# Supplementary material for: TIA1 Loss Exacerbates Fatty Liver Disease but Exerts a Dual Role in Hepatocarcinogenesis
Source: Cancers (Basel). 2022 Mar 27;14(7):1704. doi: 10.3390/cancers14071704 (PMC8997004; doi:10.3390/cancers14071704)
Supplement: Supplementary file 1 [file cancers-14-01704-s001.zip › cancers-1614817-supplementary materials/cancers-1614817-supplementary.pdf]

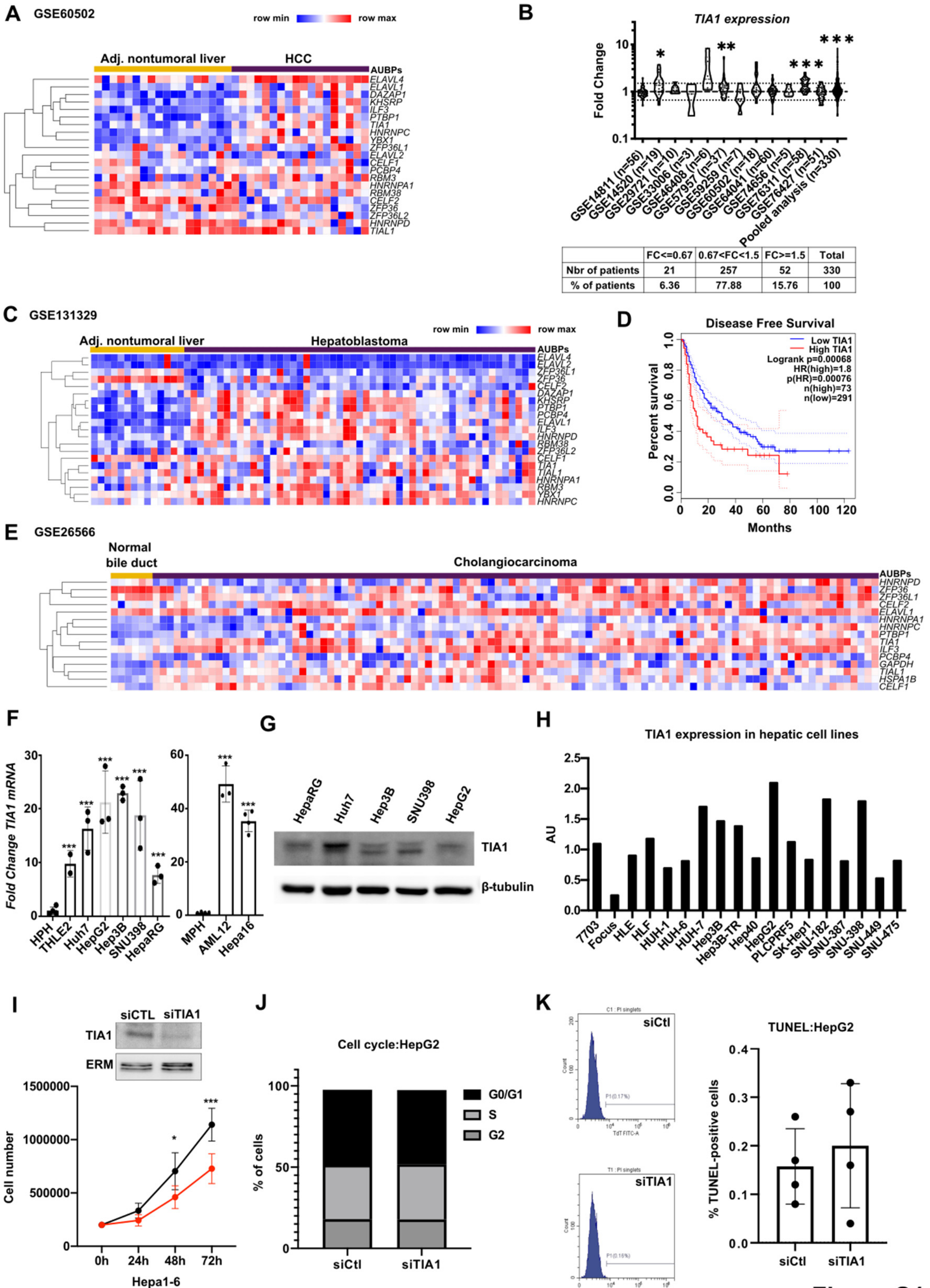

Figure S1

Figure S1. TIA1 mRNA level in HCC, ICC and hepatoblastoma and impact of its silencing on HCC cells proliferation. (A) Log2 mRNA expression of AUBPs in hepatocellular carcinoma (HCC) patients from a transcriptomic dataset (GSE60502: n=18). (B) Relative mRNA expression of TIA1 in other transcriptomic datasets of HCC patients vs. adjacent non-tumoral tissue, paired t-test was used. (C-E) Heatmaps showing the mRNA expression of AUBPs in GEO datasets of hepatoblastoma (C) and cholangiocarcinoma (ICC) (E). Graphs represent log2 expression of tumoral vs. non-tumoral tissue, hierarchical clustering was performed (One minus Pearson correlation, average linkage). (D)

Kaplan-Meier plot showing disease-free survival of patients expressing high (top 20%) and low levels (remaining 80%) of TIA1 expression in the TCGA LIHC cohort, retrieved form the GEPIA2 database (p-value calculated using the Log-rank test). (F) TIA1 mRNA level in human and mouse normal primary hepatocytes (HPH and MPH) and in a panel of human (THLE2, Huh7, HepG2, SNU398, Hep3B, HepaRG) and mouse (AML12 and Hepa1-6) hepatic cell lines (n=3), normalized by *ACTB*. (G) Western Blot showing TIA1 levels in a panel of hepatic cell lines (the western blot is representative of 3 independent experiments), β-tubulin was used as loading control. (H) *TIA1* expression in hepatic cell lines, based on data from the Cell Miner (http://www.medicalgenomics.org/cellminerhcc). (I) Cell number of Hepa1-6 (n=4) after TIA1 silencing (siTIA1) vs. control cells (siCtl). One-way ANOVA was used. The western blot validating TIA1 knockdown is shown above the graph. (J) Distribution of cell cycle phases in HepG2 cells siTIA1 and siCtl (n=3). (K) Percentage of TUNEL positive cells in HepG2 siTIA1 and siCtl (n=3). Data was represented as mean ± SD. Unpaired t-test was used for comparison of two groups, unless specified otherwise. *P<0.05, **P<0.01, ***P<0.001.

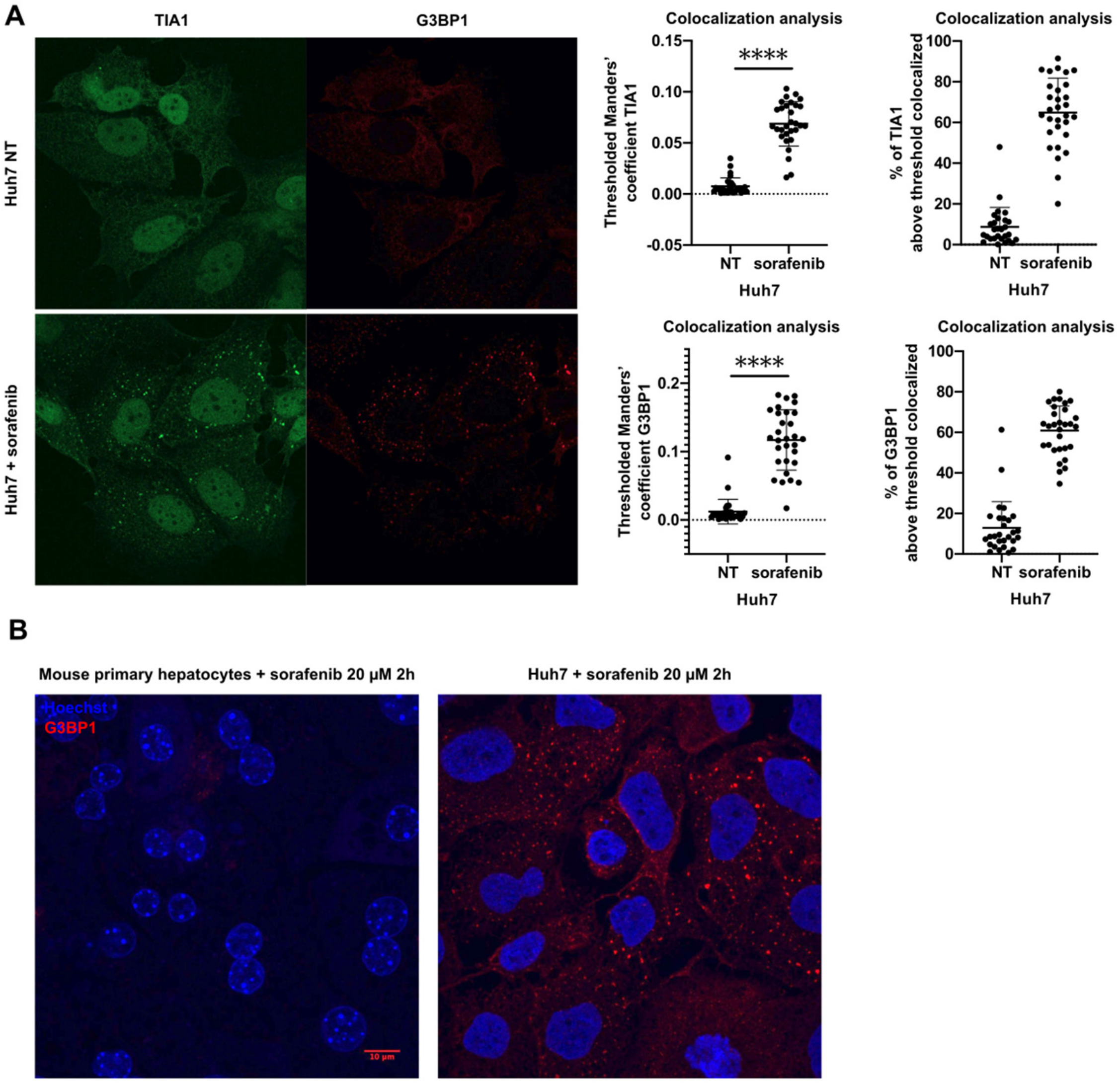

Figure S2. TIA1 is a reliable marker of stress granules in sorafenib-treated HCC cells. (A) Representative pictures of TIA1 (green) and G3BP1 (red) signals after treatment with 20 µM sorafenib (SORAF) (vs. untreated cells (NT)) in Huh7 (n=3) for 2h and quantification of their colocalization using Mander's coefficient and % of signal above threshold colocalized. (B) Representative pictures of G3BP1 (red) signal after treatment of mouse primary hepatocytes (MPH) vs. Huh7 cells for 2h with 20 µM sorafenib. Data was represented as mean ± SD. Unpaired t-test was used for comparison of two groups, unless specified otherwise. *P<0.05, **P<0.01, ***P<0.001.

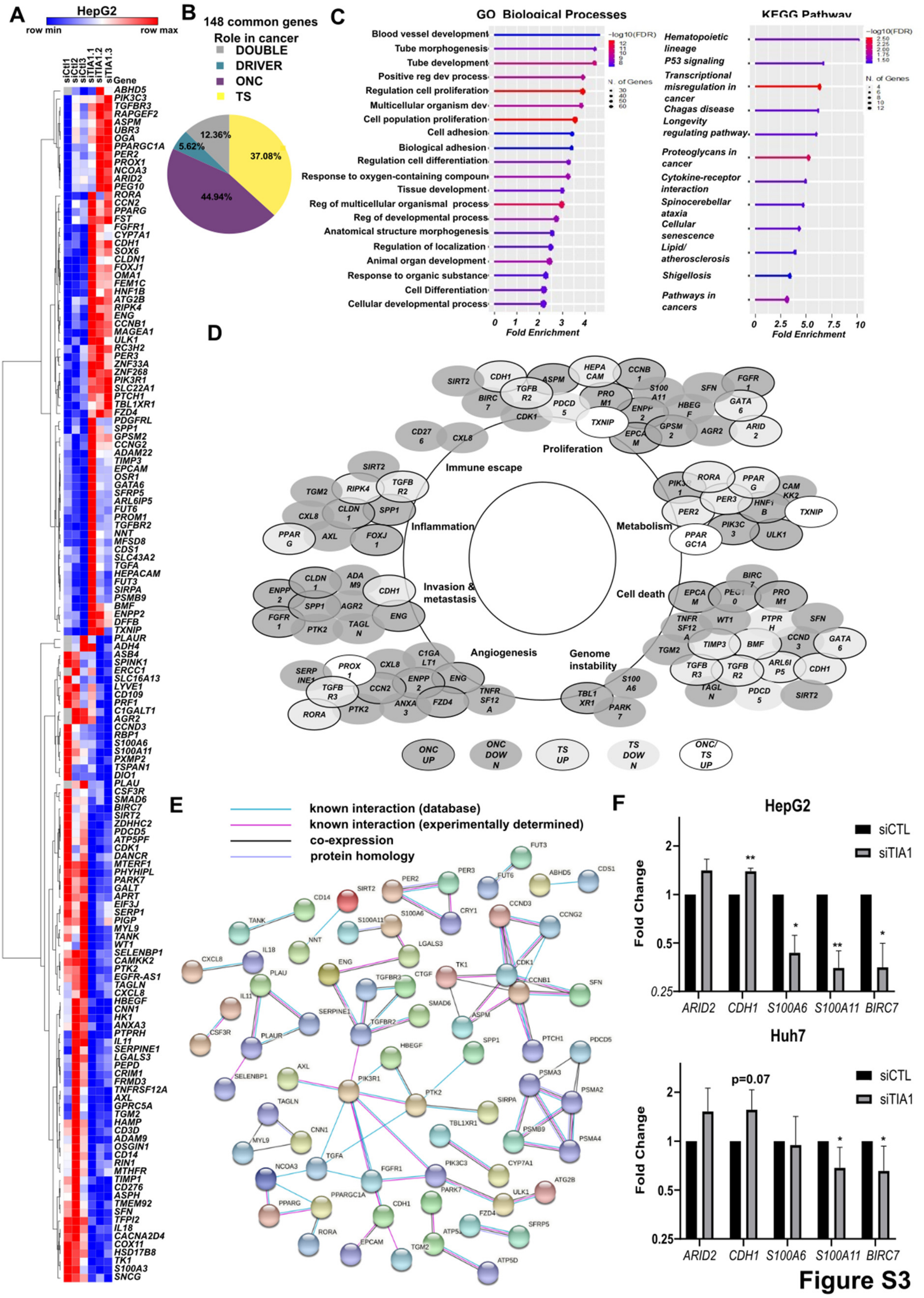

Figure S3. RNAseq on polysomes from HepG2 cells following TIA1 silencing. (A) Heat map showing deregulated genes (FC>=1.5, p-value<0.01) following an RNA sequencing of polysomal (3+) fractions HepG2 cells treated with siTIA1 or siCtl crossed with HCC-related genes (MetaCore). (B) Pie chart showing CancerMine annotation of 148 genes common between HepG2 siTIA1 deregulated genes and MetaCore HCC [1]. (C) Gene ontology enrichment analysis for biological processes and KEGG pathway (ShinyGO database: http://bioinformatics.sdstate.edu/go/) with the 148 genes

common between HepG2 siTIA1 deregulated genes and MetaCore HCC. (D) Deregulated genes classified into hallmarks of cancer based on GeneCards database. (E) Network of deregulated genes retrieved from STRING database (medium confidence). (F) qPCR analysis for strongly deregulated candidates in HepG2 and Huh7 cells. *P<0.05, **P<0.01, ***P<0.001.

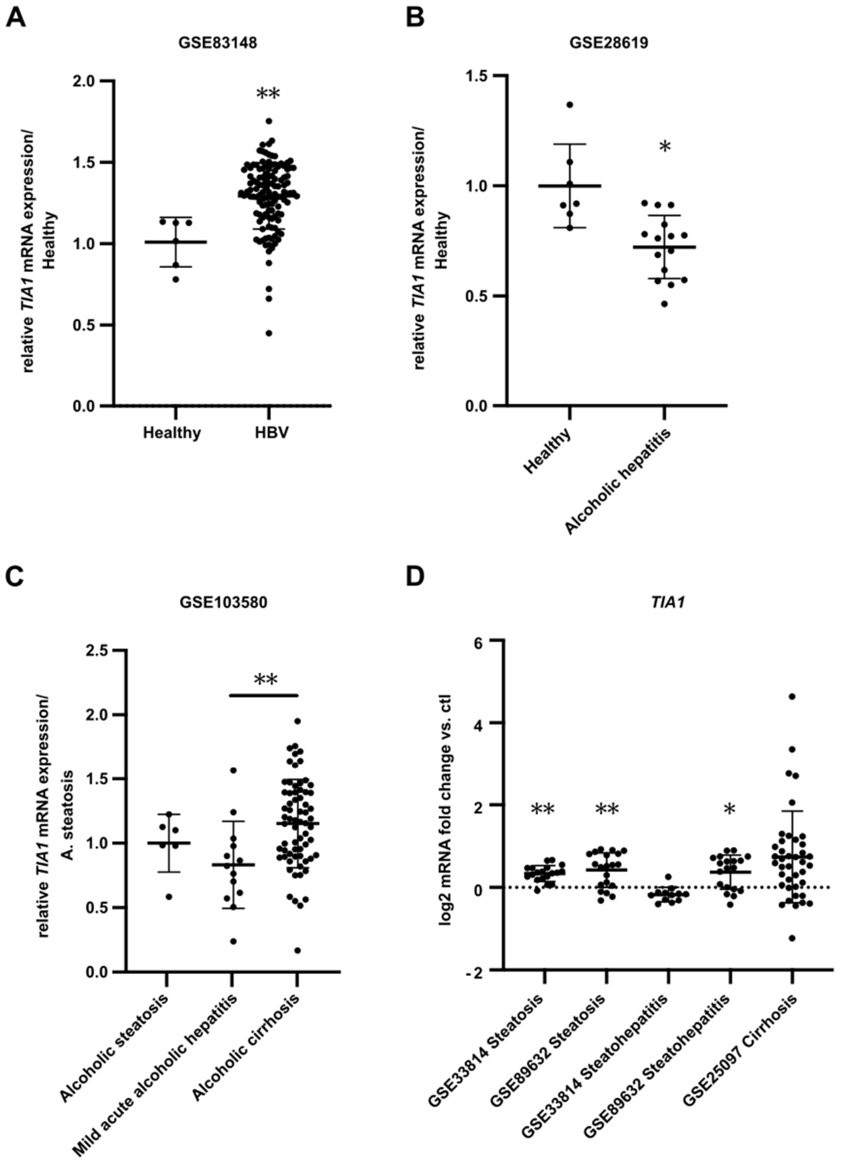

Figure S4. *TIA1* mRNA level in various chronic liver diseases. (A) Relative mRNA expression of *TIA1* in Hepatitis B virus (HBV)-infected vs. healthy patients (GSE83148). (B) *TIA1* expression in patients diagnosed with alcoholic hepatitis vs. healthy patients (GSE28619). (C) *TIA1* expression in patients diagnosed with various liver diseases (GSE103580). (D) *TIA1* expression in human liver diseases. Data was represented as log2 fold change vs. healthy patients. P-values were retrieved from GEO2R analyses. P-values were based on a GEO2R analysis (Benjamini & Hochberg, False discovery rate). *P<0.05, **P<0.01, ***P<0.001.

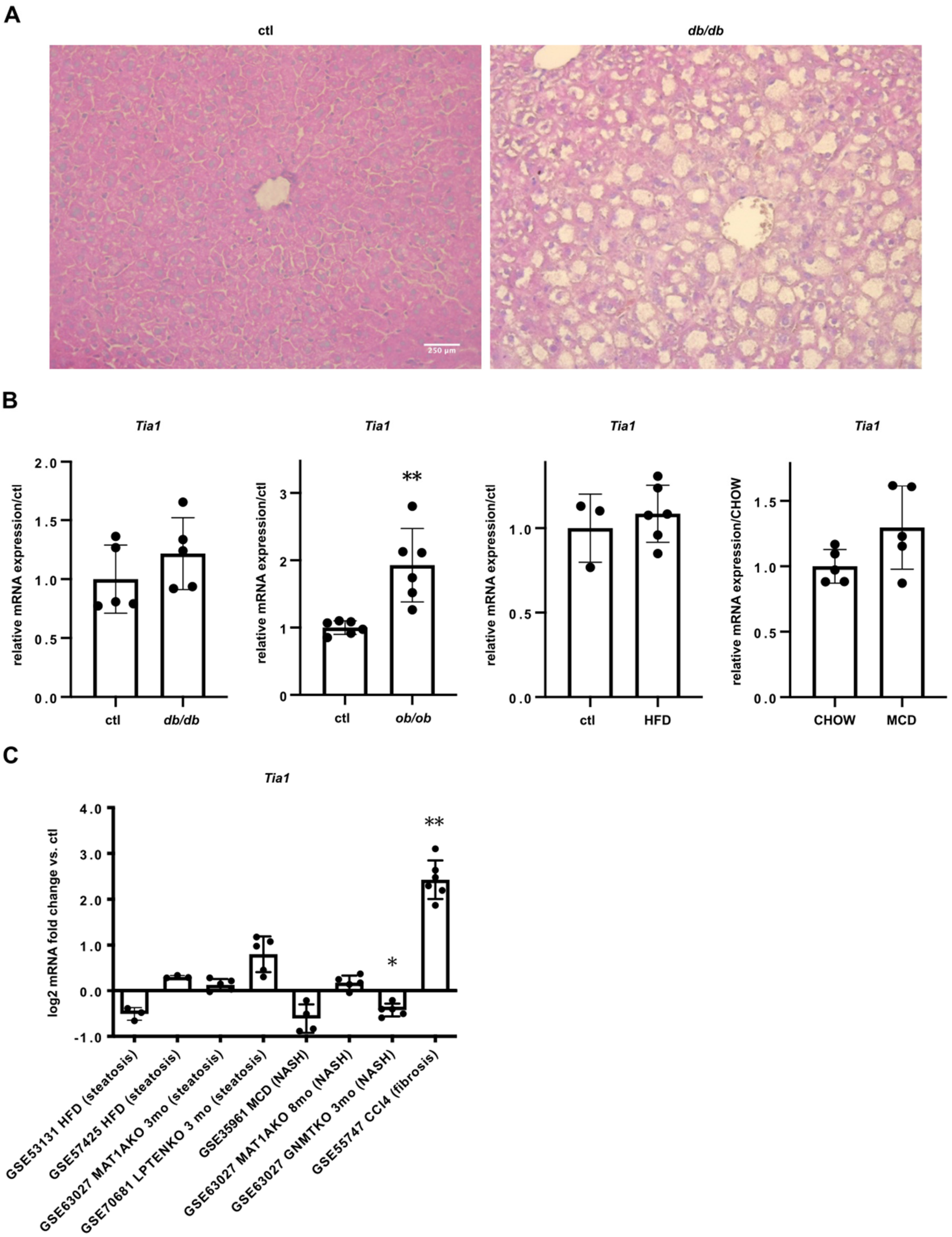

Figure S5. *TIA1* mRNA level in mouse models of NAFLD/NASH. (A) Representative H&E staining pictures of *db/db* and ctl mice. (B) Relative mRNA expression of *Tia1* in *db/db*, *ob/ob* mice and mice fed a high-fat diet (HFD) and a choline deficient diet (MCD), vs. corresponding control mice, normalized by *PPIA*. Unpaired t-test was used for comparison of two groups. (C) Relative mRNA expression of *Tia1* in mouse models of liver diseases. Data was represented as log2 fold change vs. healthy patients. GSE p-values were retrieved from GEO2R analyses (Benjamini & Hochberg (False discovery rate)). *P<0.05, **P<0.01, ***P<0.001.

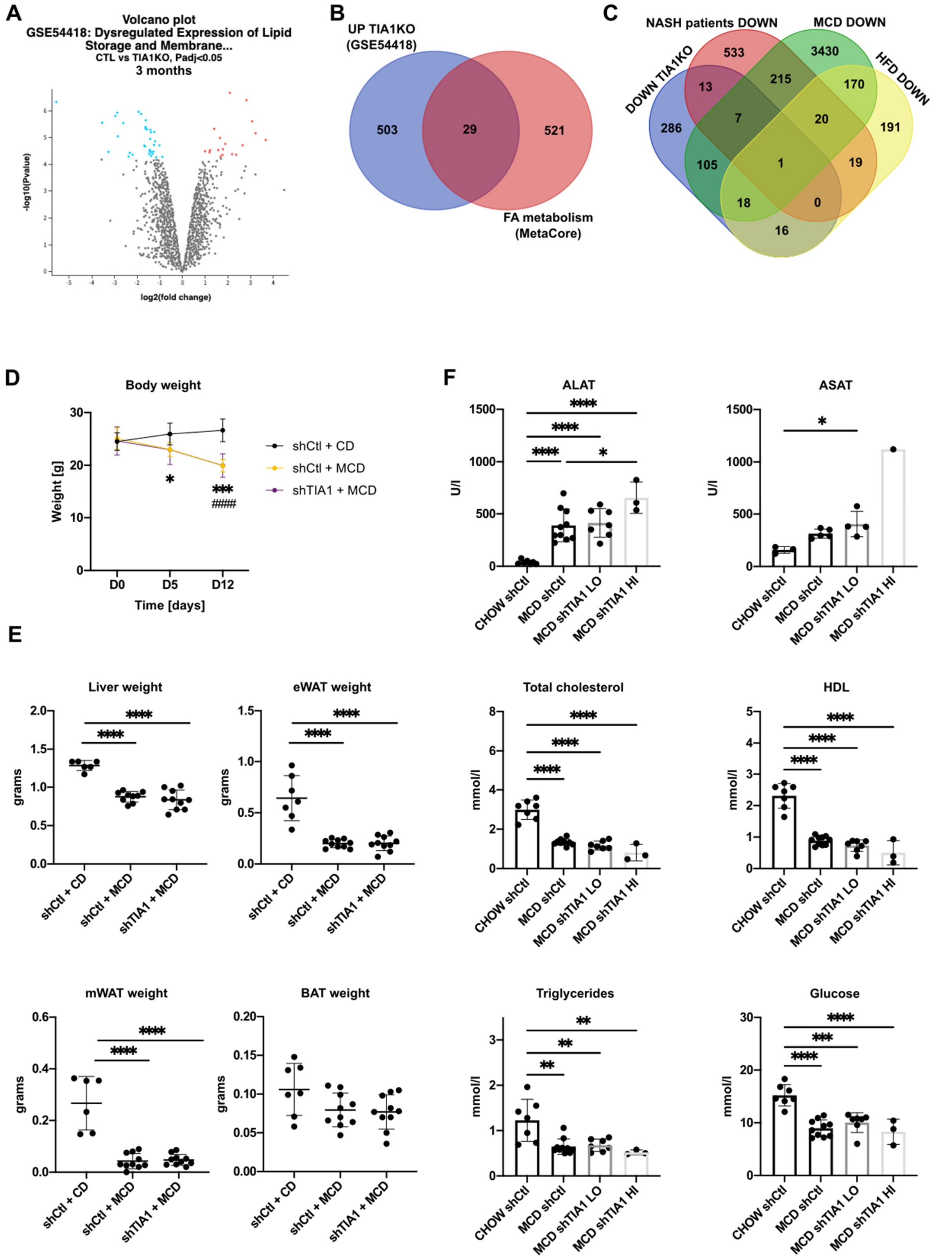

Figure S6. Impact of TIA1 loss on NAFLD and NASH development. (A) Volcano plot showing deregulated genes of livers in 3-mo TIA1KO mice (GSE54118). (B) Venn diagram showing upregulated genes of livers in TIA1KO mice (GSE54118) crossed with genes associated to fatty acid (FA) metabolism retrieved from MetaCore. (C) Venn diagram showing downregulated genes of livers in TIA1KO mice (GSE54118) crossed with genes deregulated in datasets of livers of human NASH patients (GSE33814) and mice fed an MCD (GSE35961) or a HFD (GSE57425). (D) Body weight of shCtl and shTIA1 mice fed an MCD (n = 10 per group) or CHOW (n =5) diet for 2 weeks. (E) Organ weight of shCtl and shTIA1 mice fed an MCD (n =10 per group) or CHOW (n=5) diet for 2 weeks.

In case of missing values, the organs were not retrieved at sacrifice. (F) Plasma levels of ALAT, ASAT, total cholesterol, glucose, HDL, and triglycerides in C57BL/6J mice injected with an AAV8 virus coding for shRNA against TIA1 (shTIA1) or a scrambled control (shCtl) and fed an MCD or a CHOW diet (n = 10 per group for the MCD, n=5 for the CHOW diet). In case of missing values, the quality of plasma made the analysis impossible. shTIA1 mice were divided based on their fibrosis score. One-way ANOVA with multiple comparisons was used for comparison of three or more groups. *P<0.05, **P<0.01, ***P<0.001.

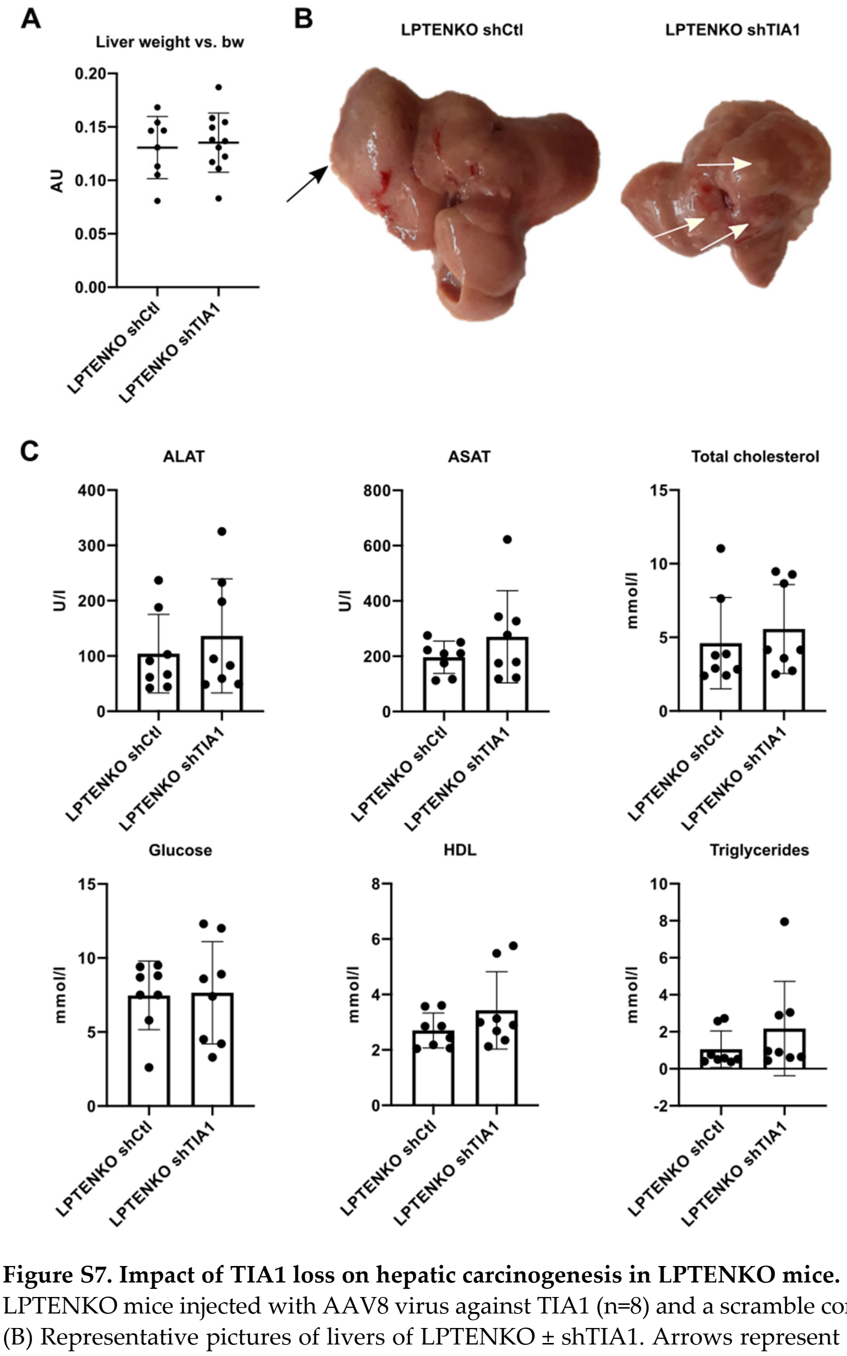

Figure S7. Impact of TIA1 loss on hepatic carcinogenesis in LPTENKO mice. (A) Liver weight of LPTENKO mice injected with AAV8 virus against TIA1 (n=8) and a scramble control (shCtl) (n=11). (B) Representative pictures of livers of LPTENKO ± shTIA1. Arrows represent tumor nodules. (C) Plasma levels of ALAT, ASAT, total cholesterol, glucose, HDL, and triglycerides in LPTENKO ± shTIA1 (n=8). Unpaired t-test was used for comparison of two groups. *P<0.05, **P<0.01, ***P<0.001.

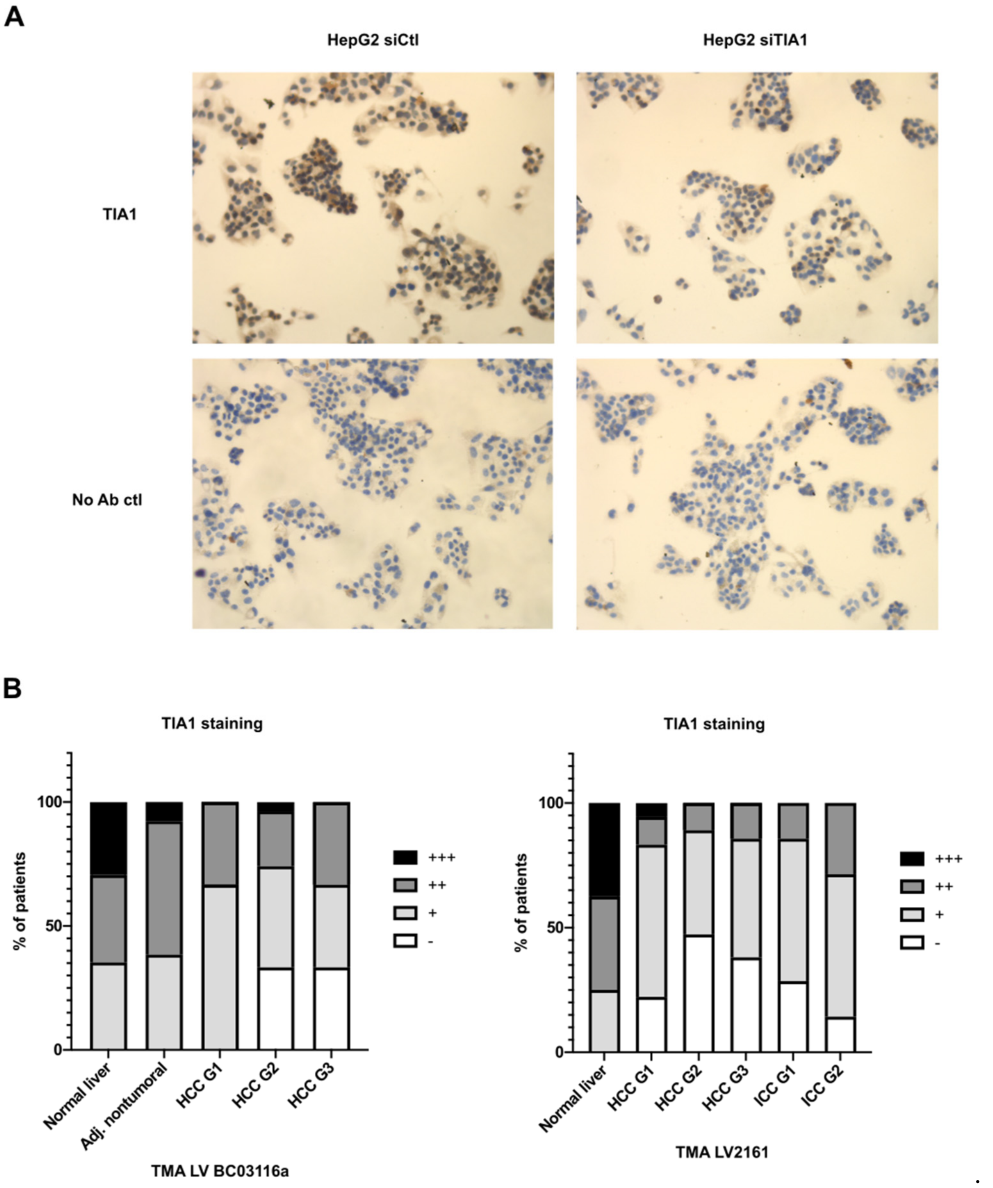

Figure S8. Immunostaining of TIA1 in HCC cells and human tissue microarrays of HCC patients. (A) Representative ICC staining for TIA1 (brown) in HepG2 cells. (B) Intensity of TIA1 staining in BC03116a and LV2161 Tissue Microarrays (TMAs) containing HCC and ICC samples based on a qualitative scale from "+++" – highly intense staining to "-", no staining (individual analysis) (n=69 and n=206, respectively). *P<0.05, **P<0.01, ***P<0.001.

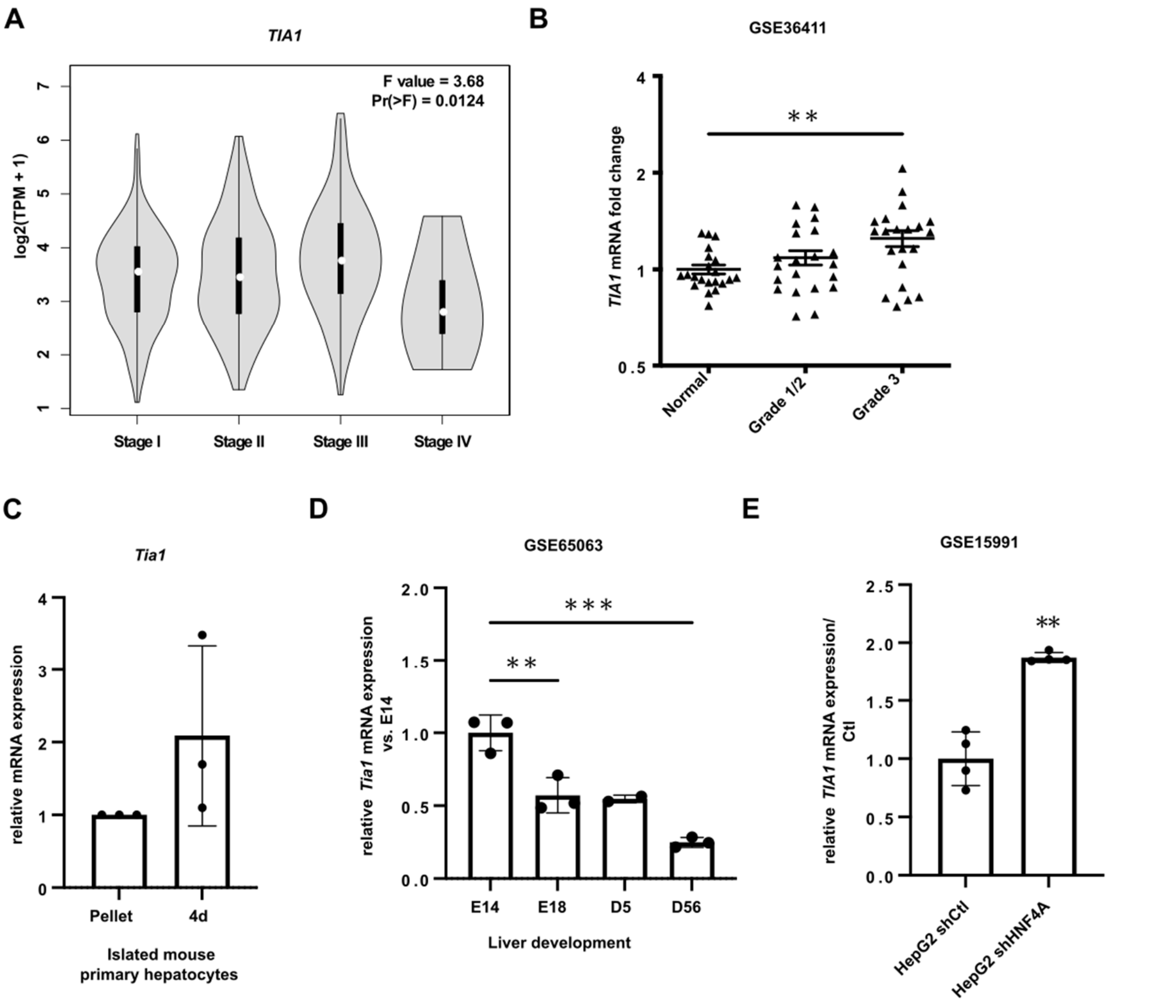

Figure S9. TIA1 mRNA level in HCC and liver development. (A) Log2(TPM+1) *TIA1* mRNA expression divided by HCC stages in the LIHC TCGA cohort. (B) Relative mRNA expression of *TIA1* in different grades of HCC in the GSE36411 dataset. (C) Relative mRNA expression of *Tia1* in mouse primary hepatocytes after isolation (pellet) and plating (D4), normalized by *18S*. (D) *Tia1* expression during mouse liver development (GSE65063). (E) Relative mRNA expression of *TIA1* in HepG2 cells treated with shRNA against HNF4α (GSE15991). Unpaired t-test was used for comparison of two groups and one-way ANOVA with multiple comparisons was used for comparison of three or more groups, unless specified otherwise. *P<0.05, **P<0.01, ***P<0.001.

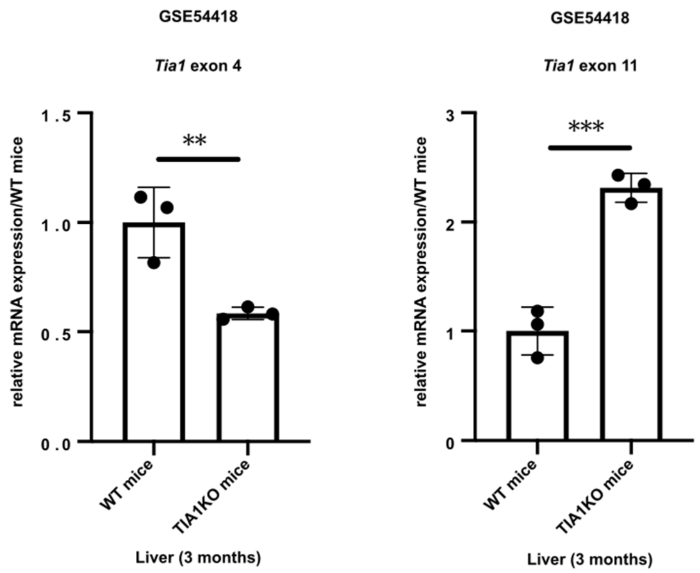

Figure S10. Relative mRNA expression of TIA1 exon 4 and exon 11 in livers of TIA1KO vs. wild type (WT) mice at 3 months (GSE54118). P-value was retrieved from GEO2R. *P<0.05, **P<0.01, ***P<0.001.

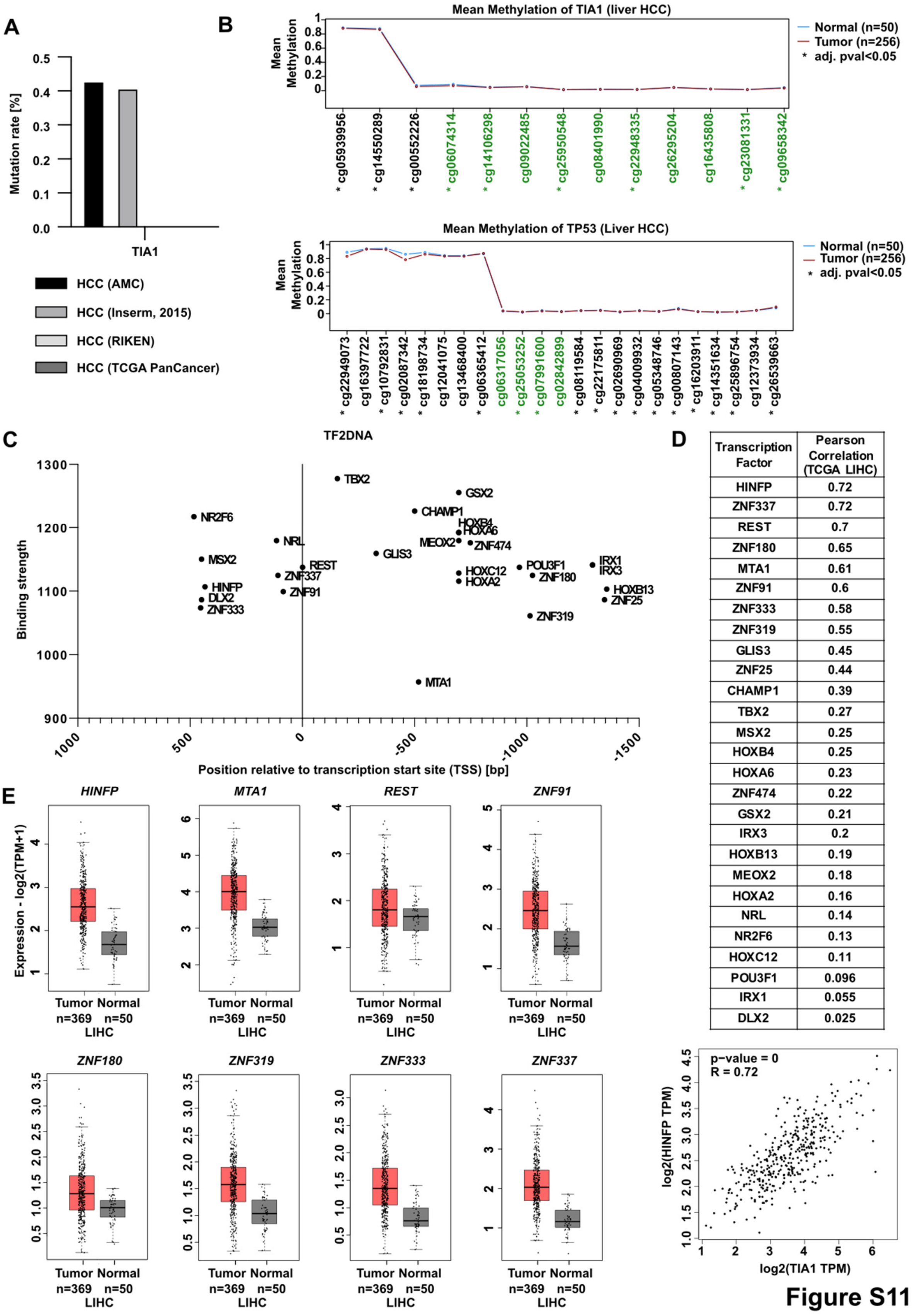

Transcription Factor	Pearson Correlation (TCGA LIHC)
HINFP	0.72
ZNF337	0.72
REST	0.7
ZNF180	0.65
MTA1	0.61
ZNF91	0.6
ZNF333	0.58
ZNF319	0.55
GLIS3	0.45
ZNF25	0.44
CHAMP1	0.39
TBX2	0.27
MSX2	0.25
HOXB4	0.25
HOXA6	0.23
ZNF474	0.22
GSX2	0.21
IRX3	0.2
HOXB13	0.19
MEOX2	0.18
HOXA2	0.16
NRL	0.14
NR2F6	0.13
HOXC12	0.11
POU3F1	0.096
IRX1	0.055
DLX2	0.025

Figure S11. Potential regulatory mechanisms of TIA1 expression. (A) Mutation rate of TIA1 in HCC patients retrieved from cBioPortal[2,3]. (B) Methylation profile of TIA1 between tumors and healthy livers from the TCGA LIHC cohort, retrieved from Wanderer[4]. Probes corresponding to CpG islands are shown in green. TP53 was used as a positive control. (C) Potential transcription

factors (TFs) binding to the promoter of TIA1 retrieved for the TF2DNA database (http://www.fiserlab.org/tf2dna_db/[5]). (D) Pearson correlation coefficient for TIA1 and its potential transcription factors in the LIHC TCGA cohort, and exemplary graph of the correlation between *TIA1* and *HINFP*, retrieved from GEPIA2 (http://gepia2.cancer-pku.cn/#index). (E) The expression of most correlated TFs in tumors vs. non-tumoral tissues of the TCGA LIHC cohort, retrieved from GEPIA2. *P<0.05, **P<0.01, ***P<0.001.

A

miRNAs regulating TIA1 (miRBase)

microRNA	miRBase ID	PMID	Expression in HCC	Expression in HCC PMID
hsa-miR-16-5p	MIMAT0000069	18362358	down	30657555
hsa-miR-15a-5p	MIMAT0000068	18362358		
hsa-miR-26b-5p	MIMAT0000083	19088304	upregulated	30593845<br>31276277
hsa-miR-33a-5p	MIMAT0000091	20371350	downregulated	28291769<br>32021242
hsa-miR-1229-3p	MIMAT0005584	23622248	unknown	
hsa-miR-331-3p	MIMAT0000760	23622248	upregulated	24825302

B

Literature-based miRs targeting TIA1

microRNA	Cancer type	PMID	Expression in HCC	Expression in HCC PMID
miR-19a-3p	Colorectal cancer	28257633	upregulated	32201518
miR-487a-3p	Gastric cancer	30144499	upregulated	27827315
miR-34a	Myeloid-derived suppressor cells	24780820	downregulated	30627547

C

Survival and expression analysis of miRs in liver cancer (OncomiR database)

miRNA Name	Cancer	Log Rank P-value	Log Rank FDR	Upregulated in:	Deceased Log2 Mean Expression	Living Log2 Mean Expression	T-Test P-value	T-Test FDR
hsa-miR-331-3p	LIHC	6.00E-05	2.10E-01	Deceased	4.95	4.63	2.12E-03	5.93E-02
hsa-miR-1229-3p	LIHC	6.67E-03	6.90E-01	Deceased	0.46	0.26	1.97E-01	5.21E-01
hsa-miR-33a-5p	LIHC	1.55E-02	2.32E-01	Deceased	5.49	5.05	1.01E-02	1.26E-01
hsa-miR-16-5p	LIHC	4.50E-02	4.66E-01	Deceased	9.03	8.84	5.03E-02	2.82E-01
hsa-miR-26b-5p	LIHC	1.03E-01	7.35E-01	Deceased	10.28	10.06	2.00E-02	1.81E-01
hsa-miR-34a-5p	LIHC	1.38E-01	6.12E-01	Living	8.54	8.6	6.01E-01	7.46E-01
hsa-miR-19a-3p	LIHC	3.47E-01	3.33E-01	Deceased	6.26	5.95	2.39E-01	5.51E-01
hsa-miR-15a-5p	LIHC	3.73E-01	4.25E-01	Living	7.86	7.9	6.06E-01	7.51E-01
hsa-miR-34a-3p	LIHC	7.98E-01	7.31E-01	Deceased	0.52	0.5	8.22E-01	8.93E-01
hsa-miR-487a-3p	LIHC	9.81E-01	7.91E-01	Living	1.12	1.43	4.39E-01	6.19E-01

D

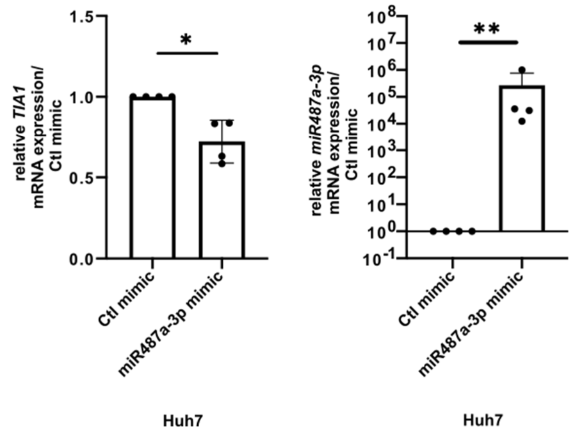

Figure S12. MicroRNAs-dependent regulation of TIA1 expression. (A) Table summarizing potential miRNAs targeting TIA1, retrieved from miRBase (https://www.mirbase.org/). (B) Literature-based screening of miRNAs targeting TIA1. (C) Survival and expression analysis of miRs in the

TCGA LIHC cohort, retrieved from the OncomiR database (http://www.oncomir.org/)[6]. (D) Relative mRNA expression of *TIA1* and *miR-487a-3p* in Huh7 cells treated with a mimic of miR-487a-3p. Data was represented as mean ± SD. Unpaired t-test was used for comparison of two groups. *P<0.05, **P<0.01, ***P<0.001.

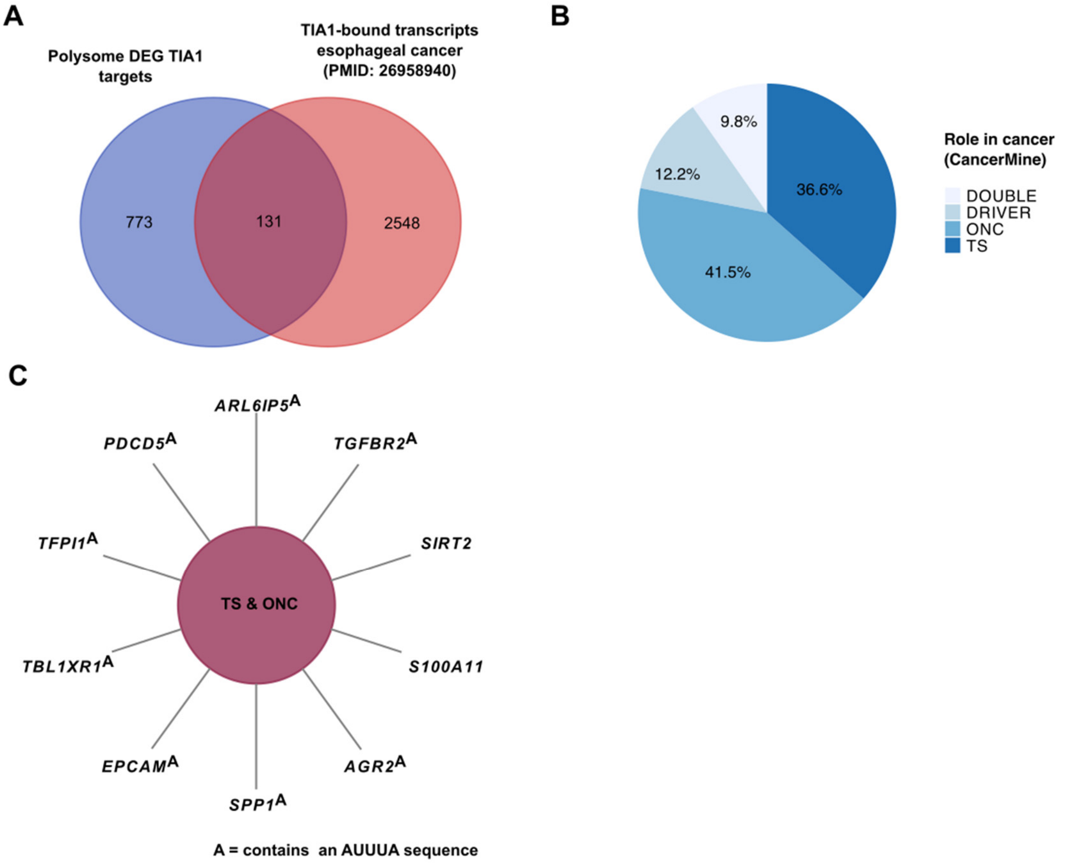

Figure S13. Potential TIA1 mRNA targets involved in hepatocarcinogenesis. (A) Venn diagram representing TIA1 targets in esophageal cancer [7] crossed with genes associated with genes deregulated in the HepG2 translatomics resulting from the polysome profiling. (B) Pie chart showing 131 common genes annotated for their role in cancer with CancerMine. (C) Sun plot showing TS and ONC containing or not an AUUUA sequence.

A

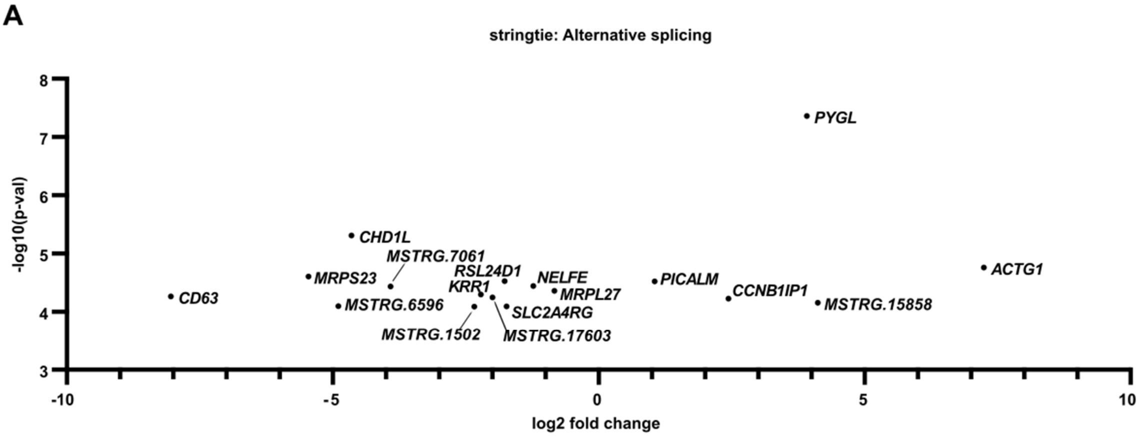

id	geneNames	geneIDs	feature	id	fc	pval	qval
66032	PYGL	MSTRG.7061	transcript	66032	15.0883317	4.38E-08	0.00075196
12253	CHD1L	MSTRG.1502	transcript	12253	0.03983295	4.90E-06	0.04202219
106069	ACTG1	MSTRG.11533	transcript	106069	151.117175	1.73E-05	0.07861371
102180	MRPS23	MSTRG.11107	transcript	102180	0.02279609	2.47E-05	0.07861371
75398	RSL24D1	MSTRG.8047	transcript	75398	0.29344062	2.98E-05	0.07861371
40825	PICALM	MSTRG.4493	transcript	40825	2.07354069	2.99E-05	0.07861371
198266	NELFE	MSTRG.20797	transcript	198266	0.42601094	3.60E-05	0.07861371
66033	.	MSTRG.7061	transcript	66033	0.06629373	3.67E-05	0.07861371
101595	MRPL27	MSTRG.11047	transcript	101595	0.56101795	4.35E-05	0.07861371
53161	KRR1	MSTRG.5716	transcript	53161	0.21525096	5.06E-05	0.07861371
50950	CD63	MSTRG.5484	transcript	50950	0.00379065	5.45E-05	0.07861371
167176	.	MSTRG.17603	transcript	167176	0.25044593	5.66E-05	0.07861371
62921	CCNB1IP1	MSTRG.6765	transcript	62921	5.43053909	5.95E-05	0.07861371
149889	.	MSTRG.15858	transcript	149889	17.3753027	6.99E-05	0.08205738
61065	.	MSTRG.6596	transcript	61065	0.03360655	7.95E-05	0.08205738
149928	SLC2A4RG	MSTRG.15858	transcript	149928	0.30086448	8.02E-05	0.08205738
12270	.	MSTRG.1502	transcript	12270	0.19761812	8.13E-05	0.08205738

B

	Role in HCC	PMID HCC
ACTG1	ONC	30881024
CCNB1IP1	overexpressed in HCC	27913571
CD63	TS	33277798
CHD1L	ONC	20335658
KRR1	-	-
MRPL27	-	-
MRPS23	ONC	28714366
PICALM	-	-
PYGL	-	-
RSL24D1	-	-
SLC2A4RG	-	-

Figure S14. TIA1 controls the splicing of several transcripts involved in HCC. (A) Graph and table showing alternative transcript usage in translatomics of HepG2 siTIA1 vs. siCtl obtained using stringtie with FDR (q-val) <0.1. (B) Summary of the role in HCC of transcripts potentially spliced alternatively by TIA1.

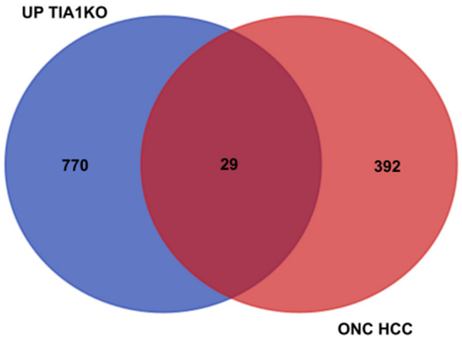

29 genes

E2F8	*CKS1B*	*CCND1*	*EGFR*	*DLC1*
FOXQ1	*STAT3*	*MYC*	*CREB1*	*CEACAM1*
EZH2	*BMP7*	*NTRK3*	*CDT1*	*UHRF1*
FOXA2	*KIF2C*	*PBK*	*GRN*	*SGK3*
CDCA4	*TCF19*	*KDM5B*	*CDKN1A*	*CSF1R*
CDX2	*DNMT3A*	*CDK9*	*DDX58*	

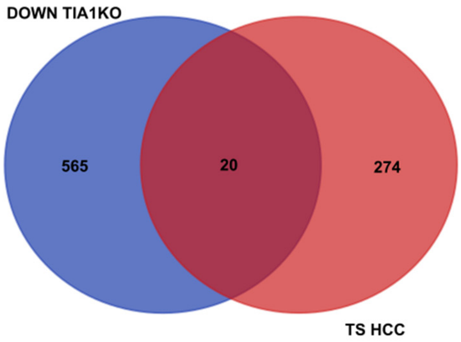

20 genes

NR3C2	*AKAP12*	*PCDH17*	*FHL2*
VAC14	*MYCN*	*CADM1*	*DLC1*
TAT	*TIAM1*	*SOX6*	*WWOX*
NR4A1	*NR0B2*	*KLF4*	*IL1RAPL1*
CDH1	*SORBS2*	*SULF1*	*TOB1*

Figure S15. Deregulation of oncogenes and tumor suppressors in the liver of TIA1KO mice. Venn diagrams showing deregulated genes of 6 months' old livers of TIA1KO mice (GSE54118) crossed with lists of oncogenes and tumor suppressors derived from CancerMine.

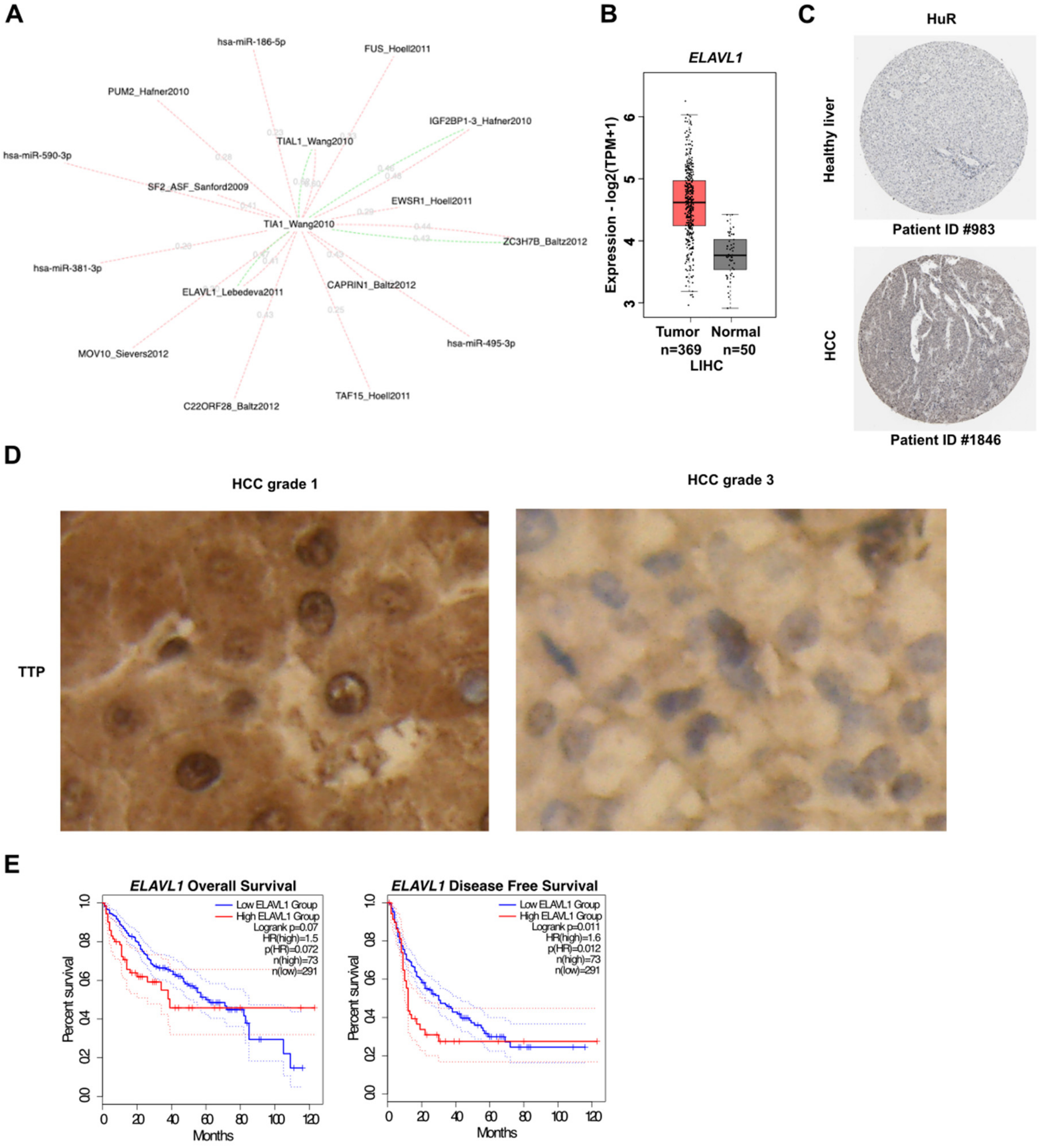

F

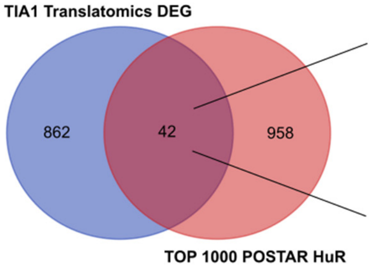

ARID2	FRMD5	NFIA	RC3H1	ZNF207
ASB3	GDAP1	NUP58	REV3L	ZNF529
ASPH	ICE1	PANK3	RORA	
ASPM	LCLAT1	PAWR	RYK	
BBX	LIN28B	PDE4D	SHOC2	
CDC73	LSM14A	PIK3C3	TBL1XR1	
CDK19	MDM4	POLQ	TIAM2	
CDKAL1	MED13L	PPM1L	TTLL5	
CENPP	MEIS2	PTK2	UBR3	
CPNE8	NEK7	RAPGEF2	UHMK1	

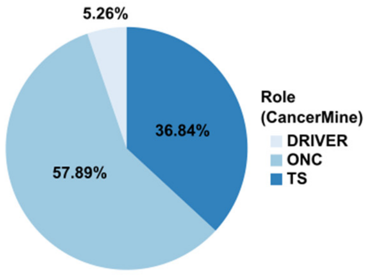

Figure S16. TIA1 is part of a network of RNA-Binding proteins involved in HCC. (A) Network of TIA1 interactors retrieved from the simiRa database (http://vsicb-simira.helmholtz-muenchen.de/). (B) *ELAVL1* expression in tumors vs. healthy tissues of the LIHC TCGA cohort. (C) Representative pictures of HuR staining in healthy and HCC livers, retrieved from the Human Protein Atlas (Patient IDs: 983, 1846, Antibody CAB005256). (D) Representative pictures showing TTP staining between grade 1 and grade 3 HCC in a human TMA LV2161. (E) Kaplan-Meier plot showing overall survival of patients expressing high (top 20%) and low levels (remaining 80%) of *ELAVL1* expression in the TCGA LIHC cohort, retrieved form the GEPIA2 database (statistics based on log-rank test).

(F) Venn diagram and table showing common gens between top 1000 candidates binding to HuR retrieved from POSTAR2 and genes deregulated in the HepG2 siTIA1 translatomics with their annotation by CancerMine.

A

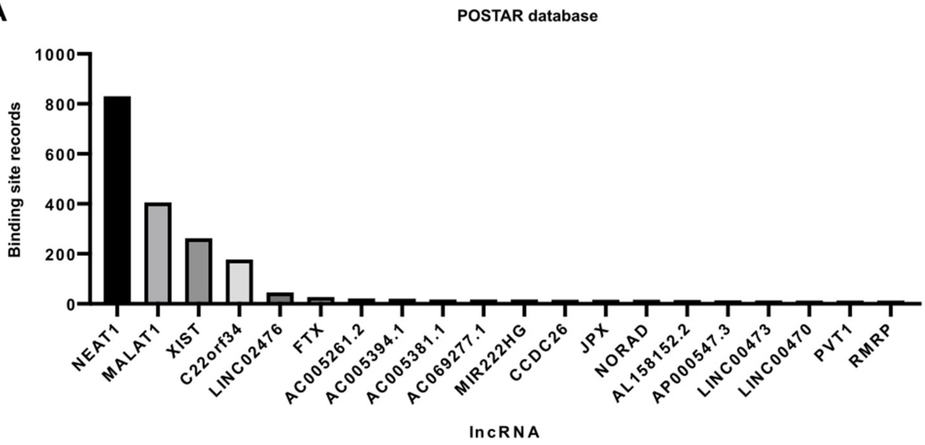

B

	Levels in HCC	Role in HCC	PMID
MALAT1	Upregulated	↑SRSF1, ↑mTOR signalling	27993818
		↑glycolysis, ↓gluconeogenesis	30914432
	Upregulated	regulation of Aurora A expression, ↑sorafenib resistance	32220970
		↑doxorubicin resistance through the miR-3129-5p/Nova1 axis	32965597
	Upregulated	↑angiogenesis through the miR-140/VEGF-A axis	31693399
NEAT1		↓lipolysis through ATGL	29764424
	Upregulated	↑proliferation and invasion, ↓apoptosis	32373951
		maintains proliferation and inhibits apoptosis under hypoxic conditions	32670881
	Upregulation	↑sorafenib resistance	32461380
	Up in HCC cell lines	↑proliferation and migration; ↓apoptosis, acts throuh the miR-320a/LAGE3 axis	32945386
	Upregualted	↑autophagy and sorafenib resistance, though miR-204/ATG3 axis	31549407

C

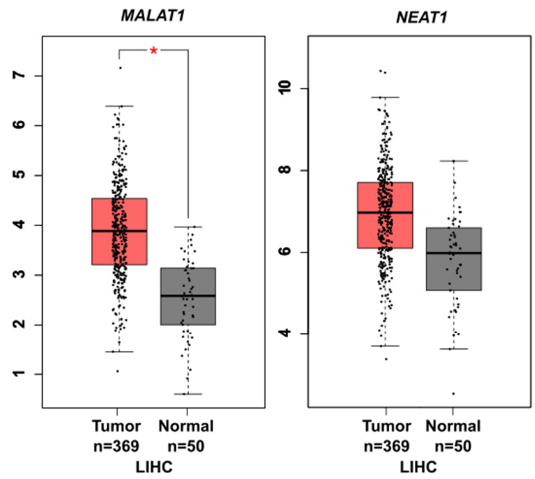

D

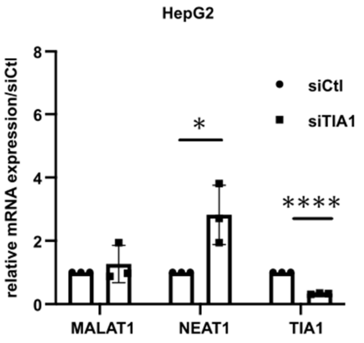

Figure S17. TIA1 binds and regulates the expression of long-non-coding RNA involved in HCC. (A) Long non-coding RNAs binding to TIA1 retrieved from the POSTAR2 database[8]. (B) Table summarizing the role of top 2 lncRNAs from (A) in HCC, based on literature. (C) mRNA expression of *MALAT1* and *NEAT1* in tumors vs. non-tumoral tissues of the TCGA LIHC cohort, retrieved from GEPIA2. (D) Relative mRNA expression of *TIA1*, *MALAT1* and *NEAT1* in HepG2 cells treated with siRNA against TIA1, *PPIA* was used as a reference gene. Unpaired t-test was used for comparison of two groups, unless specified otherwise. *P<0.05, **P<0.01, ***P<0.001.

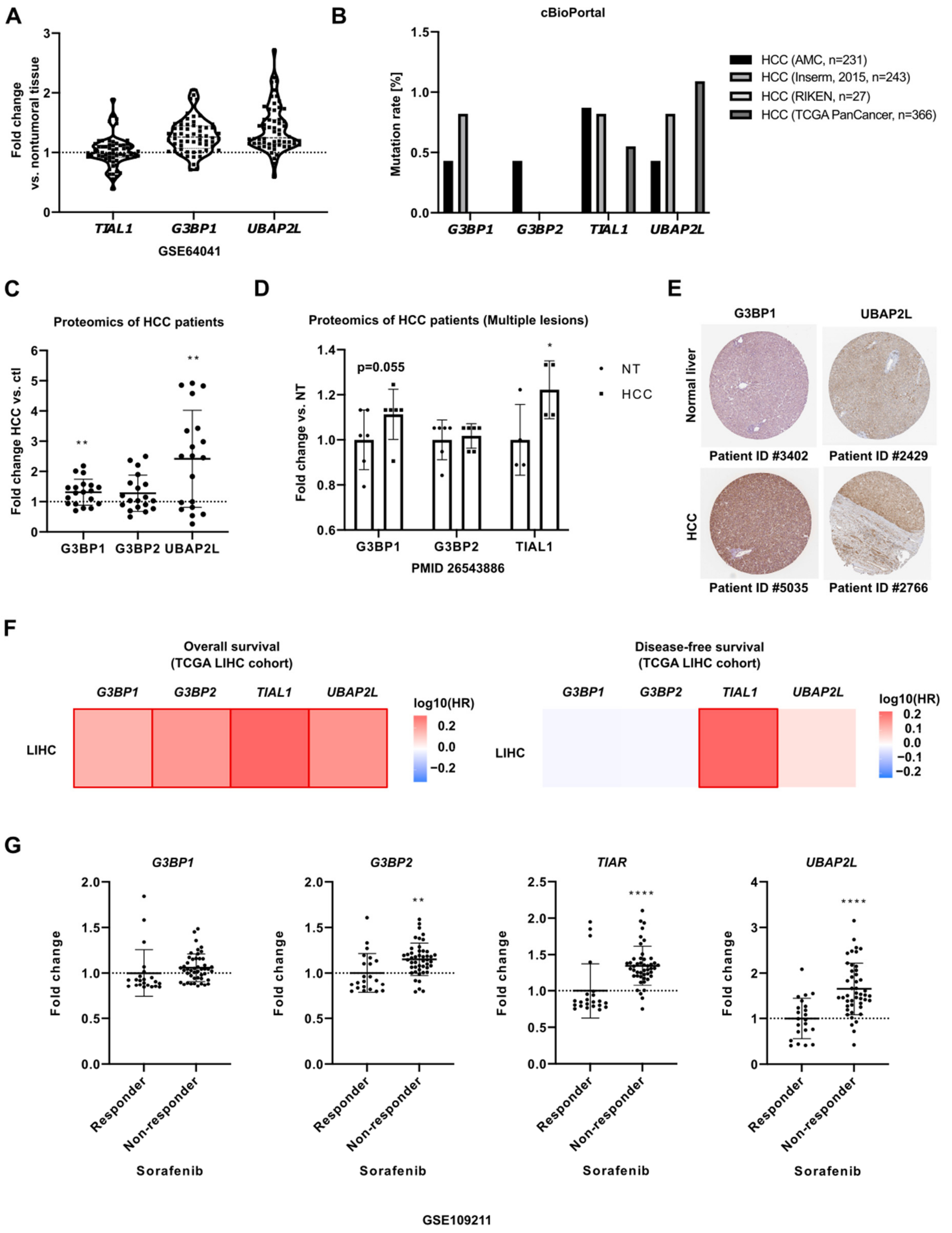

Figure S18. Expression of stress granules assembly factors in human HCC. (A) Relative mRNA expression levels of SG components in tumors of HCC patients vs. adjacent non-tumoral tissues in a transcriptomic dataset (GSE64041). (B) Mutation rates of stress granule (SG) components in different studies of HCC patients retrieved from cBioPortal (http://www.cbioportal.org)[2,3]. (C) Relative protein expression levels of SG components in a proteomics study of HCC patients (FC vs nontumoral liver) [9]. (D) Relative protein expression levels of SG components in a proteomics study of HCC patients (vs nontumoral liver, NT)[10]. (E) Representative pictures of G3BP1 staining in healthy and HCC livers, retrieved from the Human Protein Atlas. (G3BP1 patient IDs: 3402, 5035, Antibody HPA004052; UBAP2L patient IDs: 2429, 2766, Antibody HPA035068). (F) Hazard ratio of

overall and disease-free survival of 25% of patients expressing highest mRNA levels of SG components (vs. remaining patients) based on the LIHC TCGA cohort, retrieved from GEPIA2. (G) Relative mRNA expression levels of SG components in HCC patients responding to sorafenib (vs non-responders) (GSE109211). P-values were retrieved from the GEO2R (Benjamini & Hochberg (False discovery rate)) and GEPIA2 databases (log-rank test). Data was represented as mean ± SD. Unpaired t-test was used for comparison of two groups, unless specified otherwise, one-sided t-test was used in (C)-(D). *P<0.05, **P<0.01, ***P<0.001.

Table S1. Human GEO datasets used in the article.

GEO Dataset	Type	Sample Data	Disease	PMID
GSE14520	microarray	19 HCC vs matched non-tum. tissues, cohort 1	HCC	21159642
GSE14811	microarray	56 HCC vs matched non-tum. tissues	HCC	15607117
GSE15991	microarray	4 replicates of HepG2 cells treated with shHNF4A vs. control shRNA	-	21352552
GSE25097	microarray	6 healthy livers vs 40 cirrhotic livers	Cirrhosis	22634754
GSE26566	microarray	104 cholangiocarcinomas vs 6 normal bile duct	Cholangiocarcinoma	22178589
GSE28619	microarray	15 livers with alcoholic steatohepatitis vs 7 healthy livers	Alcoholic hepatitis	22637703
GSE29721	microarray	10 HCC vs matched non-tum. tissues	HCC	21747116
GSE33006	microarray	3 HCC vs matched non-tum. tissues	HCC	22923518
GSE33814	microarray	13 healthy livers vs 19 steatotic livers	Steatosis	23071592
		13 healthy livers vs 12 NASH livers	NASH	
GSE36411	microarray	21 healthy livers vs 21 cirrhotic livers	Cirrhosis	none
GSE46408	microarray	6 HCC vs matched non-tum. tissues	HCC	23922981
GSE57957	microarray	37 HCC vs matched non-tum. tissues	HCC	25093504
GSE59259	microarray	7 HCC vs matched non-tum. tissues	HCC	25945129
GSE60502	microarray	18 HCC vs matched non-tum. tissues	HCC	25376302
GSE64041	microarray	60 HCC vs matched non-tum. tissues	HCC	27499918
GSE74656	microarray	5 HCC vs matched non-tum. tissues	HCC	none
GSE76311	microarray	58 HCC vs matched non-tum. tissues	HCC	28648284
GSE76427	microarray	51 HCC vs matched non-tum. tissues	HCC	29117471
GSE83148	microarray	122 HBV-infected livers vs. 6 healthy livers	HBV	31282064
GSE89632	microarray	20 steatotic livers vs 24 healthy livers	Steatosis	25581263
		19 NASH livers vs 24 healthy livers	NASH	
GSE103580	microarray	6 steatotic livers, vs 13 with mild acute alc. hepatitis, vs 67 with alcoholic cirrhosis	Hepatitis, cirrhosis	29158192
GSE109211	microarray	22 HCC of sorafenib responers vs. 46 non-responders	HCC	30108162
GSE131329	microarray	53 hepatoblastoma samples vs. 14 noncancerous livers	Hepatoblastoma	none

Table S2. Rodent GEO datasets used in the article.

	Type	Sample Data	Disease	PMID
GSE35961	microarray	4 WT mice fed a chow or HFD+MCD for 8 weeks	NASH	23028442
GSE53131	microarray	3 WT mice fed a chow or HFD (60Kcal%fat) for 9 weeks	Steatosis	24618914
GSE54418	microarray	Livers of 3 TIA1KO mice vs. WT (3/6 months)	-	24659297
GSE55747	microarray	4 healthy WT mice vs 4 CCl4-treated mice	Fibrosis	none
GSE57425	microarray	3 WT mice fed a chow or HFD (60Kcal%fat) for 12 weeks	Steatosis	25003192
GSE63027	microarray	5 WT vs 5 MAT1A KO mice (3 months old)	Steatosis	25993042
		5 WT vs 5 GNMT KO mice (3 months old)	NASH	
		5 WT vs 5 MAT1A KO mice (8 months old)	NASH	
		4 tumors from 8 mo. GNMTKO mice vs 5 normal livers	HCC	
GSE65063	microarray	Mouse livers at different stages of development, n = 2-3	-	25738607
GSE70681	microarray	5 livers from 3 mo. LPTENKO mice vs 5 normal 3 mo livers	Steatosis	none
		3 tumors from 15 mo. LPTENKO mice vs 5 normal livers	HCC	

Table S3. is provided as a separate PDF file due to its size.

Table S4. Role in HCC and presence of AUUUA sequence of potential TIA1 targets identified in the translatomics performed in HepG2 siTIA1 cells, that were annotated by CancerMine (Figure 2).

Gene	Log2FC HepG2 Translatomic	Expression in HCC (Literature)	Final Role	AUUUA Site	PMIDs		
ENG	2.22188505	Upregulated	ONC	no	30730204	30563158	
FOXJ1	1.746218206	Upregulated	ONC	yes	22488567		
CLDN1	1.206235618	Upregulated	ONC	yes	19897486	23160379	28651932
FZD4	1.105719682	Upregulated	ONC	yes	31807066		
FGFR1	0.987952566	Upregulated	ONC	yes	33148422	32884351	24925055
ENPP2	0.933931791	Upregulated	ONC	yes	28598712		
PTCH1	0.927711168	Upregulated	ONC	yes	27744627	23359030	
PIK3R1	0.855179558	Upregulated	ONC	yes	30497511		
SPP1	0.817404616	Upregulated	ONC	yes	31814897		
CCN2	0.792191561	Upregulated	ONC	no	29967264		
PEG10	0.776274191	Upregulated	ONC	yes	28004118		
GPSM2	0.716796129	Upregulated	ONC	yes	28347229		
EPCAM	0.702406827	Upregulated	ONC	yes	29936198	29970536	
ASPM	0.698134979	Upregulated	ONC	yes	18676753	32685486	
ULK1	0.690375318	Upregulated	ONC	yes	31986961	29091866	
PIK3C3	0.618132053	Upregulated	ONC	yes	32513919		
HNF1B	0.614027629	Upregulated	ONC	yes	26311117		
FST	0.610498228	Upregulated	ONC	yes	16935389	12203361	
NCOA3	0.607125786	Upregulated	ONC	yes	33239622		
TBL1XR1	0.606592221	Upregulated	ONC	yes	34125401	26386862	
PROM1	0.590156651	Upregulated	ONC	yes	31938317		
CCNB1	0.586349292	Upregulated	ONC	yes	30363964		
TNFRSF12A	-0.598814145	Upregulated	ONC	yes	23886137	28138696	
CDK1	-0.615693371	UP TCGA RNA	ONC	yes	32133348		
PARK7	-0.618358141	Upregulated	ONC	yes	28036277	21410067	21410067
CAMKK2	-0.630679294	Upregulated	ONC	yes	25847065		
CCND3	-0.740185071	Upregulated	ONC	yes	25439221	31301177	
PTK2	-0.752458996	Upregulated	ONC	yes	30849480		
SFN	-0.758863852	Upregulated	ONC	no	33221766		
SPINK1	-0.795661846	Upregulated	ONC	no	32066292	28544403	
S100A6	-0.83817682	Upregulated	ONC	yes	33335992	24281831	
C1GALT1	-0.857300689	Upregulated	ONC	yes	25089569	23832667	
ANXA3	-0.872613931	Upregulated	ONC	yes	26095609	24375474	
TSPAN1	-0.87772547	Upregulated	ONC	no	22378020	20890423	
CXCL8	-0.958898766	Upregulated	ONC	yes	31684995	26078356	
ADAM9	-0.966654564	Upregulated	ONC	yes	32245188	29432845	34528498
CD276	-1.007711461	UP in metastatic tumors	ONC	yes	33204103	25908926	
TAGLN	-1.069060208	Up in cancer stem cells	ONC	no	20707403		
TIMP1	-1.132852118	Upregulated	ONC	no	25909286		
TGM2	-1.211797931		ONC	yes	30393774		
SERPINE1	-1.225004265	Upregulated	ONC	yes	33831787		
S100A11	-1.29457183	Upregulated	ONC	no	31919231	24376686	
SIRT2	-1.386782256	Upregulated/Downregulated	ONC	no	28992545	23348706	22014574
AGR2	-1.622282794	Upregulated	ONC	yes	22828706	32493835	
HBEGF	-1.735651141	Upregulated	ONC	yes	7958694	34028973	31867749

AXL	-1.76261532	Upregulated	ONC	no	24233839	25251599	
BIRC7	-1.887315678	Upregulated	ONC	no	24223461	24934632	
WT1	-2.087359971	Upregulated	ONC	yes	34720052	29285297	18255279
SOX6	1.169888594	Downregulated	TS	yes	23731550	28892647	
RORA	1.044418136	Downregulated	TS	yes	24798975	31365778	
ARID2	1.026616844	Downregulated	TS	yes	28238438	32071245	
PER3	1.002619793	Downregulated	TS	yes	22689435		
BMF	0.979424867	Downregulated	TS	yes	19671867		
PER2	0.936346142	Downregulated	TS	yes	29770483	32700769	18444243
TGFBR3	0.875643762	Downregulated	TS	yes	19639191	21829018	26882862
GATA6	0.814138274	Downregulated	TS	yes	30834518		
CDH1	0.794372159	Downregulated	TS	yes	32239101	31551254	26206264
TGFBR2	0.769853015	Downregulated	TS	yes	21898503	28352351	
HEPACAM	0.763219381	Downregulated	TS	no	15885354	12971969	
TIMP3	0.666643353	Downregulated	TS	yes	25171061	27222429	
PPARG	0.66164918	Unclear	TS	yes	22472882		
ARL6IP5	0.648852601	Downregulated	TS	yes	23169062		
RIPK4	0.640526912	Downregulated	TS	no	34222329		
PTPRH	-0.642675797	Down with undifferentiation	TS	yes	12879010		
RIN1	-0.738328161	Downregulated	TS	no	23765536		
PDCD5	-0.781976363	Downregulated	TS	yes	23807738	25436001	
TFPI2	-1.124111335	Downregulated	TS	yes	17464989	22866126	28053577
CD109	-1.207896145	Downregulated	TS	yes	27121053		
TXNIP	2.721786188	Upregulated	TS/ONC	yes	30627326	16607285	16607285
PPARGC1A	0.94644094	Upregulated/Downregulated	TS/ONC	yes	27081083	32298475	
OSR1	0.919008185	unclear	TS/ONC	yes	29029515	33005011	
PROX1	0.817739666	Upregulated	TS/ONC	yes	23505027	23291986	25684142
CCNG2	1.054985006	undetermined		yes	-	-	
NNT	0.758866565	Downregulated		yes	32509068		
ABHD5	0.745210489	unknown		yes	19211039		
PSMB9	0.719928944	unknown		no	-		
CSF3R	-0.652064847	unknown		no			
SELENBP1	-0.701653342	unknown		yes			
EIF3J	-0.866276419	unknown		yes			
PLAU	-0.953923443	Upregulated		yes	11079728		
LYVE1	-0.970015739	Downregulated		yes	24649290		
IL11	-1.170600785	UP in metastatic tumors		yes	23307318		
PLAUR	-1.267201866	unknown		yes			
HK1	-1.607007594	Up in HCC cell lines		yes	30474301		
RBP1	-1.681038223	unknown		no			
CNN1	-1.933660211	unclear		no	22681909	11920541	
GPRC5A	-2.149599232	unchanged		yes	32547082		
SNCG	-2.199169834	Upregulated		no	16596223		
IL18	-2.51525601	unknown		no			

Table S5. Roles of TIA1 in cancer.

Cancer	RNA Level in Tumoral Tissue	Protein Level in Tumoral Tissue	Function	PMID
colorectal	unchanged	down	TS	28257633
esophageal squamous cell carcinoma	-	Pos. staining in 47% patients	ONC	26958940
cervical (HeLa)			TS	19709424
thyroid cancer	down		TS	31555352
gastric cancer	unchanged	down	TS	30144499
cervical (HeLa)			TS	21284605
many cancers		downregulated		21284605

Table S6. Intensity of TIA1 staining in the LVC482 TMA.

Catalog Num	Position	Sex	Age	Organ	Pathology	Grade	Tnm	Type	Staining
LVC482	A1	M	58	Liver	Hepatocellular carcinoma	I	T2N0M0	Malignant	0
LVC482	A2	M	72	Liver	Hepatocellular carcinoma	I	T2N0M0	Malignant	1
LVC482	A3	M	46	Liver	Hepatocellular carcinoma	I	T3N1M0	Malignant	0
LVC482	A4	M	57	Liver	Hepatocellular carcinoma	I	T2N0M0	Malignant	1
LVC482	A5	M	30	Liver	Hepatocellular carcinoma	II	T2N0M0	Malignant	1
LVC482	A6	F	45	Liver	Hepatocellular carcinoma	II	T2N0M0	Malignant	1
LVC482	A7	M	47	Liver	Hepatocellular carcinoma	II	T3N0M0	Malignant	2
LVC482	A8	F	63	Liver	Hepatocellular carcinoma	II	T3N0M0	Malignant	1
LVC482	B1	M	58	Liver	Hepatocellular carcinoma	I	T2N0M0	Malignant	0
LVC482	B2	M	72	Liver	Hepatocellular carcinoma	I	T2N0M0	Malignant	0
LVC482	B3	M	46	Liver	Hepatocellular carcinoma	I	T3N1M0	Malignant	1
LVC482	B4	M	57	Liver	Hepatocellular carcinoma	I	T2N0M0	Malignant	1
LVC482	B5	M	30	Liver	Hepatocellular carcinoma	II	T2N0M0	Malignant	1
LVC482	B6	F	45	Liver	Hepatocellular carcinoma	II	T2N0M0	Malignant	1
LVC482	B7	M	47	Liver	Hepatocellular carcinoma	II	T3N0M0	Malignant	2
LVC482	B8	F	63	Liver	Hepatocellular carcinoma	II	T3N0M0	Malignant	1
LVC482	C1	M	58	Liver	Uninvolved Liver tissue of A1,B1			Normal	1
LVC482	C2	M	72	Liver	Uninvolved Liver tissue of A2,B2			Normal	1
LVC482	C3	M	46	Liver	Uninvolved Liver tissue of A3,B3			Normal	2
LVC482	C4	M	57	Liver	Uninvolved Liver tissue of A4,B4			Normal	2
LVC482	C5	M	30	Liver	Uninvolved Liver tissue of A5,B5			Normal	1
LVC482	C6	F	45	Liver	Uninvolved Liver tissue of A6,B6			Normal	1
LVC482	C7	M	47	Liver	Uninvolved Liver tissue of A7,B7			Normal	1
LVC482	C8	F	63	Liver	Uninvolved Liver tissue of A8,B8			Normal	1
LVC482	D1	M	48	Liver	Hepatocellular carcinoma	II	T2N0M0	Malignant	0
LVC482	D2	M	47	Liver	Hepatocellular carcinoma	II	T2N0M0	Malignant	0
LVC482	D3	M	68	Liver	Hepatocellular carcinoma	II	T2N0M0	Malignant	0
LVC482	D4	F	35	Liver	Hepatocellular carcinoma	II	T2N0M0	Malignant	0
LVC482	D5	M	57	Liver	Hepatocellular carcinoma	II	T2N0M0	Malignant	1
LVC482	D6	M	40	Liver	Hepatocellular carcinoma	II	T2N0M0	Malignant	0
LVC482	D7	F	60	Liver	Hepatocellular carcinoma	II	T2N0M0	Malignant	1
LVC482	D8	M	32	Liver	Hepatocellular carcinoma	III	T2N0M0	Malignant	1
LVC482	E1	M	48	Liver	Hepatocellular carcinoma	II	T2N0M0	Malignant	1
LVC482	E2	M	47	Liver	Hepatocellular carcinoma	II	T2N0M0	Malignant	0
LVC482	E3	M	68	Liver	Hepatocellular carcinoma	II	T2N0M0	Malignant	1
LVC482	E4	F	35	Liver	Hepatocellular carcinoma	II	T2N0M0	Malignant	1
LVC482	E5	M	57	Liver	Hepatocellular carcinoma	II	T2N0M0	Malignant	2
LVC482	E6	M	40	Liver	Hepatocellular carcinoma	II	T2N0M0	Malignant	0
LVC482	E7	F	60	Liver	Hepatocellular carcinoma	II	T2N0M0	Malignant	1
LVC482	E8	M	32	Liver	Hepatocellular carcinoma	III	T2N0M0	Malignant	2
LVC482	F1	M	48	Liver	Uninvolved Liver tissue of D1,E1			Normal	1
LVC482	F2	M	47	Liver	Uninvolved Liver tissue of D2,E2			Normal	1
LVC482	F3	M	68	Liver	Uninvolved Liver tissue of D3,E3			Normal	2
LVC482	F4	F	35	Liver	Uninvolved Liver tissue of D4,E4			Normal	2
LVC482	F5	M	57	Liver	Uninvolved Liver tissue of D5,E5			Normal	1
LVC482	F6	M	40	Liver	Uninvolved Liver tissue of D6,E6			Normal	1
LVC482	F7	F	60	Liver	Uninvolved Liver tissue of D7,E7			Normal	1
LVC482	F8	M	32	Liver	Uninvolved Liver tissue of D8,E8			Normal	1

Table S7. Intensity of TIA1 staining in the LV2161 TMA.

Position	No.	Age	Sex	Organ/Anatomic Site	Pathology Diagnosis	TNM	Grade	Stage	Type	Tissue ID.	Staining
A1	1	68	M	Liver	Hepatocellular carcinoma	T3N0M0	2	III	malignant	Dlv030034	0
A2	2	63	M	Liver	Hepatocellular carcinoma	T2N0M0	2	II	malignant	Dlv030030	0
A3	3	63	M	Liver	Hepatocellular carcinoma	T3N0M0	2	III	malignant	Dlv030341	1
A4	4	62	F	Liver	Hepatocellular carcinoma	T3N0M0	1	III	malignant	Dlv030570	1
A5	5	36	M	Liver	Hepatocellular carcinoma	T1N0M0	1	I	malignant	Dlv040080	0
A6	6	55	M	Liver	Hepatocellular carcinoma	T2N0M0	1	II	malignant	Dlv040126	1
A7	7	40	M	Liver	Hepatocellular carcinoma	T2N0M0	1	II	malignant	Dlv040142	1
A8	8	42	M	Liver	Hepatocellular carcinoma	T3N0M0	1	III	malignant	Dlv040241	1
A9	9	70	M	Liver	Hepatocellular carcinoma	T3N0M0	1	III	malignant	Dlv030662	2
A10	10	42	M	Liver	Hepatocellular carcinoma	T2N0M0	1	II	malignant	Dlv040438	1
A11	11	42	M	Liver	Mixed hepatocellular and cholangiocellular carcinoma	T2N0M0	-	II	malignant	Dlv040535	2
A12	12	47	M	Liver	Hepatocellular carcinoma	T3N0M0	1	III	malignant	Dlv031061	1
A13	13	67	M	Liver	Hepatocellular carcinoma	T2N0M0	1	II	malignant	Dlv031643	1
A14	14	49	M	Liver	Hepatocellular carcinoma	T2N0M0	1	II	malignant	Dlv031903	2
A15	15	56	M	Liver	Hepatocellular carcinoma	T3N0M0	1	III	malignant	Dlv050399	3
A16	16	35	M	Liver	Hepatocellular carcinoma	T3N0M0	1	III	malignant	Dlv030481	0
A17	17	62	M	Liver	Hepatocellular carcinoma	T1N0M0	1	I	malignant	Dlv030026	0
A18	18	65	M	Liver	Hepatocellular carcinoma	T2N0M0	1	II	malignant	Dlv030121	1
B1	19	40	M	Liver	Hepatocellular carcinoma	T3N0M0	1	III	malignant	Dlv030517	1
B2	20	36	M	Liver	Hepatocellular carcinoma with necrosis	T2N0M0	2	II	malignant	Dlv040395	2
B3	21	45	M	Liver	Hepatocellular carcinoma	T2N0M0	1	II	malignant	Dlv031174	1
B4	22	45	M	Liver	Hepatocellular carcinoma	T3N0M0	1	III	malignant	Dlv030355	0
B5	23	57	M	Liver	Hepatocellular carcinoma	T3N0M0	2	III	malignant	Dlv031993	0
B6	24	28	M	Liver	Hepatocellular carcinoma	T1N0M0	2	I	malignant	Dlv032029	0
B7	25	40	F	Liver	Hepatocellular carcinoma	T2N0M0	2	II	malignant	Dlv031033	0
B8	26	43	M	Liver	Hepatocellular carcinoma	T3N0M0	2	III	malignant	Dlv030116	0
B9	27	46	M	Liver	Hepatocellular carcinoma	T3N0M0	2	III	malignant	Dlv030002	0
B10	28	23	F	Liver	Hepatocellular carcinoma	T3N0M0	2	III	malignant	Dlv031151	2
B11	29	43	M	Liver	Hepatocellular carcinoma	T2N0M0	2	II	malignant	Dlv031995	1
B12	30	41	M	Liver	Hepatocellular carcinoma (fibrous tissue and blood vessel)	T2N0M0	-	II	malignant	Dlv030240	1
B13	31	41	M	Liver	Hepatocellular carcinoma	T2N0M0	2	II	malignant	Dlv030296	0
B14	32	46	M	Liver	Hepatocellular carcinoma	T3N0M0	2	III	malignant	Dlv030003	1
B15	33	42	M	Liver	Hepatocellular carcinoma	T3N0M0	2	III	malignant	Dlv040053	1
B16	34	37	M	Liver	Hepatocellular carcinoma	T3N0M0	2	III	malignant	Dlv040016	1
B17	35	55	M	Liver	Hepatocellular carcinoma (sparse)	T2N0M0	-	II	malignant	Dlv032033	0
B18	36	49	M	Liver	Hepatocellular carcinoma	T3N0M0	1	III	malignant	Dlv040015	1
C1	37	35	M	Liver	Hepatocellular carcinoma	T3N0M0	2	III	malignant	Dlv030189	0
C2	38	67	M	Liver	Hepatocellular carcinoma	T3N0M0	2	III	malignant	Dlv030335	1
C3	39	48	F	Liver	Hepatocellular carcinoma	T3N0M0	2	III	malignant	Dlv030364	1
C4	40	58	F	Liver	Hepatocellular carcinoma	T3N0M0	2	III	malignant	Dlv040762	0
C5	41	64	F	Liver	Hepatocellular carcinoma	T3N0M0	2	III	malignant	Dlv030417	0
C6	42	46	M	Liver	Hepatocellular carcinoma	T3N0M0	2	III	malignant	Dlv030488	2
C7	43	60	F	Liver	Hepatocellular carcinoma	T3N0M0	2	III	malignant	Dlv040275	0
C8	44	30	M	Liver	Hepatocellular carcinoma	T3N0M0	2	III	malignant	Dlv040766	0
C9	45	34	M	Liver	Hepatocellular carcinoma	T3N0M0	2	III	malignant	Dlv040279	0
C10	46	52	F	Liver	Hepatocellular carcinoma	T3N0M0	2	III	malignant	Dlv030497	0
C11	47	56	F	Liver	Hepatocellular carcinoma	T2N0M0	2	II	malignant	Dlv030632	0
C12	48	41	F	Liver	Hepatocellular carcinoma	T3N0M0	2	III	malignant	Dlv040208	1
C13	49	51	M	Liver	Hepatocellular carcinoma	T2N0M0	2	II	malignant	Dlv030676	1

C14	50	48	M	Liver	Hepatocellular carcinoma	T3N0M0	2	III	malignant	Dlv040086	1
C15	51	45	M	Liver	Hepatocellular carcinoma	T2N0M0	2	II	malignant	Dlv030804	0
C16	52	43	M	Liver	Hepatocellular carcinoma	T3N0M0	2	III	malignant	Dlv040123	1
C17	53	50	M	Liver	Hepatocellular carcinoma	T1N0M0	2	I	malignant	Dlv030651	0
C18	54	46	F	Liver	Hepatocellular carcinoma	T3N0M0	2	III	malignant	Dlv040191	0
D1	55	40	M	Liver	Hepatocellular carcinoma	T2N0M0	2	II	malignant	Dlv030717	0
D2	56	50	M	Liver	Hepatocellular carcinoma	T2N0M0	2	II	malignant	Dlv030787	2
D3	57	40	M	Liver	Hepatocellular carcinoma	T2N0M0	2	II	malignant	Dlv030793	0
D4	58	38	M	Liver	Hepatocellular carcinoma	T3N0M0	2	III	malignant	Dlv040315	1
D5	59	32	M	Liver	Hepatocellular carcinoma	T2N0M0	2	II	malignant	Dlv040335	1
D6	60	57	M	Liver	Hepatocellular carcinoma	T2N0M0	2	II	malignant	Dlv040392	1
D7	61	59	F	Liver	Hepatocellular carcinoma with necrosis	T2N0M0	2	II	malignant	Dlv040415	2
D8	62	43	F	Liver	Hepatocellular carcinoma with necrosis	T3N0M0	2	III	malignant	Dlv040416	2
D9	63	60	M	Liver	Hepatocellular carcinoma	T2N0M0	2	II	malignant	Dlv030495	0
D10	64	39	M	Liver	Hepatocellular carcinoma	T2N0M0	2	II	malignant	Dlv040396	0
D11	65	40	M	Liver	Hepatocellular carcinoma	T2N0M0	2	II	malignant	Dlv040224	0
D12	66	61	M	Liver	Hepatocellular carcinoma	T1N0M0	2	I	malignant	Dlv030801	1
D13	67	53	M	Liver	Hepatocellular carcinoma	T3N0M0	2	III	malignant	Dlv040163	0
D14	68	36	F	Liver	Hepatocellular carcinoma	T2N0M0	2	II	malignant	Dlv030806	2
D15	69	52	M	Liver	Hepatocellular carcinoma	T3N0M0	2	III	malignant	Dlv030844	1
D16	70	28	M	Liver	Hepatocellular carcinoma	T3N0M0	2	III	malignant	Dlv040342	0
D17	71	50	M	Liver	Hepatocellular carcinoma (sparse)	T2N0M0	-	II	malignant	Dlv040145	3
D18	72	52	M	Liver	Hepatocellular carcinoma	T2N0M0	2	II	malignant	Dlv040148	2
E1	73	56	M	Liver	Hepatocellular carcinoma	T2N0M0	2	II	malignant	Dlv040345	0
E2	74	46	M	Liver	Hepatocellular carcinoma	T3N0M0	2	III	malignant	Dlv040355	1
E3	75	45	M	Liver	Hepatocellular carcinoma	T1N0M0	2	I	malignant	Dlv030951	1
E4	76	34	M	Liver	Hepatocellular carcinoma	T3N0M0	2	III	malignant	Dlv030988	1
E5	77	56	F	Liver	Hepatocellular carcinoma	T3N0M0	2	III	malignant	Dlv040781	0
E6	78	49	M	Liver	Hepatocellular carcinoma	T2N0M0	2	II	malignant	Dlv030029	0
E7	79	58	M	Liver	Hepatocellular carcinoma	T3N0M0	2	III	malignant	Dlv030585	0
E8	80	56	M	Liver	Hepatocellular carcinoma	T3N0M0	2	III	malignant	Dlv030591	0
E9	81	48	F	Liver	Hepatocellular carcinoma	T3N0M0	2	III	malignant	Dlv030625	1
E10	82	58	M	Liver	Hepatocellular carcinoma	T3N0M0	2	III	malignant	Dlv031052	0
E11	83	41	M	Liver	Hepatocellular carcinoma	T3N0M0	2	III	malignant	Dlv031063	1
E12	84	58	M	Liver	Hepatocellular carcinoma	T2N0M0	2	II	malignant	Dlv040470	1
E13	85	18	M	Liver	Hepatocellular carcinoma	T3N0M0	2	III	malignant	Dlv040482	1
E14	86	59	F	Liver	Hepatocellular carcinoma	T2N0M0	2	II	malignant	Dlv040754	2
E15	87	38	M	Liver	Hepatocellular carcinoma	T3N0M0	2	III	malignant	Dlv040636	1
E16	88	50	M	Liver	Hepatocellular carcinoma	T3N0M0	2	III	malignant	Dlv031079	0
E17	89	35	M	Liver	Hepatocellular carcinoma	T3N0M0	2	III	malignant	Dlv031089	1
E18	90	51	F	Liver	Hepatocellular carcinoma	T3N0M0	2	III	malignant	Dlv031099	1
F1	91	58	M	Liver	Hepatocellular carcinoma	T3N1M0	2	IIIc	malignant	Dlv031127	1
F2	92	56	M	Liver	Hepatocellular carcinoma	T3N0M0	2	III	malignant	Dlv031237	1
F3	93	50	M	Liver	Hepatocellular carcinoma	T2N0M0	2	II	malignant	Dlv040487	0
F4	94	49	M	Liver	Hepatocellular carcinoma	T3N0M0	2	III	malignant	Dlv031311	1
F5	95	40	M	Liver	Hepatocellular carcinoma	T2N0M0	2	II	malignant	Dlv031325	1
F6	96	60	F	Liver	Hepatocellular carcinoma	T3N0M0	2	III	malignant	Dlv031329	0
F7	97	57	M	Liver	Hepatocellular carcinoma	T2N0M0	2	II	malignant	Dlv031459	1
F8	98	47	M	Liver	Hepatocellular carcinoma with necrosis	T2N0M0	2	II	malignant	Dlv031668	0
F9	99	51	M	Liver	Hepatocellular carcinoma with necrosis	T3N0M0	2	III	malignant	Dlv031843	1
F10	100	58	M	Liver	Hepatocellular carcinoma	T2N0M0	2	II	malignant	Dlv031833	0

F11	101	62	M	Liver	Hepatocellular carcinoma	T2N0M0	2	II	malignant	Dlv031722	2
F12	102	63	M	Liver	Hepatocellular carcinoma	T3N0M0	2	III	malignant	Dlv031882	0
F13	103	42	M	Liver	Hepatocellular carcinoma	T2N0M0	2	II	malignant	Dlv031884	0
F14	104	63	F	Liver	Hepatocellular carcinoma	T3N0M0	2	III	malignant	Dlv031919	0
F15	105	54	M	Liver	Hepatocellular carcinoma	T3N0M0	2	III	malignant	Dlv032007	1
F16	106	52	F	Liver	Hepatocellular carcinoma	T3N0M0	2	III	malignant	Dlv030362	1
F17	107	50	M	Liver	Hepatocellular carcinoma	T2N0M0	2	II	malignant	Dlv031874	1
F18	108	65	F	Liver	Hepatocellular carcinoma	T2N0M0	2	II	malignant	Dlv030012	0
G1	109	50	M	Liver	Hepatocellular carcinoma	T3N0M0	2	III	malignant	Dlv040690	1
G2	110	40	M	Liver	Hepatocellular carcinoma	T3N0M0	2	III	malignant	Dlv031035	0
G3	111	72	M	Liver	Hepatocellular carcinoma	T2N0M0	2	II	malignant	Dlv040667	0
G4	112	52	M	Liver	Hepatocellular carcinoma	T3N1M0	2	IIIc	malignant	Dlv031147	1
G5	113	56	F	Liver	Hepatocellular carcinoma	T2N0M0	2	II	malignant	Dlv040632	0
G6	114	58	M	Liver	Hepatocellular carcinoma	T3N0M0	2	III	malignant	Dlv030949	1
G7	115	55	M	Liver	Hepatocellular carcinoma	T2N0M0	2	II	malignant	Dlv040574	0
G8	116	41	M	Liver	Hepatocellular carcinoma	T3N0M0	2	III	malignant	Dlv031051	1
G9	117	56	M	Liver	Hepatocellular carcinoma	T3N0M0	2	III	malignant	Dlv040617	1
G10	118	47	M	Liver	Hepatocellular carcinoma	T2N0M0	2	II	malignant	Dlv040233	2
G11	119	47	M	Liver	Hepatocellular carcinoma (sparse)	T3N0M0	2	III	malignant	Dlv030194	0
G12	120	62	F	Liver	Hepatocellular carcinoma	T3N0M0	2	III	malignant	Dlv030172	0
G13	121	45	M	Liver	Hepatocellular carcinoma	T2N0M0	2	II	malignant	Dlv030792	0
G14	122	59	M	Liver	Hepatocellular carcinoma	T3N0M0	2	III	malignant	Dlv040314	1
G15	123	73	M	Liver	Hepatocellular carcinoma	T3N0M0	2	III	malignant	Dlv040281	0
G16	124	41	M	Liver	Hepatocellular carcinoma	T3N0M0	2	III	malignant	Dlv040276	1
G17	125	37	M	Liver	Hepatocellular carcinoma	T3N0M0	2	III	malignant	Dlv030453	0
G18	126	52	M	Liver	Hepatocellular carcinoma	T3N0M0	2	III	malignant	Dlv030580	0
H1	127	49	M	Liver	Hepatocellular carcinoma	T3N0M0	3	III	malignant	Dlv031751	1
H2	128	63	M	Liver	Hepatocellular carcinoma	T2N0M0	3	II	malignant	Dlv040125	2
H3	129	49	M	Liver	Hepatocellular carcinoma	T3N0M0	3	III	malignant	Dlv030993	1
H4	130	59	M	Liver	Hepatocellular carcinoma	T3N0M0	3	III	malignant	Dlv050014	0
H5	131	61	M	Liver	Hepatocellular carcinoma	T2N0M0	3	II	malignant	Dlv031113	1
H6	132	58	F	Liver	Hepatocellular carcinoma	T2N0M0	3	II	malignant	Dlv040741	1
H7	133	48	F	Liver	Hepatocellular carcinoma	T3N0M0	2	III	malignant	Dlv040746	1
H8	134	47	M	Liver	Hepatocellular carcinoma	T3N0M0	2	III	malignant	Dlv031741	1
H9	135	33	M	Liver	Hepatocellular carcinoma	T3N0M0	2	III	malignant	Dlv031799	2
H10	136	61	F	Liver	Hepatocellular carcinoma	T3N0M0	2	III	malignant	Dlv031276	1
H11	137	41	F	Liver	Hepatocellular carcinoma	T2N0M0	2	II	malignant	Dlv031887	1
H12	138	45	M	Liver	Hepatocellular carcinoma (fibrous tissue and nerve tissue)	T2N0M0	-	II	malignant	Dlv040775	0
H13	139	69	F	Liver	Hepatocellular carcinoma	T2N0M0	3	II	malignant	Dlv040605	0
H14	140	56	M	Liver	Hepatocellular carcinoma	T3N0M0	3	III	malignant	Dlv030111	0
H15	141	47	M	Liver	Hepatocellular carcinoma	T3N0M0	3	III	malignant	Dlv031977	0
H16	142	51	M	Liver	Hepatocellular carcinoma	T2N0M0	3	II	malignant	Dlv031996	1
H17	143	52	F	Liver	Hepatocellular carcinoma	T3N0M0	3	III	malignant	Dlv032005	2
H18	144	59	M	Liver	Hepatocellular carcinoma	T3N0M0	3	III	malignant	Dlv030124	0
I1	145	47	F	Liver	Hepatocellular carcinoma (fibrous tissue and blood vessel)	T3N0M0	-	III	malignant	Dlv030191	0
I2	146	55	F	Liver	Hepatocellular carcinoma	T3N0M0	3	III	malignant	Dlv030211	1
I3	147	35	M	Liver	Hepatocellular carcinoma	T2N0M0	3	II	malignant	Dlv030221	0
I4	148	43	M	Liver	Hepatocellular carcinoma	T3N0M0	2	III	malignant	Dlv032025	0
I5	149	65	M	Liver	Hepatocellular carcinoma	T2N0M0	3	II	malignant	Dlv040316	2
I6	150	41	M	Liver	Hepatocellular carcinoma	T2N0M0	3	II	malignant	Dlv030759	0
I7	151	41	F	Liver	Hepatocellular carcinoma with necrosis	T2N0M0	3	II	malignant	Dlv040779	1
I8	152	38	M	Liver	Hepatocellular carcinoma	T3N0M0	3	III	malignant	Dlv030419	1

I9	153	64	M	Liver	Hepatocellular carcinoma	T2N0M0	3	II	malignant	Dlv030439	0
I10	154	36	M	Liver	Spindle cell hepatocellular carcinoma	T1N0M0	3	I	malignant	Dlv030440	1
I11	155	69	F	Liver	Hepatocellular carcinoma	T3N0M0	3	III	malignant	Dlv030479	2
I12	156	48	F	Liver	Hepatocellular carcinoma	T3N0M0	3	III	malignant	Dlv040167	2
I13	157	62	M	Liver	Hepatocellular carcinoma	T3N0M0	3	III	malignant	Dlv040159	1
I14	158	70	M	Liver	Hepatocellular carcinoma	T3N0M0	3	III	malignant	Dlv030572	1
I15	159	49	F	Liver	Hepatocellular carcinoma	T2N0M0	3	II	malignant	Dlv030617	0
I16	160	68	F	Liver	Hepatocellular carcinoma	T2N0M0	3	II	malignant	Dlv030319	0
I17	161	42	M	Liver	Hepatocellular carcinoma	T2N0M0	3	II	malignant	Dlv030325	0
I18	162	37	M	Liver	Hepatocellular carcinoma	T3N0M0	3	III	malignant	Dlv040071	1
J1	163	54	M	Liver	Hepatocellular carcinoma	T3N0M0	3	III	malignant	Dlv040124	0
J2	164	56	F	Liver	Hepatocellular carcinoma	T2N0M0	3	II	malignant	Dlv031150	1
J3	165	69	M	Liver	Hepatocellular carcinoma	T3N0M0	3	III	malignant	Dlv031105	1
J4	166	47	M	Liver	Hepatocellular carcinoma	T2N0M0	3	II	malignant	Dlv040559	1
J5	167	52	F	Liver	Hepatocellular carcinoma	T3N0M0	3	III	malignant	Dlv030850	0
J6	168	34	M	Liver	Hepatocellular carcinoma	T2N0M0	3	II	malignant	Dlv030655	1
J7	169	72	F	Liver	Hepatocellular carcinoma	T3N0M0	3	III	malignant	Dlv040463	0
J8	170	43	M	Liver	Hepatocellular carcinoma	T3N0M0	3	III	malignant	Dlv031463	1
J9	171	46	F	Liver	Hepatocellular carcinoma	T3N0M0	3	III	malignant	Dlv030285	0
J10	172	53	M	Liver	Hepatocellular carcinoma	T2N0M0	3	II	malignant	Dlv040056	1
J11	173	38	M	Liver	Hepatocellular carcinoma	T2N0M0	3	II	malignant	Dlv050016	0
J12	174	56	M	Liver	Clear cell hepatocellular carcinoma	T2N0M0	3	II	malignant	Dlv040385	2
J13	175	46	M	Liver	Clear cell hepatocellular carcinoma	T3N0M0	3	III	malignant	Dlv030579	1
J14	176	63	F	Liver	Pleomorphic hepatocellular carcinoma	T3N0M0	3	III	malignant	Dlv040127	1
J15	177	56	F	Liver	Mixed hepatocellular and cholangiocellular carcinoma	T3N0M0	-	III	malignant	Dlv030374	0
J16	178	45	M	Liver	Mixed hepatocellular and cholangiocellular carcinoma	T3N0M0	-	III	malignant	Dlv040203	1
J17	179	58	F	Liver	Cholangiocellular carcinoma	T3N0M0	1	III	malignant	Dlv031868	0
J18	180	65	M	Liver	Cholangiocellular carcinoma	T2N0M0	1	II	malignant	Dlv040637	1
K1	181	71	M	Liver	Cholangiocellular carcinoma	T3N0M0	1	III	malignant	Dlv031819	0
K2	182	66	M	Liver	Cholangiocellular carcinoma	T2N0M0	1	II	malignant	Dlv031133	1
K3	183	47	M	Liver	Cholangiocellular carcinoma	T2N0M0	1	II	malignant	Dlv040629	1
K4	184	65	F	Liver	Cholangiocellular carcinoma	T2N1M0	1	III	malignant	Dlv040301	2
K5	185	58	F	Liver	Cholangiocellular carcinoma	T2N0M0	2	II	malignant	Dlv030924	1
K6	186	32	M	Liver	Cholangiocellular carcinoma	T3N0M0	2	III	malignant	Dlv031742	1
K7	187	26	M	Liver	Cholangiocellular carcinoma	T3N0M0	2	III	malignant	Dlv032017	2
K8	188	43	M	Liver	Cholangiocellular carcinoma	T2N0M0	2	II	malignant	Dlv031987	2
K9	189	76	M	Liver	Cholangiocellular carcinoma	T3N0M0	2	III	malignant	Dlv031752	1
K10	190	42	M	Liver	Cholangiocellular carcinoma	T3N0M0	2	III	malignant	Dlv040079	1
K11	191	49	M	Liver	Cholangiocellular carcinoma	T1N0M0	2	I	malignant	Dlv030778	0
K12	192	62	F	Liver	Cholangiocellular carcinoma	T2N0M0	2	II	malignant	Dlv030843	0
K13	193	56	F	Liver	Cholangiocellular carcinoma	T3N0M0	2	III	malignant	Dlv031916	1
K14	194	55	F	Liver	Cholangiocellular carcinoma	T2N0M0	2	II	malignant	Dlv031725	1
K15	195	49	M	Liver	Cholangiocellular carcinoma	T4N0M0	2	IIIb	malignant	Dlv040180	1
K16	196	50	F	Liver	Cholangiocellular carcinoma	T3N0M0	2	III	malignant	Dlv031894	0
K17	197	45	M	Liver	Cholangiocellular carcinoma	T3N0M0	2	III	malignant	Dlv040564	1
K18	198	64	M	Liver	Cholangiocellular carcinoma	T4N0M0	2	IIIb	malignant	Dlv040512	1
L1	199	60	F	Liver	Cholangiocellular carcinoma (tumoral necrosis)	T3N0M0	-	III	malignant	Dlv040784	0
L2	200	48	F	Liver	Cholangiocellular carcinoma	T2N0M0	2	II	malignant	Dlv031188	1
L3	201	59	F	Liver	Cholangiocellular carcinoma	T4N0M0	2	IIIb	malignant	Dlv040635	1

L4	202	53	F	Liver	Cholangiocellular carcinoma	T3N0M0	2	III	malignant	Dlv031964	2
L5	203	51	F	Liver	Cholangiocellular carcinoma	T3N0M0	2	III	malignant	Dlv040767	2
L6	204	57	M	Liver	Cholangiocellular carcinoma	T2N0M0	1	II	malignant	Dlv040580	1
L7	205	34	M	Liver	Cholangiocellular carcinoma	T3N0M0	2	III	malignant	Dlv040069	1
L8	206	51	F	Liver	Cholangiocellular carcinoma	T3N0M0	2	III	malignant	Dlv040099	2
L9	207	52	M	Liver	Cholangiocellular carcinoma	T2N0M0	2	II	malignant	Dlv040020	2
L10	208	44	M	Liver	Sarcomatoid hepatocellular carcinoma	T3N0M0	-	III	malignant	Dlv031004	2
L11	209	18	F	Liver	Normal hepatic tissue	-	-	-	normal	Dlv06N010	2
L12	210	16	M	Liver	Normal hepatic tissue	-	-	-	normal	Dlv06N007	3
L13	211	45	M	Liver	Normal hepatic tissue	-	-	-	normal	Dlv06N001	3
L14	212	35	F	Liver	Normal hepatic tissue	-	-	-	normal	Dlv03N009	2
L15	213	50	F	Liver	Normal hepatic tissue	-	-	-	normal	Dlv03N005	1
L16	214	35	M	Liver	Normal hepatic tissue	-	-	-	normal	Dlv05N010	3
L17	215	35	M	Liver	Normal hepatic tissue	-	-	-	normal	Dlv05N006	1
L18	216	40	M	Liver	Normal hepatic tissue	-	-	-	normal	Dlv05N014	2

Table S8. Intensity of TIA1 staining in the BC03116a TMA.

Position	No.	Age	Sex	Organ/ Anatomic Site	Pathology Diagnosis	TNM	Grade	Stage	Type	Tissue ID.	Staining Intensity
A1	1	42	M	Liver	Hepatocellular carcinoma	T3N0M0	2	IIIA	Malignant	Dlv040241	0
A2	2	55	M	Liver	Hepatocellular carcinoma	T2N0M0	1	II	Malignant	Dlv040126	0
A3	3	40	M	Liver	Hepatocellular carcinoma	T2N0M0	1	II	Malignant	Dlv040142	0
A4	4	52	M	Liver	Hepatocellular carcinoma	T2N0M0	2	II	Malignant	Dlv040148	2
A5	5	50	M	Liver	Hepatocellular carcinoma	T2N0M0	2	II	Malignant	Dlv040145	2
A6	6	50	M	Liver	Hepatocellular carcinoma	T3N0M0	*	IIIA	Malignant	Dlv040119	2
A7	7	37	F	Liver	Hepatocellular carcinoma	T3N0M0	2	IIIA	Malignant	Dlv010875	1
A8	8	40	M	Liver	Hepatocellular carcinoma	T2N0M0	2	II	Malignant	Dlv040224	0
A9	9	51	M	Liver	Hepatocellular carcinoma	T1N0M0	2	I	Malignant	Dlv040046	0
A10	10	49	M	Liver	Hepatocellular carcinoma	T3N0M0	2	IIIA	Malignant	Dlv040015	1
B1	11	44	M	Liver	Hepatocellular carcinoma	T2N0M0	2	II	Malignant	Dlv040134	0
B2	12	46	F	Liver	Hepatocellular carcinoma	T3N0M0	2	IIIA	Malignant	Dlv040191	0
B3	13	48	M	Liver	Hepatocellular carcinoma	T3N0M0	2	IIIA	Malignant	Dlv040086	1
B4	14	41	F	Liver	Hepatocellular carcinoma	T3N0M0	2	IIIA	Malignant	Dlv040208	1
B5	15	37	M	Liver	Hepatocellular carcinoma	T3N0M0	2	IIIA	Malignant	Dlv040016	2
B6	16	42	M	Liver	Hepatocellular carcinoma	T3N0M0	2	IIIA	Malignant	Dlv040053	0
B7	17	43	M	Liver	Hepatocellular carcinoma	T3N0M0	2	IIIA	Malignant	Dlv040123	2
B8	18	60	F	Liver	Hepatocellular carcinoma	T3N0M0	2	IIIA	Malignant	Dlv040275	1
B9	19	48	F	Liver	Hepatocellular carcinoma	T1N0M0	2	I	Malignant	Dlv040175	1
B10	20	27	F	Liver	Hepatocellular carcinoma	T2N0M0	2	II	Malignant	Dlv040018	1
C1	21	63	M	Liver	Hepatocellular carcinoma	T2N0M0	3	II	Malignant	Dlv040125	1
C2	22	55	F	Liver	Hepatocellular carcinoma	T3N0M0	2	IIIA	Malignant	Dlv010154	1
C3	23	67	M	Liver	Hepatocellular carcinoma	T3N0M0	2	IIIA	Malignant	Dlv010874	1
C4	24	48	F	Liver	Hepatocellular carcinoma	T3N0M0	2	IIIA	Malignant	Dlv040167	0
C5	25	53	M	Liver	Hepatocellular carcinoma	T3N0M0	2	IIIA	Malignant	Dlv040163	0
C6	26	49	M	Liver	Hepatocellular carcinoma	T3N0M0	2	IIIA	Malignant	Dlv010258	3
C7	27	66	M	Liver	Hepatocellular carcinoma	T1N0M0	2	I	Malignant	Dlv040277	1
C8	28	34	M	Liver	Hepatocellular carcinoma	T3N0M0	1	IIIA	Malignant	Dlv040279	2
C9	29	47	M	Liver	Hepatocellular carcinoma	T2N0M0	2	II	Malignant	Dlv040233	2
C10	30	41	M	Liver	Hepatocellular carcinoma	T3N0M0	2	IIIA	Malignant	Dlv040276	1
D1	31	59	M	Liver	Hepatocellular carcinoma	T3N0M0	2	IIIA	Malignant	Dlv040188	2
D2	32	38	M	Liver	Hepatocellular carcinoma	T3N0M0	2	IIIA	Malignant	Dlv040073	0
D3	33	62	M	Liver	Hepatocellular carcinoma	T3N0M0	3	IIIA	Malignant	Dlv040159	0
D4	34	54	M	Liver	Hepatocellular carcinoma	T3N0M0	3	IIIA	Malignant	Dlv040124	0
D5	35	73	M	Liver	Hepatocellular carcinoma	T3N0M0	3	IIIA	Malignant	Dlv040281	1
D6	36	70	M	Liver	Hepatocellular carcinoma	T2N0M0	3	II	Malignant	Dlv040063	1
D7	37	37	M	Liver	Hepatocellular carcinoma	T3N0M0	3	IIIA	Malignant	Dlv040071	2
D8	38	34	M	Liver	Hepatocellular carcinoma	T2N0M0	3	II	Malignant	Dlv040017	2
D9	39	63	F	Liver	Hepatocellular carcinoma	T3N0M0	3	IIIA	Malignant	Dlv040127	2
D10	40	53	M	Liver	Hepatocellular carcinoma	T2N0M0	3	II	Malignant	Dlv040056	0
E1	41	14	F	Liver	Liver tissue	-	-	-	Normal	Dlv03N001	1
E2	42	27	F	Liver	Liver tissue	-	-	-	Normal	Dlv07N025	2
E3	43	3	M	Liver	Liver tissue	-	-	-	Normal	Dlv07N021	1
E4	44	47	M	Liver	Liver tissue	-	-	-	Normal	Dlv06N028	3
E5	45	50	F	Liver	Liver tissue with fatty degeneration	-	-	-	Normal	Dlv03N005	2
E6	46	35	F	Liver	Liver tissue	-	-	-	Normal	Dlv03N009	2
E7	47	35	M	Liver	Liver tissue with fatty degeneration	-	-	-	Normal	Dlv05N006	1
E8	48	35	M	Liver	Liver tissue	-	-	-	Normal	Dlv05N010	2
E9	49	40	M	Liver	Liver tissue	-	-	-	Normal	Dlv05N013	3
E10	50	40	M	Liver	Liver tissue	-	-	-	Normal	Dlv05N014	3
F1	51	38	M	Liver	Liver tissue	-	-	-	Normal	Dlv05N015	1

F2	52	45	M	Liver	Liver tissue	-	-	-	Normal	Dlv06N001	3
F3	53	47	M	Liver	Liver tissue	-	-	-	Normal	Dlv06N004	1
F4	54	16	M	Liver	Liver tissue	-	-	-	Normal	Dlv06N007	2
F5	55	18	F	Liver	Liver tissue	-	-	-	Normal	Dlv06N010	2
F6	56	21	F	Liver	Liver tissue with fatty degeneration	-	-	-	Normal	Dlv11N001	1
F7	57	43	M	Liver	Liver tissue	-	-	-	Normal	Dlv06N019	3
F8	58	35	M	Liver	Adjacent normal liver tissue with fatty degeneration	-	-	-	NAT	Dlv031809	2
F9	59	34	M	Liver	Adjacent normal liver tissue	-	-	-	NAT	Dlv030943	2
F10	60	61	F	Liver	Adjacent normal liver tissue	-	-	-	NAT	Dlv041048	3
G1	61	57	F	Liver	Adjacent normal liver tissue	-	-	-	NAT	Dlv022779	1
G2	62	60	M	Liver	Adjacent normal liver tissue	-	-	-	NAT	Dlv030629	1
G3	63	65	F	Liver	Adjacent normal liver tissue	-	-	-	NAT	Dlv051119	2
G4	64	32	M	Liver	Adjacent normal liver tissue with fatty degeneration	-	-	-	NAT	Dlv041167	2
G5	65	31	M	Liver	Adjacent normal liver tissue with fatty degeneration	-	-	-	NAT	Dlv030945	1
G6	66	31	M	Liver	Adjacent normal liver tissue with fatty degeneration	-	-	-	NAT	Dlv031134	2
G7	67	27	M	Liver	Adjacent normal liver tissue	-	-	-	NAT	Dlv031249	2
G8	68	35	F	Liver	Adjacent normal liver tissue	-	-	-	NAT	Dlv060974	1
G9	69	25	F	Liver	Adjacent normal liver tissue with fatty degeneration	-	-	-	NAT	Dlv031716	1
G10	70	23	F	Liver	Adjacent normal liver tissue with fatty degeneration	-	-	-	NAT	Dlv031827	2

References

1. Lever, J.; Zhao, E.Y.; Grewal, J.; Jones, M.R.; Jones, S.J.M. CancerMine: a literature-mined resource for drivers, oncogenes and tumor suppressors in cancer. *Nat Methods* **2019**, *16*, 505-507, doi:10.1038/s41592-019-0422-y.
2. Cerami, E.; Gao, J.; Dogrusoz, U.; Gross, B.E.; Sumer, S.O.; Aksoy, B.A.; Jacobsen, A.; Byrne, C.J.; Heuer, M.L.; Larsson, E., et al. The cBio cancer genomics portal: an open platform for exploring multidimensional cancer genomics data. *Cancer Discov* **2012**, *2*, 401-404, doi:10.1158/2159-8290.CD-12-0095.
3. Gao, J.; Aksoy, B.A.; Dogrusoz, U.; Dresdner, G.; Gross, B.; Sumer, S.O.; Sun, Y.; Jacobsen, A.; Sinha, R.; Larsson, E., et al. Integrative analysis of complex cancer genomics and clinical profiles using the cBioPortal. *Sci Signal* **2013**, *6*, pl1, doi:10.1126/scisignal.2004088.
4. Diez-Villanueva, A.; Mallona, I.; Peinado, M.A. Wanderer, an interactive viewer to explore DNA methylation and gene expression data in human cancer. *Epigenetics Chromatin* **2015**, *8*, 22, doi:10.1186/s13072-015-0014-8.
5. Pujato, M.; Kieken, F.; Skiles, A.A.; Tapinos, N.; Fiser, A. Prediction of DNA binding motifs from 3D models of transcription factors; identifying TLX3 regulated genes. *Nucleic Acids Res* **2014**, *42*, 13500-13512, doi:10.1093/nar/gku1228.
6. Wong, N.W.; Chen, Y.; Chen, S.; Wang, X. OncomiR: an online resource for exploring pan-cancer microRNA dysregulation. *Bioinformatics* **2018**, *34*, 713-715, doi:10.1093/bioinformatics/btx627.
7. Hamada, J.; Shoda, K.; Masuda, K.; Fujita, Y.; Naruto, T.; Kohmoto, T.; Miyakami, Y.; Watanabe, M.; Kudo, Y.; Fujiwara, H., et al. Tumor-promoting function and prognostic significance of the RNA-binding protein T-cell intracellular antigen-1 in esophageal squamous cell carcinoma. *Oncotarget* **2016**, *7*, 17111-17128, doi:10.18632/oncotarget.7937.
8. Zhu, Y.; Xu, G.; Yang, Y.T.; Xu, Z.; Chen, X.; Shi, B.; Xie, D.; Lu, Z.J.; Wang, P. POSTAR2: deciphering the post-transcriptional regulatory logics. *Nucleic Acids Res* **2019**, *47*, D203-D211, doi:10.1093/nar/gky830.
9. Naboulsi, W.; Megger, D.A.; Bracht, T.; Kohl, M.; Turewicz, M.; Eisenacher, M.; Voss, D.M.; Schlaak, J.F.; Hoffmann, A.C.; Weber, F., et al. Quantitative Tissue Proteomics Analysis Reveals Versican as Potential Biomarker for Early-Stage Hepatocellular Carcinoma. *J Proteome Res* **2016**, *15*, 38-47, doi:10.1021/acs.jproteome.5b00420.
10. Xing, X.; Huang, Y.; Wang, S.; Chi, M.; Zeng, Y.; Chen, L.; Li, L.; Zeng, J.; Lin, M.; Han, X., et al. Dataset for the quantitative proteomics analysis of the primary hepatocellular carcinoma with single and multiple lesions. *Data Brief* **2015**, *5*, 226-240, doi:10.1016/j.dib.2015.08.036.
