# Supplementary material for: TIA1 Loss Exacerbates Fatty Liver Disease but Exerts a Dual Role in Hepatocarcinogenesis
Source: Cancers (Basel). 2022 Mar 27;14(7):1704. doi: 10.3390/cancers14071704 (PMC8997004; doi:10.3390/cancers14071704)
Supplement: Supplementary file 1 [file cancers-14-01704-s001.zip › cancers-1614817-supplementary materials/File S1-Supplementary Materials and Methods.pdf]

Supplementary Materials and Methods:

Western blot

Because Anti-TIA1 C20 antibody has been discontinued, we also used another anti-TIA1 antibody (G3). For detailed description of antibodies used, see table below. TIA1 antibodies G-3 and C-20 validation blots can be found below.

Validation blot for C-20 and G-3 TIA1 antibodies in HCC cell lines:

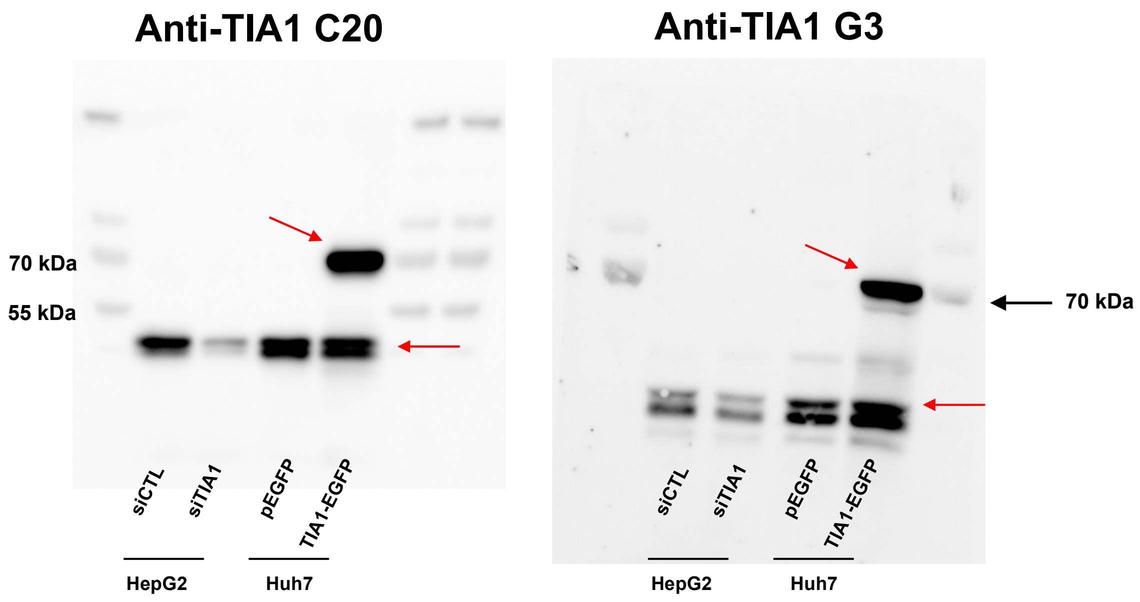

Bioinformatical analyses

Gene ontology analyses

Gene ontology (GO) enrichment analysis was performed using ShinyGO database (http://bioinformatics.sdstate.edu/go/) and PANTHER GO-SLIM (http://www.pantherdb.org/panther/goSlim.jsp)[1] on genes with p-value < 0.01 and log2FC > |0.58| (which corresponds to approx. FC > 1.5 or FC < 0.67). KEGG pathway enrichment was performed using the KEGG Mapping tool [2-4].

Identification of HCC-associated genes in the translatomic analysis

To identify genes associated to HCC and deregulated in HepG2 following TIA1 silencing, we retrieved a list of HCC-associated genes with the Metacore™ database (https://portal.genego.com/). HCC-associated genes and deregulated candidates from our translatomic were compared and represented with a venn diagram. 148 candidates were identified.

CancerMine annotation

Genes were annotated with the CancerMine tool to determine their function in cancer based on the literature[5]. Genes coming from the HepG2 siTIA1 translatomics were further re-annotated according to their role in HCC, see Table S2.

Alternative splicing analysis

Starting from the previously aligned reads, tree similar splicing analyses were performed. The stringTie/ballgown package was used to assess the differential transcript usage between conditions. The R Bioconductor package DEXSeq was used to assess the differential exon usage between the two conditions.

Chord plot

Chord plots were designed using GOplot R package (version 1.0.2).

Correlation analysis

Correlation analyses between mRNA levels in human hepatocellular carcinoma (LIHC cohort) were performed with GEPIA2 software (http://gepia2.cancer-pku.cn, accessed in June 2021) using Pearson coefficient.

Gene Expression Omnibus (GEO) datasets

Microarray/RNAseq data obtained from GEO database (https://www.ncbi.nlm.nih.gov/gds, accessed in 1 June 2021) was used to compare mRNA levels in various models of liver diseases in mice and human. The data was analyzed either by GEO2R web tool (https://www.ncbi.nlm.nih.gov/geo/geo2r). P-values were retrieved from GEO2R. For multiple available probes, the probe showing most significant differences was considered. A deregulation expression pattern of minimum 50% (0,666<FC<1.5) was considered to avoid small and biologically irrelevant alteration, as previously described [6-9]. All GEO datasets are represented in Supplementary Table 1-2.

Human protein atlas

Images of protein stainings were retrieved from the human protein atlas (https://www.proteinatlas.org/, accessed in 1 October 2021).

Interactome analysis

STRING software (http://string-db.org, accessed in 1 October 2021) (medium confidence, interaction sources: Text mining, Experiments, Databases) was used to determine interactions between genes.

Methylation data

Information on TIA1 methylation was retrieved from Wanderer (http://maplab.imppc.org/wanderer/#, accessed in 1 September 2021)[10].

miRbase

Potential miRNAs targeting TIA1 were retrieved from miRbase (http://www.mirbase.org/index.shtml, accessed in 1 September 2021)[11].

mRNA expression in the TCGA cohort

Analyses of mRNA expression in human hepatocellular carcinoma (LIHC cohort) were performed with GEPIA2 software (http://gepia2.cancer-pku.cn, accessed in 1 June 2021) using one-way ANOVA.

Mutation analysis

Mutation rate was determined using cBioPortal (https://www.cbioportal.org/, accessed in February 2021) based on HCC cohorts, where mutation data was available.

OncomiR

Data on expression and survival curves of miRNAs was retrieved from the OncomiR database[12] , (https://www.oncomir.umn.edu/omcd, accessed in 1 September 2021).

POSTAR database

TIA1 binding partners were downloaded from the CLIPdb POSTAR database (http://111.198.139.65/, accessed in 1 June 2021)[13].

Survival analyses

Survival analysis showing overall, and disease-free survival was performed using the GEPIA2 interface (http://gepia2.cancer-pku.cn/, accessed in 1 September 2021), using segregation mentioned on figures, based on gene expression in the LIHC TCGA cohort. In case of survival maps, significance is presented by bold, colored edges of respective tiles.

Transcription factor binding sites

Potential transcription factors of TIA1 were retrieved from TF2DNA database (http://www.fiserlab.org/tf2dna_db/, accessed in 1 June 2021)[14].

Additional tools

Heatmaps were created using Morpheus software (https://software.broadinstitute.org/morpheus/) with hierarchical clustering (One minus Pearson correlation, average linkage). Venn diagrams were prepared using the VennDiagram R package (version 1.6.20)

No ethical approval is required for in silico analyses performed on human samples, since the data come from publicly available, anonymized and previously approved studies.

Illustrations

Protocol illustrations were created using Servier Medical Art (https://smart.servier.com/).

Primary antibodies:

Protein Targeted	Host	Provider	Catalogue Number
TIA1 C-20	Goat	Santa Cruz (US)	sc-1751
TIA1 G-3	Mouse	Santa Cruz (US)	sc-166247
β-tubulin	Rabbit	Cell Signaling (US)	2128
G3BP1	Mouse	BD Biosciences (US)	611126

Secondary antibodies:

Protein Targeted	Host	Provider	Catalogue Number
HRP-conjugated anti-rabbit	Goat	BioRad (Switzerland)	170-6515
HRP-conjugated anti-goat	Donkey	Abcam (UK)	ab 205723
HRP-conjugated anti-mouse	Goat	BioRad (Switzerland)	170-6516
Alexa Fluor 488 anti-goat	Donkey	Abcam (UK)	ab150129
Alexa Fluor 647 anti-mouse	Donkey	Invitrogen (US)	A-31571

siRNAs:

	Provider	Catalogue Number
Human siTIA1-1	Qiagen (Basel, Switzerland)	1027417
Mouse siTIA1-1	Qiagen (Basel, Switzerland)	SI01448139
All Stars Negative Control siRNA	Qiagen (Basel, Switzerland)	1027280

Software:

OsiriX MD v.10.0.1; Quantum GX microCT software (PerkinElmer, US); ImageJ software and the Cell Counter plug-in; FlowJo v10 software (BD Biosciences, US).; StepOnePlus and QuantStudio systems (Life Technologies, Switzerland); R version 4.0.5; R Studio 1.4.1106

Mouse primers:

Gene	Forward Primer	Reverse Primer

Acta2	5′-AAAAAAAACCACGAGTAACAAATCAA-3′	5′-TCAGCGCCTCCAGTTCCT-3′
Ccn2	5′-CCTGGAGGAAAACATTAAGAAGGG-3′	5′-CTTGACAGGCTTGGCGATTT-3′
Cd36	5′-GTCTATCTACGCTGTGTTCG-3′	5′-ACAGGCTTTCCTTCTTTGC-3′
Cldn1	5′-CCTGCCCCAGTGGAAGATTT-3′	5′-AAAGATTGCGATCAGCCCCA-3′
Col1a1	5′-GCTCCTCTTAGGGGCCACT-3′	5′-CCACGTCTCACCATTGGGG-3′
Col1a2	5′-CACCCCAGCGAAGAACTCAT-3′	5′-TCTCCTCATCCAGGTACGCA-3′
Col3a1	5′-CCTGGCTCAAATGGCTCAC-3′	5′-GACCTCGTGTTCCGGGTAT-3′
Col4a1	5′-TCCGGGAGAGATTGGTTTCC-3′	5′-CTGGCCTATAAGCCCTGGT-3′
Cpt1a	5′-ATGGCAGAGGCTCACCAAGC-3′	5′-GATGAACTTCTTCTTCCAGGAGTGC-3′
Eng	5′-CCCTCTGCCCATTACCCTG-3′	5′-GTAAACGTCACCTCACCCCTT-3′
Enpp2	5'-GTACCAACTACCAAGTGGTCT G-3'	5'-GGCATTCGGGGACTCTTATTTC-3'
Epcam	5'-AACACAAGACGACGTGGACA-3'	5'-GCTCTCCGTTCACTCTCAGG-3'
Fzd4	5′-CAGCTGACAACTTTCACGCC-3′	5′-GACTGAAAGGCACATGCCAC-3′
Gpsm2	5′-ACCATTCCTTTCATGTCCGCT-3′	5′-GGCAGTCCCCTGATTTACATAGA-3′
Hnf1b	5′-AGCCAGTCGGTTTTACAGCA-3′	5′-CCCGACACTGTGATCTGCATT-3′
Il1b	5′-GACAACTGCACTACAGGC-3′	5′-CATGGAGAATATCACTTGTTGG-3′
Ncoa3	5′-GCTTCAGCAGAGGCTACAGG-3′	5′-TGGGCATTAAAGAAAGCCTGC-3′
Pcna	5′-CCTGTGCAAAGAATGGGGTG-3′	5′-TCTCTATGGTTACCGCCTCC-3′
Pik3c3	5′-CCTGGACATCAACGTGCAG-3′	5′-TGTCTCTTGGTATAGCCCAGAAA-3′
Pik3r1	5′-ACACCACGGTTTGGACTATGG-3′	5′-GGCTACAGTAGTGGGCTTGG-3′
Pparg	5′-CTCACAATGCCATCAGGT-3′	5′-GCTGGTCGATATCACTGG-3′
Ppia	5′-CAAATGCTGGACCAAACACAA-3′	5′-GCCATCCAGCCATTCAGTCT-3′
Ptch1	5′-GCCTTCGCTGTGGGATTAAAG-3′	5′-CTTCTCCTATCTTCTGACGGGT-3′
Rps9	5′-GACCAGGAGCTAAAGTTGATTGGA-3′	5′-TCTTGGCCAGGGTAAACTTGA-3′
Spp1	5'-AGCTCAGAGGAGAAGAAGCTTTA-3'	5'-CTTCTGAGATGGGTCAGGCA-3'
Srp72	5′-CACCCAGCAGACAGACAAACTG-3′	5′-GCACTCATCGTAGCGTTCCA-3′
Tbl1xr1	5′-TCTCATTCTGCGTTTACCTTTGG-3′	5′-GACAGAGACTCGATGGGTCG-3′
Tgfb	5′-GCCTGAGTGGCTGTCTTTTGA-3′	5′-GCTGAATCGAAAGCCCTGTATT-3′
Tia1	5′-CGAGCAGTAGTACCGTTGTCA-3′	5′-TCCAAATGGTGCAAACGCTG-3′
Ulk1	5'-AACATCGTGGCGCTGTATGA-3'	5'-TGCGCATAGTGTGCAGGTAG-3'
Il6	5′-AGTTGCCTTCTTGGGACTGAT-3′	5′-TCCACGATTTCCCAGAGAAC-3′
Bcl2	5′-TCGCAGAGATGTCCAGTCAG-3′	5′-ATCTCCCTGTTGACGCTCTC-3′
Tnfa	5′-AGGCTGCCCCGACTACGT-3′	5′-GACTTTCTCCTGGTATGAGATAGCAAA-3'
Mki67	5′-TGGTCACCATCAAGCGGAG-3′	5′-AGGCAGCTGGATACGAATGT-3′
Mcl1	5′-GCTCCGGAAACTGGACATTA-3′	5′-CCCGTTTCGTCCTTACAAGA-3′
Cdkn1a	5′-ACAAGAGGCCCAGTACTTCC-3′	5′-AGAAATCTGTCAGGCTGGTCT-3′
Cdkn1b	5′-CGAGTTCTACTACAGGCCCC-3′	5′-GACGAGTCAGGCATTTGGTC-3′
Cdc25a	5′-ACAGCAGTCTACAGAGAATGG G-3′	5′-GATGAGGTGAAAGGTGTCTTGG-3′
Actb	5′CTAAGGCCAACCGTGAAAAGAT-3′	5′-CACAGCCTGGATGGCTACGT-3′

Human Primers:

Gene	Forward Primer	Reverse Primer
ACTB	5′-AGCACTGTG TTG GCG TAC AG	CTC TTC CAG CCT TCC TTC CT
CD36	5′AGTCACTGCGACTGATTAATGGT-3′	5′CTGCAATACCTGGCTTTTCTCA-3′
CPT1A	5′-TCTGCCTTTACGTGGTGTCTAAATAT-3′	5′TCCAAGGCTCAGATAAAACTTCCT-3′
FASN	5′ACACCCAAGGCCAAGTACCA-3′	5′TAGGCGCCACCCGTCTT-3′
MALAT1	5′GCTCTGTGGTGTGGGATTGA-3′	5′GTGGCAAAATGGCGGACTTT-3′
miR-487a-3p	5'-GACGAATCATACAGGGACATCC-3'	5'-GAGGTATTCGCACCAGAGGA-3'
NEAT1	5′GTGGCTGTTGGAGTCGGTAT-3′	5′TAACAAACCACGGTCCATGA-3′
PPIA	5′-ATGGTCAACCCCACCGTGT-3′	5′-TCTGCTGTCTTTGGGACCTTGTC-3′

TIA1	5′-ACCCACTG TTGAATGGCGT-3′	5′-GGCAACAGGAAAGTCTAAGGGAT-3′
ARID2	5′-CAGTGTGTCGGATTATCTGCG-3′	5′-GCATGACGTGCTTGCTTTCATT-3′
CDH1	5′-TGAGTGTCCCCCGGTATCTTC-3′	5′-CAGTATCAGCCGCTTTCAGATTTT-3′
S100A11	5′-GCTGTCTTCCAGAAGTATGC-3′	5′-GACCATCACTGTTGGTGTC-3′
S100A6	GGG AGG GTG ACA AGC ACA C	5′-AGCTTCGAGCCAATGGTGAG-3′
BIRC7	5′ GGTGAGGTGCTTCTTCTGCT-3′	5′-GGAGTGAGTCTCCTGCACAC-3′

References

1. Mi, H.; Ebert, D.; Muruganujan, A.; Mills, C.; Albou, L.P.; Mushayamaha, T.; Thomas, P.D. PANTHER version 16: a revised family classification, tree-based classification tool, enhancer regions and extensive API. *Nucleic Acids Res* **2021**, *49*, D394-D403, doi:10.1093/nar/gkaa1106.
2. Kanehisa, M.; Goto, S. KEGG: kyoto encyclopedia of genes and genomes. *Nucleic Acids Res* **2000**, *28*, 27-30, doi:10.1093/nar/28.1.27.
3. Kanehisa, M. Toward understanding the origin and evolution of cellular organisms. *Protein Sci* **2019**, *28*, 1947-1951, doi:10.1002/pro.3715.
4. Kanehisa, M.; Furumichi, M.; Sato, Y.; Ishiguro-Watanabe, M.; Tanabe, M. KEGG: integrating viruses and cellular organisms. *Nucleic Acids Res* **2021**, *49*, D545-D551, doi:10.1093/nar/gkaa970.
5. Lever, J.; Zhao, E.Y.; Grewal, J.; Jones, M.R.; Jones, S.J.M. CancerMine: a literature-mined resource for drivers, oncogenes and tumor suppressors in cancer. *Nat Methods* **2019**, *16*, 505-507, doi:10.1038/s41592-019-0422-y.
6. Ibraim, I.C.; Parise, M.T.D.; Parise, D.; Sfeir, M.Z.T.; de Paula Castro, T.L.; Wattam, A.R.; Ghosh, P.; Barh, D.; Souza, E.M.; Goes-Neto, A., et al. Transcriptome profile of Corynebacterium pseudotuberculosis in response to iron limitation. *BMC Genomics* **2019**, *20*, 663, doi:10.1186/s12864-019-6018-1.
7. Della Bella, E.; Buetti-Dinh, A.; Licandro, G.; Ahmad, P.; Basoli, V.; Alini, M.; Stoddart, M.J. Dexamethasone Induces Changes in Osteogenic Differentiation of Human Mesenchymal Stromal Cells via SOX9 and PPARG, but Not RUNX2. *Int J Mol Sci* **2021**, *22*, doi:10.3390/ijms22094785.
8. Gjorgjieva, M.; Sobolewski, C.; Ay, A.S.; Abegg, D.; Correia de Sousa, M.; Portius, D.; Berthou, F.; Fournier, M.; Maeder, C.; Rantakari, P., et al. Genetic Ablation of MiR-22 Fosters Diet-Induced Obesity and NAFLD Development. *J Pers Med* **2020**, *10*, doi:10.3390/jpm10040170.
9. Sobolewski, C.; Abegg, D.; Berthou, F.; Dolicka, D.; Calo, N.; Sempoux, C.; Fournier, M.; Maeder, C.; Ay, A.S.; Clavien, P.A., et al. S100A11/ANXA2 belongs to a tumour suppressor/oncogene network deregulated early with steatosis and involved in inflammation and hepatocellular carcinoma development. *Gut* **2020**, *69*, 1841-1854, doi:10.1136/gutjnl-2019-319019.
10. Diez-Villanueva, A.; Mallona, I.; Peinado, M.A. Wanderer, an interactive viewer to explore DNA methylation and gene expression data in human cancer. *Epigenetics Chromatin* **2015**, *8*, 22, doi:10.1186/s13072-015-0014-8.
11. Kozomara, A.; Birgaoanu, M.; Griffiths-Jones, S. miRBase: from microRNA sequences to function. *Nucleic Acids Res* **2019**, *47*, D155-D162, doi:10.1093/nar/gky1141.
12. Wong, N.W.; Chen, Y.; Chen, S.; Wang, X. OncomiR: an online resource for exploring pan-cancer microRNA dysregulation. *Bioinformatics* **2018**, *34*, 713-715, doi:10.1093/bioinformatics/btx627.
13. Zhu, Y.; Xu, G.; Yang, Y.T.; Xu, Z.; Chen, X.; Shi, B.; Xie, D.; Lu, Z.J.; Wang, P. POSTAR2: deciphering the post-transcriptional regulatory logics. *Nucleic Acids Res* **2019**, *47*, D203-D211, doi:10.1093/nar/gky830.
14. Pujato, M.; Kieken, F.; Skiles, A.A.; Tapinos, N.; Fiser, A. Prediction of DNA binding motifs from 3D models of transcription factors; identifying TLX3 regulated genes. *Nucleic Acids Res* **2014**, *42*, 13500-13512, doi:10.1093/nar/gku1228.
