# Supplementary material for: TIA1 Loss Exacerbates Fatty Liver Disease but Exerts a Dual Role in Hepatocarcinogenesis
Source: Cancers (Basel). 2022 Mar 27;14(7):1704. doi: 10.3390/cancers14071704 (PMC8997004; doi:10.3390/cancers14071704)
Supplement: Supplementary file 1 [file cancers-14-01704-s001.zip › cancers-1614817-supplementary materials/File S2-Original images Western Blots_R2.pdf]

Validation TIA1 antibodies

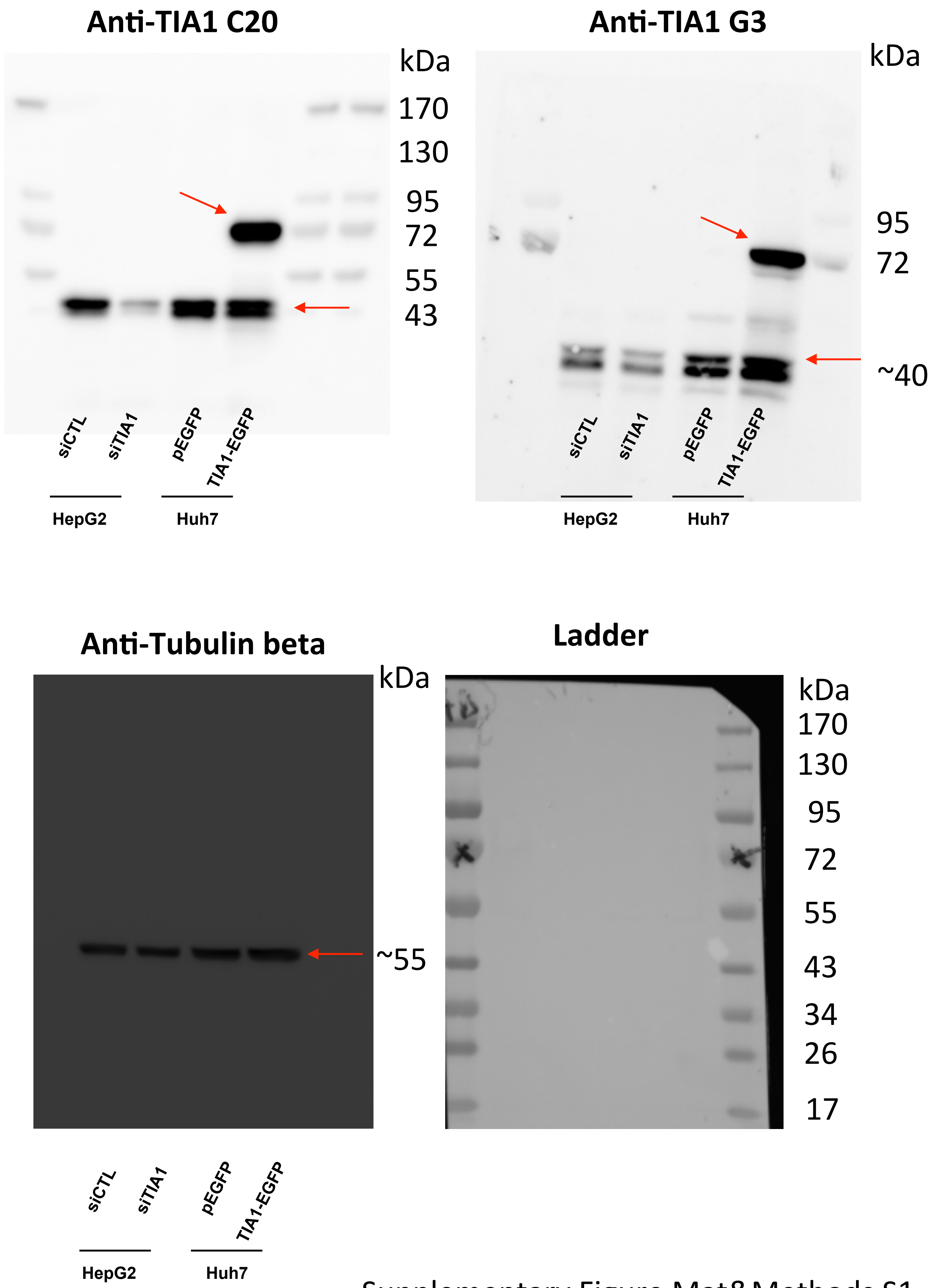

Supplementary Figure Mat&Methods S1

TIA1 expression in different cell lines

Anti-TIA1-G3

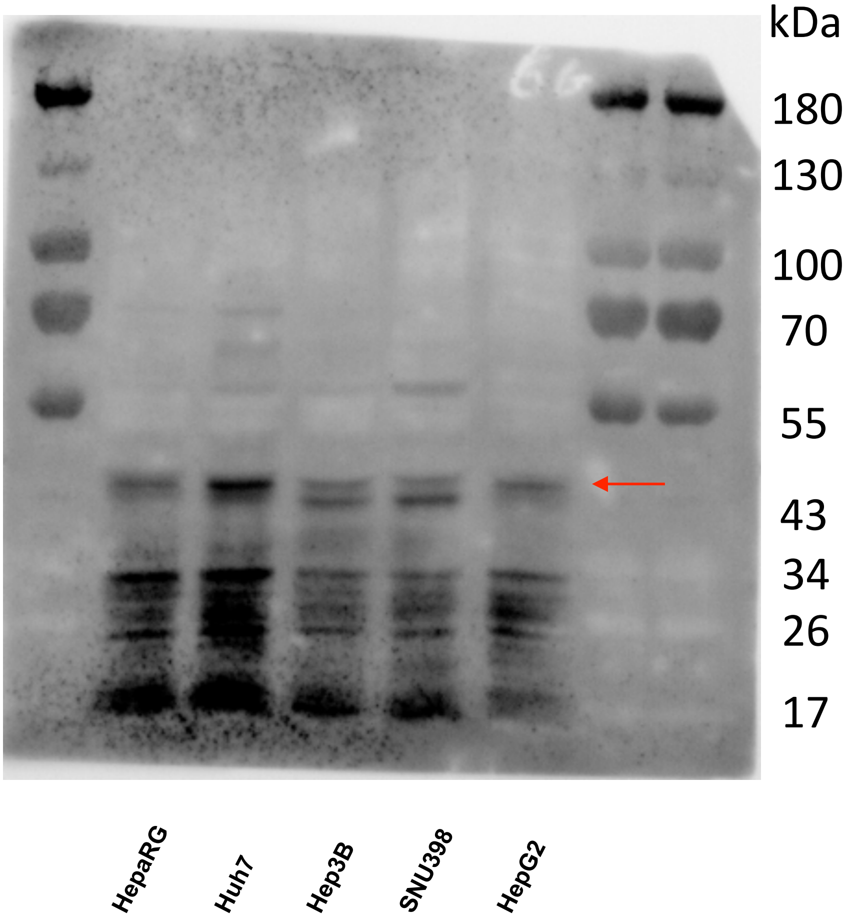

Anti-Tubulin beta

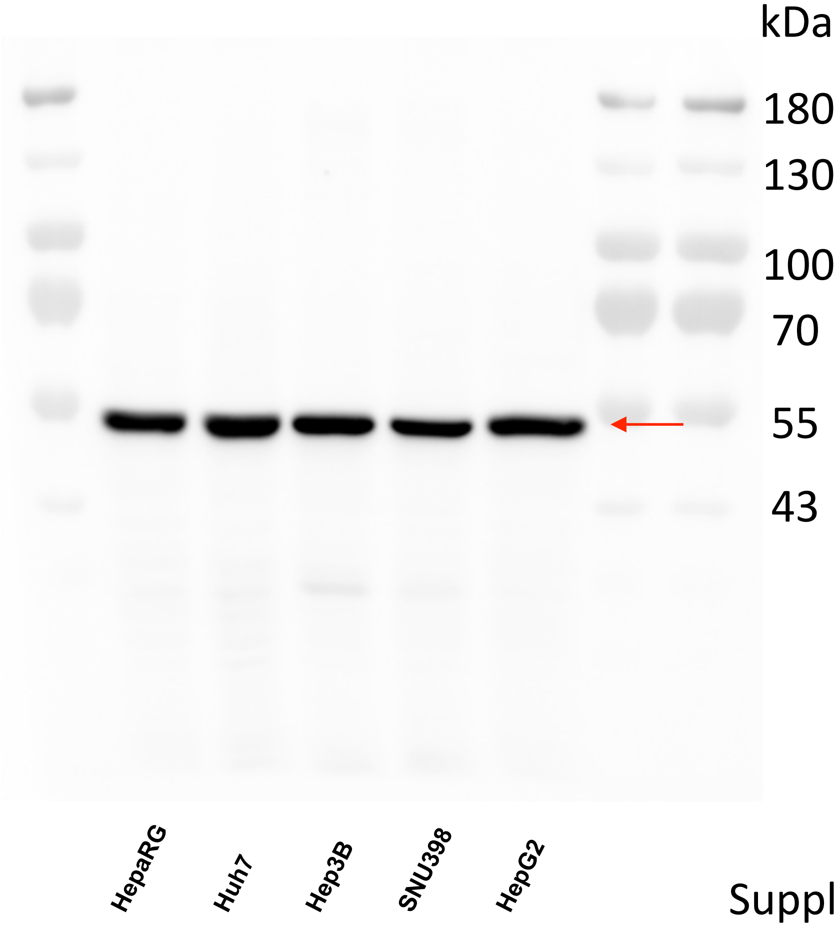

Supplementary Figure S1G

siTIA1 in Hepa1-6 cells

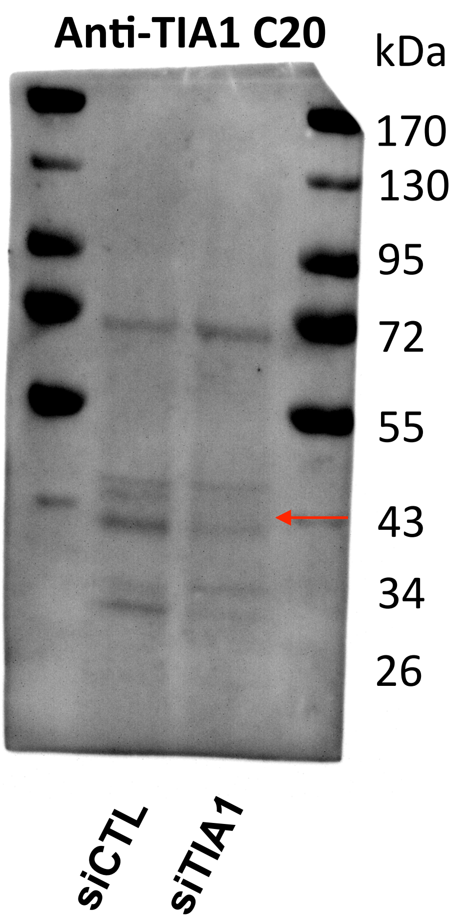

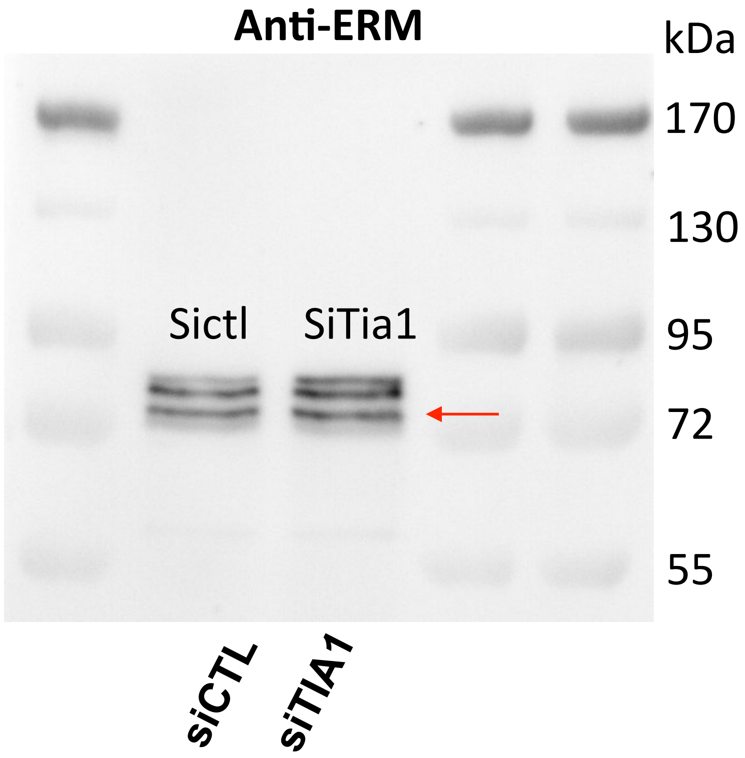

Supplementary Figure S1I

MCD + AAV8 shCTL/TIA1

Anti-TIA1-C20

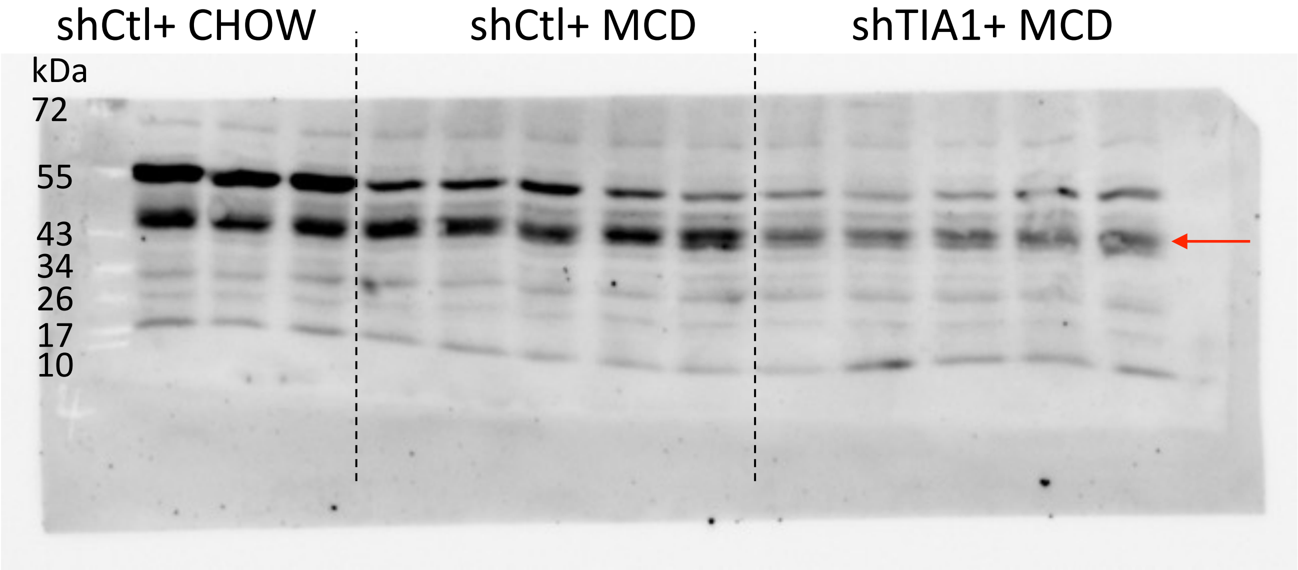

Anti-Tubulin beta

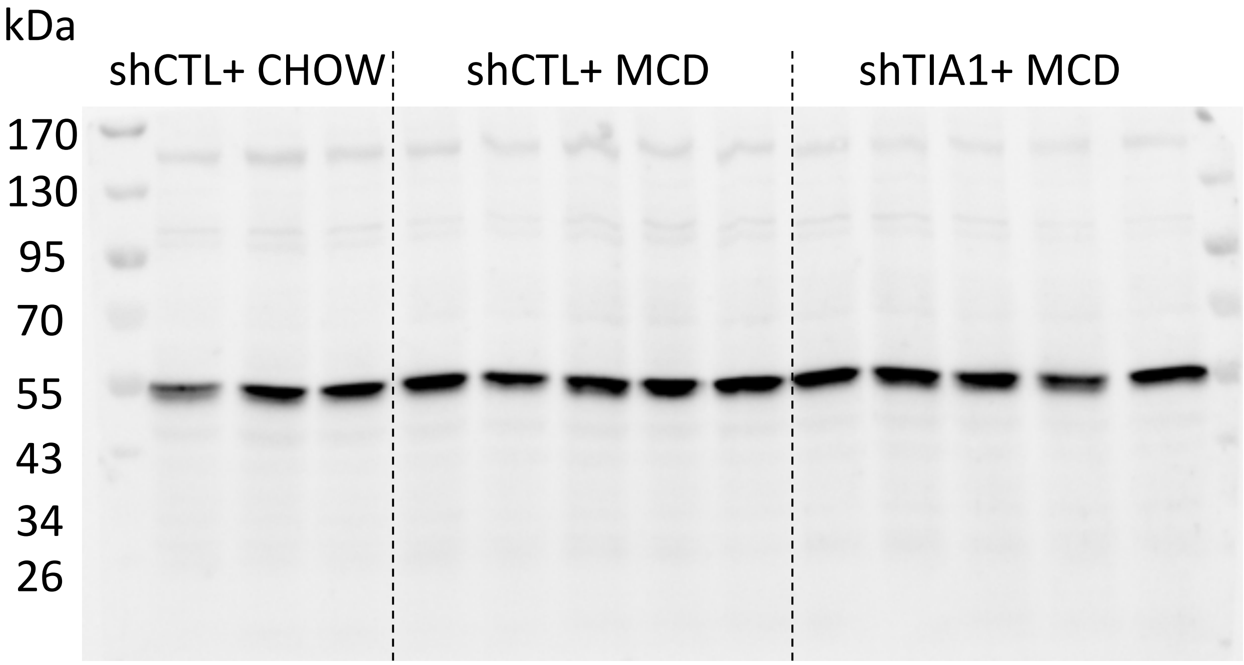

Figure 3

LPTENKO + AAV8 shCTL/TIA1

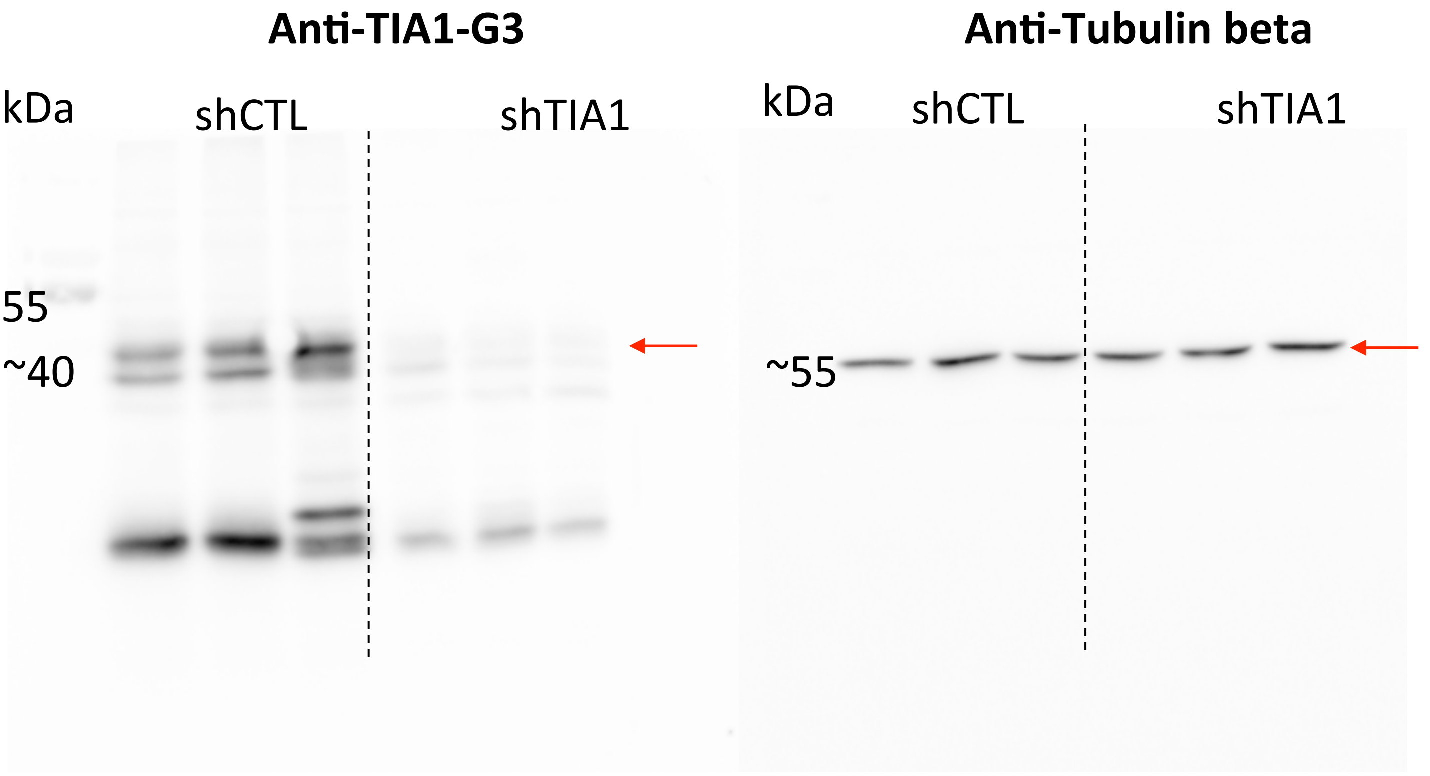

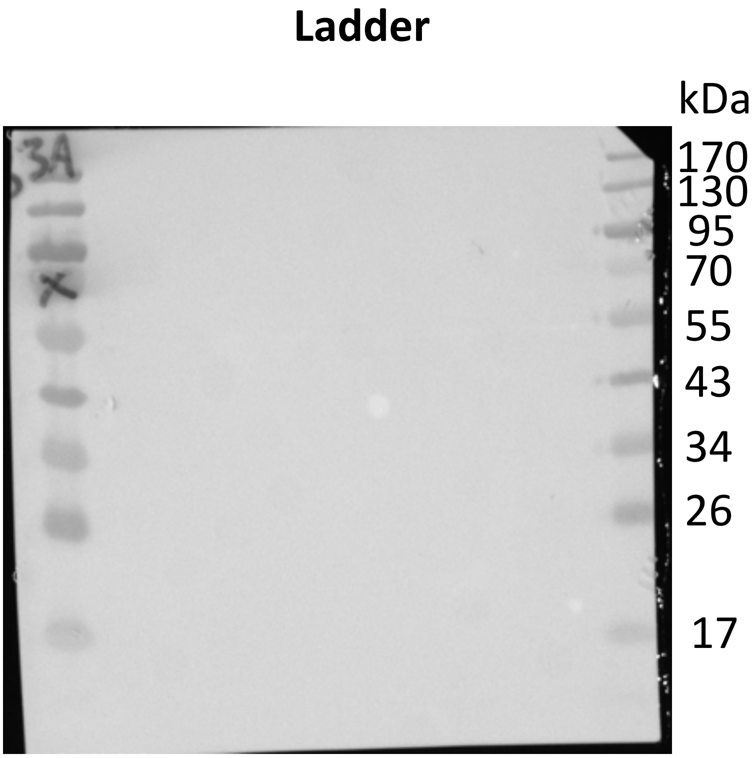

Figure 4
