# Supplementary material for: TIA1 Loss Exacerbates Fatty Liver Disease but Exerts a Dual Role in Hepatocarcinogenesis
Source: Cancers (Basel). 2022 Mar 27;14(7):1704. doi: 10.3390/cancers14071704 (PMC8997004; doi:10.3390/cancers14071704)
Supplement: Supplementary file 1 [file cancers-14-01704-s001.zip › cancers-1614817-supplementary materials/Table S3.pdf]

ensembl_id	logFC	logCPM	F	PValue	FDR	Symbol	Description
ENSG00000142227	-1,8552436	3,74296163	118,190875	1,42E-06	0,01395357	EMP3	epithelial membrane protein 3 [Source:HGNC Symbol;Acc:HGNC:3335]
ENSG00000165443	-1,6641539	5,33600408	110,296	1,91E-06	0,01395357	PHYHIPL	phytanoyl-CoA 2-hydroxylase interacting protein like [Source:HGNC Symbol;Acc:HGNC:29378]
ENSG00000005469	1,55343852	3,48542585	92,8196741	3,99E-06	0,01398238	CROT	carnitine O-octanoyltransferase [Source:HGNC Symbol;Acc:HGNC:2366]
ENSG00000052795	1,41060945	3,51701979	71,0803148	1,22E-05	0,01398238	FNIP2	folliculin interacting protein 2 [Source:HGNC Symbol;Acc:HGNC:29280]
ENSG00000101166	-1,3280138	4,23013074	68,0650047	1,46E-05	0,01398238	PRELID3B	PRELI domain containing 3B [Source:HGNC Symbol;Acc:HGNC:15892]
ENSG00000106541	-1,6222828	4,10173693	84,3515443	5,98E-06	0,01398238	AGR2	anterior gradient 2, protein disulphide isomerase family member [Source:HGNC Symbol;Acc:HGNC:328]
ENSG00000114115	-1,6810382	3,03776701	79,8288468	7,54E-06	0,01398238	RBP1	retinol binding protein 1 [Source:HGNC Symbol;Acc:HGNC:9919]
ENSG00000131238	-1,576772	7,92842637	92,1916045	4,11E-06	0,01398238	PPT1	palmitoyl-protein thioesterase 1 [Source:HGNC Symbol;Acc:HGNC:9325]
ENSG00000134240	2,61163819	0,72979159	67,2991165	1,53E-05	0,01398238	HMGCS2	3-hydroxy-3-methylglutaryl-CoA synthase 2 [Source:HGNC Symbol;Acc:HGNC:5008]
ENSG00000136305	-1,2633824	6,69205382	79,0998884	7,83E-06	0,01398238	CIDEB	cell death inducing DFFA like effector b [Source:HGNC Symbol;Acc:HGNC:1977]
ENSG00000143365	1,72615681	2,5755142	70,9159604	1,23E-05	0,01398238	RORC	RAR related orphan receptor C [Source:HGNC Symbol;Acc:HGNC:10260]
ENSG00000158887	1,93473878	4,81336079	68,8203673	1,40E-05	0,01398238	MPZ	myelin protein zero [Source:HGNC Symbol;Acc:HGNC:7225]
ENSG00000162078	1,39885739	3,85913897	67,3005353	1,53E-05	0,01398238	ZG16B	zymogen granule protein 16B [Source:HGNC Symbol;Acc:HGNC:30456]
ENSG00000162407	-1,5534949	5,70894974	75,4076741	9,56E-06	0,01398238	PLPP3	phospholipid phosphatase 3 [Source:HGNC Symbol;Acc:HGNC:9229]
ENSG00000163501	1,67553388	1,98750869	67,2960135	1,53E-05	0,01398238	IHH	Indian hedgehog signaling molecule [Source:HGNC Symbol;Acc:HGNC:5956]
ENSG00000170502	-1,2979206	5,61062111	71,0013741	1,23E-05	0,01398238	NUDT9	nudix hydrolase 9 [Source:HGNC Symbol;Acc:HGNC:8056]
ENSG00000118939	-1,3554131	2,74060243	64,1976568	1,86E-05	0,01433942	UCHL3	ubiquitin C-terminal hydrolase L3 [Source:HGNC Symbol;Acc:HGNC:12515]
ENSG00000212901	-1,6668078	2,47756332	64,1410593	1,86E-05	0,01433942	KRTAP3-1	keratin associated protein 3-1 [Source:HGNC Symbol;Acc:HGNC:16778]
ENSG00000265972	2,72178619	4,99050871	64,2775009	1,85E-05	0,01433942	TXNIP	thioredoxin interacting protein [Source:HGNC Symbol;Acc:HGNC:16952]
ENSG00000115128	-1,5232641	6,43879179	62,2626519	2,11E-05	0,01437359	SF3B6	splicing factor 3b subunit 6 [Source:HGNC Symbol;Acc:HGNC:30096]
ENSG00000135926	-1,1621618	7,70754805	62,9101907	2,02E-05	0,01437359	TMBIM1	transmembrane BAX inhibitor motif containing 1 [Source:HGNC Symbol;Acc:HGNC:23410]
ENSG00000187288	-1,6056191	2,81355348	61,1724113	2,26E-05	0,01437359	CIDEC	cell death inducing DFFA like effector c [Source:HGNC Symbol;Acc:HGNC:24229]
ENSG00000197705	1,21425912	5,08318292	61,3120785	2,24E-05	0,01437359	KLHL14	kelch like family member 14 [Source:HGNC Symbol;Acc:HGNC:29266]
ENSG00000101928	-1,2036249	3,07099856	56,9761381	3,02E-05	0,01463019	MOSPD1	motile sperm domain containing 1 [Source:HGNC Symbol;Acc:HGNC:25235]
ENSG00000113369	1,5047935	4,20210422	59,0256481	2,62E-05	0,01463019	ARRDC3	arrestin domain containing 3 [Source:HGNC Symbol;Acc:HGNC:29263]
ENSG00000116001	-1,2359645	5,17336566	57,690039	2,87E-05	0,01463019	TIA1	TIA1 cytotoxic granule associated RNA binding protein [Source:HGNC Symbol;Acc:HGNC:11802]
ENSG00000134333	-1,0157497	10,4092719	56,3902413	3,15E-05	0,01463019	LDHA	lactate dehydrogenase A [Source:HGNC Symbol;Acc:HGNC:6535]
ENSG00000134851	-1,3542066	6,0197412	56,1357846	3,20E-05	0,01463019	TMEM165	transmembrane protein 165 [Source:HGNC Symbol;Acc:HGNC:30760]
ENSG00000136881	2,06330024	4,09990021	56,3475274	3,16E-05	0,01463019	BAAT	bile acid-CoA:amino acid N-acyltransferase [Source:HGNC Symbol;Acc:HGNC:932]
ENSG00000137393	1,8306476	2,0530434	58,6762168	2,68E-05	0,01463019	RNF144B	ring finger protein 144B [Source:HGNC Symbol;Acc:HGNC:21578]
ENSG00000163191	-1,2945718	4,28107142	56,3139203	3,16E-05	0,01463019	S100A11	S100 calcium binding protein A11 [Source:HGNC Symbol;Acc:HGNC:10488]
ENSG00000197461	1,86629151	1,41525776	57,4918582	2,91E-05	0,01463019	PDGFA	platelet derived growth factor subunit A [Source:HGNC Symbol;Acc:HGNC:8799]
ENSG00000143418	-1,1894358	7,6360349	55,6372863	3,32E-05	0,01470552	CERS2	ceramide synthase 2 [Source:HGNC Symbol;Acc:HGNC:14076]
ENSG00000086696	1,07027594	6,14762091	52,7795932	4,10E-05	0,01666529	HSD17B2	hydroxysteroid 17-beta dehydrogenase 2 [Source:HGNC Symbol;Acc:HGNC:5211]
ENSG00000100605	-1,1339206	4,99943591	52,4846226	4,20E-05	0,01666529	ITPK1	inositol-tetrakisphosphate 1-kinase [Source:HGNC Symbol;Acc:HGNC:6177]
ENSG00000119326	-1,1214759	6,42409848	52,4094315	4,22E-05	0,01666529	CTNNAL1	catenin alpha like 1 [Source:HGNC Symbol;Acc:HGNC:2512]
ENSG00000198681	3,60905476	-1,0857313	53,219763	3,97E-05	0,01666529	MAGEA1	MAGE family member A1 [Source:HGNC Symbol;Acc:HGNC:6796]
ENSG00000129654	1,74621821	1,30100978	50,5162973	4,89E-05	0,0187866	FOXJ1	forkhead box J1 [Source:HGNC Symbol;Acc:HGNC:3816]
ENSG00000139278	-1,1599443	2,97269857	50,1606638	5,03E-05	0,0188255	GLIPR1	GLI pathogenesis related 1 [Source:HGNC Symbol;Acc:HGNC:17001]
ENSG00000117616	1,62140525	3,03901306	47,9071412	6,03E-05	0,02001263	RSRP1	arginine and serine rich protein 1 [Source:HGNC Symbol;Acc:HGNC:25234]
ENSG00000149591	-1,0690602	6,95911885	48,0471271	5,96E-05	0,02001263	TAGLN	transgelin [Source:HGNC Symbol;Acc:HGNC:11553]
ENSG00000154856	-2,5936523	-0,0863955	48,3354061	5,82E-05	0,02001263	APCDD1	APC down-regulated 1 [Source:HGNC Symbol;Acc:HGNC:15718]
ENSG00000155100	-1,3799246	2,27197574	48,8184737	5,60E-05	0,02001263	OTUD6B	OTU deubiquitinase 6B [Source:HGNC Symbol;Acc:HGNC:24281]

ENSG00000174804	1,10571968	5,28106047	48,2112807	5,88E-05	0,02001263	FZD4	frizzled class receptor 4 [Source:HGNC Symbol;Acc:HGNC:4042]
ENSG00000116885	1,90647936	1,25003297	47,5832324	6,19E-05	0,02009794	OSCP1	organic solute carrier partner 1 [Source:HGNC Symbol;Acc:HGNC:29971]
ENSG00000077782	0,98795257	7,75543718	46,6076245	6,72E-05	0,02132941	FGFR1	fibroblast growth factor receptor 1 [Source:HGNC Symbol;Acc:HGNC:3688]
ENSG00000102003	1,6960066	3,17887245	45,9934627	7,07E-05	0,0219903	SYP	synaptophysin [Source:HGNC Symbol;Acc:HGNC:11506]
ENSG00000169914	1,14713906	2,61950921	45,0197269	7,69E-05	0,02207138	OTUD3	OTU deubiquitinase 3 [Source:HGNC Symbol;Acc:HGNC:29038]
ENSG00000172071	-0,9986656	4,44932728	45,5586787	7,34E-05	0,02207138	EIF2AK3	eukaryotic translation initiation factor 2 alpha kinase 3 [Source:HGNC Symbol;Acc:HGNC:3255]
ENSG00000184012	1,21656218	3,0063669	45,4619703	7,40E-05	0,02207138	TMPRSS2	transmembrane serine protease 2 [Source:HGNC Symbol;Acc:HGNC:11876]
ENSG00000257103	-1,0052862	4,934818	45,0006007	7,71E-05	0,02207138	LSM14A	LSM14A mRNA processing body assembly factor [Source:HGNC Symbol;Acc:HGNC:24489]
ENSG00000168077	0,92704886	5,78161776	44,6535486	7,94E-05	0,02231093	SCARA3	scavenger receptor class A member 3 [Source:HGNC Symbol;Acc:HGNC:19000]
ENSG00000120137	-1,0438343	4,87371258	43,46294	8,82E-05	0,02431782	PANK3	pantothenate kinase 3 [Source:HGNC Symbol;Acc:HGNC:19365]
ENSG00000023734	-0,9488577	7,06583096	42,0335348	0,00010042	0,0243956	STRAP	serine/threonine kinase receptor associated protein [Source:HGNC Symbol;Acc:HGNC:30796]
ENSG00000068903	-1,3867823	4,65681503	41,7152592	0,00010341	0,0243956	SIRT2	sirtuin 2 [Source:HGNC Symbol;Acc:HGNC:10886]
ENSG00000096080	-1,0044989	6,57828219	43,1425645	9,08E-05	0,0243956	MRPS18A	mitochondrial ribosomal protein S18A [Source:HGNC Symbol;Acc:HGNC:14515]
ENSG00000105697	-1,0701233	5,94350295	42,7213219	9,43E-05	0,0243956	HAMP	hepcidin antimicrobial peptide [Source:HGNC Symbol;Acc:HGNC:15598]
ENSG00000151062	-1,0805726	4,95525898	42,2251148	9,87E-05	0,0243956	CACNA2D4	calcium voltage-gated channel auxiliary subunit alpha2delta 4 [Source:HGNC Symbol;Acc:HGNC:20202]
ENSG00000163347	1,20623562	7,00795824	42,6005443	9,54E-05	0,0243956	CLDN1	claudin 1 [Source:HGNC Symbol;Acc:HGNC:2032]
ENSG00000168216	-1,159678	2,92526092	42,7174864	9,43E-05	0,0243956	LMBRD1	LMBR1 domain containing 1 [Source:HGNC Symbol;Acc:HGNC:23038]
ENSG00000188015	-1,5461186	1,86330288	41,7026851	0,00010353	0,0243956	S100A3	S100 calcium binding protein A3 [Source:HGNC Symbol;Acc:HGNC:10493]
ENSG00000244062	1,19008772	4,18488609	42,2714314	9,83E-05	0,0243956	AC080128.1	acyl-CoA synthetase long-chain family member 3 (ACSL3) pseudogene
ENSG00000102265	-1,1328521	7,3792012	41,4283594	0,0001062	0,02462719	TIMP1	TIMP metallopeptidase inhibitor 1 [Source:HGNC Symbol;Acc:HGNC:11820]
ENSG00000086619	-1,5599819	1,50771646	41,1266386	0,00010923	0,02493459	ERO1B	endoplasmic reticulum oxidoreductase 1 beta [Source:HGNC Symbol;Acc:HGNC:14355]
ENSG00000167695	-0,896251	6,34785411	40,8488645	0,00011212	0,02519948	TLCD3A	TLC domain containing 3A [Source:HGNC Symbol;Acc:HGNC:29646]
ENSG00000130751	-2,1675831	0,32741409	40,6304444	0,00011445	0,02533434	NPAS1	neuronal PAS domain protein 1 [Source:HGNC Symbol;Acc:HGNC:7894]
ENSG00000052344	1,25773006	4,29606114	39,6085286	0,00012619	0,02617598	PRSS8	serine protease 8 [Source:HGNC Symbol;Acc:HGNC:9491]
ENSG00000121068	0,97074705	4,56972751	39,5598683	0,00012679	0,02617598	TBX2	T-box transcription factor 2 [Source:HGNC Symbol;Acc:HGNC:11597]
ENSG00000148702	4,36627648	-1,2963664	39,8537517	0,00012325	0,02617598	HABP2	hyaluronan binding protein 2 [Source:HGNC Symbol;Acc:HGNC:4798]
ENSG00000196557	1,43167374	1,9279543	39,4682069	0,00012792	0,02617598	CACNA1H	calcium voltage-gated channel subunit alpha1 H [Source:HGNC Symbol;Acc:HGNC:1395]
ENSG00000197956	-0,8381768	5,65682115	39,3807734	0,00012901	0,02617598	S100A6	S100 calcium binding protein A6 [Source:HGNC Symbol;Acc:HGNC:10496]
ENSG00000267041	1,79415767	0,82978569	39,6868621	0,00012524	0,02617598	ZNF850	zinc finger protein 850 [Source:HGNC Symbol;Acc:HGNC:27994]
ENSG00000182013	2,14902945	0,05991196	38,9583847	0,00013443	0,02690247	PNMA8A	PNMA family member 8A [Source:HGNC Symbol;Acc:HGNC:25578]
ENSG00000174456	1,00800246	3,20274155	37,537903	0,00015482	0,03056408	C12orf76	chromosome 12 open reading frame 76 [Source:HGNC Symbol;Acc:HGNC:33790]
ENSG00000111875	-1,2106878	5,4477823	36,4729797	0,00017261	0,03148131	ASF1A	anti-silencing function 1A histone chaperone [Source:HGNC Symbol;Acc:HGNC:20995]
ENSG00000126458	-1,2896325	1,78357367	36,365011	0,00017455	0,03148131	RRAS	RAS related [Source:HGNC Symbol;Acc:HGNC:10447]
ENSG00000140297	-0,820213	5,97246086	36,5248301	0,00017169	0,03148131	GCNT3	glucosaminyl (N-acetyl) transferase 3, mucin type [Source:HGNC Symbol;Acc:HGNC:4205]
ENSG00000158710	-1,2883449	2,6741442	36,8547192	0,00016596	0,03148131	TAGLN2	transgelin 2 [Source:HGNC Symbol;Acc:HGNC:11554]
ENSG00000181027	-1,058878	3,44942526	36,7440078	0,00016786	0,03148131	FKRP	fukutin related protein [Source:HGNC Symbol;Acc:HGNC:17997]
ENSG00000185745	1,23380478	1,6367149	36,4455325	0,0001731	0,03148131	IFIT1	interferon induced protein with tetratricopeptide repeats 1 [Source:HGNC Symbol;Acc:HGNC:5407]
ENSG00000228474	-1,2898517	7,40334324	36,7272082	0,00016815	0,03148131	OST4	oligosaccharyltransferase complex subunit 4, non-catalytic [Source:HGNC Symbol;Acc:HGNC:32483]
ENSG00000135454	-1,4095028	3,18151096	35,8201092	0,00018475	0,03169112	B4GALNT1	beta-1,4-N-acetyl-galactosaminyltransferase 1 [Source:HGNC Symbol;Acc:HGNC:4117]
ENSG00000162909	-1,2726338	3,72717794	35,6183363	0,00018871	0,03169112	CAPN2	calpain 2 [Source:HGNC Symbol;Acc:HGNC:1479]
ENSG00000167183	3,17268723	0,65320793	35,8060296	0,00018503	0,03169112	PRR15L	proline rich 15 like [Source:HGNC Symbol;Acc:HGNC:28149]
ENSG00000168621	2,78911714	-0,8077062	36,0254376	0,00018082	0,03169112	GDNF	glial cell derived neurotrophic factor [Source:HGNC Symbol;Acc:HGNC:4232]
ENSG00000173559	-1,4426733	2,35481575	35,6976729	0,00018714	0,03169112	NABP1	nucleic acid binding protein 1 [Source:HGNC Symbol;Acc:HGNC:26232]
ENSG00000251602	-1,4479089	0,88325002	35,6175932	0,00018873	0,03169112	AL928654.1	novel transcript, antisense to MTA1

ENSG00000100593	1,11642682	3,83766512	34,9762738	0,00020203	0,03173537	ISM2	isthmin 2 [Source:HGNC Symbol;Acc:HGNC:23176]
ENSG00000106991	2,22188505	1,46803945	35,1629167	0,00019804	0,03173537	ENG	endoglin [Source:HGNC Symbol;Acc:HGNC:3349]
ENSG00000111321	-0,7892577	6,8939747	35,0534106	0,00020037	0,03173537	LTBR	lymphotoxin beta receptor [Source:HGNC Symbol;Acc:HGNC:6718]
ENSG00000134371	-1,1153165	4,70247641	35,0477893	0,00020049	0,03173537	CDC73	cell division cycle 73 [Source:HGNC Symbol;Acc:HGNC:16783]
ENSG00000136738	-0,9998885	4,64326849	35,4057583	0,000193	0,03173537	STAM	signal transducing adaptor molecule [Source:HGNC Symbol;Acc:HGNC:11357]
ENSG00000253368	-0,8537803	5,37546029	35,1084588	0,00019919	0,03173537	TRNP1	TMF1 regulated nuclear protein 1 [Source:HGNC Symbol;Acc:HGNC:34348]
ENSG00000010244	-0,7926893	6,35758482	34,4064813	0,00021481	0,03203801	ZNF207	zinc finger protein 207 [Source:HGNC Symbol;Acc:HGNC:12998]
ENSG00000101000	-1,3272693	1,31768248	34,7564896	0,00020684	0,03203801	PROCR	protein C receptor [Source:HGNC Symbol;Acc:HGNC:9452]
ENSG00000174567	-0,8491246	5,61382221	34,5972992	0,00021042	0,03203801	GOLT1A	golgi transport 1A [Source:HGNC Symbol;Acc:HGNC:24766]
ENSG00000187764	3,17829298	-0,9330903	34,5149518	0,0002123	0,03203801	SEMA4D	semaphorin 4D [Source:HGNC Symbol;Acc:HGNC:10732]
ENSG00000198099	-1,3793427	5,44257043	34,3643677	0,00021579	0,03203801	ADH4	alcohol dehydrogenase 4 (class II), pi polypeptide [Source:HGNC Symbol;Acc:HGNC:252]
ENSG00000213390	-0,9233033	2,77829376	34,3082243	0,00021711	0,03203801	ARHGAP19	Rho GTPase activating protein 19 [Source:HGNC Symbol;Acc:HGNC:23724]
ENSG00000200087	2,65477637	-0,206777	34,0529135	0,00022324	0,03261244	SNORA73B	small nucleolar RNA, H/ACA box 73B [Source:HGNC Symbol;Acc:HGNC:10116]
ENSG00000172059	1,43695323	1,93347169	33,9229269	0,00022643	0,03275229	KLF11	Kruppel like factor 11 [Source:HGNC Symbol;Acc:HGNC:11811]
ENSG00000148835	0,92646466	3,26641834	33,500339	0,00023723	0,03397711	TAF5	TATA-box binding protein associated factor 5 [Source:HGNC Symbol;Acc:HGNC:11539]
ENSG00000106028	-0,8422419	6,98464712	33,2584978	0,00024369	0,03421374	SSBP1	single stranded DNA binding protein 1 [Source:HGNC Symbol;Acc:HGNC:11317]
ENSG00000144834	-2,6069229	-0,2167634	33,2815758	0,00024306	0,03421374	TAGLN3	transgelin 3 [Source:HGNC Symbol;Acc:HGNC:29868]
ENSG00000167106	0,82618206	5,03255068	33,177203	0,00024591	0,03421374	FAM102A	family with sequence similarity 102 member A [Source:HGNC Symbol;Acc:HGNC:31419]
ENSG00000059573	0,94942934	8,27559143	33,0817096	0,00024854	0,03425457	ALDH18A1	aldehyde dehydrogenase 18 family member A1 [Source:HGNC Symbol;Acc:HGNC:9722]
ENSG00000067445	1,27450926	2,95691708	31,9595038	0,00028229	0,03438097	TRO	trophinin [Source:HGNC Symbol;Acc:HGNC:12326]
ENSG00000079393	-2,0150685	1,92253096	31,7088226	0,00029057	0,03438097	DUSP13	dual specificity phosphatase 13 [Source:HGNC Symbol;Acc:HGNC:19681]
ENSG00000101470	-1,2730538	1,14015786	31,9415896	0,00028287	0,03438097	TNNC2	troponin C2, fast skeletal type [Source:HGNC Symbol;Acc:HGNC:11944]
ENSG00000101974	-0,93993	2,75517887	32,4104997	0,00026809	0,03438097	ATP11C	ATPase phospholipid transporting 11C [Source:HGNC Symbol;Acc:HGNC:13554]
ENSG00000106392	-0,8573007	5,68864142	32,4633197	0,00026649	0,03438097	C1GALT1	core 1 synthase, glycoprotein-N-acetylgalactosamine 3-beta-galactosyltransferase 1 [Source:HGNC Symbol;Acc:HG
ENSG00000113070	-1,7356511	1,46900379	31,7506266	0,00028917	0,03438097	HBEGF	heparin binding EGF like growth factor [Source:HGNC Symbol;Acc:HGNC:3059]
ENSG00000117620	0,92228307	2,95847703	31,6859522	0,00029134	0,03438097	SLC35A3	solute carrier family 35 member A3 [Source:HGNC Symbol;Acc:HGNC:11023]
ENSG00000130066	-0,8829433	7,7640988	32,2854338	0,00027194	0,03438097	SAT1	spermidine/spermine N1-acetyltransferase 1 [Source:HGNC Symbol;Acc:HGNC:10540]
ENSG00000139428	-0,8709776	5,66361663	31,8073576	0,00028728	0,03438097	MMAB	metabolism of cobalamin associated B [Source:HGNC Symbol;Acc:HGNC:19331]
ENSG00000139926	1,37262403	1,37068025	31,8526399	0,00028578	0,03438097	FRMD6	FERM domain containing 6 [Source:HGNC Symbol;Acc:HGNC:19839]
ENSG00000145675	0,85517956	4,20045821	31,9595099	0,00028228	0,03438097	PIK3R1	phosphoinositide-3-kinase regulatory subunit 1 [Source:HGNC Symbol;Acc:HGNC:8979]
ENSG00000155380	-0,8239058	6,13104361	32,5881479	0,00026274	0,03438097	SLC16A1	solute carrier family 16 member 1 [Source:HGNC Symbol;Acc:HGNC:10922]
ENSG00000156515	-1,6070076	0,83771485	32,7467297	0,00025808	0,03438097	HK1	hexokinase 1 [Source:HGNC Symbol;Acc:HGNC:4922]
ENSG00000166260	-0,8819349	6,15066208	31,9760139	0,00028175	0,03438097	COX11	cytochrome c oxidase copper chaperone COX11 [Source:HGNC Symbol;Acc:HGNC:2261]
ENSG00000169398	-0,752459	5,56873104	31,8685008	0,00028526	0,03438097	PTK2	protein tyrosine kinase 2 [Source:HGNC Symbol;Acc:HGNC:9611]
ENSG00000213462	-0,9736501	2,94634271	32,2934632	0,00027169	0,03438097	ERV3-1	endogenous retrovirus group 3 member 1, envelope [Source:HGNC Symbol;Acc:HGNC:3454]
ENSG00000215271	-0,8751524	4,29976771	31,671634	0,00029182	0,03438097	HOMEZ	homeobox and leucine zipper encoding [Source:HGNC Symbol;Acc:HGNC:20164]
ENSG00000275993	0,93635652	2,85173586	32,2527631	0,00027296	0,03438097	SIK1B	salt inducible kinase 1B (putative) [Source:HGNC Symbol;Acc:HGNC:52389]
ENSG00000060138	-0,7890233	6,79992395	31,3122821	0,00030429	0,03447799	YBX3	Y-box binding protein 3 [Source:HGNC Symbol;Acc:HGNC:2428]
ENSG00000101745	1,13667621	2,37579232	31,2045897	0,00030815	0,03447799	ANKRD12	ankyrin repeat domain 12 [Source:HGNC Symbol;Acc:HGNC:29135]
ENSG00000107130	-0,9689169	2,89384508	31,5617161	0,00029557	0,03447799	NCS1	neuronal calcium sensor 1 [Source:HGNC Symbol;Acc:HGNC:3953]
ENSG00000144224	-1,0230528	6,24992717	31,1707322	0,00030937	0,03447799	UBXN4	UBX domain protein 4 [Source:HGNC Symbol;Acc:HGNC:14860]
ENSG00000144959	-1,1912338	4,0587581	31,1116318	0,00031153	0,03447799	NCEH1	neutral cholesterol ester hydrolase 1 [Source:HGNC Symbol;Acc:HGNC:29260]
ENSG00000159713	3,00477492	-0,7644635	31,4852956	0,00029821	0,03447799	TPPP3	tubulin polymerization promoting protein family member 3 [Source:HGNC Symbol;Acc:HGNC:24162]
ENSG00000187772	-0,8122483	4,2738588	31,3430263	0,00030319	0,03447799	LIN28B	lin-28 homolog B [Source:HGNC Symbol;Acc:HGNC:32207]

ENSG00000188643	-0,846536	5,66694301	31,2484638	0,00030657	0,03447799	S100A16	S100 calcium binding protein A16 [Source:HGNC Symbol;Acc:HGNC:20441]
ENSG00000151693	-1,1620037	3,31866843	30,9467671	0,00031763	0,03464184	ASAP2	ArfGAP with SH3 domain, ankyrin repeat and PH domain 2 [Source:HGNC Symbol;Acc:HGNC:2721]
ENSG00000168615	-0,9666546	6,45056708	30,9436739	0,00031775	0,03464184	ADAM9	ADAM metallopeptidase domain 9 [Source:HGNC Symbol;Acc:HGNC:216]
ENSG00000117122	2,10028153	2,04211204	30,5470202	0,00033306	0,03490198	MFAP2	microfibril associated protein 2 [Source:HGNC Symbol;Acc:HGNC:7033]
ENSG00000118985	0,95529642	6,05563779	30,5722115	0,00033206	0,03490198	ELL2	elongation factor for RNA polymerase II 2 [Source:HGNC Symbol;Acc:HGNC:17064]
ENSG00000134153	-0,7852126	7,5882094	30,5901037	0,00033135	0,03490198	EMC7	ER membrane protein complex subunit 7 [Source:HGNC Symbol;Acc:HGNC:24301]
ENSG00000157315	-1,313128	1,01659964	30,5890816	0,00033139	0,03490198	TMED6	transmembrane p24 trafficking protein 6 [Source:HGNC Symbol;Acc:HGNC:28331]
ENSG00000168874	5,06026659	-2,1168007	30,5831692	0,00033163	0,03490198	ATOH8	atonal bHLH transcription factor 8 [Source:HGNC Symbol;Acc:HGNC:24126]
ENSG00000171103	-0,8475438	3,9075026	30,5115295	0,00033447	0,03490198	TRMT61B	tRNA methyltransferase 61B [Source:HGNC Symbol;Acc:HGNC:26070]
ENSG00000112297	0,87657347	4,10475836	30,4501257	0,00033693	0,03490953	CRYBG1	crystallin beta-gamma domain containing 1 [Source:HGNC Symbol;Acc:HGNC:356]
ENSG00000085465	1,11089877	1,76981244	30,276598	0,00034401	0,03539183	OVGP1	oviductal glycoprotein 1 [Source:HGNC Symbol;Acc:HGNC:8524]
ENSG00000133731	-0,9057603	3,62205376	30,1982515	0,00034726	0,03547684	IMPA1	inositol monophosphatase 1 [Source:HGNC Symbol;Acc:HGNC:6050]
ENSG00000108468	-0,8151484	6,25741715	30,1237916	0,00035039	0,03554782	CBX1	chromobox 1 [Source:HGNC Symbol;Acc:HGNC:1551]
ENSG00000104081	0,97942487	2,52948433	29,7611088	0,00036613	0,03638622	BMF	Bcl2 modifying factor [Source:HGNC Symbol;Acc:HGNC:24132]
ENSG00000180573	1,02999426	2,76040719	29,7937487	0,00036468	0,03638622	H2AC6	H2A clustered histone 6 [Source:HGNC Symbol;Acc:HGNC:4733]
ENSG00000204610	-0,8781496	4,46677261	29,7700616	0,00036573	0,03638622	TRIM15	tripartite motif containing 15 [Source:HGNC Symbol;Acc:HGNC:16284]
ENSG00000204220	-0,938214	5,51632721	29,674426	0,00037002	0,03652411	PFDN6	prefoldin subunit 6 [Source:HGNC Symbol;Acc:HGNC:4926]
ENSG00000183386	-0,8295879	4,44566054	29,5845056	0,0003741	0,0366796	FHL3	four and a half LIM domains 3 [Source:HGNC Symbol;Acc:HGNC:3704]
ENSG00000066697	-0,9269475	3,56925202	29,3881409	0,00038322	0,03727685	MSANTD3	Myb/SANT DNA binding domain containing 3 [Source:HGNC Symbol;Acc:HGNC:23370]
ENSG00000188554	0,95254638	7,01724027	29,3441469	0,0003853	0,03727685	NBR1	NBR1 autophagy cargo receptor [Source:HGNC Symbol;Acc:HGNC:6746]
ENSG00000095752	-1,1706008	3,69131668	29,285995	0,00038807	0,03729778	IL11	interleukin 11 [Source:HGNC Symbol;Acc:HGNC:5966]
ENSG00000110108	0,91010452	4,86344571	29,2210491	0,00039119	0,03735208	TMEM109	transmembrane protein 109 [Source:HGNC Symbol;Acc:HGNC:28771]
ENSG00000009413	1,12697251	3,48156467	28,7535906	0,00041458	0,03784577	REV3L	REV3 like, DNA directed polymerase zeta catalytic subunit [Source:HGNC Symbol;Acc:HGNC:9968]
ENSG00000163590	0,96187273	3,68983237	28,5939484	0,00042296	0,03784577	PPM1L	protein phosphatase, Mg2+/Mn2+ dependent 1L [Source:HGNC Symbol;Acc:HGNC:16381]
ENSG00000178057	-0,7845846	5,85007337	28,5007279	0,00042795	0,03784577	NDUFAF3	NADH:ubiquinone oxidoreductase complex assembly factor 3 [Source:HGNC Symbol;Acc:HGNC:29918]
ENSG00000188321	1,35402827	0,98936458	28,7668	0,0004139	0,03784577	ZNF559	zinc finger protein 559 [Source:HGNC Symbol;Acc:HGNC:28197]
ENSG00000189042	1,26097222	1,30732011	28,7996722	0,0004122	0,03784577	ZNF567	zinc finger protein 567 [Source:HGNC Symbol;Acc:HGNC:28696]
ENSG00000196542	1,36490935	1,22031361	29,019246	0,00040109	0,03784577	SPTSSB	serine palmitoyltransferase small subunit B [Source:HGNC Symbol;Acc:HGNC:24045]
ENSG00000198053	0,92146663	2,67729975	28,5361997	0,00042604	0,03784577	SIRPA	signal regulatory protein alpha [Source:HGNC Symbol;Acc:HGNC:9662]
ENSG00000198393	1,02078206	2,71055469	28,6777298	0,00041854	0,03784577	ZNF26	zinc finger protein 26 [Source:HGNC Symbol;Acc:HGNC:13053]
ENSG00000198758	-0,8922925	4,34048141	28,4620791	0,00043004	0,03784577	EPS8L3	EPS8 like 3 [Source:HGNC Symbol;Acc:HGNC:21297]
ENSG00000203668	-1,0121126	2,6752425	28,7711694	0,00041367	0,03784577	CHML	CHM like Rab escort protein [Source:HGNC Symbol;Acc:HGNC:1941]
ENSG00000213937	1,8472567	0,62891801	28,7883833	0,00041278	0,03784577	CLDN9	claudin 9 [Source:HGNC Symbol;Acc:HGNC:2051]
ENSG00000224057	-0,9753604	2,46265688	28,6022623	0,00042252	0,03784577	EGFR-AS1	EGFR antisense RNA 1 [Source:HGNC Symbol;Acc:HGNC:40207]
ENSG00000269821	1,43873259	2,4515195	28,646042	0,0004202	0,03784577	KCNQ1OT1	KCNQ1 opposite strand/antisense transcript 1 [Source:HGNC Symbol;Acc:HGNC:6295]
ENSG00000214855	-1,061835	3,00622842	28,4033122	0,00043324	0,03789906	APOC1P1	apolipoprotein C1 pseudogene 1 [Source:HGNC Symbol;Acc:HGNC:608]
ENSG00000197457	0,74677419	6,75878107	28,1400693	0,00044793	0,03895123	STMN3	stathmin 3 [Source:HGNC Symbol;Acc:HGNC:15926]
ENSG00000163785	0,7906968	5,63390257	28,0676302	0,00045208	0,03907944	RYK	receptor like tyrosine kinase [Source:HGNC Symbol;Acc:HGNC:10481]
ENSG00000063854	-0,7208386	6,05731967	27,9663429	0,00045796	0,03935493	HAGH	hydroxyacylglutathione hydrolase [Source:HGNC Symbol;Acc:HGNC:4805]
ENSG00000137500	-1,0444007	4,14429024	27,7669613	0,00046981	0,03955677	CCDC90B	coiled-coil domain containing 90B [Source:HGNC Symbol;Acc:HGNC:28108]
ENSG00000176871	-0,9464302	4,02793427	27,7601176	0,00047022	0,03955677	WSB2	WD repeat and SOCS box containing 2 [Source:HGNC Symbol;Acc:HGNC:19222]
ENSG00000231172	-1,2813688	1,41164044	27,7449864	0,00047114	0,03955677	AC007099.1	novel transcript, antisense to EVA1A
ENSG00000277247	-2,5434868	-0,7146124	27,7846283	0,00046875	0,03955677	AC083809.1	novel transcript, antisense to PTPRB
ENSG00000221968	-0,8908913	4,75172427	27,6698541	0,00047572	0,0397129	FADS3	fatty acid desaturase 3 [Source:HGNC Symbol;Acc:HGNC:3576]

ENSG00000103855	-1,0077115	5,68921256	27,5349598	0,00048407	0,03974706	CD276	CD276 molecule [Source:HGNC Symbol;Acc:HGNC:19137]
ENSG00000105219	1,77490227	0,23651307	27,6093268	0,00047945	0,03974706	CCNP	cyclin P [Source:HGNC Symbol;Acc:HGNC:25805]
ENSG00000108244	-0,89343	5,20828922	27,5315258	0,00048429	0,03974706	KRT23	keratin 23 [Source:HGNC Symbol;Acc:HGNC:6438]
ENSG00000040275	-0,84812	4,32164294	27,262506	0,0005015	0,04008347	SPDL1	spindle apparatus coiled-coil protein 1 [Source:HGNC Symbol;Acc:HGNC:26010]
ENSG00000080822	-0,8905919	4,14672083	27,3009766	0,000499	0,04008347	CLDND1	claudin domain containing 1 [Source:HGNC Symbol;Acc:HGNC:1322]
ENSG00000114744	-0,912924	4,62138696	27,2564541	0,0005019	0,04008347	COMMD2	COMM domain containing 2 [Source:HGNC Symbol;Acc:HGNC:24993]
ENSG00000224078	0,95220729	4,81679722	27,3161174	0,00049801	0,04008347	SNHG14	small nucleolar RNA host gene 14 [Source:HGNC Symbol;Acc:HGNC:37462]
ENSG00000279495	0,94470287	2,19216443	27,2532836	0,00050211	0,04008347	AL928654.4	novel transcript
ENSG00000132031	1,21093195	8,47394398	27,0041972	0,00051874	0,04118615	MATN3	matrilin 3 [Source:HGNC Symbol;Acc:HGNC:6909]
ENSG00000125772	1,19424388	4,26919053	26,8289999	0,00053084	0,04191913	GPCPD1	glycerophosphocholine phosphodiesterase 1 [Source:HGNC Symbol;Acc:HGNC:26957]
ENSG00000119541	0,97866073	4,61558339	26,6471097	0,00054377	0,04248086	VPS4B	vacuolar protein sorting 4 homolog B [Source:HGNC Symbol;Acc:HGNC:10895]
ENSG00000148798	3,21054553	-1,5539113	26,6814844	0,0005413	0,04248086	INA	internexin neuronal intermediate filament protein alpha [Source:HGNC Symbol;Acc:HGNC:6057]
ENSG00000169429	-0,9588988	1,86648037	26,6049797	0,00054682	0,04249185	CXCL8	C-X-C motif chemokine ligand 8 [Source:HGNC Symbol;Acc:HGNC:6025]
ENSG00000104812	-1,0018622	3,89529726	26,5247441	0,00055268	0,04272036	GYS1	glycogen synthase 1 [Source:HGNC Symbol;Acc:HGNC:4706]
ENSG00000118515	0,86326782	5,63727837	26,2090181	0,00057652	0,04386646	SGK1	serum/glucocorticoid regulated kinase 1 [Source:HGNC Symbol;Acc:HGNC:10810]
ENSG00000154930	0,8126223	6,21753194	26,2367905	0,00057437	0,04386646	ACSS1	acyl-CoA synthetase short chain family member 1 [Source:HGNC Symbol;Acc:HGNC:16091]
ENSG00000224596	-2,4822584	-0,4434633	26,2747049	0,00057146	0,04386646	ZMIZ1-AS1	ZMIZ1 antisense RNA 1 [Source:HGNC Symbol;Acc:HGNC:27433]
ENSG00000175793	-0,7588639	5,84579066	26,1656709	0,00057989	0,04389422	SFN	stratifin [Source:HGNC Symbol;Acc:HGNC:10773]
ENSG00000136960	0,93393179	2,75954838	25,9289427	0,00059872	0,04508593	ENPP2	ectonucleotide pyrophosphatase/phosphodiesterase 2 [Source:HGNC Symbol;Acc:HGNC:3357]
ENSG00000272405	-1,7193068	2,21170546	25,8765895	0,00060298	0,04517419	AL365181.3	novel transcript, antisense to BCAN
ENSG00000090612	0,85136547	3,76099758	25,7629432	0,00061237	0,04523617	ZNF268	zinc finger protein 268 [Source:HGNC Symbol;Acc:HGNC:13061]
ENSG00000112561	0,81254251	4,43943015	25,758757	0,00061272	0,04523617	TFEB	transcription factor EB [Source:HGNC Symbol;Acc:HGNC:11753]
ENSG00000162819	-0,9180062	2,64182492	25,7295543	0,00061516	0,04523617	BROX	BRO1 domain and CAAX motif containing [Source:HGNC Symbol;Acc:HGNC:26512]
ENSG00000186283	0,77867089	7,41281831	25,717193	0,0006162	0,04523617	TOR3A	torsin family 3 member A [Source:HGNC Symbol;Acc:HGNC:11997]
ENSG00000158104	-0,770779	7,83885681	25,5930781	0,00062673	0,04577939	HPD	4-hydroxyphenylpyruvate dioxygenase [Source:HGNC Symbol;Acc:HGNC:5147]
ENSG00000106384	-0,7184233	4,83332112	25,4627745	0,00063802	0,04632036	MOGAT3	monoacylglycerol O-acyltransferase 3 [Source:HGNC Symbol;Acc:HGNC:23249]
ENSG00000110628	-0,9826788	1,68909128	25,3645721	0,0006467	0,04632036	SLC22A18	solute carrier family 22 member 18 [Source:HGNC Symbol;Acc:HGNC:10964]
ENSG00000172795	0,72553231	4,6920991	25,3876783	0,00064465	0,04632036	DCP2	decapping mRNA 2 [Source:HGNC Symbol;Acc:HGNC:24452]
ENSG00000213516	-0,7834653	3,48030089	25,363264	0,00064682	0,04632036	RBMXL1	RBMX like 1 [Source:HGNC Symbol;Acc:HGNC:25073]
ENSG00000104219	-1,1773586	2,54943961	25,3171575	0,00065094	0,04638832	ZDHHC2	zinc finger DHHC-type palmitoyltransferase 2 [Source:HGNC Symbol;Acc:HGNC:18469]
ENSG00000142661	-0,8541992	3,90462534	25,197066	0,00066184	0,04686687	MYOM3	myomesin 3 [Source:HGNC Symbol;Acc:HGNC:26679]
ENSG00000196083	0,81776704	4,13612896	25,1726944	0,00066407	0,04686687	IL1RAP	interleukin 1 receptor accessory protein [Source:HGNC Symbol;Acc:HGNC:5995]
ENSG00000132879	0,74910466	4,18410785	25,1192013	0,00066902	0,04692533	FBXO44	F-box protein 44 [Source:HGNC Symbol;Acc:HGNC:24847]
ENSG00000134013	-2,056019	0,07610973	25,0600386	0,00067454	0,04692533	LOXL2	lysyl oxidase like 2 [Source:HGNC Symbol;Acc:HGNC:6666]
ENSG00000260231	-0,8173167	3,1453269	25,0708095	0,00067353	0,04692533	KDM7A-DT	KDM7A divergent transcript [Source:HGNC Symbol;Acc:HGNC:48959]
ENSG00000126775	0,80531807	3,12755976	25,011864	0,00067907	0,04701701	ATG14	autophagy related 14 [Source:HGNC Symbol;Acc:HGNC:19962]
ENSG00000133874	0,826848	3,06347907	24,8236295	0,00069716	0,0473711	RNF122	ring finger protein 122 [Source:HGNC Symbol;Acc:HGNC:21147]
ENSG00000160055	0,74236879	3,74248078	24,8341821	0,00069613	0,0473711	TMEM234	transmembrane protein 234 [Source:HGNC Symbol;Acc:HGNC:28837]
ENSG00000168778	1,02775191	3,715167	24,8679254	0,00069285	0,0473711	TCTN2	tectonic family member 2 [Source:HGNC Symbol;Acc:HGNC:25774]
ENSG00000198363	-0,9114698	7,92583026	24,8655078	0,00069308	0,0473711	ASPH	aspartate beta-hydroxylase [Source:HGNC Symbol;Acc:HGNC:757]
ENSG00000112742	0,78477438	3,86537515	24,7412791	0,00070525	0,04769935	TTK	TTK protein kinase [Source:HGNC Symbol;Acc:HGNC:12401]
ENSG00000149308	0,87618277	2,30878118	24,6421647	0,00071515	0,04770611	NPAT	nuclear protein, coactivator of histone transcription [Source:HGNC Symbol;Acc:HGNC:7896]
ENSG00000150782	-2,515256	-0,3608062	24,6689039	0,00071246	0,04770611	IL18	interleukin 18 [Source:HGNC Symbol;Acc:HGNC:5986]
ENSG00000276600	-2,6847784	-0,9112418	24,6715533	0,0007122	0,04770611	RAB7B	RAB7B, member RAS oncogene family [Source:HGNC Symbol;Acc:HGNC:30513]

ENSG00000142178	1,50233167	0,13956801	24,4463538	0,0007352	0,04838109	SIK1	salt inducible kinase 1 [Source:HGNC Symbol;Acc:HGNC:11142]
ENSG00000167601	-1,7626153	3,61373565	24,4603822	0,00073375	0,04838109	AXL	AXL receptor tyrosine kinase [Source:HGNC Symbol;Acc:HGNC:905]
ENSG00000180817	-0,6752552	7,58460812	24,4718372	0,00073256	0,04838109	PPA1	inorganic pyrophosphatase 1 [Source:HGNC Symbol;Acc:HGNC:9226]
ENSG00000140450	1,67859078	0,9295808	24,3637382	0,00074387	0,04873185	ARRDC4	arrestin domain containing 4 [Source:HGNC Symbol;Acc:HGNC:28087]
ENSG00000145996	-0,8454156	3,74883922	24,3058215	0,00075002	0,04891536	CDKAL1	CDK5 regulatory subunit associated protein 1 like 1 [Source:HGNC Symbol;Acc:HGNC:21050]
ENSG00000066654	0,69611062	5,66659412	24,0927299	0,00077319	0,04937996	THUMPD1	THUMP domain containing 1 [Source:HGNC Symbol;Acc:HGNC:23807]
ENSG00000104611	0,74302228	4,35591818	24,0559315	0,00077727	0,04937996	SH2D4A	SH2 domain containing 4A [Source:HGNC Symbol;Acc:HGNC:26102]
ENSG00000110693	1,16988859	0,9423692	24,1145883	0,00077077	0,04937996	SOX6	SRY-box transcription factor 6 [Source:HGNC Symbol;Acc:HGNC:16421]
ENSG00000124459	0,85959947	2,33242587	24,2037237	0,00076101	0,04937996	ZNF45	zinc finger protein 45 [Source:HGNC Symbol;Acc:HGNC:13111]
ENSG00000188312	-1,493687	2,1524762	24,0651611	0,00077625	0,04937996	CENPP	centromere protein P [Source:HGNC Symbol;Acc:HGNC:32933]
ENSG00000228648	-1,623296	0,01862727	24,0545782	0,00077742	0,04937996	AL353747.2	novel transcript
ENSG00000047634	-0,8414078	3,45054828	23,9934002	0,00078428	0,04959983	SCML1	Scm polycomb group protein like 1 [Source:HGNC Symbol;Acc:HGNC:10580]
ENSG00000090238	0,77771718	3,17778125	23,8155985	0,00080463	0,05032882	YPEL3	yippee like 3 [Source:HGNC Symbol;Acc:HGNC:18327]
ENSG00000112992	0,75886656	5,18047244	23,7879266	0,00080786	0,05032882	NNT	nicotinamide nucleotide transhydrogenase [Source:HGNC Symbol;Acc:HGNC:7863]
ENSG00000198835	1,09338963	1,73536948	23,7731142	0,00080959	0,05032882	GJC2	gap junction protein gamma 2 [Source:HGNC Symbol;Acc:HGNC:17494]
ENSG00000229043	-1,0382609	1,30691359	23,8360211	0,00080226	0,05032882	ZFAND2A-DT	ZFAND2A divergent transcript [Source:HGNC Symbol;Acc:HGNC:41187]
ENSG00000109819	0,94644094	2,38259266	23,714582	0,00081648	0,05054213	PPARGC1A	PPARG coactivator 1 alpha [Source:HGNC Symbol;Acc:HGNC:9237]
ENSG00000073712	-0,8604647	5,11160645	23,5499744	0,00083624	0,05095176	FERMT2	fermitin family member 2 [Source:HGNC Symbol;Acc:HGNC:15767]
ENSG00000137166	0,67316753	6,096588	23,5433926	0,00083705	0,05095176	FOXP4	forkhead box P4 [Source:HGNC Symbol;Acc:HGNC:20842]
ENSG00000139117	-2,1528833	0,17081971	23,5554931	0,00083557	0,05095176	CPNE8	copine 8 [Source:HGNC Symbol;Acc:HGNC:23498]
ENSG00000163597	-0,8419561	6,10894629	23,5540784	0,00083575	0,05095176	SNHG16	small nucleolar RNA host gene 16 [Source:HGNC Symbol;Acc:HGNC:44352]
ENSG00000086189	-0,6859605	4,7143186	23,4147159	0,00085292	0,05138014	DIMT1	DIMT1 rRNA methyltransferase and ribosome maturation factor [Source:HGNC Symbol;Acc:HGNC:30217]
ENSG00000166797	-0,89152	5,02106782	23,4010173	0,00085464	0,05138014	CIAO2A	cytosolic iron-sulfur assembly component 2A [Source:HGNC Symbol;Acc:HGNC:26235]
ENSG00000167910	1,55487056	1,51703558	23,441541	0,00084958	0,05138014	CYP7A1	cytochrome P450 family 7 subfamily A member 1 [Source:HGNC Symbol;Acc:HGNC:2651]
ENSG00000120057	1,54173672	0,57270788	23,3654943	0,00085909	0,05143651	SFRP5	secreted frizzled related protein 5 [Source:HGNC Symbol;Acc:HGNC:10779]
ENSG00000107738	-1,6589022	1,01699257	23,2288081	0,00087652	0,05184218	VSIR	V-set immunoregulatory receptor [Source:HGNC Symbol;Acc:HGNC:30085]
ENSG00000160191	-0,7944752	2,5194155	23,2535831	0,00087333	0,05184218	PDE9A	phosphodiesterase 9A [Source:HGNC Symbol;Acc:HGNC:8795]
ENSG00000260573	-0,9626244	2,32451397	23,2441319	0,00087454	0,05184218	AC007493.1	novel transcript
ENSG00000011422	-1,2672019	2,14073292	22,9046798	0,00091958	0,052526	PLAUR	plasminogen activator, urokinase receptor [Source:HGNC Symbol;Acc:HGNC:9053]
ENSG00000039068	0,79437216	3,19345782	22,5057106	0,00097618	0,052526	CDH1	cadherin 1 [Source:HGNC Symbol;Acc:HGNC:1748]
ENSG00000053501	-0,76713	3,77475381	22,7029138	0,00094769	0,052526	USE1	unconventional SNARE in the ER 1 [Source:HGNC Symbol;Acc:HGNC:30882]
ENSG00000069509	-1,1877996	3,28790964	22,6582388	0,00095405	0,052526	FUNDC1	FUN14 domain containing 1 [Source:HGNC Symbol;Acc:HGNC:28746]
ENSG00000074527	0,8773702	3,86760365	22,6972858	0,00094849	0,052526	NTN4	netrin 4 [Source:HGNC Symbol;Acc:HGNC:13658]
ENSG00000081923	1,04565805	2,2061396	22,9365323	0,00091523	0,052526	ATP8B1	ATPase phospholipid transporting 8B1 [Source:HGNC Symbol;Acc:HGNC:3706]
ENSG00000100442	-1,0603309	6,5220228	22,5213262	0,00097389	0,052526	FKBP3	FKBP prolyl isomerase 3 [Source:HGNC Symbol;Acc:HGNC:3719]
ENSG00000126107	-1,1071129	4,47545336	22,5954209	0,00096309	0,052526	HECTD3	HECT domain E3 ubiquitin protein ligase 3 [Source:HGNC Symbol;Acc:HGNC:26117]
ENSG00000129353	1,37418476	3,91916703	22,4863978	0,00097903	0,052526	SLC44A2	solute carrier family 44 member 2 [Source:HGNC Symbol;Acc:HGNC:17292]
ENSG00000130176	-1,9336602	-0,6022456	22,8413732	0,00092829	0,052526	CNN1	calponin 1 [Source:HGNC Symbol;Acc:HGNC:2155]
ENSG00000132256	-0,9069174	2,13174913	22,3823888	0,00099453	0,052526	TRIM5	tripartite motif containing 5 [Source:HGNC Symbol;Acc:HGNC:16276]
ENSG00000141448	0,81413827	2,71742484	22,8256092	0,00093047	0,052526	GATA6	GATA binding protein 6 [Source:HGNC Symbol;Acc:HGNC:4174]
ENSG00000145287	-0,7093936	4,44636421	22,4609081	0,0009828	0,052526	PLAC8	placenta associated 8 [Source:HGNC Symbol;Acc:HGNC:19254]
ENSG00000150764	-0,932901	2,44187151	23,007977	0,00090558	0,052526	DIXDC1	DIX domain containing 1 [Source:HGNC Symbol;Acc:HGNC:23695]
ENSG00000150938	-1,2067274	5,24380327	22,8320349	0,00092958	0,052526	CRIM1	cysteine rich transmembrane BMP regulator 1 [Source:HGNC Symbol;Acc:HGNC:2359]
ENSG00000154723	-0,7826684	7,37688548	22,3517132	0,00099916	0,052526	ATP5PF	ATP synthase peripheral stalk subunit F6 [Source:HGNC Symbol;Acc:HGNC:847]

ENSG00000157625	0,99565533	3,33337008	22,4235288	0,00098836	0,052526	TAB3	TGF-beta activated kinase 1 (MAP3K7) binding protein 3 [Source:HGNC Symbol;Acc:HGNC:30681]
ENSG00000167191	1,52005862	0,43282913	22,3762384	0,00099546	0,052526	GPRC5B	G protein-coupled receptor class C group 5 member B [Source:HGNC Symbol;Acc:HGNC:13308]
ENSG00000168071	-1,0074262	1,9147725	22,5445019	0,0009705	0,052526	CCDC88B	coiled-coil domain containing 88B [Source:HGNC Symbol;Acc:HGNC:26757]
ENSG00000172057	0,64940294	6,83108872	22,6318904	0,00095783	0,052526	ORMDL3	ORMDL sphingolipid biosynthesis regulator 3 [Source:HGNC Symbol;Acc:HGNC:16038]
ENSG00000173702	2,44868194	-1,2962426	22,9895724	0,00090806	0,052526	MUC13	mucin 13, cell surface associated [Source:HGNC Symbol;Acc:HGNC:7511]
ENSG00000175707	0,98764989	2,82975761	22,7523172	0,00094071	0,052526	KDF1	keratinocyte differentiation factor 1 [Source:HGNC Symbol;Acc:HGNC:26624]
ENSG00000177425	-0,7504143	4,60749346	22,6069666	0,00096142	0,052526	PAWR	pro-apoptotic WT1 regulator [Source:HGNC Symbol;Acc:HGNC:8614]
ENSG00000178826	0,92898507	2,69268037	22,8995889	0,00092028	0,052526	TMEM139	transmembrane protein 139 [Source:HGNC Symbol;Acc:HGNC:22058]
ENSG00000185551	0,73473206	3,56526841	22,8872408	0,00092197	0,052526	NR2F2	nuclear receptor subfamily 2 group F member 2 [Source:HGNC Symbol;Acc:HGNC:7976]
ENSG00000196739	0,98817288	5,61055283	22,6437437	0,00095613	0,052526	COL27A1	collagen type XXVII alpha 1 chain [Source:HGNC Symbol;Acc:HGNC:22986]
ENSG00000197050	1,28970494	1,22998925	22,349236	0,00099954	0,052526	ZNF420	zinc finger protein 420 [Source:HGNC Symbol;Acc:HGNC:20649]
ENSG00000197608	1,26661498	1,67378468	22,4284087	0,00098764	0,052526	ZNF841	zinc finger protein 841 [Source:HGNC Symbol;Acc:HGNC:27611]
ENSG00000213574	-1,3990563	0,33067766	22,5457471	0,00097031	0,052526	LDHAP5	lactate dehydrogenase A pseudogene 5 [Source:HGNC Symbol;Acc:HGNC:6540]
ENSG00000261189	-0,8080258	2,82035216	22,74902	0,00094117	0,052526	AL031058.1	novel transcript, antisense to DSP
ENSG00000266304	-1,79618	-0,0841603	23,0158283	0,00090453	0,052526	LIVAR	liver cell viability associated lncRNA [Source:HGNC Symbol;Acc:HGNC:53130]
ENSG00000173214	0,77727841	3,20774119	22,297225	0,00100745	0,05256367	MFSD4B	major facilitator superfamily domain containing 4B [Source:HGNC Symbol;Acc:HGNC:21053]
ENSG00000197619	0,85736214	2,10839122	22,306327	0,00100606	0,05256367	ZNF615	zinc finger protein 615 [Source:HGNC Symbol;Acc:HGNC:24740]
ENSG00000105711	0,83469283	2,57437931	22,1008477	0,00103802	0,05377474	SCN1B	sodium voltage-gated channel beta subunit 1 [Source:HGNC Symbol;Acc:HGNC:10586]
ENSG00000131115	0,81746391	2,8737588	22,1125484	0,00103617	0,05377474	ZNF227	zinc finger protein 227 [Source:HGNC Symbol;Acc:HGNC:13020]
ENSG00000111671	-0,8976798	2,71882897	22,0030906	0,00105366	0,0542006	SPSB2	splA/ryanodine receptor domain and SOCS box containing 2 [Source:HGNC Symbol;Acc:HGNC:29522]
ENSG00000143416	-0,7016533	4,74879004	22,0173029	0,00105137	0,0542006	SELENBP1	selenium binding protein 1 [Source:HGNC Symbol;Acc:HGNC:10719]
ENSG00000130150	-0,9153831	2,02355009	21,9253679	0,0010663	0,05427099	MOSPD2	motile sperm domain containing 2 [Source:HGNC Symbol;Acc:HGNC:28381]
ENSG00000143375	1,44916123	1,1464025	21,9267777	0,00106607	0,05427099	CGN	cingulin [Source:HGNC Symbol;Acc:HGNC:17429]
ENSG00000164323	-0,6678057	4,82114212	21,9035153	0,00106989	0,05427099	CFAP97	cilia and flagella associated protein 97 [Source:HGNC Symbol;Acc:HGNC:29276]
ENSG00000185920	0,92771117	1,96803241	21,940832	0,00106377	0,05427099	PTCH1	patched 1 [Source:HGNC Symbol;Acc:HGNC:9585]
ENSG00000014919	0,70234858	4,35859304	21,8710641	0,00107525	0,05435386	COX15	cytochrome c oxidase assembly homolog COX15 [Source:HGNC Symbol;Acc:HGNC:2263]
ENSG00000153250	-0,6868744	4,33789338	21,8426594	0,00107996	0,05440388	RBMS1	RNA binding motif single stranded interacting protein 1 [Source:HGNC Symbol;Acc:HGNC:9907]
ENSG00000026297	-0,868715	4,79465787	21,4883238	0,00114093	0,05460988	RNASET2	ribonuclease T2 [Source:HGNC Symbol;Acc:HGNC:21686]
ENSG00000101188	-1,9685431	0,30375857	21,5188352	0,00113552	0,05460988	NTSR1	neurotensin receptor 1 [Source:HGNC Symbol;Acc:HGNC:8039]
ENSG00000101197	-1,8873157	-0,6346136	21,6423579	0,00111392	0,05460988	BIRC7	baculoviral IAP repeat containing 7 [Source:HGNC Symbol;Acc:HGNC:13702]
ENSG00000102081	0,72344274	4,06420312	21,4718765	0,00114386	0,05460988	FMR1	FMRP translational regulator 1 [Source:HGNC Symbol;Acc:HGNC:3775]
ENSG00000105825	-1,1241113	0,90276732	21,4798631	0,00114243	0,05460988	TFPI2	tissue factor pathway inhibitor 2 [Source:HGNC Symbol;Acc:HGNC:11761]
ENSG00000112576	-0,7401851	3,78389011	21,6310002	0,00111589	0,05460988	CCND3	cyclin D3 [Source:HGNC Symbol;Acc:HGNC:1585]
ENSG00000115204	-0,7247647	5,81026759	21,7371636	0,00109769	0,05460988	MPV17	mitochondrial inner membrane protein MPV17 [Source:HGNC Symbol;Acc:HGNC:7224]
ENSG00000130988	-0,9070902	5,33010049	21,5747158	0,00112568	0,05460988	RGN	regucalcin [Source:HGNC Symbol;Acc:HGNC:9989]
ENSG00000139725	-0,6861834	5,1325994	21,5143948	0,0011363	0,05460988	RHOF	ras homolog family member F, filopodia associated [Source:HGNC Symbol;Acc:HGNC:15703]
ENSG00000152931	-0,8443586	2,20140889	21,5902883	0,00112296	0,05460988	PART1	prostate androgen-regulated transcript 1 [Source:HGNC Symbol;Acc:HGNC:17263]
ENSG00000165355	0,8680318	3,61923915	21,5814452	0,00112451	0,05460988	FBXO33	F-box protein 33 [Source:HGNC Symbol;Acc:HGNC:19833]
ENSG00000170906	-0,7957685	6,07396122	21,7239982	0,00109992	0,05460988	NDUFA3	NADH:ubiquinone oxidoreductase subunit A3 [Source:HGNC Symbol;Acc:HGNC:7686]
ENSG00000171421	-0,7613193	5,83362636	21,5548768	0,00112916	0,05460988	MRPL36	mitochondrial ribosomal protein L36 [Source:HGNC Symbol;Acc:HGNC:14490]
ENSG00000171877	-1,1044998	2,48800257	21,545554	0,0011308	0,05460988	FRMD5	FERM domain containing 5 [Source:HGNC Symbol;Acc:HGNC:28214]
ENSG00000198931	-0,6991758	7,95021947	21,4765605	0,00114302	0,05460988	APRT	adenine phosphoribosyltransferase [Source:HGNC Symbol;Acc:HGNC:626]
ENSG00000205436	-0,890363	2,21914128	21,7810384	0,00109027	0,05460988	EXOC3L4	exocyst complex component 3 like 4 [Source:HGNC Symbol;Acc:HGNC:20120]
ENSG00000105856	0,87250173	4,18319033	21,3877237	0,001159	0,05461864	HBP1	HMG-box transcription factor 1 [Source:HGNC Symbol;Acc:HGNC:23200]

ENSG00000139219	0,83500349	5,38768991	21,4037936	0,00115609	0,05461864	COL2A1	collagen type II alpha 1 chain [Source:HGNC Symbol;Acc:HGNC:2200]
ENSG00000143149	-0,8009309	4,83867485	21,4022847	0,00115636	0,05461864	ALDH9A1	aldehyde dehydrogenase 9 family member A1 [Source:HGNC Symbol;Acc:HGNC:412]
ENSG00000152484	-0,7469582	3,29696532	21,4291259	0,00115152	0,05461864	USP12	ubiquitin specific peptidase 12 [Source:HGNC Symbol;Acc:HGNC:20485]
ENSG00000166557	-0,7367786	8,22732775	21,306693	0,0011738	0,05478629	TMED3	transmembrane p24 trafficking protein 3 [Source:HGNC Symbol;Acc:HGNC:28889]
ENSG00000178150	-1,0753991	4,93772435	21,307321	0,00117369	0,05478629	ZNF114	zinc finger protein 114 [Source:HGNC Symbol;Acc:HGNC:12894]
ENSG00000260630	-0,7901771	3,16989074	21,3195016	0,00117145	0,05478629	SNAI3-AS1	SNAI3 antisense RNA 1 [Source:HGNC Symbol;Acc:HGNC:28327]
ENSG00000127989	-0,7394936	2,98932405	21,2449174	0,00118525	0,05498393	MTERF1	mitochondrial transcription termination factor 1 [Source:HGNC Symbol;Acc:HGNC:21463]
ENSG00000149292	-0,8323649	2,8427786	21,2432133	0,00118557	0,05498393	TTC12	tetratricopeptide repeat domain 12 [Source:HGNC Symbol;Acc:HGNC:23700]
ENSG00000034677	0,99146679	4,24463128	21,0320008	0,00122575	0,05621464	RNF19A	ring finger protein 19A, RBR E3 ubiquitin protein ligase [Source:HGNC Symbol;Acc:HGNC:13432]
ENSG00000106723	0,77946461	2,85259422	20,9668684	0,00123847	0,05621464	SPIN1	spindlin 1 [Source:HGNC Symbol;Acc:HGNC:11243]
ENSG00000110169	-0,6815533	4,68108368	21,0058078	0,00123085	0,05621464	HPX	hemopexin [Source:HGNC Symbol;Acc:HGNC:5171]
ENSG00000116273	0,78188367	4,9883267	21,0340839	0,00122534	0,05621464	PHF13	PHD finger protein 13 [Source:HGNC Symbol;Acc:HGNC:22983]
ENSG00000134146	-0,9645941	2,84935525	20,9639975	0,00123904	0,05621464	DPH6	diphthamine biosynthesis 6 [Source:HGNC Symbol;Acc:HGNC:30543]
ENSG00000135870	1,17846408	2,05665096	20,9938046	0,00123319	0,05621464	RC3H1	ring finger and CCCH-type domains 1 [Source:HGNC Symbol;Acc:HGNC:29434]
ENSG00000164151	0,7886248	4,55737129	21,0029398	0,00123141	0,05621464	ICE1	interactor of little elongation complex ELL subunit 1 [Source:HGNC Symbol;Acc:HGNC:29154]
ENSG00000088899	-0,7160651	3,74835169	20,919229	0,00124788	0,05644064	LZTS3	leucine zipper tumor suppressor family member 3 [Source:HGNC Symbol;Acc:HGNC:30139]
ENSG00000099998	-1,5708009	3,45979555	20,8268423	0,00126638	0,05684635	GGT5	gamma-glutamyltransferase 5 [Source:HGNC Symbol;Acc:HGNC:4260]
ENSG00000114854	-0,9209424	3,23322696	20,8518196	0,00126135	0,05684635	TNNC1	troponin C1, slow skeletal and cardiac type [Source:HGNC Symbol;Acc:HGNC:11943]
ENSG00000167232	0,80449628	2,4711122	20,8162273	0,00126853	0,05684635	ZNF91	zinc finger protein 91 [Source:HGNC Symbol;Acc:HGNC:13166]
ENSG00000184260	-0,9956704	2,0336316	20,7916102	0,00127352	0,05689567	H2AC20	H2A clustered histone 20 [Source:HGNC Symbol;Acc:HGNC:4738]
ENSG00000131373	-0,7220099	3,75788819	20,7475385	0,00128252	0,05712319	HACL1	2-hydroxyacyl-CoA lyase 1 [Source:HGNC Symbol;Acc:HGNC:17856]
ENSG00000198146	-0,6985011	3,65942351	20,6540188	0,00130189	0,05780934	ZNF770	zinc finger protein 770 [Source:HGNC Symbol;Acc:HGNC:26061]
ENSG00000146085	0,77263914	5,5733023	20,6224595	0,0013085	0,05792697	MMUT	methylmalonyl-CoA mutase [Source:HGNC Symbol;Acc:HGNC:7526]
ENSG00000105185	-0,7819764	6,45206549	20,5654444	0,00132056	0,05828396	PDCD5	programmed cell death 5 [Source:HGNC Symbol;Acc:HGNC:8764]
ENSG00000177058	0,93251969	4,10651707	20,5409429	0,00132578	0,05833817	SLC38A9	solute carrier family 38 member 9 [Source:HGNC Symbol;Acc:HGNC:26907]
ENSG00000131746	-1,6224711	3,9049846	20,4515691	0,00134504	0,05900796	TNS4	tensin 4 [Source:HGNC Symbol;Acc:HGNC:24352]
ENSG00000164219	-0,6895026	3,46018284	20,4294639	0,00134985	0,05904196	PGGT1B	protein geranylgeranyltransferase type I subunit beta [Source:HGNC Symbol;Acc:HGNC:8895]
ENSG00000170537	-0,9697036	3,65248402	20,3986241	0,00135661	0,05916028	TMC7	transmembrane channel like 7 [Source:HGNC Symbol;Acc:HGNC:23000]
ENSG00000131480	1,48521687	0,0254606	20,3584232	0,00136548	0,05936973	AOC2	amine oxidase copper containing 2 [Source:HGNC Symbol;Acc:HGNC:549]
ENSG00000128165	-0,7991994	3,85098531	20,338332	0,00136993	0,05938681	ADM2	adrenomedullin 2 [Source:HGNC Symbol;Acc:HGNC:28898]
ENSG00000187024	-0,7683494	2,75592259	20,3084584	0,00137659	0,05949902	PTRH1	peptidyl-tRNA hydrolase 1 homolog [Source:HGNC Symbol;Acc:HGNC:27039]
ENSG00000142765	-0,7917124	3,14184123	20,2734132	0,00138446	0,05966243	SYTL1	synaptotagmin like 1 [Source:HGNC Symbol;Acc:HGNC:15584]
ENSG00000183647	0,99324203	1,44079869	20,222786	0,00139592	0,05997931	ZNF530	zinc finger protein 530 [Source:HGNC Symbol;Acc:HGNC:29297]
ENSG00000140961	-0,9432111	5,36900317	20,1731611	0,00140726	0,06028942	OSGIN1	oxidative stress induced growth inhibitor 1 [Source:HGNC Symbol;Acc:HGNC:30093]
ENSG00000005981	-1,3942274	1,88707351	20,0934343	0,00142572	0,0603075	ASB4	ankyrin repeat and SOCS box containing 4 [Source:HGNC Symbol;Acc:HGNC:16009]
ENSG00000027869	-1,4568678	0,59123824	20,0600583	0,00143354	0,0603075	SH2D2A	SH2 domain containing 2A [Source:HGNC Symbol;Acc:HGNC:10821]
ENSG00000100079	-0,8374517	4,70160282	20,1235206	0,00141872	0,0603075	LGALS2	galectin 2 [Source:HGNC Symbol;Acc:HGNC:6562]
ENSG00000120693	0,89298175	1,57550237	20,1509672	0,00141237	0,0603075	SMAD9	SMAD family member 9 [Source:HGNC Symbol;Acc:HGNC:6774]
ENSG00000130208	-0,6427221	8,43732734	20,0471333	0,00143658	0,0603075	APOC1	apolipoprotein C1 [Source:HGNC Symbol;Acc:HGNC:607]
ENSG00000144331	1,47626133	0,06729949	20,0560125	0,00143449	0,0603075	ZNF385B	zinc finger protein 385B [Source:HGNC Symbol;Acc:HGNC:26332]
ENSG00000163513	0,76985302	8,38689328	20,1170399	0,00142023	0,0603075	TGFBR2	transforming growth factor beta receptor 2 [Source:HGNC Symbol;Acc:HGNC:11773]
ENSG00000206535	-1,1358966	0,98443516	20,0246318	0,00144189	0,06035709	LNP1	leukemia NUP98 fusion partner 1 [Source:HGNC Symbol;Acc:HGNC:28014]
ENSG00000232774	-2,6563586	-0,8504603	19,9618802	0,00145684	0,06080836	AL355916.1	novel transcript
ENSG00000102738	-0,6940263	4,72779226	19,9374553	0,0014627	0,06087932	MRPS31	mitochondrial ribosomal protein S31 [Source:HGNC Symbol;Acc:HGNC:16632]

ENSG00000137752	1,79032287	-0,2602087	19,8446446	0,00148526	0,06164264	CASP1	caspase 1 [Source:HGNC Symbol;Acc:HGNC:1499]
ENSG00000084110	-1,1841721	0,87805071	19,8236286	0,00149043	0,06168185	HAL	histidine ammonia-lyase [Source:HGNC Symbol;Acc:HGNC:4806]
ENSG00000119888	0,70240683	8,57690507	19,8011928	0,00149597	0,06173625	EPCAM	epithelial cell adhesion molecule [Source:HGNC Symbol;Acc:HGNC:11529]
ENSG00000083097	0,76161303	2,35464528	19,6576623	0,00153201	0,06204477	DOP1A	DOP1 leucine zipper like protein A [Source:HGNC Symbol;Acc:HGNC:21194]
ENSG00000100564	0,68938553	5,5053738	19,6834177	0,00152547	0,06204477	PIGH	phosphatidylinositol glycan anchor biosynthesis class H [Source:HGNC Symbol;Acc:HGNC:8964]
ENSG00000165861	0,78695427	2,83263407	19,7138048	0,00151779	0,06204477	ZFYVE1	zinc finger FYVE-type containing 1 [Source:HGNC Symbol;Acc:HGNC:13180]
ENSG00000168065	-1,6893078	1,6121949	19,6622676	0,00153084	0,06204477	SLC22A11	solute carrier family 22 member 11 [Source:HGNC Symbol;Acc:HGNC:18120]
ENSG00000172167	0,8420237	2,65346759	19,7394883	0,00151134	0,06204477	MTBP	MDM2 binding protein [Source:HGNC Symbol;Acc:HGNC:7417]
ENSG00000236809	-1,082763	1,19110061	19,6531064	0,00153318	0,06204477	SNX25P1	sorting nexin 25 pseudogene 1 [Source:HGNC Symbol;Acc:HGNC:41509]
ENSG00000250799	-0,7409219	5,62677673	19,7219458	0,00151574	0,06204477	PRODH2	proline dehydrogenase 2 [Source:HGNC Symbol;Acc:HGNC:17325]
ENSG00000108306	0,83827682	3,30161315	19,5481826	0,00156022	0,06235175	FBXL20	F-box and leucine rich repeat protein 20 [Source:HGNC Symbol;Acc:HGNC:24679]
ENSG00000123268	-0,7597781	3,94204154	19,5097589	0,00157027	0,06235175	ATF1	activating transcription factor 1 [Source:HGNC Symbol;Acc:HGNC:783]
ENSG00000138772	-0,8726139	3,46064923	19,5249802	0,00156628	0,06235175	ANXA3	annexin A3 [Source:HGNC Symbol;Acc:HGNC:541]
ENSG00000139496	-0,6580515	3,98186934	19,5083584	0,00157064	0,06235175	NUP58	nucleoporin 58 [Source:HGNC Symbol;Acc:HGNC:20261]
ENSG00000167900	-0,6450163	6,4454324	19,5860572	0,00155039	0,06235175	TK1	thymidine kinase 1 [Source:HGNC Symbol;Acc:HGNC:11830]
ENSG00000179965	-0,9619299	3,05097428	19,5170054	0,00156837	0,06235175	ZNF771	zinc finger protein 771 [Source:HGNC Symbol;Acc:HGNC:29653]
ENSG00000258655	-1,2427725	1,0944001	19,5670602	0,00155531	0,06235175	ARHGAP5-AS1	ARHGAP5 antisense RNA 1 (head to head) [Source:HGNC Symbol;Acc:HGNC:20279]
ENSG00000157326	-0,6588005	5,12116398	19,470325	0,00158067	0,06257979	DHRS4	dehydrogenase/reductase 4 [Source:HGNC Symbol;Acc:HGNC:16985]
ENSG00000104522	-0,6145571	6,3130725	19,3752856	0,00160607	0,06270276	GFUS	GDP-L-fucose synthase [Source:HGNC Symbol;Acc:HGNC:12390]
ENSG00000137601	-0,7729825	2,63466195	19,3047508	0,00162524	0,06270276	NEK1	NIMA related kinase 1 [Source:HGNC Symbol;Acc:HGNC:7744]
ENSG00000147274	-0,6059163	8,08270163	19,3045378	0,0016253	0,06270276	RBMX	RNA binding motif protein X-linked [Source:HGNC Symbol;Acc:HGNC:9910]
ENSG00000148803	-0,7203669	5,12797495	19,3819177	0,00160428	0,06270276	FUOM	fucose mutarotase [Source:HGNC Symbol;Acc:HGNC:24733]
ENSG00000151726	0,63036559	5,90602541	19,3139199	0,00162273	0,06270276	ACSL1	acyl-CoA synthetase long chain family member 1 [Source:HGNC Symbol;Acc:HGNC:3569]
ENSG00000154743	-0,7022977	3,39825214	19,3597056	0,00161028	0,06270276	TSEN2	tRNA splicing endonuclease subunit 2 [Source:HGNC Symbol;Acc:HGNC:28422]
ENSG00000167700	-0,9758608	4,7430069	19,3553263	0,00161146	0,06270276	MFSD3	major facilitator superfamily domain containing 3 [Source:HGNC Symbol;Acc:HGNC:25157]
ENSG00000173598	0,83702932	5,08079113	19,2994535	0,00162669	0,06270276	NUDT4	nudix hydrolase 4 [Source:HGNC Symbol;Acc:HGNC:8051]
ENSG00000176407	-0,589926	6,03241356	19,3921897	0,00160151	0,06270276	KCMF1	potassium channel modulatory factor 1 [Source:HGNC Symbol;Acc:HGNC:20589]
ENSG00000196693	0,8991583	3,38742278	19,3263734	0,00161933	0,06270276	ZNF33B	zinc finger protein 33B [Source:HGNC Symbol;Acc:HGNC:13097]
ENSG00000119547	1,33779418	2,17833683	19,2052924	0,00165275	0,06283122	ONECUT2	one cut homeobox 2 [Source:HGNC Symbol;Acc:HGNC:8139]
ENSG00000139132	1,19239563	1,67666777	19,2196608	0,00164874	0,06283122	FGD4	FYVE, RhoGEF and PH domain containing 4 [Source:HGNC Symbol;Acc:HGNC:19125]
ENSG00000161835	-0,8616333	2,65748921	19,2445231	0,00164183	0,06283122	TAMALIN	trafficking regulator and scaffold protein tamalin [Source:HGNC Symbol;Acc:HGNC:18707]
ENSG00000163281	0,81771479	3,18880261	19,2096497	0,00165154	0,06283122	GNPDA2	glucosamine-6-phosphate deaminase 2 [Source:HGNC Symbol;Acc:HGNC:21526]
ENSG00000164142	1,03959312	1,29856211	19,2216394	0,00164819	0,06283122	FAM160A1	family with sequence similarity 160 member A1 [Source:HGNC Symbol;Acc:HGNC:34237]
ENSG00000167286	-1,5722689	-0,0646628	19,1942995	0,00165583	0,06283122	CD3D	CD3d molecule [Source:HGNC Symbol;Acc:HGNC:1673]
ENSG00000183876	0,82266859	5,76355272	19,1632765	0,00166455	0,06299839	ARSI	arylsulfatase family member I [Source:HGNC Symbol;Acc:HGNC:32521]
ENSG00000040199	0,92773121	3,61999317	19,1379426	0,00167171	0,0631059	PHLPP2	PH domain and leucine rich repeat protein phosphatase 2 [Source:HGNC Symbol;Acc:HGNC:29149]
ENSG00000163624	0,79117628	2,86906594	19,1027093	0,00168173	0,06315779	CDS1	CDP-diacylglycerol synthase 1 [Source:HGNC Symbol;Acc:HGNC:1800]
ENSG00000204634	0,70178901	5,05294687	19,1114066	0,00167925	0,06315779	TBC1D8	TBC1 domain family member 8 [Source:HGNC Symbol;Acc:HGNC:17791]
ENSG00000154553	-0,753128	2,77209332	19,0795163	0,00168837	0,06324445	PDLIM3	PDZ and LIM domain 3 [Source:HGNC Symbol;Acc:HGNC:20767]
ENSG00000057593	-1,0736143	3,51119271	19,0370429	0,0017006	0,06331815	F7	coagulation factor VII [Source:HGNC Symbol;Acc:HGNC:3544]
ENSG00000104381	0,66446281	4,02696394	19,0276052	0,00170334	0,06331815	GDAP1	ganglioside induced differentiation associated protein 1 [Source:HGNC Symbol;Acc:HGNC:15968]
ENSG00000131435	-1,6072595	-0,1782357	19,0501375	0,00169682	0,06331815	PDLIM4	PDZ and LIM domain 4 [Source:HGNC Symbol;Acc:HGNC:16501]
ENSG00000122986	-2,4021373	-0,9278643	18,9394894	0,00172912	0,06411345	HVCN1	hydrogen voltage gated channel 1 [Source:HGNC Symbol;Acc:HGNC:28240]
ENSG00000110931	-0,6306793	4,39115954	18,9086538	0,00173825	0,06424617	CAMKK2	calcium/calmodulin dependent protein kinase kinase 2 [Source:HGNC Symbol;Acc:HGNC:1470]

ENSG00000127528	2,84519584	-1,4515113	18,8830238	0,00174589	0,06424617	KLF2	Kruppel like factor 2 [Source:HGNC Symbol;Acc:HGNC:6347]
ENSG00000196923	-0,6539541	5,5969376	18,8971557	0,00174168	0,06424617	PDLIM7	PDZ and LIM domain 7 [Source:HGNC Symbol;Acc:HGNC:22958]
ENSG00000196502	-0,5651872	7,04142679	18,8208925	0,00176458	0,06477061	SULT1A1	sulfotransferase family 1A member 1 [Source:HGNC Symbol;Acc:HGNC:11453]
ENSG00000104055	-2,6914043	-1,4009258	18,6855059	0,00180614	0,06563672	TGM5	transglutaminase 5 [Source:HGNC Symbol;Acc:HGNC:11781]
ENSG00000111961	-1,1496384	2,5322257	18,6993153	0,00180185	0,06563672	SASH1	SAM and SH3 domain containing 1 [Source:HGNC Symbol;Acc:HGNC:19182]
ENSG00000162878	0,68221904	6,32636649	18,6959337	0,0018029	0,06563672	PKDCC	protein kinase domain containing, cytoplasmic [Source:HGNC Symbol;Acc:HGNC:25123]
ENSG00000260367	-2,6126804	-1,3704417	18,7142766	0,00179721	0,06563672	AC109460.1	novel transcript, antisense to SPNS1
ENSG00000172273	0,60388325	4,85652636	18,6699189	0,00181101	0,06565009	HINFP	histone H4 transcription factor [Source:HGNC Symbol;Acc:HGNC:17850]
ENSG00000116288	-0,6183581	8,30333126	18,6336944	0,00182237	0,06589842	PARK7	Parkinsonism associated deglycase [Source:HGNC Symbol;Acc:HGNC:16369]
ENSG00000060642	-0,7698075	3,63307883	18,5799109	0,0018394	0,06634996	PIGV	phosphatidylinositol glycan anchor biosynthesis class V [Source:HGNC Symbol;Acc:HGNC:26031]
ENSG00000132437	-0,5680564	5,84165874	18,5552893	0,00184726	0,06646937	DDC	dopa decarboxylase [Source:HGNC Symbol;Acc:HGNC:2719]
ENSG00000213930	-0,6745696	3,70655806	18,5334818	0,00185425	0,06655717	GALT	galactose-1-phosphate uridylyltransferase [Source:HGNC Symbol;Acc:HGNC:4135]
ENSG00000014914	-2,4657181	-1,2683595	18,4964566	0,0018662	0,06665863	MTMR11	myotubularin related protein 11 [Source:HGNC Symbol;Acc:HGNC:24307]
ENSG00000139178	-0,7267008	4,21917858	18,5062589	0,00186303	0,06665863	C1RL	complement C1r subcomponent like [Source:HGNC Symbol;Acc:HGNC:21265]
ENSG00000157131	-0,8525117	3,87402585	18,4738118	0,00187356	0,06675817	C8A	complement C8 alpha chain [Source:HGNC Symbol;Acc:HGNC:1352]
ENSG00000099341	-0,582288	8,06792602	18,4178913	0,00189188	0,06724687	PSMD8	proteasome 26S subunit, non-ATPase 8 [Source:HGNC Symbol;Acc:HGNC:9566]
ENSG00000100129	-0,657621	6,89982539	18,3744081	0,00190627	0,06755593	EIF3L	eukaryotic translation initiation factor 3 subunit L [Source:HGNC Symbol;Acc:HGNC:18138]
ENSG00000102225	-0,6290619	6,26527742	18,3637516	0,00190982	0,06755593	CDK16	cyclin dependent kinase 16 [Source:HGNC Symbol;Acc:HGNC:8749]
ENSG00000204271	1,0911244	1,57381786	18,3485867	0,00191489	0,06757143	SPIN3	spindlin family member 3 [Source:HGNC Symbol;Acc:HGNC:27272]
ENSG00000131981	-0,6764841	6,40220508	18,3165459	0,00192564	0,0676593	LGALS3	galectin 3 [Source:HGNC Symbol;Acc:HGNC:6563]
ENSG00000158869	-0,9671979	1,82693629	18,3135732	0,00192664	0,0676593	FCER1G	Fc fragment of IgE receptor Ig [Source:HGNC Symbol;Acc:HGNC:3611]
ENSG00000273619	-0,7816013	2,39629645	18,2969523	0,00193225	0,06769359	AL121832.2	novel transcript, antisense to RPS21
ENSG00000024862	0,69982981	4,03034891	18,1838574	0,00197095	0,06810951	CCDC28A	coiled-coil domain containing 28A [Source:HGNC Symbol;Acc:HGNC:21098]
ENSG00000029725	-0,6477648	5,00845798	18,1805689	0,00197209	0,06810951	RABEP1	rabaptin, RAB GTPase binding effector protein 1 [Source:HGNC Symbol;Acc:HGNC:17677]
ENSG00000110172	-0,5546991	6,22370625	18,2168305	0,00195957	0,06810951	CHORDC1	cysteine and histidine rich domain containing 1 [Source:HGNC Symbol;Acc:HGNC:14525]
ENSG00000137409	-0,6722886	7,7026054	18,2192605	0,00195874	0,06810951	MTCH1	mitochondrial carrier 1 [Source:HGNC Symbol;Acc:HGNC:17586]
ENSG00000148672	0,7014775	8,92357149	18,1832883	0,00197115	0,06810951	GLUD1	glutamate dehydrogenase 1 [Source:HGNC Symbol;Acc:HGNC:4335]
ENSG00000159917	1,10118109	1,61431503	18,2276354	0,00195586	0,06810951	ZNF235	zinc finger protein 235 [Source:HGNC Symbol;Acc:HGNC:12866]
ENSG00000175606	0,59752838	5,45947287	18,1220251	0,00199252	0,06865251	TMEM70	transmembrane protein 70 [Source:HGNC Symbol;Acc:HGNC:26050]
ENSG00000145780	0,82737678	2,11180633	18,0921093	0,00200305	0,06869148	FEM1C	fem-1 homolog C [Source:HGNC Symbol;Acc:HGNC:16933]
ENSG00000237289	1,0465202	0,89785943	18,0968555	0,00200137	0,06869148	CKMT1A	creatine kinase, mitochondrial 1A [Source:HGNC Symbol;Acc:HGNC:31736]
ENSG00000149743	-0,7486581	4,16481165	18,031735	0,00202452	0,06926512	TRPT1	tRNA phosphotransferase 1 [Source:HGNC Symbol;Acc:HGNC:20316]
ENSG00000281406	-1,6431802	0,22779248	18,004712	0,00203422	0,06943438	BLACAT1	bladder cancer associated transcript 1 [Source:HGNC Symbol;Acc:HGNC:48597]
ENSG00000077254	0,65785461	4,30460418	17,9162901	0,00206636	0,06973223	USP33	ubiquitin specific peptidase 33 [Source:HGNC Symbol;Acc:HGNC:20059]
ENSG00000141738	0,56304106	6,34744982	17,9253117	0,00206305	0,06973223	GRB7	growth factor receptor bound protein 7 [Source:HGNC Symbol;Acc:HGNC:4567]
ENSG00000151414	1,08371287	1,9938529	17,9644367	0,00204878	0,06973223	NEK7	NIMA related kinase 7 [Source:HGNC Symbol;Acc:HGNC:13386]
ENSG00000165996	-0,6567533	4,46076204	17,9150532	0,00206681	0,06973223	HACD1	3-hydroxyacyl-CoA dehydratase 1 [Source:HGNC Symbol;Acc:HGNC:9639]
ENSG00000204852	-0,6965506	3,32637481	17,9418776	0,00205699	0,06973223	TCTN1	tectonic family member 1 [Source:HGNC Symbol;Acc:HGNC:26113]
ENSG00000104131	-0,8662764	5,77029773	17,8683776	0,00208403	0,07015118	EIF3J	eukaryotic translation initiation factor 3 subunit J [Source:HGNC Symbol;Acc:HGNC:3270]
ENSG00000148123	1,21140595	3,2072061	17,8307114	0,00209805	0,07046086	PLPPR1	phospholipid phosphatase related 1 [Source:HGNC Symbol;Acc:HGNC:25993]
ENSG00000081692	-0,6536843	5,37894132	17,8050708	0,00210767	0,07062132	JMJD4	jumonji domain containing 4 [Source:HGNC Symbol;Acc:HGNC:25724]
ENSG00000134057	0,58634929	7,8822111	17,7700134	0,0021209	0,07090197	CCNB1	cyclin B1 [Source:HGNC Symbol;Acc:HGNC:1579]
ENSG00000165533	0,71489552	3,58549362	17,7468453	0,00212969	0,07103355	TTC8	tetratricopeptide repeat domain 8 [Source:HGNC Symbol;Acc:HGNC:20087]
ENSG00000163444	-0,8183548	4,86076913	17,6943895	0,00214978	0,07154014	TMEM183A	transmembrane protein 183A [Source:HGNC Symbol;Acc:HGNC:20173]

ENSG00000011258	0,64105177	3,12295435	17,6448219	0,00216897	0,07165709	MBTD1	mbt domain containing 1 [Source:HGNC Symbol;Acc:HGNC:19866]
ENSG00000141753	-0,6038849	6,88027079	17,6592359	0,00216337	0,07165709	IGFBP4	insulin like growth factor binding protein 4 [Source:HGNC Symbol;Acc:HGNC:5473]
ENSG00000180535	-0,850583	4,17742666	17,6436335	0,00216943	0,07165709	BHLHA15	basic helix-loop-helix family member a15 [Source:HGNC Symbol;Acc:HGNC:22265]
ENSG00000196387	0,82621581	2,76233604	17,6227501	0,00217758	0,07165709	ZNF140	zinc finger protein 140 [Source:HGNC Symbol;Acc:HGNC:12925]
ENSG00000256269	-0,5473461	6,04271519	17,6221523	0,00217782	0,07165709	HMBS	hydroxymethylbilane synthase [Source:HGNC Symbol;Acc:HGNC:4982]
ENSG00000155111	0,91718727	2,98641128	17,5948472	0,00218853	0,07184782	CDK19	cyclin dependent kinase 19 [Source:HGNC Symbol;Acc:HGNC:19338]
ENSG00000165626	-0,6739434	4,03720481	17,54777	0,00220716	0,07229679	BEND7	BEN domain containing 7 [Source:HGNC Symbol;Acc:HGNC:23514]
ENSG00000277972	-0,6092687	6,06313204	17,4987673	0,00222675	0,07277537	CISD3	CDGSH iron sulfur domain 3 [Source:HGNC Symbol;Acc:HGNC:27578]
ENSG00000154269	-0,678427	4,74876502	17,4750389	0,00223631	0,07292478	ENPP3	ectonucleotide pyrophosphatase/phosphodiesterase 3 [Source:HGNC Symbol;Acc:HGNC:3358]
ENSG00000176014	-1,0267185	4,60506497	17,4622389	0,00224149	0,07293089	TUBB6	tubulin beta 6 class V [Source:HGNC Symbol;Acc:HGNC:20776]
ENSG00000057757	-0,6635929	4,91341041	17,4245788	0,00225682	0,07326633	PITHD1	PITH domain containing 1 [Source:HGNC Symbol;Acc:HGNC:25022]
ENSG00000167779	-0,6934547	4,08391721	17,3804953	0,00227492	0,07369021	IGFBP6	insulin like growth factor binding protein 6 [Source:HGNC Symbol;Acc:HGNC:5475]
ENSG00000137842	-0,6167859	4,13816643	17,319111	0,00230042	0,07435138	TMEM62	transmembrane protein 62 [Source:HGNC Symbol;Acc:HGNC:26269]
ENSG00000185808	-0,8503023	3,23847077	17,3059566	0,00230593	0,07436494	PIGP	phosphatidylinositol glycan anchor biosynthesis class P [Source:HGNC Symbol;Acc:HGNC:3046]
ENSG00000112079	0,65050832	4,49013311	17,2729424	0,00231983	0,07464842	STK38	serine/threonine kinase 38 [Source:HGNC Symbol;Acc:HGNC:17847]
ENSG00000151332	0,69549547	6,13960613	17,223347	0,0023409	0,07516097	MBIP	MAP3K12 binding inhibitory protein 1 [Source:HGNC Symbol;Acc:HGNC:20427]
ENSG00000140853	0,7553565	1,93495601	17,1878791	0,00235612	0,07531838	NLRC5	NLR family CARD domain containing 5 [Source:HGNC Symbol;Acc:HGNC:29933]
ENSG00000267002	1,30728709	1,7453112	17,1914071	0,0023546	0,07531838	AC060780.1	novel transcript
ENSG00000008277	0,72609288	2,4687831	17,1410311	0,0023764	0,07550418	ADAM22	ADAM metallopeptidase domain 22 [Source:HGNC Symbol;Acc:HGNC:201]
ENSG00000013588	-2,1495992	1,81269149	17,1476593	0,00237351	0,07550418	GPRC5A	G protein-coupled receptor class C group 5 member A [Source:HGNC Symbol;Acc:HGNC:9836]
ENSG00000089091	1,26086934	0,77509834	17,1267953	0,0023826	0,07550418	DZANK1	double zinc ribbon and ankyrin repeat domains 1 [Source:HGNC Symbol;Acc:HGNC:15858]
ENSG00000105246	-2,1052889	-1,4186274	17,1352563	0,00237891	0,07550418	EBI3	Epstein-Barr virus induced 3 [Source:HGNC Symbol;Acc:HGNC:3129]
ENSG00000134744	0,65940945	4,92609537	17,0741944	0,0024057	0,0755365	TUT4	terminal uridylyl transferase 4 [Source:HGNC Symbol;Acc:HGNC:28981]
ENSG00000135637	-0,7353058	3,24655491	17,0813612	0,00240254	0,0755365	CCDC142	coiled-coil domain containing 142 [Source:HGNC Symbol;Acc:HGNC:25889]
ENSG00000162599	0,82447378	2,2546565	17,0539912	0,00241464	0,0755365	NFIA	nuclear factor I A [Source:HGNC Symbol;Acc:HGNC:7784]
ENSG00000186466	-1,3282236	-0,0063143	17,0901481	0,00239867	0,0755365	AQP7P1	aquaporin 7 pseudogene 1 [Source:HGNC Symbol;Acc:HGNC:32048]
ENSG00000204228	-0,6703921	3,75129659	17,1088253	0,00239046	0,0755365	HSD17B8	hydroxysteroid 17-beta dehydrogenase 8 [Source:HGNC Symbol;Acc:HGNC:3554]
ENSG00000223572	1,55196109	-0,2096576	17,0650743	0,00240973	0,0755365	CKMT1A	creatine kinase, mitochondrial 1A [Source:HGNC Symbol;Acc:HGNC:31736]
ENSG00000020577	-0,6759324	2,75438816	16,9979632	0,00243966	0,07578747	SAMD4A	sterile alpha motif domain containing 4A [Source:HGNC Symbol;Acc:HGNC:23023]
ENSG00000102119	-0,8145299	4,63874558	16,9666094	0,0024538	0,07578747	EMD	emerin [Source:HGNC Symbol;Acc:HGNC:3331]
ENSG00000129514	0,59165696	5,44503107	17,0029158	0,00243744	0,07578747	FOXA1	forkhead box A1 [Source:HGNC Symbol;Acc:HGNC:5021]
ENSG00000142494	-0,6511382	5,86056862	17,0202509	0,00242967	0,07578747	SLC47A1	solute carrier family 47 member 1 [Source:HGNC Symbol;Acc:HGNC:25588]
ENSG00000174327	-0,7508706	4,1204867	16,955177	0,00245898	0,07578747	SLC16A13	solute carrier family 16 member 13 [Source:HGNC Symbol;Acc:HGNC:31037]
ENSG00000182010	0,75017961	2,49133481	16,9561637	0,00245853	0,07578747	RTKN2	rhotekin 2 [Source:HGNC Symbol;Acc:HGNC:19364]
ENSG00000254233	-0,845943	2,65773599	16,9695436	0,00245247	0,07578747	LINC02365	long intergenic non-protein coding RNA 2365 [Source:HGNC Symbol;Acc:HGNC:53285]
ENSG00000159224	-1,5268513	-0,3350698	16,9247124	0,00247285	0,07605446	GIP	gastric inhibitory polypeptide [Source:HGNC Symbol;Acc:HGNC:4270]
ENSG00000126603	2,18326219	-0,5956816	16,9014888	0,00248349	0,07611278	GLIS2	GLIS family zinc finger 2 [Source:HGNC Symbol;Acc:HGNC:29450]
ENSG00000134215	0,92122138	3,48710996	16,8978333	0,00248517	0,07611278	VAV3	vav guanine nucleotide exchange factor 3 [Source:HGNC Symbol;Acc:HGNC:12659]
ENSG00000214290	-0,6938205	2,75638781	16,8657775	0,00249995	0,07640544	COLCA2	colorectal cancer associated 2 [Source:HGNC Symbol;Acc:HGNC:26978]
ENSG00000175711	-0,6500949	2,98881845	16,8432128	0,00251042	0,07656533	B3GNTL1	UDP-GlcNAc:betaGal beta-1,3-N-acetylglucosaminyltransferase like 1 [Source:HGNC Symbol;Acc:HGNC:21727]
ENSG00000196422	0,61025177	4,30311093	16,8317289	0,00251577	0,07656866	PPP1R26	protein phosphatase 1 regulatory subunit 26 [Source:HGNC Symbol;Acc:HGNC:29089]
ENSG00000163959	-0,8487364	2,98169873	16,7868636	0,00253681	0,07704839	SLC51A	solute carrier family 51 subunit alpha [Source:HGNC Symbol;Acc:HGNC:29955]
ENSG00000178802	-0,6526136	5,26627237	16,7501338	0,00255419	0,07732733	MPI	mannose phosphate isomerase [Source:HGNC Symbol;Acc:HGNC:7216]
ENSG00000231274	-2,2270058	-0,663191	16,7451103	0,00255658	0,07732733	SBK3	SH3 domain binding kinase family member 3 [Source:HGNC Symbol;Acc:HGNC:44121]

ENSG00000168743	0,78693055	2,86765254	16,7243068	0,0025665	0,077467	NPNT	nephronectin [Source:HGNC Symbol;Acc:HGNC:27405]
ENSG00000090534	-0,6516794	3,82870854	16,6954827	0,00258033	0,07772366	THPO	thrombopoietin [Source:HGNC Symbol;Acc:HGNC:11795]
ENSG00000156413	0,70072018	4,46443521	16,6621909	0,0025964	0,07785173	FUT6	fucosyltransferase 6 [Source:HGNC Symbol;Acc:HGNC:4017]
ENSG00000170946	-0,7896611	1,94037331	16,6503518	0,00260215	0,07785173	DNAJC24	DnaJ heat shock protein family (Hsp40) member C24 [Source:HGNC Symbol;Acc:HGNC:26979]
ENSG00000183337	-0,9865803	3,74989692	16,6426617	0,00260589	0,07785173	BCOR	BCL6 corepressor [Source:HGNC Symbol;Acc:HGNC:20893]
ENSG00000274213	-1,7888369	-1,0316079	16,6499008	0,00260237	0,07785173	AC015912.3	novel transcript
ENSG00000088367	-0,5880791	6,18796884	16,603829	0,00262489	0,07794102	EPB41L1	erythrocyte membrane protein band 4.1 like 1 [Source:HGNC Symbol;Acc:HGNC:3378]
ENSG00000116194	0,93611804	2,64248736	16,6121354	0,00262081	0,07794102	ANGPTL1	angiopoietin like 1 [Source:HGNC Symbol;Acc:HGNC:489]
ENSG00000187630	-0,6094495	5,26945379	16,6142588	0,00261977	0,07794102	DHRS4L2	dehydrogenase/reductase 4 like 2 [Source:HGNC Symbol;Acc:HGNC:19731]
ENSG00000089053	0,57296726	8,53840469	16,5479195	0,00265253	0,0781267	ANAPC5	anaphase promoting complex subunit 5 [Source:HGNC Symbol;Acc:HGNC:15713]
ENSG00000105186	0,62333441	4,57038189	16,5643311	0,00264438	0,0781267	ANKRD27	ankyrin repeat domain 27 [Source:HGNC Symbol;Acc:HGNC:25310]
ENSG00000141854	0,64807711	3,10517692	16,57662	0,0026383	0,0781267	MISP3	MISP family member 3 [Source:HGNC Symbol;Acc:HGNC:26963]
ENSG00000173894	0,57481928	4,63736729	16,548667	0,00265216	0,0781267	CBX2	chromobox 2 [Source:HGNC Symbol;Acc:HGNC:1552]
ENSG00000121957	0,71679613	3,65700752	16,530384	0,00266128	0,07822652	GPSM2	G protein signaling modulator 2 [Source:HGNC Symbol;Acc:HGNC:29501]
ENSG00000184277	0,67737706	5,90690938	16,5046716	0,00267416	0,0784474	TM2D3	TM2 domain containing 3 [Source:HGNC Symbol;Acc:HGNC:24128]
ENSG00000115758	0,5859647	9,09914408	16,4556892	0,00269892	0,0789626	ODC1	ornithine decarboxylase 1 [Source:HGNC Symbol;Acc:HGNC:8109]
ENSG00000213463	-0,7439812	2,6458396	16,4485804	0,00270253	0,0789626	SYNJ2BP	synaptojanin 2 binding protein [Source:HGNC Symbol;Acc:HGNC:18955]
ENSG00000151806	0,54825232	5,70171843	16,4035277	0,00272559	0,07947727	GUF1	GUF1 homolog, GTPase [Source:HGNC Symbol;Acc:HGNC:25799]
ENSG00000115828	-1,7162629	0,06794125	16,3454718	0,00275565	0,08019386	QPCT	glutaminyl-peptide cyclotransferase [Source:HGNC Symbol;Acc:HGNC:9753]
ENSG00000117707	0,81773967	4,25072504	16,3299287	0,00276377	0,08027019	PROX1	prospero homeobox 1 [Source:HGNC Symbol;Acc:HGNC:9459]
ENSG00000126821	-0,994953	2,85915892	16,2881332	0,00278574	0,08074785	SGPP1	sphingosine-1-phosphate phosphatase 1 [Source:HGNC Symbol;Acc:HGNC:17720]
ENSG00000069702	0,87564376	6,81921301	16,263433	0,00279883	0,0808065	TGFBR3	transforming growth factor beta receptor 3 [Source:HGNC Symbol;Acc:HGNC:11774]
ENSG00000109107	-0,5684793	5,88012337	16,2649327	0,00279803	0,0808065	ALDOC	aldolase, fructose-bisphosphate C [Source:HGNC Symbol;Acc:HGNC:418]
ENSG00000067704	0,59582559	7,85890046	16,2428931	0,00280977	0,08096232	IARS2	isoleucyl-tRNA synthetase 2, mitochondrial [Source:HGNC Symbol;Acc:HGNC:29685]
ENSG00000066739	0,9306782	2,59661165	16,2284254	0,0028175	0,08099733	ATG2B	autophagy related 2B [Source:HGNC Symbol;Acc:HGNC:20187]
ENSG00000155363	0,71515427	5,71866192	16,2199072	0,00282207	0,08099733	MOV10	Mov10 RISC complex RNA helicase [Source:HGNC Symbol;Acc:HGNC:7200]
ENSG00000163235	0,62296613	3,69667263	16,1912837	0,00283748	0,08127199	TGFA	transforming growth factor alpha [Source:HGNC Symbol;Acc:HGNC:11765]
ENSG00000176155	-0,7038124	4,93280311	16,1755933	0,00284598	0,08127199	CCDC57	coiled-coil domain containing 57 [Source:HGNC Symbol;Acc:HGNC:27564]
ENSG00000181904	-0,6890281	2,77170861	16,161019	0,00285389	0,08127199	C5orf24	chromosome 5 open reading frame 24 [Source:HGNC Symbol;Acc:HGNC:26746]
ENSG00000181997	-1,424975	-0,0618246	16,1665351	0,00285089	0,08127199	AQP7P2	aquaporin 7 pseudogene 2 [Source:HGNC Symbol;Acc:HGNC:32049]
ENSG00000135317	0,61257594	5,97249665	16,0954938	0,00288982	0,0820373	SNX14	sorting nexin 14 [Source:HGNC Symbol;Acc:HGNC:14977]
ENSG00000198843	-0,6014793	6,63253044	16,0915489	0,002892	0,0820373	SELENOT	selenoprotein T [Source:HGNC Symbol;Acc:HGNC:18136]
ENSG00000139173	-1,0082432	1,69486039	16,071929	0,00290287	0,08218618	TMEM117	transmembrane protein 117 [Source:HGNC Symbol;Acc:HGNC:25308]
ENSG00000100485	0,97809028	2,54860621	16,0381394	0,00292172	0,08255971	SOS2	SOS Ras/Rho guanine nucleotide exchange factor 2 [Source:HGNC Symbol;Acc:HGNC:11188]
ENSG00000100592	0,85582542	3,58852029	15,974914	0,00295738	0,08340614	DAAM1	dishevelled associated activator of morphogenesis 1 [Source:HGNC Symbol;Acc:HGNC:18142]
ENSG00000177700	-0,6764803	7,27188404	15,9493475	0,00297195	0,08365561	POLR2L	RNA polymerase II, I and III subunit L [Source:HGNC Symbol;Acc:HGNC:9199]
ENSG00000005486	0,58737159	6,51222325	15,8698701	0,00301782	0,08367148	RHBDD2	rhomboid domain containing 2 [Source:HGNC Symbol;Acc:HGNC:23082]
ENSG00000011198	0,74521049	3,41393593	15,8493603	0,00302979	0,08367148	ABHD5	abhydrolase domain containing 5, lysophosphatidic acid acyltransferase [Source:HGNC Symbol;Acc:HGNC:21396]
ENSG00000068438	-0,5862813	5,80094048	15,91584	0,00299118	0,08367148	FTSJ1	FtsJ RNA 2'-O-methyltransferase 1 [Source:HGNC Symbol;Acc:HGNC:13254]
ENSG00000119396	-0,7318993	5,22318575	15,8707548	0,0030173	0,08367148	RAB14	RAB14, member RAS oncogene family [Source:HGNC Symbol;Acc:HGNC:16524]
ENSG00000136243	-0,5804376	4,03062597	15,8516045	0,00302848	0,08367148	NUP42	nucleoporin 42 [Source:HGNC Symbol;Acc:HGNC:17010]
ENSG00000137819	-2,0585904	-1,4851633	15,9343185	0,00298056	0,08367148	PAQR5	progestin and adipoQ receptor family member 5 [Source:HGNC Symbol;Acc:HGNC:29645]
ENSG00000173930	0,64777961	4,34316953	15,8939638	0,00300382	0,08367148	SLCO4C1	solute carrier organic anion transporter family member 4C1 [Source:HGNC Symbol;Acc:HGNC:23612]
ENSG00000175602	-0,7044631	3,51767335	15,8941888	0,00300369	0,08367148	CCDC85B	coiled-coil domain containing 85B [Source:HGNC Symbol;Acc:HGNC:24926]

ENSG00000177169	0,69037532	3,87778716	15,8525743	0,00302791	0,08367148	ULK1	unc-51 like autophagy activating kinase 1 [Source:HGNC Symbol;Acc:HGNC:12558]
ENSG00000251022	0,75818666	2,70266721	15,8707218	0,00301732	0,08367148	THAP9-AS1	THAP9 antisense RNA 1 [Source:HGNC Symbol;Acc:HGNC:44172]
ENSG00000136144	-0,5937109	3,98669647	15,8132567	0,00305101	0,08409169	RCBTB1	RCC1 and BTB domain containing protein 1 [Source:HGNC Symbol;Acc:HGNC:18243]
ENSG00000240972	-0,5695054	6,32977115	15,8039372	0,00305652	0,08409169	MIF	macrophage migration inhibitory factor [Source:HGNC Symbol;Acc:HGNC:7097]
ENSG00000166068	-0,7316152	2,2806341	15,7675689	0,00307813	0,0843684	SPRED1	sprouty related EVH1 domain containing 1 [Source:HGNC Symbol;Acc:HGNC:20249]
ENSG00000169432	0,88070648	3,91425381	15,7736624	0,00307449	0,0843684	SCN9A	sodium voltage-gated channel alpha subunit 9 [Source:HGNC Symbol;Acc:HGNC:10597]
ENSG00000106631	-2,7835377	-1,8757729	15,7394852	0,00309494	0,08451212	MYL7	myosin light chain 7 [Source:HGNC Symbol;Acc:HGNC:21719]
ENSG00000216588	-1,3712636	-0,1165264	15,7488503	0,00308932	0,08451212	IGSF23	immunoglobulin superfamily member 23 [Source:HGNC Symbol;Acc:HGNC:40040]
ENSG00000124299	-0,774549	6,50758302	15,7149014	0,00310975	0,08451309	PEPD	peptidase D [Source:HGNC Symbol;Acc:HGNC:8840]
ENSG00000143621	-0,6986415	8,47349439	15,7010783	0,00311812	0,08451309	ILF2	interleukin enhancer binding factor 2 [Source:HGNC Symbol;Acc:HGNC:6037]
ENSG00000180340	2,36001027	-0,6226904	15,706796	0,00311465	0,08451309	FZD2	frizzled class receptor 2 [Source:HGNC Symbol;Acc:HGNC:4040]
ENSG00000204248	2,76127289	-1,610107	15,7058972	0,0031152	0,08451309	COL11A2	collagen type XI alpha 2 chain [Source:HGNC Symbol;Acc:HGNC:2187]
ENSG00000165478	0,76321938	3,73614585	15,6910918	0,00312418	0,08452055	HEPACAM	hepatic and glial cell adhesion molecule [Source:HGNC Symbol;Acc:HGNC:26361]
ENSG00000137288	-0,5241631	6,9093729	15,666628	0,00313908	0,08453018	UQCC2	ubiquinol-cytochrome c reductase complex assembly factor 2 [Source:HGNC Symbol;Acc:HGNC:21237]
ENSG00000149564	-1,0313151	5,14474318	15,669712	0,0031372	0,08453018	ESAM	endothelial cell adhesion molecule [Source:HGNC Symbol;Acc:HGNC:17474]
ENSG00000261455	-1,0540043	1,24919823	15,6620375	0,00314189	0,08453018	LINC01003	long intergenic non-protein coding RNA 1003 [Source:HGNC Symbol;Acc:HGNC:48957]
ENSG00000117305	-0,6670845	4,76773269	15,6405977	0,00315504	0,08472796	HMGCL	3-hydroxy-3-methylglutaryl-CoA lyase [Source:HGNC Symbol;Acc:HGNC:5005]
ENSG00000177565	0,60659222	3,74283948	15,6296348	0,00316179	0,08475344	TBL1XR1	TBL1X receptor 1 [Source:HGNC Symbol;Acc:HGNC:29529]
ENSG00000165917	1,77917206	-0,9976913	15,60946	0,00317426	0,08477259	RAPSN	receptor associated protein of the synapse [Source:HGNC Symbol;Acc:HGNC:9863]
ENSG00000176105	-0,5410984	5,35994032	15,6003436	0,00317992	0,08477259	YES1	YES proto-oncogene 1, Src family tyrosine kinase [Source:HGNC Symbol;Acc:HGNC:12841]
ENSG00000237232	-2,6686927	-2,2260922	15,6102117	0,0031738	0,08477259	ZNF295-AS1	ZNF295 antisense RNA 1 [Source:HGNC Symbol;Acc:HGNC:23130]
ENSG00000144746	0,6488526	6,07459529	15,5747574	0,00319585	0,08484466	ARL6IP5	ADP ribosylation factor like GTPase 6 interacting protein 5 [Source:HGNC Symbol;Acc:HGNC:16937]
ENSG00000167671	-0,5702586	6,21235566	15,5587792	0,00320585	0,08484466	UBXN6	UBX domain protein 6 [Source:HGNC Symbol;Acc:HGNC:14928]
ENSG00000180354	0,79846676	2,58092471	15,5618913	0,0032039	0,08484466	MTURN	maturin, neural progenitor differentiation regulator homolog [Source:HGNC Symbol;Acc:HGNC:25457]
ENSG00000180644	-1,4002132	0,85946292	15,5848217	0,00318957	0,08484466	PRF1	perforin 1 [Source:HGNC Symbol;Acc:HGNC:9360]
ENSG00000065491	0,64848115	4,18676364	15,5321515	0,0032226	0,0850053	TBC1D22B	TBC1 domain family member 22B [Source:HGNC Symbol;Acc:HGNC:21602]
ENSG00000152454	0,76325405	2,48067407	15,5266335	0,00322608	0,0850053	ZNF256	zinc finger protein 256 [Source:HGNC Symbol;Acc:HGNC:13049]
ENSG00000244187	-0,664136	5,56319949	15,5214281	0,00322938	0,0850053	TMEM141	transmembrane protein 141 [Source:HGNC Symbol;Acc:HGNC:28211]
ENSG00000101335	-0,5921723	4,50398651	15,5021819	0,00324158	0,08517309	MYL9	myosin light chain 9 [Source:HGNC Symbol;Acc:HGNC:15754]
ENSG00000149735	-0,7863374	5,16485868	15,486788	0,00325138	0,08527727	GPHA2	glycoprotein hormone subunit alpha 2 [Source:HGNC Symbol;Acc:HGNC:18054]
ENSG00000011376	0,64720672	5,69127528	15,413317	0,00329867	0,08557427	LARS2	leucyl-tRNA synthetase 2, mitochondrial [Source:HGNC Symbol;Acc:HGNC:17095]
ENSG00000068489	0,71003861	4,96105591	15,4272268	0,00328965	0,08557427	PRR11	proline rich 11 [Source:HGNC Symbol;Acc:HGNC:25619]
ENSG00000100628	-1,4228177	3,3476521	15,4143008	0,00329803	0,08557427	ASB2	ankyrin repeat and SOCS box containing 2 [Source:HGNC Symbol;Acc:HGNC:16012]
ENSG00000119661	-0,8794216	1,5736024	15,4433969	0,00327921	0,08557427	DNAL1	dynein axonemal light chain 1 [Source:HGNC Symbol;Acc:HGNC:23247]
ENSG00000221944	-0,7392955	1,74429237	15,4055592	0,00330371	0,08557427	TIGD1	tigger transposable element derived 1 [Source:HGNC Symbol;Acc:HGNC:14523]
ENSG00000242265	0,77627419	9,81965048	15,4461616	0,00327743	0,08557427	PEG10	paternally expressed 10 [Source:HGNC Symbol;Acc:HGNC:14005]
ENSG00000247134	1,00903241	0,97353734	15,4174906	0,00329596	0,08557427	AC090204.1	novel transcript
ENSG00000100097	-0,5350656	7,53039887	15,3467384	0,00334224	0,08623952	LGALS1	galectin 1 [Source:HGNC Symbol;Acc:HGNC:6561]
ENSG00000111906	-0,5556016	5,701672	15,3551683	0,00333669	0,08623952	HDDC2	HD domain containing 2 [Source:HGNC Symbol;Acc:HGNC:21078]
ENSG00000141524	-0,7035206	6,45185157	15,3304599	0,003353	0,08623952	TMC6	transmembrane channel like 6 [Source:HGNC Symbol;Acc:HGNC:18021]
ENSG00000172183	-1,0207224	2,90543192	15,33778	0,00334816	0,08623952	ISG20	interferon stimulated exonuclease gene 20 [Source:HGNC Symbol;Acc:HGNC:6130]
ENSG00000172927	-1,731492	-0,4853112	15,267307	0,00339515	0,08717016	MYEOV	myeloma overexpressed [Source:HGNC Symbol;Acc:HGNC:7563]
ENSG00000260086	-1,4964088	-0,7387389	15,2567791	0,00340224	0,08719893	AC007611.1	novel transcript
ENSG00000120705	-0,5391401	7,49997163	15,2200944	0,00342709	0,08740484	ETF1	eukaryotic translation termination factor 1 [Source:HGNC Symbol;Acc:HGNC:3477]

ENSG00000127863	0,58075483	6,65824644	15,2184214	0,00342823	0,08740484	TNFRSF19	TNF receptor superfamily member 19 [Source:HGNC Symbol;Acc:HGNC:11915]
ENSG00000154429	0,81390265	2,52969081	15,2230081	0,00342511	0,08740484	CCSAP	centriole, cilia and spindle associated protein [Source:HGNC Symbol;Acc:HGNC:29578]
ENSG00000115239	-0,6399567	3,9357241	15,1873055	0,00344948	0,08764087	ASB3	ankyrin repeat and SOCS box containing 3 [Source:HGNC Symbol;Acc:HGNC:16013]
ENSG00000167555	1,11320059	0,41854696	15,1918657	0,00344636	0,08764087	ZNF528	zinc finger protein 528 [Source:HGNC Symbol;Acc:HGNC:29384]
ENSG00000083807	-0,6982867	3,35717156	15,176621	0,00345682	0,08767477	SLC27A5	solute carrier family 27 member 5 [Source:HGNC Symbol;Acc:HGNC:10999]
ENSG00000181894	0,9031266	2,52382582	15,1626678	0,00346643	0,08776611	ZNF329	zinc finger protein 329 [Source:HGNC Symbol;Acc:HGNC:14209]
ENSG00000134030	0,6117207	4,7408123	15,1396703	0,00348234	0,08786433	CTIF	cap binding complex dependent translation initiation factor [Source:HGNC Symbol;Acc:HGNC:23925]
ENSG00000165689	-0,6321246	5,83795417	15,1441183	0,00347925	0,08786433	ENTR1	endosome associated trafficking regulator 1 [Source:HGNC Symbol;Acc:HGNC:10667]
ENSG00000232533	-0,836693	2,11384075	15,1123584	0,00350134	0,08819163	AC093673.1	novel transcript
ENSG00000162433	0,50308597	6,22262458	15,0830115	0,00352191	0,08855697	AK4	adenylate kinase 4 [Source:HGNC Symbol;Acc:HGNC:363]
ENSG00000125967	-0,5512647	5,40025741	14,9949636	0,00358451	0,08928713	NECAB3	N-terminal EF-hand calcium binding protein 3 [Source:HGNC Symbol;Acc:HGNC:15851]
ENSG00000163507	0,56927577	4,01157825	15,0169203	0,00356877	0,08928713	CIP2A	cellular inhibitor of PP2A [Source:HGNC Symbol;Acc:HGNC:29302]
ENSG00000164896	-0,8823978	5,04696622	14,9906326	0,00358762	0,08928713	FASTK	Fas activated serine/threonine kinase [Source:HGNC Symbol;Acc:HGNC:24676]
ENSG00000166086	-0,7087945	2,21324577	14,9945145	0,00358483	0,08928713	JAM3	junctional adhesion molecule 3 [Source:HGNC Symbol;Acc:HGNC:15532]
ENSG00000173258	1,0368007	1,43135267	15,0101224	0,00357363	0,08928713	ZNF483	zinc finger protein 483 [Source:HGNC Symbol;Acc:HGNC:23384]
ENSG00000184545	-0,7796033	1,56820747	15,0306004	0,00355901	0,08928713	DUSP8	dual specificity phosphatase 8 [Source:HGNC Symbol;Acc:HGNC:3074]
ENSG00000105516	0,91480972	2,01723565	14,9647414	0,0036063	0,0895995	DBP	D-box binding PAR bZIP transcription factor [Source:HGNC Symbol;Acc:HGNC:2697]
ENSG00000140406	-0,7627382	3,16762335	14,9248705	0,00363531	0,09016681	TLNRD1	talin rod domain containing 1 [Source:HGNC Symbol;Acc:HGNC:13519]
ENSG00000056586	0,77161115	3,34226867	14,8996661	0,00365379	0,0904676	RC3H2	ring finger and CCCH-type domains 2 [Source:HGNC Symbol;Acc:HGNC:21461]
ENSG00000147679	-0,768041	3,0114583	14,8830845	0,00366602	0,0904676	UTP23	UTP23 small subunit processome component [Source:HGNC Symbol;Acc:HGNC:28224]
ENSG00000162600	0,60216315	3,28184898	14,8875028	0,00366275	0,0904676	OMA1	OMA1 zinc metallopeptidase [Source:HGNC Symbol;Acc:HGNC:29661]
ENSG00000120742	-0,6269193	6,62331806	14,8167697	0,0037154	0,0904818	SERP1	stress associated endoplasmic reticulum protein 1 [Source:HGNC Symbol;Acc:HGNC:10759]
ENSG00000124171	0,75892136	2,2816127	14,8272896	0,00370751	0,0904818	PARD6B	par-6 family cell polarity regulator beta [Source:HGNC Symbol;Acc:HGNC:16245]
ENSG00000133059	0,6272024	2,72497876	14,8145546	0,00371706	0,0904818	DSTYK	dual serine/threonine and tyrosine protein kinase [Source:HGNC Symbol;Acc:HGNC:29043]
ENSG00000137124	0,64463312	8,36692258	14,8262815	0,00370826	0,0904818	ALDH1B1	aldehyde dehydrogenase 1 family member B1 [Source:HGNC Symbol;Acc:HGNC:407]
ENSG00000139410	-0,6827763	2,42853723	14,851928	0,00368912	0,0904818	SDSL	serine dehydratase like [Source:HGNC Symbol;Acc:HGNC:30404]
ENSG00000144827	-0,5659635	4,68890794	14,8603589	0,00368285	0,0904818	ABHD10	abhydrolase domain containing 10, depalmitoylase [Source:HGNC Symbol;Acc:HGNC:25656]
ENSG00000164284	-0,6200451	3,40965864	14,8737964	0,00367288	0,0904818	GRPEL2	GrpE like 2, mitochondrial [Source:HGNC Symbol;Acc:HGNC:21060]
ENSG00000214309	-1,1180419	0,97575951	14,8075426	0,00372233	0,0904818	MBLAC1	metallo-beta-lactamase domain containing 1 [Source:HGNC Symbol;Acc:HGNC:22180]
ENSG00000235847	-1,2264965	0,38034646	14,8410925	0,00369719	0,0904818	LDHAP7	lactate dehydrogenase A pseudogene 7 [Source:HGNC Symbol;Acc:HGNC:23144]
ENSG00000163728	0,73455716	5,97083017	14,7954118	0,00373147	0,09053246	TTC14	tetratricopeptide repeat domain 14 [Source:HGNC Symbol;Acc:HGNC:24697]
ENSG00000172296	0,78098728	5,30106053	14,7883473	0,00373681	0,09053246	SPTLC3	serine palmitoyltransferase long chain base subunit 3 [Source:HGNC Symbol;Acc:HGNC:16253]
ENSG00000006740	0,77873372	1,48881372	14,7635912	0,00375559	0,09083667	ARHGAP44	Rho GTPase activating protein 44 [Source:HGNC Symbol;Acc:HGNC:29096]
ENSG00000147475	-0,7211615	5,25500029	14,7380287	0,00377509	0,09102825	ERLIN2	ER lipid raft associated 2 [Source:HGNC Symbol;Acc:HGNC:1356]
ENSG00000267680	0,82040404	2,65488491	14,7368845	0,00377597	0,09102825	ZNF224	zinc finger protein 224 [Source:HGNC Symbol;Acc:HGNC:13017]
ENSG00000072134	-0,6162041	3,08264267	14,6935497	0,00380933	0,09167417	EPN2	epsin 2 [Source:HGNC Symbol;Acc:HGNC:18639]
ENSG00000105755	-0,542591	5,36495802	14,6761074	0,00382286	0,09167417	ETHE1	ETHE1 persulfide dioxygenase [Source:HGNC Symbol;Acc:HGNC:23287]
ENSG00000128228	-0,6711563	7,09984294	14,6696766	0,00382786	0,09167417	SDF2L1	stromal cell derived factor 2 like 1 [Source:HGNC Symbol;Acc:HGNC:10676]
ENSG00000271270	-1,3757941	0,14187901	14,6743819	0,0038242	0,09167417	TMCC1-AS1	TMCC1 antisense RNA 1 (head to head) [Source:HGNC Symbol;Acc:HGNC:49060]
ENSG00000235162	-0,7792132	2,45031522	14,6589234	0,00383625	0,09172458	C12orf75	chromosome 12 open reading frame 75 [Source:HGNC Symbol;Acc:HGNC:35164]
ENSG00000108061	-0,786057	2,58828557	14,6429157	0,00384877	0,09187361	SHOC2	SHOC2 leucine rich repeat scaffold protein [Source:HGNC Symbol;Acc:HGNC:15454]
ENSG00000100234	0,66664335	8,19295515	14,6110625	0,00387383	0,0923211	TIMP3	TIMP metallopeptidase inhibitor 3 [Source:HGNC Symbol;Acc:HGNC:11822]
ENSG00000158865	-0,8753197	0,96387166	14,5873662	0,00389261	0,09246689	SLC5A11	solute carrier family 5 member 11 [Source:HGNC Symbol;Acc:HGNC:23091]
ENSG00000184937	-2,08736	-1,2084687	14,5889106	0,00389138	0,09246689	WT1	WT1 transcription factor [Source:HGNC Symbol;Acc:HGNC:12796]

ENSG00000164406	0,68145446	3,50554317	14,5557851	0,00391781	0,09291435	LEAP2	liver enriched antimicrobial peptide 2 [Source:HGNC Symbol;Acc:HGNC:29571]
ENSG00000105323	-0,6150212	6,88237971	14,5340384	0,00393527	0,09302655	HNRNPUL1	heterogeneous nuclear ribonucleoprotein U like 1 [Source:HGNC Symbol;Acc:HGNC:17011]
ENSG00000143507	0,94240443	0,95901255	14,5347822	0,00393467	0,09302655	DUSP10	dual specificity phosphatase 10 [Source:HGNC Symbol;Acc:HGNC:3065]
ENSG00000158825	-1,318663	-0,1852751	14,5035273	0,00395994	0,09342182	CDA	cytidine deaminase [Source:HGNC Symbol;Acc:HGNC:1712]
ENSG00000169598	0,59311513	3,01950559	14,4975624	0,00396478	0,09342182	DFFB	DNA fragmentation factor subunit beta [Source:HGNC Symbol;Acc:HGNC:2773]
ENSG00000090013	-0,6099327	4,97872086	14,4685157	0,00398848	0,09363819	BLVRB	biliverdin reductase B [Source:HGNC Symbol;Acc:HGNC:1063]
ENSG00000166266	-0,6452417	3,62399636	14,4627567	0,0039932	0,09363819	CUL5	cullin 5 [Source:HGNC Symbol;Acc:HGNC:2556]
ENSG00000188725	0,6201321	4,09163502	14,473262	0,00398459	0,09363819	SMIM15	small integral membrane protein 15 [Source:HGNC Symbol;Acc:HGNC:33861]
ENSG00000196458	1,20038818	0,32528152	14,4516244	0,00400234	0,0937021	ZNF605	zinc finger protein 605 [Source:HGNC Symbol;Acc:HGNC:28068]
ENSG00000132170	0,66164918	5,03377956	14,4286846	0,00402125	0,09399429	PPARG	peroxisome proliferator activated receptor gamma [Source:HGNC Symbol;Acc:HGNC:9236]
ENSG00000100380	0,56382752	8,44727139	14,3872604	0,00405568	0,09429104	ST13	ST13 Hsp70 interacting protein [Source:HGNC Symbol;Acc:HGNC:11343]
ENSG00000103460	0,63927249	4,61316953	14,3974069	0,00404721	0,09429104	TOX3	TOX high mobility group box family member 3 [Source:HGNC Symbol;Acc:HGNC:11972]
ENSG00000139433	-0,5758403	3,58808587	14,4027128	0,0040428	0,09429104	GLTP	glycolipid transfer protein [Source:HGNC Symbol;Acc:HGNC:24867]
ENSG00000165507	0,54339876	5,14509587	14,3593017	0,00407913	0,09429104	DEPP1	DEPP1 autophagy regulator [Source:HGNC Symbol;Acc:HGNC:23355]
ENSG00000204070	0,58784049	6,66864065	14,3698483	0,00407026	0,09429104	SYS1	SYS1 golgi trafficking protein [Source:HGNC Symbol;Acc:HGNC:16162]
ENSG00000260018	-0,849509	1,11495131	14,3649315	0,00407439	0,09429104	AC040169.1	novel transcript, antisense to MBTPS1
ENSG00000272655	1,05185203	0,53585348	14,3756488	0,0040654	0,09429104	POLR2J4	RNA polymerase II subunit J4, pseudogene [Source:HGNC Symbol;Acc:HGNC:28195]
ENSG00000235823	-0,9195962	1,55423931	14,3409034	0,00409464	0,0945002	OLMALINC	oligodendrocyte maturation-associated long intergenic non-coding RNA [Source:HGNC Symbol;Acc:HGNC:28060]
ENSG00000149269	1,27968789	0,35848451	14,3323963	0,00410184	0,09451704	PAK1	p21 (RAC1) activated kinase 1 [Source:HGNC Symbol;Acc:HGNC:8590]
ENSG00000171940	0,57618123	5,33323566	14,3057705	0,00412447	0,09483993	ZNF217	zinc finger protein 217 [Source:HGNC Symbol;Acc:HGNC:13009]
ENSG00000179526	-0,62114	5,04425357	14,3006573	0,00412884	0,09483993	SHARPIN	SHANK associated RH domain interactor [Source:HGNC Symbol;Acc:HGNC:25321]
ENSG00000265458	-1,4557359	0,23982286	14,2916448	0,00413654	0,09486776	AC132938.3	novel transcript, antisense NARF
ENSG00000108239	0,62981205	2,50689843	14,2793981	0,00414704	0,09495947	TBC1D12	TBC1 domain family member 12 [Source:HGNC Symbol;Acc:HGNC:29082]
ENSG00000141552	-0,6432915	6,46692776	14,2423604	0,00417899	0,09550623	ANAPC11	anaphase promoting complex subunit 11 [Source:HGNC Symbol;Acc:HGNC:14452]
ENSG00000182923	-0,6827804	3,54904639	14,2365914	0,004184	0,09550623	CEP63	centrosomal protein 63 [Source:HGNC Symbol;Acc:HGNC:25815]
ENSG00000115020	0,76407413	3,68900157	14,1995366	0,00421631	0,0956245	PIKFYVE	phosphoinositide kinase, FYVE-type zinc finger containing [Source:HGNC Symbol;Acc:HGNC:23785]
ENSG00000122711	-1,2834084	-0,3981962	14,1933519	0,00422173	0,0956245	SPINK4	serine peptidase inhibitor Kazal type 4 [Source:HGNC Symbol;Acc:HGNC:16646]
ENSG00000155629	0,71137462	3,01860269	14,1931547	0,0042219	0,0956245	PIK3AP1	phosphoinositide-3-kinase adaptor protein 1 [Source:HGNC Symbol;Acc:HGNC:30034]
ENSG00000168453	2,21164036	-1,5125395	14,2142261	0,00420346	0,0956245	HR	HR lysine demethylase and nuclear receptor corepressor [Source:HGNC Symbol;Acc:HGNC:5172]
ENSG00000198625	0,94371699	2,61352435	14,2127173	0,00420478	0,0956245	MDM4	MDM4 regulator of p53 [Source:HGNC Symbol;Acc:HGNC:6974]
ENSG00000100731	0,56682787	4,77800575	14,1790257	0,00423433	0,09575737	PCNX1	pecanex 1 [Source:HGNC Symbol;Acc:HGNC:19740]
ENSG00000108576	-0,9937766	2,36041081	14,1536575	0,00425674	0,09581928	SLC6A4	solute carrier family 6 member 4 [Source:HGNC Symbol;Acc:HGNC:11050]
ENSG00000121716	2,60967746	-1,260149	14,1569702	0,0042538	0,09581928	PILRB	paired immunoglobin like type 2 receptor beta [Source:HGNC Symbol;Acc:HGNC:18297]
ENSG00000131242	0,50737072	6,84116156	14,1671787	0,00424478	0,09581928	RAB11FIP4	RAB11 family interacting protein 4 [Source:HGNC Symbol;Acc:HGNC:30267]
ENSG00000115840	-0,6113743	2,85932098	14,1255216	0,00428177	0,09583378	SLC25A12	solute carrier family 25 member 12 [Source:HGNC Symbol;Acc:HGNC:10982]
ENSG00000130023	-0,6809867	2,37041469	14,1175083	0,00428893	0,09583378	ERMARD	ER membrane associated RNA degradation [Source:HGNC Symbol;Acc:HGNC:21056]
ENSG00000136504	0,5298437	4,98379846	14,1075376	0,00429786	0,09583378	KAT7	lysine acetyltransferase 7 [Source:HGNC Symbol;Acc:HGNC:17016]
ENSG00000153094	0,69753367	2,87403792	14,1052404	0,00429992	0,09583378	BCL2L11	BCL2 like 11 [Source:HGNC Symbol;Acc:HGNC:994]
ENSG00000173992	-0,5337753	4,99793245	14,1048094	0,00430031	0,09583378	CCS	copper chaperone for superoxide dismutase [Source:HGNC Symbol;Acc:HGNC:1613]
ENSG00000256683	0,73927539	2,62485037	14,1014749	0,0043033	0,09583378	ZNF350	zinc finger protein 350 [Source:HGNC Symbol;Acc:HGNC:16656]
ENSG00000286117	-1,0153876	1,27003359	14,1163334	0,00428998	0,09583378	AL121894.3	novel transcript
ENSG00000143479	1,22993382	0,61039364	14,0929576	0,00431096	0,09585822	DYRK3	dual specificity tyrosine phosphorylation regulated kinase 3 [Source:HGNC Symbol;Acc:HGNC:3094]
ENSG00000003249	-0,5981894	4,09457386	14,0282549	0,00436969	0,09661544	DBNDD1	dysbindin domain containing 1 [Source:HGNC Symbol;Acc:HGNC:28455]
ENSG00000163884	-0,7387468	3,20744818	14,0263135	0,00437147	0,09661544	KLF15	Kruppel like factor 15 [Source:HGNC Symbol;Acc:HGNC:14536]

ENSG00000181754	0,74731534	2,08234405	14,0462103	0,0043533	0,09661544	AMIGO1	adhesion molecule with Ig like domain 1 [Source:HGNC Symbol;Acc:HGNC:20824]
ENSG00000205336	0,80078004	4,0635265	14,0302747	0,00436784	0,09661544	ADGRG1	adhesion G protein-coupled receptor G1 [Source:HGNC Symbol;Acc:HGNC:4512]
ENSG00000173295	-0,8406708	1,88862883	14,0185913	0,00437855	0,09662569	FAM86B3P	family with sequence similarity 86 member B3, pseudogene [Source:HGNC Symbol;Acc:HGNC:44371]
ENSG00000119682	0,53661461	4,61742514	13,9892098	0,00440561	0,09707619	AREL1	apoptosis resistant E3 ubiquitin protein ligase 1 [Source:HGNC Symbol;Acc:HGNC:20363]
ENSG00000136052	0,66810194	2,67409792	13,8666717	0,00452068	0,09946185	SLC41A2	solute carrier family 41 member 2 [Source:HGNC Symbol;Acc:HGNC:31045]
ENSG00000139793	-0,8689213	1,9037289	13,8457175	0,00454073	0,09959768	MBNL2	muscleblind like splicing regulator 2 [Source:HGNC Symbol;Acc:HGNC:16746]
ENSG00000149527	-1,2165453	0,42826351	13,8388615	0,00454731	0,09959768	PLCH2	phospholipase C eta 2 [Source:HGNC Symbol;Acc:HGNC:29037]
ENSG00000150540	-0,5029511	7,48780847	13,8468303	0,00453966	0,09959768	HNMT	histamine N-methyltransferase [Source:HGNC Symbol;Acc:HGNC:5028]
ENSG00000099260	0,52138103	4,6746696	13,7625208	0,00462139	0,09959826	PALMD	palmdelphin [Source:HGNC Symbol;Acc:HGNC:15846]
ENSG00000112667	-0,6228303	4,8728674	13,7393492	0,00464417	0,09959826	DNPH1	2'-deoxynucleoside 5'-phosphate N-hydrolase 1 [Source:HGNC Symbol;Acc:HGNC:21218]
ENSG00000122861	-0,9539234	2,7046542	13,7458251	0,00463779	0,09959826	PLAU	plasminogen activator, urokinase [Source:HGNC Symbol;Acc:HGNC:9052]
ENSG00000126790	-0,586679	3,06014581	13,8047194	0,00458026	0,09959826	L3HYPDH	trans-L-3-hydroxyproline dehydratase [Source:HGNC Symbol;Acc:HGNC:20488]
ENSG00000129991	0,91538919	1,12396516	13,8163613	0,00456899	0,09959826	TNNI3	troponin I3, cardiac type [Source:HGNC Symbol;Acc:HGNC:11947]
ENSG00000134363	0,61049823	6,97126555	13,7539728	0,00462978	0,09959826	FST	follistatin [Source:HGNC Symbol;Acc:HGNC:3971]
ENSG00000139192	0,67882668	4,35509536	13,7901579	0,0045944	0,09959826	TAPBPL	TAP binding protein like [Source:HGNC Symbol;Acc:HGNC:30683]
ENSG00000157368	1,27046753	-0,2203912	13,8045509	0,00458042	0,09959826	IL34	interleukin 34 [Source:HGNC Symbol;Acc:HGNC:28529]
ENSG00000172482	-0,6468854	4,2046588	13,8146661	0,00457063	0,09959826	AGXT	alanine--glyoxylate and serine--pyruvate aminotransferase [Source:HGNC Symbol;Acc:HGNC:341]
ENSG00000172965	-0,6573842	3,38469408	13,7338427	0,0046496	0,09959826	MIR4435-2HG	MIR4435-2 host gene [Source:HGNC Symbol;Acc:HGNC:35163]
ENSG00000183520	-0,4951593	5,60101991	13,7858386	0,00459861	0,09959826	UTP11	UTP11 small subunit processome component [Source:HGNC Symbol;Acc:HGNC:24329]
ENSG00000210082	0,49986139	12,2235533	13,7588264	0,00462501	0,09959826	MT-RNR2	mitochondrially encoded 16S rRNA [Source:HGNC Symbol;Acc:HGNC:7471]
ENSG00000228315	0,95028658	0,76033239	13,7360911	0,00464738	0,09959826	GUSBP11	GUSB pseudogene 11 [Source:HGNC Symbol;Acc:HGNC:42325]
ENSG00000272702	-2,2758739	-1,9283733	13,7502532	0,00463343	0,09959826	AC010913.1	novel transcript
ENSG00000276931	-1,8791286	-1,0726433	13,7723285	0,00461179	0,09959826	AC009041.3	novel transcript, sense intronic to LMF1
ENSG00000124357	0,53761955	4,91321443	13,6962702	0,00468688	0,09987165	NAGK	N-acetylglucosamine kinase [Source:HGNC Symbol;Acc:HGNC:17174]
ENSG00000135069	-0,496978	8,60138237	13,6953874	0,00468776	0,09987165	PSAT1	phosphoserine aminotransferase 1 [Source:HGNC Symbol;Acc:HGNC:19129]
ENSG00000140832	0,71956205	2,40221613	13,6910742	0,00469206	0,09987165	MARVELD3	MARVEL domain containing 3 [Source:HGNC Symbol;Acc:HGNC:30525]
ENSG00000141480	-0,5410682	5,00763555	13,7039289	0,00467925	0,09987165	ARRB2	arrestin beta 2 [Source:HGNC Symbol;Acc:HGNC:712]
ENSG00000173786	0,56499066	7,70517186	13,686587	0,00469654	0,09987165	CNP	2',3'-cyclic nucleotide 3' phosphodiesterase [Source:HGNC Symbol;Acc:HGNC:2158]
ENSG00000142396	1,01927966	0,68155938	13,6747554	0,00470839	0,09997799	ERVK3-1	endogenous retrovirus group K3 member 1 [Source:HGNC Symbol;Acc:HGNC:30466]
ENSG00000126003	0,66742139	4,68203468	13,6663824	0,00471679	0,10001107	PLAGL2	PLAG1 like zinc finger 2 [Source:HGNC Symbol;Acc:HGNC:9047]
ENSG00000174373	0,55055636	4,92254123	13,6564678	0,00472677	0,10007732	RALGAPA1	Ral GTPase activating protein catalytic subunit alpha 1 [Source:HGNC Symbol;Acc:HGNC:17770]
ENSG00000181649	-0,9112797	2,25800541	13,6385787	0,00474483	0,10031438	PHLDA2	pleckstrin homology like domain family A member 2 [Source:HGNC Symbol;Acc:HGNC:12385]
ENSG00000167306	-0,5432607	3,91481036	13,6037652	0,00478023	0,10077102	MYO5B	myosin VB [Source:HGNC Symbol;Acc:HGNC:7603]
ENSG00000198162	-0,5527273	3,50996075	13,6098723	0,00477399	0,10077102	MAN1A2	mannosidase alpha class 1A member 2 [Source:HGNC Symbol;Acc:HGNC:6822]
ENSG00000051341	0,73782639	3,08225739	13,589574	0,00479475	0,10078626	POLQ	DNA polymerase theta [Source:HGNC Symbol;Acc:HGNC:9186]
ENSG00000153391	-0,5507163	3,70926763	13,5917759	0,00479249	0,10078626	INO80C	INO80 complex subunit C [Source:HGNC Symbol;Acc:HGNC:26994]
ENSG00000069667	1,04441814	1,18945193	13,5713853	0,00481344	0,10086412	RORA	RAR related orphan receptor A [Source:HGNC Symbol;Acc:HGNC:10258]
ENSG00000100038	-0,5666562	4,17090635	13,5586519	0,00482657	0,10086412	TOP3B	DNA topoisomerase III beta [Source:HGNC Symbol;Acc:HGNC:11993]
ENSG00000122787	0,78577643	4,41529582	13,5524655	0,00483297	0,10086412	AKR1D1	aldo-keto reductase family 1 member D1 [Source:HGNC Symbol;Acc:HGNC:388]
ENSG00000138764	1,05498501	1,57462144	13,5707284	0,00481411	0,10086412	CCNG2	cyclin G2 [Source:HGNC Symbol;Acc:HGNC:1593]
ENSG00000143337	0,66968891	6,09330253	13,5640791	0,00482097	0,10086412	TOR1AIP1	torsin 1A interacting protein 1 [Source:HGNC Symbol;Acc:HGNC:29456]
ENSG00000042286	-0,5050905	7,56937698	13,4820243	0,00490656	0,10093923	AIFM2	apoptosis inducing factor mitochondria associated 2 [Source:HGNC Symbol;Acc:HGNC:21411]
ENSG00000110844	-0,6359305	2,67938396	13,5043443	0,0048831	0,10093923	PRPF40B	pre-mRNA processing factor 40 homolog B [Source:HGNC Symbol;Acc:HGNC:25031]
ENSG00000121621	0,61117568	2,67652541	13,5019236	0,00488564	0,10093923	KIF18A	kinesin family member 18A [Source:HGNC Symbol;Acc:HGNC:29441]

ENSG00000131477	0,61566601	3,41433386	13,5243054	0,00486223	0,10093923	RAMP2	receptor activity modifying protein 2 [Source:HGNC Symbol;Acc:HGNC:9844]
ENSG00000141295	-0,6517743	3,6823607	13,4832388	0,00490528	0,10093923	SCRN2	secernin 2 [Source:HGNC Symbol;Acc:HGNC:30381]
ENSG00000165406	0,7471523	2,62926505	13,534832	0,00485127	0,10093923	MARCHF8	membrane associated ring-CH-type finger 8 [Source:HGNC Symbol;Acc:HGNC:23356]
ENSG00000177707	0,62542793	3,32577078	13,4815076	0,00490711	0,10093923	NECTIN3	nectin cell adhesion molecule 3 [Source:HGNC Symbol;Acc:HGNC:17664]
ENSG00000180398	-0,5430307	7,86136972	13,5103917	0,00487676	0,10093923	MCFD2	multiple coagulation factor deficiency 2, ER cargo receptor complex subunit [Source:HGNC Symbol;Acc:HGNC:184
ENSG00000181513	-0,661749	3,02864683	13,5006194	0,004887	0,10093923	ACBD4	acyl-CoA binding domain containing 4 [Source:HGNC Symbol;Acc:HGNC:23337]
ENSG00000186792	-0,6176663	3,68117354	13,4840612	0,00490442	0,10093923	HYAL3	hyaluronidase 3 [Source:HGNC Symbol;Acc:HGNC:5322]
ENSG00000198612	-0,5344037	6,47644391	13,4763271	0,00491257	0,10093923	COPS8	COP9 signalosome subunit 8 [Source:HGNC Symbol;Acc:HGNC:24335]
ENSG00000100949	-0,4885968	5,6786041	13,4623473	0,00492736	0,10110091	RABGGTA	Rab geranylgeranyltransferase subunit alpha [Source:HGNC Symbol;Acc:HGNC:9795]
ENSG00000136560	-0,6072839	4,70010735	13,4448221	0,00494598	0,10119863	TANK	TRAF family member associated NFKB activator [Source:HGNC Symbol;Acc:HGNC:11562]
ENSG00000174013	-0,751306	2,70392007	13,4473247	0,00494332	0,10119863	FBXO45	F-box protein 45 [Source:HGNC Symbol;Acc:HGNC:29148]
ENSG00000111674	-0,7837927	5,54579076	13,437744	0,00495352	0,10121122	ENO2	enolase 2 [Source:HGNC Symbol;Acc:HGNC:3353]
ENSG00000144749	0,54466084	5,59875444	13,4104232	0,00498277	0,10138346	LRIG1	leucine rich repeats and immunoglobulin like domains 1 [Source:HGNC Symbol;Acc:HGNC:17360]
ENSG00000178999	-0,4786839	6,25408768	13,4144763	0,00497842	0,10138346	AURKB	aurora kinase B [Source:HGNC Symbol;Acc:HGNC:11390]
ENSG00000197119	0,59637299	3,65497555	13,4215867	0,0049708	0,10138346	SLC25A29	solute carrier family 25 member 29 [Source:HGNC Symbol;Acc:HGNC:20116]
ENSG00000229953	-1,1998594	0,23208999	13,4032814	0,00499045	0,10139851	AL590666.2	novel transcript
ENSG00000166147	1,04416803	0,96578164	13,3845612	0,00501065	0,10166757	FBN1	fibrillin 1 [Source:HGNC Symbol;Acc:HGNC:3603]
ENSG00000145431	-0,740481	1,5986392	13,3662624	0,0050305	0,10192863	PDGFC	platelet derived growth factor C [Source:HGNC Symbol;Acc:HGNC:8801]
ENSG00000164073	0,62984499	4,09511031	13,3559924	0,00504168	0,10201365	MFSD8	major facilitator superfamily domain containing 8 [Source:HGNC Symbol;Acc:HGNC:28486]
ENSG00000189221	0,5847054	4,49732231	13,3472801	0,00505118	0,10206465	MAOA	monoamine oxidase A [Source:HGNC Symbol;Acc:HGNC:6833]
ENSG00000058804	-0,5511245	5,63265541	13,3074039	0,00509498	0,10239234	NDC1	NDC1 transmembrane nucleoporin [Source:HGNC Symbol;Acc:HGNC:25525]
ENSG00000136925	0,67409154	3,47086161	13,3073652	0,00509502	0,10239234	TSTD2	thiosulfate sulfurtransferase like domain containing 2 [Source:HGNC Symbol;Acc:HGNC:30087]
ENSG00000154079	-0,7029263	2,45917523	13,3069888	0,00509544	0,10239234	SDHAF4	succinate dehydrogenase complex assembly factor 4 [Source:HGNC Symbol;Acc:HGNC:20957]
ENSG00000189180	0,61441602	3,64959114	13,3246621	0,00507597	0,10239234	ZNF33A	zinc finger protein 33A [Source:HGNC Symbol;Acc:HGNC:13096]
ENSG00000137767	0,57725842	4,58617807	13,2950546	0,00510864	0,10243076	SQOR	sulfide quinone oxidoreductase [Source:HGNC Symbol;Acc:HGNC:20390]
ENSG00000174106	0,5620121	3,63651033	13,292585	0,00511137	0,10243076	LEMD3	LEM domain containing 3 [Source:HGNC Symbol;Acc:HGNC:28887]
ENSG00000049246	1,00261979	1,05787094	13,2617875	0,00514565	0,10262877	PER3	period circadian regulator 3 [Source:HGNC Symbol;Acc:HGNC:8847]
ENSG00000113448	0,75192417	2,4032495	13,2670719	0,00513975	0,10262877	PDE4D	phosphodiesterase 4D [Source:HGNC Symbol;Acc:HGNC:8783]
ENSG00000152382	0,58477225	4,5116091	13,2732111	0,0051329	0,10262877	TADA1	transcriptional adaptor 1 [Source:HGNC Symbol;Acc:HGNC:30631]
ENSG00000263528	-0,6190625	2,52381106	13,2584738	0,00514935	0,10262877	IKBKE	inhibitor of nuclear factor kappa B kinase subunit epsilon [Source:HGNC Symbol;Acc:HGNC:14552]
ENSG00000006576	0,60383246	3,38653048	13,234946	0,00517575	0,10287419	PHTF2	putative homeodomain transcription factor 2 [Source:HGNC Symbol;Acc:HGNC:13411]
ENSG00000163482	0,70018455	3,67189805	13,2408955	0,00516906	0,10287419	STK36	serine/threonine kinase 36 [Source:HGNC Symbol;Acc:HGNC:17209]
ENSG00000103034	-1,3961951	-0,464956	13,2157892	0,00519737	0,10316348	NDRG4	NDRG family member 4 [Source:HGNC Symbol;Acc:HGNC:14466]
ENSG00000183421	0,64052691	3,81118571	13,2068195	0,00520752	0,10322487	RIPK4	receptor interacting serine/threonine kinase 4 [Source:HGNC Symbol;Acc:HGNC:496]
ENSG00000047249	0,51237582	5,61319139	13,1700526	0,00524942	0,10370934	ATP6V1H	ATPase H+ transporting V1 subunit H [Source:HGNC Symbol;Acc:HGNC:18303]
ENSG00000139651	0,62242772	3,6616772	13,1754636	0,00524323	0,10370934	ZNF740	zinc finger protein 740 [Source:HGNC Symbol;Acc:HGNC:27465]
ENSG00000151632	0,50086911	8,40372085	13,1666993	0,00525326	0,10370934	AKR1C2	aldo-keto reductase family 1 member C2 [Source:HGNC Symbol;Acc:HGNC:385]
ENSG00000119640	-0,7309028	1,91806355	13,155297	0,00526635	0,10382742	ACYP1	acylphosphatase 1 [Source:HGNC Symbol;Acc:HGNC:179]
ENSG00000163818	0,72096013	3,63660497	13,1410955	0,00528271	0,10386956	LZTFL1	leucine zipper transcription factor like 1 [Source:HGNC Symbol;Acc:HGNC:6741]
ENSG00000165338	0,87464644	0,90253019	13,1472575	0,0052756	0,10386956	HECTD2	HECT domain E3 ubiquitin protein ligase 2 [Source:HGNC Symbol;Acc:HGNC:26736]
ENSG00000138434	-0,5652817	5,30510326	13,130427	0,00529504	0,10397204	ITPRID2	ITPR interacting domain containing 2 [Source:HGNC Symbol;Acc:HGNC:11319]
ENSG00000112183	-1,3177657	-0,0800383	13,1198587	0,00530728	0,10407264	RBM24	RNA binding motif protein 24 [Source:HGNC Symbol;Acc:HGNC:21539]
ENSG00000005187	0,58079196	4,85486306	13,0801174	0,00535365	0,10424285	ACSM3	acyl-CoA synthetase medium chain family member 3 [Source:HGNC Symbol;Acc:HGNC:10522]
ENSG00000099625	0,76547228	1,65011374	13,069282	0,00536638	0,10424285	CBARP	CACN subunit beta associated regulatory protein [Source:HGNC Symbol;Acc:HGNC:28617]

ENSG00000104756	-0,585405	4,00842881	13,0446339	0,00539547	0,10424285	KCTD9	potassium channel tetramerization domain containing 9 [Source:HGNC Symbol;Acc:HGNC:22401]
ENSG00000124120	-0,6108298	3,91986	13,061147	0,00537596	0,10424285	TTPAL	alpha tocopherol transfer protein like [Source:HGNC Symbol;Acc:HGNC:16114]
ENSG00000125124	0,6658738	5,0632672	13,0334566	0,00540873	0,10424285	BBS2	Bardet-Biedl syndrome 2 [Source:HGNC Symbol;Acc:HGNC:967]
ENSG00000137364	-0,6750158	4,39663767	13,0480065	0,00539148	0,10424285	TPMT	thiopurine S-methyltransferase [Source:HGNC Symbol;Acc:HGNC:12014]
ENSG00000137824	0,53461132	6,5619939	13,0493679	0,00538987	0,10424285	RMDN3	regulator of microtubule dynamics 3 [Source:HGNC Symbol;Acc:HGNC:25550]
ENSG00000144591	-0,5710713	6,80586162	13,0600753	0,00537722	0,10424285	GMPPA	GDP-mannose pyrophosphorylase A [Source:HGNC Symbol;Acc:HGNC:22923]
ENSG00000162704	-0,7043162	5,75679393	13,0708516	0,00536453	0,10424285	ARPC5	actin related protein 2/3 complex subunit 5 [Source:HGNC Symbol;Acc:HGNC:708]
ENSG00000167703	0,67019086	4,99754873	13,0958045	0,00533529	0,10424285	SLC43A2	solute carrier family 43 member 2 [Source:HGNC Symbol;Acc:HGNC:23087]
ENSG00000176894	-0,6697869	2,51561866	13,0578674	0,00537983	0,10424285	PXMP2	peroxisomal membrane protein 2 [Source:HGNC Symbol;Acc:HGNC:9716]
ENSG00000179241	-1,8479094	-0,8404809	13,0346926	0,00540726	0,10424285	LDLRAD3	low density lipoprotein receptor class A domain containing 3 [Source:HGNC Symbol;Acc:HGNC:27046]
ENSG00000187097	0,53296802	4,45077592	13,0485907	0,00539079	0,10424285	ENTPD5	ectonucleoside triphosphate diphosphohydrolase 5 (inactive) [Source:HGNC Symbol;Acc:HGNC:3367]
ENSG00000066248	0,7008191	3,61052888	13,0133603	0,00543266	0,1042845	NGEF	neuronal guanine nucleotide exchange factor [Source:HGNC Symbol;Acc:HGNC:7807]
ENSG00000100577	-0,576418	4,82667792	12,9889747	0,00546187	0,1042845	GSTZ1	glutathione S-transferase zeta 1 [Source:HGNC Symbol;Acc:HGNC:4643]
ENSG00000114439	0,83054014	2,28543444	12,9274619	0,00553643	0,1042845	BBX	BBX high mobility group box domain containing [Source:HGNC Symbol;Acc:HGNC:14422]
ENSG00000115267	0,65560955	3,05119502	12,9536287	0,00550456	0,1042845	IFIH1	interferon induced with helicase C domain 1 [Source:HGNC Symbol;Acc:HGNC:18873]
ENSG00000123684	0,51236154	4,7795307	12,9360476	0,00552595	0,1042845	LPGAT1	lysophosphatidylglycerol acyltransferase 1 [Source:HGNC Symbol;Acc:HGNC:28985]
ENSG00000129173	0,64033164	2,70283463	12,9537616	0,0055044	0,1042845	E2F8	E2F transcription factor 8 [Source:HGNC Symbol;Acc:HGNC:24727]
ENSG00000131398	-1,6675369	-0,5569222	12,9530628	0,00550525	0,1042845	KCNC3	potassium voltage-gated channel subfamily C member 3 [Source:HGNC Symbol;Acc:HGNC:6235]
ENSG00000136143	0,66344494	6,05404186	12,9250484	0,00553938	0,1042845	SUCLA2	succinate-CoA ligase ADP-forming subunit beta [Source:HGNC Symbol;Acc:HGNC:11448]
ENSG00000157020	-0,4870634	8,78209391	12,9899831	0,00546066	0,1042845	SEC13	SEC13 homolog, nuclear pore and COPII coat complex component [Source:HGNC Symbol;Acc:HGNC:10697]
ENSG00000168310	0,52792609	4,44207635	12,9948472	0,00545482	0,1042845	IRF2	interferon regulatory factor 2 [Source:HGNC Symbol;Acc:HGNC:6117]
ENSG00000171617	0,71063346	7,23768121	12,9691789	0,00548573	0,1042845	ENC1	ectodermal-neural cortex 1 [Source:HGNC Symbol;Acc:HGNC:3345]
ENSG00000174990	-0,5523542	4,87767699	12,9793406	0,00547347	0,1042845	CA5A	carbonic anhydrase 5A [Source:HGNC Symbol;Acc:HGNC:1377]
ENSG00000176092	-0,9612158	1,28441745	12,9605734	0,00549614	0,1042845	CRYBG2	crystallin beta-gamma domain containing 2 [Source:HGNC Symbol;Acc:HGNC:17295]
ENSG00000197147	-0,7658219	1,78842434	13,0056967	0,00544182	0,1042845	LRRC8B	leucine rich repeat containing 8 VRAC subunit B [Source:HGNC Symbol;Acc:HGNC:30692]
ENSG00000198315	0,91624285	2,19367664	12,9275598	0,00553631	0,1042845	ZKSCAN8	zinc finger with KRAB and SCAN domains 8 [Source:HGNC Symbol;Acc:HGNC:12983]
ENSG00000227038	1,00008759	0,37081719	13,0243439	0,00541956	0,1042845	GTF2IP7	general transcription factor IIi pseudogene 7 [Source:HGNC Symbol;Acc:HGNC:51720]
ENSG00000228696	0,71667852	1,58630247	12,9428404	0,00551767	0,1042845	ARL17A	ADP ribosylation factor like GTPase 17A [Source:HGNC Symbol;Acc:HGNC:24096]
ENSG00000262001	-1,7112356	-0,6203287	12,941429	0,00551939	0,1042845	DLGAP1-AS2	DLGAP1 antisense RNA 2 [Source:HGNC Symbol;Acc:HGNC:28146]
ENSG00000231742	-0,8474739	1,7057141	12,8956912	0,00557542	0,10482795	LINC01273	long intergenic non-protein coding RNA 1273 [Source:HGNC Symbol;Acc:HGNC:50329]
ENSG00000132326	0,93634614	3,07456083	12,8843977	0,00558936	0,10485801	PER2	period circadian regulator 2 [Source:HGNC Symbol;Acc:HGNC:8846]
ENSG00000134531	-1,6560551	0,22885632	12,8827705	0,00559137	0,10485801	EMP1	epithelial membrane protein 1 [Source:HGNC Symbol;Acc:HGNC:3333]
ENSG00000135315	0,74171315	1,62439103	12,8427551	0,00564114	0,10525082	CEP162	centrosomal protein 162 [Source:HGNC Symbol;Acc:HGNC:21107]
ENSG00000165959	0,89364844	2,3340371	12,8508491	0,00563103	0,10525082	CLMN	calmin [Source:HGNC Symbol;Acc:HGNC:19972]
ENSG00000182362	-0,652067	1,83919218	12,8457255	0,00563743	0,10525082	YBEY	ybeY metalloendoribonuclease [Source:HGNC Symbol;Acc:HGNC:1299]
ENSG00000213347	-0,7742027	1,77776825	12,8509598	0,00563089	0,10525082	MXD3	MAX dimerization protein 3 [Source:HGNC Symbol;Acc:HGNC:14008]
ENSG00000148399	-0,5220115	4,86382001	12,827174	0,00566066	0,10548037	DPH7	diphthamide biosynthesis 7 [Source:HGNC Symbol;Acc:HGNC:25199]
ENSG00000034510	-0,5560711	8,65958823	12,8201504	0,00566949	0,10551029	TMSB10	thymosin beta 10 [Source:HGNC Symbol;Acc:HGNC:11879]
ENSG00000011132	-0,5368996	3,83832235	12,7940885	0,00570239	0,10598762	APBA3	amyloid beta precursor protein binding family A member 3 [Source:HGNC Symbol;Acc:HGNC:580]
ENSG00000155366	-0,4625146	6,26792074	12,7836785	0,0057156	0,10609812	RHOC	ras homolog family member C [Source:HGNC Symbol;Acc:HGNC:669]
ENSG00000165476	-0,514679	5,39555392	12,7615476	0,00574381	0,1063514	REEP3	receptor accessory protein 3 [Source:HGNC Symbol;Acc:HGNC:23711]
ENSG00000170439	-0,5945151	5,19671021	12,7665925	0,00573736	0,1063514	METTL7B	methyltransferase like 7B [Source:HGNC Symbol;Acc:HGNC:28276]
ENSG00000158156	0,5529166	4,42720037	12,7533277	0,00575432	0,10641129	XKR8	XK related 8 [Source:HGNC Symbol;Acc:HGNC:25508]
ENSG00000100078	0,72152416	1,7576585	12,7366564	0,00577573	0,10642585	PLA2G3	phospholipase A2 group III [Source:HGNC Symbol;Acc:HGNC:17934]

ENSG00000157224	0,75152006	4,7884089	12,7356963	0,00577697	0,10642585	CLDN12	claudin 12 [Source:HGNC Symbol;Acc:HGNC:2034]
ENSG00000159873	0,51934289	5,03679498	12,7360444	0,00577652	0,10642585	CCDC117	coiled-coil domain containing 117 [Source:HGNC Symbol;Acc:HGNC:26599]
ENSG00000100077	-0,5889958	4,04752251	12,7071677	0,00581383	0,10656749	GRK3	G protein-coupled receptor kinase 3 [Source:HGNC Symbol;Acc:HGNC:290]
ENSG00000115540	-0,705016	3,38260357	12,7189129	0,00579862	0,10656749	MOB4	MOB family member 4, phocein [Source:HGNC Symbol;Acc:HGNC:17261]
ENSG00000168067	-0,5630294	3,56129491	12,7075214	0,00581337	0,10656749	MAP4K2	mitogen-activated protein kinase kinase kinase kinase 2 [Source:HGNC Symbol;Acc:HGNC:6864]
ENSG00000230952	-0,5860194	3,20588455	12,7173661	0,00580062	0,10656749	AC099344.1	novel transcript
ENSG00000170262	1,57844418	-0,6974534	12,6943345	0,00583051	0,10673927	MRAP	melanocortin 2 receptor accessory protein [Source:HGNC Symbol;Acc:HGNC:1304]
ENSG00000109756	0,79680162	3,86501247	12,6727164	0,00585874	0,10712179	RAPGEF2	Rap guanine nucleotide exchange factor 2 [Source:HGNC Symbol;Acc:HGNC:16854]
ENSG00000101191	0,47845633	6,40847831	12,6597791	0,00587571	0,10716372	DIDO1	death inducer-obliterator 1 [Source:HGNC Symbol;Acc:HGNC:2680]
ENSG00000116133	0,47986231	9,76545192	12,6543768	0,00588282	0,10716372	DHCR24	24-dehydrocholesterol reductase [Source:HGNC Symbol;Acc:HGNC:2859]
ENSG00000178229	0,74690895	1,42815369	12,6542083	0,00588304	0,10716372	ZNF543	zinc finger protein 543 [Source:HGNC Symbol;Acc:HGNC:25281]
ENSG00000006015	-0,7459254	1,86740071	12,6408141	0,0059007	0,10722044	REX1BD	required for excision 1-B domain containing [Source:HGNC Symbol;Acc:HGNC:26098]
ENSG00000065665	0,65547149	3,89863503	12,6407151	0,00590083	0,10722044	SEC61A2	SEC61 translocon subunit alpha 2 [Source:HGNC Symbol;Acc:HGNC:17702]
ENSG00000185112	0,8423303	1,28560391	12,6338872	0,00590986	0,1072511	FAM43A	family with sequence similarity 43 member A [Source:HGNC Symbol;Acc:HGNC:26888]
ENSG00000134138	0,62176634	2,60947406	12,6162032	0,00593332	0,1075433	MEIS2	Meis homeobox 2 [Source:HGNC Symbol;Acc:HGNC:7001]
ENSG00000161267	-0,5282782	3,69841919	12,6016348	0,00595274	0,1077615	BDH1	3-hydroxybutyrate dehydrogenase 1 [Source:HGNC Symbol;Acc:HGNC:1027]
ENSG00000270638	-1,8015862	-1,1824762	12,595323	0,00596117	0,10778065	AL023806.1	novel transcript
ENSG00000174032	0,59372259	4,10385394	12,5750872	0,00598832	0,10813758	SLC25A30	solute carrier family 25 member 30 [Source:HGNC Symbol;Acc:HGNC:27371]
ENSG00000162062	-0,5722334	4,15707497	12,5641283	0,00600308	0,10827033	TEDC2	tubulin epsilon and delta complex 2 [Source:HGNC Symbol;Acc:HGNC:25849]
ENSG00000171649	0,88620832	1,63989608	12,5570798	0,0060126	0,10830829	ZIK1	zinc finger protein interacting with K protein 1 [Source:HGNC Symbol;Acc:HGNC:33104]
ENSG00000196704	-0,7571167	5,55046514	12,5464839	0,00602694	0,10843297	AMZ2	archaelysin family metallopeptidase 2 [Source:HGNC Symbol;Acc:HGNC:28041]
ENSG00000146409	-0,6526586	3,28006773	12,5263511	0,00605431	0,10879141	SLC18B1	solute carrier family 18 member B1 [Source:HGNC Symbol;Acc:HGNC:21573]
ENSG00000110090	0,93455428	3,00653769	12,5011605	0,00608877	0,10902478	CPT1A	carnitine palmitoyltransferase 1A [Source:HGNC Symbol;Acc:HGNC:2328]
ENSG00000123066	0,96542643	3,25290245	12,4958816	0,00609602	0,10902478	MED13L	mediator complex subunit 13L [Source:HGNC Symbol;Acc:HGNC:22962]
ENSG00000146426	0,84852861	1,48912691	12,4950618	0,00609715	0,10902478	TIAM2	TIAM Rac1 associated GEF 2 [Source:HGNC Symbol;Acc:HGNC:11806]
ENSG00000172159	-0,9473959	1,29453198	12,5010815	0,00608888	0,10902478	FRMD3	FERM domain containing 3 [Source:HGNC Symbol;Acc:HGNC:24125]
ENSG00000103642	0,62250419	4,2101057	12,4832578	0,00611341	0,10918184	LACTB	lactamase beta [Source:HGNC Symbol;Acc:HGNC:16468]
ENSG00000144567	0,48571737	7,39682036	12,4650281	0,00613862	0,10949824	RETREG2	reticulophagy regulator family member 2 [Source:HGNC Symbol;Acc:HGNC:28450]
ENSG00000138593	0,5856167	2,8835203	12,4507326	0,00615848	0,10971851	SECISBP2L	SECIS binding protein 2 like [Source:HGNC Symbol;Acc:HGNC:28997]
ENSG00000006606	-1,9863708	-1,9071924	12,4444977	0,00616716	0,10973944	CCL26	C-C motif chemokine ligand 26 [Source:HGNC Symbol;Acc:HGNC:10625]
ENSG00000123124	-0,5116962	4,98903281	12,4344428	0,0061812	0,10985545	WWP1	WW domain containing E3 ubiquitin protein ligase 1 [Source:HGNC Symbol;Acc:HGNC:17004]
ENSG00000116717	-0,4805826	5,59785918	12,4143896	0,00620932	0,1100873	GADD45A	growth arrest and DNA damage inducible alpha [Source:HGNC Symbol;Acc:HGNC:4095]
ENSG00000170260	0,5322569	3,53780356	12,415509	0,00620775	0,1100873	ZNF212	zinc finger protein 212 [Source:HGNC Symbol;Acc:HGNC:13004]
ENSG00000006327	-0,5988141	7,14151491	12,4013223	0,00622772	0,11011864	TNFRSF12A	TNF receptor superfamily member 12A [Source:HGNC Symbol;Acc:HGNC:18152]
ENSG00000157193	-0,682303	4,50410322	12,398748	0,00623136	0,11011864	LRP8	LDL receptor related protein 8 [Source:HGNC Symbol;Acc:HGNC:6700]
ENSG00000165233	-0,5365651	3,72327729	12,3970908	0,0062337	0,11011864	CARD19	caspase recruitment domain family member 19 [Source:HGNC Symbol;Acc:HGNC:28148]
ENSG00000112425	-0,6231822	2,04019744	12,3886263	0,00624567	0,11019688	EPM2A	EPM2A glucan phosphatase, laforin [Source:HGNC Symbol;Acc:HGNC:3413]
ENSG00000039650	-0,5497926	4,49385363	12,3551173	0,00629334	0,11088681	PNKP	polynucleotide kinase 3'-phosphatase [Source:HGNC Symbol;Acc:HGNC:9154]
ENSG00000136048	0,57796594	6,07917616	12,3451951	0,00630755	0,11088681	DRAM1	DNA damage regulated autophagy modulator 1 [Source:HGNC Symbol;Acc:HGNC:25645]
ENSG00000150776	-0,6493372	3,76219989	12,3504171	0,00630007	0,11088681	NKAPD1	NKAP domain containing 1 [Source:HGNC Symbol;Acc:HGNC:25569]
ENSG00000196421	-0,7123025	4,93019606	12,3282431	0,0063319	0,11117684	C20orf204	chromosome 20 open reading frame 204 [Source:HGNC Symbol;Acc:HGNC:27655]
ENSG00000235169	-0,9451779	2,79638914	12,3231328	0,00633926	0,11117684	SMIM1	small integral membrane protein 1 (Vel blood group) [Source:HGNC Symbol;Acc:HGNC:44204]
ENSG00000101290	0,58192823	7,01499111	12,3067286	0,00636297	0,11132537	CDS2	CDP-diacylglycerol synthase 2 [Source:HGNC Symbol;Acc:HGNC:1801]
ENSG00000156535	-1,2078961	0,40419324	12,3100149	0,00635822	0,11132537	CD109	CD109 molecule [Source:HGNC Symbol;Acc:HGNC:21685]

ENSG00000087842	-0,5130001	5,51425606	12,2952216	0,00637967	0,11135078	PIR	pirin [Source:HGNC Symbol;Acc:HGNC:30048]
ENSG00000187800	-1,4285826	-0,772621	12,2991681	0,00637394	0,11135078	PEAR1	platelet endothelial aggregation receptor 1 [Source:HGNC Symbol;Acc:HGNC:33631]
ENSG00000144909	0,52957497	3,75973907	12,2785999	0,00640388	0,11164001	OSBPL11	oxysterol binding protein like 11 [Source:HGNC Symbol;Acc:HGNC:16397]
ENSG00000198088	1,13368744	0,18066292	12,2713641	0,00641446	0,11169109	NUP62CL	nucleoporin 62 C-terminal like [Source:HGNC Symbol;Acc:HGNC:25960]
ENSG00000124587	-0,6563428	3,01464196	12,2513459	0,00644383	0,11203744	PEX6	peroxisomal biogenesis factor 6 [Source:HGNC Symbol;Acc:HGNC:8859]
ENSG00000169967	0,56057422	4,32891036	12,244323	0,00645417	0,11203744	MAP3K2	mitogen-activated protein kinase kinase kinase 2 [Source:HGNC Symbol;Acc:HGNC:6854]
ENSG00000176024	0,79381236	2,43326977	12,2421614	0,00645736	0,11203744	ZNF613	zinc finger protein 613 [Source:HGNC Symbol;Acc:HGNC:25827]
ENSG00000240065	0,71992894	3,64838881	12,2229254	0,00648581	0,11239756	PSMB9	proteasome 20S subunit beta 9 [Source:HGNC Symbol;Acc:HGNC:9546]
ENSG00000049449	0,57158502	5,7958796	12,1587729	0,0065818	0,11259192	RCN1	reticulocalbin 1 [Source:HGNC Symbol;Acc:HGNC:9934]
ENSG00000102974	-0,4726441	5,58269513	12,185339	0,00654184	0,11259192	CTCF	CCCTC-binding factor [Source:HGNC Symbol;Acc:HGNC:13723]
ENSG00000103449	0,64871984	2,80995088	12,1614227	0,0065778	0,11259192	SALL1	spalt like transcription factor 1 [Source:HGNC Symbol;Acc:HGNC:10524]
ENSG00000110066	0,51586372	4,60235456	12,2030234	0,0065154	0,11259192	KMT5B	lysine methyltransferase 5B [Source:HGNC Symbol;Acc:HGNC:24283]
ENSG00000117472	-0,8777255	1,96841694	12,1615393	0,00657762	0,11259192	TSPAN1	tetraspanin 1 [Source:HGNC Symbol;Acc:HGNC:20657]
ENSG00000148343	0,5471482	4,27771808	12,2087905	0,00650681	0,11259192	MIGA2	mitoguardin 2 [Source:HGNC Symbol;Acc:HGNC:23621]
ENSG00000153071	-0,7038242	2,97946444	12,1917834	0,00653219	0,11259192	DAB2	DAB adaptor protein 2 [Source:HGNC Symbol;Acc:HGNC:2662]
ENSG00000160752	-0,4630523	9,09438262	12,1622836	0,0065765	0,11259192	FDPS	farnesyl diphosphate synthase [Source:HGNC Symbol;Acc:HGNC:3631]
ENSG00000164162	-0,7142247	2,24354745	12,1763987	0,00655525	0,11259192	ANAPC10	anaphase promoting complex subunit 10 [Source:HGNC Symbol;Acc:HGNC:24077]
ENSG00000177156	-0,4948523	7,96702956	12,1640801	0,00657379	0,11259192	TALDO1	transaldolase 1 [Source:HGNC Symbol;Acc:HGNC:11559]
ENSG00000196177	0,74984855	6,40307768	12,1626683	0,00657592	0,11259192	ACADSB	acyl-CoA dehydrogenase short/branched chain [Source:HGNC Symbol;Acc:HGNC:91]
ENSG00000128394	-0,9958378	0,30964308	12,1327109	0,00662129	0,11313504	APOBEC3F	apolipoprotein B mRNA editing enzyme catalytic subunit 3F [Source:HGNC Symbol;Acc:HGNC:17356]
ENSG00000025434	-0,5554565	4,68816278	12,1231724	0,00663582	0,11324808	NR1H3	nuclear receptor subfamily 1 group H member 3 [Source:HGNC Symbol;Acc:HGNC:7966]
ENSG00000165948	-0,8499187	3,06190171	12,1181966	0,00664341	0,11324808	IFI27L1	interferon alpha inducible protein 27 like 1 [Source:HGNC Symbol;Acc:HGNC:19754]
ENSG00000148158	0,6791776	3,17811439	12,0931686	0,00668177	0,11330751	SNX30	sorting nexin family member 30 [Source:HGNC Symbol;Acc:HGNC:23685]
ENSG00000149782	-0,7480205	3,89424377	12,1005007	0,0066705	0,11330751	PLCB3	phospholipase C beta 3 [Source:HGNC Symbol;Acc:HGNC:9056]
ENSG00000154174	0,5121637	6,70614393	12,0993391	0,00667229	0,11330751	TOMM70	translocase of outer mitochondrial membrane 70 [Source:HGNC Symbol;Acc:HGNC:11985]
ENSG00000167614	0,63297749	4,65019678	12,0906269	0,00668568	0,11330751	TTYH1	tweety family member 1 [Source:HGNC Symbol;Acc:HGNC:13476]
ENSG00000175216	-0,8668669	6,23565624	12,0936928	0,00668096	0,11330751	CKAP5	cytoskeleton associated protein 5 [Source:HGNC Symbol;Acc:HGNC:28959]
ENSG00000112394	0,62168919	4,12058694	12,0780325	0,0067051	0,11350491	SLC16A10	solute carrier family 16 member 10 [Source:HGNC Symbol;Acc:HGNC:17027]
ENSG00000087237	1,2733103	0,43248442	12,0349228	0,00677208	0,11360057	CETP	cholesteryl ester transfer protein [Source:HGNC Symbol;Acc:HGNC:1869]
ENSG00000089163	-0,9574002	0,64378911	12,0490108	0,0067501	0,11360057	SIRT4	sirtuin 4 [Source:HGNC Symbol;Acc:HGNC:14932]
ENSG00000121281	-0,6444908	1,81616971	12,0360761	0,00677028	0,11360057	ADCY7	adenylate cyclase 7 [Source:HGNC Symbol;Acc:HGNC:238]
ENSG00000161542	0,51871617	8,03751163	12,0633539	0,00672781	0,11360057	PRPSAP1	phosphoribosyl pyrophosphate synthetase associated protein 1 [Source:HGNC Symbol;Acc:HGNC:9466]
ENSG00000162300	-0,5028964	5,40848325	12,047798	0,00675199	0,11360057	ZFPL1	zinc finger protein like 1 [Source:HGNC Symbol;Acc:HGNC:12868]
ENSG00000170458	-0,8549715	1,37682221	12,0622669	0,0067295	0,11360057	CD14	CD14 molecule [Source:HGNC Symbol;Acc:HGNC:1628]
ENSG00000174282	0,69741193	2,20242768	12,0475902	0,00675232	0,11360057	ZBTB4	zinc finger and BTB domain containing 4 [Source:HGNC Symbol;Acc:HGNC:23847]
ENSG00000254004	0,69621615	2,71353996	12,0295017	0,00678057	0,11360057	ZNF260	zinc finger protein 260 [Source:HGNC Symbol;Acc:HGNC:13499]
ENSG00000275410	0,61402763	2,45523153	12,0293959	0,00678073	0,11360057	HNF1B	HNF1 homeobox B [Source:HGNC Symbol;Acc:HGNC:11630]
ENSG00000006534	-0,6468166	1,85903587	12,0200237	0,00679543	0,11371634	ALDH3B1	aldehyde dehydrogenase 3 family member B1 [Source:HGNC Symbol;Acc:HGNC:410]
ENSG00000078804	-0,608674	3,10726853	12,0059591	0,00681755	0,11382582	TP53INP2	tumor protein p53 inducible nuclear protein 2 [Source:HGNC Symbol;Acc:HGNC:16104]
ENSG00000125459	-0,5855122	3,81134467	12,0106904	0,0068101	0,11382582	MSTO1	misato mitochondrial distribution and morphology regulator 1 [Source:HGNC Symbol;Acc:HGNC:29678]
ENSG00000155719	2,72811112	-2,3170164	12,0008526	0,0068256	0,11383021	OTOA	otoancorin [Source:HGNC Symbol;Acc:HGNC:16378]
ENSG00000085433	0,71975503	2,13478655	11,9908871	0,00684136	0,11383302	WDR47	WD repeat domain 47 [Source:HGNC Symbol;Acc:HGNC:29141]
ENSG00000174791	-0,7383282	3,27592543	11,9950924	0,0068347	0,11383302	RIN1	Ras and Rab interactor 1 [Source:HGNC Symbol;Acc:HGNC:18749]
ENSG00000163041	-0,5750915	6,4080646	11,9600408	0,0068904	0,11425862	H3-3B	H3.3 histone B [Source:HGNC Symbol;Acc:HGNC:4765]

ENSG00000182327	-0,7078929	3,99597518	11,9611131	0,00688869	0,11425862	GLTPD2	glycolipid transfer protein domain containing 2 [Source:HGNC Symbol;Acc:HGNC:33756]
ENSG00000204054	-0,662522	2,99258562	11,9670661	0,00687919	0,11425862	LINC00963	long intergenic non-protein coding RNA 963 [Source:HGNC Symbol;Acc:HGNC:48716]
ENSG00000080815	0,58688611	5,39362199	11,9227958	0,00695019	0,11498903	PSEN1	presenilin 1 [Source:HGNC Symbol;Acc:HGNC:9508]
ENSG00000099251	-0,5328076	3,57707245	11,926262	0,0069446	0,11498903	HSD17B7P2	hydroxysteroid 17-beta dehydrogenase 7 pseudogene 2 [Source:HGNC Symbol;Acc:HGNC:28120]
ENSG00000066279	0,69813498	3,53549815	11,9072793	0,00697528	0,11514343	ASPM	assembly factor for spindle microtubules [Source:HGNC Symbol;Acc:HGNC:19048]
ENSG00000167105	-2,0124505	-1,5386502	11,907827	0,0069744	0,11514343	TMEM92	transmembrane protein 92 [Source:HGNC Symbol;Acc:HGNC:26579]
ENSG00000117748	0,61000224	6,01487987	11,8877804	0,00700698	0,11553607	RPA2	replication protein A2 [Source:HGNC Symbol;Acc:HGNC:10290]
ENSG00000006282	-0,4652181	6,55244866	11,8584596	0,00705497	0,11580456	SPATA20	spermatogenesis associated 20 [Source:HGNC Symbol;Acc:HGNC:26125]
ENSG00000147471	0,47225119	5,56364383	11,8641739	0,00704559	0,11580456	PLPBP	pyridoxal phosphate binding protein [Source:HGNC Symbol;Acc:HGNC:9457]
ENSG00000158169	0,53826216	4,09201425	11,8629387	0,00704761	0,11580456	FANCC	FA complementation group C [Source:HGNC Symbol;Acc:HGNC:3584]
ENSG00000197451	-0,6298817	7,61768172	11,8706805	0,00703492	0,11580456	HNRNPAB	heterogeneous nuclear ribonucleoprotein A/B [Source:HGNC Symbol;Acc:HGNC:5034]
ENSG00000112337	0,75664776	3,31913772	11,8527637	0,00706434	0,11582821	SLC17A2	solute carrier family 17 member 2 [Source:HGNC Symbol;Acc:HGNC:10930]
ENSG00000260604	-1,0213777	0,89839319	11,8477349	0,00707262	0,11583405	AL590004.3	novel transcript
ENSG00000124143	-0,8723633	1,19671515	11,8334736	0,00709618	0,11608976	ARHGAP40	Rho GTPase activating protein 40 [Source:HGNC Symbol;Acc:HGNC:16226]
ENSG00000226499	-1,4077891	-1,0636615	11,8229345	0,00711366	0,11624542	AL136380.1	ribosomal protein S2 (RPS2) pseudogene
ENSG00000173540	-0,5602455	4,82713047	11,8169225	0,00712365	0,11627861	GMPPB	GDP-mannose pyrophosphorylase B [Source:HGNC Symbol;Acc:HGNC:22932]
ENSG00000134901	0,461578	6,20504241	11,7877657	0,00717234	0,11694282	POGLUT2	protein O-glucosyltransferase 2 [Source:HGNC Symbol;Acc:HGNC:19350]
ENSG00000189410	-0,8539438	3,23512038	11,7795324	0,00718617	0,1170376	SH2D5	SH2 domain containing 5 [Source:HGNC Symbol;Acc:HGNC:28819]
ENSG00000006016	-0,4784582	9,41777177	11,7703366	0,00720165	0,11705719	CRLF1	cytokine receptor like factor 1 [Source:HGNC Symbol;Acc:HGNC:2364]
ENSG00000115641	-0,7740166	2,77287446	11,7643705	0,00721171	0,11705719	FHL2	four and a half LIM domains 2 [Source:HGNC Symbol;Acc:HGNC:3703]
ENSG00000164168	-0,6901686	2,96619678	11,7665613	0,00720801	0,11705719	TMEM184C	transmembrane protein 184C [Source:HGNC Symbol;Acc:HGNC:25587]
ENSG00000176912	-0,8338119	2,66376709	11,7598071	0,00721942	0,11705719	TYMSOS	TYMS opposite strand [Source:HGNC Symbol;Acc:HGNC:29553]
ENSG00000124151	0,60712579	5,91677025	11,7517675	0,00723303	0,11710494	NCOA3	nuclear receptor coactivator 3 [Source:HGNC Symbol;Acc:HGNC:7670]
ENSG00000204370	-0,521955	5,35409783	11,7486001	0,0072384	0,11710494	SDHD	succinate dehydrogenase complex subunit D [Source:HGNC Symbol;Acc:HGNC:10683]
ENSG00000175482	-0,9048674	0,63884924	11,7290973	0,00727157	0,11751144	POLD4	DNA polymerase delta 4, accessory subunit [Source:HGNC Symbol;Acc:HGNC:14106]
ENSG00000142733	-0,917029	0,9343494	11,7034791	0,00731542	0,11782911	MAP3K6	mitogen-activated protein kinase kinase kinase 6 [Source:HGNC Symbol;Acc:HGNC:6858]
ENSG00000177000	-0,7007698	3,1864116	11,7077066	0,00730816	0,11782911	MTHFR	methylenetetrahydrofolate reductase [Source:HGNC Symbol;Acc:HGNC:7436]
ENSG00000180767	-0,5206997	5,91744994	11,7057619	0,0073115	0,11782911	CHST13	carbohydrate sulfotransferase 13 [Source:HGNC Symbol;Acc:HGNC:21755]
ENSG00000143398	0,54092542	5,43324344	11,6961758	0,00732798	0,11790145	PIP5K1A	phosphatidylinositol-4-phosphate 5-kinase type 1 alpha [Source:HGNC Symbol;Acc:HGNC:8994]
ENSG00000129667	-0,739563	4,47285923	11,6909672	0,00733696	0,11791598	RHBDF2	rhomboid 5 homolog 2 [Source:HGNC Symbol;Acc:HGNC:20788]
ENSG00000159082	0,82192749	1,04139262	11,6827893	0,00735108	0,11801305	SYNJ1	synaptojanin 1 [Source:HGNC Symbol;Acc:HGNC:11503]
ENSG00000215440	-1,9227746	-1,8440247	11,6773836	0,00736043	0,11803345	NPEPL1	aminopeptidase like 1 [Source:HGNC Symbol;Acc:HGNC:16244]
ENSG00000085978	0,48377758	5,88529382	11,6723818	0,00736909	0,11804282	ATG16L1	autophagy related 16 like 1 [Source:HGNC Symbol;Acc:HGNC:21498]
ENSG00000235609	1,12665928	0,28162387	11,6566434	0,00739644	0,11835109	AF127577.4	novel transcript
ENSG00000167173	0,49509632	5,86655067	11,6444783	0,00741766	0,11856081	C15orf39	chromosome 15 open reading frame 39 [Source:HGNC Symbol;Acc:HGNC:24497]
ENSG00000204149	2,02409939	-2,0236979	11,6334813	0,00743691	0,11873856	AGAP6	ArfGAP with GTPase domain, ankyrin repeat and PH domain 6 [Source:HGNC Symbol;Acc:HGNC:23466]
ENSG00000179922	-0,6302224	2,16768209	11,6150134	0,00746937	0,11912667	ZNF784	zinc finger protein 784 [Source:HGNC Symbol;Acc:HGNC:33111]
ENSG00000160472	-1,6476857	-0,8050973	11,6052712	0,00748656	0,11916053	TMEM190	transmembrane protein 190 [Source:HGNC Symbol;Acc:HGNC:29632]
ENSG00000162520	-0,8680887	2,05939481	11,6045686	0,00748781	0,11916053	SYNC	syncoilin, intermediate filament protein [Source:HGNC Symbol;Acc:HGNC:28897]
ENSG00000186020	0,82433362	0,8011024	11,5689207	0,00755115	0,11990734	ZNF529	zinc finger protein 529 [Source:HGNC Symbol;Acc:HGNC:29328]
ENSG00000286115	2,23573849	-1,8398457	11,5692465	0,00755057	0,11990734	AC010092.1	novel transcript
ENSG00000146859	0,8076535	3,78460226	11,5626299	0,0075624	0,11995554	TMEM140	transmembrane protein 140 [Source:HGNC Symbol;Acc:HGNC:21870]
ENSG00000080546	0,52044136	4,72885404	11,5549287	0,00757619	0,12004403	SESN1	sestrin 1 [Source:HGNC Symbol;Acc:HGNC:21595]
ENSG00000198246	0,55450454	5,29971249	11,5471485	0,00759016	0,12013506	SLC29A3	solute carrier family 29 member 3 [Source:HGNC Symbol;Acc:HGNC:23096]

ENSG00000181666	0,7340142	3,20106223	11,5415279	0,00760027	0,1201649	ZNF875	zinc finger protein 875 [Source:HGNC Symbol;Acc:HGNC:4928]
ENSG00000149582	0,51534728	3,7666589	11,5272286	0,00762607	0,12044242	TMEM25	transmembrane protein 25 [Source:HGNC Symbol;Acc:HGNC:25890]
ENSG00000010626	-0,7326969	1,97417196	11,4768581	0,0077178	0,12139227	LRRC23	leucine rich repeat containing 23 [Source:HGNC Symbol;Acc:HGNC:19138]
ENSG00000012061	-0,616791	5,22567463	11,4868196	0,00769955	0,12139227	ERCC1	ERCC excision repair 1, endonuclease non-catalytic subunit [Source:HGNC Symbol;Acc:HGNC:3433]
ENSG00000183762	0,6460993	3,20735436	11,4759589	0,00771945	0,12139227	KREMEN1	kringle containing transmembrane protein 1 [Source:HGNC Symbol;Acc:HGNC:17550]
ENSG00000188747	-1,5567547	-0,8649769	11,4776604	0,00771633	0,12139227	NOXA1	NADPH oxidase activator 1 [Source:HGNC Symbol;Acc:HGNC:10668]
ENSG00000173267	-2,1991698	-1,7505797	11,4631793	0,00774294	0,12163079	SNCG	synuclein gamma [Source:HGNC Symbol;Acc:HGNC:11141]
ENSG00000118785	0,81740462	10,1722182	11,4583352	0,00775187	0,12164024	SPP1	secreted phosphoprotein 1 [Source:HGNC Symbol;Acc:HGNC:11255]
ENSG00000080031	-0,6426758	4,55038752	11,453177	0,00776139	0,12165896	PTPRH	protein tyrosine phosphatase receptor type H [Source:HGNC Symbol;Acc:HGNC:9672]
ENSG00000131165	0,4795035	6,4853067	11,4332342	0,00779833	0,12210704	CHMP1A	charged multivesicular body protein 1A [Source:HGNC Symbol;Acc:HGNC:8740]
ENSG00000117153	-0,4838263	4,82074108	11,4099467	0,00784175	0,12223955	KLHL12	kelch like family member 12 [Source:HGNC Symbol;Acc:HGNC:19360]
ENSG00000137802	0,57071425	2,62455184	11,4062672	0,00784863	0,12223955	MAPKBP1	mitogen-activated protein kinase binding protein 1 [Source:HGNC Symbol;Acc:HGNC:29536]
ENSG00000152332	0,7738847	2,57287835	11,4161135	0,00783022	0,12223955	UHMK1	U2AF homology motif kinase 1 [Source:HGNC Symbol;Acc:HGNC:19683]
ENSG00000167588	-0,6589098	4,82657551	11,4143759	0,00783347	0,12223955	GPD1	glycerol-3-phosphate dehydrogenase 1 [Source:HGNC Symbol;Acc:HGNC:4455]
ENSG00000189079	1,02661684	2,51073546	11,4125619	0,00783686	0,12223955	ARID2	AT-rich interaction domain 2 [Source:HGNC Symbol;Acc:HGNC:18037]
ENSG00000035115	0,71873837	2,45480194	11,3659415	0,00792459	0,12242282	SH3YL1	SH3 and SYLF domain containing 1 [Source:HGNC Symbol;Acc:HGNC:29546]
ENSG00000061656	-0,7150165	2,09646769	11,3440882	0,00796613	0,12242282	SPAG4	sperm associated antigen 4 [Source:HGNC Symbol;Acc:HGNC:11214]
ENSG00000078487	-0,9548032	0,73809223	11,3613226	0,00793335	0,12242282	ZCWPW1	zinc finger CW-type and PWWP domain containing 1 [Source:HGNC Symbol;Acc:HGNC:23486]
ENSG00000158623	0,56393787	4,51734975	11,3829725	0,0078924	0,12242282	COPG2	COPI coat complex subunit gamma 2 [Source:HGNC Symbol;Acc:HGNC:2237]
ENSG00000163293	1,60607619	-0,8794427	11,3670221	0,00792255	0,12242282	NIPAL1	NIPA like domain containing 1 [Source:HGNC Symbol;Acc:HGNC:27194]
ENSG00000183864	0,48635432	5,37430296	11,3542283	0,00794683	0,12242282	TOB2	transducer of ERBB2, 2 [Source:HGNC Symbol;Acc:HGNC:11980]
ENSG00000187808	-1,2870546	0,21284547	11,3646568	0,00792703	0,12242282	SOWAHD	sosondowah ankyrin repeat domain family member D [Source:HGNC Symbol;Acc:HGNC:32960]
ENSG00000188026	-0,5417257	3,24344918	11,3424072	0,00796934	0,12242282	RILPL1	Rab interacting lysosomal protein like 1 [Source:HGNC Symbol;Acc:HGNC:26814]
ENSG00000196411	0,4367813	7,70829412	11,3937825	0,00787206	0,12242282	EPHB4	EPH receptor B4 [Source:HGNC Symbol;Acc:HGNC:3395]
ENSG00000196754	-0,9900781	0,25753325	11,3497851	0,00795528	0,12242282	S100A2	S100 calcium binding protein A2 [Source:HGNC Symbol;Acc:HGNC:10492]
ENSG00000223496	-0,4871136	5,14132223	11,3510144	0,00795294	0,12242282	EXOSC6	exosome component 6 [Source:HGNC Symbol;Acc:HGNC:19055]
ENSG00000224790	-1,6310784	-0,945817	11,3578691	0,00793991	0,12242282	AP000704.1	novel transcript
ENSG00000230863	1,57996384	-1,5228556	11,3505595	0,00795381	0,12242282	AL137803.1	histidine acid phosphatase domain containing 1 (HISPPD1) pseudogene
ENSG00000172954	0,68265625	3,63146042	11,2990265	0,00805263	0,12357242	LCLAT1	lysocardiolipin acyltransferase 1 [Source:HGNC Symbol;Acc:HGNC:26756]
ENSG00000108094	0,54270988	4,88409648	11,2869252	0,00807606	0,12371934	CUL2	cullin 2 [Source:HGNC Symbol;Acc:HGNC:2552]
ENSG00000198408	0,63003139	4,78618655	11,2853339	0,00807915	0,12371934	OGA	O-GlcNAcase [Source:HGNC Symbol;Acc:HGNC:7056]
ENSG00000088826	-0,4663381	5,35283764	11,2725365	0,00810402	0,12388854	SMOX	spermine oxidase [Source:HGNC Symbol;Acc:HGNC:15862]
ENSG00000122126	0,53414491	4,12427339	11,2701394	0,00810869	0,12388854	OCRL	OCRL inositol polyphosphate-5-phosphatase [Source:HGNC Symbol;Acc:HGNC:8108]
ENSG00000143321	0,44790069	8,66761219	11,2665774	0,00811564	0,12388854	HDGF	heparin binding growth factor [Source:HGNC Symbol;Acc:HGNC:4856]
ENSG00000080839	0,5577358	4,25202794	11,2586308	0,00813116	0,12393728	RBL1	RB transcriptional corepressor like 1 [Source:HGNC Symbol;Acc:HGNC:9893]
ENSG00000177669	1,19736994	-0,2997589	11,2535494	0,0081411	0,12393728	MBOAT4	membrane bound O-acyltransferase domain containing 4 [Source:HGNC Symbol;Acc:HGNC:32311]
ENSG00000186416	-0,5025231	3,44186455	11,2519263	0,00814428	0,12393728	NKRF	NFKB repressing factor [Source:HGNC Symbol;Acc:HGNC:19374]
ENSG00000117000	0,56428761	3,73189813	11,2416702	0,00816441	0,12398523	RLF	RLF zinc finger [Source:HGNC Symbol;Acc:HGNC:10025]
ENSG00000187601	0,79548288	2,43391216	11,2425182	0,00816274	0,12398523	MAGEH1	MAGE family member H1 [Source:HGNC Symbol;Acc:HGNC:24092]
ENSG00000123607	0,50805577	5,05332229	11,2297237	0,00818792	0,1241343	TTC21B	tetratricopeptide repeat domain 21B [Source:HGNC Symbol;Acc:HGNC:25660]
ENSG00000133800	-0,9700157	1,61171047	11,223751	0,00819971	0,1241343	LYVE1	lymphatic vessel endothelial hyaluronan receptor 1 [Source:HGNC Symbol;Acc:HGNC:14687]
ENSG00000198937	-0,6185516	5,3516	11,2241715	0,00819888	0,1241343	CCDC167	coiled-coil domain containing 167 [Source:HGNC Symbol;Acc:HGNC:21239]
ENSG00000005889	0,57188933	3,88033436	11,1988144	0,00824916	0,12434361	ZFX	zinc finger protein X-linked [Source:HGNC Symbol;Acc:HGNC:12869]
ENSG00000137496	1,38292885	-0,0774911	11,198761	0,00824927	0,12434361	IL18BP	interleukin 18 binding protein [Source:HGNC Symbol;Acc:HGNC:5987]

ENSG00000164967	-0,5573253	4,34107031	11,1946857	0,00825738	0,12434361	RPP25L	ribonuclease P/MRP subunit p25 like [Source:HGNC Symbol;Acc:HGNC:19909]
ENSG00000164985	-0,5906461	5,03006687	11,1933357	0,00826007	0,12434361	PSIP1	PC4 and SFRS1 interacting protein 1 [Source:HGNC Symbol;Acc:HGNC:9527]
ENSG00000175003	1,59035693	-1,3911966	11,2077007	0,0082315	0,12434361	SLC22A1	solute carrier family 22 member 1 [Source:HGNC Symbol;Acc:HGNC:10963]
ENSG00000273669	-2,7115433	-2,2548835	11,1910612	0,00826461	0,12434361	AC015819.1	novel transcript
ENSG00000004660	-0,7284695	2,09398896	11,1230352	0,00840168	0,12449851	CAMKK1	calcium/calmodulin dependent protein kinase kinase 1 [Source:HGNC Symbol;Acc:HGNC:1469]
ENSG00000019485	0,59734361	2,39211726	11,1452423	0,00835663	0,12449851	PRDM11	PR/SET domain 11 [Source:HGNC Symbol;Acc:HGNC:13996]
ENSG00000087502	-0,7137164	4,73870783	11,1278502	0,00839188	0,12449851	ERGIC2	ERGIC and golgi 2 [Source:HGNC Symbol;Acc:HGNC:30208]
ENSG00000110583	0,48186308	4,43193026	11,1318066	0,00838385	0,12449851	NAA40	N-alpha-acetyltransferase 40, NatD catalytic subunit [Source:HGNC Symbol;Acc:HGNC:25845]
ENSG00000119685	0,59424694	2,83997628	11,1590491	0,00832877	0,12449851	TTLL5	tubulin tyrosine ligase like 5 [Source:HGNC Symbol;Acc:HGNC:19963]
ENSG00000132953	0,54397026	3,66401691	11,1773269	0,00829206	0,12449851	XPO4	exportin 4 [Source:HGNC Symbol;Acc:HGNC:17796]
ENSG00000137135	-0,5581101	3,87664453	11,1523096	0,00834235	0,12449851	ARHGEF39	Rho guanine nucleotide exchange factor 39 [Source:HGNC Symbol;Acc:HGNC:25909]
ENSG00000143867	0,91900819	0,47711252	11,1183322	0,00841126	0,12449851	OSR1	odd-skipped related transcription factor 1 [Source:HGNC Symbol;Acc:HGNC:8111]
ENSG00000157654	-0,9341265	2,28513301	11,1294951	0,00838854	0,12449851	PALM2AKAP2	PALM2 and AKAP2 fusion [Source:HGNC Symbol;Acc:HGNC:33529]
ENSG00000165644	-0,5623149	3,60510042	11,1319892	0,00838348	0,12449851	COMTD1	catechol-O-methyltransferase domain containing 1 [Source:HGNC Symbol;Acc:HGNC:26309]
ENSG00000165792	0,51334604	7,41052914	11,1432915	0,00836057	0,12449851	METTL17	methyltransferase like 17 [Source:HGNC Symbol;Acc:HGNC:19280]
ENSG00000170471	0,51281544	5,03911192	11,1214746	0,00840485	0,12449851	RALGAPB	Ral GTPase activating protein non-catalytic subunit beta [Source:HGNC Symbol;Acc:HGNC:29221]
ENSG00000213639	-0,4818352	4,65411849	11,1725555	0,00830162	0,12449851	PPP1CB	protein phosphatase 1 catalytic subunit beta [Source:HGNC Symbol;Acc:HGNC:9282]
ENSG00000225721	-1,6852533	-1,75658	11,1470779	0,00835292	0,12449851	AL592166.1	novel transcript
ENSG00000226950	-0,7012773	5,56553395	11,1712642	0,00830421	0,12449851	DANCR	differentiation antagonizing non-protein coding RNA [Source:HGNC Symbol;Acc:HGNC:28964]
ENSG00000259985	-1,5480311	-1,1577894	11,1539045	0,00833913	0,12449851	AC017100.1	novel transcript, antisense to B4GALT6
ENSG00000118523	0,79219156	9,03336465	11,1100397	0,00842818	0,12462272	CCN2	cellular communication network factor 2 [Source:HGNC Symbol;Acc:HGNC:2500]
ENSG00000011114	0,56326323	2,83266816	11,0971447	0,00845457	0,12476048	BTBD7	BTB domain containing 7 [Source:HGNC Symbol;Acc:HGNC:18269]
ENSG00000170113	0,49354477	4,55953765	11,0981695	0,00845247	0,12476048	NIPA1	NIPA magnesium transporter 1 [Source:HGNC Symbol;Acc:HGNC:17043]
ENSG00000143390	-0,6409818	3,9096283	11,0834056	0,00848281	0,12505085	RFX5	regulatory factor X5 [Source:HGNC Symbol;Acc:HGNC:9986]
ENSG00000148677	-1,0194535	6,07834457	11,0521052	0,00854757	0,12537297	ANKRD1	ankyrin repeat domain 1 [Source:HGNC Symbol;Acc:HGNC:15819]
ENSG00000171124	1,2594671	-0,3409376	11,0599562	0,00853127	0,12537297	FUT3	fucosyltransferase 3 (Lewis blood group) [Source:HGNC Symbol;Acc:HGNC:4014]
ENSG00000185753	0,59617206	3,36998131	11,0540952	0,00854344	0,12537297	CXorf38	chromosome X open reading frame 38 [Source:HGNC Symbol;Acc:HGNC:28589]
ENSG00000198944	-1,0182753	0,11506336	11,0652369	0,00852033	0,12537297	SOWAHA	sosondowah ankyrin repeat domain family member A [Source:HGNC Symbol;Acc:HGNC:27033]
ENSG00000227500	0,45763453	6,2026556	11,0557542	0,00853999	0,12537297	SCAMP4	secretory carrier membrane protein 4 [Source:HGNC Symbol;Acc:HGNC:30385]
ENSG00000096872	0,54546243	3,12323297	11,0384616	0,00857599	0,12555802	IFT74	intraflagellar transport 74 [Source:HGNC Symbol;Acc:HGNC:21424]
ENSG00000204710	-1,698498	-1,7363725	11,0295731	0,00859457	0,12555802	SPDYC	speedy/RINGO cell cycle regulator family member C [Source:HGNC Symbol;Acc:HGNC:32681]
ENSG00000217801	-1,0091514	0,31435611	11,0343867	0,0085845	0,12555802	AL390719.1	protein tyrosine phosphatase family pseudogene
ENSG00000244687	0,90129558	0,44553115	11,0321746	0,00858912	0,12555802	UBE2V1	ubiquitin conjugating enzyme E2 V1 [Source:HGNC Symbol;Acc:HGNC:12494]
ENSG00000186212	0,90104169	1,17819592	11,0170994	0,00862072	0,12581426	SOWAHB	sosondowah ankyrin repeat domain family member B [Source:HGNC Symbol;Acc:HGNC:32958]
ENSG00000251247	1,42950807	-0,3833619	11,0017746	0,00865298	0,12615908	ZNF345	zinc finger protein 345 [Source:HGNC Symbol;Acc:HGNC:16367]
ENSG00000110245	-0,6453683	7,19222791	10,9964242	0,00866428	0,12619787	APOC3	apolipoprotein C3 [Source:HGNC Symbol;Acc:HGNC:610]
ENSG00000089351	-0,4386152	5,66010157	10,988649	0,00868073	0,12621079	GRAMD1A	GRAM domain containing 1A [Source:HGNC Symbol;Acc:HGNC:29305]
ENSG00000118894	-0,4768807	5,56064818	10,98784	0,00868245	0,12621079	EEF2KMT	eukaryotic elongation factor 2 lysine methyltransferase [Source:HGNC Symbol;Acc:HGNC:32221]
ENSG00000137834	-0,7069264	2,07835033	10,975258	0,00870916	0,12647321	SMAD6	SMAD family member 6 [Source:HGNC Symbol;Acc:HGNC:6772]
ENSG00000103479	0,58125685	5,35608848	10,9452236	0,00877332	0,12652465	RBL2	RB transcriptional corepressor like 2 [Source:HGNC Symbol;Acc:HGNC:9894]
ENSG00000104213	0,79193091	3,88773389	10,9595442	0,00874266	0,12652465	PDGFRL	platelet derived growth factor receptor like [Source:HGNC Symbol;Acc:HGNC:8805]
ENSG00000127946	0,71977394	1,90079878	10,947926	0,00876753	0,12652465	HIP1	huntingtin interacting protein 1 [Source:HGNC Symbol;Acc:HGNC:4913]
ENSG00000144357	0,70584394	4,38656335	10,9530094	0,00875663	0,12652465	UBR3	ubiquitin protein ligase E3 component n-recognin 3 [Source:HGNC Symbol;Acc:HGNC:30467]
ENSG00000144647	0,50189932	5,35186423	10,9471007	0,0087693	0,12652465	POMGNT2	protein O-linked mannose N-acetylglucosaminyltransferase 2 (beta 1,4-) [Source:HGNC Symbol;Acc:HGNC:25902]

ENSG00000162676	0,53747152	2,66439818	10,9567921	0,00874854	0,12652465	GFI1	growth factor independent 1 transcriptional repressor [Source:HGNC Symbol;Acc:HGNC:4237]
ENSG00000164308	0,56926696	4,1774505	10,9595592	0,00874262	0,12652465	ERAP2	endoplasmic reticulum aminopeptidase 2 [Source:HGNC Symbol;Acc:HGNC:29499]
ENSG00000170312	-0,6156934	5,6455682	10,9369048	0,0087912	0,1266574	CDK1	cyclin dependent kinase 1 [Source:HGNC Symbol;Acc:HGNC:1722]
ENSG00000158806	-0,578092	3,96710447	10,9007554	0,00886939	0,12743928	NPM2	nucleophosmin/nucleoplasmin 2 [Source:HGNC Symbol;Acc:HGNC:7930]
ENSG00000173085	-0,4862437	4,93235181	10,9057907	0,00885845	0,12743928	COQ2	coenzyme Q2, polyprenyltransferase [Source:HGNC Symbol;Acc:HGNC:25223]
ENSG00000234771	1,3286675	0,31385929	10,8997238	0,00887164	0,12743928	SLC25A25-AS1	SLC25A25 antisense RNA 1 [Source:HGNC Symbol;Acc:HGNC:27844]
ENSG00000139921	-0,5345166	6,06003681	10,8879405	0,00889732	0,12755732	TMX1	thioredoxin related transmembrane protein 1 [Source:HGNC Symbol;Acc:HGNC:15487]
ENSG00000279970	-1,5792261	-0,5501103	10,8914543	0,00888965	0,12755732	AC023024.2	TEC
ENSG00000197647	0,71267952	2,38565666	10,8791432	0,00891655	0,12758462	ZNF433	zinc finger protein 433 [Source:HGNC Symbol;Acc:HGNC:20811]
ENSG00000198959	-1,2117979	4,14882542	10,8790797	0,00891669	0,12758462	TGM2	transglutaminase 2 [Source:HGNC Symbol;Acc:HGNC:11778]
ENSG00000102890	-0,7823811	1,5119609	10,8723305	0,00893148	0,1276712	ELMO3	engulfment and cell motility 3 [Source:HGNC Symbol;Acc:HGNC:17289]
ENSG00000092148	0,54987681	7,46111479	10,8666219	0,00894401	0,12772537	HECTD1	HECT domain E3 ubiquitin protein ligase 1 [Source:HGNC Symbol;Acc:HGNC:20157]
ENSG00000008282	0,49102881	6,17810164	10,8531582	0,00897365	0,12789865	SYPL1	synaptophysin like 1 [Source:HGNC Symbol;Acc:HGNC:11507]
ENSG00000182809	-0,5035158	4,42873514	10,8538524	0,00897212	0,12789865	CRIP2	cysteine rich protein 2 [Source:HGNC Symbol;Acc:HGNC:2361]
ENSG00000099899	-0,47771	4,89455608	10,8307704	0,00902321	0,12810508	TRMT2A	tRNA methyltransferase 2 homolog A [Source:HGNC Symbol;Acc:HGNC:24974]
ENSG00000130147	0,44546349	5,85800292	10,8377744	0,00900767	0,12810508	SH3BP4	SH3 domain binding protein 4 [Source:HGNC Symbol;Acc:HGNC:10826]
ENSG00000165209	0,4906198	5,4962895	10,8399614	0,00900283	0,12810508	STRBP	spermatid perinuclear RNA binding protein [Source:HGNC Symbol;Acc:HGNC:16462]
ENSG00000171150	0,73991013	3,63470286	10,8313792	0,00902186	0,12810508	SOCS5	suppressor of cytokine signaling 5 [Source:HGNC Symbol;Acc:HGNC:16852]
ENSG00000107789	0,54768208	5,95425089	10,8061364	0,00907813	0,12875965	MINPP1	multiple inositol-polyphosphate phosphatase 1 [Source:HGNC Symbol;Acc:HGNC:7102]
ENSG00000266714	-1,2614384	0,81036351	10,7942446	0,00910479	0,1290125	MYO15B	myosin XVB [Source:HGNC Symbol;Acc:HGNC:14083]
ENSG00000166912	-0,5234449	3,11171678	10,7807357	0,00913519	0,12931783	MTMR10	myotubularin related protein 10 [Source:HGNC Symbol;Acc:HGNC:25999]
ENSG00000137200	0,60177097	6,35044064	10,7723687	0,00915408	0,1293346	CMTR1	cap methyltransferase 1 [Source:HGNC Symbol;Acc:HGNC:21077]
ENSG00000186918	0,61089072	2,44104926	10,7752689	0,00914753	0,1293346	ZNF395	zinc finger protein 395 [Source:HGNC Symbol;Acc:HGNC:18737]
ENSG00000099864	0,55247919	3,10659698	10,7378573	0,00923251	0,12946456	PALM	paralemmin [Source:HGNC Symbol;Acc:HGNC:8594]
ENSG00000115841	-0,5721923	2,32137439	10,7346834	0,00923976	0,12946456	RMDN2	regulator of microtubule dynamics 2 [Source:HGNC Symbol;Acc:HGNC:26567]
ENSG00000142327	-0,4807028	5,8853503	10,7361966	0,0092363	0,12946456	RNPEPL1	arginyl aminopeptidase like 1 [Source:HGNC Symbol;Acc:HGNC:10079]
ENSG00000177984	-0,5417592	3,55877854	10,7380636	0,00923204	0,12946456	LCN15	lipocalin 15 [Source:HGNC Symbol;Acc:HGNC:33777]
ENSG00000188215	0,54630134	2,68294196	10,7423127	0,00922234	0,12946456	DCUN1D3	defective in cullin neddylation 1 domain containing 3 [Source:HGNC Symbol;Acc:HGNC:28734]
ENSG00000196136	-0,7889187	1,98091834	10,7477883	0,00920986	0,12946456	SERPINA3	serpin family A member 3 [Source:HGNC Symbol;Acc:HGNC:16]
ENSG00000205730	0,56526393	4,39963465	10,7293798	0,0092519	0,12946456	ITPRIPL2	ITPRIP like 2 [Source:HGNC Symbol;Acc:HGNC:27257]
ENSG00000206190	-0,7943433	1,75468096	10,7540732	0,00919556	0,12946456	ATP10A	ATPase phospholipid transporting 10A (putative) [Source:HGNC Symbol;Acc:HGNC:13542]
ENSG00000211452	-0,7133762	4,21513766	10,7442337	0,00921796	0,12946456	DIO1	iodothyronine deiodinase 1 [Source:HGNC Symbol;Acc:HGNC:2883]
ENSG00000215030	-0,5217434	4,29370035	10,7332267	0,00924309	0,12946456	RPL13P12	ribosomal protein L13 pseudogene 12 [Source:HGNC Symbol;Acc:HGNC:35701]
ENSG00000091140	0,61208122	7,41325057	10,7231547	0,00926617	0,12951415	DLD	dihydrolipoamide dehydrogenase [Source:HGNC Symbol;Acc:HGNC:2898]
ENSG00000137561	0,9641716	0,63993731	10,7201035	0,00927317	0,12951415	TTPA	alpha tocopherol transfer protein [Source:HGNC Symbol;Acc:HGNC:12404]
ENSG00000004059	0,46029002	7,25144216	10,6984174	0,00932315	0,12986529	ARF5	ADP ribosylation factor 5 [Source:HGNC Symbol;Acc:HGNC:658]
ENSG00000121851	-0,5886555	1,96552369	10,6934044	0,00933474	0,12986529	POLR3GL	RNA polymerase III subunit GL [Source:HGNC Symbol;Acc:HGNC:28466]
ENSG00000164266	-0,7956618	1,42020127	10,7050849	0,00930775	0,12986529	SPINK1	serine peptidase inhibitor Kazal type 1 [Source:HGNC Symbol;Acc:HGNC:11244]
ENSG00000164877	-0,9978283	1,55214852	10,6822812	0,00936054	0,12986529	MICALL2	MICAL like 2 [Source:HGNC Symbol;Acc:HGNC:29672]
ENSG00000197019	-0,5422156	3,27419313	10,6846943	0,00935494	0,12986529	SERTAD1	SERTA domain containing 1 [Source:HGNC Symbol;Acc:HGNC:17932]
ENSG00000235655	-0,6208489	2,86878377	10,6967455	0,00932701	0,12986529	H3P6	H3 histone pseudogene 6 [Source:HGNC Symbol;Acc:HGNC:42980]
ENSG00000267169	-1,2641814	-0,7543701	10,6839239	0,00935673	0,12986529	AC022098.1	novel transcript, antisense to LPHN1
ENSG00000117479	0,69290514	5,28045093	10,6735611	0,00938083	0,1299024	SLC19A2	solute carrier family 19 member 2 [Source:HGNC Symbol;Acc:HGNC:10938]
ENSG00000173846	-0,5909366	4,26727076	10,6734868	0,009381	0,1299024	PLK3	polo like kinase 3 [Source:HGNC Symbol;Acc:HGNC:2154]

ENSG00000150712	0,46397474	5,32666892	10,6612411	0,00940958	0,13017476	MTMR12	myotubularin related protein 12 [Source:HGNC Symbol;Acc:HGNC:18191]
ENSG00000132824	-0,5196293	6,54939276	10,6479566	0,0094407	0,13025551	SERINC3	serine incorporator 3 [Source:HGNC Symbol;Acc:HGNC:11699]
ENSG00000164109	0,48205406	6,95456231	10,6473342	0,00944216	0,13025551	MAD2L1	mitotic arrest deficient 2 like 1 [Source:HGNC Symbol;Acc:HGNC:6763]
ENSG00000165169	-0,8149191	1,43460451	10,6485738	0,00943925	0,13025551	DYNLT3	dynein light chain Tctex-type 3 [Source:HGNC Symbol;Acc:HGNC:11694]
ENSG00000095739	0,59344437	5,50009409	10,6310035	0,00948061	0,13033689	BAMBI	BMP and activin membrane bound inhibitor [Source:HGNC Symbol;Acc:HGNC:30251]
ENSG00000150457	0,52668262	3,95543031	10,6386109	0,00946268	0,13033689	LATS2	large tumor suppressor kinase 2 [Source:HGNC Symbol;Acc:HGNC:6515]
ENSG00000183955	-0,5522979	4,52346863	10,6318191	0,00947868	0,13033689	KMT5A	lysine methyltransferase 5A [Source:HGNC Symbol;Acc:HGNC:29489]
ENSG00000231908	-1,0260068	0,77599392	10,6296713	0,00948375	0,13033689	IDH1-AS1	IDH1 antisense RNA 1 [Source:HGNC Symbol;Acc:HGNC:40292]
ENSG00000131791	0,52654788	4,78805534	10,6187333	0,00950962	0,13049545	PRKAB2	protein kinase AMP-activated non-catalytic subunit beta 2 [Source:HGNC Symbol;Acc:HGNC:9379]
ENSG00000236090	-1,2524168	-0,6800955	10,6172401	0,00951315	0,13049545	LDHAP3	lactate dehydrogenase A pseudogene 3 [Source:HGNC Symbol;Acc:HGNC:6538]
ENSG00000104325	-0,4621956	5,3886916	10,6028612	0,0095473	0,1307271	DECR1	2,4-dienoyl-CoA reductase 1 [Source:HGNC Symbol;Acc:HGNC:2753]
ENSG00000176401	0,69021422	1,70812668	10,6025941	0,00954794	0,1307271	EID2B	EP300 interacting inhibitor of differentiation 2B [Source:HGNC Symbol;Acc:HGNC:26796]
ENSG00000097046	0,65511954	2,94212409	10,5977642	0,00955944	0,13073858	CDC7	cell division cycle 7 [Source:HGNC Symbol;Acc:HGNC:1745]
ENSG00000167785	0,71091141	1,48142246	10,5947322	0,00956667	0,13073858	ZNF558	zinc finger protein 558 [Source:HGNC Symbol;Acc:HGNC:26422]
ENSG00000168386	-0,8589286	4,97318778	10,5841604	0,00959194	0,13096135	FILIP1L	filamin A interacting protein 1 like [Source:HGNC Symbol;Acc:HGNC:24589]
ENSG00000205707	-0,9507665	0,95328953	10,560272	0,00964933	0,13162193	ETFRF1	electron transfer flavoprotein regulatory factor 1 [Source:HGNC Symbol;Acc:HGNC:27052]
ENSG00000078142	0,61813205	4,71400005	10,5510882	0,00967151	0,13180137	PIK3C3	phosphatidylinositol 3-kinase catalytic subunit type 3 [Source:HGNC Symbol;Acc:HGNC:8974]
ENSG00000197324	-0,5301449	7,23958726	10,5314406	0,00971916	0,13232734	LRP10	LDL receptor related protein 10 [Source:HGNC Symbol;Acc:HGNC:14553]
ENSG00000105383	1,54551254	-1,4298651	10,5100761	0,00977131	0,13264109	CD33	CD33 molecule [Source:HGNC Symbol;Acc:HGNC:1659]
ENSG00000107742	-0,6301888	5,75062169	10,504253	0,00978558	0,13264109	SPOCK2	SPARC (osteonectin), cwcv and kazal like domains proteoglycan 2 [Source:HGNC Symbol;Acc:HGNC:13564]
ENSG00000131781	0,58929124	4,17537837	10,514256	0,00976108	0,13264109	FMO5	flavin containing dimethylaniline monoxygenase 5 [Source:HGNC Symbol;Acc:HGNC:3773]
ENSG00000186868	0,63741732	2,72665811	10,5094096	0,00977294	0,13264109	MAPT	microtubule associated protein tau [Source:HGNC Symbol;Acc:HGNC:6893]
ENSG00000188060	-0,484423	3,97988192	10,5034273	0,0097876	0,13264109	RAB42	RAB42, member RAS oncogene family [Source:HGNC Symbol;Acc:HGNC:28702]
ENSG00000105369	-1,2890487	-0,4558067	10,4976689	0,00980175	0,13270963	CD79A	CD79a molecule [Source:HGNC Symbol;Acc:HGNC:1698]
ENSG00000233154	-1,5368423	-1,3409592	10,4816368	0,00984125	0,13312113	LINC01762	long intergenic non-protein coding RNA 1762 [Source:HGNC Symbol;Acc:HGNC:52552]
ENSG00000007062	0,59015665	4,68625185	10,4262198	0,00997931	0,13344788	PROM1	prominin 1 [Source:HGNC Symbol;Acc:HGNC:9454]
ENSG00000093144	-0,4665372	6,66437212	10,4554457	0,00990621	0,13344788	ECHDC1	ethylmalonyl-CoA decarboxylase 1 [Source:HGNC Symbol;Acc:HGNC:21489]
ENSG00000106366	-1,2250043	9,23276639	10,4392125	0,00994673	0,13344788	SERPINE1	serpin family E member 1 [Source:HGNC Symbol;Acc:HGNC:8583]
ENSG00000116191	0,53364488	3,59829349	10,4259153	0,00998008	0,13344788	RALGPS2	Ral GEF with PH domain and SH3 binding motif 2 [Source:HGNC Symbol;Acc:HGNC:30279]
ENSG00000117133	0,43631794	5,70669663	10,4415284	0,00994094	0,13344788	RPF1	ribosome production factor 1 homolog [Source:HGNC Symbol;Acc:HGNC:30350]
ENSG00000119535	-0,6520648	3,07061996	10,4469319	0,00992744	0,13344788	CSF3R	colony stimulating factor 3 receptor [Source:HGNC Symbol;Acc:HGNC:2439]
ENSG00000139438	0,53053937	3,50201483	10,4465739	0,00992833	0,13344788	FAM222A	family with sequence similarity 222 member A [Source:HGNC Symbol;Acc:HGNC:25915]
ENSG00000144199	-0,5713977	3,39873061	10,4187564	0,00999809	0,13344788	FAHD2B	fumarylacetoacetate hydrolase domain containing 2B [Source:HGNC Symbol;Acc:HGNC:25318]
ENSG00000159720	-0,5079207	6,31163677	10,4170354	0,01000243	0,13344788	ATP6V0D1	ATPase H+ transporting V0 subunit d1 [Source:HGNC Symbol;Acc:HGNC:13724]
ENSG00000171557	-0,531271	10,8668997	10,4174088	0,01000148	0,13344788	FGG	fibrinogen gamma chain [Source:HGNC Symbol;Acc:HGNC:3694]
ENSG00000187134	0,49383704	10,3516466	10,4639043	0,00988517	0,13344788	AKR1C1	aldo-keto reductase family 1 member C1 [Source:HGNC Symbol;Acc:HGNC:384]
ENSG00000187775	-0,7384284	1,68270976	10,4636605	0,00988578	0,13344788	DNAH17	dynein axonemal heavy chain 17 [Source:HGNC Symbol;Acc:HGNC:2946]
ENSG00000240476	-1,8994482	-1,9514858	10,4396717	0,00994558	0,13344788	LINC00973	long intergenic non-protein coding RNA 973 [Source:HGNC Symbol;Acc:HGNC:48868]
ENSG00000254290	-0,7220906	2,01624023	10,4213631	0,00999153	0,13344788	AC124067.4	novel transcript
ENSG00000270194	-0,788313	0,99891347	10,4175924	0,01000102	0,13344788	AC097359.2	novel transcript, antisense to GOLGA4
ENSG00000008283	0,63325692	2,90086814	10,4037585	0,01003595	0,13352998	CYB561	cytochrome b561 [Source:HGNC Symbol;Acc:HGNC:2571]
ENSG00000066027	0,68525479	3,89726327	10,3984315	0,01004944	0,13352998	PPP2R5A	protein phosphatase 2 regulatory subunit B'alpha [Source:HGNC Symbol;Acc:HGNC:9309]
ENSG00000114391	-0,4503284	9,69229623	10,3965218	0,01005428	0,13352998	RPL24	ribosomal protein L24 [Source:HGNC Symbol;Acc:HGNC:10325]
ENSG00000125503	-0,4596227	4,84141665	10,4087199	0,01002341	0,13352998	PPP1R12C	protein phosphatase 1 regulatory subunit 12C [Source:HGNC Symbol;Acc:HGNC:14947]

ENSG00000203797	0,57423985	3,06348692	10,4036885	0,01003613	0,13352998	DDO	D-aspartate oxidase [Source:HGNC Symbol;Acc:HGNC:2727]
ENSG00000146350	1,48109889	-0,723889	10,3887701	0,01007396	0,13365459	TBC1D32	TBC1 domain family member 32 [Source:HGNC Symbol;Acc:HGNC:21485]
ENSG00000231050	-1,8328037	-1,5085009	10,3856247	0,01008196	0,13365459	AL109917.1	novel transcript
ENSG00000070081	-0,5451131	6,91608448	10,3405278	0,01019751	0,13371513	NUCB2	nucleobindin 2 [Source:HGNC Symbol;Acc:HGNC:8044]
ENSG00000077616	0,59960982	2,19726545	10,3279782	0,01022995	0,13371513	NAALAD2	N-acetylated alpha-linked acidic dipeptidase 2 [Source:HGNC Symbol;Acc:HGNC:14526]
ENSG00000079689	0,54414109	4,97828929	10,328306	0,0102291	0,13371513	SCGN	secretagogin, EF-hand calcium binding protein [Source:HGNC Symbol;Acc:HGNC:16941]
ENSG00000103254	-0,635948	3,55641554	10,3573555	0,0101542	0,13371513	ANTKMT	adenine nucleotide translocase lysine methyltransferase [Source:HGNC Symbol;Acc:HGNC:14152]
ENSG00000124207	0,45405226	7,75054206	10,3270416	0,01023238	0,13371513	CSE1L	chromosome segregation 1 like [Source:HGNC Symbol;Acc:HGNC:2431]
ENSG00000125686	0,79469301	4,75031451	10,378274	0,01010068	0,13371513	MED1	mediator complex subunit 1 [Source:HGNC Symbol;Acc:HGNC:9234]
ENSG00000132591	0,46586163	8,1762423	10,3455173	0,01018465	0,13371513	ERAL1	Era like 12S mitochondrial rRNA chaperone 1 [Source:HGNC Symbol;Acc:HGNC:3424]
ENSG00000164754	0,53347083	7,00052681	10,3388606	0,01020181	0,13371513	RAD21	RAD21 cohesin complex component [Source:HGNC Symbol;Acc:HGNC:9811]
ENSG00000168394	0,57203014	6,60528716	10,356472	0,01015647	0,13371513	TAP1	transporter 1, ATP binding cassette subfamily B member [Source:HGNC Symbol;Acc:HGNC:43]
ENSG00000172661	0,54092227	4,20620521	10,3731047	0,01011388	0,13371513	WASHC2C	WASH complex subunit 2C [Source:HGNC Symbol;Acc:HGNC:23414]
ENSG00000172992	-0,6054715	3,78187013	10,3509151	0,01017075	0,13371513	DCAKD	dephospho-CoA kinase domain containing [Source:HGNC Symbol;Acc:HGNC:26238]
ENSG00000184232	-0,4868352	7,91355254	10,3268119	0,01023297	0,13371513	OAF	out at first homolog [Source:HGNC Symbol;Acc:HGNC:28752]
ENSG00000197070	-0,5731105	3,29972345	10,3359277	0,01020939	0,13371513	ARRDC1	arrestin domain containing 1 [Source:HGNC Symbol;Acc:HGNC:28633]
ENSG00000256223	0,94162685	0,84950229	10,3533832	0,01016441	0,13371513	ZNF10	zinc finger protein 10 [Source:HGNC Symbol;Acc:HGNC:12879]
ENSG00000269335	-0,4862213	3,48145644	10,3447617	0,01018659	0,13371513	IKBKG	inhibitor of nuclear factor kappa B kinase regulatory subunit gamma [Source:HGNC Symbol;Acc:HGNC:5961]
ENSG00000277075	0,86628413	0,58398409	10,3315469	0,01022071	0,13371513	H2AC8	H2A clustered histone 8 [Source:HGNC Symbol;Acc:HGNC:4724]
ENSG00000105048	0,87664373	0,46320208	10,3038832	0,0102926	0,1337763	TNNT1	troponin T1, slow skeletal type [Source:HGNC Symbol;Acc:HGNC:11948]
ENSG00000115594	0,51381276	4,54128707	10,3205528	0,01024921	0,1337763	IL1R1	interleukin 1 receptor type 1 [Source:HGNC Symbol;Acc:HGNC:5993]
ENSG00000145050	-0,49031	9,13716894	10,3113024	0,01027326	0,1337763	MANF	mesencephalic astrocyte derived neurotrophic factor [Source:HGNC Symbol;Acc:HGNC:15461]
ENSG00000170525	-0,912463	2,1929051	10,3101969	0,01027614	0,1337763	PFKFB3	6-phosphofructo-2-kinase/fructose-2,6-biphosphatase 3 [Source:HGNC Symbol;Acc:HGNC:8874]
ENSG00000183032	0,59415427	2,48148934	10,3060666	0,0102869	0,1337763	SLC25A21	solute carrier family 25 member 21 [Source:HGNC Symbol;Acc:HGNC:14411]
ENSG00000239332	1,09542488	-0,0919994	10,3101594	0,01027623	0,1337763	LINC01119	long intergenic non-protein coding RNA 1119 [Source:HGNC Symbol;Acc:HGNC:49262]
ENSG00000133138	0,57473321	2,16339578	10,2938797	0,01031874	0,13399692	TBC1D8B	TBC1 domain family member 8B [Source:HGNC Symbol;Acc:HGNC:24715]
ENSG00000160588	0,60772888	3,80721046	10,2880542	0,01033401	0,13407596	MPZL3	myelin protein zero like 3 [Source:HGNC Symbol;Acc:HGNC:27279]
ENSG00000135052	-2,0563644	-0,7914625	10,2724131	0,01037513	0,13438415	GOLM1	golgi membrane protein 1 [Source:HGNC Symbol;Acc:HGNC:15451]
ENSG00000234589	-0,5702345	2,95424033	10,2720214	0,01037616	0,13438415	AC125807.1	ribosomal protein L13a (RPL13A) pseudogene
ENSG00000115042	-0,4788322	5,02533373	10,2626456	0,01040091	0,13455237	FAHD2A	fumarylacetoacetate hydrolase domain containing 2A [Source:HGNC Symbol;Acc:HGNC:24252]
ENSG00000213551	0,45856695	6,80338139	10,2601263	0,01040757	0,13455237	DNAJC9	DnaJ heat shock protein family (Hsp40) member C9 [Source:HGNC Symbol;Acc:HGNC:19123]
ENSG00000064651	-0,5651896	2,61646258	10,2486253	0,01043805	0,13462628	SLC12A2	solute carrier family 12 member 2 [Source:HGNC Symbol;Acc:HGNC:10911]
ENSG00000083799	0,52234338	2,85524334	10,2535839	0,01042489	0,13462628	CYLD	CYLD lysine 63 deubiquitinase [Source:HGNC Symbol;Acc:HGNC:2584]
ENSG00000102554	-1,2425659	0,44040104	10,2475396	0,01044093	0,13462628	KLF5	Kruppel like factor 5 [Source:HGNC Symbol;Acc:HGNC:6349]
ENSG00000112320	0,57899357	2,62188086	10,237517	0,01046759	0,13482013	SOBP	sine oculis binding protein homolog [Source:HGNC Symbol;Acc:HGNC:29256]
ENSG00000126259	-1,3101737	0,06959077	10,2344727	0,01047571	0,13482013	KIRREL2	kirre like nephrin family adhesion molecule 2 [Source:HGNC Symbol;Acc:HGNC:18816]
ENSG00000152990	0,4873578	6,17442207	10,2314945	0,01048365	0,13482013	ADGRA3	adhesion G protein-coupled receptor A3 [Source:HGNC Symbol;Acc:HGNC:13839]
ENSG00000161544	-0,7727607	2,46354696	10,2262991	0,01049753	0,13487988	CYGB	cytoglobin [Source:HGNC Symbol;Acc:HGNC:16505]
ENSG00000179195	0,47963069	4,0068096	10,2177467	0,01052043	0,13505527	ZNF664	zinc finger protein 664 [Source:HGNC Symbol;Acc:HGNC:25406]
ENSG00000176593	0,81287147	0,95036666	10,2049777	0,01055472	0,1353766	AC008969.1	novel transcript, antisense to ZNF606
ENSG00000090054	-0,4745098	6,10099928	10,1860503	0,01060581	0,13579346	SPTLC1	serine palmitoyltransferase long chain base subunit 1 [Source:HGNC Symbol;Acc:HGNC:11277]
ENSG00000278129	1,08631038	0,92899653	10,1891699	0,01059737	0,13579346	ZNF8	zinc finger protein 8 [Source:HGNC Symbol;Acc:HGNC:13154]
ENSG00000142409	-0,4680832	5,10746355	10,175958	0,01063318	0,13602463	ZNF787	zinc finger protein 787 [Source:HGNC Symbol;Acc:HGNC:26998]
ENSG00000114745	0,51916943	4,55023465	10,1522501	0,01069781	0,13661212	GORASP1	golgi reassembly stacking protein 1 [Source:HGNC Symbol;Acc:HGNC:16769]

ENSG00000184661	0,50842427	3,83290923	10,1530867	0,01069552	0,13661212	CDCA2	cell division cycle associated 2 [Source:HGNC Symbol;Acc:HGNC:14623]
ENSG00000130939	0,45608052	5,31861473	10,1443199	0,01071953	0,13665065	UBE4B	ubiquitination factor E4B [Source:HGNC Symbol;Acc:HGNC:12500]
ENSG00000244705	-0,5317061	2,55900735	10,1443808	0,01071936	0,13665065	AC020658.1	ribosomal protein, large, P0 (RPLP0) pseudogene
ENSG00000197905	-0,443843	6,4409881	10,1396755	0,01073228	0,13669388	TEAD4	TEA domain transcription factor 4 [Source:HGNC Symbol;Acc:HGNC:11717]
ENSG00000003056	0,53303761	7,17907077	10,0956983	0,01085392	0,13751609	M6PR	mannose-6-phosphate receptor, cation dependent [Source:HGNC Symbol;Acc:HGNC:6752]
ENSG00000048405	-0,6438264	1,85119524	10,1120254	0,01080856	0,13751609	ZNF800	zinc finger protein 800 [Source:HGNC Symbol;Acc:HGNC:27267]
ENSG00000109046	0,46736188	5,40757737	10,08932	0,0108717	0,13751609	WSB1	WD repeat and SOCS box containing 1 [Source:HGNC Symbol;Acc:HGNC:19221]
ENSG00000162910	-0,5826805	5,20300151	10,0925379	0,01086273	0,13751609	MRPL55	mitochondrial ribosomal protein L55 [Source:HGNC Symbol;Acc:HGNC:16686]
ENSG00000175309	-0,6701615	2,68939088	10,0891633	0,01087214	0,13751609	PHYKPL	5-phosphohydroxy-L-lysine phospho-lyase [Source:HGNC Symbol;Acc:HGNC:28249]
ENSG00000175416	-0,4913363	5,25414129	10,093891	0,01085895	0,13751609	CLTB	clathrin light chain B [Source:HGNC Symbol;Acc:HGNC:2091]
ENSG00000240682	0,52523698	3,26797358	10,1009433	0,01083932	0,13751609	ISY1	ISY1 splicing factor homolog [Source:HGNC Symbol;Acc:HGNC:29201]
ENSG00000266709	0,45736225	5,67591385	10,1063268	0,01082437	0,13751609	AC005224.3	novel transcript
ENSG00000064309	0,64061218	2,33025656	10,0811537	0,01089452	0,13767998	CDON	cell adhesion associated, oncogene regulated [Source:HGNC Symbol;Acc:HGNC:17104]
ENSG00000108506	0,63608337	4,19708233	10,0588282	0,01095721	0,13774861	INTS2	integrator complex subunit 2 [Source:HGNC Symbol;Acc:HGNC:29241]
ENSG00000109536	0,46869084	5,36815747	10,0510588	0,01097912	0,13774861	FRG1	FSHD region gene 1 [Source:HGNC Symbol;Acc:HGNC:3954]
ENSG00000111653	0,62035627	3,07796071	10,0577692	0,01096019	0,13774861	ING4	inhibitor of growth family member 4 [Source:HGNC Symbol;Acc:HGNC:19423]
ENSG00000121579	0,52538206	5,48634087	10,0398933	0,01101072	0,13774861	NAA50	N-alpha-acetyltransferase 50, NatE catalytic subunit [Source:HGNC Symbol;Acc:HGNC:29533]
ENSG00000121879	-0,5035446	2,95601301	10,0401267	0,01101005	0,13774861	PIK3CA	phosphatidylinositol-4,5-bisphosphate 3-kinase catalytic subunit alpha [Source:HGNC Symbol;Acc:HGNC:8975]
ENSG00000143036	0,82754582	2,56785548	10,0390524	0,0110131	0,13774861	SLC44A3	solute carrier family 44 member 3 [Source:HGNC Symbol;Acc:HGNC:28689]
ENSG00000154222	-0,4718404	4,92300642	10,0577787	0,01096016	0,13774861	CC2D1B	coiled-coil and C2 domain containing 1B [Source:HGNC Symbol;Acc:HGNC:29386]
ENSG00000157617	0,45780877	4,39127343	10,0478073	0,01098831	0,13774861	C2CD2	C2 calcium dependent domain containing 2 [Source:HGNC Symbol;Acc:HGNC:1266]
ENSG00000162512	0,84477575	0,51538876	10,0510081	0,01097927	0,13774861	SDC3	syndecan 3 [Source:HGNC Symbol;Acc:HGNC:10660]
ENSG00000174842	0,59876415	2,87103427	10,0400774	0,01101019	0,13774861	GLMN	glomulin, FKBP associated protein [Source:HGNC Symbol;Acc:HGNC:14373]
ENSG00000183496	1,69116094	-1,0422304	10,0737262	0,01091533	0,13774861	MEX3B	mex-3 RNA binding family member B [Source:HGNC Symbol;Acc:HGNC:25297]
ENSG00000214954	-1,5078947	-1,2734646	10,064468	0,01094133	0,13774861	LRRC69	leucine rich repeat containing 69 [Source:HGNC Symbol;Acc:HGNC:34303]
ENSG00000162585	-0,5099041	4,81697248	10,0350646	0,01102441	0,13777217	FAAP20	FA core complex associated protein 20 [Source:HGNC Symbol;Acc:HGNC:26428]
ENSG00000143179	-0,4104669	6,397252	10,0295064	0,01104021	0,13785161	UCK2	uridine-cytidine kinase 2 [Source:HGNC Symbol;Acc:HGNC:12562]
ENSG00000141127	-0,5249588	4,9036121	10,0202812	0,01106648	0,13806165	PRPSAP2	phosphoribosyl pyrophosphate synthetase associated protein 2 [Source:HGNC Symbol;Acc:HGNC:9467]
ENSG00000091640	-0,4145371	6,1163726	10,0003615	0,01112347	0,13806401	SPAG7	sperm associated antigen 7 [Source:HGNC Symbol;Acc:HGNC:11216]
ENSG00000104518	-0,4904646	4,62319509	10,008302	0,01110071	0,13806401	GSDMD	gasdermin D [Source:HGNC Symbol;Acc:HGNC:25697]
ENSG00000113249	-1,0914368	0,05626631	10,0033663	0,01111485	0,13806401	HAVCR1	hepatitis A virus cellular receptor 1 [Source:HGNC Symbol;Acc:HGNC:17866]
ENSG00000135709	-0,7316226	1,03309226	10,002859	0,01111631	0,13806401	KIAA0513	KIAA0513 [Source:HGNC Symbol;Acc:HGNC:29058]
ENSG00000135899	0,50938972	3,78560472	9,9971053	0,01113282	0,13806401	SP110	SP110 nuclear body protein [Source:HGNC Symbol;Acc:HGNC:5401]
ENSG00000159199	-0,5311964	7,62898331	10,0023845	0,01111767	0,13806401	ATP5MC1	ATP synthase membrane subunit c locus 1 [Source:HGNC Symbol;Acc:HGNC:841]
ENSG00000173848	0,48871439	6,07558757	10,0062068	0,01110671	0,13806401	NET1	neuroepithelial cell transforming 1 [Source:HGNC Symbol;Acc:HGNC:14592]
ENSG00000109519	-0,4356849	7,07184344	9,98970774	0,0111541	0,13821057	GRPEL1	GrpE like 1, mitochondrial [Source:HGNC Symbol;Acc:HGNC:19696]
ENSG00000154839	0,50393341	3,7732812	9,98454098	0,01116899	0,13821836	SKA1	spindle and kinetochore associated complex subunit 1 [Source:HGNC Symbol;Acc:HGNC:28109]
ENSG00000198933	0,57972847	2,88947006	9,9829259	0,01117365	0,13821836	TBKBP1	TBK1 binding protein 1 [Source:HGNC Symbol;Acc:HGNC:30140]
ENSG00000163788	0,49921203	3,00067205	9,97214261	0,01120483	0,13848672	SNRK	SNF related kinase [Source:HGNC Symbol;Acc:HGNC:30598]
ENSG00000071859	-0,5245521	5,86352057	9,96251048	0,01123276	0,13859748	FAM50A	family with sequence similarity 50 member A [Source:HGNC Symbol;Acc:HGNC:18786]
ENSG00000228716	-0,4326176	5,45110145	9,96264277	0,01123238	0,13859748	DHFR	dihydrofolate reductase [Source:HGNC Symbol;Acc:HGNC:2861]
ENSG00000153560	0,52284237	5,00786943	9,95159283	0,01126453	0,13887216	UBP1	upstream binding protein 1 [Source:HGNC Symbol;Acc:HGNC:12507]
ENSG00000162551	1,41616024	-0,3314096	9,94515434	0,01128332	0,13898647	ALPL	alkaline phosphatase, biomineralization associated [Source:HGNC Symbol;Acc:HGNC:438]
ENSG00000163874	0,49896762	3,50000445	9,93956558	0,01129965	0,13907046	ZC3H12A	zinc finger CCCH-type containing 12A [Source:HGNC Symbol;Acc:HGNC:26259]

ENSG00000172366	-0,5136856	5,07812565	9,93291544	0,01131913	0,13919293	MCRIP2	MAPK regulated corepressor interacting protein 2 [Source:HGNC Symbol;Acc:HGNC:14142]
ENSG00000177853	0,50771534	3,77904912	9,91309272	0,01137744	0,13979225	ZNF518A	zinc finger protein 518A [Source:HGNC Symbol;Acc:HGNC:29009]
ENSG00000058063	0,51824704	3,12077483	9,895905	0,01142829	0,13994482	ATP11B	ATPase phospholipid transporting 11B (putative) [Source:HGNC Symbol;Acc:HGNC:13553]
ENSG00000090006	2,1133359	-1,3951753	9,90474856	0,01140209	0,13994482	LTBP4	latent transforming growth factor beta binding protein 4 [Source:HGNC Symbol;Acc:HGNC:6717]
ENSG00000100979	-0,6913473	5,5802845	9,90169969	0,01141112	0,13994482	PLTP	phospholipid transfer protein [Source:HGNC Symbol;Acc:HGNC:9093]
ENSG00000124787	0,46777662	4,94698946	9,88571334	0,01145858	0,13994482	RPP40	ribonuclease P/MRP subunit p40 [Source:HGNC Symbol;Acc:HGNC:20992]
ENSG00000131558	-0,6622231	3,92753161	9,88372129	0,01146451	0,13994482	EXOC4	exocyst complex component 4 [Source:HGNC Symbol;Acc:HGNC:30389]
ENSG00000136153	0,50857281	4,25812043	9,88378298	0,01146432	0,13994482	LMO7	LIM domain 7 [Source:HGNC Symbol;Acc:HGNC:6646]
ENSG00000160131	-0,4816376	4,12606136	9,88305608	0,01146649	0,13994482	VMA21	vacuolar ATPase assembly factor VMA21 [Source:HGNC Symbol;Acc:HGNC:22082]
ENSG00000183578	0,61495202	2,60615114	9,89005345	0,01144567	0,13994482	TNFAIP8L3	TNF alpha induced protein 8 like 3 [Source:HGNC Symbol;Acc:HGNC:20620]
ENSG00000114446	0,5047013	3,89599176	9,87852796	0,01147999	0,13999262	IFT57	intraflagellar transport 57 [Source:HGNC Symbol;Acc:HGNC:17367]
ENSG00000160818	-0,6039053	5,32881802	9,87414922	0,01149306	0,14003513	GPATCH4	G-patch domain containing 4 [Source:HGNC Symbol;Acc:HGNC:25982]
ENSG00000123353	-0,4363276	5,5528462	9,86732255	0,01151348	0,14005028	ORMDL2	ORMDL sphingolipid biosynthesis regulator 2 [Source:HGNC Symbol;Acc:HGNC:16037]
ENSG00000151881	0,60686531	2,22561872	9,87051979	0,01150391	0,14005028	TMEM267	transmembrane protein 267 [Source:HGNC Symbol;Acc:HGNC:26139]
ENSG00000157601	0,7851958	1,86930689	9,84613349	0,01157713	0,14070741	MX1	MX dynamin like GTPase 1 [Source:HGNC Symbol;Acc:HGNC:7532]
ENSG00000162959	-1,0643742	0,46418285	9,8426885	0,01158752	0,14071662	MEMO1	mediator of cell motility 1 [Source:HGNC Symbol;Acc:HGNC:14014]
ENSG00000013583	-0,6062602	5,02502638	9,83794057	0,01160186	0,14077372	HEBP1	heme binding protein 1 [Source:HGNC Symbol;Acc:HGNC:17176]
ENSG00000198909	0,48344078	4,38163101	9,83240735	0,0116186	0,14085981	MAP3K3	mitogen-activated protein kinase kinase kinase 3 [Source:HGNC Symbol;Acc:HGNC:6855]
ENSG00000111145	-0,6076256	2,07973168	9,8129445	0,0116777	0,14142244	ELK3	ETS transcription factor ELK3 [Source:HGNC Symbol;Acc:HGNC:3325]
ENSG00000160216	0,41377297	6,04464344	9,81075853	0,01168436	0,14142244	AGPAT3	1-acylglycerol-3-phosphate O-acyltransferase 3 [Source:HGNC Symbol;Acc:HGNC:326]
ENSG00000126561	0,66421553	1,8177509	9,80546247	0,01170052	0,14150078	STAT5A	signal transducer and activator of transcription 5A [Source:HGNC Symbol;Acc:HGNC:11366]
ENSG00000136840	-0,5569856	4,91849094	9,80094621	0,01171432	0,14155051	ST6GALNAC4	ST6 N-acetylgalactosaminide alpha-2,6-sialyltransferase 4 [Source:HGNC Symbol;Acc:HGNC:17846]
ENSG00000253710	0,50719194	3,13886264	9,7856542	0,0117612	0,14188227	ALG11	ALG11 alpha-1,2-mannosyltransferase [Source:HGNC Symbol;Acc:HGNC:32456]
ENSG00000268858	-0,4904283	3,37586067	9,78818212	0,01175344	0,14188227	AL118506.1	uncharacterized LOC112268269 [Source:NCBI gene (formerly Entrezgene);Acc:112268269]
ENSG00000117625	0,52243077	3,09511316	9,76268694	0,01183204	0,14226695	RCOR3	REST corepressor 3 [Source:HGNC Symbol;Acc:HGNC:25594]
ENSG00000137413	0,48362879	4,08308335	9,76605558	0,01182162	0,14226695	TAF8	TATA-box binding protein associated factor 8 [Source:HGNC Symbol;Acc:HGNC:17300]
ENSG00000160570	0,49796048	4,24797876	9,76282526	0,01183162	0,14226695	DEDD2	death effector domain containing 2 [Source:HGNC Symbol;Acc:HGNC:24450]
ENSG00000161647	-0,9071028	0,77606522	9,76923257	0,0118118	0,14226695	MPP3	membrane palmitoylated protein 3 [Source:HGNC Symbol;Acc:HGNC:7221]
ENSG00000149260	0,87158572	1,39089015	9,74698779	0,01188077	0,14273533	CAPN5	calpain 5 [Source:HGNC Symbol;Acc:HGNC:1482]
ENSG00000000460	0,52437472	3,83667467	9,71694675	0,01197469	0,14278059	C1orf112	chromosome 1 open reading frame 112 [Source:HGNC Symbol;Acc:HGNC:25565]
ENSG00000081913	0,46651661	4,31981117	9,74047978	0,01190104	0,14278059	PHLPP1	PH domain and leucine rich repeat protein phosphatase 1 [Source:HGNC Symbol;Acc:HGNC:20610]
ENSG00000110315	-0,5712348	2,89921771	9,72346757	0,01195423	0,14278059	RNF141	ring finger protein 141 [Source:HGNC Symbol;Acc:HGNC:21159]
ENSG00000110987	0,48247249	3,72537109	9,7149867	0,01198085	0,14278059	BCL7A	BAF chromatin remodeling complex subunit BCL7A [Source:HGNC Symbol;Acc:HGNC:1004]
ENSG00000162076	-0,549614	3,1868134	9,73859442	0,01190692	0,14278059	FLYWCH2	FLYWCH family member 2 [Source:HGNC Symbol;Acc:HGNC:25178]
ENSG00000177302	0,45751844	5,4112907	9,7183517	0,01197028	0,14278059	TOP3A	DNA topoisomerase III alpha [Source:HGNC Symbol;Acc:HGNC:11992]
ENSG00000183814	0,54715025	2,75783171	9,72336033	0,01195456	0,14278059	LIN9	lin-9 DREAM MuvB core complex component [Source:HGNC Symbol;Acc:HGNC:30830]
ENSG00000187741	0,41489903	5,67923096	9,71850453	0,0119698	0,14278059	FANCA	FA complementation group A [Source:HGNC Symbol;Acc:HGNC:3582]
ENSG00000197273	1,25760663	-0,1486439	9,72838697	0,01193882	0,14278059	GUCA2A	guanylate cyclase activator 2A [Source:HGNC Symbol;Acc:HGNC:4682]
ENSG00000288012	-0,8996268	0,28416816	9,71453548	0,01198227	0,14278059	AP003307.1	novel transcript, antisense to CUL5
ENSG00000153774	0,48625167	5,66208697	9,70961774	0,01199775	0,14284846	CFDP1	craniofacial development protein 1 [Source:HGNC Symbol;Acc:HGNC:1873]
ENSG00000176485	-0,7451958	1,51834879	9,70480625	0,01201291	0,14291253	PLAAT3	phospholipase A and acyltransferase 3 [Source:HGNC Symbol;Acc:HGNC:17825]
ENSG00000119703	0,64728278	2,04796366	9,6956153	0,01204194	0,14314133	ZC2HC1C	zinc finger C2HC-type containing 1C [Source:HGNC Symbol;Acc:HGNC:20354]
ENSG00000272449	-1,3925902	-1,1960993	9,6800759	0,01209122	0,14361025	AL139246.5	novel transcript
ENSG00000152359	0,67983502	2,4958255	9,67457125	0,01210873	0,14370145	POC5	POC5 centriolar protein [Source:HGNC Symbol;Acc:HGNC:26658]

ENSG00000104524	-0,5958126	3,17436435	9,65338404	0,01217644	0,14380408	PYCR3	pyrroline-5-carboxylate reductase 3 [Source:HGNC Symbol;Acc:HGNC:25846]
ENSG00000116604	0,61876414	3,05385137	9,66406546	0,01214225	0,14380408	MEF2D	myocyte enhancer factor 2D [Source:HGNC Symbol;Acc:HGNC:6997]
ENSG00000154127	-0,510928	4,869235	9,65352574	0,01217599	0,14380408	UBASH3B	ubiquitin associated and SH3 domain containing B [Source:HGNC Symbol;Acc:HGNC:29884]
ENSG00000169607	-0,5737674	3,59875245	9,65468356	0,01217228	0,14380408	CKAP2L	cytoskeleton associated protein 2 like [Source:HGNC Symbol;Acc:HGNC:26877]
ENSG00000170145	0,56694757	3,94581433	9,65866896	0,01215951	0,14380408	SIK2	salt inducible kinase 2 [Source:HGNC Symbol;Acc:HGNC:21680]
ENSG00000243829	-0,5532065	2,12139407	9,65750491	0,01216324	0,14380408	AC011495.1	ribosomal protein S9 (RPS9) pseudogene
ENSG00000168477	-1,1882925	-0,0528742	9,63914705	0,0122222	0,14422789	TNXB	tenascin XB [Source:HGNC Symbol;Acc:HGNC:11976]
ENSG00000008517	-0,4055452	7,46865302	9,62253075	0,01227587	0,14429075	IL32	interleukin 32 [Source:HGNC Symbol;Acc:HGNC:16830]
ENSG00000070540	-0,4313677	5,42683136	9,62038286	0,01228283	0,14429075	WIPI1	WD repeat domain, phosphoinositide interacting 1 [Source:HGNC Symbol;Acc:HGNC:25471]
ENSG00000122068	-0,4948803	4,3558153	9,62045099	0,01228261	0,14429075	FYTTD1	forty-two-three domain containing 1 [Source:HGNC Symbol;Acc:HGNC:25407]
ENSG00000160172	-0,6422796	1,37844768	9,61946315	0,01228581	0,14429075	FAM86C2P	family with sequence similarity 86 member C2, pseudogene [Source:HGNC Symbol;Acc:HGNC:42392]
ENSG00000181524	-0,5003844	2,71857679	9,61916218	0,01228679	0,14429075	RPL24P4	RPL24 pseudogene 4 [Source:HGNC Symbol;Acc:HGNC:21371]
ENSG00000272831	-0,9799344	0,65043419	9,63287985	0,01224241	0,14429075	AC027644.3	novel transcript, antisense to RABGEF1
ENSG00000137312	-0,4300173	7,24582064	9,61470505	0,01230125	0,14434456	FLOT1	flotillin 1 [Source:HGNC Symbol;Acc:HGNC:3757]
ENSG00000237172	0,55786462	1,95023347	9,61106035	0,01231309	0,14436755	B3GNT9	UDP-GlcNAc:betaGal beta-1,3-N-acetylglucosaminyltransferase 9 [Source:HGNC Symbol;Acc:HGNC:28714]
ENSG00000128298	-0,5428481	3,7346725	9,58024588	0,01241377	0,1444728	BAIAP2L2	BAR/IMD domain containing adaptor protein 2 like 2 [Source:HGNC Symbol;Acc:HGNC:26203]
ENSG00000138152	-0,7067399	2,32265371	9,57955014	0,01241605	0,1444728	BTBD16	BTB domain containing 16 [Source:HGNC Symbol;Acc:HGNC:26340]
ENSG00000144228	0,55161863	3,56880541	9,5789101	0,01241815	0,1444728	SPOPL	speckle type BTB/POZ protein like [Source:HGNC Symbol;Acc:HGNC:27934]
ENSG00000164649	0,94409599	1,72398225	9,59157364	0,01237664	0,1444728	CDCA7L	cell division cycle associated 7 like [Source:HGNC Symbol;Acc:HGNC:30777]
ENSG00000166167	0,4773511	4,00726088	9,58279219	0,01240541	0,1444728	BTRC	beta-transducin repeat containing E3 ubiquitin protein ligase [Source:HGNC Symbol;Acc:HGNC:1144]
ENSG00000167637	0,67822486	1,34024559	9,580535	0,01241282	0,1444728	ZNF283	zinc finger protein 283 [Source:HGNC Symbol;Acc:HGNC:13077]
ENSG00000170100	0,59598676	2,56625161	9,59711051	0,01235854	0,1444728	ZNF778	zinc finger protein 778 [Source:HGNC Symbol;Acc:HGNC:26479]
ENSG00000183648	-0,5938068	5,32703381	9,57999637	0,01241459	0,1444728	NDUFB1	NADH:ubiquinone oxidoreductase subunit B1 [Source:HGNC Symbol;Acc:HGNC:7695]
ENSG00000247595	-0,9396796	0,82897011	9,5952903	0,01236449	0,1444728	SPTY2D1OS	SPTY2D1 opposite strand [Source:HGNC Symbol;Acc:HGNC:44122]
ENSG00000282164	0,71106328	1,77312233	9,57805523	0,01242096	0,1444728	PEG13	paternally expressed 13 [Source:HGNC Symbol;Acc:HGNC:19969]
ENSG00000108846	-0,509246	5,29482517	9,56683148	0,01245791	0,14467217	ABCC3	ATP binding cassette subfamily C member 3 [Source:HGNC Symbol;Acc:HGNC:54]
ENSG00000166971	0,67880293	4,76331148	9,56803762	0,01245393	0,14467217	AKTIP	AKT interacting protein [Source:HGNC Symbol;Acc:HGNC:16710]
ENSG00000164190	0,4887078	4,40654881	9,55913997	0,01248331	0,14473699	NIPBL	NIPBL cohesin loading factor [Source:HGNC Symbol;Acc:HGNC:28862]
ENSG00000185721	0,4146892	7,34610016	9,5603578	0,01247928	0,14473699	DRG1	developmentally regulated GTP binding protein 1 [Source:HGNC Symbol;Acc:HGNC:3029]
ENSG00000109445	-0,4602389	5,07403842	9,55279803	0,01250429	0,14476791	ZNF330	zinc finger protein 330 [Source:HGNC Symbol;Acc:HGNC:15462]
ENSG00000198863	0,51701537	4,56712244	9,55234605	0,01250579	0,14476791	RUNDC1	RUN domain containing 1 [Source:HGNC Symbol;Acc:HGNC:25418]
ENSG00000076053	0,47093107	3,6895076	9,54568253	0,01252789	0,14490894	RBM7	RNA binding motif protein 7 [Source:HGNC Symbol;Acc:HGNC:9904]
ENSG00000171443	-0,5832057	2,13867022	9,54161394	0,01254141	0,14495055	ZNF524	zinc finger protein 524 [Source:HGNC Symbol;Acc:HGNC:28322]
ENSG00000115271	0,48062747	4,13372833	9,52861094	0,01258474	0,14510689	GCA	grancalcin [Source:HGNC Symbol;Acc:HGNC:15990]
ENSG00000167618	-1,1460503	-0,5191501	9,53311771	0,0125697	0,14510689	LAIR2	leukocyte associated immunoglobulin like receptor 2 [Source:HGNC Symbol;Acc:HGNC:6478]
ENSG00000214425	0,96522325	0,39983304	9,5292655	0,01258255	0,14510689	LRRC37A4P	leucine rich repeat containing 37 member A4, pseudogene [Source:HGNC Symbol;Acc:HGNC:25479]
ENSG00000159202	0,42694446	6,92992119	9,52474315	0,01259766	0,14514134	UBE2Z	ubiquitin conjugating enzyme E2 Z [Source:HGNC Symbol;Acc:HGNC:25847]
ENSG00000156398	-0,5732124	4,17028601	9,50843431	0,01265233	0,14565633	SFXN2	sideroflexin 2 [Source:HGNC Symbol;Acc:HGNC:16086]
ENSG00000105520	0,69689818	1,62717355	9,50510563	0,01266352	0,1456704	PLPPR2	phospholipid phosphatase related 2 [Source:HGNC Symbol;Acc:HGNC:29566]
ENSG00000101294	-0,4067821	8,85324119	9,46716133	0,01279199	0,1466497	HM13	histocompatibility minor 13 [Source:HGNC Symbol;Acc:HGNC:16435]
ENSG00000102763	0,63111513	4,14302925	9,46537559	0,01279807	0,1466497	VWA8	von Willebrand factor A domain containing 8 [Source:HGNC Symbol;Acc:HGNC:29071]
ENSG00000128833	0,52369719	3,93128814	9,47134154	0,01277776	0,1466497	MYO5C	myosin VC [Source:HGNC Symbol;Acc:HGNC:7604]
ENSG00000165175	-0,5193285	4,96901378	9,4653662	0,0127981	0,1466497	MID1IP1	MID1 interacting protein 1 [Source:HGNC Symbol;Acc:HGNC:20715]
ENSG00000167705	-0,5537415	2,90025913	9,46514772	0,01279885	0,1466497	RILP	Rab interacting lysosomal protein [Source:HGNC Symbol;Acc:HGNC:30266]

ENSG00000189171	-0,5342774	3,72875799	9,44702055	0,01286082	0,14724434	S100A13	S100 calcium binding protein A13 [Source:HGNC Symbol;Acc:HGNC:10490]
ENSG00000178896	-0,4827853	4,99391563	9,43780961	0,01289246	0,14743612	EXOSC4	exosome component 4 [Source:HGNC Symbol;Acc:HGNC:18189]
ENSG00000179604	0,48043109	5,46507502	9,43543764	0,01290062	0,14743612	CDC42EP4	CDC42 effector protein 4 [Source:HGNC Symbol;Acc:HGNC:17147]
ENSG00000198898	0,46231717	6,19931797	9,43333698	0,01290785	0,14743612	CAPZA2	capping actin protein of muscle Z-line subunit alpha 2 [Source:HGNC Symbol;Acc:HGNC:1490]
ENSG00000230082	-0,6857025	1,72424744	9,42700805	0,01292967	0,14757	PRRT3-AS1	PRRT3 antisense RNA 1 [Source:HGNC Symbol;Acc:HGNC:41151]
ENSG00000120685	0,73461702	3,08721509	9,42160056	0,01294835	0,14766785	PROSER1	proline and serine rich 1 [Source:HGNC Symbol;Acc:HGNC:20291]
ENSG00000115947	0,43059232	5,2723322	9,39994021	0,01302352	0,14834403	ORC4	origin recognition complex subunit 4 [Source:HGNC Symbol;Acc:HGNC:8490]
ENSG00000158571	-0,8537731	0,42324392	9,39575261	0,01303811	0,14834403	PFKFB1	6-phosphofructo-2-kinase/fructose-2,6-biphosphatase 1 [Source:HGNC Symbol;Acc:HGNC:8872]
ENSG00000240225	0,75481023	2,25608677	9,39732899	0,01303261	0,14834403	ZNF542P	zinc finger protein 542, pseudogene [Source:HGNC Symbol;Acc:HGNC:25393]
ENSG00000164930	0,79238054	3,016564	9,38854612	0,01306327	0,14844366	FZD6	frizzled class receptor 6 [Source:HGNC Symbol;Acc:HGNC:4044]
ENSG00000183665	0,58387854	1,99313765	9,38452078	0,01307735	0,14844366	TRMT12	tRNA methyltransferase 12 homolog [Source:HGNC Symbol;Acc:HGNC:26091]
ENSG00000226416	-1,0145166	-0,0353621	9,38452995	0,01307732	0,14844366	MRPL23-AS1	MRPL23 antisense RNA 1 [Source:HGNC Symbol;Acc:HGNC:42812]
ENSG00000015520	-0,7772159	5,25863922	9,36042676	0,01316201	0,14852708	NPC1L1	NPC1 like intracellular cholesterol transporter 1 [Source:HGNC Symbol;Acc:HGNC:7898]
ENSG00000133606	0,46371926	5,32268073	9,37687802	0,01310413	0,14852708	MKRN1	makorin ring finger protein 1 [Source:HGNC Symbol;Acc:HGNC:7112]
ENSG00000133816	-0,8693392	1,32328678	9,36966092	0,01312949	0,14852708	MICAL2	microtubule associated monooxygenase, calponin and LIM domain containing 2 [Source:HGNC Symbol;Acc:HGNC
ENSG00000137040	-0,5343859	3,44081543	9,36601106	0,01314233	0,14852708	RANBP6	RAN binding protein 6 [Source:HGNC Symbol;Acc:HGNC:9851]
ENSG00000148362	-0,5647765	3,99935911	9,36187426	0,01315691	0,14852708	PAXX	PAXX non-homologous end joining factor [Source:HGNC Symbol;Acc:HGNC:27849]
ENSG00000165029	0,80457251	4,90941765	9,35591235	0,01317795	0,14852708	ABCA1	ATP binding cassette subfamily A member 1 [Source:HGNC Symbol;Acc:HGNC:29]
ENSG00000170899	-0,606637	4,36507898	9,35353149	0,01318637	0,14852708	GSTA4	glutathione S-transferase alpha 4 [Source:HGNC Symbol;Acc:HGNC:4629]
ENSG00000189334	0,86874365	0,85509227	9,37088861	0,01312517	0,14852708	S100A14	S100 calcium binding protein A14 [Source:HGNC Symbol;Acc:HGNC:18901]
ENSG00000198039	0,66864299	1,61391967	9,35838517	0,01316922	0,14852708	ZNF273	zinc finger protein 273 [Source:HGNC Symbol;Acc:HGNC:13067]
ENSG00000198707	-0,7655196	2,334557	9,37336352	0,01311647	0,14852708	CEP290	centrosomal protein 290 [Source:HGNC Symbol;Acc:HGNC:29021]
ENSG00000100814	0,45234774	6,22883592	9,34648241	0,01321132	0,1486935	CCNB1IP1	cyclin B1 interacting protein 1 [Source:HGNC Symbol;Acc:HGNC:19437]
ENSG00000008441	0,74959571	2,71500757	9,31643231	0,01331835	0,14966749	NFIX	nuclear factor I X [Source:HGNC Symbol;Acc:HGNC:7788]
ENSG00000175592	-0,504049	4,1673465	9,31778939	0,01331349	0,14966749	FOSL1	FOS like 1, AP-1 transcription factor subunit [Source:HGNC Symbol;Acc:HGNC:13718]
ENSG00000135002	-0,5510094	3,99763863	9,31143021	0,01333627	0,14975366	RFK	riboflavin kinase [Source:HGNC Symbol;Acc:HGNC:30324]
ENSG00000064199	-0,6830207	2,29375272	9,30552017	0,01335748	0,14987663	SPA17	sperm autoantigenic protein 17 [Source:HGNC Symbol;Acc:HGNC:11210]
ENSG00000157227	0,70328619	3,45261779	9,30073032	0,0133747	0,14995467	MMP14	matrix metallopeptidase 14 [Source:HGNC Symbol;Acc:HGNC:7160]
ENSG00000170271	0,63012786	1,56950293	9,28910081	0,01341662	0,15030934	FAXDC2	fatty acid hydroxylase domain containing 2 [Source:HGNC Symbol;Acc:HGNC:1334]
ENSG00000276107	0,88554953	1,90469263	9,27555719	0,01346564	0,15074298	AC037198.1	novel transcript, sense intronic to THBS1
ENSG00000163631	0,60820474	14,4824109	9,26413841	0,01350715	0,15109184	ALB	albumin [Source:HGNC Symbol;Acc:HGNC:399]
ENSG00000005884	-1,3654394	0,11376741	9,25980046	0,01352296	0,15115294	ITGA3	integrin subunit alpha 3 [Source:HGNC Symbol;Acc:HGNC:6139]
ENSG00000012232	-0,4538839	4,46566882	9,23346495	0,01361942	0,15115752	EXTL3	exostosin like glycosyltransferase 3 [Source:HGNC Symbol;Acc:HGNC:3518]
ENSG00000068784	-0,4920803	4,25421471	9,2441085	0,01358033	0,15115752	SRBD1	S1 RNA binding domain 1 [Source:HGNC Symbol;Acc:HGNC:25521]
ENSG00000106080	-0,3986796	5,71441847	9,24724033	0,01356886	0,15115752	FKBP14	FKBP prolyl isomerase 14 [Source:HGNC Symbol;Acc:HGNC:18625]
ENSG00000121390	-0,4785958	5,95998755	9,24538429	0,01357566	0,15115752	PSPC1	paraspeckle component 1 [Source:HGNC Symbol;Acc:HGNC:20320]
ENSG00000171311	-0,4473439	5,16276197	9,239344	0,01359781	0,15115752	EXOSC1	exosome component 1 [Source:HGNC Symbol;Acc:HGNC:17286]
ENSG00000180917	0,6276917	2,03056533	9,24249311	0,01358626	0,15115752	CMTR2	cap methyltransferase 2 [Source:HGNC Symbol;Acc:HGNC:25635]
ENSG00000185015	0,49145225	2,63906376	9,23345628	0,01361945	0,15115752	CA13	carbonic anhydrase 13 [Source:HGNC Symbol;Acc:HGNC:14914]
ENSG00000213500	-1,8446772	-1,9972633	9,23144978	0,01362684	0,15115752	LAP3P2	leucine aminopeptidase 3 pseudogene 2 [Source:HGNC Symbol;Acc:HGNC:42365]
ENSG00000227671	1,00135331	0,64893037	9,24264538	0,0135857	0,15115752	AL390728.4	zinc finger protein pseudogene
ENSG00000275004	0,87833358	0,76863646	9,25166896	0,01355265	0,15115752	ZNF280B	zinc finger protein 280B [Source:HGNC Symbol;Acc:HGNC:23022]
ENSG00000197782	0,64997674	1,89451798	9,22781568	0,01364022	0,15119121	ZNF780A	zinc finger protein 780A [Source:HGNC Symbol;Acc:HGNC:27603]
ENSG00000170965	1,12415088	-0,310898	9,21870402	0,01367386	0,15144911	PLAC1	placenta enriched 1 [Source:HGNC Symbol;Acc:HGNC:9044]

ENSG00000256940	-1,1635577	-0,590814	9,21360399	0,01369273	0,15154322	PPP1R14B-AS1	PPP1R14B antisense RNA 1 [Source:HGNC Symbol;Acc:HGNC:54233]
ENSG00000132589	0,42375099	6,85562626	9,20675421	0,01371812	0,15159458	FLOT2	flotillin 2 [Source:HGNC Symbol;Acc:HGNC:3758]
ENSG00000235890	-1,1003146	0,16560177	9,2091696	0,01370916	0,15159458	TSPEAR-AS1	TSPEAR antisense RNA 1 [Source:HGNC Symbol;Acc:HGNC:1271]
ENSG00000168724	-0,7275246	4,92079653	9,18285827	0,01380717	0,15246326	DNAJC21	DnaJ heat shock protein family (Hsp40) member C21 [Source:HGNC Symbol;Acc:HGNC:27030]
ENSG00000132670	-0,516778	6,52089502	9,1748293	0,01383724	0,15267999	PTPRA	protein tyrosine phosphatase receptor type A [Source:HGNC Symbol;Acc:HGNC:9664]
ENSG00000169288	-0,4296694	5,07145813	9,16277957	0,01388254	0,15306412	MRPL1	mitochondrial ribosomal protein L1 [Source:HGNC Symbol;Acc:HGNC:14275]
ENSG00000141013	-0,5457695	4,04645267	9,15765749	0,01390184	0,15307391	GAS8	growth arrest specific 8 [Source:HGNC Symbol;Acc:HGNC:4166]
ENSG00000142959	-0,6410485	1,47617628	9,1569852	0,01390438	0,15307391	BEST4	bestrophin 4 [Source:HGNC Symbol;Acc:HGNC:17106]
ENSG00000172345	-0,7429849	1,05836404	9,15394828	0,01391585	0,15308478	STARD5	StAR related lipid transfer domain containing 5 [Source:HGNC Symbol;Acc:HGNC:18065]
ENSG00000117266	-0,5609962	4,20642772	9,14523375	0,01394881	0,15321668	CDK18	cyclin dependent kinase 18 [Source:HGNC Symbol;Acc:HGNC:8751]
ENSG00000148334	-0,4054355	6,87914872	9,14718216	0,01394143	0,15321668	PTGES2	prostaglandin E synthase 2 [Source:HGNC Symbol;Acc:HGNC:17822]
ENSG00000164211	-0,5433949	4,28679662	9,1406776	0,01396608	0,15329115	STARD4	StAR related lipid transfer domain containing 4 [Source:HGNC Symbol;Acc:HGNC:18058]
ENSG00000104412	-0,4412483	4,78331554	9,12539289	0,01402422	0,15358312	EMC2	ER membrane protein complex subunit 2 [Source:HGNC Symbol;Acc:HGNC:28963]
ENSG00000136149	-0,4511924	3,81224206	9,12921356	0,01400966	0,15358312	RPL13AP25	ribosomal protein L13a pseudogene 25 [Source:HGNC Symbol;Acc:HGNC:36981]
ENSG00000165171	-1,2004031	0,15877396	9,12542312	0,01402411	0,15358312	METTL27	methyltransferase like 27 [Source:HGNC Symbol;Acc:HGNC:19068]
ENSG00000239665	1,43186102	-1,5070487	9,11574622	0,01406107	0,15387129	AL157392.3	novel transcript
ENSG00000066422	0,55952968	3,83265328	9,10392106	0,0141064	0,1539062	ZBTB11	zinc finger and BTB domain containing 11 [Source:HGNC Symbol;Acc:HGNC:16740]
ENSG00000196787	1,54508672	-1,5380735	9,10428291	0,01410501	0,1539062	H2AC13	H2A clustered histone 13 [Source:HGNC Symbol;Acc:HGNC:4725]
ENSG00000197977	0,47877458	6,37071584	9,10950434	0,01408498	0,1539062	ELOVL2	ELOVL fatty acid elongase 2 [Source:HGNC Symbol;Acc:HGNC:14416]
ENSG00000213523	-0,4054221	5,84996162	9,10753914	0,01409251	0,1539062	SRA1	steroid receptor RNA activator 1 [Source:HGNC Symbol;Acc:HGNC:11281]
ENSG00000023902	-0,4586453	4,08098463	9,07257423	0,01422743	0,15415721	PLEKHO1	pleckstrin homology domain containing O1 [Source:HGNC Symbol;Acc:HGNC:24310]
ENSG00000078319	-0,6558962	3,36268692	9,07142055	0,01423191	0,15415721	PMS2P1	PMS1 homolog 2, mismatch repair system component pseudogene 1 [Source:HGNC Symbol;Acc:HGNC:9123]
ENSG00000099949	-0,5977763	2,58734797	9,07928286	0,01420142	0,15415721	LZTR1	leucine zipper like transcription regulator 1 [Source:HGNC Symbol;Acc:HGNC:6742]
ENSG00000112640	0,4310722	6,58779582	9,07064389	0,01423493	0,15415721	PPP2R5D	protein phosphatase 2 regulatory subunit B'delta [Source:HGNC Symbol;Acc:HGNC:9312]
ENSG00000117593	0,46954342	6,0145668	9,07382091	0,0142226	0,15415721	DARS2	aspartyl-tRNA synthetase 2, mitochondrial [Source:HGNC Symbol;Acc:HGNC:25538]
ENSG00000163170	-0,5221894	6,10534988	9,07840765	0,01420481	0,15415721	BOLA3	bolA family member 3 [Source:HGNC Symbol;Acc:HGNC:24415]
ENSG00000214176	0,80361774	1,35044097	9,07981588	0,01419936	0,15415721	PLEKHM1P1	pleckstrin homology and RUN domain containing M1 pseudogene 1 [Source:HGNC Symbol;Acc:HGNC:35411]
ENSG00000240344	-0,544831	4,74601781	9,09056375	0,01415782	0,15415721	PPIL3	peptidylprolyl isomerase like 3 [Source:HGNC Symbol;Acc:HGNC:9262]
ENSG00000251369	0,55721665	2,47652527	9,08598404	0,0141755	0,15415721	ZNF550	zinc finger protein 550 [Source:HGNC Symbol;Acc:HGNC:28643]
ENSG00000258474	-0,8006323	1,44483138	9,09111425	0,0141557	0,15415721	LINC02313	long intergenic non-protein coding RNA 2313 [Source:HGNC Symbol;Acc:HGNC:53232]
ENSG00000158793	-0,4371306	4,99311818	9,06527428	0,0142558	0,1542689	NIT1	nitrilase 1 [Source:HGNC Symbol;Acc:HGNC:7828]
ENSG00000030066	0,5403053	6,10252252	9,05654542	0,01428982	0,1543692	NUP160	nucleoporin 160 [Source:HGNC Symbol;Acc:HGNC:18017]
ENSG00000130348	0,42376329	5,53383397	9,05476346	0,01429677	0,1543692	QRSL1	glutaminyl-tRNA amidotransferase subunit QRSL1 [Source:HGNC Symbol;Acc:HGNC:21020]
ENSG00000147853	0,456327	6,38144946	9,05821347	0,01428331	0,1543692	AK3	adenylate kinase 3 [Source:HGNC Symbol;Acc:HGNC:17376]
ENSG00000102572	-0,4754329	6,86972915	9,04911281	0,01431886	0,15449347	STK24	serine/threonine kinase 24 [Source:HGNC Symbol;Acc:HGNC:11403]
ENSG00000105379	-0,4091997	8,3290012	9,03476326	0,01437513	0,15466473	ETFB	electron transfer flavoprotein subunit beta [Source:HGNC Symbol;Acc:HGNC:3482]
ENSG00000162851	0,43348415	5,97780478	9,03426752	0,01437708	0,15466473	TFB2M	transcription factor B2, mitochondrial [Source:HGNC Symbol;Acc:HGNC:18559]
ENSG00000165949	-1,2287275	-0,5412827	9,04164938	0,01434809	0,15466473	IFI27	interferon alpha inducible protein 27 [Source:HGNC Symbol;Acc:HGNC:5397]
ENSG00000197822	0,55805295	4,68580649	9,03440611	0,01437653	0,15466473	OCLN	occludin [Source:HGNC Symbol;Acc:HGNC:8104]
ENSG00000039537	0,71454377	0,93284265	9,0268594	0,01440624	0,15470707	C6	complement C6 [Source:HGNC Symbol;Acc:HGNC:1339]
ENSG00000111012	0,82531881	0,67332307	9,02519947	0,01441278	0,15470707	CYP27B1	cytochrome P450 family 27 subfamily B member 1 [Source:HGNC Symbol;Acc:HGNC:2606]
ENSG00000125434	-0,7459683	0,8887246	9,02810595	0,01440133	0,15470707	SLC25A35	solute carrier family 25 member 35 [Source:HGNC Symbol;Acc:HGNC:31921]
ENSG00000010810	-0,580341	2,89743853	9,01877627	0,01443814	0,15486547	FYN	FYN proto-oncogene, Src family tyrosine kinase [Source:HGNC Symbol;Acc:HGNC:4037]
ENSG00000166801	0,66980445	1,51155332	9,00634993	0,01448735	0,15510091	FAM111A	family with sequence similarity 111 member A [Source:HGNC Symbol;Acc:HGNC:24725]

ENSG00000167550	0,84073212	1,331079	9,00519405	0,01449194	0,15510091	RHEBL1	RHEB like 1 [Source:HGNC Symbol;Acc:HGNC:21166]
ENSG00000196646	0,5129375	2,47132329	9,00578597	0,01448959	0,15510091	ZNF136	zinc finger protein 136 [Source:HGNC Symbol;Acc:HGNC:12920]
ENSG00000005302	-0,730324	2,24640239	8,9911637	0,01454777	0,15513024	MSL3	MSL complex subunit 3 [Source:HGNC Symbol;Acc:HGNC:7370]
ENSG00000139687	0,49474379	3,52620743	8,99629554	0,01452732	0,15513024	RB1	RB transcriptional corepressor 1 [Source:HGNC Symbol;Acc:HGNC:9884]
ENSG00000178074	0,50168439	4,28487853	8,99199328	0,01454447	0,15513024	C2orf69	chromosome 2 open reading frame 69 [Source:HGNC Symbol;Acc:HGNC:26799]
ENSG00000196821	0,44323995	6,32582077	8,99928002	0,01451544	0,15513024	ILRUN	inflammation and lipid regulator with UBA-like and NBR1-like domains [Source:HGNC Symbol;Acc:HGNC:21215]
ENSG00000197971	0,73406939	1,53743613	8,99639074	0,01452694	0,15513024	MBP	myelin basic protein [Source:HGNC Symbol;Acc:HGNC:6925]
ENSG00000104177	0,75247415	2,35292225	8,98434899	0,01457499	0,15522166	MYEF2	myelin expression factor 2 [Source:HGNC Symbol;Acc:HGNC:17940]
ENSG00000108950	-0,5810635	2,89843119	8,98369656	0,0145776	0,15522166	FAM20A	FAM20A golgi associated secretory pathway pseudokinase [Source:HGNC Symbol;Acc:HGNC:23015]
ENSG00000035928	0,43974247	6,24757427	8,98021024	0,01459155	0,15524775	RFC1	replication factor C subunit 1 [Source:HGNC Symbol;Acc:HGNC:9969]
ENSG00000115657	-0,5181954	2,71184029	8,977775	0,0146013	0,15524775	ABCB6	ATP binding cassette subfamily B member 6 (Langereis blood group) [Source:HGNC Symbol;Acc:HGNC:47]
ENSG00000141424	0,52878464	5,80415207	8,96171726	0,01466582	0,15575085	SLC39A6	solute carrier family 39 member 6 [Source:HGNC Symbol;Acc:HGNC:18607]
ENSG00000184307	-0,567691	1,65663326	8,96069399	0,01466994	0,15575085	ZDHHC23	zinc finger DHHC-type palmitoyltransferase 23 [Source:HGNC Symbol;Acc:HGNC:28654]
ENSG00000096088	0,53230009	7,95418538	8,94283818	0,01474212	0,1564035	PGC	progastricsin [Source:HGNC Symbol;Acc:HGNC:8890]
ENSG00000160145	-0,4912176	4,18650192	8,93354603	0,01477985	0,15649207	KALRN	kalirin RhoGEF kinase [Source:HGNC Symbol;Acc:HGNC:4814]
ENSG00000166343	1,24477834	-1,0609254	8,93286983	0,0147826	0,15649207	MSS51	MSS51 mitochondrial translational activator [Source:HGNC Symbol;Acc:HGNC:21000]
ENSG00000187189	0,57907913	3,97437345	8,93798193	0,01476182	0,15649207	TSPYL4	TSPY like 4 [Source:HGNC Symbol;Acc:HGNC:21559]
ENSG00000173080	1,05610291	0,4036997	8,91914254	0,01483858	0,15674388	RXFP4	relaxin family peptide/INSL5 receptor 4 [Source:HGNC Symbol;Acc:HGNC:14666]
ENSG00000174886	-0,5366805	4,16291446	8,91955042	0,01483691	0,15674388	NDUFA11	NADH:ubiquinone oxidoreductase subunit A11 [Source:HGNC Symbol;Acc:HGNC:20371]
ENSG00000196975	-0,4003857	7,71382026	8,92040403	0,01483342	0,15674388	ANXA4	annexin A4 [Source:HGNC Symbol;Acc:HGNC:542]
ENSG00000185480	0,53418758	3,40187835	8,91540162	0,01485388	0,15679211	PARPBP	PARP1 binding protein [Source:HGNC Symbol;Acc:HGNC:26074]
ENSG00000131023	0,49993611	3,03482013	8,90033838	0,01491568	0,15733076	LATS1	large tumor suppressor kinase 1 [Source:HGNC Symbol;Acc:HGNC:6514]
ENSG00000110063	-0,4327688	5,13984755	8,89682323	0,01493014	0,15736974	DCPS	decapping enzyme, scavenger [Source:HGNC Symbol;Acc:HGNC:29812]
ENSG00000137807	-0,4869136	4,77621311	8,88920483	0,01496156	0,15752519	KIF23	kinesin family member 23 [Source:HGNC Symbol;Acc:HGNC:6392]
ENSG00000141391	-0,653808	1,39153668	8,88540874	0,01497724	0,15752519	PRELID3A	PRELI domain containing 3A [Source:HGNC Symbol;Acc:HGNC:24639]
ENSG00000163435	0,59998119	3,18615304	8,8864153	0,01497308	0,15752519	ELF3	E74 like ETS transcription factor 3 [Source:HGNC Symbol;Acc:HGNC:3318]
ENSG00000127995	0,61394931	3,21620312	8,87330203	0,01502739	0,15789304	CASD1	CAS1 domain containing 1 [Source:HGNC Symbol;Acc:HGNC:16014]
ENSG00000135821	0,46505007	9,08786272	8,87175129	0,01503383	0,15789304	GLUL	glutamate-ammonia ligase [Source:HGNC Symbol;Acc:HGNC:4341]
ENSG00000105854	-0,4543464	8,19092134	8,86802736	0,01504931	0,15794203	PON2	paraoxonase 2 [Source:HGNC Symbol;Acc:HGNC:9205]
ENSG00000124406	-0,8443442	0,17548004	8,83409724	0,01519122	0,15931693	ATP8A1	ATPase phospholipid transporting 8A1 [Source:HGNC Symbol;Acc:HGNC:13531]
ENSG00000120256	0,8593697	0,49283503	8,83010022	0,01520804	0,15937897	LRP11	LDL receptor related protein 11 [Source:HGNC Symbol;Acc:HGNC:16936]
ENSG00000105173	0,6112062	5,42707243	8,82730281	0,01521983	0,15938818	CCNE1	cyclin E1 [Source:HGNC Symbol;Acc:HGNC:1589]
ENSG00000152082	-0,5332358	6,26632957	8,8239022	0,01523418	0,15942414	MZT2B	mitotic spindle organizing protein 2B [Source:HGNC Symbol;Acc:HGNC:25886]
ENSG00000064419	0,41812326	5,09409801	8,79573957	0,01535363	0,15966871	TNPO3	transportin 3 [Source:HGNC Symbol;Acc:HGNC:17103]
ENSG00000073910	0,67365075	2,41993366	8,79546345	0,01535481	0,15966871	FRY	FRY microtubule binding protein [Source:HGNC Symbol;Acc:HGNC:20367]
ENSG00000124098	-0,3953404	6,83402731	8,81497151	0,01527193	0,15966871	FAM210B	family with sequence similarity 210 member B [Source:HGNC Symbol;Acc:HGNC:16102]
ENSG00000157540	0,58598372	3,97833432	8,80116203	0,01533054	0,15966871	DYRK1A	dual specificity tyrosine phosphorylation regulated kinase 1A [Source:HGNC Symbol;Acc:HGNC:3091]
ENSG00000167632	-0,5245771	3,00451295	8,79542589	0,01535497	0,15966871	TRAPPC9	trafficking protein particle complex 9 [Source:HGNC Symbol;Acc:HGNC:30832]
ENSG00000169762	0,64715902	3,29687978	8,7988769	0,01534027	0,15966871	TAPT1	transmembrane anterior posterior transformation 1 [Source:HGNC Symbol;Acc:HGNC:26887]
ENSG00000170430	-0,4524272	5,05362802	8,81125325	0,01528769	0,15966871	MGMT	O-6-methylguanine-DNA methyltransferase [Source:HGNC Symbol;Acc:HGNC:7059]
ENSG00000196705	0,48826361	3,12263235	8,80200138	0,01532697	0,15966871	ZNF431	zinc finger protein 431 [Source:HGNC Symbol;Acc:HGNC:20809]
ENSG00000261371	-0,6485226	1,31176409	8,79520389	0,01535591	0,15966871	PECAM1	platelet and endothelial cell adhesion molecule 1 [Source:HGNC Symbol;Acc:HGNC:8823]
ENSG00000287837	1,01050414	-0,1395889	8,79131525	0,0153725	0,15972749	AL590064.1	novel transcript
ENSG00000135929	0,39492495	9,24626548	8,78794141	0,01538691	0,15976358	CYP27A1	cytochrome P450 family 27 subfamily A member 1 [Source:HGNC Symbol;Acc:HGNC:2605]

ENSG00000128311	-0,4419544	5,36361802	8,77346562	0,01544892	0,16017976	TST	thiosulfate sulfurtransferase [Source:HGNC Symbol;Acc:HGNC:12388]
ENSG00000235257	-0,740882	0,61664718	8,77383081	0,01544735	0,16017976	ITGA9-AS1	ITGA9 antisense RNA 1 [Source:HGNC Symbol;Acc:HGNC:49668]
ENSG00000159055	0,46809919	5,03631989	8,76099673	0,01550258	0,1605083	MIS18A	MIS18 kinetochore protein A [Source:HGNC Symbol;Acc:HGNC:1286]
ENSG00000161179	-0,512265	5,09022185	8,76313163	0,01549338	0,1605083	YDJC	YdjC chitooligosaccharide deacetylase homolog [Source:HGNC Symbol;Acc:HGNC:27158]
ENSG00000039319	0,54622463	3,6741737	8,75117947	0,01554499	0,16052188	ZFYVE16	zinc finger FYVE-type containing 16 [Source:HGNC Symbol;Acc:HGNC:20756]
ENSG00000089723	-0,5008531	2,33600281	8,75576484	0,01552517	0,16052188	OTUB2	OTU deubiquitinase, ubiquitin aldehyde binding 2 [Source:HGNC Symbol;Acc:HGNC:20351]
ENSG00000114378	-0,3947142	7,35475461	8,74798261	0,01555883	0,16052188	HYAL1	hyaluronidase 1 [Source:HGNC Symbol;Acc:HGNC:5320]
ENSG00000124602	0,89962465	1,11676044	8,75319161	0,01553629	0,16052188	UNC5CL	unc-5 family C-terminal like [Source:HGNC Symbol;Acc:HGNC:21203]
ENSG00000165272	-1,2690238	3,62348093	8,7498708	0,01555066	0,16052188	AQP3	aquaporin 3 (Gill blood group) [Source:HGNC Symbol;Acc:HGNC:636]
ENSG00000125657	-1,0606022	0,99473728	8,73775046	0,01560324	0,16086639	TNFSF9	TNF superfamily member 9 [Source:HGNC Symbol;Acc:HGNC:11939]
ENSG00000166483	0,42252724	5,06632035	8,72430438	0,01566182	0,16135653	WEE1	WEE1 G2 checkpoint kinase [Source:HGNC Symbol;Acc:HGNC:12761]
ENSG00000119285	0,48644396	5,36713016	8,71839994	0,01568763	0,16139482	HEATR1	HEAT repeat containing 1 [Source:HGNC Symbol;Acc:HGNC:25517]
ENSG00000185129	-0,4891969	2,67166617	8,71843969	0,01568746	0,16139482	PURA	purine rich element binding protein A [Source:HGNC Symbol;Acc:HGNC:9701]
ENSG00000096433	-0,8904558	4,71134913	8,70996367	0,01572461	0,16166133	ITPR3	inositol 1,4,5-trisphosphate receptor type 3 [Source:HGNC Symbol;Acc:HGNC:6182]
ENSG00000136478	-0,4427669	5,85370437	8,70368424	0,01575219	0,16183108	TEX2	testis expressed 2 [Source:HGNC Symbol;Acc:HGNC:30884]
ENSG00000146215	0,97607152	0,74296668	8,69844515	0,01577526	0,16184039	CRIP3	cysteine rich protein 3 [Source:HGNC Symbol;Acc:HGNC:17751]
ENSG00000173226	-0,4943211	4,29734687	8,69954284	0,01577042	0,16184039	IQCB1	IQ motif containing B1 [Source:HGNC Symbol;Acc:HGNC:28949]
ENSG00000054967	-0,5022299	3,39464773	8,67769322	0,01586702	0,16212264	RELT	RELT TNF receptor [Source:HGNC Symbol;Acc:HGNC:13764]
ENSG00000080986	0,456021	4,3416795	8,68254089	0,01584552	0,16212264	NDC80	NDC80 kinetochore complex component [Source:HGNC Symbol;Acc:HGNC:16909]
ENSG00000106993	0,50077666	3,7133574	8,67319322	0,015887	0,16212264	CDC37L1	cell division cycle 37 like 1 [Source:HGNC Symbol;Acc:HGNC:17179]
ENSG00000109089	-0,8787859	0,85321875	8,68134171	0,01585084	0,16212264	CDR2L	cerebellar degeneration related protein 2 like [Source:HGNC Symbol;Acc:HGNC:29999]
ENSG00000147416	0,50576764	6,6785955	8,67447218	0,01588132	0,16212264	ATP6V1B2	ATPase H+ transporting V1 subunit B2 [Source:HGNC Symbol;Acc:HGNC:854]
ENSG00000162702	0,58805522	3,74464665	8,6721706	0,01589155	0,16212264	ZNF281	zinc finger protein 281 [Source:HGNC Symbol;Acc:HGNC:13075]
ENSG00000175189	0,60240101	1,47812979	8,68620244	0,01582931	0,16212264	INHBC	inhibin subunit beta C [Source:HGNC Symbol;Acc:HGNC:6068]
ENSG00000205544	-0,6178355	4,23736188	8,68077752	0,01585334	0,16212264	TMEM256	transmembrane protein 256 [Source:HGNC Symbol;Acc:HGNC:28618]
ENSG00000179899	1,67237274	-2,0206765	8,66202867	0,01593672	0,16246999	PHC1P1	polyhomeotic homolog 1 pseudogene 1 [Source:HGNC Symbol;Acc:HGNC:34502]
ENSG00000182979	-0,4083294	5,9402598	8,65945021	0,01594823	0,16247394	MTA1	metastasis associated 1 [Source:HGNC Symbol;Acc:HGNC:7410]
ENSG00000116299	-1,6568271	-2,0346362	8,65099284	0,01598605	0,16263152	ELAPOR1	endosome-lysosome associated apoptosis and autophagy regulator 1 [Source:HGNC Symbol;Acc:HGNC:29618]
ENSG00000125457	-0,552824	4,52765088	8,64604553	0,01600822	0,16263152	MIF4GD	MIF4G domain containing [Source:HGNC Symbol;Acc:HGNC:24030]
ENSG00000236801	-0,5763503	1,66904212	8,64815697	0,01599875	0,16263152	RPL24P8	RPL24 pseudogene 8 [Source:HGNC Symbol;Acc:HGNC:36409]
ENSG00000241878	0,42988263	6,95183181	8,65188373	0,01598206	0,16263152	PISD	phosphatidylserine decarboxylase [Source:HGNC Symbol;Acc:HGNC:8999]
ENSG00000102100	0,41332439	6,14830554	8,63208406	0,01607101	0,16310486	SLC35A2	solute carrier family 35 member A2 [Source:HGNC Symbol;Acc:HGNC:11022]
ENSG00000141699	0,40631676	5,17620574	8,62824833	0,01608831	0,16310486	RETREG3	reticulophagy regulator family member 3 [Source:HGNC Symbol;Acc:HGNC:27258]
ENSG00000172244	0,56286805	1,81206204	8,62915372	0,01608422	0,16310486	C5orf34	chromosome 5 open reading frame 34 [Source:HGNC Symbol;Acc:HGNC:24738]
ENSG00000141279	0,52258949	6,77611411	8,62498132	0,01610306	0,16314123	NPEPPS	aminopeptidase puromycin sensitive [Source:HGNC Symbol;Acc:HGNC:7900]
ENSG00000085185	0,96422732	2,38441633	8,60916524	0,01617473	0,16325999	BCORL1	BCL6 corepressor like 1 [Source:HGNC Symbol;Acc:HGNC:25657]
ENSG00000145817	0,52471848	5,99746121	8,61204279	0,01616166	0,16325999	YIPF5	Yip1 domain family member 5 [Source:HGNC Symbol;Acc:HGNC:24877]
ENSG00000149313	0,42772231	5,03540274	8,60288626	0,01620329	0,16325999	AASDHPPT	aminoadipate-semialdehyde dehydrogenase-phosphopantetheinyl transferase [Source:HGNC Symbol;Acc:HGNC:1
ENSG00000153029	-0,5433784	3,6997731	8,60268831	0,01620419	0,16325999	MR1	major histocompatibility complex, class I-related [Source:HGNC Symbol;Acc:HGNC:4975]
ENSG00000169604	0,44311942	4,76246069	8,61377264	0,01615381	0,16325999	ANTXR1	ANTXR cell adhesion molecule 1 [Source:HGNC Symbol;Acc:HGNC:21014]
ENSG00000182580	1,90990675	-1,6043913	8,60909554	0,01617504	0,16325999	EPHB3	EPH receptor B3 [Source:HGNC Symbol;Acc:HGNC:3394]
ENSG00000225200	-0,523079	2,35707081	8,61569063	0,01614511	0,16325999	AC246787.1	ribosomal protein S8 (RPS8) pseudogene
ENSG00000267260	2,40515172	-2,0938569	8,60414574	0,01619755	0,16325999	AC020928.1	novel transcript
ENSG00000007080	-0,4365119	5,66104119	8,59650251	0,01623239	0,16343139	CCDC124	coiled-coil domain containing 124 [Source:HGNC Symbol;Acc:HGNC:25171]

ENSG00000187736	0,84261449	1,05874432	8,58937305	0,01626496	0,16364657	NHEJ1	non-homologous end joining factor 1 [Source:HGNC Symbol;Acc:HGNC:25737]
ENSG00000081803	0,47801686	5,280742	8,56161713	0,01639255	0,16368091	CADPS2	calcium dependent secretion activator 2 [Source:HGNC Symbol;Acc:HGNC:16018]
ENSG00000115286	-0,4791893	6,21036604	8,57133142	0,01634775	0,16368091	NDUFS7	NADH:ubiquinone oxidoreductase core subunit S7 [Source:HGNC Symbol;Acc:HGNC:7714]
ENSG00000115649	0,41555811	6,49745916	8,54857497	0,01645292	0,16368091	CNPPD1	cyclin Pas1/PHO80 domain containing 1 [Source:HGNC Symbol;Acc:HGNC:25220]
ENSG00000115756	-0,437063	4,68854217	8,57059925	0,01635112	0,16368091	HPCAL1	hippocalcin like 1 [Source:HGNC Symbol;Acc:HGNC:5145]
ENSG00000126218	-0,6322966	5,88586636	8,57244703	0,01634262	0,16368091	F10	coagulation factor X [Source:HGNC Symbol;Acc:HGNC:3528]
ENSG00000131730	-0,9087402	0,75476253	8,56312231	0,0163856	0,16368091	CKMT2	creatine kinase, mitochondrial 2 [Source:HGNC Symbol;Acc:HGNC:1996]
ENSG00000135093	0,4584774	3,55913666	8,55211876	0,01643649	0,16368091	USP30	ubiquitin specific peptidase 30 [Source:HGNC Symbol;Acc:HGNC:20065]
ENSG00000135597	0,4229382	5,17213086	8,55929199	0,01640329	0,16368091	REPS1	RALBP1 associated Eps domain containing 1 [Source:HGNC Symbol;Acc:HGNC:15578]
ENSG00000139865	0,67852681	2,09033501	8,55754212	0,01641138	0,16368091	TTC6	tetratricopeptide repeat domain 6 [Source:HGNC Symbol;Acc:HGNC:19739]
ENSG00000140368	-0,7115678	2,43591524	8,58306627	0,01629384	0,16368091	PSTPIP1	proline-serine-threonine phosphatase interacting protein 1 [Source:HGNC Symbol;Acc:HGNC:9580]
ENSG00000147642	0,63606531	2,64217641	8,57039166	0,01635208	0,16368091	SYBU	syntabulin [Source:HGNC Symbol;Acc:HGNC:26011]
ENSG00000147813	-0,8699413	0,82946032	8,57873324	0,01631372	0,16368091	NAPRT	nicotinate phosphoribosyltransferase [Source:HGNC Symbol;Acc:HGNC:30450]
ENSG00000164002	0,56385261	3,85665419	8,55088734	0,0164422	0,16368091	EXO5	exonuclease 5 [Source:HGNC Symbol;Acc:HGNC:26115]
ENSG00000181274	0,45788968	4,73244644	8,56856023	0,01636052	0,16368091	FRAT2	FRAT regulator of WNT signaling pathway 2 [Source:HGNC Symbol;Acc:HGNC:16048]
ENSG00000181856	-0,4936268	2,74680475	8,54729887	0,01645884	0,16368091	SLC2A4	solute carrier family 2 member 4 [Source:HGNC Symbol;Acc:HGNC:11009]
ENSG00000184304	1,48250139	-1,2789799	8,55870272	0,01640601	0,16368091	PRKD1	protein kinase D1 [Source:HGNC Symbol;Acc:HGNC:9407]
ENSG00000228657	-0,6386284	1,21666044	8,55391288	0,01642818	0,16368091	RPL23AP58	ribosomal protein L23a pseudogene 58 [Source:HGNC Symbol;Acc:HGNC:36138]
ENSG00000183098	0,54833917	4,81874761	8,54379407	0,01647512	0,16373133	GPC6	glypican 6 [Source:HGNC Symbol;Acc:HGNC:4454]
ENSG00000186594	-0,5313066	2,45512848	8,5403181	0,01649129	0,16378056	MIR22HG	MIR22 host gene [Source:HGNC Symbol;Acc:HGNC:28219]
ENSG00000120885	-0,6226833	9,47872898	8,53723658	0,01650563	0,16381167	CLU	clusterin [Source:HGNC Symbol;Acc:HGNC:2095]
ENSG00000033800	0,46502066	4,28991988	8,5192513	0,01658967	0,16397731	PIAS1	protein inhibitor of activated STAT 1 [Source:HGNC Symbol;Acc:HGNC:2752]
ENSG00000130270	-0,5881522	1,49832647	8,51986843	0,01658678	0,16397731	ATP8B3	ATPase phospholipid transporting 8B3 [Source:HGNC Symbol;Acc:HGNC:13535]
ENSG00000169188	-0,4794535	5,11378379	8,51926846	0,01658959	0,16397731	APEX2	apurinic/apyrimidinic endodeoxyribonuclease 2 [Source:HGNC Symbol;Acc:HGNC:17889]
ENSG00000170190	-0,7069528	1,38135781	8,52427539	0,01656614	0,16397731	SLC16A5	solute carrier family 16 member 5 [Source:HGNC Symbol;Acc:HGNC:10926]
ENSG00000171346	-1,8170691	-1,10809	8,52339283	0,01657027	0,16397731	KRT15	keratin 15 [Source:HGNC Symbol;Acc:HGNC:6421]
ENSG00000175198	0,59975646	5,29126426	8,52693524	0,0165537	0,16397731	PCCA	propionyl-CoA carboxylase subunit alpha [Source:HGNC Symbol;Acc:HGNC:8653]
ENSG00000112149	0,84032571	1,34125452	8,51208145	0,01662332	0,1641988	CD83	CD83 molecule [Source:HGNC Symbol;Acc:HGNC:1703]
ENSG00000118162	-0,4667146	3,67045421	8,49801149	0,01668959	0,16448713	KPTN	kaptin, actin binding protein [Source:HGNC Symbol;Acc:HGNC:6404]
ENSG00000130227	0,44963713	5,22694627	8,49632767	0,01669754	0,16448713	XPO7	exportin 7 [Source:HGNC Symbol;Acc:HGNC:14108]
ENSG00000163703	-0,8417591	1,26888715	8,50038775	0,01667837	0,16448713	CRELD1	cysteine rich with EGF like domains 1 [Source:HGNC Symbol;Acc:HGNC:14630]
ENSG00000165449	1,08402641	-0,5814158	8,49854451	0,01668707	0,16448713	SLC16A9	solute carrier family 16 member 9 [Source:HGNC Symbol;Acc:HGNC:23520]
ENSG00000213942	-0,5972446	1,61562984	8,48854929	0,01673434	0,16473855	AC005840.1	ribosomal protein L31 (RPL31) pseudogene
ENSG00000113163	-0,459328	3,33185874	8,4846064	0,01675303	0,1648115	CERT1	ceramide transporter 1 [Source:HGNC Symbol;Acc:HGNC:2205]
ENSG00000124216	-0,6764017	2,02385049	8,4804103	0,01677295	0,16486021	SNAI1	snail family transcriptional repressor 1 [Source:HGNC Symbol;Acc:HGNC:11128]
ENSG00000225830	0,57841819	2,57877539	8,47881078	0,01678056	0,16486021	ERCC6	ERCC excision repair 6, chromatin remodeling factor [Source:HGNC Symbol;Acc:HGNC:3438]
ENSG00000183617	-0,4368978	6,17680195	8,47267022	0,01680978	0,1650363	MRPL54	mitochondrial ribosomal protein L54 [Source:HGNC Symbol;Acc:HGNC:16685]
ENSG00000135127	0,55389232	2,40317924	8,4697127	0,01682387	0,16506376	BICDL1	BICD family like cargo adaptor 1 [Source:HGNC Symbol;Acc:HGNC:28095]
ENSG00000165807	1,35772424	-1,5092588	8,46438882	0,01684928	0,1650958	PPP1R36	protein phosphatase 1 regulatory subunit 36 [Source:HGNC Symbol;Acc:HGNC:20097]
ENSG00000204385	-0,6356539	4,45394946	8,46429334	0,01684974	0,1650958	SLC44A4	solute carrier family 44 member 4 [Source:HGNC Symbol;Acc:HGNC:13941]
ENSG00000005436	0,43301014	4,83393077	8,46182326	0,01686154	0,16510074	GCFC2	GC-rich sequence DNA-binding factor 2 [Source:HGNC Symbol;Acc:HGNC:1317]
ENSG00000106077	-0,4953605	3,45888876	8,45091726	0,01691379	0,16541942	ABHD11	abhydrolase domain containing 11 [Source:HGNC Symbol;Acc:HGNC:16407]
ENSG00000120725	-0,5559845	6,55225403	8,44797272	0,01692793	0,16541942	SIL1	SIL1 nucleotide exchange factor [Source:HGNC Symbol;Acc:HGNC:24624]
ENSG00000124422	0,43315947	7,07732173	8,44794626	0,01692806	0,16541942	USP22	ubiquitin specific peptidase 22 [Source:HGNC Symbol;Acc:HGNC:12621]

ENSG00000254635	-0,6735862	1,83275164	8,44336875	0,01695007	0,1655238	WAC-AS1	WAC antisense RNA 1 (head to head) [Source:HGNC Symbol;Acc:HGNC:27347]
ENSG00000114698	0,88166685	0,03101292	8,42179299	0,0170543	0,16631926	PLSCR4	phospholipid scramblase 4 [Source:HGNC Symbol;Acc:HGNC:16497]
ENSG00000127663	-0,6850867	3,72690355	8,42257375	0,01705051	0,16631926	KDM4B	lysine demethylase 4B [Source:HGNC Symbol;Acc:HGNC:29136]
ENSG00000107262	-0,3920299	6,04453938	8,41409844	0,01709166	0,16657241	BAG1	BAG cochaperone 1 [Source:HGNC Symbol;Acc:HGNC:937]
ENSG00000099219	0,50841198	6,35294457	8,3968587	0,01717573	0,16716873	ERMP1	endoplasmic reticulum metallopeptidase 1 [Source:HGNC Symbol;Acc:HGNC:23703]
ENSG00000181315	0,47162071	2,91051576	8,39722684	0,01717393	0,16716873	ZNF322	zinc finger protein 322 [Source:HGNC Symbol;Acc:HGNC:23640]
ENSG00000067048	0,52954547	5,49285588	8,38393554	0,01723909	0,16767365	DDX3Y	DEAD-box helicase 3 Y-linked [Source:HGNC Symbol;Acc:HGNC:2699]
ENSG00000257167	-1,0058517	0,99185547	8,37864956	0,01726508	0,16781478	TMPO-AS1	TMPO antisense RNA 1 [Source:HGNC Symbol;Acc:HGNC:44158]
ENSG00000198089	-0,5109077	3,18155239	8,36985747	0,01730843	0,16812425	SFI1	SFI1 centrin binding protein [Source:HGNC Symbol;Acc:HGNC:29064]
ENSG00000007255	-0,5747501	2,11467676	8,35586528	0,01737769	0,16839297	TRAPPC6A	trafficking protein particle complex 6A [Source:HGNC Symbol;Acc:HGNC:23069]
ENSG00000119844	0,49774909	4,32798295	8,35842534	0,01736499	0,16839297	AFTPH	aftiphilin [Source:HGNC Symbol;Acc:HGNC:25951]
ENSG00000158769	0,47180262	5,41160286	8,35797309	0,01736724	0,16839297	F11R	F11 receptor [Source:HGNC Symbol;Acc:HGNC:14685]
ENSG00000175183	-0,7541124	1,63622788	8,35495641	0,0173822	0,16839297	CSRP2	cysteine and glycine rich protein 2 [Source:HGNC Symbol;Acc:HGNC:2470]
ENSG00000089327	-1,1662195	-0,4004275	8,34532337	0,0174301	0,16852173	FXYD5	FXYD domain containing ion transport regulator 5 [Source:HGNC Symbol;Acc:HGNC:4029]
ENSG00000159882	0,69861321	0,82356712	8,34685272	0,01742249	0,16852173	ZNF230	zinc finger protein 230 [Source:HGNC Symbol;Acc:HGNC:13024]
ENSG00000177105	-0,4846718	5,11604552	8,34747289	0,0174194	0,16852173	RHOG	ras homolog family member G [Source:HGNC Symbol;Acc:HGNC:672]
ENSG00000103342	-0,5218868	8,4445231	8,34287902	0,01744228	0,16852795	GSPT1	G1 to S phase transition 1 [Source:HGNC Symbol;Acc:HGNC:4621]
ENSG00000142669	-0,4069355	6,04752984	8,33606539	0,01747628	0,16874491	SH3BGRL3	SH3 domain binding glutamate rich protein like 3 [Source:HGNC Symbol;Acc:HGNC:15568]
ENSG00000018610	0,43401105	4,03043402	8,3210659	0,01755143	0,1688011	CXorf56	chromosome X open reading frame 56 [Source:HGNC Symbol;Acc:HGNC:26239]
ENSG00000138801	0,48663896	6,4516827	8,32221332	0,01754567	0,1688011	PAPSS1	3'-phosphoadenosine 5'-phosphosulfate synthase 1 [Source:HGNC Symbol;Acc:HGNC:8603]
ENSG00000146416	-0,4028337	7,07138373	8,32398864	0,01753676	0,1688011	AIG1	androgen induced 1 [Source:HGNC Symbol;Acc:HGNC:21607]
ENSG00000168672	1,34185131	-0,6720328	8,32295886	0,01754193	0,1688011	LRATD2	LRAT domain containing 2 [Source:HGNC Symbol;Acc:HGNC:24166]
ENSG00000181924	-0,5210762	5,89423497	8,32469134	0,01753323	0,1688011	COA4	cytochrome c oxidase assembly factor 4 homolog [Source:HGNC Symbol;Acc:HGNC:24604]
ENSG00000255302	0,73233667	1,7466667	8,33134207	0,01749991	0,1688011	EID1	EP300 interacting inhibitor of differentiation 1 [Source:HGNC Symbol;Acc:HGNC:1191]
ENSG00000141428	-0,4916215	3,89641855	8,31634067	0,01757519	0,16891837	C18orf21	chromosome 18 open reading frame 21 [Source:HGNC Symbol;Acc:HGNC:28802]
ENSG00000163428	-0,475377	3,28384209	8,30325638	0,01764117	0,16937278	LRRC58	leucine rich repeat containing 58 [Source:HGNC Symbol;Acc:HGNC:26968]
ENSG00000167081	0,5972508	2,24649667	8,30237004	0,01764565	0,16937278	PBX3	PBX homeobox 3 [Source:HGNC Symbol;Acc:HGNC:8634]
ENSG00000124615	-0,5367966	3,64786925	8,30000906	0,0176576	0,16937613	MOCS1	molybdenum cofactor synthesis 1 [Source:HGNC Symbol;Acc:HGNC:7190]
ENSG00000234648	-0,595579	1,54781673	8,29760238	0,01766978	0,16938179	AL162151.2	pseudogene similar to part of ribosomal protein L3 (RPL3)
ENSG00000107864	1,22056967	-0,6032684	8,27252436	0,01779736	0,16971388	CPEB3	cytoplasmic polyadenylation element binding protein 3 [Source:HGNC Symbol;Acc:HGNC:21746]
ENSG00000111670	0,45198196	4,53240836	8,27627055	0,01777823	0,16971388	GNPTAB	N-acetylglucosamine-1-phosphate transferase subunits alpha and beta [Source:HGNC Symbol;Acc:HGNC:29670]
ENSG00000152926	-1,0712749	0,12120638	8,27993111	0,01775956	0,16971388	ZNF117	zinc finger protein 117 [Source:HGNC Symbol;Acc:HGNC:12897]
ENSG00000169057	0,4867873	3,96494809	8,27750208	0,01777195	0,16971388	MECP2	methyl-CpG binding protein 2 [Source:HGNC Symbol;Acc:HGNC:6990]
ENSG00000174243	0,42400391	6,98005917	8,2860121	0,01772861	0,16971388	DDX23	DEAD-box helicase 23 [Source:HGNC Symbol;Acc:HGNC:17347]
ENSG00000182541	-0,4321252	5,60364884	8,27280493	0,01779593	0,16971388	LIMK2	LIM domain kinase 2 [Source:HGNC Symbol;Acc:HGNC:6614]
ENSG00000204227	-0,4028441	5,50914944	8,27757992	0,01777155	0,16971388	RING1	ring finger protein 1 [Source:HGNC Symbol;Acc:HGNC:10018]
ENSG00000225439	-0,496706	2,8395438	8,27280023	0,01779595	0,16971388	BOLA3-AS1	BOLA3 divergent transcript [Source:HGNC Symbol;Acc:HGNC:42922]
ENSG00000035681	-0,4198054	4,37517474	8,25509122	0,01788672	0,16979016	NSMAF	neutral sphingomyelinase activation associated factor [Source:HGNC Symbol;Acc:HGNC:8017]
ENSG00000110422	0,55993712	2,63708677	8,25540073	0,01788513	0,16979016	HIPK3	homeodomain interacting protein kinase 3 [Source:HGNC Symbol;Acc:HGNC:4915]
ENSG00000119913	-1,1631017	-0,1408644	8,26572958	0,01783212	0,16979016	TECTB	tectorin beta [Source:HGNC Symbol;Acc:HGNC:11721]
ENSG00000129925	-0,4458409	6,07395469	8,26731373	0,01782401	0,16979016	PGAP6	post-glycosylphosphatidylinositol attachment to proteins 6 [Source:HGNC Symbol;Acc:HGNC:17205]
ENSG00000153339	0,46135437	3,63147434	8,2584849	0,01786928	0,16979016	TRAPPC8	trafficking protein particle complex 8 [Source:HGNC Symbol;Acc:HGNC:29169]
ENSG00000223529	-0,6930257	1,21853402	8,26413667	0,01784029	0,16979016	EEF1A1P8	eukaryotic translation elongation factor 1 alpha 1 pseudogene 8 [Source:HGNC Symbol;Acc:HGNC:3203]
ENSG00000272931	-0,6389054	1,14184464	8,25891622	0,01786707	0,16979016	AC099568.2	novel transcript

ENSG00000114126	0,50455608	4,16612198	8,24569681	0,0179351	0,16991728	TFDP2	transcription factor Dp-2 [Source:HGNC Symbol;Acc:HGNC:11751]
ENSG00000144285	0,92616398	1,28668021	8,24929345	0,01791656	0,16991728	SCN1A	sodium voltage-gated channel alpha subunit 1 [Source:HGNC Symbol;Acc:HGNC:10585]
ENSG00000144908	-0,630003	4,4769437	8,24346179	0,01794663	0,16991728	ALDH1L1	aldehyde dehydrogenase 1 family member L1 [Source:HGNC Symbol;Acc:HGNC:3978]
ENSG00000288558	1,07045436	-0,4520063	8,24750801	0,01792576	0,16991728	DUS4L-BCAP29	DUS4L-BCAP29 readthrough [Source:HGNC Symbol;Acc:HGNC:54422]
ENSG00000155961	-1,5562912	-0,6700268	8,23685013	0,01798081	0,17002045	RAB39B	RAB39B, member RAS oncogene family [Source:HGNC Symbol;Acc:HGNC:16499]
ENSG00000184207	-0,4532448	5,55225304	8,2384627	0,01797246	0,17002045	PGP	phosphoglycolate phosphatase [Source:HGNC Symbol;Acc:HGNC:8909]
ENSG00000183283	-0,4444927	5,81698885	8,23372645	0,01799698	0,1700633	DAZAP2	DAZ associated protein 2 [Source:HGNC Symbol;Acc:HGNC:2684]
ENSG00000167130	0,40116114	5,86461786	8,22165168	0,01805966	0,17032514	DOLPP1	dolichyldiphosphatase 1 [Source:HGNC Symbol;Acc:HGNC:29565]
ENSG00000182325	-0,4952766	4,84471453	8,22201504	0,01805777	0,17032514	FBXL6	F-box and leucine rich repeat protein 6 [Source:HGNC Symbol;Acc:HGNC:13603]
ENSG00000243477	-0,615653	1,99013422	8,22249334	0,01805529	0,17032514	NAA80	N-alpha-acetyltransferase 80, NatH catalytic subunit [Source:HGNC Symbol;Acc:HGNC:30252]
ENSG00000100418	-0,4368553	5,26405779	8,2075857	0,01813303	0,17042271	DESI1	desumoylating isopeptidase 1 [Source:HGNC Symbol;Acc:HGNC:24577]
ENSG00000116701	-1,3418234	-1,2518907	8,21438306	0,01809753	0,17042271	NCF2	neutrophil cytosolic factor 2 [Source:HGNC Symbol;Acc:HGNC:7661]
ENSG00000124155	0,45819794	8,10203831	8,20625181	0,01814	0,17042271	PIGT	phosphatidylinositol glycan anchor biosynthesis class T [Source:HGNC Symbol;Acc:HGNC:14938]
ENSG00000152518	0,42418428	5,94559207	8,21030838	0,0181188	0,17042271	ZFP36L2	ZFP36 ring finger protein like 2 [Source:HGNC Symbol;Acc:HGNC:1108]
ENSG00000158985	0,59170934	3,03965557	8,20910889	0,01812507	0,17042271	CDC42SE2	CDC42 small effector 2 [Source:HGNC Symbol;Acc:HGNC:18547]
ENSG00000174292	0,44583793	3,01003357	8,2080474	0,01813061	0,17042271	TNK1	tyrosine kinase non receptor 1 [Source:HGNC Symbol;Acc:HGNC:11940]
ENSG00000104859	-0,4561153	4,91179427	8,18289111	0,01826271	0,17141919	CLASRP	CLK4 associating serine/arginine rich protein [Source:HGNC Symbol;Acc:HGNC:17731]
ENSG00000153310	-0,5333469	4,8212105	8,17937473	0,01828127	0,17141919	CYRIB	CYFIP related Rac1 interactor B [Source:HGNC Symbol;Acc:HGNC:25216]
ENSG00000185220	0,6977095	2,00347467	8,18019078	0,01827696	0,17141919	PGBD2	piggyBac transposable element derived 2 [Source:HGNC Symbol;Acc:HGNC:19399]
ENSG00000169379	0,68711806	2,45251579	8,17087884	0,01832621	0,17173032	ARL13B	ADP ribosylation factor like GTPase 13B [Source:HGNC Symbol;Acc:HGNC:25419]
ENSG00000069248	0,50878806	5,40708149	8,16368071	0,01836439	0,17197776	NUP133	nucleoporin 133 [Source:HGNC Symbol;Acc:HGNC:18016]
ENSG00000128604	0,61226071	1,62067577	8,1607033	0,01838021	0,17201565	IRF5	interferon regulatory factor 5 [Source:HGNC Symbol;Acc:HGNC:6120]
ENSG00000188786	0,63608024	1,77966612	8,15848945	0,01839198	0,17201565	MTF1	metal regulatory transcription factor 1 [Source:HGNC Symbol;Acc:HGNC:7428]
ENSG00000076685	0,44982694	5,60324125	8,14377197	0,01847049	0,17241879	NT5C2	5'-nucleotidase, cytosolic II [Source:HGNC Symbol;Acc:HGNC:8022]
ENSG00000100106	-0,4271035	4,05845948	8,14710748	0,01845266	0,17241879	TRIOBP	TRIO and F-actin binding protein [Source:HGNC Symbol;Acc:HGNC:17009]
ENSG00000178177	0,47898752	2,85254045	8,14438806	0,0184672	0,17241879	LCORL	ligand dependent nuclear receptor corepressor like [Source:HGNC Symbol;Acc:HGNC:30776]
ENSG00000128335	-0,4028553	4,91738117	8,14083983	0,01848618	0,17245506	APOL2	apolipoprotein L2 [Source:HGNC Symbol;Acc:HGNC:619]
ENSG00000074696	0,46272484	8,17577299	8,12390509	0,01857713	0,17286193	HACD3	3-hydroxyacyl-CoA dehydratase 3 [Source:HGNC Symbol;Acc:HGNC:24175]
ENSG00000085741	1,23692227	-0,9706198	8,12443066	0,01857429	0,17286193	WNT11	Wnt family member 11 [Source:HGNC Symbol;Acc:HGNC:12776]
ENSG00000172053	-0,3796558	8,03106487	8,13018074	0,01854336	0,17286193	QARS1	glutaminyl-tRNA synthetase 1 [Source:HGNC Symbol;Acc:HGNC:9751]
ENSG00000244585	-1,8029669	-2,133401	8,12395012	0,01857688	0,17286193	RPL12P33	ribosomal protein L12 pseudogene 33 [Source:HGNC Symbol;Acc:HGNC:35696]
ENSG00000230371	-0,8563398	0,36482764	8,10345724	0,01868767	0,17377987	KARS1P2	lysyl-tRNA synthetase 1 pseudogene 2 [Source:HGNC Symbol;Acc:HGNC:39208]
ENSG00000081320	-0,8154165	0,56447712	8,10083297	0,01870192	0,17378464	STK17B	serine/threonine kinase 17b [Source:HGNC Symbol;Acc:HGNC:11396]
ENSG00000133169	0,44631467	5,84738871	8,09898139	0,01871197	0,17378464	BEX1	brain expressed X-linked 1 [Source:HGNC Symbol;Acc:HGNC:1036]
ENSG00000117758	-0,4435885	3,67014915	8,09166235	0,0187518	0,1738509	STX12	syntaxin 12 [Source:HGNC Symbol;Acc:HGNC:11430]
ENSG00000132613	0,44654162	7,13734565	8,0914571	0,01875292	0,1738509	MTSS2	MTSS I-BAR domain containing 2 [Source:HGNC Symbol;Acc:HGNC:25094]
ENSG00000230269	-0,590412	2,30477876	8,09017743	0,0187599	0,1738509	LINC02525	long intergenic non-protein coding RNA 2525 [Source:HGNC Symbol;Acc:HGNC:53545]
ENSG00000234380	-0,6040752	3,03519262	8,08892792	0,01876671	0,1738509	LINC01426	long intergenic non-protein coding RNA 1426 [Source:HGNC Symbol;Acc:HGNC:50734]
ENSG00000050327	0,95292553	-0,0512917	8,08414403	0,01879282	0,17398249	ARHGEF5	Rho guanine nucleotide exchange factor 5 [Source:HGNC Symbol;Acc:HGNC:13209]
ENSG00000262172	-0,9748202	-0,1539967	8,07453995	0,01884539	0,17435861	AC116025.2	novel transcript
ENSG00000121406	0,60683632	2,12617876	8,06006494	0,01892495	0,17464364	ZNF549	zinc finger protein 549 [Source:HGNC Symbol;Acc:HGNC:26632]
ENSG00000131462	-0,4916617	7,87992355	8,06225443	0,01891289	0,17464364	TUBG1	tubulin gamma 1 [Source:HGNC Symbol;Acc:HGNC:12417]
ENSG00000136932	0,50406453	3,80593046	8,05638956	0,01894522	0,17464364	TRMO	tRNA methyltransferase O [Source:HGNC Symbol;Acc:HGNC:30967]
ENSG00000138614	0,49693914	6,1930412	8,05589934	0,01894792	0,17464364	INTS14	integrator complex subunit 14 [Source:HGNC Symbol;Acc:HGNC:25372]

ENSG00000140323	-1,3100697	-0,192352	8,05646616	0,01894479	0,17464364	DISP2	dispatched RND transporter family member 2 [Source:HGNC Symbol;Acc:HGNC:19712]
ENSG00000157259	-0,4503753	4,38606764	8,06086143	0,01892056	0,17464364	GATAD1	GATA zinc finger domain containing 1 [Source:HGNC Symbol;Acc:HGNC:29941]
ENSG00000095383	0,47824172	2,52266182	8,05126283	0,01897353	0,17467703	TBC1D2	TBC1 domain family member 2 [Source:HGNC Symbol;Acc:HGNC:18026]
ENSG00000140382	0,42837799	3,87225289	8,05091454	0,01897546	0,17467703	HMG20A	high mobility group 20A [Source:HGNC Symbol;Acc:HGNC:5001]
ENSG00000005882	0,51245994	4,58023629	8,04742896	0,01899474	0,17474443	PDK2	pyruvate dehydrogenase kinase 2 [Source:HGNC Symbol;Acc:HGNC:8810]
ENSG00000056972	0,53052196	2,60914643	8,03972794	0,01903743	0,17486516	TRAF3IP2	TRAF3 interacting protein 2 [Source:HGNC Symbol;Acc:HGNC:1343]
ENSG00000106617	0,93320528	0,21498827	8,04135935	0,01902838	0,17486516	PRKAG2	protein kinase AMP-activated non-catalytic subunit gamma 2 [Source:HGNC Symbol;Acc:HGNC:9386]
ENSG00000180776	0,49409704	3,92414858	8,03858558	0,01904377	0,17486516	ZDHHC20	zinc finger DHHC-type palmitoyltransferase 20 [Source:HGNC Symbol;Acc:HGNC:20749]
ENSG00000162444	1,01951061	0,48789148	8,03312924	0,0190741	0,17503365	RBP7	retinol binding protein 7 [Source:HGNC Symbol;Acc:HGNC:30316]
ENSG00000153395	0,39550261	6,36308143	8,026147	0,019113	0,17528051	LPCAT1	lysophosphatidylcholine acyltransferase 1 [Source:HGNC Symbol;Acc:HGNC:25718]
ENSG00000067182	-0,3954526	7,05232595	8,01609054	0,0191692	0,17546177	TNFRSF1A	TNF receptor superfamily member 1A [Source:HGNC Symbol;Acc:HGNC:11916]
ENSG00000166816	-0,6504652	3,21562478	8,01802987	0,01915835	0,17546177	LDHD	lactate dehydrogenase D [Source:HGNC Symbol;Acc:HGNC:19708]
ENSG00000197208	-1,1861114	-1,1778372	8,02005031	0,01914705	0,17546177	SLC22A4	solute carrier family 22 member 4 [Source:HGNC Symbol;Acc:HGNC:10968]
ENSG00000211456	0,44591275	4,96532801	8,013177	0,01918552	0,17546177	SACM1L	SAC1 like phosphatidylinositide phosphatase [Source:HGNC Symbol;Acc:HGNC:17059]
ENSG00000214517	0,45266585	5,7786348	8,01187462	0,01919282	0,17546177	PPME1	protein phosphatase methylesterase 1 [Source:HGNC Symbol;Acc:HGNC:30178]
ENSG00000110442	0,37439451	5,8640815	8,00844123	0,01921208	0,17552803	COMMD9	COMM domain containing 9 [Source:HGNC Symbol;Acc:HGNC:25014]
ENSG00000078674	0,46399051	6,46035705	7,99972933	0,01926107	0,17559554	PCM1	pericentriolar material 1 [Source:HGNC Symbol;Acc:HGNC:8727]
ENSG00000132434	-0,4346498	4,37730149	7,99651556	0,01927917	0,17559554	LANCL2	LanC like 2 [Source:HGNC Symbol;Acc:HGNC:6509]
ENSG00000154589	-1,107116	0,26045523	7,9921975	0,01930354	0,17559554	LY96	lymphocyte antigen 96 [Source:HGNC Symbol;Acc:HGNC:17156]
ENSG00000164091	0,43394235	5,69466773	8,00048927	0,01925679	0,17559554	WDR82	WD repeat domain 82 [Source:HGNC Symbol;Acc:HGNC:28826]
ENSG00000241112	1,47080149	-0,9482212	7,99530915	0,01928598	0,17559554	RPL29P14	ribosomal protein L29 pseudogene 14 [Source:HGNC Symbol;Acc:HGNC:36687]
ENSG00000254614	-0,7348666	0,92054116	7,999586	0,01926187	0,17559554	AP003068.2	novel transcript, antisense to CAPN1
ENSG00000274979	0,76566157	1,50165588	7,99218481	0,01930361	0,17559554	AC020656.2	novel transcript
ENSG00000072415	0,43212757	3,89030148	7,97854837	0,0193808	0,17618799	MPP5	membrane palmitoylated protein 5 [Source:HGNC Symbol;Acc:HGNC:18669]
ENSG00000185252	0,47106044	3,49203001	7,97358154	0,01940901	0,17633471	ZNF74	zinc finger protein 74 [Source:HGNC Symbol;Acc:HGNC:13144]
ENSG00000152404	0,47198131	3,75432517	7,96780319	0,01944189	0,17652367	CWF19L2	CWF19 like cell cycle control factor 2 [Source:HGNC Symbol;Acc:HGNC:26508]
ENSG00000138166	-1,0085688	4,24469343	7,95717768	0,01950254	0,17663585	DUSP5	dual specificity phosphatase 5 [Source:HGNC Symbol;Acc:HGNC:3071]
ENSG00000171631	-1,159721	-0,9136963	7,96074811	0,01948213	0,17663585	P2RY6	pyrimidinergic receptor P2Y6 [Source:HGNC Symbol;Acc:HGNC:8543]
ENSG00000175197	-0,5026507	4,74725107	7,95914428	0,01949129	0,17663585	DDIT3	DNA damage inducible transcript 3 [Source:HGNC Symbol;Acc:HGNC:2726]
ENSG00000226479	-0,4082535	5,05934382	7,95716457	0,01950261	0,17663585	TMEM185B	transmembrane protein 185B [Source:HGNC Symbol;Acc:HGNC:18896]
ENSG00000204231	0,43472383	4,98182539	7,95317215	0,01952546	0,17673321	RXRB	retinoid X receptor beta [Source:HGNC Symbol;Acc:HGNC:10478]
ENSG00000175395	-1,0511003	-0,5464555	7,94561682	0,01956878	0,17701569	ZNF25	zinc finger protein 25 [Source:HGNC Symbol;Acc:HGNC:13043]
ENSG00000160298	-0,676923	1,4686606	7,9427581	0,01958521	0,17705463	C21orf58	chromosome 21 open reading frame 58 [Source:HGNC Symbol;Acc:HGNC:1300]
ENSG00000186301	-0,4630927	4,76169959	7,93123519	0,01965158	0,17754479	MST1P2	macrophage stimulating 1 pseudogene 2 [Source:HGNC Symbol;Acc:HGNC:7383]
ENSG00000177352	-0,4236641	4,73593852	7,92697804	0,01967617	0,1776571	CCDC71	coiled-coil domain containing 71 [Source:HGNC Symbol;Acc:HGNC:25760]
ENSG00000106078	-0,5007109	2,91405958	7,9207505	0,01971221	0,17787259	COBL	cordon-bleu WH2 repeat protein [Source:HGNC Symbol;Acc:HGNC:22199]
ENSG00000135587	-0,6396353	3,48911778	7,90459935	0,01980607	0,17860917	SMPD2	sphingomyelin phosphodiesterase 2 [Source:HGNC Symbol;Acc:HGNC:11121]
ENSG00000061987	0,48903229	3,38137678	7,89704854	0,01985014	0,17889613	MON2	MON2 homolog, regulator of endosome-to-Golgi trafficking [Source:HGNC Symbol;Acc:HGNC:29177]
ENSG00000105011	-0,4187084	4,84790797	7,89037056	0,01988921	0,17908321	ASF1B	anti-silencing function 1B histone chaperone [Source:HGNC Symbol;Acc:HGNC:20996]
ENSG00000139209	1,35999275	-0,2227644	7,88586161	0,01991564	0,17908321	SLC38A4	solute carrier family 38 member 4 [Source:HGNC Symbol;Acc:HGNC:14679]
ENSG00000162769	0,43437032	4,28255193	7,88701132	0,0199089	0,17908321	FLVCR1	FLVCR heme transporter 1 [Source:HGNC Symbol;Acc:HGNC:24682]
ENSG00000166128	0,48220878	2,97383968	7,88513174	0,01991993	0,17908321	RAB8B	RAB8B, member RAS oncogene family [Source:HGNC Symbol;Acc:HGNC:30273]
ENSG00000115216	0,38145333	8,39461413	7,88293027	0,01993285	0,17908921	NRBP1	nuclear receptor binding protein 1 [Source:HGNC Symbol;Acc:HGNC:7993]
ENSG00000119632	-0,6633588	1,11837524	7,87814719	0,01996097	0,17912749	IFI27L2	interferon alpha inducible protein 27 like 2 [Source:HGNC Symbol;Acc:HGNC:19753]

ENSG00000173141	-0,4102364	6,30646193	7,87699217	0,01996777	0,17912749	MRPL57	mitochondrial ribosomal protein L57 [Source:HGNC Symbol;Acc:HGNC:14514]
ENSG00000221947	-1,8355788	-1,3978985	7,87595141	0,0199739	0,17912749	XKR9	XK related 9 [Source:HGNC Symbol;Acc:HGNC:20937]
ENSG00000138768	-0,4783466	5,81919646	7,86796893	0,02002097	0,17943952	USO1	USO1 vesicle transport factor [Source:HGNC Symbol;Acc:HGNC:30904]
ENSG00000090470	0,43142516	4,23202105	7,86411029	0,02004378	0,17953377	PDCD7	programmed cell death 7 [Source:HGNC Symbol;Acc:HGNC:8767]
ENSG00000071967	1,04269137	0,517805	7,84837479	0,02013711	0,17970863	CYBRD1	cytochrome b reductase 1 [Source:HGNC Symbol;Acc:HGNC:20797]
ENSG00000108406	0,41670129	4,39848544	7,85752333	0,02008278	0,17970863	DHX40	DEAH-box helicase 40 [Source:HGNC Symbol;Acc:HGNC:18018]
ENSG00000166529	0,44260075	3,67381901	7,85035825	0,02012532	0,17970863	ZSCAN21	zinc finger and SCAN domain containing 21 [Source:HGNC Symbol;Acc:HGNC:13104]
ENSG00000167780	-0,4956396	6,35947302	7,85132634	0,02011956	0,17970863	SOAT2	sterol O-acyltransferase 2 [Source:HGNC Symbol;Acc:HGNC:11178]
ENSG00000273311	0,64316872	1,15315158	7,85357999	0,02010618	0,17970863	DGCR11	DiGeorge syndrome critical region gene 11 [Source:HGNC Symbol;Acc:HGNC:17226]
ENSG00000275152	-0,6578515	1,87866564	7,85032545	0,02012551	0,17970863	CCL16	C-C motif chemokine ligand 16 [Source:HGNC Symbol;Acc:HGNC:10614]
ENSG00000089820	-0,8182542	0,18490162	7,84586234	0,02015206	0,17973226	ARHGAP4	Rho GTPase activating protein 4 [Source:HGNC Symbol;Acc:HGNC:674]
ENSG00000151690	1,08229107	0,84701667	7,84219406	0,02017391	0,17975058	MFSD6	major facilitator superfamily domain containing 6 [Source:HGNC Symbol;Acc:HGNC:24711]
ENSG00000169738	-0,415347	7,35635079	7,84127862	0,02017937	0,17975058	DCXR	dicarbonyl and L-xylulose reductase [Source:HGNC Symbol;Acc:HGNC:18985]
ENSG00000214222	-0,6278878	1,20888132	7,83932502	0,02019103	0,17975058	TUBBP2	tubulin beta pseudogene 2 [Source:HGNC Symbol;Acc:HGNC:12415]
ENSG00000017483	-0,3906389	6,18379632	7,82246194	0,02029197	0,17983847	SLC38A5	solute carrier family 38 member 5 [Source:HGNC Symbol;Acc:HGNC:18070]
ENSG00000109689	0,4527022	3,85044068	7,82959453	0,0202492	0,17983847	STIM2	stromal interaction molecule 2 [Source:HGNC Symbol;Acc:HGNC:19205]
ENSG00000123575	0,45706841	3,80214408	7,82569291	0,02027258	0,17983847	FAM199X	family with sequence similarity 199, X-linked [Source:HGNC Symbol;Acc:HGNC:25195]
ENSG00000125821	0,42170127	5,75819004	7,81634448	0,02032874	0,17983847	DTD1	D-aminoacyl-tRNA deacylase 1 [Source:HGNC Symbol;Acc:HGNC:16219]
ENSG00000146830	0,66438832	3,48888867	7,81852883	0,0203156	0,17983847	GIGYF1	GRB10 interacting GYF protein 1 [Source:HGNC Symbol;Acc:HGNC:9126]
ENSG00000147677	-0,3752581	7,96500317	7,81698816	0,02032487	0,17983847	EIF3H	eukaryotic translation initiation factor 3 subunit H [Source:HGNC Symbol;Acc:HGNC:3273]
ENSG00000149925	-0,416265	5,39291731	7,81508714	0,02033631	0,17983847	ALDOA	aldolase, fructose-bisphosphate A [Source:HGNC Symbol;Acc:HGNC:414]
ENSG00000171222	-0,4923394	6,16041409	7,82046715	0,02030395	0,17983847	SCAND1	SCAN domain containing 1 [Source:HGNC Symbol;Acc:HGNC:10566]
ENSG00000179833	0,42358018	3,41590476	7,81786777	0,02031958	0,17983847	SERTAD2	SERTA domain containing 2 [Source:HGNC Symbol;Acc:HGNC:30784]
ENSG00000188603	1,34599052	-1,4170311	7,81751189	0,02032172	0,17983847	CLN3	CLN3 lysosomal/endosomal transmembrane protein, battenin [Source:HGNC Symbol;Acc:HGNC:2074]
ENSG00000198945	0,78129783	0,43614997	7,82472962	0,02027836	0,17983847	L3MBTL3	L3MBTL histone methyl-lysine binding protein 3 [Source:HGNC Symbol;Acc:HGNC:23035]
ENSG00000158555	0,42541947	5,45161642	7,80884252	0,02037395	0,18006233	GDPD5	glycerophosphodiester phosphodiesterase domain containing 5 [Source:HGNC Symbol;Acc:HGNC:28804]
ENSG00000184164	-0,4428107	7,23367161	7,80488017	0,02039788	0,1801648	CRELD2	cysteine rich with EGF like domains 2 [Source:HGNC Symbol;Acc:HGNC:28150]
ENSG00000278535	-0,441976	5,02179078	7,78587148	0,02051315	0,18107344	DHRS11	dehydrogenase/reductase 11 [Source:HGNC Symbol;Acc:HGNC:28639]
ENSG00000107821	-0,5372021	3,24617572	7,77821773	0,02055978	0,18137554	KAZALD1	Kazal type serine peptidase inhibitor domain 1 [Source:HGNC Symbol;Acc:HGNC:25460]
ENSG00000178715	-0,6738289	0,84827686	7,77472362	0,02058112	0,18145418	AL450998.1	ribosomal protein S16 (RPS16) pseudogene
ENSG00000142684	-0,4277044	5,7793559	7,76982642	0,02061107	0,18160862	ZNF593	zinc finger protein 593 [Source:HGNC Symbol;Acc:HGNC:30943]
ENSG00000233593	-0,6807762	1,65112208	7,76452684	0,02064354	0,18178507	LINC02609	long intergenic non-protein coding RNA 2609 [Source:HGNC Symbol;Acc:HGNC:27140]
ENSG00000051009	0,42089427	3,83626735	7,75858888	0,02067999	0,18188144	FAM160A2	family with sequence similarity 160 member A2 [Source:HGNC Symbol;Acc:HGNC:25378]
ENSG00000081177	0,41492816	4,12920069	7,74861832	0,02074138	0,18188144	EXD2	exonuclease 3'-5' domain containing 2 [Source:HGNC Symbol;Acc:HGNC:20217]
ENSG00000111752	0,74184135	1,82135752	7,74857842	0,02074163	0,18188144	PHC1	polyhomeotic homolog 1 [Source:HGNC Symbol;Acc:HGNC:3182]
ENSG00000114735	-0,4919995	3,70523821	7,7516976	0,0207224	0,18188144	HEMK1	HemK methyltransferase family member 1 [Source:HGNC Symbol;Acc:HGNC:24923]
ENSG00000135414	-0,5782528	2,0666397	7,75017604	0,02073178	0,18188144	GDF11	growth differentiation factor 11 [Source:HGNC Symbol;Acc:HGNC:4216]
ENSG00000147144	0,62263464	1,60671562	7,75837495	0,02068131	0,18188144	CCDC120	coiled-coil domain containing 120 [Source:HGNC Symbol;Acc:HGNC:28910]
ENSG00000162194	-0,7263854	1,07583472	7,75339805	0,02071193	0,18188144	LBHD1	LBH domain containing 1 [Source:HGNC Symbol;Acc:HGNC:28351]
ENSG00000010318	-0,8856855	0,0893254	7,74646344	0,02075468	0,18188671	PHF7	PHD finger protein 7 [Source:HGNC Symbol;Acc:HGNC:18458]
ENSG00000118520	1,38919307	-0,5425426	7,73114464	0,02084952	0,18228042	ARG1	arginase 1 [Source:HGNC Symbol;Acc:HGNC:663]
ENSG00000138111	0,75854683	2,68296719	7,73469577	0,02082748	0,18228042	MFSD13A	major facilitator superfamily domain containing 13A [Source:HGNC Symbol;Acc:HGNC:26196]
ENSG00000138448	0,59767345	5,85168917	7,73434151	0,02082968	0,18228042	ITGAV	integrin subunit alpha V [Source:HGNC Symbol;Acc:HGNC:6150]
ENSG00000234906	-0,5260296	1,7351466	7,73271725	0,02083976	0,18228042	APOC2	apolipoprotein C2 [Source:HGNC Symbol;Acc:HGNC:609]

ENSG00000160209	-0,4133049	7,17299268	7,72575287	0,02088302	0,18246416	PDXK	pyridoxal kinase [Source:HGNC Symbol;Acc:HGNC:8819]
ENSG00000183255	-0,3612094	7,7173553	7,71616361	0,02094278	0,18287688	PTTG1IP	PTTG1 interacting protein [Source:HGNC Symbol;Acc:HGNC:13524]
ENSG00000146677	-0,4317817	3,01590567	7,71061658	0,02097744	0,18307012	AC004453.1	ribosomal protein L32 (RPL32) pseudogene
ENSG00000028203	0,46102209	5,66388523	7,68582677	0,02113321	0,18344801	VEZT	vezatin, adherens junctions transmembrane protein [Source:HGNC Symbol;Acc:HGNC:18258]
ENSG00000113712	-0,4668247	5,70920785	7,68194106	0,02115775	0,18344801	CSNK1A1	casein kinase 1 alpha 1 [Source:HGNC Symbol;Acc:HGNC:2451]
ENSG00000119574	-0,4700562	2,29124679	7,67977894	0,02117142	0,18344801	ZBTB45	zinc finger and BTB domain containing 45 [Source:HGNC Symbol;Acc:HGNC:23715]
ENSG00000127241	-0,6218544	3,75701984	7,69681634	0,02106398	0,18344801	MASP1	mannan binding lectin serine peptidase 1 [Source:HGNC Symbol;Acc:HGNC:6901]
ENSG00000127743	-0,5456016	1,97923554	7,68108547	0,02116316	0,18344801	IL17B	interleukin 17B [Source:HGNC Symbol;Acc:HGNC:5982]
ENSG00000130830	-0,6315437	3,69630698	7,68106977	0,02116326	0,18344801	MPP1	membrane palmitoylated protein 1 [Source:HGNC Symbol;Acc:HGNC:7219]
ENSG00000133302	0,55935866	2,21861951	7,6809028	0,02116432	0,18344801	SLF1	SMC5-SMC6 complex localization factor 1 [Source:HGNC Symbol;Acc:HGNC:25408]
ENSG00000150527	0,37138883	7,69482051	7,68147062	0,02116073	0,18344801	MIA2	MIA SH3 domain ER export factor 2 [Source:HGNC Symbol;Acc:HGNC:18432]
ENSG00000161920	-0,4309083	4,42658252	7,69050241	0,02110372	0,18344801	MED11	mediator complex subunit 11 [Source:HGNC Symbol;Acc:HGNC:32687]
ENSG00000166225	0,6106924	1,80447909	7,68834271	0,02111733	0,18344801	FRS2	fibroblast growth factor receptor substrate 2 [Source:HGNC Symbol;Acc:HGNC:16971]
ENSG00000197124	0,71979046	1,04186618	7,68732341	0,02112376	0,18344801	ZNF682	zinc finger protein 682 [Source:HGNC Symbol;Acc:HGNC:28857]
ENSG00000217027	-0,7275806	0,64937114	7,69451471	0,02107845	0,18344801	TPT1P4	tumor protein, translationally-controlled 1 pseudogene 4 [Source:HGNC Symbol;Acc:HGNC:49298]
ENSG00000246263	-1,8173898	-1,3759167	7,67729338	0,02118716	0,1834755	UBR5-AS1	UBR5 antisense RNA 1 [Source:HGNC Symbol;Acc:HGNC:51661]
ENSG00000100647	0,48270426	3,0271795	7,66498979	0,02126524	0,18385939	SUSD6	sushi domain containing 6 [Source:HGNC Symbol;Acc:HGNC:19956]
ENSG00000143761	0,36652679	9,04369672	7,66571274	0,02126064	0,18385939	ARF1	ADP ribosylation factor 1 [Source:HGNC Symbol;Acc:HGNC:652]
ENSG00000167460	-0,4038982	8,41780168	7,66436057	0,02126924	0,18385939	TPM4	tropomyosin 4 [Source:HGNC Symbol;Acc:HGNC:12013]
ENSG00000287263	-1,0122484	0,58006225	7,66140841	0,02128804	0,18391302	AC008875.3	novel transcript
ENSG00000141504	-0,4131262	6,94614569	7,65660996	0,02131863	0,18406844	SAT2	spermidine/spermine N1-acetyltransferase family member 2 [Source:HGNC Symbol;Acc:HGNC:23160]
ENSG00000074211	-0,4752891	2,82472538	7,65100766	0,02135441	0,1841597	PPP2R2C	protein phosphatase 2 regulatory subunit Bgamma [Source:HGNC Symbol;Acc:HGNC:9306]
ENSG00000166165	-0,4679538	7,46676569	7,65150205	0,02135125	0,1841597	CKB	creatine kinase B [Source:HGNC Symbol;Acc:HGNC:1991]
ENSG00000100319	-0,4328492	4,50282866	7,64013593	0,02142406	0,18420133	ZMAT5	zinc finger matrin-type 5 [Source:HGNC Symbol;Acc:HGNC:28046]
ENSG00000111911	0,47296057	4,17867106	7,6389051	0,02143196	0,18420133	HINT3	histidine triad nucleotide binding protein 3 [Source:HGNC Symbol;Acc:HGNC:18468]
ENSG00000118690	0,65011524	0,87443182	7,63737025	0,02144182	0,18420133	ARMC2	armadillo repeat containing 2 [Source:HGNC Symbol;Acc:HGNC:23045]
ENSG00000163586	-0,5710486	8,85089461	7,63752364	0,02144083	0,18420133	FABP1	fatty acid binding protein 1 [Source:HGNC Symbol;Acc:HGNC:3555]
ENSG00000168286	0,44675303	5,97207979	7,63648725	0,0214475	0,18420133	THAP11	THAP domain containing 11 [Source:HGNC Symbol;Acc:HGNC:23194]
ENSG00000168528	-0,487971	4,71201118	7,63716456	0,02144314	0,18420133	SERINC2	serine incorporator 2 [Source:HGNC Symbol;Acc:HGNC:23231]
ENSG00000196663	0,54153333	3,13092895	7,64673415	0,02138175	0,18420133	TECPR2	tectonin beta-propeller repeat containing 2 [Source:HGNC Symbol;Acc:HGNC:19957]
ENSG00000084693	-0,4932979	5,66901399	7,62972926	0,02149099	0,18437457	AGBL5	ATP/GTP binding protein like 5 [Source:HGNC Symbol;Acc:HGNC:26147]
ENSG00000103064	-0,3873106	5,03231623	7,62943166	0,02149291	0,18437457	SLC7A6	solute carrier family 7 member 6 [Source:HGNC Symbol;Acc:HGNC:11064]
ENSG00000177370	-0,4454007	5,46502899	7,62713659	0,02150771	0,18439323	TIMM22	translocase of inner mitochondrial membrane 22 [Source:HGNC Symbol;Acc:HGNC:17317]
ENSG00000055957	0,5147183	2,30011515	7,62169505	0,02154284	0,18458613	ITIH1	inter-alpha-trypsin inhibitor heavy chain 1 [Source:HGNC Symbol;Acc:HGNC:6166]
ENSG00000226917	1,34010954	-0,9914421	7,61713122	0,02157236	0,18473073	LINC01276	long intergenic non-protein coding RNA 1276 [Source:HGNC Symbol;Acc:HGNC:50333]
ENSG00000239713	-1,1348034	-0,6538452	7,61286366	0,02160001	0,18485915	APOBEC3G	apolipoprotein B mRNA editing enzyme catalytic subunit 3G [Source:HGNC Symbol;Acc:HGNC:17357]
ENSG00000158109	0,35841127	6,50289874	7,60872821	0,02162685	0,18498046	TPRG1L	tumor protein p63 regulated 1 like [Source:HGNC Symbol;Acc:HGNC:27007]
ENSG00000143515	0,39424411	4,92056207	7,60445719	0,02165461	0,18500126	ATP8B2	ATPase phospholipid transporting 8B2 [Source:HGNC Symbol;Acc:HGNC:13534]
ENSG00000235453	-0,6516261	1,13295921	7,60583369	0,02164566	0,18500126	SMIM27	small integral membrane protein 27 [Source:HGNC Symbol;Acc:HGNC:31420]
ENSG00000076513	0,4404734	4,18451833	7,59417838	0,02172159	0,18546508	ANKRD13A	ankyrin repeat domain 13A [Source:HGNC Symbol;Acc:HGNC:21268]
ENSG00000105327	0,43010121	3,7708023	7,58466693	0,02178381	0,18588763	BBC3	BCL2 binding component 3 [Source:HGNC Symbol;Acc:HGNC:17868]
ENSG00000160058	0,42503738	5,99267824	7,56926089	0,02188504	0,18664247	BSDC1	BSD domain containing 1 [Source:HGNC Symbol;Acc:HGNC:25501]
ENSG00000012174	0,50629934	4,16768485	7,56224858	0,02193131	0,18689345	MBTPS2	membrane bound transcription factor peptidase, site 2 [Source:HGNC Symbol;Acc:HGNC:15455]
ENSG00000267249	1,3314133	-1,492416	7,56092536	0,02194005	0,18689345	AP005482.2	novel transcript, sense intronic CEP76

ENSG00000138336	0,52984377	2,25779669	7,55075474	0,02200741	0,18729359	TET1	tet methylcytosine dioxygenase 1 [Source:HGNC Symbol;Acc:HGNC:29484]
ENSG00000173221	-0,3572813	6,92100251	7,54996246	0,02201267	0,18729359	GLRX	glutaredoxin [Source:HGNC Symbol;Acc:HGNC:4330]
ENSG00000021826	0,4235749	4,78734037	7,53281957	0,02212681	0,18815519	CPS1	carbamoyl-phosphate synthase 1 [Source:HGNC Symbol;Acc:HGNC:2323]
ENSG00000100209	-0,4209471	4,28425718	7,52945461	0,0221493	0,18821829	HSCB	HscB mitochondrial iron-sulfur cluster cochaperone [Source:HGNC Symbol;Acc:HGNC:28913]
ENSG00000268403	-0,6941663	0,90649891	7,52785565	0,02216	0,18821829	AC132192.2	novel transcript, antisense to ZNF143
ENSG00000105835	0,45980426	5,74518224	7,51975667	0,02221428	0,18831052	NAMPT	nicotinamide phosphoribosyltransferase [Source:HGNC Symbol;Acc:HGNC:30092]
ENSG00000108479	-0,3720068	5,65430866	7,51494876	0,02224658	0,18831052	GALK1	galactokinase 1 [Source:HGNC Symbol;Acc:HGNC:4118]
ENSG00000111261	0,45285347	3,88904519	7,5178068	0,02222737	0,18831052	MANSC1	MANSC domain containing 1 [Source:HGNC Symbol;Acc:HGNC:25505]
ENSG00000134996	-0,4692789	4,83514347	7,51087708	0,02227398	0,18831052	OSTF1	osteoclast stimulating factor 1 [Source:HGNC Symbol;Acc:HGNC:8510]
ENSG00000164296	-0,6516444	1,1592611	7,51232471	0,02226423	0,18831052	TIGD6	tigger transposable element derived 6 [Source:HGNC Symbol;Acc:HGNC:18332]
ENSG00000164616	0,88005532	0,92702419	7,52063428	0,02220839	0,18831052	FBXL21P	F-box and leucine rich repeat protein 21, pseudogene [Source:HGNC Symbol;Acc:HGNC:13600]
ENSG00000188662	2,08066364	-1,1725451	7,51203082	0,02226621	0,18831052	H1-9P	H1.9 linker histone, pseudogene [Source:HGNC Symbol;Acc:HGNC:30616]
ENSG00000231574	0,78480887	0,63251977	7,51627145	0,02223769	0,18831052	LINC02015	long intergenic non-protein coding RNA 2015 [Source:HGNC Symbol;Acc:HGNC:52850]
ENSG00000266173	0,96418912	-0,2212372	7,50585427	0,02230784	0,18848768	STRADA	STE20 related adaptor alpha [Source:HGNC Symbol;Acc:HGNC:30172]
ENSG00000100330	0,4865186	4,37790038	7,49880019	0,02235549	0,18853464	MTMR3	myotubularin related protein 3 [Source:HGNC Symbol;Acc:HGNC:7451]
ENSG00000103978	0,44976186	5,93959888	7,49548678	0,02237792	0,18853464	TMEM87A	transmembrane protein 87A [Source:HGNC Symbol;Acc:HGNC:24522]
ENSG00000143643	-0,4347644	4,34440008	7,49967241	0,02234959	0,18853464	TTC13	tetratricopeptide repeat domain 13 [Source:HGNC Symbol;Acc:HGNC:26204]
ENSG00000166595	-0,3930096	6,47227026	7,49676113	0,02236929	0,18853464	CIAO2B	cytosolic iron-sulfur assembly component 2B [Source:HGNC Symbol;Acc:HGNC:24261]
ENSG00000237187	1,23781976	-0,57241	7,50056819	0,02234354	0,18853464	NR2F1-AS1	NR2F1 antisense RNA 1 [Source:HGNC Symbol;Acc:HGNC:48622]
ENSG00000100030	0,52707996	6,38289656	7,48647364	0,02243907	0,18872332	MAPK1	mitogen-activated protein kinase 1 [Source:HGNC Symbol;Acc:HGNC:6871]
ENSG00000163291	-0,4840682	2,64504906	7,48942316	0,02241904	0,18872332	PAQR3	progestin and adipoQ receptor family member 3 [Source:HGNC Symbol;Acc:HGNC:30130]
ENSG00000164953	0,69213505	1,19081948	7,4876243	0,02243125	0,18872332	TMEM67	transmembrane protein 67 [Source:HGNC Symbol;Acc:HGNC:28396]
ENSG00000102921	0,38269268	5,00199412	7,48145802	0,02247319	0,18890151	N4BP1	NEDD4 binding protein 1 [Source:HGNC Symbol;Acc:HGNC:29850]
ENSG00000007968	0,48509209	3,23175271	7,46047431	0,02261662	0,18972441	E2F2	E2F transcription factor 2 [Source:HGNC Symbol;Acc:HGNC:3114]
ENSG00000130876	-0,6656666	0,7344174	7,4593704	0,0226242	0,18972441	SLC7A10	solute carrier family 7 member 10 [Source:HGNC Symbol;Acc:HGNC:11058]
ENSG00000163467	-1,0962249	-0,0492369	7,45765832	0,02263595	0,18972441	TSACC	TSSK6 activating cochaperone [Source:HGNC Symbol;Acc:HGNC:30636]
ENSG00000163491	0,56501636	1,31383168	7,4566418	0,02264294	0,18972441	NEK10	NIMA related kinase 10 [Source:HGNC Symbol;Acc:HGNC:18592]
ENSG00000257704	-0,6322049	1,03448458	7,45856462	0,02262973	0,18972441	INAFM1	InaF motif containing 1 [Source:HGNC Symbol;Acc:HGNC:27406]
ENSG00000268104	0,5216171	4,48395772	7,45575868	0,02264901	0,18972441	SLC6A14	solute carrier family 6 member 14 [Source:HGNC Symbol;Acc:HGNC:11047]
ENSG00000269028	0,43802989	2,9708366	7,45316714	0,02266683	0,1897649	MTRNR2L12	MT-RNR2 like 12 [Source:HGNC Symbol;Acc:HGNC:37169]
ENSG00000106692	0,60789773	2,15448609	7,44868907	0,02269767	0,18991425	FKTN	fukutin [Source:HGNC Symbol;Acc:HGNC:3622]
ENSG00000012817	0,4491134	4,68156059	7,44561865	0,02271885	0,18998261	KDM5D	lysine demethylase 5D [Source:HGNC Symbol;Acc:HGNC:11115]
ENSG00000217648	-0,563113	2,22551049	7,44314311	0,02273594	0,19001676	AL136116.3	pseudogene similar to presenilin associated, rhomboid-like PARL
ENSG00000100918	-0,9603867	0,26043169	7,42919226	0,02283254	0,19030557	REC8	REC8 meiotic recombination protein [Source:HGNC Symbol;Acc:HGNC:16879]
ENSG00000143612	-0,5509521	6,89311803	7,42791946	0,02284138	0,19030557	C1orf43	chromosome 1 open reading frame 43 [Source:HGNC Symbol;Acc:HGNC:29876]
ENSG00000175265	1,10527519	0,96643388	7,42687305	0,02284865	0,19030557	GOLGA8A	golgin A8 family member A [Source:HGNC Symbol;Acc:HGNC:31972]
ENSG00000196814	0,48393048	4,68278334	7,4344319	0,0227962	0,19030557	MVB12B	multivesicular body subunit 12B [Source:HGNC Symbol;Acc:HGNC:23368]
ENSG00000203896	1,46644509	-1,7949365	7,43019506	0,02282558	0,19030557	LIME1	Lck interacting transmembrane adaptor 1 [Source:HGNC Symbol;Acc:HGNC:26016]
ENSG00000279307	-0,9930637	-0,4852556	7,43040362	0,02282414	0,19030557	NDUFAF2P1	NDUFAF2 pseudogene 1 [Source:HGNC Symbol;Acc:HGNC:54722]
ENSG00000141179	0,52582956	5,35813476	7,423797	0,02287004	0,19037517	PCTP	phosphatidylcholine transfer protein [Source:HGNC Symbol;Acc:HGNC:8752]
ENSG00000182866	-1,2167118	-0,2318339	7,41979351	0,02289791	0,19049865	LCK	LCK proto-oncogene, Src family tyrosine kinase [Source:HGNC Symbol;Acc:HGNC:6524]
ENSG00000136856	-0,375231	6,93800675	7,41451228	0,02293475	0,19069649	SLC2A8	solute carrier family 2 member 8 [Source:HGNC Symbol;Acc:HGNC:13812]
ENSG00000205212	0,83656023	0,51832948	7,40992278	0,02296682	0,1908545	CCDC144NL	CCDC144A N-terminal pseudogene [Source:HGNC Symbol;Acc:HGNC:33735]
ENSG00000101935	0,57635776	1,57303371	7,39383863	0,02307964	0,19168301	AMMECR1	AMMECR nuclear protein 1 [Source:HGNC Symbol;Acc:HGNC:467]

ENSG00000151725	-0,4855884	4,52687808	7,39190065	0,02309328	0,19168732	CENPU	centromere protein U [Source:HGNC Symbol;Acc:HGNC:21348]
ENSG00000057252	0,48979372	5,39043523	7,38544099	0,02313881	0,19181908	SOAT1	sterol O-acyltransferase 1 [Source:HGNC Symbol;Acc:HGNC:11177]
ENSG00000164111	-0,4445188	8,59128424	7,38220433	0,02316167	0,19181908	ANXA5	annexin A5 [Source:HGNC Symbol;Acc:HGNC:543]
ENSG00000182782	-1,7092908	-1,0325798	7,38375405	0,02315072	0,19181908	HCAR2	hydroxycarboxylic acid receptor 2 [Source:HGNC Symbol;Acc:HGNC:24827]
ENSG00000184986	0,58369052	2,90459276	7,38429325	0,02314692	0,19181908	TMEM121	transmembrane protein 121 [Source:HGNC Symbol;Acc:HGNC:20511]
ENSG00000081791	0,40571619	5,63750249	7,37545463	0,02320943	0,19188825	DELE1	DAP3 binding cell death enhancer 1 [Source:HGNC Symbol;Acc:HGNC:28969]
ENSG00000173272	-0,4812707	6,25491509	7,37871328	0,02318636	0,19188825	MZT2A	mitotic spindle organizing protein 2A [Source:HGNC Symbol;Acc:HGNC:33187]
ENSG00000197599	-1,1463837	-0,9607807	7,37631319	0,02320335	0,19188825	CCDC154	coiled-coil domain containing 154 [Source:HGNC Symbol;Acc:HGNC:34454]
ENSG00000153944	-0,4088964	5,33555049	7,36683247	0,02327061	0,19228526	MSI2	musashi RNA binding protein 2 [Source:HGNC Symbol;Acc:HGNC:18585]
ENSG00000136040	0,52161085	3,69761368	7,36257002	0,02330093	0,19236216	PLXNC1	plexin C1 [Source:HGNC Symbol;Acc:HGNC:9106]
ENSG00000157036	0,43341938	3,31123208	7,35997408	0,02331942	0,19236216	EXOG	exo/endonuclease G [Source:HGNC Symbol;Acc:HGNC:3347]
ENSG00000239719	-0,6137696	1,61931178	7,36129589	0,02331	0,19236216	AC004890.1	ribosomal protein L36a (RPL36A) pseudogene
ENSG00000112200	0,43657267	4,27568893	7,35612462	0,02334687	0,19247992	ZNF451	zinc finger protein 451 [Source:HGNC Symbol;Acc:HGNC:21091]
ENSG00000010319	-0,685488	3,58851743	7,35147241	0,0233801	0,19253655	SEMA3G	semaphorin 3G [Source:HGNC Symbol;Acc:HGNC:30400]
ENSG00000246523	-1,2245302	-1,0025794	7,35181779	0,02337763	0,19253655	FZD4-DT	FZD4 divergent transcript [Source:HGNC Symbol;Acc:HGNC:54219]
ENSG00000166579	0,43953857	4,94927128	7,33829722	0,02347452	0,19298774	NDEL1	nudE neurodevelopment protein 1 like 1 [Source:HGNC Symbol;Acc:HGNC:17620]
ENSG00000174652	0,55954758	3,98578792	7,339833	0,02346349	0,19298774	ZNF266	zinc finger protein 266 [Source:HGNC Symbol;Acc:HGNC:13059]
ENSG00000185900	0,80771566	3,7896189	7,34109524	0,02345443	0,19298774	POMK	protein O-mannose kinase [Source:HGNC Symbol;Acc:HGNC:26267]
ENSG00000037042	-0,4018709	3,90240496	7,33084176	0,02352815	0,19305635	TUBG2	tubulin gamma 2 [Source:HGNC Symbol;Acc:HGNC:12419]
ENSG00000143153	0,47733531	8,09986438	7,33519086	0,02349685	0,19305635	ATP1B1	ATPase Na+/K+ transporting subunit beta 1 [Source:HGNC Symbol;Acc:HGNC:804]
ENSG00000170340	0,40810587	5,29858796	7,32979141	0,02353572	0,19305635	B3GNT2	UDP-GlcNAc:betaGal beta-1,3-N-acetylglucosaminyltransferase 2 [Source:HGNC Symbol;Acc:HGNC:15629]
ENSG00000264364	0,43354835	5,22763825	7,33305224	0,02351223	0,19305635	DYNLL2	dynein light chain LC8-type 2 [Source:HGNC Symbol;Acc:HGNC:24596]
ENSG00000139263	0,45397613	3,71871539	7,32516369	0,0235691	0,19317089	LRIG3	leucine rich repeats and immunoglobulin like domains 3 [Source:HGNC Symbol;Acc:HGNC:30991]
ENSG00000175854	-0,4910806	5,15106323	7,32419074	0,02357613	0,19317089	SWI5	SWI5 homologous recombination repair protein [Source:HGNC Symbol;Acc:HGNC:31412]
ENSG00000163864	-0,5323901	1,94694609	7,31699779	0,02362815	0,19327364	NMNAT3	nicotinamide nucleotide adenylyltransferase 3 [Source:HGNC Symbol;Acc:HGNC:20989]
ENSG00000168259	0,36630589	7,51744532	7,31514307	0,02364159	0,19327364	DNAJC7	DnaJ heat shock protein family (Hsp40) member C7 [Source:HGNC Symbol;Acc:HGNC:12392]
ENSG00000186185	0,44637615	4,67583549	7,31557861	0,02363843	0,19327364	KIF18B	kinesin family member 18B [Source:HGNC Symbol;Acc:HGNC:27102]
ENSG00000267858	-0,9104599	0,09434161	7,31585864	0,0236364	0,19327364	MZF1-AS1	MZF1 antisense RNA 1 [Source:HGNC Symbol;Acc:HGNC:51271]
ENSG00000186910	-0,4706531	5,63785999	7,30962932	0,02368159	0,19349238	SERPINA11	serpin family A member 11 [Source:HGNC Symbol;Acc:HGNC:19193]
ENSG00000116353	-0,3958004	4,45836892	7,29044787	0,0238214	0,19390209	MECR	mitochondrial trans-2-enoyl-CoA reductase [Source:HGNC Symbol;Acc:HGNC:19691]
ENSG00000126562	-0,8246465	3,64368424	7,29000433	0,02382465	0,19390209	WNK4	WNK lysine deficient protein kinase 4 [Source:HGNC Symbol;Acc:HGNC:14544]
ENSG00000127328	-0,6596509	3,25169711	7,29568572	0,02378312	0,19390209	RAB3IP	RAB3A interacting protein [Source:HGNC Symbol;Acc:HGNC:16508]
ENSG00000131269	0,4412397	4,7835953	7,29282928	0,02380399	0,19390209	ABCB7	ATP binding cassette subfamily B member 7 [Source:HGNC Symbol;Acc:HGNC:48]
ENSG00000137414	-0,4276319	4,61287358	7,29816622	0,02376502	0,19390209	FAM8A1	family with sequence similarity 8 member A1 [Source:HGNC Symbol;Acc:HGNC:16372]
ENSG00000175155	0,69981203	1,55731813	7,29785536	0,02376729	0,19390209	YPEL2	yippee like 2 [Source:HGNC Symbol;Acc:HGNC:18326]
ENSG00000179364	-0,4138042	4,88805207	7,29286112	0,02380376	0,19390209	PACS2	phosphofurin acidic cluster sorting protein 2 [Source:HGNC Symbol;Acc:HGNC:23794]
ENSG00000196792	0,45463631	5,04531294	7,28701165	0,02384655	0,19397234	STRN3	striatin 3 [Source:HGNC Symbol;Acc:HGNC:15720]
ENSG00000063761	-0,4372813	3,85375573	7,28483707	0,02386249	0,19399394	ADCK1	aarF domain containing kinase 1 [Source:HGNC Symbol;Acc:HGNC:19038]
ENSG00000142230	0,35656828	8,47537678	7,27347713	0,02394595	0,19456415	SAE1	SUMO1 activating enzyme subunit 1 [Source:HGNC Symbol;Acc:HGNC:30660]
ENSG00000178445	0,4408134	7,70230926	7,26433928	0,02401334	0,19484195	GLDC	glycine decarboxylase [Source:HGNC Symbol;Acc:HGNC:4313]
ENSG00000198018	0,38575122	4,79725091	7,26618373	0,02399972	0,19484195	ENTPD7	ectonucleoside triphosphate diphosphohydrolase 7 [Source:HGNC Symbol;Acc:HGNC:19745]
ENSG00000254093	-0,4159898	3,96490936	7,26341783	0,02402015	0,19484195	PINX1	PIN2 (TERF1) interacting telomerase inhibitor 1 [Source:HGNC Symbol;Acc:HGNC:30046]
ENSG00000168140	0,48989942	5,62401454	7,25731574	0,0240653	0,19509987	VASN	vasorin [Source:HGNC Symbol;Acc:HGNC:18517]
ENSG00000126705	0,56431413	2,57515291	7,2527746	0,02409897	0,19515622	AHDC1	AT-hook DNA binding motif containing 1 [Source:HGNC Symbol;Acc:HGNC:25230]

ENSG00000145217	-0,4236921	4,3180937	7,25335239	0,02409468	0,19515622	SLC26A1	solute carrier family 26 member 1 [Source:HGNC Symbol;Acc:HGNC:10993]
ENSG00000163535	0,43387837	3,71705999	7,25039904	0,0241166	0,19519084	SGO2	shugoshin 2 [Source:HGNC Symbol;Acc:HGNC:30812]
ENSG00000177683	-0,5964648	1,82998262	7,24756482	0,02413767	0,19525313	THAP5	THAP domain containing 5 [Source:HGNC Symbol;Acc:HGNC:23188]
ENSG00000085511	0,41793997	4,44310253	7,24537013	0,02415399	0,19527705	MAP3K4	mitogen-activated protein kinase kinase kinase 4 [Source:HGNC Symbol;Acc:HGNC:6856]
ENSG00000164972	-1,5502492	-1,5265761	7,23519566	0,02422985	0,19567377	C9orf24	chromosome 9 open reading frame 24 [Source:HGNC Symbol;Acc:HGNC:19919]
ENSG00000286132	0,95743738	-0,1593654	7,23529888	0,02422908	0,19567377	AC022415.2	novel protein
ENSG00000076067	-0,6540849	1,64775253	7,22268046	0,02432356	0,19601931	RBMS2	RNA binding motif single stranded interacting protein 2 [Source:HGNC Symbol;Acc:HGNC:9909]
ENSG00000113811	-0,3611568	7,00546171	7,22265337	0,02432376	0,19601931	SELENOK	selenoprotein K [Source:HGNC Symbol;Acc:HGNC:30394]
ENSG00000164542	0,67265634	2,22423787	7,22231473	0,02432631	0,19601931	KIAA0895	KIAA0895 [Source:HGNC Symbol;Acc:HGNC:22206]
ENSG00000175110	0,39259628	7,10186333	7,22593071	0,02429918	0,19601931	MRPS22	mitochondrial ribosomal protein S22 [Source:HGNC Symbol;Acc:HGNC:14508]
ENSG00000124253	0,55424526	1,91413875	7,21538355	0,02437841	0,19605147	PCK1	phosphoenolpyruvate carboxykinase 1 [Source:HGNC Symbol;Acc:HGNC:8724]
ENSG00000138802	0,54263464	3,88828546	7,21700794	0,02436618	0,19605147	SEC24B	SEC24 homolog B, COPII coat complex component [Source:HGNC Symbol;Acc:HGNC:10704]
ENSG00000184060	-1,2276249	-1,0371394	7,21464362	0,02438398	0,19605147	ADAP2	ArfGAP with dual PH domains 2 [Source:HGNC Symbol;Acc:HGNC:16487]
ENSG00000249669	-1,2574419	-1,4687018	7,21790943	0,0243594	0,19605147	CARMN	cardiac mesoderm enhancer-associated non-coding RNA [Source:HGNC Symbol;Acc:HGNC:42872]
ENSG00000124279	0,41423476	3,27319518	7,20852674	0,02443008	0,1962062	FASTKD3	FAST kinase domains 3 [Source:HGNC Symbol;Acc:HGNC:28758]
ENSG00000166747	0,44281126	5,72827706	7,20906072	0,02442605	0,1962062	AP1G1	adaptor related protein complex 1 subunit gamma 1 [Source:HGNC Symbol;Acc:HGNC:555]
ENSG00000053900	0,46131312	3,61337864	7,2023209	0,02447697	0,19626922	ANAPC4	anaphase promoting complex subunit 4 [Source:HGNC Symbol;Acc:HGNC:19990]
ENSG00000161021	0,41793254	4,50431628	7,20215385	0,02447823	0,19626922	MAML1	mastermind like transcriptional coactivator 1 [Source:HGNC Symbol;Acc:HGNC:13632]
ENSG00000267508	0,90961627	-0,2438728	7,20490524	0,02445743	0,19626922	ZNF285	zinc finger protein 285 [Source:HGNC Symbol;Acc:HGNC:13079]
ENSG00000104635	0,44674363	9,26147141	7,19952137	0,02449816	0,19632122	SLC39A14	solute carrier family 39 member 14 [Source:HGNC Symbol;Acc:HGNC:20858]
ENSG00000164867	-0,66519	3,54621074	7,18976861	0,02457215	0,19678931	NOS3	nitric oxide synthase 3 [Source:HGNC Symbol;Acc:HGNC:7876]
ENSG00000174353	1,28259058	-0,9762972	7,18827386	0,02458351	0,19678931	STAG3L3	stromal antigen 3-like 3 (pseudogene) [Source:HGNC Symbol;Acc:HGNC:33845]
ENSG00000099290	0,53617026	4,97816421	7,18407671	0,02461545	0,19693711	WASHC2A	WASH complex subunit 2A [Source:HGNC Symbol;Acc:HGNC:23416]
ENSG00000130770	-0,4615849	5,82407191	7,17880733	0,02465563	0,19704273	ATP5IF1	ATP synthase inhibitory factor subunit 1 [Source:HGNC Symbol;Acc:HGNC:871]
ENSG00000182841	-0,4482794	3,32003549	7,17989493	0,02464733	0,19704273	RRP7BP	ribosomal RNA processing 7 homolog B, pseudogene [Source:HGNC Symbol;Acc:HGNC:30454]
ENSG00000107872	-0,4232311	3,49330976	7,17241994	0,02470444	0,19721704	FBXL15	F-box and leucine rich repeat protein 15 [Source:HGNC Symbol;Acc:HGNC:28155]
ENSG00000175287	-0,432076	3,84176247	7,17305958	0,02469955	0,19721704	PHYHD1	phytanoyl-CoA dioxygenase domain containing 1 [Source:HGNC Symbol;Acc:HGNC:23396]
ENSG00000088854	0,56424162	3,95315018	7,16835764	0,02473555	0,1972966	C20orf194	chromosome 20 open reading frame 194 [Source:HGNC Symbol;Acc:HGNC:17721]
ENSG00000116774	-0,6465414	3,35097638	7,16587548	0,02475457	0,1972966	OLFML3	olfactomedin like 3 [Source:HGNC Symbol;Acc:HGNC:24956]
ENSG00000135108	0,53024378	4,10782606	7,1658301	0,02475492	0,1972966	FBXO21	F-box protein 21 [Source:HGNC Symbol;Acc:HGNC:13592]
ENSG00000171307	-0,5601487	5,14242488	7,1572428	0,0248209	0,19771455	ZDHHC16	zinc finger DHHC-type palmitoyltransferase 16 [Source:HGNC Symbol;Acc:HGNC:20714]
ENSG00000255823	0,49151336	2,07619923	7,14629638	0,02490531	0,19806298	MTRNR2L8	MT-RNR2 like 8 [Source:HGNC Symbol;Acc:HGNC:37165]
ENSG00000285744	1,21152293	-0,5039298	7,14714713	0,02489874	0,19806298	AC083837.1	novel transcript
ENSG00000286388	-1,1650471	0,03852816	7,14765712	0,0248948	0,19806298	AC026748.7	novel transcript
ENSG00000105552	-0,8775076	1,68984405	7,12435328	0,0250756	0,19920572	BCAT2	branched chain amino acid transaminase 2 [Source:HGNC Symbol;Acc:HGNC:977]
ENSG00000114796	0,61894659	1,30140793	7,12251675	0,02508991	0,19920572	KLHL24	kelch like family member 24 [Source:HGNC Symbol;Acc:HGNC:25947]
ENSG00000171560	-0,6926976	10,4786958	7,12318547	0,0250847	0,19920572	FGA	fibrinogen alpha chain [Source:HGNC Symbol;Acc:HGNC:3661]
ENSG00000068078	0,36847571	7,33938971	7,11739772	0,02512987	0,19928354	FGFR3	fibroblast growth factor receptor 3 [Source:HGNC Symbol;Acc:HGNC:3690]
ENSG00000131845	0,64119435	2,40036195	7,11602041	0,02514064	0,19928354	ZNF304	zinc finger protein 304 [Source:HGNC Symbol;Acc:HGNC:13505]
ENSG00000198794	0,38809004	4,48557564	7,11766838	0,02512776	0,19928354	SCAMP5	secretory carrier membrane protein 5 [Source:HGNC Symbol;Acc:HGNC:30386]
ENSG00000105136	0,48725269	2,20436364	7,11392683	0,02515701	0,19929953	ZNF419	zinc finger protein 419 [Source:HGNC Symbol;Acc:HGNC:20648]
ENSG00000108848	0,38456117	6,16420788	7,10879224	0,02519722	0,19929953	LUC7L3	LUC7 like 3 pre-mRNA splicing factor [Source:HGNC Symbol;Acc:HGNC:24309]
ENSG00000110721	0,36504667	7,1235211	7,1090964	0,02519484	0,19929953	CHKA	choline kinase alpha [Source:HGNC Symbol;Acc:HGNC:1937]
ENSG00000213169	-0,5178511	1,89081295	7,10888156	0,02519652	0,19929953	AC069218.1	ribosomal protein L8 (RPL8) pseudogene

ENSG00000198720	0,5467982	2,41250192	7,10493574	0,02522748	0,19943087	ANKRD13B	ankyrin repeat domain 13B [Source:HGNC Symbol;Acc:HGNC:26363]
ENSG00000258920	-0,6575451	1,06267611	7,10253315	0,02524635	0,19947211	FOXN3-AS1	FOXN3 antisense RNA 1 [Source:HGNC Symbol;Acc:HGNC:44290]
ENSG00000189283	-0,618149	2,1943942	7,09497411	0,02530584	0,1997261	FHIT	fragile histidine triad diadenosine triphosphatase [Source:HGNC Symbol;Acc:HGNC:3701]
ENSG00000198814	-0,5044833	5,82673823	7,09497508	0,02530583	0,1997261	GK	glycerol kinase [Source:HGNC Symbol;Acc:HGNC:4289]
ENSG00000087266	-0,5360228	4,31160532	7,09079507	0,0253388	0,19987827	SH3BP2	SH3 domain binding protein 2 [Source:HGNC Symbol;Acc:HGNC:10825]
ENSG00000117859	0,37026669	6,47601753	7,08859183	0,0253562	0,19990758	OSBPL9	oxysterol binding protein like 9 [Source:HGNC Symbol;Acc:HGNC:16386]
ENSG00000125826	-0,3660723	7,45744317	7,0829106	0,02540113	0,19991391	RBCK1	RANBP2-type and C3HC4-type zinc finger containing 1 [Source:HGNC Symbol;Acc:HGNC:15864]
ENSG00000130529	-0,8641867	1,3896355	7,07997532	0,02542439	0,19991391	TRPM4	transient receptor potential cation channel subfamily M member 4 [Source:HGNC Symbol;Acc:HGNC:17993]
ENSG00000149480	0,39480092	6,50184586	7,08295665	0,02540077	0,19991391	MTA2	metastasis associated 1 family member 2 [Source:HGNC Symbol;Acc:HGNC:7411]
ENSG00000169976	-0,4296372	7,95623954	7,0818606	0,02540945	0,19991391	SF3B5	splicing factor 3b subunit 5 [Source:HGNC Symbol;Acc:HGNC:21083]
ENSG00000205581	-0,4840808	7,15333503	7,07984455	0,02542543	0,19991391	HMGN1	high mobility group nucleosome binding domain 1 [Source:HGNC Symbol;Acc:HGNC:4984]
ENSG00000196459	0,53158953	1,43088612	7,07128747	0,02549337	0,20034032	TRAPPC2	trafficking protein particle complex 2 [Source:HGNC Symbol;Acc:HGNC:23068]
ENSG00000012124	-1,012393	0,51210295	7,06562553	0,02553845	0,2004867	CD22	CD22 molecule [Source:HGNC Symbol;Acc:HGNC:1643]
ENSG00000111602	0,39012628	5,91598103	7,06188927	0,02556825	0,2004867	TIMELESS	timeless circadian regulator [Source:HGNC Symbol;Acc:HGNC:11813]
ENSG00000126787	0,38429226	4,97128358	7,06189587	0,0255682	0,2004867	DLGAP5	DLG associated protein 5 [Source:HGNC Symbol;Acc:HGNC:16864]
ENSG00000133997	0,37428281	5,38818266	7,05980589	0,02558489	0,2004867	MED6	mediator complex subunit 6 [Source:HGNC Symbol;Acc:HGNC:19970]
ENSG00000138433	0,39346718	5,17599781	7,0466312	0,0256904	0,2004867	CIR1	corepressor interacting with RBPJ, CIR1 [Source:HGNC Symbol;Acc:HGNC:24217]
ENSG00000157303	-0,653203	1,19500807	7,05331389	0,02563682	0,2004867	SUSD3	sushi domain containing 3 [Source:HGNC Symbol;Acc:HGNC:28391]
ENSG00000163251	0,34772399	5,85986822	7,04667087	0,02569009	0,2004867	FZD5	frizzled class receptor 5 [Source:HGNC Symbol;Acc:HGNC:4043]
ENSG00000171612	-0,5437114	6,50995074	7,04924367	0,02566944	0,2004867	SLC25A33	solute carrier family 25 member 33 [Source:HGNC Symbol;Acc:HGNC:29681]
ENSG00000175283	-0,379489	5,39562815	7,05404936	0,02563093	0,2004867	DOLK	dolichol kinase [Source:HGNC Symbol;Acc:HGNC:23406]
ENSG00000183779	-0,4363916	3,01899483	7,04953022	0,02566714	0,2004867	ZNF703	zinc finger protein 703 [Source:HGNC Symbol;Acc:HGNC:25883]
ENSG00000227638	-0,8898123	-0,2266502	7,04898297	0,02567153	0,2004867	HNRNPA1P14	heterogeneous nuclear ribonucleoprotein A1 pseudogene 14 [Source:HGNC Symbol;Acc:HGNC:39132]
ENSG00000250321	-0,6193072	1,03146232	7,05739338	0,02560417	0,2004867	AC079140.2	pseudogene similar to part of ribosomal protein S17 (RPS17)
ENSG00000258867	-0,7591994	1,50678011	7,06056506	0,02557883	0,2004867	LINC01146	long intergenic non-protein coding RNA 1146 [Source:HGNC Symbol;Acc:HGNC:49467]
ENSG00000028839	0,61784067	2,90705427	7,04410592	0,02571069	0,20053789	TBPL1	TATA-box binding protein like 1 [Source:HGNC Symbol;Acc:HGNC:11589]
ENSG00000006744	0,54356928	6,90861395	7,03644921	0,02577232	0,20059019	ELAC2	elaC ribonuclease Z 2 [Source:HGNC Symbol;Acc:HGNC:14198]
ENSG00000121653	0,44333824	2,88895953	7,03966411	0,02574642	0,20059019	MAPK8IP1	mitogen-activated protein kinase 8 interacting protein 1 [Source:HGNC Symbol;Acc:HGNC:6882]
ENSG00000159210	-0,3622636	7,58855863	7,03922385	0,02574996	0,20059019	SNF8	SNF8 subunit of ESCRT-II [Source:HGNC Symbol;Acc:HGNC:17028]
ENSG00000185761	-1,1747448	-0,4804966	7,03693248	0,02576842	0,20059019	ADAMTSL5	ADAMTS like 5 [Source:HGNC Symbol;Acc:HGNC:27912]
ENSG00000217130	-0,5210607	1,60824512	7,03396682	0,02579234	0,20063911	AL139100.1	ribosomal protein S10 (RPS10) pseudogene
ENSG00000078070	0,43261695	4,88551836	7,03206667	0,02580767	0,20065157	MCCC1	methylcrotonoyl-CoA carboxylase 1 [Source:HGNC Symbol;Acc:HGNC:6936]
ENSG00000100116	-0,3840108	6,18449394	7,02954204	0,02582807	0,20070333	GCAT	glycine C-acetyltransferase [Source:HGNC Symbol;Acc:HGNC:4188]
ENSG00000013563	-0,5346561	2,54930781	7,01310352	0,02596135	0,20083227	DNASE1L1	deoxyribonuclease 1 like 1 [Source:HGNC Symbol;Acc:HGNC:2957]
ENSG00000076248	0,37160261	7,05752583	7,02513274	0,02586374	0,20083227	UNG	uracil DNA glycosylase [Source:HGNC Symbol;Acc:HGNC:12572]
ENSG00000101311	0,3806132	4,62470091	7,01403983	0,02595374	0,20083227	FERMT1	fermitin family member 1 [Source:HGNC Symbol;Acc:HGNC:15889]
ENSG00000102030	-0,3607291	5,69190051	7,00703701	0,02601075	0,20083227	NAA10	N-alpha-acetyltransferase 10, NatA catalytic subunit [Source:HGNC Symbol;Acc:HGNC:18704]
ENSG00000122970	0,47354061	2,5432047	7,00785276	0,0260041	0,20083227	IFT81	intraflagellar transport 81 [Source:HGNC Symbol;Acc:HGNC:14313]
ENSG00000148468	0,3687265	5,85253249	7,00623258	0,02601731	0,20083227	FAM171A1	family with sequence similarity 171 member A1 [Source:HGNC Symbol;Acc:HGNC:23522]
ENSG00000151746	-0,6000733	1,41979139	7,01425784	0,02595197	0,20083227	BICD1	BICD cargo adaptor 1 [Source:HGNC Symbol;Acc:HGNC:1049]
ENSG00000158220	0,5072137	2,18913738	7,00892106	0,0259954	0,20083227	ESYT3	extended synaptotagmin 3 [Source:HGNC Symbol;Acc:HGNC:24295]
ENSG00000170482	-0,6102284	2,66766198	7,02249172	0,02588513	0,20083227	SLC23A1	solute carrier family 23 member 1 [Source:HGNC Symbol;Acc:HGNC:10974]
ENSG00000173409	0,49722818	4,77377724	7,0102625	0,02598447	0,20083227	ARV1	ARV1 homolog, fatty acid homeostasis modulator [Source:HGNC Symbol;Acc:HGNC:29561]
ENSG00000196437	0,79615609	1,78865834	7,0054893	0,02602338	0,20083227	ZNF569	zinc finger protein 569 [Source:HGNC Symbol;Acc:HGNC:24737]

ENSG00000222041	-0,5022685	3,1144983	7,0179782	0,02592174	0,20083227	CYTOR	cytoskeleton regulator RNA [Source:HGNC Symbol;Acc:HGNC:28717]
ENSG00000243517	-0,4413196	2,73376707	7,01323997	0,02596024	0,20083227	AC024940.2	ribosomal protein L13a (RPL13A) pseudogene
ENSG00000096093	0,43835977	3,03232772	7,0013717	0,02605699	0,20098553	EFHC1	EF-hand domain containing 1 [Source:HGNC Symbol;Acc:HGNC:16406]
ENSG00000204922	-0,438311	5,21783719	6,99672749	0,02609497	0,20117228	UQCC3	ubiquinol-cytochrome c reductase complex assembly factor 3 [Source:HGNC Symbol;Acc:HGNC:34399]
ENSG00000167797	-0,4886013	5,76521364	6,99401766	0,02611717	0,20123717	CDK2AP2	cyclin dependent kinase 2 associated protein 2 [Source:HGNC Symbol;Acc:HGNC:30833]
ENSG00000261468	1,2799723	-1,5846391	6,99136592	0,02613891	0,20129851	AC096921.2	novel transcript
ENSG00000100503	-0,4058159	3,74792695	6,98944036	0,02615471	0,20131406	NIN	ninein [Source:HGNC Symbol;Acc:HGNC:14906]
ENSG00000167685	-0,4336888	4,80833752	6,98668942	0,0261773	0,20138186	ZNF444	zinc finger protein 444 [Source:HGNC Symbol;Acc:HGNC:16052]
ENSG00000130313	-0,386758	5,82566405	6,98276757	0,02620955	0,20141894	PGLS	6-phosphogluconolactonase [Source:HGNC Symbol;Acc:HGNC:8903]
ENSG00000179941	0,50301714	2,56516697	6,98239754	0,0262126	0,20141894	BBS10	Bardet-Biedl syndrome 10 [Source:HGNC Symbol;Acc:HGNC:26291]
ENSG00000262814	-0,4581974	4,98023963	6,98107543	0,02622348	0,20141894	MRPL12	mitochondrial ribosomal protein L12 [Source:HGNC Symbol;Acc:HGNC:10378]
ENSG00000188735	-0,4208597	4,38166509	6,9767657	0,026259	0,20158579	TMEM120B	transmembrane protein 120B [Source:HGNC Symbol;Acc:HGNC:32008]
ENSG00000148400	1,10845073	-0,5781252	6,97397613	0,02628203	0,20162825	NOTCH1	notch receptor 1 [Source:HGNC Symbol;Acc:HGNC:7881]
ENSG00000183888	-0,4167009	5,23506252	6,97275195	0,02629214	0,20162825	SRARP	steroid receptor associated and regulated protein [Source:HGNC Symbol;Acc:HGNC:28339]
ENSG00000013523	0,50429091	4,43849137	6,9609803	0,02638961	0,20192175	ANGEL1	angel homolog 1 [Source:HGNC Symbol;Acc:HGNC:19961]
ENSG00000087116	-0,6010724	0,93838123	6,96433533	0,02636179	0,20192175	ADAMTS2	ADAM metallopeptidase with thrombospondin type 1 motif 2 [Source:HGNC Symbol;Acc:HGNC:218]
ENSG00000108528	-0,3929552	6,53739127	6,96008421	0,02639705	0,20192175	SLC25A11	solute carrier family 25 member 11 [Source:HGNC Symbol;Acc:HGNC:10981]
ENSG00000108582	0,7004078	6,7814741	6,96110747	0,02638856	0,20192175	CPD	carboxypeptidase D [Source:HGNC Symbol;Acc:HGNC:2301]
ENSG00000185787	0,36982831	8,27407951	6,95978741	0,02639952	0,20192175	MORF4L1	mortality factor 4 like 1 [Source:HGNC Symbol;Acc:HGNC:16989]
ENSG00000141646	0,4255713	4,34331473	6,9430914	0,0265386	0,20281414	SMAD4	SMAD family member 4 [Source:HGNC Symbol;Acc:HGNC:6770]
ENSG00000168438	0,40969852	4,02838715	6,9424508	0,02654396	0,20281414	CDC40	cell division cycle 40 [Source:HGNC Symbol;Acc:HGNC:17350]
ENSG00000257923	-0,4078705	4,9714476	6,93898298	0,02657296	0,20292965	CUX1	cut like homeobox 1 [Source:HGNC Symbol;Acc:HGNC:2557]
ENSG00000204237	-0,4386305	4,49015801	6,93372798	0,026617	0,20295076	OXLD1	oxidoreductase like domain containing 1 [Source:HGNC Symbol;Acc:HGNC:27901]
ENSG00000253958	-0,9416248	-0,3300229	6,9336794	0,0266174	0,20295076	CLDN23	claudin 23 [Source:HGNC Symbol;Acc:HGNC:17591]
ENSG00000278619	-0,4206048	4,15326735	6,93534311	0,02660345	0,20295076	MRM1	mitochondrial rRNA methyltransferase 1 [Source:HGNC Symbol;Acc:HGNC:26202]
ENSG00000099330	-0,4669109	4,03499147	6,93179301	0,02663323	0,20296552	OCEL1	occludin/ELL domain containing 1 [Source:HGNC Symbol;Acc:HGNC:26221]
ENSG00000198624	-0,4522965	5,2342523	6,92847293	0,02666112	0,20296629	CCDC69	coiled-coil domain containing 69 [Source:HGNC Symbol;Acc:HGNC:24487]
ENSG00000215210	-1,154219	-0,4170973	6,92850477	0,02666085	0,20296629	RBMXP2	RBMX pseudogene 2 [Source:HGNC Symbol;Acc:HGNC:23284]
ENSG00000012504	-0,5576598	5,19195759	6,92624375	0,02667987	0,20300321	NR1H4	nuclear receptor subfamily 1 group H member 4 [Source:HGNC Symbol;Acc:HGNC:7967]
ENSG00000073169	-0,4633773	3,85627471	6,92443239	0,02669511	0,20301345	SELENOO	selenoprotein O [Source:HGNC Symbol;Acc:HGNC:30395]
ENSG00000068354	-0,4542873	2,82554971	6,91256136	0,02679527	0,20302703	TBC1D25	TBC1 domain family member 25 [Source:HGNC Symbol;Acc:HGNC:8092]
ENSG00000092931	-0,4112797	4,39973068	6,90776586	0,02683587	0,20302703	MFSD11	major facilitator superfamily domain containing 11 [Source:HGNC Symbol;Acc:HGNC:25458]
ENSG00000103111	0,38135021	4,85407459	6,90888303	0,0268264	0,20302703	MON1B	MON1 homolog B, secretory trafficking associated [Source:HGNC Symbol;Acc:HGNC:25020]
ENSG00000133315	-0,4027072	4,16358768	6,92201184	0,0267155	0,20302703	MACROD1	mono-ADP ribosylhydrolase 1 [Source:HGNC Symbol;Acc:HGNC:29598]
ENSG00000148291	-0,4629557	4,04784179	6,92022504	0,02673056	0,20302703	SURF2	surfeit 2 [Source:HGNC Symbol;Acc:HGNC:11475]
ENSG00000151208	0,52482994	5,55300766	6,91482795	0,02677611	0,20302703	DLG5	discs large MAGUK scaffold protein 5 [Source:HGNC Symbol;Acc:HGNC:2904]
ENSG00000166398	0,59061727	2,98097675	6,90894142	0,02682591	0,20302703	GARRE1	granule associated Rac and RHOG effector 1 [Source:HGNC Symbol;Acc:HGNC:29016]
ENSG00000178295	0,42295564	2,70541319	6,91181621	0,02680158	0,20302703	GEN1	GEN1 Holliday junction 5' flap endonuclease [Source:HGNC Symbol;Acc:HGNC:26881]
ENSG00000196862	1,01170511	-0,7132359	6,91587186	0,02676729	0,20302703	RGPD4	RANBP2 like and GRIP domain containing 4 [Source:HGNC Symbol;Acc:HGNC:32417]
ENSG00000270157	1,37934454	-1,2438122	6,91121339	0,02680668	0,20302703	AC004918.3	novel transcript
ENSG00000145331	-0,4867406	2,6233809	6,90307779	0,02687563	0,20310159	TRMT10A	tRNA methyltransferase 10A [Source:HGNC Symbol;Acc:HGNC:28403]
ENSG00000156931	0,41366758	3,34141547	6,90106783	0,0268927	0,20310159	VPS8	VPS8 subunit of CORVET complex [Source:HGNC Symbol;Acc:HGNC:29122]
ENSG00000167113	-0,376027	4,85572258	6,90481117	0,02686092	0,20310159	COQ4	coenzyme Q4 [Source:HGNC Symbol;Acc:HGNC:19693]
ENSG00000172115	-0,3751793	7,38292213	6,90005117	0,02690133	0,20310159	CYCS	cytochrome c, somatic [Source:HGNC Symbol;Acc:HGNC:19986]

ENSG00000155893	0,52090187	2,54450471	6,88978841	0,02698873	0,20322693	PXYLP1	2-phosphoxylose phosphatase 1 [Source:HGNC Symbol;Acc:HGNC:26303]
ENSG00000160075	0,44716076	6,93794242	6,89136606	0,02697527	0,20322693	SSU72	SSU72 homolog, RNA polymerase II CTD phosphatase [Source:HGNC Symbol;Acc:HGNC:25016]
ENSG00000168497	-0,4607159	3,94118797	6,89312245	0,0269603	0,20322693	CAVIN2	caveolae associated protein 2 [Source:HGNC Symbol;Acc:HGNC:10690]
ENSG00000169856	0,57067493	1,26399463	6,89303747	0,02696102	0,20322693	ONECUT1	one cut homeobox 1 [Source:HGNC Symbol;Acc:HGNC:8138]
ENSG00000170385	0,37806992	5,99956293	6,89476283	0,02694632	0,20322693	SLC30A1	solute carrier family 30 member 1 [Source:HGNC Symbol;Acc:HGNC:11012]
ENSG00000198753	1,07150757	1,31740862	6,88830343	0,0270014	0,20322693	PLXNB3	plexin B3 [Source:HGNC Symbol;Acc:HGNC:9105]
ENSG00000116213	0,37674552	5,3789922	6,88552636	0,02702512	0,20326201	WRAP73	WD repeat containing, antisense to TP73 [Source:HGNC Symbol;Acc:HGNC:12759]
ENSG00000164124	0,42058118	3,27772908	6,884501	0,02703389	0,20326201	TMEM144	transmembrane protein 144 [Source:HGNC Symbol;Acc:HGNC:25633]
ENSG00000036672	0,51846839	2,87838571	6,8793338	0,02707812	0,20348981	USP2	ubiquitin specific peptidase 2 [Source:HGNC Symbol;Acc:HGNC:12618]
ENSG00000173531	-0,4751547	5,96318743	6,87633725	0,0271038	0,20357813	MST1	macrophage stimulating 1 [Source:HGNC Symbol;Acc:HGNC:7380]
ENSG00000151779	0,54451102	5,41273966	6,87413677	0,02712269	0,20361527	NBAS	NBAS subunit of NRZ tethering complex [Source:HGNC Symbol;Acc:HGNC:15625]
ENSG00000066468	1,25784401	0,58603761	6,86742667	0,02718037	0,2039435	FGFR2	fibroblast growth factor receptor 2 [Source:HGNC Symbol;Acc:HGNC:3689]
ENSG00000004897	0,43489257	5,51270764	6,85651724	0,02727447	0,20427416	CDC27	cell division cycle 27 [Source:HGNC Symbol;Acc:HGNC:1728]
ENSG00000125995	-0,4383674	7,7785184	6,84937477	0,0273363	0,20427416	ROMO1	reactive oxygen species modulator 1 [Source:HGNC Symbol;Acc:HGNC:16185]
ENSG00000132323	0,3742803	5,888872	6,85685822	0,02727152	0,20427416	ILKAP	ILK associated serine/threonine phosphatase [Source:HGNC Symbol;Acc:HGNC:15566]
ENSG00000142405	-1,424169	-1,2141206	6,85864487	0,02725609	0,20427416	NLRP12	NLR family pyrin domain containing 12 [Source:HGNC Symbol;Acc:HGNC:22938]
ENSG00000170835	-0,6244534	1,08866553	6,84963581	0,02733404	0,20427416	CEL	carboxyl ester lipase [Source:HGNC Symbol;Acc:HGNC:1848]
ENSG00000178234	0,57653596	4,18896777	6,85211764	0,02731254	0,20427416	GALNT11	polypeptide N-acetylgalactosaminyltransferase 11 [Source:HGNC Symbol;Acc:HGNC:19875]
ENSG00000185338	0,40175408	3,23916522	6,8541159	0,02729524	0,20427416	SOCS1	suppressor of cytokine signaling 1 [Source:HGNC Symbol;Acc:HGNC:19383]
ENSG00000198075	-0,9672595	-0,7200767	6,85496044	0,02728793	0,20427416	SULT1C4	sulfotransferase family 1C member 4 [Source:HGNC Symbol;Acc:HGNC:11457]
ENSG00000054690	-0,5985801	1,83625867	6,83642237	0,02744886	0,20428323	PLEKHH1	pleckstrin homology, MyTH4 and FERM domain containing H1 [Source:HGNC Symbol;Acc:HGNC:17733]
ENSG00000064886	1,03477231	0,22441567	6,84072493	0,02741141	0,20428323	CHI3L2	chitinase 3 like 2 [Source:HGNC Symbol;Acc:HGNC:1933]
ENSG00000103591	0,37711833	5,45981857	6,83889789	0,0274273	0,20428323	AAGAB	alpha and gamma adaptin binding protein [Source:HGNC Symbol;Acc:HGNC:25662]
ENSG00000132196	-0,3747205	6,58467437	6,83636293	0,02744938	0,20428323	HSD17B7	hydroxysteroid 17-beta dehydrogenase 7 [Source:HGNC Symbol;Acc:HGNC:5215]
ENSG00000168763	0,41990204	6,68110422	6,83824146	0,02743302	0,20428323	CNNM3	cyclin and CBS domain divalent metal cation transport mediator 3 [Source:HGNC Symbol;Acc:HGNC:104]
ENSG00000183401	-1,4024619	-0,8065396	6,84401786	0,02738278	0,20428323	CCDC159	coiled-coil domain containing 159 [Source:HGNC Symbol;Acc:HGNC:26996]
ENSG00000225377	-0,4912653	1,79170731	6,84152013	0,02740449	0,20428323	NRSN2-AS1	NRSN2 antisense RNA 1 [Source:HGNC Symbol;Acc:HGNC:51222]
ENSG00000243649	-0,6349026	2,74675384	6,84589167	0,02736651	0,20428323	CFB	complement factor B [Source:HGNC Symbol;Acc:HGNC:1037]
ENSG00000188176	1,40085555	-0,5587125	6,83227913	0,02748499	0,2044441	SMTNL2	smoothelin like 2 [Source:HGNC Symbol;Acc:HGNC:24764]
ENSG00000085274	0,4831916	3,31230191	6,82245946	0,02757085	0,20487411	MYNN	myoneurin [Source:HGNC Symbol;Acc:HGNC:14955]
ENSG00000196876	0,96057385	-0,0806472	6,82318006	0,02756454	0,20487411	SCN8A	sodium voltage-gated channel alpha subunit 8 [Source:HGNC Symbol;Acc:HGNC:10596]
ENSG00000161551	0,54797059	1,60294107	6,81997835	0,02759259	0,2049268	ZNF577	zinc finger protein 577 [Source:HGNC Symbol;Acc:HGNC:28673]
ENSG00000197238	-1,1389977	-0,6654202	6,81845072	0,02760599	0,2049268	H4C8	H4 clustered histone 8 [Source:HGNC Symbol;Acc:HGNC:4788]
ENSG00000118804	-0,8618469	0,34294709	6,81424056	0,02764296	0,20494971	STBD1	starch binding domain 1 [Source:HGNC Symbol;Acc:HGNC:24854]
ENSG00000126353	-1,3239863	-1,8487671	6,81333427	0,02765093	0,20494971	CCR7	C-C motif chemokine receptor 7 [Source:HGNC Symbol;Acc:HGNC:1608]
ENSG00000196455	0,48149555	4,59026631	6,81330757	0,02765117	0,20494971	PIK3R4	phosphoinositide-3-kinase regulatory subunit 4 [Source:HGNC Symbol;Acc:HGNC:8982]
ENSG00000215375	-0,7091048	2,47238027	6,80540061	0,02772079	0,20536157	MYL5	myosin light chain 5 [Source:HGNC Symbol;Acc:HGNC:7586]
ENSG00000078900	0,68430064	0,8029687	6,80100919	0,02775955	0,20543388	TP73	tumor protein p73 [Source:HGNC Symbol;Acc:HGNC:12003]
ENSG00000107968	0,49658666	3,13288409	6,7979269	0,0277868	0,20543388	MAP3K8	mitogen-activated protein kinase kinase kinase 8 [Source:HGNC Symbol;Acc:HGNC:6860]
ENSG00000232346	-0,4744042	2,33103518	6,79949442	0,02777294	0,20543388	Z74021.1	ribosomal protein S17 (RPS17) pseudogene
ENSG00000271601	0,39126066	4,05023435	6,79801643	0,02778601	0,20543388	LIX1L	limb and CNS expressed 1 like [Source:HGNC Symbol;Acc:HGNC:28715]
ENSG00000213096	0,85944032	0,61495631	6,79254472	0,02783446	0,20568212	ZNF254	zinc finger protein 254 [Source:HGNC Symbol;Acc:HGNC:13047]
ENSG00000272973	1,05536569	-1,1032756	6,78853081	0,02787006	0,20584112	AP000350.5	novel transcript, antisense to SLC2A11
ENSG00000054277	0,62780582	4,14903054	6,77987447	0,02794704	0,20611683	OPN3	opsin 3 [Source:HGNC Symbol;Acc:HGNC:14007]

ENSG00000143614	0,78816333	3,23864727	6,77957408	0,02794972	0,20611683	GATAD2B	GATA zinc finger domain containing 2B [Source:HGNC Symbol;Acc:HGNC:30778]
ENSG00000164506	0,40331252	3,60663286	6,78109817	0,02793614	0,20611683	STXBP5	syntaxin binding protein 5 [Source:HGNC Symbol;Acc:HGNC:19665]
ENSG00000184319	-0,4569765	2,60328679	6,77389208	0,0280004	0,20638638	RPL23AP82	ribosomal protein L23a pseudogene 82 [Source:HGNC Symbol;Acc:HGNC:33730]
ENSG00000249565	-0,5969346	1,10946001	6,76773639	0,02805543	0,20668773	SERBP1P5	SERPINE1 mRNA binding protein 1 pseudogene 5 [Source:HGNC Symbol;Acc:HGNC:44632]
ENSG00000086289	0,37250281	5,5384171	6,7623801	0,02810342	0,20671388	EPDR1	ependymin related 1 [Source:HGNC Symbol;Acc:HGNC:17572]
ENSG00000164251	-0,6001593	5,8899573	6,76078299	0,02811775	0,20671388	F2RL1	F2R like trypsin receptor 1 [Source:HGNC Symbol;Acc:HGNC:3538]
ENSG00000177685	-0,5742511	3,17270173	6,76283028	0,02809939	0,20671388	CRACR2B	calcium release activated channel regulator 2B [Source:HGNC Symbol;Acc:HGNC:28703]
ENSG00000182606	0,372611	4,82081327	6,76167139	0,02810978	0,20671388	TRAK1	trafficking kinesin protein 1 [Source:HGNC Symbol;Acc:HGNC:29947]
ENSG00000224081	1,11475151	-1,0610749	6,75944913	0,02812973	0,20671388	SLC44A3-AS1	SLC44A3 antisense RNA 1 [Source:HGNC Symbol;Acc:HGNC:49057]
ENSG00000124795	-0,4630166	6,57238554	6,75279794	0,02818953	0,20704923	DEK	DEK proto-oncogene [Source:HGNC Symbol;Acc:HGNC:2768]
ENSG00000025708	-0,552114	1,5856763	6,74450845	0,02826429	0,20749399	TYMP	thymidine phosphorylase [Source:HGNC Symbol;Acc:HGNC:3148]
ENSG00000065154	-0,5039237	5,9806646	6,73818107	0,02832152	0,20755975	OAT	ornithine aminotransferase [Source:HGNC Symbol;Acc:HGNC:8091]
ENSG00000105223	-0,3394516	6,03471628	6,74157701	0,02829079	0,20755975	PLD3	phospholipase D family member 3 [Source:HGNC Symbol;Acc:HGNC:17158]
ENSG00000134461	-0,4744149	3,36709275	6,73566809	0,02834429	0,20755975	ANKRD16	ankyrin repeat domain 16 [Source:HGNC Symbol;Acc:HGNC:23471]
ENSG00000205208	-0,7529936	2,3265536	6,73812426	0,02832203	0,20755975	C4orf46	chromosome 4 open reading frame 46 [Source:HGNC Symbol;Acc:HGNC:27320]
ENSG00000230979	-0,3915318	3,33247267	6,73591306	0,02834207	0,20755975	AC079250.1	ribosomal protein L18a (RPL18A) pseudogene
ENSG00000115902	0,5512789	6,5774048	6,7320164	0,02837741	0,20769821	SLC1A4	solute carrier family 1 member 4 [Source:HGNC Symbol;Acc:HGNC:10942]
ENSG00000008394	-0,4735796	8,01100382	6,72733067	0,02841999	0,20790567	MGST1	microsomal glutathione S-transferase 1 [Source:HGNC Symbol;Acc:HGNC:7061]
ENSG00000198131	0,52334677	2,79075331	6,7243144	0,02844744	0,2080023	ZNF544	zinc finger protein 544 [Source:HGNC Symbol;Acc:HGNC:16759]
ENSG00000198563	0,39657236	4,7124579	6,72037465	0,02848334	0,20816062	DDX39B	DExD-box helicase 39B [Source:HGNC Symbol;Acc:HGNC:13917]
ENSG00000065361	0,4492406	7,36302133	6,71162472	0,02856327	0,20818482	ERBB3	erb-b2 receptor tyrosine kinase 3 [Source:HGNC Symbol;Acc:HGNC:3431]
ENSG00000122299	-0,3741274	5,47782487	6,70773999	0,02859885	0,20818482	ZC3H7A	zinc finger CCCH-type containing 7A [Source:HGNC Symbol;Acc:HGNC:30959]
ENSG00000138780	0,39919348	3,93164983	6,70527893	0,02862142	0,20818482	GSTCD	glutathione S-transferase C-terminal domain containing [Source:HGNC Symbol;Acc:HGNC:25806]
ENSG00000139722	0,40374415	4,21088672	6,70443574	0,02862915	0,20818482	VPS37B	VPS37B subunit of ESCRT-I [Source:HGNC Symbol;Acc:HGNC:25754]
ENSG00000165246	0,4170317	2,90258747	6,71150577	0,02856436	0,20818482	NLGN4Y	neuroligin 4 Y-linked [Source:HGNC Symbol;Acc:HGNC:15529]
ENSG00000165813	-0,7395676	1,17925877	6,71839119	0,02850143	0,20818482	CCDC186	coiled-coil domain containing 186 [Source:HGNC Symbol;Acc:HGNC:24349]
ENSG00000170855	-0,571845	5,85109957	6,70445514	0,02862898	0,20818482	TRIAP1	TP53 regulated inhibitor of apoptosis 1 [Source:HGNC Symbol;Acc:HGNC:26937]
ENSG00000172572	0,85054986	-0,1350434	6,70680604	0,02860741	0,20818482	PDE3A	phosphodiesterase 3A [Source:HGNC Symbol;Acc:HGNC:8778]
ENSG00000228695	-0,6041752	1,13160569	6,7105875	0,02857277	0,20818482	CES1P1	carboxylesterase 1 pseudogene 1 [Source:HGNC Symbol;Acc:HGNC:18546]
ENSG00000260267	-0,5643464	1,46787754	6,71252656	0,02855502	0,20818482	AC026471.1	novel transcript, antisense to ARMC5
ENSG00000158747	0,44845537	4,05132195	6,70222973	0,02864941	0,20822846	NBL1	NBL1, DAN family BMP antagonist [Source:HGNC Symbol;Acc:HGNC:7650]
ENSG00000134294	0,44535632	8,02191169	6,69832554	0,0286853	0,20835023	SLC38A2	solute carrier family 38 member 2 [Source:HGNC Symbol;Acc:HGNC:13448]
ENSG00000236058	-0,641406	1,49194511	6,69730523	0,02869469	0,20835023	RPL17P36	ribosomal protein L17 pseudogene 36 [Source:HGNC Symbol;Acc:HGNC:36051]
ENSG00000136758	0,37882759	7,54896229	6,68761964	0,028784	0,20860214	YME1L1	YME1 like 1 ATPase [Source:HGNC Symbol;Acc:HGNC:12843]
ENSG00000152520	0,53345482	2,47679152	6,68782669	0,02878208	0,20860214	PAN3	poly(A) specific ribonuclease subunit PAN3 [Source:HGNC Symbol;Acc:HGNC:29991]
ENSG00000182903	0,52005459	2,30014537	6,68772539	0,02878302	0,20860214	ZNF721	zinc finger protein 721 [Source:HGNC Symbol;Acc:HGNC:29425]
ENSG00000231789	-1,0335584	0,91553801	6,68734901	0,0287865	0,20860214	PIK3CD-AS2	PIK3CD antisense RNA 2 [Source:HGNC Symbol;Acc:HGNC:51334]
ENSG00000107779	0,44074864	4,53918388	6,67807029	0,02887239	0,20861441	BMPR1A	bone morphogenetic protein receptor type 1A [Source:HGNC Symbol;Acc:HGNC:1076]
ENSG00000112936	1,18608315	-1,0286785	6,68001607	0,02885435	0,20861441	C7	complement C7 [Source:HGNC Symbol;Acc:HGNC:1346]
ENSG00000123154	-0,4718489	2,95535414	6,67791053	0,02887387	0,20861441	WDR83	WD repeat domain 83 [Source:HGNC Symbol;Acc:HGNC:32672]
ENSG00000137494	0,67570716	0,74986875	6,68314901	0,02882533	0,20861441	ANKRD42	ankyrin repeat domain 42 [Source:HGNC Symbol;Acc:HGNC:26752]
ENSG00000139626	1,43983552	-1,0309825	6,68031936	0,02885154	0,20861441	ITGB7	integrin subunit beta 7 [Source:HGNC Symbol;Acc:HGNC:6162]
ENSG00000187796	-0,5610185	2,44429138	6,67986063	0,02885579	0,20861441	CARD9	caspase recruitment domain family member 9 [Source:HGNC Symbol;Acc:HGNC:16391]
ENSG00000171723	-0,6019829	4,13375492	6,67549396	0,02889629	0,20867321	GPHN	gephyrin [Source:HGNC Symbol;Acc:HGNC:15465]

ENSG00000197043	-0,536957	7,65192894	6,6721775	0,0289271	0,20879248	ANXA6	annexin A6 [Source:HGNC Symbol;Acc:HGNC:544]
ENSG00000141580	-0,3656149	6,67299347	6,66798403	0,02896611	0,20897082	WDR45B	WD repeat domain 45B [Source:HGNC Symbol;Acc:HGNC:25072]
ENSG00000157823	0,63905642	1,53262702	6,66389199	0,02900424	0,20904789	AP3S2	adaptor related protein complex 3 subunit sigma 2 [Source:HGNC Symbol;Acc:HGNC:571]
ENSG00000188760	0,35368766	6,20375607	6,66376642	0,02900541	0,20904789	TMEM198	transmembrane protein 198 [Source:HGNC Symbol;Acc:HGNC:33704]
ENSG00000132823	0,36058943	6,46200565	6,65825029	0,02905692	0,20931582	OSER1	oxidative stress responsive serine rich 1 [Source:HGNC Symbol;Acc:HGNC:16105]
ENSG00000136574	0,34755335	5,40725737	6,65407516	0,02909597	0,20949387	GATA4	GATA binding protein 4 [Source:HGNC Symbol;Acc:HGNC:4173]
ENSG00000166908	-0,361325	5,1872532	6,65059011	0,02912862	0,20952243	PIP4K2C	phosphatidylinositol-5-phosphate 4-kinase type 2 gamma [Source:HGNC Symbol;Acc:HGNC:23786]
ENSG00000281358	-1,1580974	-0,8894074	6,65142045	0,02912084	0,20952243	RASSF1-AS1	RASSF1 antisense RNA 1 [Source:HGNC Symbol;Acc:HGNC:49091]
ENSG00000106780	0,42798469	2,77714624	6,64031407	0,02922516	0,20970059	MEGF9	multiple EGF like domains 9 [Source:HGNC Symbol;Acc:HGNC:3234]
ENSG00000136813	0,38710197	6,16257406	6,64472799	0,02918365	0,20970059	ECPAS	Ecm29 proteasome adaptor and scaffold [Source:HGNC Symbol;Acc:HGNC:29020]
ENSG00000171497	0,34373796	6,83524969	6,64049366	0,02922347	0,20970059	PPID	peptidylprolyl isomerase D [Source:HGNC Symbol;Acc:HGNC:9257]
ENSG00000176170	-0,363886	7,43001781	6,64098598	0,02921884	0,20970059	SPHK1	sphingosine kinase 1 [Source:HGNC Symbol;Acc:HGNC:11240]
ENSG00000202111	-1,3980496	-1,5835822	6,6418926	0,02921031	0,20970059	VTRNA1-2	vault RNA 1-2 [Source:HGNC Symbol;Acc:HGNC:12655]
ENSG00000166866	-0,9378283	1,22426102	6,6365795	0,02926035	0,20984997	MYO1A	myosin IA [Source:HGNC Symbol;Acc:HGNC:7595]
ENSG00000185800	-0,4115893	3,59796796	6,63435223	0,02928135	0,20989759	DMWD	DM1 locus, WD repeat containing [Source:HGNC Symbol;Acc:HGNC:2936]
ENSG00000101158	0,36161639	7,69113111	6,62696227	0,02935119	0,20998605	NELFCD	negative elongation factor complex member C/D [Source:HGNC Symbol;Acc:HGNC:15934]
ENSG00000123358	0,41618693	3,83483403	6,62698256	0,029351	0,20998605	NR4A1	nuclear receptor subfamily 4 group A member 1 [Source:HGNC Symbol;Acc:HGNC:7980]
ENSG00000153044	-0,6335904	2,63977146	6,62818722	0,0293396	0,20998605	CENPH	centromere protein H [Source:HGNC Symbol;Acc:HGNC:17268]
ENSG00000256162	-0,42625	7,43716897	6,63120534	0,02931107	0,20998605	SMLR1	small leucine rich protein 1 [Source:HGNC Symbol;Acc:HGNC:44670]
ENSG00000110042	0,5608725	2,6442323	6,6156522	0,02945847	0,21013612	DTX4	deltex E3 ubiquitin ligase 4 [Source:HGNC Symbol;Acc:HGNC:29151]
ENSG00000140022	1,02109164	0,49917785	6,62219801	0,02939632	0,21013612	STON2	stonin 2 [Source:HGNC Symbol;Acc:HGNC:30652]
ENSG00000147883	-0,7445264	0,2810757	6,62103332	0,02940737	0,21013612	CDKN2B	cyclin dependent kinase inhibitor 2B [Source:HGNC Symbol;Acc:HGNC:1788]
ENSG00000149742	0,62807574	2,09343719	6,61587788	0,02945632	0,21013612	SLC22A9	solute carrier family 22 member 9 [Source:HGNC Symbol;Acc:HGNC:16261]
ENSG00000163812	-0,5584352	5,41489357	6,61756371	0,0294403	0,21013612	ZDHHC3	zinc finger DHHC-type palmitoyltransferase 3 [Source:HGNC Symbol;Acc:HGNC:18470]
ENSG00000226082	-1,0174376	-0,9740303	6,61942732	0,02942261	0,21013612	AC096915.1	ribosomal protein S10 (RPS10) pseudogene
ENSG00000075218	0,37431181	4,63149126	6,60753285	0,02953578	0,21037415	GTSE1	G2 and S-phase expressed 1 [Source:HGNC Symbol;Acc:HGNC:13698]
ENSG00000140682	-0,8567164	1,15017977	6,60632765	0,02954728	0,21037415	TGFB1I1	transforming growth factor beta 1 induced transcript 1 [Source:HGNC Symbol;Acc:HGNC:11767]
ENSG00000146872	0,38615185	5,72693905	6,60459294	0,02956384	0,21037415	TLK2	tousled like kinase 2 [Source:HGNC Symbol;Acc:HGNC:11842]
ENSG00000224063	-0,5942012	2,51512901	6,6046783	0,02956302	0,21037415	AC007319.1	novel transcript, antisense to TFPI and CALCRL
ENSG00000259347	-0,6762215	0,90611266	6,60747598	0,02953633	0,21037415	AC087482.1	novel transcript, antisense to SMAD3
ENSG00000113319	-0,426451	4,68006936	6,60057948	0,02960219	0,21054452	RASGRF2	Ras protein specific guanine nucleotide releasing factor 2 [Source:HGNC Symbol;Acc:HGNC:9876]
ENSG00000149179	0,37875386	4,39744036	6,59746596	0,02963199	0,21065388	C11orf49	chromosome 11 open reading frame 49 [Source:HGNC Symbol;Acc:HGNC:28720]
ENSG00000175886	-0,4378314	2,48869095	6,59474341	0,02965807	0,21073678	RPL7AP66	ribosomal protein L7a pseudogene 66 [Source:HGNC Symbol;Acc:HGNC:35682]
ENSG00000172586	-0,4111828	5,68841452	6,58762755	0,02972639	0,21111949	CHCHD1	coiled-coil-helix-coiled-coil-helix domain containing 1 [Source:HGNC Symbol;Acc:HGNC:23518]
ENSG00000170425	-0,3802305	4,11646955	6,58558288	0,02974605	0,2111565	ADORA2B	adenosine A2b receptor [Source:HGNC Symbol;Acc:HGNC:264]
ENSG00000099804	-0,4024696	7,51599442	6,57880491	0,02981135	0,21151728	CDC34	cell division cycle 34, ubiqiutin conjugating enzyme [Source:HGNC Symbol;Acc:HGNC:1734]
ENSG00000134690	0,3536071	5,85764505	6,57725333	0,02982633	0,21152079	CDCA8	cell division cycle associated 8 [Source:HGNC Symbol;Acc:HGNC:14629]
ENSG00000144451	0,42134197	2,93215915	6,56957926	0,02990052	0,21194408	SPAG16	sperm associated antigen 16 [Source:HGNC Symbol;Acc:HGNC:23225]
ENSG00000164379	-0,3948926	6,23691344	6,56078829	0,0299858	0,21244546	FOXQ1	forkhead box Q1 [Source:HGNC Symbol;Acc:HGNC:20951]
ENSG00000122035	-0,7501967	0,68652075	6,55737401	0,030019	0,21251549	RASL11A	RAS like family 11 member A [Source:HGNC Symbol;Acc:HGNC:23802]
ENSG00000167895	-0,8690811	1,64605566	6,55678035	0,03002478	0,21251549	TMC8	transmembrane channel like 8 [Source:HGNC Symbol;Acc:HGNC:20474]
ENSG00000038427	-0,8289298	1,6698543	6,55052309	0,03008576	0,21257675	VCAN	versican [Source:HGNC Symbol;Acc:HGNC:2464]
ENSG00000113916	0,39571203	3,98543307	6,54999761	0,03009088	0,21257675	BCL6	BCL6 transcription repressor [Source:HGNC Symbol;Acc:HGNC:1001]
ENSG00000198610	0,58557671	1,4456904	6,55410579	0,03005082	0,21257675	AKR1C4	aldo-keto reductase family 1 member C4 [Source:HGNC Symbol;Acc:HGNC:387]

ENSG00000232931	-1,704032	-1,6694955	6,54992045	0,03009164	0,21257675	LINC00342	long intergenic non-protein coding RNA 342 [Source:HGNC Symbol;Acc:HGNC:42470]
ENSG00000170345	-0,7788137	1,86027991	6,54195013	0,03016955	0,21302414	FOS	Fos proto-oncogene, AP-1 transcription factor subunit [Source:HGNC Symbol;Acc:HGNC:3796]
ENSG00000063322	-0,4078873	4,40057378	6,53178776	0,03026925	0,21302819	MED29	mediator complex subunit 29 [Source:HGNC Symbol;Acc:HGNC:23074]
ENSG00000091483	0,45301557	7,31196618	6,52439258	0,03034206	0,21302819	FH	fumarate hydratase [Source:HGNC Symbol;Acc:HGNC:3700]
ENSG00000106211	0,35837181	6,74087025	6,52524967	0,03033361	0,21302819	HSPB1	heat shock protein family B (small) member 1 [Source:HGNC Symbol;Acc:HGNC:5246]
ENSG00000114346	0,44010546	5,38209224	6,52425984	0,03034337	0,21302819	ECT2	epithelial cell transforming 2 [Source:HGNC Symbol;Acc:HGNC:3155]
ENSG00000115170	0,53767465	4,69936463	6,53998868	0,03018876	0,21302819	ACVR1	activin A receptor type 1 [Source:HGNC Symbol;Acc:HGNC:171]
ENSG00000148200	0,60319939	4,20505483	6,52858811	0,03030073	0,21302819	NR6A1	nuclear receptor subfamily 6 group A member 1 [Source:HGNC Symbol;Acc:HGNC:7985]
ENSG00000173542	0,47986515	2,07762581	6,53734876	0,03021464	0,21302819	MOB1B	MOB kinase activator 1B [Source:HGNC Symbol;Acc:HGNC:29801]
ENSG00000180211	-0,4873017	1,99527237	6,53792446	0,030209	0,21302819	FO393411.1	pseudogene similar to part of ribosomal protein L23 RPL23
ENSG00000214562	-1,1951791	-1,5128754	6,53030424	0,03028384	0,21302819	NUTM2D	NUT family member 2D [Source:HGNC Symbol;Acc:HGNC:23447]
ENSG00000235529	1,15836929	-0,6538294	6,52981128	0,03028869	0,21302819	AGAP1-IT1	AGAP1 intronic transcript 1 [Source:HGNC Symbol;Acc:HGNC:41427]
ENSG00000264920	-0,9310286	0,54469271	6,52431878	0,03034279	0,21302819	AC018521.5	novel transcript
ENSG00000272398	-0,7211735	1,6507951	6,52408389	0,03034511	0,21302819	CD24	CD24 molecule [Source:HGNC Symbol;Acc:HGNC:1645]
ENSG00000145214	-0,5099584	3,20364126	6,5186297	0,03039895	0,21330369	DGKQ	diacylglycerol kinase theta [Source:HGNC Symbol;Acc:HGNC:2856]
ENSG00000162975	-0,8697848	-0,0777512	6,51205843	0,03046398	0,21342792	KCNF1	potassium voltage-gated channel modifier subfamily F member 1 [Source:HGNC Symbol;Acc:HGNC:6246]
ENSG00000166347	-0,4908497	6,35700724	6,50893976	0,0304949	0,21342792	CYB5A	cytochrome b5 type A [Source:HGNC Symbol;Acc:HGNC:2570]
ENSG00000171823	0,46113346	2,43727028	6,50855747	0,0304987	0,21342792	FBXL14	F-box and leucine rich repeat protein 14 [Source:HGNC Symbol;Acc:HGNC:28624]
ENSG00000185839	-1,0363989	-0,9096029	6,50956876	0,03048867	0,21342792	AL035411.1	adenylate kinase 2 (AK2) pseudogene
ENSG00000186814	0,54348294	1,97486633	6,50799174	0,03050431	0,21342792	ZSCAN30	zinc finger and SCAN domain containing 30 [Source:HGNC Symbol;Acc:HGNC:33517]
ENSG00000253313	1,43732689	-1,1941351	6,51145717	0,03046994	0,21342792	C1orf210	chromosome 1 open reading frame 210 [Source:HGNC Symbol;Acc:HGNC:28755]
ENSG00000149809	-0,5652064	7,31271888	6,49774911	0,03060618	0,21403818	TM7SF2	transmembrane 7 superfamily member 2 [Source:HGNC Symbol;Acc:HGNC:11863]
ENSG00000184731	0,74980707	0,65191453	6,49456211	0,03063797	0,2141571	FAM110C	family with sequence similarity 110 member C [Source:HGNC Symbol;Acc:HGNC:33340]
ENSG00000271976	1,2842244	-1,6227737	6,49310543	0,03065251	0,2141571	AC012467.2	novel transcript, antisense to IL17RB
ENSG00000005206	-0,4744785	3,37890639	6,47420891	0,03084191	0,21418062	SPPL2B	signal peptide peptidase like 2B [Source:HGNC Symbol;Acc:HGNC:30627]
ENSG00000006831	0,33445143	6,90953111	6,48015689	0,03078214	0,21418062	ADIPOR2	adiponectin receptor 2 [Source:HGNC Symbol;Acc:HGNC:24041]
ENSG00000077238	-0,4455181	6,36488859	6,49084489	0,03067509	0,21418062	IL4R	interleukin 4 receptor [Source:HGNC Symbol;Acc:HGNC:6015]
ENSG00000116857	0,35614574	7,43036519	6,47375625	0,03084647	0,21418062	TMEM9	transmembrane protein 9 [Source:HGNC Symbol;Acc:HGNC:18823]
ENSG00000126351	0,41358465	2,62978054	6,47883378	0,03079542	0,21418062	THRA	thyroid hormone receptor alpha [Source:HGNC Symbol;Acc:HGNC:11796]
ENSG00000140262	0,38361176	4,5440472	6,48778965	0,03070564	0,21418062	TCF12	transcription factor 12 [Source:HGNC Symbol;Acc:HGNC:11623]
ENSG00000149218	-0,512049	3,36920848	6,47473691	0,0308366	0,21418062	ENDOD1	endonuclease domain containing 1 [Source:HGNC Symbol;Acc:HGNC:29129]
ENSG00000164830	0,38259921	3,70453593	6,48626913	0,03072086	0,21418062	OXR1	oxidation resistance 1 [Source:HGNC Symbol;Acc:HGNC:15822]
ENSG00000171234	-0,3458247	5,27236798	6,47629313	0,03082095	0,21418062	UGT2B7	UDP glucuronosyltransferase family 2 member B7 [Source:HGNC Symbol;Acc:HGNC:12554]
ENSG00000171811	1,01483818	-0,3472162	6,48230135	0,03076062	0,21418062	CFAP46	cilia and flagella associated protein 46 [Source:HGNC Symbol;Acc:HGNC:25247]
ENSG00000187866	-0,4021746	3,15654701	6,4812052	0,03077162	0,21418062	FAM122A	family with sequence similarity 122A [Source:HGNC Symbol;Acc:HGNC:23490]
ENSG00000197958	-0,4085506	10,6610861	6,47397923	0,03084422	0,21418062	RPL12	ribosomal protein L12 [Source:HGNC Symbol;Acc:HGNC:10302]
ENSG00000203999	-0,6762934	0,80663632	6,4860939	0,03072262	0,21418062	LINC01270	long intergenic non-protein coding RNA 1270 [Source:HGNC Symbol;Acc:HGNC:27658]
ENSG00000168952	0,41596314	5,19928488	6,4687182	0,03089722	0,2142506	STXBP6	syntaxin binding protein 6 [Source:HGNC Symbol;Acc:HGNC:19666]
ENSG00000177125	0,56746029	1,71001913	6,4664927	0,03091967	0,2142506	ZBTB34	zinc finger and BTB domain containing 34 [Source:HGNC Symbol;Acc:HGNC:31446]
ENSG00000182890	0,63774647	0,87851373	6,46658695	0,03091872	0,2142506	GLUD2	glutamate dehydrogenase 2 [Source:HGNC Symbol;Acc:HGNC:4336]
ENSG00000183475	0,41002734	3,03047101	6,47011635	0,03088312	0,2142506	ASB7	ankyrin repeat and SOCS box containing 7 [Source:HGNC Symbol;Acc:HGNC:17182]
ENSG00000253785	-0,938044	-0,1775546	6,46548171	0,03092987	0,2142506	AC008429.2	pyrophosphatase (inorganic) 1 (PPA1) pseudogene
ENSG00000123965	-0,9324498	-0,4682508	6,46383353	0,03094652	0,21426432	PMS2P5	PMS1 homolog 2, mismatch repair system component pseudogene 5 [Source:HGNC Symbol;Acc:HGNC:9130]
ENSG00000231087	-0,9141355	-0,4646402	6,46153297	0,03096978	0,21432375	FDPSP7	farnesyl diphosphate synthase pseudogene 7 [Source:HGNC Symbol;Acc:HGNC:33487]

ENSG00000272841	-1,1574956	-1,2082955	6,45719305	0,0310137	0,21452613	MAP3K4-AS1	MAP3K4 antisense RNA 1 [Source:HGNC Symbol;Acc:HGNC:55048]
ENSG00000149091	-0,4589919	4,95128998	6,45425595	0,03104348	0,21463045	DGKZ	diacylglycerol kinase zeta [Source:HGNC Symbol;Acc:HGNC:2857]
ENSG00000171621	-0,3992037	3,23985576	6,4522775	0,03106355	0,21466765	SPSB1	splA/ryanodine receptor domain and SOCS box containing 1 [Source:HGNC Symbol;Acc:HGNC:30628]
ENSG00000125388	-0,6389718	0,60214508	6,44783666	0,03110867	0,21469446	GRK4	G protein-coupled receptor kinase 4 [Source:HGNC Symbol;Acc:HGNC:4543]
ENSG00000179988	0,48674748	2,71393962	6,45024864	0,03108415	0,21469446	PSTK	phosphoseryl-tRNA kinase [Source:HGNC Symbol;Acc:HGNC:28578]
ENSG00000233927	-0,4083081	8,66919603	6,44755639	0,03111152	0,21469446	RPS28	ribosomal protein S28 [Source:HGNC Symbol;Acc:HGNC:10418]
ENSG00000156273	-0,4043967	3,08951607	6,44415145	0,03114617	0,21478708	BACH1	BTB domain and CNC homolog 1 [Source:HGNC Symbol;Acc:HGNC:935]
ENSG00000263412	-1,5224512	-1,975065	6,44334906	0,03115434	0,21478708	NFE2L1-DT	NFE2L1 divergent transcript [Source:HGNC Symbol;Acc:HGNC:54812]
ENSG00000100522	0,37984703	5,31287113	6,43587655	0,03123059	0,21510968	GNPNAT1	glucosamine-phosphate N-acetyltransferase 1 [Source:HGNC Symbol;Acc:HGNC:19980]
ENSG00000197372	0,47454337	2,06279481	6,43678108	0,03122134	0,21510968	ZNF675	zinc finger protein 675 [Source:HGNC Symbol;Acc:HGNC:30768]
ENSG00000067141	0,72262192	4,74223317	6,43275007	0,03126255	0,21520591	NEO1	neogenin 1 [Source:HGNC Symbol;Acc:HGNC:7754]
ENSG00000121749	0,43839186	3,6307155	6,43162961	0,03127402	0,21520591	TBC1D15	TBC1 domain family member 15 [Source:HGNC Symbol;Acc:HGNC:25694]
ENSG00000170542	-0,4685945	1,98374162	6,4282113	0,03130903	0,21534542	SERPINB9	serpin family B member 9 [Source:HGNC Symbol;Acc:HGNC:8955]
ENSG00000173653	0,34360301	5,87484587	6,42519222	0,03134	0,21545695	RCE1	Ras converting CAAX endopeptidase 1 [Source:HGNC Symbol;Acc:HGNC:13721]
ENSG00000078269	0,42975793	6,28253133	6,41660191	0,03142831	0,21586092	SYNJ2	synaptojanin 2 [Source:HGNC Symbol;Acc:HGNC:11504]
ENSG00000149600	-0,5084073	5,07136589	6,41663807	0,03142794	0,21586092	COMMD7	COMM domain containing 7 [Source:HGNC Symbol;Acc:HGNC:16223]
ENSG00000073464	0,41524908	2,57166329	6,41450838	0,03144988	0,21590755	CLCN4	chloride voltage-gated channel 4 [Source:HGNC Symbol;Acc:HGNC:2022]
ENSG00000111412	0,38151157	4,87098282	6,40866693	0,03151016	0,21601685	SPRING1	SREBF pathway regulator in golgi 1 [Source:HGNC Symbol;Acc:HGNC:26128]
ENSG00000118482	0,38312326	5,90038001	6,41114198	0,0314846	0,21601685	PHF3	PHD finger protein 3 [Source:HGNC Symbol;Acc:HGNC:8921]
ENSG00000213433	-1,3142603	-1,2962432	6,40892196	0,03150752	0,21601685	RPLP1P6	ribosomal protein lateral stalk subunit P1 pseudogene 6 [Source:HGNC Symbol;Acc:HGNC:35809]
ENSG00000131016	-1,378152	5,9657782	6,40307232	0,03156802	0,21631204	AKAP12	A-kinase anchoring protein 12 [Source:HGNC Symbol;Acc:HGNC:370]
ENSG00000220472	-0,3687431	3,93704227	6,39977321	0,03160221	0,21644476	AL139095.2	ribosomal protein S3 (RPS3) pseudogene
ENSG00000156026	0,41168239	5,35393565	6,39731978	0,03162766	0,21651757	MCU	mitochondrial calcium uniporter [Source:HGNC Symbol;Acc:HGNC:23526]
ENSG00000135632	0,38115993	6,52431842	6,39229501	0,03167987	0,21666931	SMYD5	SMYD family member 5 [Source:HGNC Symbol;Acc:HGNC:16258]
ENSG00000154447	-0,7562104	2,32327769	6,39062803	0,03169721	0,21666931	SH3RF1	SH3 domain containing ring finger 1 [Source:HGNC Symbol;Acc:HGNC:17650]
ENSG00000189060	0,33221496	8,418837	6,38663377	0,03173881	0,21666931	H1-0	H1.0 linker histone [Source:HGNC Symbol;Acc:HGNC:4714]
ENSG00000197961	0,49279711	3,9193281	6,38704408	0,03173454	0,21666931	ZNF121	zinc finger protein 121 [Source:HGNC Symbol;Acc:HGNC:12904]
ENSG00000204524	0,65307926	1,34292055	6,38876087	0,03171665	0,21666931	ZNF805	zinc finger protein 805 [Source:HGNC Symbol;Acc:HGNC:23272]
ENSG00000214016	-1,3302456	-1,8939572	6,38975935	0,03170625	0,21666931	RPSAP61	ribosomal protein SA pseudogene 61 [Source:HGNC Symbol;Acc:HGNC:36760]
ENSG00000123609	0,70967274	1,1781311	6,37568645	0,03185319	0,21688415	NMI	N-myc and STAT interactor [Source:HGNC Symbol;Acc:HGNC:7854]
ENSG00000177675	-0,5856141	0,94932668	6,37810866	0,03182784	0,21688415	CD163L1	CD163 molecule like 1 [Source:HGNC Symbol;Acc:HGNC:30375]
ENSG00000179913	0,5253146	3,90429598	6,37509681	0,03185936	0,21688415	B3GNT3	UDP-GlcNAc:betaGal beta-1,3-N-acetylglucosaminyltransferase 3 [Source:HGNC Symbol;Acc:HGNC:13528]
ENSG00000215114	0,44637799	2,42434813	6,3794319	0,031814	0,21688415	UBXN2B	UBX domain protein 2B [Source:HGNC Symbol;Acc:HGNC:27035]
ENSG00000225793	-1,409855	-1,7427605	6,37681724	0,03184135	0,21688415	TMEM30A-DT	TMEM30A divergent transcript [Source:HGNC Symbol;Acc:HGNC:48985]
ENSG00000227619	-1,4060648	-0,5562815	6,37612428	0,0318486	0,21688415	AL391056.1	novel transcript
ENSG00000115718	-0,394007	6,89029947	6,36827552	0,0319309	0,21726992	PROC	protein C, inactivator of coagulation factors Va and VIIIa [Source:HGNC Symbol;Acc:HGNC:9451]
ENSG00000025156	0,42988898	4,50107875	6,36355908	0,03198048	0,21734939	HSF2	heat shock transcription factor 2 [Source:HGNC Symbol;Acc:HGNC:5225]
ENSG00000105329	-0,4602152	6,98301656	6,36162979	0,03200079	0,21734939	TGFB1	transforming growth factor beta 1 [Source:HGNC Symbol;Acc:HGNC:11766]
ENSG00000187068	-0,9425682	0,08269871	6,36150634	0,03200209	0,21734939	C3orf70	chromosome 3 open reading frame 70 [Source:HGNC Symbol;Acc:HGNC:33731]
ENSG00000233980	-0,9881902	-0,7664292	6,36234551	0,03199325	0,21734939	FDPSP2	farnesyl diphosphate synthase pseudogene 2 [Source:HGNC Symbol;Acc:HGNC:3633]
ENSG00000125746	-0,8897652	2,41179491	6,35819789	0,03203696	0,21748507	EML2	EMAP like 2 [Source:HGNC Symbol;Acc:HGNC:18035]
ENSG00000108786	-0,9275564	-0,2393385	6,34996253	0,03212395	0,21784921	HSD17B1	hydroxysteroid 17-beta dehydrogenase 1 [Source:HGNC Symbol;Acc:HGNC:5210]
ENSG00000136636	0,39989057	6,27723944	6,3514924	0,03210776	0,21784921	KCTD3	potassium channel tetramerization domain containing 3 [Source:HGNC Symbol;Acc:HGNC:21305]
ENSG00000138688	0,79177595	3,44074235	6,34888687	0,03213533	0,21784921	KIAA1109	KIAA1109 [Source:HGNC Symbol;Acc:HGNC:26953]

ENSG00000108799	0,43774079	3,13673994	6,34465564	0,03218016	0,21793347	EZH1	enhancer of zeste 1 polycomb repressive complex 2 subunit [Source:HGNC Symbol;Acc:HGNC:3526]
ENSG00000112118	0,48936462	8,12035199	6,34036982	0,03222564	0,21793347	MCM3	minichromosome maintenance complex component 3 [Source:HGNC Symbol;Acc:HGNC:6945]
ENSG00000114019	0,40486846	4,55847272	6,33927598	0,03223727	0,21793347	AMOTL2	angiomotin like 2 [Source:HGNC Symbol;Acc:HGNC:17812]
ENSG00000139344	-0,5622461	3,04873106	6,34249679	0,03220306	0,21793347	AMDHD1	amidohydrolase domain containing 1 [Source:HGNC Symbol;Acc:HGNC:28577]
ENSG00000169507	0,60485296	1,55842544	6,34414808	0,03218554	0,21793347	SLC38A11	solute carrier family 38 member 11 [Source:HGNC Symbol;Acc:HGNC:26836]
ENSG00000233155	-1,2817185	-1,7211504	6,33934016	0,03223658	0,21793347	HMGA1P8	high mobility group AT-hook 1 pseudogene 8 [Source:HGNC Symbol;Acc:HGNC:39183]
ENSG00000138621	-0,4651491	3,94884421	6,33468016	0,03228615	0,21816299	PPCDC	phosphopantothenoylcysteine decarboxylase [Source:HGNC Symbol;Acc:HGNC:28107]
ENSG00000113269	0,3438772	5,65704554	6,33160541	0,03231891	0,21816833	RNF130	ring finger protein 130 [Source:HGNC Symbol;Acc:HGNC:18280]
ENSG00000164096	-0,417641	6,2359293	6,33059982	0,03232963	0,21816833	C4orf3	chromosome 4 open reading frame 3 [Source:HGNC Symbol;Acc:HGNC:19225]
ENSG00000166619	0,40702265	4,86036258	6,33040182	0,03233174	0,21816833	BLCAP	BLCAP apoptosis inducing factor [Source:HGNC Symbol;Acc:HGNC:1055]
ENSG00000147465	-0,6777829	1,82414184	6,32679838	0,0323702	0,21832701	STAR	steroidogenic acute regulatory protein [Source:HGNC Symbol;Acc:HGNC:11359]
ENSG00000092421	0,51376596	4,94365602	6,31848386	0,03245916	0,21870715	SEMA6A	semaphorin 6A [Source:HGNC Symbol;Acc:HGNC:10738]
ENSG00000119862	-0,3946962	4,2003153	6,31769811	0,03246759	0,21870715	LGALSL	galectin like [Source:HGNC Symbol;Acc:HGNC:25012]
ENSG00000244754	0,38378567	4,67293266	6,31733528	0,03247148	0,21870715	N4BP2L2	NEDD4 binding protein 2 like 2 [Source:HGNC Symbol;Acc:HGNC:26916]
ENSG00000165757	-1,8403702	-1,188123	6,31432642	0,03250376	0,21872291	JCAD	junctional cadherin 5 associated [Source:HGNC Symbol;Acc:HGNC:29283]
ENSG00000189143	-0,3494232	6,49864008	6,31454469	0,03250142	0,21872291	CLDN4	claudin 4 [Source:HGNC Symbol;Acc:HGNC:2046]
ENSG00000259153	-0,5483085	1,73880046	6,3112101	0,03253724	0,21884738	AC004816.1	novel transcript
ENSG00000102032	-0,7653131	1,31275733	6,29666807	0,03269402	0,21909494	RENBP	renin binding protein [Source:HGNC Symbol;Acc:HGNC:9959]
ENSG00000116539	0,59603158	2,44687621	6,29744152	0,03268566	0,21909494	ASH1L	ASH1 like histone lysine methyltransferase [Source:HGNC Symbol;Acc:HGNC:19088]
ENSG00000132849	0,67907498	3,12520202	6,3005564	0,03265201	0,21909494	PATJ	PATJ crumbs cell polarity complex component [Source:HGNC Symbol;Acc:HGNC:28881]
ENSG00000135469	0,40990448	3,52351475	6,3012054	0,032645	0,21909494	COQ10A	coenzyme Q10A [Source:HGNC Symbol;Acc:HGNC:26515]
ENSG00000144136	-0,4497411	7,77174714	6,297171	0,03268858	0,21909494	SLC20A1	solute carrier family 20 member 1 [Source:HGNC Symbol;Acc:HGNC:10946]
ENSG00000148834	-0,347558	8,50201556	6,30378946	0,03261713	0,21909494	GSTO1	glutathione S-transferase omega 1 [Source:HGNC Symbol;Acc:HGNC:13312]
ENSG00000254112	-1,3663089	-1,5007512	6,30464978	0,03260785	0,21909494	AP003467.2	ATP synthase, H+ transporting, mitochondrial F0 complex, subunit F6 (ATP5J) pseudogene
ENSG00000267493	-1,804191	-1,1238905	6,30099366	0,03264729	0,21909494	CIRBP-AS1	CIRBP antisense RNA 1 [Source:HGNC Symbol;Acc:HGNC:28588]
ENSG00000241370	1,32226541	-1,064829	6,29161638	0,03274871	0,21936077	RPP21	ribonuclease P/MRP subunit p21 [Source:HGNC Symbol;Acc:HGNC:21300]
ENSG00000086848	0,65581527	2,18265796	6,27819299	0,03289456	0,2195339	ALG9	ALG9 alpha-1,2-mannosyltransferase [Source:HGNC Symbol;Acc:HGNC:15672]
ENSG00000090674	-0,3892313	3,80263913	6,28118422	0,03286199	0,2195339	MCOLN1	mucolipin 1 [Source:HGNC Symbol;Acc:HGNC:13356]
ENSG00000095059	1,2396702	-1,4624233	6,27679489	0,0329098	0,2195339	DHPS	deoxyhypusine synthase [Source:HGNC Symbol;Acc:HGNC:2869]
ENSG00000103227	-0,6496403	1,51057735	6,27682952	0,03290942	0,2195339	LMF1	lipase maturation factor 1 [Source:HGNC Symbol;Acc:HGNC:14154]
ENSG00000103353	0,39155837	6,36647718	6,27989639	0,03287601	0,2195339	UBFD1	ubiquitin family domain containing 1 [Source:HGNC Symbol;Acc:HGNC:30565]
ENSG00000149531	0,51135221	2,07618741	6,27751267	0,03290197	0,2195339	FRG1BP	FSHD region gene 1 family member B, pseudogene [Source:HGNC Symbol;Acc:HGNC:15792]
ENSG00000165480	-0,4151626	3,69002813	6,27933324	0,03288214	0,2195339	SKA3	spindle and kinetochore associated complex subunit 3 [Source:HGNC Symbol;Acc:HGNC:20262]
ENSG00000167799	-0,4484468	3,97189309	6,28532953	0,03281692	0,2195339	NUDT8	nudix hydrolase 8 [Source:HGNC Symbol;Acc:HGNC:8055]
ENSG00000168090	-0,3466429	6,86886102	6,28597938	0,03280986	0,2195339	COPS6	COP9 signalosome subunit 6 [Source:HGNC Symbol;Acc:HGNC:21749]
ENSG00000157456	0,37843917	6,93878518	6,2711762	0,03297112	0,21980975	CCNB2	cyclin B2 [Source:HGNC Symbol;Acc:HGNC:1580]
ENSG00000164331	0,51102358	3,30618709	6,27025025	0,03298124	0,21980975	ANKRA2	ankyrin repeat family A member 2 [Source:HGNC Symbol;Acc:HGNC:13208]
ENSG00000079150	0,49655254	3,71402957	6,26472634	0,03304169	0,21995967	FKBP7	FKBP prolyl isomerase 7 [Source:HGNC Symbol;Acc:HGNC:3723]
ENSG00000141429	0,38078312	6,63105023	6,26185448	0,03307318	0,21995967	GALNT1	polypeptide N-acetylgalactosaminyltransferase 1 [Source:HGNC Symbol;Acc:HGNC:4123]
ENSG00000142634	-0,3574853	4,67998037	6,26163007	0,03307564	0,21995967	EFHD2	EF-hand domain family member D2 [Source:HGNC Symbol;Acc:HGNC:28670]
ENSG00000166510	-0,8003857	0,64708507	6,265661	0,03303146	0,21995967	CCDC68	coiled-coil domain containing 68 [Source:HGNC Symbol;Acc:HGNC:24350]
ENSG00000228436	-0,8224995	-0,1737519	6,26132186	0,03307902	0,21995967	AL139260.1	novel transcript
ENSG00000144034	0,34654771	5,39369054	6,25847171	0,03311031	0,22006755	TPRKB	TP53RK binding protein [Source:HGNC Symbol;Acc:HGNC:24259]
ENSG00000107854	0,51287555	4,29323972	6,25123292	0,03318994	0,2204965	TNKS2	tankyrase 2 [Source:HGNC Symbol;Acc:HGNC:15677]

ENSG00000151611	0,67096251	1,63870648	6,24878159	0,03321696	0,22057569	MMAA	metabolism of cobalamin associated A [Source:HGNC Symbol;Acc:HGNC:18871]
ENSG00000115464	0,45524488	5,13888742	6,24659122	0,03324112	0,22063588	USP34	ubiquitin specific peptidase 34 [Source:HGNC Symbol;Acc:HGNC:20066]
ENSG00000123349	0,99249368	-0,1086232	6,23930507	0,03332167	0,22077191	PFDN5	prefoldin subunit 5 [Source:HGNC Symbol;Acc:HGNC:8869]
ENSG00000144214	-1,1157262	-0,7882472	6,23851695	0,03333039	0,22077191	LYG1	lysozyme g1 [Source:HGNC Symbol;Acc:HGNC:27014]
ENSG00000186951	0,43266736	2,41655105	6,23790439	0,03333718	0,22077191	PPARA	peroxisome proliferator activated receptor alpha [Source:HGNC Symbol;Acc:HGNC:9232]
ENSG00000198689	-0,491528	2,53453142	6,24065887	0,03330668	0,22077191	SLC9A6	solute carrier family 9 member A6 [Source:HGNC Symbol;Acc:HGNC:11079]
ENSG00000270049	-0,931724	0,51005812	6,23823382	0,03333353	0,22077191	AC009061.2	novel transcript, antisense to AGRP
ENSG00000085491	-0,5686876	2,65851785	6,23493825	0,03337005	0,2208895	SLC25A24	solute carrier family 25 member 24 [Source:HGNC Symbol;Acc:HGNC:20662]
ENSG00000137267	-0,5055588	4,98379872	6,22943463	0,03343116	0,22109365	TUBB2A	tubulin beta 2A class IIa [Source:HGNC Symbol;Acc:HGNC:12412]
ENSG00000242692	-0,4464994	2,23778144	6,23016693	0,03342302	0,22109365	RPS27AP1	RPS27A pseudogene 1 [Source:HGNC Symbol;Acc:HGNC:17121]
ENSG00000116406	-0,5415838	2,76417755	6,2234979	0,03349724	0,22143037	EDEM3	ER degradation enhancing alpha-mannosidase like protein 3 [Source:HGNC Symbol;Acc:HGNC:16787]
ENSG00000005483	0,7281546	3,22276471	6,21714231	0,03356815	0,221465	KMT2E	lysine methyltransferase 2E (inactive) [Source:HGNC Symbol;Acc:HGNC:18541]
ENSG00000103540	0,46032335	2,55793643	6,219547	0,0335413	0,221465	CCP110	centriolar coiled-coil protein 110 [Source:HGNC Symbol;Acc:HGNC:24342]
ENSG00000164307	0,59045096	4,9352514	6,2165885	0,03357434	0,221465	ERAP1	endoplasmic reticulum aminopeptidase 1 [Source:HGNC Symbol;Acc:HGNC:18173]
ENSG00000182534	-0,4168559	5,5119587	6,21623656	0,03357827	0,221465	MXRA7	matrix remodeling associated 7 [Source:HGNC Symbol;Acc:HGNC:7541]
ENSG00000259755	-1,2376314	-1,5144765	6,2188213	0,0335494	0,221465	AC090907.2	novel transcript, antisense to LRRK1
ENSG00000130528	-0,8596771	2,96632624	6,21252817	0,03361975	0,22153853	HRC	histidine rich calcium binding protein [Source:HGNC Symbol;Acc:HGNC:5178]
ENSG00000214391	-0,5453118	1,58129073	6,21317459	0,03361252	0,22153853	TUBAP2	tubulin alpha pseudogene 2 [Source:HGNC Symbol;Acc:HGNC:14531]
ENSG00000144852	-0,410218	2,58267854	6,21008001	0,03364717	0,22161924	NR1I2	nuclear receptor subfamily 1 group I member 2 [Source:HGNC Symbol;Acc:HGNC:7968]
ENSG00000105708	0,41941669	2,77205998	6,20868029	0,03366286	0,22162265	ZNF14	zinc finger protein 14 [Source:HGNC Symbol;Acc:HGNC:12924]
ENSG00000205084	0,45741892	2,34084006	6,20667763	0,03368532	0,22167063	TMEM231	transmembrane protein 231 [Source:HGNC Symbol;Acc:HGNC:37234]
ENSG00000111665	-0,3784805	5,74615037	6,20401941	0,03371516	0,22175348	CDCA3	cell division cycle associated 3 [Source:HGNC Symbol;Acc:HGNC:14624]
ENSG00000185414	0,33936921	5,52232746	6,20285307	0,03372826	0,22175348	MRPL30	mitochondrial ribosomal protein L30 [Source:HGNC Symbol;Acc:HGNC:14036]
ENSG00000119729	-0,4316442	3,10536764	6,19762509	0,03378708	0,22198073	RHOQ	ras homolog family member Q [Source:HGNC Symbol;Acc:HGNC:17736]
ENSG00000143570	-0,871836	-0,1400687	6,19708024	0,03379322	0,22198073	SLC39A1	solute carrier family 39 member 1 [Source:HGNC Symbol;Acc:HGNC:12876]
ENSG00000062485	0,44834513	8,39479473	6,19545638	0,03381152	0,22200111	CS	citrate synthase [Source:HGNC Symbol;Acc:HGNC:2422]
ENSG00000134326	1,42260897	-0,9796248	6,19144777	0,03385674	0,22210137	CMPK2	cytidine/uridine monophosphate kinase 2 [Source:HGNC Symbol;Acc:HGNC:27015]
ENSG00000152620	0,36221683	5,51351125	6,19140774	0,03385719	0,22210137	NADK2	NAD kinase 2, mitochondrial [Source:HGNC Symbol;Acc:HGNC:26404]
ENSG00000171492	0,45793775	5,19731913	6,18705216	0,03390642	0,22223051	LRRC8D	leucine rich repeat containing 8 VRAC subunit D [Source:HGNC Symbol;Acc:HGNC:16992]
ENSG00000249550	0,54019357	1,51616903	6,18697396	0,0339073	0,22223051	LINC01234	long intergenic non-protein coding RNA 1234 [Source:HGNC Symbol;Acc:HGNC:49757]
ENSG00000173218	-0,3925881	3,78997265	6,1845114	0,03393518	0,22230616	VANGL1	VANGL planar cell polarity protein 1 [Source:HGNC Symbol;Acc:HGNC:15512]
ENSG00000174599	1,85458639	-1,6963001	6,18326611	0,03394928	0,22230616	TRAM1L1	translocation associated membrane protein 1 like 1 [Source:HGNC Symbol;Acc:HGNC:28371]
ENSG00000135390	-0,3379001	7,69322958	6,17831847	0,03400539	0,22257382	ATP5MC2	ATP synthase membrane subunit c locus 2 [Source:HGNC Symbol;Acc:HGNC:842]
ENSG00000246067	-0,568076	2,40530284	6,17490801	0,03404414	0,22272763	RAB30-DT	RAB30 divergent transcript [Source:HGNC Symbol;Acc:HGNC:48672]
ENSG00000105559	-0,4101614	2,80836982	6,16862348	0,03411568	0,22277405	PLEKHA4	pleckstrin homology domain containing A4 [Source:HGNC Symbol;Acc:HGNC:14339]
ENSG00000176125	-0,652427	0,40946631	6,16924596	0,03410858	0,22277405	UFSP1	UFM1 specific peptidase 1 (inactive) [Source:HGNC Symbol;Acc:HGNC:33821]
ENSG00000187091	-0,3810745	3,40878954	6,1675884	0,03412748	0,22277405	PLCD1	phospholipase C delta 1 [Source:HGNC Symbol;Acc:HGNC:9060]
ENSG00000187678	-0,5040122	5,47935875	6,17160567	0,03408171	0,22277405	SPRY4	sprouty RTK signaling antagonist 4 [Source:HGNC Symbol;Acc:HGNC:15533]
ENSG00000255398	-0,6551816	1,53708936	6,16885847	0,034113	0,22277405	HCAR3	hydroxycarboxylic acid receptor 3 [Source:HGNC Symbol;Acc:HGNC:16824]
ENSG00000108819	0,37149272	3,77472721	6,16146947	0,03419734	0,22293126	PPP1R9B	protein phosphatase 1 regulatory subunit 9B [Source:HGNC Symbol;Acc:HGNC:9298]
ENSG00000147592	0,39733697	4,06187458	6,16275847	0,03418261	0,22293126	LACTB2	lactamase beta 2 [Source:HGNC Symbol;Acc:HGNC:18512]
ENSG00000167515	-0,3768387	6,69908605	6,16175487	0,03419408	0,22293126	TRAPPC2L	trafficking protein particle complex 2 like [Source:HGNC Symbol;Acc:HGNC:30887]
ENSG00000130653	-0,9174333	0,32947562	6,15743121	0,03424354	0,22309023	PNPLA7	patatin like phospholipase domain containing 7 [Source:HGNC Symbol;Acc:HGNC:24768]
ENSG00000132388	0,4042593	4,85126269	6,1566695	0,03425227	0,22309023	UBE2G1	ubiquitin conjugating enzyme E2 G1 [Source:HGNC Symbol;Acc:HGNC:12482]

ENSG00000106348	-0,341084	5,25720302	6,15351904	0,03428838	0,22322591	IMPDH1	inosine monophosphate dehydrogenase 1 [Source:HGNC Symbol;Acc:HGNC:6052]
ENSG00000142784	0,40684487	2,92722566	6,15210887	0,03430456	0,22323176	WDTC1	WD and tetratricopeptide repeats 1 [Source:HGNC Symbol;Acc:HGNC:29175]
ENSG00000149792	0,32262422	6,27894664	6,14145769	0,03442707	0,22382955	MRPL49	mitochondrial ribosomal protein L49 [Source:HGNC Symbol;Acc:HGNC:1176]
ENSG00000231890	-1,4573289	-1,0525148	6,14249557	0,0344151	0,22382955	DARS-AS1	DARS antisense RNA 1 [Source:HGNC Symbol;Acc:HGNC:40170]
ENSG00000124532	0,43294095	7,23844476	6,13753487	0,03447232	0,22402408	MRS2	magnesium transporter MRS2 [Source:HGNC Symbol;Acc:HGNC:13785]
ENSG00000119946	0,60757407	4,10935716	6,12754817	0,03458787	0,22457517	CNNM1	cyclin and CBS domain divalent metal cation transport mediator 1 [Source:HGNC Symbol;Acc:HGNC:102]
ENSG00000128626	-0,395683	5,76414815	6,12776529	0,03458535	0,22457517	MRPS12	mitochondrial ribosomal protein S12 [Source:HGNC Symbol;Acc:HGNC:10380]
ENSG00000127054	-0,4947069	6,13364734	6,12449736	0,03462326	0,22460532	INTS11	integrator complex subunit 11 [Source:HGNC Symbol;Acc:HGNC:26052]
ENSG00000175305	-0,7429738	0,77999595	6,12521442	0,03461494	0,22460532	CCNE2	cyclin E2 [Source:HGNC Symbol;Acc:HGNC:1590]
ENSG00000141404	-1,4072168	-1,3861024	6,12246882	0,03464682	0,22465839	GNAL	G protein subunit alpha L [Source:HGNC Symbol;Acc:HGNC:4388]
ENSG00000101266	0,35340006	7,5304022	6,11708206	0,03470947	0,22466578	CSNK2A1	casein kinase 2 alpha 1 [Source:HGNC Symbol;Acc:HGNC:2457]
ENSG00000173153	-0,4187133	4,99243965	6,11713272	0,03470888	0,22466578	ESRRA	estrogen related receptor alpha [Source:HGNC Symbol;Acc:HGNC:3471]
ENSG00000213280	-0,4415503	2,75426732	6,11834254	0,0346948	0,22466578	AC090114.1	ribosomal protein S10 (RPS10) pseudogene
ENSG00000265096	-1,122114	0,34460607	6,12067625	0,03466765	0,22466578	C1QTNF1-AS1	C1QTNF1 antisense RNA 1 [Source:HGNC Symbol;Acc:HGNC:44351]
ENSG00000107404	-0,3220337	6,02916074	6,10951088	0,03479777	0,22510321	DVL1	dishevelled segment polarity protein 1 [Source:HGNC Symbol;Acc:HGNC:3084]
ENSG00000182095	0,64684577	5,76536449	6,1086466	0,03480787	0,22510321	TNRC18	trinucleotide repeat containing 18 [Source:HGNC Symbol;Acc:HGNC:11962]
ENSG00000217835	-1,1607721	-1,0455796	6,10242178	0,0348807	0,2254744	RBMXP5	RBMX pseudogene 5 [Source:HGNC Symbol;Acc:HGNC:54979]
ENSG00000269834	1,30082471	-1,0550637	6,10066215	0,03490132	0,22550793	ZNF528-AS1	ZNF528 antisense RNA 1 [Source:HGNC Symbol;Acc:HGNC:51305]
ENSG00000012171	-0,5457951	7,93805587	6,09753477	0,03493801	0,22554548	SEMA3B	semaphorin 3B [Source:HGNC Symbol;Acc:HGNC:10724]
ENSG00000160961	-0,6017989	1,16716615	6,09866448	0,03492475	0,22554548	ZNF333	zinc finger protein 333 [Source:HGNC Symbol;Acc:HGNC:15624]
ENSG00000177885	0,39675171	6,40855257	6,09384366	0,03498138	0,22572567	GRB2	growth factor receptor bound protein 2 [Source:HGNC Symbol;Acc:HGNC:4566]
ENSG00000100614	0,3793822	3,90176473	6,08592372	0,03507465	0,22593494	PPM1A	protein phosphatase, Mg2+/Mn2+ dependent 1A [Source:HGNC Symbol;Acc:HGNC:9275]
ENSG00000109065	-0,3930232	5,58281629	6,08834563	0,03504609	0,22593494	NAT9	N-acetyltransferase 9 (putative) [Source:HGNC Symbol;Acc:HGNC:23133]
ENSG00000110002	1,04721999	-0,5573157	6,086991	0,03506206	0,22593494	VWA5A	von Willebrand factor A domain containing 5A [Source:HGNC Symbol;Acc:HGNC:6658]
ENSG00000148572	-0,4288701	4,60192741	6,0845265	0,03509113	0,22593494	NRBF2	nuclear receptor binding factor 2 [Source:HGNC Symbol;Acc:HGNC:19692]
ENSG00000185834	-0,5225414	2,66394121	6,08547066	0,03507999	0,22593494	RPL12P4	ribosomal protein L12 pseudogene 4 [Source:HGNC Symbol;Acc:HGNC:16587]
ENSG00000122507	0,46178608	2,67207792	6,07800175	0,03516825	0,2263317	BBS9	Bardet-Biedl syndrome 9 [Source:HGNC Symbol;Acc:HGNC:30000]
ENSG00000182749	0,4407175	4,91624803	6,06646493	0,03530512	0,22711249	PAQR7	progestin and adipoQ receptor family member 7 [Source:HGNC Symbol;Acc:HGNC:23146]
ENSG00000215790	1,23778766	-0,8770406	6,06351586	0,03534021	0,22723817	SLC35E2A	solute carrier family 35 member E2A [Source:HGNC Symbol;Acc:HGNC:20863]
ENSG00000114993	-0,3244865	6,94454125	6,05937565	0,03538955	0,22735529	RTKN	rhotekin [Source:HGNC Symbol;Acc:HGNC:10466]
ENSG00000175567	-0,5081842	3,59571547	6,05960895	0,03538677	0,22735529	UCP2	uncoupling protein 2 [Source:HGNC Symbol;Acc:HGNC:12518]
ENSG00000056097	-0,5125277	5,51480275	6,05604923	0,03542925	0,22742596	ZFR	zinc finger RNA binding protein [Source:HGNC Symbol;Acc:HGNC:17277]
ENSG00000125968	-0,3358111	8,0705106	6,05461823	0,03544635	0,22742596	ID1	inhibitor of DNA binding 1, HLH protein [Source:HGNC Symbol;Acc:HGNC:5360]
ENSG00000227533	-1,444622	-1,7534012	6,05454269	0,03544725	0,22742596	SLC2A1-AS1	SLC2A1 antisense RNA 1 [Source:HGNC Symbol;Acc:HGNC:44187]
ENSG00000107771	-0,4399778	2,1915336	6,05018735	0,03549935	0,2274351	CCSER2	coiled-coil serine rich protein 2 [Source:HGNC Symbol;Acc:HGNC:29197]
ENSG00000167034	-0,4435151	1,9851081	6,0520266	0,03547734	0,2274351	NKX3-1	NK3 homeobox 1 [Source:HGNC Symbol;Acc:HGNC:7838]
ENSG00000196139	0,34304884	8,81906099	6,04921899	0,03551095	0,2274351	AKR1C3	aldo-keto reductase family 1 member C3 [Source:HGNC Symbol;Acc:HGNC:386]
ENSG00000213253	-0,715374	0,14623229	6,05074374	0,03549269	0,2274351	RPL12P42	ribosomal protein L12 pseudogene 42 [Source:HGNC Symbol;Acc:HGNC:36165]
ENSG00000272800	-1,0012453	-0,9498889	6,04720895	0,03553504	0,22748963	AC021851.1	novel transcript
ENSG00000164815	0,33624046	5,12515161	6,0399147	0,03562261	0,22789494	ORC5	origin recognition complex subunit 5 [Source:HGNC Symbol;Acc:HGNC:8491]
ENSG00000168002	-0,3501801	6,36404797	6,03933821	0,03562955	0,22789494	POLR2G	RNA polymerase II subunit G [Source:HGNC Symbol;Acc:HGNC:9194]
ENSG00000138678	-0,7785784	3,69507669	6,03622246	0,03566705	0,2279352	GPAT3	glycerol-3-phosphate acyltransferase 3 [Source:HGNC Symbol;Acc:HGNC:28157]
ENSG00000161298	0,64648785	0,89854273	6,0371941	0,03565535	0,2279352	ZNF382	zinc finger protein 382 [Source:HGNC Symbol;Acc:HGNC:17409]
ENSG00000102886	0,39738099	5,18407604	6,03000858	0,03574198	0,22831419	GDPD3	glycerophosphodiester phosphodiesterase domain containing 3 [Source:HGNC Symbol;Acc:HGNC:28638]

ENSG00000100916	0,41565316	4,89992342	6,02776638	0,03576906	0,22838735	BRMS1L	BRMS1 like transcriptional repressor [Source:HGNC Symbol;Acc:HGNC:20512]
ENSG00000112972	-0,386934	7,82145551	6,02536973	0,03579804	0,22847254	HMGCS1	3-hydroxy-3-methylglutaryl-CoA synthase 1 [Source:HGNC Symbol;Acc:HGNC:5007]
ENSG00000266094	1,47798654	-1,9207985	6,02228033	0,03583544	0,22861136	RASSF5	Ras association domain family member 5 [Source:HGNC Symbol;Acc:HGNC:17609]
ENSG00000107938	0,45512073	3,24423773	6,01559143	0,03591659	0,22902899	EDRF1	erythroid differentiation regulatory factor 1 [Source:HGNC Symbol;Acc:HGNC:24640]
ENSG00000137309	-0,3802636	8,30865657	6,0120892	0,03595916	0,22920043	HMGA1	high mobility group AT-hook 1 [Source:HGNC Symbol;Acc:HGNC:5010]
ENSG00000126453	-0,3496188	5,66730629	6,00197575	0,03608246	0,22988601	BCL2L12	BCL2 like 12 [Source:HGNC Symbol;Acc:HGNC:13787]
ENSG00000049883	0,38673289	3,61994982	5,99570115	0,03615922	0,23003489	PTCD2	pentatricopeptide repeat domain 2 [Source:HGNC Symbol;Acc:HGNC:25734]
ENSG00000055208	0,37408222	3,93695828	5,99724932	0,03614026	0,23003489	TAB2	TGF-beta activated kinase 1 (MAP3K7) binding protein 2 [Source:HGNC Symbol;Acc:HGNC:17075]
ENSG00000176148	-0,3830421	3,0299299	5,99491815	0,03616881	0,23003489	TCP11L1	t-complex 11 like 1 [Source:HGNC Symbol;Acc:HGNC:25655]
ENSG00000235674	-0,9161176	-0,6985619	5,99744983	0,0361378	0,23003489	LDHAP2	lactate dehydrogenase A pseudogene 2 [Source:HGNC Symbol;Acc:HGNC:6537]
ENSG00000136940	0,38729924	4,32893527	5,99153428	0,0362103	0,23019857	PDCL	phosducin like [Source:HGNC Symbol;Acc:HGNC:8770]
ENSG00000040487	-0,3436775	5,55920866	5,98442622	0,03629766	0,23041412	SLC66A1	solute carrier family 66 member 1 [Source:HGNC Symbol;Acc:HGNC:26001]
ENSG00000081014	0,42096495	2,64265327	5,98460677	0,03629543	0,23041412	AP4E1	adaptor related protein complex 4 subunit epsilon 1 [Source:HGNC Symbol;Acc:HGNC:573]
ENSG00000142102	-0,6096779	1,29667551	5,98491148	0,03629168	0,23041412	PGGHG	protein-glucosylgalactosylhydroxylysine glucosidase [Source:HGNC Symbol;Acc:HGNC:26210]
ENSG00000172590	-0,3403277	7,83453902	5,98364302	0,0363073	0,23041412	MRPL52	mitochondrial ribosomal protein L52 [Source:HGNC Symbol;Acc:HGNC:16655]
ENSG00000179630	1,33883702	-0,5586848	5,98076403	0,03634276	0,23053905	LACC1	laccase domain containing 1 [Source:HGNC Symbol;Acc:HGNC:26789]
ENSG00000124784	0,35893409	5,92188008	5,97467657	0,0364179	0,2307442	RIOK1	RIO kinase 1 [Source:HGNC Symbol;Acc:HGNC:18656]
ENSG00000163900	0,35754037	5,97400773	5,97161762	0,03645572	0,2307442	TMEM41A	transmembrane protein 41A [Source:HGNC Symbol;Acc:HGNC:30544]
ENSG00000173875	0,42262475	3,06068419	5,97672572	0,03639258	0,2307442	ZNF791	zinc finger protein 791 [Source:HGNC Symbol;Acc:HGNC:26895]
ENSG00000185515	0,34459565	4,82621477	5,9718761	0,03645252	0,2307442	BRCC3	BRCA1/BRCA2-containing complex subunit 3 [Source:HGNC Symbol;Acc:HGNC:24185]
ENSG00000188389	-2,0401369	-1,6388008	5,97071427	0,0364669	0,2307442	PDCD1	programmed cell death 1 [Source:HGNC Symbol;Acc:HGNC:8760]
ENSG00000189077	-0,3793107	6,87586621	5,96839271	0,03649565	0,2307442	TMEM120A	transmembrane protein 120A [Source:HGNC Symbol;Acc:HGNC:21697]
ENSG00000205100	-0,7336749	0,64688406	5,96792418	0,03650146	0,2307442	HSP90AA4P	heat shock protein 90 alpha family class A member 4, pseudogene [Source:HGNC Symbol;Acc:HGNC:5255]
ENSG00000261701	-0,3988418	5,09209658	5,96798252	0,03650074	0,2307442	HPR	haptoglobin-related protein [Source:HGNC Symbol;Acc:HGNC:5156]
ENSG00000127586	-0,4862789	3,0262542	5,96073081	0,03659074	0,23120856	CHTF18	chromosome transmission fidelity factor 18 [Source:HGNC Symbol;Acc:HGNC:18435]
ENSG00000170871	0,37994297	3,41438612	5,9582743	0,0366213	0,23130157	KIAA0232	KIAA0232 [Source:HGNC Symbol;Acc:HGNC:28992]
ENSG00000197165	-0,6116305	1,40395206	5,9564639	0,03664383	0,23134389	SULT1A2	sulfotransferase family 1A member 2 [Source:HGNC Symbol;Acc:HGNC:11454]
ENSG00000060566	0,56838912	3,45208246	5,94839657	0,03674447	0,23187901	CREB3L3	cAMP responsive element binding protein 3 like 3 [Source:HGNC Symbol;Acc:HGNC:18855]
ENSG00000165698	-1,1215393	-0,9708298	5,94611257	0,03677302	0,231959	SPACA9	sperm acrosome associated 9 [Source:HGNC Symbol;Acc:HGNC:1367]
ENSG00000203499	-1,0283788	-0,6163347	5,94142032	0,03683177	0,2322293	IQANK1	IQ motif and ankyrin repeat containing 1 [Source:HGNC Symbol;Acc:HGNC:49576]
ENSG00000119686	0,43969076	3,93550421	5,93632636	0,03689567	0,23233142	FLVCR2	FLVCR heme transporter 2 [Source:HGNC Symbol;Acc:HGNC:20105]
ENSG00000143537	0,36746391	6,47669554	5,93781399	0,036877	0,23233142	ADAM15	ADAM metallopeptidase domain 15 [Source:HGNC Symbol;Acc:HGNC:193]
ENSG00000224321	-0,6669896	0,3972417	5,93653778	0,03689302	0,23233142	RPL12P14	ribosomal protein L12 pseudogene 14 [Source:HGNC Symbol;Acc:HGNC:36495]
ENSG00000185219	0,48566408	3,83413939	5,93365772	0,03692921	0,2324424	ZNF445	zinc finger protein 445 [Source:HGNC Symbol;Acc:HGNC:21018]
ENSG00000166886	-0,4998521	4,87797639	5,92910874	0,03698646	0,23260232	NAB2	NGFI-A binding protein 2 [Source:HGNC Symbol;Acc:HGNC:7627]
ENSG00000232818	-0,7378041	0,71282448	5,92976221	0,03697823	0,23260232	RPS2P32	ribosomal protein S2 pseudogene 32 [Source:HGNC Symbol;Acc:HGNC:30518]
ENSG00000100836	0,47463753	2,66369983	5,92705722	0,03701231	0,23266476	PABPN1	poly(A) binding protein nuclear 1 [Source:HGNC Symbol;Acc:HGNC:8565]
ENSG00000242485	-0,3608317	6,17696962	5,92321491	0,0370608	0,23286934	MRPL20	mitochondrial ribosomal protein L20 [Source:HGNC Symbol;Acc:HGNC:14478]
ENSG00000075975	-0,3551343	4,99824307	5,91712988	0,03713775	0,23289603	MKRN2	makorin ring finger protein 2 [Source:HGNC Symbol;Acc:HGNC:7113]
ENSG00000110321	-0,3311519	9,09798628	5,91657614	0,03714476	0,23289603	EIF4G2	eukaryotic translation initiation factor 4 gamma 2 [Source:HGNC Symbol;Acc:HGNC:3297]
ENSG00000116833	0,6944664	0,74865129	5,92072658	0,03709224	0,23289603	NR5A2	nuclear receptor subfamily 5 group A member 2 [Source:HGNC Symbol;Acc:HGNC:7984]
ENSG00000182154	-0,3533367	5,75980436	5,91877641	0,03711691	0,23289603	MRPL41	mitochondrial ribosomal protein L41 [Source:HGNC Symbol;Acc:HGNC:14492]
ENSG00000280128	0,84406196	0,65263744	5,92031303	0,03709747	0,23289603	AL662795.2	TEC
ENSG00000173818	-0,5038299	2,76080095	5,91529916	0,03716093	0,2328975	ENDOV	endonuclease V [Source:HGNC Symbol;Acc:HGNC:26640]

ENSG00000185483	0,60889581	1,89137025	5,91262873	0,03719479	0,23300973	ROR1	receptor tyrosine kinase like orphan receptor 1 [Source:HGNC Symbol;Acc:HGNC:10256]
ENSG00000139291	-0,4822037	2,41012137	5,90762345	0,03725835	0,2330494	TMEM19	transmembrane protein 19 [Source:HGNC Symbol;Acc:HGNC:25605]
ENSG00000145332	-0,4280317	2,76974639	5,91043076	0,03722269	0,2330494	KLHL8	kelch like family member 8 [Source:HGNC Symbol;Acc:HGNC:18644]
ENSG00000186280	-0,7359152	0,20663028	5,90814546	0,03725172	0,2330494	KDM4D	lysine demethylase 4D [Source:HGNC Symbol;Acc:HGNC:25498]
ENSG00000217239	-1,2002138	-1,6689864	5,90710587	0,03726493	0,2330494	AL136968.2	ribosomal protein L34 (RPL34) pseudogene
ENSG00000110851	0,3569911	4,02851744	5,90137513	0,03733789	0,23340573	PRDM4	PR/SET domain 4 [Source:HGNC Symbol;Acc:HGNC:9348]
ENSG00000198718	0,4623839	2,18269719	5,89560884	0,03741147	0,23376571	TOGARAM1	TOG array regulator of axonemal microtubules 1 [Source:HGNC Symbol;Acc:HGNC:19959]
ENSG00000122863	0,45141832	4,84560801	5,89242581	0,03745217	0,23384785	CHST3	carbohydrate sulfotransferase 3 [Source:HGNC Symbol;Acc:HGNC:1971]
ENSG00000166224	0,42542943	4,97191512	5,89207699	0,03745663	0,23384785	SGPL1	sphingosine-1-phosphate lyase 1 [Source:HGNC Symbol;Acc:HGNC:10817]
ENSG00000064652	-0,5539832	3,49212689	5,88855409	0,03750175	0,23392956	SNX24	sorting nexin 24 [Source:HGNC Symbol;Acc:HGNC:21533]
ENSG00000113583	0,39350446	6,49735259	5,88914027	0,03749424	0,23392956	C5orf15	chromosome 5 open reading frame 15 [Source:HGNC Symbol;Acc:HGNC:20656]
ENSG00000072803	0,35586523	4,63638557	5,8871574	0,03751965	0,23394136	FBXW11	F-box and WD repeat domain containing 11 [Source:HGNC Symbol;Acc:HGNC:13607]
ENSG00000105778	-0,3522939	4,67043731	5,88123895	0,03759564	0,2341689	AVL9	AVL9 cell migration associated [Source:HGNC Symbol;Acc:HGNC:28994]
ENSG00000134809	-0,404427	6,13914389	5,8817359	0,03758925	0,2341689	TIMM10	translocase of inner mitochondrial membrane 10 [Source:HGNC Symbol;Acc:HGNC:11814]
ENSG00000174903	0,32088054	6,83283381	5,88057056	0,03760423	0,2341689	RAB1B	RAB1B, member RAS oncogene family [Source:HGNC Symbol;Acc:HGNC:18370]
ENSG00000141505	-0,3896379	9,31820011	5,87388407	0,03769033	0,23460507	ASGR1	asialoglycoprotein receptor 1 [Source:HGNC Symbol;Acc:HGNC:742]
ENSG00000187837	0,44197026	3,89236511	5,871626	0,03771947	0,23468641	H1-2	H1.2 linker histone, cluster member [Source:HGNC Symbol;Acc:HGNC:4716]
ENSG00000246705	-0,4427743	4,95991813	5,86041745	0,03786449	0,23548841	H2AJ	H2A.J histone [Source:HGNC Symbol;Acc:HGNC:14456]
ENSG00000237371	-1,0138834	-1,0636582	5,85798748	0,03789602	0,23558422	AL355803.1	novel transcript
ENSG00000165219	0,47403248	4,86224317	5,85262183	0,03796576	0,23581706	GAPVD1	GTPase activating protein and VPS9 domains 1 [Source:HGNC Symbol;Acc:HGNC:23375]
ENSG00000280077	1,20837723	-1,6047521	5,85340033	0,03795563	0,23581706	AL353763.2	TEC
ENSG00000140416	-0,3289945	6,65158973	5,84516842	0,03806289	0,23631994	TPM1	tropomyosin 1 [Source:HGNC Symbol;Acc:HGNC:12010]
ENSG00000114779	-0,3547696	5,20159321	5,84377387	0,0380811	0,23633256	ABHD14B	abhydrolase domain containing 14B [Source:HGNC Symbol;Acc:HGNC:28235]
ENSG00000163885	-1,9186442	-2,1329201	5,84010323	0,03812908	0,23652984	CFAP100	cilia and flagella associated protein 100 [Source:HGNC Symbol;Acc:HGNC:26842]
ENSG00000139697	0,37610551	4,75094386	5,83661547	0,03817474	0,23671257	SBNO1	strawberry notch homolog 1 [Source:HGNC Symbol;Acc:HGNC:22973]
ENSG00000166016	0,38075204	4,29760329	5,8214084	0,03837462	0,23764933	ABTB2	ankyrin repeat and BTB domain containing 2 [Source:HGNC Symbol;Acc:HGNC:23842]
ENSG00000214106	0,92815033	0,0379082	5,82199233	0,03836692	0,23764933	PAXIP1-AS2	PAXIP1 antisense RNA 2 [Source:HGNC Symbol;Acc:HGNC:48958]
ENSG00000255522	-1,8409139	-2,1309527	5,82312631	0,03835197	0,23764933	SNRPCP5	small nuclear ribonucleoprotein polypeptide C pseudogene 5 [Source:HGNC Symbol;Acc:HGNC:49820]
ENSG00000184897	-0,3536166	6,18454214	5,80756468	0,0385577	0,23868196	H1-10	H1.10 linker histone [Source:HGNC Symbol;Acc:HGNC:4722]
ENSG00000133028	0,34982054	6,30397626	5,803599	0,03861034	0,23880547	SCO1	synthesis of cytochrome C oxidase 1 [Source:HGNC Symbol;Acc:HGNC:10603]
ENSG00000136231	-0,373583	5,28894656	5,80360345	0,03861028	0,23880547	IGF2BP3	insulin like growth factor 2 mRNA binding protein 3 [Source:HGNC Symbol;Acc:HGNC:28868]
ENSG00000119953	0,38600088	4,8270375	5,80203044	0,03863119	0,23882941	SMNDC1	survival motor neuron domain containing 1 [Source:HGNC Symbol;Acc:HGNC:16900]
ENSG00000204963	-1,493675	-1,2420987	5,8008484	0,03864691	0,23882941	PCDHA7	protocadherin alpha 7 [Source:HGNC Symbol;Acc:HGNC:8673]
ENSG00000151923	0,35217771	6,85260582	5,799373	0,03866654	0,2388497	TIAL1	TIA1 cytotoxic granule associated RNA binding protein like 1 [Source:HGNC Symbol;Acc:HGNC:11804]
ENSG00000042493	-0,4140154	4,05070272	5,79370001	0,03874215	0,23911449	CAPG	capping actin protein, gelsolin like [Source:HGNC Symbol;Acc:HGNC:1474]
ENSG00000162616	-0,5292762	2,11072051	5,7943538	0,03873342	0,23911449	DNAJB4	DnaJ heat shock protein family (Hsp40) member B4 [Source:HGNC Symbol;Acc:HGNC:14886]
ENSG00000182197	0,39563401	5,77339235	5,79125617	0,03877477	0,23921479	EXT1	exostosin glycosyltransferase 1 [Source:HGNC Symbol;Acc:HGNC:3512]
ENSG00000100239	-0,3165442	6,12593166	5,7871668	0,03882944	0,23945093	PPP6R2	protein phosphatase 6 regulatory subunit 2 [Source:HGNC Symbol;Acc:HGNC:19253]
ENSG00000159128	0,3340627	6,43421758	5,78445392	0,03886576	0,23957378	IFNGR2	interferon gamma receptor 2 [Source:HGNC Symbol;Acc:HGNC:5440]
ENSG00000186198	-0,4053039	4,2925028	5,78180838	0,03890122	0,23969121	SLC51B	solute carrier family 51 subunit beta [Source:HGNC Symbol;Acc:HGNC:29956]
ENSG00000067369	0,491938	4,14490172	5,77895282	0,03893953	0,23982616	TP53BP1	tumor protein p53 binding protein 1 [Source:HGNC Symbol;Acc:HGNC:11999]
ENSG00000127980	0,43656325	3,34877224	5,77576807	0,03898233	0,23988744	PEX1	peroxisomal biogenesis factor 1 [Source:HGNC Symbol;Acc:HGNC:8850]
ENSG00000133466	-0,6111888	1,23297649	5,77661673	0,03897092	0,23988744	C1QTNF6	C1q and TNF related 6 [Source:HGNC Symbol;Acc:HGNC:14343]
ENSG00000161980	-0,342365	5,79222535	5,773767	0,03900924	0,23995201	POLR3K	RNA polymerase III subunit K [Source:HGNC Symbol;Acc:HGNC:14121]

ENSG00000196839	-0,5358873	1,91075686	5,77189753	0,03903441	0,24000576	ADA	adenosine deaminase [Source:HGNC Symbol;Acc:HGNC:186]
ENSG00000100461	-0,8266976	0,40765738	5,75961679	0,03920024	0,24024021	RBM23	RNA binding motif protein 23 [Source:HGNC Symbol;Acc:HGNC:20155]
ENSG00000112941	0,37447404	3,9501696	5,75858873	0,03921416	0,24024021	TENT4A	terminal nucleotidyltransferase 4A [Source:HGNC Symbol;Acc:HGNC:16705]
ENSG00000114988	0,3327576	6,84656506	5,76635934	0,03910908	0,24024021	LMAN2L	lectin, mannose binding 2 like [Source:HGNC Symbol;Acc:HGNC:19263]
ENSG00000115524	0,45558977	8,09567772	5,76282127	0,03915688	0,24024021	SF3B1	splicing factor 3b subunit 1 [Source:HGNC Symbol;Acc:HGNC:10768]
ENSG00000126106	-0,434757	4,69882503	5,75893099	0,03920952	0,24024021	TMEM53	transmembrane protein 53 [Source:HGNC Symbol;Acc:HGNC:26186]
ENSG00000162882	-0,3549123	4,74819255	5,76087566	0,0391832	0,24024021	HAAO	3-hydroxyanthranilate 3,4-dioxygenase [Source:HGNC Symbol;Acc:HGNC:4796]
ENSG00000170634	-0,652619	2,44341619	5,7604007	0,03918963	0,24024021	ACYP2	acylphosphatase 2 [Source:HGNC Symbol;Acc:HGNC:180]
ENSG00000175785	0,50851362	2,08383946	5,76122059	0,03917853	0,24024021	PRIMA1	proline rich membrane anchor 1 [Source:HGNC Symbol;Acc:HGNC:18319]
ENSG00000205268	0,51280275	3,33685247	5,75811759	0,03922054	0,24024021	PDE7A	phosphodiesterase 7A [Source:HGNC Symbol;Acc:HGNC:8791]
ENSG00000075399	-0,3991769	3,24984846	5,75529235	0,03925884	0,24024108	VPS9D1	VPS9 domain containing 1 [Source:HGNC Symbol;Acc:HGNC:13526]
ENSG00000100625	0,54099598	1,62568524	5,7544683	0,03927002	0,24024108	SIX4	SIX homeobox 4 [Source:HGNC Symbol;Acc:HGNC:10890]
ENSG00000219863	-1,0610043	-0,1317896	5,75501591	0,03926259	0,24024108	RPL31P28	ribosomal protein L31 pseudogene 28 [Source:HGNC Symbol;Acc:HGNC:36253]
ENSG00000224470	0,59076591	3,32114662	5,75223157	0,03930038	0,2403262	ATXN1L	ataxin 1 like [Source:HGNC Symbol;Acc:HGNC:33279]
ENSG00000196188	-0,7789522	0,15839312	5,75001621	0,03933048	0,24036399	CTSE	cathepsin E [Source:HGNC Symbol;Acc:HGNC:2530]
ENSG00000197149	-1,147094	-1,0620948	5,74935542	0,03933947	0,24036399	AC107956.1	ribosomal protein S2 (RPS2) pseudogene
ENSG00000159166	-0,400524	6,44305698	5,74774927	0,03936132	0,24039694	LAD1	ladinin 1 [Source:HGNC Symbol;Acc:HGNC:6472]
ENSG00000270878	-1,2944466	-1,9270204	5,74258819	0,03943163	0,24072571	GCATP1	glycine C-acetyltransferase pseudogene 1 [Source:HGNC Symbol;Acc:HGNC:20013]
ENSG00000140691	-0,4450715	4,58793394	5,74012466	0,03946524	0,24083028	ARMC5	armadillo repeat containing 5 [Source:HGNC Symbol;Acc:HGNC:25781]
ENSG00000169684	-0,4740338	3,1040491	5,73770139	0,03949834	0,24093163	CHRNA5	cholinergic receptor nicotinic alpha 5 subunit [Source:HGNC Symbol;Acc:HGNC:1959]
ENSG00000008853	0,44309657	2,71685428	5,73625329	0,03951814	0,24095178	RHOBTB2	Rho related BTB domain containing 2 [Source:HGNC Symbol;Acc:HGNC:18756]
ENSG00000047315	0,38329255	6,37402738	5,73000496	0,0396037	0,24135008	POLR2B	RNA polymerase II subunit B [Source:HGNC Symbol;Acc:HGNC:9188]
ENSG00000130803	0,35209114	4,72101722	5,72786712	0,03963302	0,24135008	ZNF317	zinc finger protein 317 [Source:HGNC Symbol;Acc:HGNC:13507]
ENSG00000138036	0,40395566	3,77134631	5,72809606	0,03962988	0,24135008	DYNC2LI1	dynein cytoplasmic 2 light intermediate chain 1 [Source:HGNC Symbol;Acc:HGNC:24595]
ENSG00000043514	0,42145174	4,92412309	5,72458583	0,03967809	0,24143866	TRIT1	tRNA isopentenyltransferase 1 [Source:HGNC Symbol;Acc:HGNC:20286]
ENSG00000151694	-0,4216917	4,98735132	5,72440148	0,03968062	0,24143866	ADAM17	ADAM metallopeptidase domain 17 [Source:HGNC Symbol;Acc:HGNC:195]
ENSG00000263002	0,48765041	2,13370546	5,72088479	0,039729	0,24163234	ZNF234	zinc finger protein 234 [Source:HGNC Symbol;Acc:HGNC:13027]
ENSG00000078304	0,33667921	5,57435487	5,71377902	0,03982696	0,24172499	PPP2R5C	protein phosphatase 2 regulatory subunit B'gamma [Source:HGNC Symbol;Acc:HGNC:9311]
ENSG00000103647	-0,7691038	0,31153911	5,71447554	0,03981734	0,24172499	CORO2B	coronin 2B [Source:HGNC Symbol;Acc:HGNC:2256]
ENSG00000144677	-0,3687422	5,03506563	5,71837248	0,0397636	0,24172499	CTDSPL	CTD small phosphatase like [Source:HGNC Symbol;Acc:HGNC:16890]
ENSG00000162441	-0,4404181	5,35582587	5,71608404	0,03979515	0,24172499	LZIC	leucine zipper and CTNNBIP1 domain containing [Source:HGNC Symbol;Acc:HGNC:17497]
ENSG00000221916	-0,8453692	-0,1685256	5,71610325	0,03979488	0,24172499	C19orf73	chromosome 19 open reading frame 73 [Source:HGNC Symbol;Acc:HGNC:25534]
ENSG00000110717	-0,3432698	7,27476296	5,7100487	0,03987851	0,24184785	NDUFS8	NADH:ubiquinone oxidoreductase core subunit S8 [Source:HGNC Symbol;Acc:HGNC:7715]
ENSG00000165312	0,85873644	-0,1069248	5,70991828	0,03988031	0,24184785	OTUD1	OTU deubiquitinase 1 [Source:HGNC Symbol;Acc:HGNC:27346]
ENSG00000151552	-0,3133195	6,62775106	5,70641969	0,03992873	0,24204103	QDPR	quinoid dihydropteridine reductase [Source:HGNC Symbol;Acc:HGNC:9752]
ENSG00000137103	-0,4468662	2,24286512	5,70458826	0,03995411	0,24209441	TMEM8B	transmembrane protein 8B [Source:HGNC Symbol;Acc:HGNC:21427]
ENSG00000129534	0,37875505	4,03667742	5,70186476	0,03999189	0,24212246	MIS18BP1	MIS18 binding protein 1 [Source:HGNC Symbol;Acc:HGNC:20190]
ENSG00000196396	0,33208395	5,51218203	5,70207236	0,03998901	0,24212246	PTPN1	protein tyrosine phosphatase non-receptor type 1 [Source:HGNC Symbol;Acc:HGNC:9642]
ENSG00000146242	1,26990392	0,78112303	5,69104842	0,04014235	0,24283215	TPBG	trophoblast glycoprotein [Source:HGNC Symbol;Acc:HGNC:12004]
ENSG00000167005	0,33237582	7,09781506	5,69141702	0,04013721	0,24283215	NUDT21	nudix hydrolase 21 [Source:HGNC Symbol;Acc:HGNC:13870]
ENSG00000185561	-1,0147533	-0,4394803	5,68951492	0,04016374	0,24286098	TLCD2	TLC domain containing 2 [Source:HGNC Symbol;Acc:HGNC:33522]
ENSG00000277879	1,83755565	-1,9196474	5,68633102	0,0402082	0,24302919	AL391988.1	novel transcript, antisense to SLC18A2
ENSG00000169891	1,51364428	-2,2405504	5,67745396	0,04033246	0,24347808	REPS2	RALBP1 associated Eps domain containing 2 [Source:HGNC Symbol;Acc:HGNC:9963]
ENSG00000213613	-0,7492471	-0,0855285	5,67944316	0,04030458	0,24347808	RPL11P3	ribosomal protein L11 pseudogene 3 [Source:HGNC Symbol;Acc:HGNC:36342]

ENSG00000239559	-0,4795082	1,69533873	5,67833085	0,04032017	0,24347808	RPL37P2	ribosomal protein L37 pseudogene 2 [Source:HGNC Symbol;Acc:HGNC:17092]
ENSG00000182149	0,34088151	7,82721391	5,67506155	0,04036603	0,24358009	IST1	IST1 factor associated with ESCRT-III [Source:HGNC Symbol;Acc:HGNC:28977]
ENSG00000204257	0,66574354	0,44765293	5,67047774	0,04043045	0,24386808	HLA-DMA	major histocompatibility complex, class II, DM alpha [Source:HGNC Symbol;Acc:HGNC:4934]
ENSG00000117597	0,47456811	4,14504207	5,66923001	0,04044801	0,24387329	UTP25	UTP25 small subunit processor component [Source:HGNC Symbol;Acc:HGNC:28440]
ENSG00000114933	0,72964137	1,78274693	5,66386489	0,04052361	0,24400661	INO80D	INO80 complex subunit D [Source:HGNC Symbol;Acc:HGNC:25997]
ENSG00000129521	-1,0025174	0,60181025	5,66411265	0,04052012	0,24400661	EGLN3	egl-9 family hypoxia inducible factor 3 [Source:HGNC Symbol;Acc:HGNC:14661]
ENSG00000153721	0,37282105	4,17296136	5,66055533	0,04057034	0,24400661	CNKSR3	CNKSR family member 3 [Source:HGNC Symbol;Acc:HGNC:23034]
ENSG00000159069	-0,4108845	5,84890819	5,66100308	0,04056401	0,24400661	FBXW5	F-box and WD repeat domain containing 5 [Source:HGNC Symbol;Acc:HGNC:13613]
ENSG00000164898	-0,4984882	1,48019693	5,66207535	0,04054887	0,24400661	FMC1	formation of mitochondrial complex V assembly factor 1 homolog [Source:HGNC Symbol;Acc:HGNC:26946]
ENSG00000242615	1,09208217	-0,7404421	5,66586192	0,04049545	0,24400661	AC022415.1	ribosomal protein L17 (RPL17) pseudogene
ENSG00000109436	0,379568	3,98658477	5,65081146	0,04070829	0,24423302	TBC1D9	TBC1 domain family member 9 [Source:HGNC Symbol;Acc:HGNC:21710]
ENSG00000135912	0,3318202	6,22805272	5,65231493	0,04068697	0,24423302	TTLL4	tubulin tyrosine ligase like 4 [Source:HGNC Symbol;Acc:HGNC:28976]
ENSG00000183161	0,35709054	3,79382143	5,6524057	0,04068568	0,24423302	FANCF	FA complementation group F [Source:HGNC Symbol;Acc:HGNC:3587]
ENSG00000185842	0,56416204	1,9419443	5,65642294	0,04062877	0,24423302	DNAH14	dynein axonemal heavy chain 14 [Source:HGNC Symbol;Acc:HGNC:2945]
ENSG00000230392	1,54849643	-2,0195007	5,6511047	0,04070413	0,24423302	AC004835.1	novel transcript
ENSG00000254221	1,04513821	-0,7846646	5,65350583	0,04067009	0,24423302	PCDHGB1	protocadherin gamma subfamily B, 1 [Source:HGNC Symbol;Acc:HGNC:8708]
ENSG00000230629	-0,4111315	2,84949658	5,64936673	0,04072879	0,24425572	RPS23P8	ribosomal protein S23 pseudogene 8 [Source:HGNC Symbol;Acc:HGNC:35982]
ENSG00000002919	0,34452851	4,0982816	5,64261381	0,0408248	0,24466365	SNX11	sorting nexin 11 [Source:HGNC Symbol;Acc:HGNC:14975]
ENSG00000236439	-0,6489127	0,75659004	5,64222707	0,04083031	0,24466365	AC099336.2	ribosomal protein S27 (metallopanstimulin 1) (RPS27) pseudogene
ENSG00000163521	-0,4262311	2,55998115	5,64094165	0,04084862	0,24467301	GLB1L	galactosidase beta 1 like [Source:HGNC Symbol;Acc:HGNC:28129]
ENSG00000108387	-1,5404895	-1,9100389	5,63371658	0,04095172	0,24519006	SEPTIN4	septin 4 [Source:HGNC Symbol;Acc:HGNC:9165]
ENSG00000206557	0,37286559	5,79219474	5,62913798	0,04101723	0,24548166	TRIM71	tripartite motif containing 71 [Source:HGNC Symbol;Acc:HGNC:32669]
ENSG00000088038	-0,3562756	5,60385102	5,62425639	0,04108722	0,24550822	CNOT3	CCR4-NOT transcription complex subunit 3 [Source:HGNC Symbol;Acc:HGNC:7879]
ENSG00000116819	0,98884477	-0,6806383	5,62413977	0,04108889	0,24550822	TFAP2E	transcription factor AP-2 epsilon [Source:HGNC Symbol;Acc:HGNC:30774]
ENSG00000120647	0,41345084	3,65202615	5,62730758	0,04104346	0,24550822	CCDC77	coiled-coil domain containing 77 [Source:HGNC Symbol;Acc:HGNC:28203]
ENSG00000261978	-0,7346084	-0,125925	5,62590655	0,04106354	0,24550822	AC116025.1	novel transcript, antisense to TBC1D16
ENSG00000115685	-0,4390364	5,97682645	5,6175562	0,04118352	0,24597301	PPP1R7	protein phosphatase 1 regulatory subunit 7 [Source:HGNC Symbol;Acc:HGNC:9295]
ENSG00000127337	0,47689811	4,65839227	5,61427281	0,04123081	0,24615484	YEATS4	YEATS domain containing 4 [Source:HGNC Symbol;Acc:HGNC:24859]
ENSG00000175324	-0,38764	5,06082163	5,61247857	0,04125668	0,2461615	LSM1	LSM1 homolog, mRNA degradation associated [Source:HGNC Symbol;Acc:HGNC:20472]
ENSG00000188959	1,23084838	-0,1304761	5,6118586	0,04126563	0,2461615	C9orf152	chromosome 9 open reading frame 152 [Source:HGNC Symbol;Acc:HGNC:31455]
ENSG00000274270	1,15947466	-1,313834	5,60216671	0,04140576	0,24689666	AL137060.3	novel transcript, sense intronic to DLEU1
ENSG00000152904	-0,484318	2,68402339	5,59320969	0,0415358	0,24736916	GGPS1	geranylgeranyl diphosphate synthase 1 [Source:HGNC Symbol;Acc:HGNC:4249]
ENSG00000158864	0,4371936	7,6843371	5,59388839	0,04152593	0,24736916	NDUFS2	NADH:ubiquinone oxidoreductase core subunit S2 [Source:HGNC Symbol;Acc:HGNC:7708]
ENSG00000165424	-0,5207139	2,18622123	5,59375324	0,0415279	0,24736916	ZCCHC24	zinc finger CCHC-type containing 24 [Source:HGNC Symbol;Acc:HGNC:26911]
ENSG00000092929	-0,3956489	5,61604838	5,59009375	0,04158116	0,24753838	UNC13D	unc-13 homolog D [Source:HGNC Symbol;Acc:HGNC:23147]
ENSG00000226328	-0,7954024	-0,1891396	5,58863166	0,04160246	0,24756432	NUP50-DT	NUP50 divergent transcript [Source:HGNC Symbol;Acc:HGNC:50502]
ENSG00000224093	-0,5052008	3,5404928	5,5838513	0,04167221	0,24784632	BCAR3-AS1	BCAR3 antisense RNA 1 [Source:HGNC Symbol;Acc:HGNC:40093]
ENSG00000276980	-0,5173753	2,5652366	5,58305912	0,04168378	0,24784632	AC008760.2	novel transcript, sense intronic C3
ENSG00000124257	-0,4662416	2,16902486	5,57803083	0,04175733	0,24818261	NEURL2	neuralized E3 ubiquitin protein ligase 2 [Source:HGNC Symbol;Acc:HGNC:16156]
ENSG00000119231	0,36471586	4,09156033	5,57620518	0,04178408	0,24824057	SENP5	SUMO specific peptidase 5 [Source:HGNC Symbol;Acc:HGNC:28407]
ENSG00000135503	0,47098552	4,8092748	5,57121308	0,04185731	0,24857458	ACVR1B	activin A receptor type 1B [Source:HGNC Symbol;Acc:HGNC:172]
ENSG00000148450	-0,4481763	3,47314211	5,56802555	0,04190416	0,24865062	MSRB2	methionine sulfoxide reductase B2 [Source:HGNC Symbol;Acc:HGNC:17061]
ENSG00000173465	-0,375576	4,96363933	5,56888086	0,04189158	0,24865062	ZNRD2	zinc ribbon domain containing 2 [Source:HGNC Symbol;Acc:HGNC:11328]
ENSG00000248008	0,43997055	2,45102866	5,56542942	0,04194236	0,24877626	NRAV	negative regulator of antiviral response [Source:HGNC Symbol;Acc:HGNC:48588]

ENSG00000128607	0,4070957	4,00095317	5,55029242	0,04216596	0,25000104	KLHDC10	kelch domain containing 10 [Source:HGNC Symbol;Acc:HGNC:22194]
ENSG00000140943	0,33556836	6,47893457	5,54575767	0,04223324	0,25029833	MBTPS1	membrane bound transcription factor peptidase, site 1 [Source:HGNC Symbol;Acc:HGNC:15456]
ENSG00000091009	0,44859635	1,8285275	5,54169874	0,04229357	0,25055423	RBM27	RNA binding motif protein 27 [Source:HGNC Symbol;Acc:HGNC:29243]
ENSG00000173889	0,53624544	2,4245011	5,53560417	0,04238436	0,25099029	PHC3	polyhomeotic homolog 3 [Source:HGNC Symbol;Acc:HGNC:15682]
ENSG00000176438	0,60998513	2,22134659	5,53131357	0,04244842	0,25126779	SYNE3	spectrin repeat containing nuclear envelope family member 3 [Source:HGNC Symbol;Acc:HGNC:19861]
ENSG00000168591	0,38903001	5,2905293	5,53009559	0,04246662	0,25127375	TMUB2	transmembrane and ubiquitin like domain containing 2 [Source:HGNC Symbol;Acc:HGNC:28459]
ENSG00000143374	0,45901871	5,6778133	5,52857454	0,04248937	0,25130657	TARS2	threonyl-tRNA synthetase 2, mitochondrial [Source:HGNC Symbol;Acc:HGNC:30740]
ENSG00000186364	-0,5037859	1,22038924	5,52508661	0,0425416	0,25151365	NUDT17	nudix hydrolase 17 [Source:HGNC Symbol;Acc:HGNC:26618]
ENSG00000120913	-0,4155237	3,77917993	5,52270104	0,04257737	0,25162327	PDLIM2	PDZ and LIM domain 2 [Source:HGNC Symbol;Acc:HGNC:13992]
ENSG00000109270	0,45314681	4,67864713	5,51878922	0,0426361	0,25170625	LAMTOR3	late endosomal/lysosomal adaptor, MAPK and MTOR activator 3 [Source:HGNC Symbol;Acc:HGNC:15606]
ENSG00000162302	-0,3898031	4,89570918	5,51893511	0,0426339	0,25170625	RPS6KA4	ribosomal protein S6 kinase A4 [Source:HGNC Symbol;Acc:HGNC:10433]
ENSG00000166401	-0,6404141	0,56084353	5,51717732	0,04266032	0,25170625	SERPINB8	serpin family B member 8 [Source:HGNC Symbol;Acc:HGNC:8952]
ENSG00000187688	-0,5995354	5,08595876	5,51773774	0,0426519	0,25170625	TRPV2	transient receptor potential cation channel subfamily V member 2 [Source:HGNC Symbol;Acc:HGNC:18082]
ENSG00000100227	0,3683154	6,86667114	5,51201639	0,04273802	0,25192206	POLDIP3	DNA polymerase delta interacting protein 3 [Source:HGNC Symbol;Acc:HGNC:23782]
ENSG00000110660	0,37021951	4,83062015	5,51193001	0,04273932	0,25192206	SLC35F2	solute carrier family 35 member F2 [Source:HGNC Symbol;Acc:HGNC:23615]
ENSG00000114374	0,43928512	2,66362778	5,51131211	0,04274863	0,25192206	USP9Y	ubiquitin specific peptidase 9 Y-linked [Source:HGNC Symbol;Acc:HGNC:12633]
ENSG00000130244	-0,3749572	2,72957634	5,50291178	0,04287551	0,25223098	FAM98C	family with sequence similarity 98 member C [Source:HGNC Symbol;Acc:HGNC:27119]
ENSG00000146755	-0,376742	4,28303988	5,50212706	0,04288738	0,25223098	TRIM50	tripartite motif containing 50 [Source:HGNC Symbol;Acc:HGNC:19017]
ENSG00000148120	0,33811857	4,21474165	5,50623835	0,04282521	0,25223098	AOPEP	aminopeptidase O (putative) [Source:HGNC Symbol;Acc:HGNC:1361]
ENSG00000176095	0,3247352	5,34517187	5,50307211	0,04287308	0,25223098	IP6K1	inositol hexakisphosphate kinase 1 [Source:HGNC Symbol;Acc:HGNC:18360]
ENSG00000277443	0,42420035	5,47956533	5,50410655	0,04285743	0,25223098	MARCKS	myristoylated alanine rich protein kinase C substrate [Source:HGNC Symbol;Acc:HGNC:6759]
ENSG00000136156	0,39742507	7,92694116	5,49918866	0,04293189	0,25239112	ITM2B	integral membrane protein 2B [Source:HGNC Symbol;Acc:HGNC:6174]
ENSG00000184436	-0,4096463	4,22624709	5,49758966	0,04295613	0,25243206	THAP7	THAP domain containing 7 [Source:HGNC Symbol;Acc:HGNC:23190]
ENSG00000126226	-0,3147955	5,94919996	5,49458115	0,04300179	0,25256383	PCID2	PCI domain containing 2 [Source:HGNC Symbol;Acc:HGNC:25653]
ENSG00000138823	0,55370139	5,41811225	5,49383446	0,04301313	0,25256383	MTTP	microsomal triglyceride transfer protein [Source:HGNC Symbol;Acc:HGNC:7467]
ENSG00000136522	-0,3230813	6,84466728	5,49055867	0,04306293	0,25272799	MRPL47	mitochondrial ribosomal protein L47 [Source:HGNC Symbol;Acc:HGNC:16652]
ENSG00000138629	0,32810035	6,24713413	5,48972032	0,04307569	0,25272799	UBL7	ubiquitin like 7 [Source:HGNC Symbol;Acc:HGNC:28221]
ENSG00000163739	1,62753145	-1,6392405	5,48614159	0,04313019	0,2529462	CXCL1	C-X-C motif chemokine ligand 1 [Source:HGNC Symbol;Acc:HGNC:4602]
ENSG00000151575	0,83039859	0,46843637	5,48429914	0,04315829	0,25298025	TEX9	testis expressed 9 [Source:HGNC Symbol;Acc:HGNC:29585]
ENSG00000247095	-1,061409	-0,6239342	5,4834902	0,04317063	0,25298025	MIR210HG	MIR210 host gene [Source:HGNC Symbol;Acc:HGNC:39524]
ENSG00000218426	-0,4060548	4,10316767	5,48178996	0,04319659	0,25303086	AL590867.2	60S ribosomal protein L27a (RPL27A) pseudogene
ENSG00000133056	0,59353855	2,20753867	5,47743716	0,04326313	0,25321756	PIK3C2B	phosphatidylinositol-4-phosphate 3-kinase catalytic subunit type 2 beta [Source:HGNC Symbol;Acc:HGNC:8972]
ENSG00000170876	0,39181653	6,72312787	5,478431	0,04324792	0,25321756	TMEM43	transmembrane protein 43 [Source:HGNC Symbol;Acc:HGNC:28472]
ENSG00000175745	0,41194575	3,93725476	5,47629511	0,04328061	0,25321842	NR2F1	nuclear receptor subfamily 2 group F member 1 [Source:HGNC Symbol;Acc:HGNC:7975]
ENSG00000114654	1,31724494	-1,1394739	5,47016942	0,04337451	0,25366622	EFCC1	EF-hand and coiled-coil domain containing 1 [Source:HGNC Symbol;Acc:HGNC:25692]
ENSG00000018869	0,88124485	-0,5876808	5,46730487	0,04341851	0,25382193	ZNF582	zinc finger protein 582 [Source:HGNC Symbol;Acc:HGNC:26421]
ENSG00000269837	0,94295461	-1,0226644	5,4658054	0,04344156	0,25385512	IPO5P1	importin 5 pseudogene 1 [Source:HGNC Symbol;Acc:HGNC:49687]
ENSG00000100299	-0,4143337	5,04260914	5,46143675	0,04350882	0,25385948	ARSA	arylsulfatase A [Source:HGNC Symbol;Acc:HGNC:713]
ENSG00000115966	0,3897898	4,26740427	5,46293903	0,04348568	0,25385948	ATF2	activating transcription factor 2 [Source:HGNC Symbol;Acc:HGNC:784]
ENSG00000141034	0,43356395	2,53282336	5,46124202	0,04351182	0,25385948	GID4	GID complex subunit 4 homolog [Source:HGNC Symbol;Acc:HGNC:28453]
ENSG00000279672	0,5025424	2,10183172	5,46170821	0,04350463	0,25385948	AP006621.5	TEC
ENSG00000121417	0,37207288	3,11847653	5,45983185	0,04353356	0,25388492	ZNF211	zinc finger protein 211 [Source:HGNC Symbol;Acc:HGNC:13003]
ENSG00000100225	0,31314639	7,13579404	5,45744948	0,04357031	0,25395852	FBXO7	F-box protein 7 [Source:HGNC Symbol;Acc:HGNC:13586]
ENSG00000197299	0,37795431	2,98983964	5,45676078	0,04358094	0,25395852	BLM	BLM RecQ like helicase [Source:HGNC Symbol;Acc:HGNC:1058]

ENSG00000125656	-0,3147721	6,00189494	5,45398845	0,04362378	0,25410676	CLPP	caseinolytic mitochondrial matrix peptidase proteolytic subunit [Source:HGNC Symbol;Acc:HGNC:2084]
ENSG00000133657	0,46844964	3,93493353	5,45097236	0,04367044	0,25427717	ATP13A3	ATPase 13A3 [Source:HGNC Symbol;Acc:HGNC:24113]
ENSG00000136807	-0,3317727	5,24926128	5,44970792	0,04369002	0,25428982	CDK9	cyclin dependent kinase 9 [Source:HGNC Symbol;Acc:HGNC:1780]
ENSG00000128881	0,6175169	0,94961149	5,44328876	0,04378958	0,25476783	TTBK2	tau tubulin kinase 2 [Source:HGNC Symbol;Acc:HGNC:19141]
ENSG00000168827	0,48161021	5,82748359	5,43555711	0,04390988	0,25516288	GFM1	G elongation factor mitochondrial 1 [Source:HGNC Symbol;Acc:HGNC:13780]
ENSG00000175311	-0,4429576	5,3905915	5,43557317	0,04390963	0,25516288	ANKS4B	ankyrin repeat and sterile alpha motif domain containing 4B [Source:HGNC Symbol;Acc:HGNC:26795]
ENSG00000228929	-0,3886553	2,44650945	5,43607126	0,04390187	0,25516288	RPS13P2	ribosomal protein S13 pseudogene 2 [Source:HGNC Symbol;Acc:HGNC:36364]
ENSG00000161395	-0,376324	3,44348014	5,43306138	0,0439488	0,25518603	PGAP3	post-GPI attachment to proteins phospholipase 3 [Source:HGNC Symbol;Acc:HGNC:23719]
ENSG00000228253	0,4419238	5,78278866	5,43399748	0,0439342	0,25518603	MT-ATP8	mitochondrially encoded ATP synthase membrane subunit 8 [Source:HGNC Symbol;Acc:HGNC:7415]
ENSG00000112237	0,3678364	6,35569362	5,42729376	0,04403891	0,25541301	CCNC	cyclin C [Source:HGNC Symbol;Acc:HGNC:1581]
ENSG00000117614	0,33901146	5,07640323	5,42608471	0,04405783	0,25541301	SYF2	SYF2 pre-mRNA splicing factor [Source:HGNC Symbol;Acc:HGNC:19824]
ENSG00000133665	1,15925657	-0,5269089	5,42611742	0,04405732	0,25541301	DYDC2	DPY30 domain containing 2 [Source:HGNC Symbol;Acc:HGNC:23468]
ENSG00000134324	-0,5196788	3,39345349	5,42633839	0,04405386	0,25541301	LPIN1	lipin 1 [Source:HGNC Symbol;Acc:HGNC:13345]
ENSG00000008869	0,43820282	3,45605275	5,42352135	0,04409797	0,255443	HEATR5B	HEAT repeat containing 5B [Source:HGNC Symbol;Acc:HGNC:29273]
ENSG00000158480	0,40884448	4,08413158	5,42397819	0,04409081	0,255443	SPATA2	spermatogenesis associated 2 [Source:HGNC Symbol;Acc:HGNC:14681]
ENSG00000188811	-0,3475888	3,97896118	5,41811663	0,04418276	0,25583269	NHLRC3	NHL repeat containing 3 [Source:HGNC Symbol;Acc:HGNC:33751]
ENSG00000065615	-0,4031538	3,46297528	5,4139037	0,04424899	0,25601325	CYB5R4	cytochrome b5 reductase 4 [Source:HGNC Symbol;Acc:HGNC:20147]
ENSG00000274588	-0,4072868	3,43550951	5,41396699	0,04424799	0,25601325	DGKK	diacylglycerol kinase kappa [Source:HGNC Symbol;Acc:HGNC:32395]
ENSG00000094804	-0,3390177	6,90129507	5,408857	0,04432849	0,2563369	CDC6	cell division cycle 6 [Source:HGNC Symbol;Acc:HGNC:1744]
ENSG00000214367	0,43338134	2,74990641	5,40812602	0,04434002	0,2563369	HAUS3	HAUS augmin like complex subunit 3 [Source:HGNC Symbol;Acc:HGNC:28719]
ENSG00000263535	0,71379162	0,36675364	5,40541959	0,04438274	0,25648238	AK4P1	adenylate kinase 4 pseudogene 1 [Source:HGNC Symbol;Acc:HGNC:364]
ENSG00000121210	0,44202826	3,32094603	5,39984091	0,04447096	0,25662337	TMEM131L	transmembrane 131 like [Source:HGNC Symbol;Acc:HGNC:29146]
ENSG00000162521	0,3269903	6,95332236	5,39943423	0,0444774	0,25662337	RBBP4	RB binding protein 4, chromatin remodeling factor [Source:HGNC Symbol;Acc:HGNC:9887]
ENSG00000163617	1,37698192	-1,4120561	5,40053666	0,04445995	0,25662337	CCDC191	coiled-coil domain containing 191 [Source:HGNC Symbol;Acc:HGNC:29272]
ENSG00000267226	-0,7722715	-0,1456729	5,40208544	0,04443544	0,25662337	AC104971.1	novel transcript, antisense to MALT1
ENSG00000196776	0,53921267	2,51497017	5,39653835	0,04452329	0,25678673	CD47	CD47 molecule [Source:HGNC Symbol;Acc:HGNC:1682]
ENSG00000138081	0,33296423	4,98677034	5,38841038	0,04465241	0,25712536	FBXO11	F-box protein 11 [Source:HGNC Symbol;Acc:HGNC:13590]
ENSG00000163694	-0,5987689	1,7866118	5,39011919	0,04462523	0,25712536	RBM47	RNA binding motif protein 47 [Source:HGNC Symbol;Acc:HGNC:30358]
ENSG00000182934	0,42917644	7,98680639	5,3903787	0,0446211	0,25712536	SRPRA	SRP receptor subunit alpha [Source:HGNC Symbol;Acc:HGNC:11307]
ENSG00000250479	-0,3600683	5,63276622	5,3884408	0,04465193	0,25712536	CHCHD10	coiled-coil-helix-coiled-coil-helix domain containing 10 [Source:HGNC Symbol;Acc:HGNC:15559]
ENSG00000136717	-0,7320515	0,39293178	5,38611259	0,04468899	0,25723464	BIN1	bridging integrator 1 [Source:HGNC Symbol;Acc:HGNC:1052]
ENSG00000074276	-0,462408	5,05778356	5,36314968	0,04505667	0,25768165	CDHR2	cadherin related family member 2 [Source:HGNC Symbol;Acc:HGNC:18231]
ENSG00000093010	-0,3285625	7,9784172	5,37220539	0,04491122	0,25768165	COMT	catechol-O-methyltransferase [Source:HGNC Symbol;Acc:HGNC:2228]
ENSG00000102302	0,40730184	2,43074218	5,36311899	0,04505716	0,25768165	FGD1	FYVE, RhoGEF and PH domain containing 1 [Source:HGNC Symbol;Acc:HGNC:3663]
ENSG00000104432	1,55267075	-0,4078186	5,36532653	0,04502165	0,25768165	IL7	interleukin 7 [Source:HGNC Symbol;Acc:HGNC:6023]
ENSG00000111144	0,34404759	6,57484482	5,37524481	0,04486254	0,25768165	LTA4H	leukotriene A4 hydrolase [Source:HGNC Symbol;Acc:HGNC:6710]
ENSG00000115446	0,37974621	6,68261544	5,37419512	0,04487935	0,25768165	UNC50	unc-50 inner nuclear membrane RNA binding protein [Source:HGNC Symbol;Acc:HGNC:16046]
ENSG00000124588	-0,4255518	5,70733051	5,3657089	0,04501551	0,25768165	NQO2	N-ribosyldihydronicotinamide:quinone reductase 2 [Source:HGNC Symbol;Acc:HGNC:7856]
ENSG00000127720	0,51381513	1,83038507	5,36789549	0,04498037	0,25768165	METTL25	methyltransferase like 25 [Source:HGNC Symbol;Acc:HGNC:26228]
ENSG00000132383	0,40975064	6,95728783	5,36051245	0,04509913	0,25768165	RPA1	replication protein A1 [Source:HGNC Symbol;Acc:HGNC:10289]
ENSG00000156603	-0,3580626	3,96332548	5,36404756	0,04504222	0,25768165	MED19	mediator complex subunit 19 [Source:HGNC Symbol;Acc:HGNC:29600]
ENSG00000166855	0,46098289	5,68820231	5,36597479	0,04501123	0,25768165	CLPX	caseinolytic mitochondrial matrix peptidase chaperone subunit X [Source:HGNC Symbol;Acc:HGNC:2088]
ENSG00000183765	-0,3225937	5,17772054	5,35849585	0,04513164	0,25768165	CHEK2	checkpoint kinase 2 [Source:HGNC Symbol;Acc:HGNC:16627]
ENSG00000184508	-0,4164194	5,1410988	5,36403809	0,04504237	0,25768165	HDDC3	HD domain containing 3 [Source:HGNC Symbol;Acc:HGNC:30522]

ENSG00000186026	0,94998144	-0,9598378	5,36428142	0,04503846	0,25768165	ZNF284	zinc finger protein 284 [Source:HGNC Symbol;Acc:HGNC:13078]
ENSG00000186523	-0,8975548	0,60878787	5,36013789	0,04510517	0,25768165	FAM86B1	family with sequence similarity 86 member B1 [Source:HGNC Symbol;Acc:HGNC:28268]
ENSG00000197982	-0,3485764	5,1747519	5,36141959	0,04508452	0,25768165	C1orf122	chromosome 1 open reading frame 122 [Source:HGNC Symbol;Acc:HGNC:24789]
ENSG00000219932	-1,0504982	-0,7758991	5,37904035	0,04480183	0,25768165	RPL12P8	ribosomal protein L12 pseudogene 8 [Source:HGNC Symbol;Acc:HGNC:23735]
ENSG00000221886	0,50685986	1,07890241	5,35815966	0,04513706	0,25768165	ZBED8	zinc finger BED-type containing 8 [Source:HGNC Symbol;Acc:HGNC:30804]
ENSG00000223822	-1,0229427	-1,0033721	5,37429496	0,04487775	0,25768165	EEF1A1P1	eukaryotic translation elongation factor 1 alpha 1 pseudogene 1 [Source:HGNC Symbol;Acc:HGNC:3191]
ENSG00000225489	-0,8606312	-0,4272344	5,37729184	0,04482979	0,25768165	AL354707.1	novel transcript
ENSG00000240540	-0,5803791	0,71570427	5,36126459	0,04508702	0,25768165	RPL3P9	ribosomal protein L3 pseudogene 9 [Source:HGNC Symbol;Acc:HGNC:36839]
ENSG00000006432	0,38360083	3,34727235	5,3559222	0,04517317	0,25778704	MAP3K9	mitogen-activated protein kinase kinase kinase 9 [Source:HGNC Symbol;Acc:HGNC:6861]
ENSG00000142937	-0,3156734	11,0060715	5,35293768	0,04522139	0,25793152	RPS8	ribosomal protein S8 [Source:HGNC Symbol;Acc:HGNC:10441]
ENSG00000179981	0,47472467	1,73506774	5,35217006	0,0452338	0,25793152	TSHZ1	teashirt zinc finger homeobox 1 [Source:HGNC Symbol;Acc:HGNC:10669]
ENSG00000060982	-0,3824361	4,76662758	5,34164252	0,04540445	0,25795869	BCAT1	branched chain amino acid transaminase 1 [Source:HGNC Symbol;Acc:HGNC:976]
ENSG00000088305	0,42520261	4,60822845	5,34128019	0,04541034	0,25795869	DNMT3B	DNA methyltransferase 3 beta [Source:HGNC Symbol;Acc:HGNC:2979]
ENSG00000117475	0,44002352	3,46213768	5,34410035	0,04536454	0,25795869	BLZF1	basic leucine zipper nuclear factor 1 [Source:HGNC Symbol;Acc:HGNC:1065]
ENSG00000123384	-0,8042767	6,71613922	5,34348845	0,04537447	0,25795869	LRP1	LDL receptor related protein 1 [Source:HGNC Symbol;Acc:HGNC:6692]
ENSG00000147676	0,5371045	6,24090104	5,33966184	0,04543664	0,25795869	MAL2	mal, T cell differentiation protein 2 [Source:HGNC Symbol;Acc:HGNC:13634]
ENSG00000154309	-0,490162	1,92540984	5,33539993	0,04550602	0,25795869	DISP1	dispatched RND transporter family member 1 [Source:HGNC Symbol;Acc:HGNC:19711]
ENSG00000166851	0,33202977	8,24887295	5,33447544	0,04552108	0,25795869	PLK1	polo like kinase 1 [Source:HGNC Symbol;Acc:HGNC:9077]
ENSG00000169155	0,56542652	2,29291349	5,33838719	0,04545738	0,25795869	ZBTB43	zinc finger and BTB domain containing 43 [Source:HGNC Symbol;Acc:HGNC:17908]
ENSG00000180822	-0,4256451	4,42425778	5,34528406	0,04534533	0,25795869	PSMG4	proteasome assembly chaperone 4 [Source:HGNC Symbol;Acc:HGNC:21108]
ENSG00000196730	0,32732008	4,60507252	5,33749204	0,04547195	0,25795869	DAPK1	death associated protein kinase 1 [Source:HGNC Symbol;Acc:HGNC:2674]
ENSG00000198952	0,32577203	6,46131861	5,33918436	0,04544441	0,25795869	SMG5	SMG5 nonsense mediated mRNA decay factor [Source:HGNC Symbol;Acc:HGNC:24644]
ENSG00000204356	-0,2999788	7,10985459	5,34311606	0,04538051	0,25795869	NELFE	negative elongation factor complex member E [Source:HGNC Symbol;Acc:HGNC:13974]
ENSG00000228274	-0,8525492	0,22672353	5,33772588	0,04546814	0,25795869	AL021707.2	novel transcript, antisense to CBY1
ENSG00000275494	-0,781344	0,15807005	5,33476359	0,04551639	0,25795869	AC133552.5	novel transcript
ENSG00000277232	1,23198974	-1,9814742	5,3409619	0,04541551	0,25795869	GTSE1-DT	GTSE1 divergent transcript [Source:HGNC Symbol;Acc:HGNC:27163]
ENSG00000280789	-0,391809	2,99831699	5,33995115	0,04543194	0,25795869	PAGR1	PAXIP1 associated glutamate rich protein 1 [Source:HGNC Symbol;Acc:HGNC:28707]
ENSG00000117226	-1,352912	-1,6479325	5,33018191	0,04559113	0,25805536	GBP3	guanylate binding protein 3 [Source:HGNC Symbol;Acc:HGNC:4184]
ENSG00000185201	-0,3673399	5,28778325	5,33122222	0,04557415	0,25805536	IFITM2	interferon induced transmembrane protein 2 [Source:HGNC Symbol;Acc:HGNC:5413]
ENSG00000262943	0,4423739	1,63818099	5,33187955	0,04556342	0,25805536	ALOX12P2	arachidonate 12-lipoxygenase pseudogene 2 [Source:HGNC Symbol;Acc:HGNC:432]
ENSG00000179935	0,98330084	-0,3665304	5,32838155	0,04562055	0,25812183	LINC00652	long intergenic non-protein coding RNA 652 [Source:HGNC Symbol;Acc:HGNC:25003]
ENSG00000118193	0,37791575	2,76603039	5,32389854	0,04569389	0,25813691	KIF14	kinesin family member 14 [Source:HGNC Symbol;Acc:HGNC:19181]
ENSG00000130935	0,39663872	7,01060886	5,32429607	0,04568738	0,25813691	NOL11	nucleolar protein 11 [Source:HGNC Symbol;Acc:HGNC:24557]
ENSG00000162496	0,36103838	6,30252392	5,3239194	0,04569355	0,25813691	DHRS3	dehydrogenase/reductase 3 [Source:HGNC Symbol;Acc:HGNC:17693]
ENSG00000188785	0,47056538	1,56047883	5,32592135	0,04566078	0,25813691	ZNF548	zinc finger protein 548 [Source:HGNC Symbol;Acc:HGNC:26561]
ENSG00000085831	-0,7665304	-0,0177901	5,32074237	0,04574561	0,25832922	TTC39A	tetratricopeptide repeat domain 39A [Source:HGNC Symbol;Acc:HGNC:18657]
ENSG00000169490	0,31725081	6,85769489	5,31646003	0,04581591	0,2584265	TM2D2	TM2 domain containing 2 [Source:HGNC Symbol;Acc:HGNC:24127]
ENSG00000183763	0,36440512	4,34691656	5,31830452	0,04578562	0,2584265	TRAIP	TRAF interacting protein [Source:HGNC Symbol;Acc:HGNC:30764]
ENSG00000255517	-0,6996592	0,51941801	5,31659051	0,04581377	0,2584265	AP002748.4	novel transcript
ENSG00000125703	-0,5310898	2,34074104	5,31411814	0,04585441	0,25854384	ATG4C	autophagy related 4C cysteine peptidase [Source:HGNC Symbol;Acc:HGNC:16040]
ENSG00000167552	-0,3905265	5,65076795	5,31229024	0,04588449	0,25861362	TUBA1A	tubulin alpha 1a [Source:HGNC Symbol;Acc:HGNC:20766]
ENSG00000174485	0,41781196	3,88092032	5,3109513	0,04590654	0,2586381	DENND4A	DENN domain containing 4A [Source:HGNC Symbol;Acc:HGNC:24321]
ENSG00000234390	-0,742055	0,41206103	5,30782493	0,04595807	0,25882861	USP27X-AS1	USP27X antisense RNA 1 (head to head) [Source:HGNC Symbol;Acc:HGNC:27249]
ENSG00000146678	-0,6041628	8,17740914	5,30429176	0,04601639	0,25886194	IGFBP1	insulin like growth factor binding protein 1 [Source:HGNC Symbol;Acc:HGNC:5469]

ENSG00000168904	-0,4911147	2,62018283	5,30424626	0,04601714	0,25886194	LRRC28	leucine rich repeat containing 28 [Source:HGNC Symbol;Acc:HGNC:28355]
ENSG00000198910	-1,0055304	2,8056106	5,30516574	0,04600196	0,25886194	L1CAM	L1 cell adhesion molecule [Source:HGNC Symbol;Acc:HGNC:6470]
ENSG00000124067	-0,3467373	6,01587771	5,30112477	0,04606875	0,25905249	SLC12A4	solute carrier family 12 member 4 [Source:HGNC Symbol;Acc:HGNC:10913]
ENSG00000125352	-0,3655282	4,22704931	5,29979483	0,04609076	0,25907653	RNF113A	ring finger protein 113A [Source:HGNC Symbol;Acc:HGNC:12974]
ENSG00000087053	0,37023684	4,80151292	5,29178503	0,04622359	0,25936547	MTMR2	myotubularin related protein 2 [Source:HGNC Symbol;Acc:HGNC:7450]
ENSG00000104979	-0,3515416	6,47634654	5,29343198	0,04619624	0,25936547	C19orf53	chromosome 19 open reading frame 53 [Source:HGNC Symbol;Acc:HGNC:24991]
ENSG00000130590	-0,4876146	2,78028141	5,29296155	0,04620405	0,25936547	SAMD10	sterile alpha motif domain containing 10 [Source:HGNC Symbol;Acc:HGNC:16129]
ENSG00000133393	-0,3190513	5,87784083	5,29042684	0,04624616	0,25936547	CEP20	centrosomal protein 20 [Source:HGNC Symbol;Acc:HGNC:26435]
ENSG00000183773	-0,571756	2,91198854	5,29027483	0,04624869	0,25936547	AIFM3	apoptosis inducing factor mitochondria associated 3 [Source:HGNC Symbol;Acc:HGNC:26398]
ENSG00000186952	1,20601055	-1,6370503	5,29118893	0,04623349	0,25936547	TMEM232	transmembrane protein 232 [Source:HGNC Symbol;Acc:HGNC:37270]
ENSG00000286177	-1,0855446	-0,9865202	5,28743412	0,04629594	0,25953085	AC011462.5	novel transcript, antisense to CCDC97 and HNRNPUL1
ENSG00000133119	0,31744567	5,23628492	5,27383415	0,04652301	0,26040905	RFC3	replication factor C subunit 3 [Source:HGNC Symbol;Acc:HGNC:9971]
ENSG00000204592	0,36935558	7,45267755	5,2761731	0,04648386	0,26040905	HLA-E	major histocompatibility complex, class I, E [Source:HGNC Symbol;Acc:HGNC:4962]
ENSG00000213598	-0,6805897	0,00723205	5,27572393	0,04649137	0,26040905	AL049873.1	ribosomal protein L31 (RPL31) pseudogene
ENSG00000229666	-1,8758841	-1,7156232	5,2737811	0,0465239	0,26040905	MAST4-AS1	MAST4 antisense RNA 1 [Source:HGNC Symbol;Acc:HGNC:40865]
ENSG00000124370	-0,3390292	4,6436291	5,27170564	0,04655867	0,26050388	MCEE	methylmalonyl-CoA epimerase [Source:HGNC Symbol;Acc:HGNC:16732]
ENSG00000236914	-1,018504	-1,1167053	5,26866385	0,0466097	0,26068953	LINC01852	long intergenic non-protein coding RNA 1852 [Source:HGNC Symbol;Acc:HGNC:52668]
ENSG00000062370	0,8192947	0,62641516	5,26165291	0,04672757	0,26086758	ZNF112	zinc finger protein 112 [Source:HGNC Symbol;Acc:HGNC:12892]
ENSG00000132463	0,34822954	7,27208457	5,26524938	0,04666705	0,26086758	GRSF1	G-rich RNA sequence binding factor 1 [Source:HGNC Symbol;Acc:HGNC:4610]
ENSG00000152804	0,42409518	6,13446714	5,26216471	0,04671895	0,26086758	HHEX	hematopoietically expressed homeobox [Source:HGNC Symbol;Acc:HGNC:4901]
ENSG00000164845	-0,6978649	0,88430589	5,26039993	0,04674867	0,26086758	FAM86FP	family with sequence similarity 86 member F, pseudogene [Source:HGNC Symbol;Acc:HGNC:42357]
ENSG00000213939	-0,5253109	1,89475874	5,26213621	0,04671943	0,26086758	AC091153.1	ribosomal protein S12 (RPS12) pseudogene
ENSG00000228110	1,08422312	-0,9831291	5,2605054	0,04674689	0,26086758	ST13P19	ST13, Hsp70 interacting protein pseudogene 19 [Source:HGNC Symbol;Acc:HGNC:38862]
ENSG00000237440	0,73055382	0,31220987	5,25932486	0,04676679	0,26086903	ZNF737	zinc finger protein 737 [Source:HGNC Symbol;Acc:HGNC:32468]
ENSG00000056277	-1,0753748	-0,1032626	5,25627101	0,0468183	0,26093189	ZNF280C	zinc finger protein 280C [Source:HGNC Symbol;Acc:HGNC:25955]
ENSG00000115073	-0,3087093	6,91253802	5,25471245	0,04684462	0,26093189	ACTR1B	actin related protein 1B [Source:HGNC Symbol;Acc:HGNC:168]
ENSG00000141385	0,3301279	7,59918712	5,25442322	0,0468495	0,26093189	AFG3L2	AFG3 like matrix AAA peptidase subunit 2 [Source:HGNC Symbol;Acc:HGNC:315]
ENSG00000272419	0,9656174	-1,0609411	5,25485874	0,04684214	0,26093189	LINC01145	long intergenic non-protein coding RNA 1145 [Source:HGNC Symbol;Acc:HGNC:49462]
ENSG00000140675	-1,1153983	-1,4898288	5,24833491	0,04695249	0,26140586	SLC5A2	solute carrier family 5 member 2 [Source:HGNC Symbol;Acc:HGNC:11037]
ENSG00000223768	0,37830652	2,8870474	5,24361886	0,04703247	0,26175135	LINC00205	long intergenic non-protein coding RNA 205 [Source:HGNC Symbol;Acc:HGNC:16420]
ENSG00000227939	-0,3643538	2,77911159	5,23890708	0,04711253	0,26209711	RPL3P2	ribosomal protein L3 pseudogene 2 [Source:HGNC Symbol;Acc:HGNC:17834]
ENSG00000054148	-0,4116163	6,78511651	5,23651362	0,04715327	0,26222389	PHPT1	phosphohistidine phosphatase 1 [Source:HGNC Symbol;Acc:HGNC:30033]
ENSG00000105939	0,56243385	4,44733689	5,23329992	0,04720804	0,26229508	ZC3HAV1	zinc finger CCCH-type containing, antiviral 1 [Source:HGNC Symbol;Acc:HGNC:23721]
ENSG00000181359	-1,3593178	-1,4024516	5,23260249	0,04721994	0,26229508	HSP90AA6P	heat shock protein 90 alpha family class A member 6, pseudogene [Source:HGNC Symbol;Acc:HGNC:32536]
ENSG00000279738	1,38725907	-1,8585824	5,23448058	0,04718791	0,26229508	AL022311.1	novel transcript, sense overlapping to EIF3L
ENSG00000115507	0,50107673	2,50068344	5,22667844	0,04732113	0,2624738	OTX1	orthodenticle homeobox 1 [Source:HGNC Symbol;Acc:HGNC:8521]
ENSG00000160256	-0,3115725	5,70637944	5,22728878	0,04731069	0,2624738	FAM207A	family with sequence similarity 207 member A [Source:HGNC Symbol;Acc:HGNC:15811]
ENSG00000168209	-0,3107259	7,07043183	5,22546211	0,04734194	0,2624738	DDIT4	DNA damage inducible transcript 4 [Source:HGNC Symbol;Acc:HGNC:24944]
ENSG00000176371	0,31998204	4,89441213	5,22633436	0,04732702	0,2624738	ZSCAN2	zinc finger and SCAN domain containing 2 [Source:HGNC Symbol;Acc:HGNC:20994]
ENSG00000258704	-0,4135525	3,12929794	5,22656432	0,04732309	0,2624738	SRP54-AS1	SRP54 antisense RNA 1 (head to head) [Source:HGNC Symbol;Acc:HGNC:50643]
ENSG00000105204	-0,7521848	0,79916576	5,22254512	0,0473919	0,26259553	DYRK1B	dual specificity tyrosine phosphorylation regulated kinase 1B [Source:HGNC Symbol;Acc:HGNC:3092]
ENSG00000107651	0,3614025	5,68821833	5,22027704	0,04743079	0,26259553	SEC23IP	SEC23 interacting protein [Source:HGNC Symbol;Acc:HGNC:17018]
ENSG00000185022	-0,3831802	3,51565468	5,22114577	0,04741589	0,26259553	MAFF	MAF bZIP transcription factor F [Source:HGNC Symbol;Acc:HGNC:6780]
ENSG00000241258	0,38106068	3,51778445	5,21998484	0,0474358	0,26259553	CRCP	CGRP receptor component [Source:HGNC Symbol;Acc:HGNC:17888]

ENSG00000131507	0,43097413	5,55852514	5,21786367	0,04747221	0,26269754	NDFIP1	Nedd4 family interacting protein 1 [Source:HGNC Symbol;Acc:HGNC:17592]
ENSG00000005022	-0,295722	9,20513147	5,21366727	0,04754434	0,26289755	SLC25A5	solute carrier family 25 member 5 [Source:HGNC Symbol;Acc:HGNC:10991]
ENSG00000288586	-1,3725056	-1,8378522	5,21390322	0,04754028	0,26289755	AL357874.3	novel transcript
ENSG00000240652	-0,9652718	-0,2616833	5,2106031	0,0475971	0,26308969	AP001024.1	ribosomal protein, large, P2 (RPLP2) pseudogene
ENSG00000269556	0,49073302	2,10473808	5,20940462	0,04761776	0,26310431	TMEM185A	transmembrane protein 185A [Source:HGNC Symbol;Acc:HGNC:17125]
ENSG00000138604	-0,6051412	1,92427285	5,20549326	0,04768524	0,26327804	GLCE	glucuronic acid epimerase [Source:HGNC Symbol;Acc:HGNC:17855]
ENSG00000148358	0,43628716	5,67657924	5,20568273	0,04768197	0,26327804	GPR107	G protein-coupled receptor 107 [Source:HGNC Symbol;Acc:HGNC:17830]
ENSG00000276570	-1,2386509	-1,399462	5,20338595	0,04772165	0,26337952	AC010327.6	novel transcript
ENSG00000148842	0,34129317	4,88926538	5,2002487	0,04777592	0,26347994	CNNM2	cyclin and CBS domain divalent metal cation transport mediator 2 [Source:HGNC Symbol;Acc:HGNC:103]
ENSG00000165806	0,4153093	5,49047411	5,20113633	0,04776055	0,26347994	CASP7	caspase 7 [Source:HGNC Symbol;Acc:HGNC:1508]
ENSG00000011566	0,41388792	4,72058224	5,19708081	0,04783079	0,26358357	MAP4K3	mitogen-activated protein kinase kinase kinase kinase 3 [Source:HGNC Symbol;Acc:HGNC:6865]
ENSG00000280734	-0,7457349	0,63481036	5,19719339	0,04782884	0,26358357	LINC01232	long intergenic non-protein coding RNA 1232 [Source:HGNC Symbol;Acc:HGNC:49755]
ENSG00000147535	-0,3527927	5,33510394	5,19242613	0,04791156	0,26368827	PLPP5	phospholipid phosphatase 5 [Source:HGNC Symbol;Acc:HGNC:25026]
ENSG00000168792	0,35426073	3,58061977	5,19182601	0,04792199	0,26368827	ABHD15	abhydrolase domain containing 15 [Source:HGNC Symbol;Acc:HGNC:26971]
ENSG00000182912	-0,6754629	0,09415069	5,19207696	0,04791763	0,26368827	TSPEAR-AS2	TSPEAR antisense RNA 2 [Source:HGNC Symbol;Acc:HGNC:16428]
ENSG00000196584	0,44085243	2,69782243	5,19381245	0,04788749	0,26368827	XRCC2	X-ray repair cross complementing 2 [Source:HGNC Symbol;Acc:HGNC:12829]
ENSG00000170469	-0,3761805	2,79720222	5,18902479	0,04797069	0,26385688	SPATA24	spermatogenesis associated 24 [Source:HGNC Symbol;Acc:HGNC:27322]
ENSG00000129460	0,31859092	7,79876411	5,18640475	0,0480163	0,26400835	NGDN	neuroguidin [Source:HGNC Symbol;Acc:HGNC:20271]
ENSG00000171084	-0,741158	0,58750995	5,18510784	0,0480389	0,26403322	FAM86JP	family with sequence similarity 86 member J, pseudogene [Source:HGNC Symbol;Acc:HGNC:44097]
ENSG00000087995	0,3081445	6,08218312	5,18298041	0,048076	0,26413773	METTL2A	methyltransferase like 2A [Source:HGNC Symbol;Acc:HGNC:25755]
ENSG00000076662	-0,4670724	3,81018182	5,18113531	0,0481082	0,26421383	ICAM3	intercellular adhesion molecule 3 [Source:HGNC Symbol;Acc:HGNC:5346]
ENSG00000109381	0,36184707	4,39809555	5,17992016	0,04812942	0,26421383	ELF2	E74 like ETS transcription factor 2 [Source:HGNC Symbol;Acc:HGNC:3317]
ENSG00000228594	0,41711783	4,01503046	5,17907977	0,0481441	0,26421383	FNDC10	fibronectin type III domain containing 10 [Source:HGNC Symbol;Acc:HGNC:42951]
ENSG00000139180	0,31940656	5,35564363	5,17550737	0,04820659	0,26435811	NDUFA9	NADH:ubiquinone oxidoreductase subunit A9 [Source:HGNC Symbol;Acc:HGNC:7693]
ENSG00000164638	0,42210013	4,63252676	5,17560112	0,04820494	0,26435811	SLC29A4	solute carrier family 29 member 4 [Source:HGNC Symbol;Acc:HGNC:23097]
ENSG00000163362	0,35187446	3,86954582	5,17346328	0,04824238	0,26445515	INAVA	innate immunity activator [Source:HGNC Symbol;Acc:HGNC:25599]
ENSG00000174684	0,30711666	7,36036778	5,17186645	0,04827037	0,26450931	B4GAT1	beta-1,4-glucuronyltransferase 1 [Source:HGNC Symbol;Acc:HGNC:15685]
ENSG00000119979	-0,3543444	4,28368552	5,16859389	0,04832779	0,26472149	DENND10	DENN domain containing 10 [Source:HGNC Symbol;Acc:HGNC:31793]
ENSG00000273066	0,56111825	1,36639387	5,16759523	0,04834533	0,26472149	AL355987.4	novel transcript
ENSG00000126934	-0,3266387	7,49524467	5,16552959	0,04838163	0,26482101	MAP2K2	mitogen-activated protein kinase kinase 2 [Source:HGNC Symbol;Acc:HGNC:6842]
ENSG00000146278	0,39121997	4,14565596	5,15741522	0,04852457	0,2653052	PNRC1	proline rich nuclear receptor coactivator 1 [Source:HGNC Symbol;Acc:HGNC:17278]
ENSG00000169627	-0,5775722	0,63937504	5,15749737	0,04852312	0,2653052	BOLA2B	bolA family member 2B [Source:HGNC Symbol;Acc:HGNC:32479]
ENSG00000172164	0,30994095	6,52239016	5,15937823	0,04848995	0,2653052	SNTB1	syntrophin beta 1 [Source:HGNC Symbol;Acc:HGNC:11168]
ENSG00000119915	-0,9410191	-0,8672496	5,14398389	0,04876233	0,26645932	ELOVL3	ELOVL fatty acid elongase 3 [Source:HGNC Symbol;Acc:HGNC:18047]
ENSG00000136261	0,30648787	7,28932579	5,14240481	0,04879038	0,26645932	BZW2	basic leucine zipper and W2 domains 2 [Source:HGNC Symbol;Acc:HGNC:18808]
ENSG00000177455	-0,6144718	0,70936602	5,14311544	0,04877776	0,26645932	CD19	CD19 molecule [Source:HGNC Symbol;Acc:HGNC:1633]
ENSG00000134824	0,34186865	6,37226484	5,14134554	0,04880921	0,26646253	FADS2	fatty acid desaturase 2 [Source:HGNC Symbol;Acc:HGNC:3575]
ENSG00000126246	-0,9505866	0,41963966	5,13859858	0,04885807	0,26662965	IGFLR1	IGF like family receptor 1 [Source:HGNC Symbol;Acc:HGNC:23620]
ENSG00000148290	-0,3197349	6,2039884	5,1318675	0,04897806	0,26708492	SURF1	SURF1 cytochrome c oxidase assembly factor [Source:HGNC Symbol;Acc:HGNC:11474]
ENSG00000184481	0,43303718	1,63919845	5,13247566	0,04896721	0,26708492	FOXO4	forkhead box O4 [Source:HGNC Symbol;Acc:HGNC:7139]
ENSG00000092470	0,48533488	2,48994997	5,12743428	0,04905729	0,26718349	WDR76	WD repeat domain 76 [Source:HGNC Symbol;Acc:HGNC:25773]
ENSG00000175536	-0,5270433	1,10259871	5,12580777	0,0490864	0,26718349	LIPT2	lipoyl(octanoyl) transferase 2 [Source:HGNC Symbol;Acc:HGNC:37216]
ENSG00000187325	0,54865751	3,55772563	5,12752171	0,04905573	0,26718349	TAF9B	TATA-box binding protein associated factor 9b [Source:HGNC Symbol;Acc:HGNC:17306]
ENSG00000272273	-0,8228935	0,47640319	5,125203	0,04909723	0,26718349	IER3-AS1	IER3 antisense RNA 1 [Source:HGNC Symbol;Acc:HGNC:53629]

ENSG00000275964	1,5424234	-1,7984739	5,12472042	0,04910587	0,26718349	AL355001.2	novel transcript
ENSG00000278730	0,4213666	2,59291349	5,12759613	0,0490544	0,26718349	AC005332.6	novel transcript
ENSG00000165471	0,47268171	2,76126985	5,11864728	0,0492148	0,26744198	MBL2	mannose binding lectin 2 [Source:HGNC Symbol;Acc:HGNC:6922]
ENSG00000170017	0,36065051	5,39675404	5,12067232	0,04917845	0,26744198	ALCAM	activated leukocyte cell adhesion molecule [Source:HGNC Symbol;Acc:HGNC:400]
ENSG00000189164	0,55611443	0,68459301	5,11799013	0,04922661	0,26744198	ZNF527	zinc finger protein 527 [Source:HGNC Symbol;Acc:HGNC:29385]
ENSG00000197142	-0,4750231	7,6345631	5,11966866	0,04919646	0,26744198	ACSL5	acyl-CoA synthetase long chain family member 5 [Source:HGNC Symbol;Acc:HGNC:16526]
ENSG00000137266	0,33372041	4,91924388	5,11645197	0,04925425	0,2674927	SLC22A23	solute carrier family 22 member 23 [Source:HGNC Symbol;Acc:HGNC:21106]
ENSG00000145220	-0,316119	6,42543001	5,11506119	0,04927926	0,26752908	LYAR	Ly1 antibody reactive [Source:HGNC Symbol;Acc:HGNC:26021]
ENSG00000105928	-0,3534903	4,14901059	5,10769724	0,04941197	0,26764391	GSDME	gasdermin E [Source:HGNC Symbol;Acc:HGNC:2810]
ENSG00000127589	-0,4277209	2,65030969	5,10896043	0,04938917	0,26764391	TUBBP1	tubulin beta pseudogene 1 [Source:HGNC Symbol;Acc:HGNC:12414]
ENSG00000127824	-0,3957268	2,15200375	5,10575845	0,04944698	0,26764391	TUBA4A	tubulin alpha 4a [Source:HGNC Symbol;Acc:HGNC:12407]
ENSG00000132854	0,36167169	4,21258508	5,10700255	0,04942451	0,26764391	KANK4	KN motif and ankyrin repeat domains 4 [Source:HGNC Symbol;Acc:HGNC:27263]
ENSG00000182718	-0,3228673	6,47848644	5,10656294	0,04943245	0,26764391	ANXA2	annexin A2 [Source:HGNC Symbol;Acc:HGNC:537]
ENSG00000198513	1,10666263	-1,1087873	5,1109125	0,04935397	0,26764391	ATL1	atlastin GTPase 1 [Source:HGNC Symbol;Acc:HGNC:11231]
ENSG00000260806	-0,7448217	0,92859325	5,10694884	0,04942548	0,26764391	AL163051.1	novel transcript, antisense to PAPOLA
ENSG00000273259	-0,6265377	0,26199362	5,10706349	0,04942341	0,26764391	AL049839.2	novel protein
ENSG00000139350	0,3516317	3,27825685	5,10271532	0,049502	0,2677433	NEDD1	NEDD1 gamma-tubulin ring complex targeting factor [Source:HGNC Symbol;Acc:HGNC:7723]
ENSG00000261012	-0,9235587	0,25422152	5,10288352	0,04949895	0,2677433	AC115619.1	novel transcript
ENSG00000052802	0,36052432	7,60787556	5,0948015	0,04964543	0,26792392	MSMO1	methylsterol monooxygenase 1 [Source:HGNC Symbol;Acc:HGNC:10545]
ENSG00000121858	0,57215389	1,8586499	5,09740327	0,04959822	0,26792392	TNFSF10	TNF superfamily member 10 [Source:HGNC Symbol;Acc:HGNC:11925]
ENSG00000158062	0,41091754	3,43516	5,09844228	0,04957938	0,26792392	UBXN11	UBX domain protein 11 [Source:HGNC Symbol;Acc:HGNC:30600]
ENSG00000159625	-0,954738	-1,0660528	5,09482729	0,04964496	0,26792392	DRC7	dynein regulatory complex subunit 7 [Source:HGNC Symbol;Acc:HGNC:25289]
ENSG00000178917	0,64751609	1,20911567	5,09727358	0,04960057	0,26792392	ZNF852	zinc finger protein 852 [Source:HGNC Symbol;Acc:HGNC:27713]
ENSG00000263843	-0,5040056	1,33714123	5,09525402	0,04963721	0,26792392	AC022211.2	novel transcript, antisense to SLC25A19
ENSG00000244313	-0,3546737	4,19453628	5,09323415	0,0496739	0,26797857	AC024293.1	ribosomal protein S10 (RPS10) pseudogene
ENSG00000198650	-0,6224369	1,36937773	5,09131764	0,04970874	0,26806752	TAT	tyrosine aminotransferase [Source:HGNC Symbol;Acc:HGNC:11573]
ENSG00000120690	0,41620707	4,91750798	5,08879723	0,0497546	0,26821583	ELF1	E74 like ETS transcription factor 1 [Source:HGNC Symbol;Acc:HGNC:3316]
ENSG00000135218	0,93230642	-0,4489981	5,0864765	0,04979687	0,26834471	CD36	CD36 molecule [Source:HGNC Symbol;Acc:HGNC:1663]
ENSG00000231770	-0,8351877	0,81893547	5,08355698	0,04985012	0,26853259	TMEM44-AS1	TMEM44 antisense RNA 1 [Source:HGNC Symbol;Acc:HGNC:44272]
ENSG00000155016	-0,4962353	1,52410872	5,08244601	0,0498704	0,26854282	CYP2U1	cytochrome P450 family 2 subfamily U member 1 [Source:HGNC Symbol;Acc:HGNC:20582]
ENSG00000108219	0,33865286	7,16261055	5,07923428	0,04992909	0,26875978	TSPAN14	tetraspanin 14 [Source:HGNC Symbol;Acc:HGNC:23303]
ENSG00000234498	-0,4408223	1,65460059	5,07810423	0,04994976	0,26877201	RPL13AP20	ribosomal protein L13a pseudogene 20 [Source:HGNC Symbol;Acc:HGNC:35709]
ENSG00000175573	-0,3643465	4,6211187	5,07677955	0,049974	0,26880345	C11orf68	chromosome 11 open reading frame 68 [Source:HGNC Symbol;Acc:HGNC:28801]
ENSG00000100003	-0,5210583	1,06503242	5,07028773	0,05009302	0,26911957	SEC14L2	SEC14 like lipid binding 2 [Source:HGNC Symbol;Acc:HGNC:10699]
ENSG00000198055	-0,2995762	5,7170908	5,07229702	0,05005615	0,26911957	GRK6	G protein-coupled receptor kinase 6 [Source:HGNC Symbol;Acc:HGNC:4545]
ENSG00000235663	-1,0728513	-0,9694347	5,07148225	0,05007109	0,26911957	SAPCD1-AS1	SAPCD1 antisense RNA 1 [Source:HGNC Symbol;Acc:HGNC:39824]
ENSG00000269825	1,19148717	-1,4631868	5,06955607	0,05010646	0,26911957	AC022150.4	novel transcript, sense intronic to ZNF83
ENSG00000146700	-0,4542429	4,70844549	5,06798095	0,0501354	0,26917605	SSC4D	scavenger receptor cysteine rich family member with 4 domains [Source:HGNC Symbol;Acc:HGNC:14461]
ENSG00000007516	-0,8115669	-0,1167127	5,06117431	0,0502607	0,26928059	BAIAP3	BAI1 associated protein 3 [Source:HGNC Symbol;Acc:HGNC:948]
ENSG00000136930	-0,310282	8,21891349	5,06259363	0,05023454	0,26928059	PSMB7	proteasome 20S subunit beta 7 [Source:HGNC Symbol;Acc:HGNC:9544]
ENSG00000154114	0,43703988	2,23108544	5,06122118	0,05025984	0,26928059	TBCEL	tubulin folding cofactor E like [Source:HGNC Symbol;Acc:HGNC:28115]
ENSG00000155660	-0,4836509	10,6380417	5,06259562	0,0502345	0,26928059	PDIA4	protein disulfide isomerase family A member 4 [Source:HGNC Symbol;Acc:HGNC:30167]
ENSG00000215450	-1,3256566	-2,1562702	5,06389591	0,05021055	0,26928059	AL022342.1	ribosomal protein S2 (RPS2) pseudogene
ENSG00000265437	-1,3087578	-1,4008196	5,06091603	0,05026546	0,26928059	AP005212.1	family with sequence similarity 207, member A (FAM207A) pseudogene

ENSG00000122741	-0,3749913	2,82731939	5,05563909	0,05036289	0,26952146	DCAF10	DDB1 and CUL4 associated factor 10 [Source:HGNC Symbol;Acc:HGNC:23686]
ENSG00000231826	-1,2197717	-1,2717987	5,05590882	0,0503579	0,26952146	LINC01819	long intergenic non-protein coding RNA 1819 [Source:HGNC Symbol;Acc:HGNC:52624]
ENSG00000259431	-0,4621583	2,07719017	5,05548298	0,05036577	0,26952146	THTPA	thiamine triphosphatase [Source:HGNC Symbol;Acc:HGNC:18987]
ENSG00000124574	0,41636058	4,93336951	5,05340235	0,05040425	0,26952992	ABCC10	ATP binding cassette subfamily C member 10 [Source:HGNC Symbol;Acc:HGNC:52]
ENSG00000261557	-0,4271938	2,28055999	5,05353181	0,05040186	0,26952992	EEF1A1P38	eukaryotic translation elongation factor 1 alpha 1 pseudogene 38 [Source:HGNC Symbol;Acc:HGNC:37916]
ENSG00000182459	0,39929536	2,411509	5,0513672	0,05044193	0,26963268	TEX19	testis expressed 19 [Source:HGNC Symbol;Acc:HGNC:33802]
ENSG00000112473	0,32178871	9,22614945	5,04995427	0,0504681	0,26967394	SLC39A7	solute carrier family 39 member 7 [Source:HGNC Symbol;Acc:HGNC:4927]
ENSG00000070018	0,44853398	4,93321028	5,04346749	0,0505885	0,26981445	LRP6	LDL receptor related protein 6 [Source:HGNC Symbol;Acc:HGNC:6698]
ENSG00000092096	-0,9170013	-0,5693047	5,04467155	0,05056613	0,26981445	SLC22A17	solute carrier family 22 member 17 [Source:HGNC Symbol;Acc:HGNC:23095]
ENSG00000133687	-0,6594927	0,31714384	5,03859912	0,05067909	0,26981445	TMTC1	transmembrane O-mannosyltransferase targeting cadherins 1 [Source:HGNC Symbol;Acc:HGNC:24099]
ENSG00000143033	0,47785439	4,58472051	5,03886839	0,05067408	0,26981445	MTF2	metal response element binding transcription factor 2 [Source:HGNC Symbol;Acc:HGNC:29535]
ENSG00000168566	0,37660261	5,27579065	5,03937666	0,05066461	0,26981445	SNRNP48	small nuclear ribonucleoprotein U11/U12 subunit 48 [Source:HGNC Symbol;Acc:HGNC:21368]
ENSG00000173418	-0,2917216	6,33469428	5,04105048	0,05063345	0,26981445	NAA20	N-alpha-acetyltransferase 20, NatB catalytic subunit [Source:HGNC Symbol;Acc:HGNC:15908]
ENSG00000178035	-0,3132669	8,58784629	5,0419522	0,05061667	0,26981445	IMPDH2	inosine monophosphate dehydrogenase 2 [Source:HGNC Symbol;Acc:HGNC:6053]
ENSG00000196696	0,84973908	-0,8062737	5,04157275	0,05062373	0,26981445	PDXDC2P-NPIPB14P	nuclear pore complex-interacting protein [Source:NCBI gene (formerly Entrezgene);Acc:283970]
ENSG00000197747	-0,3584298	6,11174279	5,04198012	0,05061616	0,26981445	S100A10	S100 calcium binding protein A10 [Source:HGNC Symbol;Acc:HGNC:10487]
ENSG00000250988	-0,7274418	0,40187885	5,04176843	0,05062009	0,26981445	SNHG21	small nucleolar RNA host gene 21 [Source:HGNC Symbol;Acc:HGNC:50284]
ENSG00000183258	-0,291154	6,21791503	5,03557646	0,05073544	0,26996394	DDX41	DEAD-box helicase 41 [Source:HGNC Symbol;Acc:HGNC:18674]
ENSG00000218582	-0,4154475	2,10182412	5,03511077	0,05074413	0,26996394	GAPDHP63	glyceraldehyde 3 phosphate dehydrogenase pseudogene 63 [Source:HGNC Symbol;Acc:HGNC:4157]
ENSG00000023287	-0,3665913	3,98221781	5,03306903	0,05078224	0,27006836	RB1CC1	RB1 inducible coiled-coil 1 [Source:HGNC Symbol;Acc:HGNC:15574]
ENSG00000129128	-0,302273	6,36175436	5,0277883	0,05088099	0,27039663	SPCS3	signal peptidase complex subunit 3 [Source:HGNC Symbol;Acc:HGNC:26212]
ENSG00000184465	0,55847257	2,03508662	5,02790807	0,05087874	0,27039663	WDR27	WD repeat domain 27 [Source:HGNC Symbol;Acc:HGNC:21248]
ENSG00000140750	0,40934846	6,00831026	5,02616517	0,05091139	0,27045979	ARHGAP17	Rho GTPase activating protein 17 [Source:HGNC Symbol;Acc:HGNC:18239]
ENSG00000107242	0,58379989	4,29765197	5,02199693	0,05098955	0,27077659	PIP5K1B	phosphatidylinositol-4-phosphate 5-kinase type 1 beta [Source:HGNC Symbol;Acc:HGNC:8995]
ENSG00000183624	0,29982316	6,2014673	5,02050484	0,05101757	0,27082693	HMCES	5-hydroxymethylcytosine binding, ES cell specific [Source:HGNC Symbol;Acc:HGNC:24446]
ENSG00000049130	0,67927027	0,42571662	5,01674028	0,05108835	0,27093655	KITLG	KIT ligand [Source:HGNC Symbol;Acc:HGNC:6343]
ENSG00000055917	0,34651963	5,02184515	5,01744604	0,05107507	0,27093655	PUM2	pumilio RNA binding family member 2 [Source:HGNC Symbol;Acc:HGNC:14958]
ENSG00000090447	0,34409744	3,73705271	5,01200968	0,05117746	0,27093655	TFAP4	transcription factor AP-4 [Source:HGNC Symbol;Acc:HGNC:11745]
ENSG00000103042	0,3709201	4,60789086	5,01058755	0,05120428	0,27093655	SLC38A7	solute carrier family 38 member 7 [Source:HGNC Symbol;Acc:HGNC:25582]
ENSG00000148110	0,43511743	4,82809146	5,01054248	0,05120514	0,27093655	MFSD14B	major facilitator superfamily domain containing 14B [Source:HGNC Symbol;Acc:HGNC:23376]
ENSG00000163635	0,42528825	2,07450997	5,01285221	0,05116157	0,27093655	ATXN7	ataxin 7 [Source:HGNC Symbol;Acc:HGNC:10560]
ENSG00000244625	1,14096922	-1,7260614	5,01189238	0,05117967	0,27093655	MIATNB	MIAT neighbor [Source:HGNC Symbol;Acc:HGNC:50731]
ENSG00000273270	-0,7152414	0,05154523	5,01386547	0,05114248	0,27093655	AC090114.2	novel transcript
ENSG00000280145	-1,0566967	-1,1304561	5,01161236	0,05118495	0,27093655	CU638689.4	novel transcript
ENSG00000283103	-0,5962851	2,39101113	5,00901779	0,05123392	0,27099069	AC010642.2	novel transcript, antisense to ERVK3-1
ENSG00000079557	1,43950588	-1,230718	5,00683549	0,05127515	0,27111061	AFM	afamin [Source:HGNC Symbol;Acc:HGNC:316]
ENSG00000165240	-0,5750703	0,65571589	5,00436566	0,05132186	0,27125942	ATP7A	ATPase copper transporting alpha [Source:HGNC Symbol;Acc:HGNC:869]
ENSG00000106665	-0,5278519	4,2947513	5,00108366	0,05138401	0,27139156	CLIP2	CAP-Gly domain containing linker protein 2 [Source:HGNC Symbol;Acc:HGNC:2586]
ENSG00000143869	0,33196775	4,00971887	5,00158467	0,05137452	0,27139156	GDF7	growth differentiation factor 7 [Source:HGNC Symbol;Acc:HGNC:4222]
ENSG00000162032	0,35591804	3,72345484	4,99938155	0,05141629	0,27146387	SPSB3	splA/ryanodine receptor domain and SOCS box containing 3 [Source:HGNC Symbol;Acc:HGNC:30629]
ENSG00000229214	0,73225863	-0,0718931	4,99260842	0,05154495	0,27204488	LINC00242	long intergenic non-protein coding RNA 242 [Source:HGNC Symbol;Acc:HGNC:21249]
ENSG00000163820	0,69607865	2,60164051	4,98477392	0,05169429	0,2727345	FYCO1	FYVE and coiled-coil domain autophagy adaptor 1 [Source:HGNC Symbol;Acc:HGNC:14673]
ENSG00000240376	-0,3906929	2,09211915	4,98312588	0,05172577	0,27280207	AC010343.1	ribosomal protein S8 (RPS8) pseudogene
ENSG00000169062	-0,3561184	5,34972407	4,98142255	0,05175833	0,2728753	UPF3A	UPF3A regulator of nonsense mediated mRNA decay [Source:HGNC Symbol;Acc:HGNC:20332]

ENSG00000233369	0,47293428	2,85800157	4,98022597	0,05178122	0,27289749	GTF2IP4	general transcription factor IIi pseudogene 4 [Source:HGNC Symbol;Acc:HGNC:51716]
ENSG00000104164	0,40711419	4,01678551	4,97454077	0,05189015	0,27295516	BLOC1S6	biogenesis of lysosomal organelles complex 1 subunit 6 [Source:HGNC Symbol;Acc:HGNC:8549]
ENSG00000110092	-0,3402507	6,66954278	4,97615542	0,05185918	0,27295516	CCND1	cyclin D1 [Source:HGNC Symbol;Acc:HGNC:1582]
ENSG00000112996	0,36647659	5,44562309	4,96894292	0,05199769	0,27295516	MRPS30	mitochondrial ribosomal protein S30 [Source:HGNC Symbol;Acc:HGNC:8769]
ENSG00000130703	0,32032006	5,73771253	4,97779152	0,05182783	0,27295516	OSBPL2	oxysterol binding protein like 2 [Source:HGNC Symbol;Acc:HGNC:15761]
ENSG00000144354	0,39610218	5,25624968	4,97314648	0,05191691	0,27295516	CDCA7	cell division cycle associated 7 [Source:HGNC Symbol;Acc:HGNC:14628]
ENSG00000168062	0,39357673	2,84132252	4,96963744	0,05198433	0,27295516	BATF2	basic leucine zipper ATF-like transcription factor 2 [Source:HGNC Symbol;Acc:HGNC:25163]
ENSG00000181915	-0,3524642	3,6526113	4,96896545	0,05199725	0,27295516	ADO	2-aminoethanethiol dioxygenase [Source:HGNC Symbol;Acc:HGNC:23506]
ENSG00000187372	-0,7858129	0,57684879	4,96895699	0,05199742	0,27295516	PCDHB13	protocadherin beta 13 [Source:HGNC Symbol;Acc:HGNC:8684]
ENSG00000198399	0,46270107	3,18869795	4,9751339	0,05187877	0,27295516	ITSN2	intersectin 2 [Source:HGNC Symbol;Acc:HGNC:6184]
ENSG00000213176	-0,7568769	-0,0627817	4,96984022	0,05198043	0,27295516	RPL13P6	ribosomal protein L13 pseudogene 6 [Source:HGNC Symbol;Acc:HGNC:19809]
ENSG00000213862	-0,38391	2,67132623	4,97309434	0,05191791	0,27295516	AC044787.1	ribosomal protein L7a (RPL7A) pseudogene
ENSG00000011260	0,36934247	6,81010553	4,96707354	0,05203366	0,27299031	UTP18	UTP18 small subunit processome component [Source:HGNC Symbol;Acc:HGNC:24274]
ENSG00000125611	-0,357361	5,01337966	4,96596424	0,05205502	0,27299031	CHCHD5	coiled-coil-helix-coiled-coil-helix domain containing 5 [Source:HGNC Symbol;Acc:HGNC:17840]
ENSG00000279059	1,18728392	-0,3440859	4,96568277	0,05206044	0,27299031	AC007485.2	TEC
ENSG00000116663	0,41506956	3,84069938	4,96349094	0,05210269	0,2731138	FBXO6	F-box protein 6 [Source:HGNC Symbol;Acc:HGNC:13585]
ENSG00000126005	-0,5416597	2,05097908	4,96195496	0,05213232	0,27317109	MMP24OS	MMP24 opposite strand [Source:HGNC Symbol;Acc:HGNC:44421]
ENSG00000066777	0,3718033	3,84914545	4,95606709	0,05224609	0,27317935	ARFGEF1	ADP ribosylation factor guanine nucleotide exchange factor 1 [Source:HGNC Symbol;Acc:HGNC:15772]
ENSG00000136444	0,30916477	6,16306343	4,95660696	0,05223564	0,27317935	RSAD1	radical S-adenosyl methionine domain containing 1 [Source:HGNC Symbol;Acc:HGNC:25634]
ENSG00000146243	0,62905513	0,8308589	4,95771101	0,05221429	0,27317935	IRAK1BP1	interleukin 1 receptor associated kinase 1 binding protein 1 [Source:HGNC Symbol;Acc:HGNC:17368]
ENSG00000164687	-0,4072722	5,62768364	4,95666582	0,05223451	0,27317935	FABP5	fatty acid binding protein 5 [Source:HGNC Symbol;Acc:HGNC:3560]
ENSG00000196154	-0,49545	2,71930366	4,9596157	0,05217748	0,27317935	S100A4	S100 calcium binding protein A4 [Source:HGNC Symbol;Acc:HGNC:10494]
ENSG00000233762	-0,3680951	3,53942714	4,95705416	0,05222699	0,27317935	RPS15P4	ribosomal protein S15 pseudogene 4 [Source:HGNC Symbol;Acc:HGNC:35712]
ENSG00000251669	-0,7258799	1,17437446	4,95482266	0,05227018	0,27320751	FAM86EP	family with sequence similarity 86 member E, pseudogene [Source:HGNC Symbol;Acc:HGNC:28017]
ENSG00000172732	-0,315345	4,59340689	4,9507584	0,05234894	0,27342803	MUS81	MUS81 structure-specific endonuclease subunit [Source:HGNC Symbol;Acc:HGNC:29814]
ENSG00000230897	-0,3887183	2,165384	4,95071391	0,0523498	0,27342803	RPS18P12	ribosomal protein S18 pseudogene 12 [Source:HGNC Symbol;Acc:HGNC:35967]
ENSG00000151348	0,34711215	7,05366855	4,94755544	0,05241111	0,2735529	EXT2	exostosin glycosyltransferase 2 [Source:HGNC Symbol;Acc:HGNC:3513]
ENSG00000156384	-0,5755022	2,07969699	4,94659107	0,05242985	0,2735529	SFR1	SWI5 dependent homologous recombination repair protein 1 [Source:HGNC Symbol;Acc:HGNC:29574]
ENSG00000196591	0,31695911	7,16966954	4,94658935	0,05242988	0,2735529	HDAC2	histone deacetylase 2 [Source:HGNC Symbol;Acc:HGNC:4853]
ENSG00000080824	-0,575781	10,9153039	4,94553557	0,05245037	0,27356208	HSP90AA1	heat shock protein 90 alpha family class A member 1 [Source:HGNC Symbol;Acc:HGNC:5253]
ENSG00000087074	-0,3624327	5,46053133	4,93120593	0,05272991	0,27399856	PPP1R15A	protein phosphatase 1 regulatory subunit 15A [Source:HGNC Symbol;Acc:HGNC:14375]
ENSG00000126878	0,4441173	4,57356115	4,9297144	0,05275912	0,27399856	AIF1L	allograft inflammatory factor 1 like [Source:HGNC Symbol;Acc:HGNC:28904]
ENSG00000134815	0,3538817	4,66658849	4,94003498	0,05255745	0,27399856	DHX34	DExH-box helicase 34 [Source:HGNC Symbol;Acc:HGNC:16719]
ENSG00000136167	-0,7167681	-0,0848371	4,93714073	0,05261391	0,27399856	LCP1	lymphocyte cytosolic protein 1 [Source:HGNC Symbol;Acc:HGNC:6528]
ENSG00000171566	0,3090342	5,81728508	4,93235404	0,05270745	0,27399856	PLRG1	pleiotropic regulator 1 [Source:HGNC Symbol;Acc:HGNC:9089]
ENSG00000189159	-0,3080802	8,3117903	4,93217231	0,052711	0,27399856	JPT1	Jupiter microtubule associated homolog 1 [Source:HGNC Symbol;Acc:HGNC:14569]
ENSG00000196544	-0,4275846	3,03520962	4,92989069	0,05275567	0,27399856	BORCS6	BLOC-1 related complex subunit 6 [Source:HGNC Symbol;Acc:HGNC:25939]
ENSG00000198887	0,35251974	3,96728841	4,9312691	0,05272868	0,27399856	SMC5	structural maintenance of chromosomes 5 [Source:HGNC Symbol;Acc:HGNC:20465]
ENSG00000242588	1,56501505	-1,5044049	4,93678371	0,05262088	0,27399856	AC108010.1	novel transcript
ENSG00000248727	-0,7813135	0,09462548	4,93043053	0,05274509	0,27399856	LINC01948	long intergenic non-protein coding RNA 1948 [Source:HGNC Symbol;Acc:HGNC:52772]
ENSG00000250317	-0,3852668	4,47488074	4,93085112	0,05273686	0,27399856	SMIM20	small integral membrane protein 20 [Source:HGNC Symbol;Acc:HGNC:37260]
ENSG00000253320	1,49819565	-2,14024	4,93037382	0,0527462	0,27399856	MAILR	macrophage interferon regulatory lncRNA [Source:HGNC Symbol;Acc:HGNC:51558]
ENSG00000104529	-0,3138145	8,85171606	4,92674537	0,05281731	0,27403898	EEF1D	eukaryotic translation elongation factor 1 delta [Source:HGNC Symbol;Acc:HGNC:3211]
ENSG00000108021	-0,3654511	4,80650373	4,92718878	0,05280862	0,27403898	TASOR2	transcription activation suppressor family member 2 [Source:HGNC Symbol;Acc:HGNC:23484]

ENSG00000155158	0,34321051	5,28988118	4,9264465	0,05282317	0,27403898	TTC39B	tetratricopeptide repeat domain 39B [Source:HGNC Symbol;Acc:HGNC:23704]
ENSG00000213315	-0,5788141	0,70941909	4,92432545	0,0528648	0,27408346	AL122020.1	ribosomal protein S18 (RPS18) pseudogene
ENSG00000214063	-0,3054555	6,1186576	4,92409781	0,05286927	0,27408346	TSPAN4	tetraspanin 4 [Source:HGNC Symbol;Acc:HGNC:11859]
ENSG00000130711	-1,0110239	-0,6504648	4,92187739	0,0529129	0,27411508	PRDM12	PR/SET domain 12 [Source:HGNC Symbol;Acc:HGNC:13997]
ENSG00000235776	-0,4878108	1,45878476	4,92232527	0,05290409	0,27411508	AC000089.1	ribosomal protein L7a (RPL7A) pseudogene
ENSG00000268996	-0,5816293	0,91836778	4,91818305	0,05298558	0,27439432	MAN1B1-DT	MAN1B1 divergent transcript [Source:HGNC Symbol;Acc:HGNC:48715]
ENSG00000177943	-0,7696831	0,64728508	4,9158329	0,05303189	0,2745368	MAMDC4	MAM domain containing 4 [Source:HGNC Symbol;Acc:HGNC:24083]
ENSG00000197008	0,52763822	2,24002475	4,9145931	0,05305633	0,27456606	ZNF138	zinc finger protein 138 [Source:HGNC Symbol;Acc:HGNC:12922]
ENSG00000070614	0,38942539	5,30930679	4,91165803	0,05311427	0,27465032	NDST1	N-deacetylase and N-sulfotransferase 1 [Source:HGNC Symbol;Acc:HGNC:7680]
ENSG00000143630	0,51209561	1,38977435	4,91211355	0,05310527	0,27465032	HCN3	hyperpolarization activated cyclic nucleotide gated potassium channel 3 [Source:HGNC Symbol;Acc:HGNC:19183]
ENSG00000198736	-0,4014935	6,52745429	4,91091135	0,05312902	0,27465032	MSRB1	methionine sulfoxide reductase B1 [Source:HGNC Symbol;Acc:HGNC:14133]
ENSG00000120265	0,31052996	8,3083355	4,90814504	0,05318371	0,27466305	PCMT1	protein-L-isoaspartate (D-aspartate) O-methyltransferase [Source:HGNC Symbol;Acc:HGNC:8728]
ENSG00000205885	0,95768264	-0,7672295	4,90883216	0,05317012	0,27466305	C1RL-AS1	C1RL antisense RNA 1 [Source:HGNC Symbol;Acc:HGNC:27461]
ENSG00000243083	-0,7923038	-0,5904288	4,90793409	0,05318788	0,27466305	LINC00870	long intergenic non-protein coding RNA 870 [Source:HGNC Symbol;Acc:HGNC:27319]
ENSG00000135932	-0,3799176	4,63692297	4,90653043	0,05321566	0,27470941	CAB39	calcium binding protein 39 [Source:HGNC Symbol;Acc:HGNC:20292]
ENSG00000135837	0,5023747	3,49236171	4,90261655	0,05329322	0,27481659	CEP350	centrosomal protein 350 [Source:HGNC Symbol;Acc:HGNC:24238]
ENSG00000173812	-0,3212489	9,20307543	4,90407861	0,05326423	0,27481659	EIF1	eukaryotic translation initiation factor 1 [Source:HGNC Symbol;Acc:HGNC:3249]
ENSG00000180304	0,35536904	4,35226941	4,90305304	0,05328456	0,27481659	OAZ2	ornithine decarboxylase antizyme 2 [Source:HGNC Symbol;Acc:HGNC:8096]
ENSG00000258457	-1,2945964	-1,5524648	4,9016865	0,05331167	0,27481659	AL132780.2	novel transcript, antisense to C14orf93
ENSG00000126838	-0,6943826	2,69082636	4,89413155	0,05346185	0,27546301	PZP	PZP alpha-2-macroglobulin like [Source:HGNC Symbol;Acc:HGNC:9750]
ENSG00000128513	-0,4032387	2,59176036	4,89253558	0,05349364	0,27546301	POT1	protection of telomeres 1 [Source:HGNC Symbol;Acc:HGNC:17284]
ENSG00000204394	-0,46998	7,85732978	4,89293948	0,05348559	0,27546301	VARS1	valyl-tRNA synthetase 1 [Source:HGNC Symbol;Acc:HGNC:12651]
ENSG00000142892	-0,3992302	5,2319385	4,88784994	0,05358711	0,27574996	PIGK	phosphatidylinositol glycan anchor biosynthesis class K [Source:HGNC Symbol;Acc:HGNC:8965]
ENSG00000267321	-0,5109097	2,63582258	4,88872063	0,05356973	0,27574996	SNHG30	small nucleolar RNA host gene 30 [Source:HGNC Symbol;Acc:HGNC:52836]
ENSG00000198925	0,36911332	4,92114285	4,88516641	0,05364074	0,27592871	ATG9A	autophagy related 9A [Source:HGNC Symbol;Acc:HGNC:22408]
ENSG00000147586	-0,3225741	4,5431314	4,88143302	0,05371545	0,27621579	MRPS28	mitochondrial ribosomal protein S28 [Source:HGNC Symbol;Acc:HGNC:14513]
ENSG00000196715	-0,3451837	5,55383744	4,87847769	0,05377469	0,27642311	VKORC1L1	vitamin K epoxide reductase complex subunit 1 like 1 [Source:HGNC Symbol;Acc:HGNC:21492]
ENSG00000121690	0,36429699	3,37215858	4,8736635	0,05387136	0,27682263	DEPDC7	DEP domain containing 7 [Source:HGNC Symbol;Acc:HGNC:29899]
ENSG00000134444	0,38902646	3,7014291	4,86873257	0,0539706	0,27704024	RELCH	RAB11 binding and LisH domain, coiled-coil and HEAT repeat containing [Source:HGNC Symbol;Acc:HGNC:29289]
ENSG00000172594	0,50040368	3,65931019	4,86924322	0,05396031	0,27704024	SMPDL3A	sphingomyelin phosphodiesterase acid like 3A [Source:HGNC Symbol;Acc:HGNC:17389]
ENSG00000213867	-0,377665	2,37059247	4,87031413	0,05393875	0,27704024	AL049830.1	ribosomal protein L27 (RPL27) pseudogene
ENSG00000196208	0,42688205	5,5757009	4,86622481	0,05402116	0,27720237	GREB1	growth regulating estrogen receptor binding 1 [Source:HGNC Symbol;Acc:HGNC:24885]
ENSG00000055147	-0,3721925	3,5113336	4,86197974	0,05410688	0,27744052	FAM114A2	family with sequence similarity 114 member A2 [Source:HGNC Symbol;Acc:HGNC:1333]
ENSG00000107643	-0,4326892	3,22710849	4,86110601	0,05412454	0,27744052	MAPK8	mitogen-activated protein kinase 8 [Source:HGNC Symbol;Acc:HGNC:6881]
ENSG00000107951	0,39151294	5,24246681	4,86242641	0,05409785	0,27744052	MTPAP	mitochondrial poly(A) polymerase [Source:HGNC Symbol;Acc:HGNC:25532]
ENSG00000253552	-0,6469945	2,19806549	4,85862127	0,05417482	0,27760081	HOXA-AS2	HOXA cluster antisense RNA 2 [Source:HGNC Symbol;Acc:HGNC:43745]
ENSG00000115290	0,62938455	0,62498008	4,85396402	0,0542692	0,27798695	GRB14	growth factor receptor bound protein 14 [Source:HGNC Symbol;Acc:HGNC:4565]
ENSG00000128645	0,32868612	4,67546988	4,85112752	0,05432679	0,27818439	HOXD1	homeobox D1 [Source:HGNC Symbol;Acc:HGNC:5132]
ENSG00000186298	-0,3932336	6,8967877	4,84923783	0,0543652	0,27828351	PPP1CC	protein phosphatase 1 catalytic subunit gamma [Source:HGNC Symbol;Acc:HGNC:9283]
ENSG00000212719	0,35290791	4,14154495	4,84266621	0,05449902	0,27877339	LINC02693	long intergenic non-protein coding RNA 2693 [Source:HGNC Symbol;Acc:HGNC:27904]
ENSG00000260942	0,53132148	2,10535317	4,84266427	0,05449906	0,27877339	CAPN10-DT	CAPN10 divergent transcript [Source:HGNC Symbol;Acc:HGNC:48839]
ENSG00000103222	-0,5164948	6,24607196	4,83854616	0,05458313	0,2789847	ABCC1	ATP binding cassette subfamily C member 1 [Source:HGNC Symbol;Acc:HGNC:51]
ENSG00000160352	0,46510032	1,725854	4,83668214	0,05462124	0,2789847	ZNF714	zinc finger protein 714 [Source:HGNC Symbol;Acc:HGNC:27124]
ENSG00000169972	-0,3621375	4,46471118	4,83737444	0,05460708	0,2789847	PUSL1	pseudouridine synthase like 1 [Source:HGNC Symbol;Acc:HGNC:26914]

ENSG00000225756	-1,2629849	-0,1532849	4,83686762	0,05461745	0,2789847	DBH-AS1	DBH antisense RNA 1 [Source:HGNC Symbol;Acc:HGNC:24155]
ENSG00000244171	-1,3480506	-2,0632879	4,83596767	0,05463586	0,2789847	PBX2P1	PBX homeobox 2 pseudogene 1 [Source:HGNC Symbol;Acc:HGNC:8635]
ENSG00000185829	0,52592677	0,9500648	4,83404875	0,05467513	0,27908771	ARL17A	ADP ribosylation factor like GTPase 17A [Source:HGNC Symbol;Acc:HGNC:24096]
ENSG00000107816	0,40247843	6,05921167	4,83134404	0,05473056	0,27909709	LZTS2	leucine zipper tumor suppressor 2 [Source:HGNC Symbol;Acc:HGNC:29381]
ENSG00000120451	1,35754754	-2,0273213	4,83116216	0,05473428	0,27909709	SNX19	sorting nexin 19 [Source:HGNC Symbol;Acc:HGNC:21532]
ENSG00000225828	1,21584631	-0,7503849	4,83260008	0,05470481	0,27909709	FAM229A	family with sequence similarity 229 member A [Source:HGNC Symbol;Acc:HGNC:44652]
ENSG00000083223	0,41228682	3,51956	4,82899549	0,05477874	0,27922629	TUT7	terminal uridylyl transferase 7 [Source:HGNC Symbol;Acc:HGNC:25817]
ENSG00000163162	0,32939109	6,64062493	4,82574691	0,05484547	0,27937149	RNF149	ring finger protein 149 [Source:HGNC Symbol;Acc:HGNC:23137]
ENSG00000167619	0,64625224	0,87749329	4,82595969	0,05484109	0,27937149	TMEM145	transmembrane protein 145 [Source:HGNC Symbol;Acc:HGNC:26912]
ENSG00000174099	0,61429251	0,47677563	4,82314446	0,054899	0,2794493	MSRB3	methionine sulfoxide reductase B3 [Source:HGNC Symbol;Acc:HGNC:27375]
ENSG00000188985	-0,9579558	-1,3140734	4,82382076	0,05488508	0,2794493	DHFRP1	dihydrofolate reductase pseudogene 1 [Source:HGNC Symbol;Acc:HGNC:2862]
ENSG00000104129	-0,350373	4,1111801	4,81479106	0,05507127	0,27949762	DNAJC17	DnaJ heat shock protein family (Hsp40) member C17 [Source:HGNC Symbol;Acc:HGNC:25556]
ENSG00000105612	0,35192821	5,60093887	4,82129895	0,054937	0,27949762	DNASE2	deoxyribonuclease 2, lysosomal [Source:HGNC Symbol;Acc:HGNC:2960]
ENSG00000133794	-0,5007497	1,73873464	4,81734533	0,05501852	0,27949762	ARNTL	aryl hydrocarbon receptor nuclear translocator like [Source:HGNC Symbol;Acc:HGNC:701]
ENSG00000145041	0,51387142	3,9977812	4,81433577	0,05508068	0,27949762	DCAF1	DDB1 and CUL4 associated factor 1 [Source:HGNC Symbol;Acc:HGNC:30911]
ENSG00000177485	0,38337382	3,08940065	4,81602084	0,05504587	0,27949762	ZBTB33	zinc finger and BTB domain containing 33 [Source:HGNC Symbol;Acc:HGNC:16682]
ENSG00000184922	-0,7042438	-0,0157526	4,81972579	0,05496942	0,27949762	FMNL1	formin like 1 [Source:HGNC Symbol;Acc:HGNC:1212]
ENSG00000185238	-0,3174014	4,45500435	4,81906102	0,05498313	0,27949762	PRMT3	protein arginine methyltransferase 3 [Source:HGNC Symbol;Acc:HGNC:30163]
ENSG00000196204	0,42332001	3,13876396	4,81708636	0,05502387	0,27949762	RNF216P1	ring finger protein 216 pseudogene 1 [Source:HGNC Symbol;Acc:HGNC:33610]
ENSG00000214413	-0,5550814	2,12126865	4,8148022	0,05507104	0,27949762	BBIP1	BBSome interacting protein 1 [Source:HGNC Symbol;Acc:HGNC:28093]
ENSG00000065268	-0,387874	6,39360428	4,81137602	0,05514189	0,27964123	WDR18	WD repeat domain 18 [Source:HGNC Symbol;Acc:HGNC:17956]
ENSG00000120656	-0,4426592	3,34663835	4,81081954	0,05515341	0,27964123	TAF12	TATA-box binding protein associated factor 12 [Source:HGNC Symbol;Acc:HGNC:11545]
ENSG00000251474	0,47656861	1,72915971	4,81019189	0,05516641	0,27964123	RPL32P3	ribosomal protein L32 pseudogene 3 [Source:HGNC Symbol;Acc:HGNC:27024]
ENSG00000112697	0,31693136	6,15954931	4,80894598	0,05519221	0,27967501	TMEM30A	transmembrane protein 30A [Source:HGNC Symbol;Acc:HGNC:16667]
ENSG00000123415	-0,4722159	4,03566243	4,80720211	0,05522836	0,27976114	SMUG1	single-strand-selective monofunctional uracil-DNA glycosylase 1 [Source:HGNC Symbol;Acc:HGNC:17148]
ENSG00000110237	0,41892791	4,04846408	4,80459028	0,05528256	0,2799386	ARHGEF17	Rho guanine nucleotide exchange factor 17 [Source:HGNC Symbol;Acc:HGNC:21726]
ENSG00000174840	0,46105007	5,47701827	4,80018903	0,05537403	0,28030464	PDE12	phosphodiesterase 12 [Source:HGNC Symbol;Acc:HGNC:25386]
ENSG00000196295	0,73240969	0,86903589	4,79394674	0,05550409	0,28086569	GARS1-DT	GARS1 divergent transcript [Source:HGNC Symbol;Acc:HGNC:48951]
ENSG00000128602	0,33931297	5,73118903	4,79201074	0,0555445	0,28097288	SMO	smoothened, frizzled class receptor [Source:HGNC Symbol;Acc:HGNC:11119]
ENSG00000107560	0,53833972	1,22075732	4,79023046	0,0555817	0,28101258	RAB11FIP2	RAB11 family interacting protein 2 [Source:HGNC Symbol;Acc:HGNC:29152]
ENSG00000145692	0,6249545	2,01057502	4,78979406	0,05559082	0,28101258	BHMT	betaine--homocysteine S-methyltransferase [Source:HGNC Symbol;Acc:HGNC:1047]
ENSG00000180957	0,30412148	5,71058881	4,78879376	0,05561174	0,28102108	PITPNB	phosphatidylinositol transfer protein beta [Source:HGNC Symbol;Acc:HGNC:9002]
ENSG00000092978	0,4114844	2,79516985	4,78742532	0,05564037	0,28105576	GPATCH2	G-patch domain containing 2 [Source:HGNC Symbol;Acc:HGNC:25499]
ENSG00000117461	-0,3927729	2,36301857	4,78486036	0,05569409	0,28105576	PIK3R3	phosphoinositide-3-kinase regulatory subunit 3 [Source:HGNC Symbol;Acc:HGNC:8981]
ENSG00000178458	-0,7867425	-0,1995036	4,78387247	0,0557148	0,28105576	H3P16	H3 histone pseudogene 16 [Source:HGNC Symbol;Acc:HGNC:42982]
ENSG00000231365	0,53569238	0,98570334	4,78635853	0,05566271	0,28105576	WARS2-AS1	WARS2 antisense RNA 1 [Source:HGNC Symbol;Acc:HGNC:40612]
ENSG00000260920	0,98920373	-0,2402001	4,78398146	0,05571251	0,28105576	AL031985.3	novel transcript, sense overlapping with ZNF643
ENSG00000224046	-1,2943131	-1,2078679	4,78021701	0,0557915	0,28132526	AC005076.1	novel transcript, antisense to DMTF1
ENSG00000273802	0,84089077	-0,3574732	4,77949164	0,05580674	0,28132526	H2BC4	H2B clustered histone 4 [Source:HGNC Symbol;Acc:HGNC:4757]
ENSG00000107159	-0,8065945	1,65557509	4,77539588	0,05589286	0,28161846	CA9	carbonic anhydrase 9 [Source:HGNC Symbol;Acc:HGNC:1383]
ENSG00000115363	-0,324637	5,76353469	4,77397745	0,05592273	0,28161846	EVA1A	eva-1 homolog A, regulator of programmed cell death [Source:HGNC Symbol;Acc:HGNC:25816]
ENSG00000235173	-0,3677555	5,0868047	4,77442273	0,05591335	0,28161846	HGH1	HGH1 homolog [Source:HGNC Symbol;Acc:HGNC:24161]
ENSG00000107796	-0,3732701	5,77313129	4,75937064	0,05623145	0,28229237	ACTA2	actin alpha 2, smooth muscle [Source:HGNC Symbol;Acc:HGNC:130]
ENSG00000133961	0,34093516	5,38098256	4,76316866	0,05615097	0,28229237	NUMB	NUMB endocytic adaptor protein [Source:HGNC Symbol;Acc:HGNC:8060]

ENSG00000157014	0,40412363	3,47738515	4,76339158	0,05614625	0,28229237	TATDN2	TatD DNase domain containing 2 [Source:HGNC Symbol;Acc:HGNC:28988]
ENSG00000164961	0,38033099	4,14066772	4,76217411	0,05617203	0,28229237	WASHC5	WASH complex subunit 5 [Source:HGNC Symbol;Acc:HGNC:28984]
ENSG00000167526	-0,3302403	10,679697	4,76243684	0,05616647	0,28229237	RPL13	ribosomal protein L13 [Source:HGNC Symbol;Acc:HGNC:10303]
ENSG00000169826	0,42701378	3,26421344	4,75892676	0,05624086	0,28229237	CSGALNACT2	chondroitin sulfate N-acetylgalactosaminyltransferase 2 [Source:HGNC Symbol;Acc:HGNC:24292]
ENSG00000172216	-0,3016596	5,5856201	4,76036185	0,05621043	0,28229237	CEBPB	CCAAT enhancer binding protein beta [Source:HGNC Symbol;Acc:HGNC:1834]
ENSG00000172260	1,30468618	-1,3518113	4,76491326	0,05611405	0,28229237	NEGR1	neuronal growth regulator 1 [Source:HGNC Symbol;Acc:HGNC:17302]
ENSG00000245532	0,4523697	3,92949782	4,76135226	0,05618944	0,28229237	NEAT1	nuclear paraspeckle assembly transcript 1 [Source:HGNC Symbol;Acc:HGNC:30815]
ENSG00000256073	-0,4762504	1,73896844	4,75850618	0,05624978	0,28229237	URB1-AS1	URB1 antisense RNA 1 (head to head) [Source:HGNC Symbol;Acc:HGNC:23128]
ENSG00000176261	-0,3950769	5,14286254	4,75331679	0,05636002	0,2826514	ZBTB8OS	zinc finger and BTB domain containing 8 opposite strand [Source:HGNC Symbol;Acc:HGNC:24094]
ENSG00000184675	0,42755689	3,03635658	4,75392925	0,056347	0,2826514	AMER1	APC membrane recruitment protein 1 [Source:HGNC Symbol;Acc:HGNC:26837]
ENSG00000136111	0,4049774	5,40094807	4,75196175	0,05638885	0,28269893	TBC1D4	TBC1 domain family member 4 [Source:HGNC Symbol;Acc:HGNC:19165]
ENSG00000198455	0,4591205	2,0310396	4,74565445	0,05652328	0,28327568	ZXDB	zinc finger X-linked duplicated B [Source:HGNC Symbol;Acc:HGNC:13199]
ENSG00000134308	-0,3153192	8,07527464	4,74464756	0,05654478	0,28328624	YWHAQ	tyrosine 3-monooxygenase/tryptophan 5-monooxygenase activation protein theta [Source:HGNC Symbol;Acc:HG
ENSG00000260083	0,66158542	0,2699275	4,74066059	0,05663	0,28361594	MIR762HG	MIR762 host gene [Source:HGNC Symbol;Acc:HGNC:51386]
ENSG00000116830	0,40479302	4,94111575	4,73866371	0,05667275	0,28373275	TTF2	transcription termination factor 2 [Source:HGNC Symbol;Acc:HGNC:12398]
ENSG00000102359	-0,6770807	0,37493149	4,73400792	0,05677256	0,28376037	SRPX2	sushi repeat containing protein X-linked 2 [Source:HGNC Symbol;Acc:HGNC:30668]
ENSG00000119280	0,36311095	4,53787765	4,73350511	0,05678336	0,28376037	C1orf198	chromosome 1 open reading frame 198 [Source:HGNC Symbol;Acc:HGNC:25900]
ENSG00000167992	-0,4113392	6,66724644	4,73730963	0,05670176	0,28376037	VWCE	von Willebrand factor C and EGF domains [Source:HGNC Symbol;Acc:HGNC:26487]
ENSG00000169951	-0,3420352	3,56817734	4,73216356	0,05681217	0,28376037	ZNF764	zinc finger protein 764 [Source:HGNC Symbol;Acc:HGNC:28200]
ENSG00000177106	-0,4032556	6,87971553	4,73206753	0,05681423	0,28376037	EPS8L2	EPS8 like 2 [Source:HGNC Symbol;Acc:HGNC:21296]
ENSG00000179841	0,9481515	-1,1988785	4,73361491	0,056781	0,28376037	AKAP5	A-kinase anchoring protein 5 [Source:HGNC Symbol;Acc:HGNC:375]
ENSG00000186648	1,36519406	-1,5876396	4,73406483	0,05677134	0,28376037	CARMIL3	capping protein regulator and myosin 1 linker 3 [Source:HGNC Symbol;Acc:HGNC:20272]
ENSG00000231607	-0,5549286	1,10104908	4,72969253	0,05686528	0,28391828	DLEU2	deleted in lymphocytic leukemia 2 [Source:HGNC Symbol;Acc:HGNC:13748]
ENSG00000271943	-0,7961927	-0,3553513	4,72754853	0,05691141	0,28405154	AC098614.2	tropomyosin 4 (TPM4) pseudogene
ENSG00000106004	0,59417406	0,38113339	4,72331267	0,0570027	0,28417015	HOXA5	homeobox A5 [Source:HGNC Symbol;Acc:HGNC:5106]
ENSG00000147010	-0,3239992	4,14096387	4,72437933	0,05697969	0,28417015	SH3KBP1	SH3 domain containing kinase binding protein 1 [Source:HGNC Symbol;Acc:HGNC:13867]
ENSG00000179029	-0,5477171	1,00591009	4,72283582	0,05701299	0,28417015	TMEM107	transmembrane protein 107 [Source:HGNC Symbol;Acc:HGNC:28128]
ENSG00000268654	0,59552792	0,51789789	4,72427063	0,05698204	0,28417015	MIMT1	MER1 repeat containing imprinted transcript 1 [Source:HGNC Symbol;Acc:HGNC:33464]
ENSG00000175938	-0,4127866	3,28639191	4,72071261	0,05705882	0,28430161	ORAI3	ORAI calcium release-activated calcium modulator 3 [Source:HGNC Symbol;Acc:HGNC:28185]
ENSG00000165923	-1,0881259	-1,3750579	4,71809546	0,05711538	0,28438944	AGBL2	ATP/GTP binding protein like 2 [Source:HGNC Symbol;Acc:HGNC:26296]
ENSG00000243779	-0,8212939	-0,7556288	4,71884466	0,05709918	0,28438944	AP001086.1	ribosomal protein L36a-like (RPL36AL) pseudogene
ENSG00000100124	0,32538361	5,37461806	4,71557169	0,05716999	0,28446744	ANKRD54	ankyrin repeat domain 54 [Source:HGNC Symbol;Acc:HGNC:25185]
ENSG00000137575	0,39374892	5,18821407	4,71573237	0,05716651	0,28446744	SDCBP	syndecan binding protein [Source:HGNC Symbol;Acc:HGNC:10662]
ENSG00000015153	-0,4094492	2,46382058	4,71150962	0,05725802	0,28478119	YAF2	YY1 associated factor 2 [Source:HGNC Symbol;Acc:HGNC:17363]
ENSG00000179051	0,37482519	6,19568799	4,71086379	0,05727203	0,28478119	RCC2	regulator of chromosome condensation 2 [Source:HGNC Symbol;Acc:HGNC:30297]
ENSG00000110200	-0,5387531	4,17305814	4,70963196	0,05729877	0,2848172	ANAPC15	anaphase promoting complex subunit 15 [Source:HGNC Symbol;Acc:HGNC:24531]
ENSG00000119760	0,36069327	6,18316188	4,7040653	0,05741979	0,28496595	SUPT7L	SPT7 like, STAGA complex subunit gamma [Source:HGNC Symbol;Acc:HGNC:30632]
ENSG00000141965	-0,3024996	5,87169024	4,70634664	0,05737016	0,28496595	FEM1A	fem-1 homolog A [Source:HGNC Symbol;Acc:HGNC:16934]
ENSG00000142082	0,42053143	4,16234464	4,7058293	0,05738141	0,28496595	SIRT3	sirtuin 3 [Source:HGNC Symbol;Acc:HGNC:14931]
ENSG00000143669	0,83024609	-0,2622903	4,70532051	0,05739248	0,28496595	LYST	lysosomal trafficking regulator [Source:HGNC Symbol;Acc:HGNC:1968]
ENSG00000155846	-0,7395764	0,30363778	4,70376975	0,05742623	0,28496595	PPARGC1B	PPARG coactivator 1 beta [Source:HGNC Symbol;Acc:HGNC:30022]
ENSG00000099849	-0,3096863	4,66217547	4,69810444	0,05754974	0,28525606	RASSF7	Ras association domain family member 7 [Source:HGNC Symbol;Acc:HGNC:1166]
ENSG00000100528	0,30342421	7,63703636	4,69806841	0,05755053	0,28525606	CNIH1	cornichon family AMPA receptor auxiliary protein 1 [Source:HGNC Symbol;Acc:HGNC:19431]
ENSG00000140807	-0,2896693	8,38961331	4,69991188	0,0575103	0,28525606	NKD1	NKD inhibitor of WNT signaling pathway 1 [Source:HGNC Symbol;Acc:HGNC:17045]

ENSG00000163346	0,51978636	4,60489487	4,69661249	0,05758232	0,28525606	PBXIP1	PBX homeobox interacting protein 1 [Source:HGNC Symbol;Acc:HGNC:21199]
ENSG00000234996	-0,7280504	0,41868955	4,69704848	0,0575728	0,28525606	ACTG1P25	actin gamma 1 pseudogene 25 [Source:HGNC Symbol;Acc:HGNC:54872]
ENSG00000086827	0,40308072	4,87299005	4,68827394	0,05776485	0,28538608	ZW10	zw10 kinetochore protein [Source:HGNC Symbol;Acc:HGNC:13194]
ENSG00000108641	-0,4002326	4,36681571	4,68850327	0,05775982	0,28538608	B9D1	B9 domain containing 1 [Source:HGNC Symbol;Acc:HGNC:24123]
ENSG00000113494	0,45112686	1,35550928	4,69068966	0,05771189	0,28538608	PRLR	prolactin receptor [Source:HGNC Symbol;Acc:HGNC:9446]
ENSG00000141736	0,33874604	7,5038096	4,68848923	0,05776012	0,28538608	ERBB2	erb-b2 receptor tyrosine kinase 2 [Source:HGNC Symbol;Acc:HGNC:3430]
ENSG00000169299	-0,3874583	4,56672056	4,68844539	0,05776109	0,28538608	PGM2	phosphoglucomutase 2 [Source:HGNC Symbol;Acc:HGNC:8906]
ENSG00000182134	0,41030689	2,99883863	4,68829866	0,0577643	0,28538608	TDRKH	tudor and KH domain containing [Source:HGNC Symbol;Acc:HGNC:11713]
ENSG00000239672	-0,319975	7,58309039	4,69254524	0,05767126	0,28538608	NME1	NME/NM23 nucleoside diphosphate kinase 1 [Source:HGNC Symbol;Acc:HGNC:7849]
ENSG00000258377	-1,2880095	-1,4477185	4,69445802	0,05762941	0,28538608	AL139099.1	novel transcript
ENSG00000126016	0,92458529	-0,5858175	4,68381113	0,05786283	0,28540138	AMOT	angiomotin [Source:HGNC Symbol;Acc:HGNC:17810]
ENSG00000137193	0,33067195	4,80692406	4,68290692	0,05788271	0,28540138	PIM1	Pim-1 proto-oncogene, serine/threonine kinase [Source:HGNC Symbol;Acc:HGNC:8986]
ENSG00000163545	-0,500239	3,28031541	4,68658422	0,05780192	0,28540138	NUAK2	NUAK family kinase 2 [Source:HGNC Symbol;Acc:HGNC:29558]
ENSG00000185513	0,7706794	-0,0696905	4,68279547	0,05788516	0,28540138	L3MBTL1	L3MBTL histone methyl-lysine binding protein 1 [Source:HGNC Symbol;Acc:HGNC:15905]
ENSG00000220583	-0,3903995	1,89871528	4,68541353	0,05782763	0,28540138	RPL35P2	ribosomal protein L35 pseudogene 2 [Source:HGNC Symbol;Acc:HGNC:35789]
ENSG00000254469	0,56680949	0,41085711	4,68492688	0,05783831	0,28540138	AP002495.1	XRCC1 N-terminal domain containing 1-like [Source:NCBI gene (formerly Entrezgene);Acc:100133315]
ENSG00000115806	-0,3017917	7,55125462	4,67884411	0,05797213	0,2855293	GORASP2	golgi reassembly stacking protein 2 [Source:HGNC Symbol;Acc:HGNC:17500]
ENSG00000168397	0,30299448	6,43643469	4,67974435	0,0579523	0,2855293	ATG4B	autophagy related 4B cysteine peptidase [Source:HGNC Symbol;Acc:HGNC:20790]
ENSG00000168461	-0,9405078	-0,2841038	4,67850568	0,05797959	0,2855293	RAB31	RAB31, member RAS oncogene family [Source:HGNC Symbol;Acc:HGNC:9771]
ENSG00000176531	-0,4027133	3,41926375	4,67806568	0,05798928	0,2855293	PHLDB3	pleckstrin homology like domain family B member 3 [Source:HGNC Symbol;Acc:HGNC:30499]
ENSG00000138346	0,32339161	3,35912434	4,67565793	0,05804238	0,28569444	DNA2	DNA replication helicase/nuclease 2 [Source:HGNC Symbol;Acc:HGNC:2939]
ENSG00000107185	-0,4401053	3,92221615	4,67151991	0,05813377	0,28577245	RGP1	RGP1 homolog, RAB6A GEF complex partner 1 [Source:HGNC Symbol;Acc:HGNC:21965]
ENSG00000145375	0,74637217	1,85933849	4,67338041	0,05809266	0,28577245	SPATA5	spermatogenesis associated 5 [Source:HGNC Symbol;Acc:HGNC:18119]
ENSG00000176055	-0,4324746	1,98785623	4,67313798	0,05809801	0,28577245	MBLAC2	metallo-beta-lactamase domain containing 2 [Source:HGNC Symbol;Acc:HGNC:33711]
ENSG00000187079	0,55768126	2,57168884	4,67139776	0,05813647	0,28577245	TEAD1	TEA domain transcription factor 1 [Source:HGNC Symbol;Acc:HGNC:11714]
ENSG00000018280	-1,1217877	-0,5570407	4,66974631	0,058173	0,28585581	SLC11A1	solute carrier family 11 member 1 [Source:HGNC Symbol;Acc:HGNC:10907]
ENSG00000090621	0,32173717	7,45955552	4,66794117	0,05821296	0,28595598	PABPC4	poly(A) binding protein cytoplasmic 4 [Source:HGNC Symbol;Acc:HGNC:8557]
ENSG00000203761	-0,5741136	1,20760945	4,66387015	0,0583032	0,286303	MSTO2P	misato family member 2, pseudogene [Source:HGNC Symbol;Acc:HGNC:26329]
ENSG00000109103	0,43812893	3,94232309	4,65677247	0,05846095	0,28636368	UNC119	unc-119 lipid binding chaperone [Source:HGNC Symbol;Acc:HGNC:12565]
ENSG00000113368	0,33797419	6,6380444	4,65625958	0,05847237	0,28636368	LMNB1	lamin B1 [Source:HGNC Symbol;Acc:HGNC:6637]
ENSG00000122547	-0,3222081	4,88671029	4,65777249	0,05843869	0,28636368	EEPD1	endonuclease/exonuclease/phosphatase family domain containing 1 [Source:HGNC Symbol;Acc:HGNC:22223]
ENSG00000130818	0,5071325	2,25964816	4,66088099	0,05836957	0,28636368	ZNF426	zinc finger protein 426 [Source:HGNC Symbol;Acc:HGNC:20725]
ENSG00000154864	0,37863954	3,35929991	4,66084201	0,05837044	0,28636368	PIEZO2	piezo type mechanosensitive ion channel component 2 [Source:HGNC Symbol;Acc:HGNC:26270]
ENSG00000157500	-0,3428124	3,87844873	4,6592938	0,05840485	0,28636368	APPL1	adaptor protein, phosphotyrosine interacting with PH domain and leucine zipper 1 [Source:HGNC Symbol;Acc:HG
ENSG00000228232	-0,3631114	3,82964318	4,65891358	0,05841331	0,28636368	GAPDHP1	glyceraldehyde-3-phosphate dehydrogenase pseudogene 1 [Source:HGNC Symbol;Acc:HGNC:4159]
ENSG00000257108	1,33312349	-1,4972449	4,65792372	0,05843533	0,28636368	NHLRC4	NHL repeat containing 4 [Source:HGNC Symbol;Acc:HGNC:26700]
ENSG00000101350	0,34991187	4,98106113	4,65336683	0,05853683	0,2864972	KIF3B	kinesin family member 3B [Source:HGNC Symbol;Acc:HGNC:6320]
ENSG00000137806	-0,3211783	3,64853657	4,65316054	0,05854143	0,2864972	NDUFAF1	NADH:ubiquinone oxidoreductase complex assembly factor 1 [Source:HGNC Symbol;Acc:HGNC:18828]
ENSG00000171606	0,38072303	4,4935615	4,65239688	0,05855847	0,2864972	ZNF274	zinc finger protein 274 [Source:HGNC Symbol;Acc:HGNC:13068]
ENSG00000116747	-0,3453144	3,18258666	4,65132392	0,05858241	0,28651839	RO60	Ro60, Y RNA binding protein [Source:HGNC Symbol;Acc:HGNC:11313]
ENSG00000178821	-0,460366	4,44234161	4,64552948	0,05871193	0,28697531	TMEM52	transmembrane protein 52 [Source:HGNC Symbol;Acc:HGNC:27916]
ENSG00000241741	-0,403848	1,83238846	4,6453868	0,05871512	0,28697531	RPL7AP30	ribosomal protein L7a pseudogene 30 [Source:HGNC Symbol;Acc:HGNC:35757]
ENSG00000101951	0,8718167	-0,2223038	4,63991064	0,05883786	0,28703357	PAGE4	PAGE family member 4 [Source:HGNC Symbol;Acc:HGNC:4108]
ENSG00000120333	-0,2830338	5,96183339	4,6438497	0,05874954	0,28703357	MRPS14	mitochondrial ribosomal protein S14 [Source:HGNC Symbol;Acc:HGNC:14049]

ENSG00000132541	-0,3780778	4,53079419	4,63990678	0,05883795	0,28703357	RIDA	reactive intermediate imine deaminase A homolog [Source:HGNC Symbol;Acc:HGNC:16897]
ENSG00000143401	-0,3112398	5,24039746	4,63889478	0,05886067	0,28703357	ANP32E	acidic nuclear phosphoprotein 32 family member E [Source:HGNC Symbol;Acc:HGNC:16673]
ENSG00000196116	0,35468491	3,81992983	4,63872076	0,05886457	0,28703357	TDRD7	tudor domain containing 7 [Source:HGNC Symbol;Acc:HGNC:30831]
ENSG00000198160	0,33578032	3,17587232	4,64068069	0,05882058	0,28703357	MIER1	MIER1 transcriptional regulator [Source:HGNC Symbol;Acc:HGNC:29657]
ENSG00000198482	0,99371791	-0,1634083	4,64110917	0,05881097	0,28703357	ZNF808	zinc finger protein 808 [Source:HGNC Symbol;Acc:HGNC:33230]
ENSG00000119977	0,31618662	7,32768172	4,63550234	0,0589369	0,28729035	TCTN3	tectonic family member 3 [Source:HGNC Symbol;Acc:HGNC:24519]
ENSG00000120899	-0,7454238	0,53090069	4,62903682	0,05908254	0,28742546	PTK2B	protein tyrosine kinase 2 beta [Source:HGNC Symbol;Acc:HGNC:9612]
ENSG00000136827	0,29765191	6,52059281	4,63287168	0,0589961	0,28742546	TOR1A	torsin family 1 member A [Source:HGNC Symbol;Acc:HGNC:3098]
ENSG00000163935	0,34644653	4,15614227	4,63252197	0,05900398	0,28742546	SFMBT1	Scm like with four mbt domains 1 [Source:HGNC Symbol;Acc:HGNC:20255]
ENSG00000171988	0,51570721	3,86354128	4,63160101	0,05902472	0,28742546	JMJD1C	jumonji domain containing 1C [Source:HGNC Symbol;Acc:HGNC:12313]
ENSG00000224646	-0,7599968	-0,4228635	4,62958403	0,05907019	0,28742546	RPS28P5	ribosomal protein S28 pseudogene 5 [Source:HGNC Symbol;Acc:HGNC:36802]
ENSG00000277369	-0,8919965	-0,8280744	4,62903114	0,05908266	0,28742546	AC010654.1	novel transcript
ENSG00000117448	-0,2913977	7,07899318	4,6265045	0,0591397	0,28760714	AKR1A1	aldo-keto reductase family 1 member A1 [Source:HGNC Symbol;Acc:HGNC:380]
ENSG00000178537	0,33073523	4,87289656	4,62195505	0,05924257	0,28801154	SLC25A20	solute carrier family 25 member 20 [Source:HGNC Symbol;Acc:HGNC:1421]
ENSG00000140939	-0,413858	1,70917553	4,60965729	0,05952176	0,28927259	NOL3	nucleolar protein 3 [Source:HGNC Symbol;Acc:HGNC:7869]
ENSG00000134287	0,3139278	4,62375547	4,60125398	0,05971348	0,2900114	ARF3	ADP ribosylation factor 3 [Source:HGNC Symbol;Acc:HGNC:654]
ENSG00000197381	0,34358143	3,23066011	4,60183896	0,05970011	0,2900114	ADARB1	adenosine deaminase RNA specific B1 [Source:HGNC Symbol;Acc:HGNC:226]
ENSG00000092036	0,30628717	4,95438786	4,59949011	0,05975383	0,2900145	HAUS4	HAUS augmin like complex subunit 4 [Source:HGNC Symbol;Acc:HGNC:20163]
ENSG00000197279	0,39848542	2,67764312	4,59974723	0,05974794	0,2900145	ZNF165	zinc finger protein 165 [Source:HGNC Symbol;Acc:HGNC:12953]
ENSG00000198142	0,28698513	5,478297	4,59776469	0,05979332	0,29003711	SOWAHC	sosondowah ankyrin repeat domain family member C [Source:HGNC Symbol;Acc:HGNC:26149]
ENSG00000217026	-0,483012	1,4576996	4,59755198	0,05979819	0,29003711	RPL10P1	ribosomal protein L10 pseudogene 1 [Source:HGNC Symbol;Acc:HGNC:10300]
ENSG00000183011	-0,3613496	5,59053128	4,59464394	0,05986484	0,29017265	NAA38	N-alpha-acetyltransferase 38, NatC auxiliary subunit [Source:HGNC Symbol;Acc:HGNC:28212]
ENSG00000186193	-0,3208212	6,31056801	4,59459929	0,05986586	0,29017265	SAPCD2	suppressor APC domain containing 2 [Source:HGNC Symbol;Acc:HGNC:28055]
ENSG00000198856	-0,3147787	8,58922002	4,59313161	0,05989953	0,29023956	OSTC	oligosaccharyltransferase complex non-catalytic subunit [Source:HGNC Symbol;Acc:HGNC:24448]
ENSG00000018408	0,32086666	6,0559263	4,59011803	0,05996875	0,29047858	WWTR1	WW domain containing transcription regulator 1 [Source:HGNC Symbol;Acc:HGNC:24042]
ENSG00000128059	-0,3081135	5,07815401	4,58875525	0,06000008	0,29053402	PPAT	phosphoribosyl pyrophosphate amidotransferase [Source:HGNC Symbol;Acc:HGNC:9238]
ENSG00000165752	-0,3582486	4,46620742	4,58417811	0,06010546	0,29085149	STK32C	serine/threonine kinase 32C [Source:HGNC Symbol;Acc:HGNC:21332]
ENSG00000282793	-1,31866	-1,2618283	4,58491883	0,06008839	0,29085149	AC090164.4	novel transcript
ENSG00000165802	-0,3273203	5,62772836	4,58319391	0,06012815	0,29086495	NSMF	NMDA receptor synaptonuclear signaling and neuronal migration factor [Source:HGNC Symbol;Acc:HGNC:29843]
ENSG00000087589	-1,2735139	-2,0380254	4,57990877	0,06020396	0,29103895	CASS4	Cas scaffold protein family member 4 [Source:HGNC Symbol;Acc:HGNC:15878]
ENSG00000128699	-0,4066924	5,12783547	4,58003216	0,06020111	0,29103895	ORMDL1	ORMDL sphingolipid biosynthesis regulator 1 [Source:HGNC Symbol;Acc:HGNC:16036]
ENSG00000070718	-0,3260174	3,5533107	4,57763273	0,06025656	0,29119686	AP3M2	adaptor related protein complex 3 subunit mu 2 [Source:HGNC Symbol;Acc:HGNC:570]
ENSG00000165046	-0,787111	1,41960107	4,57638453	0,06028543	0,29124003	LETM2	leucine zipper and EF-hand containing transmembrane protein 2 [Source:HGNC Symbol;Acc:HGNC:14648]
ENSG00000213949	0,54343456	4,09829788	4,57546311	0,06030675	0,29124672	ITGA1	integrin subunit alpha 1 [Source:HGNC Symbol;Acc:HGNC:6134]
ENSG00000176473	-0,3857461	2,31521511	4,57448684	0,06032935	0,29125959	WDR25	WD repeat domain 25 [Source:HGNC Symbol;Acc:HGNC:21064]
ENSG00000242814	-0,5099362	0,81050859	4,57256678	0,06037384	0,29137805	AC113398.1	ribosomal protein L28 (RPL28) pseudogene
ENSG00000242083	-0,4748111	1,25727118	4,56886286	0,06045976	0,29169638	RPL7AP31	ribosomal protein L7a pseudogene 31 [Source:HGNC Symbol;Acc:HGNC:36857]
ENSG00000133597	0,40890305	4,89333099	4,56667898	0,06051049	0,29174846	ADCK2	aarF domain containing kinase 2 [Source:HGNC Symbol;Acc:HGNC:19039]
ENSG00000151553	0,47232688	2,36749025	4,56741534	0,06049338	0,29174846	FAM160B1	family with sequence similarity 160 member B1 [Source:HGNC Symbol;Acc:HGNC:29320]
ENSG00000133103	0,3781562	3,45401627	4,56551325	0,0605376	0,29178283	COG6	component of oligomeric golgi complex 6 [Source:HGNC Symbol;Acc:HGNC:18621]
ENSG00000223877	-0,3695524	2,5405442	4,56011908	0,06066321	0,29229183	RPS8P10	ribosomal protein S8 pseudogene 10 [Source:HGNC Symbol;Acc:HGNC:35628]
ENSG00000257017	-0,318935	7,35382733	4,55773931	0,06071873	0,29246288	HP	haptoglobin [Source:HGNC Symbol;Acc:HGNC:5141]
ENSG00000234616	0,39663648	4,14130586	4,55474069	0,06078878	0,29270378	JRK	Jrk helix-turn-helix protein [Source:HGNC Symbol;Acc:HGNC:6199]
ENSG00000041802	0,44049316	6,25566499	4,55074588	0,06088226	0,29304255	LSG1	large 60S subunit nuclear export GTPase 1 [Source:HGNC Symbol;Acc:HGNC:25652]

ENSG00000125895	0,51539123	1,77038428	4,55002033	0,06089925	0,29304255	TMEM74B	transmembrane protein 74B [Source:HGNC Symbol;Acc:HGNC:15893]
ENSG00000146670	0,36985036	6,40557733	4,5489906	0,06092339	0,29306215	CDCA5	cell division cycle associated 5 [Source:HGNC Symbol;Acc:HGNC:14626]
ENSG00000099834	-1,141683	0,08230323	4,54666827	0,06097786	0,29312013	CDHR5	cadherin related family member 5 [Source:HGNC Symbol;Acc:HGNC:7521]
ENSG00000118292	-1,1591248	-1,5881473	4,54505647	0,0610157	0,29312013	C1orf54	chromosome 1 open reading frame 54 [Source:HGNC Symbol;Acc:HGNC:26258]
ENSG00000154639	0,34913436	6,58032507	4,54559781	0,06100298	0,29312013	CXADR	CXADR Ig-like cell adhesion molecule [Source:HGNC Symbol;Acc:HGNC:2559]
ENSG00000181031	-0,3300342	2,86552572	4,54507805	0,06101519	0,29312013	RPH3AL	rabphilin 3A like (without C2 domains) [Source:HGNC Symbol;Acc:HGNC:10296]
ENSG00000079482	0,68670486	0,38256189	4,54256015	0,06107436	0,29330551	OPHN1	oligophrenin 1 [Source:HGNC Symbol;Acc:HGNC:8148]
ENSG00000134255	-0,327825	3,90068361	4,53932437	0,06115051	0,29357469	CEPT1	choline/ethanolamine phosphotransferase 1 [Source:HGNC Symbol;Acc:HGNC:24289]
ENSG00000148335	-0,3093929	6,57677868	4,53838418	0,06117266	0,29358454	NTMT1	N-terminal Xaa-Pro-Lys N-methyltransferase 1 [Source:HGNC Symbol;Acc:HGNC:23373]
ENSG00000006007	0,31707817	7,46068871	4,53203738	0,06132243	0,29362811	GDE1	glycerophosphodiester phosphodiesterase 1 [Source:HGNC Symbol;Acc:HGNC:29644]
ENSG00000104760	0,44341548	7,1410388	4,53292784	0,06130139	0,29362811	FGL1	fibrinogen like 1 [Source:HGNC Symbol;Acc:HGNC:3695]
ENSG00000106799	0,32830093	3,87807691	4,53489872	0,06125485	0,29362811	TGFBR1	transforming growth factor beta receptor 1 [Source:HGNC Symbol;Acc:HGNC:11772]
ENSG00000121931	0,30490268	4,78155695	4,53245213	0,06131263	0,29362811	LRIF1	ligand dependent nuclear receptor interacting factor 1 [Source:HGNC Symbol;Acc:HGNC:30299]
ENSG00000131116	0,32226546	3,79955574	4,53295661	0,06130071	0,29362811	ZNF428	zinc finger protein 428 [Source:HGNC Symbol;Acc:HGNC:20804]
ENSG00000177613	0,38124903	3,03483449	4,53302941	0,06129899	0,29362811	CSTF2T	cleavage stimulation factor subunit 2 tau variant [Source:HGNC Symbol;Acc:HGNC:17086]
ENSG00000203880	0,37099513	4,15747381	4,53425331	0,06127009	0,29362811	PCMTD2	protein-L-isoaspartate (D-aspartate) O-methyltransferase domain containing 2 [Source:HGNC Symbol;Acc:HGNC:1
ENSG00000092208	0,29846526	6,34435303	4,5264401	0,06145489	0,29368484	GEMIN2	gem nuclear organelle associated protein 2 [Source:HGNC Symbol;Acc:HGNC:10884]
ENSG00000107960	-0,4842351	4,0930054	4,5276841	0,06142542	0,29368484	STN1	STN1 subunit of CST complex [Source:HGNC Symbol;Acc:HGNC:26200]
ENSG00000138835	-0,3326092	5,14600551	4,52653786	0,06145258	0,29368484	RGS3	regulator of G protein signaling 3 [Source:HGNC Symbol;Acc:HGNC:9999]
ENSG00000151136	-0,3536253	2,49604362	4,52889793	0,06139668	0,29368484	BTBD11	BTB domain containing 11 [Source:HGNC Symbol;Acc:HGNC:23844]
ENSG00000160691	-0,2872414	6,40296144	4,5268173	0,06144596	0,29368484	SHC1	SHC adaptor protein 1 [Source:HGNC Symbol;Acc:HGNC:10840]
ENSG00000213339	-0,3429757	5,39808123	4,52983212	0,06137458	0,29368484	QTRT1	queuine tRNA-ribosyltransferase catalytic subunit 1 [Source:HGNC Symbol;Acc:HGNC:23797]
ENSG00000124713	-0,5865754	1,66764059	4,52423175	0,06150726	0,29381619	GNMT	glycine N-methyltransferase [Source:HGNC Symbol;Acc:HGNC:4415]
ENSG00000164125	0,3762298	5,16126779	4,52358491	0,0615226	0,29381619	GASK1B	golgi associated kinase 1B [Source:HGNC Symbol;Acc:HGNC:25312]
ENSG00000101138	0,3114061	6,16537994	4,51791241	0,0616574	0,29432114	CSTF1	cleavage stimulation factor subunit 1 [Source:HGNC Symbol;Acc:HGNC:2483]
ENSG00000139187	1,25490441	-0,9118839	4,51744064	0,06166863	0,29432114	KLRG1	killer cell lectin like receptor G1 [Source:HGNC Symbol;Acc:HGNC:6380]
ENSG00000100490	-0,791697	-0,0107419	4,5133623	0,06176579	0,29450475	CDKL1	cyclin dependent kinase like 1 [Source:HGNC Symbol;Acc:HGNC:1781]
ENSG00000114805	0,5276748	1,02796528	4,51253224	0,06178559	0,29450475	PLCH1	phospholipase C eta 1 [Source:HGNC Symbol;Acc:HGNC:29185]
ENSG00000179271	-0,28977	7,0095167	4,51228708	0,06179144	0,29450475	GADD45GIP1	GADD45G interacting protein 1 [Source:HGNC Symbol;Acc:HGNC:29996]
ENSG00000204386	-0,2769314	10,0687865	4,51120001	0,06181739	0,29450475	NEU1	neuraminidase 1 [Source:HGNC Symbol;Acc:HGNC:7758]
ENSG00000231169	-0,4647715	1,29183867	4,51462583	0,06173567	0,29450475	EEF1B2P1	eukaryotic translation elongation factor 1 beta 2 pseudogene 1 [Source:HGNC Symbol;Acc:HGNC:3207]
ENSG00000231720	-0,8061015	0,14227453	4,51075322	0,06182805	0,29450475	AL353747.3	novel transcript
ENSG00000256087	0,53843507	1,62560765	4,50977816	0,06185134	0,29451965	ZNF432	zinc finger protein 432 [Source:HGNC Symbol;Acc:HGNC:20810]
ENSG00000203485	-0,3339018	5,09519227	4,50250653	0,06202536	0,29515588	INF2	inverted formin 2 [Source:HGNC Symbol;Acc:HGNC:23791]
ENSG00000281087	-0,3675765	2,54146539	4,50301838	0,0620131	0,29515588	AC135068.7	ribosomal protein S8 (RPS8) pseudogene
ENSG00000118855	0,31703097	5,54829774	4,50029956	0,0620783	0,2953116	MFSD1	major facilitator superfamily domain containing 1 [Source:HGNC Symbol;Acc:HGNC:25874]
ENSG00000117139	-0,4059627	4,78805853	4,49803084	0,06213278	0,29534057	KDM5B	lysine demethylase 5B [Source:HGNC Symbol;Acc:HGNC:18039]
ENSG00000135074	-0,7273183	0,21496326	4,49752057	0,06214504	0,29534057	ADAM19	ADAM metallopeptidase domain 19 [Source:HGNC Symbol;Acc:HGNC:197]
ENSG00000166450	0,61451628	3,74584562	4,49854145	0,06212051	0,29534057	PRTG	protogenin [Source:HGNC Symbol;Acc:HGNC:26373]
ENSG00000275074	-0,4892926	2,07582939	4,49633272	0,06217359	0,29538018	NUDT18	nudix hydrolase 18 [Source:HGNC Symbol;Acc:HGNC:26194]
ENSG00000136379	0,48339624	2,39788871	4,49153435	0,06228911	0,29583276	ABHD17C	abhydrolase domain containing 17C, depalmitoylase [Source:HGNC Symbol;Acc:HGNC:26925]
ENSG00000141503	0,33965473	5,50046117	4,48725534	0,06239234	0,29584218	MINK1	misshapen like kinase 1 [Source:HGNC Symbol;Acc:HGNC:17565]
ENSG00000168246	-0,4136465	3,65300085	4,48856883	0,06236063	0,29584218	UBTD2	ubiquitin domain containing 2 [Source:HGNC Symbol;Acc:HGNC:24463]
ENSG00000169679	0,33690168	5,97770346	4,48771529	0,06238124	0,29584218	BUB1	BUB1 mitotic checkpoint serine/threonine kinase [Source:HGNC Symbol;Acc:HGNC:1148]

ENSG00000218175	-0,4121688	2,58526991	4,48928744	0,06234329	0,29584218	AC016739.1	ribosomal protein, large, P1 (RPLP1) pseudogene
ENSG00000259746	-0,5212318	1,26759668	4,48977253	0,06233159	0,29584218	HSPE1P3	heat shock protein family E (Hsp10) member 1 pseudogene 3 [Source:HGNC Symbol;Acc:HGNC:49322]
ENSG00000167723	0,95875089	-1,3066361	4,48487626	0,06244984	0,2960187	TRPV3	transient receptor potential cation channel subfamily V member 3 [Source:HGNC Symbol;Acc:HGNC:18084]
ENSG00000083093	0,35166109	4,75093183	4,48316585	0,06249121	0,29610618	PALB2	partner and localizer of BRCA2 [Source:HGNC Symbol;Acc:HGNC:26144]
ENSG00000166831	0,57837355	0,78443863	4,48243792	0,06250883	0,29610618	RBPMS2	RNA binding protein, mRNA processing factor 2 [Source:HGNC Symbol;Acc:HGNC:19098]
ENSG00000172062	0,38983581	3,75815455	4,47888906	0,06259481	0,29641737	SMN1	survival of motor neuron 1, telomeric [Source:HGNC Symbol;Acc:HGNC:11117]
ENSG00000160949	-0,3496124	5,02407284	4,47431984	0,06270574	0,29665417	TONSL	tonsoku like, DNA repair protein [Source:HGNC Symbol;Acc:HGNC:7801]
ENSG00000212907	0,50645999	7,97438435	4,47486604	0,06269246	0,29665417	MT-ND4L	mitochondrially encoded NADH:ubiquinone oxidoreductase core subunit 4L [Source:HGNC Symbol;Acc:HGNC:746
ENSG00000263001	0,38218962	5,7163965	4,47589913	0,06266737	0,29665417	GTF2I	general transcription factor IIi [Source:HGNC Symbol;Acc:HGNC:4659]
ENSG00000198189	0,3041434	7,32921133	4,47279746	0,06274275	0,29673318	HSD17B11	hydroxysteroid 17-beta dehydrogenase 11 [Source:HGNC Symbol;Acc:HGNC:22960]
ENSG00000163399	0,42126322	10,1845101	4,47084522	0,06279025	0,29686174	ATP1A1	ATPase Na+/K+ transporting subunit alpha 1 [Source:HGNC Symbol;Acc:HGNC:799]
ENSG00000134109	-0,3432586	4,5807202	4,46824886	0,0628535	0,29696926	EDEM1	ER degradation enhancing alpha-mannosidase like protein 1 [Source:HGNC Symbol;Acc:HGNC:18967]
ENSG00000136381	0,35434321	5,10265496	4,46824262	0,06285365	0,29696926	IREB2	iron responsive element binding protein 2 [Source:HGNC Symbol;Acc:HGNC:6115]
ENSG00000165782	0,37207298	5,95491003	4,46693837	0,06288545	0,29702346	PIP4P1	phosphatidylinositol-4,5-bisphosphate 4-phosphatase 1 [Source:HGNC Symbol;Acc:HGNC:19299]
ENSG00000156510	-0,7470739	5,52840233	4,4653982	0,06292303	0,2971049	HKDC1	hexokinase domain containing 1 [Source:HGNC Symbol;Acc:HGNC:23302]
ENSG00000015676	0,30307666	5,76286537	4,46370982	0,06296426	0,29710751	NUDCD3	NudC domain containing 3 [Source:HGNC Symbol;Acc:HGNC:22208]
ENSG00000108256	0,32299122	5,0801613	4,46431108	0,06294957	0,29710751	NUFIP2	nuclear FMR1 interacting protein 2 [Source:HGNC Symbol;Acc:HGNC:17634]
ENSG00000107863	0,47534049	4,00578482	4,4624304	0,06299552	0,29715906	ARHGAP21	Rho GTPase activating protein 21 [Source:HGNC Symbol;Acc:HGNC:23725]
ENSG00000196865	0,33575547	3,43286056	4,45929522	0,06307222	0,29723291	NHLRC2	NHL repeat containing 2 [Source:HGNC Symbol;Acc:HGNC:24731]
ENSG00000234743	-0,4619154	1,31816504	4,46012584	0,06305189	0,29723291	EIF5AP4	eukaryotic translation initiation factor 5A pseudogene 4 [Source:HGNC Symbol;Acc:HGNC:31442]
ENSG00000264278	-0,9152994	-1,2169388	4,46035232	0,06304635	0,29723291	ZNF236-DT	ZNF236 divergent transcript [Source:HGNC Symbol;Acc:HGNC:53779]
ENSG00000132436	0,39701527	2,95904145	4,45256468	0,06323726	0,2972436	FIGNL1	fidgetin like 1 [Source:HGNC Symbol;Acc:HGNC:13286]
ENSG00000143578	-0,3555261	3,74406063	4,45394027	0,06320348	0,2972436	CREB3L4	cAMP responsive element binding protein 3 like 4 [Source:HGNC Symbol;Acc:HGNC:18854]
ENSG00000151692	0,35412333	2,75011001	4,4526931	0,0632341	0,2972436	RNF144A	ring finger protein 144A [Source:HGNC Symbol;Acc:HGNC:20457]
ENSG00000169660	-0,3429637	3,28202581	4,45575155	0,06315905	0,2972436	HEXD	hexosaminidase D [Source:HGNC Symbol;Acc:HGNC:26307]
ENSG00000182492	-0,7736376	-0,2029106	4,45408487	0,06319993	0,2972436	BGN	biglycan [Source:HGNC Symbol;Acc:HGNC:1044]
ENSG00000196152	-0,4141459	2,86496857	4,45456102	0,06318825	0,2972436	ZNF79	zinc finger protein 79 [Source:HGNC Symbol;Acc:HGNC:13153]
ENSG00000196296	-0,4963528	1,06562108	4,4530428	0,06322552	0,2972436	ATP2A1	ATPase sarcoplasmic/endoplasmic reticulum Ca2+ transporting 1 [Source:HGNC Symbol;Acc:HGNC:811]
ENSG00000272555	-0,6738391	0,37270981	4,45307974	0,06322461	0,2972436	AC009974.1	novel transcript
ENSG00000018510	0,3384703	6,42176257	4,44864615	0,0633336	0,29747307	AGPS	alkylglycerone phosphate synthase [Source:HGNC Symbol;Acc:HGNC:327]
ENSG00000122121	-0,5295456	1,82472364	4,44957634	0,06331071	0,29747307	XPNPEP2	X-prolyl aminopeptidase 2 [Source:HGNC Symbol;Acc:HGNC:12823]
ENSG00000144747	-0,382508	4,49089455	4,44809482	0,06334716	0,29747307	TMF1	TATA element modulatory factor 1 [Source:HGNC Symbol;Acc:HGNC:11870]
ENSG00000113594	0,44680279	2,10142343	4,44395086	0,06344927	0,29785681	LIFR	LIF receptor subunit alpha [Source:HGNC Symbol;Acc:HGNC:6597]
ENSG00000088836	0,49787263	4,33969322	4,44227201	0,0634907	0,29787489	SLC4A11	solute carrier family 4 member 11 [Source:HGNC Symbol;Acc:HGNC:16438]
ENSG00000105618	-0,2754883	6,47566273	4,44131634	0,06351429	0,29787489	PRPF31	pre-mRNA processing factor 31 [Source:HGNC Symbol;Acc:HGNC:15446]
ENSG00000110244	-0,4400939	2,13102359	4,44173463	0,06350396	0,29787489	APOA4	apolipoprotein A4 [Source:HGNC Symbol;Acc:HGNC:602]
ENSG00000070159	0,34641726	4,81219267	4,43951191	0,06355887	0,2979299	PTPN3	protein tyrosine phosphatase non-receptor type 3 [Source:HGNC Symbol;Acc:HGNC:9655]
ENSG00000183207	-0,3714406	7,34212405	4,43919093	0,06356681	0,2979299	RUVBL2	RuvB like AAA ATPase 2 [Source:HGNC Symbol;Acc:HGNC:10475]
ENSG00000005812	0,35509341	3,79984171	4,43603864	0,0636448	0,29794458	FBXL3	F-box and leucine rich repeat protein 3 [Source:HGNC Symbol;Acc:HGNC:13599]
ENSG00000101194	-0,2935739	7,45286723	4,43793139	0,06359796	0,29794458	SLC17A9	solute carrier family 17 member 9 [Source:HGNC Symbol;Acc:HGNC:16192]
ENSG00000125755	-0,4119458	5,6243485	4,43458696	0,06368076	0,29794458	SYMPK	symplekin [Source:HGNC Symbol;Acc:HGNC:22935]
ENSG00000138756	0,40479015	2,76051745	4,4348392	0,06367451	0,29794458	BMP2K	BMP2 inducible kinase [Source:HGNC Symbol;Acc:HGNC:18041]
ENSG00000149231	-0,3390903	3,10695325	4,43412074	0,06369231	0,29794458	CCDC82	coiled-coil domain containing 82 [Source:HGNC Symbol;Acc:HGNC:26282]
ENSG00000214029	0,55926768	1,51611932	4,43625289	0,06363949	0,29794458	ZNF891	zinc finger protein 891 [Source:HGNC Symbol;Acc:HGNC:38709]

ENSG00000146281	0,35646709	3,77962041	4,43298718	0,06372041	0,29798062	PM20D2	peptidase M20 domain containing 2 [Source:HGNC Symbol;Acc:HGNC:21408]
ENSG00000104957	-0,3117085	3,44805073	4,42995552	0,06379564	0,29823696	CCDC130	coiled-coil domain containing 130 [Source:HGNC Symbol;Acc:HGNC:28118]
ENSG00000132623	-0,3805664	2,85111516	4,42809163	0,06384195	0,29830159	ANKEF1	ankyrin repeat and EF-hand domain containing 1 [Source:HGNC Symbol;Acc:HGNC:15803]
ENSG00000188338	0,40738628	6,16506388	4,42775554	0,0638503	0,29830159	SLC38A3	solute carrier family 38 member 3 [Source:HGNC Symbol;Acc:HGNC:18044]
ENSG00000266472	-0,3128202	6,12806768	4,42535562	0,06391	0,29848503	MRPS21	mitochondrial ribosomal protein S21 [Source:HGNC Symbol;Acc:HGNC:14046]
ENSG00000135341	-0,2988096	5,14040276	4,42364184	0,06395267	0,29849347	MAP3K7	mitogen-activated protein kinase kinase kinase 7 [Source:HGNC Symbol;Acc:HGNC:6859]
ENSG00000205138	-0,3392496	4,77521216	4,42370699	0,06395105	0,29849347	SDHAF1	succinate dehydrogenase complex assembly factor 1 [Source:HGNC Symbol;Acc:HGNC:33867]
ENSG00000158470	0,29635399	5,40638089	4,42174468	0,06399995	0,2985234	B4GALT5	beta-1,4-galactosyltransferase 5 [Source:HGNC Symbol;Acc:HGNC:928]
ENSG00000174721	-0,4945401	1,34381003	4,42180379	0,06399848	0,2985234	FGFBP3	fibroblast growth factor binding protein 3 [Source:HGNC Symbol;Acc:HGNC:23428]
ENSG00000143320	-0,8115973	-0,7214536	4,42031114	0,06403571	0,29859484	CRABP2	cellular retinoic acid binding protein 2 [Source:HGNC Symbol;Acc:HGNC:2339]
ENSG00000121542	0,31510346	3,91115893	4,41568691	0,06415121	0,29885646	SEC22A	SEC22 homolog A, vesicle trafficking protein [Source:HGNC Symbol;Acc:HGNC:20260]
ENSG00000189343	-0,3245049	3,61465913	4,41560811	0,06415318	0,29885646	RPS2P46	ribosomal protein S2 pseudogene 46 [Source:HGNC Symbol;Acc:HGNC:35839]
ENSG00000237765	0,66729591	0,55536024	4,41719701	0,06411346	0,29885646	FAM200B	family with sequence similarity 200 member B [Source:HGNC Symbol;Acc:HGNC:27740]
ENSG00000133639	0,41164724	3,47095724	4,41213192	0,06424019	0,29888055	BTG1	BTG anti-proliferation factor 1 [Source:HGNC Symbol;Acc:HGNC:1130]
ENSG00000142544	-0,5410681	0,97769674	4,41284728	0,06422227	0,29888055	CTU1	cytosolic thiouridylase subunit 1 [Source:HGNC Symbol;Acc:HGNC:29590]
ENSG00000149761	-0,4324189	4,74711003	4,41271092	0,06422569	0,29888055	NUDT22	nudix hydrolase 22 [Source:HGNC Symbol;Acc:HGNC:28189]
ENSG00000188229	-0,371749	9,53942584	4,41423542	0,06418752	0,29888055	TUBB4B	tubulin beta 4B class IVb [Source:HGNC Symbol;Acc:HGNC:20771]
ENSG00000240674	-0,8303028	-0,6711338	4,41117586	0,06426414	0,29889681	AC106872.2	ribosomal protein L21 (RPL21) pseudogene
ENSG00000224687	1,45189647	-1,4541238	4,40948877	0,06430644	0,29890322	RASAL2-AS1	RASAL2 antisense RNA 1 [Source:HGNC Symbol;Acc:HGNC:44170]
ENSG00000287569	0,88856058	-1,045613	4,40986498	0,06429701	0,29890322	AL078587.2	novel transcript
ENSG00000004777	0,90646518	-0,428996	4,40576077	0,06440004	0,29916503	ARHGAP33	Rho GTPase activating protein 33 [Source:HGNC Symbol;Acc:HGNC:23085]
ENSG00000099817	-0,2798997	8,52620194	4,40561403	0,06440372	0,29916503	POLR2E	RNA polymerase II, I and III subunit E [Source:HGNC Symbol;Acc:HGNC:9192]
ENSG00000175701	-0,3456177	4,2024832	4,39848569	0,06458317	0,29980794	MTLN	mitoregulin [Source:HGNC Symbol;Acc:HGNC:27339]
ENSG00000233913	-0,366001	2,7782409	4,39867965	0,06457828	0,29980794	RPL10P9	ribosomal protein L10 pseudogene 9 [Source:HGNC Symbol;Acc:HGNC:35579]
ENSG00000186866	0,44731005	5,82642364	4,39611895	0,06464289	0,29998984	POFUT2	protein O-fucosyltransferase 2 [Source:HGNC Symbol;Acc:HGNC:14683]
ENSG00000117906	0,29485269	7,93680965	4,39133776	0,06476374	0,30045524	RCN2	reticulocalbin 2 [Source:HGNC Symbol;Acc:HGNC:9935]
ENSG00000111816	-0,4538936	2,20087175	4,39024239	0,06479147	0,30048845	FRK	fyn related Src family tyrosine kinase [Source:HGNC Symbol;Acc:HGNC:3955]
ENSG00000173276	0,40675129	2,05819996	4,38861079	0,0648328	0,3005847	ZBTB21	zinc finger and BTB domain containing 21 [Source:HGNC Symbol;Acc:HGNC:13083]
ENSG00000170836	0,31698473	4,94538309	4,38769892	0,06485591	0,30059645	PPM1D	protein phosphatase, Mg2+/Mn2+ dependent 1D [Source:HGNC Symbol;Acc:HGNC:9277]
ENSG00000087088	-0,3474514	7,35984419	4,38037419	0,06504194	0,30061201	BAX	BCL2 associated X, apoptosis regulator [Source:HGNC Symbol;Acc:HGNC:959]
ENSG00000100749	-0,3133177	4,95807674	4,37368011	0,06521253	0,30061201	VRK1	VRK serine/threonine kinase 1 [Source:HGNC Symbol;Acc:HGNC:12718]
ENSG00000110492	0,29454044	9,61990912	4,37409931	0,06520183	0,30061201	MDK	midkine [Source:HGNC Symbol;Acc:HGNC:6972]
ENSG00000111364	-0,3533446	5,30427284	4,38228439	0,06499336	0,30061201	DDX55	DEAD-box helicase 55 [Source:HGNC Symbol;Acc:HGNC:20085]
ENSG00000114302	0,59420728	5,26046236	4,37905316	0,06507556	0,30061201	PRKAR2A	protein kinase cAMP-dependent type II regulatory subunit alpha [Source:HGNC Symbol;Acc:HGNC:9391]
ENSG00000116898	-0,316819	7,18689535	4,37681158	0,06513266	0,30061201	MRPS15	mitochondrial ribosomal protein S15 [Source:HGNC Symbol;Acc:HGNC:14504]
ENSG00000126456	-0,2727151	6,09328282	4,38183214	0,06500486	0,30061201	IRF3	interferon regulatory factor 3 [Source:HGNC Symbol;Acc:HGNC:6118]
ENSG00000138018	0,37947815	5,74126568	4,38391238	0,064952	0,30061201	SELENOI	selenoprotein I [Source:HGNC Symbol;Acc:HGNC:29361]
ENSG00000139437	-0,4474031	3,26342742	4,3731498	0,06522607	0,30061201	TCHP	trichoplein keratin filament binding [Source:HGNC Symbol;Acc:HGNC:28135]
ENSG00000148090	0,31846206	5,07395394	4,3743044	0,0651966	0,30061201	AUH	AU RNA binding methylglutaconyl-CoA hydratase [Source:HGNC Symbol;Acc:HGNC:890]
ENSG00000161091	-0,2789289	5,87612071	4,37780335	0,06510739	0,30061201	MFSD12	major facilitator superfamily domain containing 12 [Source:HGNC Symbol;Acc:HGNC:28299]
ENSG00000163975	-0,4414108	1,75133218	4,37915614	0,06507294	0,30061201	MELTF	melanotransferrin [Source:HGNC Symbol;Acc:HGNC:7037]
ENSG00000167566	0,4338133	4,2774294	4,3813626	0,0650168	0,30061201	NCKAP5L	NCK associated protein 5 like [Source:HGNC Symbol;Acc:HGNC:29321]
ENSG00000175087	0,7068036	0,38588197	4,3726095	0,06523987	0,30061201	PDIK1L	PDLIM1 interacting kinase 1 like [Source:HGNC Symbol;Acc:HGNC:18981]
ENSG00000176654	-0,795711	-0,5176343	4,38450756	0,06493688	0,30061201	NANOGP1	Nanog homeobox pseudogene 1 [Source:HGNC Symbol;Acc:HGNC:23099]

ENSG00000225285	-1,2783025	-1,3642931	4,37220361	0,06525024	0,30061201	LINC01770	long intergenic non-protein coding RNA 1770 [Source:HGNC Symbol;Acc:HGNC:52560]
ENSG00000244026	-0,3853475	2,80307845	4,37648989	0,06514086	0,30061201	FAM86DP	family with sequence similarity 86 member D, pseudogene [Source:HGNC Symbol;Acc:HGNC:32659]
ENSG00000275202	-0,966673	-0,3859184	4,3819269	0,06500245	0,30061201	AL161421.1	novel transcript, antisense to FNDC3A
ENSG00000277559	-1,0434318	-0,9052467	4,38390453	0,06495219	0,30061201	AC018553.1	novel transcript
ENSG00000008988	-0,3571924	10,0388448	4,37027378	0,06529956	0,30074439	RPS20	ribosomal protein S20 [Source:HGNC Symbol;Acc:HGNC:10405]
ENSG00000104660	-0,2908594	5,83076422	4,36851658	0,06534451	0,30076178	LEPROTL1	leptin receptor overlapping transcript like 1 [Source:HGNC Symbol;Acc:HGNC:6555]
ENSG00000205362	-0,4633332	1,05999642	4,36902673	0,06533145	0,30076178	MT1A	metallothionein 1A [Source:HGNC Symbol;Acc:HGNC:7393]
ENSG00000102981	0,42257392	2,31774653	4,3669287	0,06538516	0,30085409	PARD6A	par-6 family cell polarity regulator alpha [Source:HGNC Symbol;Acc:HGNC:15943]
ENSG00000111696	-0,4387634	2,71790546	4,36237308	0,06550196	0,30122346	NT5DC3	5'-nucleotidase domain containing 3 [Source:HGNC Symbol;Acc:HGNC:30826]
ENSG00000178814	-0,6059891	3,89992726	4,36218961	0,06550667	0,30122346	OPLAH	5-oxoprolinase, ATP-hydrolysing [Source:HGNC Symbol;Acc:HGNC:8149]
ENSG00000100379	-0,4515591	1,73792947	4,36066814	0,06554574	0,30122901	KCTD17	potassium channel tetramerization domain containing 17 [Source:HGNC Symbol;Acc:HGNC:25705]
ENSG00000163888	0,98376407	-1,4048401	4,35995089	0,06556417	0,30122901	CAMK2N2	calcium/calmodulin dependent protein kinase II inhibitor 2 [Source:HGNC Symbol;Acc:HGNC:24197]
ENSG00000197329	0,40495336	3,53016209	4,35973441	0,06556973	0,30122901	PELI1	pellino E3 ubiquitin protein ligase 1 [Source:HGNC Symbol;Acc:HGNC:8827]
ENSG00000225918	-0,8584101	-0,8502253	4,35529444	0,06568397	0,30165896	RPL7P59	ribosomal protein L7 pseudogene 59 [Source:HGNC Symbol;Acc:HGNC:49215]
ENSG00000090905	0,617627	3,79108175	4,35384443	0,06572134	0,3017357	TNRC6A	trinucleotide repeat containing adaptor 6A [Source:HGNC Symbol;Acc:HGNC:11969]
ENSG00000061794	0,30836083	6,53325116	4,34332403	0,06599321	0,30288873	MRPS35	mitochondrial ribosomal protein S35 [Source:HGNC Symbol;Acc:HGNC:16635]
ENSG00000115419	0,4444457	6,02959733	4,34132201	0,06604511	0,30293658	GLS	glutaminase [Source:HGNC Symbol;Acc:HGNC:4331]
ENSG00000229980	-0,7165496	-0,1141242	4,34134855	0,06604442	0,30293658	TOB1-AS1	TOB1 antisense RNA 1 [Source:HGNC Symbol;Acc:HGNC:44340]
ENSG00000080819	0,33097583	5,62926896	4,33982334	0,06608399	0,30295259	CPOX	coproporphyrinogen oxidase [Source:HGNC Symbol;Acc:HGNC:2321]
ENSG00000125458	-0,2938098	5,26176931	4,33958901	0,06609007	0,30295259	NT5C	5', 3'-nucleotidase, cytosolic [Source:HGNC Symbol;Acc:HGNC:17144]
ENSG00000102804	0,35237017	5,29622699	4,33710089	0,0661547	0,30295589	TSC22D1	TSC22 domain family member 1 [Source:HGNC Symbol;Acc:HGNC:16826]
ENSG00000109775	-0,3062232	4,45584983	4,33601482	0,06618293	0,30295589	UFSP2	UFM1 specific peptidase 2 [Source:HGNC Symbol;Acc:HGNC:25640]
ENSG00000119669	0,34490372	4,03241924	4,33449281	0,06622253	0,30295589	IRF2BPL	interferon regulatory factor 2 binding protein like [Source:HGNC Symbol;Acc:HGNC:14282]
ENSG00000119737	1,07128427	-1,2600743	4,33361726	0,06624531	0,30295589	GPR75	G protein-coupled receptor 75 [Source:HGNC Symbol;Acc:HGNC:4526]
ENSG00000156453	0,7769325	-0,2561569	4,33318017	0,06625669	0,30295589	PCDH1	protocadherin 1 [Source:HGNC Symbol;Acc:HGNC:8655]
ENSG00000168118	0,41599416	6,67675957	4,33615621	0,06617926	0,30295589	RAB4A	RAB4A, member RAS oncogene family [Source:HGNC Symbol;Acc:HGNC:9781]
ENSG00000179262	-0,283042	7,30006292	4,33782621	0,06613585	0,30295589	RAD23A	RAD23 homolog A, nucleotide excision repair protein [Source:HGNC Symbol;Acc:HGNC:9812]
ENSG00000240356	-0,420063	1,62456837	4,33431719	0,0662271	0,30295589	RPL23AP7	ribosomal protein L23a pseudogene 7 [Source:HGNC Symbol;Acc:HGNC:17336]
ENSG00000104953	-1,2580047	-1,5156915	4,32709518	0,06641538	0,3033724	TLE6	TLE family member 6, subcortical maternal complex member [Source:HGNC Symbol;Acc:HGNC:30788]
ENSG00000111845	-0,2917816	6,06824072	4,32570834	0,06645162	0,3033724	PAK1IP1	PAK1 interacting protein 1 [Source:HGNC Symbol;Acc:HGNC:20882]
ENSG00000156313	0,39296015	3,55814085	4,32624255	0,06643766	0,3033724	RPGR	retinitis pigmentosa GTPase regulator [Source:HGNC Symbol;Acc:HGNC:10295]
ENSG00000198586	0,31739451	4,56591428	4,32701525	0,06641747	0,3033724	TLK1	tousled like kinase 1 [Source:HGNC Symbol;Acc:HGNC:11841]
ENSG00000234322	0,82550321	-0,9783655	4,32574422	0,06645068	0,3033724	ST13P18	ST13, Hsp70 interacting protein pseudogene 18 [Source:HGNC Symbol;Acc:HGNC:38861]
ENSG00000263934	1,06544131	-1,5901129	4,32092656	0,06657674	0,30384867	SNORD3A	small nucleolar RNA, C/D box 3A [Source:HGNC Symbol;Acc:HGNC:33189]
ENSG00000128692	-0,4296618	1,39439071	4,31671815	0,0666871	0,30397251	EIF2S2P4	eukaryotic translation initiation factor 2 subunit 2 beta pseudogene 4 [Source:HGNC Symbol;Acc:HGNC:37626]
ENSG00000168522	-0,3159952	4,56550812	4,31676909	0,06668577	0,30397251	FNTA	farnesyltransferase, CAAX box, alpha [Source:HGNC Symbol;Acc:HGNC:3782]
ENSG00000197385	0,97162197	-1,13365	4,31716159	0,06667546	0,30397251	ZNF860	zinc finger protein 860 [Source:HGNC Symbol;Acc:HGNC:34513]
ENSG00000235082	0,88857424	-1,2785954	4,31797452	0,06665413	0,30397251	SUMO1P3	SUMO1 pseudogene 3 [Source:HGNC Symbol;Acc:HGNC:33150]
ENSG00000166037	0,28324762	5,22281077	4,31549204	0,0667193	0,30402441	CEP57	centrosomal protein 57 [Source:HGNC Symbol;Acc:HGNC:30794]
ENSG00000081307	-0,2794598	5,68131088	4,31293392	0,06678654	0,30409156	UBA5	ubiquitin like modifier activating enzyme 5 [Source:HGNC Symbol;Acc:HGNC:23230]
ENSG00000087470	0,29655758	5,61422375	4,31316739	0,0667804	0,30409156	DNM1L	dynamin 1 like [Source:HGNC Symbol;Acc:HGNC:2973]
ENSG00000153714	0,46198237	1,97441826	4,31255586	0,06679648	0,30409156	LURAP1L	leucine rich adaptor protein 1 like [Source:HGNC Symbol;Acc:HGNC:31452]
ENSG00000000003	0,32434315	7,845668	4,31076339	0,06684366	0,30421152	TSPAN6	tetraspanin 6 [Source:HGNC Symbol;Acc:HGNC:11858]
ENSG00000163013	0,4255105	2,66835211	4,30900394	0,06689	0,30426559	FBXO41	F-box protein 41 [Source:HGNC Symbol;Acc:HGNC:29409]

ENSG00000205771	-0,841627	-0,6456587	4,30873108	0,06689719	0,30426559	CATSPER2P1	cation channel sperm associated 2 pseudogene 1 [Source:HGNC Symbol;Acc:HGNC:31054]
ENSG00000102543	0,45769143	1,64729591	4,30657985	0,06695392	0,30435387	CDADC1	cytidine and dCMP deaminase domain containing 1 [Source:HGNC Symbol;Acc:HGNC:20299]
ENSG00000128512	0,55265182	2,91083158	4,30641495	0,06695827	0,30435387	DOCK4	dedicator of cytokinesis 4 [Source:HGNC Symbol;Acc:HGNC:19192]
ENSG00000027001	0,38546282	5,0101867	4,29527963	0,06725289	0,30550296	MIPEP	mitochondrial intermediate peptidase [Source:HGNC Symbol;Acc:HGNC:7104]
ENSG00000170242	0,34391949	4,83880981	4,29538981	0,06724997	0,30550296	USP47	ubiquitin specific peptidase 47 [Source:HGNC Symbol;Acc:HGNC:20076]
ENSG00000140848	-0,4483798	2,7347062	4,29276857	0,06731956	0,30571072	CPNE2	copine 2 [Source:HGNC Symbol;Acc:HGNC:2315]
ENSG00000204934	-0,5514849	0,86372431	4,2894082	0,0674089	0,30594403	ATP6V0E2-AS1	ATP6V0E2 antisense RNA 1 [Source:HGNC Symbol;Acc:HGNC:44180]
ENSG00000225573	-0,3710111	2,90369704	4,28926091	0,06741282	0,30594403	RPL35P5	ribosomal protein L35 pseudogene 5 [Source:HGNC Symbol;Acc:HGNC:36955]
ENSG00000111859	0,34293183	6,30311755	4,28827741	0,06743899	0,30596778	NEDD9	neural precursor cell expressed, developmentally down-regulated 9 [Source:HGNC Symbol;Acc:HGNC:7733]
ENSG00000205403	-0,3285052	5,36925709	4,28216702	0,06760192	0,30661175	CFI	complement factor I [Source:HGNC Symbol;Acc:HGNC:5394]
ENSG00000138207	-0,2824886	10,7221833	4,28115319	0,067629	0,30662709	RBP4	retinol binding protein 4 [Source:HGNC Symbol;Acc:HGNC:9922]
ENSG00000185278	0,55783278	1,20227558	4,28046915	0,06764728	0,30662709	ZBTB37	zinc finger and BTB domain containing 37 [Source:HGNC Symbol;Acc:HGNC:28365]
ENSG00000164944	0,34166894	4,8230432	4,27878739	0,06769225	0,30673574	VIRMA	vir like m6A methyltransferase associated [Source:HGNC Symbol;Acc:HGNC:24500]
ENSG00000108091	0,4382356	4,60106362	4,27018177	0,06792293	0,30685021	CCDC6	coiled-coil domain containing 6 [Source:HGNC Symbol;Acc:HGNC:18782]
ENSG00000122971	-0,380837	4,69482261	4,26775282	0,06798822	0,30685021	ACADS	acyl-CoA dehydrogenase short chain [Source:HGNC Symbol;Acc:HGNC:90]
ENSG00000137404	0,33082633	5,94995253	4,27501725	0,06779319	0,30685021	NRM	nurim [Source:HGNC Symbol;Acc:HGNC:8003]
ENSG00000148154	0,46029373	3,42632971	4,2720167	0,06787366	0,30685021	UGCG	UDP-glucose ceramide glucosyltransferase [Source:HGNC Symbol;Acc:HGNC:12524]
ENSG00000150477	0,56359171	1,15174863	4,27406388	0,06781874	0,30685021	KIAA1328	KIAA1328 [Source:HGNC Symbol;Acc:HGNC:29248]
ENSG00000154016	-0,4901996	0,88747338	4,26812358	0,06797825	0,30685021	GRAP	GRB2 related adaptor protein [Source:HGNC Symbol;Acc:HGNC:4562]
ENSG00000175792	-0,3579657	6,76366835	4,26614584	0,06803146	0,30685021	RUVBL1	RuvB like AAA ATPase 1 [Source:HGNC Symbol;Acc:HGNC:10474]
ENSG00000182899	-0,3144481	9,64927563	4,26657409	0,06801993	0,30685021	RPL35A	ribosomal protein L35a [Source:HGNC Symbol;Acc:HGNC:10345]
ENSG00000183801	0,777168	0,94187241	4,26970592	0,06793571	0,30685021	OLFML1	olfactomedin like 1 [Source:HGNC Symbol;Acc:HGNC:24473]
ENSG00000188559	0,50790955	3,65141862	4,27483745	0,06779801	0,30685021	RALGAPA2	Ral GTPase activating protein catalytic subunit alpha 2 [Source:HGNC Symbol;Acc:HGNC:16207]
ENSG00000226360	-0,3250046	3,04938317	4,26731249	0,06800006	0,30685021	RPL10AP6	ribosomal protein L10a pseudogene 6 [Source:HGNC Symbol;Acc:HGNC:36504]
ENSG00000228878	-0,7923158	-0,6674446	4,27071288	0,06790866	0,30685021	SEPTIN7-DT	SEPTIN7 divergent transcript [Source:HGNC Symbol;Acc:HGNC:51153]
ENSG00000242114	-0,3992082	1,8776814	4,26981313	0,06793283	0,30685021	MTFP1	mitochondrial fission process 1 [Source:HGNC Symbol;Acc:HGNC:26945]
ENSG00000259674	-1,3516686	-1,4274622	4,26610446	0,06803257	0,30685021	AC092868.1	ribosomal protein L7a (RPL7A) pseudogene
ENSG00000275835	-0,3457907	3,52813043	4,26679943	0,06801387	0,30685021	TUBGCP5	tubulin gamma complex associated protein 5 [Source:HGNC Symbol;Acc:HGNC:18600]
ENSG00000187266	0,34742636	2,36587472	4,26494465	0,0680638	0,30689631	EPOR	erythropoietin receptor [Source:HGNC Symbol;Acc:HGNC:3416]
ENSG00000156958	-0,3236667	4,22947366	4,26132569	0,06816136	0,30720439	GALK2	galactokinase 2 [Source:HGNC Symbol;Acc:HGNC:4119]
ENSG00000169599	0,29442156	6,02846987	4,26007135	0,06819521	0,30720439	NFU1	NFU1 iron-sulfur cluster scaffold [Source:HGNC Symbol;Acc:HGNC:16287]
ENSG00000213281	0,36094839	4,88060357	4,2608422	0,0681744	0,30720439	NRAS	NRAS proto-oncogene, GTPase [Source:HGNC Symbol;Acc:HGNC:7989]
ENSG00000224531	-0,3391012	3,60991983	4,25768071	0,06825979	0,30740053	SMIM13	small integral membrane protein 13 [Source:HGNC Symbol;Acc:HGNC:27356]
ENSG00000162576	0,32811358	4,9356203	4,2567111	0,06828601	0,30742382	MXRA8	matrix remodeling associated 8 [Source:HGNC Symbol;Acc:HGNC:7542]
ENSG00000067057	-0,6517817	1,21046978	4,25028783	0,06846	0,30744915	PFKP	phosphofructokinase, platelet [Source:HGNC Symbol;Acc:HGNC:8878]
ENSG00000130707	-0,2740909	6,19663841	4,2525305	0,06839919	0,30744915	ASS1	argininosuccinate synthase 1 [Source:HGNC Symbol;Acc:HGNC:758]
ENSG00000143499	-0,2880566	5,94526056	4,25102216	0,06844008	0,30744915	SMYD2	SET and MYND domain containing 2 [Source:HGNC Symbol;Acc:HGNC:20982]
ENSG00000144035	-0,3746961	2,32390768	4,25074125	0,0684477	0,30744915	NAT8	N-acetyltransferase 8 (putative) [Source:HGNC Symbol;Acc:HGNC:18069]
ENSG00000164932	0,53150616	3,59325929	4,25168495	0,0684221	0,30744915	CTHRC1	collagen triple helix repeat containing 1 [Source:HGNC Symbol;Acc:HGNC:18831]
ENSG00000166822	0,39155104	3,21333156	4,2514298	0,06842902	0,30744915	TMEM170A	transmembrane protein 170A [Source:HGNC Symbol;Acc:HGNC:29577]
ENSG00000184203	-0,452601	4,37130707	4,25233895	0,06840438	0,30744915	PPP1R2	protein phosphatase 1 regulatory inhibitor subunit 2 [Source:HGNC Symbol;Acc:HGNC:9288]
ENSG00000251562	1,27946755	3,54179643	4,25241129	0,06840242	0,30744915	MALAT1	metastasis associated lung adenocarcinoma transcript 1 [Source:HGNC Symbol;Acc:HGNC:29665]
ENSG00000109118	0,30305133	4,24110703	4,24470314	0,06861172	0,30752407	PHF12	PHD finger protein 12 [Source:HGNC Symbol;Acc:HGNC:20816]
ENSG00000140859	0,32798302	6,28809185	4,24647807	0,06856345	0,30752407	KIFC3	kinesin family member C3 [Source:HGNC Symbol;Acc:HGNC:6326]

ENSG00000142484	-0,3169637	6,69549526	4,24252199	0,06867109	0,30752407	TM4SF5	transmembrane 4 L six family member 5 [Source:HGNC Symbol;Acc:HGNC:11857]
ENSG00000168661	0,43343757	1,76079535	4,24259099	0,06866921	0,30752407	ZNF30	zinc finger protein 30 [Source:HGNC Symbol;Acc:HGNC:13090]
ENSG00000173599	-0,4153864	5,20442967	4,24403784	0,06862982	0,30752407	PC	pyruvate carboxylase [Source:HGNC Symbol;Acc:HGNC:8636]
ENSG00000184363	-0,368302	5,40286839	4,2421495	0,06868124	0,30752407	PKP3	plakophilin 3 [Source:HGNC Symbol;Acc:HGNC:9025]
ENSG00000196943	0,44656447	6,20488737	4,24392481	0,0686329	0,30752407	NOP9	NOP9 nucleolar protein [Source:HGNC Symbol;Acc:HGNC:19826]
ENSG00000216866	-0,4022193	2,14320531	4,24533019	0,06859466	0,30752407	RPS2P55	ribosomal protein S2 pseudogene 55 [Source:HGNC Symbol;Acc:HGNC:36140]
ENSG00000272079	-0,3910956	1,97689285	4,24224633	0,0686786	0,30752407	AC004233.2	novel transcript
ENSG00000272341	0,87644556	-0,9217046	4,24193125	0,06868718	0,30752407	AL137003.1	novel transcript
ENSG00000126581	-0,4316934	5,88397399	4,24047196	0,06872695	0,30760786	BECN1	beclin 1 [Source:HGNC Symbol;Acc:HGNC:1034]
ENSG00000105514	-0,4465151	1,72818116	4,23672156	0,06882929	0,30763439	RAB3D	RAB3D, member RAS oncogene family [Source:HGNC Symbol;Acc:HGNC:9779]
ENSG00000116128	0,4911935	2,03477409	4,2348556	0,06888029	0,30763439	BCL9	BCL9 transcription coactivator [Source:HGNC Symbol;Acc:HGNC:1008]
ENSG00000120868	0,45832752	2,49903672	4,23530604	0,06886797	0,30763439	APAF1	apoptotic peptidase activating factor 1 [Source:HGNC Symbol;Acc:HGNC:576]
ENSG00000130164	-0,5728933	7,65086225	4,23544923	0,06886406	0,30763439	LDLR	low density lipoprotein receptor [Source:HGNC Symbol;Acc:HGNC:6547]
ENSG00000174177	-0,2825455	5,10858562	4,23851748	0,06878026	0,30763439	CTU2	cytosolic thiouridylase subunit 2 [Source:HGNC Symbol;Acc:HGNC:28005]
ENSG00000196597	-0,5090308	0,77267038	4,23602868	0,06884822	0,30763439	ZNF782	zinc finger protein 782 [Source:HGNC Symbol;Acc:HGNC:33110]
ENSG00000237310	-0,7729118	0,84943829	4,23578912	0,06885477	0,30763439	GS1-124K5.4	uncharacterized LOC100289098 [Source:NCBI gene (formerly Entrezgene);Acc:100289098]
ENSG00000119392	0,28290655	5,21776542	4,23289932	0,06893379	0,30777928	GLE1	GLE1 RNA export mediator [Source:HGNC Symbol;Acc:HGNC:4315]
ENSG00000129691	-0,2766033	6,05436364	4,23106003	0,06898415	0,30781596	ASH2L	ASH2 like, histone lysine methyltransferase complex subunit [Source:HGNC Symbol;Acc:HGNC:744]
ENSG00000274308	-0,9539604	-1,5512178	4,23114933	0,06898171	0,30781596	AC244093.1	ribosomal protein S2 (RPS2) pseudogene
ENSG00000181135	0,43802081	1,9486239	4,23008101	0,06901097	0,30784163	ZNF707	zinc finger protein 707 [Source:HGNC Symbol;Acc:HGNC:27815]
ENSG00000112419	0,38585505	3,1431764	4,22375525	0,0691846	0,30784813	PHACTR2	phosphatase and actin regulator 2 [Source:HGNC Symbol;Acc:HGNC:20956]
ENSG00000114790	0,40029885	2,24447252	4,22257833	0,06921697	0,30784813	ARHGEF26	Rho guanine nucleotide exchange factor 26 [Source:HGNC Symbol;Acc:HGNC:24490]
ENSG00000117335	0,36088259	7,65608704	4,22441125	0,06916657	0,30784813	CD46	CD46 molecule [Source:HGNC Symbol;Acc:HGNC:6953]
ENSG00000159714	1,23845702	-1,1038245	4,22824911	0,0690612	0,30784813	ZDHHC1	zinc finger DHHC-type containing 1 [Source:HGNC Symbol;Acc:HGNC:17916]
ENSG00000162522	0,34042654	4,61156283	4,22301386	0,06920499	0,30784813	KIAA1522	KIAA1522 [Source:HGNC Symbol;Acc:HGNC:29301]
ENSG00000171564	-0,552523	8,22578979	4,22519067	0,06914516	0,30784813	FGB	fibrinogen beta chain [Source:HGNC Symbol;Acc:HGNC:3662]
ENSG00000198841	0,33007763	3,31631198	4,22624506	0,0691162	0,30784813	KTI12	KTI12 chromatin associated homolog [Source:HGNC Symbol;Acc:HGNC:25160]
ENSG00000253945	-1,0266651	-1,6537664	4,22878488	0,06904651	0,30784813	AC024995.1	ribosomal protein L36a-like (RPL36AL) pseudogene
ENSG00000265778	-0,984013	-1,3579625	4,2223533	0,06922316	0,30784813	AC018413.1	novel transcript, antisense to ZFN156
ENSG00000271643	-0,7075693	0,06055694	4,22274742	0,06921232	0,30784813	AC112220.2	novel transcript
ENSG00000104408	0,27136849	8,58834029	4,21863505	0,06932554	0,30810586	EIF3E	eukaryotic translation initiation factor 3 subunit E [Source:HGNC Symbol;Acc:HGNC:3277]
ENSG00000163644	0,35171073	2,7464181	4,21746819	0,06935771	0,30810586	PPM1K	protein phosphatase, Mg2+/Mn2+ dependent 1K [Source:HGNC Symbol;Acc:HGNC:25415]
ENSG00000178691	0,32699381	3,78177232	4,21642231	0,06938656	0,30810586	SUZ12	SUZ12 polycomb repressive complex 2 subunit [Source:HGNC Symbol;Acc:HGNC:17101]
ENSG00000197860	-0,3159886	3,65409284	4,21653944	0,06938333	0,30810586	SGTB	small glutamine rich tetratricopeptide repeat containing beta [Source:HGNC Symbol;Acc:HGNC:23567]
ENSG00000285410	0,39718724	3,69367272	4,21756023	0,06935517	0,30810586	GABPB1-IT1	GABPB1 intronic transcript [Source:HGNC Symbol;Acc:HGNC:25469]
ENSG00000126903	-0,2960638	4,9738904	4,21274854	0,06948802	0,30836517	SLC10A3	solute carrier family 10 member 3 [Source:HGNC Symbol;Acc:HGNC:22979]
ENSG00000134490	-0,3821911	2,31016391	4,21337401	0,06947073	0,30836517	TMEM241	transmembrane protein 241 [Source:HGNC Symbol;Acc:HGNC:31723]
ENSG00000151445	-0,3108842	3,84741877	4,21125243	0,06952939	0,30836517	VIPAS39	VPS33B interacting protein, apical-basolateral polarity regulator, spe-39 homolog [Source:HGNC Symbol;Acc:HGN
ENSG00000167380	0,35391939	2,74925508	4,2116591	0,06951814	0,30836517	ZNF226	zinc finger protein 226 [Source:HGNC Symbol;Acc:HGNC:13019]
ENSG00000179750	-0,3514339	2,96808799	4,2097432	0,06957116	0,30845676	APOBEC3B	apolipoprotein B mRNA editing enzyme catalytic subunit 3B [Source:HGNC Symbol;Acc:HGNC:17352]
ENSG00000111181	-0,5228435	1,27690615	4,20591595	0,06967721	0,30862183	SLC6A12	solute carrier family 6 member 12 [Source:HGNC Symbol;Acc:HGNC:11045]
ENSG00000142444	0,31000724	4,27886125	4,20763555	0,06962954	0,30862183	TIMM29	translocase of inner mitochondrial membrane 29 [Source:HGNC Symbol;Acc:HGNC:25152]
ENSG00000153446	0,70567621	1,7287164	4,2053508	0,06969289	0,30862183	C16orf89	chromosome 16 open reading frame 89 [Source:HGNC Symbol;Acc:HGNC:28687]
ENSG00000175970	0,34595447	4,40596592	4,20603926	0,06967379	0,30862183	UNC119B	unc-119 lipid binding chaperone B [Source:HGNC Symbol;Acc:HGNC:16488]

ENSG00000171703	-0,4087036	3,84114774	4,20380087	0,06973591	0,30871875	TCEA2	transcription elongation factor A2 [Source:HGNC Symbol;Acc:HGNC:11614]
ENSG00000273004	-0,8569386	-0,7877284	4,20205113	0,06978451	0,30884034	AL078644.1	novel transcript
ENSG00000156239	0,39809557	3,22108438	4,19552618	0,06996614	0,30920259	N6AMT1	N-6 adenine-specific DNA methyltransferase 1 [Source:HGNC Symbol;Acc:HGNC:16021]
ENSG00000160221	-0,3826453	2,26346802	4,19531229	0,06997211	0,30920259	GATD3A	glutamine amidotransferase like class 1 domain containing 3A [Source:HGNC Symbol;Acc:HGNC:1273]
ENSG00000167508	-0,2849894	7,20079688	4,19764293	0,06990716	0,30920259	MVD	mevalonate diphosphate decarboxylase [Source:HGNC Symbol;Acc:HGNC:7529]
ENSG00000185189	-0,6849534	0,475655	4,19530917	0,06997219	0,30920259	NRBP2	nuclear receptor binding protein 2 [Source:HGNC Symbol;Acc:HGNC:19339]
ENSG00000270231	0,63696527	0,14079975	4,19663038	0,06993536	0,30920259	NBPF8	NBPF member 8 [Source:HGNC Symbol;Acc:HGNC:31990]
ENSG00000135119	0,36090359	2,40509491	4,19336932	0,07002631	0,30925465	RNFT2	ring finger protein, transmembrane 2 [Source:HGNC Symbol;Acc:HGNC:25905]
ENSG00000137251	1,05266601	-1,1233725	4,19377057	0,07001511	0,30925465	TINAG	tubulointerstitial nephritis antigen [Source:HGNC Symbol;Acc:HGNC:14599]
ENSG00000177600	-0,3136184	10,4288258	4,19144933	0,07007993	0,3093979	RPLP2	ribosomal protein lateral stalk subunit P2 [Source:HGNC Symbol;Acc:HGNC:10377]
ENSG00000101442	0,30422763	4,81446602	4,1904108	0,07010895	0,30943252	ACTR5	actin related protein 5 [Source:HGNC Symbol;Acc:HGNC:14671]
ENSG00000124702	0,34737761	7,04737802	4,18613651	0,07022856	0,3095724	KLHDC3	kelch domain containing 3 [Source:HGNC Symbol;Acc:HGNC:20704]
ENSG00000143256	-0,2858159	6,64596294	4,18774599	0,07018349	0,3095724	PFDN2	prefoldin subunit 2 [Source:HGNC Symbol;Acc:HGNC:8867]
ENSG00000187555	0,29034294	8,27245229	4,18549259	0,0702466	0,3095724	USP7	ubiquitin specific peptidase 7 [Source:HGNC Symbol;Acc:HGNC:12630]
ENSG00000233621	-1,271241	-1,3209275	4,1883609	0,07016628	0,3095724	LINC01137	long intergenic non-protein coding RNA 1137 [Source:HGNC Symbol;Acc:HGNC:49453]
ENSG00000258102	0,71311351	-0,4554651	4,18551818	0,07024588	0,3095724	MAP1LC3B2	microtubule associated protein 1 light chain 3 beta 2 [Source:HGNC Symbol;Acc:HGNC:34390]
ENSG00000112144	0,33930802	2,96867636	4,18217637	0,0703396	0,30977682	CILK1	ciliogenesis associated kinase 1 [Source:HGNC Symbol;Acc:HGNC:21219]
ENSG00000125089	-0,4570696	4,85210352	4,18157099	0,07035659	0,30977682	SH3TC1	SH3 domain and tetratricopeptide repeats 1 [Source:HGNC Symbol;Acc:HGNC:26009]
ENSG00000186806	0,35498761	2,9731587	4,1816812	0,0703535	0,30977682	VSIG10L	V-set and immunoglobulin domain containing 10 like [Source:HGNC Symbol;Acc:HGNC:27111]
ENSG00000164494	0,32362567	4,71456731	4,17909643	0,07042612	0,30989612	PDSS2	decaprenyl diphosphate synthase subunit 2 [Source:HGNC Symbol;Acc:HGNC:23041]
ENSG00000197774	-0,6380449	1,84421421	4,17949581	0,07041489	0,30989612	EME2	essential meiotic structure-specific endonuclease subunit 2 [Source:HGNC Symbol;Acc:HGNC:27289]
ENSG00000188186	-0,3877259	5,97440804	4,17773901	0,07046429	0,30997073	LAMTOR4	late endosomal/lysosomal adaptor, MAPK and MTOR activator 4 [Source:HGNC Symbol;Acc:HGNC:33772]
ENSG00000134107	-0,3771629	4,61584341	4,17603845	0,07051215	0,30999457	BHLHE40	basic helix-loop-helix family member e40 [Source:HGNC Symbol;Acc:HGNC:1046]
ENSG00000153363	-0,476931	1,80587522	4,17628349	0,07050525	0,30999457	LINC00467	long intergenic non-protein coding RNA 467 [Source:HGNC Symbol;Acc:HGNC:28227]
ENSG00000076003	0,30986071	5,69053355	4,17295334	0,07059907	0,31002175	MCM6	minichromosome maintenance complex component 6 [Source:HGNC Symbol;Acc:HGNC:6949]
ENSG00000139988	0,52327722	1,61958086	4,17360969	0,07058057	0,31002175	RDH12	retinol dehydrogenase 12 [Source:HGNC Symbol;Acc:HGNC:19977]
ENSG00000165434	0,64785102	-0,1733129	4,17402494	0,07056887	0,31002175	PGM2L1	phosphoglucomutase 2 like 1 [Source:HGNC Symbol;Acc:HGNC:20898]
ENSG00000220842	-0,4866083	0,82731786	4,17280654	0,07060321	0,31002175	RPL21P16	ribosomal protein L21 pseudogene 16 [Source:HGNC Symbol;Acc:HGNC:31396]
ENSG00000267056	1,69323677	-1,7131119	4,17079985	0,07065983	0,31017713	AC005336.1	inositol polyphosphate multikinase (IPMK) pseudogene
ENSG00000023041	0,41346077	5,28882259	4,16762489	0,07074953	0,31029117	ZDHHC6	zinc finger DHHC-type palmitoyltransferase 6 [Source:HGNC Symbol;Acc:HGNC:19160]
ENSG00000154133	-1,3482773	-0,9092763	4,16796197	0,07074	0,31029117	ROBO4	roundabout guidance receptor 4 [Source:HGNC Symbol;Acc:HGNC:17985]
ENSG00000167701	-0,6278651	1,234084	4,16911159	0,07070751	0,31029117	GPT	glutamic--pyruvic transaminase [Source:HGNC Symbol;Acc:HGNC:4552]
ENSG00000108666	0,33538831	4,42234631	4,1667242	0,070775	0,31030973	C17orf75	chromosome 17 open reading frame 75 [Source:HGNC Symbol;Acc:HGNC:30173]
ENSG00000087157	0,32797554	4,81547683	4,16570911	0,07080372	0,31034251	PGS1	phosphatidylglycerophosphate synthase 1 [Source:HGNC Symbol;Acc:HGNC:30029]
ENSG00000129083	0,35010165	7,22104222	4,15843157	0,07101006	0,31115357	COPB1	COPI coat complex subunit beta 1 [Source:HGNC Symbol;Acc:HGNC:2231]
ENSG00000101052	0,26574237	6,45214023	4,15741625	0,07103891	0,31118664	IFT52	intraflagellar transport 52 [Source:HGNC Symbol;Acc:HGNC:15901]
ENSG00000101972	0,30980685	4,19898388	4,15496891	0,07110851	0,31139813	STAG2	stromal antigen 2 [Source:HGNC Symbol;Acc:HGNC:11355]
ENSG00000105750	0,62110576	0,61113017	4,14605016	0,07136285	0,31150975	ZNF85	zinc finger protein 85 [Source:HGNC Symbol;Acc:HGNC:13160]
ENSG00000136783	-0,3392984	2,78004889	4,15074841	0,07122872	0,31150975	NIPSNAP3A	nipsnap homolog 3A [Source:HGNC Symbol;Acc:HGNC:23619]
ENSG00000151413	0,31574479	4,92544374	4,15121308	0,07121548	0,31150975	NUBPL	nucleotide binding protein like [Source:HGNC Symbol;Acc:HGNC:20278]
ENSG00000152601	0,36770583	2,59823098	4,14736921	0,07132516	0,31150975	MBNL1	muscleblind like splicing regulator 1 [Source:HGNC Symbol;Acc:HGNC:6923]
ENSG00000164828	0,39516552	5,21231996	4,14891569	0,07128101	0,31150975	SUN1	Sad1 and UNC84 domain containing 1 [Source:HGNC Symbol;Acc:HGNC:18587]
ENSG00000183576	-0,2734919	5,8500164	4,14850924	0,07129261	0,31150975	SETD3	SET domain containing 3, actin histidine methyltransferase [Source:HGNC Symbol;Acc:HGNC:20493]
ENSG00000196110	1,19993239	-1,0934488	4,14740101	0,07132425	0,31150975	ZNF699	zinc finger protein 699 [Source:HGNC Symbol;Acc:HGNC:24750]

ENSG00000219481	0,35320581	2,78387062	4,1480393	0,07130603	0,31150975	NBPF1	NBPF member 1 [Source:HGNC Symbol;Acc:HGNC:26088]
ENSG00000226564	-0,3525945	2,2626422	4,14610315	0,07136134	0,31150975	FTH1P20	ferritin heavy chain 1 pseudogene 20 [Source:HGNC Symbol;Acc:HGNC:37639]
ENSG00000256393	-0,5189515	0,79332989	4,14585086	0,07136855	0,31150975	RPL41P5	ribosomal protein L41 pseudogene 5 [Source:HGNC Symbol;Acc:HGNC:10355]
ENSG00000261609	0,49709566	2,23231107	4,14862812	0,07128922	0,31150975	GAN	gigaxonin [Source:HGNC Symbol;Acc:HGNC:4137]
ENSG00000174165	-0,3017645	4,23313784	4,14429323	0,07141309	0,31151802	ZDHHC24	zinc finger DHHC-type containing 24 [Source:HGNC Symbol;Acc:HGNC:27387]
ENSG00000188895	0,31494248	5,81051508	4,14499078	0,07139314	0,31151802	MSL1	MSL complex subunit 1 [Source:HGNC Symbol;Acc:HGNC:27905]
ENSG00000188488	0,3460377	9,53096557	4,14271979	0,07145812	0,31152841	SERPINA5	serpin family A member 5 [Source:HGNC Symbol;Acc:HGNC:8723]
ENSG00000216775	0,35575451	3,23883663	4,14340844	0,07143841	0,31152841	AL109918.1	heterogeneous nuclear ribonucleoprotein A/B (HNRNPAB) pseudogene
ENSG00000138449	0,40386966	3,17173788	4,14059258	0,07151906	0,31159628	SLC40A1	solute carrier family 40 member 1 [Source:HGNC Symbol;Acc:HGNC:10909]
ENSG00000174917	-0,2794834	5,80114867	4,14117552	0,07150235	0,31159628	MICOS13	mitochondrial contact site and cristae organizing system subunit 13 [Source:HGNC Symbol;Acc:HGNC:33702]
ENSG00000187699	-0,5151365	0,90114267	4,13994297	0,07153768	0,31159628	C2orf88	chromosome 2 open reading frame 88 [Source:HGNC Symbol;Acc:HGNC:28191]
ENSG00000159753	0,41342012	1,96608596	4,13771412	0,07160161	0,31178182	CARMIL2	capping protein regulator and myosin 1 linker 2 [Source:HGNC Symbol;Acc:HGNC:27089]
ENSG00000196167	-0,6135493	0,32362582	4,13674858	0,07162934	0,31180958	COLCA1	colorectal cancer associated 1 [Source:HGNC Symbol;Acc:HGNC:33789]
ENSG00000142655	0,30646766	5,46417842	4,13430526	0,07169954	0,31200247	PEX14	peroxisomal biogenesis factor 14 [Source:HGNC Symbol;Acc:HGNC:8856]
ENSG00000180525	-0,4439657	1,33542753	4,13336666	0,07172653	0,31200247	PRR26	proline rich 26 [Source:HGNC Symbol;Acc:HGNC:30724]
ENSG00000236423	-1,0192123	-0,9627055	4,13297796	0,07173772	0,31200247	LINC01134	long intergenic non-protein coding RNA 1134 [Source:HGNC Symbol;Acc:HGNC:49449]
ENSG00000173914	-0,3106205	4,05357538	4,13138217	0,07178365	0,31210932	RBM4B	RNA binding motif protein 4B [Source:HGNC Symbol;Acc:HGNC:28842]
ENSG00000090520	-0,2795909	8,12775317	4,12486973	0,07197148	0,31221501	DNAJB11	DnaJ heat shock protein family (Hsp40) member B11 [Source:HGNC Symbol;Acc:HGNC:14889]
ENSG00000100292	0,387539	7,04395147	4,12364142	0,07200697	0,31221501	HMOX1	heme oxygenase 1 [Source:HGNC Symbol;Acc:HGNC:5013]
ENSG00000106789	-0,2843745	5,85141542	4,12361443	0,07200775	0,31221501	CORO2A	coronin 2A [Source:HGNC Symbol;Acc:HGNC:2255]
ENSG00000111596	-0,3251792	5,19699081	4,1275932	0,07189285	0,31221501	CNOT2	CCR4-NOT transcription complex subunit 2 [Source:HGNC Symbol;Acc:HGNC:7878]
ENSG00000122376	0,40251564	4,7558575	4,12313295	0,07202167	0,31221501	SHLD2	shieldin complex subunit 2 [Source:HGNC Symbol;Acc:HGNC:28773]
ENSG00000132274	-1,2742463	-1,7670718	4,12360719	0,07200796	0,31221501	TRIM22	tripartite motif containing 22 [Source:HGNC Symbol;Acc:HGNC:16379]
ENSG00000137656	0,29321108	5,1541574	4,12821289	0,07187498	0,31221501	BUD13	BUD13 homolog [Source:HGNC Symbol;Acc:HGNC:28199]
ENSG00000153201	0,52101068	5,96727908	4,12671341	0,07191824	0,31221501	RANBP2	RAN binding protein 2 [Source:HGNC Symbol;Acc:HGNC:9848]
ENSG00000163602	0,34707566	3,34664322	4,12603787	0,07193774	0,31221501	RYBP	RING1 and YY1 binding protein [Source:HGNC Symbol;Acc:HGNC:10480]
ENSG00000228526	0,43294547	1,94287958	4,12321408	0,07201933	0,31221501	MIR34AHG	MIR34A host gene [Source:HGNC Symbol;Acc:HGNC:51913]
ENSG00000118007	0,32323171	3,91322526	4,12116716	0,07207854	0,31236884	STAG1	stromal antigen 1 [Source:HGNC Symbol;Acc:HGNC:11354]
ENSG00000070778	-0,4494968	1,63614204	4,11698462	0,07219972	0,31243061	PTPN21	protein tyrosine phosphatase non-receptor type 21 [Source:HGNC Symbol;Acc:HGNC:9651]
ENSG00000071794	0,41109653	3,16698929	4,11940069	0,07212969	0,31243061	HLTF	helicase like transcription factor [Source:HGNC Symbol;Acc:HGNC:11099]
ENSG00000087302	-0,2726732	7,1932911	4,11729687	0,07219067	0,31243061	RTRAF	RNA transcription, translation and transport factor [Source:HGNC Symbol;Acc:HGNC:23169]
ENSG00000131469	-0,2886973	10,6696833	4,11802209	0,07216964	0,31243061	RPL27	ribosomal protein L27 [Source:HGNC Symbol;Acc:HGNC:10328]
ENSG00000185875	0,40746273	1,72345676	4,11710447	0,07219625	0,31243061	THNSL1	threonine synthase like 1 [Source:HGNC Symbol;Acc:HGNC:26160]
ENSG00000162889	-0,3002263	5,52143013	4,11512263	0,07225375	0,31257183	MAPKAPK2	MAPK activated protein kinase 2 [Source:HGNC Symbol;Acc:HGNC:6887]
ENSG00000078668	0,28958306	7,97624583	4,11370561	0,0722949	0,31265727	VDAC3	voltage dependent anion channel 3 [Source:HGNC Symbol;Acc:HGNC:12674]
ENSG00000167077	-1,1013434	-0,717557	4,10957957	0,0724149	0,31308354	MEI1	meiotic double-stranded break formation protein 1 [Source:HGNC Symbol;Acc:HGNC:28613]
ENSG00000157796	0,39838094	3,27797611	4,10812512	0,07245726	0,31317399	WDR19	WD repeat domain 19 [Source:HGNC Symbol;Acc:HGNC:18340]
ENSG00000105607	-0,3408238	5,0939553	4,10525571	0,07254092	0,31344284	GCDH	glutaryl-CoA dehydrogenase [Source:HGNC Symbol;Acc:HGNC:4189]
ENSG00000163528	-0,3609788	4,40621955	4,10301528	0,07260632	0,31363269	CHCHD4	coiled-coil-helix-coiled-coil-helix domain containing 4 [Source:HGNC Symbol;Acc:HGNC:26467]
ENSG00000048028	-0,3459213	3,6877979	4,09982162	0,07269968	0,31376436	USP28	ubiquitin specific peptidase 28 [Source:HGNC Symbol;Acc:HGNC:12625]
ENSG00000070269	0,43530567	3,31777581	4,09976852	0,07270124	0,31376436	TMEM260	transmembrane protein 260 [Source:HGNC Symbol;Acc:HGNC:20185]
ENSG00000255394	1,17616102	-1,976829	4,10103259	0,07266426	0,31376436	C8orf49	chromosome 8 open reading frame 49 [Source:HGNC Symbol;Acc:HGNC:32200]
ENSG00000185155	-0,2919393	4,87007824	4,09764528	0,07276339	0,31384717	MIXL1	Mix paired-like homeobox [Source:HGNC Symbol;Acc:HGNC:13363]
ENSG00000198840	-0,2963367	7,2761857	4,09768145	0,07276233	0,31384717	MT-ND3	mitochondrially encoded NADH:ubiquinone oxidoreductase core subunit 3 [Source:HGNC Symbol;Acc:HGNC:7458

ENSG00000163960	0,55053104	4,01472302	4,09635183	0,07280129	0,31389991	UBXN7	UBX domain protein 7 [Source:HGNC Symbol;Acc:HGNC:29119]
ENSG00000185495	0,87312094	-0,3071865	4,09576147	0,07281859	0,31389991	AC138393.1	septin 7 (SEPT7) pseudogene
ENSG00000146094	0,92064153	-0,938671	4,09202017	0,07292838	0,31428043	DOK3	docking protein 3 [Source:HGNC Symbol;Acc:HGNC:24583]
ENSG00000106133	-0,3920443	1,91488326	4,09101727	0,07295784	0,31431469	NSUN5P2	NSUN5 pseudogene 2 [Source:HGNC Symbol;Acc:HGNC:16609]
ENSG00000118503	0,44656774	5,88761165	4,08787035	0,0730504	0,3143286	TNFAIP3	TNF alpha induced protein 3 [Source:HGNC Symbol;Acc:HGNC:11896]
ENSG00000138078	0,45935788	4,58083823	4,08772956	0,07305454	0,3143286	PREPL	prolyl endopeptidase like [Source:HGNC Symbol;Acc:HGNC:30228]
ENSG00000234004	-0,4517303	1,23615466	4,08740807	0,073064	0,3143286	AC092017.1	pseudogene similar to ribosomal protein S25 (RPS25)
ENSG00000253616	-0,8878906	-0,766844	4,08725025	0,07306865	0,3143286	AC107959.3	novel transcript
ENSG00000267656	1,05193027	-1,2269532	4,08909248	0,07301443	0,3143286	AP001357.2	mRNA turnover 4 homolog (S. cerevisiae) (MRTO4) pseudogene
ENSG00000253361	-0,7391282	-0,6880755	4,08522073	0,07312844	0,31449318	AC069120.1	novel transcript
ENSG00000115211	-0,3095856	6,66720162	4,08437724	0,0731533	0,31450754	EIF2B4	eukaryotic translation initiation factor 2B subunit delta [Source:HGNC Symbol;Acc:HGNC:3260]
ENSG00000250722	0,28730548	11,0729824	4,08360076	0,0731762	0,31451343	SELENOP	selenoprotein P [Source:HGNC Symbol;Acc:HGNC:10751]
ENSG00000163848	0,386995	2,56180036	4,08018586	0,07327702	0,31476155	ZNF148	zinc finger protein 148 [Source:HGNC Symbol;Acc:HGNC:12933]
ENSG00000169991	0,42286251	4,00081574	4,08025473	0,07327499	0,31476155	IFFO2	intermediate filament family orphan 2 [Source:HGNC Symbol;Acc:HGNC:27006]
ENSG00000113580	0,31761134	3,69917267	4,07704477	0,07336991	0,31497534	NR3C1	nuclear receptor subfamily 3 group C member 1 [Source:HGNC Symbol;Acc:HGNC:7978]
ENSG00000172955	-0,2907472	5,37689097	4,07726218	0,07336348	0,31497534	ADH6	alcohol dehydrogenase 6 (class V) [Source:HGNC Symbol;Acc:HGNC:255]
ENSG00000272146	-0,9597582	-1,1639169	4,07515901	0,07342575	0,31512245	ARF4-AS1	ARF4 antisense RNA 1 [Source:HGNC Symbol;Acc:HGNC:51593]
ENSG00000287080	-0,6579532	0,30632551	4,07408384	0,07345761	0,31516659	H3C3	H3 clustered histone 3 [Source:HGNC Symbol;Acc:HGNC:4768]
ENSG00000099822	1,32643976	-1,561714	4,07269309	0,07349885	0,31525094	HCN2	hyperpolarization activated cyclic nucleotide gated potassium and sodium channel 2 [Source:HGNC Symbol;Acc:H
ENSG00000104450	0,32689054	2,68904764	4,07157756	0,07353195	0,31530033	SPAG1	sperm associated antigen 1 [Source:HGNC Symbol;Acc:HGNC:11212]
ENSG00000117528	0,30925183	6,76345347	4,06664837	0,07367842	0,31583569	ABCD3	ATP binding cassette subfamily D member 3 [Source:HGNC Symbol;Acc:HGNC:67]
ENSG00000213799	0,38748928	2,52501675	4,06560367	0,07370951	0,31587628	ZNF845	zinc finger protein 845 [Source:HGNC Symbol;Acc:HGNC:25112]
ENSG00000123472	0,26468289	6,14222582	4,06355753	0,07377045	0,31604474	ATPAF1	ATP synthase mitochondrial F1 complex assembly factor 1 [Source:HGNC Symbol;Acc:HGNC:18803]
ENSG00000026950	0,42780999	2,67985006	4,06018293	0,0738711	0,31629105	BTN3A1	butyrophilin subfamily 3 member A1 [Source:HGNC Symbol;Acc:HGNC:1138]
ENSG00000156052	0,34511389	2,52280383	4,06017799	0,07387125	0,31629105	GNAQ	G protein subunit alpha q [Source:HGNC Symbol;Acc:HGNC:4390]
ENSG00000165195	0,33081939	2,5390426	4,05807878	0,07393395	0,31646675	PIGA	phosphatidylinositol glycan anchor biosynthesis class A [Source:HGNC Symbol;Acc:HGNC:8957]
ENSG00000223547	0,64198029	0,51046292	4,05718491	0,07396066	0,31648838	ZNF844	zinc finger protein 844 [Source:HGNC Symbol;Acc:HGNC:25932]
ENSG00000143569	0,47970111	6,81954601	4,05641601	0,07398365	0,31649406	UBAP2L	ubiquitin associated protein 2 like [Source:HGNC Symbol;Acc:HGNC:29877]
ENSG00000068079	-0,4176072	5,32116352	4,04920619	0,07419968	0,31732527	IFI35	interferon induced protein 35 [Source:HGNC Symbol;Acc:HGNC:5399]
ENSG00000197771	0,29624431	6,08733246	4,0477243	0,07424418	0,31742266	MCMBP	minichromosome maintenance complex binding protein [Source:HGNC Symbol;Acc:HGNC:25782]
ENSG00000133943	0,29614675	4,06257827	4,04117446	0,07444127	0,31771012	DGLUCY	D-glutamate cyclase [Source:HGNC Symbol;Acc:HGNC:20498]
ENSG00000146477	0,31508204	5,45587226	4,04240914	0,07440407	0,31771012	SLC22A3	solute carrier family 22 member 3 [Source:HGNC Symbol;Acc:HGNC:10967]
ENSG00000173890	0,42971656	2,416037	4,04115341	0,0744419	0,31771012	GPR160	G protein-coupled receptor 160 [Source:HGNC Symbol;Acc:HGNC:23693]
ENSG00000176225	0,43457577	2,47283584	4,04256706	0,07439931	0,31771012	RTTN	rotatin [Source:HGNC Symbol;Acc:HGNC:18654]
ENSG00000219607	-0,6431774	-0,2031693	4,04250665	0,07440113	0,31771012	PPP1R3G	protein phosphatase 1 regulatory subunit 3G [Source:HGNC Symbol;Acc:HGNC:14945]
ENSG00000279602	1,17841821	-1,3165956	4,04464508	0,07433675	0,31771012	AC109326.1	TEC
ENSG00000234718	-0,5539078	0,31695661	4,03956858	0,07448969	0,31782123	AC007161.1	ribosomal protein L23a (RPL23A) pseudogene
ENSG00000143742	-0,3274612	6,15067027	4,03770624	0,07454589	0,31796817	SRP9	signal recognition particle 9 [Source:HGNC Symbol;Acc:HGNC:11304]
ENSG00000179104	-0,7963182	-0,2453796	4,0364565	0,07458364	0,31803632	TMTC2	transmembrane O-mannosyltransferase targeting cadherins 2 [Source:HGNC Symbol;Acc:HGNC:25440]
ENSG00000186638	0,42643516	1,49876054	4,03425299	0,07465025	0,31822748	KIF24	kinesin family member 24 [Source:HGNC Symbol;Acc:HGNC:19916]
ENSG00000168300	0,53207063	1,8369645	4,03165451	0,0747289	0,31837916	PCMTD1	protein-L-isoaspartate (D-aspartate) O-methyltransferase domain containing 1 [Source:HGNC Symbol;Acc:HGNC:3
ENSG00000256092	-0,8651827	-1,0009057	4,03163737	0,07472942	0,31837916	SBNO1-AS1	SBNO1 antisense RNA 1 [Source:HGNC Symbol;Acc:HGNC:54355]
ENSG00000139354	0,67354474	0,71878936	4,03011293	0,07477561	0,31848306	GAS2L3	growth arrest specific 2 like 3 [Source:HGNC Symbol;Acc:HGNC:27475]
ENSG00000095951	0,51873208	2,04989711	4,02613046	0,07489645	0,31848958	HIVEP1	HIVEP zinc finger 1 [Source:HGNC Symbol;Acc:HGNC:4920]

ENSG00000151689	-0,2767343	5,58670701	4,02643398	0,07488723	0,31848958	INPP1	inositol polyphosphate-1-phosphatase [Source:HGNC Symbol;Acc:HGNC:6071]
ENSG00000152952	-0,3565877	6,28542626	4,02855112	0,07482297	0,31848958	PLOD2	procollagen-lysine,2-oxoglutarate 5-dioxygenase 2 [Source:HGNC Symbol;Acc:HGNC:9082]
ENSG00000166888	-0,2959733	5,01616205	4,02566019	0,07491073	0,31848958	STAT6	signal transducer and activator of transcription 6 [Source:HGNC Symbol;Acc:HGNC:11368]
ENSG00000183688	-0,3313339	3,48963644	4,02488401	0,07493432	0,31848958	RFLNB	refilin B [Source:HGNC Symbol;Acc:HGNC:28705]
ENSG00000225392	-1,0074128	-1,6252285	4,02389232	0,07496446	0,31848958	MRPL36P1	mitochondrial ribosomal protein L36 pseudogene 1 [Source:HGNC Symbol;Acc:HGNC:29708]
ENSG00000228649	-0,5712414	0,90162906	4,0236001	0,07497335	0,31848958	SNHG26	small nucleolar RNA host gene 26 [Source:HGNC Symbol;Acc:HGNC:53131]
ENSG00000241343	0,80231194	-0,7750166	4,0265748	0,07488295	0,31848958	RPL36A	ribosomal protein L36a [Source:HGNC Symbol;Acc:HGNC:10359]
ENSG00000277283	-0,4428115	1,57570422	4,02629466	0,07489146	0,31848958	AC004812.2	novel transcript, antisense to RAB35
ENSG00000144935	-0,4394029	1,2248228	4,02184066	0,07502688	0,31862434	TRPC1	transient receptor potential cation channel subfamily C member 1 [Source:HGNC Symbol;Acc:HGNC:12333]
ENSG00000100439	-0,3252229	5,08642014	4,02048409	0,07506819	0,31870712	ABHD4	abhydrolase domain containing 4, N-acyl phospholipase B [Source:HGNC Symbol;Acc:HGNC:20154]
ENSG00000213553	-0,2817988	5,14733412	4,01760643	0,07515591	0,31898683	RPLP0P6	ribosomal protein lateral stalk subunit P0 pseudogene 6 [Source:HGNC Symbol;Acc:HGNC:36404]
ENSG00000073008	-0,2932306	7,02072988	4,01453665	0,07524963	0,31910647	PVR	PVR cell adhesion molecule [Source:HGNC Symbol;Acc:HGNC:9705]
ENSG00000108106	-0,3554445	5,0749483	4,01470957	0,07524434	0,31910647	UBE2S	ubiquitin conjugating enzyme E2 S [Source:HGNC Symbol;Acc:HGNC:17895]
ENSG00000145014	-0,35193	3,29467628	4,01504927	0,07523397	0,31910647	TMEM44	transmembrane protein 44 [Source:HGNC Symbol;Acc:HGNC:25120]
ENSG00000105784	0,8934987	-0,5406126	4,01242073	0,07531431	0,3192337	RUNDC3B	RUN domain containing 3B [Source:HGNC Symbol;Acc:HGNC:30286]
ENSG00000178301	0,37171128	4,69935352	4,01105597	0,07535607	0,3192337	AQP11	aquaporin 11 [Source:HGNC Symbol;Acc:HGNC:19940]
ENSG00000236753	-0,5493847	0,51579783	4,01203665	0,07532606	0,3192337	MKLN1-AS	MKLN1 antisense RNA [Source:HGNC Symbol;Acc:HGNC:40374]
ENSG00000255737	0,37238886	2,08626059	4,01069765	0,07536704	0,3192337	AGAP2-AS1	AGAP2 antisense RNA 1 [Source:HGNC Symbol;Acc:HGNC:48633]
ENSG00000106927	0,32216049	11,968922	4,00894979	0,07542057	0,31936784	AMBP	alpha-1-microglobulin/bikunin precursor [Source:HGNC Symbol;Acc:HGNC:453]
ENSG00000115687	0,32600974	4,07982743	4,00750015	0,075465	0,31944018	PASK	PAS domain containing serine/threonine kinase [Source:HGNC Symbol;Acc:HGNC:17270]
ENSG00000273604	-0,269554	7,93099281	4,00696601	0,07548138	0,31944018	EPOP	elongin BC and polycomb repressive complex 2 associated protein [Source:HGNC Symbol;Acc:HGNC:34493]
ENSG00000186625	0,32682873	4,57127494	4,00610441	0,07550781	0,3194595	KATNA1	katanin catalytic subunit A1 [Source:HGNC Symbol;Acc:HGNC:6216]
ENSG00000139055	-0,5205951	2,48843848	4,00365705	0,07558296	0,31968484	ERP27	endoplasmic reticulum protein 27 [Source:HGNC Symbol;Acc:HGNC:26495]
ENSG00000175782	0,327721	4,77886265	4,00253524	0,07561743	0,31969378	SLC35E3	solute carrier family 35 member E3 [Source:HGNC Symbol;Acc:HGNC:20864]
ENSG00000237350	-0,7031105	-0,4404756	4,00216429	0,07562884	0,31969378	CDC42P6	cell division cycle 42 pseudogene 6 [Source:HGNC Symbol;Acc:HGNC:44430]
ENSG00000136108	0,2806633	5,1621578	3,99900301	0,07572612	0,32001239	CKAP2	cytoskeleton associated protein 2 [Source:HGNC Symbol;Acc:HGNC:1990]
ENSG00000103365	0,41383956	6,12010586	3,99817519	0,07575162	0,32001989	GGA2	golgi associated, gamma adaptin ear containing, ARF binding protein 2 [Source:HGNC Symbol;Acc:HGNC:16064]
ENSG00000106948	-0,3682161	2,32833557	3,99610235	0,07581551	0,32001989	AKNA	AT-hook transcription factor [Source:HGNC Symbol;Acc:HGNC:24108]
ENSG00000114268	-0,3798141	3,97099039	3,99622131	0,07581185	0,32001989	PFKFB4	6-phosphofructo-2-kinase/fructose-2,6-biphosphatase 4 [Source:HGNC Symbol;Acc:HGNC:8875]
ENSG00000157077	0,33029667	4,22271026	3,99630447	0,07580928	0,32001989	ZFYVE9	zinc finger FYVE-type containing 9 [Source:HGNC Symbol;Acc:HGNC:6775]
ENSG00000162231	0,31254919	5,91961219	3,99529233	0,0758405	0,3200329	NXF1	nuclear RNA export factor 1 [Source:HGNC Symbol;Acc:HGNC:8071]
ENSG00000170881	0,42968032	4,83566696	3,99437988	0,07586866	0,32005928	RNF139	ring finger protein 139 [Source:HGNC Symbol;Acc:HGNC:17023]
ENSG00000140948	0,37958142	3,82071995	3,9924185	0,07592924	0,32022237	ZCCHC14	zinc finger CCHC-type containing 14 [Source:HGNC Symbol;Acc:HGNC:24134]
ENSG00000213719	-0,3235908	10,3496516	3,99169075	0,07595173	0,32022478	CLIC1	chloride intracellular channel 1 [Source:HGNC Symbol;Acc:HGNC:2062]
ENSG00000153922	0,46717726	4,55073849	3,98914362	0,07603052	0,32046448	CHD1	chromodomain helicase DNA binding protein 1 [Source:HGNC Symbol;Acc:HGNC:1915]
ENSG00000136051	0,36121069	3,4857985	3,98755946	0,07607958	0,32057875	WASHC4	WASH complex subunit 4 [Source:HGNC Symbol;Acc:HGNC:29174]
ENSG00000005961	-1,4949879	-1,8234925	3,98434723	0,07617917	0,32081333	ITGA2B	integrin subunit alpha 2b [Source:HGNC Symbol;Acc:HGNC:6138]
ENSG00000083535	-0,3685767	2,31756258	3,98454597	0,076173	0,32081333	PIBF1	progesterone immunomodulatory binding factor 1 [Source:HGNC Symbol;Acc:HGNC:23352]
ENSG00000160991	-0,421572	2,46763183	3,98081484	0,07628887	0,32118274	ORAI2	ORAI calcium release-activated calcium modulator 2 [Source:HGNC Symbol;Acc:HGNC:21667]
ENSG00000080709	1,0508137	-1,1416631	3,97866995	0,07635558	0,32118592	KCNN2	potassium calcium-activated channel subfamily N member 2 [Source:HGNC Symbol;Acc:HGNC:6291]
ENSG00000121057	0,31274496	7,7175141	3,97945646	0,07633111	0,32118592	AKAP1	A-kinase anchoring protein 1 [Source:HGNC Symbol;Acc:HGNC:367]
ENSG00000135318	0,37106604	4,30439865	3,97912726	0,07634135	0,32118592	NT5E	5'-nucleotidase ecto [Source:HGNC Symbol;Acc:HGNC:8021]
ENSG00000100196	-0,3887329	5,12858109	3,97761376	0,07638846	0,32123172	KDELR3	KDEL endoplasmic reticulum protein retention receptor 3 [Source:HGNC Symbol;Acc:HGNC:6306]
ENSG00000219891	0,8051105	-0,7189381	3,97678258	0,07641434	0,3212481	ZSCAN12P1	zinc finger and SCAN domain containing 12 pseudogene 1 [Source:HGNC Symbol;Acc:HGNC:13850]

ENSG00000134049	0,41391017	4,32291677	3,97605922	0,07643688	0,3212504	IER3IP1	immediate early response 3 interacting protein 1 [Source:HGNC Symbol;Acc:HGNC:18550]
ENSG00000135931	0,34483414	3,06002731	3,97395316	0,07650254	0,32143389	ARMC9	armadillo repeat containing 9 [Source:HGNC Symbol;Acc:HGNC:20730]
ENSG00000069329	0,36471376	6,45379218	3,97031962	0,076616	0,32181802	VPS35	VPS35 retromer complex component [Source:HGNC Symbol;Acc:HGNC:13487]
ENSG00000162267	0,44499239	6,7131462	3,96484776	0,07678725	0,32244464	ITIH3	inter-alpha-trypsin inhibitor heavy chain 3 [Source:HGNC Symbol;Acc:HGNC:6168]
ENSG00000073578	0,38531269	4,80692323	3,96359791	0,07682643	0,32251648	SDHA	succinate dehydrogenase complex flavoprotein subunit A [Source:HGNC Symbol;Acc:HGNC:10680]
ENSG00000224152	0,87961647	-1,3456653	3,96159808	0,07688918	0,32268717	AC009506.1	novel transcript, antisense to BAZ2B
ENSG00000186448	0,35032423	2,37536141	3,96062642	0,07691969	0,32272251	ZNF197	zinc finger protein 197 [Source:HGNC Symbol;Acc:HGNC:12988]
ENSG00000163001	0,37412938	5,28078984	3,95961159	0,07695158	0,32276359	CFAP36	cilia and flagella associated protein 36 [Source:HGNC Symbol;Acc:HGNC:30540]
ENSG00000020922	0,32478609	4,31308453	3,9505078	0,07723834	0,32361997	MRE11	MRE11 homolog, double strand break repair nuclease [Source:HGNC Symbol;Acc:HGNC:7230]
ENSG00000095002	0,31844267	5,83429623	3,95008677	0,07725163	0,32361997	MSH2	mutS homolog 2 [Source:HGNC Symbol;Acc:HGNC:7325]
ENSG00000100300	-0,8537762	-0,679029	3,95178385	0,07719806	0,32361997	TSPO	translocator protein [Source:HGNC Symbol;Acc:HGNC:1158]
ENSG00000137770	-0,3603036	3,08429287	3,95106895	0,07722062	0,32361997	CTDSPL2	CTD small phosphatase like 2 [Source:HGNC Symbol;Acc:HGNC:26936]
ENSG00000198498	-0,3915477	4,61511827	3,94961569	0,07726651	0,32361997	TMA16	translation machinery associated 16 homolog [Source:HGNC Symbol;Acc:HGNC:25638]
ENSG00000102871	-0,3589343	2,91486499	3,94598031	0,07738145	0,32385726	TRADD	TNFRSF1A associated via death domain [Source:HGNC Symbol;Acc:HGNC:12030]
ENSG00000130299	-0,3134168	4,86859976	3,94700135	0,07734915	0,32385726	GTPBP3	GTP binding protein 3, mitochondrial [Source:HGNC Symbol;Acc:HGNC:14880]
ENSG00000136628	-0,4680794	6,32030898	3,94362054	0,07745618	0,32385726	EPRS1	glutamyl-prolyl-tRNA synthetase 1 [Source:HGNC Symbol;Acc:HGNC:3418]
ENSG00000173928	-0,3196883	2,80545501	3,94382697	0,07744964	0,32385726	SWSAP1	SWIM-type zinc finger 7 associated protein 1 [Source:HGNC Symbol;Acc:HGNC:26638]
ENSG00000186577	-0,3635536	3,26320759	3,94530701	0,07740276	0,32385726	SMIM29	small integral membrane protein 29 [Source:HGNC Symbol;Acc:HGNC:1340]
ENSG00000235297	-0,5728954	0,61900888	3,94437341	0,07743233	0,32385726	FAUP1	FAU pseudogene 1 [Source:HGNC Symbol;Acc:HGNC:17984]
ENSG00000136100	0,32635607	4,67704942	3,94155137	0,07752177	0,32403879	VPS36	vacuolar protein sorting 36 homolog [Source:HGNC Symbol;Acc:HGNC:20312]
ENSG00000172113	-0,2782183	5,1497057	3,94059037	0,07755226	0,32407351	NME6	NME/NM23 nucleoside diphosphate kinase 6 [Source:HGNC Symbol;Acc:HGNC:20567]
ENSG00000143624	0,28980259	5,21625905	3,93858614	0,0776159	0,32415399	INTS3	integrator complex subunit 3 [Source:HGNC Symbol;Acc:HGNC:26153]
ENSG00000185608	-0,2608106	6,7945641	3,93923487	0,07759529	0,32415399	MRPL40	mitochondrial ribosomal protein L40 [Source:HGNC Symbol;Acc:HGNC:14491]
ENSG00000269893	-0,3781978	5,20426485	3,93623429	0,07769066	0,32437348	SNHG8	small nucleolar RNA host gene 8 [Source:HGNC Symbol;Acc:HGNC:33098]
ENSG00000017260	0,35112291	5,74256795	3,93434201	0,07775087	0,32443944	ATP2C1	ATPase secretory pathway Ca2+ transporting 1 [Source:HGNC Symbol;Acc:HGNC:13211]
ENSG00000233741	-0,4922879	1,01110404	3,93437375	0,07774986	0,32443944	RPL27P7	ribosomal protein L27 pseudogene 7 [Source:HGNC Symbol;Acc:HGNC:36107]
ENSG00000008838	0,43644203	6,04993824	3,9320522	0,07782382	0,32458992	MED24	mediator complex subunit 24 [Source:HGNC Symbol;Acc:HGNC:22963]
ENSG00000236281	-0,6030474	0,41760953	3,9318152	0,07783137	0,32458992	NDUFB9P2	NADH:ubiquinone oxidoreductase subunit B9 pseudogene 2 [Source:HGNC Symbol;Acc:HGNC:52270]
ENSG00000182957	0,43836464	2,35569222	3,93016534	0,07788399	0,32471666	SPATA13	spermatogenesis associated 13 [Source:HGNC Symbol;Acc:HGNC:23222]
ENSG00000170456	0,30081097	4,04902479	3,92743264	0,07797124	0,32491461	DENND5B	DENN domain containing 5B [Source:HGNC Symbol;Acc:HGNC:28338]
ENSG00000247271	-0,6993064	-0,2121777	3,92728524	0,07797595	0,32491461	ZBED5-AS1	ZBED5 antisense RNA 1 [Source:HGNC Symbol;Acc:HGNC:48646]
ENSG00000213856	0,80641971	-0,8218157	3,92486555	0,07805331	0,32514424	VDAC1P2	voltage dependent anion channel 1 pseudogene 2 [Source:HGNC Symbol;Acc:HGNC:12670]
ENSG00000105617	-0,3199428	3,22755909	3,92344422	0,0780988	0,325241	LENG1	leukocyte receptor cluster member 1 [Source:HGNC Symbol;Acc:HGNC:15502]
ENSG00000160282	-0,3237227	5,41420936	3,9219682	0,07814608	0,32534513	FTCD	formimidoyltransferase cyclodeaminase [Source:HGNC Symbol;Acc:HGNC:3974]
ENSG00000272631	0,94485508	-1,5337951	3,92008202	0,07820654	0,3255041	AC067750.1	novel transcript, antisense to BMPR1A
ENSG00000255135	-0,4838779	1,26215053	3,91895068	0,07824284	0,32556241	AP002360.1	novel transcript
ENSG00000106701	0,42843208	1,4936042	3,91712163	0,07830156	0,32571399	FSD1L	fibronectin type III and SPRY domain containing 1 like [Source:HGNC Symbol;Acc:HGNC:13753]
ENSG00000119899	0,35839718	5,20159345	3,91437271	0,07838993	0,32594172	SLC17A5	solute carrier family 17 member 5 [Source:HGNC Symbol;Acc:HGNC:10933]
ENSG00000197020	0,51323522	0,81668904	3,91334254	0,07842307	0,32594172	ZNF100	zinc finger protein 100 [Source:HGNC Symbol;Acc:HGNC:12880]
ENSG00000198346	0,36953165	1,97415624	3,91328544	0,07842491	0,32594172	ZNF813	zinc finger protein 813 [Source:HGNC Symbol;Acc:HGNC:33257]
ENSG00000229644	0,75163031	-0,4721421	3,91264414	0,07844555	0,32594172	NAMPTP1	nicotinamide phosphoribosyltransferase pseudogene 1 [Source:HGNC Symbol;Acc:HGNC:17633]
ENSG00000127184	-0,3669971	7,76724833	3,9116528	0,07847748	0,32598166	COX7C	cytochrome c oxidase subunit 7C [Source:HGNC Symbol;Acc:HGNC:2292]
ENSG00000006695	0,37524242	3,76070784	3,90887906	0,07856689	0,32611988	COX10	cytochrome c oxidase assembly factor heme A:farnesyltransferase COX10 [Source:HGNC Symbol;Acc:HGNC:2260]
ENSG00000165304	-0,301081	5,35782295	3,90785151	0,07860005	0,32611988	MELK	maternal embryonic leucine zipper kinase [Source:HGNC Symbol;Acc:HGNC:16870]

ENSG00000185049	0,36396371	5,47567724	3,90841353	0,07858191	0,32611988	NELFA	negative elongation factor complex member A [Source:HGNC Symbol;Acc:HGNC:12768]
ENSG00000262294	0,85788192	-0,9360628	3,90971417	0,07853996	0,32611988	AC129507.2	novel transcript, antisense to RPH3AL
ENSG00000110218	0,33672576	4,64537687	3,90324318	0,07874896	0,32664498	PANX1	pannexin 1 [Source:HGNC Symbol;Acc:HGNC:8599]
ENSG00000136943	0,31549305	4,67205152	3,89957029	0,07886791	0,32696043	CTSV	cathepsin V [Source:HGNC Symbol;Acc:HGNC:2538]
ENSG00000138119	-1,1314241	-0,4820192	3,89951257	0,07886978	0,32696043	MYOF	myoferlin [Source:HGNC Symbol;Acc:HGNC:3656]
ENSG00000102710	0,29883955	4,74646342	3,89856078	0,07890064	0,32699557	SUPT20H	SPT20 homolog, SAGA complex component [Source:HGNC Symbol;Acc:HGNC:20596]
ENSG00000102218	0,38868975	2,95260676	3,89530471	0,07900632	0,32724502	RP2	RP2 activator of ARL3 GTPase [Source:HGNC Symbol;Acc:HGNC:10274]
ENSG00000169359	-0,3293983	3,2192891	3,89521136	0,07900936	0,32724502	SLC33A1	solute carrier family 33 member 1 [Source:HGNC Symbol;Acc:HGNC:95]
ENSG00000173662	0,5719791	1,9641324	3,89463681	0,07902803	0,32724502	TAS1R1	taste 1 receptor member 1 [Source:HGNC Symbol;Acc:HGNC:14448]
ENSG00000160606	-0,2887986	4,11421317	3,89351262	0,07906457	0,32730357	TLCD1	TLC domain containing 1 [Source:HGNC Symbol;Acc:HGNC:25177]
ENSG00000082438	0,32859462	5,29891062	3,89097035	0,07914729	0,32755319	COBLL1	cordon-bleu WH2 repeat protein like 1 [Source:HGNC Symbol;Acc:HGNC:23571]
ENSG00000186222	-0,322173	4,42051753	3,88934165	0,07920034	0,32767992	BLOC1S4	biogenesis of lysosomal organelles complex 1 subunit 4 [Source:HGNC Symbol;Acc:HGNC:24206]
ENSG00000127415	-0,9738263	-1,4773753	3,88703318	0,07927561	0,32782008	IDUA	alpha-L-iduronidase [Source:HGNC Symbol;Acc:HGNC:5391]
ENSG00000186567	-0,9422215	-1,0441968	3,88692633	0,0792791	0,32782008	CEACAM19	CEA cell adhesion molecule 19 [Source:HGNC Symbol;Acc:HGNC:31951]
ENSG00000180096	-0,5814634	0,06554437	3,88609435	0,07930625	0,32783956	SEPTIN1	septin 1 [Source:HGNC Symbol;Acc:HGNC:2879]
ENSG00000065989	-0,3327533	2,43568885	3,88361717	0,07938716	0,32791794	PDE4A	phosphodiesterase 4A [Source:HGNC Symbol;Acc:HGNC:8780]
ENSG00000108423	0,45781237	3,8498255	3,8794322	0,07952409	0,32791794	TUBD1	tubulin delta 1 [Source:HGNC Symbol;Acc:HGNC:16811]
ENSG00000109205	-0,5617297	4,57893758	3,88058768	0,07948625	0,32791794	ODAM	odontogenic, ameloblast associated [Source:HGNC Symbol;Acc:HGNC:26043]
ENSG00000145604	0,36471104	6,00090487	3,88300431	0,0794072	0,32791794	SKP2	S-phase kinase associated protein 2 [Source:HGNC Symbol;Acc:HGNC:10901]
ENSG00000157985	0,30603042	4,88958239	3,88156176	0,07945438	0,32791794	AGAP1	ArfGAP with GTPase domain, ankyrin repeat and PH domain 1 [Source:HGNC Symbol;Acc:HGNC:16922]
ENSG00000174233	0,36111581	4,33349272	3,88242821	0,07942603	0,32791794	ADCY6	adenylate cyclase 6 [Source:HGNC Symbol;Acc:HGNC:237]
ENSG00000178401	-0,3332766	5,09825097	3,88064086	0,07948451	0,32791794	DNAJC22	DnaJ heat shock protein family (Hsp40) member C22 [Source:HGNC Symbol;Acc:HGNC:25802]
ENSG00000180423	-0,3512144	1,94531232	3,88426377	0,07936603	0,32791794	HARBI1	harbinger transposase derived 1 [Source:HGNC Symbol;Acc:HGNC:26522]
ENSG00000204860	0,50314492	1,15155299	3,87933252	0,07952736	0,32791794	FAM201A	family with sequence similarity 201 member A [Source:HGNC Symbol;Acc:HGNC:27239]
ENSG00000231806	0,43536512	1,53020167	3,87865132	0,07954967	0,32791794	PCAT7	prostate cancer associated transcript 7 [Source:HGNC Symbol;Acc:HGNC:48824]
ENSG00000220267	-1,1863252	-1,5039436	3,8743393	0,07969113	0,32840838	ACTBP8	ACTB pseudogene 8 [Source:HGNC Symbol;Acc:HGNC:141]
ENSG00000165502	-0,3211545	7,82246245	3,87330808	0,079725	0,32845533	RPL36AL	ribosomal protein L36a like [Source:HGNC Symbol;Acc:HGNC:10346]
ENSG00000126231	-0,368941	2,29573099	3,87232433	0,07975734	0,3284959	PROZ	protein Z, vitamin K dependent plasma glycoprotein [Source:HGNC Symbol;Acc:HGNC:9460]
ENSG00000167107	0,3227259	5,81538195	3,87072594	0,07980991	0,3285293	ACSF2	acyl-CoA synthetase family member 2 [Source:HGNC Symbol;Acc:HGNC:26101]
ENSG00000184677	0,41471663	3,74336021	3,87002697	0,07983291	0,3285293	ZBTB40	zinc finger and BTB domain containing 40 [Source:HGNC Symbol;Acc:HGNC:29045]
ENSG00000227615	-0,3482238	3,5497082	3,87052392	0,07981656	0,3285293	AP001324.1	ribosomal protein S12 (RPS12) pseudogene
ENSG00000117410	0,27218657	8,32927679	3,86848461	0,0798837	0,32864572	ATP6V0B	ATPase H+ transporting V0 subunit b [Source:HGNC Symbol;Acc:HGNC:861]
ENSG00000127423	0,28773334	4,30390361	3,8665219	0,07994839	0,32866256	AUNIP	aurora kinase A and ninein interacting protein [Source:HGNC Symbol;Acc:HGNC:28363]
ENSG00000176533	-0,3290706	2,79188664	3,86631276	0,07995528	0,32866256	GNG7	G protein subunit gamma 7 [Source:HGNC Symbol;Acc:HGNC:4410]
ENSG00000275454	-1,2992557	-1,7761702	3,86641428	0,07995194	0,32866256	AC105020.6	novel transcript, antisense to IMP3
ENSG00000144045	-0,3919598	4,60465773	3,86522654	0,07999112	0,32871736	DQX1	DEAQ-box RNA dependent ATPase 1 [Source:HGNC Symbol;Acc:HGNC:20410]
ENSG00000174574	0,29431656	7,15768941	3,86077914	0,08013804	0,32913596	AKIRIN1	akirin 1 [Source:HGNC Symbol;Acc:HGNC:25744]
ENSG00000188917	0,36578793	2,95506836	3,86115001	0,08012577	0,32913596	TRMT2B	tRNA methyltransferase 2 homolog B [Source:HGNC Symbol;Acc:HGNC:25748]
ENSG00000126522	-0,3140635	5,61515466	3,85819394	0,0802236	0,32930221	ASL	argininosuccinate lyase [Source:HGNC Symbol;Acc:HGNC:746]
ENSG00000228278	-0,2628206	9,1898629	3,85885736	0,08020163	0,32930221	ORM2	orosomucoid 2 [Source:HGNC Symbol;Acc:HGNC:8499]
ENSG00000237506	-0,3400458	2,21111502	3,8573803	0,08025055	0,32932031	RPSAP15	ribosomal protein SA pseudogene 15 [Source:HGNC Symbol;Acc:HGNC:31464]
ENSG00000104884	-0,3739833	5,46468928	3,8546347	0,08034159	0,32941135	ERCC2	ERCC excision repair 2, TFIIH core complex helicase subunit [Source:HGNC Symbol;Acc:HGNC:3434]
ENSG00000110925	0,31556135	3,07188316	3,854724	0,08033862	0,32941135	CSRNP2	cysteine and serine rich nuclear protein 2 [Source:HGNC Symbol;Acc:HGNC:16006]
ENSG00000113360	0,27919505	5,1219171	3,85393032	0,08036496	0,32941135	DROSHA	drosha ribonuclease III [Source:HGNC Symbol;Acc:HGNC:17904]

ENSG00000132600	-0,3506349	5,17823628	3,85331224	0,08038548	0,32941135	PRMT7	protein arginine methyltransferase 7 [Source:HGNC Symbol;Acc:HGNC:25557]
ENSG00000280433	1,16447027	-2,0428498	3,85426006	0,08035402	0,32941135	FP565260.6	novel protein, similar to trafficking protein particle complex 10 TRAPPC10
ENSG00000163636	0,2767391	6,85016975	3,85068435	0,08047279	0,32952036	PSMD6	proteasome 26S subunit, non-ATPase 6 [Source:HGNC Symbol;Acc:HGNC:9564]
ENSG00000169733	-0,2714125	6,76882547	3,84994446	0,0804974	0,32952036	RFNG	RFNG O-fucosylpeptide 3-beta-N-acetylglucosaminyltransferase [Source:HGNC Symbol;Acc:HGNC:9974]
ENSG00000184378	0,85047876	-0,5946283	3,84997773	0,08049629	0,32952036	ACTRT3	actin related protein T3 [Source:HGNC Symbol;Acc:HGNC:24022]
ENSG00000204959	1,13903685	-1,5010759	3,84921603	0,08052163	0,32952036	ARHGEF34P	Rho guanine nucleotide exchange factor 34, pseudogene [Source:HGNC Symbol;Acc:HGNC:38086]
ENSG00000213772	-0,6609063	0,03420585	3,84911896	0,08052486	0,32952036	EIF1P7	eukaryotic translation initiation factor 1 pseudogene 7 [Source:HGNC Symbol;Acc:HGNC:49620]
ENSG00000196586	0,47669254	1,92223595	3,84708709	0,08059251	0,32970483	MYO6	myosin VI [Source:HGNC Symbol;Acc:HGNC:7605]
ENSG00000065675	1,01072626	-1,4882411	3,84578021	0,08063606	0,32979063	PRKCQ	protein kinase C theta [Source:HGNC Symbol;Acc:HGNC:9410]
ENSG00000115590	-0,5485937	1,16835975	3,84345921	0,08071347	0,33001485	IL1R2	interleukin 1 receptor type 2 [Source:HGNC Symbol;Acc:HGNC:5994]
ENSG00000125691	-0,2852423	10,7560678	3,84118172	0,08078952	0,33020672	RPL23	ribosomal protein L23 [Source:HGNC Symbol;Acc:HGNC:10316]
ENSG00000167600	-0,6402008	1,77820374	3,84002443	0,0808282	0,33020672	CYP2S1	cytochrome P450 family 2 subfamily S member 1 [Source:HGNC Symbol;Acc:HGNC:15654]
ENSG00000227088	-1,0196832	-1,6670379	3,84051934	0,08081166	0,33020672	AC012494.1	novel transcript
ENSG00000130520	-0,2920081	7,07478793	3,83518428	0,08099023	0,33060386	LSM4	LSM4 homolog, U6 small nuclear RNA and mRNA degradation associated [Source:HGNC Symbol;Acc:HGNC:17259
ENSG00000132563	0,38943945	3,74301335	3,83509251	0,08099331	0,33060386	REEP2	receptor accessory protein 2 [Source:HGNC Symbol;Acc:HGNC:17975]
ENSG00000155755	-0,2693911	5,08749269	3,83521994	0,08098904	0,33060386	TMEM237	transmembrane protein 237 [Source:HGNC Symbol;Acc:HGNC:14432]
ENSG00000135535	0,29173457	8,15136307	3,83353568	0,08104551	0,33072455	CD164	CD164 molecule [Source:HGNC Symbol;Acc:HGNC:1632]
ENSG00000143921	-0,2889735	4,24569315	3,83071483	0,08114021	0,33092613	ABCG8	ATP binding cassette subfamily G member 8 [Source:HGNC Symbol;Acc:HGNC:13887]
ENSG00000211459	0,25641113	9,46473541	3,83110943	0,08112696	0,33092613	MT-RNR1	mitochondrially encoded 12S rRNA [Source:HGNC Symbol;Acc:HGNC:7470]
ENSG00000181458	-0,4507873	4,11761717	3,82895492	0,08119937	0,33107495	TMEM45A	transmembrane protein 45A [Source:HGNC Symbol;Acc:HGNC:25480]
ENSG00000247626	-0,4660724	5,02917083	3,82767141	0,08124254	0,33115857	MARS2	methionyl-tRNA synthetase 2, mitochondrial [Source:HGNC Symbol;Acc:HGNC:25133]
ENSG00000103202	-0,2783985	7,54163749	3,82557113	0,08131326	0,33123538	NME4	NME/NM23 nucleoside diphosphate kinase 4 [Source:HGNC Symbol;Acc:HGNC:7852]
ENSG00000154217	-0,486373	1,36595479	3,82613782	0,08129417	0,33123538	PITPNC1	phosphatidylinositol transfer protein cytoplasmic 1 [Source:HGNC Symbol;Acc:HGNC:21045]
ENSG00000175600	0,39296395	3,89644757	3,82509172	0,08132941	0,33123538	SUGCT	succinyl-CoA:glutarate-CoA transferase [Source:HGNC Symbol;Acc:HGNC:16001]
ENSG00000019144	-0,4551122	4,07213914	3,81807835	0,08156616	0,3315124	PHLDB1	pleckstrin homology like domain family B member 1 [Source:HGNC Symbol;Acc:HGNC:23697]
ENSG00000090263	-0,3165411	5,60426965	3,81778949	0,08157593	0,3315124	MRPS33	mitochondrial ribosomal protein S33 [Source:HGNC Symbol;Acc:HGNC:16634]
ENSG00000118246	0,29057422	6,03587218	3,81702902	0,08160166	0,3315124	FASTKD2	FAST kinase domains 2 [Source:HGNC Symbol;Acc:HGNC:29160]
ENSG00000136874	0,37209723	3,42250573	3,82035515	0,08148921	0,3315124	STX17	syntaxin 17 [Source:HGNC Symbol;Acc:HGNC:11432]
ENSG00000145740	0,4026053	4,93605224	3,8170827	0,08159984	0,3315124	SLC30A5	solute carrier family 30 member 5 [Source:HGNC Symbol;Acc:HGNC:19089]
ENSG00000174010	0,41966311	1,35590676	3,81796906	0,08156986	0,3315124	KLHL15	kelch like family member 15 [Source:HGNC Symbol;Acc:HGNC:29347]
ENSG00000205629	0,30724529	6,60463554	3,81934638	0,08152329	0,3315124	LCMT1	leucine carboxyl methyltransferase 1 [Source:HGNC Symbol;Acc:HGNC:17557]
ENSG00000230337	-0,9378343	-1,3172604	3,81949376	0,08151831	0,3315124	EXOSC10-AS1	EXOSC10 antisense RNA 1 [Source:HGNC Symbol;Acc:HGNC:40456]
ENSG00000234814	-1,4314179	-1,634838	3,81960622	0,08151451	0,3315124	SVIL2P	supervillin family member 2, pseudogene [Source:HGNC Symbol;Acc:HGNC:44959]
ENSG00000128567	0,5632436	0,73000183	3,81204634	0,08177048	0,33203153	PODXL	podocalyxin like [Source:HGNC Symbol;Acc:HGNC:9171]
ENSG00000130713	-0,2967426	5,99636157	3,81124642	0,08179762	0,33203153	EXOSC2	exosome component 2 [Source:HGNC Symbol;Acc:HGNC:17097]
ENSG00000130714	0,3553224	4,76940176	3,81125168	0,08179744	0,33203153	POMT1	protein O-mannosyltransferase 1 [Source:HGNC Symbol;Acc:HGNC:9202]
ENSG00000244086	-0,9978371	-1,7218308	3,80995226	0,08184156	0,33211761	AC110749.1	ribosomal protein S20 pseudogene 35
ENSG00000229314	-0,260854	9,69596874	3,8084641	0,08189212	0,3322305	ORM1	orosomucoid 1 [Source:HGNC Symbol;Acc:HGNC:8498]
ENSG00000012963	0,29402898	4,44433456	3,8056719	0,0819871	0,3325025	UBR7	ubiquitin protein ligase E3 component n-recognin 7 [Source:HGNC Symbol;Acc:HGNC:20344]
ENSG00000205643	-0,4179692	2,57423888	3,8051552	0,08200469	0,3325025	CDPF1	cysteine rich DPF motif domain containing 1 [Source:HGNC Symbol;Acc:HGNC:33710]
ENSG00000089123	-0,3510653	3,5974438	3,80137528	0,08213352	0,3326159	TASP1	taspase 1 [Source:HGNC Symbol;Acc:HGNC:15859]
ENSG00000094880	0,28501334	4,74187238	3,80139444	0,08213287	0,3326159	CDC23	cell division cycle 23 [Source:HGNC Symbol;Acc:HGNC:1724]
ENSG00000160087	-0,2738946	5,87316755	3,80113103	0,08214185	0,3326159	UBE2J2	ubiquitin conjugating enzyme E2 J2 [Source:HGNC Symbol;Acc:HGNC:19268]
ENSG00000169683	-0,3442299	4,90530352	3,80121094	0,08213913	0,3326159	LRRC45	leucine rich repeat containing 45 [Source:HGNC Symbol;Acc:HGNC:28302]

ENSG00000171853	-0,2937623	5,07602165	3,80099497	0,0821465	0,3326159	TRAPPC12	trafficking protein particle complex 12 [Source:HGNC Symbol;Acc:HGNC:24284]
ENSG00000141219	0,2782106	4,98050755	3,79937231	0,08220189	0,33274796	C17orf80	chromosome 17 open reading frame 80 [Source:HGNC Symbol;Acc:HGNC:29601]
ENSG00000137106	-0,2627392	6,91267306	3,79827177	0,08223949	0,33280793	GRHPR	glyoxylate and hydroxypyruvate reductase [Source:HGNC Symbol;Acc:HGNC:4570]
ENSG00000140525	0,26928739	5,14771547	3,79594996	0,08231887	0,33303695	FANCI	FA complementation group I [Source:HGNC Symbol;Acc:HGNC:25568]
ENSG00000283828	-0,3935074	1,8441137	3,79043174	0,08250795	0,33370946	AL137002.2	WAS/WASL-interacting protein family member 2-like [Source:NCBI gene (formerly Entrezgene);Acc:102724474]
ENSG00000167767	-0,6013777	0,218818	3,7880259	0,08259055	0,3339511	KRT80	keratin 80 [Source:HGNC Symbol;Acc:HGNC:27056]
ENSG00000125977	-0,3000152	8,55677578	3,78566486	0,08267171	0,33411034	EIF2S2	eukaryotic translation initiation factor 2 subunit beta [Source:HGNC Symbol;Acc:HGNC:3266]
ENSG00000184381	-0,4292271	1,40407285	3,78554987	0,08267567	0,33411034	PLA2G6	phospholipase A2 group VI [Source:HGNC Symbol;Acc:HGNC:9039]
ENSG00000140521	0,33013732	5,77854561	3,78395508	0,08273055	0,33423968	POLG	DNA polymerase gamma, catalytic subunit [Source:HGNC Symbol;Acc:HGNC:9179]
ENSG00000059804	-0,3913196	8,10224949	3,77941883	0,08288692	0,33474377	SLC2A3	solute carrier family 2 member 3 [Source:HGNC Symbol;Acc:HGNC:11007]
ENSG00000272556	1,32472886	0,36909194	3,77900651	0,08290115	0,33474377	GTF2IP13	general transcription factor IIi pseudogene 13 [Source:HGNC Symbol;Acc:HGNC:51725]
ENSG00000157450	-0,4872788	2,41626415	3,77613974	0,08300019	0,33486598	RNF111	ring finger protein 111 [Source:HGNC Symbol;Acc:HGNC:17384]
ENSG00000230383	-0,3609143	2,10944061	3,77651885	0,08298708	0,33486598	AC009245.1	ribosomal protein L6 (RPL6) pseudogene
ENSG00000241553	0,28162724	6,02890549	3,77714002	0,08296561	0,33486598	ARPC4	actin related protein 2/3 complex subunit 4 [Source:HGNC Symbol;Acc:HGNC:707]
ENSG00000240914	-0,4225365	1,50608114	3,77382208	0,08308036	0,3350969	RPL15P2	ribosomal protein L15 pseudogene 2 [Source:HGNC Symbol;Acc:HGNC:19803]
ENSG00000103152	-0,3041266	6,58382755	3,76941051	0,08323324	0,33529896	MPG	N-methylpurine DNA glycosylase [Source:HGNC Symbol;Acc:HGNC:7211]
ENSG00000104517	0,38753464	5,40025672	3,76813947	0,08327735	0,33529896	UBR5	ubiquitin protein ligase E3 component n-recognin 5 [Source:HGNC Symbol;Acc:HGNC:16806]
ENSG00000140153	0,30795658	4,16343914	3,7688352	0,0832532	0,33529896	WDR20	WD repeat domain 20 [Source:HGNC Symbol;Acc:HGNC:19667]
ENSG00000153107	0,2925333	5,00215644	3,76774295	0,08329112	0,33529896	ANAPC1	anaphase promoting complex subunit 1 [Source:HGNC Symbol;Acc:HGNC:19988]
ENSG00000164180	0,32042767	3,1532808	3,76808088	0,08327938	0,33529896	TMEM161B	transmembrane protein 161B [Source:HGNC Symbol;Acc:HGNC:28483]
ENSG00000188706	0,28272725	5,98894373	3,76971547	0,08322266	0,33529896	ZDHHC9	zinc finger DHHC-type palmitoyltransferase 9 [Source:HGNC Symbol;Acc:HGNC:18475]
ENSG00000273361	-0,895807	-1,4646694	3,76855335	0,08326298	0,33529896	AC021016.2	novel transcript, antisense to CTDSP1
ENSG00000171658	-0,7637471	-0,3360242	3,76686116	0,08332174	0,33532985	NMRAL2P	NmrA like redox sensor 2, pseudogene [Source:HGNC Symbol;Acc:HGNC:52332]
ENSG00000071073	-0,2803593	5,3311195	3,76026921	0,08355115	0,33553651	MGAT4A	alpha-1,3-mannosyl-glycoprotein 4-beta-N-acetylglucosaminyltransferase A [Source:HGNC Symbol;Acc:HGNC:704
ENSG00000081019	0,39135486	2,24552972	3,75922926	0,08358741	0,33553651	RSBN1	round spermatid basic protein 1 [Source:HGNC Symbol;Acc:HGNC:25642]
ENSG00000092203	0,30482919	6,63096115	3,75813971	0,08362543	0,33553651	TOX4	TOX high mobility group box family member 4 [Source:HGNC Symbol;Acc:HGNC:20161]
ENSG00000105281	-0,3546744	8,13552742	3,76397584	0,08342206	0,33553651	SLC1A5	solute carrier family 1 member 5 [Source:HGNC Symbol;Acc:HGNC:10943]
ENSG00000108591	-0,290045	5,39689383	3,75582086	0,08370641	0,33553651	DRG2	developmentally regulated GTP binding protein 2 [Source:HGNC Symbol;Acc:HGNC:3030]
ENSG00000115694	-0,2836098	7,0445435	3,75932877	0,08358394	0,33553651	STK25	serine/threonine kinase 25 [Source:HGNC Symbol;Acc:HGNC:11404]
ENSG00000119969	0,31795346	4,15505808	3,76306643	0,08345371	0,33553651	HELLS	helicase, lymphoid specific [Source:HGNC Symbol;Acc:HGNC:4861]
ENSG00000125779	0,25938374	6,44735221	3,75550022	0,08371761	0,33553651	PANK2	pantothenate kinase 2 [Source:HGNC Symbol;Acc:HGNC:15894]
ENSG00000135457	-0,284238	4,28853853	3,76169626	0,08350142	0,33553651	TFCP2	transcription factor CP2 [Source:HGNC Symbol;Acc:HGNC:11748]
ENSG00000147912	0,3499125	2,46822505	3,75767675	0,08364159	0,33553651	FBXO10	F-box protein 10 [Source:HGNC Symbol;Acc:HGNC:13589]
ENSG00000213236	0,98192116	-1,5238536	3,75682888	0,08367119	0,33553651	YWHAZP2	tyrosine 3-monooxygenase/tryptophan 5-monooxygenase activation protein zeta pseudogene 2 [Source:HGNC Sy
ENSG00000213619	0,25970407	7,15026813	3,75583481	0,08370592	0,33553651	NDUFS3	NADH:ubiquinone oxidoreductase core subunit S3 [Source:HGNC Symbol;Acc:HGNC:7710]
ENSG00000226124	0,85230937	-0,3628122	3,75635535	0,08368773	0,33553651	FTCDNL1	formiminotransferase cyclodeaminase N-terminal like [Source:HGNC Symbol;Acc:HGNC:48661]
ENSG00000269713	0,48401534	1,4264028	3,76209742	0,08348745	0,33553651	NBPF9	NBPF member 9 [Source:HGNC Symbol;Acc:HGNC:31991]
ENSG00000275645	0,86486647	-0,6088112	3,76127346	0,08351615	0,33553651	AC068338.3	novel transcript, antisense to NEIL1
ENSG00000161016	-0,2698676	11,0141542	3,75432915	0,08375855	0,33560852	RPL8	ribosomal protein L8 [Source:HGNC Symbol;Acc:HGNC:10368]
ENSG00000117751	0,28809248	5,85254399	3,75289953	0,08380856	0,33571684	PPP1R8	protein phosphatase 1 regulatory subunit 8 [Source:HGNC Symbol;Acc:HGNC:9296]
ENSG00000275832	-0,9776891	-1,3919011	3,75151968	0,08385687	0,33581827	ARHGAP23	Rho GTPase activating protein 23 [Source:HGNC Symbol;Acc:HGNC:29293]
ENSG00000166135	-0,2839637	5,2824182	3,75009691	0,08390672	0,33592581	HIF1AN	hypoxia inducible factor 1 subunit alpha inhibitor [Source:HGNC Symbol;Acc:HGNC:17113]
ENSG00000104320	-0,298824	4,66627638	3,74187556	0,08419549	0,33643895	NBN	nibrin [Source:HGNC Symbol;Acc:HGNC:7652]
ENSG00000129932	0,28939382	4,40039228	3,74198794	0,08419154	0,33643895	DOHH	deoxyhypusine hydroxylase [Source:HGNC Symbol;Acc:HGNC:28662]

ENSG00000172888	0,35182805	3,06687937	3,74505953	0,08408351	0,33643895	ZNF621	zinc finger protein 621 [Source:HGNC Symbol;Acc:HGNC:24787]
ENSG00000173110	-2,1079066	1,01186572	3,7431516	0,08415059	0,33643895	HSPA6	heat shock protein family A (Hsp70) member 6 [Source:HGNC Symbol;Acc:HGNC:5239]
ENSG00000176444	-0,2794299	4,57524255	3,74185841	0,0841961	0,33643895	CLK2	CDC like kinase 2 [Source:HGNC Symbol;Acc:HGNC:2069]
ENSG00000182272	0,38034779	3,96539983	3,74403332	0,08411958	0,33643895	B4GALNT4	beta-1,4-N-acetyl-galactosaminyltransferase 4 [Source:HGNC Symbol;Acc:HGNC:26315]
ENSG00000274756	-0,8488036	-1,2511902	3,74219966	0,08418409	0,33643895	AC243732.1	ribosomal protein S2 (RPS2) pseudogene
ENSG00000163214	0,37560845	4,89024465	3,73989251	0,08426534	0,33662354	DHX57	DExH-box helicase 57 [Source:HGNC Symbol;Acc:HGNC:20086]
ENSG00000234009	-0,3610535	2,4856247	3,73850384	0,08431429	0,33672702	RPL5P34	ribosomal protein L5 pseudogene 34 [Source:HGNC Symbol;Acc:HGNC:36463]
ENSG00000213398	-0,2835686	5,21643304	3,73467356	0,0844495	0,33699914	LCAT	lecithin-cholesterol acyltransferase [Source:HGNC Symbol;Acc:HGNC:6522]
ENSG00000251587	-0,7856869	-0,9864943	3,73461321	0,08445163	0,33699914	LDHAP1	lactate dehydrogenase A pseudogene 1 [Source:HGNC Symbol;Acc:HGNC:6536]
ENSG00000277182	0,87034307	-0,8112499	3,73535942	0,08442526	0,33699914	AC006449.5	novel transcript, antisense to PCGF2
ENSG00000021574	0,30662427	4,00905105	3,72886389	0,0846551	0,33717121	SPAST	spastin [Source:HGNC Symbol;Acc:HGNC:11233]
ENSG00000101439	-0,255016	10,8305638	3,73092432	0,08458211	0,33717121	CST3	cystatin C [Source:HGNC Symbol;Acc:HGNC:2475]
ENSG00000146733	0,32842126	6,79925354	3,73196928	0,08454512	0,33717121	PSPH	phosphoserine phosphatase [Source:HGNC Symbol;Acc:HGNC:9577]
ENSG00000180992	-0,2620827	7,97669144	3,73072259	0,08458925	0,33717121	MRPL14	mitochondrial ribosomal protein L14 [Source:HGNC Symbol;Acc:HGNC:14279]
ENSG00000184047	0,93358738	-1,2269701	3,73198364	0,08454461	0,33717121	DIABLO	diablo IAP-binding mitochondrial protein [Source:HGNC Symbol;Acc:HGNC:21528]
ENSG00000204899	-0,3296359	3,82124872	3,72882979	0,08465631	0,33717121	MZT1	mitotic spindle organizing protein 1 [Source:HGNC Symbol;Acc:HGNC:33830]
ENSG00000205309	0,95463052	-1,2777965	3,7299762	0,08461568	0,33717121	NT5M	5',3'-nucleotidase, mitochondrial [Source:HGNC Symbol;Acc:HGNC:15769]
ENSG00000183145	0,56144207	0,32389284	3,72775348	0,08469447	0,33723125	RIPPLY3	ripply transcriptional repressor 3 [Source:HGNC Symbol;Acc:HGNC:3047]
ENSG00000005020	0,29854092	3,49525742	3,72303363	0,08486207	0,3377145	SKAP2	src kinase associated phosphoprotein 2 [Source:HGNC Symbol;Acc:HGNC:15687]
ENSG00000008323	0,29693808	4,52187511	3,72362938	0,08484089	0,3377145	PLEKHG6	pleckstrin homology and RhoGEF domain containing G6 [Source:HGNC Symbol;Acc:HGNC:25562]
ENSG00000063978	0,33709344	5,31522793	3,72214549	0,08489365	0,33774818	RNF4	ring finger protein 4 [Source:HGNC Symbol;Acc:HGNC:10067]
ENSG00000112874	-0,3828753	1,55351705	3,7211329	0,08492968	0,33779952	NUDT12	nudix hydrolase 12 [Source:HGNC Symbol;Acc:HGNC:18826]
ENSG00000143891	0,26229683	6,63497972	3,72034689	0,08495766	0,33781884	GALM	galactose mutarotase [Source:HGNC Symbol;Acc:HGNC:24063]
ENSG00000214595	0,35561643	3,08198794	3,71663114	0,08509008	0,33825334	EML6	EMAP like 6 [Source:HGNC Symbol;Acc:HGNC:35412]
ENSG00000151239	-0,2636411	5,46456373	3,7148564	0,08515343	0,33841306	TWF1	twinfilin actin binding protein 1 [Source:HGNC Symbol;Acc:HGNC:9620]
ENSG00000163608	0,2858419	4,49808854	3,71100343	0,08529115	0,3388682	NEPRO	nucleolus and neural progenitor protein [Source:HGNC Symbol;Acc:HGNC:24496]
ENSG00000120833	-0,6127949	0,32516211	3,70961055	0,085341	0,3389741	SOCS2	suppressor of cytokine signaling 2 [Source:HGNC Symbol;Acc:HGNC:19382]
ENSG00000049283	-0,6587473	1,47811289	3,70776362	0,08540717	0,3391447	EPN3	epsin 3 [Source:HGNC Symbol;Acc:HGNC:18235]
ENSG00000136709	0,26356476	6,50942193	3,70332047	0,08556661	0,33966679	WDR33	WD repeat domain 33 [Source:HGNC Symbol;Acc:HGNC:25651]
ENSG00000179085	-0,2888975	4,64020298	3,70280453	0,08558515	0,33966679	DPM3	dolichyl-phosphate mannosyltransferase subunit 3, regulatory [Source:HGNC Symbol;Acc:HGNC:3007]
ENSG00000054793	0,49923387	3,53303783	3,70130108	0,0856392	0,33969574	ATP9A	ATPase phospholipid transporting 9A (putative) [Source:HGNC Symbol;Acc:HGNC:13540]
ENSG00000169895	-0,2705135	5,74835485	3,7010598	0,08564788	0,33969574	SYAP1	synapse associated protein 1 [Source:HGNC Symbol;Acc:HGNC:16273]
ENSG00000184678	0,5801467	0,21459435	3,70066168	0,0856622	0,33969574	H2BC21	H2B clustered histone 21 [Source:HGNC Symbol;Acc:HGNC:4760]
ENSG00000125166	0,39604605	8,40923994	3,69862604	0,08573548	0,33989353	GOT2	glutamic-oxaloacetic transaminase 2 [Source:HGNC Symbol;Acc:HGNC:4433]
ENSG00000184056	-0,5721097	0,77369589	3,69798386	0,08575861	0,33989353	VPS33B	VPS33B late endosome and lysosome associated [Source:HGNC Symbol;Acc:HGNC:12712]
ENSG00000152894	0,29797474	6,95251037	3,69732374	0,0857824	0,3398956	PTPRK	protein tyrosine phosphatase receptor type K [Source:HGNC Symbol;Acc:HGNC:9674]
ENSG00000118257	0,58300025	1,21690814	3,69557135	0,08584559	0,34005374	NRP2	neuropilin 2 [Source:HGNC Symbol;Acc:HGNC:8005]
ENSG00000164989	0,78870815	-0,2196463	3,69430644	0,08589123	0,34014233	CCDC171	coiled-coil domain containing 171 [Source:HGNC Symbol;Acc:HGNC:29828]
ENSG00000106258	0,82571668	-0,1906081	3,69292699	0,08594105	0,34024528	CYP3A5	cytochrome P450 family 3 subfamily A member 5 [Source:HGNC Symbol;Acc:HGNC:2638]
ENSG00000135951	-0,4453252	1,51882935	3,69229702	0,08596381	0,34024528	TSGA10	testis specific 10 [Source:HGNC Symbol;Acc:HGNC:14927]
ENSG00000143512	-1,284468	-1,5854664	3,69057155	0,0860262	0,34029069	HHIPL2	HHIP like 2 [Source:HGNC Symbol;Acc:HGNC:25842]
ENSG00000182057	-0,3714967	1,84890392	3,69004724	0,08604516	0,34029069	OGFRP1	opioid growth factor receptor pseudogene 1 [Source:HGNC Symbol;Acc:HGNC:50511]
ENSG00000214021	0,67243786	0,40136662	3,69025374	0,08603769	0,34029069	TTLL3	tubulin tyrosine ligase like 3 [Source:HGNC Symbol;Acc:HGNC:24483]
ENSG00000049167	-0,3316276	3,38363891	3,68650721	0,08617337	0,3403961	ERCC8	ERCC excision repair 8, CSA ubiquitin ligase complex subunit [Source:HGNC Symbol;Acc:HGNC:3439]

ENSG00000105771	0,31754043	4,75821292	3,68545217	0,08621162	0,3403961	SMG9	SMG9 nonsense mediated mRNA decay factor [Source:HGNC Symbol;Acc:HGNC:25763]
ENSG00000135045	-0,3180831	3,80763876	3,68547882	0,08621066	0,3403961	C9orf40	chromosome 9 open reading frame 40 [Source:HGNC Symbol;Acc:HGNC:23433]
ENSG00000170889	-0,2521945	10,3445666	3,68663682	0,08616867	0,3403961	RPS9	ribosomal protein S9 [Source:HGNC Symbol;Acc:HGNC:10442]
ENSG00000177692	0,41534232	1,49032431	3,68639807	0,08617732	0,3403961	DNAJC28	DnaJ heat shock protein family (Hsp40) member C28 [Source:HGNC Symbol;Acc:HGNC:1297]
ENSG00000234750	-1,0126542	-1,8876304	3,68554178	0,08620837	0,3403961	AC012618.2	ribosomal protein S2 (RPS2) pseudogene
ENSG00000101400	-0,3043837	4,81715384	3,68466986	0,08624	0,34041615	SNTA1	syntrophin alpha 1 [Source:HGNC Symbol;Acc:HGNC:11167]
ENSG00000134779	0,3799707	5,61962974	3,68308169	0,08629765	0,34051805	TPGS2	tubulin polyglutamylase complex subunit 2 [Source:HGNC Symbol;Acc:HGNC:24561]
ENSG00000180172	-1,1812904	-1,89024	3,68267471	0,08631243	0,34051805	RPS12P23	ribosomal protein S12 pseudogene 23 [Source:HGNC Symbol;Acc:HGNC:35573]
ENSG00000109618	0,39634245	2,7040707	3,67849734	0,08646434	0,34102525	SEPSECS	Sep (O-phosphoserine) tRNA:Sec (selenocysteine) tRNA synthase [Source:HGNC Symbol;Acc:HGNC:30605]
ENSG00000124783	-0,2573346	8,4691182	3,67780583	0,08648952	0,34103249	SSR1	signal sequence receptor subunit 1 [Source:HGNC Symbol;Acc:HGNC:11323]
ENSG00000034693	0,2876088	5,40329906	3,6757887	0,08656302	0,34123019	PEX3	peroxisomal biogenesis factor 3 [Source:HGNC Symbol;Acc:HGNC:8858]
ENSG00000077157	0,76391465	1,49113461	3,67418942	0,08662134	0,34136801	PPP1R12B	protein phosphatase 1 regulatory subunit 12B [Source:HGNC Symbol;Acc:HGNC:7619]
ENSG00000106144	0,36561845	3,45130089	3,67319845	0,08665751	0,34141845	CASP2	caspase 2 [Source:HGNC Symbol;Acc:HGNC:1503]
ENSG00000100612	0,31185554	5,56524841	3,66741176	0,08686909	0,34179116	DHRS7	dehydrogenase/reductase 7 [Source:HGNC Symbol;Acc:HGNC:21524]
ENSG00000136631	0,28552162	3,88404648	3,66793585	0,0868499	0,34179116	VPS45	vacuolar protein sorting 45 homolog [Source:HGNC Symbol;Acc:HGNC:14579]
ENSG00000160785	0,47152722	2,70706095	3,66806248	0,08684527	0,34179116	SLC25A44	solute carrier family 25 member 44 [Source:HGNC Symbol;Acc:HGNC:29036]
ENSG00000185100	-0,5653659	0,28799463	3,66839865	0,08683296	0,34179116	ADSS1	adenylosuccinate synthase 1 [Source:HGNC Symbol;Acc:HGNC:20093]
ENSG00000213918	0,42446766	1,34454473	3,66828904	0,08683698	0,34179116	DNASE1	deoxyribonuclease 1 [Source:HGNC Symbol;Acc:HGNC:2956]
ENSG00000226781	-0,8021187	-1,1480743	3,66637544	0,08690705	0,34184845	TBCAP1	tubulin folding cofactor A pseudogene 1 [Source:HGNC Symbol;Acc:HGNC:42630]
ENSG00000132305	0,34475098	7,40979781	3,66553444	0,08693788	0,34187764	IMMT	inner membrane mitochondrial protein [Source:HGNC Symbol;Acc:HGNC:6047]
ENSG00000100354	0,54018003	3,55252063	3,66448865	0,08697622	0,34193372	TNRC6B	trinucleotide repeat containing adaptor 6B [Source:HGNC Symbol;Acc:HGNC:29190]
ENSG00000163071	-0,4023882	1,58957976	3,66386905	0,08699895	0,34193372	SPATA18	spermatogenesis associated 18 [Source:HGNC Symbol;Acc:HGNC:29579]
ENSG00000104331	-0,2722377	5,56710668	3,66247498	0,08705012	0,34202817	BPNT2	3'(2'), 5'-bisphosphate nucleotidase 2 [Source:HGNC Symbol;Acc:HGNC:26019]
ENSG00000125733	0,27283485	5,95421486	3,66052983	0,08712157	0,34202817	TRIP10	thyroid hormone receptor interactor 10 [Source:HGNC Symbol;Acc:HGNC:12304]
ENSG00000151090	0,34053787	2,75906426	3,65939068	0,08716345	0,34202817	THRB	thyroid hormone receptor beta [Source:HGNC Symbol;Acc:HGNC:11799]
ENSG00000159459	0,41151941	2,83165524	3,65986038	0,08714618	0,34202817	UBR1	ubiquitin protein ligase E3 component n-recognin 1 [Source:HGNC Symbol;Acc:HGNC:16808]
ENSG00000261770	1,02288779	-0,2263243	3,66125721	0,08709484	0,34202817	AC006504.1	novel transcript
ENSG00000270419	-0,9932981	-1,6320833	3,66185116	0,08707302	0,34202817	CAHM	colon adenocarcinoma hypermethylated [Source:HGNC Symbol;Acc:HGNC:42860]
ENSG00000111058	0,36062674	6,85494973	3,6574394	0,08723525	0,3420343	ACSS3	acyl-CoA synthetase short chain family member 3 [Source:HGNC Symbol;Acc:HGNC:24723]
ENSG00000148356	-0,451872	3,79811495	3,65838781	0,08720035	0,3420343	LRSAM1	leucine rich repeat and sterile alpha motif containing 1 [Source:HGNC Symbol;Acc:HGNC:25135]
ENSG00000177606	-0,6001629	6,00673571	3,65788173	0,08721897	0,3420343	JUN	Jun proto-oncogene, AP-1 transcription factor subunit [Source:HGNC Symbol;Acc:HGNC:6204]
ENSG00000266074	0,48968583	1,08711175	3,65461539	0,0873393	0,34235036	BAHCC1	BAH domain and coiled-coil containing 1 [Source:HGNC Symbol;Acc:HGNC:29279]
ENSG00000081181	-0,3839741	6,01599489	3,65319097	0,08739184	0,34237258	ARG2	arginase 2 [Source:HGNC Symbol;Acc:HGNC:664]
ENSG00000188266	-0,3681475	2,49955831	3,65378088	0,08737007	0,34237258	HYKK	hydroxylysine kinase [Source:HGNC Symbol;Acc:HGNC:34403]
ENSG00000100263	-0,3037396	4,4299477	3,65062681	0,08748651	0,3426355	RHBDD3	rhomboid domain containing 3 [Source:HGNC Symbol;Acc:HGNC:1308]
ENSG00000129055	-0,3194873	5,16714096	3,64999786	0,08750976	0,3426355	ANAPC13	anaphase promoting complex subunit 13 [Source:HGNC Symbol;Acc:HGNC:24540]
ENSG00000229814	-0,8560005	-1,0414351	3,6488347	0,08755276	0,3426355	RPL35AP21	ribosomal protein L35a pseudogene 21 [Source:HGNC Symbol;Acc:HGNC:36814]
ENSG00000262454	-0,6877683	0,00456614	3,64942963	0,08753076	0,3426355	MIR193BHG	MIR193B host gene [Source:HGNC Symbol;Acc:HGNC:51945]
ENSG00000176919	0,41008631	2,20715475	3,64538385	0,08768051	0,34295169	C8G	complement C8 gamma chain [Source:HGNC Symbol;Acc:HGNC:1354]
ENSG00000260404	0,95096536	1,31871018	3,64563256	0,0876713	0,34295169	AC110079.1	general transcription factor IIi (GTF2I) pseudogene
ENSG00000152683	0,31422012	4,89770287	3,63883012	0,08792377	0,34381113	SLC30A6	solute carrier family 30 member 6 [Source:HGNC Symbol;Acc:HGNC:19305]
ENSG00000014123	0,40729835	4,31965836	3,63651155	0,08801004	0,34387232	UFL1	UFM1 specific ligase 1 [Source:HGNC Symbol;Acc:HGNC:23039]
ENSG00000152056	-0,4818841	1,26523157	3,63662951	0,08800564	0,34387232	AP1S3	adaptor related protein complex 1 subunit sigma 3 [Source:HGNC Symbol;Acc:HGNC:18971]
ENSG00000162402	0,31796216	5,07300559	3,63653835	0,08800904	0,34387232	USP24	ubiquitin specific peptidase 24 [Source:HGNC Symbol;Acc:HGNC:12623]

ENSG00000106803	-0,3422902	7,5070963	3,63578631	0,08803704	0,34388587	SEC61B	SEC61 translocon subunit beta [Source:HGNC Symbol;Acc:HGNC:16993]
ENSG00000105640	-0,2820538	9,9956838	3,63334328	0,08812809	0,34408419	RPL18A	ribosomal protein L18a [Source:HGNC Symbol;Acc:HGNC:10311]
ENSG00000179528	-0,3732176	2,55107835	3,63316009	0,08813492	0,34408419	LBX2	ladybird homeobox 2 [Source:HGNC Symbol;Acc:HGNC:15525]
ENSG00000248932	-1,1185231	-1,5881841	3,63017247	0,08824644	0,34442751	AC046134.2	novel transcript, antisense to RBP2, RBP1 & NMNAT3
ENSG00000163596	0,58689886	-0,0088571	3,62796432	0,08832897	0,34465757	ICA1L	islet cell autoantigen 1 like [Source:HGNC Symbol;Acc:HGNC:14442]
ENSG00000225093	-0,3583389	1,90160849	3,62498254	0,08844058	0,34500092	RPL3P7	ribosomal protein L3 pseudogene 7 [Source:HGNC Symbol;Acc:HGNC:36797]
ENSG00000143771	0,26817221	5,9198081	3,62246163	0,08853508	0,345018	CNIH4	cornichon family AMPA receptor auxiliary protein 4 [Source:HGNC Symbol;Acc:HGNC:25013]
ENSG00000167925	-0,2838539	5,85782622	3,62131407	0,08857814	0,345018	GHDC	GH3 domain containing [Source:HGNC Symbol;Acc:HGNC:24438]
ENSG00000176953	0,26687256	5,50262413	3,62108695	0,08858666	0,345018	NFATC2IP	nuclear factor of activated T cells 2 interacting protein [Source:HGNC Symbol;Acc:HGNC:25906]
ENSG00000186115	-0,4210539	1,5429832	3,62205072	0,08855049	0,345018	CYP4F2	cytochrome P450 family 4 subfamily F member 2 [Source:HGNC Symbol;Acc:HGNC:2645]
ENSG00000223802	0,33709618	2,86493527	3,62356842	0,08849357	0,345018	CERS1	ceramide synthase 1 [Source:HGNC Symbol;Acc:HGNC:14253]
ENSG00000253506	-0,5057532	0,38292436	3,6241846	0,08847048	0,345018	NACA2	nascent polypeptide associated complex subunit alpha 2 [Source:HGNC Symbol;Acc:HGNC:23290]
ENSG00000173530	0,37898401	5,05799184	3,61904771	0,08866325	0,34522426	TNFRSF10D	TNF receptor superfamily member 10d [Source:HGNC Symbol;Acc:HGNC:11907]
ENSG00000183798	0,7735426	-0,5910295	3,61600196	0,0887778	0,34557818	EMILIN3	elastin microfibril interfacer 3 [Source:HGNC Symbol;Acc:HGNC:16123]
ENSG00000145916	-0,3018695	4,66194269	3,6143445	0,08884022	0,34572902	RMND5B	required for meiotic nuclear division 5 homolog B [Source:HGNC Symbol;Acc:HGNC:26181]
ENSG00000164405	-0,3278193	7,38273953	3,61321249	0,08888288	0,34579497	UQCRQ	ubiquinol-cytochrome c reductase complex III subunit VII [Source:HGNC Symbol;Acc:HGNC:29594]
ENSG00000180008	0,46885794	2,02620066	3,61263886	0,08890451	0,34579497	SOCS4	suppressor of cytokine signaling 4 [Source:HGNC Symbol;Acc:HGNC:19392]
ENSG00000267534	0,74565321	-0,1299589	3,60804706	0,08907787	0,34637706	S1PR2	sphingosine-1-phosphate receptor 2 [Source:HGNC Symbol;Acc:HGNC:3169]
ENSG00000273204	-0,8679296	-0,7927	3,60653122	0,08913519	0,34650773	AC104506.1	novel transcript, antisense to EXTL2
ENSG00000232176	-0,2917256	4,28924751	3,60412012	0,08922647	0,34667807	AL161909.1	ribosomal protein S6 (RPS6) pseudogene
ENSG00000259612	-0,3809069	2,63555752	3,60435302	0,08921765	0,34667807	EEF1A1P22	eukaryotic translation elongation factor 1 alpha 1 pseudogene 22 [Source:HGNC Symbol;Acc:HGNC:37895]
ENSG00000011105	0,2571495	5,62234962	3,60193971	0,08930912	0,3468147	TSPAN9	tetraspanin 9 [Source:HGNC Symbol;Acc:HGNC:21640]
ENSG00000130414	0,32549469	7,27231898	3,60200048	0,08930681	0,3468147	NDUFA10	NADH:ubiquinone oxidoreductase subunit A10 [Source:HGNC Symbol;Acc:HGNC:7684]
ENSG00000058668	0,60367517	3,54035921	3,59857354	0,0894369	0,34719107	ATP2B4	ATPase plasma membrane Ca2+ transporting 4 [Source:HGNC Symbol;Acc:HGNC:817]
ENSG00000118557	-1,0443638	-1,806791	3,59696149	0,08949817	0,34719107	PMFBP1	polyamine modulated factor 1 binding protein 1 [Source:HGNC Symbol;Acc:HGNC:17728]
ENSG00000157911	-0,3214883	5,16134437	3,59625976	0,08952487	0,34719107	PEX10	peroxisomal biogenesis factor 10 [Source:HGNC Symbol;Acc:HGNC:8851]
ENSG00000167969	-0,2779823	6,45406137	3,59647009	0,08951686	0,34719107	ECI1	enoyl-CoA delta isomerase 1 [Source:HGNC Symbol;Acc:HGNC:2703]
ENSG00000237298	0,77119027	-0,6345114	3,59641657	0,0895189	0,34719107	TTN-AS1	TTN antisense RNA 1 [Source:HGNC Symbol;Acc:HGNC:44124]
ENSG00000178385	0,4872416	0,93080158	3,59384359	0,08961684	0,34745553	PLEKHM3	pleckstrin homology domain containing M3 [Source:HGNC Symbol;Acc:HGNC:34006]
ENSG00000140396	0,58891018	3,09921742	3,59158365	0,08970298	0,34761734	NCOA2	nuclear receptor coactivator 2 [Source:HGNC Symbol;Acc:HGNC:7669]
ENSG00000164081	0,25925862	6,68840212	3,59095245	0,08972705	0,34761734	TEX264	testis expressed 264, ER-phagy receptor [Source:HGNC Symbol;Acc:HGNC:30247]
ENSG00000184117	-0,2669517	6,91275497	3,59087627	0,08972996	0,34761734	NIPSNAP1	nipsnap homolog 1 [Source:HGNC Symbol;Acc:HGNC:7827]
ENSG00000006634	0,34119003	4,93272962	3,58836145	0,08982597	0,347874	DBF4	DBF4 zinc finger [Source:HGNC Symbol;Acc:HGNC:17364]
ENSG00000125449	-0,3070187	4,35454176	3,58755315	0,08985686	0,347874	ARMC7	armadillo repeat containing 7 [Source:HGNC Symbol;Acc:HGNC:26168]
ENSG00000221949	-1,1348064	-1,5485257	3,58727088	0,08986765	0,347874	LINC01465	long intergenic non-protein coding RNA 1465 [Source:HGNC Symbol;Acc:HGNC:26364]
ENSG00000259376	-0,8931826	-0,9795536	3,5850316	0,0899533	0,34811332	AC090907.1	novel transcript, antisense to LRRK1
ENSG00000172409	0,27298147	4,68851654	3,58352992	0,0900108	0,34824358	CLP1	cleavage factor polyribonucleotide kinase subunit 1 [Source:HGNC Symbol;Acc:HGNC:16999]
ENSG00000100823	-0,2518502	8,54900906	3,58222916	0,09006064	0,34825195	APEX1	apurinic/apyrimidinic endodeoxyribonuclease 1 [Source:HGNC Symbol;Acc:HGNC:587]
ENSG00000177728	0,29086047	5,4988244	3,58236645	0,09005538	0,34825195	TMEM94	transmembrane protein 94 [Source:HGNC Symbol;Acc:HGNC:28983]
ENSG00000003402	-0,290594	5,42143598	3,57571031	0,09031095	0,34908561	CFLAR	CASP8 and FADD like apoptosis regulator [Source:HGNC Symbol;Acc:HGNC:1876]
ENSG00000033050	-0,3508811	4,18318468	3,57537056	0,09032402	0,34908561	ABCF2	ATP binding cassette subfamily F member 2 [Source:HGNC Symbol;Acc:HGNC:71]
ENSG00000087274	0,31052477	6,6258793	3,57122043	0,09048386	0,34926633	ADD1	adducin 1 [Source:HGNC Symbol;Acc:HGNC:243]
ENSG00000174705	-0,8083194	1,95914673	3,57160779	0,09046893	0,34926633	SH3PXD2B	SH3 and PX domains 2B [Source:HGNC Symbol;Acc:HGNC:29242]
ENSG00000178502	0,69503501	1,28715787	3,57105307	0,09049032	0,34926633	KLHL11	kelch like family member 11 [Source:HGNC Symbol;Acc:HGNC:19008]

ENSG00000196323	0,41635085	2,86210787	3,57206392	0,09045135	0,34926633	ZBTB44	zinc finger and BTB domain containing 44 [Source:HGNC Symbol;Acc:HGNC:25001]
ENSG00000229873	-0,6065754	0,0893124	3,57257704	0,09043157	0,34926633	OGFR-AS1	OGFR antisense RNA 1 [Source:HGNC Symbol;Acc:HGNC:40724]
ENSG00000120784	0,42171396	2,42943388	3,56857062	0,09058611	0,34954371	ZFP30	ZFP30 zinc finger protein [Source:HGNC Symbol;Acc:HGNC:29555]
ENSG00000070501	0,32984127	5,48807289	3,56506769	0,0907215	0,3499737	POLB	DNA polymerase beta [Source:HGNC Symbol;Acc:HGNC:9174]
ENSG00000129195	-0,295706	4,60546187	3,56442878	0,09074622	0,34997666	PIMREG	PICALM interacting mitotic regulator [Source:HGNC Symbol;Acc:HGNC:25483]
ENSG00000131188	-0,3779984	2,02487229	3,56117434	0,09087228	0,3503271	PRR7	proline rich 7, synaptic [Source:HGNC Symbol;Acc:HGNC:28130]
ENSG00000178966	0,40153712	2,59991059	3,560845	0,09088505	0,3503271	RMI1	RecQ mediated genome instability 1 [Source:HGNC Symbol;Acc:HGNC:25764]
ENSG00000144655	-0,3047554	4,40320075	3,55922351	0,09094795	0,35047709	CSRNP1	cysteine and serine rich nuclear protein 1 [Source:HGNC Symbol;Acc:HGNC:14300]
ENSG00000075426	-0,3323943	3,62193381	3,55439352	0,09113565	0,35071541	FOSL2	FOS like 2, AP-1 transcription factor subunit [Source:HGNC Symbol;Acc:HGNC:3798]
ENSG00000128739	0,41195504	4,09920586	3,5541116	0,09114662	0,35071541	SNRPN	small nuclear ribonucleoprotein polypeptide N [Source:HGNC Symbol;Acc:HGNC:11164]
ENSG00000129910	-0,5286331	2,04169109	3,55525311	0,09110221	0,35071541	CDH15	cadherin 15 [Source:HGNC Symbol;Acc:HGNC:1754]
ENSG00000133065	0,4910085	0,86811267	3,55299838	0,09118995	0,35071541	SLC41A1	solute carrier family 41 member 1 [Source:HGNC Symbol;Acc:HGNC:19429]
ENSG00000155792	0,50708399	0,42177035	3,55628415	0,09106212	0,35071541	DEPTOR	DEP domain containing MTOR interacting protein [Source:HGNC Symbol;Acc:HGNC:22953]
ENSG00000158423	1,36576095	-1,8002783	3,5527022	0,09120149	0,35071541	RIBC1	RIB43A domain with coiled-coils 1 [Source:HGNC Symbol;Acc:HGNC:26537]
ENSG00000167693	-0,4235001	5,99591324	3,55269288	0,09120185	0,35071541	NXN	nucleoredoxin [Source:HGNC Symbol;Acc:HGNC:18008]
ENSG00000176720	0,29872275	5,5082981	3,55640762	0,09105732	0,35071541	BOK	BCL2 family apoptosis regulator BOK [Source:HGNC Symbol;Acc:HGNC:1087]
ENSG00000092377	0,7036271	-0,1694588	3,55109433	0,09126414	0,35073945	TBL1Y	transducin beta like 1 Y-linked [Source:HGNC Symbol;Acc:HGNC:18502]
ENSG00000133703	0,31532004	3,15079578	3,55177169	0,09123774	0,35073945	KRAS	KRAS proto-oncogene, GTPase [Source:HGNC Symbol;Acc:HGNC:6407]
ENSG00000186205	0,25537911	5,95886134	3,55006845	0,09130414	0,35073945	MTARC1	mitochondrial amidoxime reducing component 1 [Source:HGNC Symbol;Acc:HGNC:26189]
ENSG00000224411	-0,5969176	2,39702952	3,55015201	0,09130088	0,35073945	HSP90AA2P	heat shock protein 90 alpha family class A member 2, pseudogene [Source:HGNC Symbol;Acc:HGNC:5256]
ENSG00000157764	0,28924082	3,02234799	3,54829953	0,09137316	0,35091234	BRAF	B-Raf proto-oncogene, serine/threonine kinase [Source:HGNC Symbol;Acc:HGNC:1097]
ENSG00000164466	0,24534395	6,45404698	3,54444349	0,09152386	0,35121409	SFXN1	sideroflexin 1 [Source:HGNC Symbol;Acc:HGNC:16085]
ENSG00000173327	-0,292412	5,7870667	3,54535642	0,09148815	0,35121409	MAP3K11	mitogen-activated protein kinase kinase kinase 11 [Source:HGNC Symbol;Acc:HGNC:6850]
ENSG00000204194	-0,4182298	1,10812177	3,54450908	0,09152129	0,35121409	RPL12P1	ribosomal protein L12 pseudogene 1 [Source:HGNC Symbol;Acc:HGNC:13976]
ENSG00000121691	0,30652514	6,70268975	3,5385353	0,09175536	0,35164064	CAT	catalase [Source:HGNC Symbol;Acc:HGNC:1516]
ENSG00000152217	1,12113119	-1,2767615	3,5388285	0,09174386	0,35164064	SETBP1	SET binding protein 1 [Source:HGNC Symbol;Acc:HGNC:15573]
ENSG00000166821	0,38187965	3,56304004	3,53874894	0,09174698	0,35164064	PEX11A	peroxisomal biogenesis factor 11 alpha [Source:HGNC Symbol;Acc:HGNC:8852]
ENSG00000233785	-1,1002663	-1,3765467	3,5394514	0,09171942	0,35164064	AC131011.1	novel transcript
ENSG00000236901	0,76774201	0,16803828	3,54070694	0,09167018	0,35164064	MIR600HG	MIR600 host gene [Source:HGNC Symbol;Acc:HGNC:23642]
ENSG00000021852	-0,5176568	1,37880967	3,53393887	0,09193598	0,35206266	C8B	complement C8 beta chain [Source:HGNC Symbol;Acc:HGNC:1353]
ENSG00000122557	0,3188932	3,40512822	3,53298332	0,09197359	0,35206266	HERPUD2	HERPUD family member 2 [Source:HGNC Symbol;Acc:HGNC:21915]
ENSG00000134532	0,33444644	3,13473258	3,53228246	0,09200118	0,35206266	SOX5	SRY-box transcription factor 5 [Source:HGNC Symbol;Acc:HGNC:11201]
ENSG00000136718	-0,2439925	7,15666432	3,53205656	0,09201008	0,35206266	IMP4	IMP U3 small nucleolar ribonucleoprotein 4 [Source:HGNC Symbol;Acc:HGNC:30856]
ENSG00000177854	-0,3839847	2,65931772	3,53402052	0,09193277	0,35206266	TMEM187	transmembrane protein 187 [Source:HGNC Symbol;Acc:HGNC:13705]
ENSG00000232434	-0,4308027	1,15277666	3,53352206	0,09195238	0,35206266	AJM1	apical junction component 1 homolog [Source:HGNC Symbol;Acc:HGNC:37284]
ENSG00000146067	0,28033899	3,75596818	3,53088453	0,09205625	0,35214711	FAM193B	family with sequence similarity 193 member B [Source:HGNC Symbol;Acc:HGNC:25524]
ENSG00000177731	-0,532116	6,92566273	3,52912155	0,09212577	0,35222445	FLII	FLII actin remodeling protein [Source:HGNC Symbol;Acc:HGNC:3750]
ENSG00000205323	0,48838867	0,94611281	3,52918943	0,09212309	0,35222445	SARNP	SAP domain containing ribonucleoprotein [Source:HGNC Symbol;Acc:HGNC:24432]
ENSG00000234690	-0,6495715	-0,3053565	3,52853765	0,0921488	0,35222445	EPCAM-DT	EPCAM divergent transcript [Source:HGNC Symbol;Acc:HGNC:52639]
ENSG00000068394	-0,3940582	4,90419532	3,52685293	0,09221531	0,35229432	GPKOW	G-patch domain and KOW motifs [Source:HGNC Symbol;Acc:HGNC:30677]
ENSG00000277476	0,97476243	-0,3530904	3,5270743	0,09220657	0,35229432	AC005332.5	novel transcript
ENSG00000129354	-0,4092546	5,51736035	3,52609358	0,09224531	0,35230754	AP1M2	adaptor related protein complex 1 subunit mu 2 [Source:HGNC Symbol;Acc:HGNC:558]
ENSG00000214114	0,36205181	3,82959951	3,52554466	0,092267	0,35230754	MYCBP	MYC binding protein [Source:HGNC Symbol;Acc:HGNC:7554]
ENSG00000130332	-0,2857856	6,72941641	3,52439443	0,09231248	0,35238907	LSM7	LSM7 homolog, U6 small nuclear RNA and mRNA degradation associated [Source:HGNC Symbol;Acc:HGNC:20470

ENSG00000099968	-0,2937111	4,21374407	3,51990522	0,09249024	0,35240689	BCL2L13	BCL2 like 13 [Source:HGNC Symbol;Acc:HGNC:17164]
ENSG00000108599	0,33162272	3,30007107	3,5170848	0,09260214	0,35240689	AKAP10	A-kinase anchoring protein 10 [Source:HGNC Symbol;Acc:HGNC:368]
ENSG00000124217	-0,2710898	4,20712229	3,51776936	0,09257496	0,35240689	MOCS3	molybdenum cofactor synthesis 3 [Source:HGNC Symbol;Acc:HGNC:15765]
ENSG00000130255	-0,3121411	8,71205655	3,51840931	0,09254957	0,35240689	RPL36	ribosomal protein L36 [Source:HGNC Symbol;Acc:HGNC:13631]
ENSG00000135097	0,89448945	-1,3574667	3,52034031	0,09247299	0,35240689	MSI1	musashi RNA binding protein 1 [Source:HGNC Symbol;Acc:HGNC:7330]
ENSG00000139631	0,47363914	1,01044168	3,51614669	0,0926394	0,35240689	CSAD	cysteine sulfinic acid decarboxylase [Source:HGNC Symbol;Acc:HGNC:18966]
ENSG00000140332	0,34223889	3,77593841	3,5213203	0,09243416	0,35240689	TLE3	TLE family member 3, transcriptional corepressor [Source:HGNC Symbol;Acc:HGNC:11839]
ENSG00000160688	-0,2715084	5,35886558	3,51626824	0,09263457	0,35240689	FLAD1	flavin adenine dinucleotide synthetase 1 [Source:HGNC Symbol;Acc:HGNC:24671]
ENSG00000172840	0,36231845	3,95682225	3,51817659	0,0925588	0,35240689	PDP2	pyruvate dehyrogenase phosphatase catalytic subunit 2 [Source:HGNC Symbol;Acc:HGNC:30263]
ENSG00000185000	-0,3324164	6,65138756	3,51575752	0,09265486	0,35240689	DGAT1	diacylglycerol O-acyltransferase 1 [Source:HGNC Symbol;Acc:HGNC:2843]
ENSG00000198169	0,29780611	3,13600167	3,51724543	0,09259576	0,35240689	ZNF251	zinc finger protein 251 [Source:HGNC Symbol;Acc:HGNC:13045]
ENSG00000215251	0,2747885	6,06528888	3,51793888	0,09256824	0,35240689	FASTKD5	FAST kinase domains 5 [Source:HGNC Symbol;Acc:HGNC:25790]
ENSG00000228496	0,68614181	-0,2695076	3,52320391	0,09235958	0,35240689	AC106875.1	uncharacterized LOC100506405 [Source:NCBI gene (formerly Entrezgene);Acc:100506405]
ENSG00000233184	-0,503209	0,31106721	3,52111338	0,09244236	0,35240689	AC093157.1	novel transcript
ENSG00000005100	0,34378453	5,33203351	3,509991	0,09288435	0,35263419	DHX33	DEAH-box helicase 33 [Source:HGNC Symbol;Acc:HGNC:16718]
ENSG00000110906	0,27209958	4,79005416	3,51216255	0,09279785	0,35263419	KCTD10	potassium channel tetramerization domain containing 10 [Source:HGNC Symbol;Acc:HGNC:23236]
ENSG00000119698	0,33968993	2,96915997	3,51109014	0,09284055	0,35263419	PPP4R4	protein phosphatase 4 regulatory subunit 4 [Source:HGNC Symbol;Acc:HGNC:23788]
ENSG00000137760	0,32297416	2,62052027	3,50944178	0,09290625	0,35263419	ALKBH8	alkB homolog 8, tRNA methyltransferase [Source:HGNC Symbol;Acc:HGNC:25189]
ENSG00000144674	-0,5205404	5,83380226	3,51033388	0,09287069	0,35263419	GOLGA4	golgin A4 [Source:HGNC Symbol;Acc:HGNC:4427]
ENSG00000164089	-0,3941509	1,33730048	3,50940466	0,09290773	0,35263419	ETNPPL	ethanolamine-phosphate phospho-lyase [Source:HGNC Symbol;Acc:HGNC:14404]
ENSG00000171224	-0,3281517	2,21160715	3,50999322	0,09288426	0,35263419	FAM241B	family with sequence similarity 241 member B [Source:HGNC Symbol;Acc:HGNC:23519]
ENSG00000214593	-0,5299294	0,72631354	3,50970577	0,09289572	0,35263419	AL354710.1	ribosomal protein S10 (RPS10) pseudogene
ENSG00000083937	-0,2956538	4,42174994	3,50531153	0,09307111	0,35315442	CHMP2B	charged multivesicular body protein 2B [Source:HGNC Symbol;Acc:HGNC:24537]
ENSG00000242299	-0,3021705	3,92241846	3,50476037	0,09309314	0,35315442	AC073861.1	ribosomal protein S18 (RPS18) pseudogene
ENSG00000099617	-0,3471787	4,3059932	3,49862451	0,09333882	0,3538972	EFNA2	ephrin A2 [Source:HGNC Symbol;Acc:HGNC:3222]
ENSG00000111846	-0,2808831	4,88890153	3,4970244	0,09340303	0,3538972	GCNT2	glucosaminyl (N-acetyl) transferase 2 (I blood group) [Source:HGNC Symbol;Acc:HGNC:4204]
ENSG00000116752	0,26175219	5,62870136	3,49768703	0,09337643	0,3538972	BCAS2	BCAS2 pre-mRNA processing factor [Source:HGNC Symbol;Acc:HGNC:975]
ENSG00000188994	0,42776297	2,29241264	3,4973968	0,09338808	0,3538972	ZNF292	zinc finger protein 292 [Source:HGNC Symbol;Acc:HGNC:18410]
ENSG00000254332	-0,8974807	-1,6837237	3,49684912	0,09341006	0,3538972	AF201337.1	tropomyosin 4 (TPM4) pseudogene
ENSG00000180488	0,37162574	2,0305913	3,49535509	0,09347007	0,35403272	MIGA1	mitoguardin 1 [Source:HGNC Symbol;Acc:HGNC:24741]
ENSG00000131844	0,35850354	7,23222166	3,49333848	0,09355113	0,35424793	MCCC2	methylcrotonoyl-CoA carboxylase 2 [Source:HGNC Symbol;Acc:HGNC:6937]
ENSG00000171453	0,27684172	6,02586527	3,49214969	0,09359897	0,35433721	POLR1C	RNA polymerase I and III subunit C [Source:HGNC Symbol;Acc:HGNC:20194]
ENSG00000181929	0,28287134	4,76939533	3,49032156	0,09367258	0,35450274	PRKAG1	protein kinase AMP-activated non-catalytic subunit gamma 1 [Source:HGNC Symbol;Acc:HGNC:9385]
ENSG00000225177	-0,7367394	0,20697143	3,48985902	0,09369122	0,35450274	AL590617.2	novel transcript
ENSG00000053254	0,41617219	4,08945407	3,48868608	0,09373851	0,35458981	FOXN3	forkhead box N3 [Source:HGNC Symbol;Acc:HGNC:1928]
ENSG00000144840	-0,2933534	3,99823518	3,48534142	0,0938735	0,35491667	RABL3	RAB, member of RAS oncogene family like 3 [Source:HGNC Symbol;Acc:HGNC:18072]
ENSG00000174206	0,39060048	2,61714371	3,48569323	0,09385929	0,35491667	C12orf66	chromosome 12 open reading frame 66 [Source:HGNC Symbol;Acc:HGNC:26517]
ENSG00000155903	0,46233367	1,63903776	3,48321007	0,09395966	0,35515048	RASA2	RAS p21 protein activator 2 [Source:HGNC Symbol;Acc:HGNC:9872]
ENSG00000126970	0,32694469	2,98352303	3,48087408	0,0940542	0,35529661	ZC4H2	zinc finger C4H2-type containing [Source:HGNC Symbol;Acc:HGNC:24931]
ENSG00000137720	-0,2839732	3,63740496	3,48099025	0,09404949	0,35529661	C11orf1	chromosome 11 open reading frame 1 [Source:HGNC Symbol;Acc:HGNC:1163]
ENSG00000139239	-0,2759444	4,02055553	3,47985209	0,0940956	0,35529661	RPL14P1	ribosomal protein L14 pseudogene 1 [Source:HGNC Symbol;Acc:HGNC:31384]
ENSG00000167755	-0,7938045	0,83824696	3,480082	0,09408628	0,35529661	KLK6	kallikrein related peptidase 6 [Source:HGNC Symbol;Acc:HGNC:6367]
ENSG00000275718	-0,5159459	1,02854296	3,47845473	0,09415224	0,35541863	CCL15	C-C motif chemokine ligand 15 [Source:HGNC Symbol;Acc:HGNC:10613]
ENSG00000285184	-0,6862584	-0,5761715	3,47775784	0,09418051	0,35543348	AC244033.2	novel transcript

ENSG00000175768	-0,3282564	3,26188023	3,47704939	0,09420925	0,35545014	TOMM5	translocase of outer mitochondrial membrane 5 [Source:HGNC Symbol;Acc:HGNC:31369]
ENSG00000070831	-0,3375031	6,92582218	3,47488768	0,09429703	0,35564637	CDC42	cell division cycle 42 [Source:HGNC Symbol;Acc:HGNC:1736]
ENSG00000071082	-0,3084716	10,138176	3,47409787	0,09432913	0,35564637	RPL31	ribosomal protein L31 [Source:HGNC Symbol;Acc:HGNC:10334]
ENSG00000092841	-0,3069865	8,10454491	3,47337199	0,09435864	0,35564637	MYL6	myosin light chain 6 [Source:HGNC Symbol;Acc:HGNC:7587]
ENSG00000186104	-1,2478921	-1,6698563	3,47353572	0,09435198	0,35564637	CYP2R1	cytochrome P450 family 2 subfamily R member 1 [Source:HGNC Symbol;Acc:HGNC:20580]
ENSG00000144306	-0,3862708	1,96974665	3,46925865	0,09452609	0,35618562	SCRN3	secernin 3 [Source:HGNC Symbol;Acc:HGNC:30382]
ENSG00000133858	0,39160649	2,60501674	3,4677078	0,09458932	0,35625009	ZFC3H1	zinc finger C3H1-type containing [Source:HGNC Symbol;Acc:HGNC:28328]
ENSG00000232333	1,02444744	-1,0735786	3,4673275	0,09460484	0,35625009	RPS27AP2	RPS27A pseudogene 2 [Source:HGNC Symbol;Acc:HGNC:16572]
ENSG00000272462	0,56210266	0,49248742	3,46704517	0,09461636	0,35625009	U91328.1	novel transcript
ENSG00000177350	-0,4502645	0,88991571	3,46185719	0,09482835	0,35686434	RPL13AP3	ribosomal protein L13a pseudogene 3 [Source:HGNC Symbol;Acc:HGNC:23539]
ENSG00000237476	-0,9476241	-1,813553	3,46190645	0,09482634	0,35686434	LINC01637	long intergenic non-protein coding RNA 1637 [Source:HGNC Symbol;Acc:HGNC:52424]
ENSG00000140391	0,29369185	8,04189869	3,46099471	0,09486365	0,35690525	TSPAN3	tetraspanin 3 [Source:HGNC Symbol;Acc:HGNC:17752]
ENSG00000095932	0,36592102	6,7797173	3,45617429	0,09506126	0,35741402	SMIM24	small integral membrane protein 24 [Source:HGNC Symbol;Acc:HGNC:37244]
ENSG00000126368	-0,3305309	3,86951749	3,4564875	0,09504841	0,35741402	NR1D1	nuclear receptor subfamily 1 group D member 1 [Source:HGNC Symbol;Acc:HGNC:7962]
ENSG00000140497	0,35985321	7,34072001	3,45471452	0,09512121	0,35741402	SCAMP2	secretory carrier membrane protein 2 [Source:HGNC Symbol;Acc:HGNC:10564]
ENSG00000221838	-0,3114285	3,13208875	3,45484558	0,09511582	0,35741402	AP4M1	adaptor related protein complex 4 subunit mu 1 [Source:HGNC Symbol;Acc:HGNC:574]
ENSG00000226525	-0,2831529	3,71655917	3,45556706	0,09508619	0,35741402	RPS7P10	ribosomal protein S7 pseudogene 10 [Source:HGNC Symbol;Acc:HGNC:39903]
ENSG00000134574	-0,2630184	5,56093003	3,44982157	0,09532249	0,35807822	DDB2	damage specific DNA binding protein 2 [Source:HGNC Symbol;Acc:HGNC:2718]
ENSG00000166441	-0,2982602	10,5038447	3,44773401	0,09540853	0,35825552	RPL27A	ribosomal protein L27a [Source:HGNC Symbol;Acc:HGNC:10329]
ENSG00000188707	0,29448098	5,23101933	3,44690369	0,09544277	0,35825552	ZBED6CL	ZBED6 C-terminal like [Source:HGNC Symbol;Acc:HGNC:21720]
ENSG00000251550	-0,5898572	-0,0866206	3,4468921	0,09544325	0,35825552	AC091133.5	COX assembly mitochondrial protein homolog (S. cerevisiae) (CMC1) pseudogene
ENSG00000075240	-0,3246108	3,63698482	3,44440898	0,09554577	0,3583641	GRAMD4	GRAM domain containing 4 [Source:HGNC Symbol;Acc:HGNC:29113]
ENSG00000187566	-1,1821468	-1,7908091	3,44531582	0,09550832	0,3583641	NHLRC1	NHL repeat containing E3 ubiquitin protein ligase 1 [Source:HGNC Symbol;Acc:HGNC:21576]
ENSG00000204713	-0,2492899	6,79298612	3,44470984	0,09553334	0,3583641	TRIM27	tripartite motif containing 27 [Source:HGNC Symbol;Acc:HGNC:9975]
ENSG00000107371	-0,4291133	4,4168379	3,44331866	0,09559083	0,35844108	EXOSC3	exosome component 3 [Source:HGNC Symbol;Acc:HGNC:17944]
ENSG00000171161	-0,3079438	4,06672534	3,44247952	0,09562553	0,35847917	ZNF672	zinc finger protein 672 [Source:HGNC Symbol;Acc:HGNC:26179]
ENSG00000137145	0,41478821	3,45366479	3,43776033	0,09582096	0,35902753	DENND4C	DENN domain containing 4C [Source:HGNC Symbol;Acc:HGNC:26079]
ENSG00000198824	0,32484638	4,26968476	3,4381077	0,09580655	0,35902753	CHAMP1	chromosome alignment maintaining phosphoprotein 1 [Source:HGNC Symbol;Acc:HGNC:20311]
ENSG00000110435	0,35984429	6,46304287	3,43643581	0,0958759	0,35904006	PDHX	pyruvate dehydrogenase complex component X [Source:HGNC Symbol;Acc:HGNC:21350]
ENSG00000123636	0,69322508	0,92933973	3,43609665	0,09588997	0,35904006	BAZ2B	bromodomain adjacent to zinc finger domain 2B [Source:HGNC Symbol;Acc:HGNC:963]
ENSG00000147509	-0,8458042	-0,7850209	3,43590253	0,09589803	0,35904006	RGS20	regulator of G protein signaling 20 [Source:HGNC Symbol;Acc:HGNC:14600]
ENSG00000117519	-0,2912275	7,60272902	3,43103818	0,09610021	0,35915271	CNN3	calponin 3 [Source:HGNC Symbol;Acc:HGNC:2157]
ENSG00000117862	-0,2912768	4,47872902	3,43296288	0,09602015	0,35915271	TXNDC12	thioredoxin domain containing 12 [Source:HGNC Symbol;Acc:HGNC:24626]
ENSG00000122490	0,27514598	5,36883769	3,43220281	0,09605175	0,35915271	SLC66A2	solute carrier family 66 member 2 [Source:HGNC Symbol;Acc:HGNC:26188]
ENSG00000157106	0,56291384	5,21348672	3,43197772	0,09606112	0,35915271	SMG1	SMG1 nonsense mediated mRNA decay associated PI3K related kinase [Source:HGNC Symbol;Acc:HGNC:30045]
ENSG00000175215	0,3074262	5,65315941	3,43259039	0,09603563	0,35915271	CTDSP2	CTD small phosphatase 2 [Source:HGNC Symbol;Acc:HGNC:17077]
ENSG00000184205	-0,6888492	3,60932151	3,43189209	0,09606468	0,35915271	TSPYL2	TSPY like 2 [Source:HGNC Symbol;Acc:HGNC:24358]
ENSG00000270344	0,72205545	-0,7836982	3,43108258	0,09609836	0,35915271	POC1B-AS1	POC1B antisense RNA 1 [Source:HGNC Symbol;Acc:HGNC:52949]
ENSG00000009780	-0,2966983	2,70358304	3,42714204	0,09626253	0,35937352	FAM76A	family with sequence similarity 76 member A [Source:HGNC Symbol;Acc:HGNC:28530]
ENSG00000050405	0,42502676	3,71142982	3,42772223	0,09623834	0,35937352	LIMA1	LIM domain and actin binding 1 [Source:HGNC Symbol;Acc:HGNC:24636]
ENSG00000111912	0,33957473	3,61435031	3,42640594	0,09629324	0,35937352	NCOA7	nuclear receptor coactivator 7 [Source:HGNC Symbol;Acc:HGNC:21081]
ENSG00000137563	0,26232033	7,77734309	3,42872115	0,0961967	0,35937352	GGH	gamma-glutamyl hydrolase [Source:HGNC Symbol;Acc:HGNC:4248]
ENSG00000137880	-0,41332	5,01278338	3,4260789	0,09630689	0,35937352	GCHFR	GTP cyclohydrolase I feedback regulator [Source:HGNC Symbol;Acc:HGNC:4194]
ENSG00000237973	0,27192207	4,49529693	3,42725565	0,0962578	0,35937352	MTCO1P12	MT-CO1 pseudogene 12 [Source:HGNC Symbol;Acc:HGNC:52014]

ENSG00000274641	-1,287413	-1,5430156	3,42530396	0,09633924	0,35940242	H2BC17	H2B clustered histone 17 [Source:HGNC Symbol;Acc:HGNC:4758]
ENSG00000118689	0,37608773	3,57928179	3,42455354	0,09637057	0,35942754	FOXO3	forkhead box O3 [Source:HGNC Symbol;Acc:HGNC:3821]
ENSG00000101146	0,27223203	6,99477624	3,42224211	0,09646718	0,35943381	RAE1	ribonucleic acid export 1 [Source:HGNC Symbol;Acc:HGNC:9828]
ENSG00000118579	0,27923357	6,78714155	3,42232948	0,09646352	0,35943381	MED28	mediator complex subunit 28 [Source:HGNC Symbol;Acc:HGNC:24628]
ENSG00000148841	-0,5066389	4,0314232	3,42222913	0,09646772	0,35943381	ITPRIP	inositol 1,4,5-trisphosphate receptor interacting protein [Source:HGNC Symbol;Acc:HGNC:29370]
ENSG00000234327	-0,8293505	-0,2789104	3,42215868	0,09647067	0,35943381	ZNF232-AS1	ZNF232 antisense RNA 1 [Source:HGNC Symbol;Acc:HGNC:40623]
ENSG00000072121	0,30723346	4,07293269	3,42050304	0,09653994	0,3596002	ZFYVE26	zinc finger FYVE-type containing 26 [Source:HGNC Symbol;Acc:HGNC:20761]
ENSG00000104983	-0,3599524	2,10839075	3,41787576	0,09665	0,35991839	CCDC61	coiled-coil domain containing 61 [Source:HGNC Symbol;Acc:HGNC:33629]
ENSG00000134871	-0,8358421	-0,5839174	3,41705391	0,09668446	0,35995497	COL4A2	collagen type IV alpha 2 chain [Source:HGNC Symbol;Acc:HGNC:2203]
ENSG00000136895	-0,4157325	0,97573577	3,41576652	0,09673847	0,3600643	GARNL3	GTPase activating Rap/RanGAP domain like 3 [Source:HGNC Symbol;Acc:HGNC:25425]
ENSG00000165092	0,35483487	8,39470517	3,41489861	0,09677491	0,36010816	ALDH1A1	aldehyde dehydrogenase 1 family member A1 [Source:HGNC Symbol;Acc:HGNC:402]
ENSG00000260260	-0,3742018	2,80875192	3,4127493	0,09686521	0,3603524	SNHG19	small nucleolar RNA host gene 19 [Source:HGNC Symbol;Acc:HGNC:49574]
ENSG00000113522	0,68583738	0,55976908	3,411652	0,09691136	0,36038967	RAD50	RAD50 double strand break repair protein [Source:HGNC Symbol;Acc:HGNC:9816]
ENSG00000213569	0,78444474	-1,265549	3,41133792	0,09692457	0,36038967	GTF3C6P2	GTF3C6 pseudogene 2 [Source:HGNC Symbol;Acc:HGNC:55024]
ENSG00000114107	0,31100902	2,53783411	3,41071548	0,09695076	0,36039534	CEP70	centrosomal protein 70 [Source:HGNC Symbol;Acc:HGNC:29972]
ENSG00000118655	0,37766472	3,40489633	3,40845925	0,09704578	0,36040386	DCLRE1B	DNA cross-link repair 1B [Source:HGNC Symbol;Acc:HGNC:17641]
ENSG00000138640	0,52571541	0,46322367	3,40936833	0,09700748	0,36040386	FAM13A	family with sequence similarity 13 member A [Source:HGNC Symbol;Acc:HGNC:19367]
ENSG00000164134	-0,3800538	4,83261783	3,40831791	0,09705174	0,36040386	NAA15	N-alpha-acetyltransferase 15, NatA auxiliary subunit [Source:HGNC Symbol;Acc:HGNC:30782]
ENSG00000233276	-0,3314896	7,00264973	3,40990355	0,09698494	0,36040386	GPX1	glutathione peroxidase 1 [Source:HGNC Symbol;Acc:HGNC:4553]
ENSG00000105072	-0,2920124	3,02827338	3,40753683	0,09708466	0,36043451	C19orf44	chromosome 19 open reading frame 44 [Source:HGNC Symbol;Acc:HGNC:26141]
ENSG00000138246	0,33462805	4,30605905	3,40653254	0,09712701	0,36050013	DNAJC13	DnaJ heat shock protein family (Hsp40) member C13 [Source:HGNC Symbol;Acc:HGNC:30343]
ENSG00000230461	-0,9637446	-0,8254952	3,405741	0,09716041	0,36053249	PROX1-AS1	PROX1 antisense RNA 1 [Source:HGNC Symbol;Acc:HGNC:43656]
ENSG00000062822	-0,4376848	4,55326558	3,40168217	0,0973319	0,36107711	POLD1	DNA polymerase delta 1, catalytic subunit [Source:HGNC Symbol;Acc:HGNC:9175]
ENSG00000123843	0,29700348	7,47183256	3,39928264	0,09743346	0,3610884	C4BPB	complement component 4 binding protein beta [Source:HGNC Symbol;Acc:HGNC:1328]
ENSG00000125398	0,30799782	6,58868713	3,39835235	0,09747287	0,3610884	SOX9	SRY-box transcription factor 9 [Source:HGNC Symbol;Acc:HGNC:11204]
ENSG00000138095	0,34820195	7,96733684	3,39810762	0,09748324	0,3610884	LRPPRC	leucine rich pentatricopeptide repeat containing [Source:HGNC Symbol;Acc:HGNC:15714]
ENSG00000138107	0,26967316	7,2139571	3,39884306	0,09745208	0,3610884	ACTR1A	actin related protein 1A [Source:HGNC Symbol;Acc:HGNC:167]
ENSG00000154655	0,35603656	3,16556774	3,39816821	0,09748067	0,3610884	L3MBTL4	L3MBTL histone methyl-lysine binding protein 4 [Source:HGNC Symbol;Acc:HGNC:26677]
ENSG00000224043	-0,603379	0,56323231	3,40017814	0,09739554	0,3610884	CCNT2-AS1	CCNT2 antisense RNA 1 [Source:HGNC Symbol;Acc:HGNC:40130]
ENSG00000120992	-0,3005606	5,18017249	3,39746602	0,09751044	0,36109758	LYPLA1	lysophospholipase 1 [Source:HGNC Symbol;Acc:HGNC:6737]
ENSG00000161813	-0,3269733	5,15443413	3,39591955	0,09757603	0,36124891	LARP4	La ribonucleoprotein 4 [Source:HGNC Symbol;Acc:HGNC:24320]
ENSG00000120727	0,32621151	5,62851778	3,39244651	0,09772354	0,36142865	PAIP2	poly(A) binding protein interacting protein 2 [Source:HGNC Symbol;Acc:HGNC:17970]
ENSG00000142657	0,29865879	8,93311108	3,39393031	0,09766048	0,36142865	PGD	phosphogluconate dehydrogenase [Source:HGNC Symbol;Acc:HGNC:8891]
ENSG00000146574	0,273588	4,3549728	3,39310113	0,09769571	0,36142865	CCZ1B	CCZ1 homolog B, vacuolar protein trafficking and biogenesis associated [Source:HGNC Symbol;Acc:HGNC:21717]
ENSG00000261824	-0,4011937	1,81558753	3,39266636	0,09771419	0,36142865	LINC00662	long intergenic non-protein coding RNA 662 [Source:HGNC Symbol;Acc:HGNC:27122]
ENSG00000038358	0,44854175	5,02864945	3,39127962	0,09777316	0,36142919	EDC4	enhancer of mRNA decapping 4 [Source:HGNC Symbol;Acc:HGNC:17157]
ENSG00000183726	0,31598766	6,59470213	3,39171341	0,09775471	0,36142919	TMEM50A	transmembrane protein 50A [Source:HGNC Symbol;Acc:HGNC:30590]
ENSG00000100478	0,43985852	2,08470989	3,39026536	0,09781632	0,36149726	AP4S1	adaptor related protein complex 4 subunit sigma 1 [Source:HGNC Symbol;Acc:HGNC:575]
ENSG00000170043	-0,257203	6,32773923	3,3890499	0,09786808	0,36150562	TRAPPC1	trafficking protein particle complex 1 [Source:HGNC Symbol;Acc:HGNC:19894]
ENSG00000198715	0,28404645	7,80094443	3,38937943	0,09785404	0,36150562	GLMP	glycosylated lysosomal membrane protein [Source:HGNC Symbol;Acc:HGNC:29436]
ENSG00000171163	-0,2816782	3,8260589	3,38776336	0,09792289	0,36161668	ZNF692	zinc finger protein 692 [Source:HGNC Symbol;Acc:HGNC:26049]
ENSG00000146410	0,3797985	3,78357861	3,38650727	0,09797645	0,36163163	MTFR2	mitochondrial fission regulator 2 [Source:HGNC Symbol;Acc:HGNC:21115]
ENSG00000230356	-0,3351302	3,02121071	3,38690877	0,09795933	0,36163163	NCAPD2P1	non-SMC condensin I complex subunit D2 pseudogene 1 [Source:HGNC Symbol;Acc:HGNC:43831]
ENSG00000204954	-0,2935367	3,90374398	3,38544907	0,0980216	0,36170689	C12orf73	chromosome 12 open reading frame 73 [Source:HGNC Symbol;Acc:HGNC:34450]

ENSG00000075643	-0,3223005	5,26013961	3,38308278	0,09812266	0,36179012	MOCOS	molybdenum cofactor sulfurase [Source:HGNC Symbol;Acc:HGNC:18234]
ENSG00000132359	0,27274052	3,7925898	3,38344887	0,09810702	0,36179012	RAP1GAP2	RAP1 GTPase activating protein 2 [Source:HGNC Symbol;Acc:HGNC:29176]
ENSG00000163738	-0,3295614	2,86029668	3,3826018	0,09814322	0,36179012	MTHFD2L	methylenetetrahydrofolate dehydrogenase (NADP+ dependent) 2 like [Source:HGNC Symbol;Acc:HGNC:31865]
ENSG00000218748	-0,8649648	-0,6635367	3,38392953	0,09808648	0,36179012	DBIP1	DBI pseudogene 1 [Source:HGNC Symbol;Acc:HGNC:2692]
ENSG00000134755	0,32099386	3,9871017	3,38081197	0,09821976	0,36183728	DSC2	desmocollin 2 [Source:HGNC Symbol;Acc:HGNC:3036]
ENSG00000160813	-0,2868913	4,46526264	3,38056535	0,09823031	0,36183728	PPP1R35	protein phosphatase 1 regulatory subunit 35 [Source:HGNC Symbol;Acc:HGNC:28320]
ENSG00000265666	-1,0664985	-1,2975412	3,38123224	0,09820178	0,36183728	RARA-AS1	RARA antisense RNA 1 [Source:HGNC Symbol;Acc:HGNC:49577]
ENSG00000118960	-0,2917127	5,50660895	3,37839702	0,09832316	0,36197943	HS1BP3	HCLS1 binding protein 3 [Source:HGNC Symbol;Acc:HGNC:24979]
ENSG00000158792	0,32497217	4,79037945	3,3787375	0,09830858	0,36197943	SPATA2L	spermatogenesis associated 2 like [Source:HGNC Symbol;Acc:HGNC:28393]
ENSG00000160094	0,32152927	3,53145467	3,37567422	0,09843991	0,36197943	ZNF362	zinc finger protein 362 [Source:HGNC Symbol;Acc:HGNC:18079]
ENSG00000163930	-0,2789445	5,59449164	3,37571963	0,09843796	0,36197943	BAP1	BRCA1 associated protein 1 [Source:HGNC Symbol;Acc:HGNC:950]
ENSG00000179886	-0,3078115	2,97057518	3,37641587	0,09840809	0,36197943	TIGD5	tigger transposable element derived 5 [Source:HGNC Symbol;Acc:HGNC:18336]
ENSG00000242276	-0,5716238	-0,0791209	3,37561737	0,09844235	0,36197943	RPL5P3	ribosomal protein L5 pseudogene 3 [Source:HGNC Symbol;Acc:HGNC:31034]
ENSG00000254166	-0,3605372	3,85652098	3,37762011	0,09835646	0,36197943	CASC19	cancer susceptibility 19 [Source:HGNC Symbol;Acc:HGNC:49476]
ENSG00000173852	0,2851737	4,17502262	3,37417251	0,09850438	0,36211636	DPY19L1	dpy-19 like C-mannosyltransferase 1 [Source:HGNC Symbol;Acc:HGNC:22205]
ENSG00000176624	0,30095326	3,72934418	3,37333137	0,09854051	0,36215805	MEX3C	mex-3 RNA binding family member C [Source:HGNC Symbol;Acc:HGNC:28040]
ENSG00000005421	-0,7620653	-0,4029555	3,3723965	0,09858069	0,3621593	PON1	paraoxonase 1 [Source:HGNC Symbol;Acc:HGNC:9204]
ENSG00000198746	0,25508512	5,37654541	3,37216991	0,09859043	0,3621593	GPATCH3	G-patch domain containing 3 [Source:HGNC Symbol;Acc:HGNC:25720]
ENSG00000114982	0,29737834	5,41115687	3,36842992	0,09875139	0,36229509	KANSL3	KAT8 regulatory NSL complex subunit 3 [Source:HGNC Symbol;Acc:HGNC:25473]
ENSG00000137693	0,26726486	5,75274546	3,36859097	0,09874445	0,36229509	YAP1	Yes1 associated transcriptional regulator [Source:HGNC Symbol;Acc:HGNC:16262]
ENSG00000143324	0,30464299	4,10100587	3,36913121	0,09872118	0,36229509	XPR1	xenotropic and polytropic retrovirus receptor 1 [Source:HGNC Symbol;Acc:HGNC:12827]
ENSG00000161677	-0,7399428	0,79032838	3,37024975	0,09867302	0,36229509	JOSD2	Josephin domain containing 2 [Source:HGNC Symbol;Acc:HGNC:28853]
ENSG00000204685	0,46451553	0,98979983	3,36979058	0,09869279	0,36229509	STARD7-AS1	STARD7 antisense RNA 1 [Source:HGNC Symbol;Acc:HGNC:40827]
ENSG00000130856	0,46376521	1,4807344	3,36772075	0,09878195	0,36230137	ZNF236	zinc finger protein 236 [Source:HGNC Symbol;Acc:HGNC:13028]
ENSG00000172292	0,3169538	4,9994688	3,36723928	0,0988027	0,36230137	CERS6	ceramide synthase 6 [Source:HGNC Symbol;Acc:HGNC:23826]
ENSG00000162623	0,29622224	4,86728168	3,36627523	0,09884427	0,36236286	TYW3	tRNA-yW synthesizing protein 3 homolog [Source:HGNC Symbol;Acc:HGNC:24757]
ENSG00000089693	-0,4208915	6,65227383	3,36561778	0,09887264	0,36237591	MLF2	myeloid leukemia factor 2 [Source:HGNC Symbol;Acc:HGNC:7126]
ENSG00000157483	-0,3658617	4,50420299	3,3649647	0,09890082	0,3623883	MYO1E	myosin IE [Source:HGNC Symbol;Acc:HGNC:7599]
ENSG00000100100	0,42888401	1,04793431	3,36395916	0,09894424	0,36245647	PIK3IP1	phosphoinositide-3-kinase interacting protein 1 [Source:HGNC Symbol;Acc:HGNC:24942]
ENSG00000183172	-0,3114283	4,57588193	3,36212175	0,09902364	0,36265639	SMDT1	single-pass membrane protein with aspartate rich tail 1 [Source:HGNC Symbol;Acc:HGNC:25055]
ENSG00000105341	-0,2413965	5,93848528	3,35942128	0,09914048	0,36275078	DMAC2	distal membrane arm assembly complex 2 [Source:HGNC Symbol;Acc:HGNC:25496]
ENSG00000115568	0,32389073	4,17013198	3,35939473	0,09914163	0,36275078	ZNF142	zinc finger protein 142 [Source:HGNC Symbol;Acc:HGNC:12927]
ENSG00000197622	-0,2907229	3,26315477	3,35923062	0,09914873	0,36275078	CDC42SE1	CDC42 small effector 1 [Source:HGNC Symbol;Acc:HGNC:17719]
ENSG00000213366	0,39392611	1,36175876	3,35937307	0,09914257	0,36275078	GSTM2	glutathione S-transferase mu 2 [Source:HGNC Symbol;Acc:HGNC:4634]
ENSG00000067225	-0,4006251	10,1249527	3,354688	0,09934569	0,36277956	PKM	pyruvate kinase M1/2 [Source:HGNC Symbol;Acc:HGNC:9021]
ENSG00000120029	0,30690977	4,34080716	3,35601358	0,09928817	0,36277956	ARMH3	armadillo like helical domain containing 3 [Source:HGNC Symbol;Acc:HGNC:25788]
ENSG00000126457	-0,2367521	8,59265499	3,35413797	0,09936957	0,36277956	PRMT1	protein arginine methyltransferase 1 [Source:HGNC Symbol;Acc:HGNC:5187]
ENSG00000169241	-0,2770777	5,1015343	3,35520375	0,09932331	0,36277956	SLC50A1	solute carrier family 50 member 1 [Source:HGNC Symbol;Acc:HGNC:30657]
ENSG00000170545	0,28272243	3,60424282	3,35490883	0,09933611	0,36277956	SMAGP	small cell adhesion glycoprotein [Source:HGNC Symbol;Acc:HGNC:26918]
ENSG00000188873	-0,4669247	0,7045727	3,35403024	0,09937425	0,36277956	RPL10AP2	ribosomal protein L10a pseudogene 2 [Source:HGNC Symbol;Acc:HGNC:31081]
ENSG00000198885	-0,2879466	3,69468835	3,35544971	0,09931263	0,36277956	ITPRIPL1	ITPRIP like 1 [Source:HGNC Symbol;Acc:HGNC:29371]
ENSG00000230364	0,87008013	-0,3451652	3,35389573	0,0993801	0,36277956	RPL4P3	ribosomal protein L4 pseudogene 3 [Source:HGNC Symbol;Acc:HGNC:35773]
ENSG00000240087	-0,3280385	2,01845679	3,35845673	0,09918225	0,36277956	RPSAP12	ribosomal protein SA pseudogene 12 [Source:HGNC Symbol;Acc:HGNC:6504]
ENSG00000166716	0,30067799	4,22068952	3,35235215	0,09944716	0,36293371	ZNF592	zinc finger protein 592 [Source:HGNC Symbol;Acc:HGNC:28986]

ENSG00000106153	-0,2804606	9,06503444	3,34641061	0,09970587	0,36375058	CHCHD2	coiled-coil-helix-coiled-coil-helix domain containing 2 [Source:HGNC Symbol;Acc:HGNC:21645]
ENSG00000125753	-0,2631648	7,37270624	3,34254368	0,0998747	0,36375058	VASP	vasodilator stimulated phosphoprotein [Source:HGNC Symbol;Acc:HGNC:12652]
ENSG00000130544	0,41310432	2,11481125	3,34594073	0,09972636	0,36375058	ZNF557	zinc finger protein 557 [Source:HGNC Symbol;Acc:HGNC:28632]
ENSG00000132664	0,28042219	4,85040855	3,34150773	0,09991999	0,36375058	POLR3F	RNA polymerase III subunit F [Source:HGNC Symbol;Acc:HGNC:15763]
ENSG00000146083	0,29274703	4,28660427	3,34244421	0,09987904	0,36375058	RNF44	ring finger protein 44 [Source:HGNC Symbol;Acc:HGNC:19180]
ENSG00000151116	-0,3210999	2,39714224	3,34168023	0,09991244	0,36375058	UEVLD	UEV and lactate/malate dehyrogenase domains [Source:HGNC Symbol;Acc:HGNC:30866]
ENSG00000156970	0,36826817	4,88041242	3,34310417	0,0998502	0,36375058	BUB1B	BUB1 mitotic checkpoint serine/threonine kinase B [Source:HGNC Symbol;Acc:HGNC:1149]
ENSG00000160360	-0,6011424	0,08801376	3,34160338	0,0999158	0,36375058	GPSM1	G protein signaling modulator 1 [Source:HGNC Symbol;Acc:HGNC:17858]
ENSG00000184014	-0,2686783	5,25574751	3,34470923	0,0997801	0,36375058	DENND5A	DENN domain containing 5A [Source:HGNC Symbol;Acc:HGNC:19344]
ENSG00000198862	-0,3320068	3,09800481	3,34301817	0,09985396	0,36375058	LTN1	listerin E3 ubiquitin protein ligase 1 [Source:HGNC Symbol;Acc:HGNC:13082]
ENSG00000138138	0,27909805	5,68630303	3,34068623	0,09995592	0,36379074	ATAD1	ATPase family AAA domain containing 1 [Source:HGNC Symbol;Acc:HGNC:25903]
ENSG00000101849	0,27460152	4,21085247	3,33974679	0,09999703	0,36384973	TBL1X	transducin beta like 1 X-linked [Source:HGNC Symbol;Acc:HGNC:11585]
ENSG00000091428	1,36436169	-1,6267362	3,33438584	0,10023206	0,36452332	RAPGEF4	Rap guanine nucleotide exchange factor 4 [Source:HGNC Symbol;Acc:HGNC:16626]
ENSG00000152234	0,36121072	10,2767622	3,33453699	0,10022542	0,36452332	ATP5F1A	ATP synthase F1 subunit alpha [Source:HGNC Symbol;Acc:HGNC:823]
ENSG00000177508	0,29973104	4,33520381	3,33368098	0,10026301	0,36454514	IRX3	iroquois homeobox 3 [Source:HGNC Symbol;Acc:HGNC:14360]
ENSG00000234040	-0,8422561	-1,4289845	3,33306645	0,10029001	0,36455257	RPL10P12	ribosomal protein L10 pseudogene 12 [Source:HGNC Symbol;Acc:HGNC:52345]
ENSG00000102034	-0,3916617	1,33911169	3,33227226	0,10032491	0,36456895	ELF4	E74 like ETS transcription factor 4 [Source:HGNC Symbol;Acc:HGNC:3319]
ENSG00000160746	0,29690418	4,42544925	3,33182837	0,10034443	0,36456895	ANO10	anoctamin 10 [Source:HGNC Symbol;Acc:HGNC:25519]
ENSG00000135916	0,26484878	7,12515413	3,33088631	0,10038586	0,3646288	ITM2C	integral membrane protein 2C [Source:HGNC Symbol;Acc:HGNC:6175]
ENSG00000111727	0,48654749	1,6001406	3,32967124	0,10043933	0,3646513	HCFC2	host cell factor C2 [Source:HGNC Symbol;Acc:HGNC:24972]
ENSG00000228205	-0,3253956	2,05356523	3,32961118	0,10044198	0,3646513	AC131235.1	ribosomal protein S3 (RPS3) pseudogene
ENSG00000145439	-0,2796243	3,77708848	3,3280365	0,10051133	0,36469296	CBR4	carbonyl reductase 4 [Source:HGNC Symbol;Acc:HGNC:25891]
ENSG00000204611	0,34341481	2,70564351	3,32849517	0,10049112	0,36469296	ZNF616	zinc finger protein 616 [Source:HGNC Symbol;Acc:HGNC:28062]
ENSG00000225715	-0,8818992	-1,3458911	3,32765038	0,10052834	0,36469296	PSMA1P1	PSMA1 pseudogene 1 [Source:HGNC Symbol;Acc:HGNC:55003]
ENSG00000112715	-0,2459126	6,23245795	3,32482217	0,10065308	0,36505484	VEGFA	vascular endothelial growth factor A [Source:HGNC Symbol;Acc:HGNC:12680]
ENSG00000175147	-0,542895	0,3697744	3,32375986	0,10069999	0,36513431	TMEM51-AS1	TMEM51 antisense RNA 1 [Source:HGNC Symbol;Acc:HGNC:26301]
ENSG00000170364	-0,2781889	3,48740489	3,32235701	0,10076197	0,36513827	SETMAR	SET domain and mariner transposase fusion gene [Source:HGNC Symbol;Acc:HGNC:10762]
ENSG00000196504	-0,3710697	6,49636747	3,32203824	0,10077606	0,36513827	PRPF40A	pre-mRNA processing factor 40 homolog A [Source:HGNC Symbol;Acc:HGNC:16463]
ENSG00000234949	-1,2349866	-1,821829	3,32217639	0,10076996	0,36513827	AC104667.2	novel transcript, antisense to RAB17
ENSG00000074842	-0,2504406	9,14662679	3,32096475	0,10082354	0,3651604	MYDGF	myeloid derived growth factor [Source:HGNC Symbol;Acc:HGNC:16948]
ENSG00000164105	0,33818952	3,88535585	3,32076978	0,10083216	0,3651604	SAP30	Sin3A associated protein 30 [Source:HGNC Symbol;Acc:HGNC:10532]
ENSG00000118217	0,32603486	6,29398903	3,3198804	0,10087152	0,3652124	ATF6	activating transcription factor 6 [Source:HGNC Symbol;Acc:HGNC:791]
ENSG00000070950	0,31176602	3,91505516	3,31752161	0,10097599	0,3654136	RAD18	RAD18 E3 ubiquitin protein ligase [Source:HGNC Symbol;Acc:HGNC:18278]
ENSG00000232024	0,61052141	-0,3508988	3,31749626	0,10097712	0,3654136	LSM12P1	LSM12 pseudogene 1 [Source:HGNC Symbol;Acc:HGNC:32453]
ENSG00000100601	0,27517576	4,17219952	3,31692421	0,10100248	0,36541485	ALKBH1	alkB homolog 1, histone H2A dioxygenase [Source:HGNC Symbol;Acc:HGNC:17911]
ENSG00000106266	-0,2734299	5,5113279	3,31600942	0,10104304	0,36547111	SNX8	sorting nexin 8 [Source:HGNC Symbol;Acc:HGNC:14972]
ENSG00000180581	-0,65305	-0,11467	3,31411823	0,10112698	0,36559367	SRP9P1	signal recognition particle 9 pseudogene 1 [Source:HGNC Symbol;Acc:HGNC:23402]
ENSG00000196365	-0,4260474	7,197711	3,31431013	0,10111846	0,36559367	LONP1	lon peptidase 1, mitochondrial [Source:HGNC Symbol;Acc:HGNC:9479]
ENSG00000124789	0,33503154	5,73988012	3,31305974	0,10117399	0,36566281	NUP153	nucleoporin 153 [Source:HGNC Symbol;Acc:HGNC:8062]
ENSG00000182985	0,27555632	6,11954439	3,31256089	0,10119616	0,36566281	CADM1	cell adhesion molecule 1 [Source:HGNC Symbol;Acc:HGNC:5951]
ENSG00000115762	0,29490888	7,16945797	3,31172653	0,10123325	0,36570638	PLEKHB2	pleckstrin homology domain containing B2 [Source:HGNC Symbol;Acc:HGNC:19236]
ENSG00000103160	0,31878553	5,38463264	3,30970748	0,10132308	0,36594039	HSDL1	hydroxysteroid dehydrogenase like 1 [Source:HGNC Symbol;Acc:HGNC:16475]
ENSG00000067082	-0,3073436	6,09216033	3,30875797	0,10136536	0,36598245	KLF6	Kruppel like factor 6 [Source:HGNC Symbol;Acc:HGNC:2235]
ENSG00000123983	0,33025048	9,56402759	3,30803852	0,10139741	0,36598245	ACSL3	acyl-CoA synthetase long chain family member 3 [Source:HGNC Symbol;Acc:HGNC:3570]

ENSG00000185404	0,34015337	3,62717369	3,30775861	0,10140988	0,36598245	SP140L	SP140 nuclear body protein like [Source:HGNC Symbol;Acc:HGNC:25105]
ENSG00000095139	0,34297748	6,72649642	3,3067199	0,10145618	0,36605912	ARCN1	archain 1 [Source:HGNC Symbol;Acc:HGNC:649]
ENSG00000198130	0,27425763	5,6846713	3,30575863	0,10149906	0,36612339	HIBCH	3-hydroxyisobutyryl-CoA hydrolase [Source:HGNC Symbol;Acc:HGNC:4908]
ENSG00000106443	-0,3290682	5,30308506	3,30432454	0,10156306	0,36617344	PHF14	PHD finger protein 14 [Source:HGNC Symbol;Acc:HGNC:22203]
ENSG00000164068	-0,3000994	5,21538735	3,30451762	0,10155444	0,36617344	RNF123	ring finger protein 123 [Source:HGNC Symbol;Acc:HGNC:21148]
ENSG00000103995	-0,4787226	1,83054085	3,30018064	0,1017483	0,36630593	CEP152	centrosomal protein 152 [Source:HGNC Symbol;Acc:HGNC:29298]
ENSG00000141298	0,3395226	2,57586836	3,30253918	0,10164282	0,36630593	SSH2	slingshot protein phosphatase 2 [Source:HGNC Symbol;Acc:HGNC:30580]
ENSG00000146038	0,25453201	5,78039921	3,2990168	0,1018004	0,36630593	DCDC2	doublecortin domain containing 2 [Source:HGNC Symbol;Acc:HGNC:18141]
ENSG00000156795	0,32638826	4,73179656	3,29911539	0,10179599	0,36630593	NTAQ1	N-terminal glutamine amidase 1 [Source:HGNC Symbol;Acc:HGNC:25490]
ENSG00000164171	-0,7230581	5,33059836	3,29997216	0,10175763	0,36630593	ITGA2	integrin subunit alpha 2 [Source:HGNC Symbol;Acc:HGNC:6137]
ENSG00000178381	-0,2573226	6,78181128	3,29921342	0,1017916	0,36630593	ZFAND2A	zinc finger AN1-type containing 2A [Source:HGNC Symbol;Acc:HGNC:28073]
ENSG00000233954	-0,3593127	4,03570309	3,29927412	0,10178888	0,36630593	UQCRHL	ubiquinol-cytochrome c reductase hinge protein like [Source:HGNC Symbol;Acc:HGNC:51714]
ENSG00000251169	-0,5276903	0,61431854	3,29955438	0,10177633	0,36630593	LINC01843	long intergenic non-protein coding RNA 1843 [Source:HGNC Symbol;Acc:HGNC:52659]
ENSG00000102316	0,32272795	7,6010812	3,29733331	0,10187583	0,36648706	MAGED2	MAGE family member D2 [Source:HGNC Symbol;Acc:HGNC:16353]
ENSG00000280303	-1,3595784	-1,990733	3,29358917	0,10204383	0,36700107	ERICD	E2F1-regulated inhibitor of cell death [Source:HGNC Symbol;Acc:HGNC:49404]
ENSG00000122912	-0,308531	3,06382903	3,29257369	0,10208946	0,36700378	SLC25A16	solute carrier family 25 member 16 [Source:HGNC Symbol;Acc:HGNC:10986]
ENSG00000287710	-0,7123473	0,45638476	3,2924542	0,10209483	0,36700378	AC067852.5	novel transcript, antisense to PSMC3IP
ENSG00000245248	-0,3476675	1,88851666	3,28989644	0,10220987	0,36732695	USP2-AS1	USP2 antisense RNA 1 (head to head) [Source:HGNC Symbol;Acc:HGNC:48673]
ENSG00000164414	0,35193562	2,94787987	3,28923325	0,10223973	0,36734388	SLC35A1	solute carrier family 35 member A1 [Source:HGNC Symbol;Acc:HGNC:11021]
ENSG00000109670	0,29004196	2,84758427	3,28643709	0,10236574	0,36770618	FBXW7	F-box and WD repeat domain containing 7 [Source:HGNC Symbol;Acc:HGNC:16712]
ENSG00000060656	-0,3065778	5,59648729	3,28523232	0,10242009	0,36781098	PTPRU	protein tyrosine phosphatase receptor type U [Source:HGNC Symbol;Acc:HGNC:9683]
ENSG00000141002	0,25505839	6,32800713	3,28448208	0,10245395	0,36784217	TCF25	transcription factor 25 [Source:HGNC Symbol;Acc:HGNC:29181]
ENSG00000124198	0,40629094	5,46822167	3,28092414	0,10261475	0,36832896	ARFGEF2	ADP ribosylation factor guanine nucleotide exchange factor 2 [Source:HGNC Symbol;Acc:HGNC:15853]
ENSG00000111077	-0,4480334	3,80200645	3,27943893	0,10268197	0,36834341	TNS2	tensin 2 [Source:HGNC Symbol;Acc:HGNC:19737]
ENSG00000112276	0,95520254	-1,5037938	3,27919653	0,10269294	0,36834341	BVES	blood vessel epicardial substance [Source:HGNC Symbol;Acc:HGNC:1152]
ENSG00000165591	0,41413878	1,08479652	3,27799803	0,10274723	0,36834341	FAAH2	fatty acid amide hydrolase 2 [Source:HGNC Symbol;Acc:HGNC:26440]
ENSG00000183431	-0,3833032	7,14113962	3,27911943	0,10269643	0,36834341	SF3A3	splicing factor 3a subunit 3 [Source:HGNC Symbol;Acc:HGNC:10767]
ENSG00000228665	-0,6180837	-0,2014789	3,27797835	0,10274812	0,36834341	BX679664.1	ribosomal protein S27 (metallopanstimulin 1) (RPS27) pseudogene
ENSG00000266962	-0,2967393	4,35323294	3,27749438	0,10277006	0,36834341	AC067852.2	novel transcript, antisense to HSD17B1
ENSG00000167977	0,24824116	5,2911424	3,2756221	0,10285496	0,3683766	KCTD5	potassium channel tetramerization domain containing 5 [Source:HGNC Symbol;Acc:HGNC:21423]
ENSG00000196741	-0,6151473	0,23303352	3,27585572	0,10284436	0,3683766	LINC01560	long intergenic non-protein coding RNA 1560 [Source:HGNC Symbol;Acc:HGNC:27333]
ENSG00000241852	-0,682003	0,2999088	3,27665773	0,10280799	0,3683766	C8orf58	chromosome 8 open reading frame 58 [Source:HGNC Symbol;Acc:HGNC:32233]
ENSG00000059728	-0,2934286	3,08002235	3,27069543	0,10307881	0,36854586	MXD1	MAX dimerization protein 1 [Source:HGNC Symbol;Acc:HGNC:6761]
ENSG00000099994	-0,6506528	4,95397672	3,27102673	0,10306374	0,36854586	SUSD2	sushi domain containing 2 [Source:HGNC Symbol;Acc:HGNC:30667]
ENSG00000118271	0,23560685	9,80739889	3,27117441	0,10305702	0,36854586	TTR	transthyretin [Source:HGNC Symbol;Acc:HGNC:12405]
ENSG00000123815	-0,3525822	3,99309627	3,27345197	0,10295349	0,36854586	COQ8B	coenzyme Q8B [Source:HGNC Symbol;Acc:HGNC:19041]
ENSG00000198060	0,30245701	5,32467827	3,27363473	0,10294519	0,36854586	MARCHF5	membrane associated ring-CH-type finger 5 [Source:HGNC Symbol;Acc:HGNC:26025]
ENSG00000214049	-1,0882928	-1,8944263	3,27102962	0,10306361	0,36854586	UCA1	urothelial cancer associated 1 [Source:HGNC Symbol;Acc:HGNC:37126]
ENSG00000224861	-0,2828524	3,12187305	3,27251747	0,10299595	0,36854586	YBX1P1	Y-box binding protein 1 pseudogene 1 [Source:HGNC Symbol;Acc:HGNC:8015]
ENSG00000119227	-0,3929135	3,13684845	3,26882276	0,10316406	0,36876041	PIGZ	phosphatidylinositol glycan anchor biosynthesis class Z [Source:HGNC Symbol;Acc:HGNC:30596]
ENSG00000160963	0,40250254	4,64818417	3,26699007	0,10324758	0,36887843	COL26A1	collagen type XXVI alpha 1 chain [Source:HGNC Symbol;Acc:HGNC:18038]
ENSG00000284194	-0,265129	5,22474414	3,26745257	0,1032265	0,36887843	SCO2	synthesis of cytochrome C oxidase 2 [Source:HGNC Symbol;Acc:HGNC:10604]
ENSG00000125375	-0,3050353	3,68527867	3,26503016	0,10333699	0,36892719	DMAC2L	distal membrane arm assembly complex 2 like [Source:HGNC Symbol;Acc:HGNC:18799]
ENSG00000160469	0,78998332	-0,7268131	3,26551444	0,10331489	0,36892719	BRSK1	BR serine/threonine kinase 1 [Source:HGNC Symbol;Acc:HGNC:18994]

ENSG00000253251	0,49060512	0,66432722	3,26573708	0,10330473	0,36892719	SHLD3	shieldin complex subunit 3 [Source:HGNC Symbol;Acc:HGNC:53826]
ENSG00000117791	-0,2970082	2,78038478	3,26342518	0,10341028	0,36899321	MTARC2	mitochondrial amidoxime reducing component 2 [Source:HGNC Symbol;Acc:HGNC:26064]
ENSG00000156928	0,24693585	6,01278891	3,263407	0,10341111	0,36899321	MALSU1	mitochondrial assembly of ribosomal large subunit 1 [Source:HGNC Symbol;Acc:HGNC:21721]
ENSG00000185009	0,26695129	5,12191936	3,26296616	0,10343125	0,36899321	AP3M1	adaptor related protein complex 3 subunit mu 1 [Source:HGNC Symbol;Acc:HGNC:569]
ENSG00000133895	0,24440987	6,06379525	3,26022237	0,10355674	0,36935067	MEN1	menin 1 [Source:HGNC Symbol;Acc:HGNC:7010]
ENSG00000113595	0,43105694	3,00540016	3,25907038	0,10360948	0,36942955	TRIM23	tripartite motif containing 23 [Source:HGNC Symbol;Acc:HGNC:660]
ENSG00000176915	0,27004071	6,11440913	3,25863487	0,10362943	0,36942955	ANKLE2	ankyrin repeat and LEM domain containing 2 [Source:HGNC Symbol;Acc:HGNC:29101]
ENSG00000123191	0,37645615	6,10249927	3,25686711	0,10371045	0,36943035	ATP7B	ATPase copper transporting beta [Source:HGNC Symbol;Acc:HGNC:870]
ENSG00000130382	0,28261754	4,83438424	3,25544608	0,10377563	0,36943035	MLLT1	MLLT1 super elongation complex subunit [Source:HGNC Symbol;Acc:HGNC:7134]
ENSG00000137821	0,81989337	-0,7140654	3,2557841	0,10376012	0,36943035	LRRC49	leucine rich repeat containing 49 [Source:HGNC Symbol;Acc:HGNC:25965]
ENSG00000143702	0,41722124	3,14820152	3,2553209	0,10378138	0,36943035	CEP170	centrosomal protein 170 [Source:HGNC Symbol;Acc:HGNC:28920]
ENSG00000168899	-0,2954702	4,10918881	3,25559182	0,10376894	0,36943035	VAMP5	vesicle associated membrane protein 5 [Source:HGNC Symbol;Acc:HGNC:12646]
ENSG00000262712	0,71408219	0,4125944	3,2578594	0,10366496	0,36943035	AC012676.1	novel transcript
ENSG00000126088	-0,2658442	7,28630432	3,25391622	0,10384587	0,36956987	UROD	uroporphyrinogen decarboxylase [Source:HGNC Symbol;Acc:HGNC:12591]
ENSG00000101447	0,25303978	5,76242543	3,25207458	0,1039305	0,36969094	FAM83D	family with sequence similarity 83 member D [Source:HGNC Symbol;Acc:HGNC:16122]
ENSG00000108946	-0,3537025	6,65895036	3,25209302	0,10392965	0,36969094	PRKAR1A	protein kinase cAMP-dependent type I regulatory subunit alpha [Source:HGNC Symbol;Acc:HGNC:9388]
ENSG00000072274	0,38673657	8,15756927	3,24968863	0,10404028	0,36999134	TFRC	transferrin receptor [Source:HGNC Symbol;Acc:HGNC:11763]
ENSG00000174951	1,05280151	-1,7414504	3,24829962	0,10410426	0,37012876	FUT1	fucosyltransferase 1 (H blood group) [Source:HGNC Symbol;Acc:HGNC:4012]
ENSG00000090060	-0,2683053	5,82986533	3,24738665	0,10414633	0,37018827	PAPOLA	poly(A) polymerase alpha [Source:HGNC Symbol;Acc:HGNC:14981]
ENSG00000160678	-0,3302911	5,98889563	3,24401995	0,10430169	0,37065029	S100A1	S100 calcium binding protein A1 [Source:HGNC Symbol;Acc:HGNC:10486]
ENSG00000169016	0,27569582	5,25261826	3,24311718	0,10434339	0,37070833	E2F6	E2F transcription factor 6 [Source:HGNC Symbol;Acc:HGNC:3120]
ENSG00000161999	-0,5168771	0,37901612	3,24063015	0,1044584	0,37102671	JMJD8	jumonji domain containing 8 [Source:HGNC Symbol;Acc:HGNC:14148]
ENSG00000138606	-0,2551904	4,74311139	3,23852144	0,10455605	0,37112763	SHF	Src homology 2 domain containing F [Source:HGNC Symbol;Acc:HGNC:25116]
ENSG00000161642	-0,5118756	1,09343662	3,23881986	0,10454222	0,37112763	ZNF385A	zinc finger protein 385A [Source:HGNC Symbol;Acc:HGNC:17521]
ENSG00000236480	-0,6524257	-0,5932079	3,23797281	0,10458147	0,37112763	PKMP1	pyruvate kinase M1/2 pseudogene 1 [Source:HGNC Symbol;Acc:HGNC:44243]
ENSG00000236735	-0,6441967	-0,5658602	3,2378225	0,10458844	0,37112763	RPL31P63	ribosomal protein L31 pseudogene 63 [Source:HGNC Symbol;Acc:HGNC:35666]
ENSG00000274070	0,44073194	3,67606954	3,23700916	0,10462614	0,37117128	CASTOR2	cytosolic arginine sensor for mTORC1 subunit 2 [Source:HGNC Symbol;Acc:HGNC:37073]
ENSG00000012211	-0,3475716	2,28031041	3,23171623	0,10487195	0,37168046	PRICKLE3	prickle planar cell polarity protein 3 [Source:HGNC Symbol;Acc:HGNC:6645]
ENSG00000090061	0,31373895	4,54927843	3,23118029	0,10489688	0,37168046	CCNK	cyclin K [Source:HGNC Symbol;Acc:HGNC:1596]
ENSG00000123453	-0,3114591	5,33992051	3,23220041	0,10484943	0,37168046	SARDH	sarcosine dehydrogenase [Source:HGNC Symbol;Acc:HGNC:10536]
ENSG00000161011	-0,4162305	8,53668468	3,23180248	0,10486794	0,37168046	SQSTM1	sequestosome 1 [Source:HGNC Symbol;Acc:HGNC:11280]
ENSG00000198056	0,29307639	3,80661508	3,23255914	0,10483276	0,37168046	PRIM1	DNA primase subunit 1 [Source:HGNC Symbol;Acc:HGNC:9369]
ENSG00000134086	0,3537638	4,6352281	3,22988276	0,10495727	0,37171412	VHL	von Hippel-Lindau tumor suppressor [Source:HGNC Symbol;Acc:HGNC:12687]
ENSG00000141376	0,34732713	3,28487108	3,22993166	0,10495499	0,37171412	BCAS3	BCAS3 microtubule associated cell migration factor [Source:HGNC Symbol;Acc:HGNC:14347]
ENSG00000025800	0,53811727	5,21518889	3,22742343	0,10507185	0,3718638	KPNA6	karyopherin subunit alpha 6 [Source:HGNC Symbol;Acc:HGNC:6399]
ENSG00000111786	-0,2505081	7,62060227	3,22733671	0,1050759	0,3718638	SRSF9	serine and arginine rich splicing factor 9 [Source:HGNC Symbol;Acc:HGNC:10791]
ENSG00000124383	0,27984162	6,69553618	3,22797681	0,10504606	0,3718638	MPHOSPH10	M-phase phosphoprotein 10 [Source:HGNC Symbol;Acc:HGNC:7213]
ENSG00000236432	-0,6932911	-0,0933092	3,22387791	0,10523732	0,37234489	MFF-DT	MFF divergent transcript [Source:HGNC Symbol;Acc:HGNC:41067]
ENSG00000107021	0,41495251	4,49631457	3,22255335	0,10529922	0,37241533	TBC1D13	TBC1 domain family member 13 [Source:HGNC Symbol;Acc:HGNC:25571]
ENSG00000120738	-0,7157931	3,93939166	3,22072615	0,10538469	0,37241533	EGR1	early growth response 1 [Source:HGNC Symbol;Acc:HGNC:3238]
ENSG00000131037	-0,4761844	0,48393058	3,22211796	0,10531958	0,37241533	EPS8L1	EPS8 like 1 [Source:HGNC Symbol;Acc:HGNC:21295]
ENSG00000132300	0,28295175	7,57686203	3,22114581	0,10536506	0,37241533	PTCD3	pentatricopeptide repeat domain 3 [Source:HGNC Symbol;Acc:HGNC:24717]
ENSG00000221867	0,6450449	-0,2084066	3,22174438	0,10533705	0,37241533	MAGEA3	MAGE family member A3 [Source:HGNC Symbol;Acc:HGNC:6801]
ENSG00000137285	-0,3571149	3,84085252	3,21831131	0,10549779	0,37272483	TUBB2B	tubulin beta 2B class IIb [Source:HGNC Symbol;Acc:HGNC:30829]

ENSG00000145192	0,31116023	12,8994429	3,21340319	0,10572812	0,37344829	AHSG	alpha 2-HS glycoprotein [Source:HGNC Symbol;Acc:HGNC:349]
ENSG00000172809	-0,2724475	9,42346003	3,21271369	0,10576053	0,37347247	RPL38	ribosomal protein L38 [Source:HGNC Symbol;Acc:HGNC:10349]
ENSG00000101751	0,38468522	2,42018572	3,21134268	0,10582501	0,37349503	POLI	DNA polymerase iota [Source:HGNC Symbol;Acc:HGNC:9182]
ENSG00000102575	0,46112516	1,67922317	3,21171127	0,10580767	0,37349503	ACP5	acid phosphatase 5, tartrate resistant [Source:HGNC Symbol;Acc:HGNC:124]
ENSG00000124535	0,24537609	6,55791876	3,21040408	0,10586918	0,37349503	WRNIP1	WRN helicase interacting protein 1 [Source:HGNC Symbol;Acc:HGNC:20876]
ENSG00000171345	-0,4474865	7,99255419	3,21086135	0,10584766	0,37349503	KRT19	keratin 19 [Source:HGNC Symbol;Acc:HGNC:6436]
ENSG00000205339	0,26317933	5,98847009	3,20963476	0,10590541	0,37353262	IPO7	importin 7 [Source:HGNC Symbol;Acc:HGNC:9852]
ENSG00000171943	-0,5712133	0,69185981	3,20720267	0,10602003	0,37378893	SRGAP2C	SLIT-ROBO Rho GTPase activating protein 2C [Source:HGNC Symbol;Acc:HGNC:30584]
ENSG00000183735	0,29823394	4,57330599	3,2070071	0,10602925	0,37378893	TBK1	TANK binding kinase 1 [Source:HGNC Symbol;Acc:HGNC:11584]
ENSG00000090857	0,59945836	4,37712043	3,20347682	0,10619593	0,3742862	PDPR	pyruvate dehydrogenase phosphatase regulatory subunit [Source:HGNC Symbol;Acc:HGNC:30264]
ENSG00000005059	0,33047671	2,98202305	3,20276433	0,1062296	0,37431459	MCUB	mitochondrial calcium uniporter dominant negative subunit beta [Source:HGNC Symbol;Acc:HGNC:26076]
ENSG00000106852	-0,8592692	-0,5585418	3,19829806	0,10644103	0,37439375	LHX6	LIM homeobox 6 [Source:HGNC Symbol;Acc:HGNC:21735]
ENSG00000114857	0,6439153	4,25723209	3,19957134	0,1063807	0,37439375	NKTR	natural killer cell triggering receptor [Source:HGNC Symbol;Acc:HGNC:7833]
ENSG00000134962	0,37081384	6,13801119	3,19999736	0,10636053	0,37439375	KLB	klotho beta [Source:HGNC Symbol;Acc:HGNC:15527]
ENSG00000149532	0,27041386	5,82926262	3,19828152	0,10644182	0,37439375	CPSF7	cleavage and polyadenylation specific factor 7 [Source:HGNC Symbol;Acc:HGNC:30098]
ENSG00000158604	-0,2551811	7,11888245	3,20076601	0,10632414	0,37439375	TMED4	transmembrane p24 trafficking protein 4 [Source:HGNC Symbol;Acc:HGNC:22301]
ENSG00000166313	-0,4814288	0,73068446	3,2001738	0,10635217	0,37439375	APBB1	amyloid beta precursor protein binding family B member 1 [Source:HGNC Symbol;Acc:HGNC:581]
ENSG00000173273	0,34690177	3,85636108	3,1998745	0,10636635	0,37439375	TNKS	tankyrase [Source:HGNC Symbol;Acc:HGNC:11941]
ENSG00000230989	-0,3119588	6,55830438	3,19795937	0,10645709	0,37439375	HSBP1	heat shock factor binding protein 1 [Source:HGNC Symbol;Acc:HGNC:5203]
ENSG00000105497	0,87217423	-0,9345187	3,19281367	0,1067014	0,37448911	ZNF175	zinc finger protein 175 [Source:HGNC Symbol;Acc:HGNC:12964]
ENSG00000117408	-0,2954156	4,67325107	3,19260375	0,10671138	0,37448911	IPO13	importin 13 [Source:HGNC Symbol;Acc:HGNC:16853]
ENSG00000160888	-0,3395036	5,40624313	3,19554969	0,10657141	0,37448911	IER2	immediate early response 2 [Source:HGNC Symbol;Acc:HGNC:28871]
ENSG00000167733	-0,4763501	0,85454196	3,19252946	0,10671491	0,37448911	HSD11B1L	hydroxysteroid 11-beta dehydrogenase 1 like [Source:HGNC Symbol;Acc:HGNC:30419]
ENSG00000167904	-0,3212615	4,1388175	3,19389595	0,10664995	0,37448911	TMEM68	transmembrane protein 68 [Source:HGNC Symbol;Acc:HGNC:26510]
ENSG00000171840	-0,6001206	-0,1812647	3,19342149	0,1066725	0,37448911	NINJ2	ninjurin 2 [Source:HGNC Symbol;Acc:HGNC:7825]
ENSG00000198496	0,39295804	1,89234929	3,1944419	0,10662401	0,37448911	NBR2	neighbor of BRCA1 lncRNA 2 [Source:HGNC Symbol;Acc:HGNC:20691]
ENSG00000203914	-0,7546221	-0,6929749	3,19313709	0,10668602	0,37448911	HSP90B3P	heat shock protein 90 beta family member 3, pseudogene [Source:HGNC Symbol;Acc:HGNC:12100]
ENSG00000214922	1,20164245	-1,1527548	3,19524373	0,10658593	0,37448911	HLA-F-AS1	HLA-F antisense RNA 1 [Source:HGNC Symbol;Acc:HGNC:26645]
ENSG00000101353	-0,3512819	1,77734458	3,18969393	0,10684986	0,37478263	MROH8	maestro heat like repeat family member 8 [Source:HGNC Symbol;Acc:HGNC:16125]
ENSG00000180098	-0,3394276	4,42192961	3,19018172	0,10682663	0,37478263	TRNAU1AP	tRNA selenocysteine 1 associated protein 1 [Source:HGNC Symbol;Acc:HGNC:30813]
ENSG00000143842	0,25996671	4,22012736	3,1859618	0,10702782	0,37523339	SOX13	SRY-box transcription factor 13 [Source:HGNC Symbol;Acc:HGNC:11192]
ENSG00000184368	0,37467991	3,75914211	3,18592153	0,10702974	0,37523339	MAP7D2	MAP7 domain containing 2 [Source:HGNC Symbol;Acc:HGNC:25899]
ENSG00000072210	0,23600537	7,29022561	3,18404249	0,10711949	0,37533808	ALDH3A2	aldehyde dehydrogenase 3 family member A2 [Source:HGNC Symbol;Acc:HGNC:403]
ENSG00000140006	-0,3103583	3,1149226	3,18474745	0,10708581	0,37533808	WDR89	WD repeat domain 89 [Source:HGNC Symbol;Acc:HGNC:20489]
ENSG00000204305	-0,5082312	0,31886168	3,18368266	0,10713668	0,37533808	AGER	advanced glycosylation end-product specific receptor [Source:HGNC Symbol;Acc:HGNC:320]
ENSG00000087253	0,29700536	4,17820504	3,1830729	0,10716583	0,37535018	LPCAT2	lysophosphatidylcholine acyltransferase 2 [Source:HGNC Symbol;Acc:HGNC:26032]
ENSG00000241061	-0,374545	1,69911664	3,18215203	0,10720987	0,37541443	RPL5P1	ribosomal protein L5 pseudogene 1 [Source:HGNC Symbol;Acc:HGNC:10361]
ENSG00000156873	-0,35389	5,45763258	3,1791902	0,10735167	0,375461	PHKG2	phosphorylase kinase catalytic subunit gamma 2 [Source:HGNC Symbol;Acc:HGNC:8931]
ENSG00000169100	-0,2500943	9,51990159	3,17934938	0,10734405	0,375461	SLC25A6	solute carrier family 25 member 6 [Source:HGNC Symbol;Acc:HGNC:10992]
ENSG00000176623	0,24779656	5,60012876	3,18092779	0,10726845	0,375461	RMDN1	regulator of microtubule dynamics 1 [Source:HGNC Symbol;Acc:HGNC:24285]
ENSG00000178464	-0,2655344	3,52706512	3,17937363	0,10734288	0,375461	RPL10P16	ribosomal protein L10 pseudogene 16 [Source:HGNC Symbol;Acc:HGNC:36882]
ENSG00000198780	0,32686661	4,76763354	3,18030628	0,10729821	0,375461	FAM169A	family with sequence similarity 169 member A [Source:HGNC Symbol;Acc:HGNC:29138]
ENSG00000157240	0,5138104	0,42154227	3,17630033	0,10749026	0,37567589	FZD1	frizzled class receptor 1 [Source:HGNC Symbol;Acc:HGNC:4038]
ENSG00000223916	-0,4524777	0,5818955	3,17707775	0,10745296	0,37567589	AC097638.1	ribosomal protein L18a (RPL18A) pseudogene

ENSG00000260916	-0,5522213	1,53032442	3,1763183	0,1074894	0,37567589	CCPG1	cell cycle progression 1 [Source:HGNC Symbol;Acc:HGNC:24227]
ENSG00000070423	-0,2583059	5,72580927	3,17269924	0,10766328	0,37584126	RNF126	ring finger protein 126 [Source:HGNC Symbol;Acc:HGNC:21151]
ENSG00000095787	0,29610502	4,73267525	3,173969	0,10760223	0,37584126	WAC	WW domain containing adaptor with coiled-coil [Source:HGNC Symbol;Acc:HGNC:17327]
ENSG00000133983	-0,5366938	0,7621099	3,17198978	0,10769741	0,37584126	COX16	cytochrome c oxidase assembly factor COX16 [Source:HGNC Symbol;Acc:HGNC:20213]
ENSG00000136933	-0,3232716	6,22830729	3,17156879	0,10771767	0,37584126	RABEPK	Rab9 effector protein with kelch motifs [Source:HGNC Symbol;Acc:HGNC:16896]
ENSG00000137074	0,24569889	6,07226465	3,17258998	0,10766853	0,37584126	APTX	aprataxin [Source:HGNC Symbol;Acc:HGNC:15984]
ENSG00000142794	0,50683865	0,58381469	3,17412993	0,1075945	0,37584126	NBPF3	NBPF member 3 [Source:HGNC Symbol;Acc:HGNC:25076]
ENSG00000229320	-0,6693441	0,76888354	3,17197829	0,10769796	0,37584126	KRT8P12	keratin 8 pseudogene 12 [Source:HGNC Symbol;Acc:HGNC:28057]
ENSG00000055163	0,24969604	5,05038096	3,16834257	0,10787307	0,3761942	CYFIP2	cytoplasmic FMR1 interacting protein 2 [Source:HGNC Symbol;Acc:HGNC:13760]
ENSG00000071575	-0,9834491	-0,9929095	3,16524022	0,10802278	0,3761942	TRIB2	tribbles pseudokinase 2 [Source:HGNC Symbol;Acc:HGNC:30809]
ENSG00000095564	0,32003221	3,78525195	3,16270157	0,10814549	0,3761942	BTAF1	B-TFIID TATA-box binding protein associated factor 1 [Source:HGNC Symbol;Acc:HGNC:17307]
ENSG00000101255	-0,24136	8,39146791	3,15987474	0,10828233	0,3761942	TRIB3	tribbles pseudokinase 3 [Source:HGNC Symbol;Acc:HGNC:16228]
ENSG00000116497	0,2843876	3,75397504	3,16067617	0,10824352	0,3761942	S100PBP	S100P binding protein [Source:HGNC Symbol;Acc:HGNC:25768]
ENSG00000135047	0,27491179	7,38125789	3,1660454	0,1079839	0,3761942	CTSL	cathepsin L [Source:HGNC Symbol;Acc:HGNC:2537]
ENSG00000143322	0,37850772	2,99474708	3,16670507	0,10795206	0,3761942	ABL2	ABL proto-oncogene 2, non-receptor tyrosine kinase [Source:HGNC Symbol;Acc:HGNC:77]
ENSG00000155508	0,28951685	4,82797752	3,16164111	0,1081968	0,3761942	CNOT8	CCR4-NOT transcription complex subunit 8 [Source:HGNC Symbol;Acc:HGNC:9207]
ENSG00000155858	0,2968164	2,83153854	3,1640915	0,10807829	0,3761942	LSM11	LSM11, U7 small nuclear RNA associated [Source:HGNC Symbol;Acc:HGNC:30860]
ENSG00000169375	0,29552594	4,69467378	3,16225851	0,10816692	0,3761942	SIN3A	SIN3 transcription regulator family member A [Source:HGNC Symbol;Acc:HGNC:19353]
ENSG00000169905	0,27192292	4,35799052	3,16029147	0,10826215	0,3761942	TOR1AIP2	torsin 1A interacting protein 2 [Source:HGNC Symbol;Acc:HGNC:24055]
ENSG00000172500	-0,2401055	6,07688959	3,16297472	0,10813228	0,3761942	FIBP	FGF1 intracellular binding protein [Source:HGNC Symbol;Acc:HGNC:3705]
ENSG00000198755	-0,2411372	10,6518891	3,16710451	0,10793279	0,3761942	RPL10A	ribosomal protein L10a [Source:HGNC Symbol;Acc:HGNC:10299]
ENSG00000204304	-0,243061	5,62914008	3,16228103	0,10816583	0,3761942	PBX2	PBX homeobox 2 [Source:HGNC Symbol;Acc:HGNC:8633]
ENSG00000230251	0,83002034	-0,7796729	3,16117267	0,10821948	0,3761942	PHBP4	prohibitin pseudogene 4 [Source:HGNC Symbol;Acc:HGNC:8916]
ENSG00000272525	-0,493126	0,51625988	3,1675342	0,10791206	0,3761942	AC099522.2	novel transcript
ENSG00000273080	-0,5177784	0,33206563	3,167944	0,10789229	0,3761942	AC009309.1	novel transcript, antisense to IMMT
ENSG00000287825	-0,9172435	-1,2943812	3,16265999	0,1081475	0,3761942	AL035587.2	novel transcript, antisense to CNPY3
ENSG00000205808	0,27881363	4,18027212	3,15795865	0,10837522	0,37642737	PLPP6	phospholipid phosphatase 6 [Source:HGNC Symbol;Acc:HGNC:23682]
ENSG00000187546	1,29948282	-1,5219303	3,15684596	0,1084292	0,37652536	AGMO	alkylglycerol monooxygenase [Source:HGNC Symbol;Acc:HGNC:33784]
ENSG00000173933	0,31154553	2,41201106	3,15320676	0,108606	0,37704969	RBM4	RNA binding motif protein 4 [Source:HGNC Symbol;Acc:HGNC:9901]
ENSG00000170234	0,33130519	3,05954753	3,15218083	0,10865591	0,37713334	PWWP2A	PWWP domain containing 2A [Source:HGNC Symbol;Acc:HGNC:29406]
ENSG00000257605	-1,3346526	-1,2721208	3,15156484	0,10868589	0,37714779	MYG1-AS1	MYG1 antisense RNA 1 [Source:HGNC Symbol;Acc:HGNC:54810]
ENSG00000173275	0,50698137	0,50337911	3,1503686	0,10874414	0,37726032	ZNF449	zinc finger protein 449 [Source:HGNC Symbol;Acc:HGNC:21039]
ENSG00000065518	-0,2762137	6,72322161	3,14917893	0,10880211	0,37737182	NDUFB4	NADH:ubiquinone oxidoreductase subunit B4 [Source:HGNC Symbol;Acc:HGNC:7699]
ENSG00000066583	0,33964563	5,93867142	3,14641769	0,10893682	0,37739103	ISOC1	isochorismatase domain containing 1 [Source:HGNC Symbol;Acc:HGNC:24254]
ENSG00000179361	0,36998297	3,90366143	3,14737279	0,1088902	0,37739103	ARID3B	AT-rich interaction domain 3B [Source:HGNC Symbol;Acc:HGNC:14350]
ENSG00000232573	-0,2936098	3,0110269	3,14754381	0,10888186	0,37739103	RPL3P4	ribosomal protein L3 pseudogene 4 [Source:HGNC Symbol;Acc:HGNC:19805]
ENSG00000283390	-0,9069709	-1,9541427	3,14675157	0,10892052	0,37739103	AC068631.3	PET100 homolog (PET100) pseudogene
ENSG00000284024	0,36798708	3,8313109	3,14826273	0,10884679	0,37739103	AC069544.2	zinc finger and SCAN domain containing 29 [Source:NCBI gene (formerly Entrezgene);Acc:100421372]
ENSG00000167244	0,91193105	4,24008135	3,14527637	0,10899256	0,37749462	IGF2	insulin like growth factor 2 [Source:HGNC Symbol;Acc:HGNC:5466]
ENSG00000130813	-0,2509253	4,66533132	3,14350249	0,10907926	0,37770537	SHFL	shiftless antiviral inhibitor of ribosomal frameshifting [Source:HGNC Symbol;Acc:HGNC:25649]
ENSG00000259820	0,88340931	-1,5402128	3,14032097	0,109235	0,37815499	AC083843.2	novel transcript
ENSG00000152104	0,50354731	2,3347338	3,1394764	0,10927638	0,37820864	PTPN14	protein tyrosine phosphatase non-receptor type 14 [Source:HGNC Symbol;Acc:HGNC:9647]
ENSG00000163006	0,31604479	3,37660633	3,13559991	0,10946661	0,37877728	CCDC138	coiled-coil domain containing 138 [Source:HGNC Symbol;Acc:HGNC:26531]
ENSG00000140694	0,31668263	5,67590725	3,13446157	0,10952255	0,37879482	PARN	poly(A)-specific ribonuclease [Source:HGNC Symbol;Acc:HGNC:8609]

ENSG00000243830	-0,9152851	-1,3471302	3,13444151	0,10952353	0,37879482	AC092865.1	ribosomal protein L15 (RPL15) pseudogene
ENSG00000147121	-0,2848885	3,15909402	3,13214271	0,10963662	0,37909617	KRBOX4	KRAB box domain containing 4 [Source:HGNC Symbol;Acc:HGNC:26007]
ENSG00000143126	0,53266285	3,58580044	3,13027064	0,10972881	0,37932519	CELSR2	cadherin EGF LAG seven-pass G-type receptor 2 [Source:HGNC Symbol;Acc:HGNC:3231]
ENSG00000144597	0,26535221	3,78423127	3,12784537	0,10984841	0,37937952	EAF1	ELL associated factor 1 [Source:HGNC Symbol;Acc:HGNC:20907]
ENSG00000159214	-0,3722392	1,30877886	3,12793497	0,10984398	0,37937952	CCDC24	coiled-coil domain containing 24 [Source:HGNC Symbol;Acc:HGNC:28688]
ENSG00000168275	-0,2969194	5,81946134	3,12790199	0,10984561	0,37937952	COA6	cytochrome c oxidase assembly factor 6 [Source:HGNC Symbol;Acc:HGNC:18025]
ENSG00000230202	-0,3164845	2,9111232	3,12871833	0,10980534	0,37937952	AL450405.1	ribosomal protein L29 (RPL29) pseudogene
ENSG00000136859	-0,3942924	1,71911963	3,12636294	0,10992159	0,37954253	ANGPTL2	angiopoietin like 2 [Source:HGNC Symbol;Acc:HGNC:490]
ENSG00000159063	0,29716495	5,8128225	3,12481943	0,10999785	0,37971611	ALG8	ALG8 alpha-1,3-glucosyltransferase [Source:HGNC Symbol;Acc:HGNC:23161]
ENSG00000143314	-0,2483501	5,94448977	3,12329203	0,11007338	0,37988709	MRPL24	mitochondrial ribosomal protein L24 [Source:HGNC Symbol;Acc:HGNC:14037]
ENSG00000091129	0,39111438	3,77733889	3,12199827	0,11013741	0,37992857	NRCAM	neuronal cell adhesion molecule [Source:HGNC Symbol;Acc:HGNC:7994]
ENSG00000227097	-0,3361681	2,89806439	3,12247023	0,11011405	0,37992857	RPS28P7	ribosomal protein S28 pseudogene 7 [Source:HGNC Symbol;Acc:HGNC:35787]
ENSG00000160193	-0,2792273	4,95950484	3,11670538	0,11039987	0,38074402	WDR4	WD repeat domain 4 [Source:HGNC Symbol;Acc:HGNC:12756]
ENSG00000094631	-0,3581047	5,21775412	3,11484072	0,11049252	0,38079377	HDAC6	histone deacetylase 6 [Source:HGNC Symbol;Acc:HGNC:14064]
ENSG00000109390	-0,313775	5,63086798	3,11569341	0,11045014	0,38079377	NDUFC1	NADH:ubiquinone oxidoreductase subunit C1 [Source:HGNC Symbol;Acc:HGNC:7705]
ENSG00000152380	-0,8754224	-0,7949553	3,11379304	0,11054462	0,38079377	FAM151B	family with sequence similarity 151 member B [Source:HGNC Symbol;Acc:HGNC:33716]
ENSG00000182919	-0,2890652	2,56488493	3,11389633	0,11053948	0,38079377	C11orf54	chromosome 11 open reading frame 54 [Source:HGNC Symbol;Acc:HGNC:30204]
ENSG00000255198	-0,7256722	0,14450447	3,11444325	0,11051228	0,38079377	SNHG9	small nucleolar RNA host gene 9 [Source:HGNC Symbol;Acc:HGNC:33102]
ENSG00000114853	-0,6879562	0,2375255	3,1132277	0,11057275	0,38080087	ZBTB47	zinc finger and BTB domain containing 47 [Source:HGNC Symbol;Acc:HGNC:26955]
ENSG00000223501	-0,287102	5,21285529	3,11246504	0,11061071	0,38084182	VPS52	VPS52 subunit of GARP complex [Source:HGNC Symbol;Acc:HGNC:10518]
ENSG00000114423	0,43134113	3,0455241	3,11155496	0,11065603	0,38090808	CBLB	Cbl proto-oncogene B [Source:HGNC Symbol;Acc:HGNC:1542]
ENSG00000229656	-0,9778355	-1,2748091	3,1101428	0,11072639	0,38106051	ITGB1-DT	ITGB1 divergent transcript [Source:HGNC Symbol;Acc:HGNC:53718]
ENSG00000141485	-0,2496452	6,04295448	3,1085201	0,11080732	0,38124921	SLC13A5	solute carrier family 13 member 5 [Source:HGNC Symbol;Acc:HGNC:23089]
ENSG00000125872	0,66770358	-0,6172457	3,10641563	0,11091239	0,38152086	LRRN4	leucine rich repeat neuronal 4 [Source:HGNC Symbol;Acc:HGNC:16208]
ENSG00000127314	-0,3791372	4,10075746	3,10496004	0,11098514	0,38168123	RAP1B	RAP1B, member of RAS oncogene family [Source:HGNC Symbol;Acc:HGNC:9857]
ENSG00000107745	0,28212501	6,30715817	3,10346391	0,11105997	0,38175886	MICU1	mitochondrial calcium uptake 1 [Source:HGNC Symbol;Acc:HGNC:1530]
ENSG00000280208	-0,7355332	-0,5311425	3,10374002	0,11104616	0,38175886	GGT4P	gamma-glutamyltransferase 4 pseudogene [Source:HGNC Symbol;Acc:HGNC:33207]
ENSG00000161653	-0,4235174	3,43795943	3,1020038	0,11113307	0,38183913	NAGS	N-acetylglutamate synthase [Source:HGNC Symbol;Acc:HGNC:17996]
ENSG00000259230	-0,3567205	1,57238344	3,10195328	0,1111356	0,38183913	LINC02323	long intergenic non-protein coding RNA 2323 [Source:HGNC Symbol;Acc:HGNC:53242]
ENSG00000185347	-0,2798248	3,97290261	3,10051155	0,11120784	0,38199749	TEDC1	tubulin epsilon and delta complex 1 [Source:HGNC Symbol;Acc:HGNC:20127]
ENSG00000204620	-1,0287103	-1,7279165	3,09910347	0,11127845	0,38215018	AC115618.1	novel transcript, antisense to RBM3
ENSG00000155329	0,31735735	4,26285282	3,09704678	0,11138169	0,38241483	ZCCHC10	zinc finger CCHC-type containing 10 [Source:HGNC Symbol;Acc:HGNC:25954]
ENSG00000120963	0,26382557	5,61529144	3,09482195	0,11149351	0,38252904	ZNF706	zinc finger protein 706 [Source:HGNC Symbol;Acc:HGNC:24992]
ENSG00000177602	0,28906775	3,27976894	3,0952169	0,11147365	0,38252904	HASPIN	histone H3 associated protein kinase [Source:HGNC Symbol;Acc:HGNC:19682]
ENSG00000206562	0,40793451	3,73679249	3,09487608	0,11149079	0,38252904	METTL6	methyltransferase like 6 [Source:HGNC Symbol;Acc:HGNC:28343]
ENSG00000141401	-0,3919446	5,2029711	3,09378351	0,11154575	0,38261842	IMPA2	inositol monophosphatase 2 [Source:HGNC Symbol;Acc:HGNC:6051]
ENSG00000105948	0,33088631	2,35627573	3,09007842	0,11173239	0,38316867	TTC26	tetratricopeptide repeat domain 26 [Source:HGNC Symbol;Acc:HGNC:21882]
ENSG00000189190	0,30250589	2,95429375	3,08805681	0,1118344	0,38325607	ZNF600	zinc finger protein 600 [Source:HGNC Symbol;Acc:HGNC:30951]
ENSG00000213177	-0,9872293	-1,7989179	3,08801362	0,11183658	0,38325607	PRDX2P4	peroxiredoxin 2 pseudogene 4 [Source:HGNC Symbol;Acc:HGNC:44969]
ENSG00000286760	-0,9194795	-1,6092028	3,08872991	0,11180042	0,38325607	AL022069.3	novel transcript
ENSG00000058600	-0,2344182	6,85872742	3,08407424	0,1120357	0,38383243	POLR3E	RNA polymerase III subunit E [Source:HGNC Symbol;Acc:HGNC:30347]
ENSG00000132906	0,28594607	3,78323432	3,08364723	0,11205732	0,38383243	CASP9	caspase 9 [Source:HGNC Symbol;Acc:HGNC:1511]
ENSG00000260708	-0,3291223	2,63203358	3,08216456	0,1121324	0,38399957	AL118516.1	novel transcript, antisense to TBC1D22A
ENSG00000105373	-0,2800585	6,42157564	3,08036195	0,11222376	0,38411535	NOP53	NOP53 ribosome biogenesis factor [Source:HGNC Symbol;Acc:HGNC:4333]

ENSG00000114956	0,24645958	7,20250102	3,07942335	0,11227138	0,38411535	DGUOK	deoxyguanosine kinase [Source:HGNC Symbol;Acc:HGNC:2858]
ENSG00000120158	-0,2564874	5,2250984	3,08002813	0,11224069	0,38411535	RCL1	RNA terminal phosphate cyclase like 1 [Source:HGNC Symbol;Acc:HGNC:17687]
ENSG00000224631	-0,2629828	4,89933232	3,0798265	0,11225092	0,38411535	RPS27AP16	RPS27A pseudogene 16 [Source:HGNC Symbol;Acc:HGNC:36855]
ENSG00000159403	-0,4518821	1,08108778	3,07882448	0,11230177	0,38412937	C1R	complement C1r [Source:HGNC Symbol;Acc:HGNC:1246]
ENSG00000116127	0,48212173	3,1928076	3,07777804	0,1123549	0,38414216	ALMS1	ALMS1 centrosome and basal body associated protein [Source:HGNC Symbol;Acc:HGNC:428]
ENSG00000137944	0,28795122	4,55525775	3,07771507	0,1123581	0,38414216	KYAT3	kynurenine aminotransferase 3 [Source:HGNC Symbol;Acc:HGNC:33238]
ENSG00000130816	-0,7047049	6,11680386	3,0765445	0,11241757	0,38425557	DNMT1	DNA methyltransferase 1 [Source:HGNC Symbol;Acc:HGNC:2976]
ENSG00000067992	0,27814463	3,51006656	3,07545794	0,11247281	0,38435446	PDK3	pyruvate dehydrogenase kinase 3 [Source:HGNC Symbol;Acc:HGNC:8811]
ENSG00000070367	0,27411306	3,67419832	3,07450982	0,11252104	0,38442936	EXOC5	exocyst complex component 5 [Source:HGNC Symbol;Acc:HGNC:10696]
ENSG00000175105	0,34704835	1,78249004	3,07379371	0,11255749	0,38446397	ZNF654	zinc finger protein 654 [Source:HGNC Symbol;Acc:HGNC:25612]
ENSG00000274602	-0,2956294	2,96221365	3,07258508	0,11261904	0,38453942	PI4KAP1	phosphatidylinositol 4-kinase alpha pseudogene 1 [Source:HGNC Symbol;Acc:HGNC:33576]
ENSG00000288044	-0,3282505	2,66230284	3,07232629	0,11263222	0,38453942	AL096870.12	novel transcript, antisense to RABGGTAand DHRS1
ENSG00000163882	0,27839485	6,74362806	3,06894612	0,11280461	0,38494803	POLR2H	RNA polymerase II, I and III subunit H [Source:HGNC Symbol;Acc:HGNC:9195]
ENSG00000182600	0,28253017	4,97005833	3,06900252	0,11280173	0,38494803	SNORC	secondary ossification center associated regulator of chondrocyte maturation [Source:HGNC Symbol;Acc:HGNC:3[illegible]
ENSG00000177427	0,25741906	4,01307916	3,06758162	0,11287429	0,38494867	MIEF2	mitochondrial elongation factor 2 [Source:HGNC Symbol;Acc:HGNC:17920]
ENSG00000184517	0,3135528	3,00184153	3,066879	0,1129102	0,38494867	ZFP1	ZFP1 zinc finger protein [Source:HGNC Symbol;Acc:HGNC:23328]
ENSG00000215190	0,3226641	2,21519443	3,06767209	0,11286967	0,38494867	LINC00680	long intergenic non-protein coding RNA 680 [Source:HGNC Symbol;Acc:HGNC:44417]
ENSG00000241468	-0,2842712	6,29710145	3,06688623	0,11290983	0,38494867	ATP5MF	ATP synthase membrane subunit f [Source:HGNC Symbol;Acc:HGNC:848]
ENSG00000105677	-0,2531897	7,63077674	3,06333002	0,11309177	0,38530324	TMEM147	transmembrane protein 147 [Source:HGNC Symbol;Acc:HGNC:30414]
ENSG00000233837	-0,9718212	-1,8731477	3,06333679	0,11309143	0,38530324	EIF3LP2	eukaryotic translation initiation factor 3 subunit L pseudogene 2 [Source:HGNC Symbol;Acc:HGNC:24657]
ENSG00000281404	0,99702904	-1,574086	3,06329983	0,11309332	0,38530324	LINC01176	long intergenic non-protein coding RNA 1176 [Source:HGNC Symbol;Acc:HGNC:49548]
ENSG00000007392	0,24145688	5,963732	3,06262442	0,11312792	0,38533125	LUC7L	LUC7 like [Source:HGNC Symbol;Acc:HGNC:6723]
ENSG00000108465	0,50324721	0,84276476	3,06174537	0,11317297	0,38539484	CDK5RAP3	CDK5 regulatory subunit associated protein 3 [Source:HGNC Symbol;Acc:HGNC:18673]
ENSG00000072954	0,364823	2,18718998	3,06066846	0,11322819	0,38549304	TMEM38A	transmembrane protein 38A [Source:HGNC Symbol;Acc:HGNC:28462]
ENSG00000142686	0,74238519	-0,7034225	3,0591195	0,11330768	0,38550403	C1orf216	chromosome 1 open reading frame 216 [Source:HGNC Symbol;Acc:HGNC:26800]
ENSG00000148341	-0,2616547	4,0321706	3,05973191	0,11327624	0,38550403	SH3GLB2	SH3 domain containing GRB2 like, endophilin B2 [Source:HGNC Symbol;Acc:HGNC:10834]
ENSG00000162390	0,32221899	2,65803471	3,05906294	0,11331058	0,38550403	ACOT11	acyl-CoA thioesterase 11 [Source:HGNC Symbol;Acc:HGNC:18156]
ENSG00000152767	0,23166292	7,44512907	3,05756774	0,11338738	0,3856755	FARP1	FERM, ARH/RhoGEF and pleckstrin domain protein 1 [Source:HGNC Symbol;Acc:HGNC:3591]
ENSG00000131828	0,27037497	7,75694693	3,05530188	0,11350389	0,38571258	PDHA1	pyruvate dehydrogenase E1 subunit alpha 1 [Source:HGNC Symbol;Acc:HGNC:8806]
ENSG00000151743	-0,3762749	2,49910326	3,0562593	0,11345464	0,38571258	AMN1	antagonist of mitotic exit network 1 homolog [Source:HGNC Symbol;Acc:HGNC:27281]
ENSG00000172775	-0,2751355	5,64599928	3,05668872	0,11343256	0,38571258	PSME3IP1	proteasome activator subunit 3 interacting protein 1 [Source:HGNC Symbol;Acc:HGNC:29856]
ENSG00000241081	-0,4109519	0,83578063	3,05538312	0,11349971	0,38571258	RPL22P2	ribosomal protein L22 pseudogene 2 [Source:HGNC Symbol;Acc:HGNC:19794]
ENSG00000129116	-0,5222998	1,13622431	3,05099413	0,11372582	0,38606586	PALLD	palladin, cytoskeletal associated protein [Source:HGNC Symbol;Acc:HGNC:17068]
ENSG00000135537	-0,2869044	3,02058287	3,05000929	0,11377664	0,38606586	AFG1L	AFG1 like ATPase [Source:HGNC Symbol;Acc:HGNC:16411]
ENSG00000135956	0,28119722	5,82998513	3,04867185	0,11384569	0,38606586	TMEM127	transmembrane protein 127 [Source:HGNC Symbol;Acc:HGNC:26038]
ENSG00000149823	0,25019522	6,1747345	3,05142462	0,11370362	0,38606586	VPS51	VPS51 subunit of GARP complex [Source:HGNC Symbol;Acc:HGNC:1172]
ENSG00000159147	-0,2494634	5,08795746	3,05099756	0,11372565	0,38606586	DONSON	DNA replication fork stabilization factor DONSON [Source:HGNC Symbol;Acc:HGNC:2993]
ENSG00000189306	-0,2452377	6,56334577	3,0501704	0,11376832	0,38606586	RRP7A	ribosomal RNA processing 7 homolog A [Source:HGNC Symbol;Acc:HGNC:24286]
ENSG00000267080	-0,4332935	1,389982	3,04913769	0,11382163	0,38606586	ASB16-AS1	ASB16 antisense RNA 1 [Source:HGNC Symbol;Acc:HGNC:25442]
ENSG00000270647	-0,3225284	6,59868088	3,05179092	0,11368473	0,38606586	TAF15	TATA-box binding protein associated factor 15 [Source:HGNC Symbol;Acc:HGNC:11547]
ENSG00000273820	0,29453694	3,08360195	3,04908924	0,11382414	0,38606586	USP27X	ubiquitin specific peptidase 27 X-linked [Source:HGNC Symbol;Acc:HGNC:13486]
ENSG00000267123	-0,7436554	-0,704686	3,0475719	0,11390253	0,38616895	SCAT1	S-phase cancer associated transcript 1 [Source:HGNC Symbol;Acc:HGNC:52930]
ENSG00000141384	-0,515277	0,44893708	3,045618	0,11400357	0,38624262	TAF4B	TATA-box binding protein associated factor 4b [Source:HGNC Symbol;Acc:HGNC:11538]
ENSG00000198431	-0,2429223	6,64146021	3,04576457	0,11399599	0,38624262	TXNRD1	thioredoxin reductase 1 [Source:HGNC Symbol;Acc:HGNC:12437]

ENSG00000274349	0,52730526	0,56936415	3,04629125	0,11396874	0,38624262	ZNF658	zinc finger protein 658 [Source:HGNC Symbol;Acc:HGNC:25226]
ENSG00000075790	0,76082086	-1,1711686	3,04381987	0,11409666	0,38628926	BCAP29	B cell receptor associated protein 29 [Source:HGNC Symbol;Acc:HGNC:24131]
ENSG00000100241	0,22652514	6,65041636	3,0440762	0,11408339	0,38628926	SBF1	SET binding factor 1 [Source:HGNC Symbol;Acc:HGNC:10542]
ENSG00000129493	0,31676389	3,65846687	3,04417062	0,1140785	0,38628926	HEATR5A	HEAT repeat containing 5A [Source:HGNC Symbol;Acc:HGNC:20276]
ENSG00000070214	-0,2867774	5,15067133	3,03993951	0,11429789	0,38678447	SLC44A1	solute carrier family 44 member 1 [Source:HGNC Symbol;Acc:HGNC:18798]
ENSG00000118707	0,22619182	6,03711568	3,0389153	0,11435108	0,38678447	TGIF2	TGFB induced factor homeobox 2 [Source:HGNC Symbol;Acc:HGNC:15764]
ENSG00000136816	-0,2893604	5,23760424	3,03844881	0,11437531	0,38678447	TOR1B	torsin family 1 member B [Source:HGNC Symbol;Acc:HGNC:11995]
ENSG00000182383	-0,3578461	1,61901913	3,03954534	0,11431835	0,38678447	RPL27AP5	ribosomal protein L27a pseudogene 5 [Source:HGNC Symbol;Acc:HGNC:36420]
ENSG00000186166	-0,3070857	3,23842761	3,03866866	0,11436389	0,38678447	CENATAC	centrosomal AT-AC splicing factor [Source:HGNC Symbol;Acc:HGNC:30460]
ENSG00000105193	-0,2589068	10,2504788	3,03547269	0,11453009	0,38702695	RPS16	ribosomal protein S16 [Source:HGNC Symbol;Acc:HGNC:10396]
ENSG00000108176	0,31414941	3,30917633	3,03457353	0,1145769	0,38702695	DNAJC12	DnaJ heat shock protein family (Hsp40) member C12 [Source:HGNC Symbol;Acc:HGNC:28908]
ENSG00000120733	0,41963415	5,36110855	3,03452409	0,11457947	0,38702695	KDM3B	lysine demethylase 3B [Source:HGNC Symbol;Acc:HGNC:1337]
ENSG00000263753	-0,3185437	3,30346532	3,03454259	0,11457851	0,38702695	LINC00667	long intergenic non-protein coding RNA 667 [Source:HGNC Symbol;Acc:HGNC:27906]
ENSG00000285872	-0,7347333	0,24064716	3,03529514	0,11453933	0,38702695	AC007240.3	novel transcript
ENSG00000266028	0,363423	3,70557326	3,0330732	0,11465507	0,38719276	SRGAP2	SLIT-ROBO Rho GTPase activating protein 2 [Source:HGNC Symbol;Acc:HGNC:19751]
ENSG00000143224	-0,2784952	3,69734036	3,03098128	0,11476417	0,38739812	PPOX	protoporphyrinogen oxidase [Source:HGNC Symbol;Acc:HGNC:9280]
ENSG00000152315	-0,3636264	2,4313183	3,03089041	0,11476891	0,38739812	KCNK13	potassium two pore domain channel subfamily K member 13 [Source:HGNC Symbol;Acc:HGNC:6275]
ENSG00000048342	-0,3286668	2,5911905	3,0300375	0,11481344	0,38744167	CC2D2A	coiled-coil and C2 domain containing 2A [Source:HGNC Symbol;Acc:HGNC:29253]
ENSG00000101365	0,30483215	7,57227143	3,02962732	0,11483486	0,38744167	IDH3B	isocitrate dehydrogenase (NAD(+)) 3 non-catalytic subunit beta [Source:HGNC Symbol;Acc:HGNC:5385]
ENSG00000221823	-0,2779772	3,92041789	3,02703944	0,11497012	0,38780847	PPP3R1	protein phosphatase 3 regulatory subunit B, alpha [Source:HGNC Symbol;Acc:HGNC:9317]
ENSG00000125356	-0,2635939	7,71430478	3,02422685	0,11511736	0,3881366	NDUFA1	NADH:ubiquinone oxidoreductase subunit A1 [Source:HGNC Symbol;Acc:HGNC:7683]
ENSG00000274487	0,37993166	1,28224034	3,02416621	0,11512053	0,3881366	NPEPPSP1	NPEPPS pseudogene 1 [Source:HGNC Symbol;Acc:HGNC:44259]
ENSG00000185896	0,35658007	8,59760192	3,02204531	0,11523173	0,38842185	LAMP1	lysosomal associated membrane protein 1 [Source:HGNC Symbol;Acc:HGNC:6499]
ENSG00000136146	0,2532139	5,5726269	3,01752064	0,1154694	0,38903823	MED4	mediator complex subunit 4 [Source:HGNC Symbol;Acc:HGNC:17903]
ENSG00000143971	0,36612756	2,29904271	3,01789891	0,11544951	0,38903823	ETAA1	ETAA1 activator of ATR kinase [Source:HGNC Symbol;Acc:HGNC:24648]
ENSG00000151466	-0,3668423	2,16530912	3,01651811	0,11552215	0,38903823	SCLT1	sodium channel and clathrin linker 1 [Source:HGNC Symbol;Acc:HGNC:26406]
ENSG00000168159	0,25729018	6,64721704	3,01678887	0,1155079	0,38903823	RNF187	ring finger protein 187 [Source:HGNC Symbol;Acc:HGNC:27146]
ENSG00000168434	0,31033621	4,45767237	3,01552613	0,11557437	0,38903823	COG7	component of oligomeric golgi complex 7 [Source:HGNC Symbol;Acc:HGNC:18622]
ENSG00000172000	-0,3336717	3,71110991	3,01558844	0,11557109	0,38903823	ZNF556	zinc finger protein 556 [Source:HGNC Symbol;Acc:HGNC:25669]
ENSG00000136942	-0,242829	9,93871396	3,01314556	0,11569981	0,38935605	RPL35	ribosomal protein L35 [Source:HGNC Symbol;Acc:HGNC:10344]
ENSG00000159445	-0,2728808	4,07221298	3,01207669	0,11575619	0,38935605	THEM4	thioesterase superfamily member 4 [Source:HGNC Symbol;Acc:HGNC:17947]
ENSG00000233006	0,79462188	-1,3713739	3,01258228	0,11572952	0,38935605	MIR3936HG	MIR3936 host gene [Source:HGNC Symbol;Acc:HGNC:40538]
ENSG00000240509	-0,3982062	1,41564289	3,01171294	0,11577539	0,38935605	RPL34P18	ribosomal protein L34 pseudogene 18 [Source:HGNC Symbol;Acc:HGNC:35968]
ENSG00000091262	-0,2779265	5,50368166	3,01002627	0,11586445	0,38956588	ABCC6	ATP binding cassette subfamily C member 6 [Source:HGNC Symbol;Acc:HGNC:57]
ENSG00000072062	-0,3324187	5,21141801	3,00729418	0,1160089	0,38987209	PRKACA	protein kinase cAMP-activated catalytic subunit alpha [Source:HGNC Symbol;Acc:HGNC:9380]
ENSG00000213079	0,31896843	4,97383176	3,00747239	0,11599947	0,38987209	SCAF8	SR-related CTD associated factor 8 [Source:HGNC Symbol;Acc:HGNC:20959]
ENSG00000218283	0,27047889	3,89193049	3,00637117	0,11605775	0,38994656	MORF4L1P1	mortality factor 4 like 1 pseudogene 1 [Source:HGNC Symbol;Acc:HGNC:20400]
ENSG00000173706	0,54880531	4,76955251	3,00475863	0,11614316	0,39014381	HEG1	heart development protein with EGF like domains 1 [Source:HGNC Symbol;Acc:HGNC:29227]
ENSG00000164970	-0,304667	2,67149829	3,0040462	0,11618092	0,39018093	FAM219A	family with sequence similarity 219 member A [Source:HGNC Symbol;Acc:HGNC:19920]
ENSG00000166473	-0,3533839	1,67634397	3,00318045	0,11622683	0,3902454	PKD1L2	polycystin 1 like 2 (gene/pseudogene) [Source:HGNC Symbol;Acc:HGNC:21715]
ENSG00000251393	-0,8874365	-0,1051429	3,0026516	0,11625488	0,39024991	AC005280.1	novel transcript, antisense to NUMB and HEATR4
ENSG00000110429	0,31421073	4,00989747	3,00145711	0,11631828	0,39037302	FBXO3	F-box protein 3 [Source:HGNC Symbol;Acc:HGNC:13582]
ENSG00000100031	-0,5205405	1,20831186	3,00060875	0,11636333	0,39043453	GGT1	gamma-glutamyltransferase 1 [Source:HGNC Symbol;Acc:HGNC:4250]
ENSG00000116209	-0,2688103	6,82482694	2,99911167	0,11644289	0,39061177	TMEM59	transmembrane protein 59 [Source:HGNC Symbol;Acc:HGNC:1239]

ENSG00000197766	-0,4126728	1,02506279	2,99828403	0,11648691	0,3906697	CFD	complement factor D [Source:HGNC Symbol;Acc:HGNC:2771]
ENSG00000104343	0,49337414	1,89074231	2,99768798	0,11651862	0,39068637	UBE2W	ubiquitin conjugating enzyme E2 W [Source:HGNC Symbol;Acc:HGNC:25616]
ENSG00000239305	0,29383469	4,21963016	2,99657448	0,11657789	0,3907954	RNF103	ring finger protein 103 [Source:HGNC Symbol;Acc:HGNC:12859]
ENSG00000184697	0,31269897	6,35638804	2,99504784	0,11665921	0,3909783	CLDN6	claudin 6 [Source:HGNC Symbol;Acc:HGNC:2048]
ENSG00000177697	-0,2704389	7,49678163	2,99114923	0,11686722	0,39122667	CD151	CD151 molecule (Raph blood group) [Source:HGNC Symbol;Acc:HGNC:1630]
ENSG00000187239	0,35796891	4,35757427	2,99307704	0,1167643	0,39122667	FNBP1	formin binding protein 1 [Source:HGNC Symbol;Acc:HGNC:17069]
ENSG00000204128	0,23544112	6,50872988	2,99137878	0,11685496	0,39122667	C2orf72	chromosome 2 open reading frame 72 [Source:HGNC Symbol;Acc:HGNC:27418]
ENSG00000256148	0,84257874	-1,5022941	2,99170248	0,11683767	0,39122667	AP000763.2	mitochondrially encoded cytochrome c oxidase I (MT-COI) pseudogene
ENSG00000279799	-1,0053071	-1,5629128	2,99255878	0,11679196	0,39122667	AC006077.2	TEC
ENSG00000223705	0,39354862	1,11507204	2,98938853	0,11696131	0,39145197	NSUN5P1	NSUN5 pseudogene 1 [Source:HGNC Symbol;Acc:HGNC:19146]
ENSG00000144802	0,43703009	2,64740789	2,98621032	0,11713141	0,39188758	NFKBIZ	NFKB inhibitor zeta [Source:HGNC Symbol;Acc:HGNC:29805]
ENSG00000149476	-0,3872456	6,75327188	2,98545381	0,11717195	0,39188758	TKFC	triokinase and FMN cyclase [Source:HGNC Symbol;Acc:HGNC:24552]
ENSG00000177303	0,34170478	3,81811779	2,98550795	0,11716905	0,39188758	CASKIN2	CASK interacting protein 2 [Source:HGNC Symbol;Acc:HGNC:18200]
ENSG00000099624	-0,2959055	6,66017311	2,9843742	0,11722983	0,39190172	ATP5F1D	ATP synthase F1 subunit delta [Source:HGNC Symbol;Acc:HGNC:837]
ENSG00000248461	-0,7925499	-0,4623733	2,984853	0,11720415	0,39190172	LINC02119	long intergenic non-protein coding RNA 2119 [Source:HGNC Symbol;Acc:HGNC:52975]
ENSG00000239569	-0,4367931	1,9836046	2,98340301	0,11728192	0,39198618	KMT2E-AS1	KMT2E antisense RNA 1 [Source:HGNC Symbol;Acc:HGNC:40845]
ENSG00000124570	-0,2234567	6,17175829	2,98260018	0,11732501	0,3920405	SERPINB6	serpin family B member 6 [Source:HGNC Symbol;Acc:HGNC:8950]
ENSG00000137275	-0,3320506	4,53494049	2,98033773	0,11744655	0,39235688	RIPK1	receptor interacting serine/threonine kinase 1 [Source:HGNC Symbol;Acc:HGNC:10019]
ENSG00000103253	-0,3882245	1,80622925	2,9787781	0,11753042	0,39236791	HAGHL	hydroxyacylglutathione hydrolase like [Source:HGNC Symbol;Acc:HGNC:14177]
ENSG00000130165	-0,3429129	3,96399764	2,97889025	0,11752439	0,39236791	ELOF1	elongation factor 1 homolog [Source:HGNC Symbol;Acc:HGNC:28691]
ENSG00000148175	0,27130429	6,57025938	2,97932381	0,11750107	0,39236791	STOM	stomatin [Source:HGNC Symbol;Acc:HGNC:3383]
ENSG00000120054	-0,2757945	5,25338161	2,97781353	0,11758234	0,39245153	CPN1	carboxypeptidase N subunit 1 [Source:HGNC Symbol;Acc:HGNC:2312]
ENSG00000188938	-0,3218524	4,41753383	2,97706222	0,11762279	0,39249688	FAM120AOS	family with sequence similarity 120A opposite strand [Source:HGNC Symbol;Acc:HGNC:23389]
ENSG00000149554	0,3012637	4,93765296	2,97596052	0,11768215	0,39260527	CHEK1	checkpoint kinase 1 [Source:HGNC Symbol;Acc:HGNC:1925]
ENSG00000126070	0,32339801	3,23522654	2,97446807	0,11776261	0,39278402	AGO3	argonaute RISC catalytic component 3 [Source:HGNC Symbol;Acc:HGNC:18421]
ENSG00000186908	0,28181714	3,11258394	2,97339898	0,1178203	0,39288672	ZDHHC17	zinc finger DHHC-type palmitoyltransferase 17 [Source:HGNC Symbol;Acc:HGNC:18412]
ENSG00000083812	0,36348351	2,84272963	2,97247254	0,11787032	0,39296382	ZNF324	zinc finger protein 324 [Source:HGNC Symbol;Acc:HGNC:14096]
ENSG00000114770	0,33577606	4,12977138	2,97066105	0,11796819	0,39302106	ABCC5	ATP binding cassette subfamily C member 5 [Source:HGNC Symbol;Acc:HGNC:56]
ENSG00000138600	0,24715601	5,24836528	2,97075986	0,11796285	0,39302106	SPPL2A	signal peptide peptidase like 2A [Source:HGNC Symbol;Acc:HGNC:30227]
ENSG00000148429	0,26558062	4,14645028	2,97069773	0,11796621	0,39302106	USP6NL	USP6 N-terminal like [Source:HGNC Symbol;Acc:HGNC:16858]
ENSG00000213160	0,26375491	4,31221719	2,96607398	0,11821651	0,39375855	KLHL23	kelch like family member 23 [Source:HGNC Symbol;Acc:HGNC:27506]
ENSG00000160972	-0,2837349	5,44949133	2,96329609	0,11836722	0,39399103	PPP1R16A	protein phosphatase 1 regulatory subunit 16A [Source:HGNC Symbol;Acc:HGNC:14941]
ENSG00000172915	0,4730891	0,68214479	2,96376635	0,11834169	0,39399103	NBEA	neurobeachin [Source:HGNC Symbol;Acc:HGNC:7648]
ENSG00000175634	-0,2608371	6,50550841	2,96385247	0,11833701	0,39399103	RPS6KB2	ribosomal protein S6 kinase B2 [Source:HGNC Symbol;Acc:HGNC:10437]
ENSG00000129255	0,23493535	7,29697227	2,96145461	0,11846725	0,39414441	MPDU1	mannose-P-dolichol utilization defect 1 [Source:HGNC Symbol;Acc:HGNC:7207]
ENSG00000158163	-0,812009	-0,723029	2,96191774	0,11844208	0,39414441	DZIP1L	DAZ interacting zinc finger protein 1 like [Source:HGNC Symbol;Acc:HGNC:26551]
ENSG00000196268	0,66025272	-0,7406407	2,96021989	0,11853439	0,39427799	ZNF493	zinc finger protein 493 [Source:HGNC Symbol;Acc:HGNC:23708]
ENSG00000065809	0,23351075	5,96926107	2,95758251	0,11867796	0,39430665	FAM107B	family with sequence similarity 107 member B [Source:HGNC Symbol;Acc:HGNC:23726]
ENSG00000089050	0,30368115	3,85409059	2,95864589	0,11862005	0,39430665	RBBP9	RB binding protein 9, serine hydrolase [Source:HGNC Symbol;Acc:HGNC:9892]
ENSG00000171813	-0,319175	1,79469657	2,9584462	0,11863092	0,39430665	PWWP2B	PWWP domain containing 2B [Source:HGNC Symbol;Acc:HGNC:25150]
ENSG00000188690	-0,2672606	5,29776629	2,95806431	0,11865172	0,39430665	UROS	uroporphyrinogen III synthase [Source:HGNC Symbol;Acc:HGNC:12592]
ENSG00000224126	-0,9691846	-1,9325116	2,95906433	0,11859727	0,39430665	UBE2SP2	ubiquitin conjugating enzyme E2 S pseudogene 2 [Source:HGNC Symbol;Acc:HGNC:32196]
ENSG00000198816	-0,3626339	3,29109103	2,95626207	0,11874992	0,39445603	ZNF358	zinc finger protein 358 [Source:HGNC Symbol;Acc:HGNC:16838]
ENSG00000122952	-0,267845	6,32810745	2,95548622	0,11879223	0,39450687	ZWINT	ZW10 interacting kinetochore protein [Source:HGNC Symbol;Acc:HGNC:13195]

ENSG00000129235	-0,2670225	6,4930822	2,95285654	0,11893578	0,39464433	TXNDC17	thioredoxin domain containing 17 [Source:HGNC Symbol;Acc:HGNC:28218]
ENSG00000155876	-0,2463387	6,36108734	2,95196212	0,11898466	0,39464433	RRAGA	Ras related GTP binding A [Source:HGNC Symbol;Acc:HGNC:16963]
ENSG00000158615	-0,2635015	5,08131274	2,95413857	0,11886577	0,39464433	PPP1R15B	protein phosphatase 1 regulatory subunit 15B [Source:HGNC Symbol;Acc:HGNC:14951]
ENSG00000176909	-0,3018594	2,66179525	2,95229707	0,11896635	0,39464433	MAMSTR	MEF2 activating motif and SAP domain containing transcriptional regulator [Source:HGNC Symbol;Acc:HGNC:266
ENSG00000178665	0,86559072	-1,1546782	2,95175998	0,11899571	0,39464433	ZNF713	zinc finger protein 713 [Source:HGNC Symbol;Acc:HGNC:22043]
ENSG00000227118	-0,6529756	-0,6686243	2,95269646	0,11894453	0,39464433	BTF3P13	basic transcription factor 3 pseudogene 13 [Source:HGNC Symbol;Acc:HGNC:1128]
ENSG00000144134	-0,7179581	0,14137272	2,95110005	0,11903179	0,3946744	RABL2A	RAB, member of RAS oncogene family like 2A [Source:HGNC Symbol;Acc:HGNC:9799]
ENSG00000163867	-0,423451	0,83134653	2,94949058	0,11911985	0,39487676	ZMYM6	zinc finger MYM-type containing 6 [Source:HGNC Symbol;Acc:HGNC:13050]
ENSG00000166881	0,31362696	4,00592856	2,9488716	0,11915374	0,39489949	NEMP1	nuclear envelope integral membrane protein 1 [Source:HGNC Symbol;Acc:HGNC:29001]
ENSG00000151092	0,26347168	5,79908992	2,94613773	0,11930356	0,39521672	NGLY1	N-glycanase 1 [Source:HGNC Symbol;Acc:HGNC:17646]
ENSG00000198585	-0,2604286	4,96362472	2,94659386	0,11927855	0,39521672	NUDT16	nudix hydrolase 16 [Source:HGNC Symbol;Acc:HGNC:26442]
ENSG00000084092	0,25036222	5,34933843	2,94484477	0,1193745	0,39536208	NOA1	nitric oxide associated 1 [Source:HGNC Symbol;Acc:HGNC:28473]
ENSG00000078237	-0,6048027	-0,2659904	2,94210175	0,11952519	0,39562249	TIGAR	TP53 induced glycolysis regulatory phosphatase [Source:HGNC Symbol;Acc:HGNC:1185]
ENSG00000104880	0,37523399	4,48640156	2,9419347	0,11953437	0,39562249	ARHGEF18	Rho/Rac guanine nucleotide exchange factor 18 [Source:HGNC Symbol;Acc:HGNC:17090]
ENSG00000284976	-0,3064371	4,15760755	2,9426262	0,11949636	0,39562249	BX255925.3	novel RING finger protein
ENSG00000156253	-0,3483284	3,60999174	2,94001738	0,11963985	0,39588191	RWDD2B	RWD domain containing 2B [Source:HGNC Symbol;Acc:HGNC:1302]
ENSG00000141644	0,24399957	4,89994205	2,93706736	0,11980238	0,39624021	MBD1	methyl-CpG binding domain protein 1 [Source:HGNC Symbol;Acc:HGNC:6916]
ENSG00000197291	0,90971941	-0,958739	2,93748483	0,11977936	0,39624021	RAMP2-AS1	RAMP2 antisense RNA 1 [Source:HGNC Symbol;Acc:HGNC:44358]
ENSG00000136603	-0,3305686	1,65239296	2,9323729	0,1200616	0,39637965	SKIL	SKI like proto-oncogene [Source:HGNC Symbol;Acc:HGNC:10897]
ENSG00000156011	0,42983966	1,64410972	2,93286365	0,12003447	0,39637965	PSD3	pleckstrin and Sec7 domain containing 3 [Source:HGNC Symbol;Acc:HGNC:19093]
ENSG00000170890	-1,0656375	-1,7125466	2,93313765	0,12001932	0,39637965	PLA2G1B	phospholipase A2 group IB [Source:HGNC Symbol;Acc:HGNC:9030]
ENSG00000227674	-0,5272767	0,96081543	2,93293527	0,12003051	0,39637965	LINC00355	long intergenic non-protein coding RNA 355 [Source:HGNC Symbol;Acc:HGNC:27061]
ENSG00000256340	-0,2642296	4,47015851	2,933486	0,12000007	0,39637965	ABCC6P1	ATP binding cassette subfamily C member 6 pseudogene 1 [Source:HGNC Symbol;Acc:HGNC:33352]
ENSG00000262879	0,32689348	2,65133822	2,93314671	0,12001882	0,39637965	AC005670.3	novel transcript
ENSG00000271122	-0,4376886	1,34508416	2,93494649	0,1199194	0,39637965	AC018647.2	novel transcript
ENSG00000275464	-0,3792951	2,043903	2,93539843	0,11989445	0,39637965	FP565260.1	periodic tryptophan protein 2 homolog [Source:NCBI gene (formerly Entrezgene);Acc:102724159]
ENSG00000274523	0,27492814	6,92014388	2,93181857	0,12009226	0,39639128	RCC1L	RCC1 like [Source:HGNC Symbol;Acc:HGNC:14948]
ENSG00000152253	0,30732172	4,33513212	2,92810178	0,12029807	0,39698092	SPC25	SPC25 component of NDC80 kinetochore complex [Source:HGNC Symbol;Acc:HGNC:24031]
ENSG00000144445	-0,4573852	0,59340573	2,9273732	0,12033847	0,39702455	KANSL1L	KAT8 regulatory NSL complex subunit 1 like [Source:HGNC Symbol;Acc:HGNC:26310]
ENSG00000267100	-0,2893058	3,46469209	2,92666831	0,12037757	0,39706388	ILF3-DT	ILF3 divergent transcript [Source:HGNC Symbol;Acc:HGNC:27115]
ENSG00000115318	0,9900276	-1,2709075	2,92563071	0,12043515	0,39716415	LOXL3	lysyl oxidase like 3 [Source:HGNC Symbol;Acc:HGNC:13869]
ENSG00000177628	0,35206544	5,35257219	2,92505046	0,12046737	0,39718074	GBA	glucosylceramidase beta [Source:HGNC Symbol;Acc:HGNC:4177]
ENSG00000107902	-0,2976152	2,62051258	2,92446011	0,12050016	0,39719921	LHPP	phospholysine phosphohistidine inorganic pyrophosphate phosphatase [Source:HGNC Symbol;Acc:HGNC:30042]
ENSG00000146918	0,26316927	5,07029704	2,92267767	0,12059924	0,39743611	NCAPG2	non-SMC condensin II complex subunit G2 [Source:HGNC Symbol;Acc:HGNC:21904]
ENSG00000084463	0,22926111	6,68040232	2,91267747	0,12115703	0,39796784	WBP11	WW domain binding protein 11 [Source:HGNC Symbol;Acc:HGNC:16461]
ENSG00000090432	0,22830067	6,8412755	2,91475279	0,121041	0,39796784	MUL1	mitochondrial E3 ubiquitin protein ligase 1 [Source:HGNC Symbol;Acc:HGNC:25762]
ENSG00000090686	0,2470769	5,53697155	2,91222705	0,12118223	0,39796784	USP48	ubiquitin specific peptidase 48 [Source:HGNC Symbol;Acc:HGNC:18533]
ENSG00000100150	0,42455248	1,50897413	2,91874461	0,12081822	0,39796784	DEPDC5	DEP domain containing 5, GATOR1 subcomplex subunit [Source:HGNC Symbol;Acc:HGNC:18423]
ENSG00000111339	0,41926023	3,37379177	2,91197301	0,12119645	0,39796784	ART4	ADP-ribosyltransferase 4 (Dombrock blood group) [Source:HGNC Symbol;Acc:HGNC:726]
ENSG00000127483	0,22716949	5,67691498	2,91811448	0,12085336	0,39796784	HP1BP3	heterochromatin protein 1 binding protein 3 [Source:HGNC Symbol;Acc:HGNC:24973]
ENSG00000152154	1,40610422	-1,1519701	2,91479773	0,12103849	0,39796784	TMEM178A	transmembrane protein 178A [Source:HGNC Symbol;Acc:HGNC:28517]
ENSG00000158092	-0,3055119	3,53036318	2,91549414	0,12099959	0,39796784	NCK1	NCK adaptor protein 1 [Source:HGNC Symbol;Acc:HGNC:7664]
ENSG00000158987	0,54583671	0,90124461	2,91202791	0,12119338	0,39796784	RAPGEF6	Rap guanine nucleotide exchange factor 6 [Source:HGNC Symbol;Acc:HGNC:20655]
ENSG00000162755	0,59230048	0,80820932	2,91795869	0,12086204	0,39796784	KLHDC9	kelch domain containing 9 [Source:HGNC Symbol;Acc:HGNC:28489]

ENSG00000170027	0,22100648	6,15323431	2,91473635	0,12104192	0,39796784	YWHAG	tyrosine 3-monooxygenase/tryptophan 5-monooxygenase activation protein gamma [Source:HGNC Symbol;Acc:H
ENSG00000180432	-0,9203793	-0,8838916	2,91225976	0,1211804	0,39796784	CYP8B1	cytochrome P450 family 8 subfamily B member 1 [Source:HGNC Symbol;Acc:HGNC:2653]
ENSG00000198551	-0,3306028	3,18508756	2,91469042	0,12104449	0,39796784	ZNF627	zinc finger protein 627 [Source:HGNC Symbol;Acc:HGNC:30570]
ENSG00000214796	-0,3710048	2,18402645	2,91590925	0,12097641	0,39796784	TUBA5P	tubulin alpha 5, pseudogene [Source:HGNC Symbol;Acc:HGNC:54799]
ENSG00000221909	-0,3083865	2,39647358	2,91686868	0,12092285	0,39796784	FAM200A	family with sequence similarity 200 member A [Source:HGNC Symbol;Acc:HGNC:25401]
ENSG00000243364	0,26647097	4,77181162	2,91383814	0,12109212	0,39796784	EFNA4	ephrin A4 [Source:HGNC Symbol;Acc:HGNC:3224]
ENSG00000036054	0,30403168	4,26320477	2,90974047	0,12132148	0,39807977	TBC1D23	TBC1 domain family member 23 [Source:HGNC Symbol;Acc:HGNC:25622]
ENSG00000103351	0,26515789	3,29945141	2,91017898	0,12129691	0,39807977	CLUAP1	clusterin associated protein 1 [Source:HGNC Symbol;Acc:HGNC:19009]
ENSG00000164713	-0,2937724	6,13483769	2,90893231	0,12136678	0,39807977	BRI3	brain protein I3 [Source:HGNC Symbol;Acc:HGNC:1109]
ENSG00000181827	0,53291252	2,65009101	2,91055055	0,12127609	0,39807977	RFX7	regulatory factor X7 [Source:HGNC Symbol;Acc:HGNC:25777]
ENSG00000198954	0,33370926	4,70013371	2,90934456	0,12134367	0,39807977	KIFBP	kinesin family binding protein [Source:HGNC Symbol;Acc:HGNC:23419]
ENSG00000126653	-0,3554281	4,2189127	2,90573861	0,12154602	0,39824464	NSRP1	nuclear speckle splicing regulatory protein 1 [Source:HGNC Symbol;Acc:HGNC:25305]
ENSG00000164048	0,34234231	2,63224188	2,9056081	0,12155335	0,39824464	ZNF589	zinc finger protein 589 [Source:HGNC Symbol;Acc:HGNC:16747]
ENSG00000204650	0,3606935	1,84627594	2,90603389	0,12152943	0,39824464	LINC02210	long intergenic non-protein coding RNA 2210 [Source:HGNC Symbol;Acc:HGNC:26327]
ENSG00000215006	-0,4462815	0,65467947	2,90714954	0,12146679	0,39824464	CHCHD2P2	coiled-coil-helix-coiled-coil-helix domain containing 2 pseudogene 2 [Source:HGNC Symbol;Acc:HGNC:39586]
ENSG00000231767	-0,3634722	1,51461309	2,90640289	0,12150871	0,39824464	RPS27AP5	ribosomal protein S27a pseudogene 5 [Source:HGNC Symbol;Acc:HGNC:36867]
ENSG00000256045	0,53523056	0,25166081	2,90440231	0,12162111	0,39837731	MTRNR2L10	MT-RNR2 like 10 [Source:HGNC Symbol;Acc:HGNC:37167]
ENSG00000131368	-0,2347219	6,08856725	2,90288494	0,12170645	0,3985675	MRPS25	mitochondrial ribosomal protein S25 [Source:HGNC Symbol;Acc:HGNC:14511]
ENSG00000105696	-0,360965	4,41848961	2,90197865	0,12175747	0,398599	TMEM59L	transmembrane protein 59 like [Source:HGNC Symbol;Acc:HGNC:13237]
ENSG00000158286	-0,4581974	0,66743863	2,90174458	0,12177064	0,398599	RNF207	ring finger protein 207 [Source:HGNC Symbol;Acc:HGNC:32947]
ENSG00000025293	-0,2833607	4,54787562	2,90030984	0,12185147	0,39876877	PHF20	PHD finger protein 20 [Source:HGNC Symbol;Acc:HGNC:16098]
ENSG00000204217	0,28681578	3,6232061	2,89985504	0,1218771	0,39876877	BMPR2	bone morphogenetic protein receptor type 2 [Source:HGNC Symbol;Acc:HGNC:1078]
ENSG00000138658	0,39767174	1,67366875	2,89918241	0,12191502	0,39880354	ZGRF1	zinc finger GRF-type containing 1 [Source:HGNC Symbol;Acc:HGNC:25654]
ENSG00000133027	-0,2293207	5,67873376	2,89686872	0,12204559	0,39907641	PEMT	phosphatidylethanolamine N-methyltransferase [Source:HGNC Symbol;Acc:HGNC:8830]
ENSG00000214013	-0,2638164	2,99144438	2,89673615	0,12205308	0,39907641	GANC	glucosidase alpha, neutral C [Source:HGNC Symbol;Acc:HGNC:4139]
ENSG00000168038	-0,3157237	3,2844334	2,89501211	0,12215049	0,39930556	ULK4	unc-51 like kinase 4 [Source:HGNC Symbol;Acc:HGNC:15784]
ENSG00000103549	0,28997679	7,07159235	2,8936638	0,12222675	0,39946545	RNF40	ring finger protein 40 [Source:HGNC Symbol;Acc:HGNC:16867]
ENSG00000108590	0,29530884	3,35982831	2,89107408	0,12237339	0,39985524	MED31	mediator complex subunit 31 [Source:HGNC Symbol;Acc:HGNC:24260]
ENSG00000076321	0,39676557	3,04151274	2,89026828	0,12241906	0,39991503	KLHL20	kelch like family member 20 [Source:HGNC Symbol;Acc:HGNC:25056]
ENSG00000125484	0,24679848	4,85672529	2,88925919	0,12247628	0,40001253	GTF3C4	general transcription factor IIIC subunit 4 [Source:HGNC Symbol;Acc:HGNC:4667]
ENSG00000092847	0,35956934	3,94954771	2,88772516	0,12256335	0,40004729	AGO1	argonaute RISC component 1 [Source:HGNC Symbol;Acc:HGNC:3262]
ENSG00000103429	0,27601428	7,31686521	2,88762419	0,12256908	0,40004729	BFAR	bifunctional apoptosis regulator [Source:HGNC Symbol;Acc:HGNC:17613]
ENSG00000135744	0,31894905	10,8914713	2,8877104	0,12256418	0,40004729	AGT	angiotensinogen [Source:HGNC Symbol;Acc:HGNC:333]
ENSG00000166938	0,25465614	5,1820176	2,88489829	0,12272399	0,40010595	DIS3L	DIS3 like exosome 3'-5' exoribonuclease [Source:HGNC Symbol;Acc:HGNC:28698]
ENSG00000167664	0,95829826	-1,8938942	2,88572184	0,12267716	0,40010595	TMIGD2	transmembrane and immunoglobulin domain containing 2 [Source:HGNC Symbol;Acc:HGNC:28324]
ENSG00000197471	0,94452735	0,54263076	2,88514086	0,12271019	0,40010595	SPN	sialophorin [Source:HGNC Symbol;Acc:HGNC:11249]
ENSG00000213363	-0,3973941	0,84828706	2,88626929	0,12264604	0,40010595	RPS3P6	ribosomal protein S3 pseudogene 6 [Source:HGNC Symbol;Acc:HGNC:36061]
ENSG00000271918	-0,6157196	-0,004344	2,88529214	0,12270159	0,40010595	AC034236.2	novel transcript, antisense to COMMD10
ENSG00000119383	0,24870168	6,55294463	2,88361757	0,12279686	0,40025419	PTPA	protein phosphatase 2 phosphatase activator [Source:HGNC Symbol;Acc:HGNC:9308]
ENSG00000074855	0,50370663	1,26515961	2,88193123	0,12289289	0,40047785	ANO8	anoctamin 8 [Source:HGNC Symbol;Acc:HGNC:29329]
ENSG00000277969	0,39130494	2,53069386	2,88086742	0,12295352	0,40058607	AC006449.6	novel transcript
ENSG00000188493	-0,3096006	2,9621971	2,88023701	0,12298947	0,40061385	C19orf54	chromosome 19 open reading frame 54 [Source:HGNC Symbol;Acc:HGNC:24758]
ENSG00000196917	-0,4466892	0,50055909	2,87825981	0,1231023	0,40080265	HCAR1	hydroxycarboxylic acid receptor 1 [Source:HGNC Symbol;Acc:HGNC:4532]
ENSG00000234118	-0,5387288	-0,0619004	2,87847995	0,12308973	0,40080265	RPL13AP6	ribosomal protein L13a pseudogene 6 [Source:HGNC Symbol;Acc:HGNC:23737]

ENSG00000115365	0,26463724	5,09130565	2,87633224	0,12321243	0,40107182	LANCL1	LanC like 1 [Source:HGNC Symbol;Acc:HGNC:6508]
ENSG00000062598	0,27835253	5,23191113	2,87135126	0,12349759	0,40151025	ELMO2	engulfment and cell motility 2 [Source:HGNC Symbol;Acc:HGNC:17233]
ENSG00000100580	0,42529405	1,98997495	2,87296084	0,12340535	0,40151025	TMED8	transmembrane p24 trafficking protein family member 8 [Source:HGNC Symbol;Acc:HGNC:18633]
ENSG00000113300	0,29966432	3,37663349	2,87158491	0,1234842	0,40151025	CNOT6	CCR4-NOT transcription complex subunit 6 [Source:HGNC Symbol;Acc:HGNC:14099]
ENSG00000122026	-0,2665737	9,57294583	2,87014145	0,12356698	0,40151025	RPL21	ribosomal protein L21 [Source:HGNC Symbol;Acc:HGNC:10313]
ENSG00000135083	0,30836902	3,1713656	2,87337053	0,12338189	0,40151025	CCNJL	cyclin J like [Source:HGNC Symbol;Acc:HGNC:25876]
ENSG00000145592	-0,2707411	10,2341186	2,87222419	0,12344756	0,40151025	RPL37	ribosomal protein L37 [Source:HGNC Symbol;Acc:HGNC:10347]
ENSG00000152782	0,31164358	4,39698676	2,87051694	0,12354544	0,40151025	PANK1	pantothenate kinase 1 [Source:HGNC Symbol;Acc:HGNC:8598]
ENSG00000163781	0,26146035	5,60896532	2,87085026	0,12352632	0,40151025	TOPBP1	DNA topoisomerase II binding protein 1 [Source:HGNC Symbol;Acc:HGNC:17008]
ENSG00000219438	-0,3617281	3,79151375	2,86836293	0,12366909	0,40175265	TAFA5	TAFA chemokine like family member 5 [Source:HGNC Symbol;Acc:HGNC:21592]
ENSG00000099769	0,67010258	0,01687803	2,86753299	0,12371677	0,40179577	IGFALS	insulin like growth factor binding protein acid labile subunit [Source:HGNC Symbol;Acc:HGNC:5468]
ENSG00000104626	0,27555079	3,35914383	2,86717463	0,12373736	0,40179577	ERI1	exoribonuclease 1 [Source:HGNC Symbol;Acc:HGNC:23994]
ENSG00000112210	0,4041252	2,8989699	2,86597334	0,12380644	0,40193073	RAB23	RAB23, member RAS oncogene family [Source:HGNC Symbol;Acc:HGNC:14263]
ENSG00000106853	-0,2490799	4,20954664	2,86487976	0,12386936	0,4019628	PTGR1	prostaglandin reductase 1 [Source:HGNC Symbol;Acc:HGNC:18429]
ENSG00000159592	0,24922753	5,30538392	2,86484529	0,12387135	0,4019628	GPBP1L1	GC-rich promoter binding protein 1 like 1 [Source:HGNC Symbol;Acc:HGNC:28843]
ENSG00000144231	0,23973268	5,86235427	2,8641092	0,12391372	0,40201102	POLR2D	RNA polymerase II subunit D [Source:HGNC Symbol;Acc:HGNC:9191]
ENSG00000176393	-0,2600494	5,79600939	2,86332285	0,12395902	0,40206867	RNPEP	arginyl aminopeptidase [Source:HGNC Symbol;Acc:HGNC:10078]
ENSG00000121897	-0,2480186	4,10225844	2,86202415	0,12403387	0,40222214	LIAS	lipoic acid synthetase [Source:HGNC Symbol;Acc:HGNC:16429]
ENSG00000104549	0,24536495	7,94697565	2,86035186	0,12413033	0,40226709	SQLE	squalene epoxidase [Source:HGNC Symbol;Acc:HGNC:11279]
ENSG00000142166	0,23156595	5,06613809	2,86041698	0,12412658	0,40226709	IFNAR1	interferon alpha and beta receptor subunit 1 [Source:HGNC Symbol;Acc:HGNC:5432]
ENSG00000213246	-0,3139947	2,4161639	2,86071715	0,12410925	0,40226709	SUPT4H1	SPT4 homolog, DSIF elongation factor subunit [Source:HGNC Symbol;Acc:HGNC:11467]
ENSG00000156599	0,25416125	6,1027714	2,85848904	0,1242379	0,40244331	ZDHHC5	zinc finger DHHC-type palmitoyltransferase 5 [Source:HGNC Symbol;Acc:HGNC:18472]
ENSG00000160318	-0,3459458	1,94228383	2,85845611	0,12423981	0,40244331	CLDND2	claudin domain containing 2 [Source:HGNC Symbol;Acc:HGNC:28511]
ENSG00000069974	-0,3329077	2,83903347	2,85633022	0,12436272	0,40259415	RAB27A	RAB27A, member RAS oncogene family [Source:HGNC Symbol;Acc:HGNC:9766]
ENSG00000095321	-0,2710186	5,54645693	2,8524147	0,12458951	0,40259415	CRAT	carnitine O-acetyltransferase [Source:HGNC Symbol;Acc:HGNC:2342]
ENSG00000101670	0,32093052	5,09573268	2,85483196	0,12444944	0,40259415	LIPG	lipase G, endothelial type [Source:HGNC Symbol;Acc:HGNC:6623]
ENSG00000105655	-0,2938162	4,8461597	2,85509699	0,12443409	0,40259415	ISYNA1	inositol-3-phosphate synthase 1 [Source:HGNC Symbol;Acc:HGNC:29821]
ENSG00000109062	0,24970588	8,55142749	2,8560367	0,1243797	0,40259415	SLC9A3R1	SLC9A3 regulator 1 [Source:HGNC Symbol;Acc:HGNC:11075]
ENSG00000135776	0,24336051	5,20214283	2,8529661	0,12455754	0,40259415	ABCB10	ATP binding cassette subfamily B member 10 [Source:HGNC Symbol;Acc:HGNC:41]
ENSG00000139714	0,51339823	0,44996747	2,85261667	0,1245778	0,40259415	MORN3	MORN repeat containing 3 [Source:HGNC Symbol;Acc:HGNC:29807]
ENSG00000162384	-0,2306155	5,40286239	2,85388297	0,1245044	0,40259415	CZIB	CXXC motif containing zinc binding protein [Source:HGNC Symbol;Acc:HGNC:26059]
ENSG00000176973	0,62763311	-0,4092932	2,85530166	0,12442224	0,40259415	FAM89B	family with sequence similarity 89 member B [Source:HGNC Symbol;Acc:HGNC:16708]
ENSG00000182093	0,36755252	3,37326676	2,85286488	0,12456341	0,40259415	GET1	guided entry of tail-anchored proteins factor 1 [Source:HGNC Symbol;Acc:HGNC:12790]
ENSG00000283627	0,46824168	0,42394135	2,85362576	0,12451931	0,40259415	AL137785.1	novel transcript
ENSG00000260274	-0,448671	0,79189455	2,85025937	0,12471458	0,40290916	AC068338.2	novel transcript
ENSG00000092330	-0,2582111	5,80529145	2,84794903	0,12484882	0,40295408	TINF2	TERF1 interacting nuclear factor 2 [Source:HGNC Symbol;Acc:HGNC:11824]
ENSG00000117151	-0,3431234	2,11196573	2,84820557	0,1248339	0,40295408	CTBS	chitobiase [Source:HGNC Symbol;Acc:HGNC:2496]
ENSG00000120438	0,22378846	9,12506165	2,84854212	0,12481434	0,40295408	TCP1	t-complex 1 [Source:HGNC Symbol;Acc:HGNC:11655]
ENSG00000159176	-0,2251705	5,64730412	2,84771867	0,12486221	0,40295408	CSRP1	cysteine and glycine rich protein 1 [Source:HGNC Symbol;Acc:HGNC:2469]
ENSG00000175224	0,26904998	6,05187196	2,84764677	0,12486639	0,40295408	ATG13	autophagy related 13 [Source:HGNC Symbol;Acc:HGNC:29091]
ENSG00000272606	-0,5521614	0,28874824	2,84612089	0,12495517	0,40315152	AC015982.2	novel transcript
ENSG00000131653	-0,2511078	5,98435433	2,84551638	0,12499036	0,40317603	TRAF7	TNF receptor associated factor 7 [Source:HGNC Symbol;Acc:HGNC:20456]
ENSG00000067066	-0,2875445	3,7746209	2,84363239	0,12510013	0,40344101	SP100	SP100 nuclear antigen [Source:HGNC Symbol;Acc:HGNC:11206]
ENSG00000167074	0,26872619	3,39428237	2,84280576	0,12514833	0,40344891	TEF	TEF transcription factor, PAR bZIP family member [Source:HGNC Symbol;Acc:HGNC:11722]

ENSG00000176054	-0,4489487	0,91817066	2,84264323	0,12515781	0,40344891	RPL23P2	ribosomal protein L23 pseudogene 2 [Source:HGNC Symbol;Acc:HGNC:10324]
ENSG00000182512	0,23328194	7,07498536	2,83967846	0,1253309	0,40391773	GLRX5	glutaredoxin 5 [Source:HGNC Symbol;Acc:HGNC:20134]
ENSG00000102984	-0,2826555	2,75261148	2,83777866	0,12544197	0,40414838	ZNF821	zinc finger protein 821 [Source:HGNC Symbol;Acc:HGNC:28043]
ENSG00000131375	0,24822975	4,68013302	2,83750822	0,12545779	0,40414838	CAPN7	calpain 7 [Source:HGNC Symbol;Acc:HGNC:1484]
ENSG00000085449	0,256745	4,3083238	2,83623595	0,12553226	0,40417267	WDFY1	WD repeat and FYVE domain containing 1 [Source:HGNC Symbol;Acc:HGNC:20451]
ENSG00000091527	-0,248711	5,77228791	2,83327932	0,12570554	0,40417267	CDV3	CDV3 homolog [Source:HGNC Symbol;Acc:HGNC:26928]
ENSG00000101452	0,24203352	5,20179543	2,83265796	0,12574199	0,40417267	DHX35	DEAH-box helicase 35 [Source:HGNC Symbol;Acc:HGNC:15861]
ENSG00000145703	0,37261244	6,24099721	2,8354838	0,12557631	0,40417267	IQGAP2	IQ motif containing GTPase activating protein 2 [Source:HGNC Symbol;Acc:HGNC:6111]
ENSG00000205639	-0,8839173	-1,4108316	2,83414824	0,12565458	0,40417267	MFSD2B	major facilitator superfamily domain containing 2B [Source:HGNC Symbol;Acc:HGNC:37207]
ENSG00000237424	0,46313501	0,37870437	2,83645792	0,12551927	0,40417267	FOXD2-AS1	FOXD2 adjacent opposite strand RNA 1 [Source:HGNC Symbol;Acc:HGNC:44256]
ENSG00000258708	-0,313448	1,73259919	2,83301131	0,12572126	0,40417267	SLC25A21-AS1	SLC25A21 antisense RNA 1 [Source:HGNC Symbol;Acc:HGNC:44298]
ENSG00000259500	-1,1048968	-2,1301152	2,83478271	0,12561739	0,40417267	KRT8P24	keratin 8 pseudogene 24 [Source:HGNC Symbol;Acc:HGNC:33376]
ENSG00000287104	0,73495254	-0,6694006	2,83357061	0,12568845	0,40417267	AC097382.3	novel transcript, antisense to TBC1D14
ENSG00000287356	0,80719301	-1,4558808	2,83370486	0,12568058	0,40417267	AL590822.3	novel transcript, antisense to MORN1
ENSG00000113739	-0,3701024	7,2284491	2,82918069	0,12594625	0,40474017	STC2	stanniocalcin 2 [Source:HGNC Symbol;Acc:HGNC:11374]
ENSG00000154359	-0,2823006	2,68930816	2,82829332	0,12599845	0,40481885	LONRF1	LON peptidase N-terminal domain and ring finger 1 [Source:HGNC Symbol;Acc:HGNC:26302]
ENSG00000171861	0,23865644	5,49069875	2,82761354	0,12603845	0,40485834	MRM3	mitochondrial rRNA methyltransferase 3 [Source:HGNC Symbol;Acc:HGNC:18485]
ENSG00000138459	0,2706212	3,76625931	2,82697104	0,12607627	0,40489081	SLC35A5	solute carrier family 35 member A5 [Source:HGNC Symbol;Acc:HGNC:20792]
ENSG00000206573	-0,3440029	3,05842262	2,82578116	0,12614636	0,40493786	THUMPD3-AS1	THUMPD3 antisense RNA 1 [Source:HGNC Symbol;Acc:HGNC:44478]
ENSG00000271781	-1,1582284	-1,606236	2,82607953	0,12612878	0,40493786	AC026740.1	novel transcript, antisense to TPPP
ENSG00000272275	1,11711309	-2,0845577	2,82405361	0,12624821	0,40517576	AC092687.3	novel transcript
ENSG00000170275	0,24141695	7,25859253	2,82327995	0,12629385	0,40523323	CRTAP	cartilage associated protein [Source:HGNC Symbol;Acc:HGNC:2379]
ENSG00000198894	0,33655669	2,37719986	2,82271644	0,12632711	0,40525094	CIPC	CLOCK interacting pacemaker [Source:HGNC Symbol;Acc:HGNC:20365]
ENSG00000162063	0,2759244	5,26336788	2,82081203	0,1264396	0,40552274	CCNF	cyclin F [Source:HGNC Symbol;Acc:HGNC:1591]
ENSG00000100311	0,71798426	0,31306343	2,81918388	0,12653587	0,4056846	PDGFB	platelet derived growth factor subunit B [Source:HGNC Symbol;Acc:HGNC:8800]
ENSG00000119139	0,48203782	0,86892456	2,81901931	0,1265456	0,4056846	TJP2	tight junction protein 2 [Source:HGNC Symbol;Acc:HGNC:11828]
ENSG00000284634	0,87113392	-1,8158857	2,8161831	0,12671355	0,40613387	AC092821.3	novel transcript
ENSG00000104904	-0,3081539	9,42230828	2,81204834	0,1269589	0,40633661	OAZ1	ornithine decarboxylase antizyme 1 [Source:HGNC Symbol;Acc:HGNC:8095]
ENSG00000107362	0,28894426	3,47860633	2,81379013	0,12685547	0,40633661	ABHD17B	abhydrolase domain containing 17B, depalmitoylase [Source:HGNC Symbol;Acc:HGNC:24278]
ENSG00000116793	-0,3075846	3,56746977	2,81161215	0,12698481	0,40633661	PHTF1	putative homeodomain transcription factor 1 [Source:HGNC Symbol;Acc:HGNC:8939]
ENSG00000121964	-0,3741783	1,91904724	2,80959145	0,12710497	0,40633661	GTDC1	glycosyltransferase like domain containing 1 [Source:HGNC Symbol;Acc:HGNC:20887]
ENSG00000125122	-0,8637152	-0,4748168	2,81053663	0,12704875	0,40633661	LRRC29	leucine rich repeat containing 29 [Source:HGNC Symbol;Acc:HGNC:13605]
ENSG00000133789	-0,3267048	3,58336547	2,80984074	0,12709014	0,40633661	SWAP70	switching B cell complex subunit SWAP70 [Source:HGNC Symbol;Acc:HGNC:17070]
ENSG00000144524	0,33949458	5,48696122	2,81249598	0,1269323	0,40633661	COPS7B	COP9 signalosome subunit 7B [Source:HGNC Symbol;Acc:HGNC:16760]
ENSG00000160948	-0,2633709	5,16869469	2,81091311	0,12702637	0,40633661	VPS28	VPS28 subunit of ESCRT-I [Source:HGNC Symbol;Acc:HGNC:18178]
ENSG00000166925	0,24547662	4,3348827	2,80996808	0,12708257	0,40633661	TSC22D4	TSC22 domain family member 4 [Source:HGNC Symbol;Acc:HGNC:21696]
ENSG00000198468	-0,3624353	2,20395053	2,81094796	0,12702429	0,40633661	FLVCR1-DT	FLVCR1 divergent transcript [Source:HGNC Symbol;Acc:HGNC:39077]
ENSG00000225733	0,29408001	4,27119387	2,80949742	0,12711057	0,40633661	FGD5-AS1	FGD5 antisense RNA 1 [Source:HGNC Symbol;Acc:HGNC:40410]
ENSG00000259005	0,79842188	-1,1843834	2,81142239	0,12699609	0,40633661	AC005479.1	novel transcript
ENSG00000104823	-0,2342179	6,78083357	2,80540792	0,12735421	0,40650694	ECH1	enoyl-CoA hydratase 1 [Source:HGNC Symbol;Acc:HGNC:3149]
ENSG00000106682	-0,2241712	7,26839866	2,80500098	0,12737849	0,40650694	EIF4H	eukaryotic translation initiation factor 4H [Source:HGNC Symbol;Acc:HGNC:12741]
ENSG00000139644	0,23480338	9,49800441	2,80660903	0,12728259	0,40650694	TMBIM6	transmembrane BAX inhibitor motif containing 6 [Source:HGNC Symbol;Acc:HGNC:11723]
ENSG00000145781	0,29931229	3,73178777	2,80486738	0,12738646	0,40650694	COMMD10	COMM domain containing 10 [Source:HGNC Symbol;Acc:HGNC:30201]
ENSG00000160447	-0,2311852	4,90591204	2,80630387	0,12730078	0,40650694	PKN3	protein kinase N3 [Source:HGNC Symbol;Acc:HGNC:17999]

ENSG00000168237	-0,313716	5,80174951	2,80765253	0,12722041	0,40650694	GLYCTK	glycerate kinase [Source:HGNC Symbol;Acc:HGNC:24247]
ENSG00000177335	-0,8082155	-0,2685612	2,80767708	0,12721895	0,40650694	C8orf31	chromosome 8 open reading frame 31 [Source:HGNC Symbol;Acc:HGNC:26731]
ENSG00000198074	-0,3010617	7,25586352	2,80543811	0,12735241	0,40650694	AKR1B10	aldo-keto reductase family 1 member B10 [Source:HGNC Symbol;Acc:HGNC:382]
ENSG00000154803	0,24338552	4,20808652	2,80414952	0,1274293	0,40655485	FLCN	folliculin [Source:HGNC Symbol;Acc:HGNC:27310]
ENSG00000163704	-0,8684152	0,03719323	2,801801	0,1275696	0,4069136	PRRT3	proline rich transmembrane protein 3 [Source:HGNC Symbol;Acc:HGNC:26591]
ENSG00000196517	0,30482482	3,18604688	2,80107246	0,12761316	0,40696369	SLC6A9	solute carrier family 6 member 9 [Source:HGNC Symbol;Acc:HGNC:11056]
ENSG00000197063	0,25221077	4,99801872	2,80058294	0,12764244	0,40696823	MAFG	MAF bZIP transcription factor G [Source:HGNC Symbol;Acc:HGNC:6781]
ENSG00000229932	0,81384751	-1,3372773	2,79953218	0,12770532	0,40707988	YWHAZP3	tyrosine 3-monooxygenase/tryptophan 5-monooxygenase activation protein zeta pseudogene 3 [Source:HGNC Sy
ENSG00000186591	0,2522738	3,88082282	2,79888979	0,12774379	0,40711365	UBE2H	ubiquitin conjugating enzyme E2 H [Source:HGNC Symbol;Acc:HGNC:12484]
ENSG00000170915	-0,2676957	5,9485942	2,79746731	0,12782901	0,4072964	PAQR8	progestin and adipoQ receptor family member 8 [Source:HGNC Symbol;Acc:HGNC:15708]
ENSG00000024048	0,344601	4,95752611	2,79453543	0,12800489	0,40776788	UBR2	ubiquitin protein ligase E3 component n-recognin 2 [Source:HGNC Symbol;Acc:HGNC:21289]
ENSG00000110955	0,34593029	10,4447097	2,79327133	0,12808083	0,40779136	ATP5F1B	ATP synthase F1 subunit beta [Source:HGNC Symbol;Acc:HGNC:830]
ENSG00000152137	-1,0929831	-1,1820195	2,79339678	0,12807329	0,40779136	HSPB8	heat shock protein family B (small) member 8 [Source:HGNC Symbol;Acc:HGNC:30171]
ENSG00000171681	-0,3532671	1,65438813	2,79301873	0,128096	0,40779136	ATF7IP	activating transcription factor 7 interacting protein [Source:HGNC Symbol;Acc:HGNC:20092]
ENSG00000140465	-0,5927109	1,23261065	2,79252627	0,12812561	0,40779673	CYP1A1	cytochrome P450 family 1 subfamily A member 1 [Source:HGNC Symbol;Acc:HGNC:2595]
ENSG00000154518	-0,2509329	9,19747736	2,7919218	0,12816195	0,40782356	ATP5MC3	ATP synthase membrane subunit c locus 3 [Source:HGNC Symbol;Acc:HGNC:843]
ENSG00000104679	-0,2333788	5,32630872	2,79054242	0,12824494	0,40799876	R3HCC1	R3H domain and coiled-coil containing 1 [Source:HGNC Symbol;Acc:HGNC:27329]
ENSG00000198911	0,25070212	7,40436171	2,78920998	0,12832517	0,40816512	SREBF2	sterol regulatory element binding transcription factor 2 [Source:HGNC Symbol;Acc:HGNC:11290]
ENSG00000261879	1,15780856	-1,9818285	2,7880096	0,1283975	0,4083063	AC087500.1	novel transcript, antisense to C17orf87
ENSG00000235587	-0,3456576	2,95138764	2,78746735	0,1284302	0,40832138	GAPDHP65	glyceraldehyde 3 phosphate dehydrogenase pseudogene 65 [Source:HGNC Symbol;Acc:HGNC:4143]
ENSG00000160293	0,33376972	6,11084599	2,78699189	0,12845887	0,40832368	VAV2	vav guanine nucleotide exchange factor 2 [Source:HGNC Symbol;Acc:HGNC:12658]
ENSG00000008382	-0,3599176	4,47095125	2,78572575	0,12853527	0,40847766	MPND	MPN domain containing [Source:HGNC Symbol;Acc:HGNC:25934]
ENSG00000158636	0,35104718	2,33327285	2,78524334	0,1285644	0,40848136	EMSY	EMSY transcriptional repressor, BRCA2 interacting [Source:HGNC Symbol;Acc:HGNC:18071]
ENSG00000145362	-0,5802895	1,6549886	2,78461783	0,12860217	0,40851254	ANK2	ankyrin 2 [Source:HGNC Symbol;Acc:HGNC:493]
ENSG00000137731	-0,363528	1,7509472	2,78197555	0,12876191	0,4087261	FXYD2	FXYD domain containing ion transport regulator 2 [Source:HGNC Symbol;Acc:HGNC:4026]
ENSG00000176532	0,28332213	2,95134855	2,78215052	0,12875132	0,4087261	PRR15	proline rich 15 [Source:HGNC Symbol;Acc:HGNC:22310]
ENSG00000236751	-0,6104354	-0,1231591	2,78165483	0,12878131	0,4087261	LINC01186	long intergenic non-protein coding RNA 1186 [Source:HGNC Symbol;Acc:HGNC:49573]
ENSG00000284948	-0,6016204	-0,3295608	2,78204658	0,12875761	0,4087261	AC107959.4	novel transcript
ENSG00000112282	0,31750681	3,75406079	2,78105201	0,1288178	0,4087498	MED23	mediator complex subunit 23 [Source:HGNC Symbol;Acc:HGNC:2372]
ENSG00000162065	0,29966457	3,17489105	2,780607	0,12884474	0,4087498	TBC1D24	TBC1 domain family member 24 [Source:HGNC Symbol;Acc:HGNC:29203]
ENSG00000167720	-0,4419651	1,72859135	2,77836838	0,12898039	0,40909129	SRR	serine racemase [Source:HGNC Symbol;Acc:HGNC:14398]
ENSG00000106268	-0,2317401	5,08051517	2,77652352	0,12909231	0,40922919	NUDT1	nudix hydrolase 1 [Source:HGNC Symbol;Acc:HGNC:8048]
ENSG00000157827	0,29596979	5,21288889	2,77718044	0,12905244	0,40922919	FMNL2	formin like 2 [Source:HGNC Symbol;Acc:HGNC:18267]
ENSG00000242169	-0,6912486	-1,2361764	2,77626668	0,1291079	0,40922919	AC093789.1	ribosomal protein L7a (RPL7A) pseudogene
ENSG00000149806	-0,2490768	8,95490663	2,77518209	0,12917377	0,40934916	FAU	FAU ubiquitin like and ribosomal protein S30 fusion [Source:HGNC Symbol;Acc:HGNC:3597]
ENSG00000104341	0,25144251	8,32490686	2,77360804	0,12926944	0,40946963	LAPTM4B	lysosomal protein transmembrane 4 beta [Source:HGNC Symbol;Acc:HGNC:13646]
ENSG00000104671	-0,2813215	3,51814163	2,7717916	0,12937996	0,40946963	DCTN6	dynactin subunit 6 [Source:HGNC Symbol;Acc:HGNC:16964]
ENSG00000108671	-0,2796812	7,78155772	2,7718179	0,12937836	0,40946963	PSMD11	proteasome 26S subunit, non-ATPase 11 [Source:HGNC Symbol;Acc:HGNC:9556]
ENSG00000131174	-0,2760851	7,54351056	2,77387748	0,12925306	0,40946963	COX7B	cytochrome c oxidase subunit 7B [Source:HGNC Symbol;Acc:HGNC:2291]
ENSG00000151353	0,23170698	6,33143889	2,77248602	0,12933769	0,40946963	TMEM18	transmembrane protein 18 [Source:HGNC Symbol;Acc:HGNC:25257]
ENSG00000249592	-0,5498363	0,50070339	2,7722889	0,12934969	0,40946963	AC139887.2	novel transcript, antisense to PCGF3
ENSG00000153936	-0,2297526	5,4762269	2,76890676	0,12955573	0,40975962	HS2ST1	heparan sulfate 2-O-sulfotransferase 1 [Source:HGNC Symbol;Acc:HGNC:5193]
ENSG00000198815	0,33575985	3,60234115	2,76917451	0,12953941	0,40975962	FOXJ3	forkhead box J3 [Source:HGNC Symbol;Acc:HGNC:29178]
ENSG00000243592	-1,0617626	-1,6184687	2,76982415	0,1294998	0,40975962	RPL17P22	ribosomal protein L17 pseudogene 22 [Source:HGNC Symbol;Acc:HGNC:35761]

ENSG00000119402	0,28149076	5,03594497	2,76577697	0,12974678	0,41009751	FBXW2	F-box and WD repeat domain containing 2 [Source:HGNC Symbol;Acc:HGNC:13608]
ENSG00000173517	0,55473906	2,39640521	2,76634968	0,12971179	0,41009751	PEAK1	pseudopodium enriched atypical kinase 1 [Source:HGNC Symbol;Acc:HGNC:29431]
ENSG00000198646	0,44287367	4,70773729	2,76620156	0,12972084	0,41009751	NCOA6	nuclear receptor coactivator 6 [Source:HGNC Symbol;Acc:HGNC:15936]
ENSG00000181418	-0,3943765	1,16304413	2,76514366	0,12978548	0,4101311	DDN	dendrin [Source:HGNC Symbol;Acc:HGNC:24458]
ENSG00000166164	0,21649659	7,43971799	2,76445751	0,12982743	0,41017494	BRD7	bromodomain containing 7 [Source:HGNC Symbol;Acc:HGNC:14310]
ENSG00000099860	-0,4083233	3,95601163	2,76341688	0,12989108	0,41019862	GADD45B	growth arrest and DNA damage inducible beta [Source:HGNC Symbol;Acc:HGNC:4096]
ENSG00000257702	-0,2943927	4,79903134	2,76346256	0,12988829	0,41019862	LBX2-AS1	LBX2 antisense RNA 1 [Source:HGNC Symbol;Acc:HGNC:25136]
ENSG00000142552	-0,3788709	1,73695013	2,76225353	0,12996229	0,41024612	RCN3	reticulocalbin 3 [Source:HGNC Symbol;Acc:HGNC:21145]
ENSG00000260219	-0,6195615	0,64680974	2,76259106	0,12994162	0,41024612	CD2BP2-DT	CD2BP2 divergent transcript [Source:HGNC Symbol;Acc:HGNC:53029]
ENSG00000079616	0,29848647	7,23190927	2,75843245	0,13019652	0,41056037	KIF22	kinesin family member 22 [Source:HGNC Symbol;Acc:HGNC:6391]
ENSG00000094914	0,26056264	6,34839687	2,75877732	0,13017536	0,41056037	AAAS	aladin WD repeat nucleoporin [Source:HGNC Symbol;Acc:HGNC:13666]
ENSG00000112130	0,34870415	5,30584064	2,75804456	0,13022033	0,41056037	RNF8	ring finger protein 8 [Source:HGNC Symbol;Acc:HGNC:10071]
ENSG00000130513	-0,6143433	9,62155789	2,75795261	0,13022598	0,41056037	GDF15	growth differentiation factor 15 [Source:HGNC Symbol;Acc:HGNC:30142]
ENSG00000176890	0,23417351	8,30214831	2,75787961	0,13023046	0,41056037	TYMS	thymidylate synthetase [Source:HGNC Symbol;Acc:HGNC:12441]
ENSG00000234072	-0,2950796	3,18563473	2,75867659	0,13018154	0,41056037	AC074117.1	novel transcript, antisense to GTF3C2 and EIF2B4
ENSG00000178980	0,26641042	4,05417467	2,75676444	0,13029895	0,41059907	SELENOW	selenoprotein W [Source:HGNC Symbol;Acc:HGNC:10752]
ENSG00000255690	0,63667412	-0,5130628	2,75686703	0,13029264	0,41059907	TRIL	TLR4 interactor with leucine rich repeats [Source:HGNC Symbol;Acc:HGNC:22200]
ENSG00000145545	-0,350305	2,28836824	2,75551143	0,13037595	0,41075314	SRD5A1	steroid 5 alpha-reductase 1 [Source:HGNC Symbol;Acc:HGNC:11284]
ENSG00000103187	-0,2523316	5,27623007	2,75394159	0,13047252	0,41092837	COTL1	coactosin like F-actin binding protein 1 [Source:HGNC Symbol;Acc:HGNC:18304]
ENSG00000134882	0,24001354	7,09097571	2,75323581	0,13051596	0,41092837	UBAC2	UBA domain containing 2 [Source:HGNC Symbol;Acc:HGNC:20486]
ENSG00000198700	0,27671966	5,68253659	2,75354343	0,13049702	0,41092837	IPO9	importin 9 [Source:HGNC Symbol;Acc:HGNC:19425]
ENSG00000162976	-0,515505	0,80078292	2,75215554	0,13058249	0,4109607	SLC66A3	solute carrier family 66 member 3 [Source:HGNC Symbol;Acc:HGNC:28503]
ENSG00000196428	0,38691844	2,45693611	2,75235236	0,13057036	0,4109607	TSC22D2	TSC22 domain family member 2 [Source:HGNC Symbol;Acc:HGNC:29095]
ENSG00000115520	0,25459544	4,9358776	2,74920853	0,13076421	0,41120306	COQ10B	coenzyme Q10B [Source:HGNC Symbol;Acc:HGNC:25819]
ENSG00000157833	1,08128112	-0,6266057	2,75029326	0,13069728	0,41120306	GAREM2	GRB2 associated regulator of MAPK1 subtype 2 [Source:HGNC Symbol;Acc:HGNC:27172]
ENSG00000197859	-0,4554035	3,30444784	2,7486372	0,13079948	0,41120306	ADAMTSL2	ADAMTS like 2 [Source:HGNC Symbol;Acc:HGNC:14631]
ENSG00000221978	0,27504246	5,63505097	2,74862494	0,13080023	0,41120306	CCNL2	cyclin L2 [Source:HGNC Symbol;Acc:HGNC:20570]
ENSG00000230701	0,75426623	-0,7316151	2,74922738	0,13076305	0,41120306	FBXW4P1	F-box and WD repeat domain containing 4 pseudogene 1 [Source:HGNC Symbol;Acc:HGNC:13609]
ENSG00000102897	-0,3070177	5,58225527	2,74783019	0,13084931	0,41126885	LYRM1	LYR motif containing 1 [Source:HGNC Symbol;Acc:HGNC:25074]
ENSG00000144485	-0,3654839	5,49587105	2,74683485	0,13091081	0,41137364	HES6	hes family bHLH transcription factor 6 [Source:HGNC Symbol;Acc:HGNC:18254]
ENSG00000257594	1,0159629	-1,4236166	2,74438845	0,13106213	0,41176057	GALNT4	polypeptide N-acetylgalactosaminyltransferase 4 [Source:HGNC Symbol;Acc:HGNC:4126]
ENSG00000067836	-0,320223	2,88695776	2,74338585	0,13112421	0,4117785	ROGDI	rogdi atypical leucine zipper [Source:HGNC Symbol;Acc:HGNC:29478]
ENSG00000165643	1,57136452	-1,920523	2,74354218	0,13111453	0,4117785	SOHLH1	spermatogenesis and oogenesis specific basic helix-loop-helix 1 [Source:HGNC Symbol;Acc:HGNC:27845]
ENSG00000056736	0,21900509	7,3247577	2,74016781	0,13132372	0,41217592	IL17RB	interleukin 17 receptor B [Source:HGNC Symbol;Acc:HGNC:18015]
ENSG00000143369	-0,6687547	-0,1981911	2,73952506	0,13136362	0,41217592	ECM1	extracellular matrix protein 1 [Source:HGNC Symbol;Acc:HGNC:3153]
ENSG00000180884	0,27953125	2,90835252	2,74079697	0,13128468	0,41217592	ZNF792	zinc finger protein 792 [Source:HGNC Symbol;Acc:HGNC:24751]
ENSG00000230291	-0,3371887	1,51287623	2,7397716	0,13134831	0,41217592	AC078817.1	ribosomal protein L26 (RPL26) pseudogene
ENSG00000182612	-0,4844829	0,23228982	2,7385047	0,13142698	0,41222795	TSPAN10	tetraspanin 10 [Source:HGNC Symbol;Acc:HGNC:29942]
ENSG00000187109	-0,2649066	7,3385192	2,73698741	0,13152128	0,41222795	NAP1L1	nucleosome assembly protein 1 like 1 [Source:HGNC Symbol;Acc:HGNC:7637]
ENSG00000221963	0,37753137	2,83163763	2,73756175	0,13148558	0,41222795	APOL6	apolipoprotein L6 [Source:HGNC Symbol;Acc:HGNC:14870]
ENSG00000233328	-0,3900444	1,37824723	2,73706798	0,13151627	0,41222795	PFN1P1	profilin 1 pseudogene 1 [Source:HGNC Symbol;Acc:HGNC:42989]
ENSG00000257790	-0,3447367	1,58992745	2,73760538	0,13148287	0,41222795	EIF4A1P4	eukaryotic translation initiation factor 4A1 pseudogene 4 [Source:HGNC Symbol;Acc:HGNC:37923]
ENSG00000120688	0,26995656	4,16038183	2,73512567	0,13163711	0,41250248	WBP4	WW domain binding protein 4 [Source:HGNC Symbol;Acc:HGNC:12739]
ENSG00000178685	0,27826366	4,07588652	2,7344208	0,131681	0,41255152	PARP10	poly(ADP-ribose) polymerase family member 10 [Source:HGNC Symbol;Acc:HGNC:25895]

ENSG00000188290	-0,3941528	1,07948468	2,73390158	0,13171334	0,41256436	HES4	hes family bHLH transcription factor 4 [Source:HGNC Symbol;Acc:HGNC:24149]
ENSG00000273716	-0,468837	0,49863866	2,73342504	0,13174303	0,41256891	AC092670.1	ribosomal protein L41 (RPL41) pseudogene
ENSG00000197748	-0,6705651	-0,3727658	2,73289302	0,13177619	0,4125843	CFAP43	cilia and flagella associated protein 43 [Source:HGNC Symbol;Acc:HGNC:26684]
ENSG00000104731	-0,3225759	4,92966399	2,73227543	0,13181469	0,41261643	KLHDC4	kelch domain containing 4 [Source:HGNC Symbol;Acc:HGNC:25272]
ENSG00000109466	0,32007625	2,75332125	2,73173029	0,13184869	0,41263444	KLHL2	kelch like family member 2 [Source:HGNC Symbol;Acc:HGNC:6353]
ENSG00000103269	-0,5542729	-0,3875445	2,72542104	0,13224302	0,4128233	RHBDL1	rhomboid like 1 [Source:HGNC Symbol;Acc:HGNC:10007]
ENSG00000117394	0,34586856	10,4769718	2,7274578	0,13211556	0,4128233	SLC2A1	solute carrier family 2 member 1 [Source:HGNC Symbol;Acc:HGNC:11005]
ENSG00000134686	-0,2152904	6,74934093	2,72657728	0,13217064	0,4128233	PHC2	polyhomeotic homolog 2 [Source:HGNC Symbol;Acc:HGNC:3183]
ENSG00000135316	0,28264346	7,17863196	2,72541181	0,1322436	0,4128233	SYNCRIP	synaptotagmin binding cytoplasmic RNA interacting protein [Source:HGNC Symbol;Acc:HGNC:16918]
ENSG00000151491	0,30622431	5,89896081	2,72972431	0,1319739	0,4128233	EPS8	epidermal growth factor receptor pathway substrate 8 [Source:HGNC Symbol;Acc:HGNC:3420]
ENSG00000166189	-0,2529749	4,41346588	2,72752219	0,13211153	0,4128233	HPS6	HPS6 biogenesis of lysosomal organelles complex 2 subunit 3 [Source:HGNC Symbol;Acc:HGNC:18817]
ENSG00000166348	-0,4616846	3,34397652	2,72633623	0,13218573	0,4128233	USP54	ubiquitin specific peptidase 54 [Source:HGNC Symbol;Acc:HGNC:23513]
ENSG00000184792	0,82160887	-1,3498793	2,72768201	0,13210153	0,4128233	OSBP2	oxysterol binding protein 2 [Source:HGNC Symbol;Acc:HGNC:8504]
ENSG00000197603	0,43583446	1,97999768	2,72599563	0,13220704	0,4128233	CPLANE1	ciliogenesis and planar polarity effector 1 [Source:HGNC Symbol;Acc:HGNC:25801]
ENSG00000215252	0,43272764	1,7986276	2,72841595	0,13205565	0,4128233	GOLGA8B	golgin A8 family member B [Source:HGNC Symbol;Acc:HGNC:31973]
ENSG00000232442	-0,3481011	1,75285768	2,72533932	0,13224814	0,4128233	MHENCR	melanoma highly expressed competing endogenous lncRNA for miR-425 and miR-489 [Source:HGNC Symbol;Acc:[illegible]
ENSG00000244485	-0,5525153	-0,4483432	2,72891047	0,13202474	0,4128233	RPL18P13	ribosomal protein L18 pseudogene 13 [Source:HGNC Symbol;Acc:HGNC:35599]
ENSG00000103024	-0,2681956	3,31117083	2,72426853	0,13231521	0,41294445	NME3	NME/NM23 nucleoside diphosphate kinase 3 [Source:HGNC Symbol;Acc:HGNC:7851]
ENSG00000120539	0,24672589	3,92683688	2,72112987	0,13251209	0,41347054	MASTL	microtubule associated serine/threonine kinase like [Source:HGNC Symbol;Acc:HGNC:19042]
ENSG00000166002	-0,3312367	3,58207198	2,71981666	0,13259457	0,41363956	SMCO4	single-pass membrane protein with coiled-coil domains 4 [Source:HGNC Symbol;Acc:HGNC:24810]
ENSG00000177721	-0,5935608	0,46022905	2,71911492	0,13263867	0,41368881	ANXA2R	annexin A2 receptor [Source:HGNC Symbol;Acc:HGNC:33463]
ENSG00000176410	-0,2528291	4,28084118	2,71819882	0,13269628	0,41369183	DNAJC30	DnaJ heat shock protein family (Hsp40) member C30 [Source:HGNC Symbol;Acc:HGNC:16410]
ENSG00000279406	0,75722037	-0,1861576	2,71855643	0,13267379	0,41369183	AL359183.1	TEC
ENSG00000211455	0,26970568	3,93019972	2,71419838	0,1329482	0,4143888	STK38L	serine/threonine kinase 38 like [Source:HGNC Symbol;Acc:HGNC:17848]
ENSG00000151292	0,24752085	3,25613123	2,70956357	0,13324085	0,41515634	CSNK1G3	casein kinase 1 gamma 3 [Source:HGNC Symbol;Acc:HGNC:2456]
ENSG00000273314	-0,7909863	-0,7328319	2,70939847	0,13325129	0,41515634	AC005229.4	novel transcript, antisense to CUL1
ENSG00000105404	-0,3369716	2,11393464	2,70737583	0,13337928	0,41546649	RABAC1	Rab acceptor 1 [Source:HGNC Symbol;Acc:HGNC:9794]
ENSG00000026103	0,29463716	4,43779238	2,70646386	0,13343703	0,41550049	FAS	Fas cell surface death receptor [Source:HGNC Symbol;Acc:HGNC:11920]
ENSG00000100804	-0,2141186	10,0198605	2,70630539	0,13344707	0,41550049	PSMB5	proteasome 20S subunit beta 5 [Source:HGNC Symbol;Acc:HGNC:9542]
ENSG00000173545	0,30348473	6,9221907	2,70496743	0,13353188	0,41567595	ZNF622	zinc finger protein 622 [Source:HGNC Symbol;Acc:HGNC:30958]
ENSG00000125037	0,23215212	6,8607416	2,70357505	0,13362021	0,41586229	EMC3	ER membrane protein complex subunit 3 [Source:HGNC Symbol;Acc:HGNC:23999]
ENSG00000112624	0,57602795	1,27020033	2,70121791	0,13376991	0,41623953	BICRAL	BRD4 interacting chromatin remodeling complex associated protein like [Source:HGNC Symbol;Acc:HGNC:21111]
ENSG00000055070	0,26612855	5,86777488	2,69806828	0,13397028	0,41624241	SZRD1	SUZ RNA binding domain containing 1 [Source:HGNC Symbol;Acc:HGNC:30232]
ENSG00000109063	-1,0025328	0,95339847	2,69830672	0,1339551	0,41624241	MYH3	myosin heavy chain 3 [Source:HGNC Symbol;Acc:HGNC:7573]
ENSG00000132481	-0,2445195	6,31857236	2,69963606	0,13387049	0,41624241	TRIM47	tripartite motif containing 47 [Source:HGNC Symbol;Acc:HGNC:19020]
ENSG00000160803	0,24066263	5,56300499	2,70053698	0,13381319	0,41624241	UBQLN4	ubiquilin 4 [Source:HGNC Symbol;Acc:HGNC:1237]
ENSG00000176153	-0,3046724	8,66510378	2,6990211	0,13390962	0,41624241	GPX2	glutathione peroxidase 2 [Source:HGNC Symbol;Acc:HGNC:4554]
ENSG00000260001	-0,7811682	-0,7199439	2,69963055	0,13387084	0,41624241	TGFBR3L	transforming growth factor beta receptor 3 like [Source:HGNC Symbol;Acc:HGNC:44152]
ENSG00000275441	-0,8308787	-1,1918552	2,6982008	0,13396184	0,41624241	AC020765.2	novel transcript, antisense to CCDC101
ENSG00000005001	-1,1165465	-1,7028025	2,69578313	0,1341159	0,41660624	PRSS22	serine protease 22 [Source:HGNC Symbol;Acc:HGNC:14368]
ENSG00000090932	-0,5234025	0,48678747	2,69525278	0,13414972	0,41662272	DLL3	delta like canonical Notch ligand 3 [Source:HGNC Symbol;Acc:HGNC:2909]
ENSG00000169621	0,45838625	1,62807156	2,69366974	0,13425075	0,41684787	APLF	aprataxin and PNKP like factor [Source:HGNC Symbol;Acc:HGNC:28724]
ENSG00000072571	-0,3162584	4,22279651	2,69073012	0,13443862	0,41694074	HMMR	hyaluronan mediated motility receptor [Source:HGNC Symbol;Acc:HGNC:5012]
ENSG00000082641	0,2906964	7,15929841	2,69082328	0,13443266	0,41694074	NFE2L1	nuclear factor, erythroid 2 like 1 [Source:HGNC Symbol;Acc:HGNC:7781]

ENSG00000138029	0,31730555	8,3567712	2,69098381	0,13442239	0,41694074	HADHB	hydroxyacyl-CoA dehydrogenase trifunctional multienzyme complex subunit beta [Source:HGNC Symbol;Acc:HGN
ENSG00000172531	-0,2213903	8,20689696	2,69206791	0,13435308	0,41694074	PPP1CA	protein phosphatase 1 catalytic subunit alpha [Source:HGNC Symbol;Acc:HGNC:9281]
ENSG00000179862	0,29724134	4,05272793	2,69033938	0,13446362	0,41694074	CITED4	Cbp/p300 interacting transactivator with Glu/Asp rich carboxy-terminal domain 4 [Source:HGNC Symbol;Acc:HGN
ENSG00000215482	0,52824549	-0,0585215	2,69007641	0,13448044	0,41694074	CALM2P3	calmodulin 2 pseudogene 3 [Source:HGNC Symbol;Acc:HGNC:1448]
ENSG00000275183	-0,5533485	1,60555198	2,69120598	0,13440818	0,41694074	LENG9	leukocyte receptor cluster member 9 [Source:HGNC Symbol;Acc:HGNC:16306]
ENSG00000072422	0,33428194	4,07663789	2,6893403	0,13452756	0,41699832	RHOBTB1	Rho related BTB domain containing 1 [Source:HGNC Symbol;Acc:HGNC:18738]
ENSG00000177054	0,33058989	3,47567025	2,68823021	0,13459865	0,41713019	ZDHHC13	zinc finger DHHC-type palmitoyltransferase 13 [Source:HGNC Symbol;Acc:HGNC:18413]
ENSG00000038002	0,27309128	4,54114058	2,68504733	0,13480276	0,41730618	AGA	aspartylglucosaminidase [Source:HGNC Symbol;Acc:HGNC:318]
ENSG00000070366	0,37760403	4,39888209	2,68464696	0,13482847	0,41730618	SMG6	SMG6 nonsense mediated mRNA decay factor [Source:HGNC Symbol;Acc:HGNC:17809]
ENSG00000125875	0,24254397	6,09780886	2,68561384	0,1347664	0,41730618	TBC1D20	TBC1 domain family member 20 [Source:HGNC Symbol;Acc:HGNC:16133]
ENSG00000139131	0,2578039	6,14740402	2,68638481	0,13471695	0,41730618	YARS2	tyrosyl-tRNA synthetase 2 [Source:HGNC Symbol;Acc:HGNC:24249]
ENSG00000166046	0,56316124	0,63095146	2,68561146	0,13476656	0,41730618	TCP11L2	t-complex 11 like 2 [Source:HGNC Symbol;Acc:HGNC:28627]
ENSG00000166479	-0,317682	4,29247282	2,68532356	0,13478503	0,41730618	TMX3	thioredoxin related transmembrane protein 3 [Source:HGNC Symbol;Acc:HGNC:24718]
ENSG00000212695	-0,6183611	-0,570287	2,68378288	0,13488396	0,41730618	AL583805.1	ribosomal protein 18 (RPL18) pseudogene
ENSG00000272145	-0,5564507	0,09295805	2,68403818	0,13486756	0,41730618	NFYC-AS1	NFYC antisense RNA 1 [Source:HGNC Symbol;Acc:HGNC:49451]
ENSG00000140854	-0,2329961	5,50169945	2,68333014	0,13491305	0,41730694	KATNB1	katanin regulatory subunit B1 [Source:HGNC Symbol;Acc:HGNC:6217]
ENSG00000165487	0,28578273	4,94505572	2,68272556	0,13495191	0,41730694	MICU2	mitochondrial calcium uptake 2 [Source:HGNC Symbol;Acc:HGNC:31830]
ENSG00000233057	-0,633961	-0,3926908	2,68244562	0,1349699	0,41730694	EEF1A1P14	eukaryotic translation elongation factor 1 alpha 1 pseudogene 14 [Source:HGNC Symbol;Acc:HGNC:3197]
ENSG00000180855	-0,302818	3,48038956	2,67614022	0,13537609	0,41843843	ZNF443	zinc finger protein 443 [Source:HGNC Symbol;Acc:HGNC:20878]
ENSG00000235508	-0,3839316	0,96273994	2,67575814	0,13540075	0,41843843	RPS2P7	ribosomal protein S2 pseudogene 7 [Source:HGNC Symbol;Acc:HGNC:15838]
ENSG00000242071	-0,2584463	4,13123052	2,67543231	0,13542179	0,41843843	RPL7AP6	ribosomal protein L7a pseudogene 6 [Source:HGNC Symbol;Acc:HGNC:19785]
ENSG00000197696	0,23973205	6,04889168	2,67404169	0,13551162	0,41862747	NMB	neuromedin B [Source:HGNC Symbol;Acc:HGNC:7842]
ENSG00000101945	0,24285664	4,35903654	2,67303928	0,13557643	0,41873911	SUV39H1	suppressor of variegation 3-9 homolog 1 [Source:HGNC Symbol;Acc:HGNC:11479]
ENSG00000152413	-0,295561	2,372552	2,67201708	0,13564255	0,41885479	HOMER1	homer scaffold protein 1 [Source:HGNC Symbol;Acc:HGNC:17512]
ENSG00000145216	0,26826326	4,74167081	2,66999398	0,13577354	0,41917068	FIP1L1	factor interacting with PAPOLA and CPSF1 [Source:HGNC Symbol;Acc:HGNC:19124]
ENSG00000137094	-0,4820268	1,1354942	2,66747913	0,1359366	0,41957316	DNAJB5	DnaJ heat shock protein family (Hsp40) member B5 [Source:HGNC Symbol;Acc:HGNC:14887]
ENSG00000180210	-0,4020859	9,55720909	2,66709785	0,13596135	0,41957316	F2	coagulation factor II, thrombin [Source:HGNC Symbol;Acc:HGNC:3535]
ENSG00000083520	0,2936035	4,9899278	2,66622298	0,13601815	0,41963712	DIS3	DIS3 homolog, exosome endoribonuclease and 3'-5' exoribonuclease [Source:HGNC Symbol;Acc:HGNC:20604]
ENSG00000100162	-0,260694	5,16680759	2,66376539	0,13617786	0,41963712	CENPM	centromere protein M [Source:HGNC Symbol;Acc:HGNC:18352]
ENSG00000100591	0,23191784	8,32137771	2,66368419	0,13618314	0,41963712	AHSA1	activator of HSP90 ATPase activity 1 [Source:HGNC Symbol;Acc:HGNC:1189]
ENSG00000104856	-0,278044	2,90310502	2,66447159	0,13613194	0,41963712	RELB	RELB proto-oncogene, NF-kB subunit [Source:HGNC Symbol;Acc:HGNC:9956]
ENSG00000127993	0,29605521	2,34129926	2,66505817	0,13609382	0,41963712	RBM48	RNA binding motif protein 48 [Source:HGNC Symbol;Acc:HGNC:21785]
ENSG00000174586	-0,5484486	-0,3421499	2,66580635	0,13604521	0,41963712	ZNF497	zinc finger protein 497 [Source:HGNC Symbol;Acc:HGNC:23714]
ENSG00000197191	-0,3463748	1,27243894	2,66454019	0,13612748	0,41963712	CYSRT1	cysteine rich tail 1 [Source:HGNC Symbol;Acc:HGNC:30529]
ENSG00000168734	0,38658959	3,90047876	2,66246483	0,13626248	0,41965801	PKIG	cAMP-dependent protein kinase inhibitor gamma [Source:HGNC Symbol;Acc:HGNC:9019]
ENSG00000173041	0,34980707	1,33666321	2,66225562	0,1362761	0,41965801	ZNF680	zinc finger protein 680 [Source:HGNC Symbol;Acc:HGNC:26897]
ENSG00000242193	0,44810934	0,36661079	2,66272511	0,13624554	0,41965801	CRYZL2P	crystallin zeta like 2, pseudogene [Source:HGNC Symbol;Acc:HGNC:52164]
ENSG00000198105	0,40892823	2,63786236	2,66136244	0,13633426	0,41974863	ZNF248	zinc finger protein 248 [Source:HGNC Symbol;Acc:HGNC:13041]
ENSG00000083817	0,26319024	3,07425893	2,65935527	0,13646508	0,41992359	ZNF416	zinc finger protein 416 [Source:HGNC Symbol;Acc:HGNC:20645]
ENSG00000171055	-0,2355393	4,64853957	2,65992671	0,13642782	0,41992359	FEZ2	fasciculation and elongation protein zeta 2 [Source:HGNC Symbol;Acc:HGNC:3660]
ENSG00000198208	-0,2788074	2,25029515	2,65872696	0,13650607	0,41992359	RPS6KL1	ribosomal protein S6 kinase like 1 [Source:HGNC Symbol;Acc:HGNC:20222]
ENSG00000281501	-0,6432934	-0,4294838	2,65882356	0,13649977	0,41992359	SEPSECS-AS1	SEPSECS antisense RNA 1 (head to head) [Source:HGNC Symbol;Acc:HGNC:27737]
ENSG00000168010	-0,6356277	-0,3232642	2,6577023	0,13657294	0,42004086	ATG16L2	autophagy related 16 like 2 [Source:HGNC Symbol;Acc:HGNC:25464]
ENSG00000054611	-0,2599019	4,97147679	2,65540091	0,13672329	0,42019711	TBC1D22A	TBC1 domain family member 22A [Source:HGNC Symbol;Acc:HGNC:1309]

ENSG00000132970	0,31387277	1,87730407	2,65644749	0,13665489	0,42019711	WASF3	WASP family member 3 [Source:HGNC Symbol;Acc:HGNC:12734]
ENSG00000144182	-0,3579019	1,64350153	2,65449789	0,13678234	0,42019711	LIPT1	lipoyltransferase 1 [Source:HGNC Symbol;Acc:HGNC:29569]
ENSG00000146223	0,23314552	7,52676462	2,65584975	0,13669395	0,42019711	RPL7L1	ribosomal protein L7 like 1 [Source:HGNC Symbol;Acc:HGNC:21370]
ENSG00000187650	-0,6271916	-0,4066238	2,65428415	0,13679632	0,42019711	VMAC	vimentin type intermediate filament associated coiled-coil protein [Source:HGNC Symbol;Acc:HGNC:33803]
ENSG00000245937	-0,3764603	1,67616319	2,65454639	0,13677917	0,42019711	LINC01184	long intergenic non-protein coding RNA 1184 [Source:HGNC Symbol;Acc:HGNC:49565]
ENSG00000271784	-0,8564079	-1,1987932	2,65361481	0,13684012	0,42024329	AL031055.1	novel transcript, antisense to TP53RK
ENSG00000173113	-0,2114523	8,10355031	2,65207621	0,13694087	0,42037595	TRMT112	tRNA methyltransferase subunit 11-2 [Source:HGNC Symbol;Acc:HGNC:26940]
ENSG00000241015	0,45263255	0,7095155	2,65210987	0,13693866	0,42037595	TPM3P9	tropomyosin 3 pseudogene 9 [Source:HGNC Symbol;Acc:HGNC:44142]
ENSG00000147408	0,41081393	1,16890898	2,65149422	0,136979	0,42040467	CSGALNACT1	chondroitin sulfate N-acetylgalactosaminyltransferase 1 [Source:HGNC Symbol;Acc:HGNC:24290]
ENSG00000128708	0,22739188	6,28640079	2,64979052	0,13709071	0,42065914	HAT1	histone acetyltransferase 1 [Source:HGNC Symbol;Acc:HGNC:4821]
ENSG00000144659	0,2763992	5,6433218	2,64881825	0,13715451	0,42075645	SLC25A38	solute carrier family 25 member 38 [Source:HGNC Symbol;Acc:HGNC:26054]
ENSG00000219747	-0,3558842	1,09926037	2,64842969	0,13718002	0,42075645	AL133260.1	60S ribosomal protein L32 (RPL32) pseudogene
ENSG00000137210	-0,3150641	5,58562777	2,64764121	0,13723181	0,42080091	TMEM14B	transmembrane protein 14B [Source:HGNC Symbol;Acc:HGNC:21384]
ENSG00000140577	0,35633174	2,69930926	2,64733186	0,13725213	0,42080091	CRTC3	CREB regulated transcription coactivator 3 [Source:HGNC Symbol;Acc:HGNC:26148]
ENSG00000124762	-0,2456175	5,7054452	2,64682593	0,13728538	0,42081453	CDKN1A	cyclin dependent kinase inhibitor 1A [Source:HGNC Symbol;Acc:HGNC:1784]
ENSG00000132423	0,23531152	4,18835315	2,64510652	0,13739844	0,42105595	COQ3	coenzyme Q3, methyltransferase [Source:HGNC Symbol;Acc:HGNC:18175]
ENSG00000164659	0,31129194	1,77995028	2,64457697	0,13743329	0,42105595	ELAPOR2	endosome-lysosome associated apoptosis and autophagy regulator family member 2 [Source:HGNC Symbol;Acc:H
ENSG00000171067	-0,2469303	6,35391623	2,64431398	0,1374506	0,42105595	C11orf24	chromosome 11 open reading frame 24 [Source:HGNC Symbol;Acc:HGNC:1174]
ENSG00000165527	-0,2556289	5,11045775	2,64255425	0,1375665	0,42132264	ARF6	ADP ribosylation factor 6 [Source:HGNC Symbol;Acc:HGNC:659]
ENSG00000174564	-0,737062	-1,3089365	2,64104831	0,13766579	0,4214739	IL20RB	interleukin 20 receptor subunit beta [Source:HGNC Symbol;Acc:HGNC:6004]
ENSG00000185684	-0,2749428	2,7164635	2,64033905	0,13771258	0,4214739	EP400P1	EP400 pseudogene 1 [Source:HGNC Symbol;Acc:HGNC:26602]
ENSG00000233990	-0,7910109	-1,6145007	2,64005554	0,13773129	0,4214739	AL353754.1	mitochondrial ribosomal protein L13 (MRPL13) pseudogene
ENSG00000238287	-0,8046697	-0,7887497	2,64067543	0,13769038	0,4214739	AL603839.3	novel transcript
ENSG00000156304	0,29275084	4,68764556	2,63909705	0,13779457	0,42149984	SCAF4	SR-related CTD associated factor 4 [Source:HGNC Symbol;Acc:HGNC:19304]
ENSG00000167656	-0,6389578	-0,4700167	2,6390531	0,13779747	0,42149984	LY6D	lymphocyte antigen 6 family member D [Source:HGNC Symbol;Acc:HGNC:13348]
ENSG00000138594	0,2627166	4,47202085	2,63813612	0,13785805	0,42155865	TMOD3	tropomodulin 3 [Source:HGNC Symbol;Acc:HGNC:11873]
ENSG00000167264	-0,2774209	4,18725021	2,63788855	0,13787441	0,42155865	DUS2	dihydrouridine synthase 2 [Source:HGNC Symbol;Acc:HGNC:26014]
ENSG00000165490	-0,3187138	2,15289735	2,63722614	0,13791819	0,42160429	DDIAS	DNA damage induced apoptosis suppressor [Source:HGNC Symbol;Acc:HGNC:26351]
ENSG00000122406	-0,2180665	10,5598069	2,63617379	0,13798779	0,42172881	RPL5	ribosomal protein L5 [Source:HGNC Symbol;Acc:HGNC:10360]
ENSG00000051523	-0,2214825	8,11909448	2,63481058	0,13807802	0,42176907	CYBA	cytochrome b-245 alpha chain [Source:HGNC Symbol;Acc:HGNC:2577]
ENSG00000179912	-0,5134048	1,71547911	2,63466627	0,13808758	0,42176907	R3HDM2	R3H domain containing 2 [Source:HGNC Symbol;Acc:HGNC:29167]
ENSG00000255717	0,26407455	5,78411922	2,63545786	0,13803517	0,42176907	SNHG1	small nucleolar RNA host gene 1 [Source:HGNC Symbol;Acc:HGNC:32688]
ENSG00000142459	-0,5025557	1,1554637	2,63154133	0,13829472	0,42222667	EVI5L	ecotropic viral integration site 5 like [Source:HGNC Symbol;Acc:HGNC:30464]
ENSG00000234432	-0,6816479	-0,917743	2,63153403	0,1382952	0,42222667	AC092171.3	uncharacterized LOC100129484 [Source:NCBI gene (formerly Entrezgene);Acc:100129484]
ENSG00000134202	0,24125018	6,536918	2,63086543	0,13833957	0,42227389	GSTM3	glutathione S-transferase mu 3 [Source:HGNC Symbol;Acc:HGNC:4635]
ENSG00000096060	-0,2306262	5,27373176	2,62843773	0,13850084	0,42267784	FKBP5	FKBP prolyl isomerase 5 [Source:HGNC Symbol;Acc:HGNC:3721]
ENSG00000167553	-0,2643912	9,55425998	2,62766587	0,13855217	0,42274616	TUBA1C	tubulin alpha 1c [Source:HGNC Symbol;Acc:HGNC:20768]
ENSG00000100029	-0,304745	6,99270377	2,62641699	0,13863526	0,42283077	PES1	pescadillo ribosomal biogenesis factor 1 [Source:HGNC Symbol;Acc:HGNC:8848]
ENSG00000113391	0,35719532	1,68776961	2,62637912	0,13863778	0,42283077	FAM172A	family with sequence similarity 172 member A [Source:HGNC Symbol;Acc:HGNC:25365]
ENSG00000111247	0,24947991	4,31305463	2,62244784	0,13889978	0,42345303	RAD51AP1	RAD51 associated protein 1 [Source:HGNC Symbol;Acc:HGNC:16956]
ENSG00000169019	0,25474085	3,6684108	2,62282501	0,13887462	0,42345303	COMMD8	COMM domain containing 8 [Source:HGNC Symbol;Acc:HGNC:26036]
ENSG00000127445	-0,2549557	5,16905916	2,61360572	0,13949142	0,42516797	PIN1	peptidylprolyl cis/trans isomerase, NIMA-interacting 1 [Source:HGNC Symbol;Acc:HGNC:8988]
ENSG00000006062	-0,2507141	3,6593854	2,61258477	0,13955994	0,4252881	MAP3K14	mitogen-activated protein kinase kinase kinase 14 [Source:HGNC Symbol;Acc:HGNC:6853]
ENSG00000099810	0,22933523	4,9345075	2,61093705	0,13967062	0,4253592	MTAP	methylthioadenosine phosphorylase [Source:HGNC Symbol;Acc:HGNC:7413]

ENSG00000115750	0,24281011	4,81993449	2,61165267	0,13962254	0,4253592	TAF1B	TATA-box binding protein associated factor, RNA polymerase I subunit B [Source:HGNC Symbol;Acc:HGNC:11533]
ENSG00000174827	0,42033775	3,53353612	2,61096558	0,1396687	0,4253592	PDZK1	PDZ domain containing 1 [Source:HGNC Symbol;Acc:HGNC:8821]
ENSG00000134285	-0,2543747	3,42561543	2,61018984	0,13972085	0,42542348	FKBP11	FKBP prolyl isomerase 11 [Source:HGNC Symbol;Acc:HGNC:18624]
ENSG00000164164	0,25470234	3,89843958	2,6095859	0,13976146	0,42542365	OTUD4	OTU deubiquitinase 4 [Source:HGNC Symbol;Acc:HGNC:24949]
ENSG00000189376	0,27247722	2,43734351	2,60918415	0,13978849	0,42542365	C8orf76	chromosome 8 open reading frame 76 [Source:HGNC Symbol;Acc:HGNC:25924]
ENSG00000197586	0,20848535	8,59725139	2,60889025	0,13980827	0,42542365	ENTPD6	ectonucleoside triphosphate diphosphohydrolase 6 [Source:HGNC Symbol;Acc:HGNC:3368]
ENSG00000129566	-0,4401686	3,24377052	2,60728686	0,13991622	0,42557485	TEP1	telomerase associated protein 1 [Source:HGNC Symbol;Acc:HGNC:11726]
ENSG00000169955	-0,2865497	2,40972464	2,60771266	0,13988754	0,42557485	ZNF747	zinc finger protein 747 [Source:HGNC Symbol;Acc:HGNC:28350]
ENSG00000169718	-0,222515	7,70028602	2,60511956	0,1400623	0,42591183	DUS1L	dihydrouridine synthase 1 like [Source:HGNC Symbol;Acc:HGNC:30086]
ENSG00000247596	-0,2304128	5,27992004	2,60477845	0,14008531	0,42591183	TWF2	twinfilin actin binding protein 2 [Source:HGNC Symbol;Acc:HGNC:9621]
ENSG00000235106	0,33096384	1,92486834	2,60299348	0,14020581	0,42618948	BRD3OS	BRD3 opposite strand [Source:HGNC Symbol;Acc:HGNC:24742]
ENSG00000028528	0,23348285	6,935772	2,60075139	0,14035734	0,42641594	SNX1	sorting nexin 1 [Source:HGNC Symbol;Acc:HGNC:11172]
ENSG00000070476	0,24154872	4,2317053	2,60109619	0,14033403	0,42641594	ZXDC	ZXD family zinc finger C [Source:HGNC Symbol;Acc:HGNC:28160]
ENSG00000106367	0,22181868	5,76795762	2,60059573	0,14036787	0,42641594	AP1S1	adaptor related protein complex 1 subunit sigma 1 [Source:HGNC Symbol;Acc:HGNC:559]
ENSG00000143549	0,21086899	8,28559651	2,59941653	0,14044767	0,42642634	TPM3	tropomyosin 3 [Source:HGNC Symbol;Acc:HGNC:12012]
ENSG00000221821	-0,342377	2,74265864	2,59925111	0,14045886	0,42642634	C6orf226	chromosome 6 open reading frame 226 [Source:HGNC Symbol;Acc:HGNC:34431]
ENSG00000254373	0,83855934	-1,3041233	2,59942043	0,1404474	0,42642634	AC112191.2	ubiquinol-cytochrome c reductase binding protein (UQCRB) pseudogene
ENSG00000178026	-0,2876969	3,24537899	2,59745842	0,1405803	0,4265444	LRRC75B	leucine rich repeat containing 75B [Source:HGNC Symbol;Acc:HGNC:33155]
ENSG00000275437	0,41432757	0,83682058	2,59804412	0,14054061	0,4265444	AL121832.3	novel transcript, sense intronic to CABLES2
ENSG00000276234	0,27652866	3,3873407	2,59738395	0,14058534	0,4265444	TADA2A	transcriptional adaptor 2A [Source:HGNC Symbol;Acc:HGNC:11531]
ENSG00000286923	0,85322712	-1,4323663	2,59651021	0,14064458	0,42663552	AC004522.5	novel transcript
ENSG00000128309	-0,2274688	7,18932376	2,59545457	0,14071619	0,42667556	MPST	mercaptopyruvate sulfurtransferase [Source:HGNC Symbol;Acc:HGNC:7223]
ENSG00000215895	-0,4856819	2,11189983	2,59559702	0,14070652	0,42667556	HSPA5P1	HSPA5 pseudogene 1 [Source:HGNC Symbol;Acc:HGNC:54532]
ENSG00000130734	-0,3225026	4,07393651	2,59433167	0,14079242	0,4268181	ATG4D	autophagy related 4D cysteine peptidase [Source:HGNC Symbol;Acc:HGNC:20789]
ENSG00000241923	-0,3678968	1,03615924	2,59385963	0,14082448	0,42682672	RPL14P3	ribosomal protein L14 pseudogene 3 [Source:HGNC Symbol;Acc:HGNC:36638]
ENSG00000052126	0,30155041	3,28452942	2,59195305	0,14095406	0,42700479	PLEKHA5	pleckstrin homology domain containing A5 [Source:HGNC Symbol;Acc:HGNC:30036]
ENSG00000123560	1,03537338	0,47022942	2,59238361	0,14092479	0,42700479	PLP1	proteolipid protein 1 [Source:HGNC Symbol;Acc:HGNC:9086]
ENSG00000171680	-0,2633344	2,54995292	2,59170528	0,14097092	0,42700479	PLEKHG5	pleckstrin homology and RhoGEF domain containing G5 [Source:HGNC Symbol;Acc:HGNC:29105]
ENSG00000124275	0,2833078	4,70881271	2,59054697	0,14104973	0,42706269	MTRR	5-methyltetrahydrofolate-homocysteine methyltransferase reductase [Source:HGNC Symbol;Acc:HGNC:7473]
ENSG00000179627	-0,3902394	2,20079558	2,59075561	0,14103553	0,42706269	ZBTB42	zinc finger and BTB domain containing 42 [Source:HGNC Symbol;Acc:HGNC:32550]
ENSG00000197713	0,22631376	6,27873458	2,59013571	0,14107773	0,42706269	RPE	ribulose-5-phosphate-3-epimerase [Source:HGNC Symbol;Acc:HGNC:10293]
ENSG00000196236	0,40660773	4,09622321	2,58914398	0,14114527	0,42717864	XPNPEP3	X-prolyl aminopeptidase 3 [Source:HGNC Symbol;Acc:HGNC:28052]
ENSG00000166900	-0,2182654	5,26120599	2,58836255	0,14119852	0,42725129	STX3	syntaxin 3 [Source:HGNC Symbol;Acc:HGNC:11438]
ENSG00000170293	0,28885196	4,87915926	2,58590305	0,1413663	0,42767037	CMTM8	CKLF like MARVEL transmembrane domain containing 8 [Source:HGNC Symbol;Acc:HGNC:19179]
ENSG00000118298	0,35165486	1,79782946	2,58407497	0,14149116	0,4279595	CA14	carbonic anhydrase 14 [Source:HGNC Symbol;Acc:HGNC:1372]
ENSG00000165055	0,24337487	4,79848956	2,58285153	0,14157481	0,42812387	METTL2B	methyltransferase like 2B [Source:HGNC Symbol;Acc:HGNC:18272]
ENSG00000126391	0,24453143	5,02874794	2,58063129	0,14172678	0,42840605	FRMD8	FERM domain containing 8 [Source:HGNC Symbol;Acc:HGNC:25462]
ENSG00000137955	-0,2103964	6,94329388	2,58082101	0,14171378	0,42840605	RABGGTB	Rab geranylgeranyltransferase subunit beta [Source:HGNC Symbol;Acc:HGNC:9796]
ENSG00000196388	-0,604122	-0,7640646	2,5801997	0,14175634	0,42840678	INCA1	inhibitor of CDK, cyclin A1 interacting protein 1 [Source:HGNC Symbol;Acc:HGNC:32224]
ENSG00000174501	-0,5753023	-0,5103698	2,57838231	0,14188092	0,4286946	ANKRD36C	ankyrin repeat domain 36C [Source:HGNC Symbol;Acc:HGNC:32946]
ENSG00000109686	-0,2532477	5,22151934	2,57654005	0,14200735	0,42872193	SH3D19	SH3 domain containing 19 [Source:HGNC Symbol;Acc:HGNC:30418]
ENSG00000110057	-0,280497	3,59029715	2,57734628	0,14195201	0,42872193	UNC93B1	unc-93 homolog B1, TLR signaling regulator [Source:HGNC Symbol;Acc:HGNC:13481]
ENSG00000121741	-0,2458752	3,41062672	2,57674381	0,14199336	0,42872193	ZMYM2	zinc finger MYM-type containing 2 [Source:HGNC Symbol;Acc:HGNC:12989]
ENSG00000145386	0,2315472	6,57472511	2,57673153	0,14199421	0,42872193	CCNA2	cyclin A2 [Source:HGNC Symbol;Acc:HGNC:1578]

ENSG00000101856	0,40719418	7,18287118	2,57360452	0,14220912	0,42924236	PGRMC1	progesterone receptor membrane component 1 [Source:HGNC Symbol;Acc:HGNC:16090]
ENSG00000090097	-0,2267829	5,77496269	2,57292878	0,14225562	0,42929401	PCBP4	poly(rC) binding protein 4 [Source:HGNC Symbol;Acc:HGNC:8652]
ENSG00000177311	0,26320469	3,78302173	2,5708213	0,14240076	0,42964326	ZBTB38	zinc finger and BTB domain containing 38 [Source:HGNC Symbol;Acc:HGNC:26636]
ENSG00000152192	0,51912288	-0,1951032	2,56974473	0,14247498	0,42972786	POU4F1	POU class 4 homeobox 1 [Source:HGNC Symbol;Acc:HGNC:9218]
ENSG00000250347	0,79577114	-1,5367201	2,56956125	0,14248763	0,42972786	AC005740.2	mitochondrial ribosomal protein L11 (MRPL11) pseudogene
ENSG00000231313	-0,4892156	0,87071025	2,56878334	0,14254129	0,42980098	CLIC1P1	chloride intracellular channel 1 pseudogene 1 [Source:HGNC Symbol;Acc:HGNC:32691]
ENSG00000197056	0,39646188	1,26650618	2,5682343	0,14257919	0,42982652	ZMYM1	zinc finger MYM-type containing 1 [Source:HGNC Symbol;Acc:HGNC:26253]
ENSG00000099800	-0,2409741	7,30400102	2,5665675	0,1426943	0,43008481	TIMM13	translocase of inner mitochondrial membrane 13 [Source:HGNC Symbol;Acc:HGNC:11816]
ENSG00000132846	0,44613528	1,74208562	2,56399937	0,1428719	0,43038038	ZBED3	zinc finger BED-type containing 3 [Source:HGNC Symbol;Acc:HGNC:20711]
ENSG00000152642	0,2515384	3,40298951	2,56387155	0,14288075	0,43038038	GPD1L	glycerol-3-phosphate dehydrogenase 1 like [Source:HGNC Symbol;Acc:HGNC:28956]
ENSG00000196642	-0,2624317	6,7061357	2,56464723	0,14282707	0,43038038	RABL6	RAB, member RAS oncogene family like 6 [Source:HGNC Symbol;Acc:HGNC:24703]
ENSG00000279170	-0,4614083	0,23277647	2,56192439	0,14301561	0,4306978	TSTD3	thiosulfate sulfurtransferase like domain containing 3 [Source:HGNC Symbol;Acc:HGNC:40910]
ENSG00000125885	-0,2441421	3,36961735	2,56124462	0,14306273	0,43075091	MCM8	minichromosome maintenance 8 homologous recombination repair factor [Source:HGNC Symbol;Acc:HGNC:1614
ENSG00000140740	-0,2607994	9,11276964	2,56033468	0,14312583	0,43085211	UQCRC2	ubiquinol-cytochrome c reductase core protein 2 [Source:HGNC Symbol;Acc:HGNC:12586]
ENSG00000136059	-0,6584983	-0,2139449	2,55947769	0,1431853	0,43094233	VILL	villin like [Source:HGNC Symbol;Acc:HGNC:30906]
ENSG00000070961	0,23185462	5,41169576	2,55792518	0,14329311	0,43108918	ATP2B1	ATPase plasma membrane Ca2+ transporting 1 [Source:HGNC Symbol;Acc:HGNC:814]
ENSG00000160062	-0,684798	-0,4768861	2,5580221	0,14328638	0,43108918	ZBTB8A	zinc finger and BTB domain containing 8A [Source:HGNC Symbol;Acc:HGNC:24172]
ENSG00000174109	-0,2594365	4,35036259	2,5574707	0,14332469	0,43109541	C16orf91	chromosome 16 open reading frame 91 [Source:HGNC Symbol;Acc:HGNC:27558]
ENSG00000124596	0,27506458	4,84856039	2,55690948	0,1433637	0,43112398	OARD1	O-acyl-ADP-ribose deacylase 1 [Source:HGNC Symbol;Acc:HGNC:21257]
ENSG00000168476	-0,2773843	6,16040497	2,55496655	0,14349886	0,43144161	REEP4	receptor accessory protein 4 [Source:HGNC Symbol;Acc:HGNC:26176]
ENSG00000136279	-0,2695	5,88443395	2,55249923	0,14367073	0,4315965	DBNL	drebrin like [Source:HGNC Symbol;Acc:HGNC:2696]
ENSG00000157741	0,66902309	1,52730724	2,55234735	0,14368132	0,4315965	UBN2	ubinuclein 2 [Source:HGNC Symbol;Acc:HGNC:21931]
ENSG00000165916	-0,2571028	7,99679103	2,55313408	0,14362648	0,4315965	PSMC3	proteasome 26S subunit, ATPase 3 [Source:HGNC Symbol;Acc:HGNC:9549]
ENSG00000167315	0,2212416	6,76875847	2,55210686	0,14369809	0,4315965	ACAA2	acetyl-CoA acyltransferase 2 [Source:HGNC Symbol;Acc:HGNC:83]
ENSG00000241127	-0,2892332	3,3050011	2,55222703	0,14368971	0,4315965	YAE1	YAE1 maturation factor of ABCE1 [Source:HGNC Symbol;Acc:HGNC:24857]
ENSG00000147996	0,27305707	3,87043833	2,55052795	0,14380824	0,43161562	CBWD5	COBW domain containing 5 [Source:HGNC Symbol;Acc:HGNC:24584]
ENSG00000171759	-0,215613	7,53062398	2,55137689	0,143749	0,43161562	PAH	phenylalanine hydroxylase [Source:HGNC Symbol;Acc:HGNC:8582]
ENSG00000176022	-0,228297	5,05443814	2,55043421	0,14381479	0,43161562	B3GALT6	beta-1,3-galactosyltransferase 6 [Source:HGNC Symbol;Acc:HGNC:17978]
ENSG00000213625	0,21900134	5,5170128	2,55032184	0,14382263	0,43161562	LEPROT	leptin receptor overlapping transcript [Source:HGNC Symbol;Acc:HGNC:29477]
ENSG00000235582	-0,5269274	-0,2948601	2,54913126	0,14390578	0,43177644	AL365258.2	ribosomal protein L24 (RPL24) pseudogene
ENSG00000156502	0,23402391	5,70454702	2,54848912	0,14395065	0,43182238	SUPV3L1	Suv3 like RNA helicase [Source:HGNC Symbol;Acc:HGNC:11471]
ENSG00000235631	1,2238283	-1,7491985	2,54691172	0,14406095	0,43206454	RNF148	ring finger protein 148 [Source:HGNC Symbol;Acc:HGNC:22411]
ENSG00000100599	0,29685168	3,54684528	2,54471614	0,14421466	0,43226378	RIN3	Ras and Rab interactor 3 [Source:HGNC Symbol;Acc:HGNC:18751]
ENSG00000139323	0,32687444	2,93559203	2,54476049	0,14421155	0,43226378	POC1B	POC1 centriolar protein B [Source:HGNC Symbol;Acc:HGNC:30836]
ENSG00000198453	1,02291562	-1,8002633	2,54427263	0,14424573	0,43226378	ZNF568	zinc finger protein 568 [Source:HGNC Symbol;Acc:HGNC:25392]
ENSG00000204498	-0,2334106	4,19203465	2,54458244	0,14422402	0,43226378	NFKBIL1	NFKB inhibitor like 1 [Source:HGNC Symbol;Acc:HGNC:7800]
ENSG00000073670	-0,9502305	-1,1077391	2,54331329	0,14431298	0,43228795	ADAM11	ADAM metallopeptidase domain 11 [Source:HGNC Symbol;Acc:HGNC:189]
ENSG00000185386	0,87214131	-0,9348947	2,54372684	0,14428399	0,43228795	MAPK11	mitogen-activated protein kinase 11 [Source:HGNC Symbol;Acc:HGNC:6873]
ENSG00000084734	-0,3333838	2,26125485	2,54084002	0,14448654	0,43263043	GCKR	glucokinase regulator [Source:HGNC Symbol;Acc:HGNC:4196]
ENSG00000229638	-0,2457428	3,89567281	2,54092394	0,14448065	0,43263043	RPL4P4	ribosomal protein L4 pseudogene 4 [Source:HGNC Symbol;Acc:HGNC:36179]
ENSG00000138073	-0,2718777	7,63232193	2,53855432	0,14464718	0,43302267	PREB	prolactin regulatory element binding [Source:HGNC Symbol;Acc:HGNC:9356]
ENSG00000198796	-0,8488243	-0,9268237	2,53729058	0,14473609	0,43320008	ALPK2	alpha kinase 2 [Source:HGNC Symbol;Acc:HGNC:20565]
ENSG00000111647	0,25258196	3,72942548	2,53516978	0,14488547	0,43337658	UHRF1BP1L	UHRF1 binding protein 1 like [Source:HGNC Symbol;Acc:HGNC:29102]
ENSG00000124920	0,35409325	5,65830727	2,53476892	0,14491373	0,43337658	MYRF	myelin regulatory factor [Source:HGNC Symbol;Acc:HGNC:1181]

ENSG00000167962	-0,2826041	5,33026323	2,5351782	0,14488488	0,43337658	ZNF598	zinc finger protein 598, E3 ubiquitin ligase [Source:HGNC Symbol;Acc:HGNC:28079]
ENSG00000258289	0,32141024	1,77020478	2,53601193	0,14482613	0,43337658	CHURC1	churchill domain containing 1 [Source:HGNC Symbol;Acc:HGNC:20099]
ENSG00000143776	-0,2982906	2,81927841	2,53333867	0,1450146	0,43352826	CDC42BPA	CDC42 binding protein kinase alpha [Source:HGNC Symbol;Acc:HGNC:1737]
ENSG00000186496	0,6257444	-0,8004639	2,53320843	0,14502379	0,43352826	ZNF396	zinc finger protein 396 [Source:HGNC Symbol;Acc:HGNC:18824]
ENSG00000082516	0,3351032	5,66491253	2,5324053	0,14508048	0,433609	GEMIN5	gem nuclear organelle associated protein 5 [Source:HGNC Symbol;Acc:HGNC:20043]
ENSG00000099992	0,28287518	2,00616351	2,5301604	0,14523909	0,43384368	TBC1D10A	TBC1 domain family member 10A [Source:HGNC Symbol;Acc:HGNC:23609]
ENSG00000108510	0,2652939	3,82871027	2,52961315	0,14527779	0,43384368	MED13	mediator complex subunit 13 [Source:HGNC Symbol;Acc:HGNC:22474]
ENSG00000109861	0,31315938	7,75659138	2,5297755	0,14526631	0,43384368	CTSC	cathepsin C [Source:HGNC Symbol;Acc:HGNC:2528]
ENSG00000114742	0,26319361	4,55140894	2,53001188	0,1452496	0,43384368	WDR48	WD repeat domain 48 [Source:HGNC Symbol;Acc:HGNC:30914]
ENSG00000071051	0,23490609	4,51765668	2,52604456	0,14553048	0,43450947	NCK2	NCK adaptor protein 2 [Source:HGNC Symbol;Acc:HGNC:7665]
ENSG00000219451	-0,2557151	2,86440899	2,52556873	0,14556422	0,43452139	RPL23P8	ribosomal protein L23 pseudogene 8 [Source:HGNC Symbol;Acc:HGNC:36225]
ENSG00000253683	-0,467989	0,79933821	2,52454073	0,14563714	0,43465025	AC027309.2	coiled-coil domain containing 72 (CCDC72) pseudogene
ENSG00000104872	-0,3627076	6,01026904	2,52344932	0,14571461	0,4347126	PIH1D1	PIH1 domain containing 1 [Source:HGNC Symbol;Acc:HGNC:26075]
ENSG00000164362	-0,3343992	2,46953401	2,52340798	0,14571754	0,4347126	TERT	telomerase reverse transcriptase [Source:HGNC Symbol;Acc:HGNC:11730]
ENSG00000134955	0,382968	1,49935914	2,52286943	0,14575579	0,43473792	SLC37A2	solute carrier family 37 member 2 [Source:HGNC Symbol;Acc:HGNC:20644]
ENSG00000140350	-0,2086482	7,89293514	2,51926735	0,14601195	0,43541305	ANP32A	acidic nuclear phosphoprotein 32 family member A [Source:HGNC Symbol;Acc:HGNC:13233]
ENSG00000175906	-0,2810603	4,56418948	2,51756136	0,14613347	0,43568649	ARL4D	ADP ribosylation factor like GTPase 4D [Source:HGNC Symbol;Acc:HGNC:656]
ENSG00000065485	-0,2472184	7,28572766	2,51224856	0,14651276	0,43596008	PDIA5	protein disulfide isomerase family A member 5 [Source:HGNC Symbol;Acc:HGNC:24811]
ENSG00000070759	0,29336397	2,45704139	2,5158432	0,14625599	0,43596008	TESK2	testis associated actin remodelling kinase 2 [Source:HGNC Symbol;Acc:HGNC:11732]
ENSG00000112759	0,26419238	6,22301816	2,50959465	0,1467027	0,43596008	SLC29A1	solute carrier family 29 member 1 (Augustine blood group) [Source:HGNC Symbol;Acc:HGNC:11003]
ENSG00000123374	0,21193537	5,41370092	2,51536077	0,14629041	0,43596008	CDK2	cyclin dependent kinase 2 [Source:HGNC Symbol;Acc:HGNC:1771]
ENSG00000154380	-0,2745147	4,40297781	2,51377358	0,14640375	0,43596008	ENAH	ENAH actin regulator [Source:HGNC Symbol;Acc:HGNC:18271]
ENSG00000162378	0,27740514	2,39462154	2,51168808	0,14655284	0,43596008	ZYG11B	zyg-11 family member B, cell cycle regulator [Source:HGNC Symbol;Acc:HGNC:25820]
ENSG00000166997	-0,2598404	4,59281564	2,51140638	0,146573	0,43596008	CNPY4	canopy FGF signaling regulator 4 [Source:HGNC Symbol;Acc:HGNC:28631]
ENSG00000171766	-0,9813779	-1,6797627	2,51267388	0,14648234	0,43596008	GATM	glycine amidinotransferase [Source:HGNC Symbol;Acc:HGNC:4175]
ENSG00000183579	0,24354106	6,67079515	2,51325238	0,14644099	0,43596008	ZNRF3	zinc and ring finger 3 [Source:HGNC Symbol;Acc:HGNC:18126]
ENSG00000184454	0,4185704	0,40702601	2,50982754	0,14668602	0,43596008	NCMAP	non-compact myelin associated protein [Source:HGNC Symbol;Acc:HGNC:29332]
ENSG00000186666	-0,3136519	2,3297005	2,51452224	0,14635028	0,43596008	BCDIN3D	BCDIN3 domain containing RNA methyltransferase [Source:HGNC Symbol;Acc:HGNC:27050]
ENSG00000234036	0,57674974	-0,2370512	2,51474847	0,14633412	0,43596008	TXNP6	thioredoxin pseudogene 6 [Source:HGNC Symbol;Acc:HGNC:49486]
ENSG00000241935	0,29813945	2,5111011	2,51197885	0,14653204	0,43596008	HOGA1	4-hydroxy-2-oxoglutarate aldolase 1 [Source:HGNC Symbol;Acc:HGNC:25155]
ENSG00000247572	0,49098131	0,52746277	2,51020611	0,14665891	0,43596008	CKMT2-AS1	CKMT2 antisense RNA 1 [Source:HGNC Symbol;Acc:HGNC:48997]
ENSG00000259420	-0,721775	-1,1798128	2,51083222	0,14661409	0,43596008	AC046168.2	novel transcript
ENSG00000267216	0,98730652	-1,4274593	2,51049728	0,14663806	0,43596008	ZNF8-ERVK3-1	ZNF8-ERVK3-1 readthrough [Source:NCBI gene (formerly Entrezgene);Acc:108903150]
ENSG00000116014	-0,2780125	3,22891786	2,50847586	0,14678287	0,43602093	KISS1R	KISS1 receptor [Source:HGNC Symbol;Acc:HGNC:4510]
ENSG00000225528	-0,7542879	-1,7552516	2,5087615	0,1467624	0,43602093	Z82206.1	novel protein similar to translation machinery associated 7 homolog (S. cerevisiae) TMA7
ENSG00000112818	-0,6047619	1,49229333	2,50610826	0,14695272	0,43643672	MEP1A	meprin A subunit alpha [Source:HGNC Symbol;Acc:HGNC:7015]
ENSG00000151151	-0,4533706	0,68816126	2,50429289	0,14708312	0,43673522	IPMK	inositol polyphosphate multikinase [Source:HGNC Symbol;Acc:HGNC:20739]
ENSG00000153832	0,43709541	1,51384468	2,50127321	0,14730036	0,43720256	FBXO36	F-box protein 36 [Source:HGNC Symbol;Acc:HGNC:27020]
ENSG00000204438	-0,2753679	4,81782193	2,50144434	0,14728804	0,43720256	GPANK1	G-patch domain and ankyrin repeats 1 [Source:HGNC Symbol;Acc:HGNC:13920]
ENSG00000111325	-0,4438864	0,49423081	2,49889721	0,14747159	0,43736718	OGFOD2	2-oxoglutarate and iron dependent oxygenase domain containing 2 [Source:HGNC Symbol;Acc:HGNC:25823]
ENSG00000113161	0,22316814	8,32338085	2,49884196	0,14747558	0,43736718	HMGCR	3-hydroxy-3-methylglutaryl-CoA reductase [Source:HGNC Symbol;Acc:HGNC:5006]
ENSG00000138709	-0,2272992	5,2432235	2,49965285	0,14741711	0,43736718	LARP1B	La ribonucleoprotein 1B [Source:HGNC Symbol;Acc:HGNC:24704]
ENSG00000158828	0,30750922	4,25901868	2,49894328	0,14746827	0,43736718	PINK1	PTEN induced kinase 1 [Source:HGNC Symbol;Acc:HGNC:14581]
ENSG00000255224	-0,6707659	-0,359618	2,4974042	0,14757932	0,43758602	AC109322.1	TEC

ENSG00000100417	0,2368412	5,67575668	2,49661078	0,14763661	0,43766706	PMM1	phosphomannomutase 1 [Source:HGNC Symbol;Acc:HGNC:9114]
ENSG00000112249	0,30163023	4,83511052	2,49618964	0,14766703	0,43766843	ASCC3	activating signal cointegrator 1 complex subunit 3 [Source:HGNC Symbol;Acc:HGNC:18697]
ENSG00000167862	-0,2113274	6,17556055	2,49457397	0,14778382	0,43792572	MRPL58	mitochondrial ribosomal protein L58 [Source:HGNC Symbol;Acc:HGNC:5359]
ENSG00000173334	-0,2557876	5,8284828	2,4902807	0,14809473	0,43875805	TRIB1	tribbles pseudokinase 1 [Source:HGNC Symbol;Acc:HGNC:16891]
ENSG00000151422	0,28702704	3,72972304	2,4879802	0,14826168	0,43916361	FER	FER tyrosine kinase [Source:HGNC Symbol;Acc:HGNC:3655]
ENSG00000072163	-0,737023	-0,5329881	2,48597792	0,14840719	0,4395055	LIMS2	LIM zinc finger domain containing 2 [Source:HGNC Symbol;Acc:HGNC:16084]
ENSG00000170322	0,26567197	4,549685	2,48543908	0,14844638	0,43953246	NFRKB	nuclear factor related to kappaB binding protein [Source:HGNC Symbol;Acc:HGNC:7802]
ENSG00000164741	0,23463349	5,98009867	2,48477951	0,14849437	0,43958546	DLC1	DLC1 Rho GTPase activating protein [Source:HGNC Symbol;Acc:HGNC:2897]
ENSG00000066056	-0,7117595	1,03030213	2,48407533	0,14854562	0,4396036	TIE1	tyrosine kinase with immunoglobulin like and EGF like domains 1 [Source:HGNC Symbol;Acc:HGNC:11809]
ENSG00000157933	0,37813296	4,03598851	2,48386856	0,14856068	0,4396036	SKI	SKI proto-oncogene [Source:HGNC Symbol;Acc:HGNC:10896]
ENSG00000118058	0,72507459	3,71406337	2,48335273	0,14859825	0,43962571	KMT2A	lysine methyltransferase 2A [Source:HGNC Symbol;Acc:HGNC:7132]
ENSG00000111880	0,29817508	4,64308319	2,48129832	0,14874799	0,43989055	RNGTT	RNA guanylyltransferase and 5'-phosphatase [Source:HGNC Symbol;Acc:HGNC:10073]
ENSG00000234031	0,60328291	-0,337561	2,48164537	0,14872268	0,43989055	RPS3AP44	RPS3A pseudogene 44 [Source:HGNC Symbol;Acc:HGNC:36761]
ENSG00000111725	0,22728229	5,5264276	2,48047368	0,14880815	0,43995197	PRKAB1	protein kinase AMP-activated non-catalytic subunit beta 1 [Source:HGNC Symbol;Acc:HGNC:9378]
ENSG00000257800	0,70417728	-0,9616049	2,48018816	0,14882898	0,43995197	FNBP1P1	formin binding protein 1 pseudogene 1 [Source:HGNC Symbol;Acc:HGNC:44528]
ENSG00000059588	0,2336757	4,22217588	2,47518949	0,1491944	0,44038577	TARBP1	TAR (HIV-1) RNA binding protein 1 [Source:HGNC Symbol;Acc:HGNC:11568]
ENSG00000089022	0,23075546	5,55554112	2,47549427	0,14917209	0,44038577	MAPKAPK5	MAPK activated protein kinase 5 [Source:HGNC Symbol;Acc:HGNC:6889]
ENSG00000104808	1,0521101	-1,2402877	2,47428461	0,14926067	0,44038577	DHDH	dihydrodiol dehydrogenase [Source:HGNC Symbol;Acc:HGNC:17887]
ENSG00000136938	-0,2767772	7,78259794	2,47605607	0,14913097	0,44038577	ANP32B	acidic nuclear phosphoprotein 32 family member B [Source:HGNC Symbol;Acc:HGNC:16677]
ENSG00000165474	1,10184599	-1,3356148	2,47425565	0,1492628	0,44038577	GJB2	gap junction protein beta 2 [Source:HGNC Symbol;Acc:HGNC:4284]
ENSG00000168575	-0,2143208	6,19129568	2,47405932	0,14927718	0,44038577	SLC20A2	solute carrier family 20 member 2 [Source:HGNC Symbol;Acc:HGNC:10947]
ENSG00000170949	0,30241791	2,18636665	2,4768939	0,14906967	0,44038577	ZNF160	zinc finger protein 160 [Source:HGNC Symbol;Acc:HGNC:12948]
ENSG00000174749	-0,2782086	2,23708571	2,47421499	0,14926577	0,44038577	FAM241A	family with sequence similarity 241 member A [Source:HGNC Symbol;Acc:HGNC:26813]
ENSG00000196632	0,81721616	-0,8378941	2,47515358	0,14919703	0,44038577	WNK3	WNK lysine deficient protein kinase 3 [Source:HGNC Symbol;Acc:HGNC:14543]
ENSG00000261499	0,91057395	-1,0463791	2,47672624	0,14908193	0,44038577	AC233699.1	aminopeptidase puromycin sensitive (NPEPPS) pseudogene
ENSG00000136104	0,29689217	4,46962074	2,47322978	0,14933798	0,44047618	RNASEH2B	ribonuclease H2 subunit B [Source:HGNC Symbol;Acc:HGNC:25671]
ENSG00000127080	0,26870393	4,12536279	2,47224522	0,14941018	0,44049792	IPPK	inositol-pentakisphosphate 2-kinase [Source:HGNC Symbol;Acc:HGNC:14645]
ENSG00000258604	-0,7750254	-1,1906268	2,47189597	0,1494358	0,44049792	AL161668.4	novel transcript
ENSG00000278845	0,20353741	7,78200572	2,47192577	0,14943362	0,44049792	MRPL45	mitochondrial ribosomal protein L45 [Source:HGNC Symbol;Acc:HGNC:16651]
ENSG00000126749	-0,2610234	6,99885087	2,47109878	0,14949431	0,44058148	EMG1	EMG1 N1-specific pseudouridine methyltransferase [Source:HGNC Symbol;Acc:HGNC:16912]
ENSG00000043143	0,24131718	4,37214705	2,46986619	0,14958483	0,44071491	JADE2	jade family PHD finger 2 [Source:HGNC Symbol;Acc:HGNC:22984]
ENSG00000153207	0,25621167	5,67969729	2,46966085	0,14959992	0,44071491	AHCTF1	AT-hook containing transcription factor 1 [Source:HGNC Symbol;Acc:HGNC:24618]
ENSG00000012048	0,23339009	4,3967312	2,46481625	0,14995645	0,44137546	BRCA1	BRCA1 DNA repair associated [Source:HGNC Symbol;Acc:HGNC:1100]
ENSG00000111231	-0,2369348	4,60079284	2,46319462	0,15007604	0,44137546	GPN3	GPN-loop GTPase 3 [Source:HGNC Symbol;Acc:HGNC:30186]
ENSG00000130706	-0,2634753	8,07432989	2,46615798	0,1498576	0,44137546	ADRM1	adhesion regulating molecule 1 [Source:HGNC Symbol;Acc:HGNC:15759]
ENSG00000146112	-0,3057985	3,50483214	2,46372054	0,15003724	0,44137546	PPP1R18	protein phosphatase 1 regulatory subunit 18 [Source:HGNC Symbol;Acc:HGNC:29413]
ENSG00000159217	0,43691424	8,08633331	2,46470429	0,1499647	0,44137546	IGF2BP1	insulin like growth factor 2 mRNA binding protein 1 [Source:HGNC Symbol;Acc:HGNC:28866]
ENSG00000164742	0,46618028	0,57705424	2,46556569	0,14990122	0,44137546	ADCY1	adenylate cyclase 1 [Source:HGNC Symbol;Acc:HGNC:232]
ENSG00000166012	-0,2291104	5,92068641	2,46213911	0,15015394	0,44137546	TAF1D	TATA-box binding protein associated factor, RNA polymerase I subunit D [Source:HGNC Symbol;Acc:HGNC:28759]
ENSG00000179832	0,27188482	2,39235109	2,46172955	0,15018419	0,44137546	MROH1	maestro heat like repeat family member 1 [Source:HGNC Symbol;Acc:HGNC:26958]
ENSG00000183298	-0,2762154	2,71379479	2,46230856	0,15014143	0,44137546	RPSAP19	ribosomal protein SA pseudogene 19 [Source:HGNC Symbol;Acc:HGNC:36508]
ENSG00000230076	-0,5118482	-0,0770446	2,46377677	0,15003309	0,44137546	RPL10P6	ribosomal protein L10 pseudogene 6 [Source:HGNC Symbol;Acc:HGNC:52343]
ENSG00000237595	-0,979647	-1,8125189	2,46208354	0,15015805	0,44137546	LINC01275	long intergenic non-protein coding RNA 1275 [Source:HGNC Symbol;Acc:HGNC:50331]
ENSG00000258828	-0,7265646	-0,2977628	2,46169562	0,15018669	0,44137546	KRT8P2	keratin 8 pseudogene 2 [Source:HGNC Symbol;Acc:HGNC:20282]

ENSG00000187164	-0,25858	4,29706569	2,46056648	0,15027012	0,44144302	SHTN1	shootin 1 [Source:HGNC Symbol;Acc:HGNC:29319]
ENSG00000241746	-0,5807812	-0,6974626	2,46073255	0,15025784	0,44144302	AC104212.3	ribosomal protein L13a (RPL13A) pseudogene
ENSG00000110011	-0,3321973	4,01197763	2,45985233	0,15032291	0,44148441	DNAJC4	DnaJ heat shock protein family (Hsp40) member C4 [Source:HGNC Symbol;Acc:HGNC:5271]
ENSG00000125817	-0,2301233	6,2608327	2,45839704	0,15043057	0,44148441	CENPB	centromere protein B [Source:HGNC Symbol;Acc:HGNC:1852]
ENSG00000180806	0,69883066	-0,839094	2,458333	0,15043531	0,44148441	HOXC9	homeobox C9 [Source:HGNC Symbol;Acc:HGNC:5130]
ENSG00000204920	0,4088836	0,91799243	2,45934989	0,15036007	0,44148441	ZNF155	zinc finger protein 155 [Source:HGNC Symbol;Acc:HGNC:12940]
ENSG00000261236	-0,3753776	6,7459891	2,45858424	0,15041671	0,44148441	BOP1	BOP1 ribosomal biogenesis factor [Source:HGNC Symbol;Acc:HGNC:15519]
ENSG00000168913	0,95865232	-0,7102249	2,45673458	0,15055367	0,44174304	ENHO	energy homeostasis associated [Source:HGNC Symbol;Acc:HGNC:24838]
ENSG00000067177	0,29996262	2,57184055	2,45434177	0,15073109	0,44190859	PHKA1	phosphorylase kinase regulatory subunit alpha 1 [Source:HGNC Symbol;Acc:HGNC:8925]
ENSG00000113569	0,32728088	5,60412688	2,45438327	0,15072801	0,44190859	NUP155	nucleoporin 155 [Source:HGNC Symbol;Acc:HGNC:8063]
ENSG00000118260	-0,2863138	4,39673636	2,45473741	0,15070174	0,44190859	CREB1	cAMP responsive element binding protein 1 [Source:HGNC Symbol;Acc:HGNC:2345]
ENSG00000272086	0,41112552	1,27988334	2,45469027	0,15070524	0,44190859	AC025181.2	novel transcript
ENSG00000046647	-0,2520283	3,1304353	2,45266839	0,15085533	0,44218409	GEMIN8	gem nuclear organelle associated protein 8 [Source:HGNC Symbol;Acc:HGNC:26044]
ENSG00000140320	0,30378366	4,59968854	2,45201713	0,15090372	0,44222488	BAHD1	bromo adjacent homology domain containing 1 [Source:HGNC Symbol;Acc:HGNC:29153]
ENSG00000163220	-0,8272721	-1,0503286	2,45166636	0,15092979	0,44222488	S100A9	S100 calcium binding protein A9 [Source:HGNC Symbol;Acc:HGNC:10499]
ENSG00000214135	0,38096531	2,28812187	2,45082248	0,15099253	0,44232001	AC132008.2	succinate dehydrogenase complex, subunit A, flavoprotein (Fp) (SDHA) pseudogene
ENSG00000171928	0,28947937	4,47854842	2,44992356	0,1510594	0,44242719	TVP23B	trans-golgi network vesicle protein 23 homolog B [Source:HGNC Symbol;Acc:HGNC:20399]
ENSG00000135763	0,26394251	4,68296738	2,44768753	0,15122592	0,4428261	URB2	URB2 ribosome biogenesis homolog [Source:HGNC Symbol;Acc:HGNC:28967]
ENSG00000198435	0,81357885	-1,7250866	2,44544097	0,15139345	0,44322785	NRARP	NOTCH regulated ankyrin repeat protein [Source:HGNC Symbol;Acc:HGNC:33843]
ENSG00000063241	-0,2546772	6,50733057	2,44497815	0,151428	0,44324016	ISOC2	isochorismatase domain containing 2 [Source:HGNC Symbol;Acc:HGNC:26278]
ENSG00000256546	-0,5912694	-0,2526179	2,44438543	0,15147226	0,44328089	AC156455.1	novel transcript
ENSG00000163463	0,53894315	-0,4507792	2,44308128	0,15156969	0,44347719	KRTCAP2	keratinocyte associated protein 2 [Source:HGNC Symbol;Acc:HGNC:28942]
ENSG00000255468	-0,4984141	1,13384377	2,44136249	0,15169823	0,4437644	AP001107.9	novel transcript, antisense to SLC29A2
ENSG00000068697	0,27160488	8,78792053	2,43905175	0,15187126	0,44383623	LAPTM4A	lysosomal protein transmembrane 4 alpha [Source:HGNC Symbol;Acc:HGNC:6924]
ENSG00000138162	-0,2639962	5,26354874	2,43808077	0,15194404	0,44383623	TACC2	transforming acidic coiled-coil containing protein 2 [Source:HGNC Symbol;Acc:HGNC:11523]
ENSG00000155008	0,29084421	3,25036986	2,43889869	0,15188273	0,44383623	APOOL	apolipoprotein O like [Source:HGNC Symbol;Acc:HGNC:24009]
ENSG00000169020	-0,265224	7,19725197	2,4385709	0,1519073	0,44383623	ATP5ME	ATP synthase membrane subunit e [Source:HGNC Symbol;Acc:HGNC:846]
ENSG00000178927	-0,2095689	5,97780918	2,43938136	0,15184656	0,44383623	CYBC1	cytochrome b-245 chaperone 1 [Source:HGNC Symbol;Acc:HGNC:28672]
ENSG00000181192	0,29757567	3,17806423	2,4377902	0,15196583	0,44383623	DHTKD1	dehydrogenase E1 and transketolase domain containing 1 [Source:HGNC Symbol;Acc:HGNC:23537]
ENSG00000235374	-0,5217739	0,61050702	2,43955096	0,15183385	0,44383623	SSR4P1	signal sequence receptor subunit 4 pseudogene 1 [Source:HGNC Symbol;Acc:HGNC:23131]
ENSG00000250959	0,68671088	-1,0596481	2,43942352	0,1518434	0,44383623	GLUD1P3	glutamate dehydrogenase 1 pseudogene 3 [Source:HGNC Symbol;Acc:HGNC:4338]
ENSG00000204104	0,2627416	3,79448664	2,43607835	0,15209429	0,44412261	TRAF3IP1	TRAF3 interacting protein 1 [Source:HGNC Symbol;Acc:HGNC:17861]
ENSG00000198730	0,4337751	5,0301288	2,4352305	0,15215796	0,44421975	CTR9	CTR9 homolog, Paf1/RNA polymerase II complex component [Source:HGNC Symbol;Acc:HGNC:16850]
ENSG00000157881	-0,3443706	4,22230657	2,43475671	0,15219356	0,4442349	PANK4	pantothenate kinase 4 (inactive) [Source:HGNC Symbol;Acc:HGNC:19366]
ENSG00000112306	-0,2657997	10,359695	2,43237539	0,15237264	0,44457995	RPS12	ribosomal protein S12 [Source:HGNC Symbol;Acc:HGNC:10385]
ENSG00000171462	0,35225806	1,42763186	2,43255265	0,1523593	0,44457995	DLK2	delta like non-canonical Notch ligand 2 [Source:HGNC Symbol;Acc:HGNC:21113]
ENSG00000006451	-0,2900016	5,01487249	2,42475816	0,15294731	0,44495688	RALA	RAS like proto-oncogene A [Source:HGNC Symbol;Acc:HGNC:9839]
ENSG00000114978	0,25048718	5,85984563	2,42787292	0,15271198	0,44495688	MOB1A	MOB kinase activator 1A [Source:HGNC Symbol;Acc:HGNC:16015]
ENSG00000139266	0,2712854	3,24371072	2,42852589	0,1526627	0,44495688	MARCHF9	membrane associated ring-CH-type finger 9 [Source:HGNC Symbol;Acc:HGNC:25139]
ENSG00000146576	0,25872478	4,74020078	2,42597475	0,15285534	0,44495688	C7orf26	chromosome 7 open reading frame 26 [Source:HGNC Symbol;Acc:HGNC:21702]
ENSG00000152223	0,35668314	2,77259701	2,42786381	0,15271267	0,44495688	EPG5	ectopic P-granules autophagy protein 5 homolog [Source:HGNC Symbol;Acc:HGNC:29331]
ENSG00000173511	-0,2664899	4,69031971	2,42479151	0,15294479	0,44495688	VEGFB	vascular endothelial growth factor B [Source:HGNC Symbol;Acc:HGNC:12681]
ENSG00000186787	0,40467218	0,88452928	2,42909523	0,15261976	0,44495688	SPIN2B	spindlin family member 2B [Source:HGNC Symbol;Acc:HGNC:33147]
ENSG00000196159	1,12948545	-0,8421643	2,42741129	0,15274683	0,44495688	FAT4	FAT atypical cadherin 4 [Source:HGNC Symbol;Acc:HGNC:23109]

ENSG00000198522	0,21307446	6,29803194	2,42521978	0,1529124	0,44495688	GPN1	GPN-loop GTPase 1 [Source:HGNC Symbol;Acc:HGNC:17030]
ENSG00000214362	0,95615075	-1,626318	2,4278216	0,15271585	0,44495688	RPS3AP36	RPS3A pseudogene 36 [Source:HGNC Symbol;Acc:HGNC:35596]
ENSG00000217094	-0,3049121	2,61474724	2,42584103	0,15286544	0,44495688	PPIAP31	peptidylprolyl isomerase A pseudogene 31 [Source:HGNC Symbol;Acc:HGNC:44962]
ENSG00000226419	-0,5020933	0,90332848	2,42712175	0,15276869	0,44495688	SLC16A1-AS1	SLC16A1 antisense RNA 1 [Source:HGNC Symbol;Acc:HGNC:49445]
ENSG00000258738	-0,2736259	2,17362535	2,42460778	0,15295869	0,44495688	AL121603.2	novel transcript, antisense to BAZ1A
ENSG00000260778	-0,7872582	-0,8286226	2,42728739	0,15275618	0,44495688	AC009065.4	novel transcript
ENSG00000261270	0,7103949	-1,020671	2,42653203	0,15281323	0,44495688	AC012181.2	novel transcript, sense intronic to HERPUD1
ENSG00000157107	0,42986242	2,25298058	2,4239728	0,15300672	0,44499433	FCHO2	FCH and mu domain containing endocytic adaptor 2 [Source:HGNC Symbol;Acc:HGNC:25180]
ENSG00000240889	0,68513033	-0,6237719	2,42363242	0,15303248	0,44499433	NDUFB2-AS1	NDUFB2 antisense RNA 1 [Source:HGNC Symbol;Acc:HGNC:40396]
ENSG00000005810	0,40385219	3,81927034	2,42075189	0,1532507	0,4453792	MYCBP2	MYC binding protein 2 [Source:HGNC Symbol;Acc:HGNC:23386]
ENSG00000008735	0,39751528	4,36343759	2,4186691	0,15340873	0,4453792	MAPK8IP2	mitogen-activated protein kinase 8 interacting protein 2 [Source:HGNC Symbol;Acc:HGNC:6883]
ENSG00000110719	-0,3150491	4,36705187	2,42033544	0,15328228	0,4453792	TCIRG1	T cell immune regulator 1, ATPase H+ transporting V0 subunit a3 [Source:HGNC Symbol;Acc:HGNC:11647]
ENSG00000137825	-0,2340138	5,71384294	2,42094905	0,15323575	0,4453792	ITPKA	inositol-trisphosphate 3-kinase A [Source:HGNC Symbol;Acc:HGNC:6178]
ENSG00000172171	0,25994039	3,42443179	2,42019754	0,15329274	0,4453792	TEFM	transcription elongation factor, mitochondrial [Source:HGNC Symbol;Acc:HGNC:26223]
ENSG00000218014	-0,9815952	-1,582579	2,41974932	0,15332674	0,4453792	KRT19P1	keratin 19 pseudogene 1 [Source:HGNC Symbol;Acc:HGNC:33422]
ENSG00000233966	-0,4588159	0,32147872	2,41885098	0,15339492	0,4453792	UBE2SP1	ubiquitin conjugating enzyme E2 S pseudogene 1 [Source:HGNC Symbol;Acc:HGNC:30864]
ENSG00000281706	-0,3627728	0,83913784	2,41921973	0,15336693	0,4453792	LINC01012	long intergenic non-protein coding RNA 1012 [Source:NCBI gene (formerly Entrezgene);Acc:100507173]
ENSG00000007047	-0,2365517	4,21660227	2,41435215	0,15373696	0,44556878	MARK4	microtubule affinity regulating kinase 4 [Source:HGNC Symbol;Acc:HGNC:13538]
ENSG00000078902	0,22300504	6,28316204	2,4121989	0,15390103	0,44556878	TOLLIP	toll interacting protein [Source:HGNC Symbol;Acc:HGNC:16476]
ENSG00000119820	0,32858426	5,48329952	2,41694324	0,15353984	0,44556878	YIPF4	Yip1 domain family member 4 [Source:HGNC Symbol;Acc:HGNC:28145]
ENSG00000124243	0,24642927	3,3361513	2,41384239	0,15377578	0,44556878	BCAS4	breast carcinoma amplified sequence 4 [Source:HGNC Symbol;Acc:HGNC:14367]
ENSG00000125107	0,20888222	7,15348246	2,41256296	0,15387327	0,44556878	CNOT1	CCR4-NOT transcription complex subunit 1 [Source:HGNC Symbol;Acc:HGNC:7877]
ENSG00000135387	0,28021314	7,57211874	2,41420588	0,1537481	0,44556878	CAPRIN1	cell cycle associated protein 1 [Source:HGNC Symbol;Acc:HGNC:6743]
ENSG00000147889	-0,3951096	0,72320406	2,41630488	0,15358838	0,44556878	CDKN2A	cyclin dependent kinase inhibitor 2A [Source:HGNC Symbol;Acc:HGNC:1787]
ENSG00000164062	-0,3049809	7,44965969	2,41367176	0,15378878	0,44556878	APEH	acylaminoacyl-peptide hydrolase [Source:HGNC Symbol;Acc:HGNC:586]
ENSG00000164904	0,26901168	7,38422568	2,41676641	0,15355329	0,44556878	ALDH7A1	aldehyde dehydrogenase 7 family member A1 [Source:HGNC Symbol;Acc:HGNC:877]
ENSG00000172869	0,29285394	1,90690881	2,41258264	0,15387177	0,44556878	DMXL1	Dmx like 1 [Source:HGNC Symbol;Acc:HGNC:2937]
ENSG00000183044	-0,2839666	6,91466462	2,41296874	0,15384234	0,44556878	ABAT	4-aminobutyrate aminotransferase [Source:HGNC Symbol;Acc:HGNC:23]
ENSG00000185909	0,29242932	3,83829411	2,41574455	0,15363099	0,44556878	KLHDC8B	kelch domain containing 8B [Source:HGNC Symbol;Acc:HGNC:28557]
ENSG00000264112	0,61254457	0,77026837	2,41616016	0,15359938	0,44556878	AC015813.1	novel transcript
ENSG00000268001	-0,5992223	-0,7433111	2,4126681	0,15386526	0,44556878	CARD8-AS1	CARD8 antisense RNA 1 [Source:HGNC Symbol;Acc:HGNC:51408]
ENSG00000120756	0,40016836	3,91406078	2,4114009	0,15396188	0,44558489	PLS1	plastin 1 [Source:HGNC Symbol;Acc:HGNC:9090]
ENSG00000166444	0,25018156	4,36046303	2,41132613	0,15396759	0,44558489	DENND2B	DENN domain containing 2B [Source:HGNC Symbol;Acc:HGNC:11350]
ENSG00000133114	-0,2262584	3,8477456	2,40935617	0,15411797	0,44593175	GPALPP1	GPALPP motifs containing 1 [Source:HGNC Symbol;Acc:HGNC:20298]
ENSG00000105953	-0,4235142	5,3028159	2,40864592	0,15417224	0,44600044	OGDH	oxoglutarate dehydrogenase [Source:HGNC Symbol;Acc:HGNC:8124]
ENSG00000280187	-0,6061721	-0,5025798	2,40786401	0,15423201	0,44608501	AC022107.1	TEC
ENSG00000063169	0,50166546	1,22262486	2,40531578	0,154427	0,44629557	BICRA	BRD4 interacting chromatin remodeling complex associated protein [Source:HGNC Symbol;Acc:HGNC:4332]
ENSG00000078687	0,86495455	2,0039848	2,40561138	0,15440437	0,44629557	TNRC6C	trinucleotide repeat containing adaptor 6C [Source:HGNC Symbol;Acc:HGNC:29318]
ENSG00000109674	0,31207841	1,50197011	2,40642935	0,15434175	0,44629557	NEIL3	nei like DNA glycosylase 3 [Source:HGNC Symbol;Acc:HGNC:24573]
ENSG00000110911	-0,2365882	5,42340701	2,40566702	0,15440011	0,44629557	SLC11A2	solute carrier family 11 member 2 [Source:HGNC Symbol;Acc:HGNC:10908]
ENSG00000137269	0,24041362	5,05610037	2,40472082	0,15447258	0,44632286	LRRC1	leucine rich repeat containing 1 [Source:HGNC Symbol;Acc:HGNC:14307]
ENSG00000205358	-0,6113199	-1,0644437	2,40439492	0,15449755	0,44632286	MT1H	metallothionein 1H [Source:HGNC Symbol;Acc:HGNC:7400]
ENSG00000189362	-0,2944961	3,64226679	2,40374264	0,15454755	0,44637902	NEMP2	nuclear envelope integral membrane protein 2 [Source:HGNC Symbol;Acc:HGNC:33700]
ENSG00000171827	0,48083219	1,09680695	2,40211757	0,1546722	0,44665074	ZNF570	zinc finger protein 570 [Source:HGNC Symbol;Acc:HGNC:26416]

ENSG00000100219	0,22021324	9,12385094	2,4009636	0,15476079	0,44681826	XBP1	X-box binding protein 1 [Source:HGNC Symbol;Acc:HGNC:12801]
ENSG00000123933	-0,217263	5,48286926	2,40055526	0,15479216	0,44682051	MXD4	MAX dimerization protein 4 [Source:HGNC Symbol;Acc:HGNC:13906]
ENSG00000243679	0,64112858	0,39631336	2,39903061	0,15490934	0,44707044	AC018638.5	novel pseudogene
ENSG00000130159	-0,2921147	5,69159099	2,39680874	0,15508032	0,4474755	ECSIT	ECSIT signaling integrator [Source:HGNC Symbol;Acc:HGNC:29548]
ENSG00000066933	0,39937749	2,01904495	2,39476483	0,15523783	0,44781304	MYO9A	myosin IXA [Source:HGNC Symbol;Acc:HGNC:7608]
ENSG00000103266	-0,2208694	6,6177966	2,39449533	0,15525861	0,44781304	STUB1	STIP1 homology and U-box containing protein 1 [Source:HGNC Symbol;Acc:HGNC:11427]
ENSG00000139718	0,56886169	3,06860243	2,39314714	0,15536264	0,44783122	SETD1B	SET domain containing 1B, histone lysine methyltransferase [Source:HGNC Symbol;Acc:HGNC:29187]
ENSG00000164061	0,68017785	1,46917649	2,39192053	0,15545736	0,44783122	BSN	bassoon presynaptic cytomatrix protein [Source:HGNC Symbol;Acc:HGNC:1117]
ENSG00000166153	0,44764159	0,25884581	2,39197894	0,15545285	0,44783122	DEPDC4	DEP domain containing 4 [Source:HGNC Symbol;Acc:HGNC:22952]
ENSG00000197077	0,35300191	3,59546988	2,39277303	0,15539152	0,44783122	KIAA1671	KIAA1671 [Source:HGNC Symbol;Acc:HGNC:29345]
ENSG00000198874	0,23331418	4,71676345	2,39163402	0,1554795	0,44783122	TYW1	tRNA-yW synthesizing protein 1 homolog [Source:HGNC Symbol;Acc:HGNC:25598]
ENSG00000226580	-0,9956502	-1,3457926	2,39385794	0,15530778	0,44783122	RPL39P40	ribosomal protein L39 pseudogene 40 [Source:HGNC Symbol;Acc:HGNC:35823]
ENSG00000254612	0,67777942	-0,583589	2,39321994	0,15535702	0,44783122	DNAJB6P1	DNAJB6 pseudogene 1 [Source:HGNC Symbol;Acc:HGNC:54754]
ENSG00000146802	0,31973158	2,54807571	2,39109468	0,15552118	0,44786298	TMEM168	transmembrane protein 168 [Source:HGNC Symbol;Acc:HGNC:25826]
ENSG00000113013	0,3929908	9,57887049	2,39049551	0,1555675	0,44786553	HSPA9	heat shock protein family A (Hsp70) member 9 [Source:HGNC Symbol;Acc:HGNC:5244]
ENSG00000167085	0,20169305	10,0160818	2,38989382	0,15561403	0,44786553	PHB	prohibitin [Source:HGNC Symbol;Acc:HGNC:8912]
ENSG00000178723	0,62623044	-0,8587871	2,38995912	0,15560898	0,44786553	GLULP4	glutamate-ammonia ligase pseudogene 4 [Source:HGNC Symbol;Acc:HGNC:4345]
ENSG00000204396	-0,4878973	0,03569013	2,38736751	0,15580962	0,44834011	VWA7	von Willebrand factor A domain containing 7 [Source:HGNC Symbol;Acc:HGNC:13939]
ENSG00000139540	-0,2227631	6,8412466	2,38600245	0,15591544	0,44855624	SLC39A5	solute carrier family 39 member 5 [Source:HGNC Symbol;Acc:HGNC:20502]
ENSG00000053371	-0,2114739	6,52126445	2,3852348	0,15597498	0,44863921	AKR7A2	aldo-keto reductase family 7 member A2 [Source:HGNC Symbol;Acc:HGNC:389]
ENSG00000112599	-0,8184158	-1,4321812	2,38478634	0,15600978	0,44865097	GUCA1B	guanylate cyclase activator 1B [Source:HGNC Symbol;Acc:HGNC:4679]
ENSG00000149357	-0,2495796	6,54265707	2,38348821	0,15611058	0,44876416	LAMTOR1	late endosomal/lysosomal adaptor, MAPK and MTOR activator 1 [Source:HGNC Symbol;Acc:HGNC:26068]
ENSG00000214331	0,31911655	1,87331251	2,38373105	0,15609172	0,44876416	AC009053.1	pyruvate dehydrogenase phosphatase regulatory subunit (PDPR) pseudogene
ENSG00000111203	-0,2433424	2,90952699	2,38288365	0,15615755	0,44881087	ITFG2	integrin alpha FG-GAP repeat containing 2 [Source:HGNC Symbol;Acc:HGNC:30879]
ENSG00000171219	0,43139003	1,44632989	2,3819295	0,15623172	0,44884743	CDC42BPG	CDC42 binding protein kinase gamma [Source:HGNC Symbol;Acc:HGNC:29829]
ENSG00000198466	0,40045867	2,03351239	2,38224812	0,15620695	0,44884743	ZNF587	zinc finger protein 587 [Source:HGNC Symbol;Acc:HGNC:30955]
ENSG00000122591	0,24877193	4,37690537	2,3808567	0,15631517	0,44891061	FAM126A	family with sequence similarity 126 member A [Source:HGNC Symbol;Acc:HGNC:24587]
ENSG00000181896	-0,32468	3,67339584	2,38089553	0,15631214	0,44891061	ZNF101	zinc finger protein 101 [Source:HGNC Symbol;Acc:HGNC:12881]
ENSG00000132122	0,71493774	-0,199463	2,38004887	0,15637804	0,44900291	SPATA6	spermatogenesis associated 6 [Source:HGNC Symbol;Acc:HGNC:18309]
ENSG00000130695	0,28787231	4,56888124	2,37853672	0,15649582	0,4492528	CEP85	centrosomal protein 85 [Source:HGNC Symbol;Acc:HGNC:25309]
ENSG00000110696	0,28067742	7,31126237	2,37695707	0,15661899	0,44951804	C11orf58	chromosome 11 open reading frame 58 [Source:HGNC Symbol;Acc:HGNC:16990]
ENSG00000005448	0,38602201	2,54831372	2,37438489	0,15681981	0,44967699	WDR54	WD repeat domain 54 [Source:HGNC Symbol;Acc:HGNC:25770]
ENSG00000096717	0,23232463	3,51846437	2,37481558	0,15678616	0,44967699	SIRT1	sirtuin 1 [Source:HGNC Symbol;Acc:HGNC:14929]
ENSG00000164684	0,53105416	2,57470866	2,37484793	0,15678363	0,44967699	ZNF704	zinc finger protein 704 [Source:HGNC Symbol;Acc:HGNC:32291]
ENSG00000172748	0,47954144	0,35680926	2,37427657	0,15682828	0,44967699	ZNF596	zinc finger protein 596 [Source:HGNC Symbol;Acc:HGNC:27268]
ENSG00000180879	-0,2037998	8,91430345	2,37464341	0,15679961	0,44967699	SSR4	signal sequence receptor subunit 4 [Source:HGNC Symbol;Acc:HGNC:11326]
ENSG00000197044	0,31243328	2,97690371	2,37340153	0,15689668	0,44978484	ZNF441	zinc finger protein 441 [Source:HGNC Symbol;Acc:HGNC:20875]
ENSG00000263235	-0,8062865	-1,0770318	2,37180252	0,15702177	0,45005513	AC006111.2	novel transcript, sense overlapping TRAP1
ENSG00000099246	0,22265862	5,3470828	2,37129635	0,15706139	0,4500804	RAB18	RAB18, member RAS oncogene family [Source:HGNC Symbol;Acc:HGNC:14244]
ENSG00000115207	0,2077814	6,6993167	2,36940297	0,15720973	0,45041714	GTF3C2	general transcription factor IIIC subunit 2 [Source:HGNC Symbol;Acc:HGNC:4665]
ENSG00000007237	0,447683	0,13199913	2,36835349	0,15729203	0,45056457	GAS7	growth arrest specific 7 [Source:HGNC Symbol;Acc:HGNC:4169]
ENSG00000245888	0,80858294	-1,2221587	2,36754135	0,15735576	0,45065876	NSMCE1-DT	NSMCE1 divergent transcript [Source:HGNC Symbol;Acc:HGNC:51368]
ENSG00000102230	0,23876002	3,85648716	2,36588839	0,15748557	0,45067712	PCYT1B	phosphate cytidylyltransferase 1, choline, beta [Source:HGNC Symbol;Acc:HGNC:8755]
ENSG00000154978	0,21448251	5,73847316	2,36703373	0,15739561	0,45067712	VOPP1	VOPP1 WW domain binding protein [Source:HGNC Symbol;Acc:HGNC:34518]

ENSG00000185246	0,24573525	3,99444661	2,36639392	0,15744586	0,45067712	PRPF39	pre-mRNA processing factor 39 [Source:HGNC Symbol;Acc:HGNC:20314]
ENSG00000266200	-1,0383209	-2,0379484	2,36592497	0,1574827	0,45067712	PNLIPRP2	pancreatic lipase related protein 2 (gene/pseudogene) [Source:HGNC Symbol;Acc:HGNC:9157]
ENSG00000110777	-0,4702977	0,21128661	2,36452214	0,15759297	0,45080786	POU2AF1	POU class 2 homeobox associating factor 1 [Source:HGNC Symbol;Acc:HGNC:9211]
ENSG00000116266	-0,2866584	3,59297063	2,36483944	0,15756802	0,45080786	STXBP3	syntaxin binding protein 3 [Source:HGNC Symbol;Acc:HGNC:11446]
ENSG00000089916	0,33721099	3,33085688	2,3631074	0,15770428	0,45097638	GPATCH2L	G-patch domain containing 2 like [Source:HGNC Symbol;Acc:HGNC:20210]
ENSG00000128655	0,90681406	-1,1640456	2,36249491	0,15775251	0,45097638	PDE11A	phosphodiesterase 11A [Source:HGNC Symbol;Acc:HGNC:8773]
ENSG00000143815	0,25105616	6,29905024	2,36220473	0,15777536	0,45097638	LBR	lamin B receptor [Source:HGNC Symbol;Acc:HGNC:6518]
ENSG00000285230	-0,390802	0,94787088	2,36268275	0,15773772	0,45097638	RALY-AS1	RALY antisense RNA 1 [Source:HGNC Symbol;Acc:HGNC:50743]
ENSG00000148248	0,20324898	8,95475228	2,3612396	0,1578514	0,45110547	SURF4	surfeit 4 [Source:HGNC Symbol;Acc:HGNC:11476]
ENSG00000147548	0,22728316	3,97768013	2,35987041	0,15795936	0,45123778	NSD3	nuclear receptor binding SET domain protein 3 [Source:HGNC Symbol;Acc:HGNC:12767]
ENSG00000232000	-0,538497	-0,6088124	2,35986894	0,15795948	0,45123778	CLCN3P1	chloride voltage-gated channel 3 pseudogene 1 [Source:HGNC Symbol;Acc:HGNC:49775]
ENSG00000225580	-0,5827178	-0,6908872	2,3591012	0,15802005	0,45132257	PA2G4P5	proliferation-associated 2G4 pseudogene 5 [Source:HGNC Symbol;Acc:HGNC:32218]
ENSG00000110514	0,29056629	4,9749117	2,35571757	0,1582874	0,45179234	MADD	MAP kinase activating death domain [Source:HGNC Symbol;Acc:HGNC:6766]
ENSG00000239883	0,38371286	1,06850274	2,35582308	0,15827905	0,45179234	PARGP1	poly(ADP-ribose) glycohydrolase pseudogene 1 [Source:HGNC Symbol;Acc:HGNC:45008]
ENSG00000254543	0,4458236	0,77293405	2,35558728	0,1582977	0,45179234	HSPD1P2	heat shock protein family D (Hsp60) member 1 pseudogene 2 [Source:HGNC Symbol;Acc:HGNC:5263]
ENSG00000268061	-0,9777435	-1,5399959	2,35545417	0,15830823	0,45179234	NAPA-AS1	NAPA antisense RNA 1 [Source:HGNC Symbol;Acc:HGNC:44118]
ENSG00000095319	0,31786347	6,13538414	2,35405077	0,15841931	0,45179864	NUP188	nucleoporin 188 [Source:HGNC Symbol;Acc:HGNC:17859]
ENSG00000186106	0,29994723	3,62373201	2,35453852	0,15838069	0,45179864	ANKRD46	ankyrin repeat domain 46 [Source:HGNC Symbol;Acc:HGNC:27229]
ENSG00000186312	0,39126512	1,73245401	2,35419451	0,15840793	0,45179864	CA5BP1	carbonic anhydrase 5B pseudogene 1 [Source:HGNC Symbol;Acc:HGNC:29544]
ENSG00000198792	0,23075392	5,1277188	2,35386342	0,15843415	0,45179864	TMEM184B	transmembrane protein 184B [Source:HGNC Symbol;Acc:HGNC:1310]
ENSG00000151148	0,27741897	3,81297369	2,35305209	0,15849842	0,45189371	UBE3B	ubiquitin protein ligase E3B [Source:HGNC Symbol;Acc:HGNC:13478]
ENSG00000218537	-0,6476856	-0,7418082	2,35247731	0,15854397	0,45193538	MIF-AS1	MIF antisense RNA 1 [Source:HGNC Symbol;Acc:HGNC:27669]
ENSG00000102996	0,22744216	7,58940593	2,35124566	0,15864163	0,45194923	MMP15	matrix metallopeptidase 15 [Source:HGNC Symbol;Acc:HGNC:7161]
ENSG00000107833	-0,2289809	6,98599022	2,35167271	0,15860776	0,45194923	NPM3	nucleophosmin/nucleoplasmin 3 [Source:HGNC Symbol;Acc:HGNC:7931]
ENSG00000196547	0,30285153	5,40928183	2,35138546	0,15863054	0,45194923	MAN2A2	mannosidase alpha class 2A member 2 [Source:HGNC Symbol;Acc:HGNC:6825]
ENSG00000125734	-0,2370958	5,4614438	2,3507707	0,15867932	0,45196845	GPR108	G protein-coupled receptor 108 [Source:HGNC Symbol;Acc:HGNC:17829]
ENSG00000090924	-0,3901174	2,54795978	2,35033683	0,15871375	0,45197841	PLEKHG2	pleckstrin homology and RhoGEF domain containing G2 [Source:HGNC Symbol;Acc:HGNC:29515]
ENSG00000113597	0,28065376	3,48086962	2,349554	0,15877591	0,45206728	TRAPPC13	trafficking protein particle complex 13 [Source:HGNC Symbol;Acc:HGNC:25828]
ENSG00000136045	0,21175609	6,76412048	2,34766157	0,15892629	0,45240729	PWP1	PWP1 homolog, endonuclein [Source:HGNC Symbol;Acc:HGNC:17015]
ENSG00000157429	-0,4143769	1,7170003	2,34705701	0,15897438	0,452456	ZNF19	zinc finger protein 19 [Source:HGNC Symbol;Acc:HGNC:12981]
ENSG00000136205	0,38179203	5,76517869	2,34549339	0,15909882	0,45272199	TNS3	tensin 3 [Source:HGNC Symbol;Acc:HGNC:21616]
ENSG00000167861	-0,3470403	2,26411519	2,3436221	0,15924792	0,45305801	HID1	HID1 domain containing [Source:HGNC Symbol;Acc:HGNC:15736]
ENSG00000204947	-0,7860692	-0,2971379	2,34262687	0,15932729	0,45319557	ZNF425	zinc finger protein 425 [Source:HGNC Symbol;Acc:HGNC:20690]
ENSG00000162775	0,25200452	3,5989701	2,34133157	0,15943068	0,45340135	RBM15	RNA binding motif protein 15 [Source:HGNC Symbol;Acc:HGNC:14959]
ENSG00000099974	-0,7705613	-1,7508302	2,34011341	0,15952798	0,45346481	DDTL	D-dopachrome tautomerase like [Source:HGNC Symbol;Acc:HGNC:33446]
ENSG00000173611	0,29528405	2,2709773	2,34053145	0,15949458	0,45346481	SCAI	suppressor of cancer cell invasion [Source:HGNC Symbol;Acc:HGNC:26709]
ENSG00000188130	-0,2389796	4,92036352	2,33988656	0,15954611	0,45346481	MAPK12	mitogen-activated protein kinase 12 [Source:HGNC Symbol;Acc:HGNC:6874]
ENSG00000108107	-0,230107	9,98892602	2,33933884	0,15958989	0,45350102	RPL28	ribosomal protein L28 [Source:HGNC Symbol;Acc:HGNC:10330]
ENSG00000270706	-0,9660956	-1,1231854	2,33850686	0,15965643	0,45360186	PRMT1P1	protein arginine methyltransferase 1 pseudogene 1 [Source:HGNC Symbol;Acc:HGNC:49611]
ENSG00000216624	-0,4254175	0,52027266	2,33778681	0,15971404	0,45367732	GAPDHP72	glyceraldehyde-3-phosphate dehydrogenase pseudogene 72 [Source:HGNC Symbol;Acc:HGNC:22955]
ENSG00000176208	-0,2820327	2,23876366	2,3360851	0,15985031	0,45397613	ATAD5	ATPase family AAA domain containing 5 [Source:HGNC Symbol;Acc:HGNC:25752]
ENSG00000154781	-0,2781044	4,19135868	2,33532025	0,15991161	0,45406194	CCDC174	coiled-coil domain containing 174 [Source:HGNC Symbol;Acc:HGNC:28033]
ENSG00000168404	-0,2311598	3,32166194	2,33454015	0,15997416	0,45415128	MLKL	mixed lineage kinase domain like pseudokinase [Source:HGNC Symbol;Acc:HGNC:26617]
ENSG00000138759	0,51033229	5,65466038	2,33374123	0,16003825	0,45415672	FRAS1	Fraser extracellular matrix complex subunit 1 [Source:HGNC Symbol;Acc:HGNC:19185]

ENSG00000151718	0,23151295	4,58719459	2,33411526	0,16000824	0,45415672	WWC2	WW and C2 domain containing 2 [Source:HGNC Symbol;Acc:HGNC:24148]
ENSG00000143198	0,19970866	6,44572214	2,33221877	0,16016048	0,45425416	MGST3	microsomal glutathione S-transferase 3 [Source:HGNC Symbol;Acc:HGNC:7064]
ENSG00000176273	0,40786495	1,53250973	2,33215171	0,16016587	0,45425416	SLC35G1	solute carrier family 35 member G1 [Source:HGNC Symbol;Acc:HGNC:26607]
ENSG00000258651	-0,6419979	-0,4755571	2,33222109	0,1601603	0,45425416	SEC23A-AS1	SEC23A antisense RNA 1 [Source:HGNC Symbol;Acc:HGNC:53213]
ENSG00000163093	0,30609223	2,46274081	2,33116172	0,16024542	0,45439157	BBS5	Bardet-Biedl syndrome 5 [Source:HGNC Symbol;Acc:HGNC:970]
ENSG00000077585	-0,7913029	-0,8758335	2,32986524	0,16034968	0,45452979	GPR137B	G protein-coupled receptor 137B [Source:HGNC Symbol;Acc:HGNC:11862]
ENSG00000104964	0,22528428	7,15927346	2,32900993	0,16041851	0,45452979	TLE5	TLE family member 5, transcriptional modulator [Source:HGNC Symbol;Acc:HGNC:307]
ENSG00000120594	0,59577474	-0,4135804	2,32900853	0,16041862	0,45452979	PLXDC2	plexin domain containing 2 [Source:HGNC Symbol;Acc:HGNC:21013]
ENSG00000169908	-0,2297499	10,011348	2,32931642	0,16039384	0,45452979	TM4SF1	transmembrane 4 L six family member 1 [Source:HGNC Symbol;Acc:HGNC:11853]
ENSG00000009335	0,20527891	5,82704732	2,32687838	0,16059021	0,454681	UBE3C	ubiquitin protein ligase E3C [Source:HGNC Symbol;Acc:HGNC:16803]
ENSG00000136854	-0,3714315	2,82402652	2,32794264	0,16050445	0,454681	STXBP1	syntaxin binding protein 1 [Source:HGNC Symbol;Acc:HGNC:11444]
ENSG00000140264	-0,2540436	9,20488802	2,32680056	0,16059648	0,454681	SERF2	small EDRK-rich factor 2 [Source:HGNC Symbol;Acc:HGNC:10757]
ENSG00000165879	-0,270275	2,35705323	2,32734576	0,16055254	0,454681	FRAT1	FRAT regulator of WNT signaling pathway 1 [Source:HGNC Symbol;Acc:HGNC:3944]
ENSG00000103852	0,37150191	2,79052628	2,32523117	0,16072306	0,4549512	TTC23	tetratricopeptide repeat domain 23 [Source:HGNC Symbol;Acc:HGNC:25730]
ENSG00000134716	0,33639885	2,47306422	2,32455085	0,16077797	0,45501847	CYP2J2	cytochrome P450 family 2 subfamily J member 2 [Source:HGNC Symbol;Acc:HGNC:2634]
ENSG00000023516	0,55703816	2,43884997	2,32120757	0,16104817	0,45525423	AKAP11	A-kinase anchoring protein 11 [Source:HGNC Symbol;Acc:HGNC:369]
ENSG00000137968	-0,4855875	0,15162316	2,32283425	0,16091663	0,45525423	SLC44A5	solute carrier family 44 member 5 [Source:HGNC Symbol;Acc:HGNC:28524]
ENSG00000175550	-0,2406665	5,76513961	2,32082154	0,16107941	0,45525423	DRAP1	DR1 associated protein 1 [Source:HGNC Symbol;Acc:HGNC:3019]
ENSG00000182224	0,48036365	0,62903547	2,32227445	0,16096188	0,45525423	CYB5D1	cytochrome b5 domain containing 1 [Source:HGNC Symbol;Acc:HGNC:26516]
ENSG00000204316	0,7479626	-1,1419412	2,32091157	0,16107213	0,45525423	MRPL38	mitochondrial ribosomal protein L38 [Source:HGNC Symbol;Acc:HGNC:14033]
ENSG00000230490	-0,4860473	0,10847057	2,32185072	0,16099615	0,45525423	AL139383.1	novel transcript
ENSG00000236255	0,44691	0,72295721	2,32224978	0,16096388	0,45525423	AC009404.1	novel transcript
ENSG00000105499	-1,099713	-1,9419585	2,32026962	0,16112409	0,45529241	PLA2G4C	phospholipase A2 group IVC [Source:HGNC Symbol;Acc:HGNC:9037]
ENSG00000149923	-0,2181331	7,94853052	2,31974471	0,16116659	0,45530433	PPP4C	protein phosphatase 4 catalytic subunit [Source:HGNC Symbol;Acc:HGNC:9319]
ENSG00000240891	-0,5420829	-0,0605786	2,31944784	0,16119063	0,45530433	PLCXD2	phosphatidylinositol specific phospholipase C X domain containing 2 [Source:HGNC Symbol;Acc:HGNC:26462]
ENSG00000099326	-0,509542	0,22993942	2,31771654	0,16133096	0,4553654	MZF1	myeloid zinc finger 1 [Source:HGNC Symbol;Acc:HGNC:13108]
ENSG00000100744	-0,2735882	3,41535869	2,31674137	0,16141006	0,4553654	GSKIP	GSK3B interacting protein [Source:HGNC Symbol;Acc:HGNC:20343]
ENSG00000127580	-0,278969	1,89813113	2,31876436	0,16124601	0,4553654	WDR24	WD repeat domain 24 [Source:HGNC Symbol;Acc:HGNC:20852]
ENSG00000135913	0,23116436	3,83618934	2,31577449	0,16148855	0,4553654	USP37	ubiquitin specific peptidase 37 [Source:HGNC Symbol;Acc:HGNC:20063]
ENSG00000144455	0,24585765	5,72581593	2,3155937	0,16150323	0,4553654	SUMF1	sulfatase modifying factor 1 [Source:HGNC Symbol;Acc:HGNC:20376]
ENSG00000185658	0,22768495	3,59723123	2,31710828	0,16138029	0,4553654	BRWD1	bromodomain and WD repeat domain containing 1 [Source:HGNC Symbol;Acc:HGNC:12760]
ENSG00000196510	0,20155484	6,18726206	2,31533018	0,16152463	0,4553654	ANAPC7	anaphase promoting complex subunit 7 [Source:HGNC Symbol;Acc:HGNC:17380]
ENSG00000253910	0,64856036	-0,4977314	2,31766421	0,1613352	0,4553654	PCDHGB2	protocadherin gamma subfamily B, 2 [Source:HGNC Symbol;Acc:HGNC:8709]
ENSG00000268362	0,6620193	0,42906821	2,31589556	0,16147872	0,4553654	AC092279.1	novel transcript
ENSG00000271576	-0,4836866	-0,0034967	2,31495476	0,16155513	0,4553654	AL359504.1	novel transcript
ENSG00000286156	0,36556554	1,16765794	2,31603654	0,16146727	0,4553654	AC026273.1	BMS1, ribosome biogenesis factor (BMS1) pseudogene
ENSG00000196497	0,37924521	0,59647791	2,31453182	0,16158949	0,4553744	IPO4	importin 4 [Source:HGNC Symbol;Acc:HGNC:19426]
ENSG00000237748	0,67818696	-0,9004494	2,31335926	0,16168482	0,45555516	UQCRBP1	ubiquinol-cytochrome c reductase binding protein pseudogene 1 [Source:HGNC Symbol;Acc:HGNC:12583]
ENSG00000213186	0,725548	-0,7054217	2,31246587	0,1617575	0,45567206	TRIM59	tripartite motif containing 59 [Source:HGNC Symbol;Acc:HGNC:30834]
ENSG00000186076	-0,378883	0,6079227	2,31193715	0,16180053	0,45570541	AC012085.1	phosphoglycerate mutase 1 (brain) (PGAM1) pseudogene
ENSG00000127334	0,28652878	2,22394862	2,31145002	0,16184019	0,45572926	DYRK2	dual specificity tyrosine phosphorylation regulated kinase 2 [Source:HGNC Symbol;Acc:HGNC:3093]
ENSG00000121075	-0,6290632	-0,4666291	2,31047772	0,16191939	0,45577658	TBX4	T-box transcription factor 4 [Source:HGNC Symbol;Acc:HGNC:11603]
ENSG00000283050	0,55701695	0,82870258	2,31051957	0,16191598	0,45577658	GTF2IP12	general transcription factor IIi pseudogene 12 [Source:HGNC Symbol;Acc:HGNC:51723]
ENSG00000128789	0,26056777	6,12695407	2,30956702	0,16199362	0,455819	PSMG2	proteasome assembly chaperone 2 [Source:HGNC Symbol;Acc:HGNC:24929]

ENSG00000163875	0,24962088	5,2891383	2,30952722	0,16199687	0,455819	MEAF6	MYST/Esa1 associated factor 6 [Source:HGNC Symbol;Acc:HGNC:25674]
ENSG00000121067	0,23157921	5,75303473	2,30851868	0,16207913	0,45587485	SPOP	speckle type BTB/POZ protein [Source:HGNC Symbol;Acc:HGNC:11254]
ENSG00000139537	0,6184029	-0,3155611	2,30876459	0,16205907	0,45587485	CCDC65	coiled-coil domain containing 65 [Source:HGNC Symbol;Acc:HGNC:29937]
ENSG00000010270	-0,3088557	4,83276902	2,3075305	0,16215978	0,45592613	STARD3NL	STARD3 N-terminal like [Source:HGNC Symbol;Acc:HGNC:19169]
ENSG00000204520	0,22554813	7,10906007	2,30769878	0,16214604	0,45592613	MICA	MHC class I polypeptide-related sequence A [Source:HGNC Symbol;Acc:HGNC:7090]
ENSG00000072135	-0,2213454	4,47511977	2,30443359	0,16241288	0,45609156	PTPN18	protein tyrosine phosphatase non-receptor type 18 [Source:HGNC Symbol;Acc:HGNC:9649]
ENSG00000107263	0,2960149	4,76974598	2,30516656	0,16235293	0,45609156	RAPGEF1	Rap guanine nucleotide exchange factor 1 [Source:HGNC Symbol;Acc:HGNC:4568]
ENSG00000116120	-0,2546784	6,83837221	2,30375537	0,16246837	0,45609156	FARSB	phenylalanyl-tRNA synthetase subunit beta [Source:HGNC Symbol;Acc:HGNC:17800]
ENSG00000121892	0,21330807	5,71028769	2,30540722	0,16233325	0,45609156	PDS5A	PDS5 cohesin associated factor A [Source:HGNC Symbol;Acc:HGNC:29088]
ENSG00000165097	-0,2581732	4,01871542	2,30522972	0,16234776	0,45609156	KDM1B	lysine demethylase 1B [Source:HGNC Symbol;Acc:HGNC:21577]
ENSG00000170903	0,24476991	3,62211517	2,30611155	0,16227568	0,45609156	MSANTD4	Myb/SANT DNA binding domain containing 4 with coiled-coils [Source:HGNC Symbol;Acc:HGNC:29383]
ENSG00000174442	0,22515927	4,33448558	2,30398938	0,16244922	0,45609156	ZWILCH	zwilch kinetochore protein [Source:HGNC Symbol;Acc:HGNC:25468]
ENSG00000175556	-0,3198221	2,38296055	2,30473719	0,16238804	0,45609156	LONRF3	LON peptidase N-terminal domain and ring finger 3 [Source:HGNC Symbol;Acc:HGNC:21152]
ENSG00000122482	0,28587908	3,72534639	2,30291295	0,16253734	0,4561975	ZNF644	zinc finger protein 644 [Source:HGNC Symbol;Acc:HGNC:29222]
ENSG00000166803	0,37824869	2,25854029	2,30204109	0,16260876	0,45631029	PCLAF	PCNA clamp associated factor [Source:HGNC Symbol;Acc:HGNC:28961]
ENSG00000136810	0,23349556	8,65017262	2,30015725	0,16276321	0,45648067	TXN	thioredoxin [Source:HGNC Symbol;Acc:HGNC:12435]
ENSG00000143811	-0,2127392	4,80665648	2,30023785	0,1627566	0,45648067	PYCR2	pyrroline-5-carboxylate reductase 2 [Source:HGNC Symbol;Acc:HGNC:30262]
ENSG00000171700	-0,2723968	3,70129874	2,30070824	0,16271802	0,45648067	RGS19	regulator of G protein signaling 19 [Source:HGNC Symbol;Acc:HGNC:13735]
ENSG00000253719	0,2211225	5,38136623	2,29958197	0,16281042	0,45652542	ATXN7L3B	ataxin 7 like 3B [Source:HGNC Symbol;Acc:HGNC:37931]
ENSG00000179455	0,50864822	0,87891049	2,29703391	0,16301972	0,45702458	MKRN3	makorin ring finger protein 3 [Source:HGNC Symbol;Acc:HGNC:7114]
ENSG00000143248	-0,8073411	-1,5257068	2,29595617	0,16310835	0,45718533	RGS5	regulator of G protein signaling 5 [Source:HGNC Symbol;Acc:HGNC:10001]
ENSG00000173145	-0,2978238	4,69996864	2,29434289	0,16324115	0,45746977	NOC3L	NOC3 like DNA replication regulator [Source:HGNC Symbol;Acc:HGNC:24034]
ENSG00000078967	0,2762826	3,88564414	2,28965085	0,16362817	0,45774973	UBE2D4	ubiquitin conjugating enzyme E2 D4 (putative) [Source:HGNC Symbol;Acc:HGNC:21647]
ENSG00000100815	0,27898719	3,54603618	2,29172739	0,16345674	0,45774973	TRIP11	thyroid hormone receptor interactor 11 [Source:HGNC Symbol;Acc:HGNC:12305]
ENSG00000120696	0,25202937	2,5032477	2,2912537	0,16349582	0,45774973	KBTBD7	kelch repeat and BTB domain containing 7 [Source:HGNC Symbol;Acc:HGNC:25266]
ENSG00000132017	-0,2994139	4,53186292	2,28915532	0,16366911	0,45774973	DCAF15	DDB1 and CUL4 associated factor 15 [Source:HGNC Symbol;Acc:HGNC:25095]
ENSG00000132688	-0,7660409	5,93400945	2,2918389	0,16344754	0,45774973	NES	nestin [Source:HGNC Symbol;Acc:HGNC:7756]
ENSG00000150455	-0,4440089	1,93138973	2,29058307	0,16355118	0,45774973	TIRAP	TIR domain containing adaptor protein [Source:HGNC Symbol;Acc:HGNC:17192]
ENSG00000166987	0,30088453	3,07068608	2,29032009	0,16357289	0,45774973	MBD6	methyl-CpG binding domain protein 6 [Source:HGNC Symbol;Acc:HGNC:20445]
ENSG00000168268	-0,2025564	8,10362247	2,28946673	0,16364338	0,45774973	NT5DC2	5'-nucleotidase domain containing 2 [Source:HGNC Symbol;Acc:HGNC:25717]
ENSG00000196652	0,22942739	4,18218183	2,29095132	0,16352078	0,45774973	ZKSCAN5	zinc finger with KRAB and SCAN domains 5 [Source:HGNC Symbol;Acc:HGNC:12867]
ENSG00000227337	0,6950793	-1,3033311	2,28895448	0,16368571	0,45774973	RPL23AP43	ribosomal protein L23a pseudogene 43 [Source:HGNC Symbol;Acc:HGNC:35751]
ENSG00000228335	0,54124381	-0,270452	2,29137696	0,16348565	0,45774973	AC073063.1	cytokine induced protein 29 kDa (CIP29) pseudogene
ENSG00000100532	0,21886399	4,13550039	2,28790898	0,16377216	0,45787902	CGRRF1	cell growth regulator with ring finger domain 1 [Source:HGNC Symbol;Acc:HGNC:15528]
ENSG00000249459	0,94848519	-0,6182831	2,28763724	0,16379463	0,45787902	ZNF286B	zinc finger protein 286B (pseudogene) [Source:HGNC Symbol;Acc:HGNC:33241]
ENSG00000222038	-0,3521006	1,86729553	2,2853541	0,16398365	0,4583197	POTEJ	POTE ankyrin domain family member J [Source:HGNC Symbol;Acc:HGNC:37094]
ENSG00000108788	-0,2265792	6,26133217	2,28486478	0,16402419	0,45834533	MLX	MAX dimerization protein MLX [Source:HGNC Symbol;Acc:HGNC:11645]
ENSG00000085721	0,2594154	6,10260032	2,28344931	0,16414155	0,45849789	RRN3	RRN3 homolog, RNA polymerase I transcription factor [Source:HGNC Symbol;Acc:HGNC:30346]
ENSG00000169093	-0,3102983	3,9932245	2,28356754	0,16413175	0,45849789	ASMTL	acetylserotonin O-methyltransferase like [Source:HGNC Symbol;Acc:HGNC:751]
ENSG00000235001	-0,2908341	2,20974642	2,28206822	0,16425617	0,45873034	EIF4A1P2	eukaryotic translation initiation factor 4A1 pseudogene 2 [Source:HGNC Symbol;Acc:HGNC:23946]
ENSG00000103423	-0,2555112	6,52385027	2,28148526	0,16430459	0,45877785	DNAJA3	DnaJ heat shock protein family (Hsp40) member A3 [Source:HGNC Symbol;Acc:HGNC:11808]
ENSG00000168701	-0,22818	6,19064099	2,28075323	0,1643654	0,45883116	TMEM208	transmembrane protein 208 [Source:HGNC Symbol;Acc:HGNC:25015]
ENSG00000185650	-0,2817262	4,81461	2,28049947	0,16438649	0,45883116	ZFP36L1	ZFP36 ring finger protein like 1 [Source:HGNC Symbol;Acc:HGNC:1107]
ENSG00000135070	-0,276707	4,46611632	2,27990491	0,16443592	0,45888145	ISCA1	iron-sulfur cluster assembly 1 [Source:HGNC Symbol;Acc:HGNC:28660]

ENSG00000123352	0,22121881	4,54785716	2,27803607	0,16459141	0,45901389	SPATS2	spermatogenesis associated serine rich 2 [Source:HGNC Symbol;Acc:HGNC:18650]
ENSG00000149196	0,24628233	6,14153361	2,27864863	0,16454042	0,45901389	HIKESHI	heat shock protein nuclear import factor hikeshi [Source:HGNC Symbol;Acc:HGNC:26938]
ENSG00000235411	0,60618642	-0,4668296	2,27837028	0,16456359	0,45901389	DRG1P1	DRG1 pseudogene 1 [Source:HGNC Symbol;Acc:HGNC:54701]
ENSG00000265681	-0,3346241	4,27232084	2,27782407	0,16460906	0,45901389	RPL17	ribosomal protein L17 [Source:HGNC Symbol;Acc:HGNC:10307]
ENSG00000111897	0,29481274	6,46690761	2,27650514	0,16471893	0,45917267	SERINC1	serine incorporator 1 [Source:HGNC Symbol;Acc:HGNC:13464]
ENSG00000213442	-0,2502259	3,05813295	2,27638594	0,16472886	0,45917267	RPL18AP3	ribosomal protein L18a pseudogene 3 [Source:HGNC Symbol;Acc:HGNC:31387]
ENSG00000109881	-0,3825636	4,07976396	2,2754658	0,16480557	0,45929886	CCDC34	coiled-coil domain containing 34 [Source:HGNC Symbol;Acc:HGNC:25079]
ENSG00000090273	-0,2509081	8,61878283	2,272323	0,16506794	0,4595487	NUDC	nuclear distribution C, dynein complex regulator [Source:HGNC Symbol;Acc:HGNC:8045]
ENSG00000092607	-1,1056742	-1,5624041	2,27299825	0,16501152	0,4595487	TBX15	T-box transcription factor 15 [Source:HGNC Symbol;Acc:HGNC:11594]
ENSG00000100028	0,2279422	3,86050816	2,27193197	0,16510062	0,4595487	SNRPD3	small nuclear ribonucleoprotein D3 polypeptide [Source:HGNC Symbol;Acc:HGNC:11160]
ENSG00000102786	-0,2284373	4,63623551	2,27133203	0,16515078	0,4595487	INTS6	integrator complex subunit 6 [Source:HGNC Symbol;Acc:HGNC:14879]
ENSG00000103066	-0,2302622	5,56352462	2,27067775	0,16520551	0,4595487	PLA2G15	phospholipase A2 group XV [Source:HGNC Symbol;Acc:HGNC:17163]
ENSG00000141519	-0,3927598	0,99600263	2,27062656	0,16520979	0,4595487	CCDC40	coiled-coil domain containing 40 [Source:HGNC Symbol;Acc:HGNC:26090]
ENSG00000156298	0,25191854	4,53327711	2,27140634	0,16514457	0,4595487	TSPAN7	tetraspanin 7 [Source:HGNC Symbol;Acc:HGNC:11854]
ENSG00000165416	0,21355981	6,30166649	2,27317231	0,16499698	0,4595487	SUGT1	SGT1 homolog, MIS12 kinetochore complex assembly cochaperone [Source:HGNC Symbol;Acc:HGNC:16987]
ENSG00000196911	0,40239248	2,39932303	2,27170174	0,16511987	0,4595487	KPNA5	karyopherin subunit alpha 5 [Source:HGNC Symbol;Acc:HGNC:6398]
ENSG00000239246	-0,3561807	0,87057172	2,27071797	0,16520214	0,4595487	AC008026.1	ribosomal protein S10 (RPS10) pseudogene
ENSG00000116138	0,24865386	4,47833258	2,27010547	0,16525339	0,45958248	DNAJC16	DnaJ heat shock protein family (Hsp40) member C16 [Source:HGNC Symbol;Acc:HGNC:29157]
ENSG00000001460	0,33078313	1,36317224	2,26693556	0,16551897	0,45988334	STPG1	sperm tail PG-rich repeat containing 1 [Source:HGNC Symbol;Acc:HGNC:28070]
ENSG00000106462	-0,205614	5,339731	2,26748862	0,16547259	0,45988334	EZH2	enhancer of zeste 2 polycomb repressive complex 2 subunit [Source:HGNC Symbol;Acc:HGNC:3527]
ENSG00000132330	0,33901849	2,13545995	2,26756482	0,1654662	0,45988334	SCLY	selenocysteine lyase [Source:HGNC Symbol;Acc:HGNC:18161]
ENSG00000256628	-0,3985034	1,25218327	2,26711423	0,16550398	0,45988334	ZBTB11-AS1	ZBTB11 antisense RNA 1 [Source:HGNC Symbol;Acc:HGNC:48573]
ENSG00000258818	-0,3884293	1,79154339	2,26840577	0,16539572	0,45988334	RNASE4	ribonuclease A family member 4 [Source:HGNC Symbol;Acc:HGNC:10047]
ENSG00000180867	-0,3519274	3,23610833	2,26650024	0,16555548	0,45989733	PDIA3P1	protein disulfide isomerase family A member 3 pseudogene 1 [Source:HGNC Symbol;Acc:HGNC:4607]
ENSG00000169550	-0,4300354	0,67625315	2,26493463	0,1656869	0,46017488	MUC15	mucin 15, cell surface associated [Source:HGNC Symbol;Acc:HGNC:14956]
ENSG00000060971	0,21171843	8,02885986	2,25781417	0,1662863	0,46060135	ACAA1	acetyl-CoA acyltransferase 1 [Source:HGNC Symbol;Acc:HGNC:82]
ENSG00000067533	-0,2703912	5,08235492	2,25682528	0,16636977	0,46060135	RRP15	ribosomal RNA processing 15 homolog [Source:HGNC Symbol;Acc:HGNC:24255]
ENSG00000073614	0,25101501	4,20509058	2,26164584	0,16596339	0,46060135	KDM5A	lysine demethylase 5A [Source:HGNC Symbol;Acc:HGNC:9886]
ENSG00000085999	0,22912694	5,37717962	2,26077954	0,16603632	0,46060135	RAD54L	RAD54 like [Source:HGNC Symbol;Acc:HGNC:9826]
ENSG00000086504	-0,2193898	7,17745946	2,25674631	0,16637643	0,46060135	MRPL28	mitochondrial ribosomal protein L28 [Source:HGNC Symbol;Acc:HGNC:14484]
ENSG00000118412	0,26682458	2,4517944	2,25983676	0,16611574	0,46060135	CASP8AP2	caspase 8 associated protein 2 [Source:HGNC Symbol;Acc:HGNC:1510]
ENSG00000125731	0,33510917	1,13163219	2,25698443	0,16635633	0,46060135	SH2D3A	SH2 domain containing 3A [Source:HGNC Symbol;Acc:HGNC:16885]
ENSG00000131871	-0,2059788	6,61457217	2,25732353	0,1663277	0,46060135	SELENOS	selenoprotein S [Source:HGNC Symbol;Acc:HGNC:30396]
ENSG00000142864	-0,1950473	8,73073969	2,2569859	0,1663562	0,46060135	SERBP1	SERPINE1 mRNA binding protein 1 [Source:HGNC Symbol;Acc:HGNC:17860]
ENSG00000165891	-0,3103995	1,38997561	2,25734536	0,16632586	0,46060135	E2F7	E2F transcription factor 7 [Source:HGNC Symbol;Acc:HGNC:23820]
ENSG00000167840	-0,2826605	4,18784498	2,26235383	0,16590382	0,46060135	ZNF232	zinc finger protein 232 [Source:HGNC Symbol;Acc:HGNC:13026]
ENSG00000171223	-0,5531064	3,62415688	2,26019155	0,16608585	0,46060135	JUNB	JunB proto-oncogene, AP-1 transcription factor subunit [Source:HGNC Symbol;Acc:HGNC:6205]
ENSG00000182158	0,41856061	6,43448962	2,25694011	0,16636007	0,46060135	CREB3L2	cAMP responsive element binding protein 3 like 2 [Source:HGNC Symbol;Acc:HGNC:23720]
ENSG00000186871	0,28348928	2,59955369	2,26008791	0,16609458	0,46060135	ERCC6L	ERCC excision repair 6 like, spindle assembly checkpoint helicase [Source:HGNC Symbol;Acc:HGNC:20794]
ENSG00000198728	0,24164246	4,41085378	2,26124738	0,16599693	0,46060135	LDB1	LIM domain binding 1 [Source:HGNC Symbol;Acc:HGNC:6532]
ENSG00000205302	0,20092006	6,05273006	2,259186	0,16617059	0,46060135	SNX2	sorting nexin 2 [Source:HGNC Symbol;Acc:HGNC:11173]
ENSG00000229161	0,75465198	-1,2187116	2,2575557	0,16630811	0,46060135	TCP1P1	t-complex 1 pseudogene 1 [Source:HGNC Symbol;Acc:HGNC:11659]
ENSG00000139547	1,14229414	-1,881151	2,25560741	0,16647264	0,46078037	RDH16	retinol dehydrogenase 16 [Source:HGNC Symbol;Acc:HGNC:29674]
ENSG00000215256	-0,3028818	3,27793501	2,25516845	0,16650974	0,46079575	DHRS4-AS1	DHRS4 antisense RNA 1 [Source:HGNC Symbol;Acc:HGNC:23175]

ENSG00000254685	-0,2959091	1,93918209	2,25337104	0,16666176	0,46112911	FPGT	fucose-1-phosphate guanylyltransferase [Source:HGNC Symbol;Acc:HGNC:3825]
ENSG00000123473	0,23412225	3,38110042	2,25246063	0,16673883	0,46116767	STIL	STIL centriolar assembly protein [Source:HGNC Symbol;Acc:HGNC:10879]
ENSG00000140830	0,26688878	4,39810716	2,25270734	0,16671795	0,46116767	TXNL4B	thioredoxin like 4B [Source:HGNC Symbol;Acc:HGNC:26041]
ENSG00000158859	-0,7161177	0,81237535	2,25001606	0,16694602	0,46163957	ADAMTS4	ADAM metallopeptidase with thrombospondin type 1 motif 4 [Source:HGNC Symbol;Acc:HGNC:220]
ENSG00000236017	0,9220227	-1,9100781	2,24970203	0,16697265	0,46163957	ASMTL-AS1	ASMTL antisense RNA 1 [Source:HGNC Symbol;Acc:HGNC:25811]
ENSG00000082898	0,20405756	6,30416734	2,24901747	0,16703074	0,4617128	XPO1	exportin 1 [Source:HGNC Symbol;Acc:HGNC:12825]
ENSG00000225616	-0,3414866	1,10002607	2,24733113	0,16717396	0,46202125	AL138785.1	ribosomal protein L27 (RPL27) pseudogene
ENSG00000234608	-0,2376594	3,30181991	2,24667506	0,16722972	0,46208794	MAPKAPK5-AS1	MAPKAPK5 antisense RNA 1 [Source:HGNC Symbol;Acc:HGNC:24091]
ENSG00000009844	0,22911102	6,24987199	2,24609278	0,16727922	0,46213733	VTA1	vesicle trafficking 1 [Source:HGNC Symbol;Acc:HGNC:20954]
ENSG00000135473	0,25508458	3,18137542	2,24516113	0,16735848	0,4622411	PAN2	poly(A) specific ribonuclease subunit PAN2 [Source:HGNC Symbol;Acc:HGNC:20074]
ENSG00000164007	0,33711713	2,52091928	2,24476346	0,16739232	0,4622411	CLDN19	claudin 19 [Source:HGNC Symbol;Acc:HGNC:2040]
ENSG00000236493	-0,5739443	-0,7048454	2,24453573	0,16741171	0,4622411	EIF2S2P3	eukaryotic translation initiation factor 2 subunit 2 beta pseudogene 3 [Source:HGNC Symbol;Acc:HGNC:31664]
ENSG00000068745	-0,2197703	5,91497209	2,24275194	0,16756366	0,46231113	IP6K2	inositol hexakisphosphate kinase 2 [Source:HGNC Symbol;Acc:HGNC:17313]
ENSG00000072849	0,21202588	6,37423074	2,24317757	0,16752738	0,46231113	DERL2	derlin 2 [Source:HGNC Symbol;Acc:HGNC:17943]
ENSG00000134056	-0,2245922	5,70553757	2,24293311	0,16754822	0,46231113	MRPS36	mitochondrial ribosomal protein S36 [Source:HGNC Symbol;Acc:HGNC:16631]
ENSG00000164402	-0,2147954	4,97356564	2,24303584	0,16753946	0,46231113	SEPTIN8	septin 8 [Source:HGNC Symbol;Acc:HGNC:16511]
ENSG00000137502	0,33668259	1,5747325	2,24219432	0,16761119	0,46235497	RAB30	RAB30, member RAS oncogene family [Source:HGNC Symbol;Acc:HGNC:9770]
ENSG00000128564	-0,8945511	3,33095846	2,23959533	0,16783299	0,46261738	VGF	VGF nerve growth factor inducible [Source:HGNC Symbol;Acc:HGNC:12684]
ENSG00000130940	0,29945786	2,15754918	2,23983133	0,16781283	0,46261738	CASZ1	castor zinc finger 1 [Source:HGNC Symbol;Acc:HGNC:26002]
ENSG00000175470	-0,2185009	4,93364561	2,23972555	0,16782187	0,46261738	PPP2R2D	protein phosphatase 2 regulatory subunit Bdelta [Source:HGNC Symbol;Acc:HGNC:23732]
ENSG00000250182	-0,2338181	5,11786997	2,23992776	0,1678046	0,46261738	EEF1A1P13	eukaryotic translation elongation factor 1 alpha 1 pseudogene 13 [Source:HGNC Symbol;Acc:HGNC:3196]
ENSG00000249936	-0,3786694	0,80193984	2,23878501	0,16790222	0,46272091	RAC1P2	Rac family small GTPase 1 pseudogene 2 [Source:HGNC Symbol;Acc:HGNC:31414]
ENSG00000112531	0,22872546	5,26364705	2,23825985	0,16794711	0,46275732	QKI	QKI, KH domain containing RNA binding [Source:HGNC Symbol;Acc:HGNC:21100]
ENSG00000108518	-0,204358	10,0567839	2,23742109	0,16801883	0,46278038	PFN1	profilin 1 [Source:HGNC Symbol;Acc:HGNC:8881]
ENSG00000109475	-0,2215517	9,4192562	2,23758034	0,16800521	0,46278038	RPL34	ribosomal protein L34 [Source:HGNC Symbol;Acc:HGNC:10340]
ENSG00000084090	0,27758465	7,02129875	2,23506507	0,16822052	0,46316125	STARD7	StAR related lipid transfer domain containing 7 [Source:HGNC Symbol;Acc:HGNC:18063]
ENSG00000136830	-0,3165425	5,77006289	2,23535331	0,16819583	0,46316125	NIBAN2	niban apoptosis regulator 2 [Source:HGNC Symbol;Acc:HGNC:25282]
ENSG00000119616	0,28961684	3,66036951	2,23396425	0,16831487	0,46330941	FCF1	FCF1 rRNA-processing protein [Source:HGNC Symbol;Acc:HGNC:20220]
ENSG00000262246	-0,6015594	1,29845619	2,23369724	0,16833776	0,46330941	CORO7	coronin 7 [Source:HGNC Symbol;Acc:HGNC:26161]
ENSG00000230807	-0,6548344	-0,3602477	2,23327947	0,16837359	0,46332073	AC099535.1	ubiquitin pseudogene
ENSG00000109501	0,26918896	7,1711871	2,23102088	0,16856747	0,46333046	WFS1	wolframin ER transmembrane glycoprotein [Source:HGNC Symbol;Acc:HGNC:12762]
ENSG00000109572	0,2400536	4,16270565	2,23028328	0,16863085	0,46333046	CLCN3	chloride voltage-gated channel 3 [Source:HGNC Symbol;Acc:HGNC:2021]
ENSG00000114127	0,36426648	2,23019895	2,23125447	0,1685474	0,46333046	XRN1	5'-3' exoribonuclease 1 [Source:HGNC Symbol;Acc:HGNC:30654]
ENSG00000137700	-0,2373774	6,52312354	2,23087446	0,16858005	0,46333046	SLC37A4	solute carrier family 37 member 4 [Source:HGNC Symbol;Acc:HGNC:4061]
ENSG00000149929	-0,2325563	4,87019106	2,23150284	0,16852607	0,46333046	HIRIP3	HIRA interacting protein 3 [Source:HGNC Symbol;Acc:HGNC:4917]
ENSG00000228217	-0,8891605	-1,7922495	2,23145072	0,16853055	0,46333046	PNRC2P1	proline rich nuclear receptor coactivator 2 pseudogene 1 [Source:HGNC Symbol;Acc:HGNC:51933]
ENSG00000228477	-0,4495443	-0,0111478	2,23252748	0,16843811	0,46333046	AL663070.1	ribosomal protein S2 (RPS2) pseudogene
ENSG00000263731	0,62027559	1,02194725	2,23048538	0,16861348	0,46333046	AC145207.5	novel transcript
ENSG00000018699	0,25058939	5,55079128	2,2287398	0,16876357	0,46352966	TTC27	tetratricopeptide repeat domain 27 [Source:HGNC Symbol;Acc:HGNC:25986]
ENSG00000131061	0,35953282	1,45820303	2,22828293	0,16880289	0,46352966	ZNF341	zinc finger protein 341 [Source:HGNC Symbol;Acc:HGNC:15992]
ENSG00000138587	0,63773231	-0,7315769	2,22844448	0,16878898	0,46352966	MNS1	meiosis specific nuclear structural 1 [Source:HGNC Symbol;Acc:HGNC:29636]
ENSG00000158435	0,21088364	6,92591303	2,22796489	0,16883026	0,46352966	CNOT11	CCR4-NOT transcription complex subunit 11 [Source:HGNC Symbol;Acc:HGNC:25217]
ENSG00000096063	-0,2526948	6,42336451	2,22366294	0,16920112	0,46355263	SRPK1	SRSF protein kinase 1 [Source:HGNC Symbol;Acc:HGNC:11305]
ENSG00000135596	-0,364258	3,50420854	2,22667931	0,16894097	0,46355263	MICAL1	microtubule associated monooxygenase, calponin and LIM domain containing 1 [Source:HGNC Symbol;Acc:HGNC

ENSG00000138160	0,20740071	4,9904077	2,2244865	0,16913004	0,46355263	KIF11	kinesin family member 11 [Source:HGNC Symbol;Acc:HGNC:6388]
ENSG00000139737	-0,4766312	0,25427745	2,22345117	0,1692194	0,46355263	SLAIN1	SLAIN motif family member 1 [Source:HGNC Symbol;Acc:HGNC:26387]
ENSG00000152556	-0,4644394	1,0136204	2,22447782	0,16913079	0,46355263	PFKM	phosphofructokinase, muscle [Source:HGNC Symbol;Acc:HGNC:8877]
ENSG00000176386	-0,3071068	3,39435016	2,22708768	0,1689058	0,46355263	CDC26	cell division cycle 26 [Source:HGNC Symbol;Acc:HGNC:17839]
ENSG00000180035	-0,2082469	4,69710095	2,22389761	0,16918086	0,46355263	ZNF48	zinc finger protein 48 [Source:HGNC Symbol;Acc:HGNC:13114]
ENSG00000187961	-0,3546402	2,5191801	2,22431344	0,16914497	0,46355263	KLHL17	kelch like family member 17 [Source:HGNC Symbol;Acc:HGNC:24023]
ENSG00000188763	-0,4414911	1,43581123	2,22596186	0,1690028	0,46355263	FZD9	frizzled class receptor 9 [Source:HGNC Symbol;Acc:HGNC:4047]
ENSG00000188825	-0,3819083	0,78942781	2,22434931	0,16914187	0,46355263	LINC00910	long intergenic non-protein coding RNA 910 [Source:HGNC Symbol;Acc:HGNC:44361]
ENSG00000230734	-0,4004297	0,80751883	2,2271951	0,16889654	0,46355263	RPL10P3	ribosomal protein L10 pseudogene 3 [Source:HGNC Symbol;Acc:HGNC:23300]
ENSG00000236104	-0,270911	2,74685989	2,22456038	0,16912366	0,46355263	ZBTB22	zinc finger and BTB domain containing 22 [Source:HGNC Symbol;Acc:HGNC:13085]
ENSG00000177337	-0,417544	0,75711859	2,2220167	0,16934331	0,46380511	DLGAP1-AS1	DLGAP1 antisense RNA 1 [Source:HGNC Symbol;Acc:HGNC:31676]
ENSG00000196205	-0,2219841	6,21359443	2,22127358	0,16940756	0,46383446	EEF1A1P5	eukaryotic translation elongation factor 1 alpha 1 pseudogene 5 [Source:HGNC Symbol;Acc:HGNC:3200]
ENSG00000243725	0,33031758	1,22885503	2,22115826	0,16941753	0,46383446	TTC4	tetratricopeptide repeat domain 4 [Source:HGNC Symbol;Acc:HGNC:12394]
ENSG00000176390	0,29095758	3,99337767	2,2205218	0,16947258	0,46389824	CRLF3	cytokine receptor like factor 3 [Source:HGNC Symbol;Acc:HGNC:17177]
ENSG00000101210	-0,8886874	0,02667593	2,2185346	0,16964461	0,46398682	EEF1A2	eukaryotic translation elongation factor 1 alpha 2 [Source:HGNC Symbol;Acc:HGNC:3192]
ENSG00000152558	-0,2641732	8,43331167	2,21758082	0,16972726	0,46398682	TMEM123	transmembrane protein 123 [Source:HGNC Symbol;Acc:HGNC:30138]
ENSG00000157778	-0,2576198	5,22309092	2,21892475	0,16961082	0,46398682	PSMG3	proteasome assembly chaperone 3 [Source:HGNC Symbol;Acc:HGNC:22420]
ENSG00000183072	-0,7809084	-1,5958444	2,21915866	0,16959056	0,46398682	NKX2-5	NK2 homeobox 5 [Source:HGNC Symbol;Acc:HGNC:2488]
ENSG00000205181	-0,4134333	0,32187054	2,21821357	0,16967242	0,46398682	LINC00654	long intergenic non-protein coding RNA 654 [Source:HGNC Symbol;Acc:HGNC:27154]
ENSG00000228834	-0,5889193	-0,9967324	2,21873546	0,16962721	0,46398682	ATP5MFP2	ATP synthase membrane subunit f pseudogene 2 [Source:HGNC Symbol;Acc:HGNC:21540]
ENSG00000231948	-0,2903919	2,17713254	2,21784605	0,16970427	0,46398682	HS1BP3-IT1	HS1BP3 intronic transcript 1 [Source:HGNC Symbol;Acc:HGNC:41474]
ENSG00000142676	-0,2141157	10,6564388	2,21621114	0,16984605	0,46405823	RPL11	ribosomal protein L11 [Source:HGNC Symbol;Acc:HGNC:10301]
ENSG00000147255	0,28607105	6,82008395	2,21618078	0,16984868	0,46405823	IGSF1	immunoglobulin superfamily member 1 [Source:HGNC Symbol;Acc:HGNC:5948]
ENSG00000154710	0,55846367	-0,5606724	2,21619489	0,16984746	0,46405823	RABGEF1	RAB guanine nucleotide exchange factor 1 [Source:HGNC Symbol;Acc:HGNC:17676]
ENSG00000154548	0,33786366	1,29264466	2,21345799	0,17008514	0,46444371	SRSF12	serine and arginine rich splicing factor 12 [Source:HGNC Symbol;Acc:HGNC:21220]
ENSG00000198805	-0,2024452	6,79598263	2,21361235	0,17007172	0,46444371	PNP	purine nucleoside phosphorylase [Source:HGNC Symbol;Acc:HGNC:7892]
ENSG00000241058	-0,2615264	3,01213221	2,2138163	0,170054	0,46444371	NSUN6	NOP2/Sun RNA methyltransferase 6 [Source:HGNC Symbol;Acc:HGNC:23529]
ENSG00000040633	0,20595511	6,04235484	2,20706161	0,17064234	0,46501835	PHF23	PHD finger protein 23 [Source:HGNC Symbol;Acc:HGNC:28428]
ENSG00000117395	-0,2824594	7,1894948	2,2066283	0,17068018	0,46501835	EBNA1BP2	EBNA1 binding protein 2 [Source:HGNC Symbol;Acc:HGNC:15531]
ENSG00000136492	0,23691089	3,07459116	2,20825558	0,17053815	0,46501835	BRIP1	BRCA1 interacting protein C-terminal helicase 1 [Source:HGNC Symbol;Acc:HGNC:20473]
ENSG00000146476	0,2359486	4,69474953	2,20640373	0,17069979	0,46501835	ARMT1	acidic residue methyltransferase 1 [Source:HGNC Symbol;Acc:HGNC:17872]
ENSG00000151498	0,21677722	4,30901767	2,20793027	0,17056653	0,46501835	ACAD8	acyl-CoA dehydrogenase family member 8 [Source:HGNC Symbol;Acc:HGNC:87]
ENSG00000153179	0,22433906	3,86969198	2,2091386	0,17046115	0,46501835	RASSF3	Ras association domain family member 3 [Source:HGNC Symbol;Acc:HGNC:14271]
ENSG00000160194	-0,2003079	6,173974	2,20938006	0,1704401	0,46501835	NDUFV3	NADH:ubiquinone oxidoreductase subunit V3 [Source:HGNC Symbol;Acc:HGNC:7719]
ENSG00000169213	-0,3804251	2,28951624	2,20629388	0,17070938	0,46501835	RAB3B	RAB3B, member RAS oncogene family [Source:HGNC Symbol;Acc:HGNC:9778]
ENSG00000171126	0,36867397	1,05842848	2,21016743	0,17037149	0,46501835	KCNG3	potassium voltage-gated channel modifier subfamily G member 3 [Source:HGNC Symbol;Acc:HGNC:18306]
ENSG00000178078	-0,2128199	5,50305598	2,20971877	0,17041058	0,46501835	STAP2	signal transducing adaptor family member 2 [Source:HGNC Symbol;Acc:HGNC:30430]
ENSG00000237036	-0,3937434	1,228948	2,2084749	0,17051902	0,46501835	ZEB1-AS1	ZEB1 antisense RNA 1 [Source:HGNC Symbol;Acc:HGNC:42354]
ENSG00000254783	1,05012848	-1,4593795	2,20998411	0,17038746	0,46501835	AP003084.1	immunoglobin superfamily, member 21 (IGSF21) pseudogene
ENSG00000282508	0,88046773	-1,7154624	2,2069105	0,17065554	0,46501835	LINC01002	long intergenic non-protein coding RNA 1002 [Source:HGNC Symbol;Acc:HGNC:38538]
ENSG00000118518	0,24015285	2,92444106	2,20311987	0,17098692	0,46568752	RNF146	ring finger protein 146 [Source:HGNC Symbol;Acc:HGNC:21336]
ENSG00000164366	0,2023243	5,540924	2,20222481	0,17106529	0,46581412	CCDC127	coiled-coil domain containing 127 [Source:HGNC Symbol;Acc:HGNC:30520]
ENSG00000171824	0,27783189	6,03871835	2,20128164	0,17114792	0,4659088	EXOSC10	exosome component 10 [Source:HGNC Symbol;Acc:HGNC:9138]
ENSG00000227008	0,54274415	-0,1724615	2,20110001	0,17116384	0,4659088	AL009174.1	ribosomal protein S24 (RPS24) pseudogene

ENSG00000105518	-0,2500908	6,11410965	2,20024532	0,17123878	0,46593915	TMEM205	transmembrane protein 205 [Source:HGNC Symbol;Acc:HGNC:29631]
ENSG00000206344	0,85436315	-1,4800353	2,20033706	0,17123073	0,46593915	HCG27	HLA complex group 27 [Source:HGNC Symbol;Acc:HGNC:27366]
ENSG00000073792	-0,3289154	6,20226862	2,19952895	0,17130162	0,46602334	IGF2BP2	insulin like growth factor 2 mRNA binding protein 2 [Source:HGNC Symbol;Acc:HGNC:28867]
ENSG00000177192	-0,2157119	5,31504313	2,19440892	0,17175165	0,46716065	PUS1	pseudouridine synthase 1 [Source:HGNC Symbol;Acc:HGNC:15508]
ENSG00000116863	0,20168648	5,81391852	2,1914576	0,17201177	0,46760312	ADPRS	ADP-ribosylserine hydrolase [Source:HGNC Symbol;Acc:HGNC:21304]
ENSG00000147403	-0,2061369	11,0693399	2,19074828	0,17207436	0,46760312	RPL10	ribosomal protein L10 [Source:HGNC Symbol;Acc:HGNC:10298]
ENSG00000168014	0,27021623	3,16408202	2,191427	0,17201447	0,46760312	C2CD3	C2 domain containing 3 centriole elongation regulator [Source:HGNC Symbol;Acc:HGNC:24564]
ENSG00000269958	0,39091656	1,45396373	2,19094015	0,17205743	0,46760312	AL049840.5	novel transcript, sense intronic to KLC1
ENSG00000287093	-0,7279713	-1,2644635	2,19191925	0,17197105	0,46760312	AC003093.1	novel transcript
ENSG00000111530	-0,2382925	5,33010277	2,1892264	0,17220876	0,46769863	CAND1	cullin associated and neddylation dissociated 1 [Source:HGNC Symbol;Acc:HGNC:30688]
ENSG00000119986	-0,2761581	5,04198368	2,18890043	0,17223757	0,46769863	AVPI1	arginine vasopressin induced 1 [Source:HGNC Symbol;Acc:HGNC:30898]
ENSG00000204444	-0,238443	8,52625209	2,18982513	0,17215587	0,46769863	APOM	apolipoprotein M [Source:HGNC Symbol;Acc:HGNC:13916]
ENSG00000213958	-0,5981625	-0,64474	2,18890814	0,17223689	0,46769863	KRT18P29	keratin 18 pseudogene 29 [Source:HGNC Symbol;Acc:HGNC:33398]
ENSG00000072840	0,38035687	1,50573949	2,18748	0,17236316	0,46788925	EVC	EvC ciliary complex subunit 1 [Source:HGNC Symbol;Acc:HGNC:3497]
ENSG00000171105	0,31227027	4,81489327	2,1873821	0,17237182	0,46788925	INSR	insulin receptor [Source:HGNC Symbol;Acc:HGNC:6091]
ENSG00000109180	0,25863535	6,31076578	2,18647267	0,1724523	0,46802075	OCIAD1	OCIA domain containing 1 [Source:HGNC Symbol;Acc:HGNC:16074]
ENSG00000121274	0,22901835	3,94748765	2,1852881	0,17255721	0,4681526	TENT4B	terminal nucleotidyltransferase 4B [Source:HGNC Symbol;Acc:HGNC:30758]
ENSG00000214753	0,30925071	3,59127796	2,18520041	0,17256498	0,4681526	HNRNPUL2	heterogeneous nuclear ribonucleoprotein U like 2 [Source:HGNC Symbol;Acc:HGNC:25451]
ENSG00000186480	0,8820376	-1,7289336	2,18482883	0,1725979	0,46815498	INSIG1	insulin induced gene 1 [Source:HGNC Symbol;Acc:HGNC:6083]
ENSG00000122778	0,40714043	3,21866378	2,18322298	0,17274029	0,46845422	KIAA1549	KIAA1549 [Source:HGNC Symbol;Acc:HGNC:22219]
ENSG00000164117	-0,3239058	2,53184929	2,18278862	0,17277883	0,46847178	FBXO8	F-box protein 8 [Source:HGNC Symbol;Acc:HGNC:13587]
ENSG00000177239	0,20145577	7,19091668	2,18239111	0,17281411	0,46848049	MAN1B1	mannosidase alpha class 1B member 1 [Source:HGNC Symbol;Acc:HGNC:6823]
ENSG00000196453	-0,2386016	4,22002449	2,18181432	0,17286532	0,46853237	ZNF777	zinc finger protein 777 [Source:HGNC Symbol;Acc:HGNC:22213]
ENSG00000105792	0,42133702	0,41572109	2,17945002	0,17307545	0,46883962	CFAP69	cilia and flagella associated protein 69 [Source:HGNC Symbol;Acc:HGNC:26107]
ENSG00000168807	0,42543616	3,29557259	2,17937324	0,17308227	0,46883962	SNTB2	syntrophin beta 2 [Source:HGNC Symbol;Acc:HGNC:11169]
ENSG00000204389	-0,6290444	6,05437136	2,17909471	0,17310705	0,46883962	HSPA1A	heat shock protein family A (Hsp70) member 1A [Source:HGNC Symbol;Acc:HGNC:5232]
ENSG00000233766	-0,9216121	-1,7245366	2,18007435	0,17301993	0,46883962	CAVIN2-AS1	CAVIN2 and TMEFF2 antisense RNA 1 [Source:HGNC Symbol;Acc:HGNC:40517]
ENSG00000135968	0,33997498	3,97006872	2,17830067	0,17317771	0,46885716	GCC2	GRIP and coiled-coil domain containing 2 [Source:HGNC Symbol;Acc:HGNC:23218]
ENSG00000164292	0,22727053	4,24369422	2,17852136	0,17315807	0,46885716	RHOBTB3	Rho related BTB domain containing 3 [Source:HGNC Symbol;Acc:HGNC:18757]
ENSG00000196465	-0,313766	5,28780338	2,17758387	0,17324153	0,46894304	MYL6B	myosin light chain 6B [Source:HGNC Symbol;Acc:HGNC:29823]
ENSG00000035862	-0,232925	5,18156002	2,1767721	0,17331385	0,46905187	TIMP2	TIMP metallopeptidase inhibitor 2 [Source:HGNC Symbol;Acc:HGNC:11821]
ENSG00000134504	0,27515811	2,01953302	2,17580889	0,1733997	0,46919731	KCTD1	potassium channel tetramerization domain containing 1 [Source:HGNC Symbol;Acc:HGNC:18249]
ENSG00000156990	-0,2406694	5,55668986	2,17494427	0,17347682	0,46931905	RPUSD3	RNA pseudouridine synthase D3 [Source:HGNC Symbol;Acc:HGNC:28437]
ENSG00000131779	0,24229939	4,23214115	2,17409337	0,17355276	0,46943755	PEX11B	peroxisomal biogenesis factor 11 beta [Source:HGNC Symbol;Acc:HGNC:8853]
ENSG00000077380	-0,2653999	6,10711713	2,16993592	0,17392441	0,46974703	DYNC1I2	dynein cytoplasmic 1 intermediate chain 2 [Source:HGNC Symbol;Acc:HGNC:2964]
ENSG00000107331	-0,3901677	1,15944577	2,17164604	0,17377141	0,46974703	ABCA2	ATP binding cassette subfamily A member 2 [Source:HGNC Symbol;Acc:HGNC:32]
ENSG00000143436	0,20070542	6,60450313	2,17151751	0,1737829	0,46974703	MRPL9	mitochondrial ribosomal protein L9 [Source:HGNC Symbol;Acc:HGNC:14277]
ENSG00000162086	0,7369917	-1,3716655	2,17020103	0,17390068	0,46974703	ZNF75A	zinc finger protein 75a [Source:HGNC Symbol;Acc:HGNC:13146]
ENSG00000167182	0,23331575	3,21632195	2,17090888	0,17383734	0,46974703	SP2	Sp2 transcription factor [Source:HGNC Symbol;Acc:HGNC:11207]
ENSG00000169136	-0,294399	3,82993865	2,17239139	0,17370478	0,46974703	ATF5	activating transcription factor 5 [Source:HGNC Symbol;Acc:HGNC:790]
ENSG00000173137	-0,2913207	2,96799408	2,17056941	0,17386771	0,46974703	ADCK5	aarF domain containing kinase 5 [Source:HGNC Symbol;Acc:HGNC:21738]
ENSG00000256771	-0,2361614	3,27579209	2,17055517	0,17386898	0,46974703	ZNF253	zinc finger protein 253 [Source:HGNC Symbol;Acc:HGNC:13497]
ENSG00000143476	0,2169955	4,48891946	2,16933635	0,17397809	0,46980517	DTL	denticleless E3 ubiquitin protein ligase homolog [Source:HGNC Symbol;Acc:HGNC:30288]
ENSG00000106299	-0,2269567	3,51665056	2,16881483	0,17402481	0,46984085	WASL	WASP like actin nucleation promoting factor [Source:HGNC Symbol;Acc:HGNC:12735]

ENSG00000109787	0,21719343	5,48124624	2,16843002	0,17405929	0,46984085	KLF3	Kruppel like factor 3 [Source:HGNC Symbol;Acc:HGNC:16516]
ENSG00000162437	0,23139131	3,26657264	2,16811197	0,17408779	0,46984085	RAVER2	ribonucleoprotein, PTB binding 2 [Source:HGNC Symbol;Acc:HGNC:25577]
ENSG00000013364	-0,4248899	5,09124312	2,16533092	0,17433729	0,47020594	MVP	major vault protein [Source:HGNC Symbol;Acc:HGNC:7531]
ENSG00000046604	-0,3606538	6,43327717	2,16499469	0,17436749	0,47020594	DSG2	desmoglein 2 [Source:HGNC Symbol;Acc:HGNC:3049]
ENSG00000169902	-0,2450305	5,1916059	2,16481096	0,174384	0,47020594	TPST1	tyrosylprotein sulfotransferase 1 [Source:HGNC Symbol;Acc:HGNC:12020]
ENSG00000214900	-0,6759179	-0,9240071	2,16504143	0,17436329	0,47020594	LINC01588	long intergenic non-protein coding RNA 1588 [Source:HGNC Symbol;Acc:HGNC:27503]
ENSG00000271869	-0,762711	-1,5885654	2,16593085	0,17428343	0,47020594	AC026979.2	novel transcript
ENSG00000027697	0,27897571	6,56493934	2,16383949	0,17447129	0,47035452	IFNGR1	interferon gamma receptor 1 [Source:HGNC Symbol;Acc:HGNC:5439]
ENSG00000071462	-0,1904842	7,06631492	2,1627124	0,17457265	0,47039467	BUD23	BUD23 rRNA methyltransferase and ribosome maturation factor [Source:HGNC Symbol;Acc:HGNC:16405]
ENSG00000142089	-0,2118132	8,10439527	2,16314069	0,17453413	0,47039467	IFITM3	interferon induced transmembrane protein 3 [Source:HGNC Symbol;Acc:HGNC:5414]
ENSG00000185559	0,19682641	11,143956	2,16259976	0,17458278	0,47039467	DLK1	delta like non-canonical Notch ligand 1 [Source:HGNC Symbol;Acc:HGNC:2907]
ENSG00000178623	-0,2800411	4,1139622	2,16153456	0,17467865	0,47056618	GPR35	G protein-coupled receptor 35 [Source:HGNC Symbol;Acc:HGNC:4492]
ENSG00000279227	-0,3274648	1,97521422	2,15909268	0,17489868	0,47107207	AC009303.4	novel transcript
ENSG00000127399	-0,259137	5,22361518	2,15683877	0,1751021	0,47130149	LRRC61	leucine rich repeat containing 61 [Source:HGNC Symbol;Acc:HGNC:21704]
ENSG00000204177	-0,3998447	1,0299975	2,1572122	0,17506838	0,47130149	BMS1P1	BMS1 pseudogene 1 [Source:HGNC Symbol;Acc:HGNC:23649]
ENSG00000224597	0,30466058	3,33579778	2,15685949	0,17510023	0,47130149	SVIL-AS1	SVIL antisense RNA 1 [Source:HGNC Symbol;Acc:HGNC:51219]
ENSG00000273568	0,58222107	-0,6398532	2,15671915	0,17511291	0,47130149	AC131009.3	novel transcript, sense intronic to PUS1
ENSG00000165282	-0,2834037	5,23744282	2,15565193	0,17520934	0,4714335	PIGO	phosphatidylinositol glycan anchor biosynthesis class O [Source:HGNC Symbol;Acc:HGNC:23215]
ENSG00000288398	-0,773659	-1,7437262	2,15546219	0,1752265	0,4714335	AL109627.1	novel transcript
ENSG00000083642	0,28597444	3,65161459	2,15236618	0,17550669	0,47149269	PDS5B	PDS5 cohesin associated factor B [Source:HGNC Symbol;Acc:HGNC:20418]
ENSG00000091073	-0,236854	3,61994999	2,15383413	0,17537377	0,47149269	DTX2	deltex E3 ubiquitin ligase 2 [Source:HGNC Symbol;Acc:HGNC:15973]
ENSG00000100281	0,24729126	3,21820544	2,15334393	0,17541814	0,47149269	HMGXB4	HMG-box containing 4 [Source:HGNC Symbol;Acc:HGNC:5003]
ENSG00000130338	0,48484701	3,42262851	2,15268569	0,17547775	0,47149269	TULP4	TUB like protein 4 [Source:HGNC Symbol;Acc:HGNC:15530]
ENSG00000140265	0,21929691	4,04162701	2,15302476	0,17544704	0,47149269	ZSCAN29	zinc finger and SCAN domain containing 29 [Source:HGNC Symbol;Acc:HGNC:26673]
ENSG00000151849	0,22173504	3,58078703	2,1543414	0,17532786	0,47149269	CENPJ	centromere protein J [Source:HGNC Symbol;Acc:HGNC:17272]
ENSG00000165801	0,20637035	5,46173415	2,15369572	0,17538629	0,47149269	ARHGEF40	Rho guanine nucleotide exchange factor 40 [Source:HGNC Symbol;Acc:HGNC:25516]
ENSG00000185825	0,20124441	8,85365878	2,15441803	0,17532093	0,47149269	BCAP31	B cell receptor associated protein 31 [Source:HGNC Symbol;Acc:HGNC:16695]
ENSG00000187514	-0,2741913	8,72866787	2,15155803	0,17557993	0,47160271	PTMA	prothymosin alpha [Source:HGNC Symbol;Acc:HGNC:9623]
ENSG00000105701	-0,244047	7,07109975	2,15117709	0,17561446	0,47160877	FKBP8	FKBP prolyl isomerase 8 [Source:HGNC Symbol;Acc:HGNC:3724]
ENSG00000054965	-0,2401557	3,10334075	2,15040444	0,17568454	0,4716836	FAM168A	family with sequence similarity 168 member A [Source:HGNC Symbol;Acc:HGNC:28999]
ENSG00000110107	0,19527796	7,41272342	2,15005304	0,17571642	0,4716836	PRPF19	pre-mRNA processing factor 19 [Source:HGNC Symbol;Acc:HGNC:17896]
ENSG00000172478	0,33185178	2,62585225	2,14980213	0,17573919	0,4716836	MAB21L4	mab-21 like 4 [Source:HGNC Symbol;Acc:HGNC:26216]
ENSG00000197969	0,35611285	4,00593591	2,14903489	0,17580884	0,47175741	VPS13A	vacuolar protein sorting 13 homolog A [Source:HGNC Symbol;Acc:HGNC:1908]
ENSG00000244398	-0,2559469	2,82048137	2,14878785	0,17583127	0,47175741	AC116533.1	ribosomal protein L36a (RPL36A) pseudogene
ENSG00000088812	0,35142506	6,28893337	2,14830128	0,17587547	0,47178934	ATRN	attractin [Source:HGNC Symbol;Acc:HGNC:885]
ENSG00000171100	0,25529026	4,03831623	2,14757779	0,17594122	0,47187906	MTM1	myotubularin 1 [Source:HGNC Symbol;Acc:HGNC:7448]
ENSG00000134278	0,24910952	3,9358216	2,14680479	0,17601149	0,4719809	SPIRE1	spire type actin nucleation factor 1 [Source:HGNC Symbol;Acc:HGNC:30622]
ENSG00000246763	-0,509816	-0,3237004	2,14606667	0,17607864	0,47207429	RGMB-AS1	RGMB antisense RNA 1 [Source:HGNC Symbol;Acc:HGNC:48666]
ENSG00000165832	-0,2211452	4,06559025	2,14512224	0,1761646	0,47221809	TRUB1	TruB pseudouridine synthase family member 1 [Source:HGNC Symbol;Acc:HGNC:16060]
ENSG00000149968	-0,7040152	1,09165386	2,14472546	0,17620073	0,47222829	MMP3	matrix metallopeptidase 3 [Source:HGNC Symbol;Acc:HGNC:7173]
ENSG00000228043	-0,7026533	-1,2671186	2,14414545	0,17625356	0,47228324	AC114763.1	novel transcript
ENSG00000275765	-0,7453849	-1,3506185	2,14342674	0,17631905	0,4723721	AC091982.3	novel transcript
ENSG00000164329	0,23033853	4,21241537	2,14245834	0,17640735	0,47252201	TENT2	terminal nucleotidyltransferase 2 [Source:HGNC Symbol;Acc:HGNC:26776]
ENSG00000204351	-0,33691	5,82002611	2,14205445	0,1764442	0,47253406	SKIV2L	Ski2 like RNA helicase [Source:HGNC Symbol;Acc:HGNC:10898]

ENSG00000010256	0,2773275	9,06573933	2,14153814	0,17649131	0,47257361	UQCRC1	ubiquinol-cytochrome c reductase core protein 1 [Source:HGNC Symbol;Acc:HGNC:12585]
ENSG00000167461	0,19175205	5,99774446	2,14096956	0,17654322	0,47262596	RAB8A	RAB8A, member RAS oncogene family [Source:HGNC Symbol;Acc:HGNC:7007]
ENSG00000064012	0,30196236	4,81058463	2,13958401	0,17666979	0,47287814	CASP8	caspase 8 [Source:HGNC Symbol;Acc:HGNC:1509]
ENSG00000235957	-0,6827095	-0,6966964	2,13911573	0,17671259	0,47290606	COX7CP1	cytochrome c oxidase subunit 7C pseudogene 1 [Source:HGNC Symbol;Acc:HGNC:2293]
ENSG00000152133	-0,2329885	3,29345151	2,13838462	0,17677944	0,47299833	GPATCH11	G-patch domain containing 11 [Source:HGNC Symbol;Acc:HGNC:26768]
ENSG00000214975	-0,3359137	1,78594816	2,13784796	0,17682854	0,47302872	PPIAP29	peptidylprolyl isomerase A pseudogene 29 [Source:HGNC Symbol;Acc:HGNC:43021]
ENSG00000286714	-0,504895	0,4606589	2,1375527	0,17685556	0,47302872	AC093001.2	novel transcript
ENSG00000114021	-0,2070839	5,42120442	2,1360264	0,17699532	0,47331586	NIT2	nitrilase family member 2 [Source:HGNC Symbol;Acc:HGNC:29878]
ENSG00000186130	0,27769035	2,13400774	2,13536453	0,17705596	0,4733914	ZBTB6	zinc finger and BTB domain containing 6 [Source:HGNC Symbol;Acc:HGNC:16764]
ENSG00000141380	0,24046064	5,04664974	2,13429881	0,17715368	0,47356599	SS18	SS18 subunit of BAF chromatin remodeling complex [Source:HGNC Symbol;Acc:HGNC:11340]
ENSG00000047457	0,33904162	4,96784115	2,13372284	0,17720652	0,47362057	CP	ceruloplasmin [Source:HGNC Symbol;Acc:HGNC:2295]
ENSG00000072609	0,24370958	3,98820451	2,13114053	0,17744368	0,47373441	CHFR	checkpoint with forkhead and ring finger domains [Source:HGNC Symbol;Acc:HGNC:20455]
ENSG00000122390	0,25179388	5,43548004	2,13164007	0,17739777	0,47373441	NAA60	N-alpha-acetyltransferase 60, NatF catalytic subunit [Source:HGNC Symbol;Acc:HGNC:25875]
ENSG00000135679	0,22705458	5,82507865	2,13274404	0,17729636	0,47373441	MDM2	MDM2 proto-oncogene [Source:HGNC Symbol;Acc:HGNC:6973]
ENSG00000143727	-0,2287921	8,38375336	2,13121908	0,17743646	0,47373441	ACP1	acid phosphatase 1 [Source:HGNC Symbol;Acc:HGNC:122]
ENSG00000145390	0,31847977	1,76795506	2,13121724	0,17743663	0,47373441	USP53	ubiquitin specific peptidase 53 [Source:HGNC Symbol;Acc:HGNC:29255]
ENSG00000261713	-0,3920032	1,76337779	2,13179023	0,17738397	0,47373441	SSTR5-AS1	SSTR5 antisense RNA 1 [Source:HGNC Symbol;Acc:HGNC:26502]
ENSG00000213963	-0,7974905	-1,6548328	2,13060204	0,17749319	0,47378	AC019080.1	novel transcript
ENSG00000131910	0,21609017	7,83105909	2,12995008	0,17755315	0,47381997	NR0B2	nuclear receptor subfamily 0 group B member 2 [Source:HGNC Symbol;Acc:HGNC:7961]
ENSG00000271855	-0,4687407	-0,1715672	2,12973402	0,17757303	0,47381997	AC073195.1	novel transcript
ENSG00000188501	-0,744776	-0,0672034	2,12923218	0,17761921	0,47385665	LCTL	lactase like [Source:HGNC Symbol;Acc:HGNC:15583]
ENSG00000081386	0,30993254	2,47147602	2,12860121	0,1776773	0,47392262	ZNF510	zinc finger protein 510 [Source:HGNC Symbol;Acc:HGNC:29161]
ENSG00000101347	0,26273793	5,28450533	2,12755465	0,1777737	0,47392262	SAMHD1	SAM and HD domain containing deoxynucleoside triphosphate triphosphohydrolase 1 [Source:HGNC Symbol;Acc:
ENSG00000167646	1,03298919	-1,8413315	2,12779841	0,17775124	0,47392262	DNAAF3	dynein axonemal assembly factor 3 [Source:HGNC Symbol;Acc:HGNC:30492]
ENSG00000171174	-0,3034641	3,5708868	2,12802591	0,17773028	0,47392262	RBKS	ribokinase [Source:HGNC Symbol;Acc:HGNC:30325]
ENSG00000053770	-0,2887998	3,00077997	2,12690343	0,17783372	0,47393022	AP5M1	adaptor related protein complex 5 subunit mu 1 [Source:HGNC Symbol;Acc:HGNC:20192]
ENSG00000174307	-0,2859185	5,16250012	2,12681978	0,17784143	0,47393022	PHLDA3	pleckstrin homology like domain family A member 3 [Source:HGNC Symbol;Acc:HGNC:8934]
ENSG00000140612	0,21182959	8,47790307	2,12534327	0,17797763	0,47420666	SEC11A	SEC11 homolog A, signal peptidase complex subunit [Source:HGNC Symbol;Acc:HGNC:17718]
ENSG00000185627	-0,1964177	7,21860217	2,12444126	0,1780609	0,47434202	PSMD13	proteasome 26S subunit, non-ATPase 13 [Source:HGNC Symbol;Acc:HGNC:9558]
ENSG00000179134	0,25411831	4,1802318	2,12050248	0,17842511	0,4752256	SAMD4B	sterile alpha motif domain containing 4B [Source:HGNC Symbol;Acc:HGNC:25492]
ENSG00000127564	-0,2145835	5,33432371	2,11877529	0,17858513	0,4753918	PKMYT1	protein kinase, membrane associated tyrosine/threonine 1 [Source:HGNC Symbol;Acc:HGNC:29650]
ENSG00000187583	-1,0077015	-0,7121204	2,11889496	0,17857404	0,4753918	PLEKHN1	pleckstrin homology domain containing N1 [Source:HGNC Symbol;Acc:HGNC:25284]
ENSG00000231083	-0,7594007	-1,3886272	2,11881653	0,17858131	0,4753918	AC011747.1	novel transcript
ENSG00000239789	-0,2635841	2,50730565	2,11742328	0,17871053	0,47563893	MRPS17	mitochondrial ribosomal protein S17 [Source:HGNC Symbol;Acc:HGNC:14047]
ENSG00000163697	0,23897717	3,6793668	2,11554527	0,1788849	0,47592961	APBB2	amyloid beta precursor protein binding family B member 2 [Source:HGNC Symbol;Acc:HGNC:582]
ENSG00000197557	0,37710042	0,65355638	2,11560101	0,17887972	0,47592961	TTC30A	tetratricopeptide repeat domain 30A [Source:HGNC Symbol;Acc:HGNC:25853]
ENSG00000198948	0,58822491	-0,0414647	2,11469067	0,17896432	0,47605422	MFAP3L	microfibril associated protein 3 like [Source:HGNC Symbol;Acc:HGNC:29083]
ENSG00000213585	0,20842605	8,54299539	2,11416544	0,17901316	0,47609744	VDAC1	voltage dependent anion channel 1 [Source:HGNC Symbol;Acc:HGNC:12669]
ENSG00000172771	-0,6076795	0,01786894	2,11375552	0,17905129	0,47611216	EFCAB12	EF-hand calcium binding domain 12 [Source:HGNC Symbol;Acc:HGNC:28061]
ENSG00000104870	-0,2180933	8,01847056	2,11301043	0,17912061	0,47614574	FCGRT	Fc fragment of IgG receptor and transporter [Source:HGNC Symbol;Acc:HGNC:3621]
ENSG00000116678	0,74457739	-1,0979751	2,11291928	0,1791291	0,47614574	LEPR	leptin receptor [Source:HGNC Symbol;Acc:HGNC:6554]
ENSG00000186532	0,22861218	3,77000018	2,11251915	0,17916635	0,47615811	SMYD4	SET and MYND domain containing 4 [Source:HGNC Symbol;Acc:HGNC:21067]
ENSG00000234171	-0,289301	3,32524829	2,10925783	0,17947033	0,47687923	RNASEH1-AS1	RNASEH1 antisense RNA 1 [Source:HGNC Symbol;Acc:HGNC:49289]
ENSG00000079974	-0,3042211	2,18827879	2,10713878	0,1796682	0,4769879	RABL2B	RAB, member of RAS oncogene family like 2B [Source:HGNC Symbol;Acc:HGNC:9800]

ENSG00000124003	-0,5245321	0,08201607	2,10777095	0,17960914	0,4769879	MOGAT1	monoacylglycerol O-acyltransferase 1 [Source:HGNC Symbol;Acc:HGNC:18210]
ENSG00000137331	-0,3963985	7,51521538	2,10532583	0,17983773	0,4769879	IER3	immediate early response 3 [Source:HGNC Symbol;Acc:HGNC:5392]
ENSG00000147364	0,23182233	5,04555174	2,10726451	0,17965646	0,4769879	FBXO25	F-box protein 25 [Source:HGNC Symbol;Acc:HGNC:13596]
ENSG00000162627	0,27017981	5,19254742	2,10599534	0,1797751	0,4769879	SNX7	sorting nexin 7 [Source:HGNC Symbol;Acc:HGNC:14971]
ENSG00000166130	-0,2036353	6,00060468	2,10606358	0,17976872	0,4769879	IKBIP	IKBKB interacting protein [Source:HGNC Symbol;Acc:HGNC:26430]
ENSG00000183048	-0,2499121	4,42316304	2,1081481	0,17957392	0,4769879	SLC25A10	solute carrier family 25 member 10 [Source:HGNC Symbol;Acc:HGNC:10980]
ENSG00000187867	0,45286108	1,53938946	2,10557722	0,17981421	0,4769879	PALM3	paralemmin 3 [Source:HGNC Symbol;Acc:HGNC:33274]
ENSG00000224875	0,6481267	-0,8986443	2,10586652	0,17978715	0,4769879	EML4-AS1	EML4 antisense RNA 1 [Source:HGNC Symbol;Acc:HGNC:40112]
ENSG00000250337	-0,3189534	1,69606048	2,10567219	0,17980532	0,4769879	PURPL	p53 upregulated regulator of p53 levels [Source:HGNC Symbol;Acc:HGNC:48995]
ENSG00000198886	0,23118925	12,360993	2,1046298	0,17990287	0,47707406	MT-ND4	mitochondrially encoded NADH:ubiquinone oxidoreductase core subunit 4 [Source:HGNC Symbol;Acc:HGNC:7459
ENSG00000138767	0,27068035	3,38304696	2,10394034	0,17996742	0,47715863	CNOT6L	CCR4-NOT transcription complex subunit 6 like [Source:HGNC Symbol;Acc:HGNC:18042]
ENSG00000079335	-0,4309097	0,70155928	2,10318671	0,18003802	0,47717262	CDC14A	cell division cycle 14A [Source:HGNC Symbol;Acc:HGNC:1718]
ENSG00000144580	0,20014501	6,44123356	2,10344427	0,18001389	0,47717262	CNOT9	CCR4-NOT transcription complex subunit 9 [Source:HGNC Symbol;Acc:HGNC:10445]
ENSG00000117298	0,38181708	5,80971874	2,10260218	0,18009281	0,47721292	ECE1	endothelin converting enzyme 1 [Source:HGNC Symbol;Acc:HGNC:3146]
ENSG00000140093	-0,2138839	5,74175898	2,10232747	0,18011856	0,47721292	SERPINA10	serpin family A member 10 [Source:HGNC Symbol;Acc:HGNC:15996]
ENSG00000175548	0,30026301	1,65776431	2,10149657	0,18019649	0,47733281	ALG10B	ALG10 alpha-1,2-glucosyltransferase B [Source:HGNC Symbol;Acc:HGNC:31088]
ENSG00000182952	0,27125886	4,78363369	2,10057678	0,1802828	0,47747488	HMGN4	high mobility group nucleosomal binding domain 4 [Source:HGNC Symbol;Acc:HGNC:4989]
ENSG00000170412	0,23439603	7,9540582	2,09977172	0,18035839	0,4775885	GPRC5C	G protein-coupled receptor class C group 5 member C [Source:HGNC Symbol;Acc:HGNC:13309]
ENSG00000147536	-0,2295107	3,45869835	2,09938707	0,18039453	0,47759761	GINS4	GINS complex subunit 4 [Source:HGNC Symbol;Acc:HGNC:28226]
ENSG00000148965	-0,3470587	1,2783938	2,09690663	0,18062776	0,47812846	SAA4	serum amyloid A4, constitutive [Source:HGNC Symbol;Acc:HGNC:10516]
ENSG00000110344	0,30134958	4,79944509	2,0953427	0,18077502	0,47843158	UBE4A	ubiquitination factor E4A [Source:HGNC Symbol;Acc:HGNC:12499]
ENSG00000198833	-0,243993	5,56497444	2,09419976	0,18088274	0,47854328	UBE2J1	ubiquitin conjugating enzyme E2 J1 [Source:HGNC Symbol;Acc:HGNC:17598]
ENSG00000206418	-0,2270831	3,38008253	2,0943834	0,18086543	0,47854328	RAB12	RAB12, member RAS oncogene family [Source:HGNC Symbol;Acc:HGNC:31332]
ENSG00000058056	-0,2527299	3,63609717	2,09293415	0,18100212	0,47874294	USP13	ubiquitin specific peptidase 13 [Source:HGNC Symbol;Acc:HGNC:12611]
ENSG00000224418	-0,702079	-1,3356981	2,09270496	0,18102375	0,47874294	STK24-AS1	STK24 antisense RNA 1 [Source:HGNC Symbol;Acc:HGNC:39935]
ENSG00000109323	0,31494262	5,72151268	2,09102257	0,18118264	0,47879842	MANBA	mannosidase beta [Source:HGNC Symbol;Acc:HGNC:6831]
ENSG00000151233	0,25411083	4,52181964	2,0912266	0,18116336	0,47879842	GXYLT1	glucoside xylosyltransferase 1 [Source:HGNC Symbol;Acc:HGNC:27482]
ENSG00000174516	-0,3388079	1,73063276	2,09086796	0,18119725	0,47879842	PELI3	pellino E3 ubiquitin protein ligase family member 3 [Source:HGNC Symbol;Acc:HGNC:30010]
ENSG00000188542	-0,4411304	0,9036768	2,0907478	0,1812086	0,47879842	DUSP28	dual specificity phosphatase 28 [Source:HGNC Symbol;Acc:HGNC:33237]
ENSG00000248626	-0,2933972	2,18787828	2,09126599	0,18115964	0,47879842	GAPDHP40	glyceraldehyde 3 phosphate dehydrogenase pseudogene 40 [Source:HGNC Symbol;Acc:HGNC:37797]
ENSG00000130005	-0,2505228	7,32689192	2,08990932	0,18128787	0,47884845	GAMT	guanidinoacetate N-methyltransferase [Source:HGNC Symbol;Acc:HGNC:4136]
ENSG00000184557	-0,6014294	-0,0820477	2,0898541	0,18129309	0,47884845	SOCS3	suppressor of cytokine signaling 3 [Source:HGNC Symbol;Acc:HGNC:19391]
ENSG00000018189	-0,2511436	2,92360371	2,0878703	0,18148083	0,47903139	RUFY3	RUN and FYVE domain containing 3 [Source:HGNC Symbol;Acc:HGNC:30285]
ENSG00000067646	0,38700737	1,86892985	2,08835989	0,18143447	0,47903139	ZFY	zinc finger protein Y-linked [Source:HGNC Symbol;Acc:HGNC:12870]
ENSG00000069275	-0,2824987	6,23796411	2,08700329	0,18156296	0,47903139	NUCKS1	nuclear casein kinase and cyclin dependent kinase substrate 1 [Source:HGNC Symbol;Acc:HGNC:29923]
ENSG00000080802	0,26407381	2,58978009	2,08669806	0,18159188	0,47903139	CNOT4	CCR4-NOT transcription complex subunit 4 [Source:HGNC Symbol;Acc:HGNC:7880]
ENSG00000166170	-0,2122092	4,59311034	2,08686408	0,18157615	0,47903139	BAG5	BAG cochaperone 5 [Source:HGNC Symbol;Acc:HGNC:941]
ENSG00000213533	0,34809195	1,97389864	2,08700611	0,18156269	0,47903139	STIMATE	STIM activating enhancer [Source:HGNC Symbol;Acc:HGNC:30526]
ENSG00000237729	-0,375755	0,6015101	2,08823313	0,18144647	0,47903139	AC002075.2	ribosomal protein S27 (RPS27) pseudogene
ENSG00000171109	0,2685608	4,77970535	2,08630045	0,18162957	0,47904431	MFN1	mitofusin 1 [Source:HGNC Symbol;Acc:HGNC:18262]
ENSG00000185917	0,25059947	3,83623579	2,08494306	0,18175832	0,47929734	SETD4	SET domain containing 4 [Source:HGNC Symbol;Acc:HGNC:1258]
ENSG00000163110	-0,4698183	6,11369743	2,08381414	0,18186548	0,47949338	PDLIM5	PDZ and LIM domain 5 [Source:HGNC Symbol;Acc:HGNC:17468]
ENSG00000168675	-0,3767117	0,67762268	2,0834379	0,18190122	0,47950106	LDLRAD4	low density lipoprotein receptor class A domain containing 4 [Source:HGNC Symbol;Acc:HGNC:1224]
ENSG00000100991	0,29227221	7,78541043	2,08211313	0,18202711	0,4796211	TRPC4AP	transient receptor potential cation channel subfamily C member 4 associated protein [Source:HGNC Symbol;Acc:H

ENSG00000182156	-0,3484997	1,4109423	2,08260223	0,18198062	0,4796211	ENPP7	ectonucleotide pyrophosphatase/phosphodiesterase 7 [Source:HGNC Symbol;Acc:HGNC:23764]
ENSG00000260765	-0,5776409	-0,3869114	2,08192242	0,18204525	0,4796211	CES1P2	carboxylesterase 1 pseudogene 2 [Source:HGNC Symbol;Acc:HGNC:48928]
ENSG00000023330	0,2180605	4,97613704	2,08112983	0,18212064	0,47963681	ALAS1	5'-aminolevulinate synthase 1 [Source:HGNC Symbol;Acc:HGNC:396]
ENSG00000148331	0,2043217	4,66976879	2,08042919	0,18218731	0,47963681	ASB6	ankyrin repeat and SOCS box containing 6 [Source:HGNC Symbol;Acc:HGNC:17181]
ENSG00000184903	-0,2353103	3,57162775	2,08049103	0,18218143	0,47963681	IMMP2L	inner mitochondrial membrane peptidase subunit 2 [Source:HGNC Symbol;Acc:HGNC:14598]
ENSG00000236762	-0,3722745	1,36651492	2,08013448	0,18221537	0,47963681	RPL19P16	ribosomal protein L19 pseudogene 16 [Source:HGNC Symbol;Acc:HGNC:36596]
ENSG00000240776	-0,3035363	1,21945093	2,08114028	0,18211964	0,47963681	AC079944.1	ribosomal protein S10 (RPS10) pseudogene
ENSG00000186314	-0,4275372	1,61942118	2,07952856	0,18227307	0,47970226	PRELID2	PRELI domain containing 2 [Source:HGNC Symbol;Acc:HGNC:28306]
ENSG00000230580	-0,3939293	0,27254322	2,07855705	0,18236563	0,47985943	RPL19P5	ribosomal protein L19 pseudogene 5 [Source:HGNC Symbol;Acc:HGNC:36325]
ENSG00000168306	-0,238067	5,91469633	2,07809277	0,18240989	0,47988945	ACOX2	acyl-CoA oxidase 2 [Source:HGNC Symbol;Acc:HGNC:120]
ENSG00000204580	0,23220951	5,93139748	2,07693157	0,18252065	0,48006182	DDR1	discoidin domain receptor tyrosine kinase 1 [Source:HGNC Symbol;Acc:HGNC:2730]
ENSG00000240771	0,31013066	1,91582747	2,07671691	0,18254113	0,48006182	ARHGEF25	Rho guanine nucleotide exchange factor 25 [Source:HGNC Symbol;Acc:HGNC:30275]
ENSG00000143632	0,28636142	4,34619108	2,074762	0,18272782	0,48037984	ACTA1	actin alpha 1, skeletal muscle [Source:HGNC Symbol;Acc:HGNC:129]
ENSG00000169696	-0,2033218	7,79008008	2,0750556	0,18269977	0,48037984	ASPSCR1	ASPSCR1 tether for SLC2A4, UBX domain containing [Source:HGNC Symbol;Acc:HGNC:13825]
ENSG00000135048	0,3852978	7,22277666	2,07329547	0,18286804	0,48048906	CEMIP2	cell migration inducing hyaluronidase 2 [Source:HGNC Symbol;Acc:HGNC:11869]
ENSG00000156587	0,3028427	5,091816	2,07380832	0,18281899	0,48048906	UBE2L6	ubiquitin conjugating enzyme E2 L6 [Source:HGNC Symbol;Acc:HGNC:12490]
ENSG00000183150	-0,7690253	-1,6196068	2,07339596	0,18285843	0,48048906	GPR19	G protein-coupled receptor 19 [Source:HGNC Symbol;Acc:HGNC:4473]
ENSG00000163931	0,21118753	9,91910405	2,07273842	0,18292134	0,48054267	TKT	transketolase [Source:HGNC Symbol;Acc:HGNC:11834]
ENSG00000102181	-0,2702594	3,87282682	2,07119667	0,18306896	0,48067117	CD99L2	CD99 molecule like 2 [Source:HGNC Symbol;Acc:HGNC:18237]
ENSG00000109083	-0,2859366	2,80555022	2,07180026	0,18301115	0,48067117	IFT20	intraflagellar transport 20 [Source:HGNC Symbol;Acc:HGNC:30989]
ENSG00000275066	0,26090118	4,01370012	2,07138797	0,18305063	0,48067117	SYNRG	synergin gamma [Source:HGNC Symbol;Acc:HGNC:557]
ENSG00000203883	-0,2391312	3,01697231	2,06771701	0,18340271	0,48146095	SOX18	SRY-box transcription factor 18 [Source:HGNC Symbol;Acc:HGNC:11194]
ENSG00000100364	-0,200051	6,07159298	2,06423622	0,18373738	0,48178446	KIAA0930	KIAA0930 [Source:HGNC Symbol;Acc:HGNC:1314]
ENSG00000103742	-0,7317136	-1,5829015	2,06389121	0,18377059	0,48178446	IGDCC4	immunoglobulin superfamily DCC subclass member 4 [Source:HGNC Symbol;Acc:HGNC:13770]
ENSG00000111790	0,26329645	4,44887038	2,06598674	0,18356897	0,48178446	FGFR1OP2	FGFR1 oncogene partner 2 [Source:HGNC Symbol;Acc:HGNC:23098]
ENSG00000113194	0,2675352	5,52786582	2,06396777	0,18376322	0,48178446	FAF2	Fas associated factor family member 2 [Source:HGNC Symbol;Acc:HGNC:24666]
ENSG00000115808	0,33602897	2,96186217	2,06474985	0,18368794	0,48178446	STRN	striatin [Source:HGNC Symbol;Acc:HGNC:11424]
ENSG00000116906	0,20695608	6,64389053	2,06304265	0,18385232	0,48178446	GNPAT	glyceronephosphate O-acyltransferase [Source:HGNC Symbol;Acc:HGNC:4416]
ENSG00000117280	0,28093388	3,08353193	2,06390611	0,18376916	0,48178446	RAB29	RAB29, member RAS oncogene family [Source:HGNC Symbol;Acc:HGNC:9789]
ENSG00000129071	0,26243903	4,57248746	2,06384189	0,18377534	0,48178446	MBD4	methyl-CpG binding domain 4, DNA glycosylase [Source:HGNC Symbol;Acc:HGNC:6919]
ENSG00000260526	-0,6497931	-1,4410877	2,06300727	0,18385573	0,48178446	AC109347.1	novel transcript, antisense to AP1AR
ENSG00000274849	0,62659721	-0,7911253	2,06309908	0,18384689	0,48178446	AC023043.4	novel transcript, sense intronic to MOCOS
ENSG00000141696	-0,2480693	6,61238179	2,06164035	0,18398749	0,48204327	P3H4	prolyl 3-hydroxylase family member 4 (inactive) [Source:HGNC Symbol;Acc:HGNC:16946]
ENSG00000033627	0,31583643	5,39625645	2,0610029	0,18404898	0,48209136	ATP6V0A1	ATPase H+ transporting V0 subunit a1 [Source:HGNC Symbol;Acc:HGNC:865]
ENSG00000115255	-0,2388262	5,86998049	2,06042398	0,18410485	0,48209136	REEP6	receptor accessory protein 6 [Source:HGNC Symbol;Acc:HGNC:30078]
ENSG00000159348	-0,200873	5,24869485	2,06062488	0,18408546	0,48209136	CYB5R1	cytochrome b5 reductase 1 [Source:HGNC Symbol;Acc:HGNC:13397]
ENSG00000158941	-0,2679222	6,6318453	2,059566	0,18418768	0,48222184	CCAR2	cell cycle and apoptosis regulator 2 [Source:HGNC Symbol;Acc:HGNC:23360]
ENSG00000101557	0,20832633	6,0315392	2,05891852	0,18425023	0,48229915	USP14	ubiquitin specific peptidase 14 [Source:HGNC Symbol;Acc:HGNC:12612]
ENSG00000007520	-0,2052289	5,78019492	2,05775832	0,18436237	0,48244117	TSR3	TSR3 ribosome maturation factor [Source:HGNC Symbol;Acc:HGNC:14175]
ENSG00000144867	0,18862536	7,7670225	2,05733252	0,18440355	0,48244117	SRPRB	SRP receptor subunit beta [Source:HGNC Symbol;Acc:HGNC:24085]
ENSG00000158195	0,25772701	4,25877164	2,05748045	0,18438924	0,48244117	WASF2	WASP family member 2 [Source:HGNC Symbol;Acc:HGNC:12733]
ENSG00000008086	0,41869599	0,90068368	2,05654965	0,1844793	0,48255292	CDKL5	cyclin dependent kinase like 5 [Source:HGNC Symbol;Acc:HGNC:11411]
ENSG00000269439	0,64342589	-0,602115	2,0561113	0,18452173	0,4825775	AC010618.3	novel transcript
ENSG00000123552	-0,2665441	2,20132997	2,05434263	0,18469306	0,48287105	USP45	ubiquitin specific peptidase 45 [Source:HGNC Symbol;Acc:HGNC:20080]

ENSG00000130522	-0,2159372	5,5457833	2,05427023	0,18470008	0,48287105	JUND	JunD proto-oncogene, AP-1 transcription factor subunit [Source:HGNC Symbol;Acc:HGNC:6206]
ENSG00000197296	0,23572154	5,43928807	2,05346228	0,18477842	0,48298942	FITM2	fat storage inducing transmembrane protein 2 [Source:HGNC Symbol;Acc:HGNC:16135]
ENSG00000069535	-0,2225191	4,78982042	2,05288257	0,18483465	0,48304999	MAOB	monoamine oxidase B [Source:HGNC Symbol;Acc:HGNC:6834]
ENSG00000128829	0,20160324	5,8930085	2,05133012	0,18498536	0,48306365	EIF2AK4	eukaryotic translation initiation factor 2 alpha kinase 4 [Source:HGNC Symbol;Acc:HGNC:19687]
ENSG00000131149	0,41336001	5,15222354	2,05120281	0,18499773	0,48306365	GSE1	Gse1 coiled-coil protein [Source:HGNC Symbol;Acc:HGNC:28979]
ENSG00000164187	-0,4291511	0,08863634	2,05100927	0,18501653	0,48306365	LMBRD2	LMBR1 domain containing 2 [Source:HGNC Symbol;Acc:HGNC:25287]
ENSG00000203778	-0,2662639	2,25453494	2,05078549	0,18503828	0,48306365	FAM229B	family with sequence similarity 229 member B [Source:HGNC Symbol;Acc:HGNC:33858]
ENSG00000239521	-0,4027097	2,429452	2,05248468	0,18487326	0,48306365	CASTOR3	CASTOR family member 3 [Source:HGNC Symbol;Acc:HGNC:29954]
ENSG00000277998	0,69805122	-1,2555816	2,05108724	0,18500896	0,48306365	AC107075.1	eukaryotic translation initiation factor 3, subunit E (EIF3E) pseudogene
ENSG00000130349	-0,2305577	3,01217735	2,05025263	0,18509006	0,48311251	MTRES1	mitochondrial transcription rescue factor 1 [Source:HGNC Symbol;Acc:HGNC:17971]
ENSG00000073921	0,25544257	6,12912013	2,0498858	0,18512572	0,48311928	PICALM	phosphatidylinositol binding clathrin assembly protein [Source:HGNC Symbol;Acc:HGNC:15514]
ENSG00000059691	0,2741789	6,89044957	2,04697884	0,18540866	0,48319571	GATB	glutamyl-tRNA amidotransferase subunit B [Source:HGNC Symbol;Acc:HGNC:8849]
ENSG00000103510	-0,1955811	5,71209138	2,04724514	0,18538271	0,48319571	KAT8	lysine acetyltransferase 8 [Source:HGNC Symbol;Acc:HGNC:17933]
ENSG00000115368	-0,2612031	5,57369216	2,04732779	0,18537466	0,48319571	WDR75	WD repeat domain 75 [Source:HGNC Symbol;Acc:HGNC:25725]
ENSG00000157110	-0,2058115	6,19997347	2,04754575	0,18535344	0,48319571	RBPMS	RNA binding protein, mRNA processing factor [Source:HGNC Symbol;Acc:HGNC:19097]
ENSG00000162994	0,3306176	1,12457698	2,04607111	0,18549712	0,48319571	CLHC1	clathrin heavy chain linker domain containing 1 [Source:HGNC Symbol;Acc:HGNC:26453]
ENSG00000169122	-0,3444066	1,67118351	2,04709853	0,185397	0,48319571	FAM110B	family with sequence similarity 110 member B [Source:HGNC Symbol;Acc:HGNC:28587]
ENSG00000171608	-0,2750129	4,83092086	2,0461842	0,1854861	0,48319571	PIK3CD	phosphatidylinositol-4,5-bisphosphate 3-kinase catalytic subunit delta [Source:HGNC Symbol;Acc:HGNC:8977]
ENSG00000173013	-0,6320328	-0,8726055	2,04671549	0,18543432	0,48319571	CCDC96	coiled-coil domain containing 96 [Source:HGNC Symbol;Acc:HGNC:26900]
ENSG00000173039	-0,1943921	6,08989066	2,04564041	0,18553912	0,48319571	RELA	RELA proto-oncogene, NF-kB subunit [Source:HGNC Symbol;Acc:HGNC:9955]
ENSG00000242358	-0,4332343	-0,0056326	2,04550918	0,18555192	0,48319571	RPS21P4	ribosomal protein S21 pseudogene 4 [Source:HGNC Symbol;Acc:HGNC:35922]
ENSG00000242372	-0,1888491	8,39145409	2,04654032	0,18545139	0,48319571	EIF6	eukaryotic translation initiation factor 6 [Source:HGNC Symbol;Acc:HGNC:6159]
ENSG00000269313	-0,5579242	-0,5887196	2,04761552	0,18534664	0,48319571	MAGIX	MAGI family member, X-linked [Source:HGNC Symbol;Acc:HGNC:30006]
ENSG00000212232	0,41424929	0,61045944	2,0444856	0,18565178	0,48336961	SNORD17	small nucleolar RNA, C/D box 17 [Source:HGNC Symbol;Acc:HGNC:32713]
ENSG00000131263	0,32197537	4,12536182	2,04318431	0,18577884	0,48353403	RLIM	ring finger protein, LIM domain interacting [Source:HGNC Symbol;Acc:HGNC:13429]
ENSG00000134910	0,24022222	7,54419145	2,0431609	0,18578113	0,48353403	STT3A	STT3 oligosaccharyltransferase complex catalytic subunit A [Source:HGNC Symbol;Acc:HGNC:6172]
ENSG00000137970	-0,2486609	2,96865226	2,04218434	0,18587656	0,48369623	RPL7P9	ribosomal protein L7 pseudogene 9 [Source:HGNC Symbol;Acc:HGNC:37028]
ENSG00000140931	0,20533655	5,71949307	2,04018944	0,1860717	0,48411781	CMTM3	CKLF like MARVEL transmembrane domain containing 3 [Source:HGNC Symbol;Acc:HGNC:19174]
ENSG00000172922	-0,2529116	4,97694242	2,03965704	0,18612383	0,48416721	RNASEH2C	ribonuclease H2 subunit C [Source:HGNC Symbol;Acc:HGNC:24116]
ENSG00000156711	0,19158983	6,00548233	2,03917696	0,18617085	0,48420331	MAPK13	mitogen-activated protein kinase 13 [Source:HGNC Symbol;Acc:HGNC:6875]
ENSG00000188582	0,20107412	5,50210738	2,03879536	0,18620824	0,48421434	PAQR9	progestin and adipoQ receptor family member 9 [Source:HGNC Symbol;Acc:HGNC:30131]
ENSG00000162664	0,28494592	4,69873695	2,03794285	0,1862918	0,48425923	ZNF326	zinc finger protein 326 [Source:HGNC Symbol;Acc:HGNC:14104]
ENSG00000278464	-0,8653618	-1,2649387	2,03820378	0,18626622	0,48425923	AC068506.1	novel transcript
ENSG00000174514	-0,554099	-0,6209409	2,03710589	0,18637388	0,48433451	MFSD4A	major facilitator superfamily domain containing 4A [Source:HGNC Symbol;Acc:HGNC:25433]
ENSG00000229119	-0,2336344	2,52603753	2,03697152	0,18638707	0,48433451	AC026403.1	60S acidic ribosomal protein (RPLP0) pseudogene
ENSG00000173548	-0,2201795	4,58414498	2,03633403	0,18644962	0,48441091	SNX33	sorting nexin 33 [Source:HGNC Symbol;Acc:HGNC:28468]
ENSG00000140983	-0,2937594	5,91405689	2,0359157	0,18649069	0,48443145	RHOT2	ras homolog family member T2 [Source:HGNC Symbol;Acc:HGNC:21169]
ENSG00000103274	0,2957221	6,1559814	2,03508316	0,18657246	0,48455769	NUBP1	nucleotide binding protein 1 [Source:HGNC Symbol;Acc:HGNC:8041]
ENSG00000227232	-0,4978602	0,43360251	2,03469474	0,18661062	0,48457067	WASH7P	WASP family homolog 7, pseudogene [Source:HGNC Symbol;Acc:HGNC:38034]
ENSG00000174028	-0,4596071	-0,0699636	2,03412403	0,18666671	0,48463018	FAM3C2P	family with sequence similarity 3 member C2, pseudogene [Source:HGNC Symbol;Acc:HGNC:34501]
ENSG00000170653	0,29697148	2,27809582	2,03277311	0,18679958	0,48471671	ATF7	activating transcription factor 7 [Source:HGNC Symbol;Acc:HGNC:792]
ENSG00000184402	0,24004638	3,9556432	2,03336851	0,18674101	0,48471671	SS18L1	SS18L1 subunit of BAF chromatin remodeling complex [Source:HGNC Symbol;Acc:HGNC:15592]
ENSG00000257815	-0,4158144	0,40189801	2,03305056	0,18677228	0,48471671	PRANCR	progenitor renewal associated non-coding RNA [Source:HGNC Symbol;Acc:HGNC:51126]
ENSG00000070404	-0,2378503	3,66406217	2,03097102	0,18697702	0,48498782	FSTL3	follistatin like 3 [Source:HGNC Symbol;Acc:HGNC:3973]

ENSG00000204531	-0,815474	-0,8385905	2,03070072	0,18700365	0,48498782	POU5F1	POU class 5 homeobox 1 [Source:HGNC Symbol;Acc:HGNC:9221]
ENSG00000286101	-0,4980929	0,2347087	2,03087942	0,18698605	0,48498782	AC093894.2	novel transcript
ENSG00000163349	0,44728783	3,70971661	2,02978258	0,18709416	0,48513642	HIPK1	homeodomain interacting protein kinase 1 [Source:HGNC Symbol;Acc:HGNC:19006]
ENSG00000076944	-0,2464493	4,33828204	2,02925481	0,18714621	0,48518527	STXBP2	syntaxin binding protein 2 [Source:HGNC Symbol;Acc:HGNC:11445]
ENSG00000172339	-0,2026288	5,13028657	2,0278877	0,18728113	0,4852767	ALG14	ALG14 UDP-N-acetylglucosaminyltransferase subunit [Source:HGNC Symbol;Acc:HGNC:28287]
ENSG00000180769	-0,8047057	-1,7368297	2,0282265	0,18724768	0,4852767	WDFY3-AS2	WDFY3 antisense RNA 2 [Source:HGNC Symbol;Acc:HGNC:21603]
ENSG00000184445	0,22657037	3,72455728	2,02795386	0,1872746	0,4852767	KNTC1	kinetochore associated 1 [Source:HGNC Symbol;Acc:HGNC:17255]
ENSG00000131446	0,19016148	7,36815053	2,02611964	0,18745581	0,48561852	MGAT1	alpha-1,3-mannosyl-glycoprotein 2-beta-N-acetylglucosaminyltransferase [Source:HGNC Symbol;Acc:HGNC:7044]
ENSG00000219545	-0,2560654	2,84935961	2,02587979	0,18747953	0,48561852	UMAD1	UBAP1-MVB12-associated (UMA) domain containing 1 [Source:HGNC Symbol;Acc:HGNC:48955]
ENSG00000254531	-0,3191042	1,26073294	2,02514562	0,18755214	0,48572047	FLJ20021	uncharacterized LOC90024 [Source:NCBI gene (formerly Entrezgene);Acc:90024]
ENSG00000132840	0,21730342	3,55684122	2,02426807	0,18763898	0,48585924	BHMT2	betaine--homocysteine S-methyltransferase 2 [Source:HGNC Symbol;Acc:HGNC:1048]
ENSG00000112695	-0,2167531	7,80090272	2,02129603	0,18793348	0,48652002	COX7A2	cytochrome c oxidase subunit 7A2 [Source:HGNC Symbol;Acc:HGNC:2288]
ENSG00000168938	0,22275687	5,85532311	2,02102093	0,18796078	0,48652002	PPIC	peptidylprolyl isomerase C [Source:HGNC Symbol;Acc:HGNC:9256]
ENSG00000048707	0,35517792	3,48590422	2,02008828	0,18805334	0,48667338	VPS13D	vacuolar protein sorting 13 homolog D [Source:HGNC Symbol;Acc:HGNC:23595]
ENSG00000144395	0,33076828	2,05750929	2,0182755	0,18823343	0,48688069	CCDC150	coiled-coil domain containing 150 [Source:HGNC Symbol;Acc:HGNC:26834]
ENSG00000171160	0,28744862	1,87290393	2,01855054	0,18820609	0,48688069	MORN4	MORN repeat containing 4 [Source:HGNC Symbol;Acc:HGNC:24001]
ENSG00000186001	0,35005253	4,10004633	2,01862227	0,18819896	0,48688069	LRCH3	leucine rich repeats and calponin homology domain containing 3 [Source:HGNC Symbol;Acc:HGNC:28637]
ENSG00000105220	-0,2795056	8,27843239	2,01637987	0,18842199	0,48723175	GPI	glucose-6-phosphate isomerase [Source:HGNC Symbol;Acc:HGNC:4458]
ENSG00000179021	0,2754883	3,96112646	2,01624055	0,18843586	0,48723175	C3orf38	chromosome 3 open reading frame 38 [Source:HGNC Symbol;Acc:HGNC:28384]
ENSG00000205476	0,23714503	5,54069251	2,01571777	0,18848791	0,4872801	CCDC85C	coiled-coil domain containing 85C [Source:HGNC Symbol;Acc:HGNC:35459]
ENSG00000031823	0,21241864	6,60383529	2,01305673	0,18875315	0,48766837	RANBP3	RAN binding protein 3 [Source:HGNC Symbol;Acc:HGNC:9850]
ENSG00000118620	-0,3342669	2,07489372	2,01184867	0,18887374	0,48766837	ZNF430	zinc finger protein 430 [Source:HGNC Symbol;Acc:HGNC:20808]
ENSG00000141756	-0,2634163	8,19178433	2,01166148	0,18889243	0,48766837	FKBP10	FKBP prolyl isomerase 10 [Source:HGNC Symbol;Acc:HGNC:18169]
ENSG00000146904	0,21305131	4,19518261	2,01153412	0,18890515	0,48766837	EPHA1	EPH receptor A1 [Source:HGNC Symbol;Acc:HGNC:3385]
ENSG00000157916	-0,2079044	7,21322704	2,01278459	0,18878031	0,48766837	RER1	retention in endoplasmic reticulum sorting receptor 1 [Source:HGNC Symbol;Acc:HGNC:30309]
ENSG00000179101	-0,7013856	-1,0356969	2,01247606	0,1888111	0,48766837	H3P47	H3 histone pseudogene 47 [Source:HGNC Symbol;Acc:HGNC:54475]
ENSG00000237821	0,48717064	-0,4572667	2,01257755	0,18880097	0,48766837	ST13P7	ST13, Hsp70 interacting protein pseudogene 7 [Source:HGNC Symbol;Acc:HGNC:18558]
ENSG00000282826	0,26456345	4,02345574	2,01157562	0,188901	0,48766837	FRG1CP	FSHD region gene 1 family member C, pseudogene [Source:HGNC Symbol;Acc:HGNC:51762]
ENSG00000075618	0,24581996	6,03586964	2,0103016	0,1890283	0,48773781	FSCN1	fascin actin-bundling protein 1 [Source:HGNC Symbol;Acc:HGNC:11148]
ENSG00000104361	-0,4938088	-0,2504612	2,00959478	0,18909898	0,48773781	NIPAL2	NIPA like domain containing 2 [Source:HGNC Symbol;Acc:HGNC:25854]
ENSG00000198826	-0,2100702	4,05090224	2,0097174	0,18908672	0,48773781	ARHGAP11A	Rho GTPase activating protein 11A [Source:HGNC Symbol;Acc:HGNC:15783]
ENSG00000215012	-0,2177271	4,86684828	2,01057816	0,18900066	0,48773781	RTL10	retrotransposon Gag like 10 [Source:HGNC Symbol;Acc:HGNC:26112]
ENSG00000259319	-0,8899893	-1,9036085	2,0096573	0,18909273	0,48773781	AF111167.2	novel transcript, antisense to JDP2
ENSG00000126264	-0,6051642	-1,2222268	2,00911568	0,1891469	0,48775771	HCST	hematopoietic cell signal transducer [Source:HGNC Symbol;Acc:HGNC:16977]
ENSG00000197756	-0,2107282	11,0591576	2,00885018	0,18917347	0,48775771	RPL37A	ribosomal protein L37a [Source:HGNC Symbol;Acc:HGNC:10348]
ENSG00000101639	0,21252551	3,28400743	2,00665011	0,18939379	0,48785193	CEP192	centrosomal protein 192 [Source:HGNC Symbol;Acc:HGNC:25515]
ENSG00000110367	0,34430029	5,21738541	2,00732912	0,18932576	0,48785193	DDX6	DEAD-box helicase 6 [Source:HGNC Symbol;Acc:HGNC:2747]
ENSG00000148985	0,22333152	5,36180008	2,00712327	0,18934638	0,48785193	PGAP2	post-GPI attachment to proteins 2 [Source:HGNC Symbol;Acc:HGNC:17893]
ENSG00000171914	0,36805477	4,47235623	2,0064847	0,18941037	0,48785193	TLN2	talin 2 [Source:HGNC Symbol;Acc:HGNC:15447]
ENSG00000236992	-0,3953463	0,3450618	2,00660792	0,18939802	0,48785193	RPL12P12	ribosomal protein L12 pseudogene 12 [Source:HGNC Symbol;Acc:HGNC:15951]
ENSG00000272690	-0,6100286	-1,2128852	2,00652801	0,18940603	0,48785193	LINC02018	long intergenic non-protein coding RNA 2018 [Source:HGNC Symbol;Acc:HGNC:52853]
ENSG00000127947	0,21279556	5,12680415	2,00505558	0,18955369	0,48798619	PTPN12	protein tyrosine phosphatase non-receptor type 12 [Source:HGNC Symbol;Acc:HGNC:9645]
ENSG00000166233	0,21296204	4,76705561	2,00549692	0,18950942	0,48798619	ARIH1	ariadne RBR E3 ubiquitin protein ligase 1 [Source:HGNC Symbol;Acc:HGNC:689]
ENSG00000287236	-0,7080281	-1,0720245	2,00496571	0,18956271	0,48798619	Z73420.1	novel transcript, antisense to ANKRD54

ENSG00000160255	-0,2457114	3,68767381	2,00442773	0,1896167	0,48803918	ITGB2	integrin subunit beta 2 [Source:HGNC Symbol;Acc:HGNC:6155]
ENSG00000173212	0,34861303	1,2714622	2,00372083	0,18968768	0,48806114	MAB21L3	mab-21 like 3 [Source:HGNC Symbol;Acc:HGNC:26787]
ENSG00000198961	0,24145254	4,72326832	2,00367729	0,18969205	0,48806114	PJA2	praja ring finger ubiquitin ligase 2 [Source:HGNC Symbol;Acc:HGNC:17481]
ENSG00000070413	0,20407706	6,25014497	2,00274897	0,18978531	0,48806427	DGCR2	DiGeorge syndrome critical region gene 2 [Source:HGNC Symbol;Acc:HGNC:2845]
ENSG00000180914	-0,4251908	0,38863661	2,00215506	0,18984501	0,48806427	OXTR	oxytocin receptor [Source:HGNC Symbol;Acc:HGNC:8529]
ENSG00000185361	0,2249544	4,82765776	2,00246183	0,18981417	0,48806427	TNFAIP8L1	TNF alpha induced protein 8 like 1 [Source:HGNC Symbol;Acc:HGNC:28279]
ENSG00000187994	-0,2638456	2,29643878	2,00200292	0,18986031	0,48806427	RINL	Ras and Rab interactor like [Source:HGNC Symbol;Acc:HGNC:24795]
ENSG00000198804	0,18439127	12,5760159	2,00264368	0,18979589	0,48806427	MT-CO1	mitochondrially encoded cytochrome c oxidase I [Source:HGNC Symbol;Acc:HGNC:7419]
ENSG00000065970	0,26531839	1,92926036	2,0002232	0,19003937	0,4882009	FOXJ2	forkhead box J2 [Source:HGNC Symbol;Acc:HGNC:24818]
ENSG00000107929	0,20646637	5,18493951	2,00002415	0,19005942	0,4882009	LARP4B	La ribonucleoprotein 4B [Source:HGNC Symbol;Acc:HGNC:28987]
ENSG00000204311	0,65629616	-1,4446797	2,00004493	0,19005732	0,4882009	PJVK	pejvakin [Source:HGNC Symbol;Acc:HGNC:29502]
ENSG00000213174	-0,865707	-1,7724506	2,00092124	0,18996912	0,4882009	AC026316.1	ribosomal protein L28 (RPL28) pseudogene
ENSG00000285976	0,57610269	-0,8169702	1,99981429	0,19008055	0,4882009	AL135905.2	novel protein
ENSG00000059758	0,30034866	3,4852093	1,99713561	0,19035056	0,48852388	CDK17	cyclin dependent kinase 17 [Source:HGNC Symbol;Acc:HGNC:8750]
ENSG00000102854	-0,378808	1,44485095	1,9969083	0,1903735	0,48852388	MSLN	mesothelin [Source:HGNC Symbol;Acc:HGNC:7371]
ENSG00000104969	0,20515783	7,44498522	1,99799446	0,19026393	0,48852388	SGTA	small glutamine rich tetratricopeptide repeat containing alpha [Source:HGNC Symbol;Acc:HGNC:10819]
ENSG00000213763	-0,3118999	2,09851765	1,99749458	0,19031435	0,48852388	ACTBP2	ACTB pseudogene 2 [Source:HGNC Symbol;Acc:HGNC:135]
ENSG00000249264	-0,2313091	4,25838709	1,99713375	0,19035075	0,48852388	EEF1A1P9	eukaryotic translation elongation factor 1 alpha 1 pseudogene 9 [Source:HGNC Symbol;Acc:HGNC:3204]
ENSG00000091947	0,19544586	6,62735211	1,99634298	0,19043056	0,48858449	TMEM101	transmembrane protein 101 [Source:HGNC Symbol;Acc:HGNC:28653]
ENSG00000103037	0,24042743	4,36373533	1,9955775	0,19050786	0,48861119	SETD6	SET domain containing 6, protein lysine methyltransferase [Source:HGNC Symbol;Acc:HGNC:26116]
ENSG00000144635	0,21915254	5,63539987	1,99572139	0,19049333	0,48861119	DYNC1LI1	dynein cytoplasmic 1 light intermediate chain 1 [Source:HGNC Symbol;Acc:HGNC:18745]
ENSG00000141698	0,23775536	5,90841206	1,99517836	0,19054818	0,48862882	NT5C3B	5'-nucleotidase, cytosolic IIIB [Source:HGNC Symbol;Acc:HGNC:28300]
ENSG00000135338	0,47231882	0,75376787	1,9926943	0,19079938	0,48918711	LCA5	lebercilin LCA5 [Source:HGNC Symbol;Acc:HGNC:31923]
ENSG00000100908	-0,2637953	6,29177777	1,99217482	0,19085197	0,48923608	EMC9	ER membrane protein complex subunit 9 [Source:HGNC Symbol;Acc:HGNC:20273]
ENSG00000065526	0,62247423	5,03803333	1,99142572	0,19092784	0,4893447	SPEN	spen family transcriptional repressor [Source:HGNC Symbol;Acc:HGNC:17575]
ENSG00000078699	0,24160094	4,18364107	1,98909441	0,19116421	0,48982797	CBFA2T2	CBFA2/RUNX1 partner transcriptional co-repressor 2 [Source:HGNC Symbol;Acc:HGNC:1536]
ENSG00000081870	0,24101817	5,13319491	1,98890473	0,19118345	0,48982797	HSPB11	heat shock protein family B (small) member 11 [Source:HGNC Symbol;Acc:HGNC:25019]
ENSG00000005194	0,19284708	7,41956561	1,98736169	0,19134014	0,49003223	CIAPIN1	cytokine induced apoptosis inhibitor 1 [Source:HGNC Symbol;Acc:HGNC:28050]
ENSG00000102898	-0,2437646	6,50714812	1,98739663	0,19133659	0,49003223	NUTF2	nuclear transport factor 2 [Source:HGNC Symbol;Acc:HGNC:13722]
ENSG00000120008	0,29398649	4,66721614	1,98672755	0,19140458	0,49003223	WDR11	WD repeat domain 11 [Source:HGNC Symbol;Acc:HGNC:13831]
ENSG00000130638	0,19802296	6,73825401	1,98646864	0,1914309	0,49003223	ATXN10	ataxin 10 [Source:HGNC Symbol;Acc:HGNC:10549]
ENSG00000184900	0,2384387	5,93241339	1,98649602	0,19142811	0,49003223	SUMO3	small ubiquitin like modifier 3 [Source:HGNC Symbol;Acc:HGNC:11124]
ENSG00000162923	0,21039632	5,7027363	1,98584517	0,19149429	0,49010864	WDR26	WD repeat domain 26 [Source:HGNC Symbol;Acc:HGNC:21208]
ENSG00000152127	0,42547871	5,73074059	1,9846044	0,19162054	0,49018	MGAT5	alpha-1,6-mannosylglycoprotein 6-beta-N-acetylglucosaminyltransferase [Source:HGNC Symbol;Acc:HGNC:7049]
ENSG00000168556	-0,3296683	2,60150577	1,98500595	0,19157967	0,49018	ING2	inhibitor of growth family member 2 [Source:HGNC Symbol;Acc:HGNC:6063]
ENSG00000169548	-0,4191157	0,19721479	1,98450563	0,1916306	0,49018	ZNF280A	zinc finger protein 280A [Source:HGNC Symbol;Acc:HGNC:18597]
ENSG00000235605	-0,3587898	0,68755578	1,98425231	0,19165639	0,49018	AL355472.1	ribosomal protein S15 (RPS15) pseudogene
ENSG00000066557	0,26341439	4,04205242	1,98253657	0,19183119	0,49020093	LRRC40	leucine rich repeat containing 40 [Source:HGNC Symbol;Acc:HGNC:26004]
ENSG00000074266	0,20716268	4,15485725	1,9825229	0,19183258	0,49020093	EED	embryonic ectoderm development [Source:HGNC Symbol;Acc:HGNC:3188]
ENSG00000077044	-0,2381962	4,89175769	1,98323479	0,19176003	0,49020093	DGKD	diacylglycerol kinase delta [Source:HGNC Symbol;Acc:HGNC:2851]
ENSG00000117411	-0,2298531	6,64767774	1,98360183	0,19172263	0,49020093	B4GALT2	beta-1,4-galactosyltransferase 2 [Source:HGNC Symbol;Acc:HGNC:925]
ENSG00000162460	-0,5670227	0,65171061	1,98219614	0,1918659	0,49020093	TMEM82	transmembrane protein 82 [Source:HGNC Symbol;Acc:HGNC:32350]
ENSG00000166562	0,23156723	8,118969	1,98256949	0,19182783	0,49020093	SEC11C	SEC11 homolog C, signal peptidase complex subunit [Source:HGNC Symbol;Acc:HGNC:23400]
ENSG00000129214	-0,3368876	4,23885676	1,98073075	0,1920154	0,49049711	SHBG	sex hormone binding globulin [Source:HGNC Symbol;Acc:HGNC:10839]

ENSG00000197386	0,30674181	5,38550104	1,97986982	0,19210331	0,49063588	HTT	huntingtin [Source:HGNC Symbol;Acc:HGNC:4851]
ENSG00000145495	0,23767543	5,59288229	1,97903668	0,19218843	0,49076747	MARCHF6	membrane associated ring-CH-type finger 6 [Source:HGNC Symbol;Acc:HGNC:30550]
ENSG00000143067	0,28522699	2,41988545	1,97702193	0,19239448	0,49112955	ZNF697	zinc finger protein 697 [Source:HGNC Symbol;Acc:HGNC:32034]
ENSG00000196419	0,23454935	9,79331772	1,97699283	0,19239745	0,49112955	XRCC6	X-ray repair cross complementing 6 [Source:HGNC Symbol;Acc:HGNC:4055]
ENSG00000166965	-0,2203811	3,62557512	1,97503114	0,19259836	0,49155652	RCCD1	RCC1 domain containing 1 [Source:HGNC Symbol;Acc:HGNC:30457]
ENSG00000149328	0,70512204	-1,2650987	1,97414932	0,19268877	0,49170135	GLB1L2	galactosidase beta 1 like 2 [Source:HGNC Symbol;Acc:HGNC:25129]
ENSG00000255769	0,26873134	2,31042488	1,97370068	0,19273478	0,49173288	GOLGA2P10	GOLGA2 pseudogene 10 [Source:HGNC Symbol;Acc:HGNC:26229]
ENSG00000165458	0,21187048	5,05707992	1,97260516	0,19284721	0,49193381	INPPL1	inositol polyphosphate phosphatase like 1 [Source:HGNC Symbol;Acc:HGNC:6080]
ENSG00000139637	-0,5414288	-0,6486209	1,9721763	0,19289125	0,49196024	MYG1	MYG1 exonuclease [Source:HGNC Symbol;Acc:HGNC:17590]
ENSG00000065923	-0,3096808	2,96519044	1,97082804	0,19302978	0,49222761	SLC9A7	solute carrier family 9 member A7 [Source:HGNC Symbol;Acc:HGNC:17123]
ENSG00000164663	0,39276291	0,96076889	1,96997953	0,19311702	0,49236415	USP49	ubiquitin specific peptidase 49 [Source:HGNC Symbol;Acc:HGNC:20078]
ENSG00000243680	-0,3444894	0,83233601	1,96960647	0,1931554	0,49237607	RPL37P23	ribosomal protein L37 pseudogene 23 [Source:HGNC Symbol;Acc:HGNC:37003]
ENSG00000105963	-0,379371	1,26569011	1,96904782	0,19321289	0,49238161	ADAP1	ArfGAP with dual PH domains 1 [Source:HGNC Symbol;Acc:HGNC:16486]
ENSG00000154957	-0,265772	2,25293497	1,96893032	0,19322498	0,49238161	ZNF18	zinc finger protein 18 [Source:HGNC Symbol;Acc:HGNC:12969]
ENSG00000138942	0,20521874	4,80222302	1,96771904	0,19334972	0,49239959	RNF185	ring finger protein 185 [Source:HGNC Symbol;Acc:HGNC:26783]
ENSG00000159461	0,21375742	7,3505153	1,96671312	0,19345339	0,49239959	AMFR	autocrine motility factor receptor [Source:HGNC Symbol;Acc:HGNC:463]
ENSG00000178605	-0,1829112	6,31865512	1,96675091	0,19344949	0,49239959	GTPBP6	GTP binding protein 6 (putative) [Source:HGNC Symbol;Acc:HGNC:30189]
ENSG00000185269	-0,2319839	9,10904174	1,96799622	0,19332116	0,49239959	NOTUM	notum, palmitoleoyl-protein carboxylesterase [Source:HGNC Symbol;Acc:HGNC:27106]
ENSG00000185596	-0,2363882	2,64616761	1,96824224	0,19329583	0,49239959	WASH3P	WASP family homolog 3, pseudogene [Source:HGNC Symbol;Acc:HGNC:24362]
ENSG00000197498	-0,211283	6,24576658	1,96744528	0,19337792	0,49239959	RPF2	ribosome production factor 2 homolog [Source:HGNC Symbol;Acc:HGNC:20870]
ENSG00000197989	-0,2726327	3,143959	1,96657161	0,19346797	0,49239959	SNHG12	small nucleolar RNA host gene 12 [Source:HGNC Symbol;Acc:HGNC:30062]
ENSG00000132002	0,26601065	7,81093602	1,96517149	0,19361241	0,49247569	DNAJB1	DnaJ heat shock protein family (Hsp40) member B1 [Source:HGNC Symbol;Acc:HGNC:5270]
ENSG00000137486	-0,2144663	5,77200548	1,96517705	0,19361183	0,49247569	ARRB1	arrestin beta 1 [Source:HGNC Symbol;Acc:HGNC:711]
ENSG00000156804	0,71725707	-1,0637482	1,96496657	0,19363356	0,49247569	FBXO32	F-box protein 32 [Source:HGNC Symbol;Acc:HGNC:16731]
ENSG00000182185	0,30497236	2,48946369	1,96464817	0,19366643	0,49247569	RAD51B	RAD51 paralog B [Source:HGNC Symbol;Acc:HGNC:9822]
ENSG00000213740	-0,3510955	0,79529845	1,96493113	0,19363722	0,49247569	SERBP1P1	SERPINE1 mRNA binding protein 1 pseudogene 1 [Source:HGNC Symbol;Acc:HGNC:32247]
ENSG00000103966	-0,3431636	3,94616558	1,9630043	0,19383625	0,49265028	EHD4	EH domain containing 4 [Source:HGNC Symbol;Acc:HGNC:3245]
ENSG00000117834	-0,2240862	3,99647218	1,96332908	0,19380269	0,49265028	SLC5A9	solute carrier family 5 member 9 [Source:HGNC Symbol;Acc:HGNC:22146]
ENSG00000174173	0,21382639	5,43274457	1,96324964	0,1938109	0,49265028	TRMT10C	tRNA methyltransferase 10C, mitochondrial RNase P subunit [Source:HGNC Symbol;Acc:HGNC:26022]
ENSG00000163755	0,2380784	4,17966753	1,96199725	0,19394039	0,49282921	HPS3	HPS3 biogenesis of lysosomal organelles complex 2 subunit 1 [Source:HGNC Symbol;Acc:HGNC:15597]
ENSG00000170873	0,25200603	3,99379883	1,96153664	0,19398804	0,49286458	MTSS1	MTSS I-BAR domain containing 1 [Source:HGNC Symbol;Acc:HGNC:20443]
ENSG00000125046	0,22399504	5,80515741	1,96052159	0,19409311	0,49296006	SSUH2	ssu-2 homolog [Source:HGNC Symbol;Acc:HGNC:24809]
ENSG00000129535	-0,2983658	1,2583271	1,96082471	0,19406173	0,49296006	NRL	neural retina leucine zipper [Source:HGNC Symbol;Acc:HGNC:8002]
ENSG00000001561	0,87960926	-0,7810059	1,95881302	0,19427014	0,49330988	ENPP4	ectonucleotide pyrophosphatase/phosphodiesterase 4 [Source:HGNC Symbol;Acc:HGNC:3359]
ENSG00000113638	0,23775186	3,31287112	1,95854071	0,19429838	0,49330988	TTC33	tetratricopeptide repeat domain 33 [Source:HGNC Symbol;Acc:HGNC:29959]
ENSG00000037749	0,25128328	4,44863697	1,95726518	0,19443071	0,49347433	MFAP3	microfibril associated protein 3 [Source:HGNC Symbol;Acc:HGNC:7034]
ENSG00000197045	0,21531157	4,32319601	1,95740633	0,19441606	0,49347433	GMFB	glia maturation factor beta [Source:HGNC Symbol;Acc:HGNC:4373]
ENSG00000115282	-0,2748261	4,39209539	1,95632335	0,1945285	0,49355102	TTC31	tetratricopeptide repeat domain 31 [Source:HGNC Symbol;Acc:HGNC:25759]
ENSG00000268412	-0,639596	-1,454484	1,9564382	0,19451657	0,49355102	TRMT112P6	tRNA methyltransferase subunit 11-2 pseudogene 6 [Source:HGNC Symbol;Acc:HGNC:44025]
ENSG00000136521	0,23205776	5,15304822	1,95443069	0,1947252	0,49387856	NDUFB5	NADH:ubiquinone oxidoreductase subunit B5 [Source:HGNC Symbol;Acc:HGNC:7700]
ENSG00000214756	-0,2398435	2,72717827	1,95459049	0,19470858	0,49387856	CSKMT	citrate synthase lysine methyltransferase [Source:HGNC Symbol;Acc:HGNC:33113]
ENSG00000082805	0,5490799	3,84602161	1,95328378	0,19484453	0,49395294	ERC1	ELKS/RAB6-interacting/CAST family member 1 [Source:HGNC Symbol;Acc:HGNC:17072]
ENSG00000137822	-0,1972248	5,01111621	1,95330184	0,19484265	0,49395294	TUBGCP4	tubulin gamma complex associated protein 4 [Source:HGNC Symbol;Acc:HGNC:16691]
ENSG00000197951	0,29363507	2,3809515	1,95317394	0,19485596	0,49395294	ZNF71	zinc finger protein 71 [Source:HGNC Symbol;Acc:HGNC:13141]

ENSG00000156500	-0,6277012	-1,2821741	1,95220158	0,19495722	0,49412387	FAM122C	family with sequence similarity 122C [Source:HGNC Symbol;Acc:HGNC:25202]
ENSG00000111605	-0,1945964	5,85177588	1,94993649	0,19519336	0,4943381	CPSF6	cleavage and polyadenylation specific factor 6 [Source:HGNC Symbol;Acc:HGNC:13871]
ENSG00000135976	0,29994092	1,26546049	1,94976805	0,19521093	0,4943381	ANKRD36	ankyrin repeat domain 36 [Source:HGNC Symbol;Acc:HGNC:24079]
ENSG00000159423	0,26547187	7,3405215	1,94980073	0,19520752	0,4943381	ALDH4A1	aldehyde dehydrogenase 4 family member A1 [Source:HGNC Symbol;Acc:HGNC:406]
ENSG00000188566	-0,3496346	3,53875427	1,9499543	0,1951915	0,4943381	NDOR1	NADPH dependent diflavin oxidoreductase 1 [Source:HGNC Symbol;Acc:HGNC:29838]
ENSG00000242242	0,96374777	-1,8047862	1,95005642	0,19518084	0,4943381	NECTIN3-AS1	NECTIN3 antisense RNA 1 [Source:HGNC Symbol;Acc:HGNC:40813]
ENSG00000033170	0,23394856	4,16961285	1,94757843	0,19543959	0,4943726	FUT8	fucosyltransferase 8 [Source:HGNC Symbol;Acc:HGNC:4019]
ENSG00000044574	-0,3640482	11,945208	1,94879927	0,19531206	0,4943726	HSPA5	heat shock protein family A (Hsp70) member 5 [Source:HGNC Symbol;Acc:HGNC:5238]
ENSG00000069399	0,21359948	4,10624943	1,94736402	0,195462	0,4943726	BCL3	BCL3 transcription coactivator [Source:HGNC Symbol;Acc:HGNC:998]
ENSG00000100888	0,30374762	6,1250961	1,94662584	0,19553918	0,4943726	CHD8	chromodomain helicase DNA binding protein 8 [Source:HGNC Symbol;Acc:HGNC:20153]
ENSG00000132763	-0,2113752	3,88756583	1,94639851	0,19556296	0,4943726	MMACHC	metabolism of cobalamin associated C [Source:HGNC Symbol;Acc:HGNC:24525]
ENSG00000143919	0,25066692	3,31887037	1,94662778	0,19553898	0,4943726	CAMKMT	calmodulin-lysine N-methyltransferase [Source:HGNC Symbol;Acc:HGNC:26276]
ENSG00000175538	-0,6804597	-0,9805285	1,94687116	0,19551353	0,4943726	KCNE3	potassium voltage-gated channel subfamily E regulatory subunit 3 [Source:HGNC Symbol;Acc:HGNC:6243]
ENSG00000226180	0,36601451	1,06101236	1,94843956	0,19534962	0,4943726	AC010536.1	novel transcript
ENSG00000241506	0,39209825	0,45813756	1,94811733	0,19538328	0,4943726	PSMC1P1	proteasome 26S subunit, ATPase 1 pseudogene 1 [Source:HGNC Symbol;Acc:HGNC:30147]
ENSG00000245648	0,72648898	-1,2922856	1,9476213	0,19543511	0,4943726	KLRK1-AS1	KLRK1 antisense RNA 1 [Source:HGNC Symbol;Acc:HGNC:54868]
ENSG00000220924	-0,743219	-1,6850031	1,94520213	0,19568815	0,4946035	OSTCP4	oligosaccharyltransferase complex subunit pseudogene 4 [Source:HGNC Symbol;Acc:HGNC:42866]
ENSG00000133104	-0,2211587	4,20618892	1,94468921	0,19574186	0,49465235	SPART	spartin [Source:HGNC Symbol;Acc:HGNC:18514]
ENSG00000163257	0,24664898	3,23005011	1,9443709	0,1957752	0,49465235	DCAF16	DDB1 and CUL4 associated factor 16 [Source:HGNC Symbol;Acc:HGNC:25987]
ENSG00000001461	0,2452316	2,65712976	1,94266036	0,19595449	0,49467758	NIPAL3	NIPA like domain containing 3 [Source:HGNC Symbol;Acc:HGNC:25233]
ENSG00000075303	-0,2502693	2,35267577	1,9428812	0,19593133	0,49467758	SLC25A40	solute carrier family 25 member 40 [Source:HGNC Symbol;Acc:HGNC:29680]
ENSG00000102753	-0,2163891	5,41468185	1,94295841	0,19592323	0,49467758	KPNA3	karyopherin subunit alpha 3 [Source:HGNC Symbol;Acc:HGNC:6396]
ENSG00000122483	-0,3400455	1,96197073	1,94280522	0,1959393	0,49467758	CCDC18	coiled-coil domain containing 18 [Source:HGNC Symbol;Acc:HGNC:30370]
ENSG00000125744	0,22150583	3,82823101	1,94321358	0,19589648	0,49467758	RTN2	reticulon 2 [Source:HGNC Symbol;Acc:HGNC:10468]
ENSG00000168487	-0,2530853	4,1188191	1,94099028	0,19612975	0,49494896	BMP1	bone morphogenetic protein 1 [Source:HGNC Symbol;Acc:HGNC:1067]
ENSG00000249471	-0,3202422	1,28578339	1,94108473	0,19611983	0,49494896	ZNF324B	zinc finger protein 324B [Source:HGNC Symbol;Acc:HGNC:33107]
ENSG00000147669	-0,2592289	4,59712644	1,94014599	0,19621843	0,49499233	POLR2K	RNA polymerase II, I and III subunit K [Source:HGNC Symbol;Acc:HGNC:9198]
ENSG00000178498	0,2863571	2,60291555	1,93985905	0,19624858	0,49499233	DTX3	deltex E3 ubiquitin ligase 3 [Source:HGNC Symbol;Acc:HGNC:24457]
ENSG00000272384	-0,5738467	-0,7117825	1,94029873	0,19620239	0,49499233	AC016405.3	novel transcript
ENSG00000165526	0,19763071	5,98998452	1,93949766	0,19628657	0,49500267	RPUSD4	RNA pseudouridine synthase D4 [Source:HGNC Symbol;Acc:HGNC:25898]
ENSG00000119318	-0,1858464	7,11694818	1,93654268	0,19659752	0,49548712	RAD23B	RAD23 homolog B, nucleotide excision repair protein [Source:HGNC Symbol;Acc:HGNC:9813]
ENSG00000131013	0,23560737	5,73779781	1,93655313	0,19659642	0,49548712	PPIL4	peptidylprolyl isomerase like 4 [Source:HGNC Symbol;Acc:HGNC:15702]
ENSG00000132881	-0,7794185	-1,2465567	1,93638303	0,19661433	0,49548712	CPLANE2	ciliogenesis and planar polarity effector 2 [Source:HGNC Symbol;Acc:HGNC:28127]
ENSG00000139269	-0,3538652	5,90917001	1,93734593	0,19651293	0,49548712	INHBE	inhibin subunit beta E [Source:HGNC Symbol;Acc:HGNC:24029]
ENSG00000156802	0,3136073	6,25540101	1,93537245	0,19672084	0,49567001	ATAD2	ATPase family AAA domain containing 2 [Source:HGNC Symbol;Acc:HGNC:30123]
ENSG00000174672	0,69107678	-1,290649	1,93449706	0,19681316	0,49581711	BRSK2	BR serine/threonine kinase 2 [Source:HGNC Symbol;Acc:HGNC:11405]
ENSG00000162337	0,20162125	7,66321513	1,93345539	0,19692309	0,49584673	LRP5	LDL receptor related protein 5 [Source:HGNC Symbol;Acc:HGNC:6697]
ENSG00000162783	-0,3116842	3,08423933	1,93342083	0,19692674	0,49584673	IER5	immediate early response 5 [Source:HGNC Symbol;Acc:HGNC:5393]
ENSG00000235084	-0,4014966	0,35092675	1,93366181	0,1969013	0,49584673	CHCHD2P6	coiled-coil-helix-coiled-coil-helix domain containing 2 pseudogene 6 [Source:HGNC Symbol;Acc:HGNC:39590]
ENSG00000046651	0,25214489	3,39754751	1,9325528	0,19701841	0,49599206	OFD1	OFD1 centriole and centriolar satellite protein [Source:HGNC Symbol;Acc:HGNC:2567]
ENSG00000104976	0,2017025	4,61187154	1,93012669	0,19727493	0,49638124	SNAPC2	small nuclear RNA activating complex polypeptide 2 [Source:HGNC Symbol;Acc:HGNC:11135]
ENSG00000226471	-0,5511756	-0,58717	1,93022972	0,19726403	0,49638124	Z93930.2	novel transcript
ENSG00000228175	-0,5243214	-0,7091324	1,93063713	0,19722092	0,49638124	GEMIN8P4	gem nuclear organelle associated protein 8 pseudogene 4 [Source:HGNC Symbol;Acc:HGNC:37979]
ENSG00000155760	-0,7025769	-1,1024408	1,92856924	0,19743984	0,49671063	FZD7	frizzled class receptor 7 [Source:HGNC Symbol;Acc:HGNC:4045]

ENSG00000259330	-0,3664037	1,87136269	1,92756991	0,19754575	0,49680597	INAFM2	InaF motif containing 2 [Source:HGNC Symbol;Acc:HGNC:35165]
ENSG00000264538	0,24763341	2,04627754	1,92777122	0,19752441	0,49680597	SUZ12P1	SUZ12 pseudogene 1 [Source:HGNC Symbol;Acc:HGNC:32421]
ENSG00000094975	0,2830491	4,61449381	1,92617074	0,19769417	0,49709364	SUCO	SUN domain containing ossification factor [Source:HGNC Symbol;Acc:HGNC:1240]
ENSG00000125962	-0,247094	2,81129744	1,92575958	0,19773781	0,49711781	ARMCX5	armadillo repeat containing X-linked 5 [Source:HGNC Symbol;Acc:HGNC:25772]
ENSG00000035141	0,21595043	7,56126771	1,92454836	0,19786644	0,49717466	FAM136A	family with sequence similarity 136 member A [Source:HGNC Symbol;Acc:HGNC:25911]
ENSG00000099795	-0,1869748	6,79540095	1,92426507	0,19789655	0,49717466	NDUFB7	NADH:ubiquinone oxidoreductase subunit B7 [Source:HGNC Symbol;Acc:HGNC:7702]
ENSG00000115109	0,2335098	5,64763547	1,92484105	0,19783535	0,49717466	EPB41L5	erythrocyte membrane protein band 4.1 like 5 [Source:HGNC Symbol;Acc:HGNC:19819]
ENSG00000198855	-0,2365099	3,03579461	1,92513046	0,19780461	0,49717466	FICD	FIC domain protein adenylyltransferase [Source:HGNC Symbol;Acc:HGNC:18416]
ENSG00000010017	0,21843198	5,51595857	1,92319732	0,19801006	0,49723038	RANBP9	RAN binding protein 9 [Source:HGNC Symbol;Acc:HGNC:13727]
ENSG00000104312	0,21705951	4,1642107	1,92181661	0,19815698	0,49723038	RIPK2	receptor interacting serine/threonine kinase 2 [Source:HGNC Symbol;Acc:HGNC:10020]
ENSG00000106546	0,27316789	2,6108556	1,92288146	0,19804366	0,49723038	AHR	aryl hydrocarbon receptor [Source:HGNC Symbol;Acc:HGNC:348]
ENSG00000139436	0,29376835	4,13977595	1,92195044	0,19814273	0,49723038	GIT2	GIT ArfGAP 2 [Source:HGNC Symbol;Acc:HGNC:4273]
ENSG00000149187	0,37104403	5,28656006	1,92220839	0,19811527	0,49723038	CELF1	CUGBP Elav-like family member 1 [Source:HGNC Symbol;Acc:HGNC:2549]
ENSG00000169715	-0,2535959	7,10416322	1,92236786	0,1980983	0,49723038	MT1E	metallothionein 1E [Source:HGNC Symbol;Acc:HGNC:7397]
ENSG00000176731	-0,2683138	4,69849683	1,92305747	0,19802493	0,49723038	RBIS	ribosomal biogenesis factor [Source:HGNC Symbol;Acc:HGNC:32235]
ENSG00000091138	-0,7175967	-1,5627887	1,91873642	0,19848525	0,49754136	SLC26A3	solute carrier family 26 member 3 [Source:HGNC Symbol;Acc:HGNC:3018]
ENSG00000095380	-0,1877528	7,50284788	1,91943091	0,19841117	0,49754136	NANS	N-acetylneuraminate synthase [Source:HGNC Symbol;Acc:HGNC:19237]
ENSG00000108984	0,25163628	3,76887102	1,9189725	0,19846007	0,49754136	MAP2K6	mitogen-activated protein kinase kinase 6 [Source:HGNC Symbol;Acc:HGNC:6846]
ENSG00000176209	-0,237299	2,81472753	1,91931528	0,1984235	0,49754136	SMIM19	small integral membrane protein 19 [Source:HGNC Symbol;Acc:HGNC:25166]
ENSG00000178096	-0,3136776	1,25892218	1,91981015	0,19837074	0,49754136	BOLA1	bolA family member 1 [Source:HGNC Symbol;Acc:HGNC:24263]
ENSG00000196843	-0,2305066	4,01399277	1,91876685	0,19848201	0,49754136	ARID5A	AT-rich interaction domain 5A [Source:HGNC Symbol;Acc:HGNC:17361]
ENSG00000100075	-0,2082985	7,96934907	1,91801295	0,19856246	0,49764952	SLC25A1	solute carrier family 25 member 1 [Source:HGNC Symbol;Acc:HGNC:10979]
ENSG00000084676	0,35163236	2,68159198	1,91601848	0,19877553	0,49771912	NCOA1	nuclear receptor coactivator 1 [Source:HGNC Symbol;Acc:HGNC:7668]
ENSG00000113273	-0,1972734	6,57502281	1,91651583	0,19872237	0,49771912	ARSB	arylsulfatase B [Source:HGNC Symbol;Acc:HGNC:714]
ENSG00000142002	0,19911241	6,45919826	1,91739266	0,19862869	0,49771912	DPP9	dipeptidyl peptidase 9 [Source:HGNC Symbol;Acc:HGNC:18648]
ENSG00000180190	0,31161008	3,27299085	1,91667769	0,19870507	0,49771912	TDRP	testis development related protein [Source:HGNC Symbol;Acc:HGNC:26951]
ENSG00000227963	-0,6539101	-1,096193	1,91624414	0,19875141	0,49771912	RBM15-AS1	RBM15 antisense RNA 1 [Source:HGNC Symbol;Acc:HGNC:53636]
ENSG00000228623	0,50642385	0,32990135	1,91583959	0,19879465	0,49771912	ZNF883	zinc finger protein 883 [Source:HGNC Symbol;Acc:HGNC:27271]
ENSG00000257773	0,846283	-1,6488287	1,91495559	0,1988892	0,49787051	ST13P3	ST13, Hsp70 interacting protein pseudogene 3 [Source:HGNC Symbol;Acc:HGNC:18552]
ENSG00000162757	0,56221183	0,04544443	1,91428464	0,198961	0,49796492	C1orf74	chromosome 1 open reading frame 74 [Source:HGNC Symbol;Acc:HGNC:26319]
ENSG00000085840	0,20366858	5,02806408	1,91383695	0,19900892	0,49799955	ORC1	origin recognition complex subunit 1 [Source:HGNC Symbol;Acc:HGNC:8487]
ENSG00000143157	-0,2143696	3,92381706	1,9130875	0,19908919	0,49804259	POGK	pogo transposable element derived with KRAB domain [Source:HGNC Symbol;Acc:HGNC:18800]
ENSG00000257135	-0,3417884	1,02202282	1,91303976	0,1990943	0,49804259	ODC1-DT	ODC1 divergent transcript [Source:HGNC Symbol;Acc:HGNC:54070]
ENSG00000102796	-0,463637	-0,011705	1,91210697	0,19919427	0,49812207	DHRS12	dehydrogenase/reductase 12 [Source:HGNC Symbol;Acc:HGNC:25832]
ENSG00000213024	0,18726704	5,46864689	1,91237322	0,19916573	0,49812207	NUP62	nucleoporin 62 [Source:HGNC Symbol;Acc:HGNC:8066]
ENSG00000076641	0,57143315	-0,5824929	1,90999694	0,19942065	0,49826666	PAG1	phosphoprotein membrane anchor with glycosphingolipid microdomains 1 [Source:HGNC Symbol;Acc:HGNC:300
ENSG00000100906	-0,3383014	7,05933723	1,90722858	0,19971818	0,49826666	NFKBIA	NFKB inhibitor alpha [Source:HGNC Symbol;Acc:HGNC:7797]
ENSG00000109917	-0,1944897	6,64105356	1,90862837	0,19956766	0,49826666	ZPR1	ZPR1 zinc finger [Source:HGNC Symbol;Acc:HGNC:13051]
ENSG00000114631	0,24334743	6,6997953	1,90825569	0,19960772	0,49826666	PODXL2	podocalyxin like 2 [Source:HGNC Symbol;Acc:HGNC:17936]
ENSG00000140107	0,80352309	-0,9402967	1,90712257	0,19972959	0,49826666	SLC25A47	solute carrier family 25 member 47 [Source:HGNC Symbol;Acc:HGNC:20115]
ENSG00000140451	-0,2585427	3,16146856	1,90767582	0,19967007	0,49826666	PIF1	PIF1 5'-to-3' DNA helicase [Source:HGNC Symbol;Acc:HGNC:26220]
ENSG00000151846	-0,4857065	-0,1659274	1,90921101	0,19950506	0,49826666	PABPC3	poly(A) binding protein cytoplasmic 3 [Source:HGNC Symbol;Acc:HGNC:8556]
ENSG00000167711	0,25607078	9,09948181	1,90770576	0,19966685	0,49826666	SERPINF2	serpin family F member 2 [Source:HGNC Symbol;Acc:HGNC:9075]
ENSG00000186940	-0,3654241	0,55703141	1,90778063	0,1996588	0,49826666	CHCHD2P9	coiled-coil-helix-coiled-coil-helix domain containing 2 pseudogene 9 [Source:HGNC Symbol;Acc:HGNC:23676]

ENSG00000197343	0,22351421	3,5362124	1,90974193	0,19944803	0,49826666	ZNF655	zinc finger protein 655 [Source:HGNC Symbol;Acc:HGNC:30899]
ENSG00000237976	-0,6189998	-0,7550598	1,91077872	0,19933674	0,49826666	RFX5-AS1	RFX5 antisense RNA 1 [Source:HGNC Symbol;Acc:HGNC:40503]
ENSG00000258168	0,53081965	-0,4938664	1,90788496	0,19964758	0,49826666	AC025569.1	novel transcript, antisense to PTPRB
ENSG00000274818	0,57114579	-1,1737959	1,90964811	0,19945811	0,49826666	AC004825.2	novel transcript
ENSG00000275395	-0,2781746	3,41090677	1,91063512	0,19935215	0,49826666	FCGBP	Fc fragment of IgG binding protein [Source:HGNC Symbol;Acc:HGNC:13572]
ENSG00000069011	0,19509086	4,73644348	1,90522149	0,19993427	0,49853147	PITX1	paired like homeodomain 1 [Source:HGNC Symbol;Acc:HGNC:9004]
ENSG00000113083	-0,2885789	5,38802657	1,90415622	0,20004908	0,49853147	LOX	lysyl oxidase [Source:HGNC Symbol;Acc:HGNC:6664]
ENSG00000128266	-0,2307893	3,655586	1,90309047	0,20016404	0,49853147	GNAZ	G protein subunit alpha z [Source:HGNC Symbol;Acc:HGNC:4395]
ENSG00000155561	0,23699101	5,67191566	1,90358904	0,20011025	0,49853147	NUP205	nucleoporin 205 [Source:HGNC Symbol;Acc:HGNC:18658]
ENSG00000163510	0,26088874	5,0815296	1,90443616	0,2000189	0,49853147	CWC22	CWC22 spliceosome associated protein homolog [Source:HGNC Symbol;Acc:HGNC:29322]
ENSG00000173588	-0,2499956	2,71194003	1,90342375	0,20012808	0,49853147	CEP83	centrosomal protein 83 [Source:HGNC Symbol;Acc:HGNC:17966]
ENSG00000179406	0,59499812	-0,1258862	1,90454333	0,20000735	0,49853147	LINC00174	long intergenic non-protein coding RNA 174 [Source:HGNC Symbol;Acc:HGNC:27788]
ENSG00000185065	0,85553537	-1,4841194	1,90499578	0,19995859	0,49853147	AC000068.1	novel transcript, antisense to C22orf39
ENSG00000256294	0,39958382	1,20979722	1,90297049	0,20017699	0,49853147	ZNF225	zinc finger protein 225 [Source:HGNC Symbol;Acc:HGNC:13018]
ENSG00000257497	1,09969835	-2,0581352	1,90537291	0,19991795	0,49853147	AC121761.1	novel transcript, antisense to GLIPR1
ENSG00000135452	0,26640313	4,08673425	1,89881745	0,20062583	0,499479	TSPAN31	tetraspanin 31 [Source:HGNC Symbol;Acc:HGNC:10539]
ENSG00000142687	0,21373575	5,73467894	1,89902637	0,20060322	0,499479	KIAA0319L	KIAA0319 like [Source:HGNC Symbol;Acc:HGNC:30071]
ENSG00000129355	-0,284922	1,92217398	1,89791783	0,20072324	0,49963636	CDKN2D	cyclin dependent kinase inhibitor 2D [Source:HGNC Symbol;Acc:HGNC:1790]
ENSG00000058729	0,24183992	5,23317621	1,89665383	0,2008602	0,49989211	RIOK2	RIO kinase 2 [Source:HGNC Symbol;Acc:HGNC:18999]
ENSG00000287394	0,6258982	-1,344086	1,8948974	0,20105074	0,50028107	AC244102.4	novel transcript, antisense to GABRQ
ENSG00000050820	-0,2254349	6,49982623	1,89440589	0,2011041	0,50032863	BCAR1	BCAR1 scaffold protein, Cas family member [Source:HGNC Symbol;Acc:HGNC:971]
ENSG00000154080	0,27020893	2,28719503	1,89352054	0,20120026	0,50048266	CHST9	carbohydrate sulfotransferase 9 [Source:HGNC Symbol;Acc:HGNC:19898]
ENSG00000143947	-0,2238351	10,9828276	1,89286027	0,20127202	0,50057592	RPS27A	ribosomal protein S27a [Source:HGNC Symbol;Acc:HGNC:10417]
ENSG00000034533	0,28190666	2,78053596	1,89136651	0,20143449	0,50089472	ASTE1	asteroid homolog 1 [Source:HGNC Symbol;Acc:HGNC:25021]
ENSG00000131503	0,3272938	0,84795091	1,89016571	0,20156522	0,5011345	ANKHD1	ankyrin repeat and KH domain containing 1 [Source:HGNC Symbol;Acc:HGNC:24714]
ENSG00000143442	0,2349675	4,20115236	1,88967057	0,20161916	0,50118332	POGZ	pogo transposable element derived with ZNF domain [Source:HGNC Symbol;Acc:HGNC:18801]
ENSG00000185614	0,97792064	-1,5943931	1,88677832	0,20193463	0,50179673	INKA1	inka box actin regulator 1 [Source:HGNC Symbol;Acc:HGNC:32480]
ENSG00000282886	-0,4649775	-0,3699276	1,88691832	0,20191934	0,50179673	PTMAP12	prothymosin alpha pseudogene 12 [Source:HGNC Symbol;Acc:HGNC:53982]
ENSG00000130347	-0,2322632	4,05123931	1,88596752	0,20202318	0,5018591	RTN4IP1	reticulon 4 interacting protein 1 [Source:HGNC Symbol;Acc:HGNC:18647]
ENSG00000143952	0,22116784	5,68853228	1,88552272	0,20207178	0,5018591	VPS54	VPS54 subunit of GARP complex [Source:HGNC Symbol;Acc:HGNC:18652]
ENSG00000145244	-0,7174953	-1,528876	1,88497649	0,20213149	0,5018591	CORIN	corin, serine peptidase [Source:HGNC Symbol;Acc:HGNC:19012]
ENSG00000235036	0,7740065	-1,207541	1,88516909	0,20211043	0,5018591	AL035456.1	pseudogene similar to DNAJ protein
ENSG00000254901	-0,3069306	2,6972696	1,88550467	0,20207376	0,5018591	BORCS8	BLOC-1 related complex subunit 8 [Source:HGNC Symbol;Acc:HGNC:37247]
ENSG00000122958	0,19318767	5,97686503	1,88261101	0,20239033	0,50207511	VPS26A	VPS26, retromer complex component A [Source:HGNC Symbol;Acc:HGNC:12711]
ENSG00000183340	-0,2739628	1,66985282	1,88274216	0,20237597	0,50207511	JRKL	JRK like [Source:HGNC Symbol;Acc:HGNC:6200]
ENSG00000206172	-0,7931417	-1,2291957	1,88331923	0,20231279	0,50207511	HBA2	hemoglobin subunit alpha 2 [Source:HGNC Symbol;Acc:HGNC:4824]
ENSG00000249042	0,39876155	1,29451996	1,88287724	0,20236117	0,50207511	AC008771.1	novel transcript
ENSG00000273319	-0,8961832	-1,2250262	1,88285964	0,2023631	0,50207511	AC058791.1	novel transcript
ENSG00000113790	0,23809561	4,59568196	1,87974272	0,20270478	0,50226618	EHHADH	enoyl-CoA hydratase and 3-hydroxyacyl CoA dehydrogenase [Source:HGNC Symbol;Acc:HGNC:3247]
ENSG00000122643	0,208662	4,32990042	1,87981352	0,20269701	0,50226618	NT5C3A	5'-nucleotidase, cytosolic IIIA [Source:HGNC Symbol;Acc:HGNC:17820]
ENSG00000131788	-0,3383341	3,40886685	1,88128791	0,2025353	0,50226618	PIAS3	protein inhibitor of activated STAT 3 [Source:HGNC Symbol;Acc:HGNC:16861]
ENSG00000136875	0,20308386	6,43692094	1,88038881	0,20263389	0,50226618	PRPF4	pre-mRNA processing factor 4 [Source:HGNC Symbol;Acc:HGNC:17349]
ENSG00000175756	-0,1829708	7,37304646	1,87971328	0,20270801	0,50226618	AURKAIP1	aurora kinase A interacting protein 1 [Source:HGNC Symbol;Acc:HGNC:24114]
ENSG00000177954	-0,2448753	9,64051787	1,88106675	0,20255955	0,50226618	RPS27	ribosomal protein S27 [Source:HGNC Symbol;Acc:HGNC:10416]

ENSG00000180257	0,26058299	2,19164488	1,88088695	0,20257926	0,50226618	ZNF816	zinc finger protein 816 [Source:HGNC Symbol;Acc:HGNC:26995]
ENSG00000100056	0,20807928	4,54987287	1,87874582	0,20281423	0,50239567	ESS2	ess-2 splicing factor homolog [Source:HGNC Symbol;Acc:HGNC:16817]
ENSG00000176542	0,66832888	0,49420922	1,87861081	0,20282906	0,50239567	USF3	upstream transcription factor family member 3 [Source:HGNC Symbol;Acc:HGNC:30494]
ENSG00000138085	-0,2083409	7,91534081	1,87696369	0,20301008	0,50275881	ATRAID	all-trans retinoic acid induced differentiation factor [Source:HGNC Symbol;Acc:HGNC:24090]
ENSG00000221983	-0,2104783	9,68559974	1,87648295	0,20306295	0,50280452	UBA52	ubiquitin A-52 residue ribosomal protein fusion product 1 [Source:HGNC Symbol;Acc:HGNC:12458]
ENSG00000087448	-0,2404318	3,47716345	1,87166969	0,20359337	0,50284431	KLHL42	kelch like family member 42 [Source:HGNC Symbol;Acc:HGNC:29252]
ENSG00000100109	0,25440622	4,71579206	1,87163258	0,20359746	0,50284431	TFIP11	tuftelin interacting protein 11 [Source:HGNC Symbol;Acc:HGNC:17165]
ENSG00000109929	0,22294822	6,34070652	1,87243565	0,20350883	0,50284431	SC5D	sterol-C5-desaturase [Source:HGNC Symbol;Acc:HGNC:10547]
ENSG00000115561	0,24681679	4,19194966	1,87425527	0,20330821	0,50284431	CHMP3	charged multivesicular body protein 3 [Source:HGNC Symbol;Acc:HGNC:29865]
ENSG00000119673	0,24397206	5,80604376	1,8735437	0,20338663	0,50284431	ACOT2	acyl-CoA thioesterase 2 [Source:HGNC Symbol;Acc:HGNC:18431]
ENSG00000123213	0,22441581	6,05770473	1,87471241	0,20325785	0,50284431	NLN	neurolysin [Source:HGNC Symbol;Acc:HGNC:16058]
ENSG00000129028	-0,682694	-0,8595097	1,87430308	0,20330294	0,50284431	THAP10	THAP domain containing 10 [Source:HGNC Symbol;Acc:HGNC:23193]
ENSG00000129197	-0,1816771	5,78695279	1,87207676	0,20354844	0,50284431	RPAIN	RPA interacting protein [Source:HGNC Symbol;Acc:HGNC:28641]
ENSG00000132603	-0,1815989	6,86188385	1,8710285	0,20366416	0,50284431	NIP7	nucleolar pre-rRNA processing protein NIP7 [Source:HGNC Symbol;Acc:HGNC:24328]
ENSG00000148935	0,23119357	4,1937564	1,87505692	0,2032199	0,50284431	GAS2	growth arrest specific 2 [Source:HGNC Symbol;Acc:HGNC:4167]
ENSG00000149089	0,22653572	4,92975059	1,87119827	0,20364542	0,50284431	APIP	APAF1 interacting protein [Source:HGNC Symbol;Acc:HGNC:17581]
ENSG00000152147	-0,2117538	5,55139804	1,87299923	0,20344667	0,50284431	GEMIN6	gem nuclear organelle associated protein 6 [Source:HGNC Symbol;Acc:HGNC:20044]
ENSG00000215089	-0,5598382	-0,7191065	1,87358353	0,20338224	0,50284431	KRT18P11	keratin 18 pseudogene 11 [Source:HGNC Symbol;Acc:HGNC:6431]
ENSG00000227361	-0,5714383	-1,2712282	1,87229458	0,2035244	0,50284431	RPS24P7	ribosomal protein S24 pseudogene 7 [Source:HGNC Symbol;Acc:HGNC:35737]
ENSG00000234284	0,33417558	0,71121079	1,87124139	0,20364065	0,50284431	ZNF879	zinc finger protein 879 [Source:HGNC Symbol;Acc:HGNC:37273]
ENSG00000239857	0,49443141	0,38723546	1,87172979	0,20358673	0,50284431	GET4	guided entry of tail-anchored proteins factor 4 [Source:HGNC Symbol;Acc:HGNC:21690]
ENSG00000260549	-0,5564687	-0,8106811	1,87271919	0,20347755	0,50284431	MT1L	metallothionein 1L, pseudogene [Source:HGNC Symbol;Acc:HGNC:7404]
ENSG00000106290	0,18654825	5,78718381	1,86980736	0,20379909	0,50309242	TAF6	TATA-box binding protein associated factor 6 [Source:HGNC Symbol;Acc:HGNC:11540]
ENSG00000096401	0,2673869	6,19064068	1,86870464	0,20392104	0,50313839	CDC5L	cell division cycle 5 like [Source:HGNC Symbol;Acc:HGNC:1743]
ENSG00000109790	-0,1899492	4,96988303	1,86905502	0,20388228	0,50313839	KLHL5	kelch like family member 5 [Source:HGNC Symbol;Acc:HGNC:6356]
ENSG00000143013	-0,2172574	4,18980025	1,86877176	0,20391361	0,50313839	LMO4	LIM domain only 4 [Source:HGNC Symbol;Acc:HGNC:6644]
ENSG00000204264	0,18365591	5,64774273	1,86803727	0,20399489	0,50323561	PSMB8	proteasome 20S subunit beta 8 [Source:HGNC Symbol;Acc:HGNC:9545]
ENSG00000256537	-0,2897429	4,44823376	1,86770523	0,20403164	0,50324131	SMIM10L1	small integral membrane protein 10 like 1 [Source:HGNC Symbol;Acc:HGNC:49847]
ENSG00000159840	-0,2313554	7,60033503	1,86704682	0,20410455	0,50325121	ZYX	zyxin [Source:HGNC Symbol;Acc:HGNC:13200]
ENSG00000185666	0,33803816	0,82081535	1,86729144	0,20407746	0,50325121	SYN3	synapsin III [Source:HGNC Symbol;Acc:HGNC:11496]
ENSG00000109458	-0,2845305	2,11842571	1,86567923	0,20425611	0,50329795	GAB1	GRB2 associated binding protein 1 [Source:HGNC Symbol;Acc:HGNC:4066]
ENSG00000153140	-0,211801	4,70345034	1,86501097	0,20433022	0,50329795	CETN3	centrin 3 [Source:HGNC Symbol;Acc:HGNC:1868]
ENSG00000163961	-0,2442133	3,14841076	1,86580375	0,2042423	0,50329795	RNF168	ring finger protein 168 [Source:HGNC Symbol;Acc:HGNC:26661]
ENSG00000185869	0,7147134	-0,9541994	1,86518264	0,20431118	0,50329795	ZNF829	zinc finger protein 829 [Source:HGNC Symbol;Acc:HGNC:34032]
ENSG00000198276	-0,1788811	6,13776471	1,86552544	0,20427316	0,50329795	UCKL1	uridine-cytidine kinase 1 like 1 [Source:HGNC Symbol;Acc:HGNC:15938]
ENSG00000198888	0,18672488	10,3513397	1,86627473	0,2041901	0,50329795	MT-ND1	mitochondrially encoded NADH:ubiquinone oxidoreductase core subunit 1 [Source:HGNC Symbol;Acc:HGNC:7455
ENSG00000164078	-0,4966347	0,16063652	1,86390993	0,2044524	0,50351402	MST1R	macrophage stimulating 1 receptor [Source:HGNC Symbol;Acc:HGNC:7381]
ENSG00000164040	0,28424011	6,11909728	1,86289144	0,20456552	0,50362279	PGRMC2	progesterone receptor membrane component 2 [Source:HGNC Symbol;Acc:HGNC:16089]
ENSG00000185133	-0,6364454	-1,3905013	1,86304377	0,20454859	0,50362279	INPP5J	inositol polyphosphate-5-phosphatase J [Source:HGNC Symbol;Acc:HGNC:8956]
ENSG00000156232	0,21619732	3,42016247	1,86231367	0,20462972	0,5036695	WHAMM	WASP homolog associated with actin, golgi membranes and microtubules [Source:HGNC Symbol;Acc:HGNC:3049[illegible]
ENSG00000167112	0,23212976	6,13094318	1,86210026	0,20465344	0,5036695	TRUB2	TruB pseudouridine synthase family member 2 [Source:HGNC Symbol;Acc:HGNC:17170]
ENSG00000247011	-0,9423069	-1,518678	1,86092947	0,20478365	0,50382019	AC005920.1	novel transcript
ENSG00000260236	-0,5326405	-0,2024432	1,86105914	0,20476922	0,50382019	AC099778.1	novel transcript, antisense to PTPN23
ENSG00000107566	0,18914418	6,97371906	1,85954093	0,20493821	0,5040307	ERLIN1	ER lipid raft associated 1 [Source:HGNC Symbol;Acc:HGNC:16947]

ENSG00000115364	0,18172632	7,29666207	1,85960187	0,20493143	0,5040307	MRPL19	mitochondrial ribosomal protein L19 [Source:HGNC Symbol;Acc:HGNC:14052]
ENSG00000144713	-0,1904047	10,3157507	1,85867259	0,20503495	0,50416031	RPL32	ribosomal protein L32 [Source:HGNC Symbol;Acc:HGNC:10336]
ENSG00000237214	-0,3127766	1,42991119	1,85844847	0,20505993	0,50416031	AL080243.2	ribosomal protein S9 (RPS9) pseudogene
ENSG00000111737	0,18727565	5,3126821	1,8577506	0,20513774	0,50426674	RAB35	RAB35, member RAS oncogene family [Source:HGNC Symbol;Acc:HGNC:9774]
ENSG00000090382	-0,2125623	11,7758227	1,85483784	0,20546291	0,50448241	LYZ	lysozyme [Source:HGNC Symbol;Acc:HGNC:6740]
ENSG00000104853	0,2129628	6,89168825	1,85481166	0,20546583	0,50448241	CLPTM1	CLPTM1 regulator of GABA type A receptor forward trafficking [Source:HGNC Symbol;Acc:HGNC:2087]
ENSG00000158042	-0,1773793	6,82277097	1,85560269	0,20537746	0,50448241	MRPL17	mitochondrial ribosomal protein L17 [Source:HGNC Symbol;Acc:HGNC:14053]
ENSG00000176845	-0,4305148	1,1068775	1,85602516	0,20533028	0,50448241	METRNL	meteorin like, glial cell differentiation regulator [Source:HGNC Symbol;Acc:HGNC:27584]
ENSG00000186395	-0,2996838	3,89768978	1,85598556	0,2053347	0,50448241	KRT10	keratin 10 [Source:HGNC Symbol;Acc:HGNC:6413]
ENSG00000214485	-0,2977095	1,41945368	1,85635541	0,20529341	0,50448241	RPL7P1	ribosomal protein L7 pseudogene 1 [Source:HGNC Symbol;Acc:HGNC:10367]
ENSG00000244067	-0,2940246	2,09302245	1,85479943	0,2054672	0,50448241	GSTA2	glutathione S-transferase alpha 2 [Source:HGNC Symbol;Acc:HGNC:4627]
ENSG00000101577	-0,2348698	5,45535718	1,85289324	0,20568038	0,50458182	LPIN2	lipin 2 [Source:HGNC Symbol;Acc:HGNC:14450]
ENSG00000103707	0,21548512	3,64608384	1,8533662	0,20562746	0,50458182	MTFMT	mitochondrial methionyl-tRNA formyltransferase [Source:HGNC Symbol;Acc:HGNC:29666]
ENSG00000157349	0,19543365	4,79049717	1,85377007	0,20558228	0,50458182	DDX19B	DEAD-box helicase 19B [Source:HGNC Symbol;Acc:HGNC:2742]
ENSG00000170310	-0,2455001	4,91460397	1,85299548	0,20566894	0,50458182	STX8	syntaxin 8 [Source:HGNC Symbol;Acc:HGNC:11443]
ENSG00000214530	-0,2362923	7,62967149	1,85354	0,20560802	0,50458182	STARD10	StAR related lipid transfer domain containing 10 [Source:HGNC Symbol;Acc:HGNC:10666]
ENSG00000259556	0,83599566	-1,732212	1,85202104	0,20577803	0,5047366	AC090971.3	Leo1, Paf1/RNA polymerase II complex component, homolog (S. cerevisiae) (LEO1) pseudogene
ENSG00000111266	0,18947167	5,58807527	1,8514585	0,20584103	0,50480639	DUSP16	dual specificity phosphatase 16 [Source:HGNC Symbol;Acc:HGNC:17909]
ENSG00000168569	-0,2016237	5,03966206	1,85096452	0,20589639	0,50485739	TMEM223	transmembrane protein 223 [Source:HGNC Symbol;Acc:HGNC:28464]
ENSG00000162772	-0,2602071	3,38898913	1,84956143	0,20605372	0,50500728	ATF3	activating transcription factor 3 [Source:HGNC Symbol;Acc:HGNC:785]
ENSG00000165724	0,22017774	5,15428556	1,85002427	0,2060018	0,50500728	ZMYND19	zinc finger MYND-type containing 19 [Source:HGNC Symbol;Acc:HGNC:21146]
ENSG00000225125	-0,7818278	-1,2191962	1,84949452	0,20606122	0,50500728	RANP4	RAN pseudogene 4 [Source:HGNC Symbol;Acc:HGNC:39859]
ENSG00000055130	0,18667777	6,07743005	1,84631326	0,20641855	0,50547922	CUL1	cullin 1 [Source:HGNC Symbol;Acc:HGNC:2551]
ENSG00000076826	0,23813257	5,01886374	1,84559982	0,20649881	0,50547922	CAMSAP3	calmodulin regulated spectrin associated protein family member 3 [Source:HGNC Symbol;Acc:HGNC:29307]
ENSG00000156050	-0,3674055	0,88646689	1,84664979	0,20638071	0,50547922	FAM161B	FAM161 centrosomal protein B [Source:HGNC Symbol;Acc:HGNC:19854]
ENSG00000162645	-0,4235098	1,21113203	1,84531731	0,20653059	0,50547922	GBP2	guanylate binding protein 2 [Source:HGNC Symbol;Acc:HGNC:4183]
ENSG00000166024	0,20767939	4,0042316	1,84593874	0,20646068	0,50547922	R3HCC1L	R3H domain and coiled-coil containing 1 like [Source:HGNC Symbol;Acc:HGNC:23512]
ENSG00000172172	0,20066586	7,03103979	1,84696335	0,20634547	0,50547922	MRPL13	mitochondrial ribosomal protein L13 [Source:HGNC Symbol;Acc:HGNC:14278]
ENSG00000178974	0,21237441	4,01666584	1,84667371	0,20637802	0,50547922	FBXO34	F-box protein 34 [Source:HGNC Symbol;Acc:HGNC:20201]
ENSG00000196267	0,509441	-0,4731015	1,84576584	0,20648013	0,50547922	ZNF836	zinc finger protein 836 [Source:HGNC Symbol;Acc:HGNC:34333]
ENSG00000148634	0,22421522	3,6547845	1,84453458	0,20661871	0,50552549	HERC4	HECT and RLD domain containing E3 ubiquitin protein ligase 4 [Source:HGNC Symbol;Acc:HGNC:24521]
ENSG00000153113	-0,1976289	6,46977147	1,84478326	0,20659071	0,50552549	CAST	calpastatin [Source:HGNC Symbol;Acc:HGNC:1515]
ENSG00000232341	-0,5260551	-0,831335	1,84314154	0,20677564	0,50574006	RPL4P2	ribosomal protein L4 pseudogene 2 [Source:HGNC Symbol;Acc:HGNC:36836]
ENSG00000278828	0,39387555	1,31049465	1,84320674	0,2067683	0,50574006	H3C12	H3 clustered histone 12 [Source:HGNC Symbol;Acc:HGNC:4774]
ENSG00000138080	-0,3529247	4,00913349	1,84245094	0,20685351	0,50576115	EMILIN1	elastin microfibril interfacer 1 [Source:HGNC Symbol;Acc:HGNC:19880]
ENSG00000214293	-0,2449089	2,8799943	1,84255516	0,20684175	0,50576115	APTR	Alu-mediated CDKN1A/p21 transcriptional regulator [Source:HGNC Symbol;Acc:HGNC:44173]
ENSG00000248508	-0,5655613	-1,1795473	1,84190338	0,20691527	0,50582751	SRP14-AS1	SRP14 antisense RNA1 (head to head) [Source:HGNC Symbol;Acc:HGNC:48619]
ENSG00000089195	-0,2041264	5,46246666	1,84052133	0,20707127	0,50612417	TRMT6	tRNA methyltransferase 6 [Source:HGNC Symbol;Acc:HGNC:20900]
ENSG00000151835	0,27599306	2,85241989	1,84019191	0,20710848	0,50613043	SACS	sacsin molecular chaperone [Source:HGNC Symbol;Acc:HGNC:10519]
ENSG00000184924	-0,2637996	4,74051586	1,83762919	0,20739824	0,50667101	PTRHD1	peptidyl-tRNA hydrolase domain containing 1 [Source:HGNC Symbol;Acc:HGNC:33782]
ENSG00000258359	1,10484816	-1,6105382	1,83762206	0,20739904	0,50667101	PCNPP1	PEST containing nuclear protein pseudogene 1 [Source:HGNC Symbol;Acc:HGNC:32440]
ENSG00000076864	-0,2108246	4,57041853	1,83705378	0,20746337	0,50674342	RAP1GAP	RAP1 GTPase activating protein [Source:HGNC Symbol;Acc:HGNC:9858]
ENSG00000120709	0,22985348	3,88585535	1,83659527	0,20751529	0,50678551	FAM53C	family with sequence similarity 53 member C [Source:HGNC Symbol;Acc:HGNC:1336]
ENSG00000008952	0,20998799	6,46648055	1,83519618	0,20767383	0,50681681	SEC62	SEC62 homolog, preprotein translocation factor [Source:HGNC Symbol;Acc:HGNC:11846]

ENSG00000135677	0,2967373	6,76452256	1,83564978	0,20762242	0,50681681	GNS	glucosamine (N-acetyl)-6-sulfatase [Source:HGNC Symbol;Acc:HGNC:4422]
ENSG00000196757	0,25579429	2,84319642	1,83524736	0,20766803	0,50681681	ZNF700	zinc finger protein 700 [Source:HGNC Symbol;Acc:HGNC:25292]
ENSG00000242262	-0,2814973	1,28276627	1,83495156	0,20770157	0,50681681	AC092597.1	ribosomal protein L15 (RPL15) pseudogene
ENSG00000286522	0,65193635	-1,2241273	1,83609446	0,20757203	0,50681681	H3C2	H3 clustered histone 2 [Source:HGNC Symbol;Acc:HGNC:4776]
ENSG00000172890	-0,2023214	5,07020224	1,83441713	0,20776219	0,50685666	NADSYN1	NAD synthetase 1 [Source:HGNC Symbol;Acc:HGNC:29832]
ENSG00000176700	0,26541957	2,23358527	1,83419585	0,20778729	0,50685666	SCAND2P	SCAN domain containing 2 pseudogene [Source:HGNC Symbol;Acc:HGNC:10567]
ENSG00000162695	-0,2388357	3,89408768	1,83357421	0,20785784	0,5069441	SLC30A7	solute carrier family 30 member 7 [Source:HGNC Symbol;Acc:HGNC:19306]
ENSG00000091157	0,38592216	2,01250908	1,83155414	0,20808731	0,50708824	WDR7	WD repeat domain 7 [Source:HGNC Symbol;Acc:HGNC:13490]
ENSG00000132635	-0,2246404	3,63916566	1,83152613	0,20809049	0,50708824	PCED1A	PC-esterase domain containing 1A [Source:HGNC Symbol;Acc:HGNC:16212]
ENSG00000165699	0,19690851	4,34331748	1,83241712	0,20798924	0,50708824	TSC1	TSC complex subunit 1 [Source:HGNC Symbol;Acc:HGNC:12362]
ENSG00000221990	-0,3869366	1,75016113	1,8317586	0,20806407	0,50708824	EXOC3-AS1	EXOC3 antisense RNA 1 [Source:HGNC Symbol;Acc:HGNC:25175]
ENSG00000235029	-0,7937048	-1,1585871	1,83268486	0,20795882	0,50708824	MNX1-AS2	MNX1 antisense RNA 2 [Source:HGNC Symbol;Acc:HGNC:40278]
ENSG00000227992	-0,8931403	-2,106673	1,83023607	0,20823722	0,50736116	AC108463.1	pseudogene similar to part of ubiquitin carboxyl-terminal hydrolase L5 UCHL5
ENSG00000138463	0,22387274	4,69643615	1,82912673	0,2083635	0,50758419	SLC49A4	solute carrier family 49 member 4 [Source:HGNC Symbol;Acc:HGNC:16628]
ENSG00000114737	0,30532413	1,99244645	1,8271079	0,20859358	0,50780598	CISH	cytokine inducible SH2 containing protein [Source:HGNC Symbol;Acc:HGNC:1984]
ENSG00000173950	0,18251529	5,83152056	1,82727015	0,20857508	0,50780598	XXYLT1	xyloside xylosyltransferase 1 [Source:HGNC Symbol;Acc:HGNC:26639]
ENSG00000204642	-0,2295379	2,72483625	1,82796396	0,20849598	0,50780598	HLA-F	major histocompatibility complex, class I, F [Source:HGNC Symbol;Acc:HGNC:4963]
ENSG00000226549	-0,5577643	-0,7936557	1,82713006	0,20859106	0,50780598	SCDP1	stearoyl-CoA desaturase pseudogene 1 [Source:HGNC Symbol;Acc:HGNC:10572]
ENSG00000136937	0,21417359	5,67712374	1,82572961	0,20875086	0,50810419	NCBP1	nuclear cap binding protein subunit 1 [Source:HGNC Symbol;Acc:HGNC:7658]
ENSG00000127561	-0,3031695	0,96843181	1,82307389	0,20905436	0,50855568	SYNGR3	synaptogyrin 3 [Source:HGNC Symbol;Acc:HGNC:11501]
ENSG00000133641	0,20118456	4,15682636	1,82288821	0,2090756	0,50855568	C12orf29	chromosome 12 open reading frame 29 [Source:HGNC Symbol;Acc:HGNC:25322]
ENSG00000136213	0,19054253	4,68861642	1,82298359	0,20906469	0,50855568	CHST12	carbohydrate sulfotransferase 12 [Source:HGNC Symbol;Acc:HGNC:17423]
ENSG00000196372	-0,2063042	3,74042233	1,82323495	0,20903593	0,50855568	ASB13	ankyrin repeat and SOCS box containing 13 [Source:HGNC Symbol;Acc:HGNC:19765]
ENSG00000006530	0,26031901	5,28912608	1,82212624	0,2091628	0,50865258	AGK	acylglycerol kinase [Source:HGNC Symbol;Acc:HGNC:21869]
ENSG00000105298	-0,2646206	4,71806085	1,82140395	0,2092455	0,50865258	CACTIN	cactin, spliceosome C complex subunit [Source:HGNC Symbol;Acc:HGNC:29938]
ENSG00000204371	-0,2789604	6,08224765	1,82120728	0,20926803	0,50865258	EHMT2	euchromatic histone lysine methyltransferase 2 [Source:HGNC Symbol;Acc:HGNC:14129]
ENSG00000234742	-0,3679036	0,85783897	1,82101963	0,20928953	0,50865258	AC144530.1	ribosomal protein L17 (RPL17) pseudogene
ENSG00000286116	-0,4129287	1,01361535	1,82141749	0,20924395	0,50865258	AL157394.3	FAS antisense RNA 1
ENSG00000172530	-0,2144399	3,861637	1,82067712	0,20932877	0,50866334	BANP	BTG3 associated nuclear protein [Source:HGNC Symbol;Acc:HGNC:13450]
ENSG00000164919	-0,2065966	7,49293376	1,81988442	0,20941964	0,50879951	COX6C	cytochrome c oxidase subunit 6C [Source:HGNC Symbol;Acc:HGNC:2285]
ENSG00000237522	-0,7506458	-1,1507653	1,81928651	0,20948821	0,50888148	NONOP2	non-POU domain containing, octamer-binding pseudogene 2 [Source:HGNC Symbol;Acc:HGNC:42032]
ENSG00000102109	0,20805808	4,51395725	1,81821727	0,20961091	0,5090949	PCSK1N	proprotein convertase subtilisin/kexin type 1 inhibitor [Source:HGNC Symbol;Acc:HGNC:17301]
ENSG00000164172	-0,2063984	5,67421145	1,81592948	0,20987378	0,5096486	MOCS2	molybdenum cofactor synthesis 2 [Source:HGNC Symbol;Acc:HGNC:7193]
ENSG00000071889	-0,2556168	4,4359003	1,81402462	0,21009298	0,50996932	FAM3A	FAM3 metabolism regulating signaling molecule A [Source:HGNC Symbol;Acc:HGNC:13749]
ENSG00000172638	0,22375917	4,26623563	1,81387191	0,21011057	0,50996932	EFEMP2	EGF containing fibulin extracellular matrix protein 2 [Source:HGNC Symbol;Acc:HGNC:3219]
ENSG00000175329	0,40130796	1,1573325	1,81439889	0,21004989	0,50996932	ISX	intestine specific homeobox [Source:HGNC Symbol;Acc:HGNC:28084]
ENSG00000259343	-0,8138072	-1,2485796	1,81298427	0,21021283	0,51013277	TMC3-AS1	TMC3 antisense RNA 1 [Source:HGNC Symbol;Acc:HGNC:51424]
ENSG00000105339	-0,4779479	0,29661519	1,81099112	0,21044271	0,51018257	DENND3	DENN domain containing 3 [Source:HGNC Symbol;Acc:HGNC:29134]
ENSG00000113048	0,19671255	6,82966086	1,81157502	0,21037533	0,51018257	MRPS27	mitochondrial ribosomal protein S27 [Source:HGNC Symbol;Acc:HGNC:14512]
ENSG00000115661	-0,3085341	3,77131909	1,8109023	0,21045296	0,51018257	STK16	serine/threonine kinase 16 [Source:HGNC Symbol;Acc:HGNC:11394]
ENSG00000125510	-0,6535746	-1,6480116	1,81044364	0,21050591	0,51018257	OPRL1	opioid related nociceptin receptor 1 [Source:HGNC Symbol;Acc:HGNC:8155]
ENSG00000164050	0,21718681	6,32428725	1,81038454	0,21051273	0,51018257	PLXNB1	plexin B1 [Source:HGNC Symbol;Acc:HGNC:9103]
ENSG00000185420	0,20612427	4,45830448	1,81244339	0,21027518	0,51018257	SMYD3	SET and MYND domain containing 3 [Source:HGNC Symbol;Acc:HGNC:15513]
ENSG00000256210	-0,7777588	-1,7423562	1,8114234	0,21039282	0,51018257	AC005255.1	transmembrane protein 167A (TMEM167A) pseudogene

ENSG00000260963	-0,280349	1,51842176	1,81087688	0,21045589	0,51018257	AC026462.3	novel transcript
ENSG00000091136	-0,4084865	8,04946748	1,81008141	0,21054774	0,51018277	LAMB1	laminin subunit beta 1 [Source:HGNC Symbol;Acc:HGNC:6486]
ENSG00000153147	0,22877777	6,14863798	1,80890531	0,21068363	0,51035707	SMARCA5	SWI/SNF related, matrix associated, actin dependent regulator of chromatin, subfamily a, member 5 [Source:HGN
ENSG00000213791	-0,74663	-0,6812678	1,80885419	0,21068954	0,51035707	AC104212.2	ribosomal protein S5 (RPS5) pseudogene
ENSG00000090339	-0,3392551	7,48164789	1,80848601	0,21073211	0,51037556	ICAM1	intercellular adhesion molecule 1 [Source:HGNC Symbol;Acc:HGNC:5344]
ENSG00000154719	0,18029133	6,09179772	1,80730914	0,21086825	0,51040172	MRPL39	mitochondrial ribosomal protein L39 [Source:HGNC Symbol;Acc:HGNC:14027]
ENSG00000161791	0,47234371	-0,2483236	1,80718464	0,21088266	0,51040172	FMNL3	formin like 3 [Source:HGNC Symbol;Acc:HGNC:23698]
ENSG00000180448	-0,3432696	1,13662718	1,80764326	0,21082959	0,51040172	ARHGAP45	Rho GTPase activating protein 45 [Source:HGNC Symbol;Acc:HGNC:17102]
ENSG00000186815	0,20143661	4,50938652	1,80747236	0,21084936	0,51040172	TPCN1	two pore segment channel 1 [Source:HGNC Symbol;Acc:HGNC:18182]
ENSG00000183751	-0,3107349	5,42273285	1,80602368	0,21101709	0,51061513	TBL3	transducin beta like 3 [Source:HGNC Symbol;Acc:HGNC:11587]
ENSG00000242208	-0,7809522	-1,4210262	1,80581953	0,21104074	0,51061513	RPL5P29	ribosomal protein L5 pseudogene 29 [Source:HGNC Symbol;Acc:HGNC:36335]
ENSG00000095485	0,18971003	4,86186264	1,80350794	0,21130879	0,51090433	CWF19L1	CWF19 like cell cycle control factor 1 [Source:HGNC Symbol;Acc:HGNC:25613]
ENSG00000102048	-0,3721456	2,49777104	1,80328102	0,21133513	0,51090433	ASB9	ankyrin repeat and SOCS box containing 9 [Source:HGNC Symbol;Acc:HGNC:17184]
ENSG00000140299	0,2390231	4,90953063	1,80374779	0,21128095	0,51090433	BNIP2	BCL2 interacting protein 2 [Source:HGNC Symbol;Acc:HGNC:1083]
ENSG00000150787	-0,2072842	4,23562755	1,80369237	0,21128739	0,51090433	PTS	6-pyruvoyltetrahydropterin synthase [Source:HGNC Symbol;Acc:HGNC:9689]
ENSG00000171475	0,22114961	3,91705274	1,80379783	0,21127515	0,51090433	WIPF2	WAS/WASL interacting protein family member 2 [Source:HGNC Symbol;Acc:HGNC:30923]
ENSG00000168806	-0,2531301	3,45915691	1,8018924	0,2114964	0,51120961	LCMT2	leucine carboxyl methyltransferase 2 [Source:HGNC Symbol;Acc:HGNC:17558]
ENSG00000116151	-0,3091919	1,58742769	1,8014692	0,21154558	0,5112439	MORN1	MORN repeat containing 1 [Source:HGNC Symbol;Acc:HGNC:25852]
ENSG00000106683	-0,2060076	4,96001649	1,79906121	0,21182572	0,51124433	LIMK1	LIM domain kinase 1 [Source:HGNC Symbol;Acc:HGNC:6613]
ENSG00000128581	0,27533862	3,48779745	1,80015077	0,2116989	0,51124433	IFT22	intraflagellar transport 22 [Source:HGNC Symbol;Acc:HGNC:21895]
ENSG00000134283	-0,1914213	5,76216082	1,79955302	0,21176846	0,51124433	PPHLN1	periphilin 1 [Source:HGNC Symbol;Acc:HGNC:19369]
ENSG00000140398	0,34462713	1,09474651	1,79981716	0,21173772	0,51124433	NEIL1	nei like DNA glycosylase 1 [Source:HGNC Symbol;Acc:HGNC:18448]
ENSG00000147533	0,20697491	5,41772624	1,7998207	0,21173731	0,51124433	GOLGA7	golgin A7 [Source:HGNC Symbol;Acc:HGNC:24876]
ENSG00000211460	0,18489321	7,18788395	1,80024427	0,21168802	0,51124433	TSN	translin [Source:HGNC Symbol;Acc:HGNC:12379]
ENSG00000280594	-0,8648566	-2,0207663	1,79944037	0,21178158	0,51124433	BTG3-AS1	BTG3 antisense RNA 1 [Source:HGNC Symbol;Acc:HGNC:53145]
ENSG00000286162	1,25923488	-1,74795	1,79918814	0,21181094	0,51124433	AL162253.2	novel transcript
ENSG00000105135	-0,1836093	7,07819977	1,79726869	0,21203458	0,51153824	ILVBL	ilvB acetolactate synthase like [Source:HGNC Symbol;Acc:HGNC:6041]
ENSG00000118420	0,23236254	3,2684964	1,79558201	0,21223136	0,51153824	UBE3D	ubiquitin protein ligase E3D [Source:HGNC Symbol;Acc:HGNC:21381]
ENSG00000121104	0,20400346	4,66459041	1,79628997	0,21214874	0,51153824	FAM117A	family with sequence similarity 117 member A [Source:HGNC Symbol;Acc:HGNC:24179]
ENSG00000179292	0,77500546	-1,2071122	1,79602863	0,21217923	0,51153824	TMEM151A	transmembrane protein 151A [Source:HGNC Symbol;Acc:HGNC:28497]
ENSG00000181264	-0,2336953	2,66003637	1,79531421	0,21226263	0,51153824	TLCD5	TLC domain containing 5 [Source:HGNC Symbol;Acc:HGNC:28280]
ENSG00000212789	0,67488798	-0,5080741	1,79568917	0,21221885	0,51153824	ST13P5	ST13, Hsp70 interacting protein pseudogene 5 [Source:HGNC Symbol;Acc:HGNC:18556]
ENSG00000226268	-0,4207779	0,10657849	1,79659167	0,21211354	0,51153824	AC135977.1	solute carrier family 25, member 33 (SLC25A33) pseudogene
ENSG00000279457	-0,2831766	1,80470509	1,79625623	0,21215267	0,51153824	WASH9P	WAS protein family homolog 9, pseudogene [Source:HGNC Symbol;Acc:HGNC:53981]
ENSG00000287787	-0,4372337	-0,1168072	1,79552046	0,21223855	0,51153824	AC092275.1	novel transcript, sense intronic to GSE1
ENSG00000134250	0,46088806	1,69396576	1,7948219	0,21232013	0,5115924	NOTCH2	notch receptor 2 [Source:HGNC Symbol;Acc:HGNC:7882]
ENSG00000214012	-0,6693517	-1,1315046	1,79275411	0,21256185	0,51209037	KRT18P38	keratin 18 pseudogene 38 [Source:HGNC Symbol;Acc:HGNC:33407]
ENSG00000115163	0,2586837	4,78510008	1,79186564	0,21266582	0,51225638	CENPA	centromere protein A [Source:HGNC Symbol;Acc:HGNC:1851]
ENSG00000175193	-0,2097554	3,99862022	1,79099892	0,21276732	0,51241637	PARL	presenilin associated rhomboid like [Source:HGNC Symbol;Acc:HGNC:18253]
ENSG00000178146	-0,6570771	-1,0634189	1,79020367	0,2128605	0,51244719	GK4P	glycerol kinase 4 pseudogene [Source:HGNC Symbol;Acc:HGNC:4295]
ENSG00000179454	0,29291286	1,19590693	1,7904794	0,21282818	0,51244719	KLHL28	kelch like family member 28 [Source:HGNC Symbol;Acc:HGNC:19741]
ENSG00000224389	-0,5283055	4,75965628	1,78999173	0,21288534	0,51244719	C4B	complement C4B (Chido blood group) [Source:HGNC Symbol;Acc:HGNC:1324]
ENSG00000130724	-0,19688	5,95036158	1,78810901	0,2131062	0,51280984	CHMP2A	charged multivesicular body protein 2A [Source:HGNC Symbol;Acc:HGNC:30216]
ENSG00000197702	0,18211112	5,60256869	1,78822266	0,21309286	0,51280984	PARVA	parvin alpha [Source:HGNC Symbol;Acc:HGNC:14652]

ENSG00000144560	0,21511568	4,30803365	1,78709919	0,2132248	0,51301071	VGLL4	vestigial like family member 4 [Source:HGNC Symbol;Acc:HGNC:28966]
ENSG00000111300	-0,2296745	4,76276371	1,78661533	0,21328165	0,51302347	NAA25	N-alpha-acetyltransferase 25, NatB auxiliary subunit [Source:HGNC Symbol;Acc:HGNC:25783]
ENSG00000258597	-0,3269412	2,23572671	1,78645635	0,21330033	0,51302347	SERPINA2	serpin family A member 2 (gene/pseudogene) [Source:HGNC Symbol;Acc:HGNC:8985]
ENSG00000182117	-0,216722	7,205143	1,78577214	0,21338077	0,51313247	NOP10	NOP10 ribonucleoprotein [Source:HGNC Symbol;Acc:HGNC:14378]
ENSG00000121644	0,24932117	3,60461564	1,785182	0,21345019	0,51320642	DESI2	desumoylating isopeptidase 2 [Source:HGNC Symbol;Acc:HGNC:24264]
ENSG00000140990	-0,2308261	7,19936171	1,78491343	0,21348179	0,51320642	NDUFB10	NADH:ubiquinone oxidoreductase subunit B10 [Source:HGNC Symbol;Acc:HGNC:7696]
ENSG00000123643	0,34027121	2,15510808	1,78395737	0,21359433	0,51339249	SLC36A1	solute carrier family 36 member 1 [Source:HGNC Symbol;Acc:HGNC:18761]
ENSG00000141569	-0,1967746	4,00697781	1,78293963	0,21371422	0,51351168	TRIM65	tripartite motif containing 65 [Source:HGNC Symbol;Acc:HGNC:27316]
ENSG00000257681	-0,9914119	-1,9421427	1,78298899	0,2137084	0,51351168	AC025265.1	novel transcript, antisense to STAB2
ENSG00000099250	0,32194384	3,87150581	1,78094904	0,21394897	0,51357593	NRP1	neuropilin 1 [Source:HGNC Symbol;Acc:HGNC:8004]
ENSG00000099940	-0,2208873	5,01916873	1,78153952	0,2138793	0,51357593	SNAP29	synaptosome associated protein 29 [Source:HGNC Symbol;Acc:HGNC:11133]
ENSG00000120889	-0,1907529	7,68626449	1,78094943	0,21394893	0,51357593	TNFRSF10B	TNF receptor superfamily member 10b [Source:HGNC Symbol;Acc:HGNC:11905]
ENSG00000132952	0,24830992	3,57511683	1,78062655	0,21398704	0,51357593	USPL1	ubiquitin specific peptidase like 1 [Source:HGNC Symbol;Acc:HGNC:20294]
ENSG00000138413	0,1754537	9,52952186	1,78072165	0,21397581	0,51357593	IDH1	isocitrate dehydrogenase (NADP(+)) 1 [Source:HGNC Symbol;Acc:HGNC:5382]
ENSG00000158526	0,22822979	5,62043392	1,78116971	0,21392293	0,51357593	TSR2	TSR2 ribosome maturation factor [Source:HGNC Symbol;Acc:HGNC:25455]
ENSG00000198168	-0,2228209	3,79957082	1,78141568	0,21389391	0,51357593	SVIP	small VCP interacting protein [Source:HGNC Symbol;Acc:HGNC:25238]
ENSG00000102078	0,27368924	3,58796455	1,77895562	0,21418441	0,51396519	SLC25A14	solute carrier family 25 member 14 [Source:HGNC Symbol;Acc:HGNC:10984]
ENSG00000116574	-0,2141261	4,74644334	1,77834953	0,21425607	0,51399349	RHOU	ras homolog family member U [Source:HGNC Symbol;Acc:HGNC:17794]
ENSG00000188211	0,47443195	4,35768525	1,7782607	0,21426657	0,51399349	NCR3LG1	natural killer cell cytotoxicity receptor 3 ligand 1 [Source:HGNC Symbol;Acc:HGNC:42400]
ENSG00000016391	0,17270337	6,62041378	1,77638459	0,21448859	0,51407354	CHDH	choline dehydrogenase [Source:HGNC Symbol;Acc:HGNC:24288]
ENSG00000066813	-0,2400335	2,40056825	1,77638993	0,21448796	0,51407354	ACSM2B	acyl-CoA synthetase medium chain family member 2B [Source:HGNC Symbol;Acc:HGNC:30931]
ENSG00000105254	0,1767494	7,24631448	1,77560066	0,21458145	0,51407354	TBCB	tubulin folding cofactor B [Source:HGNC Symbol;Acc:HGNC:1989]
ENSG00000109084	0,22377553	7,41925331	1,77727032	0,21438373	0,51407354	TMEM97	transmembrane protein 97 [Source:HGNC Symbol;Acc:HGNC:28106]
ENSG00000132128	0,23107357	6,57846375	1,77593305	0,21454207	0,51407354	LRRC41	leucine rich repeat containing 41 [Source:HGNC Symbol;Acc:HGNC:16917]
ENSG00000133112	-0,2043763	10,6853739	1,77704801	0,21441004	0,51407354	TPT1	tumor protein, translationally-controlled 1 [Source:HGNC Symbol;Acc:HGNC:12022]
ENSG00000161944	0,18451631	9,98830894	1,77583258	0,21455397	0,51407354	ASGR2	asialoglycoprotein receptor 2 [Source:HGNC Symbol;Acc:HGNC:743]
ENSG00000215840	-0,3945267	0,57744268	1,77760379	0,21434427	0,51407354	AL592295.1	ribosomal protein S2 (RPS2) pseudogene
ENSG00000072958	0,1932027	6,15332033	1,77519641	0,21462936	0,514104	AP1M1	adaptor related protein complex 1 subunit mu 1 [Source:HGNC Symbol;Acc:HGNC:13667]
ENSG00000138356	-0,2806799	3,54653309	1,77286478	0,21490596	0,51468216	AOX1	aldehyde oxidase 1 [Source:HGNC Symbol;Acc:HGNC:553]
ENSG00000100764	0,21719607	6,87648806	1,77228606	0,21497469	0,51476238	PSMC1	proteasome 26S subunit, ATPase 1 [Source:HGNC Symbol;Acc:HGNC:9547]
ENSG00000189057	0,2570055	2,800018	1,7718284	0,21502906	0,51480819	FAM111B	family with sequence similarity 111 member B [Source:HGNC Symbol;Acc:HGNC:24200]
ENSG00000102024	0,26628183	6,69768866	1,77025162	0,21521654	0,5150882	PLS3	plastin 3 [Source:HGNC Symbol;Acc:HGNC:9091]
ENSG00000197483	-0,307816	2,43452486	1,77038907	0,21520018	0,5150882	ZNF628	zinc finger protein 628 [Source:HGNC Symbol;Acc:HGNC:28054]
ENSG00000136490	-0,2209825	3,6789654	1,76869192	0,2154022	0,51536369	LIMD2	LIM domain containing 2 [Source:HGNC Symbol;Acc:HGNC:28142]
ENSG00000273045	0,43112749	0,86087376	1,7689293	0,21537392	0,51536369	C2orf15	chromosome 2 open reading frame 15 [Source:HGNC Symbol;Acc:HGNC:28436]
ENSG00000152700	0,18424362	7,08764169	1,76807986	0,21547511	0,51545372	SAR1B	secretion associated Ras related GTPase 1B [Source:HGNC Symbol;Acc:HGNC:10535]
ENSG00000236155	-0,4607243	1,06407268	1,76740029	0,21555611	0,51556306	ZPLD2P	zona pellucida like domain containing 2, pseudogene [Source:HGNC Symbol;Acc:HGNC:54510]
ENSG00000137218	0,4764757	0,53457757	1,76621254	0,21569778	0,5157829	FRS3	fibroblast growth factor receptor substrate 3 [Source:HGNC Symbol;Acc:HGNC:16970]
ENSG00000174276	-0,2266595	2,29885325	1,76603773	0,21571864	0,5157829	ZNHIT2	zinc finger HIT-type containing 2 [Source:HGNC Symbol;Acc:HGNC:1177]
ENSG00000185033	-0,2508085	4,38246734	1,76439784	0,21591446	0,51616665	SEMA4B	semaphorin 4B [Source:HGNC Symbol;Acc:HGNC:10730]
ENSG00000105398	-0,172037	7,14776041	1,76309485	0,21607023	0,51637003	SULT2A1	sulfotransferase family 2A member 1 [Source:HGNC Symbol;Acc:HGNC:11458]
ENSG00000176171	-0,2343164	6,33423834	1,76318668	0,21605925	0,51637003	BNIP3	BCL2 interacting protein 3 [Source:HGNC Symbol;Acc:HGNC:1084]
ENSG00000178567	0,25756616	3,69558075	1,76225758	0,2161704	0,51637416	EPM2AIP1	EPM2A interacting protein 1 [Source:HGNC Symbol;Acc:HGNC:19735]
ENSG00000197375	0,2152847	3,36426416	1,76219413	0,216178	0,51637416	SLC22A5	solute carrier family 22 member 5 [Source:HGNC Symbol;Acc:HGNC:10969]

ENSG00000198298	-0,3502583	1,30520502	1,7627729	0,21610874	0,51637416	ZNF485	zinc finger protein 485 [Source:HGNC Symbol;Acc:HGNC:23440]
ENSG00000114098	0,23881761	4,57246002	1,76135266	0,21627874	0,51653035	ARMC8	armadillo repeat containing 8 [Source:HGNC Symbol;Acc:HGNC:24999]
ENSG00000101608	-0,1958769	7,71368222	1,76021567	0,21641497	0,51656043	MYL12A	myosin light chain 12A [Source:HGNC Symbol;Acc:HGNC:16701]
ENSG00000114395	-0,2080693	3,69704604	1,76004958	0,21643488	0,51656043	CYB561D2	cytochrome b561 family member D2 [Source:HGNC Symbol;Acc:HGNC:30253]
ENSG00000131051	0,18231742	7,8953274	1,75947731	0,21650349	0,51656043	RBM39	RNA binding motif protein 39 [Source:HGNC Symbol;Acc:HGNC:15923]
ENSG00000144848	0,18831672	5,7391149	1,75976403	0,21646911	0,51656043	ATG3	autophagy related 3 [Source:HGNC Symbol;Acc:HGNC:20962]
ENSG00000171729	-0,2028618	5,29100336	1,7605347	0,21637673	0,51656043	TMEM51	transmembrane protein 51 [Source:HGNC Symbol;Acc:HGNC:25488]
ENSG00000241429	-0,5175292	-0,4173095	1,76091871	0,21633072	0,51656043	EEF1A1P25	eukaryotic translation elongation factor 1 alpha 1 pseudogene 25 [Source:HGNC Symbol;Acc:HGNC:37898]
ENSG00000117174	0,24570902	4,0098058	1,75636901	0,2168767	0,51656486	ZNHIT6	zinc finger HIT-type containing 6 [Source:HGNC Symbol;Acc:HGNC:26089]
ENSG00000135040	0,21018196	5,31859262	1,75622263	0,2168943	0,51656486	NAA35	N-alpha-acetyltransferase 35, NatC auxiliary subunit [Source:HGNC Symbol;Acc:HGNC:24340]
ENSG00000138495	-0,2720782	4,7046623	1,75698663	0,21680248	0,51656486	COX17	cytochrome c oxidase copper chaperone COX17 [Source:HGNC Symbol;Acc:HGNC:2264]
ENSG00000149201	0,66406135	-1,7052302	1,75704143	0,21679589	0,51656486	CCDC81	coiled-coil domain containing 81 [Source:HGNC Symbol;Acc:HGNC:26281]
ENSG00000171298	-0,2848774	7,14012657	1,75814317	0,21666358	0,51656486	GAA	glucosidase alpha, acid [Source:HGNC Symbol;Acc:HGNC:4065]
ENSG00000184992	0,17876229	5,93782199	1,75779355	0,21670555	0,51656486	BRI3BP	BRI3 binding protein [Source:HGNC Symbol;Acc:HGNC:14251]
ENSG00000186073	-0,2329717	2,70889761	1,75768334	0,21671879	0,51656486	CDIN1	CDAN1 interacting nuclease 1 [Source:HGNC Symbol;Acc:HGNC:26929]
ENSG00000187051	-0,1924739	6,60980951	1,7571243	0,21678594	0,51656486	RPS19BP1	ribosomal protein S19 binding protein 1 [Source:HGNC Symbol;Acc:HGNC:28749]
ENSG00000224546	0,33767402	0,91248666	1,75753205	0,21673696	0,51656486	EIF4BP3	eukaryotic translation initiation factor 4B pseudogene 3 [Source:HGNC Symbol;Acc:HGNC:37936]
ENSG00000236552	-0,2018254	4,63945001	1,75872795	0,21659339	0,51656486	RPL13AP5	ribosomal protein L13a pseudogene 5 [Source:HGNC Symbol;Acc:HGNC:23736]
ENSG00000261575	-0,597554	-1,4308043	1,75675312	0,21683054	0,51656486	AC005829.1	C17orf58 pseudogene
ENSG00000243388	-0,7125264	-1,7784027	1,75545922	0,21698611	0,51669927	RPL3P3	ribosomal protein L3 pseudogene 3 [Source:HGNC Symbol;Acc:HGNC:23550]
ENSG00000168092	0,18627837	5,27542355	1,75490955	0,21705224	0,51677252	PAFAH1B2	platelet activating factor acetylhydrolase 1b catalytic subunit 2 [Source:HGNC Symbol;Acc:HGNC:8575]
ENSG00000101986	-0,1827981	5,27747043	1,75397464	0,21716479	0,51695624	ABCD1	ATP binding cassette subfamily D member 1 [Source:HGNC Symbol;Acc:HGNC:61]
ENSG00000047644	-0,4005755	0,57561607	1,75367136	0,21720132	0,51695895	WWC3	WWC family member 3 [Source:HGNC Symbol;Acc:HGNC:29237]
ENSG00000067606	-0,1933955	5,62589986	1,75201411	0,21740106	0,51717285	PRKCZ	protein kinase C zeta [Source:HGNC Symbol;Acc:HGNC:9412]
ENSG00000144792	0,63188073	-0,6620027	1,75175103	0,21743279	0,51717285	ZNF660	zinc finger protein 660 [Source:HGNC Symbol;Acc:HGNC:26720]
ENSG00000224905	-1,151088	-2,2107019	1,75214363	0,21738544	0,51717285	AP001347.1	novel transcript, antisense to LIPI
ENSG00000267530	-0,5805907	-0,6854679	1,75190515	0,2174142	0,51717285	LINC01836	long intergenic non-protein coding RNA 1836 [Source:HGNC Symbol;Acc:HGNC:52652]
ENSG00000276293	0,19054992	5,84372293	1,75137857	0,21747773	0,51719553	PIP4K2B	phosphatidylinositol-5-phosphate 4-kinase type 2 beta [Source:HGNC Symbol;Acc:HGNC:8998]
ENSG00000179094	0,23446702	2,76859211	1,75007772	0,21763477	0,51748475	PER1	period circadian regulator 1 [Source:HGNC Symbol;Acc:HGNC:8845]
ENSG00000242110	0,35053975	1,18388153	1,74918574	0,21774253	0,51765674	AMACR	alpha-methylacyl-CoA racemase [Source:HGNC Symbol;Acc:HGNC:451]
ENSG00000133067	0,62142928	-0,8722416	1,74837495	0,21784056	0,51780551	LGR6	leucine rich repeat containing G protein-coupled receptor 6 [Source:HGNC Symbol;Acc:HGNC:19719]
ENSG00000103035	-0,1912758	8,24269325	1,74616701	0,21810779	0,51785092	PSMD7	proteasome 26S subunit, non-ATPase 7 [Source:HGNC Symbol;Acc:HGNC:9565]
ENSG00000129422	0,22141644	4,99741697	1,7465939	0,21805609	0,51785092	MTUS1	microtubule associated scaffold protein 1 [Source:HGNC Symbol;Acc:HGNC:29789]
ENSG00000130741	0,18002054	7,04438116	1,74665025	0,21804926	0,51785092	EIF2S3	eukaryotic translation initiation factor 2 subunit gamma [Source:HGNC Symbol;Acc:HGNC:3267]
ENSG00000131043	0,19527961	6,65533296	1,74617665	0,21810662	0,51785092	AAR2	AAR2 splicing factor [Source:HGNC Symbol;Acc:HGNC:15886]
ENSG00000141449	0,80663623	-1,7372782	1,7469671	0,2180109	0,51785092	GREB1L	GREB1 like retinoic acid receptor coactivator [Source:HGNC Symbol;Acc:HGNC:31042]
ENSG00000163516	-0,2228156	4,84244891	1,74658384	0,21805731	0,51785092	ANKZF1	ankyrin repeat and zinc finger peptidyl tRNA hydrolase 1 [Source:HGNC Symbol;Acc:HGNC:25527]
ENSG00000246174	-0,4210949	0,99955961	1,74650378	0,218067	0,51785092	KCTD21-AS1	KCTD21 antisense RNA 1 [Source:HGNC Symbol;Acc:HGNC:48674]
ENSG00000198618	-0,2225361	4,26106814	1,7450332	0,21824519	0,51805614	PPIAP22	peptidylprolyl isomerase A pseudogene 22 [Source:HGNC Symbol;Acc:HGNC:17236]
ENSG00000228218	-0,3101366	0,90428819	1,74486861	0,21826515	0,51805614	ATF4P3	activating transcription factor 4 pseudogene 3 [Source:HGNC Symbol;Acc:HGNC:788]
ENSG00000124177	0,45214767	4,44624136	1,74409165	0,21835939	0,51819562	CHD6	chromodomain helicase DNA binding protein 6 [Source:HGNC Symbol;Acc:HGNC:19057]
ENSG00000146826	-0,2594372	3,61273631	1,74184324	0,2186324	0,51850662	MAP11	microtubule associated protein 11 [Source:HGNC Symbol;Acc:HGNC:25604]
ENSG00000204406	0,25577789	3,15880953	1,7419713	0,21861684	0,51850662	MBD5	methyl-CpG binding domain protein 5 [Source:HGNC Symbol;Acc:HGNC:20444]
ENSG00000229349	-0,4976432	-0,5856205	1,74218649	0,21859069	0,51850662	ACTG1P9	actin gamma 1 pseudogene 9 [Source:HGNC Symbol;Acc:HGNC:154]

ENSG00000287956	-0,8042795	-1,6497485	1,74266931	0,21853204	0,51850662	AC010872.2	novel transcript
ENSG00000106460	0,26929736	4,03304387	1,74119357	0,21871138	0,51860972	TMEM106B	transmembrane protein 106B [Source:HGNC Symbol;Acc:HGNC:22407]
ENSG00000260807	-0,7101592	-1,7373001	1,74074596	0,21876581	0,51865461	CEROX1	cytoplasmic endogenous regulator of oxidative phosphorylation 1 [Source:HGNC Symbol;Acc:HGNC:53928]
ENSG00000233780	0,64975141	-1,5639092	1,73914843	0,21896023	0,51903132	HNRNPA1P30	heterogeneous nuclear ribonucleoprotein A1 pseudogene 30 [Source:HGNC Symbol;Acc:HGNC:39548]
ENSG00000131584	-0,2064241	4,35000253	1,73859154	0,21902806	0,51910788	ACAP3	ArfGAP with coiled-coil, ankyrin repeat and PH domains 3 [Source:HGNC Symbol;Acc:HGNC:16754]
ENSG00000144152	0,30013513	1,05735681	1,7365786	0,21927348	0,51960524	FBLN7	fibulin 7 [Source:HGNC Symbol;Acc:HGNC:26740]
ENSG00000216316	0,8818556	-0,7697445	1,73580249	0,2193682	0,51974539	AL022722.1	60S Ribosomal Protein L13A (RPL13) pseudogene
ENSG00000109133	0,25225055	5,56907413	1,73505483	0,21945951	0,51982589	TMEM33	transmembrane protein 33 [Source:HGNC Symbol;Acc:HGNC:25541]
ENSG00000236296	0,83138527	-1,9988567	1,73494155	0,21947334	0,51982589	GUSBP5	GUSB pseudogene 5 [Source:HGNC Symbol;Acc:HGNC:42319]
ENSG00000106537	0,20460374	5,61646068	1,73389618	0,2196011	0,52004417	TSPAN13	tetraspanin 13 [Source:HGNC Symbol;Acc:HGNC:21643]
ENSG00000089159	-0,2141121	5,14111836	1,73042526	0,22002601	0,52030155	PXN	paxillin [Source:HGNC Symbol;Acc:HGNC:9718]
ENSG00000102805	0,25620592	3,31839066	1,73057335	0,22000786	0,52030155	CLN5	CLN5 intracellular trafficking protein [Source:HGNC Symbol;Acc:HGNC:2076]
ENSG00000141030	0,17522718	6,24208112	1,73122759	0,21992769	0,52030155	COPS3	COP9 signalosome subunit 3 [Source:HGNC Symbol;Acc:HGNC:2239]
ENSG00000145494	-0,1853995	6,78833846	1,73249012	0,2197731	0,52030155	NDUFS6	NADH:ubiquinone oxidoreductase subunit S6 [Source:HGNC Symbol;Acc:HGNC:7713]
ENSG00000151224	-0,2217417	6,66209932	1,73062054	0,22000208	0,52030155	MAT1A	methionine adenosyltransferase 1A [Source:HGNC Symbol;Acc:HGNC:6903]
ENSG00000170270	0,21218639	3,81969329	1,73184917	0,21985156	0,52030155	GON7	GON7 subunit of KEOPS complex [Source:HGNC Symbol;Acc:HGNC:20356]
ENSG00000179314	0,44126864	0,56608569	1,73230798	0,21979539	0,52030155	WSCD1	WSC domain containing 1 [Source:HGNC Symbol;Acc:HGNC:29060]
ENSG00000197405	-0,8585018	-0,2888195	1,73039011	0,22003032	0,52030155	C5AR1	complement C5a receptor 1 [Source:HGNC Symbol;Acc:HGNC:1338]
ENSG00000260032	0,20611968	5,81799929	1,73094973	0,21996173	0,52030155	NORAD	non-coding RNA activated by DNA damage [Source:HGNC Symbol;Acc:HGNC:44311]
ENSG00000112699	0,18412388	8,4623669	1,72920923	0,22017514	0,52030713	GMDS	GDP-mannose 4,6-dehydratase [Source:HGNC Symbol;Acc:HGNC:4369]
ENSG00000138623	0,29668824	5,06151189	1,72952626	0,22013625	0,52030713	SEMA7A	semaphorin 7A (John Milton Hagen blood group) [Source:HGNC Symbol;Acc:HGNC:10741]
ENSG00000172845	0,20210517	4,79406341	1,72991141	0,22008901	0,52030713	SP3	Sp3 transcription factor [Source:HGNC Symbol;Acc:HGNC:11208]
ENSG00000196151	0,21759727	3,72114274	1,72936556	0,22015596	0,52030713	WDSUB1	WD repeat, sterile alpha motif and U-box domain containing 1 [Source:HGNC Symbol;Acc:HGNC:26697]
ENSG00000113441	0,32209574	3,62220411	1,72849276	0,22026307	0,52034658	LNPEP	leucyl and cystinyl aminopeptidase [Source:HGNC Symbol;Acc:HGNC:6656]
ENSG00000176396	0,25375636	3,82008925	1,72859372	0,22025068	0,52034658	EID2	EP300 interacting inhibitor of differentiation 2 [Source:HGNC Symbol;Acc:HGNC:28292]
ENSG00000135720	0,24133285	5,80766694	1,72788285	0,22033796	0,52043934	DYNC1LI2	dynein cytoplasmic 1 light intermediate chain 2 [Source:HGNC Symbol;Acc:HGNC:2966]
ENSG00000233426	0,21377911	2,75114714	1,7273448	0,22040406	0,5205113	EIF3FP3	eukaryotic translation initiation factor 3 subunit F pseudogene 3 [Source:HGNC Symbol;Acc:HGNC:37625]
ENSG00000176248	-0,234276	4,50063421	1,72663489	0,22049131	0,52061627	ANAPC2	anaphase promoting complex subunit 2 [Source:HGNC Symbol;Acc:HGNC:19989]
ENSG00000182141	0,42157756	0,99484764	1,72640329	0,22051978	0,52061627	ZNF708	zinc finger protein 708 [Source:HGNC Symbol;Acc:HGNC:12945]
ENSG00000106089	-0,2699219	2,12091369	1,72542842	0,22063969	0,5207456	STX1A	syntaxin 1A [Source:HGNC Symbol;Acc:HGNC:11433]
ENSG00000143363	0,2872675	3,96750346	1,7253783	0,22064585	0,5207456	PRUNE1	prune exopolyphosphatase 1 [Source:HGNC Symbol;Acc:HGNC:13420]
ENSG00000100320	0,20508782	4,84728905	1,72421713	0,2207888	0,520813	RBFOX2	RNA binding fox-1 homolog 2 [Source:HGNC Symbol;Acc:HGNC:9906]
ENSG00000105372	-0,2092423	10,8999874	1,7243419	0,22077343	0,520813	RPS19	ribosomal protein S19 [Source:HGNC Symbol;Acc:HGNC:10402]
ENSG00000159658	0,21513812	3,82593761	1,72398808	0,22081701	0,520813	EFCAB14	EF-hand calcium binding domain 14 [Source:HGNC Symbol;Acc:HGNC:29051]
ENSG00000161921	0,18192087	5,35998694	1,72447929	0,22075652	0,520813	CXCL16	C-X-C motif chemokine ligand 16 [Source:HGNC Symbol;Acc:HGNC:16642]
ENSG00000120910	-0,1986873	3,78193523	1,72205805	0,22105492	0,52119913	PPP3CC	protein phosphatase 3 catalytic subunit gamma [Source:HGNC Symbol;Acc:HGNC:9316]
ENSG00000172006	0,26048751	1,69908539	1,72179192	0,22108776	0,52119913	ZNF554	zinc finger protein 554 [Source:HGNC Symbol;Acc:HGNC:26629]
ENSG00000181789	-0,3065208	7,05175154	1,72198281	0,22106421	0,52119913	COPG1	COPI coat complex subunit gamma 1 [Source:HGNC Symbol;Acc:HGNC:2236]
ENSG00000271207	-0,6560161	-1,6770364	1,72072251	0,22121976	0,52142618	MTCO1P22	MT-CO1 pseudogene 22 [Source:HGNC Symbol;Acc:HGNC:52087]
ENSG00000014164	-0,2428669	4,38571635	1,7198047	0,22133313	0,52160925	ZC3H3	zinc finger CCCH-type containing 3 [Source:HGNC Symbol;Acc:HGNC:28972]
ENSG00000143845	0,29914436	4,16461336	1,71821445	0,22152976	0,52190425	ETNK2	ethanolamine kinase 2 [Source:HGNC Symbol;Acc:HGNC:25575]
ENSG00000242445	-0,2553033	1,84936801	1,71824845	0,22152555	0,52190425	RPL7AP11	ribosomal protein L7a pseudogene 11 [Source:HGNC Symbol;Acc:HGNC:35242]
ENSG00000147224	-0,1912327	6,17340966	1,71699765	0,22168037	0,52217487	PRPS1	phosphoribosyl pyrophosphate synthetase 1 [Source:HGNC Symbol;Acc:HGNC:9462]
ENSG00000130748	-0,2074145	3,36105289	1,71635805	0,22175959	0,52219811	TMEM160	transmembrane protein 160 [Source:HGNC Symbol;Acc:HGNC:26042]

ENSG00000234268	-0,3184491	0,88367517	1,71634083	0,22176173	0,52219811	AP000936.3	ribosomal protein S27 (metallopanstimulin 1) (RPS27) pseudogene
ENSG00000170185	0,24637696	3,10450199	1,71565944	0,22184617	0,52231276	USP38	ubiquitin specific peptidase 38 [Source:HGNC Symbol;Acc:HGNC:20067]
ENSG00000144381	0,28015041	11,5845635	1,71463324	0,22197342	0,5225279	HSPD1	heat shock protein family D (Hsp60) member 1 [Source:HGNC Symbol;Acc:HGNC:5261]
ENSG00000166166	-0,2610491	5,2020545	1,71434583	0,22200908	0,5225279	TRMT61A	tRNA methyltransferase 61A [Source:HGNC Symbol;Acc:HGNC:23790]
ENSG00000065135	0,23870317	6,30514039	1,71373199	0,22208527	0,52262302	GNAI3	G protein subunit alpha i3 [Source:HGNC Symbol;Acc:HGNC:4387]
ENSG00000165030	-0,2080855	6,04275591	1,71314122	0,22215862	0,52267141	NFIL3	nuclear factor, interleukin 3 regulated [Source:HGNC Symbol;Acc:HGNC:7787]
ENSG00000260912	-0,5067482	-0,3697159	1,71299014	0,22217739	0,52267141	AL158206.1	novel transcript, overlapping ACER2
ENSG00000164715	0,26998555	4,52186273	1,71197135	0,22230398	0,52280085	LMTK2	lemur tyrosine kinase 2 [Source:HGNC Symbol;Acc:HGNC:17880]
ENSG00000178252	0,28023204	6,19537763	1,71219562	0,22227611	0,52280085	WDR6	WD repeat domain 6 [Source:HGNC Symbol;Acc:HGNC:12758]
ENSG00000146701	-0,1908026	8,55571208	1,71144247	0,22236974	0,52287132	MDH2	malate dehydrogenase 2 [Source:HGNC Symbol;Acc:HGNC:6971]
ENSG00000141458	0,28726466	6,87118951	1,71036122	0,22250425	0,52310342	NPC1	NPC intracellular cholesterol transporter 1 [Source:HGNC Symbol;Acc:HGNC:7897]
ENSG00000099812	0,33030019	4,21052895	1,70995302	0,22255507	0,52313869	MISP	mitotic spindle positioning [Source:HGNC Symbol;Acc:HGNC:27000]
ENSG00000243859	-0,4150487	-0,1734062	1,7088655	0,22269052	0,52337287	RPL5P17	ribosomal protein L5 pseudogene 17 [Source:HGNC Symbol;Acc:HGNC:36605]
ENSG00000169855	0,32247813	6,41974464	1,70668438	0,22296251	0,52392783	ROBO1	roundabout guidance receptor 1 [Source:HGNC Symbol;Acc:HGNC:10249]
ENSG00000129824	-0,1757709	7,64678311	1,70271146	0,22345911	0,52486436	RPS4Y1	ribosomal protein S4 Y-linked 1 [Source:HGNC Symbol;Acc:HGNC:10425]
ENSG00000142798	-0,5682245	4,86275718	1,70291829	0,22343322	0,52486436	HSPG2	heparan sulfate proteoglycan 2 [Source:HGNC Symbol;Acc:HGNC:5273]
ENSG00000174437	-0,2043957	7,75848797	1,70263372	0,22346884	0,52486436	ATP2A2	ATPase sarcoplasmic/endoplasmic reticulum Ca2+ transporting 2 [Source:HGNC Symbol;Acc:HGNC:812]
ENSG00000119782	0,28485712	2,35137508	1,70102248	0,22367068	0,52516956	FKBP1B	FKBP prolyl isomerase 1B [Source:HGNC Symbol;Acc:HGNC:3712]
ENSG00000287828	-0,6181875	-1,5238079	1,70104347	0,22366805	0,52516956	AL354743.2	novel transcript, antisense to PRDM16
ENSG00000060303	-0,3276099	0,83370162	1,70043123	0,22374481	0,52525919	RPS17P5	ribosomal protein S17 pseudogene 5 [Source:HGNC Symbol;Acc:HGNC:21352]
ENSG00000143178	0,70941865	-1,1102521	1,69973114	0,22383263	0,52529652	TBX19	T-box transcription factor 19 [Source:HGNC Symbol;Acc:HGNC:11596]
ENSG00000165688	0,2248053	7,62073862	1,69986726	0,22381555	0,52529652	PMPCA	peptidase, mitochondrial processing subunit alpha [Source:HGNC Symbol;Acc:HGNC:18667]
ENSG00000260459	0,45159036	0,43111218	1,69891292	0,22393532	0,52545311	FTLP14	ferritin light chain pseudogene 14 [Source:HGNC Symbol;Acc:HGNC:37964]
ENSG00000103018	0,23211294	7,58384805	1,69848037	0,22398963	0,52549616	CYB5B	cytochrome b5 type B [Source:HGNC Symbol;Acc:HGNC:24374]
ENSG00000073350	0,20373137	6,43722379	1,69432548	0,22451227	0,52555771	LLGL2	LLGL scribble cell polarity complex component 2 [Source:HGNC Symbol;Acc:HGNC:6629]
ENSG00000085382	0,30421514	2,15320781	1,6972862	0,22413968	0,52555771	HACE1	HECT domain and ankyrin repeat containing E3 ubiquitin protein ligase 1 [Source:HGNC Symbol;Acc:HGNC:21033]
ENSG00000091513	0,23245417	12,4072417	1,69582266	0,22432375	0,52555771	TF	transferrin [Source:HGNC Symbol;Acc:HGNC:11740]
ENSG00000092964	0,29366351	5,03912668	1,69480127	0,22445234	0,52555771	DPYSL2	dihydropyrimidinase like 2 [Source:HGNC Symbol;Acc:HGNC:3014]
ENSG00000099875	0,17326778	6,2108139	1,69443127	0,22449895	0,52555771	MKNK2	MAPK interacting serine/threonine kinase 2 [Source:HGNC Symbol;Acc:HGNC:7111]
ENSG00000120071	0,3069887	4,46532205	1,69465942	0,22447021	0,52555771	KANSL1	KAT8 regulatory NSL complex subunit 1 [Source:HGNC Symbol;Acc:HGNC:24565]
ENSG00000140463	0,21094972	3,26206934	1,69426794	0,22451952	0,52555771	BBS4	Bardet-Biedl syndrome 4 [Source:HGNC Symbol;Acc:HGNC:969]
ENSG00000143222	-0,2141513	6,40122398	1,69512388	0,22441171	0,52555771	UFC1	ubiquitin-fold modifier conjugating enzyme 1 [Source:HGNC Symbol;Acc:HGNC:26941]
ENSG00000145506	-0,4566809	-0,535008	1,69567445	0,22434241	0,52555771	NKD2	NKD inhibitor of WNT signaling pathway 2 [Source:HGNC Symbol;Acc:HGNC:17046]
ENSG00000213445	-0,2150712	3,366272	1,69752089	0,22411018	0,52555771	SIPA1	signal-induced proliferation-associated 1 [Source:HGNC Symbol;Acc:HGNC:10885]
ENSG00000225663	-0,202703	5,93470937	1,6952769	0,22439245	0,52555771	MCRIP1	MAPK regulated corepressor interacting protein 1 [Source:HGNC Symbol;Acc:HGNC:28007]
ENSG00000226243	-0,2221637	2,40714579	1,69647206	0,22424205	0,52555771	RPL37AP1	ribosomal protein L37a pseudogene 1 [Source:HGNC Symbol;Acc:HGNC:16548]
ENSG00000249464	0,56495481	-0,3546307	1,69523206	0,22439809	0,52555771	LINC01091	long intergenic non-protein coding RNA 1091 [Source:HGNC Symbol;Acc:HGNC:27721]
ENSG00000272716	-0,471046	0,50732495	1,69639986	0,22425113	0,52555771	AL121658.1	novel transcript
ENSG00000084093	0,309286	3,33363823	1,69288255	0,22469416	0,52567979	REST	RE1 silencing transcription factor [Source:HGNC Symbol;Acc:HGNC:9966]
ENSG00000100519	0,19528614	7,08861469	1,69349887	0,22461645	0,52567979	PSMC6	proteasome 26S subunit, ATPase 6 [Source:HGNC Symbol;Acc:HGNC:9553]
ENSG00000215014	-0,6611845	-1,2942612	1,69271621	0,22471515	0,52567979	AL645728.1	novel transcript
ENSG00000215093	-0,5026678	-0,9884842	1,69271257	0,2247156	0,52567979	EEF1A1P29	eukaryotic translation elongation factor 1 alpha 1 pseudogene 29 [Source:HGNC Symbol;Acc:HGNC:37904]
ENSG00000125148	-0,1923735	7,93714049	1,69111026	0,22491785	0,52605773	MT2A	metallothionein 2A [Source:HGNC Symbol;Acc:HGNC:7406]
ENSG00000171806	-0,3105336	2,00032341	1,69078634	0,22495877	0,52605773	METTL18	methyltransferase like 18 [Source:HGNC Symbol;Acc:HGNC:28793]

ENSG00000256742	0,77671437	-1,8794809	1,69057717	0,22498519	0,52605773	KDM2B-DT	KDM2B divergent transcript [Source:HGNC Symbol;Acc:HGNC:53287]
ENSG00000198464	0,25577471	2,791158	1,68996819	0,22506215	0,52615347	ZNF480	zinc finger protein 480 [Source:HGNC Symbol;Acc:HGNC:23305]
ENSG00000114026	-0,1946549	5,27755561	1,68967637	0,22509905	0,52615552	OGG1	8-oxoguanine DNA glycosylase [Source:HGNC Symbol;Acc:HGNC:8125]
ENSG00000188167	0,41960785	1,24686828	1,68917684	0,22516222	0,52621898	TMPPE	transmembrane protein with metallophosphoesterase domain [Source:HGNC Symbol;Acc:HGNC:33865]
ENSG00000174718	0,25374206	2,32764988	1,68834737	0,22526717	0,52638004	RESF1	retroelement silencing factor 1 [Source:HGNC Symbol;Acc:HGNC:25559]
ENSG00000179632	-0,2241876	6,59029712	1,68768447	0,22535109	0,52649193	MAF1	MAF1 homolog, negative regulator of RNA polymerase III [Source:HGNC Symbol;Acc:HGNC:24966]
ENSG00000203993	-0,2712177	2,32778932	1,68669067	0,22547698	0,52670182	ARRDC1-AS1	ARRDC1 antisense RNA 1 [Source:HGNC Symbol;Acc:HGNC:23395]
ENSG00000140365	-0,1888073	6,69993149	1,68627136	0,22553013	0,52674174	COMMD4	COMM domain containing 4 [Source:HGNC Symbol;Acc:HGNC:26027]
ENSG00000126767	0,20443025	3,86318063	1,68585032	0,22558351	0,5267822	ELK1	ETS transcription factor ELK1 [Source:HGNC Symbol;Acc:HGNC:3321]
ENSG00000165632	-0,2379338	3,15547387	1,6850547	0,22568443	0,52693364	TAF3	TATA-box binding protein associated factor 3 [Source:HGNC Symbol;Acc:HGNC:17303]
ENSG00000077721	-0,1944744	5,30181422	1,68378313	0,22584585	0,52720322	UBE2A	ubiquitin conjugating enzyme E2 A [Source:HGNC Symbol;Acc:HGNC:12472]
ENSG00000111666	0,20721337	7,99308863	1,68357676	0,22587206	0,52720322	CHPT1	choline phosphotransferase 1 [Source:HGNC Symbol;Acc:HGNC:17852]
ENSG00000148814	-0,3361171	1,31993105	1,68261803	0,22599389	0,52739351	LRRC27	leucine rich repeat containing 27 [Source:HGNC Symbol;Acc:HGNC:29346]
ENSG00000229358	-0,5226583	-0,5115347	1,68236714	0,22602579	0,52739351	DPY19L1P1	DPY19L1 pseudogene 1 [Source:HGNC Symbol;Acc:HGNC:22395]
ENSG00000139133	0,27364994	2,26634104	1,68153969	0,22613103	0,52755481	ALG10	ALG10 alpha-1,2-glucosyltransferase [Source:HGNC Symbol;Acc:HGNC:23162]
ENSG00000178425	-0,1929964	5,24833153	1,68106838	0,226191	0,52761047	NT5DC1	5'-nucleotidase domain containing 1 [Source:HGNC Symbol;Acc:HGNC:21556]
ENSG00000168795	-0,210202	3,89200435	1,68040062	0,22627601	0,5277245	ZBTB5	zinc finger and BTB domain containing 5 [Source:HGNC Symbol;Acc:HGNC:23836]
ENSG00000161914	-0,3385571	1,40305347	1,67987428	0,22634305	0,52779658	ZNF653	zinc finger protein 653 [Source:HGNC Symbol;Acc:HGNC:25196]
ENSG00000055950	0,17754706	6,55312055	1,67917195	0,22643254	0,52790985	MRPL43	mitochondrial ribosomal protein L43 [Source:HGNC Symbol;Acc:HGNC:14517]
ENSG00000231414	-0,3048384	0,88527409	1,67892598	0,22646389	0,52790985	RPL39P15	ribosomal protein L39 pseudogene 15 [Source:HGNC Symbol;Acc:HGNC:35856]
ENSG00000221953	-0,7831179	-1,043941	1,6771615	0,22668899	0,52835025	C1orf229	chromosome 1 open reading frame 229 [Source:HGNC Symbol;Acc:HGNC:33759]
ENSG00000009830	0,22842904	4,34928081	1,67493678	0,22697322	0,52835221	POMT2	protein O-mannosyltransferase 2 [Source:HGNC Symbol;Acc:HGNC:19743]
ENSG00000064687	-0,260386	2,37535311	1,67539115	0,22691513	0,52835221	ABCA7	ATP binding cassette subfamily A member 7 [Source:HGNC Symbol;Acc:HGNC:37]
ENSG00000100151	0,22333006	4,17755509	1,67662174	0,2267579	0,52835221	PICK1	protein interacting with PRKCA 1 [Source:HGNC Symbol;Acc:HGNC:9394]
ENSG00000109919	0,16858211	8,57825733	1,67553409	0,22689686	0,52835221	MTCH2	mitochondrial carrier 2 [Source:HGNC Symbol;Acc:HGNC:17587]
ENSG00000135164	-0,2399346	3,33992798	1,67467559	0,22700662	0,52835221	DMTF1	cyclin D binding myb like transcription factor 1 [Source:HGNC Symbol;Acc:HGNC:14603]
ENSG00000135334	0,20319929	5,58781872	1,67460758	0,22701532	0,52835221	AKIRIN2	akirin 2 [Source:HGNC Symbol;Acc:HGNC:21407]
ENSG00000234785	-0,2670162	1,70931475	1,67515255	0,22694563	0,52835221	EEF1GP5	eukaryotic translation elongation factor 1 gamma pseudogene 5 [Source:HGNC Symbol;Acc:HGNC:44560]
ENSG00000235545	-0,6492306	-1,869782	1,6746411	0,22701104	0,52835221	AC103923.1	novel transcript
ENSG00000276045	-0,2205773	5,44031024	1,67550931	0,22690003	0,52835221	ORAI1	ORAI calcium release-activated calcium modulator 1 [Source:HGNC Symbol;Acc:HGNC:25896]
ENSG00000136982	0,2040762	3,52224692	1,67370224	0,22713116	0,52853761	DSCC1	DNA replication and sister chromatid cohesion 1 [Source:HGNC Symbol;Acc:HGNC:24453]
ENSG00000163249	0,19879856	4,71329599	1,67146327	0,22741798	0,52912076	CCNYL1	cyclin Y like 1 [Source:HGNC Symbol;Acc:HGNC:26868]
ENSG00000073905	0,38130322	0,10304762	1,67046586	0,22754591	0,52924983	VDAC1P1	voltage dependent anion channel 1 pseudogene 1 [Source:HGNC Symbol;Acc:HGNC:12671]
ENSG00000131471	-0,1986662	3,31863446	1,67057683	0,22753167	0,52924983	AOC3	amine oxidase copper containing 3 [Source:HGNC Symbol;Acc:HGNC:550]
ENSG00000164053	-0,2205148	3,6552692	1,67013963	0,22758778	0,52926294	ATRIP	ATR interacting protein [Source:HGNC Symbol;Acc:HGNC:33499]
ENSG00000100316	-0,1750412	11,409237	1,66688923	0,22800547	0,5299812	RPL3	ribosomal protein L3 [Source:HGNC Symbol;Acc:HGNC:10332]
ENSG00000113645	-0,1943414	4,56902636	1,66695664	0,22799679	0,5299812	WWC1	WW and C2 domain containing 1 [Source:HGNC Symbol;Acc:HGNC:29435]
ENSG00000180011	-0,2096888	4,11957992	1,6674132	0,22793806	0,5299812	ZADH2	zinc binding alcohol dehydrogenase domain containing 2 [Source:HGNC Symbol;Acc:HGNC:28697]
ENSG00000244462	-0,1861728	5,58897928	1,66656319	0,22804742	0,5299944	RBM12	RNA binding motif protein 12 [Source:HGNC Symbol;Acc:HGNC:9898]
ENSG00000137216	0,19847744	5,96422692	1,66550475	0,2281837	0,53005814	TMEM63B	transmembrane protein 63B [Source:HGNC Symbol;Acc:HGNC:17735]
ENSG00000146828	0,19828728	4,75833266	1,66572509	0,22815532	0,53005814	SLC12A9	solute carrier family 12 member 9 [Source:HGNC Symbol;Acc:HGNC:17435]
ENSG00000177469	0,23597337	5,43989184	1,66591744	0,22813055	0,53005814	CAVIN1	caveolae associated protein 1 [Source:HGNC Symbol;Acc:HGNC:9688]
ENSG00000146839	-0,6034418	-0,8577798	1,6646996	0,22828743	0,53016031	ZAN	zonadhesin [Source:HGNC Symbol;Acc:HGNC:12857]
ENSG00000215039	0,38063707	0,93989397	1,66460008	0,22830026	0,53016031	CD27-AS1	CD27 antisense RNA 1 [Source:HGNC Symbol;Acc:HGNC:43896]

ENSG00000085733	-0,2320894	6,52603088	1,66263333	0,22855395	0,53060962	CTTN	cortactin [Source:HGNC Symbol;Acc:HGNC:3338]
ENSG00000173209	0,26038442	3,8087033	1,66253701	0,22856639	0,53060962	AHSA2P	activator of HSP90 ATPase homolog 2, pseudogene [Source:HGNC Symbol;Acc:HGNC:20437]
ENSG00000107554	-0,2815775	3,15610427	1,66174662	0,22866845	0,53076223	DNMBP	dynamin binding protein [Source:HGNC Symbol;Acc:HGNC:30373]
ENSG00000139914	0,38499046	0,73125246	1,66097532	0,22876812	0,5309092	FITM1	fat storage inducing transmembrane protein 1 [Source:HGNC Symbol;Acc:HGNC:33714]
ENSG00000086506	-0,3600105	0,95382643	1,65924616	0,22899177	0,53100679	HBQ1	hemoglobin subunit theta 1 [Source:HGNC Symbol;Acc:HGNC:4833]
ENSG00000119950	-0,3392533	1,55121135	1,66012729	0,22887776	0,53100679	MXI1	MAX interactor 1, dimerization protein [Source:HGNC Symbol;Acc:HGNC:7534]
ENSG00000160539	-0,5366528	-0,3883905	1,65924508	0,22899191	0,53100679	PLPP7	phospholipid phosphatase 7 (inactive) [Source:HGNC Symbol;Acc:HGNC:28174]
ENSG00000204209	0,25359087	6,67767514	1,65953879	0,2289539	0,53100679	DAXX	death domain associated protein [Source:HGNC Symbol;Acc:HGNC:2681]
ENSG00000267064	-0,639329	-0,9857483	1,66001588	0,22889217	0,53100679	UXT-AS1	UXT antisense RNA 1 [Source:HGNC Symbol;Acc:HGNC:49239]
ENSG00000113648	-0,1820738	8,0871152	1,65831672	0,2291121	0,53111696	MACROH2A1	macroH2A.1 histone [Source:HGNC Symbol;Acc:HGNC:4740]
ENSG00000174132	0,24653625	2,60161477	1,65831651	0,22911213	0,53111696	FAM174A	family with sequence similarity 174 member A [Source:HGNC Symbol;Acc:HGNC:24943]
ENSG00000122783	-0,2449218	5,52991906	1,65760882	0,22920381	0,53113026	CYREN	cell cycle regulator of NHEJ [Source:HGNC Symbol;Acc:HGNC:22432]
ENSG00000185019	0,18727111	4,65155572	1,65767988	0,2291946	0,53113026	UBOX5	U-box domain containing 5 [Source:HGNC Symbol;Acc:HGNC:17777]
ENSG00000240489	-0,4313969	-0,0669178	1,6574304	0,22922694	0,53113026	SETP14	SET pseudogene 14 [Source:HGNC Symbol;Acc:HGNC:42933]
ENSG00000100883	-0,2338437	7,4188397	1,65697453	0,22928603	0,53118294	SRP54	signal recognition particle 54 [Source:HGNC Symbol;Acc:HGNC:11301]
ENSG00000111669	-0,1936611	9,91703288	1,65583241	0,22943417	0,53144186	TPI1	triosephosphate isomerase 1 [Source:HGNC Symbol;Acc:HGNC:12009]
ENSG00000152443	-0,2225475	2,25955221	1,65511147	0,22952775	0,53150304	ZNF776	zinc finger protein 776 [Source:HGNC Symbol;Acc:HGNC:26765]
ENSG00000153574	0,21062701	5,84081314	1,65506839	0,22953335	0,53150304	RPIA	ribose 5-phosphate isomerase A [Source:HGNC Symbol;Acc:HGNC:10297]
ENSG00000006194	0,19041945	5,24691946	1,65343678	0,22974533	0,53152899	ZNF263	zinc finger protein 263 [Source:HGNC Symbol;Acc:HGNC:13056]
ENSG00000105983	0,2086629	4,33021713	1,65350178	0,22973688	0,53152899	LMBR1	limb development membrane protein 1 [Source:HGNC Symbol;Acc:HGNC:13243]
ENSG00000116954	-0,3032782	2,98862537	1,65323993	0,22977093	0,53152899	RRAGC	Ras related GTP binding C [Source:HGNC Symbol;Acc:HGNC:19902]
ENSG00000132141	0,75573553	-1,6370286	1,65360833	0,22972303	0,53152899	CCT6B	chaperonin containing TCP1 subunit 6B [Source:HGNC Symbol;Acc:HGNC:1621]
ENSG00000156471	0,21438774	7,56626342	1,65274247	0,22983562	0,53152899	PTDSS1	phosphatidylserine synthase 1 [Source:HGNC Symbol;Acc:HGNC:9587]
ENSG00000164104	0,199837	8,0198557	1,65368677	0,22971284	0,53152899	HMGB2	high mobility group box 2 [Source:HGNC Symbol;Acc:HGNC:5000]
ENSG00000173825	-0,2377449	2,14043694	1,65284931	0,22982173	0,53152899	TIGD3	tigger transposable element derived 3 [Source:HGNC Symbol;Acc:HGNC:18334]
ENSG00000204653	-0,6031316	-0,3890908	1,65436224	0,22962506	0,53152899	ASPDH	aspartate dehydrogenase domain containing [Source:HGNC Symbol;Acc:HGNC:33856]
ENSG00000079459	0,19274334	9,95863446	1,65243484	0,22987564	0,5315374	FDFT1	farnesyl-diphosphate farnesyltransferase 1 [Source:HGNC Symbol;Acc:HGNC:3629]
ENSG00000240463	-0,4257683	0,10652509	1,65179926	0,22995836	0,53157087	RPS19P3	ribosomal protein S19 pseudogene 3 [Source:HGNC Symbol;Acc:HGNC:23560]
ENSG00000250731	0,63329611	-1,092034	1,65176441	0,22996289	0,53157087	TPM3P6	tropomyosin 3 pseudogene 6 [Source:HGNC Symbol;Acc:HGNC:39169]
ENSG00000070669	0,84976694	-1,4711401	1,65053358	0,23012319	0,53176132	ASNS	asparagine synthetase (glutamine-hydrolyzing) [Source:HGNC Symbol;Acc:HGNC:753]
ENSG00000131748	0,21784261	6,14133306	1,64820696	0,23042662	0,53176132	STARD3	StAR related lipid transfer domain containing 3 [Source:HGNC Symbol;Acc:HGNC:17579]
ENSG00000135211	0,2755383	3,61026857	1,64940086	0,23027085	0,53176132	TMEM60	transmembrane protein 60 [Source:HGNC Symbol;Acc:HGNC:21754]
ENSG00000139163	-0,1848569	4,54628556	1,64806098	0,23044568	0,53176132	ETNK1	ethanolamine kinase 1 [Source:HGNC Symbol;Acc:HGNC:24649]
ENSG00000168612	0,19915644	3,03261025	1,65056606	0,23011896	0,53176132	ZSWIM1	zinc finger SWIM-type containing 1 [Source:HGNC Symbol;Acc:HGNC:16155]
ENSG00000170515	-0,1674281	8,15370683	1,64993346	0,23020141	0,53176132	PA2G4	proliferation-associated 2G4 [Source:HGNC Symbol;Acc:HGNC:8550]
ENSG00000196233	0,32572529	3,81651831	1,6482043	0,23042697	0,53176132	LCOR	ligand dependent nuclear receptor corepressor [Source:HGNC Symbol;Acc:HGNC:29503]
ENSG00000205609	-0,3180692	2,42817836	1,64860518	0,23037465	0,53176132	EIF3CL	eukaryotic translation initiation factor 3 subunit C like [Source:HGNC Symbol;Acc:HGNC:26347]
ENSG00000242622	-0,3577055	0,37466264	1,64830764	0,23041348	0,53176132	AC092910.3	novel transcript
ENSG00000267106	-0,6693953	0,28331307	1,64882801	0,23034557	0,53176132	ZNF561-AS1	ZNF561 antisense RNA 1 (head to head) [Source:HGNC Symbol;Acc:HGNC:27613]
ENSG00000273062	-0,9160249	-1,8213433	1,64911478	0,23030816	0,53176132	AL449106.1	novel transcript, antisense to RALGPS2
ENSG00000129946	-0,2418908	2,51677582	1,64739531	0,2305326	0,53187789	SHC2	SHC adaptor protein 2 [Source:HGNC Symbol;Acc:HGNC:29869]
ENSG00000205423	0,21677937	2,65890506	1,64639515	0,23066329	0,53209537	CNEP1R1	CTD nuclear envelope phosphatase 1 regulatory subunit 1 [Source:HGNC Symbol;Acc:HGNC:26759]
ENSG00000112294	0,24778838	5,46556374	1,64510649	0,23083182	0,53216983	ALDH5A1	aldehyde dehydrogenase 5 family member A1 [Source:HGNC Symbol;Acc:HGNC:408]
ENSG00000152795	0,18381463	8,45418866	1,64555177	0,23077357	0,53216983	HNRNPDL	heterogeneous nuclear ribonucleoprotein D like [Source:HGNC Symbol;Acc:HGNC:5037]

ENSG00000167792	0,22627398	7,74313761	1,645444	0,23078767	0,53216983	NDUFV1	NADH:ubiquinone oxidoreductase core subunit V1 [Source:HGNC Symbol;Acc:HGNC:7716]
ENSG00000171503	0,22906337	3,73198108	1,64503423	0,23084128	0,53216983	ETFDH	electron transfer flavoprotein dehydrogenase [Source:HGNC Symbol;Acc:HGNC:3483]
ENSG00000116985	0,65530811	-0,0406953	1,64360781	0,23102803	0,53221515	BMP8B	bone morphogenetic protein 8b [Source:HGNC Symbol;Acc:HGNC:1075]
ENSG00000142208	-0,2000881	6,69554349	1,64408495	0,23096554	0,53221515	AKT1	AKT serine/threonine kinase 1 [Source:HGNC Symbol;Acc:HGNC:391]
ENSG00000156162	-0,2177787	2,70647061	1,64348628	0,23104395	0,53221515	DPY19L4	dpy-19 like 4 [Source:HGNC Symbol;Acc:HGNC:27829]
ENSG00000163541	0,18176576	7,20270971	1,6435117	0,23104062	0,53221515	SUCLG1	succinate-CoA ligase GDP/ADP-forming subunit alpha [Source:HGNC Symbol;Acc:HGNC:11449]
ENSG00000188283	0,32258296	1,16121764	1,64297651	0,23111075	0,53221515	ZNF383	zinc finger protein 383 [Source:HGNC Symbol;Acc:HGNC:18609]
ENSG00000229133	-0,299871	1,0023397	1,64455546	0,23090394	0,53221515	RPS7P4	ribosomal protein S7 pseudogene 4 [Source:HGNC Symbol;Acc:HGNC:36000]
ENSG00000245849	0,50926447	-1,0889398	1,64293685	0,23111595	0,53221515	RAD51-AS1	RAD51 antisense RNA 1 [Source:HGNC Symbol;Acc:HGNC:48621]
ENSG00000001630	-0,2419825	3,79618103	1,64088593	0,23138497	0,53267797	CYP51A1	cytochrome P450 family 51 subfamily A member 1 [Source:HGNC Symbol;Acc:HGNC:2649]
ENSG00000255200	-0,5576861	-1,2657672	1,64084875	0,23138985	0,53267797	PGAM1P8	phosphoglycerate mutase 1 pseudogene 8 [Source:HGNC Symbol;Acc:HGNC:42455]
ENSG00000173156	-0,6481898	-1,2468049	1,64022264	0,23147207	0,53278328	RHOD	ras homolog family member D [Source:HGNC Symbol;Acc:HGNC:670]
ENSG00000235552	-0,2449629	3,40421462	1,63852584	0,23169508	0,53321257	RPL6P27	ribosomal protein L6 pseudogene 27 [Source:HGNC Symbol;Acc:HGNC:36133]
ENSG00000149573	0,21147236	5,49786101	1,63694138	0,23190359	0,53360837	MPZL2	myelin protein zero like 2 [Source:HGNC Symbol;Acc:HGNC:3496]
ENSG00000089902	0,21853062	4,23810577	1,63543044	0,23210266	0,53398233	RCOR1	REST corepressor 1 [Source:HGNC Symbol;Acc:HGNC:17441]
ENSG00000099365	-0,8472774	-0,9003317	1,63464274	0,23220654	0,53405311	STX1B	syntaxin 1B [Source:HGNC Symbol;Acc:HGNC:18539]
ENSG00000185813	-0,1695414	8,36026398	1,63482049	0,23218309	0,53405311	PCYT2	phosphate cytidylyltransferase 2, ethanolamine [Source:HGNC Symbol;Acc:HGNC:8756]
ENSG00000151366	0,20132157	4,22256508	1,63142739	0,23263122	0,53493021	NDUFC2	NADH:ubiquinone oxidoreductase subunit C2 [Source:HGNC Symbol;Acc:HGNC:7706]
ENSG00000226972	-0,6491035	-1,8685455	1,63120117	0,23266114	0,53493021	RPL12P19	ribosomal protein L12 pseudogene 19 [Source:HGNC Symbol;Acc:HGNC:36248]
ENSG00000077147	0,20317634	7,06604636	1,63023605	0,23278884	0,53513959	TM9SF3	transmembrane 9 superfamily member 3 [Source:HGNC Symbol;Acc:HGNC:21529]
ENSG00000088448	0,18748876	4,60754206	1,62742009	0,23316198	0,53515293	ANKRD10	ankyrin repeat domain 10 [Source:HGNC Symbol;Acc:HGNC:20265]
ENSG00000107036	0,23824725	3,36592297	1,62848707	0,2330205	0,53515293	RIC1	RIC1 homolog, RAB6A GEF complex partner 1 [Source:HGNC Symbol;Acc:HGNC:17686]
ENSG00000125386	0,22761885	4,40702092	1,62869021	0,23299358	0,53515293	FAM193A	family with sequence similarity 193 member A [Source:HGNC Symbol;Acc:HGNC:16822]
ENSG00000126773	-0,2239958	4,35227484	1,627064	0,23320923	0,53515293	PCNX4	pecanex 4 [Source:HGNC Symbol;Acc:HGNC:20349]
ENSG00000131591	-0,2339352	2,78336608	1,62852262	0,23301579	0,53515293	C1orf159	chromosome 1 open reading frame 159 [Source:HGNC Symbol;Acc:HGNC:26062]
ENSG00000166986	-0,2271628	7,01094822	1,62797044	0,23308899	0,53515293	MARS1	methionyl-tRNA synthetase 1 [Source:HGNC Symbol;Acc:HGNC:6898]
ENSG00000169118	0,24587279	1,58801311	1,62770546	0,23312413	0,53515293	CSNK1G1	casein kinase 1 gamma 1 [Source:HGNC Symbol;Acc:HGNC:2454]
ENSG00000174197	0,36625406	4,52272197	1,62796603	0,23308957	0,53515293	MGA	MAX dimerization protein MGA [Source:HGNC Symbol;Acc:HGNC:14010]
ENSG00000213337	-0,2401876	3,71129088	1,62929875	0,23291295	0,53515293	ANKRD39	ankyrin repeat domain 39 [Source:HGNC Symbol;Acc:HGNC:28640]
ENSG00000213857	-0,5642209	-1,2173082	1,62702706	0,23321413	0,53515293	AL080243.1	actin, beta (ACTB) pseudogene
ENSG00000266412	0,20821152	7,92413545	1,62899047	0,23295379	0,53515293	NCOA4	nuclear receptor coactivator 4 [Source:HGNC Symbol;Acc:HGNC:7671]
ENSG00000272141	-0,6977186	-1,732077	1,62687566	0,23323422	0,53515293	AL390719.2	novel transcript
ENSG00000001631	0,34041162	0,95514376	1,62502698	0,23347974	0,53520872	KRIT1	KRIT1 ankyrin repeat containing [Source:HGNC Symbol;Acc:HGNC:1573]
ENSG00000076706	0,2743354	5,16479359	1,62317366	0,23372623	0,53520872	MCAM	melanoma cell adhesion molecule [Source:HGNC Symbol;Acc:HGNC:6934]
ENSG00000106351	0,17012029	6,97934231	1,62507235	0,23347371	0,53520872	AGFG2	ArfGAP with FG repeats 2 [Source:HGNC Symbol;Acc:HGNC:5177]
ENSG00000124214	0,18348899	8,36199428	1,62317637	0,23372587	0,53520872	STAU1	staufen double-stranded RNA binding protein 1 [Source:HGNC Symbol;Acc:HGNC:11370]
ENSG00000132842	0,24832085	5,18190956	1,62310931	0,23373479	0,53520872	AP3B1	adaptor related protein complex 3 subunit beta 1 [Source:HGNC Symbol;Acc:HGNC:566]
ENSG00000139289	-0,1939694	7,41608274	1,6238592	0,23363501	0,53520872	PHLDA1	pleckstrin homology like domain family A member 1 [Source:HGNC Symbol;Acc:HGNC:8933]
ENSG00000144711	0,2701903	4,87830756	1,62375755	0,23364853	0,53520872	IQSEC1	IQ motif and Sec7 domain ArfGEF 1 [Source:HGNC Symbol;Acc:HGNC:29112]
ENSG00000147457	0,17621182	5,28106214	1,62473297	0,23351882	0,53520872	CHMP7	charged multivesicular body protein 7 [Source:HGNC Symbol;Acc:HGNC:28439]
ENSG00000148219	-0,3200865	3,10681629	1,6263137	0,23330881	0,53520872	ASTN2	astrotactin 2 [Source:HGNC Symbol;Acc:HGNC:17021]
ENSG00000160741	0,19807969	4,18403898	1,6252807	0,23344602	0,53520872	CRTC2	CREB regulated transcription coactivator 2 [Source:HGNC Symbol;Acc:HGNC:27301]
ENSG00000162642	0,20984265	3,86517686	1,62468528	0,23352516	0,53520872	C1orf52	chromosome 1 open reading frame 52 [Source:HGNC Symbol;Acc:HGNC:24871]
ENSG00000179172	-0,359536	0,58336663	1,6234519	0,2336892	0,53520872	HNRNPCL1	heterogeneous nuclear ribonucleoprotein C like 1 [Source:HGNC Symbol;Acc:HGNC:29295]

ENSG00000204261	0,64828077	-1,0887854	1,62363149	0,23366531	0,53520872	PSMB8-AS1	PSMB8 antisense RNA 1 (head to head) [Source:HGNC Symbol;Acc:HGNC:39758]
ENSG00000136997	-0,1753151	8,16241966	1,6216989	0,23392263	0,53532369	MYC	MYC proto-oncogene, bHLH transcription factor [Source:HGNC Symbol;Acc:HGNC:7553]
ENSG00000146263	0,23690494	2,71118783	1,62179935	0,23390924	0,53532369	MMS22L	MMS22 like, DNA repair protein [Source:HGNC Symbol;Acc:HGNC:21475]
ENSG00000173898	0,29814791	1,3777309	1,6216317	0,23393158	0,53532369	SPTBN2	spectrin beta, non-erythrocytic 2 [Source:HGNC Symbol;Acc:HGNC:11276]
ENSG00000175806	-0,2154205	3,59295727	1,62219266	0,23385685	0,53532369	MSRA	methionine sulfoxide reductase A [Source:HGNC Symbol;Acc:HGNC:7377]
ENSG00000093000	0,17265575	5,70881211	1,62089757	0,23402943	0,53546375	NUP50	nucleoporin 50 [Source:HGNC Symbol;Acc:HGNC:8065]
ENSG00000075702	-0,2285683	4,63732144	1,61952473	0,23421258	0,53570666	WDR62	WD repeat domain 62 [Source:HGNC Symbol;Acc:HGNC:24502]
ENSG00000101246	-0,214186	5,74525658	1,61953696	0,23421095	0,53570666	ARFRP1	ADP ribosylation factor related protein 1 [Source:HGNC Symbol;Acc:HGNC:662]
ENSG00000196659	0,40741792	0,72047005	1,61927725	0,23424561	0,53570666	TTC30B	tetratricopeptide repeat domain 30B [Source:HGNC Symbol;Acc:HGNC:26425]
ENSG00000170264	-0,301378	1,30479465	1,61893673	0,23429108	0,53572677	FAM161A	FAM161 centrosomal protein A [Source:HGNC Symbol;Acc:HGNC:25808]
ENSG00000083720	0,3079181	2,37704102	1,61853487	0,23434475	0,53576564	OXCT1	3-oxoacid CoA-transferase 1 [Source:HGNC Symbol;Acc:HGNC:8527]
ENSG00000287222	-0,316059	1,00598463	1,61756177	0,23447478	0,53597905	AC234917.3	novel transcript, antisense to PUF60
ENSG00000089682	-0,2077251	2,68288714	1,61692257	0,23456025	0,53600668	RBM41	RNA binding motif protein 41 [Source:HGNC Symbol;Acc:HGNC:25617]
ENSG00000213970	-0,3529321	0,69849855	1,61704514	0,23454386	0,53600668	AC006122.1	ribosomal protein S15 (RPS15) pseudogene
ENSG00000001629	-0,1988354	3,02742993	1,61584675	0,2347042	0,53617306	ANKIB1	ankyrin repeat and IBR domain containing 1 [Source:HGNC Symbol;Acc:HGNC:22215]
ENSG00000082515	0,17014046	6,33913162	1,61582983	0,23470646	0,53617306	MRPL22	mitochondrial ribosomal protein L22 [Source:HGNC Symbol;Acc:HGNC:14480]
ENSG00000269743	-0,3474951	0,30531038	1,61542842	0,23476021	0,53621198	SLC25A53	solute carrier family 25 member 53 [Source:HGNC Symbol;Acc:HGNC:31894]
ENSG00000277203	0,20739636	3,0657664	1,61505081	0,23481078	0,53624365	F8A2	coagulation factor VIII associated 2 [Source:HGNC Symbol;Acc:HGNC:31849]
ENSG00000180592	-0,3468258	0,28325965	1,61389774	0,23496529	0,53642881	SKIDA1	SKI/DACH domain containing 1 [Source:HGNC Symbol;Acc:HGNC:32697]
ENSG00000281398	0,2462297	2,87198992	1,61400758	0,23495057	0,53642881	SNHG4	small nucleolar RNA host gene 4 [Source:HGNC Symbol;Acc:HGNC:32964]
ENSG00000215068	0,5166055	-0,7620026	1,61351204	0,23501701	0,53646304	AC025171.2	novel transcript, antisense to ANXA2R
ENSG00000103343	-0,1912542	3,90237199	1,61318229	0,23506123	0,53648017	ZNF174	zinc finger protein 174 [Source:HGNC Symbol;Acc:HGNC:12963]
ENSG00000198677	0,21416776	4,77127976	1,61208566	0,23520839	0,53673218	TTC37	tetratricopeptide repeat domain 37 [Source:HGNC Symbol;Acc:HGNC:23639]
ENSG00000232098	0,31405455	1,44016077	1,61160277	0,23527324	0,5367963	AC012313.1	novel transcript
ENSG00000197150	-0,1716446	5,7042773	1,61079759	0,23538141	0,53695925	ABCB8	ATP binding cassette subfamily B member 8 [Source:HGNC Symbol;Acc:HGNC:49]
ENSG00000071994	-0,1742121	6,89649969	1,60821979	0,23572818	0,53725823	PDCD2	programmed cell death 2 [Source:HGNC Symbol;Acc:HGNC:8762]
ENSG00000089505	0,73687759	-1,3645685	1,60943488	0,23556464	0,53725823	CMTM1	CKLF like MARVEL transmembrane domain containing 1 [Source:HGNC Symbol;Acc:HGNC:19172]
ENSG00000139182	0,33532151	5,79788299	1,60924334	0,23559041	0,53725823	CLSTN3	calsyntenin 3 [Source:HGNC Symbol;Acc:HGNC:18371]
ENSG00000146950	0,42657392	1,38083591	1,60833175	0,23571311	0,53725823	SHROOM2	shroom family member 2 [Source:HGNC Symbol;Acc:HGNC:630]
ENSG00000260565	-0,4344191	-0,132688	1,60882077	0,23564727	0,53725823	ERVK13-1	endogenous retrovirus group K13 member 1 [Source:HGNC Symbol;Acc:HGNC:27548]
ENSG00000266338	0,25376925	1,68076542	1,60818308	0,23573313	0,53725823	NBPF15	NBPF member 15 [Source:HGNC Symbol;Acc:HGNC:28791]
ENSG00000123405	0,30645617	2,30044254	1,60691834	0,23590353	0,53756272	NFE2	nuclear factor, erythroid 2 [Source:HGNC Symbol;Acc:HGNC:7780]
ENSG00000130810	-0,3204107	1,84110491	1,60652168	0,235957	0,53760072	PPAN	peter pan homolog [Source:HGNC Symbol;Acc:HGNC:9227]
ENSG00000165819	-0,2238925	6,29265693	1,60615602	0,23600631	0,53762923	METTL3	methyltransferase like 3 [Source:HGNC Symbol;Acc:HGNC:17563]
ENSG00000114503	0,19710346	6,1837877	1,6054484	0,23610178	0,53766417	NCBP2	nuclear cap binding protein subunit 2 [Source:HGNC Symbol;Acc:HGNC:7659]
ENSG00000136643	0,22721445	4,10219785	1,60512828	0,23614499	0,53766417	RPS6KC1	ribosomal protein S6 kinase C1 [Source:HGNC Symbol;Acc:HGNC:10439]
ENSG00000164031	0,20915118	3,78793803	1,60486801	0,23618013	0,53766417	DNAJB14	DnaJ heat shock protein family (Hsp40) member B14 [Source:HGNC Symbol;Acc:HGNC:25881]
ENSG00000204869	-0,6125835	-0,7356222	1,60485442	0,23618196	0,53766417	IGFL4	IGF like family member 4 [Source:HGNC Symbol;Acc:HGNC:32931]
ENSG00000213606	-0,7222327	-0,7811803	1,60467882	0,23620567	0,53766417	AKR1B10P1	aldo-keto reductase family 1 member B10 pseudogene 1 [Source:HGNC Symbol;Acc:HGNC:45062]
ENSG00000267757	-0,760752	-1,2675225	1,60418725	0,23627206	0,53773151	EML2-AS1	EML2 antisense RNA 1 [Source:HGNC Symbol;Acc:HGNC:48331]
ENSG00000023318	-0,2188144	7,26676937	1,60368411	0,23634004	0,53780244	ERP44	endoplasmic reticulum protein 44 [Source:HGNC Symbol;Acc:HGNC:18311]
ENSG00000099889	-0,2138753	3,5623709	1,60285777	0,23645175	0,53797284	ARVCF	ARVCF delta catenin family member [Source:HGNC Symbol;Acc:HGNC:728]
ENSG00000105705	-0,2243991	4,85089207	1,60194285	0,23657551	0,53808683	SUGP1	SURP and G-patch domain containing 1 [Source:HGNC Symbol;Acc:HGNC:18643]
ENSG00000159692	0,18258572	6,01467518	1,60220357	0,23654024	0,53808683	CTBP1	C-terminal binding protein 1 [Source:HGNC Symbol;Acc:HGNC:2494]

ENSG00000124164	-0,1789584	6,3234804	1,6013179	0,2366601	0,53819543	VAPB	VAMP associated protein B and C [Source:HGNC Symbol;Acc:HGNC:12649]
ENSG00000125304	0,23487628	7,07918272	1,59985642	0,23685809	0,53831049	TM9SF2	transmembrane 9 superfamily member 2 [Source:HGNC Symbol;Acc:HGNC:11865]
ENSG00000134070	0,27656381	4,65743991	1,59997335	0,23684224	0,53831049	IRAK2	interleukin 1 receptor associated kinase 2 [Source:HGNC Symbol;Acc:HGNC:6113]
ENSG00000176142	-0,2101013	5,40146582	1,60038937	0,23678586	0,53831049	TMEM39A	transmembrane protein 39A [Source:HGNC Symbol;Acc:HGNC:25600]
ENSG00000267198	0,72761357	-1,7653249	1,60048944	0,23677231	0,53831049	AC091132.4	novel transcript
ENSG00000169689	-0,2199393	6,50631835	1,59949977	0,23690644	0,53833662	CENPX	centromere protein X [Source:HGNC Symbol;Acc:HGNC:11422]
ENSG00000100284	0,23559312	6,50201646	1,59886779	0,23699214	0,53844763	TOM1	target of myb1 membrane trafficking protein [Source:HGNC Symbol;Acc:HGNC:11982]
ENSG00000115009	-0,1818281	8,92434412	1,59797852	0,23711282	0,53853766	CCL20	C-C motif chemokine ligand 20 [Source:HGNC Symbol;Acc:HGNC:10619]
ENSG00000170037	-0,1982172	4,03166512	1,59777017	0,2371411	0,53853766	CNTROB	centrobin, centriole duplication and spindle assembly protein [Source:HGNC Symbol;Acc:HGNC:29616]
ENSG00000231369	-0,2360189	1,85697401	1,59776087	0,23714236	0,53853766	Z97353.1	ribosomal protein L15 (RPL15) pseudogene
ENSG00000060491	-0,166028	6,68556398	1,59698667	0,23724751	0,53860899	OGFR	opioid growth factor receptor [Source:HGNC Symbol;Acc:HGNC:15768]
ENSG00000230715	0,63986034	-0,4054925	1,59711535	0,23723003	0,53860899	AC018638.2	novel pseudogene
ENSG00000110497	-0,2122671	5,37481352	1,59611722	0,23736566	0,53862612	AMBRA1	autophagy and beclin 1 regulator 1 [Source:HGNC Symbol;Acc:HGNC:25990]
ENSG00000134313	0,26737005	5,28213516	1,59617968	0,23735717	0,53862612	KIDINS220	kinase D interacting substrate 220 [Source:HGNC Symbol;Acc:HGNC:29508]
ENSG00000141543	0,17442791	8,19018761	1,59648488	0,23731569	0,53862612	EIF4A3	eukaryotic translation initiation factor 4A3 [Source:HGNC Symbol;Acc:HGNC:18683]
ENSG00000254389	0,54262776	-0,5354889	1,59402414	0,23765044	0,53918858	RHPN1-AS1	RHPN1 antisense RNA 1 (head to head) [Source:HGNC Symbol;Acc:HGNC:28457]
ENSG00000116212	0,17191789	5,96795264	1,5932966	0,23774953	0,53928492	LRRC42	leucine rich repeat containing 42 [Source:HGNC Symbol;Acc:HGNC:28792]
ENSG00000172878	0,19821453	3,85004599	1,5931704	0,23776673	0,53928492	METAP1D	methionyl aminopeptidase type 1D, mitochondrial [Source:HGNC Symbol;Acc:HGNC:32583]
ENSG00000143878	-0,1634901	8,44615579	1,59071465	0,23810167	0,53996077	RHOB	ras homolog family member B [Source:HGNC Symbol;Acc:HGNC:668]
ENSG00000160593	-0,2359919	2,30411841	1,58991738	0,23821055	0,54001421	JAML	junction adhesion molecule like [Source:HGNC Symbol;Acc:HGNC:19084]
ENSG00000161203	0,24531453	7,23154128	1,58967376	0,23824383	0,54001421	AP2M1	adaptor related protein complex 2 subunit mu 1 [Source:HGNC Symbol;Acc:HGNC:564]
ENSG00000168264	0,17468847	7,33824369	1,59026705	0,23816279	0,54001421	IRF2BP2	interferon regulatory factor 2 binding protein 2 [Source:HGNC Symbol;Acc:HGNC:21729]
ENSG00000279691	0,63027461	-0,9645951	1,58945964	0,23827309	0,54001421	AC113410.3	TEC
ENSG00000100665	0,23424836	5,19009083	1,58769374	0,23851458	0,54037549	SERPINA4	serpin family A member 4 [Source:HGNC Symbol;Acc:HGNC:8948]
ENSG00000154124	0,22059358	4,1561345	1,58788371	0,23848859	0,54037549	OTULIN	OTU deubiquitinase with linear linkage specificity [Source:HGNC Symbol;Acc:HGNC:25118]
ENSG00000276805	0,71799295	0,03601778	1,58748268	0,23854347	0,54037549	AL133216.2	general transcription factor IIi (GTF2I) pseudogene
ENSG00000131153	0,25406858	6,76908094	1,58669525	0,23865128	0,54053589	GINS2	GINS complex subunit 2 [Source:HGNC Symbol;Acc:HGNC:24575]
ENSG00000110700	-0,1887096	9,51295453	1,58569861	0,23878783	0,54059876	RPS13	ribosomal protein S13 [Source:HGNC Symbol;Acc:HGNC:10386]
ENSG00000114030	-0,2546763	5,09039139	1,58513468	0,23886514	0,54059876	KPNA1	karyopherin subunit alpha 1 [Source:HGNC Symbol;Acc:HGNC:6394]
ENSG00000115539	-0,2270321	5,57732724	1,58502566	0,23888009	0,54059876	PDCL3	phosducin like 3 [Source:HGNC Symbol;Acc:HGNC:28860]
ENSG00000123545	0,16950883	5,51948773	1,58515204	0,23886276	0,54059876	NDUFAF4	NADH:ubiquinone oxidoreductase complex assembly factor 4 [Source:HGNC Symbol;Acc:HGNC:21034]
ENSG00000240950	-0,2583599	1,29952759	1,58516933	0,23886039	0,54059876	AC021074.1	ribosomal protein L8 (RPL8) pseudogene
ENSG00000254999	-0,2502694	5,61357262	1,58487274	0,23890106	0,54059876	BRK1	BRICK1 subunit of SCAR/WAVE actin nucleating complex [Source:HGNC Symbol;Acc:HGNC:23057]
ENSG00000165895	0,3415509	1,17212191	1,58457189	0,23894233	0,5406084	ARHGAP42	Rho GTPase activating protein 42 [Source:HGNC Symbol;Acc:HGNC:26545]
ENSG00000085719	0,22934136	4,5183154	1,58412543	0,23900359	0,54066327	CPNE3	copine 3 [Source:HGNC Symbol;Acc:HGNC:2316]
ENSG00000131848	0,24152129	3,05371807	1,58208327	0,23928406	0,54119985	ZSCAN5A	zinc finger and SCAN domain containing 5A [Source:HGNC Symbol;Acc:HGNC:23710]
ENSG00000253954	-0,5954591	-1,0066197	1,58185911	0,23931488	0,54119985	HMGN1P38	high mobility group nucleosome binding domain 1 pseudogene 38 [Source:HGNC Symbol;Acc:HGNC:39422]
ENSG00000152527	0,36297256	0,73108054	1,58129791	0,23939205	0,54129058	PLEKHH2	pleckstrin homology, MyTH4 and FERM domain containing H2 [Source:HGNC Symbol;Acc:HGNC:30506]
ENSG00000077232	-0,1662192	7,49390651	1,5805592	0,23949368	0,54143658	DNAJC10	DnaJ heat shock protein family (Hsp40) member C10 [Source:HGNC Symbol;Acc:HGNC:24637]
ENSG00000155189	0,18393364	5,72470998	1,57998393	0,23957287	0,5415318	AGPAT5	1-acylglycerol-3-phosphate O-acyltransferase 5 [Source:HGNC Symbol;Acc:HGNC:20886]
ENSG00000159200	0,17706496	5,1744452	1,57933875	0,23966172	0,54164883	RCAN1	regulator of calcineurin 1 [Source:HGNC Symbol;Acc:HGNC:3040]
ENSG00000121989	0,19281573	4,05336589	1,57880474	0,2397353	0,54167911	ACVR2A	activin A receptor type 2A [Source:HGNC Symbol;Acc:HGNC:173]
ENSG00000160049	-0,1769561	5,70578214	1,57870333	0,23974927	0,54167911	DFFA	DNA fragmentation factor subunit alpha [Source:HGNC Symbol;Acc:HGNC:2772]
ENSG00000115425	0,1771973	6,427839	1,57711409	0,23996844	0,54200664	PECR	peroxisomal trans-2-enoyl-CoA reductase [Source:HGNC Symbol;Acc:HGNC:18281]

ENSG00000185043	-0,1835284	6,68412358	1,57729937	0,23994287	0,54200664	CIB1	calcium and integrin binding 1 [Source:HGNC Symbol;Acc:HGNC:16920]
ENSG00000136908	-0,2008307	5,19163568	1,57683007	0,24000764	0,54201138	DPM2	dolichyl-phosphate mannosyltransferase subunit 2, regulatory [Source:HGNC Symbol;Acc:HGNC:3006]
ENSG00000071205	0,58106604	-0,7887322	1,57580304	0,24014945	0,54216402	ARHGAP10	Rho GTPase activating protein 10 [Source:HGNC Symbol;Acc:HGNC:26099]
ENSG00000102900	0,23192147	6,84528989	1,57589958	0,24013612	0,54216402	NUP93	nucleoporin 93 [Source:HGNC Symbol;Acc:HGNC:28958]
ENSG00000134575	-0,2377806	6,38129217	1,57483334	0,24028346	0,54231878	ACP2	acid phosphatase 2, lysosomal [Source:HGNC Symbol;Acc:HGNC:123]
ENSG00000233503	-0,6058236	-1,3584353	1,57476973	0,24029225	0,54231878	HNRNPLP1	heterogeneous nuclear ribonucleoprotein L pseudogene 1 [Source:HGNC Symbol;Acc:HGNC:48748]
ENSG00000164946	0,28003338	6,09352505	1,5732748	0,24049905	0,54270167	FREM1	FRAS1 related extracellular matrix 1 [Source:HGNC Symbol;Acc:HGNC:23399]
ENSG00000157212	0,19193327	3,78129486	1,57284373	0,24055872	0,5427525	PAXIP1	PAX interacting protein 1 [Source:HGNC Symbol;Acc:HGNC:8624]
ENSG00000113838	-0,2727943	2,49170095	1,57141612	0,24075651	0,54290368	TBCCD1	TBCC domain containing 1 [Source:HGNC Symbol;Acc:HGNC:25546]
ENSG00000122133	-0,4295605	1,74724872	1,57099925	0,2408143	0,54290368	PAEP	progestagen associated endometrial protein [Source:HGNC Symbol;Acc:HGNC:8573]
ENSG00000160218	0,2180491	3,4863355	1,57101427	0,24081222	0,54290368	TRAPPC10	trafficking protein particle complex 10 [Source:HGNC Symbol;Acc:HGNC:11868]
ENSG00000169372	0,19226567	3,853379	1,57078188	0,24084445	0,54290368	CRADD	CASP2 and RIPK1 domain containing adaptor with death domain [Source:HGNC Symbol;Acc:HGNC:2340]
ENSG00000186318	-0,2195232	2,93813651	1,57076289	0,24084708	0,54290368	BACE1	beta-secretase 1 [Source:HGNC Symbol;Acc:HGNC:933]
ENSG00000232917	-0,3174024	0,59151418	1,57075116	0,2408487	0,54290368	HSPE1P6	heat shock protein family E (Hsp10) member 1 pseudogene 6 [Source:HGNC Symbol;Acc:HGNC:49325]
ENSG00000055044	-0,1918349	6,98720193	1,56976623	0,24098536	0,543051	NOP58	NOP58 ribonucleoprotein [Source:HGNC Symbol;Acc:HGNC:29926]
ENSG00000140941	-0,1676331	6,25051001	1,56974428	0,24098841	0,543051	MAP1LC3B	microtubule associated protein 1 light chain 3 beta [Source:HGNC Symbol;Acc:HGNC:13352]
ENSG00000029639	0,18903156	6,50460623	1,56865299	0,24113995	0,54328313	TFB1M	transcription factor B1, mitochondrial [Source:HGNC Symbol;Acc:HGNC:17037]
ENSG00000116525	0,31652565	0,81333045	1,56846694	0,24116579	0,54328313	TRIM62	tripartite motif containing 62 [Source:HGNC Symbol;Acc:HGNC:25574]
ENSG00000048162	-0,175986	6,66294728	1,56782466	0,24125506	0,54340042	NOP16	NOP16 nucleolar protein [Source:HGNC Symbol;Acc:HGNC:26934]
ENSG00000005801	-0,1913302	4,11940617	1,56539725	0,24159282	0,54357455	ZNF195	zinc finger protein 195 [Source:HGNC Symbol;Acc:HGNC:12986]
ENSG00000011523	-0,2331136	3,97006437	1,56689274	0,24138465	0,54357455	CEP68	centrosomal protein 68 [Source:HGNC Symbol;Acc:HGNC:29076]
ENSG00000101911	0,21628171	4,26944116	1,56564996	0,24155763	0,54357455	PRPS2	phosphoribosyl pyrophosphate synthetase 2 [Source:HGNC Symbol;Acc:HGNC:9465]
ENSG00000166387	-0,2274166	3,7375092	1,56563122	0,24156024	0,54357455	PPFIBP2	PPFIA binding protein 2 [Source:HGNC Symbol;Acc:HGNC:9250]
ENSG00000233111	0,69250478	-1,8385968	1,56610049	0,2414949	0,54357455	RAB1C	RAB1C, member RAS oncogene family pseudogene [Source:HGNC Symbol;Acc:HGNC:23683]
ENSG00000244405	0,18552973	5,02557907	1,565944	0,24151669	0,54357455	ETV5	ETS variant transcription factor 5 [Source:HGNC Symbol;Acc:HGNC:3494]
ENSG00000277053	0,26383327	1,91022142	1,56580832	0,24153558	0,54357455	GTF2IP1	general transcription factor IIi pseudogene 1 [Source:HGNC Symbol;Acc:HGNC:4660]
ENSG00000272476	-0,3958441	0,06430755	1,5639473	0,24179489	0,54394542	AL024507.2	novel transcript, antisense to SEC63
ENSG00000141452	0,19049086	4,37744741	1,56322763	0,24189527	0,54408745	RMC1	regulator of MON1-CCZ1 [Source:HGNC Symbol;Acc:HGNC:24326]
ENSG00000130158	-0,2510611	3,89803456	1,55898397	0,24248835	0,54533748	DOCK6	dedicator of cytokinesis 6 [Source:HGNC Symbol;Acc:HGNC:19189]
ENSG00000173744	0,18692797	6,45481947	1,55793072	0,24263586	0,54550127	AGFG1	ArfGAP with FG repeats 1 [Source:HGNC Symbol;Acc:HGNC:5175]
ENSG00000188486	-0,2056821	7,35299287	1,5580476	0,24261948	0,54550127	H2AX	H2A.X variant histone [Source:HGNC Symbol;Acc:HGNC:4739]
ENSG00000177946	-0,1996752	4,79546152	1,55733934	0,24271874	0,54554073	CENPBD1	CENPB DNA-binding domain containing 1 [Source:HGNC Symbol;Acc:HGNC:28272]
ENSG00000188112	-0,4650982	0,35172604	1,55718961	0,24273973	0,54554073	C6orf132	chromosome 6 open reading frame 132 [Source:HGNC Symbol;Acc:HGNC:21288]
ENSG00000197976	-0,1822492	5,27638977	1,55673993	0,24280278	0,54554073	AKAP17A	A-kinase anchoring protein 17A [Source:HGNC Symbol;Acc:HGNC:18783]
ENSG00000285679	0,64287294	-1,5546431	1,5568499	0,24278736	0,54554073	AC079142.1	novel transcript
ENSG00000266680	-0,4102127	0,45384735	1,5561502	0,2428855	0,54564267	AL135905.1	novel transcript, antisense to PTP4A1
ENSG00000214144	-0,4360132	-0,1012847	1,55566086	0,24295418	0,54571303	AL390728.1	heat shock 70kDa protein 8 (HSPA8) pseudogene
ENSG00000154920	0,18451517	3,58360165	1,5548396	0,24306949	0,54571803	EME1	essential meiotic structure-specific endonuclease 1 [Source:HGNC Symbol;Acc:HGNC:24965]
ENSG00000156469	0,18672966	4,55236459	1,55458093	0,24310582	0,54571803	MTERF3	mitochondrial transcription termination factor 3 [Source:HGNC Symbol;Acc:HGNC:24258]
ENSG00000198870	-0,791389	-0,5486048	1,55510922	0,24303162	0,54571803	STKLD1	serine/threonine kinase like domain containing 1 [Source:HGNC Symbol;Acc:HGNC:28669]
ENSG00000243742	-0,6918641	-2,0671634	1,55506817	0,24303739	0,54571803	RPLP0P2	ribosomal protein lateral stalk subunit P0 pseudogene 2 [Source:HGNC Symbol;Acc:HGNC:17960]
ENSG00000243199	-0,2326167	3,28651441	1,5539887	0,24318904	0,54582097	AC115223.1	ribosomal protein L6 (RPL6) pseudogene
ENSG00000154640	-0,1841619	4,32573936	1,55326642	0,24329059	0,545965	BTG3	BTG anti-proliferation factor 3 [Source:HGNC Symbol;Acc:HGNC:1132]
ENSG00000124508	0,18688967	3,6709053	1,55279997	0,24335619	0,54602836	BTN2A2	butyrophilin subfamily 2 member A2 [Source:HGNC Symbol;Acc:HGNC:1137]

ENSG00000138796	0,16872247	6,33895327	1,5520496	0,24346179	0,5461814	HADH	hydroxyacyl-CoA dehydrogenase [Source:HGNC Symbol;Acc:HGNC:4799]
ENSG00000104691	0,18513043	5,51454293	1,55084251	0,24363179	0,54647886	UBXN8	UBX domain protein 8 [Source:HGNC Symbol;Acc:HGNC:30307]
ENSG00000143190	0,49823427	2,37177159	1,54886364	0,24391084	0,54680665	POU2F1	POU class 2 homeobox 1 [Source:HGNC Symbol;Acc:HGNC:9212]
ENSG00000155957	0,26287672	1,74037869	1,54712537	0,24415632	0,54680665	TMBIM4	transmembrane BAX inhibitor motif containing 4 [Source:HGNC Symbol;Acc:HGNC:24257]
ENSG00000161513	-0,1799599	6,93407017	1,54799878	0,24403293	0,54680665	FDXR	ferredoxin reductase [Source:HGNC Symbol;Acc:HGNC:3642]
ENSG00000181396	0,16247246	6,47585161	1,54753465	0,24409849	0,54680665	OGFOD3	2-oxoglutarate and iron dependent oxygenase domain containing 3 [Source:HGNC Symbol;Acc:HGNC:26174]
ENSG00000188681	-0,3008771	1,97929939	1,54641728	0,24425642	0,54680665	TEKT4P2	tektin 4 pseudogene 2 [Source:HGNC Symbol;Acc:HGNC:40046]
ENSG00000197472	0,29567281	1,09399776	1,54639518	0,24425954	0,54680665	ZNF695	zinc finger protein 695 [Source:HGNC Symbol;Acc:HGNC:30954]
ENSG00000205250	0,18381702	6,49916709	1,54802715	0,24402893	0,54680665	E2F4	E2F transcription factor 4 [Source:HGNC Symbol;Acc:HGNC:3118]
ENSG00000205726	-0,2519082	3,96763582	1,54797519	0,24403626	0,54680665	ITSN1	intersectin 1 [Source:HGNC Symbol;Acc:HGNC:6183]
ENSG00000220201	-0,5700222	-1,0814341	1,54906089	0,243883	0,54680665	ZGLP1	zinc finger GATA like protein 1 [Source:HGNC Symbol;Acc:HGNC:37245]
ENSG00000225695	0,40281591	-0,2218476	1,54636006	0,24426451	0,54680665	HNRNPA1P35	heterogeneous nuclear ribonucleoprotein A1 pseudogene 35 [Source:HGNC Symbol;Acc:HGNC:48764]
ENSG00000245498	-0,6007307	-1,1657303	1,54645162	0,24425156	0,54680665	AP000866.1	novel transcript, antisense to C11orf61
ENSG00000258947	-0,3338149	0,53772554	1,54645426	0,24425119	0,54680665	TUBB3	tubulin beta 3 class III [Source:HGNC Symbol;Acc:HGNC:20772]
ENSG00000278384	0,69802806	-1,5490571	1,54849255	0,24396321	0,54680665	nan	nan
ENSG00000115548	-0,239729	4,86267227	1,54506178	0,24444819	0,54707412	KDM3A	lysine demethylase 3A [Source:HGNC Symbol;Acc:HGNC:20815]
ENSG00000217325	-0,4970308	-0,9149189	1,54498621	0,24445889	0,54707412	PRELID1P1	PRELID1 pseudogene 1 [Source:HGNC Symbol;Acc:HGNC:43886]
ENSG00000007545	0,28145556	2,55362807	1,5447048	0,24449873	0,54707948	CRAMP1	cramped chromatin regulator homolog 1 [Source:HGNC Symbol;Acc:HGNC:14122]
ENSG00000153046	-0,1805509	4,56074129	1,54429459	0,24455682	0,54712566	CDYL	chromodomain Y like [Source:HGNC Symbol;Acc:HGNC:1811]
ENSG00000246560	0,43069428	0,36910806	1,54331	0,24469633	0,54735396	UBE2D3-AS1	UBE2D3 antisense RNA 1 [Source:HGNC Symbol;Acc:HGNC:54095]
ENSG00000112701	-0,1914188	5,00232108	1,54090012	0,24503827	0,54795102	SENP6	SUMO specific peptidase 6 [Source:HGNC Symbol;Acc:HGNC:20944]
ENSG00000196576	0,25006631	7,20794383	1,54114313	0,24500376	0,54795102	PLXNB2	plexin B2 [Source:HGNC Symbol;Acc:HGNC:9104]
ENSG00000119723	-0,2195258	3,99151718	1,54053542	0,24509007	0,54796346	COQ6	coenzyme Q6, monooxygenase [Source:HGNC Symbol;Acc:HGNC:20233]
ENSG00000168101	-0,1924341	5,58977065	1,54033288	0,24511885	0,54796346	NUDT16L1	nudix hydrolase 16 like 1 [Source:HGNC Symbol;Acc:HGNC:28154]
ENSG00000101278	-0,2855828	1,16245059	1,539378	0,24525458	0,548183	RPS10P5	ribosomal protein S10 pseudogene 5 [Source:HGNC Symbol;Acc:HGNC:15795]
ENSG00000065000	-0,3357724	6,99049382	1,53762312	0,24550429	0,54865721	AP3D1	adaptor related protein complex 3 subunit delta 1 [Source:HGNC Symbol;Acc:HGNC:568]
ENSG00000243147	-0,1971301	6,78115353	1,53728345	0,24555267	0,54868139	MRPL33	mitochondrial ribosomal protein L33 [Source:HGNC Symbol;Acc:HGNC:14487]
ENSG00000185359	-0,3059212	6,25336062	1,53568664	0,24578026	0,54910594	HGS	hepatocyte growth factor-regulated tyrosine kinase substrate [Source:HGNC Symbol;Acc:HGNC:4897]
ENSG00000234093	-0,2144374	2,44861371	1,53400131	0,24602078	0,54955926	RPS15AP11	ribosomal protein S15a pseudogene 11 [Source:HGNC Symbol;Acc:HGNC:36707]
ENSG00000135766	-0,2337053	2,1482356	1,53365126	0,24607078	0,54958691	EGLN1	egl-9 family hypoxia inducible factor 1 [Source:HGNC Symbol;Acc:HGNC:1232]
ENSG00000100296	0,16122283	6,0977628	1,53273457	0,24620177	0,54965171	THOC5	THO complex 5 [Source:HGNC Symbol;Acc:HGNC:19074]
ENSG00000105695	-0,3414445	0,34218491	1,53309784	0,24614985	0,54965171	MAG	myelin associated glycoprotein [Source:HGNC Symbol;Acc:HGNC:6783]
ENSG00000232671	-0,6693355	-0,4413566	1,53265839	0,24621266	0,54965171	ZNF687-AS1	ZNF687 antisense RNA 1 [Source:HGNC Symbol;Acc:HGNC:54048]
ENSG00000139880	0,37959515	0,57943158	1,53202188	0,24630369	0,5497709	CDH24	cadherin 24 [Source:HGNC Symbol;Acc:HGNC:14265]
ENSG00000215086	0,55405311	-0,116342	1,52995943	0,24659894	0,55034216	NPM1P24	nucleophosmin 1 pseudogene 24 [Source:HGNC Symbol;Acc:HGNC:45097]
ENSG00000236404	-0,5402496	-0,9626176	1,52970804	0,24663496	0,55034216	VLDLR-AS1	VLDLR antisense RNA 1 [Source:HGNC Symbol;Acc:HGNC:49621]
ENSG00000149548	0,3586604	0,91720692	1,52928184	0,24669605	0,55035354	CCDC15	coiled-coil domain containing 15 [Source:HGNC Symbol;Acc:HGNC:25798]
ENSG00000174953	0,25105561	5,19544939	1,52914682	0,2467154	0,55035354	DHX36	DEAH-box helicase 36 [Source:HGNC Symbol;Acc:HGNC:14410]
ENSG00000116704	-0,1773724	4,62203595	1,52886376	0,24675599	0,55036004	SLC35D1	solute carrier family 35 member D1 [Source:HGNC Symbol;Acc:HGNC:20800]
ENSG00000075391	0,32371877	2,98410873	1,52621897	0,24713566	0,55107235	RASAL2	RAS protein activator like 2 [Source:HGNC Symbol;Acc:HGNC:9874]
ENSG00000101017	-0,1813423	4,59991447	1,52611362	0,2471508	0,55107235	CD40	CD40 molecule [Source:HGNC Symbol;Acc:HGNC:11919]
ENSG00000269044	-0,5155731	-1,0641728	1,52480384	0,24733914	0,55140812	AC024075.2	novel transcript, sense intronic C19orf42
ENSG00000180530	0,18710159	4,68937982	1,52386338	0,24747449	0,55145742	NRIP1	nuclear receptor interacting protein 1 [Source:HGNC Symbol;Acc:HGNC:8001]
ENSG00000242960	0,40626893	0,69643142	1,52425408	0,24741825	0,55145742	FTH1P23	ferritin heavy chain 1 pseudogene 23 [Source:HGNC Symbol;Acc:HGNC:37641]

ENSG00000265451	-0,7158317	-1,7820291	1,52411443	0,24743835	0,55145742	AC012447.1	novel transcript, antisense to CLASP1
ENSG00000067208	-0,3022103	1,63386112	1,52356353	0,24751767	0,55146951	EVI5	ecotropic viral integration site 5 [Source:HGNC Symbol;Acc:HGNC:3501]
ENSG00000132376	0,20589411	3,01289469	1,52329353	0,24755655	0,55147205	INPP5K	inositol polyphosphate-5-phosphatase K [Source:HGNC Symbol;Acc:HGNC:33882]
ENSG00000249115	-0,2201482	3,4237981	1,52206473	0,24773364	0,5517824	HAUS5	HAUS augmin like complex subunit 5 [Source:HGNC Symbol;Acc:HGNC:29130]
ENSG00000140548	-0,2310794	3,92121251	1,52141307	0,24782763	0,55182347	ZNF710	zinc finger protein 710 [Source:HGNC Symbol;Acc:HGNC:25352]
ENSG00000249855	-0,2169259	3,74525701	1,52157296	0,24780456	0,55182347	EEF1A1P19	eukaryotic translation elongation factor 1 alpha 1 pseudogene 19 [Source:HGNC Symbol;Acc:HGNC:37892]
ENSG00000143756	0,18687166	4,27579916	1,5201782	0,24800586	0,55205205	FBXO28	F-box protein 28 [Source:HGNC Symbol;Acc:HGNC:29046]
ENSG00000224914	0,34169229	0,44713398	1,52043856	0,24796827	0,55205205	LINC00863	long intergenic non-protein coding RNA 863 [Source:HGNC Symbol;Acc:HGNC:45162]
ENSG00000113593	0,19111462	4,67513226	1,51862394	0,24823044	0,55246779	PPWD1	peptidylprolyl isomerase domain and WD repeat containing 1 [Source:HGNC Symbol;Acc:HGNC:28954]
ENSG00000154914	0,36932659	1,32427342	1,51794867	0,24832811	0,55260096	USP43	ubiquitin specific peptidase 43 [Source:HGNC Symbol;Acc:HGNC:20072]
ENSG00000104205	-0,28918	1,87197867	1,51764088	0,24837264	0,55261588	SGK3	serum/glucocorticoid regulated kinase family member 3 [Source:HGNC Symbol;Acc:HGNC:10812]
ENSG00000152939	0,20534559	3,49320367	1,51593494	0,24861966	0,55308125	MARVELD2	MARVEL domain containing 2 [Source:HGNC Symbol;Acc:HGNC:26401]
ENSG00000140534	0,27424057	4,14095353	1,51460582	0,24881235	0,55334139	TICRR	TOPBP1 interacting checkpoint and replication regulator [Source:HGNC Symbol;Acc:HGNC:28704]
ENSG00000149636	0,17865502	5,55878507	1,51472266	0,2487954	0,55334139	DSN1	DSN1 component of MIS12 kinetochore complex [Source:HGNC Symbol;Acc:HGNC:16165]
ENSG00000130413	0,23943797	2,64105736	1,51414554	0,24887913	0,55340566	STK33	serine/threonine kinase 33 [Source:HGNC Symbol;Acc:HGNC:14568]
ENSG00000071282	0,26044966	2,16278387	1,51365098	0,24895091	0,55341146	LMCD1	LIM and cysteine rich domains 1 [Source:HGNC Symbol;Acc:HGNC:6633]
ENSG00000143278	-0,5199882	-0,1915099	1,51360556	0,2489575	0,55341146	F13B	coagulation factor XIII B chain [Source:HGNC Symbol;Acc:HGNC:3534]
ENSG00000013297	0,34337124	1,08084937	1,51143071	0,2492735	0,55390435	CLDN11	claudin 11 [Source:HGNC Symbol;Acc:HGNC:8514]
ENSG00000036549	0,18341473	4,38155196	1,51077555	0,24936881	0,55390435	ZZZ3	zinc finger ZZ-type containing 3 [Source:HGNC Symbol;Acc:HGNC:24523]
ENSG00000112208	-0,1952009	5,81714427	1,51081869	0,24936253	0,55390435	BAG2	BAG cochaperone 2 [Source:HGNC Symbol;Acc:HGNC:938]
ENSG00000162366	0,6643066	-0,8388854	1,51081485	0,24936309	0,55390435	PDZK1IP1	PDZK1 interacting protein 1 [Source:HGNC Symbol;Acc:HGNC:16887]
ENSG00000232134	-0,3118549	0,82083549	1,51082817	0,24936115	0,55390435	RPS15AP12	ribosomal protein S15a pseudogene 12 [Source:HGNC Symbol;Acc:HGNC:36759]
ENSG00000141627	0,20052087	4,80987834	1,50865435	0,24967771	0,55447613	DYM	dymeclin [Source:HGNC Symbol;Acc:HGNC:21317]
ENSG00000167775	-0,1671947	8,22048984	1,50848681	0,24970214	0,55447613	CD320	CD320 molecule [Source:HGNC Symbol;Acc:HGNC:16692]
ENSG00000222011	0,22447916	2,20241987	1,50821116	0,24974232	0,55448109	FAM185A	family with sequence similarity 185 member A [Source:HGNC Symbol;Acc:HGNC:22412]
ENSG00000119446	-0,2221821	4,41188534	1,50617435	0,25003954	0,55480372	RBM18	RNA binding motif protein 18 [Source:HGNC Symbol;Acc:HGNC:28413]
ENSG00000127948	0,24010121	8,61690807	1,50686025	0,2499394	0,55480372	POR	cytochrome p450 oxidoreductase [Source:HGNC Symbol;Acc:HGNC:9208]
ENSG00000142279	0,21426647	3,21679517	1,50634762	0,25001424	0,55480372	WTIP	WT1 interacting protein [Source:HGNC Symbol;Acc:HGNC:20964]
ENSG00000147180	0,55631177	-0,7767906	1,50654281	0,24998574	0,55480372	ZNF711	zinc finger protein 711 [Source:HGNC Symbol;Acc:HGNC:13128]
ENSG00000131725	0,20642386	2,33718714	1,50577343	0,25009811	0,55484939	WDR44	WD repeat domain 44 [Source:HGNC Symbol;Acc:HGNC:30512]
ENSG00000133193	-0,2148375	4,77431488	1,50528453	0,25016955	0,55492361	FAM104A	family with sequence similarity 104 member A [Source:HGNC Symbol;Acc:HGNC:25918]
ENSG00000102393	-0,171911	6,32342644	1,50368014	0,25040418	0,55494503	GLA	galactosidase alpha [Source:HGNC Symbol;Acc:HGNC:4296]
ENSG00000114315	-0,2760358	5,00939304	1,50471126	0,25025335	0,55494503	HES1	hes family bHLH transcription factor 1 [Source:HGNC Symbol;Acc:HGNC:5192]
ENSG00000115825	0,19481256	3,90790032	1,50318845	0,25047615	0,55494503	PRKD3	protein kinase D3 [Source:HGNC Symbol;Acc:HGNC:9408]
ENSG00000168894	-0,200523	6,07703635	1,50314102	0,25048309	0,55494503	RNF181	ring finger protein 181 [Source:HGNC Symbol;Acc:HGNC:28037]
ENSG00000170325	0,3009869	2,20175904	1,50330221	0,25045949	0,55494503	PRDM10	PR/SET domain 10 [Source:HGNC Symbol;Acc:HGNC:13995]
ENSG00000174007	0,49845941	-1,1507875	1,5035722	0,25041998	0,55494503	CEP19	centrosomal protein 19 [Source:HGNC Symbol;Acc:HGNC:28209]
ENSG00000239306	0,2380283	5,9444206	1,50405886	0,25034877	0,55494503	RBM14	RNA binding motif protein 14 [Source:HGNC Symbol;Acc:HGNC:14219]
ENSG00000241556	-0,3365008	0,83262714	1,50338567	0,25044728	0,55494503	AC018475.1	ribosomal protein L29 (RPL29) pseudogene
ENSG00000197013	-0,6331599	-0,7603147	1,50249116	0,25057826	0,55507169	ZNF429	zinc finger protein 429 [Source:HGNC Symbol;Acc:HGNC:20817]
ENSG00000115839	0,22881279	5,40555448	1,50222799	0,25061681	0,55507293	RAB3GAP1	RAB3 GTPase activating protein catalytic subunit 1 [Source:HGNC Symbol;Acc:HGNC:17063]
ENSG00000181555	0,25514377	5,56729428	1,5015058	0,25072265	0,55522317	SETD2	SET domain containing 2, histone lysine methyltransferase [Source:HGNC Symbol;Acc:HGNC:18420]
ENSG00000116786	-0,1630422	5,891841	1,49902802	0,25108626	0,55552312	PLEKHM2	pleckstrin homology and RUN domain containing M2 [Source:HGNC Symbol;Acc:HGNC:29131]
ENSG00000166226	0,23854924	8,19685286	1,49916453	0,25106621	0,55552312	CCT2	chaperonin containing TCP1 subunit 2 [Source:HGNC Symbol;Acc:HGNC:1615]

ENSG00000169018	0,19403153	3,83917465	1,49981994	0,25096997	0,55552312	FEM1B	fem-1 homolog B [Source:HGNC Symbol;Acc:HGNC:3649]
ENSG00000170365	-0,2237794	2,96649419	1,49957873	0,25100538	0,55552312	SMAD1	SMAD family member 1 [Source:HGNC Symbol;Acc:HGNC:6767]
ENSG00000229589	0,72905418	-1,935629	1,49903918	0,25108462	0,55552312	ACVR2B-AS1	ACVR2B antisense RNA 1 [Source:HGNC Symbol;Acc:HGNC:44161]
ENSG00000235560	-0,3707105	0,83887683	1,49971643	0,25098516	0,55552312	AC002310.1	novel transcript, antisense to ZNF747
ENSG00000137947	0,18123142	5,38285761	1,49872411	0,2511309	0,55553776	GTF2B	general transcription factor IIB [Source:HGNC Symbol;Acc:HGNC:4648]
ENSG00000100445	-0,1639829	5,83321626	1,49823939	0,25120214	0,55561121	SDR39U1	short chain dehydrogenase/reductase family 39U member 1 [Source:HGNC Symbol;Acc:HGNC:20275]
ENSG00000162729	0,1936741	6,13369076	1,49768018	0,25128435	0,55570892	IGSF8	immunoglobulin superfamily member 8 [Source:HGNC Symbol;Acc:HGNC:17813]
ENSG00000128000	0,24172839	1,61069714	1,49673444	0,25142348	0,55593244	ZNF780B	zinc finger protein 780B [Source:HGNC Symbol;Acc:HGNC:33109]
ENSG00000139517	-0,2226025	3,35977446	1,49550688	0,25160423	0,5560796	LNX2	ligand of numb-protein X 2 [Source:HGNC Symbol;Acc:HGNC:20421]
ENSG00000163687	-0,5304005	-1,2956786	1,49559777	0,25159084	0,5560796	DNASE1L3	deoxyribonuclease 1 like 3 [Source:HGNC Symbol;Acc:HGNC:2959]
ENSG00000174669	0,30392837	1,60569743	1,49574917	0,25156854	0,5560796	SLC29A2	solute carrier family 29 member 2 [Source:HGNC Symbol;Acc:HGNC:11004]
ENSG00000069424	-0,2811577	3,18370369	1,49498898	0,25168054	0,55613973	KCNAB2	potassium voltage-gated channel subfamily A regulatory beta subunit 2 [Source:HGNC Symbol;Acc:HGNC:6229]
ENSG00000163348	0,17395802	4,74413452	1,49454731	0,25174564	0,55613973	PYGO2	pygopus family PHD finger 2 [Source:HGNC Symbol;Acc:HGNC:30257]
ENSG00000273010	-0,7013646	-1,8848554	1,4945864	0,25173988	0,55613973	AL360270.3	novel transcript, antisense to LRIF1
ENSG00000124209	0,21257157	4,06696454	1,49342397	0,25191132	0,55626435	RAB22A	RAB22A, member RAS oncogene family [Source:HGNC Symbol;Acc:HGNC:9764]
ENSG00000261485	-0,6028793	-1,6356636	1,49339087	0,25191621	0,55626435	PAN3-AS1	PAN3 antisense RNA 1 [Source:HGNC Symbol;Acc:HGNC:39932]
ENSG00000272047	-0,2325318	5,4565038	1,49339037	0,25191628	0,55626435	GTF2H5	general transcription factor IIH subunit 5 [Source:HGNC Symbol;Acc:HGNC:21157]
ENSG00000054267	0,32584786	3,44344179	1,49215738	0,25209832	0,55658219	ARID4B	AT-rich interaction domain 4B [Source:HGNC Symbol;Acc:HGNC:15550]
ENSG00000159674	0,18283494	4,93718043	1,49184067	0,25214511	0,55660137	SPON2	spondin 2 [Source:HGNC Symbol;Acc:HGNC:11253]
ENSG00000100422	0,18373882	5,25901094	1,49096595	0,2522744	0,55673255	CERK	ceramide kinase [Source:HGNC Symbol;Acc:HGNC:19256]
ENSG00000196511	-0,6348561	-1,5095058	1,49092297	0,25228075	0,55673255	TPK1	thiamin pyrophosphokinase 1 [Source:HGNC Symbol;Acc:HGNC:17358]
ENSG00000140471	-0,2307361	1,79728204	1,48977041	0,25245125	0,55685646	LINS1	lines homolog 1 [Source:HGNC Symbol;Acc:HGNC:30922]
ENSG00000188313	0,18019394	4,80613611	1,49016408	0,252393	0,55685646	PLSCR1	phospholipid scramblase 1 [Source:HGNC Symbol;Acc:HGNC:9092]
ENSG00000237550	-0,180608	6,98782011	1,4899067	0,25243108	0,55685646	AC243919.1	ribosomal protein L9 pseudogene 9
ENSG00000050130	0,21913474	5,57192509	1,48846137	0,25264509	0,55690982	JKAMP	JNK1/MAPK8 associated membrane protein [Source:HGNC Symbol;Acc:HGNC:20184]
ENSG00000103005	0,20254032	4,79058767	1,48898221	0,25256794	0,55690982	USB1	U6 snRNA biogenesis phosphodiesterase 1 [Source:HGNC Symbol;Acc:HGNC:25792]
ENSG00000114209	0,21685643	5,25924606	1,48909482	0,25255127	0,55690982	PDCD10	programmed cell death 10 [Source:HGNC Symbol;Acc:HGNC:8761]
ENSG00000184216	-0,1759724	7,49520582	1,48831994	0,25266605	0,55690982	IRAK1	interleukin 1 receptor associated kinase 1 [Source:HGNC Symbol;Acc:HGNC:6112]
ENSG00000269176	-0,4861976	-0,5572263	1,48854211	0,25263313	0,55690982	AP001160.3	novel transcript, antisense to TAF6L
ENSG00000007168	0,16564897	5,92925909	1,48650676	0,25293491	0,55699312	PAFAH1B1	platelet activating factor acetylhydrolase 1b regulatory subunit 1 [Source:HGNC Symbol;Acc:HGNC:8574]
ENSG00000088356	0,16312122	6,41910608	1,48577636	0,25304333	0,55699312	PDRG1	p53 and DNA damage regulated 1 [Source:HGNC Symbol;Acc:HGNC:16119]
ENSG00000089157	-0,1794434	11,5792066	1,48616523	0,2529856	0,55699312	RPLP0	ribosomal protein lateral stalk subunit P0 [Source:HGNC Symbol;Acc:HGNC:10371]
ENSG00000104897	0,17878604	5,92990431	1,48478755	0,25319021	0,55699312	SF3A2	splicing factor 3a subunit 2 [Source:HGNC Symbol;Acc:HGNC:10766]
ENSG00000107862	-0,2672107	5,21923932	1,48648287	0,25293846	0,55699312	GBF1	golgi brefeldin A resistant guanine nucleotide exchange factor 1 [Source:HGNC Symbol;Acc:HGNC:4181]
ENSG00000124181	-0,2389516	6,69689271	1,48599499	0,25301087	0,55699312	PLCG1	phospholipase C gamma 1 [Source:HGNC Symbol;Acc:HGNC:9065]
ENSG00000156875	0,3449137	1,0272524	1,4858769	0,25302841	0,55699312	MFSD14A	major facilitator superfamily domain containing 14A [Source:HGNC Symbol;Acc:HGNC:23363]
ENSG00000157870	0,18530562	4,12837673	1,48556783	0,2530743	0,55699312	PRXL2B	peroxiredoxin like 2B [Source:HGNC Symbol;Acc:HGNC:28390]
ENSG00000185730	-0,1888242	3,3125204	1,48725148	0,25282444	0,55699312	ZNF696	zinc finger protein 696 [Source:HGNC Symbol;Acc:HGNC:25872]
ENSG00000186501	0,16600933	5,80858103	1,48504251	0,25315233	0,55699312	TMEM222	transmembrane protein 222 [Source:HGNC Symbol;Acc:HGNC:25363]
ENSG00000238082	-0,5156099	-1,1486946	1,48477293	0,25319239	0,55699312	NUDCP2	nuclear distribution C pseudogene 2 [Source:HGNC Symbol;Acc:HGNC:8047]
ENSG00000246089	-0,2585754	2,49883952	1,48753668	0,25278214	0,55699312	AC016065.1	novel transcript
ENSG00000261612	0,50075178	-0,6583676	1,48472514	0,25319949	0,55699312	SUB1P3	SUB1 pseudogene 3 [Source:HGNC Symbol;Acc:HGNC:43993]
ENSG00000237668	-0,2432861	1,64466303	1,48443663	0,25324237	0,55700358	RPS15AP38	ribosomal protein S15a pseudogene 38 [Source:HGNC Symbol;Acc:HGNC:36523]
ENSG00000187860	0,49029195	0,12696345	1,48286694	0,25347584	0,55733204	CCDC157	coiled-coil domain containing 157 [Source:HGNC Symbol;Acc:HGNC:33854]

ENSG00000218510	0,39466986	0,104934	1,48310129	0,25344097	0,55733204	LINC00339	long intergenic non-protein coding RNA 339 [Source:HGNC Symbol;Acc:HGNC:25011]
ENSG00000287091	0,29893326	1,14121532	1,48266329	0,25350615	0,55733204	AC009961.3	novel transcript, sense intronic to CD302and LY75-CD302
ENSG00000163472	-0,2712235	1,52281362	1,48238472	0,25354763	0,55733934	TMEM79	transmembrane protein 79 [Source:HGNC Symbol;Acc:HGNC:28196]
ENSG00000075131	-0,2037023	4,13724029	1,47932276	0,2540041	0,55745877	TIPIN	TIMELESS interacting protein [Source:HGNC Symbol;Acc:HGNC:30750]
ENSG00000103356	0,2361413	6,45375737	1,47961155	0,253961	0,55745877	EARS2	glutamyl-tRNA synthetase 2, mitochondrial [Source:HGNC Symbol;Acc:HGNC:29419]
ENSG00000112787	-0,1740373	4,90859998	1,47935785	0,25399886	0,55745877	FBRSL1	fibrosin like 1 [Source:HGNC Symbol;Acc:HGNC:29308]
ENSG00000146109	-0,194971	6,35095142	1,48028801	0,25386008	0,55745877	ABT1	activator of basal transcription 1 [Source:HGNC Symbol;Acc:HGNC:17369]
ENSG00000149489	-0,3964369	0,57816027	1,47928809	0,25400928	0,55745877	ROM1	retinal outer segment membrane protein 1 [Source:HGNC Symbol;Acc:HGNC:10254]
ENSG00000158856	-0,2099674	4,49128987	1,47883984	0,2540762	0,55745877	DMTN	dematin actin binding protein [Source:HGNC Symbol;Acc:HGNC:3382]
ENSG00000172456	-0,1940452	2,89596678	1,47896453	0,25405758	0,55745877	FGGY	FGGY carbohydrate kinase domain containing [Source:HGNC Symbol;Acc:HGNC:25610]
ENSG00000174606	0,1840376	3,24923113	1,48031734	0,25385571	0,55745877	ANGEL2	angel homolog 2 [Source:HGNC Symbol;Acc:HGNC:30534]
ENSG00000175505	-0,3431063	0,81881027	1,4786937	0,25409802	0,55745877	CLCF1	cardiotrophin like cytokine factor 1 [Source:HGNC Symbol;Acc:HGNC:17412]
ENSG00000176387	-0,1856511	3,77548193	1,48068089	0,25380149	0,55745877	HSD11B2	hydroxysteroid 11-beta dehydrogenase 2 [Source:HGNC Symbol;Acc:HGNC:5209]
ENSG00000214706	-0,1975924	6,70071651	1,47997115	0,25390734	0,55745877	IFRD2	interferon related developmental regulator 2 [Source:HGNC Symbol;Acc:HGNC:5457]
ENSG00000215421	0,37330233	2,27601434	1,47933376	0,25400246	0,55745877	ZNF407	zinc finger protein 407 [Source:HGNC Symbol;Acc:HGNC:19904]
ENSG00000256229	0,31157534	0,65070412	1,48023296	0,25386829	0,55745877	ZNF486	zinc finger protein 486 [Source:HGNC Symbol;Acc:HGNC:20807]
ENSG00000186660	-0,2119712	3,1139454	1,478303	0,25415638	0,55750308	ZFP91	ZFP91 zinc finger protein, atypical E3 ubiquitin ligase [Source:HGNC Symbol;Acc:HGNC:14983]
ENSG00000008311	-0,2015703	3,80061526	1,47558649	0,25456265	0,55761325	AASS	aminoadipate-semialdehyde synthase [Source:HGNC Symbol;Acc:HGNC:17366]
ENSG00000091592	0,62516727	-1,5631833	1,47678019	0,25438401	0,55761325	NLRP1	NLR family pyrin domain containing 1 [Source:HGNC Symbol;Acc:HGNC:14374]
ENSG00000137868	1,0785965	-1,918085	1,47624203	0,25446452	0,55761325	STRA6	signaling receptor and transporter of retinol STRA6 [Source:HGNC Symbol;Acc:HGNC:30650]
ENSG00000155592	0,20226529	4,15871552	1,47710154	0,25433595	0,55761325	ZKSCAN2	zinc finger with KRAB and SCAN domains 2 [Source:HGNC Symbol;Acc:HGNC:25677]
ENSG00000160932	-0,2030476	5,82955867	1,47554166	0,25456936	0,55761325	LY6E	lymphocyte antigen 6 family member E [Source:HGNC Symbol;Acc:HGNC:6727]
ENSG00000170089	0,18307673	4,26546705	1,47637315	0,2544449	0,55761325	AC106795.1	THO complex 3 (THOC3) pseudogene
ENSG00000177173	-0,6699282	-1,623262	1,47733084	0,25430167	0,55761325	NAP1L4P1	nucleosome assembly protein 1 like 4 pseudogene 1 [Source:HGNC Symbol;Acc:HGNC:39740]
ENSG00000248487	-0,2174398	4,37558648	1,4763603	0,25444683	0,55761325	ABHD14A	abhydrolase domain containing 14A [Source:HGNC Symbol;Acc:HGNC:24538]
ENSG00000256453	-0,6820628	-1,6910167	1,47541521	0,25458829	0,55761325	DND1	DND microRNA-mediated repression inhibitor 1 [Source:HGNC Symbol;Acc:HGNC:23799]
ENSG00000258301	0,32618833	1,16844044	1,47604848	0,25449349	0,55761325	VASH1-AS1	VASH1 antisense RNA 1 [Source:HGNC Symbol;Acc:HGNC:53215]
ENSG00000121073	-0,1657354	8,10180842	1,4748879	0,25466727	0,55761902	SLC35B1	solute carrier family 35 member B1 [Source:HGNC Symbol;Acc:HGNC:20798]
ENSG00000286190	-0,5136225	-0,2241733	1,47511206	0,25463369	0,55761902	AC055839.2	uncharacterized LOC728392 [Source:NCBI gene (formerly Entrezgene);Acc:728392]
ENSG00000179859	0,88026661	-1,6887881	1,47418881	0,25477203	0,5577648	RNF227	ring finger protein 227 [Source:HGNC Symbol;Acc:HGNC:27571]
ENSG00000136715	0,28111724	4,85538213	1,47366635	0,25485036	0,55776911	SAP130	Sin3A associated protein 130 [Source:HGNC Symbol;Acc:HGNC:29813]
ENSG00000164879	0,58887697	-1,537129	1,47380883	0,25482899	0,55776911	CA3	carbonic anhydrase 3 [Source:HGNC Symbol;Acc:HGNC:1374]
ENSG00000003509	0,20173656	5,13144602	1,47061184	0,25530897	0,55837455	NDUFAF7	NADH:ubiquinone oxidoreductase complex assembly factor 7 [Source:HGNC Symbol;Acc:HGNC:28816]
ENSG00000140545	0,2344461	3,55365244	1,47029695	0,25535631	0,55837455	MFGE8	milk fat globule EGF and factor V/VIII domain containing [Source:HGNC Symbol;Acc:HGNC:7036]
ENSG00000167258	0,23269949	6,28459746	1,47103423	0,25524548	0,55837455	CDK12	cyclin dependent kinase 12 [Source:HGNC Symbol;Acc:HGNC:24224]
ENSG00000175115	0,25210275	4,55080913	1,47042662	0,25533682	0,55837455	PACS1	phosphofurin acidic cluster sorting protein 1 [Source:HGNC Symbol;Acc:HGNC:30032]
ENSG00000247077	0,24397489	6,15853278	1,47139624	0,25519109	0,55837455	PGAM5	PGAM family member 5, mitochondrial serine/threonine protein phosphatase [Source:HGNC Symbol;Acc:HGNC:2
ENSG00000261120	-0,6338814	-1,7368778	1,47070417	0,25529509	0,55837455	AL133299.1	novel transcript, antisense to LRRC9
ENSG00000172939	-0,2069114	4,75350245	1,46955506	0,25546791	0,55853496	OXSR1	oxidative stress responsive kinase 1 [Source:HGNC Symbol;Acc:HGNC:8508]
ENSG00000130669	0,16252162	5,5521415	1,46905321	0,25554343	0,55861648	PAK4	p21 (RAC1) activated kinase 4 [Source:HGNC Symbol;Acc:HGNC:16059]
ENSG00000148943	-0,1870388	3,71280524	1,46779414	0,25573305	0,55893009	LIN7C	lin-7 homolog C, crumbs cell polarity complex component [Source:HGNC Symbol;Acc:HGNC:17789]
ENSG00000186615	-0,322472	1,81219502	1,46759264	0,25576341	0,55893009	KTN1-AS1	KTN1 antisense RNA 1 [Source:HGNC Symbol;Acc:HGNC:19842]
ENSG00000106636	0,17438293	5,91233482	1,46567588	0,2560525	0,55899074	YKT6	YKT6 v-SNARE homolog [Source:HGNC Symbol;Acc:HGNC:16959]
ENSG00000145425	-0,1740687	10,6663364	1,46573085	0,2560442	0,55899074	RPS3A	ribosomal protein S3A [Source:HGNC Symbol;Acc:HGNC:10421]

ENSG00000147118	0,24948682	1,96939053	1,46549486	0,25607982	0,55899074	ZNF182	zinc finger protein 182 [Source:HGNC Symbol;Acc:HGNC:13001]
ENSG00000147202	0,23270418	2,57450728	1,46577674	0,25603727	0,55899074	DIAPH2	diaphanous related formin 2 [Source:HGNC Symbol;Acc:HGNC:2877]
ENSG00000165655	-0,191077	4,36897786	1,46711005	0,25583615	0,55899074	ZNF503	zinc finger protein 503 [Source:HGNC Symbol;Acc:HGNC:23589]
ENSG00000172602	-0,2069445	2,67896948	1,46649018	0,25592963	0,55899074	RND1	Rho family GTPase 1 [Source:HGNC Symbol;Acc:HGNC:18314]
ENSG00000196187	0,18675442	4,17381088	1,46529396	0,25611015	0,55899074	TMEM63A	transmembrane protein 63A [Source:HGNC Symbol;Acc:HGNC:29118]
ENSG00000196230	-0,1656445	11,2797618	1,46539678	0,25609463	0,55899074	TUBB	tubulin beta class I [Source:HGNC Symbol;Acc:HGNC:20778]
ENSG00000197744	-0,423997	0,26919726	1,46512588	0,25613553	0,55899074	PTMAP2	prothymosin alpha pseudogene 2 [Source:HGNC Symbol;Acc:HGNC:9625]
ENSG00000086712	-0,2132047	3,83880888	1,46403887	0,25629975	0,55926558	TXLNG	taxilin gamma [Source:HGNC Symbol;Acc:HGNC:18578]
ENSG00000114529	-0,2983851	0,98539123	1,46322416	0,25642293	0,5593433	C3orf52	chromosome 3 open reading frame 52 [Source:HGNC Symbol;Acc:HGNC:26255]
ENSG00000123810	-0,2533866	2,00417168	1,46343052	0,25639172	0,5593433	B9D2	B9 domain containing 2 [Source:HGNC Symbol;Acc:HGNC:28636]
ENSG00000131650	0,29096158	1,59024347	1,46304366	0,25645023	0,5593433	KREMEN2	kringle containing transmembrane protein 2 [Source:HGNC Symbol;Acc:HGNC:18797]
ENSG00000048140	-0,2085725	4,23081184	1,46213425	0,25658785	0,5593929	TSPAN17	tetraspanin 17 [Source:HGNC Symbol;Acc:HGNC:13594]
ENSG00000122545	0,20602189	5,78118297	1,46242242	0,25654423	0,5593929	SEPTIN7	septin 7 [Source:HGNC Symbol;Acc:HGNC:1717]
ENSG00000169347	0,2452762	2,24027373	1,46227813	0,25656607	0,5593929	GP2	glycoprotein 2 [Source:HGNC Symbol;Acc:HGNC:4441]
ENSG00000100342	-0,2426611	5,12082012	1,46160499	0,25666798	0,55943317	APOL1	apolipoprotein L1 [Source:HGNC Symbol;Acc:HGNC:618]
ENSG00000148498	-0,1872328	6,29986426	1,46149885	0,25668406	0,55943317	PARD3	par-3 family cell polarity regulator [Source:HGNC Symbol;Acc:HGNC:16051]
ENSG00000161036	-0,3120132	4,48835604	1,46125365	0,2567212	0,55943317	LRWD1	leucine rich repeats and WD repeat domain containing 1 [Source:HGNC Symbol;Acc:HGNC:21769]
ENSG00000175063	-0,2173111	5,84167816	1,46073874	0,25679922	0,55951973	UBE2C	ubiquitin conjugating enzyme E2 C [Source:HGNC Symbol;Acc:HGNC:15937]
ENSG00000100218	-0,6481249	-1,7679033	1,45987863	0,25692961	0,55972036	RSPH14	radial spoke head 14 homolog [Source:HGNC Symbol;Acc:HGNC:13437]
ENSG00000113119	-0,1970806	2,61090795	1,45845322	0,25714592	0,56006402	TMCO6	transmembrane and coiled-coil domains 6 [Source:HGNC Symbol;Acc:HGNC:28814]
ENSG00000165006	0,22390605	4,81368001	1,45808145	0,25720237	0,56006402	UBAP1	ubiquitin associated protein 1 [Source:HGNC Symbol;Acc:HGNC:12461]
ENSG00000245275	-0,8044712	-1,7217327	1,45823998	0,2571783	0,56006402	SAP30L-AS1	SAP30L antisense RNA 1 (head to head) [Source:HGNC Symbol;Acc:HGNC:26760]
ENSG00000105443	-0,1703113	4,75582608	1,4571421	0,2573451	0,56016359	CYTH2	cytohesin 2 [Source:HGNC Symbol;Acc:HGNC:9502]
ENSG00000126858	0,2029546	4,08249849	1,45700033	0,25736665	0,56016359	RHOT1	ras homolog family member T1 [Source:HGNC Symbol;Acc:HGNC:21168]
ENSG00000136158	0,21564974	3,06548632	1,45651911	0,25743982	0,56016359	SPRY2	sprouty RTK signaling antagonist 2 [Source:HGNC Symbol;Acc:HGNC:11270]
ENSG00000137075	0,24790467	2,22768651	1,4566595	0,25741847	0,56016359	RNF38	ring finger protein 38 [Source:HGNC Symbol;Acc:HGNC:18052]
ENSG00000184752	-0,1626686	6,41648152	1,45717064	0,25734076	0,56016359	NDUFA12	NADH:ubiquinone oxidoreductase subunit A12 [Source:HGNC Symbol;Acc:HGNC:23987]
ENSG00000168538	0,19239221	3,87989462	1,45493397	0,25768104	0,56060497	TRAPPC11	trafficking protein particle complex 11 [Source:HGNC Symbol;Acc:HGNC:25751]
ENSG00000127838	-0,1736799	6,52663931	1,45391115	0,25783686	0,56082856	PNKD	PNKD metallo-beta-lactamase domain containing [Source:HGNC Symbol;Acc:HGNC:9153]
ENSG00000198964	0,26639751	2,97845386	1,4537554	0,2578606	0,56082856	SGMS1	sphingomyelin synthase 1 [Source:HGNC Symbol;Acc:HGNC:29799]
ENSG00000171208	-0,2573803	2,33796161	1,45327707	0,25793352	0,56085512	NETO2	neuropilin and tolloid like 2 [Source:HGNC Symbol;Acc:HGNC:14644]
ENSG00000176476	0,19901765	4,3878539	1,45291997	0,25798798	0,56085512	SGF29	SAGA complex associated factor 29 [Source:HGNC Symbol;Acc:HGNC:25156]
ENSG00000269968	-0,5092579	-0,3888147	1,45297421	0,25797971	0,56085512	AC006064.4	novel transcript, antisense to GAPDH
ENSG00000148926	-0,1867847	4,56870275	1,4525897	0,25803836	0,56088118	ADM	adrenomedullin [Source:HGNC Symbol;Acc:HGNC:259]
ENSG00000044446	0,18157874	4,57943768	1,45169846	0,25817439	0,56092648	PHKA2	phosphorylase kinase regulatory subunit alpha 2 [Source:HGNC Symbol;Acc:HGNC:8926]
ENSG00000120437	-0,1598363	9,31763308	1,45186391	0,25814913	0,56092648	ACAT2	acetyl-CoA acetyltransferase 2 [Source:HGNC Symbol;Acc:HGNC:94]
ENSG00000227885	-0,8361772	-1,7169269	1,45219163	0,25809911	0,56092648	AL590652.1	novel transcript
ENSG00000130725	-0,1765771	5,88220217	1,45110813	0,25826455	0,56095551	UBE2M	ubiquitin conjugating enzyme E2 M [Source:HGNC Symbol;Acc:HGNC:12491]
ENSG00000244731	-0,4841138	4,61680547	1,45118961	0,2582521	0,56095551	C4A	complement C4A (Rodgers blood group) [Source:HGNC Symbol;Acc:HGNC:1323]
ENSG00000105258	-0,2001253	5,32304214	1,45025624	0,25839472	0,56098804	POLR2I	RNA polymerase II subunit I [Source:HGNC Symbol;Acc:HGNC:9196]
ENSG00000143147	0,81298098	-1,3013955	1,45071845	0,25832408	0,56098804	GPR161	G protein-coupled receptor 161 [Source:HGNC Symbol;Acc:HGNC:23694]
ENSG00000213542	-0,4995062	-1,1609361	1,45026392	0,25839355	0,56098804	AC007000.1	ribosomal protein L7a (RPL7A) pseudogene
ENSG00000197714	0,39532143	1,90681881	1,44851894	0,25866048	0,56148157	ZNF460	zinc finger protein 460 [Source:HGNC Symbol;Acc:HGNC:21628]
ENSG00000168060	0,24673804	2,85350994	1,4477191	0,25878296	0,56166399	NAALADL1	N-acetylated alpha-linked acidic dipeptidase like 1 [Source:HGNC Symbol;Acc:HGNC:23536]

ENSG00000181690	0,75416351	-0,1875865	1,44446019	0,25928285	0,56266535	PLAG1	PLAG1 zinc finger [Source:HGNC Symbol;Acc:HGNC:9045]
ENSG00000011454	0,27533449	4,37404202	1,44381594	0,25938183	0,56271297	RABGAP1	RAB GTPase activating protein 1 [Source:HGNC Symbol;Acc:HGNC:17155]
ENSG00000211445	-0,1850237	7,27837285	1,44399597	0,25935417	0,56271297	GPX3	glutathione peroxidase 3 [Source:HGNC Symbol;Acc:HGNC:4555]
ENSG00000130827	-0,3761123	3,30570794	1,44284877	0,25953052	0,56295195	PLXNA3	plexin A3 [Source:HGNC Symbol;Acc:HGNC:9101]
ENSG00000240370	-0,3147993	1,0088782	1,44245149	0,25959163	0,56300092	RPL13P5	ribosomal protein L13 pseudogene 5 [Source:HGNC Symbol;Acc:HGNC:30363]
ENSG00000131724	-0,1682241	6,76787476	1,44067424	0,25986527	0,56326403	IL13RA1	interleukin 13 receptor subunit alpha 1 [Source:HGNC Symbol;Acc:HGNC:5974]
ENSG00000135374	-0,6077021	-0,7531417	1,44102515	0,25981121	0,56326403	ELF5	E74 like ETS transcription factor 5 [Source:HGNC Symbol;Acc:HGNC:3320]
ENSG00000155097	0,16507333	5,02397349	1,44077035	0,25985046	0,56326403	ATP6V1C1	ATPase H+ transporting V1 subunit C1 [Source:HGNC Symbol;Acc:HGNC:856]
ENSG00000180694	-0,2304959	2,43017533	1,44066184	0,25986718	0,56326403	TMEM64	transmembrane protein 64 [Source:HGNC Symbol;Acc:HGNC:25441]
ENSG00000115226	-0,2005473	2,92298583	1,43987892	0,25998785	0,56341839	FNDC4	fibronectin type III domain containing 4 [Source:HGNC Symbol;Acc:HGNC:20239]
ENSG00000278053	-0,2426448	4,84042406	1,43969941	0,26001552	0,56341839	DDX52	DExD-box helicase 52 [Source:HGNC Symbol;Acc:HGNC:20038]
ENSG00000122692	-0,1924598	6,03323022	1,43935887	0,26006804	0,56344862	SMU1	SMU1 DNA replication regulator and spliceosomal factor [Source:HGNC Symbol;Acc:HGNC:18247]
ENSG00000231500	-0,1810057	11,2755629	1,43878013	0,26015733	0,56355849	RPS18	ribosomal protein S18 [Source:HGNC Symbol;Acc:HGNC:10401]
ENSG00000086544	-0,1806908	4,97198015	1,43691022	0,26044612	0,56410042	ITPKC	inositol-trisphosphate 3-kinase C [Source:HGNC Symbol;Acc:HGNC:14897]
ENSG00000165868	0,22671913	2,6892956	1,43610592	0,26057047	0,5642861	HSPA12A	heat shock protein family A (Hsp70) member 12A [Source:HGNC Symbol;Acc:HGNC:19022]
ENSG00000127585	0,56137668	-1,148768	1,43544775	0,26067229	0,56442293	FBXL16	F-box and leucine rich repeat protein 16 [Source:HGNC Symbol;Acc:HGNC:14150]
ENSG00000236924	-0,3379453	0,43995264	1,4344739	0,26082305	0,56466567	AL162411.1	novel transcript
ENSG00000117407	0,80776873	-1,8194651	1,4335906	0,2609599	0,56471088	ARTN	artemin [Source:HGNC Symbol;Acc:HGNC:727]
ENSG00000250346	-0,606075	-1,7426641	1,43383683	0,26092174	0,56471088	EEF1GP2	eukaryotic translation elongation factor 1 gamma pseudogene 2 [Source:HGNC Symbol;Acc:HGNC:44557]
ENSG00000255561	0,36292506	0,63939295	1,43371634	0,26094041	0,56471088	FDXACB1	ferredoxin-fold anticodon binding domain containing 1 [Source:HGNC Symbol;Acc:HGNC:25110]
ENSG00000234985	-0,5865703	-0,4395026	1,43324768	0,26101305	0,56474225	AC074085.2	ribosomal protein L18 (RPL18) pseudogene
ENSG00000156172	-0,3942125	-0,0947026	1,43273308	0,26109285	0,56483124	C8orf37	chromosome 8 open reading frame 37 [Source:HGNC Symbol;Acc:HGNC:27232]
ENSG00000090661	0,16761376	6,82823223	1,43214446	0,26118416	0,56494512	CERS4	ceramide synthase 4 [Source:HGNC Symbol;Acc:HGNC:23747]
ENSG00000180263	0,36386093	1,31848493	1,43181444	0,26123537	0,56497225	FGD6	FYVE, RhoGEF and PH domain containing 6 [Source:HGNC Symbol;Acc:HGNC:21740]
ENSG00000180389	-0,3209173	1,50333411	1,43099241	0,26136301	0,56516462	ATP5F1EP2	ATP synthase F1 subunit epsilon pseudogene 2 [Source:HGNC Symbol;Acc:HGNC:34026]
ENSG00000171604	0,24856392	5,31432409	1,43062229	0,2614205	0,56520528	CXXC5	CXXC finger protein 5 [Source:HGNC Symbol;Acc:HGNC:26943]
ENSG00000164920	-0,3957509	0,25666472	1,43021463	0,26148385	0,56525859	OSR2	odd-skipped related transciption factor 2 [Source:HGNC Symbol;Acc:HGNC:15830]
ENSG00000272114	-0,536944	-0,9728487	1,42995517	0,26152418	0,56526212	AL136131.3	novel transcript, antisense to VEGFA
ENSG00000020633	-0,4304721	-0,6740468	1,42823783	0,26179133	0,5655679	RUNX3	RUNX family transcription factor 3 [Source:HGNC Symbol;Acc:HGNC:10473]
ENSG00000101162	-0,6741902	-0,3065393	1,42868274	0,26172208	0,5655679	TUBB1	tubulin beta 1 class VI [Source:HGNC Symbol;Acc:HGNC:16257]
ENSG00000143850	-0,250794	4,01242571	1,42771727	0,26187238	0,5655679	PLEKHA6	pleckstrin homology domain containing A6 [Source:HGNC Symbol;Acc:HGNC:17053]
ENSG00000157538	-0,1845967	4,85186091	1,42851483	0,26174821	0,5655679	VPS26C	VPS26 endosomal protein sorting factor C [Source:HGNC Symbol;Acc:HGNC:3044]
ENSG00000221986	-0,3067028	0,87116757	1,42755324	0,26189793	0,5655679	MYBPHL	myosin binding protein H like [Source:HGNC Symbol;Acc:HGNC:30434]
ENSG00000288061	0,64507209	-1,9769974	1,42761821	0,26188781	0,5655679	AP000926.2	novel transcript, antisense to HSPA8and CLMP
ENSG00000145414	-0,2047611	3,18885184	1,4267875	0,26201724	0,56574192	NAF1	nuclear assembly factor 1 ribonucleoprotein [Source:HGNC Symbol;Acc:HGNC:25126]
ENSG00000009950	-0,2232446	5,10285556	1,42566919	0,26219162	0,56581102	MLXIPL	MLX interacting protein like [Source:HGNC Symbol;Acc:HGNC:12744]
ENSG00000096968	0,25505613	1,46418741	1,42627967	0,26209641	0,56581102	JAK2	Janus kinase 2 [Source:HGNC Symbol;Acc:HGNC:6192]
ENSG00000118816	-0,2504162	5,30404521	1,42591083	0,26215393	0,56581102	CCNI	cyclin I [Source:HGNC Symbol;Acc:HGNC:1595]
ENSG00000169189	-0,2528269	6,10081433	1,42558881	0,26220416	0,56581102	NSMCE1	NSE1 homolog, SMC5-SMC6 complex component [Source:HGNC Symbol;Acc:HGNC:29897]
ENSG00000256433	-0,4768538	-0,4140308	1,42461243	0,26235655	0,56605625	AC005840.2	novel transcript
ENSG00000125743	-0,1876616	8,30779997	1,4227438	0,26264855	0,56629715	SNRPD2	small nuclear ribonucleoprotein D2 polypeptide [Source:HGNC Symbol;Acc:HGNC:11159]
ENSG00000133318	0,2421955	6,91284925	1,42265765	0,26266202	0,56629715	RTN3	reticulon 3 [Source:HGNC Symbol;Acc:HGNC:10469]
ENSG00000170852	0,18925693	4,27045715	1,42321212	0,26257533	0,56629715	KBTBD2	kelch repeat and BTB domain containing 2 [Source:HGNC Symbol;Acc:HGNC:21751]
ENSG00000179978	0,8018482	-1,940182	1,42296525	0,26261392	0,56629715	NAIPP2	NAIP pseudogene 2 [Source:HGNC Symbol;Acc:HGNC:16042]

ENSG00000253716	0,35935662	1,49982316	1,42305334	0,26260015	0,56629715	MINCR	MYC-induced long non-coding RNA [Source:HGNC Symbol;Acc:HGNC:51653]
ENSG00000087008	-0,2040329	3,99068617	1,41966689	0,26313035	0,56641245	ACOX3	acyl-CoA oxidase 3, pristanoyl [Source:HGNC Symbol;Acc:HGNC:121]
ENSG00000099942	0,16437473	6,28529865	1,42103353	0,2629162	0,56641245	CRKL	CRK like proto-oncogene, adaptor protein [Source:HGNC Symbol;Acc:HGNC:2363]
ENSG00000104825	-0,1771722	4,51724788	1,41969279	0,26312629	0,56641245	NFKBIB	NFKB inhibitor beta [Source:HGNC Symbol;Acc:HGNC:7798]
ENSG00000125482	-0,2066245	3,84163746	1,41995082	0,26308584	0,56641245	TTF1	transcription termination factor 1 [Source:HGNC Symbol;Acc:HGNC:12397]
ENSG00000130592	-0,5930412	-0,6991401	1,42081713	0,26295009	0,56641245	LSP1	lymphocyte specific protein 1 [Source:HGNC Symbol;Acc:HGNC:6707]
ENSG00000130699	0,21679948	3,97362073	1,41975939	0,26311585	0,56641245	TAF4	TATA-box binding protein associated factor 4 [Source:HGNC Symbol;Acc:HGNC:11537]
ENSG00000165501	-0,2016729	4,04437062	1,42181125	0,26279444	0,56641245	LRR1	leucine rich repeat protein 1 [Source:HGNC Symbol;Acc:HGNC:19742]
ENSG00000166794	-0,1709186	11,5156343	1,41970466	0,26312443	0,56641245	PPIB	peptidylprolyl isomerase B [Source:HGNC Symbol;Acc:HGNC:9255]
ENSG00000182831	0,20389939	4,80037697	1,41959267	0,26314199	0,56641245	C16orf72	chromosome 16 open reading frame 72 [Source:HGNC Symbol;Acc:HGNC:30103]
ENSG00000188157	-0,3863845	6,13643951	1,421066	0,26291112	0,56641245	AGRN	agrin [Source:HGNC Symbol;Acc:HGNC:329]
ENSG00000236312	-0,3587705	0,42562865	1,42009674	0,26306297	0,56641245	RPL34P34	ribosomal protein L34 pseudogene 34 [Source:HGNC Symbol;Acc:HGNC:35848]
ENSG00000005156	0,21261668	5,6326886	1,41852498	0,26330947	0,56653159	LIG3	DNA ligase 3 [Source:HGNC Symbol;Acc:HGNC:6600]
ENSG00000160781	-0,6719603	-1,3415915	1,41825111	0,26335245	0,56653159	PAQR6	progestin and adipoQ receptor family member 6 [Source:HGNC Symbol;Acc:HGNC:30132]
ENSG00000161265	-0,4550384	-0,145247	1,41846453	0,26331896	0,56653159	U2AF1L4	U2 small nuclear RNA auxiliary factor 1 like 4 [Source:HGNC Symbol;Acc:HGNC:23020]
ENSG00000277117	-0,3533122	0,65238152	1,4189021	0,2632503	0,56653159	FP565260.3	ICOS ligand [Source:NCBI gene (formerly Entrezgene);Acc:102723996]
ENSG00000066117	0,19420694	6,52553409	1,41712157	0,26352984	0,56674627	SMARCD1	SWI/SNF related, matrix associated, actin dependent regulator of chromatin, subfamily d, member 1 [Source:HGN
ENSG00000116649	-0,1973317	8,28255891	1,41731636	0,26349924	0,56674627	SRM	spermidine synthase [Source:HGNC Symbol;Acc:HGNC:11296]
ENSG00000158467	0,29412838	3,00579563	1,41627829	0,26366238	0,56694786	AHCYL2	adenosylhomocysteinase like 2 [Source:HGNC Symbol;Acc:HGNC:22204]
ENSG00000040608	-0,5471169	-0,8699339	1,41155053	0,26440719	0,56704707	RTN4R	reticulon 4 receptor [Source:HGNC Symbol;Acc:HGNC:18601]
ENSG00000068137	-0,1737428	3,85102239	1,41368794	0,2640701	0,56704707	PLEKHH3	pleckstrin homology, MyTH4 and FERM domain containing H3 [Source:HGNC Symbol;Acc:HGNC:26105]
ENSG00000083838	0,24144506	2,59972874	1,41184305	0,26436102	0,56704707	ZNF446	zinc finger protein 446 [Source:HGNC Symbol;Acc:HGNC:21036]
ENSG00000114999	-0,1838577	5,67529381	1,4121842	0,26430719	0,56704707	TTL	tubulin tyrosine ligase [Source:HGNC Symbol;Acc:HGNC:21586]
ENSG00000134762	0,70802267	-1,2789007	1,41419364	0,26399043	0,56704707	DSC3	desmocollin 3 [Source:HGNC Symbol;Acc:HGNC:3037]
ENSG00000149577	-0,1882873	4,19603337	1,41358709	0,26408599	0,56704707	SIDT2	SID1 transmembrane family member 2 [Source:HGNC Symbol;Acc:HGNC:24272]
ENSG00000160957	-0,1878242	4,96531147	1,41189181	0,26435333	0,56704707	RECQL4	RecQ like helicase 4 [Source:HGNC Symbol;Acc:HGNC:9949]
ENSG00000162383	-0,5390795	-1,2160058	1,41232933	0,2642843	0,56704707	SLC1A7	solute carrier family 1 member 7 [Source:HGNC Symbol;Acc:HGNC:10945]
ENSG00000162714	0,16902567	4,81366187	1,41333555	0,26412563	0,56704707	ZNF496	zinc finger protein 496 [Source:HGNC Symbol;Acc:HGNC:23713]
ENSG00000172819	0,47820955	0,03421032	1,41177119	0,26437236	0,56704707	RARG	retinoic acid receptor gamma [Source:HGNC Symbol;Acc:HGNC:9866]
ENSG00000184979	0,23285033	3,9859885	1,41388062	0,26403974	0,56704707	USP18	ubiquitin specific peptidase 18 [Source:HGNC Symbol;Acc:HGNC:12616]
ENSG00000213965	0,2136116	2,0267527	1,41274946	0,26421803	0,56704707	NUDT19	nudix hydrolase 19 [Source:HGNC Symbol;Acc:HGNC:32036]
ENSG00000215835	0,42009052	-0,0222836	1,41260437	0,26424091	0,56704707	AL596087.1	Ewing sarcoma breakpoint region 1 (EWSR1) pseudogene
ENSG00000241494	-0,2653425	1,31958701	1,41571969	0,26375023	0,56704707	AL355032.1	ribosomal protein L26-like 1 (RPL26L1) pseudogene
ENSG00000243317	-0,2162795	5,16412844	1,41231533	0,2642865	0,56704707	STMP1	short transmembrane mitochondrial protein 1 [Source:HGNC Symbol;Acc:HGNC:41909]
ENSG00000244184	0,57145863	-1,6504379	1,41228674	0,26429101	0,56704707	AC091153.3	novel transcript
ENSG00000265590	-0,4251732	-0,1584293	1,41464415	0,26391949	0,56704707	CFAP298-TCP10L	CFAP298-TCP10L readthrough [Source:HGNC Symbol;Acc:HGNC:54636]
ENSG00000277161	0,18910736	4,10324889	1,41236286	0,26427901	0,56704707	PIGW	phosphatidylinositol glycan anchor biosynthesis class W [Source:HGNC Symbol;Acc:HGNC:23213]
ENSG00000163605	0,16479633	5,55952039	1,41085017	0,26451777	0,56720096	PPP4R2	protein phosphatase 4 regulatory subunit 2 [Source:HGNC Symbol;Acc:HGNC:18296]
ENSG00000140575	0,22830828	3,64826084	1,409955	0,26465921	0,56742096	IQGAP1	IQ motif containing GTPase activating protein 1 [Source:HGNC Symbol;Acc:HGNC:6110]
ENSG00000246898	0,44298788	-0,3959619	1,40966951	0,26470434	0,56743444	LINC00920	long intergenic non-protein coding RNA 920 [Source:HGNC Symbol;Acc:HGNC:48611]
ENSG00000025770	-0,1792298	5,79118657	1,40739076	0,26506495	0,56751911	NCAPH2	non-SMC condensin II complex subunit H2 [Source:HGNC Symbol;Acc:HGNC:25071]
ENSG00000037897	-0,1699317	4,83187797	1,40761063	0,26503013	0,56751911	METTL1	methyltransferase like 1 [Source:HGNC Symbol;Acc:HGNC:7030]
ENSG00000128242	-0,3410118	1,39566055	1,40789265	0,26498547	0,56751911	GAL3ST1	galactose-3-O-sulfotransferase 1 [Source:HGNC Symbol;Acc:HGNC:24240]
ENSG00000143127	-0,4069522	1,8189374	1,40877612	0,26484564	0,56751911	ITGA10	integrin subunit alpha 10 [Source:HGNC Symbol;Acc:HGNC:6135]

ENSG00000159263	0,37186739	1,18443123	1,40872303	0,26485404	0,56751911	SIM2	SIM bHLH transcription factor 2 [Source:HGNC Symbol;Acc:HGNC:10883]
ENSG00000161904	0,24424609	6,20125712	1,40808456	0,26495509	0,56751911	LEMD2	LEM domain nuclear envelope protein 2 [Source:HGNC Symbol;Acc:HGNC:21244]
ENSG00000179115	-0,2521977	7,10642813	1,40864383	0,26486657	0,56751911	FARSA	phenylalanyl-tRNA synthetase subunit alpha [Source:HGNC Symbol;Acc:HGNC:3592]
ENSG00000181523	-0,2274877	5,9052616	1,4068664	0,26514803	0,56751911	SGSH	N-sulfoglucosamine sulfohydrolase [Source:HGNC Symbol;Acc:HGNC:10818]
ENSG00000182670	0,17095928	5,74683296	1,40653708	0,26520022	0,56751911	TTC3	tetratricopeptide repeat domain 3 [Source:HGNC Symbol;Acc:HGNC:12393]
ENSG00000214784	-0,2571039	1,38026222	1,40647536	0,26521001	0,56751911	RPS3AP21	RPS3A pseudogene 21 [Source:HGNC Symbol;Acc:HGNC:36841]
ENSG00000221988	0,16379393	5,35948136	1,40754767	0,2650401	0,56751911	PPT2	palmitoyl-protein thioesterase 2 [Source:HGNC Symbol;Acc:HGNC:9326]
ENSG00000247809	0,67907805	-1,6531823	1,40691072	0,26514101	0,56751911	NR2F2-AS1	NR2F2 antisense RNA 1 [Source:HGNC Symbol;Acc:HGNC:44222]
ENSG00000144724	0,18277101	5,12069712	1,4060918	0,26527082	0,56756611	PTPRG	protein tyrosine phosphatase receptor type G [Source:HGNC Symbol;Acc:HGNC:9671]
ENSG00000088970	-0,1849194	5,43846752	1,40384647	0,2656272	0,56766555	KIZ	kizuna centrosomal protein [Source:HGNC Symbol;Acc:HGNC:15865]
ENSG00000102934	0,18395955	7,83712312	1,40384045	0,26562816	0,56766555	PLLP	plasmolipin [Source:HGNC Symbol;Acc:HGNC:18553]
ENSG00000118762	0,31929125	1,51691496	1,40437816	0,26554275	0,56766555	PKD2	polycystin 2, transient receptor potential cation channel [Source:HGNC Symbol;Acc:HGNC:9009]
ENSG00000139998	0,17525391	3,59412183	1,40552331	0,26536099	0,56766555	RAB15	RAB15, member RAS oncogene family [Source:HGNC Symbol;Acc:HGNC:20150]
ENSG00000161714	-0,2338242	4,9520817	1,40431354	0,26555301	0,56766555	PLCD3	phospholipase C delta 3 [Source:HGNC Symbol;Acc:HGNC:9061]
ENSG00000197170	-0,1844118	6,48943025	1,40478393	0,26547832	0,56766555	PSMD12	proteasome 26S subunit, non-ATPase 12 [Source:HGNC Symbol;Acc:HGNC:9557]
ENSG00000205220	0,26067474	1,73078629	1,40416891	0,26557598	0,56766555	PSMB10	proteasome 20S subunit beta 10 [Source:HGNC Symbol;Acc:HGNC:9538]
ENSG00000231167	-0,2450996	1,4135862	1,40460393	0,2655069	0,56766555	YBX1P2	Y-box binding protein 1 pseudogene 2 [Source:HGNC Symbol;Acc:HGNC:22635]
ENSG00000141540	-0,4381015	-0,4269333	1,40353358	0,26567691	0,56768671	TTYH2	tweety family member 2 [Source:HGNC Symbol;Acc:HGNC:13877]
ENSG00000107959	0,22469419	5,61806306	1,40326643	0,26571937	0,5676944	PITRM1	pitrilysin metallopeptidase 1 [Source:HGNC Symbol;Acc:HGNC:17663]
ENSG00000162607	-0,2385209	4,93757842	1,40253935	0,26583497	0,56785833	USP1	ubiquitin specific peptidase 1 [Source:HGNC Symbol;Acc:HGNC:12607]
ENSG00000110619	-0,3057698	5,45434567	1,40123255	0,26604293	0,5680915	CARS1	cysteinyl-tRNA synthetase 1 [Source:HGNC Symbol;Acc:HGNC:1493]
ENSG00000234769	0,57925769	-1,2603045	1,40123713	0,2660422	0,5680915	WASH4P	WASP family homolog 4, pseudogene [Source:HGNC Symbol;Acc:HGNC:14126]
ENSG00000241945	-0,2746475	2,63525134	1,40112039	0,26606079	0,5680915	PWP2	PWP2 small subunit processome component [Source:HGNC Symbol;Acc:HGNC:9711]
ENSG00000226608	-0,1961585	4,99274229	1,40055232	0,26615126	0,56820164	FTLP3	ferritin light chain pseudogene 3 [Source:HGNC Symbol;Acc:HGNC:4000]
ENSG00000198938	0,15277086	12,2623155	1,39848466	0,26648095	0,56882234	MT-CO3	mitochondrially encoded cytochrome c oxidase III [Source:HGNC Symbol;Acc:HGNC:7422]
ENSG00000168710	-0,1891681	6,08601637	1,39821756	0,26652358	0,56883023	AHCYL1	adenosylhomocysteinase like 1 [Source:HGNC Symbol;Acc:HGNC:344]
ENSG00000166704	0,21407856	2,71240395	1,39723693	0,26668017	0,56899872	ZNF606	zinc finger protein 606 [Source:HGNC Symbol;Acc:HGNC:25879]
ENSG00000225484	0,24009688	1,73976637	1,39723539	0,26668042	0,56899872	NUTM2B-AS1	NUTM2B antisense RNA 1 [Source:HGNC Symbol;Acc:HGNC:51204]
ENSG00000140459	0,4463302	-0,3965367	1,39637741	0,26681753	0,56912503	CYP11A1	cytochrome P450 family 11 subfamily A member 1 [Source:HGNC Symbol;Acc:HGNC:2590]
ENSG00000188647	-0,2056055	2,94523777	1,39652187	0,26679444	0,56912503	PTAR1	protein prenyltransferase alpha subunit repeat containing 1 [Source:HGNC Symbol;Acc:HGNC:30449]
ENSG00000160999	-0,6227594	-1,3039863	1,39603517	0,26687225	0,56915865	SH2B2	SH2B adaptor protein 2 [Source:HGNC Symbol;Acc:HGNC:17381]
ENSG00000117054	0,19002366	5,40588426	1,39562692	0,26693755	0,5692148	ACADM	acyl-CoA dehydrogenase medium chain [Source:HGNC Symbol;Acc:HGNC:89]
ENSG00000101654	-0,1999898	4,47054526	1,3945497	0,26710995	0,56923488	RNMT	RNA guanine-7 methyltransferase [Source:HGNC Symbol;Acc:HGNC:10075]
ENSG00000155545	0,21401246	2,89721024	1,39466715	0,26709114	0,56923488	MIER3	MIER family member 3 [Source:HGNC Symbol;Acc:HGNC:26678]
ENSG00000163517	-0,1967766	3,82017614	1,39435086	0,26714179	0,56923488	HDAC11	histone deacetylase 11 [Source:HGNC Symbol;Acc:HGNC:19086]
ENSG00000224281	-0,7330839	-1,4461324	1,39506999	0,26702666	0,56923488	SLC25A5-AS1	SLC25A5 antisense RNA 1 [Source:HGNC Symbol;Acc:HGNC:43438]
ENSG00000233476	-0,1955724	4,30210705	1,39457077	0,26710657	0,56923488	EEF1A1P6	eukaryotic translation elongation factor 1 alpha 1 pseudogene 6 [Source:HGNC Symbol;Acc:HGNC:3201]
ENSG00000205765	0,20856904	3,21953295	1,39356209	0,26726814	0,56942107	C5orf51	chromosome 5 open reading frame 51 [Source:HGNC Symbol;Acc:HGNC:27750]
ENSG00000247092	-0,2377158	2,66277053	1,39262129	0,26741896	0,56965932	SNHG10	small nucleolar RNA host gene 10 [Source:HGNC Symbol;Acc:HGNC:27510]
ENSG00000115268	-0,1763553	9,88710615	1,39206111	0,26750882	0,56976766	RPS15	ribosomal protein S15 [Source:HGNC Symbol;Acc:HGNC:10388]
ENSG00000108515	-0,1700553	4,59745795	1,39077328	0,26771556	0,56977726	ENO3	enolase 3 [Source:HGNC Symbol;Acc:HGNC:3354]
ENSG00000110711	-0,1608535	5,92770621	1,39081504	0,26770886	0,56977726	AIP	aryl hydrocarbon receptor interacting protein [Source:HGNC Symbol;Acc:HGNC:358]
ENSG00000116698	0,30085051	4,2508742	1,39022258	0,26780404	0,56977726	SMG7	SMG7 nonsense mediated mRNA decay factor [Source:HGNC Symbol;Acc:HGNC:16792]
ENSG00000122515	0,1940063	2,92800176	1,39076646	0,26771666	0,56977726	ZMIZ2	zinc finger MIZ-type containing 2 [Source:HGNC Symbol;Acc:HGNC:22229]

ENSG00000130024	-0,1548027	6,1676847	1,39022463	0,26780371	0,56977726	PHF10	PHD finger protein 10 [Source:HGNC Symbol;Acc:HGNC:18250]
ENSG00000156110	-0,166068	6,19364605	1,38984735	0,26786435	0,56977726	ADK	adenosine kinase [Source:HGNC Symbol;Acc:HGNC:257]
ENSG00000185686	0,83935041	-1,6953791	1,39045088	0,26776735	0,56977726	PRAME	PRAME nuclear receptor transcriptional regulator [Source:HGNC Symbol;Acc:HGNC:9336]
ENSG00000198754	-0,7433773	-1,6853985	1,38985579	0,26786299	0,56977726	OXCT2	3-oxoacid CoA-transferase 2 [Source:HGNC Symbol;Acc:HGNC:18606]
ENSG00000272391	0,35849244	4,43900274	1,39024806	0,26779994	0,56977726	POM121C	POM121 transmembrane nucleoporin C [Source:HGNC Symbol;Acc:HGNC:34005]
ENSG00000101391	0,20006123	6,67970467	1,38777046	0,26819849	0,56990717	CDK5RAP1	CDK5 regulatory subunit associated protein 1 [Source:HGNC Symbol;Acc:HGNC:15880]
ENSG00000117676	-0,1675957	5,62851876	1,38778322	0,26819644	0,56990717	RPS6KA1	ribosomal protein S6 kinase A1 [Source:HGNC Symbol;Acc:HGNC:10430]
ENSG00000123411	0,36660749	0,07351162	1,38827477	0,2681173	0,56990717	IKZF4	IKAROS family zinc finger 4 [Source:HGNC Symbol;Acc:HGNC:13179]
ENSG00000146013	-0,5520745	-1,1873167	1,38844407	0,26809005	0,56990717	GFRA3	GDNF family receptor alpha 3 [Source:HGNC Symbol;Acc:HGNC:4245]
ENSG00000162542	-0,2139223	2,79433805	1,38809945	0,26814552	0,56990717	TMCO4	transmembrane and coiled-coil domains 4 [Source:HGNC Symbol;Acc:HGNC:27393]
ENSG00000237190	-0,2051927	4,87948972	1,38852133	0,26807762	0,56990717	CDKN2AIPNL	CDKN2A interacting protein N-terminal like [Source:HGNC Symbol;Acc:HGNC:30545]
ENSG00000258839	-0,2658007	1,8502513	1,3882386	0,26812312	0,56990717	MC1R	melanocortin 1 receptor [Source:HGNC Symbol;Acc:HGNC:6929]
ENSG00000225760	-0,7614645	-1,2763234	1,38749149	0,26824342	0,56991974	LINC00431	long intergenic non-protein coding RNA 431 [Source:HGNC Symbol;Acc:HGNC:42766]
ENSG00000114850	-0,1717303	8,50649218	1,38700162	0,26832234	0,57000452	SSR3	signal sequence receptor subunit 3 [Source:HGNC Symbol;Acc:HGNC:11325]
ENSG00000133398	0,18646678	4,69872205	1,38635606	0,26842639	0,57014264	MED10	mediator complex subunit 10 [Source:HGNC Symbol;Acc:HGNC:28760]
ENSG00000114388	-0,1853099	4,68397304	1,38569712	0,26853265	0,57028544	NPRL2	NPR2 like, GATOR1 complex subunit [Source:HGNC Symbol;Acc:HGNC:24969]
ENSG00000133805	-0,8674343	-1,7603617	1,38526076	0,26860306	0,57035204	AMPD3	adenosine monophosphate deaminase 3 [Source:HGNC Symbol;Acc:HGNC:470]
ENSG00000198133	-0,7772323	-1,4378513	1,38463696	0,26870374	0,57048292	TMEM229B	transmembrane protein 229B [Source:HGNC Symbol;Acc:HGNC:20130]
ENSG00000270906	0,56432451	-1,4915321	1,3839039	0,26882214	0,57065135	MTND4P35	MT-ND4 pseudogene 35 [Source:HGNC Symbol;Acc:HGNC:51949]
ENSG00000082146	0,25768937	5,16656575	1,38171455	0,26917616	0,57115389	STRADB	STE20 related adaptor beta [Source:HGNC Symbol;Acc:HGNC:13205]
ENSG00000118961	0,17574011	5,45325984	1,38173835	0,26917231	0,57115389	LDAH	lipid droplet associated hydrolase [Source:HGNC Symbol;Acc:HGNC:26145]
ENSG00000143409	-0,2014271	2,47882856	1,38177829	0,26916585	0,57115389	MINDY1	MINDY lysine 48 deubiquitinase 1 [Source:HGNC Symbol;Acc:HGNC:25648]
ENSG00000186468	-0,1856451	10,3612198	1,38122498	0,26925542	0,57123909	RPS23	ribosomal protein S23 [Source:HGNC Symbol;Acc:HGNC:10410]
ENSG00000142751	0,18502887	5,00690371	1,38030183	0,26940495	0,57147335	GPN2	GPN-loop GTPase 2 [Source:HGNC Symbol;Acc:HGNC:25513]
ENSG00000156096	-0,2414496	4,53040911	1,3787746	0,26965259	0,57183259	UGT2B4	UDP glucuronosyltransferase family 2 member B4 [Source:HGNC Symbol;Acc:HGNC:12553]
ENSG00000225648	0,230151	3,76962748	1,37896824	0,26962118	0,57183259	SBDSP1	SBDS pseudogene 1 [Source:HGNC Symbol;Acc:HGNC:21646]
ENSG00000021355	-0,1825676	5,85377679	1,37657643	0,27000959	0,57192624	SERPINB1	serpin family B member 1 [Source:HGNC Symbol;Acc:HGNC:3311]
ENSG00000030110	0,18179032	5,22689468	1,37741888	0,26987269	0,57192624	BAK1	BCL2 antagonist/killer 1 [Source:HGNC Symbol;Acc:HGNC:949]
ENSG00000105197	0,16260694	7,3595416	1,37638187	0,27004122	0,57192624	TIMM50	translocase of inner mitochondrial membrane 50 [Source:HGNC Symbol;Acc:HGNC:23656]
ENSG00000151065	0,19132618	3,62459178	1,3769723	0,26994525	0,57192624	DCP1B	decapping mRNA 1B [Source:HGNC Symbol;Acc:HGNC:24451]
ENSG00000163913	0,2105359	3,28553699	1,37668366	0,26999216	0,57192624	IFT122	intraflagellar transport 122 [Source:HGNC Symbol;Acc:HGNC:13556]
ENSG00000166598	-0,2781415	10,6994728	1,37794316	0,26978755	0,57192624	HSP90B1	heat shock protein 90 beta family member 1 [Source:HGNC Symbol;Acc:HGNC:12028]
ENSG00000172936	0,17819632	4,88635153	1,3781358	0,26975627	0,57192624	MYD88	MYD88 innate immune signal transduction adaptor [Source:HGNC Symbol;Acc:HGNC:7562]
ENSG00000223861	-0,5006184	-1,2259186	1,3772866	0,26989418	0,57192624	AL365436.1	ribosomal protein S10 (RPS10) pseudogene
ENSG00000261716	0,44425205	0,09317444	1,37633346	0,27004909	0,57192624	H2BC20P	H2B clustered histone 20, pseudogene [Source:HGNC Symbol;Acc:HGNC:20516]
ENSG00000064703	0,19369156	4,14577432	1,37463217	0,2703259	0,57193402	DDX20	DEAD-box helicase 20 [Source:HGNC Symbol;Acc:HGNC:2743]
ENSG00000106479	0,61037181	-1,4340706	1,37509338	0,27025082	0,57193402	ZNF862	zinc finger protein 862 [Source:HGNC Symbol;Acc:HGNC:34519]
ENSG00000163808	0,29908609	4,42006939	1,37501268	0,27026396	0,57193402	KIF15	kinesin family member 15 [Source:HGNC Symbol;Acc:HGNC:17273]
ENSG00000180425	-0,2476301	1,56412683	1,37462658	0,27032681	0,57193402	C11orf71	chromosome 11 open reading frame 71 [Source:HGNC Symbol;Acc:HGNC:25937]
ENSG00000197223	0,19814408	5,11322972	1,37480654	0,27029752	0,57193402	C1D	C1D nuclear receptor corepressor [Source:HGNC Symbol;Acc:HGNC:29911]
ENSG00000233597	-0,2150105	2,42081631	1,37536617	0,27020643	0,57193402	AC133435.1	ribosomal protein L19 (RPL19) pseudogene
ENSG00000255040	0,54562232	-1,6019728	1,37522281	0,27022976	0,57193402	MORF4L1P3	mortality factor 4 like 1 pseudogene 3 [Source:HGNC Symbol;Acc:HGNC:20402]
ENSG00000008513	-0,1996669	4,44865225	1,37401385	0,27042661	0,57206231	ST3GAL1	ST3 beta-galactoside alpha-2,3-sialyltransferase 1 [Source:HGNC Symbol;Acc:HGNC:10862]
ENSG00000126953	-0,2284464	4,49753896	1,37331475	0,27054053	0,57218079	TIMM8A	translocase of inner mitochondrial membrane 8A [Source:HGNC Symbol;Acc:HGNC:11817]

ENSG00000140950	-0,1865334	3,8222144	1,3727091	0,27063928	0,57218079	MEAK7	MTOR associated protein, eak-7 homolog [Source:HGNC Symbol;Acc:HGNC:29325]
ENSG00000170099	0,1564553	9,01017669	1,37285799	0,270615	0,57218079	SERPINA6	serpin family A member 6 [Source:HGNC Symbol;Acc:HGNC:1540]
ENSG00000178772	-0,2390026	6,91201478	1,37299172	0,2705932	0,57218079	CPN2	carboxypeptidase N subunit 2 [Source:HGNC Symbol;Acc:HGNC:2313]
ENSG00000124193	-0,1551921	6,90569911	1,37048033	0,27100312	0,57234853	SRSF6	serine and arginine rich splicing factor 6 [Source:HGNC Symbol;Acc:HGNC:10788]
ENSG00000137936	0,25365843	3,69514024	1,37108677	0,27090405	0,57234853	BCAR3	BCAR3 adaptor protein, NSP family member [Source:HGNC Symbol;Acc:HGNC:973]
ENSG00000164023	-0,4415962	-0,7892866	1,37173091	0,27079888	0,57234853	SGMS2	sphingomyelin synthase 2 [Source:HGNC Symbol;Acc:HGNC:28395]
ENSG00000164924	0,1921846	7,93524457	1,37030329	0,27103205	0,57234853	YWHAZ	tyrosine 3-monooxygenase/tryptophan 5-monooxygenase activation protein zeta [Source:HGNC Symbol;Acc:HGN
ENSG00000205269	0,32387364	2,00909726	1,37089624	0,27093517	0,57234853	TMEM170B	transmembrane protein 170B [Source:HGNC Symbol;Acc:HGNC:34244]
ENSG00000219773	-0,638397	-1,8092764	1,37061418	0,27098125	0,57234853	RPSAP45	ribosomal protein SA pseudogene 45 [Source:HGNC Symbol;Acc:HGNC:35857]
ENSG00000233594	0,47711137	-0,9204208	1,37054579	0,27099242	0,57234853	BTF3P5	basic transcription factor 3 pseudogene 5 [Source:HGNC Symbol;Acc:HGNC:38565]
ENSG00000265254	-0,5610189	-0,0721438	1,37098355	0,27092091	0,57234853	AC015917.2	novel transcript, antisense to SLC46A1
ENSG00000070756	-0,2093528	8,94896012	1,36996982	0,27108655	0,57238089	PABPC1	poly(A) binding protein cytoplasmic 1 [Source:HGNC Symbol;Acc:HGNC:8554]
ENSG00000100083	-0,164222	4,74955468	1,36845978	0,27133355	0,57243966	GGA1	golgi associated, gamma adaptin ear containing, ARF binding protein 1 [Source:HGNC Symbol;Acc:HGNC:17842]
ENSG00000133131	-0,2192585	5,08031019	1,36797838	0,27141236	0,57243966	MORC4	MORC family CW-type zinc finger 4 [Source:HGNC Symbol;Acc:HGNC:23485]
ENSG00000167523	0,23093904	3,57736016	1,36764452	0,27146704	0,57243966	SPATA33	spermatogenesis associated 33 [Source:HGNC Symbol;Acc:HGNC:26463]
ENSG00000168026	0,5579928	-1,4921736	1,36953823	0,27115711	0,57243966	TTC21A	tetratricopeptide repeat domain 21A [Source:HGNC Symbol;Acc:HGNC:30761]
ENSG00000169439	0,22276868	8,62081771	1,36765144	0,27146591	0,57243966	SDC2	syndecan 2 [Source:HGNC Symbol;Acc:HGNC:10659]
ENSG00000185641	-0,2190672	1,82017409	1,36861404	0,27130831	0,57243966	AC034236.1	ribosomal protein S25 (RPS25) pseudogene
ENSG00000235954	-0,3197275	1,43379827	1,36786628	0,27143072	0,57243966	TTC28-AS1	TTC28 antisense RNA 1 [Source:HGNC Symbol;Acc:HGNC:29336]
ENSG00000259803	-0,1588905	6,08908734	1,36794991	0,27141703	0,57243966	SLC22A31	solute carrier family 22 member 31 [Source:HGNC Symbol;Acc:HGNC:27091]
ENSG00000287733	-0,4305022	0,02129096	1,36858003	0,27131387	0,57243966	AC083862.2	novel transcript
ENSG00000089127	-0,2709844	3,71257917	1,36664256	0,27163122	0,57270321	OAS1	2'-5'-oligoadenylate synthetase 1 [Source:HGNC Symbol;Acc:HGNC:8086]
ENSG00000085760	0,18498695	6,30323931	1,36629878	0,27168759	0,57273938	MTIF2	mitochondrial translational initiation factor 2 [Source:HGNC Symbol;Acc:HGNC:7441]
ENSG00000143545	-0,2344882	2,92767087	1,36526713	0,27185683	0,57301348	RAB13	RAB13, member RAS oncogene family [Source:HGNC Symbol;Acc:HGNC:9762]
ENSG00000129315	0,20582103	3,9901472	1,36429824	0,27201591	0,57326607	CCNT1	cyclin T1 [Source:HGNC Symbol;Acc:HGNC:1599]
ENSG00000091039	0,2008234	3,53062403	1,3632711	0,2721847	0,57351416	OSBPL8	oxysterol binding protein like 8 [Source:HGNC Symbol;Acc:HGNC:16396]
ENSG00000213988	0,38497316	0,58874384	1,36310419	0,27221214	0,57351416	ZNF90	zinc finger protein 90 [Source:HGNC Symbol;Acc:HGNC:13165]
ENSG00000146833	0,17855121	4,409104	1,36281567	0,27225959	0,57353141	TRIM4	tripartite motif containing 4 [Source:HGNC Symbol;Acc:HGNC:16275]
ENSG00000232883	-0,446828	-0,6356143	1,36152081	0,27247266	0,57389751	AL451081.2	ribosomal protein, large, P0 (RPLP0) pseudogene
ENSG00000099282	0,35646816	0,98723913	1,36066563	0,27261351	0,57411141	TSPAN15	tetraspanin 15 [Source:HGNC Symbol;Acc:HGNC:23298]
ENSG00000102221	0,18327498	3,28865901	1,35745266	0,27314362	0,57448247	JADE3	jade family PHD finger 3 [Source:HGNC Symbol;Acc:HGNC:22982]
ENSG00000105852	0,17669874	5,17567925	1,3574553	0,27314319	0,57448247	PON3	paraoxonase 3 [Source:HGNC Symbol;Acc:HGNC:9206]
ENSG00000111361	0,15448025	5,8786414	1,3578684	0,27307495	0,57448247	EIF2B1	eukaryotic translation initiation factor 2B subunit alpha [Source:HGNC Symbol;Acc:HGNC:3257]
ENSG00000128578	0,24221111	1,45680588	1,35887404	0,27290893	0,57448247	STRIP2	striatin interacting protein 2 [Source:HGNC Symbol;Acc:HGNC:22209]
ENSG00000139194	-0,2108366	3,26197328	1,35822289	0,27301641	0,57448247	RBP5	retinol binding protein 5 [Source:HGNC Symbol;Acc:HGNC:15847]
ENSG00000158019	0,17421607	5,09004179	1,35932393	0,27283471	0,57448247	BABAM2	BRISC and BRCA1 A complex member 2 [Source:HGNC Symbol;Acc:HGNC:1106]
ENSG00000188846	-0,1529483	10,6440548	1,3583007	0,27300357	0,57448247	RPL14	ribosomal protein L14 [Source:HGNC Symbol;Acc:HGNC:10305]
ENSG00000198873	-0,1730118	4,29177028	1,35819171	0,27302156	0,57448247	GRK5	G protein-coupled receptor kinase 5 [Source:HGNC Symbol;Acc:HGNC:4544]
ENSG00000254298	-0,7972625	-1,5705038	1,35805879	0,27304351	0,57448247	AC008641.1	novel transcript
ENSG00000137154	-0,1633681	11,3481631	1,35656562	0,27329023	0,57470808	RPS6	ribosomal protein S6 [Source:HGNC Symbol;Acc:HGNC:10429]
ENSG00000054523	0,42464979	4,96988473	1,35457249	0,27362006	0,57477274	KIF1B	kinesin family member 1B [Source:HGNC Symbol;Acc:HGNC:16636]
ENSG00000095015	0,29222278	3,33993869	1,35426794	0,27367051	0,57477274	MAP3K1	mitogen-activated protein kinase kinase kinase 1 [Source:HGNC Symbol;Acc:HGNC:6848]
ENSG00000095261	0,2355189	4,32734523	1,35424035	0,27367508	0,57477274	PSMD5	proteasome 26S subunit, non-ATPase 5 [Source:HGNC Symbol;Acc:HGNC:9563]
ENSG00000115421	0,22357266	3,02407795	1,35570057	0,27343331	0,57477274	PAPOLG	poly(A) polymerase gamma [Source:HGNC Symbol;Acc:HGNC:14982]

ENSG00000117222	0,21003285	4,05219236	1,35568482	0,27343592	0,57477274	RBBP5	RB binding protein 5, histone lysine methyltransferase complex subunit [Source:HGNC Symbol;Acc:HGNC:9888]
ENSG00000124549	0,21018721	2,23040469	1,35427024	0,27367013	0,57477274	BTN2A3P	butyrophilin subfamily 2 member A3, pseudogene [Source:HGNC Symbol;Acc:HGNC:13229]
ENSG00000177981	0,17080437	3,82140266	1,354466	0,2736377	0,57477274	ASB8	ankyrin repeat and SOCS box containing 8 [Source:HGNC Symbol;Acc:HGNC:17183]
ENSG00000185803	-0,1555967	7,19124582	1,3551632	0,27352225	0,57477274	SLC52A2	solute carrier family 52 member 2 [Source:HGNC Symbol;Acc:HGNC:30224]
ENSG00000226976	-0,2238156	1,84884978	1,35535597	0,27349034	0,57477274	COX6A1P2	cytochrome c oxidase subunit 6A1 pseudogene 2 [Source:HGNC Symbol;Acc:HGNC:35239]
ENSG00000011007	0,18397877	6,71556932	1,35391207	0,27372947	0,57480435	ELOA	elongin A [Source:HGNC Symbol;Acc:HGNC:11620]
ENSG00000166484	0,18178361	3,31582246	1,35356762	0,27378656	0,5748416	MAPK7	mitogen-activated protein kinase 7 [Source:HGNC Symbol;Acc:HGNC:6880]
ENSG00000196961	-0,169732	6,49030078	1,35309237	0,27386535	0,57492441	AP2A1	adaptor related protein complex 2 subunit alpha 1 [Source:HGNC Symbol;Acc:HGNC:561]
ENSG00000213420	0,49039957	-0,7551666	1,35281844	0,27391079	0,57493717	GPC2	glypican 2 [Source:HGNC Symbol;Acc:HGNC:4450]
ENSG00000085365	-0,2449719	2,9825376	1,3519812	0,27404971	0,57514613	SCAMP1	secretory carrier membrane protein 1 [Source:HGNC Symbol;Acc:HGNC:10563]
ENSG00000174151	0,20148393	2,50136447	1,3508349	0,27424008	0,57546298	CYB561D1	cytochrome b561 family member D1 [Source:HGNC Symbol;Acc:HGNC:26804]
ENSG00000088387	0,52133507	-1,3469647	1,35018629	0,27434787	0,5754869	DOCK9	dedicator of cytokinesis 9 [Source:HGNC Symbol;Acc:HGNC:14132]
ENSG00000125445	-0,1703835	7,72592644	1,34934471	0,27448783	0,5754869	MRPS7	mitochondrial ribosomal protein S7 [Source:HGNC Symbol;Acc:HGNC:14499]
ENSG00000153904	0,2369844	4,87463177	1,34996511	0,27438465	0,5754869	DDAH1	dimethylarginine dimethylaminohydrolase 1 [Source:HGNC Symbol;Acc:HGNC:2715]
ENSG00000166946	0,18648337	4,28480139	1,35029631	0,27432958	0,5754869	CCNDBP1	cyclin D1 binding protein 1 [Source:HGNC Symbol;Acc:HGNC:1587]
ENSG00000268471	-0,3115635	1,34082615	1,34940791	0,27447732	0,5754869	MIR4453HG	MIR4453 host gene [Source:HGNC Symbol;Acc:HGNC:25288]
ENSG00000272870	0,55827601	-0,6842059	1,34948292	0,27446484	0,5754869	SAP30-DT	SAP30 divergent transcript [Source:HGNC Symbol;Acc:HGNC:54424]
ENSG00000148824	-0,1832644	3,79960721	1,34899786	0,27454554	0,5755253	MTG1	mitochondrial ribosome associated GTPase 1 [Source:HGNC Symbol;Acc:HGNC:32159]
ENSG00000124839	0,18152683	5,44073912	1,34868082	0,27459831	0,57555333	RAB17	RAB17, member RAS oncogene family [Source:HGNC Symbol;Acc:HGNC:16523]
ENSG00000128342	-0,3692919	3,38000624	1,34821185	0,27467639	0,57563439	LIF	LIF interleukin 6 family cytokine [Source:HGNC Symbol;Acc:HGNC:6596]
ENSG00000159650	-0,4497754	-0,5128048	1,34767713	0,27476545	0,57570284	UROC1	urocanate hydratase 1 [Source:HGNC Symbol;Acc:HGNC:26444]
ENSG00000198429	0,28470522	1,7219139	1,34741235	0,27480957	0,57570284	ZNF69	zinc finger protein 69 [Source:HGNC Symbol;Acc:HGNC:13138]
ENSG00000232260	0,71308357	-1,8416436	1,34730612	0,27482727	0,57570284	BTF3L4P1	basic transcription factor 3 like 4 pseudogene 1 [Source:HGNC Symbol;Acc:HGNC:39645]
ENSG00000055609	0,55185928	3,2081304	1,34525627	0,27516918	0,57619743	KMT2C	lysine methyltransferase 2C [Source:HGNC Symbol;Acc:HGNC:13726]
ENSG00000108379	-0,2394415	1,71670353	1,34329292	0,27549723	0,57619743	WNT3	Wnt family member 3 [Source:HGNC Symbol;Acc:HGNC:12782]
ENSG00000116761	-0,2705549	4,62153046	1,34337675	0,27548321	0,57619743	CTH	cystathionine gamma-lyase [Source:HGNC Symbol;Acc:HGNC:2501]
ENSG00000123562	0,20817329	7,79938554	1,34432903	0,27532404	0,57619743	MORF4L2	mortality factor 4 like 2 [Source:HGNC Symbol;Acc:HGNC:16849]
ENSG00000149177	0,25139023	3,55116593	1,34394216	0,27538869	0,57619743	PTPRJ	protein tyrosine phosphatase receptor type J [Source:HGNC Symbol;Acc:HGNC:9673]
ENSG00000167747	-0,2333161	6,52552669	1,34519368	0,27517963	0,57619743	C19orf48	chromosome 19 open reading frame 48 [Source:HGNC Symbol;Acc:HGNC:29667]
ENSG00000172986	-0,2395822	2,7687391	1,34486759	0,27523408	0,57619743	GXYLT2	glucoside xylosyltransferase 2 [Source:HGNC Symbol;Acc:HGNC:33383]
ENSG00000183828	-0,1877873	3,23741267	1,34332793	0,27549138	0,57619743	NUDT14	nudix hydrolase 14 [Source:HGNC Symbol;Acc:HGNC:20141]
ENSG00000187790	0,22295069	2,84024971	1,34511458	0,27519284	0,57619743	FANCM	FA complementation group M [Source:HGNC Symbol;Acc:HGNC:23168]
ENSG00000197535	-0,3361429	2,08355204	1,34367574	0,27543322	0,57619743	MYO5A	myosin VA [Source:HGNC Symbol;Acc:HGNC:7602]
ENSG00000237489	-0,5760249	-1,5793712	1,34333577	0,27549007	0,57619743	C10orf143	chromosome 10 open reading frame 143 [Source:HGNC Symbol;Acc:HGNC:48677]
ENSG00000064995	0,20478937	5,84813937	1,34258401	0,27561581	0,57636293	TAF11	TATA-box binding protein associated factor 11 [Source:HGNC Symbol;Acc:HGNC:11544]
ENSG00000099869	0,41877083	0,62683684	1,34215088	0,2756883	0,57643201	IGF2-AS	IGF2 antisense RNA [Source:HGNC Symbol;Acc:HGNC:14062]
ENSG00000107223	-0,1652299	8,41543948	1,34140908	0,27581251	0,57660918	EDF1	endothelial differentiation related factor 1 [Source:HGNC Symbol;Acc:HGNC:3164]
ENSG00000185163	-0,215623	4,20421223	1,34091548	0,2758952	0,57669953	DDX51	DEAD-box helicase 51 [Source:HGNC Symbol;Acc:HGNC:20082]
ENSG00000137501	-0,7648822	-1,6830839	1,339917	0,27606259	0,57696686	SYTL2	synaptotagmin like 2 [Source:HGNC Symbol;Acc:HGNC:15585]
ENSG00000236993	-0,6147797	-1,8861784	1,33958057	0,27611902	0,57700225	GAPDHP21	glyceraldehyde 3 phosphate dehydrogenase pseudogene 21 [Source:HGNC Symbol;Acc:HGNC:37773]
ENSG00000117155	0,18135557	3,2603352	1,33913495	0,27619379	0,57707596	SSX2IP	SSX family member 2 interacting protein [Source:HGNC Symbol;Acc:HGNC:16509]
ENSG00000196683	-0,1902646	6,79599428	1,33865213	0,27627484	0,57716274	TOMM7	translocase of outer mitochondrial membrane 7 [Source:HGNC Symbol;Acc:HGNC:21648]
ENSG00000158874	-0,1618953	13,3330531	1,33830454	0,2763332	0,57720213	APOA2	apolipoprotein A2 [Source:HGNC Symbol;Acc:HGNC:601]
ENSG00000241839	0,2418269	2,64868011	1,33734018	0,27649522	0,577458	PLEKHO2	pleckstrin homology domain containing O2 [Source:HGNC Symbol;Acc:HGNC:30026]

ENSG00000116641	0,20141159	5,29071818	1,33489177	0,27690719	0,57747293	DOCK7	dedicator of cytokinesis 7 [Source:HGNC Symbol;Acc:HGNC:19190]
ENSG00000121900	-0,3352502	0,52078068	1,33539914	0,27682175	0,57747293	TMEM54	transmembrane protein 54 [Source:HGNC Symbol;Acc:HGNC:24143]
ENSG00000122008	0,21357208	2,51835404	1,33400996	0,27705577	0,57747293	POLK	DNA polymerase kappa [Source:HGNC Symbol;Acc:HGNC:9183]
ENSG00000131374	0,26541102	3,7003019	1,33651397	0,27663414	0,57747293	TBC1D5	TBC1 domain family member 5 [Source:HGNC Symbol;Acc:HGNC:19166]
ENSG00000135476	0,21907458	4,55059369	1,3367892	0,27658786	0,57747293	ESPL1	extra spindle pole bodies like 1, separase [Source:HGNC Symbol;Acc:HGNC:16856]
ENSG00000136450	0,16128296	5,04317951	1,33580553	0,27675334	0,57747293	SRSF1	serine and arginine rich splicing factor 1 [Source:HGNC Symbol;Acc:HGNC:10780]
ENSG00000153767	0,18538909	3,73967153	1,33470981	0,27693784	0,57747293	GTF2E1	general transcription factor IIE subunit 1 [Source:HGNC Symbol;Acc:HGNC:4650]
ENSG00000166321	-0,2436116	1,39631653	1,33487357	0,27691025	0,57747293	NUDT13	nudix hydrolase 13 [Source:HGNC Symbol;Acc:HGNC:18827]
ENSG00000174628	0,23142526	2,66906413	1,33418975	0,27702547	0,57747293	IQCK	IQ motif containing K [Source:HGNC Symbol;Acc:HGNC:28556]
ENSG00000182118	1,08454728	-1,9796419	1,33408114	0,27704377	0,57747293	FAM89A	family with sequence similarity 89 member A [Source:HGNC Symbol;Acc:HGNC:25057]
ENSG00000187605	0,43956081	3,07714172	1,3353921	0,27682293	0,57747293	TET3	tet methylcytosine dioxygenase 3 [Source:HGNC Symbol;Acc:HGNC:28313]
ENSG00000213213	-0,5081915	-0,3909733	1,33581585	0,2767516	0,57747293	CCDC183	coiled-coil domain containing 183 [Source:HGNC Symbol;Acc:HGNC:28236]
ENSG00000229017	0,40787021	-0,1536631	1,33519494	0,27685613	0,57747293	LINC01277	long intergenic non-protein coding RNA 1277 [Source:HGNC Symbol;Acc:HGNC:50334]
ENSG00000259417	0,27349458	2,33244398	1,33699002	0,27655409	0,57747293	CTXND1	cortexin domain containing 1 [Source:HGNC Symbol;Acc:HGNC:50507]
ENSG00000080947	0,6525361	-1,5282557	1,33301517	0,27722353	0,57774016	CROCCP3	CROCC pseudogene 3 [Source:HGNC Symbol;Acc:HGNC:29405]
ENSG00000025039	0,71259055	-1,5924447	1,33192757	0,2774071	0,57795783	RRAGD	Ras related GTP binding D [Source:HGNC Symbol;Acc:HGNC:19903]
ENSG00000170606	0,20921275	8,16905049	1,33213453	0,27737215	0,57795783	HSPA4	heat shock protein family A (Hsp70) member 4 [Source:HGNC Symbol;Acc:HGNC:5237]
ENSG00000114520	0,17256027	5,28040168	1,33099388	0,27756483	0,57820399	SNX4	sorting nexin 4 [Source:HGNC Symbol;Acc:HGNC:11175]
ENSG00000131089	-0,5374454	-0,0519228	1,33048292	0,2776512	0,57821901	ARHGEF9	Cdc42 guanine nucleotide exchange factor 9 [Source:HGNC Symbol;Acc:HGNC:14561]
ENSG00000216285	-0,4464206	-0,994964	1,33059353	0,2776325	0,57821901	AC078819.1	phosphoglycerate mutase 1 (brain) (PGAM1) pseudogene
ENSG00000168283	0,40458521	1,73894558	1,33009145	0,2777174	0,57827444	BMI1	BMI1 proto-oncogene, polycomb ring finger [Source:HGNC Symbol;Acc:HGNC:1066]
ENSG00000108298	-0,149712	11,7297416	1,3297866	0,27776896	0,5782813	RPL19	ribosomal protein L19 [Source:HGNC Symbol;Acc:HGNC:10312]
ENSG00000176490	0,35914799	0,60733726	1,32960398	0,27779986	0,5782813	DIRAS1	DIRAS family GTPase 1 [Source:HGNC Symbol;Acc:HGNC:19127]
ENSG00000163814	0,54013997	0,71042234	1,32928374	0,27785405	0,57831171	CDCP1	CUB domain containing protein 1 [Source:HGNC Symbol;Acc:HGNC:24357]
ENSG00000235381	0,49046541	-0,5257314	1,32897374	0,27790653	0,57833853	AL596202.1	novel transcript
ENSG00000114573	0,21893641	5,04203683	1,32854987	0,2779783	0,57834555	ATP6V1A	ATPase H+ transporting V1 subunit A [Source:HGNC Symbol;Acc:HGNC:851]
ENSG00000167220	0,35899485	1,02919314	1,32848623	0,27798908	0,57834555	HDHD2	haloacid dehalogenase like hydrolase domain containing 2 [Source:HGNC Symbol;Acc:HGNC:25364]
ENSG00000180233	-0,3921356	0,39513523	1,32719079	0,2782086	0,57871985	ZNRF2	zinc and ring finger 2 [Source:HGNC Symbol;Acc:HGNC:22316]
ENSG00000126778	0,18884641	2,68219537	1,32666987	0,27829695	0,5788212	SIX1	SIX homeobox 1 [Source:HGNC Symbol;Acc:HGNC:10887]
ENSG00000065308	0,32713908	6,48361105	1,32334144	0,27886236	0,57885856	TRAM2	translocation associated membrane protein 2 [Source:HGNC Symbol;Acc:HGNC:16855]
ENSG00000100325	-0,1620716	5,65291062	1,3248367	0,27860815	0,57885856	ASCC2	activating signal cointegrator 1 complex subunit 2 [Source:HGNC Symbol;Acc:HGNC:24103]
ENSG00000103319	-0,1723288	4,91495177	1,32321088	0,27888457	0,57885856	EEF2K	eukaryotic elongation factor 2 kinase [Source:HGNC Symbol;Acc:HGNC:24615]
ENSG00000106829	0,51615877	-0,9294732	1,32452711	0,27866076	0,57885856	TLE4	TLE family member 4, transcriptional corepressor [Source:HGNC Symbol;Acc:HGNC:11840]
ENSG00000112379	-0,4004095	2,97478658	1,32500062	0,2785803	0,57885856	ARFGEF3	ARFGEF family member 3 [Source:HGNC Symbol;Acc:HGNC:21213]
ENSG00000114062	0,18083066	4,84660349	1,32378162	0,27878749	0,57885856	UBE3A	ubiquitin protein ligase E3A [Source:HGNC Symbol;Acc:HGNC:12496]
ENSG00000123106	0,21866217	3,19590239	1,32487133	0,27860227	0,57885856	CCDC91	coiled-coil domain containing 91 [Source:HGNC Symbol;Acc:HGNC:24855]
ENSG00000126062	0,1926331	6,67974322	1,32438686	0,27868459	0,57885856	TMEM115	transmembrane protein 115 [Source:HGNC Symbol;Acc:HGNC:30055]
ENSG00000126216	0,1865767	4,40187025	1,32430269	0,2786989	0,57885856	TUBGCP3	tubulin gamma complex associated protein 3 [Source:HGNC Symbol;Acc:HGNC:18598]
ENSG00000128016	-0,402982	2,41064348	1,32316766	0,27889192	0,57885856	ZFP36	ZFP36 ring finger protein [Source:HGNC Symbol;Acc:HGNC:12862]
ENSG00000134909	0,3665039	2,10451659	1,32306575	0,27890926	0,57885856	ARHGAP32	Rho GTPase activating protein 32 [Source:HGNC Symbol;Acc:HGNC:17399]
ENSG00000167766	0,25796386	1,4037837	1,32617633	0,27838068	0,57885856	ZNF83	zinc finger protein 83 [Source:HGNC Symbol;Acc:HGNC:13158]
ENSG00000177189	0,15896409	8,07792646	1,32404899	0,27874203	0,57885856	RPS6KA3	ribosomal protein S6 kinase A3 [Source:HGNC Symbol;Acc:HGNC:10432]
ENSG00000183718	-0,2916117	1,25007367	1,32464598	0,27864056	0,57885856	TRIM52	tripartite motif containing 52 [Source:HGNC Symbol;Acc:HGNC:19024]
ENSG00000237406	0,6724338	-1,8791353	1,32491352	0,2785951	0,57885856	NDUFA9P1	NADH:ubiquinone oxidoreductase subunit A9 pseudogene 1 [Source:HGNC Symbol;Acc:HGNC:19252]

ENSG00000167815	-0,1644445	9,0495326	1,32272612	0,27896706	0,57889628	PRDX2	peroxiredoxin 2 [Source:HGNC Symbol;Acc:HGNC:9353]
ENSG00000204842	0,23161934	4,14442655	1,32214541	0,27906593	0,5790192	ATXN2	ataxin 2 [Source:HGNC Symbol;Acc:HGNC:10555]
ENSG00000114331	0,25604387	2,95042707	1,32135834	0,27920001	0,57913289	ACAP2	ArfGAP with coiled-coil, ankyrin repeat and PH domains 2 [Source:HGNC Symbol;Acc:HGNC:16469]
ENSG00000254986	0,21793601	6,73533079	1,32148111	0,27917909	0,57913289	DPP3	dipeptidyl peptidase 3 [Source:HGNC Symbol;Acc:HGNC:3008]
ENSG00000172466	0,16994975	5,09709374	1,31915647	0,27957558	0,5798296	ZNF24	zinc finger protein 24 [Source:HGNC Symbol;Acc:HGNC:13032]
ENSG00000023228	0,25119189	7,42484853	1,31874452	0,27964593	0,57984874	NDUFS1	NADH:ubiquinone oxidoreductase core subunit S1 [Source:HGNC Symbol;Acc:HGNC:7707]
ENSG00000159433	0,46055701	1,80195733	1,3184696	0,27969289	0,57984874	STARD9	StAR related lipid transfer domain containing 9 [Source:HGNC Symbol;Acc:HGNC:19162]
ENSG00000224877	-0,226018	4,48868771	1,31840523	0,27970388	0,57984874	NDUFAF8	NADH:ubiquinone oxidoreductase complex assembly factor 8 [Source:HGNC Symbol;Acc:HGNC:33551]
ENSG00000152439	0,25911783	2,06096052	1,31738781	0,27987778	0,57996234	ZNF773	zinc finger protein 773 [Source:HGNC Symbol;Acc:HGNC:30487]
ENSG00000167114	-0,1546133	6,21708463	1,3177495	0,27981594	0,57996234	SLC27A4	solute carrier family 27 member 4 [Source:HGNC Symbol;Acc:HGNC:10998]
ENSG00000280120	0,73340697	-2,0221417	1,31746724	0,2798642	0,57996234	AC073857.1	novel transcript
ENSG00000188257	0,35647995	2,72247945	1,31676049	0,27998508	0,5801024	PLA2G2A	phospholipase A2 group IIA [Source:HGNC Symbol;Acc:HGNC:9031]
ENSG00000159377	-0,1584238	8,5452372	1,31533596	0,28022895	0,58052534	PSMB4	proteasome 20S subunit beta 4 [Source:HGNC Symbol;Acc:HGNC:9541]
ENSG00000162601	0,34310742	2,13911248	1,31437183	0,28039417	0,58078526	MYSM1	Myb like, SWIRM and MPN domains 1 [Source:HGNC Symbol;Acc:HGNC:29401]
ENSG00000186998	-0,6694352	-1,3834962	1,3132146	0,28059267	0,58103164	EMID1	EMI domain containing 1 [Source:HGNC Symbol;Acc:HGNC:18036]
ENSG00000187534	-0,4837571	-0,7348452	1,31326781	0,28058353	0,58103164	PRR13P5	proline rich 13 pseudogene 5 [Source:HGNC Symbol;Acc:HGNC:50618]
ENSG00000072110	-0,3373028	8,23235019	1,31147243	0,28089187	0,58140399	ACTN1	actinin alpha 1 [Source:HGNC Symbol;Acc:HGNC:163]
ENSG00000106477	0,1876918	3,88468355	1,31172257	0,28084888	0,58140399	CEP41	centrosomal protein 41 [Source:HGNC Symbol;Acc:HGNC:12370]
ENSG00000157426	-0,2048508	2,36400699	1,31159894	0,28087013	0,58140399	AASDH	aminoadipate-semialdehyde dehydrogenase [Source:HGNC Symbol;Acc:HGNC:23993]
ENSG00000116750	0,16119026	5,43253951	1,31084387	0,28099993	0,58141654	UCHL5	ubiquitin C-terminal hydrolase L5 [Source:HGNC Symbol;Acc:HGNC:19678]
ENSG00000129351	-0,22168	8,09695112	1,31074268	0,28101733	0,58141654	ILF3	interleukin enhancer binding factor 3 [Source:HGNC Symbol;Acc:HGNC:6038]
ENSG00000174444	-0,1567951	11,0574327	1,3111288	0,28095094	0,58141654	RPL4	ribosomal protein L4 [Source:HGNC Symbol;Acc:HGNC:10353]
ENSG00000151876	-0,2144231	2,31749199	1,31023044	0,28110545	0,5815165	FBXO4	F-box protein 4 [Source:HGNC Symbol;Acc:HGNC:13583]
ENSG00000227896	-0,414725	-0,4474556	1,30992608	0,28115782	0,58154249	AL731569.1	novel transcript
ENSG00000141027	0,30208162	5,49144429	1,30968219	0,2811998	0,58154698	NCOR1	nuclear receptor corepressor 1 [Source:HGNC Symbol;Acc:HGNC:7672]
ENSG00000142039	-0,1938811	3,65692352	1,30856324	0,28139251	0,58186315	CCDC97	coiled-coil domain containing 97 [Source:HGNC Symbol;Acc:HGNC:28289]
ENSG00000196781	0,15874901	5,54007038	1,30708419	0,28164752	0,58230804	TLE1	TLE family member 1, transcriptional corepressor [Source:HGNC Symbol;Acc:HGNC:11837]
ENSG00000034053	-0,2083857	1,97811359	1,30663948	0,28172426	0,58233164	APBA2	amyloid beta precursor protein binding family A member 2 [Source:HGNC Symbol;Acc:HGNC:579]
ENSG00000258634	0,49684265	0,40509364	1,30655606	0,28173866	0,58233164	AL160006.1	novel transcript, antisense to ALX3
ENSG00000174943	-0,2216094	1,72764492	1,30559358	0,28190486	0,58259274	KCTD13	potassium channel tetramerization domain containing 13 [Source:HGNC Symbol;Acc:HGNC:22234]
ENSG00000138326	-0,1636778	10,1531232	1,3026782	0,28240913	0,58355233	RPS24	ribosomal protein S24 [Source:HGNC Symbol;Acc:HGNC:10411]
ENSG00000255153	0,45627504	-0,6458067	1,30227279	0,28247936	0,58361489	TOLLIP-AS1	TOLLIP antisense RNA 1 (head to head) [Source:HGNC Symbol;Acc:HGNC:27403]
ENSG00000100568	0,20625561	5,41447991	1,30021251	0,28283662	0,584243	VTI1B	vesicle transport through interaction with t-SNAREs 1B [Source:HGNC Symbol;Acc:HGNC:17793]
ENSG00000255277	-0,2925568	1,93019214	1,30005848	0,28286335	0,584243	ABCC6P2	ATP binding cassette subfamily C member 6 pseudogene 2 [Source:HGNC Symbol;Acc:HGNC:33353]
ENSG00000032389	-0,1742345	6,14965552	1,29900486	0,28304633	0,58453829	EIPR1	EARP complex and GARP complex interacting protein 1 [Source:HGNC Symbol;Acc:HGNC:12383]
ENSG00000132570	-0,1786745	3,08870108	1,29786644	0,28324422	0,58478163	PCBD2	pterin-4 alpha-carbinolamine dehydratase 2 [Source:HGNC Symbol;Acc:HGNC:24474]
ENSG00000138032	0,21192274	2,77860715	1,29789319	0,28323957	0,58478163	PPM1B	protein phosphatase, Mg2+/Mn2+ dependent 1B [Source:HGNC Symbol;Acc:HGNC:9276]
ENSG00000260747	0,35322187	-0,1276924	1,29754732	0,28329973	0,58481359	AC022968.1	ATP synthase, H+ transporting, mitochondrial F0 complex, subunit B1 (ATP5F1) pseudogene
ENSG00000196505	0,19450099	3,96367934	1,29679829	0,28343007	0,585	GDAP2	ganglioside induced differentiation associated protein 2 [Source:HGNC Symbol;Acc:HGNC:18010]
ENSG00000107679	0,18341628	3,17468676	1,29576999	0,28360916	0,58528694	PLEKHA1	pleckstrin homology domain containing A1 [Source:HGNC Symbol;Acc:HGNC:14335]
ENSG00000101444	0,18350256	9,91606154	1,29486145	0,28376752	0,58533001	AHCY	adenosylhomocysteinase [Source:HGNC Symbol;Acc:HGNC:343]
ENSG00000106526	-0,3169717	1,14987443	1,29473082	0,2837903	0,58533001	ACTR3C	actin related protein 3C [Source:HGNC Symbol;Acc:HGNC:37282]
ENSG00000136878	-0,2385997	2,51419177	1,29494304	0,28375329	0,58533001	USP20	ubiquitin specific peptidase 20 [Source:HGNC Symbol;Acc:HGNC:12619]
ENSG00000152229	0,54220141	-1,7244094	1,29493188	0,28375524	0,58533001	PSTPIP2	proline-serine-threonine phosphatase interacting protein 2 [Source:HGNC Symbol;Acc:HGNC:9581]

ENSG00000109971	-0,1661588	10,8302149	1,29330843	0,28403851	0,58567658	HSPA8	heat shock protein family A (Hsp70) member 8 [Source:HGNC Symbol;Acc:HGNC:5241]
ENSG00000112494	0,25527607	3,77900133	1,2934159	0,28401974	0,58567658	UNC93A	unc-93 homolog A [Source:HGNC Symbol;Acc:HGNC:12570]
ENSG00000275457	-0,5038921	0,38867591	1,29283339	0,28412147	0,58576497	AL117332.1	novel transcript, antisense to XRN2
ENSG00000128944	0,17025445	5,64782778	1,29224137	0,28422491	0,58589554	KNSTRN	kinetochore localized astrin (SPAG5) binding protein [Source:HGNC Symbol;Acc:HGNC:30767]
ENSG00000116035	0,35302683	1,38558292	1,29128037	0,28439294	0,58591121	VAX2	ventral anterior homeobox 2 [Source:HGNC Symbol;Acc:HGNC:12661]
ENSG00000137076	-0,4263942	6,94380927	1,29135936	0,28437912	0,58591121	TLN1	talin 1 [Source:HGNC Symbol;Acc:HGNC:11845]
ENSG00000169592	-0,1603036	6,01331183	1,29172783	0,28431468	0,58591121	INO80E	INO80 complex subunit E [Source:HGNC Symbol;Acc:HGNC:26905]
ENSG00000188219	-0,2432537	2,15802296	1,29190353	0,28428396	0,58591121	POTEE	POTE ankyrin domain family member E [Source:HGNC Symbol;Acc:HGNC:33895]
ENSG00000107949	-0,2065546	6,82912529	1,29053444	0,28452346	0,58609746	BCCIP	BRCA2 and CDKN1A interacting protein [Source:HGNC Symbol;Acc:HGNC:978]
ENSG00000079819	0,29577333	6,73327263	1,29025284	0,28457275	0,58611635	EPB41L2	erythrocyte membrane protein band 4.1 like 2 [Source:HGNC Symbol;Acc:HGNC:3379]
ENSG00000038274	0,16920419	5,37634773	1,28978174	0,28465524	0,58615354	MAT2B	methionine adenosyltransferase 2B [Source:HGNC Symbol;Acc:HGNC:6905]
ENSG00000136819	0,18814631	5,57987689	1,28959286	0,28468833	0,58615354	C9orf78	chromosome 9 open reading frame 78 [Source:HGNC Symbol;Acc:HGNC:24932]
ENSG00000159164	0,28374471	2,01341718	1,28946245	0,28471117	0,58615354	SV2A	synaptic vesicle glycoprotein 2A [Source:HGNC Symbol;Acc:HGNC:20566]
ENSG00000091622	0,62416317	-1,6550996	1,28852146	0,2848761	0,58641045	PITPNM3	PITPNM family member 3 [Source:HGNC Symbol;Acc:HGNC:21043]
ENSG00000065357	-0,2971552	0,78416112	1,2878738	0,28498969	0,586479	DGKA	diacylglycerol kinase alpha [Source:HGNC Symbol;Acc:HGNC:2849]
ENSG00000129515	0,17408477	6,78163866	1,28798551	0,2849701	0,586479	SNX6	sorting nexin 6 [Source:HGNC Symbol;Acc:HGNC:14970]
ENSG00000171291	0,339669	0,95466883	1,28688327	0,28516355	0,5867206	ZNF439	zinc finger protein 439 [Source:HGNC Symbol;Acc:HGNC:20873]
ENSG00000198799	-0,24031	3,59034269	1,28674734	0,28518742	0,5867206	LRIG2	leucine rich repeats and immunoglobulin like domains 2 [Source:HGNC Symbol;Acc:HGNC:20889]
ENSG00000103942	0,19448299	2,5869529	1,28411957	0,28564941	0,58733332	HOMER2	homer scaffold protein 2 [Source:HGNC Symbol;Acc:HGNC:17513]
ENSG00000105662	-0,3083732	2,7974238	1,28362175	0,28573706	0,58733332	CRTC1	CREB regulated transcription coactivator 1 [Source:HGNC Symbol;Acc:HGNC:16062]
ENSG00000115904	0,16764551	3,94483941	1,28431767	0,28561455	0,58733332	SOS1	SOS Ras/Rac guanine nucleotide exchange factor 1 [Source:HGNC Symbol;Acc:HGNC:11187]
ENSG00000131408	-0,219918	4,05857273	1,28381172	0,28570361	0,58733332	NR1H2	nuclear receptor subfamily 1 group H member 2 [Source:HGNC Symbol;Acc:HGNC:7965]
ENSG00000132467	-0,1735584	5,47659693	1,28471525	0,28554459	0,58733332	UTP3	UTP3 small subunit processome component [Source:HGNC Symbol;Acc:HGNC:24477]
ENSG00000164758	0,26387075	3,68103499	1,28371828	0,28572006	0,58733332	MED30	mediator complex subunit 30 [Source:HGNC Symbol;Acc:HGNC:23032]
ENSG00000275632	-0,58736	-1,4238678	1,28345361	0,28576667	0,58733332	AL035461.2	novel transcript
ENSG00000119403	0,16672121	5,58590328	1,28281932	0,28587841	0,58748034	PHF19	PHD finger protein 19 [Source:HGNC Symbol;Acc:HGNC:24566]
ENSG00000007541	-0,1869016	3,9523857	1,2823705	0,28595752	0,58749068	PIGQ	phosphatidylinositol glycan anchor biosynthesis class Q [Source:HGNC Symbol;Acc:HGNC:14135]
ENSG00000169193	0,35224067	0,96531258	1,28233443	0,28596388	0,58749068	CCDC126	coiled-coil domain containing 126 [Source:HGNC Symbol;Acc:HGNC:22398]
ENSG00000065054	-0,1704547	5,25038023	1,28077686	0,28623865	0,58755943	SLC9A3R2	SLC9A3 regulator 2 [Source:HGNC Symbol;Acc:HGNC:11076]
ENSG00000095209	0,16055674	5,34407831	1,28087017	0,28622218	0,58755943	TMEM38B	transmembrane protein 38B [Source:HGNC Symbol;Acc:HGNC:25535]
ENSG00000163933	-0,1930454	5,04463723	1,28156413	0,28609972	0,58755943	RFT1	RFT1 homolog [Source:HGNC Symbol;Acc:HGNC:30220]
ENSG00000166289	-0,2287262	1,53174731	1,28156935	0,2860988	0,58755943	PLEKHF1	pleckstrin homology and FYVE domain containing 1 [Source:HGNC Symbol;Acc:HGNC:20764]
ENSG00000205060	0,17019869	3,64392471	1,28079168	0,28623603	0,58755943	SLC35B4	solute carrier family 35 member B4 [Source:HGNC Symbol;Acc:HGNC:20584]
ENSG00000227081	-0,245886	1,60755357	1,28095207	0,28620772	0,58755943	AC005912.1	ribosomal protein S27 (RPS27) pseudogene
ENSG00000090615	0,31824308	5,17385056	1,27980065	0,28641106	0,58783073	GOLGA3	golgin A3 [Source:HGNC Symbol;Acc:HGNC:4426]
ENSG00000109184	-0,1777771	3,17816332	1,27944757	0,28647345	0,58787619	DCUN1D4	defective in cullin neddylation 1 domain containing 4 [Source:HGNC Symbol;Acc:HGNC:28998]
ENSG00000169919	0,20846816	6,80605215	1,27877011	0,28659322	0,58803937	GUSB	glucuronidase beta [Source:HGNC Symbol;Acc:HGNC:4696]
ENSG00000176244	0,57425677	-0,8773902	1,27780364	0,28676421	0,5881424	ACBD7	acyl-CoA binding domain containing 7 [Source:HGNC Symbol;Acc:HGNC:17715]
ENSG00000204977	0,19192339	4,09442636	1,27808941	0,28671363	0,5881424	TRIM13	tripartite motif containing 13 [Source:HGNC Symbol;Acc:HGNC:9976]
ENSG00000213683	-0,3809111	-0,1604998	1,27794798	0,28673866	0,5881424	AC002056.1	small nuclear ribonucleoprotein polypeptide A' (SNRPA1) pseudogene
ENSG00000214889	-0,3953907	-0,6079543	1,27669245	0,28696098	0,58846335	RPS9P1	ribosomal protein S9 pseudogene 1 [Source:HGNC Symbol;Acc:HGNC:10443]
ENSG00000160200	-0,228458	2,59057075	1,27644894	0,28700412	0,58846923	CBS	cystathionine beta-synthase [Source:HGNC Symbol;Acc:HGNC:1550]
ENSG00000100554	0,22505658	5,2506546	1,27520951	0,28722388	0,58873972	ATP6V1D	ATPase H+ transporting V1 subunit D [Source:HGNC Symbol;Acc:HGNC:13527]
ENSG00000111801	0,30803631	0,9409226	1,27482643	0,28729185	0,58873972	BTN3A3	butyrophilin subfamily 3 member A3 [Source:HGNC Symbol;Acc:HGNC:1140]

ENSG00000116171	0,1701946	6,23550031	1,27392535	0,28745181	0,58873972	SCP2	sterol carrier protein 2 [Source:HGNC Symbol;Acc:HGNC:10606]
ENSG00000118564	0,17413868	4,18116717	1,27400798	0,28743714	0,58873972	FBXL5	F-box and leucine rich repeat protein 5 [Source:HGNC Symbol;Acc:HGNC:13602]
ENSG00000135924	0,16285821	5,0979328	1,27445258	0,2873582	0,58873972	DNAJB2	DnaJ heat shock protein family (Hsp40) member B2 [Source:HGNC Symbol;Acc:HGNC:5228]
ENSG00000136891	0,17329469	4,86931839	1,27477394	0,28730116	0,58873972	TEX10	testis expressed 10 [Source:HGNC Symbol;Acc:HGNC:25988]
ENSG00000145715	0,18419135	3,31385527	1,27366114	0,28749874	0,58873972	RASA1	RAS p21 protein activator 1 [Source:HGNC Symbol;Acc:HGNC:9871]
ENSG00000244045	0,16055083	5,11235389	1,27397734	0,28744258	0,58873972	TMEM199	transmembrane protein 199 [Source:HGNC Symbol;Acc:HGNC:18085]
ENSG00000248334	0,50364582	-0,5801524	1,27384688	0,28746575	0,58873972	WHAMMP2	WAS protein homolog associated with actin, golgi membranes and microtubules pseudogene 2 [Source:HGNC Syr
ENSG00000164252	-0,2827097	2,03400402	1,27335412	0,28755329	0,58876888	AGGF1	angiogenic factor with G-patch and FHA domains 1 [Source:HGNC Symbol;Acc:HGNC:24684]
ENSG00000065243	0,22539394	4,27988036	1,27144699	0,28789245	0,58877455	PKN2	protein kinase N2 [Source:HGNC Symbol;Acc:HGNC:9406]
ENSG00000075539	0,23224739	2,29529276	1,27062006	0,28803968	0,58877455	FRYL	FRY like transcription coactivator [Source:HGNC Symbol;Acc:HGNC:29127]
ENSG00000100890	0,24342499	1,89329836	1,27107812	0,28795811	0,58877455	PRORP	protein only RNase P catalytic subunit [Source:HGNC Symbol;Acc:HGNC:19958]
ENSG00000147164	0,16980243	4,54222603	1,27102054	0,28796836	0,58877455	SNX12	sorting nexin 12 [Source:HGNC Symbol;Acc:HGNC:14976]
ENSG00000148516	-0,2604705	2,64758297	1,27097025	0,28797732	0,58877455	ZEB1	zinc finger E-box binding homeobox 1 [Source:HGNC Symbol;Acc:HGNC:11642]
ENSG00000187624	0,53070323	-0,9214414	1,27280612	0,28765068	0,58877455	C17orf97	chromosome 17 open reading frame 97 [Source:HGNC Symbol;Acc:HGNC:33800]
ENSG00000196968	-0,2125413	2,76232408	1,27209219	0,28777764	0,58877455	FUT11	fucosyltransferase 11 [Source:HGNC Symbol;Acc:HGNC:19233]
ENSG00000198093	0,23312275	2,47678893	1,27188667	0,2878142	0,58877455	ZNF649	zinc finger protein 649 [Source:HGNC Symbol;Acc:HGNC:25741]
ENSG00000235100	-0,5635797	-1,6813527	1,27195794	0,28780153	0,58877455	AL157400.3	novel transcript
ENSG00000244701	-0,6104672	-1,7381817	1,27245799	0,28771258	0,58877455	AC004918.1	novel transcript, antisense to AGK
ENSG00000259020	-0,5876146	-1,793128	1,27240684	0,28772168	0,58877455	AL049872.1	prothymosin, alpha (PTMA) pseudogene
ENSG00000285219	0,28610865	0,86367811	1,27066287	0,28803206	0,58877455	HULC	hepatocellular carcinoma up-regulated long non-coding RNA [Source:HGNC Symbol;Acc:HGNC:34232]
ENSG00000229132	-0,1886787	3,95338315	1,27026858	0,2881023	0,58882015	EIF4A1P10	eukaryotic translation initiation factor 4A1 pseudogene 10 [Source:HGNC Symbol;Acc:HGNC:37930]
ENSG00000062282	0,16477561	7,4505415	1,26917093	0,28829796	0,58903893	DGAT2	diacylglycerol O-acyltransferase 2 [Source:HGNC Symbol;Acc:HGNC:16940]
ENSG00000173402	0,22875393	7,17832402	1,26909363	0,28831175	0,58903893	DAG1	dystroglycan 1 [Source:HGNC Symbol;Acc:HGNC:2666]
ENSG00000197429	0,26036702	2,11075705	1,26898959	0,2883303	0,58903893	IPP	intracisternal A particle-promoted polypeptide [Source:HGNC Symbol;Acc:HGNC:6108]
ENSG00000111850	-0,2515477	1,60595692	1,26812028	0,28848542	0,58922114	SMIM8	small integral membrane protein 8 [Source:HGNC Symbol;Acc:HGNC:21401]
ENSG00000146963	0,2017535	3,44053103	1,26803774	0,28850016	0,58922114	LUC7L2	LUC7 like 2, pre-mRNA splicing factor [Source:HGNC Symbol;Acc:HGNC:21608]
ENSG00000120800	0,1826293	5,12350719	1,26711094	0,28866568	0,58944258	UTP20	UTP20 small subunit processome component [Source:HGNC Symbol;Acc:HGNC:17897]
ENSG00000181773	-0,2368	1,93559506	1,26655247	0,28876548	0,58944258	GPR3	G protein-coupled receptor 3 [Source:HGNC Symbol;Acc:HGNC:4484]
ENSG00000185115	0,1765725	4,89240778	1,26664028	0,28874979	0,58944258	NSMCE3	NSE3 homolog, SMC5-SMC6 complex component [Source:HGNC Symbol;Acc:HGNC:7677]
ENSG00000218358	-0,5496627	-1,643292	1,26652735	0,28876997	0,58944258	RAET1K	retinoic acid early transcript 1K pseudogene [Source:HGNC Symbol;Acc:HGNC:16797]
ENSG00000164054	-0,1519895	5,88407897	1,26522897	0,28900221	0,58975181	SHISA5	shisa family member 5 [Source:HGNC Symbol;Acc:HGNC:30376]
ENSG00000164347	0,16643338	5,17539911	1,2653058	0,28898846	0,58975181	GFM2	GTP dependent ribosome recycling factor mitochondrial 2 [Source:HGNC Symbol;Acc:HGNC:29682]
ENSG00000213082	-0,5188831	-1,3878301	1,26471462	0,28909428	0,5898573	PPP1R14BP2	protein phosphatase 1 regulatory inhibitor subunit 14B pseudogene 2 [Source:HGNC Symbol;Acc:HGNC:16329]
ENSG00000141084	0,20437038	4,08330491	1,26391795	0,28923697	0,58998364	RANBP10	RAN binding protein 10 [Source:HGNC Symbol;Acc:HGNC:29285]
ENSG00000164032	-0,187204	8,85935038	1,26397054	0,28922754	0,58998364	H2AZ1	H2A.Z variant histone 1 [Source:HGNC Symbol;Acc:HGNC:4741]
ENSG00000149100	0,15366083	7,92632375	1,26308934	0,28938548	0,59012179	EIF3M	eukaryotic translation initiation factor 3 subunit M [Source:HGNC Symbol;Acc:HGNC:24460]
ENSG00000164548	0,17348198	5,71174969	1,26317447	0,28937022	0,59012179	TRA2A	transformer 2 alpha homolog [Source:HGNC Symbol;Acc:HGNC:16645]
ENSG00000092199	-0,1537932	9,8103354	1,2625575	0,28948087	0,59015155	HNRNPC	heterogeneous nuclear ribonucleoprotein C [Source:HGNC Symbol;Acc:HGNC:5035]
ENSG00000137504	0,16582351	3,77738063	1,26261004	0,28947144	0,59015155	CREBZF	CREB/ATF bZIP transcription factor [Source:HGNC Symbol;Acc:HGNC:24905]
ENSG00000149311	0,2614219	3,46026823	1,26173399	0,28962865	0,5902008	ATM	ATM serine/threonine kinase [Source:HGNC Symbol;Acc:HGNC:795]
ENSG00000166262	0,57635202	-0,9957753	1,26197703	0,28958502	0,5902008	FAM227B	family with sequence similarity 227 member B [Source:HGNC Symbol;Acc:HGNC:26543]
ENSG00000224415	-0,2101071	2,04552042	1,26159001	0,2896545	0,5902008	AC007683.1	ribosomal protein L19 (RPL19) pseudogene
ENSG00000273841	-0,2077699	5,98302447	1,26152245	0,28966663	0,5902008	TAF9	TATA-box binding protein associated factor 9 [Source:HGNC Symbol;Acc:HGNC:11542]
ENSG00000092094	-0,1643101	6,04791721	1,26082373	0,28979212	0,59034526	OSGEP	O-sialoglycoprotein endopeptidase [Source:HGNC Symbol;Acc:HGNC:18028]

ENSG00000278376	-0,477309	-0,5228861	1,26067777	0,28981835	0,59034526	AP004609.3	novel transcript
ENSG00000095397	-0,2513958	1,45133049	1,25811092	0,2902801	0,59096235	WHRN	whirlin [Source:HGNC Symbol;Acc:HGNC:16361]
ENSG00000117148	-0,5282306	-1,0327211	1,25786965	0,29032355	0,59096235	ACTL8	actin like 8 [Source:HGNC Symbol;Acc:HGNC:24018]
ENSG00000132661	-0,1955159	5,49763647	1,25824111	0,29025665	0,59096235	NXT1	nuclear transport factor 2 like export factor 1 [Source:HGNC Symbol;Acc:HGNC:15913]
ENSG00000132694	0,20315804	4,16606461	1,25801184	0,29029794	0,59096235	ARHGEF11	Rho guanine nucleotide exchange factor 11 [Source:HGNC Symbol;Acc:HGNC:14580]
ENSG00000149541	-0,1739356	5,65202708	1,25800038	0,29030001	0,59096235	B3GAT3	beta-1,3-glucuronyltransferase 3 [Source:HGNC Symbol;Acc:HGNC:923]
ENSG00000147799	0,23085724	2,03416784	1,25739507	0,29040906	0,59105404	ARHGAP39	Rho GTPase activating protein 39 [Source:HGNC Symbol;Acc:HGNC:29351]
ENSG00000064787	-0,428412	-0,7011081	1,25582189	0,29069276	0,59147536	BCAS1	brain enriched myelin associated protein 1 [Source:HGNC Symbol;Acc:HGNC:974]
ENSG00000187446	0,1659612	5,53707159	1,25579814	0,29069704	0,59147536	CHP1	calcineurin like EF-hand protein 1 [Source:HGNC Symbol;Acc:HGNC:17433]
ENSG00000153443	-0,1805193	4,28157734	1,25386599	0,29104602	0,59202051	UBALD1	UBA like domain containing 1 [Source:HGNC Symbol;Acc:HGNC:29576]
ENSG00000174227	0,21795092	5,31597188	1,25393948	0,29103274	0,59202051	PIGG	phosphatidylinositol glycan anchor biosynthesis class G [Source:HGNC Symbol;Acc:HGNC:25985]
ENSG00000099991	0,20570676	5,64943324	1,2531915	0,29116799	0,59210372	CABIN1	calcineurin binding protein 1 [Source:HGNC Symbol;Acc:HGNC:24187]
ENSG00000173726	0,14765927	9,09942024	1,2533814	0,29113364	0,59210372	TOMM20	translocase of outer mitochondrial membrane 20 [Source:HGNC Symbol;Acc:HGNC:20947]
ENSG00000138668	-0,1881541	8,25695842	1,25290744	0,29121938	0,5921258	HNRNPD	heterogeneous nuclear ribonucleoprotein D [Source:HGNC Symbol;Acc:HGNC:5036]
ENSG00000171119	0,37953526	1,36278528	1,25254558	0,29128486	0,59217652	NRTN	neurturin [Source:HGNC Symbol;Acc:HGNC:8007]
ENSG00000115091	0,16452987	7,9048274	1,25141436	0,29148969	0,59244653	ACTR3	actin related protein 3 [Source:HGNC Symbol;Acc:HGNC:170]
ENSG00000214717	0,16996118	5,90265131	1,25136419	0,29149878	0,59244653	ZBED1	zinc finger BED-type containing 1 [Source:HGNC Symbol;Acc:HGNC:447]
ENSG00000198744	0,1778835	3,58888085	1,2511007	0,29154652	0,59246114	MTCO3P12	MT-CO3 pseudogene 12 [Source:HGNC Symbol;Acc:HGNC:52042]
ENSG00000166452	-0,1646218	4,11499487	1,25075433	0,2916093	0,5925063	AKIP1	A-kinase interacting protein 1 [Source:HGNC Symbol;Acc:HGNC:1170]
ENSG00000129474	0,14943387	6,82609418	1,2503689	0,29167918	0,59256587	AJUBA	ajuba LIM protein [Source:HGNC Symbol;Acc:HGNC:20250]
ENSG00000140400	-0,2223974	4,19362799	1,24998528	0,29174876	0,59259006	MAN2C1	mannosidase alpha class 2C member 1 [Source:HGNC Symbol;Acc:HGNC:6827]
ENSG00000141446	0,19347017	3,47312204	1,24940886	0,29185334	0,59259006	ESCO1	establishment of sister chromatid cohesion N-acetyltransferase 1 [Source:HGNC Symbol;Acc:HGNC:24645]
ENSG00000188807	0,21427167	4,98099825	1,24984546	0,29177412	0,59259006	TMEM201	transmembrane protein 201 [Source:HGNC Symbol;Acc:HGNC:33719]
ENSG00000238083	-0,3204662	0,81382779	1,24948689	0,29183918	0,59259006	LRRC37A2	leucine rich repeat containing 37 member A2 [Source:HGNC Symbol;Acc:HGNC:32404]
ENSG00000119013	-0,1770761	6,06112251	1,24848114	0,29202178	0,59283791	NDUFB3	NADH:ubiquinone oxidoreductase subunit B3 [Source:HGNC Symbol;Acc:HGNC:7698]
ENSG00000135945	-0,1648996	4,54184872	1,24828963	0,29205657	0,59283791	REV1	REV1 DNA directed polymerase [Source:HGNC Symbol;Acc:HGNC:14060]
ENSG00000010165	0,15021838	5,71841534	1,24660582	0,2923627	0,59297557	EEF1AKNMT	eEF1A lysine and N-terminal methyltransferase [Source:HGNC Symbol;Acc:HGNC:24248]
ENSG00000104695	-0,1608429	5,34998345	1,24655434	0,29237206	0,59297557	PPP2CB	protein phosphatase 2 catalytic subunit beta [Source:HGNC Symbol;Acc:HGNC:9300]
ENSG00000132386	0,18393331	10,3101084	1,24635404	0,29240851	0,59297557	SERPINF1	serpin family F member 1 [Source:HGNC Symbol;Acc:HGNC:8824]
ENSG00000160208	0,19814682	5,83397725	1,24714469	0,29226468	0,59297557	RRP1B	ribosomal RNA processing 1B [Source:HGNC Symbol;Acc:HGNC:23818]
ENSG00000168056	0,1854306	5,37836127	1,24747037	0,29220546	0,59297557	LTBP3	latent transforming growth factor beta binding protein 3 [Source:HGNC Symbol;Acc:HGNC:6716]
ENSG00000218502	-0,286237	0,73290102	1,24648008	0,29238558	0,59297557	H2AZP3	H2A.Z histone pseudogene 3 [Source:HGNC Symbol;Acc:HGNC:38013]
ENSG00000224059	-0,5885043	-1,6714119	1,24742856	0,29221306	0,59297557	HSPA8P16	heat shock protein family A (Hsp70) member 8 pseudogene 16 [Source:HGNC Symbol;Acc:HGNC:44931]
ENSG00000004455	-0,1862301	7,44797309	1,24578485	0,29251212	0,59310334	AK2	adenylate kinase 2 [Source:HGNC Symbol;Acc:HGNC:362]
ENSG00000074319	0,15532707	6,50633199	1,24409566	0,29281991	0,5932402	TSG101	tumor susceptibility 101 [Source:HGNC Symbol;Acc:HGNC:15971]
ENSG00000157087	-0,3429918	1,5732196	1,24407725	0,29282327	0,5932402	ATP2B2	ATPase plasma membrane Ca2+ transporting 2 [Source:HGNC Symbol;Acc:HGNC:815]
ENSG00000160959	0,23345636	3,31713286	1,24425901	0,29279013	0,5932402	LRRC14	leucine rich repeat containing 14 [Source:HGNC Symbol;Acc:HGNC:20419]
ENSG00000161277	-0,2423713	2,01598345	1,24459042	0,29272971	0,5932402	THAP8	THAP domain containing 8 [Source:HGNC Symbol;Acc:HGNC:23191]
ENSG00000163939	0,27402805	4,40383218	1,24499089	0,29265673	0,5932402	PBRM1	polybromo 1 [Source:HGNC Symbol;Acc:HGNC:30064]
ENSG00000263563	-0,1947598	3,48172021	1,24412981	0,29281368	0,5932402	UBBP4	ubiquitin B pseudogene 4 [Source:HGNC Symbol;Acc:HGNC:12467]
ENSG00000034713	0,18431705	5,65130204	1,24241338	0,2931269	0,59361856	GABARAPL2	GABA type A receptor associated protein like 2 [Source:HGNC Symbol;Acc:HGNC:13291]
ENSG00000051108	0,15325799	8,06680609	1,24247654	0,29311537	0,59361856	HERPUD1	homocysteine inducible ER protein with ubiquitin like domain 1 [Source:HGNC Symbol;Acc:HGNC:13744]
ENSG00000139190	0,38069499	-0,04034	1,24216341	0,29317256	0,59361856	VAMP1	vesicle associated membrane protein 1 [Source:HGNC Symbol;Acc:HGNC:12642]
ENSG00000142541	-0,1578978	11,6584746	1,24232022	0,29314392	0,59361856	RPL13A	ribosomal protein L13a [Source:HGNC Symbol;Acc:HGNC:10304]

ENSG00000139679	0,19928932	4,45269941	1,24119383	0,29334974	0,593895	LPAR6	lysophosphatidic acid receptor 6 [Source:HGNC Symbol;Acc:HGNC:15520]
ENSG00000140876	0,28993237	2,56275677	1,24087064	0,29340884	0,59393234	NUDT7	nudix hydrolase 7 [Source:HGNC Symbol;Acc:HGNC:8054]
ENSG00000142065	0,31246617	0,61138264	1,24035924	0,29350238	0,59395709	ZFP14	ZFP14 zinc finger protein [Source:HGNC Symbol;Acc:HGNC:29312]
ENSG00000237943	-0,2321932	1,4460571	1,24051962	0,29347304	0,59395709	PRKCQ-AS1	PRKCQ antisense RNA 1 [Source:HGNC Symbol;Acc:HGNC:44689]
ENSG00000006118	-0,1705333	5,04758288	1,239801	0,29360454	0,59397991	TMEM132A	transmembrane protein 132A [Source:HGNC Symbol;Acc:HGNC:31092]
ENSG00000174839	-0,202328	2,1317617	1,23963114	0,29363563	0,59397991	DENND6A	DENN domain containing 6A [Source:HGNC Symbol;Acc:HGNC:26635]
ENSG00000196284	-0,1711172	4,08526959	1,23987334	0,29359129	0,59397991	SUPT3H	SPT3 homolog, SAGA and STAGA complex component [Source:HGNC Symbol;Acc:HGNC:11466]
ENSG00000221926	0,23999838	1,74089097	1,2392594	0,2937037	0,59403535	TRIM16	tripartite motif containing 16 [Source:HGNC Symbol;Acc:HGNC:17241]
ENSG00000165684	-0,2039909	4,23890339	1,23769539	0,29399032	0,59447912	SNAPC4	small nuclear RNA activating complex polypeptide 4 [Source:HGNC Symbol;Acc:HGNC:11137]
ENSG00000182774	0,24426664	1,55250411	1,23761814	0,29400449	0,59447912	RPS17	ribosomal protein S17 [Source:HGNC Symbol;Acc:HGNC:10397]
ENSG00000228393	-0,5082791	-1,1019607	1,23735022	0,29405364	0,59449621	LINC01004	long intergenic non-protein coding RNA 1004 [Source:HGNC Symbol;Acc:HGNC:48961]
ENSG00000280409	0,37316202	-0,1013536	1,23595502	0,29430974	0,59493165	LINC01101	long intergenic non-protein coding RNA 1101 [Source:HGNC Symbol;Acc:HGNC:25923]
ENSG00000005075	-0,1870401	5,31909038	1,23551791	0,29439005	0,59501165	POLR2J	RNA polymerase II subunit J [Source:HGNC Symbol;Acc:HGNC:9197]
ENSG00000134759	-0,1599598	5,86160335	1,23468764	0,29454266	0,59523776	ELP2	elongator acetyltransferase complex subunit 2 [Source:HGNC Symbol;Acc:HGNC:18248]
ENSG00000162814	0,42588104	-0,3550674	1,23405088	0,29465979	0,59530976	SPATA17	spermatogenesis associated 17 [Source:HGNC Symbol;Acc:HGNC:25184]
ENSG00000214193	-0,2524826	2,36546647	1,2341381	0,29464374	0,59530976	SH3D21	SH3 domain containing 21 [Source:HGNC Symbol;Acc:HGNC:26236]
ENSG00000140836	0,62395198	3,86159429	1,23351152	0,29475905	0,59542795	ZFHX3	zinc finger homeobox 3 [Source:HGNC Symbol;Acc:HGNC:777]
ENSG00000260081	0,57974733	-1,2134178	1,23323575	0,29480982	0,59544817	AF274858.1	Morf4 family associated protein 1 (MRFAP1) pseudogene
ENSG00000106628	-0,1882851	6,85824102	1,23263687	0,29492012	0,59550629	POLD2	DNA polymerase delta 2, accessory subunit [Source:HGNC Symbol;Acc:HGNC:9176]
ENSG00000235315	0,71066068	-1,8919224	1,23276961	0,29489566	0,59550629	RPL23AP69	ribosomal protein L23a pseudogene 69 [Source:HGNC Symbol;Acc:HGNC:36625]
ENSG00000104388	0,22217105	5,44985768	1,23191214	0,29505367	0,59552903	RAB2A	RAB2A, member RAS oncogene family [Source:HGNC Symbol;Acc:HGNC:9763]
ENSG00000165215	0,6685776	-0,7197494	1,23210624	0,29501789	0,59552903	CLDN3	claudin 3 [Source:HGNC Symbol;Acc:HGNC:2045]
ENSG00000259248	-0,7053911	-0,3398904	1,23225109	0,2949912	0,59552903	USP3-AS1	USP3 antisense RNA 1 [Source:HGNC Symbol;Acc:HGNC:44140]
ENSG00000215003	-0,5451482	-1,4761263	1,23158062	0,29511479	0,59557011	RPL15P20	ribosomal protein L15 pseudogene 20 [Source:HGNC Symbol;Acc:HGNC:36130]
ENSG00000218208	-0,3751577	-0,2472149	1,23100992	0,29522006	0,59570025	RPS27AP11	RPS27A pseudogene 11 [Source:HGNC Symbol;Acc:HGNC:36126]
ENSG00000005513	-0,6981889	-1,6605217	1,23061516	0,2952929	0,59576494	SOX8	SRY-box transcription factor 8 [Source:HGNC Symbol;Acc:HGNC:11203]
ENSG00000003096	0,44912429	-0,8942622	1,22881059	0,29562621	0,59600982	KLHL13	kelch like family member 13 [Source:HGNC Symbol;Acc:HGNC:22931]
ENSG00000113328	-0,1867272	6,52709002	1,22822886	0,29573378	0,59600982	CCNG1	cyclin G1 [Source:HGNC Symbol;Acc:HGNC:1592]
ENSG00000116017	0,17898948	7,21540458	1,22820314	0,29573853	0,59600982	ARID3A	AT-rich interaction domain 3A [Source:HGNC Symbol;Acc:HGNC:3031]
ENSG00000140563	0,25750145	1,71925754	1,22798341	0,29577918	0,59600982	MCTP2	multiple C2 and transmembrane domain containing 2 [Source:HGNC Symbol;Acc:HGNC:25636]
ENSG00000145012	-0,5132115	4,17003278	1,22775063	0,29582224	0,59600982	LPP	LIM domain containing preferred translocation partner in lipoma [Source:HGNC Symbol;Acc:HGNC:6679]
ENSG00000150687	-0,5495394	-1,8288997	1,22867474	0,29565133	0,59600982	PRSS23	serine protease 23 [Source:HGNC Symbol;Acc:HGNC:14370]
ENSG00000162244	-0,1744626	9,70983295	1,22933847	0,29552865	0,59600982	RPL29	ribosomal protein L29 [Source:HGNC Symbol;Acc:HGNC:10331]
ENSG00000186352	-0,2701808	1,62682769	1,22895284	0,29559992	0,59600982	ANKRD37	ankyrin repeat domain 37 [Source:HGNC Symbol;Acc:HGNC:29593]
ENSG00000241973	0,21223763	5,62102036	1,22929903	0,29553594	0,59600982	PI4KA	phosphatidylinositol 4-kinase alpha [Source:HGNC Symbol;Acc:HGNC:8983]
ENSG00000275055	0,35393893	-0,0872123	1,22784759	0,2958043	0,59600982	AC011468.5	novel transcript, antisense to ZNF432
ENSG00000131732	-0,1494937	5,65482354	1,22703804	0,29595414	0,59602895	ZCCHC9	zinc finger CCHC-type containing 9 [Source:HGNC Symbol;Acc:HGNC:25424]
ENSG00000145908	-0,2858931	1,21752685	1,22706347	0,29594943	0,59602895	ZNF300	zinc finger protein 300 [Source:HGNC Symbol;Acc:HGNC:13091]
ENSG00000171862	0,21882822	3,82043048	1,22712532	0,29593798	0,59602895	PTEN	phosphatase and tensin homolog [Source:HGNC Symbol;Acc:HGNC:9588]
ENSG00000124006	-0,1944288	6,82375558	1,22424339	0,29647221	0,59699001	OBSL1	obscurin like cytoskeletal adaptor 1 [Source:HGNC Symbol;Acc:HGNC:29092]
ENSG00000106638	-0,1873037	6,55744134	1,22395926	0,29652496	0,59701394	TBL2	transducin beta like 2 [Source:HGNC Symbol;Acc:HGNC:11586]
ENSG00000282978	0,20453692	1,74499474	1,22343542	0,29662224	0,5971275	AC110994.2	basic transcription factor 3 (BTF3) pseudogene
ENSG00000101850	0,47362255	-1,0939449	1,22225699	0,29684124	0,59748605	GPR143	G protein-coupled receptor 143 [Source:HGNC Symbol;Acc:HGNC:20145]
ENSG00000231312	-0,2184951	2,42698965	1,22167743	0,29694904	0,59762068	MAP4K3-DT	MAP4K3 divergent transcript [Source:HGNC Symbol;Acc:HGNC:54056]

ENSG00000178053	0,37512303	-0,0843903	1,22096605	0,29708143	0,59780476	MLF1	myeloid leukemia factor 1 [Source:HGNC Symbol;Acc:HGNC:7125]
ENSG00000137818	-0,1884277	10,5110542	1,21894212	0,29745855	0,59848119	RPLP1	ribosomal protein lateral stalk subunit P1 [Source:HGNC Symbol;Acc:HGNC:10372]
ENSG00000145349	-0,185125	4,70421518	1,21846059	0,29754837	0,59857948	CAMK2D	calcium/calmodulin dependent protein kinase II delta [Source:HGNC Symbol;Acc:HGNC:1462]
ENSG00000030582	0,23695002	8,43663239	1,21351408	0,29847335	0,59880372	GRN	granulin precursor [Source:HGNC Symbol;Acc:HGNC:4601]
ENSG00000080823	0,2342034	2,53445386	1,21598309	0,29801114	0,59880372	MOK	MOK protein kinase [Source:HGNC Symbol;Acc:HGNC:9833]
ENSG00000088808	0,21645714	3,75596526	1,21391783	0,2983977	0,59880372	PPP1R13B	protein phosphatase 1 regulatory subunit 13B [Source:HGNC Symbol;Acc:HGNC:14950]
ENSG00000100084	0,18220162	3,71556703	1,21378587	0,29842242	0,59880372	HIRA	histone cell cycle regulator [Source:HGNC Symbol;Acc:HGNC:4916]
ENSG00000101126	0,15151777	5,3671446	1,21284954	0,29859794	0,59880372	ADNP	activity dependent neuroprotector homeobox [Source:HGNC Symbol;Acc:HGNC:15766]
ENSG00000102967	0,15266255	5,51928863	1,21712914	0,29779694	0,59880372	DHODH	dihydroorotate dehydrogenase (quinone) [Source:HGNC Symbol;Acc:HGNC:2867]
ENSG00000117523	0,51150424	6,37545584	1,21539622	0,29812091	0,59880372	PRRC2C	proline rich coiled-coil 2C [Source:HGNC Symbol;Acc:HGNC:24903]
ENSG00000132024	0,23882538	4,51665562	1,21135689	0,29887804	0,59880372	CC2D1A	coiled-coil and C2 domain containing 1A [Source:HGNC Symbol;Acc:HGNC:30237]
ENSG00000135365	-0,2546173	1,36074591	1,21339689	0,29849532	0,59880372	PHF21A	PHD finger protein 21A [Source:HGNC Symbol;Acc:HGNC:24156]
ENSG00000170827	0,55540537	-1,517395	1,21393983	0,29839358	0,59880372	CELP	carboxyl ester lipase pseudogene [Source:HGNC Symbol;Acc:HGNC:1849]
ENSG00000176402	0,6222684	-1,2687856	1,21486971	0,29821945	0,59880372	GJC3	gap junction protein gamma 3 [Source:HGNC Symbol;Acc:HGNC:17495]
ENSG00000177082	0,15539054	4,63246556	1,21129584	0,2988895	0,59880372	WDR73	WD repeat domain 73 [Source:HGNC Symbol;Acc:HGNC:25928]
ENSG00000182628	-0,185336	5,38397259	1,21555155	0,29809185	0,59880372	SKA2	spindle and kinetochore associated complex subunit 2 [Source:HGNC Symbol;Acc:HGNC:28006]
ENSG00000185090	0,16721446	6,16147438	1,21210905	0,29873684	0,59880372	MANEAL	mannosidase endo-alpha like [Source:HGNC Symbol;Acc:HGNC:26452]
ENSG00000197358	-0,4070699	-0,6450304	1,21478059	0,29823613	0,59880372	BNIP3P1	BCL2 interacting protein 3 pseudogene 1 [Source:HGNC Symbol;Acc:HGNC:19922]
ENSG00000197808	0,5850628	-0,038229	1,21132695	0,29888366	0,59880372	ZNF461	zinc finger protein 461 [Source:HGNC Symbol;Acc:HGNC:21629]
ENSG00000198108	-0,639041	-1,0687092	1,21248175	0,29866692	0,59880372	CHSY3	chondroitin sulfate synthase 3 [Source:HGNC Symbol;Acc:HGNC:24293]
ENSG00000198203	0,16646513	3,2408766	1,21184056	0,29878723	0,59880372	SULT1C2	sulfotransferase family 1C member 2 [Source:HGNC Symbol;Acc:HGNC:11456]
ENSG00000198825	0,199595	3,92472551	1,21305684	0,29855907	0,59880372	INPP5F	inositol polyphosphate-5-phosphatase F [Source:HGNC Symbol;Acc:HGNC:17054]
ENSG00000224186	-0,487335	-0,4796641	1,21740649	0,29774514	0,59880372	C5orf66	chromosome 5 open reading frame 66 [Source:HGNC Symbol;Acc:HGNC:48332]
ENSG00000227682	-0,5420691	-1,869591	1,21615311	0,29797935	0,59880372	ATP5F1AP2	ATP synthase F1 subunit alpha pseudogene 2 [Source:HGNC Symbol;Acc:HGNC:828]
ENSG00000231747	-0,2762584	0,73787679	1,21281025	0,2986053	0,59880372	AC079922.1	ribosomal protein S20 (RPS20) pseudogene
ENSG00000233380	-0,2677679	0,67238048	1,21706864	0,29780825	0,59880372	RPS2P48	ribosomal protein S2 pseudogene 48 [Source:HGNC Symbol;Acc:HGNC:35985]
ENSG00000235194	-0,2085529	2,13155844	1,21316704	0,2985384	0,59880372	PPP1R3E	protein phosphatase 1 regulatory subunit 3E [Source:HGNC Symbol;Acc:HGNC:14943]
ENSG00000237004	0,67277126	-1,6752196	1,21439959	0,29830746	0,59880372	ZNRF2P1	zinc and ring finger 2 pseudogene 1 [Source:HGNC Symbol;Acc:HGNC:42792]
ENSG00000242411	-0,5629169	-1,0643798	1,21683889	0,29785117	0,59880372	AC011506.1	ribosomal protein L13 (RPL13) pseudogene
ENSG00000255284	-0,2631454	0,9709078	1,21312882	0,29854557	0,59880372	AP006621.3	novel transcript
ENSG00000255581	0,55755103	-1,2089202	1,21186559	0,29878253	0,59880372	AC006511.2	heat shock 60kDa protein 1 (chaperonin) (HSPD1) pseudogene
ENSG00000256967	0,5353314	-1,4710585	1,2142703	0,29833168	0,59880372	AC018653.3	novel transcript
ENSG00000279342	-0,7670944	-2,0575491	1,21154163	0,29884335	0,59880372	AP000866.6	novel transcript, sense intronic to MSANTD2
ENSG00000033100	0,14556335	5,86539216	1,20829529	0,29945374	0,59919066	CHPF2	chondroitin polymerizing factor 2 [Source:HGNC Symbol;Acc:HGNC:29270]
ENSG00000054654	0,36326213	0,25634033	1,20766354	0,29957273	0,59919066	SYNE2	spectrin repeat containing nuclear envelope protein 2 [Source:HGNC Symbol;Acc:HGNC:17084]
ENSG00000076382	-0,1511455	5,38684666	1,20714035	0,29967132	0,59919066	SPAG5	sperm associated antigen 5 [Source:HGNC Symbol;Acc:HGNC:13452]
ENSG00000086730	0,49519277	-0,9786126	1,20713742	0,29967188	0,59919066	LAT2	linker for activation of T cells family member 2 [Source:HGNC Symbol;Acc:HGNC:12749]
ENSG00000100867	-0,2017852	11,095142	1,20833417	0,29944642	0,59919066	DHRS2	dehydrogenase/reductase 2 [Source:HGNC Symbol;Acc:HGNC:18349]
ENSG00000166847	0,14225246	7,28922424	1,20773299	0,29955964	0,59919066	DCTN5	dynactin subunit 5 [Source:HGNC Symbol;Acc:HGNC:24594]
ENSG00000168944	-0,1919948	3,33167098	1,20699956	0,29969787	0,59919066	CEP120	centrosomal protein 120 [Source:HGNC Symbol;Acc:HGNC:26690]
ENSG00000180376	-0,1865328	2,84793628	1,20724511	0,29965158	0,59919066	CCDC66	coiled-coil domain containing 66 [Source:HGNC Symbol;Acc:HGNC:27709]
ENSG00000196150	-0,2284808	2,35913669	1,20975934	0,29917824	0,59919066	ZNF250	zinc finger protein 250 [Source:HGNC Symbol;Acc:HGNC:13044]
ENSG00000233016	-0,1863537	5,36805112	1,20740856	0,29962077	0,59919066	SNHG7	small nucleolar RNA host gene 7 [Source:HGNC Symbol;Acc:HGNC:28254]
ENSG00000236384	0,63820617	-1,7215735	1,20855735	0,2994044	0,59919066	LINC00479	long intergenic non-protein coding RNA 479 [Source:HGNC Symbol;Acc:HGNC:19727]

ENSG00000243302	0,67776033	-0,0573054	1,20866884	0,29938341	0,59919066	AC018638.4	novel pseudogene
ENSG00000248015	0,30182668	1,34114689	1,20862195	0,29939224	0,59919066	AC005329.1	novel transcript, antisense to NDUFS7
ENSG00000250462	0,31925696	0,74443967	1,20922609	0,29927854	0,59919066	LRRC37BP1	leucine rich repeat containing 37B pseudogene 1 [Source:HGNC Symbol;Acc:HGNC:25390]
ENSG00000276791	-0,5198386	-0,5782784	1,20851443	0,29941248	0,59919066	AC092117.1	novel transcript
ENSG00000158669	0,15354528	6,23454009	1,20593229	0,29989916	0,59942904	GPAT4	glycerol-3-phosphate acyltransferase 4 [Source:HGNC Symbol;Acc:HGNC:20880]
ENSG00000183597	-0,1763899	3,09889534	1,20608839	0,29986971	0,59942904	TANGO2	transport and golgi organization 2 homolog [Source:HGNC Symbol;Acc:HGNC:25439]
ENSG00000146433	0,17271146	6,08489536	1,2051939	0,30003855	0,5996256	TMEM181	transmembrane protein 181 [Source:HGNC Symbol;Acc:HGNC:20958]
ENSG00000177706	-0,2089649	4,76439174	1,20475875	0,30012073	0,59970781	FAM20C	FAM20C golgi associated secretory pathway kinase [Source:HGNC Symbol;Acc:HGNC:22140]
ENSG00000228801	-0,2791578	0,93732053	1,20434905	0,30019814	0,59978044	AC064807.1	novel transcript
ENSG00000263956	0,28614103	1,17381707	1,20340506	0,3003766	0,60000434	NBPF11	NBPF member 11 [Source:HGNC Symbol;Acc:HGNC:31993]
ENSG00000273329	-0,7187754	-0,7456875	1,20332183	0,30039235	0,60000434	AC078846.1	novel transcript
ENSG00000118200	0,20397816	2,38151154	1,20287208	0,30047743	0,60009225	CAMSAP2	calmodulin regulated spectrin associated protein family member 2 [Source:HGNC Symbol;Acc:HGNC:29188]
ENSG00000154642	0,23375853	2,15022063	1,20214491	0,30061508	0,60028508	C21orf91	chromosome 21 open reading frame 91 [Source:HGNC Symbol;Acc:HGNC:16459]
ENSG00000102384	-0,2011087	2,83960612	1,20187145	0,30066686	0,60030644	CENPI	centromere protein I [Source:HGNC Symbol;Acc:HGNC:3968]
ENSG00000177807	-0,3668085	1,16440729	1,20128782	0,30077743	0,60044513	KCNJ10	potassium inwardly rectifying channel subfamily J member 10 [Source:HGNC Symbol;Acc:HGNC:6256]
ENSG00000136897	0,16618876	7,26340977	1,20004151	0,30101374	0,60083477	MRPL50	mitochondrial ribosomal protein L50 [Source:HGNC Symbol;Acc:HGNC:16654]
ENSG00000142871	-0,376033	6,76109662	1,19936521	0,30114208	0,60092673	CCN1	cellular communication network factor 1 [Source:HGNC Symbol;Acc:HGNC:2654]
ENSG00000157693	0,20497939	2,13345711	1,19944449	0,30112703	0,60092673	TMEM268	transmembrane protein 268 [Source:HGNC Symbol;Acc:HGNC:24513]
ENSG00000134709	0,1995494	3,78012953	1,19890368	0,30122971	0,6010195	HOOK1	hook microtubule tethering protein 1 [Source:HGNC Symbol;Acc:HGNC:19884]
ENSG00000124523	-0,2011276	4,17551737	1,19788565	0,30142313	0,60114861	SIRT5	sirtuin 5 [Source:HGNC Symbol;Acc:HGNC:14933]
ENSG00000138075	-0,164561	3,44416651	1,19730086	0,30153432	0,60114861	ABCG5	ATP binding cassette subfamily G member 5 [Source:HGNC Symbol;Acc:HGNC:13886]
ENSG00000163166	-0,1854917	6,74644458	1,19831438	0,30134165	0,60114861	IWS1	interacts with SUPT6H, CTD assembly factor 1 [Source:HGNC Symbol;Acc:HGNC:25467]
ENSG00000166411	0,19584014	5,1044072	1,19777333	0,30144448	0,60114861	IDH3A	isocitrate dehydrogenase (NAD(+)) 3 catalytic subunit alpha [Source:HGNC Symbol;Acc:HGNC:5384]
ENSG00000177479	-0,1616499	6,31286693	1,19723027	0,30154774	0,60114861	ARIH2	ariadne RBR E3 ubiquitin protein ligase 2 [Source:HGNC Symbol;Acc:HGNC:690]
ENSG00000204516	0,19193125	5,33386065	1,19757181	0,30148279	0,60114861	MICB	MHC class I polypeptide-related sequence B [Source:HGNC Symbol;Acc:HGNC:7091]
ENSG00000264070	-0,4328637	-0,4630268	1,19704775	0,30158246	0,60114861	DND1P1	DND microRNA-mediated repression inhibitor 1 pseudogene 1 [Source:HGNC Symbol;Acc:HGNC:31444]
ENSG00000133424	0,16342174	4,42646802	1,19646129	0,30169405	0,60124013	LARGE1	LARGE xylosyl- and glucuronyltransferase 1 [Source:HGNC Symbol;Acc:HGNC:6511]
ENSG00000250144	-0,2257847	3,82314454	1,19637389	0,30171069	0,60124013	AC104619.3	eukaryotic translation elongation factor 1 alpha 1 (EEF1A1) pseudogene
ENSG00000110921	0,14171297	7,30541804	1,19565346	0,30184786	0,60126744	MVK	mevalonate kinase [Source:HGNC Symbol;Acc:HGNC:7530]
ENSG00000123737	-0,1720704	5,04759623	1,19577164	0,30182535	0,60126744	EXOSC9	exosome component 9 [Source:HGNC Symbol;Acc:HGNC:9137]
ENSG00000204267	0,18987935	3,2087029	1,19605164	0,30177203	0,60126744	TAP2	transporter 2, ATP binding cassette subfamily B member [Source:HGNC Symbol;Acc:HGNC:44]
ENSG00000213757	-0,1834607	2,32159646	1,19513653	0,30194634	0,60138161	RPS3AP20	RPS3A pseudogene 20 [Source:HGNC Symbol;Acc:HGNC:36141]
ENSG00000090581	-0,1822752	6,03425282	1,19406316	0,30215098	0,60170716	GNPTG	N-acetylglucosamine-1-phosphate transferase subunit gamma [Source:HGNC Symbol;Acc:HGNC:23026]
ENSG00000215154	-0,2514517	1,64417415	1,19374837	0,30221104	0,60174473	AC141586.1	potassium channel tetramerisation domain containing 5 (KCTD5) pseudogene
ENSG00000164305	0,16386361	6,41640604	1,19234127	0,30247968	0,6021155	CASP3	caspase 3 [Source:HGNC Symbol;Acc:HGNC:1504]
ENSG00000172331	-0,2214737	2,1482367	1,19249391	0,30245052	0,6021155	BPGM	bisphosphoglycerate mutase [Source:HGNC Symbol;Acc:HGNC:1093]
ENSG00000167965	-0,1697002	5,76475377	1,19084402	0,30276592	0,60260317	MLST8	MTOR associated protein, LST8 homolog [Source:HGNC Symbol;Acc:HGNC:24825]
ENSG00000184831	-0,1650076	4,47257564	1,19061638	0,30280947	0,60260776	APOO	apolipoprotein O [Source:HGNC Symbol;Acc:HGNC:28727]
ENSG00000143353	-0,1868769	2,61425779	1,19021677	0,30288595	0,60267786	LYPLAL1	lysophospholipase like 1 [Source:HGNC Symbol;Acc:HGNC:20440]
ENSG00000108395	0,18785354	4,50728266	1,18788965	0,30333186	0,60301678	TRIM37	tripartite motif containing 37 [Source:HGNC Symbol;Acc:HGNC:7523]
ENSG00000116161	0,15157802	7,74248991	1,18742777	0,30342048	0,60301678	CACYBP	calcyclin binding protein [Source:HGNC Symbol;Acc:HGNC:30423]
ENSG00000131389	-0,3100532	4,37379867	1,1884628	0,30322195	0,60301678	SLC6A6	solute carrier family 6 member 6 [Source:HGNC Symbol;Acc:HGNC:11052]
ENSG00000158186	-0,189512	3,63831418	1,18751045	0,30340461	0,60301678	MRAS	muscle RAS oncogene homolog [Source:HGNC Symbol;Acc:HGNC:7227]
ENSG00000159111	-0,1519221	7,43041649	1,18800623	0,3033095	0,60301678	MRPL10	mitochondrial ribosomal protein L10 [Source:HGNC Symbol;Acc:HGNC:14055]

ENSG00000162408	0,22284838	4,87029162	1,18763855	0,30338004	0,60301678	NOL9	nucleolar protein 9 [Source:HGNC Symbol;Acc:HGNC:26265]
ENSG00000164610	-0,1660774	3,52134744	1,18881403	0,30315462	0,60301678	RP9	RP9 pre-mRNA splicing factor [Source:HGNC Symbol;Acc:HGNC:10288]
ENSG00000170222	0,22145263	2,23827942	1,18805109	0,3033009	0,60301678	ADPRM	ADP-ribose/CDP-alcohol diphosphatase, manganese dependent [Source:HGNC Symbol;Acc:HGNC:30925]
ENSG00000177595	-0,165117	4,63062567	1,18738976	0,30342777	0,60301678	PIDD1	p53-induced death domain protein 1 [Source:HGNC Symbol;Acc:HGNC:16491]
ENSG00000204160	0,15306708	4,9973425	1,18715887	0,30347209	0,60302282	ZDHHC18	zinc finger DHHC-type palmitoyltransferase 18 [Source:HGNC Symbol;Acc:HGNC:20712]
ENSG00000224837	-0,4115459	-0,4958045	1,1864519	0,30360783	0,6032105	GCSHP5	glycine cleavage system protein H pseudogene 5 [Source:HGNC Symbol;Acc:HGNC:44195]
ENSG00000099999	-0,2201006	2,22317818	1,1860064	0,30369342	0,60323425	RNF215	ring finger protein 215 [Source:HGNC Symbol;Acc:HGNC:33434]
ENSG00000188827	0,30981932	2,29647081	1,18595983	0,30370237	0,60323425	SLX4	SLX4 structure-specific endonuclease subunit [Source:HGNC Symbol;Acc:HGNC:23845]
ENSG00000134852	0,20176462	2,24738196	1,18555787	0,30377962	0,60325781	CLOCK	clock circadian regulator [Source:HGNC Symbol;Acc:HGNC:2082]
ENSG00000198691	-0,5692879	-1,2135721	1,18546842	0,30379682	0,60325781	ABCA4	ATP binding cassette subfamily A member 4 [Source:HGNC Symbol;Acc:HGNC:34]
ENSG00000183010	-0,1523772	8,51542333	1,185169	0,30385439	0,60329012	PYCR1	pyrroline-5-carboxylate reductase 1 [Source:HGNC Symbol;Acc:HGNC:9721]
ENSG00000171530	-0,1590271	7,07994095	1,18435885	0,30401023	0,60351753	TBCA	tubulin folding cofactor A [Source:HGNC Symbol;Acc:HGNC:11579]
ENSG00000185324	-0,4402305	0,5560577	1,18328874	0,30421627	0,60376245	CDK10	cyclin dependent kinase 10 [Source:HGNC Symbol;Acc:HGNC:1770]
ENSG00000234518	0,32997753	0,45573613	1,18339233	0,30419631	0,60376245	PTGES3P1	prostaglandin E synthase 3 pseudogene 1 [Source:HGNC Symbol;Acc:HGNC:43824]
ENSG00000148303	-0,1562567	11,6773872	1,18225736	0,30441503	0,60407487	RPL7A	ribosomal protein L7a [Source:HGNC Symbol;Acc:HGNC:10364]
ENSG00000177380	-0,2116137	3,33539328	1,1820425	0,30445646	0,60407503	PPFIA3	PTPRF interacting protein alpha 3 [Source:HGNC Symbol;Acc:HGNC:9247]
ENSG00000014641	0,15565869	8,50155724	1,17889239	0,30506483	0,60422165	MDH1	malate dehydrogenase 1 [Source:HGNC Symbol;Acc:HGNC:6970]
ENSG00000115459	-0,2555434	2,5371524	1,17962602	0,30492299	0,60422165	ELMOD3	ELMO domain containing 3 [Source:HGNC Symbol;Acc:HGNC:26158]
ENSG00000121988	-0,1698166	3,88169407	1,18120577	0,30461788	0,60422165	ZRANB3	zinc finger RANBP2-type containing 3 [Source:HGNC Symbol;Acc:HGNC:25249]
ENSG00000161970	-0,1790802	7,50310995	1,17925289	0,30499512	0,60422165	RPL26	ribosomal protein L26 [Source:HGNC Symbol;Acc:HGNC:10327]
ENSG00000166181	0,15783022	5,99424836	1,17963099	0,30492203	0,60422165	API5	apoptosis inhibitor 5 [Source:HGNC Symbol;Acc:HGNC:594]
ENSG00000167971	0,391469	-0,363016	1,18054889	0,3047447	0,60422165	CASKIN1	CASK interacting protein 1 [Source:HGNC Symbol;Acc:HGNC:20879]
ENSG00000181191	-0,1989498	3,0986117	1,17994172	0,30486198	0,60422165	PJA1	praja ring finger ubiquitin ligase 1 [Source:HGNC Symbol;Acc:HGNC:16648]
ENSG00000182004	-0,161372	6,79003717	1,17908238	0,30502809	0,60422165	SNRPE	small nuclear ribonucleoprotein polypeptide E [Source:HGNC Symbol;Acc:HGNC:11161]
ENSG00000205659	0,17211223	3,98117279	1,17887582	0,30506803	0,60422165	LIN52	lin-52 DREAM MuvB core complex component [Source:HGNC Symbol;Acc:HGNC:19856]
ENSG00000234751	-0,5491334	-1,8308794	1,18028845	0,304795	0,60422165	AP002381.1	ribosomal protein L15 (RPL15) pseudogene
ENSG00000238000	0,16669669	3,93043028	1,17891698	0,30506007	0,60422165	PSME2P1	proteasome activator subunit 2 pseudogene 1 [Source:HGNC Symbol;Acc:HGNC:20632]
ENSG00000249378	0,70756282	-1,1253493	1,1791662	0,30501188	0,60422165	LINC01060	long intergenic non-protein coding RNA 1060 [Source:HGNC Symbol;Acc:HGNC:49081]
ENSG00000273297	-0,3558197	-0,263	1,17926863	0,30499207	0,60422165	AC009275.1	novel transcript
ENSG00000153827	0,19620025	6,54786855	1,17846649	0,30514721	0,60429655	TRIP12	thyroid hormone receptor interactor 12 [Source:HGNC Symbol;Acc:HGNC:12306]
ENSG00000113356	-0,2330993	2,119777	1,17815914	0,30520669	0,60433241	POLR3G	RNA polymerase III subunit G [Source:HGNC Symbol;Acc:HGNC:30075]
ENSG00000214389	-0,1736307	3,45023759	1,17793358	0,30525035	0,60433695	RPS3AP26	RPS3A pseudogene 26 [Source:HGNC Symbol;Acc:HGNC:36513]
ENSG00000171159	-0,1976967	4,1691109	1,17771145	0,30529335	0,60434018	C9orf16	chromosome 9 open reading frame 16 [Source:HGNC Symbol;Acc:HGNC:17823]
ENSG00000213654	0,41640001	0,34905892	1,17721142	0,30539018	0,60444996	GPSM3	G protein signaling modulator 3 [Source:HGNC Symbol;Acc:HGNC:13945]
ENSG00000149131	0,31571806	2,25532828	1,17661855	0,30550505	0,60459541	SERPING1	serpin family G member 1 [Source:HGNC Symbol;Acc:HGNC:1228]
ENSG00000109220	0,21814371	1,97046505	1,17628004	0,30557067	0,60464336	CHIC2	cysteine rich hydrophobic domain 2 [Source:HGNC Symbol;Acc:HGNC:1935]
ENSG00000068383	0,18734406	4,06152417	1,17492273	0,30583397	0,604909	INPP5A	inositol polyphosphate-5-phosphatase A [Source:HGNC Symbol;Acc:HGNC:6076]
ENSG00000075945	-0,2152686	2,7493821	1,1753627	0,30574859	0,604909	KIFAP3	kinesin associated protein 3 [Source:HGNC Symbol;Acc:HGNC:17060]
ENSG00000164916	0,54619084	4,70049143	1,17473435	0,30587054	0,604909	FOXK1	forkhead box K1 [Source:HGNC Symbol;Acc:HGNC:23480]
ENSG00000213305	-0,437333	-0,5855116	1,17483435	0,30585113	0,604909	HNRNPCP6	heterogeneous nuclear ribonucleoprotein C pseudogene 6 [Source:HGNC Symbol;Acc:HGNC:48817]
ENSG00000179532	-0,4336421	-0,1862659	1,17411909	0,30599002	0,60506338	DNHD1	dynein heavy chain domain 1 [Source:HGNC Symbol;Acc:HGNC:26532]
ENSG00000214269	-0,4524005	-1,043262	1,17390507	0,3060316	0,6050637	LGMNP1	legumain pseudogene 1 [Source:HGNC Symbol;Acc:HGNC:23079]
ENSG00000075234	-0,150567	6,41607879	1,17335007	0,30613946	0,6051539	TTC38	tetratricopeptide repeat domain 38 [Source:HGNC Symbol;Acc:HGNC:26082]
ENSG00000083544	-0,1946202	3,00236958	1,17324404	0,30616007	0,6051539	TDRD3	tudor domain containing 3 [Source:HGNC Symbol;Acc:HGNC:20612]

ENSG00000005893	0,21683609	7,27427108	1,17198133	0,30640569	0,60524179	LAMP2	lysosomal associated membrane protein 2 [Source:HGNC Symbol;Acc:HGNC:6501]
ENSG00000134058	0,14846174	6,0558186	1,17190719	0,30642012	0,60524179	CDK7	cyclin dependent kinase 7 [Source:HGNC Symbol;Acc:HGNC:1778]
ENSG00000179218	-0,2117712	11,6136624	1,17162882	0,30647432	0,60524179	CALR	calreticulin [Source:HGNC Symbol;Acc:HGNC:1455]
ENSG00000197728	-0,1737602	8,42574743	1,1721216	0,30637839	0,60524179	RPS26	ribosomal protein S26 [Source:HGNC Symbol;Acc:HGNC:10414]
ENSG00000205559	-0,4991205	-0,4531182	1,17174691	0,30645132	0,60524179	CHKB-DT	CHKB divergent transcript [Source:HGNC Symbol;Acc:HGNC:40146]
ENSG00000214655	0,25420082	4,52406364	1,1719995	0,30640216	0,60524179	ZSWIM8	zinc finger SWIM-type containing 8 [Source:HGNC Symbol;Acc:HGNC:23528]
ENSG00000241282	0,64279504	-2,0490807	1,17152492	0,30649454	0,60524179	RPL34P33	ribosomal protein L34 pseudogene 33 [Source:HGNC Symbol;Acc:HGNC:36666]
ENSG00000115919	-0,158852	5,56458655	1,17074254	0,30664694	0,60527582	KYNU	kynureninase [Source:HGNC Symbol;Acc:HGNC:6469]
ENSG00000136802	-0,1551711	5,59251367	1,17069075	0,30665703	0,60527582	LRRC8A	leucine rich repeat containing 8 VRAC subunit A [Source:HGNC Symbol;Acc:HGNC:19027]
ENSG00000231856	0,46158027	-1,1505658	1,1705857	0,3066775	0,60527582	AL162377.1	novel transcript
ENSG00000261058	-0,6256407	-2,0353664	1,17089985	0,30661629	0,60527582	AC099508.2	novel transcript
ENSG00000109606	0,19863798	6,85268056	1,16942126	0,30690455	0,60564212	DHX15	DEAH-box helicase 15 [Source:HGNC Symbol;Acc:HGNC:2738]
ENSG00000143870	-0,1901943	11,1609633	1,16914176	0,30695909	0,60566793	PDIA6	protein disulfide isomerase family A member 6 [Source:HGNC Symbol;Acc:HGNC:30168]
ENSG00000113761	-0,2158874	3,6877222	1,1687854	0,30702864	0,60570113	ZNF346	zinc finger protein 346 [Source:HGNC Symbol;Acc:HGNC:16403]
ENSG00000180198	-0,1465415	7,47388297	1,16863072	0,30705884	0,60570113	RCC1	regulator of chromosome condensation 1 [Source:HGNC Symbol;Acc:HGNC:1913]
ENSG00000145741	0,1399385	10,1839612	1,16782869	0,30721548	0,6059283	BTF3	basic transcription factor 3 [Source:HGNC Symbol;Acc:HGNC:1125]
ENSG00000273148	0,5238842	-0,244868	1,16752013	0,30727577	0,60596541	LINC00653	long intergenic non-protein coding RNA 653 [Source:HGNC Symbol;Acc:HGNC:44308]
ENSG00000171169	-0,1783837	2,72395949	1,16701677	0,30737417	0,60607764	NAIF1	nuclear apoptosis inducing factor 1 [Source:HGNC Symbol;Acc:HGNC:25446]
ENSG00000142677	0,15816921	4,08094709	1,16598537	0,30757593	0,6063118	IL22RA1	interleukin 22 receptor subunit alpha 1 [Source:HGNC Symbol;Acc:HGNC:13700]
ENSG00000170917	-0,2107113	2,08260788	1,1661642	0,30754093	0,6063118	NUDT6	nudix hydrolase 6 [Source:HGNC Symbol;Acc:HGNC:8053]
ENSG00000125430	0,15145119	4,71459841	1,16537277	0,30769585	0,60646637	HS3ST3B1	heparan sulfate-glucosamine 3-sulfotransferase 3B1 [Source:HGNC Symbol;Acc:HGNC:5198]
ENSG00000160284	-0,1941899	3,21953581	1,16480635	0,3078068	0,6066032	SPATC1L	spermatogenesis and centriole associated 1 like [Source:HGNC Symbol;Acc:HGNC:1298]
ENSG00000105357	-0,3392269	3,29805588	1,1637263	0,30801851	0,60687256	MYH14	myosin heavy chain 14 [Source:HGNC Symbol;Acc:HGNC:23212]
ENSG00000233558	-0,2294489	1,98518408	1,16368524	0,30802656	0,60687256	AL050331.1	RPS5 (40S Ribosomal Protein S5) pseudogene
ENSG00000130731	-0,1856909	5,7238221	1,16326127	0,30810973	0,60695456	METTL26	methyltransferase like 26 [Source:HGNC Symbol;Acc:HGNC:14141]
ENSG00000179562	0,17170259	4,60098563	1,16283241	0,30819389	0,60703849	GCC1	GRIP and coiled-coil domain containing 1 [Source:HGNC Symbol;Acc:HGNC:19095]
ENSG00000008130	-0,1546319	5,75314947	1,16108903	0,30853635	0,60706752	NADK	NAD kinase [Source:HGNC Symbol;Acc:HGNC:29831]
ENSG00000047621	0,19228752	3,18083418	1,15987417	0,30877532	0,60706752	C12orf4	chromosome 12 open reading frame 4 [Source:HGNC Symbol;Acc:HGNC:1184]
ENSG00000051596	0,16074514	4,66654823	1,15977975	0,3087939	0,60706752	THOC3	THO complex 3 [Source:HGNC Symbol;Acc:HGNC:19072]
ENSG00000112308	-0,1690221	3,9965102	1,16132001	0,30849095	0,60706752	C6orf62	chromosome 6 open reading frame 62 [Source:HGNC Symbol;Acc:HGNC:20998]
ENSG00000150433	-0,1931221	2,83751458	1,16040562	0,30867075	0,60706752	TMEM218	transmembrane protein 218 [Source:HGNC Symbol;Acc:HGNC:27344]
ENSG00000165280	-0,2495023	8,96563602	1,16119187	0,30851614	0,60706752	VCP	valosin containing protein [Source:HGNC Symbol;Acc:HGNC:12666]
ENSG00000171865	0,19907788	3,38251153	1,16107928	0,30853827	0,60706752	RNASEH1	ribonuclease H1 [Source:HGNC Symbol;Acc:HGNC:18466]
ENSG00000230510	-0,6278217	-1,7776199	1,16050983	0,30865025	0,60706752	PPP5D1	PPP5 tetratricopeptide repeat domain containing 1 [Source:HGNC Symbol;Acc:HGNC:44209]
ENSG00000237753	-0,6491567	-1,4662244	1,16152728	0,30845021	0,60706752	AC079922.2	novel transcript (FLJ42351)
ENSG00000240342	-0,1504614	7,44546294	1,16037433	0,30867691	0,60706752	RPS2P5	ribosomal protein S2 pseudogene 5 [Source:HGNC Symbol;Acc:HGNC:31386]
ENSG00000246283	-0,6140649	-1,4193481	1,16174484	0,30840746	0,60706752	AC090510.1	novel transcript
ENSG00000257122	0,24099212	1,85880756	1,1597514	0,30879949	0,60706752	RRN3P3	RRN3 pseudogene 3 [Source:HGNC Symbol;Acc:HGNC:37620]
ENSG00000265241	-0,1856685	6,90672815	1,1597101	0,30880762	0,60706752	RBM8A	RNA binding motif protein 8A [Source:HGNC Symbol;Acc:HGNC:9905]
ENSG00000272572	-0,8562612	-1,6927249	1,16004373	0,30874195	0,60706752	AL138762.1	novel transcript, antisense to FAM178A
ENSG00000273723	-0,6242595	-1,6319915	1,15958653	0,30883194	0,60706752	SUGT1-DT	SUGT1 divergent transcript [Source:HGNC Symbol;Acc:HGNC:54809]
ENSG00000169258	0,17081743	2,74193352	1,15893037	0,30896116	0,60723981	GPRIN1	G protein regulated inducer of neurite outgrowth 1 [Source:HGNC Symbol;Acc:HGNC:24835]
ENSG00000068323	-0,1757937	4,80318579	1,15787274	0,3091696	0,60726314	TFE3	transcription factor binding to IGHM enhancer 3 [Source:HGNC Symbol;Acc:HGNC:11752]
ENSG00000160051	-0,1937253	2,50994947	1,1578455	0,30917497	0,60726314	IQCC	IQ motif containing C [Source:HGNC Symbol;Acc:HGNC:25545]

ENSG00000162927	-0,1594835	4,45327577	1,1578156	0,30918087	0,60726314	PUS10	pseudouridine synthase 10 [Source:HGNC Symbol;Acc:HGNC:26505]
ENSG00000180902	-0,2637282	3,98779705	1,15846662	0,30905253	0,60726314	D2HGDH	D-2-hydroxyglutarate dehydrogenase [Source:HGNC Symbol;Acc:HGNC:28358]
ENSG00000276672	0,50173474	-1,400488	1,15827862	0,30908958	0,60726314	AL161891.1	novel transcript, sense intronic to RFC3
ENSG00000106003	-0,375914	1,22431648	1,15729898	0,30928276	0,60729998	LFNG	LFNG O-fucosylpeptide 3-beta-N-acetylglucosaminyltransferase [Source:HGNC Symbol;Acc:HGNC:6560]
ENSG00000196976	-0,1889225	3,33764274	1,15737127	0,3092685	0,60729998	LAGE3	L antigen family member 3 [Source:HGNC Symbol;Acc:HGNC:26058]
ENSG00000154479	0,80589484	-1,9862172	1,15703329	0,30933518	0,60732129	CCDC173	coiled-coil domain containing 173 [Source:HGNC Symbol;Acc:HGNC:25064]
ENSG00000065802	-0,1513509	5,60544953	1,15498802	0,30973917	0,60752262	ASB1	ankyrin repeat and SOCS box containing 1 [Source:HGNC Symbol;Acc:HGNC:16011]
ENSG00000119778	0,22243781	2,85473037	1,15535007	0,3096676	0,60752262	ATAD2B	ATPase family AAA domain containing 2B [Source:HGNC Symbol;Acc:HGNC:29230]
ENSG00000130726	0,1904339	8,90407216	1,15562672	0,30961293	0,60752262	TRIM28	tripartite motif containing 28 [Source:HGNC Symbol;Acc:HGNC:16384]
ENSG00000148688	0,15190574	5,49235196	1,1556934	0,30959975	0,60752262	RPP30	ribonuclease P/MRP subunit p30 [Source:HGNC Symbol;Acc:HGNC:17688]
ENSG00000177855	0,30871099	0,92927345	1,15555089	0,30962791	0,60752262	CACYBPP2	calcyclin binding protein pseudogene 2 [Source:HGNC Symbol;Acc:HGNC:45123]
ENSG00000182400	0,187491	3,86424475	1,15482998	0,30977041	0,60752262	TRAPPC6B	trafficking protein particle complex 6B [Source:HGNC Symbol;Acc:HGNC:23066]
ENSG00000189136	-0,7059191	-1,3042389	1,15614343	0,30951085	0,60752262	UBE2Q2P1	ubiquitin conjugating enzyme E2 Q2 pseudogene 1 [Source:HGNC Symbol;Acc:HGNC:37439]
ENSG00000204622	-0,3065646	1,39832293	1,15500595	0,30973562	0,60752262	HLA-J	major histocompatibility complex, class I, J (pseudogene) [Source:HGNC Symbol;Acc:HGNC:4967]
ENSG00000063046	0,18977124	8,60702157	1,15274916	0,31018226	0,60769886	EIF4B	eukaryotic translation initiation factor 4B [Source:HGNC Symbol;Acc:HGNC:3285]
ENSG00000103316	-0,3367763	0,22262083	1,15297289	0,31013794	0,60769886	CRYM	crystallin mu [Source:HGNC Symbol;Acc:HGNC:2418]
ENSG00000151914	0,308356	4,4635347	1,15248471	0,31023466	0,60769886	DST	dystonin [Source:HGNC Symbol;Acc:HGNC:1090]
ENSG00000206228	-0,2130658	1,63858143	1,15368727	0,30999649	0,60769886	HNRNPA1P4	heterogeneous nuclear ribonucleoprotein A1 pseudogene 4 [Source:HGNC Symbol;Acc:HGNC:32234]
ENSG00000214455	0,38393535	0,41063312	1,15287678	0,31015698	0,60769886	RCN1P2	reticulocalbin 1 pseudogene 2 [Source:HGNC Symbol;Acc:HGNC:39204]
ENSG00000234737	0,67495119	-0,5074537	1,15261689	0,31020847	0,60769886	KRT18P15	keratin 18 pseudogene 15 [Source:HGNC Symbol;Acc:HGNC:32449]
ENSG00000267500	0,47980965	-1,2264611	1,15276888	0,31017835	0,60769886	ZNF887P	zinc finger protein 887, pseudogene [Source:HGNC Symbol;Acc:HGNC:38700]
ENSG00000271870	0,58974885	-1,4115078	1,15320791	0,31009139	0,60769886	AC024060.2	novel transcript, antisense to CRBN
ENSG00000272333	0,30669441	4,41487902	1,15295836	0,31014082	0,60769886	KMT2B	lysine methyltransferase 2B [Source:HGNC Symbol;Acc:HGNC:15840]
ENSG00000111879	0,1821811	3,47539739	1,1512544	0,3104786	0,60776596	FAM184A	family with sequence similarity 184 member A [Source:HGNC Symbol;Acc:HGNC:20991]
ENSG00000136451	0,16123093	4,52882442	1,15119213	0,31049096	0,60776596	VEZF1	vascular endothelial zinc finger 1 [Source:HGNC Symbol;Acc:HGNC:12949]
ENSG00000137310	0,24309069	4,13785166	1,15021855	0,3106842	0,60776596	TCF19	transcription factor 19 [Source:HGNC Symbol;Acc:HGNC:11629]
ENSG00000139726	0,16411694	5,86018995	1,15181854	0,31036671	0,60776596	DENR	density regulated re-initiation and release factor [Source:HGNC Symbol;Acc:HGNC:2769]
ENSG00000155313	0,20406082	3,56754696	1,15087762	0,31055337	0,60776596	USP25	ubiquitin specific peptidase 25 [Source:HGNC Symbol;Acc:HGNC:12624]
ENSG00000158292	0,96054295	-2,0317955	1,15149431	0,31043101	0,60776596	GPR153	G protein-coupled receptor 153 [Source:HGNC Symbol;Acc:HGNC:23618]
ENSG00000166860	0,17593312	2,8524556	1,15075493	0,31057772	0,60776596	ZBTB39	zinc finger and BTB domain containing 39 [Source:HGNC Symbol;Acc:HGNC:29014]
ENSG00000177076	0,37962089	0,29689107	1,15074209	0,31058026	0,60776596	ACER2	alkaline ceramidase 2 [Source:HGNC Symbol;Acc:HGNC:23675]
ENSG00000228264	-0,5291245	-1,5281975	1,15021489	0,31068493	0,60776596	PSMD8P1	proteasome 26S subunit, non-ATPase, 8 pseudogene 1 [Source:HGNC Symbol;Acc:HGNC:34013]
ENSG00000244968	0,5600608	-0,9540764	1,15026017	0,31067594	0,60776596	LIFR-AS1	LIFR antisense RNA 1 [Source:HGNC Symbol;Acc:HGNC:43600]
ENSG00000152078	-0,3287829	1,61919892	1,14990107	0,31074726	0,60780649	TLCD4	TLC domain containing 4 [Source:HGNC Symbol;Acc:HGNC:26477]
ENSG00000153982	0,20599723	2,1362758	1,1494349	0,31083988	0,60787947	GDPD1	glycerophosphodiester phosphodiesterase domain containing 1 [Source:HGNC Symbol;Acc:HGNC:20883]
ENSG00000186432	0,16334763	5,66733103	1,14929447	0,31086779	0,60787947	KPNA4	karyopherin subunit alpha 4 [Source:HGNC Symbol;Acc:HGNC:6397]
ENSG00000109189	0,25299098	2,86508001	1,14608026	0,31150757	0,6087909	USP46	ubiquitin specific peptidase 46 [Source:HGNC Symbol;Acc:HGNC:20075]
ENSG00000146063	-0,1654178	3,58973572	1,14486176	0,31175062	0,6087909	TRIM41	tripartite motif containing 41 [Source:HGNC Symbol;Acc:HGNC:19013]
ENSG00000163625	0,23518583	2,91975102	1,14573857	0,3115757	0,6087909	WDFY3	WD repeat and FYVE domain containing 3 [Source:HGNC Symbol;Acc:HGNC:20751]
ENSG00000165898	0,17233671	4,77545369	1,14635587	0,31145264	0,6087909	ISCA2	iron-sulfur cluster assembly 2 [Source:HGNC Symbol;Acc:HGNC:19857]
ENSG00000177200	0,28204433	2,80755898	1,14587602	0,31154829	0,6087909	CHD9	chromodomain helicase DNA binding protein 9 [Source:HGNC Symbol;Acc:HGNC:25701]
ENSG00000205464	-0,2616155	0,82455991	1,14643729	0,31143641	0,6087909	ATP6AP1L	ATPase H+ transporting accessory protein 1 like [Source:HGNC Symbol;Acc:HGNC:28091]
ENSG00000213762	0,18868499	4,52745747	1,14503687	0,31171567	0,6087909	ZNF134	zinc finger protein 134 [Source:HGNC Symbol;Acc:HGNC:12918]
ENSG00000251322	0,20922755	3,18010814	1,14489666	0,31174365	0,6087909	SHANK3	SH3 and multiple ankyrin repeat domains 3 [Source:HGNC Symbol;Acc:HGNC:14294]

ENSG00000257337	-0,3918127	-0,5362647	1,14539623	0,31164398	0,6087909	AC068888.1	novel transcript, antisense to TENC1 & EIF4B
ENSG00000258527	0,51007625	-1,5395997	1,14557329	0,31160866	0,6087909	ASB9P1	ankyrin repeat and SOCS box containing 9 pseudogene 1 [Source:HGNC Symbol;Acc:HGNC:20972]
ENSG00000127526	0,18617802	5,12934071	1,1445825	0,31180635	0,60881837	SLC35E1	solute carrier family 35 member E1 [Source:HGNC Symbol;Acc:HGNC:20803]
ENSG00000102699	-0,2739869	5,34392054	1,14426946	0,31186885	0,60885903	PARP4	poly(ADP-ribose) polymerase family member 4 [Source:HGNC Symbol;Acc:HGNC:271]
ENSG00000071127	-0,2438266	8,64332582	1,1439163	0,31193939	0,60891535	WDR1	WD repeat domain 1 [Source:HGNC Symbol;Acc:HGNC:12754]
ENSG00000108349	-0,2122954	5,38818955	1,14345428	0,31203169	0,60895483	CASC3	CASC3 exon junction complex subunit [Source:HGNC Symbol;Acc:HGNC:17040]
ENSG00000171428	-0,2160502	1,99991054	1,14315671	0,31209116	0,60895483	NAT1	N-acetyltransferase 1 [Source:HGNC Symbol;Acc:HGNC:7645]
ENSG00000196834	-0,2432892	1,42415059	1,14279742	0,312163	0,60895483	POTEI	POTE ankyrin domain family member I [Source:HGNC Symbol;Acc:HGNC:37093]
ENSG00000197062	0,20287415	3,08303605	1,14310178	0,31210215	0,60895483	ZSCAN26	zinc finger and SCAN domain containing 26 [Source:HGNC Symbol;Acc:HGNC:12978]
ENSG00000220749	0,27982406	1,22189017	1,14277224	0,31216803	0,60895483	RPL21P28	ribosomal protein L21 pseudogene 28 [Source:HGNC Symbol;Acc:HGNC:36258]
ENSG00000108587	0,1473851	5,13200245	1,14204448	0,3123136	0,60907615	GOSR1	golgi SNAP receptor complex member 1 [Source:HGNC Symbol;Acc:HGNC:4430]
ENSG00000141098	0,21212857	3,98957624	1,14213162	0,31229617	0,60907615	GFOD2	glucose-fructose oxidoreductase domain containing 2 [Source:HGNC Symbol;Acc:HGNC:28159]
ENSG00000140067	0,44531915	-0,1527384	1,14137508	0,31244759	0,60909352	FAM181A	family with sequence similarity 181 member A [Source:HGNC Symbol;Acc:HGNC:20491]
ENSG00000166311	0,18573154	4,9696992	1,14158339	0,31240588	0,60909352	SMPD1	sphingomyelin phosphodiesterase 1 [Source:HGNC Symbol;Acc:HGNC:11120]
ENSG00000235098	0,22143785	3,82283847	1,14145268	0,31243205	0,60909352	ANKRD65	ankyrin repeat domain 65 [Source:HGNC Symbol;Acc:HGNC:42950]
ENSG00000206053	0,1641262	6,30434567	1,14058651	0,31260554	0,60932012	JPT2	Jupiter microtubule associated homolog 2 [Source:HGNC Symbol;Acc:HGNC:14137]
ENSG00000119333	-0,1654329	5,26382587	1,13944867	0,31283364	0,60968339	DYNC2I2	dynein 2 intermediate chain 2 [Source:HGNC Symbol;Acc:HGNC:28296]
ENSG00000103260	-0,1990162	4,51629069	1,13913774	0,31289602	0,60971478	METRN	meteorin, glial cell differentiation regulator [Source:HGNC Symbol;Acc:HGNC:14151]
ENSG00000133134	0,41330396	0,39461056	1,13895234	0,31293322	0,60971478	BEX2	brain expressed X-linked 2 [Source:HGNC Symbol;Acc:HGNC:30933]
ENSG00000177191	0,25946308	2,69604093	1,13871803	0,31298024	0,60972508	B3GNT8	UDP-GlcNAc:betaGal beta-1,3-N-acetylglucosaminyltransferase 8 [Source:HGNC Symbol;Acc:HGNC:24139]
ENSG00000131148	0,14986625	6,76001398	1,13847215	0,3130296	0,60972854	EMC8	ER membrane protein complex subunit 8 [Source:HGNC Symbol;Acc:HGNC:7864]
ENSG00000169217	-0,1467558	6,72861218	1,13829339	0,31306549	0,60972854	CD2BP2	CD2 cytoplasmic tail binding protein 2 [Source:HGNC Symbol;Acc:HGNC:1656]
ENSG00000137078	-0,6225503	-1,3580928	1,13808282	0,31310778	0,60972961	SIT1	signaling threshold regulating transmembrane adaptor 1 [Source:HGNC Symbol;Acc:HGNC:17710]
ENSG00000100373	0,19284459	4,24232691	1,13770474	0,31318372	0,60979503	UPK3A	uroplakin 3A [Source:HGNC Symbol;Acc:HGNC:12580]
ENSG00000112304	0,1862806	5,39146258	1,13718173	0,31328883	0,60979503	ACOT13	acyl-CoA thioesterase 13 [Source:HGNC Symbol;Acc:HGNC:20999]
ENSG00000113742	-0,2772022	2,37016769	1,13708467	0,31330834	0,60979503	CPEB4	cytoplasmic polyadenylation element binding protein 4 [Source:HGNC Symbol;Acc:HGNC:21747]
ENSG00000143493	0,17280211	4,08129314	1,1374298	0,31323897	0,60979503	INTS7	integrator complex subunit 7 [Source:HGNC Symbol;Acc:HGNC:24484]
ENSG00000138101	-0,1691709	3,8225366	1,13644133	0,3134377	0,60993631	DTNB	dystrobrevin beta [Source:HGNC Symbol;Acc:HGNC:3058]
ENSG00000172428	-0,2219781	5,39865399	1,13630848	0,31346443	0,60993631	COPS9	COP9 signalosome subunit 9 [Source:HGNC Symbol;Acc:HGNC:21314]
ENSG00000142961	0,44490133	0,97884103	1,13608891	0,3135086	0,60994102	MOB3C	MOB kinase activator 3C [Source:HGNC Symbol;Acc:HGNC:29800]
ENSG00000087586	0,14837455	7,73124461	1,13508301	0,31371109	0,61025371	AURKA	aurora kinase A [Source:HGNC Symbol;Acc:HGNC:11393]
ENSG00000113924	-0,1455099	7,17935376	1,13466388	0,31379552	0,61033668	HGD	homogentisate 1,2-dioxygenase [Source:HGNC Symbol;Acc:HGNC:4892]
ENSG00000124333	0,18066941	4,02918613	1,13400357	0,3139286	0,61051424	VAMP7	vesicle associated membrane protein 7 [Source:HGNC Symbol;Acc:HGNC:11486]
ENSG00000028310	-0,1671453	5,00367589	1,13325666	0,31407923	0,61053076	BRD9	bromodomain containing 9 [Source:HGNC Symbol;Acc:HGNC:25818]
ENSG00000100697	-0,1966946	4,0197495	1,13278094	0,31417523	0,61053076	DICER1	dicer 1, ribonuclease III [Source:HGNC Symbol;Acc:HGNC:17098]
ENSG00000105364	-0,1493786	6,97679953	1,13342353	0,31404557	0,61053076	MRPL4	mitochondrial ribosomal protein L4 [Source:HGNC Symbol;Acc:HGNC:14276]
ENSG00000110887	-0,5085457	-0,7040011	1,13237496	0,31425718	0,61053076	DAO	D-amino acid oxidase [Source:HGNC Symbol;Acc:HGNC:2671]
ENSG00000116580	-0,2576203	3,94239476	1,13273967	0,31418356	0,61053076	GON4L	gon-4 like [Source:HGNC Symbol;Acc:HGNC:25973]
ENSG00000173480	0,33882431	1,02095585	1,13230441	0,31427143	0,61053076	ZNF417	zinc finger protein 417 [Source:HGNC Symbol;Acc:HGNC:20646]
ENSG00000272277	-0,2812098	0,60054482	1,13241041	0,31425002	0,61053076	AL031963.3	novel transcript, antisense to RIPK1
ENSG00000272518	0,57567313	-0,8971806	1,13298572	0,3141339	0,61053076	AC036214.2	novel transcript, sense intronic to MRPS28
ENSG00000080371	0,19591403	4,24450602	1,13026249	0,31468413	0,61059709	RAB21	RAB21, member RAS oncogene family [Source:HGNC Symbol;Acc:HGNC:18263]
ENSG00000100139	0,18561024	5,31386057	1,13153506	0,31442683	0,61059709	MICALL1	MICAL like 1 [Source:HGNC Symbol;Acc:HGNC:29804]
ENSG00000102309	-0,2205263	4,60841631	1,13077239	0,314581	0,61059709	PIN4	peptidylprolyl cis/trans isomerase, NIMA-interacting 4 [Source:HGNC Symbol;Acc:HGNC:8992]

ENSG00000112655	0,2288259	7,59980646	1,13085549	0,3145642	0,61059709	PTK7	protein tyrosine kinase 7 (inactive) [Source:HGNC Symbol;Acc:HGNC:9618]
ENSG00000122218	-0,2867557	7,01914345	1,13006776	0,31472353	0,61059709	COPA	COPI coat complex subunit alpha [Source:HGNC Symbol;Acc:HGNC:2230]
ENSG00000167670	-0,1850459	5,60152514	1,13181971	0,31436932	0,61059709	CHAF1A	chromatin assembly factor 1 subunit A [Source:HGNC Symbol;Acc:HGNC:1910]
ENSG00000186522	0,14067317	5,67591651	1,13117622	0,31449935	0,61059709	SEPTIN10	septin 10 [Source:HGNC Symbol;Acc:HGNC:14349]
ENSG00000205871	0,24163641	1,4637398	1,13059344	0,31461719	0,61059709	RPS3AP47	RPS3A pseudogene 47 [Source:HGNC Symbol;Acc:HGNC:36744]
ENSG00000227946	-0,2142141	2,20056209	1,13039843	0,31465663	0,61059709	AC007383.1	novel transcript
ENSG00000274253	0,48605018	-0,55089	1,13079851	0,31457572	0,61059709	AC138649.1	novel transcript, sense overlapping NIPA1
ENSG00000079950	0,22547541	4,80618142	1,12909415	0,31492063	0,61064892	STX7	syntaxin 7 [Source:HGNC Symbol;Acc:HGNC:11442]
ENSG00000122033	-0,2233225	4,28138265	1,12878314	0,31498362	0,61064892	MTIF3	mitochondrial translational initiation factor 3 [Source:HGNC Symbol;Acc:HGNC:29788]
ENSG00000125629	0,26332906	5,47603423	1,1286531	0,31500997	0,61064892	INSIG2	insulin induced gene 2 [Source:HGNC Symbol;Acc:HGNC:20452]
ENSG00000141582	0,17362084	5,08323573	1,12936762	0,31486525	0,61064892	CBX4	chromobox 4 [Source:HGNC Symbol;Acc:HGNC:1554]
ENSG00000162511	-0,5092669	-1,6339814	1,1283138	0,31507873	0,61064892	LAPTM5	lysosomal protein transmembrane 5 [Source:HGNC Symbol;Acc:HGNC:29612]
ENSG00000163297	-0,2679678	4,32201631	1,1284737	0,31504632	0,61064892	ANTXR2	ANTXR cell adhesion molecule 2 [Source:HGNC Symbol;Acc:HGNC:21732]
ENSG00000177971	-0,1479049	6,87357412	1,12828462	0,31508464	0,61064892	IMP3	IMP U3 small nucleolar ribonucleoprotein 3 [Source:HGNC Symbol;Acc:HGNC:14497]
ENSG00000271737	-0,5546703	-0,7818677	1,1285243	0,31503607	0,61064892	AC008608.2	novel transcript
ENSG00000176454	-0,158868	4,91687989	1,12797339	0,31514773	0,61069018	LPCAT4	lysophosphatidylcholine acyltransferase 4 [Source:HGNC Symbol;Acc:HGNC:30059]
ENSG00000256806	-0,2077739	2,64615881	1,12770147	0,31520287	0,61071601	C17orf100	chromosome 17 open reading frame 100 [Source:HGNC Symbol;Acc:HGNC:34494]
ENSG00000181409	-0,2018377	2,44233801	1,1271427	0,31531622	0,6108313	AATK	apoptosis associated tyrosine kinase [Source:HGNC Symbol;Acc:HGNC:21]
ENSG00000189403	-0,1881942	8,01775953	1,12699594	0,315346	0,6108313	HMGB1	high mobility group box 1 [Source:HGNC Symbol;Acc:HGNC:4983]
ENSG00000168803	-0,2907594	3,02929615	1,12650865	0,3154449	0,61094188	ADAL	adenosine deaminase like [Source:HGNC Symbol;Acc:HGNC:31853]
ENSG00000011451	-0,193175	4,13243707	1,12595741	0,31555685	0,61105231	WIZ	WIZ zinc finger [Source:HGNC Symbol;Acc:HGNC:30917]
ENSG00000077152	-0,1783478	6,0302356	1,12580242	0,31558833	0,61105231	UBE2T	ubiquitin conjugating enzyme E2 T [Source:HGNC Symbol;Acc:HGNC:25009]
ENSG00000109771	-0,5678025	-1,6753849	1,12561013	0,3156274	0,61105231	LRP2BP	LRP2 binding protein [Source:HGNC Symbol;Acc:HGNC:25434]
ENSG00000020256	-0,1542779	4,82280954	1,12424023	0,31590593	0,61126752	ZFP64	ZFP64 zinc finger protein [Source:HGNC Symbol;Acc:HGNC:15940]
ENSG00000116478	0,1668673	7,05975375	1,12475283	0,31580167	0,61126752	HDAC1	histone deacetylase 1 [Source:HGNC Symbol;Acc:HGNC:4852]
ENSG00000136044	0,19699242	3,57952516	1,1244096	0,31587148	0,61126752	APPL2	adaptor protein, phosphotyrosine interacting with PH domain and leucine zipper 2 [Source:HGNC Symbol;Acc:HG
ENSG00000152582	-0,4183336	-0,2428451	1,12463079	0,31582649	0,61126752	SPEF2	sperm flagellar 2 [Source:HGNC Symbol;Acc:HGNC:26293]
ENSG00000140988	-0,1593599	11,1288949	1,12379272	0,315997	0,6112818	RPS2	ribosomal protein S2 [Source:HGNC Symbol;Acc:HGNC:10404]
ENSG00000186575	0,1673211	5,20174317	1,12387981	0,31597927	0,6112818	NF2	neurofibromin 2 [Source:HGNC Symbol;Acc:HGNC:7773]
ENSG00000120915	-0,2072455	3,82889449	1,12311804	0,31613437	0,61130469	EPHX2	epoxide hydrolase 2 [Source:HGNC Symbol;Acc:HGNC:3402]
ENSG00000159885	0,39668592	1,02326915	1,12334819	0,3160875	0,61130469	ZNF222	zinc finger protein 222 [Source:HGNC Symbol;Acc:HGNC:13015]
ENSG00000172086	-0,2666488	1,32953756	1,12330492	0,31609631	0,61130469	KRCC1	lysine rich coiled-coil 1 [Source:HGNC Symbol;Acc:HGNC:28039]
ENSG00000088986	-0,1813257	8,33308203	1,12283707	0,3161916	0,61133075	DYNLL1	dynein light chain LC8-type 1 [Source:HGNC Symbol;Acc:HGNC:15476]
ENSG00000104805	-0,2188642	8,4028671	1,1218199	0,31639892	0,61133075	NUCB1	nucleobindin 1 [Source:HGNC Symbol;Acc:HGNC:8043]
ENSG00000130584	-0,2235055	2,37352873	1,12210056	0,3163417	0,61133075	ZBTB46	zinc finger and BTB domain containing 46 [Source:HGNC Symbol;Acc:HGNC:16094]
ENSG00000136720	-0,1582646	6,8466495	1,12246691	0,31626702	0,61133075	HS6ST1	heparan sulfate 6-O-sulfotransferase 1 [Source:HGNC Symbol;Acc:HGNC:5201]
ENSG00000204991	-0,1842101	2,87087949	1,122148	0,31633202	0,61133075	SPIRE2	spire type actin nucleation factor 2 [Source:HGNC Symbol;Acc:HGNC:30623]
ENSG00000237651	-0,3301723	0,02829791	1,12188488	0,31638567	0,61133075	C2orf74	chromosome 2 open reading frame 74 [Source:HGNC Symbol;Acc:HGNC:34439]
ENSG00000065621	-0,2200083	2,22602276	1,12086559	0,31659361	0,61146905	GSTO2	glutathione S-transferase omega 2 [Source:HGNC Symbol;Acc:HGNC:23064]
ENSG00000111664	0,55337269	-1,2153011	1,12114063	0,31653748	0,61146905	GNB3	G protein subunit beta 3 [Source:HGNC Symbol;Acc:HGNC:4400]
ENSG00000180815	0,25763308	1,58906476	1,12085356	0,31659606	0,61146905	MAP3K15	mitogen-activated protein kinase kinase kinase 15 [Source:HGNC Symbol;Acc:HGNC:31689]
ENSG00000237649	0,2326613	5,71080731	1,12050597	0,31666702	0,61152524	KIFC1	kinesin family member C1 [Source:HGNC Symbol;Acc:HGNC:6389]
ENSG00000137492	0,16461886	4,43793168	1,11981324	0,3168085	0,6117176	THAP12	THAP domain containing 12 [Source:HGNC Symbol;Acc:HGNC:9440]
ENSG00000145022	-0,1989909	3,85881045	1,11907802	0,31695876	0,61176516	TCTA	T cell leukemia translocation altered [Source:HGNC Symbol;Acc:HGNC:11692]

ENSG00000170558	0,30741081	5,68175123	1,11935663	0,3169018	0,61176516	CDH2	cadherin 2 [Source:HGNC Symbol;Acc:HGNC:1759]
ENSG00000173457	-0,1868638	6,86032842	1,1190963	0,31695502	0,61176516	PPP1R14B	protein phosphatase 1 regulatory inhibitor subunit 14B [Source:HGNC Symbol;Acc:HGNC:9057]
ENSG00000123091	0,18430955	3,36445026	1,11814354	0,31714989	0,61188801	RNF11	ring finger protein 11 [Source:HGNC Symbol;Acc:HGNC:10056]
ENSG00000214223	-0,2031309	1,91286691	1,11855235	0,31706625	0,61188801	HNRNPA1P10	heterogeneous nuclear ribonucleoprotein A1 pseudogene 10 [Source:HGNC Symbol;Acc:HGNC:39128]
ENSG00000233994	0,367216	0,00763869	1,11794781	0,31718994	0,61188801	GDI2P2	GDP dissociation inhibitor 2 pseudogene 2 [Source:HGNC Symbol;Acc:HGNC:37976]
ENSG00000261783	-0,3876788	-0,0487629	1,11829429	0,31711905	0,61188801	AC009054.2	novel transcript, sense intronic to CFDP1
ENSG00000196313	0,32753009	4,56808364	1,11769513	0,31724167	0,61190698	POM121	POM121 transmembrane nucleoporin [Source:HGNC Symbol;Acc:HGNC:19702]
ENSG00000178149	-0,1629781	4,5481845	1,11697317	0,31738951	0,61211133	DALRD3	DALR anticodon binding domain containing 3 [Source:HGNC Symbol;Acc:HGNC:25536]
ENSG00000100058	-0,2225525	2,86449191	1,11590451	0,31760853	0,61217186	CRYBB2P1	crystallin beta B2 pseudogene 1 [Source:HGNC Symbol;Acc:HGNC:2399]
ENSG00000100479	-0,1549973	3,70269995	1,11642705	0,31750141	0,61217186	POLE2	DNA polymerase epsilon 2, accessory subunit [Source:HGNC Symbol;Acc:HGNC:9178]
ENSG00000109111	0,30893164	6,98994798	1,11579781	0,31763041	0,61217186	SUPT6H	SPT6 homolog, histone chaperone and transcription elongation factor [Source:HGNC Symbol;Acc:HGNC:11470]
ENSG00000117984	0,17322652	8,27985291	1,11579942	0,31763008	0,61217186	CTSD	cathepsin D [Source:HGNC Symbol;Acc:HGNC:2529]
ENSG00000155850	0,35523365	1,66767986	1,11585657	0,31761836	0,61217186	SLC26A2	solute carrier family 26 member 2 [Source:HGNC Symbol;Acc:HGNC:10994]
ENSG00000104643	0,18770048	2,84005156	1,11487656	0,31781942	0,61237456	MTMR9	myotubularin related protein 9 [Source:HGNC Symbol;Acc:HGNC:14596]
ENSG00000242100	-0,3044562	0,96633501	1,11488713	0,31781725	0,61237456	RPL9P32	ribosomal protein L9 pseudogene 32 [Source:HGNC Symbol;Acc:HGNC:36418]
ENSG00000135999	0,19398327	2,98091246	1,11455714	0,31788499	0,61242013	EPC2	enhancer of polycomb homolog 2 [Source:HGNC Symbol;Acc:HGNC:24543]
ENSG00000276550	-0,1856241	2,5156209	1,11416593	0,31796533	0,61249414	HERC2P2	hect domain and RLD 2 pseudogene 2 [Source:HGNC Symbol;Acc:HGNC:4870]
ENSG00000106484	0,16786733	6,46409946	1,11395742	0,31800816	0,61249588	MEST	mesoderm specific transcript [Source:HGNC Symbol;Acc:HGNC:7028]
ENSG00000100393	0,47279126	4,90685636	1,11342909	0,31811672	0,61262421	EP300	E1A binding protein p300 [Source:HGNC Symbol;Acc:HGNC:3373]
ENSG00000168872	0,17462211	4,70931604	1,11306412	0,31819175	0,61268793	DDX19A	DEAD-box helicase 19A [Source:HGNC Symbol;Acc:HGNC:25628]
ENSG00000096092	-0,1973488	5,30813569	1,11227026	0,31835504	0,61276004	TMEM14A	transmembrane protein 14A [Source:HGNC Symbol;Acc:HGNC:21076]
ENSG00000183696	-0,1741379	3,62058889	1,1123475	0,31833914	0,61276004	UPP1	uridine phosphorylase 1 [Source:HGNC Symbol;Acc:HGNC:12576]
ENSG00000271551	0,49609531	-0,9210302	1,11264682	0,31827757	0,61276004	AL355297.3	novel transcript, antisense to ARID1B
ENSG00000222033	-0,2012153	2,81518309	1,11198824	0,31841307	0,61279101	LINC01124	long intergenic non-protein coding RNA 1124 [Source:HGNC Symbol;Acc:HGNC:49270]
ENSG00000237149	-0,1736776	2,73667661	1,11118085	0,31857931	0,61303018	ZNF503-AS2	ZNF503 antisense RNA 2 [Source:HGNC Symbol;Acc:HGNC:23525]
ENSG00000108352	0,50230345	-0,0764058	1,11053479	0,31871242	0,61318936	RAPGEFL1	Rap guanine nucleotide exchange factor like 1 [Source:HGNC Symbol;Acc:HGNC:17428]
ENSG00000182963	0,38607852	-0,3265984	1,11033485	0,31875363	0,61318936	GJC1	gap junction protein gamma 1 [Source:HGNC Symbol;Acc:HGNC:4280]
ENSG00000247121	0,69354005	-0,984106	1,11016835	0,31878795	0,61318936	AC009126.1	novel transcript, antisense to LNPEP and ERAP2
ENSG00000101624	0,17274461	3,62311902	1,10995101	0,31883277	0,61319482	CEP76	centrosomal protein 76 [Source:HGNC Symbol;Acc:HGNC:25727]
ENSG00000179163	0,1666551	5,77574653	1,10940648	0,31894508	0,61330588	FUCA1	alpha-L-fucosidase 1 [Source:HGNC Symbol;Acc:HGNC:4006]
ENSG00000179240	0,34960801	0,47010123	1,10926403	0,31897448	0,61330588	GVQW3	GVQW motif containing 3 [Source:HGNC Symbol;Acc:HGNC:51239]
ENSG00000150054	0,20334249	2,88435959	1,10817047	0,31920024	0,61365921	MPP7	membrane palmitoylated protein 7 [Source:HGNC Symbol;Acc:HGNC:26542]
ENSG00000166192	-0,2893754	0,91450524	1,10793748	0,31924837	0,61367099	SENP8	SUMO peptidase family member, NEDD8 specific [Source:HGNC Symbol;Acc:HGNC:22992]
ENSG00000185624	-0,2364697	12,2051631	1,10738607	0,31936233	0,61380927	P4HB	prolyl 4-hydroxylase subunit beta [Source:HGNC Symbol;Acc:HGNC:8548]
ENSG00000204152	0,30295013	0,41512401	1,10697208	0,31944792	0,61381227	TIMM23B	translocase of inner mitochondrial membrane 23 homolog B [Source:HGNC Symbol;Acc:HGNC:23581]
ENSG00000261556	0,54298341	-1,5801541	1,10715264	0,31941059	0,61381227	SMG1P7	SMG1 pseudogene 7 [Source:HGNC Symbol;Acc:HGNC:49864]
ENSG00000272554	-0,5464957	-1,7229147	1,10627415	0,3195923	0,61400892	AC012087.2	novel transcript
ENSG00000132646	-0,2187507	8,90559295	1,10479459	0,31989868	0,61411298	PCNA	proliferating cell nuclear antigen [Source:HGNC Symbol;Acc:HGNC:8729]
ENSG00000134780	0,2122474	4,11556025	1,1053319	0,31978736	0,61411298	DAGLA	diacylglycerol lipase alpha [Source:HGNC Symbol;Acc:HGNC:1165]
ENSG00000138028	-0,199771	4,58898287	1,10508451	0,31983861	0,61411298	CGREF1	cell growth regulator with EF-hand domain 1 [Source:HGNC Symbol;Acc:HGNC:16962]
ENSG00000143093	0,22111062	5,14402965	1,10518089	0,31981864	0,61411298	STRIP1	striatin interacting protein 1 [Source:HGNC Symbol;Acc:HGNC:25916]
ENSG00000196183	-0,4790892	-1,4591519	1,10479963	0,31989763	0,61411298	RPS2P4	ribosomal protein S2 pseudogene 4 [Source:HGNC Symbol;Acc:HGNC:19814]
ENSG00000232112	-0,2117817	7,09547658	1,1057137	0,3197083	0,61411298	TMA7	translation machinery associated 7 homolog [Source:HGNC Symbol;Acc:HGNC:26932]
ENSG00000010803	0,21046746	4,09990148	1,10363728	0,32013863	0,61425075	SCMH1	Scm polycomb group protein homolog 1 [Source:HGNC Symbol;Acc:HGNC:19003]

ENSG00000103194	0,16366433	6,57107848	1,10389147	0,3200859	0,61425075	USP10	ubiquitin specific peptidase 10 [Source:HGNC Symbol;Acc:HGNC:12608]
ENSG00000156735	0,16998878	4,24246695	1,10374798	0,32011566	0,61425075	BAG4	BAG cochaperone 4 [Source:HGNC Symbol;Acc:HGNC:940]
ENSG00000224272	-0,284301	2,24066916	1,10405272	0,32005246	0,61425075	AC131097.2	novel transcript
ENSG00000082269	-0,2460488	2,42492258	1,10199054	0,3204805	0,61454194	FAM135A	family with sequence similarity 135 member A [Source:HGNC Symbol;Acc:HGNC:21084]
ENSG00000118046	-0,2083734	4,91444992	1,10240093	0,32039525	0,61454194	STK11	serine/threonine kinase 11 [Source:HGNC Symbol;Acc:HGNC:11389]
ENSG00000127554	-0,1540991	5,5236447	1,1017694	0,32052646	0,61454194	GFER	growth factor, augmenter of liver regeneration [Source:HGNC Symbol;Acc:HGNC:4236]
ENSG00000156968	-0,1966383	4,3419848	1,10164135	0,32055307	0,61454194	MPV17L	MPV17 mitochondrial inner membrane protein like [Source:HGNC Symbol;Acc:HGNC:26827]
ENSG00000198915	0,84946402	-1,5602146	1,10148842	0,32058485	0,61454194	RASGEF1A	RasGEF domain family member 1A [Source:HGNC Symbol;Acc:HGNC:24246]
ENSG00000203724	-0,2153245	2,12686213	1,10164126	0,32055309	0,61454194	C1orf53	chromosome 1 open reading frame 53 [Source:HGNC Symbol;Acc:HGNC:30003]
ENSG00000231434	-0,352569	0,79042591	1,10203944	0,32047034	0,61454194	HSP90AA3P	heat shock protein 90 alpha family class A member 3, pseudogene [Source:HGNC Symbol;Acc:HGNC:5254]
ENSG00000073150	-0,3004504	0,39630895	1,10125529	0,32063332	0,61455421	PANX2	pannexin 2 [Source:HGNC Symbol;Acc:HGNC:8600]
ENSG00000124380	0,15677585	5,2621397	1,1000459	0,32088492	0,61490884	SNRNP27	small nuclear ribonucleoprotein U4/U6.U5 subunit 27 [Source:HGNC Symbol;Acc:HGNC:30240]
ENSG00000165943	0,17058578	4,21553304	1,0999613	0,32090253	0,61490884	MOAP1	modulator of apoptosis 1 [Source:HGNC Symbol;Acc:HGNC:16658]
ENSG00000215915	-0,2209382	1,4993526	1,09967384	0,32096238	0,61494287	ATAD3C	ATPase family AAA domain containing 3C [Source:HGNC Symbol;Acc:HGNC:32151]
ENSG00000173660	-0,1631689	8,02360138	1,09937796	0,321024	0,61498027	UQCRH	ubiquinol-cytochrome c reductase hinge protein [Source:HGNC Symbol;Acc:HGNC:12590]
ENSG00000101040	0,1728233	6,62191638	1,09820112	0,32126926	0,61503092	ZMYND8	zinc finger MYND-type containing 8 [Source:HGNC Symbol;Acc:HGNC:9397]
ENSG00000124541	-0,1496288	7,09613657	1,09825709	0,32125759	0,61503092	RRP36	ribosomal RNA processing 36 [Source:HGNC Symbol;Acc:HGNC:21374]
ENSG00000143258	0,2277097	3,65319478	1,09858029	0,32119021	0,61503092	USP21	ubiquitin specific peptidase 21 [Source:HGNC Symbol;Acc:HGNC:12620]
ENSG00000143434	-0,3664178	0,5262582	1,09880197	0,321144	0,61503092	SEMA6C	semaphorin 6C [Source:HGNC Symbol;Acc:HGNC:10740]
ENSG00000163344	0,16270469	4,659071	1,09806	0,32129868	0,61503092	PMVK	phosphomevalonate kinase [Source:HGNC Symbol;Acc:HGNC:9141]
ENSG00000215022	0,65749399	-2,0014978	1,09803916	0,32130303	0,61503092	AL008729.1	novel transcript, antisense to PHACTR1
ENSG00000139990	-0,1674752	4,3733401	1,09742916	0,32143028	0,61511331	DCAF5	DDB1 and CUL4 associated factor 5 [Source:HGNC Symbol;Acc:HGNC:20224]
ENSG00000164169	0,19855491	2,90351913	1,09761803	0,32139087	0,61511331	PRMT9	protein arginine methyltransferase 9 [Source:HGNC Symbol;Acc:HGNC:25099]
ENSG00000101115	0,48036338	-1,5083106	1,09667892	0,3215869	0,61512235	SALL4	spalt like transcription factor 4 [Source:HGNC Symbol;Acc:HGNC:15924]
ENSG00000102053	0,62205194	-1,6608395	1,09690347	0,32154001	0,61512235	ZC3H12B	zinc finger CCCH-type containing 12B [Source:HGNC Symbol;Acc:HGNC:17407]
ENSG00000131697	0,18312425	3,64031963	1,09634475	0,32165669	0,61512235	NPHP4	nephrocystin 4 [Source:HGNC Symbol;Acc:HGNC:19104]
ENSG00000132155	0,17986839	6,0888595	1,09674435	0,32157323	0,61512235	RAF1	Raf-1 proto-oncogene, serine/threonine kinase [Source:HGNC Symbol;Acc:HGNC:9829]
ENSG00000137522	0,15466531	5,22739626	1,09599505	0,32172975	0,61512235	RNF121	ring finger protein 121 [Source:HGNC Symbol;Acc:HGNC:21070]
ENSG00000164934	0,18664879	6,1325187	1,09602314	0,32172388	0,61512235	DCAF13	DDB1 and CUL4 associated factor 13 [Source:HGNC Symbol;Acc:HGNC:24535]
ENSG00000166439	0,16411431	3,58258743	1,09614782	0,32169783	0,61512235	RNF169	ring finger protein 169 [Source:HGNC Symbol;Acc:HGNC:26961]
ENSG00000144589	0,20462759	3,70272116	1,09541919	0,32185011	0,61527196	STK11IP	serine/threonine kinase 11 interacting protein [Source:HGNC Symbol;Acc:HGNC:19184]
ENSG00000029363	0,21081406	6,41883705	1,09447114	0,32204841	0,61532897	BCLAF1	BCL2 associated transcription factor 1 [Source:HGNC Symbol;Acc:HGNC:16863]
ENSG00000135917	-0,1895974	3,34603981	1,09479995	0,32197962	0,61532897	SLC19A3	solute carrier family 19 member 3 [Source:HGNC Symbol;Acc:HGNC:16266]
ENSG00000153531	-0,5252859	-1,6484759	1,09478311	0,32198314	0,61532897	ADPRHL1	ADP-ribosylhydrolase like 1 [Source:HGNC Symbol;Acc:HGNC:21303]
ENSG00000213235	-0,4447664	-0,536215	1,09449329	0,32204378	0,61532897	EEF1A1P16	eukaryotic translation elongation factor 1 alpha 1 pseudogene 16 [Source:HGNC Symbol;Acc:HGNC:37889]
ENSG00000091164	0,199163	6,56753716	1,09008285	0,32296864	0,61564735	TXNL1	thioredoxin like 1 [Source:HGNC Symbol;Acc:HGNC:12436]
ENSG00000101187	0,17959262	5,84418387	1,09155122	0,32266029	0,61564735	SLCO4A1	solute carrier organic anion transporter family member 4A1 [Source:HGNC Symbol;Acc:HGNC:10953]
ENSG00000113851	0,1491085	4,63553445	1,09140638	0,32269069	0,61564735	CRBN	cereblon [Source:HGNC Symbol;Acc:HGNC:30185]
ENSG00000118454	0,17080482	3,85767222	1,09016364	0,32295166	0,61564735	ANKRD13C	ankyrin repeat domain 13C [Source:HGNC Symbol;Acc:HGNC:25374]
ENSG00000131495	-0,1677398	6,40330014	1,09170018	0,32262904	0,61564735	NDUFA2	NADH:ubiquinone oxidoreductase subunit A2 [Source:HGNC Symbol;Acc:HGNC:7685]
ENSG00000134419	-0,1494333	9,54661569	1,09006539	0,32297231	0,61564735	RPS15A	ribosomal protein S15a [Source:HGNC Symbol;Acc:HGNC:10389]
ENSG00000153558	0,2609051	1,76180889	1,09179713	0,32260869	0,61564735	FBXL2	F-box and leucine rich repeat protein 2 [Source:HGNC Symbol;Acc:HGNC:13598]
ENSG00000156030	0,37031553	2,99744722	1,09058732	0,32286266	0,61564735	MIDEAS	mitotic deacetylase associated SANT domain protein [Source:HGNC Symbol;Acc:HGNC:19853]
ENSG00000170142	-0,1914961	4,8953523	1,09070811	0,32283729	0,61564735	UBE2E1	ubiquitin conjugating enzyme E2 E1 [Source:HGNC Symbol;Acc:HGNC:12477]

ENSG00000170677	0,14904601	4,84361858	1,09026324	0,32293073	0,61564735	SOCS6	suppressor of cytokine signaling 6 [Source:HGNC Symbol;Acc:HGNC:16833]
ENSG00000181511	-0,5542969	-1,4354072	1,09176995	0,3226144	0,61564735	GLRX5P1	GLRX5 pseudogene 1 [Source:HGNC Symbol;Acc:HGNC:55062]
ENSG00000187391	-0,344482	0,91729245	1,09087994	0,3228012	0,61564735	MAGI2	membrane associated guanylate kinase, WW and PDZ domain containing 2 [Source:HGNC Symbol;Acc:HGNC:1895
ENSG00000188368	0,43833964	0,15240941	1,09005925	0,3229736	0,61564735	PRR19	proline rich 19 [Source:HGNC Symbol;Acc:HGNC:33728]
ENSG00000204392	-0,1679625	6,20503576	1,09006516	0,32297236	0,61564735	LSM2	LSM2 homolog, U6 small nuclear RNA and mRNA degradation associated [Source:HGNC Symbol;Acc:HGNC:13940
ENSG00000204556	-0,5679387	-1,3528386	1,09017385	0,32294952	0,61564735	AL450124.1	zinc-finger pseudogene
ENSG00000214078	0,15502361	8,77586715	1,09289807	0,32237784	0,61564735	CPNE1	copine 1 [Source:HGNC Symbol;Acc:HGNC:2314]
ENSG00000215492	0,18808611	1,99827727	1,09132601	0,32270756	0,61564735	HNRNPA1P7	heterogeneous nuclear ribonucleoprotein A1 pseudogene 7 [Source:HGNC Symbol;Acc:HGNC:31015]
ENSG00000227036	-0,1572607	4,36255433	1,09120434	0,3227331	0,61564735	LINC00511	long intergenic non-protein coding RNA 511 [Source:HGNC Symbol;Acc:HGNC:43564]
ENSG00000130254	-0,3415279	4,86736472	1,08959894	0,32307035	0,61575144	SAFB2	scaffold attachment factor B2 [Source:HGNC Symbol;Acc:HGNC:21605]
ENSG00000092758	-0,2739281	6,74359585	1,0892175	0,32315056	0,61582396	COL9A3	collagen type IX alpha 3 chain [Source:HGNC Symbol;Acc:HGNC:2219]
ENSG00000206149	0,2960436	1,06685104	1,08815309	0,32337453	0,61617041	HERC2P9	hect domain and RLD 2 pseudogene 9 [Source:HGNC Symbol;Acc:HGNC:30495]
ENSG00000101361	-0,1528429	9,32256982	1,08763967	0,32348264	0,61621567	NOP56	NOP56 ribonucleoprotein [Source:HGNC Symbol;Acc:HGNC:15911]
ENSG00000125971	-0,1752765	4,64206536	1,08770945	0,32346795	0,61621567	DYNLRB1	dynein light chain roadblock-type 1 [Source:HGNC Symbol;Acc:HGNC:15468]
ENSG00000111832	-0,2093159	5,8206303	1,08720405	0,32357442	0,6162497	RWDD1	RWD domain containing 1 [Source:HGNC Symbol;Acc:HGNC:20993]
ENSG00000114902	-0,172987	6,56133756	1,08695427	0,32362706	0,6162497	SPCS1	signal peptidase complex subunit 1 [Source:HGNC Symbol;Acc:HGNC:23401]
ENSG00000225345	0,4780033	-0,0673165	1,08697216	0,32362329	0,6162497	SNX18P3	sorting nexin 18 pseudogene 3 [Source:HGNC Symbol;Acc:HGNC:39611]
ENSG00000082258	0,18990051	4,47122449	1,08565327	0,32390144	0,61645077	CCNT2	cyclin T2 [Source:HGNC Symbol;Acc:HGNC:1600]
ENSG00000124782	0,39224494	4,67096825	1,08569328	0,32389299	0,61645077	RREB1	ras responsive element binding protein 1 [Source:HGNC Symbol;Acc:HGNC:10449]
ENSG00000132549	0,28602473	2,56474938	1,0857112	0,32388921	0,61645077	VPS13B	vacuolar protein sorting 13 homolog B [Source:HGNC Symbol;Acc:HGNC:2183]
ENSG00000162396	0,20555602	3,02836823	1,08585887	0,32385805	0,61645077	PARS2	prolyl-tRNA synthetase 2, mitochondrial [Source:HGNC Symbol;Acc:HGNC:30563]
ENSG00000052723	-0,151001	4,3858262	1,08533409	0,32396881	0,61649633	SIKE1	suppressor of IKBKE 1 [Source:HGNC Symbol;Acc:HGNC:26119]
ENSG00000164182	-0,1708937	5,72698483	1,08514001	0,32400978	0,61649633	NDUFAF2	NADH:ubiquinone oxidoreductase complex assembly factor 2 [Source:HGNC Symbol;Acc:HGNC:28086]
ENSG00000164983	-0,2397084	1,66321202	1,08493337	0,32405341	0,61649906	TMEM65	transmembrane protein 65 [Source:HGNC Symbol;Acc:HGNC:25203]
ENSG00000010322	-0,1842751	4,82567777	1,08407529	0,32423469	0,61668332	NISCH	nischarin [Source:HGNC Symbol;Acc:HGNC:18006]
ENSG00000225975	-0,6073784	-1,1526783	1,08418627	0,32421124	0,61668332	LINC01534	long intergenic non-protein coding RNA 1534 [Source:HGNC Symbol;Acc:HGNC:51281]
ENSG00000106400	-0,1652031	6,15684279	1,08344094	0,3243688	0,61685809	ZNHIT1	zinc finger HIT-type containing 1 [Source:HGNC Symbol;Acc:HGNC:21688]
ENSG00000114767	-0,1482089	6,37087245	1,08280624	0,32450307	0,6170331	RRP9	ribosomal RNA processing 9, U3 small nucleolar RNA binding protein [Source:HGNC Symbol;Acc:HGNC:16829]
ENSG00000228169	-0,4365037	-0,4536833	1,08167657	0,32474224	0,61740752	PPIAP19	peptidylprolyl isomerase A pseudogene 19 [Source:HGNC Symbol;Acc:HGNC:31663]
ENSG00000240303	-0,4561929	-0,6421373	1,08067294	0,32495495	0,61773153	ACAD11	acyl-CoA dehydrogenase family member 11 [Source:HGNC Symbol;Acc:HGNC:30211]
ENSG00000116062	0,16790402	6,31287353	1,0802619	0,32504212	0,61773648	MSH6	mutS homolog 6 [Source:HGNC Symbol;Acc:HGNC:7329]
ENSG00000138617	-0,2334659	3,21374765	1,08032504	0,32502873	0,61773648	PARP16	poly(ADP-ribose) polymerase family member 16 [Source:HGNC Symbol;Acc:HGNC:26040]
ENSG00000166200	0,15908946	6,11439221	1,0799017	0,32511854	0,61778789	COPS2	COP9 signalosome subunit 2 [Source:HGNC Symbol;Acc:HGNC:30747]
ENSG00000227403	0,36651091	-0,4127171	1,07973582	0,32515374	0,61778789	LINC01806	long intergenic non-protein coding RNA 1806 [Source:HGNC Symbol;Acc:HGNC:52599]
ENSG00000119711	-0,2085943	4,5465495	1,07925229	0,32525639	0,61790254	ALDH6A1	aldehyde dehydrogenase 6 family member A1 [Source:HGNC Symbol;Acc:HGNC:7179]
ENSG00000148730	0,20332428	5,21011863	1,07772105	0,32558175	0,61837623	EIF4EBP2	eukaryotic translation initiation factor 4E binding protein 2 [Source:HGNC Symbol;Acc:HGNC:3289]
ENSG00000166889	0,1749045	4,66657615	1,07768044	0,32559039	0,61837623	PATL1	PAT1 homolog 1, processing body mRNA decay factor [Source:HGNC Symbol;Acc:HGNC:26721]
ENSG00000049245	0,18945823	6,2341437	1,07556075	0,3260416	0,618415	VAMP3	vesicle associated membrane protein 3 [Source:HGNC Symbol;Acc:HGNC:12644]
ENSG00000055211	0,14469419	5,96790248	1,07619499	0,32590649	0,618415	GINM1	glycoprotein integral membrane 1 [Source:HGNC Symbol;Acc:HGNC:21074]
ENSG00000081377	0,15657399	3,91592957	1,0752565	0,32610644	0,618415	CDC14B	cell division cycle 14B [Source:HGNC Symbol;Acc:HGNC:1719]
ENSG00000101407	0,14405133	5,40034645	1,07103654	0,32700773	0,618415	TTI1	TELO2 interacting protein 1 [Source:HGNC Symbol;Acc:HGNC:29029]
ENSG00000101464	0,14931691	6,48133849	1,07409329	0,32635451	0,618415	PIGU	phosphatidylinositol glycan anchor biosynthesis class U [Source:HGNC Symbol;Acc:HGNC:15791]
ENSG00000107281	0,21258473	2,65459693	1,07187346	0,32682869	0,618415	NPDC1	neural proliferation, differentiation and control 1 [Source:HGNC Symbol;Acc:HGNC:7899]
ENSG00000108064	-0,1595033	6,03862774	1,07235938	0,3267248	0,618415	TFAM	transcription factor A, mitochondrial [Source:HGNC Symbol;Acc:HGNC:11741]

ENSG00000111711	0,15693229	4,59335691	1,07157072	0,32689344	0,618415	GOLT1B	golgi transport 1B [Source:HGNC Symbol;Acc:HGNC:20175]
ENSG00000114648	0,16973064	4,49029064	1,07131156	0,32694888	0,618415	KLHL18	kelch like family member 18 [Source:HGNC Symbol;Acc:HGNC:29120]
ENSG00000115875	-0,1407083	8,22012767	1,07648338	0,32584509	0,618415	SRSF7	serine and arginine rich splicing factor 7 [Source:HGNC Symbol;Acc:HGNC:10789]
ENSG00000125912	-0,1779752	8,02562348	1,07273712	0,32664408	0,618415	NCLN	nicalin [Source:HGNC Symbol;Acc:HGNC:26923]
ENSG00000134644	0,20507923	5,67066547	1,07148995	0,32691071	0,618415	PUM1	pumilio RNA binding family member 1 [Source:HGNC Symbol;Acc:HGNC:14957]
ENSG00000136848	0,18514116	5,61165953	1,07222292	0,32675397	0,618415	DAB2IP	DAB2 interacting protein [Source:HGNC Symbol;Acc:HGNC:17294]
ENSG00000137869	-0,2998203	2,68777819	1,07201484	0,32679846	0,618415	CYP19A1	cytochrome P450 family 19 subfamily A member 1 [Source:HGNC Symbol;Acc:HGNC:2594]
ENSG00000146232	-0,1587569	4,79145487	1,07237393	0,32672169	0,618415	NFKBIE	NFKB inhibitor epsilon [Source:HGNC Symbol;Acc:HGNC:7799]
ENSG00000146776	-0,2637096	1,39874176	1,07278238	0,32663441	0,618415	ATXN7L1	ataxin 7 like 1 [Source:HGNC Symbol;Acc:HGNC:22210]
ENSG00000147316	0,16652638	4,4573411	1,07641536	0,32585957	0,618415	MCPH1	microcephalin 1 [Source:HGNC Symbol;Acc:HGNC:6954]
ENSG00000156006	0,38792581	-0,5151583	1,07236369	0,32672388	0,618415	NAT2	N-acetyltransferase 2 [Source:HGNC Symbol;Acc:HGNC:7646]
ENSG00000157514	0,38391629	0,97893458	1,07149067	0,32691056	0,618415	TSC22D3	TSC22 domain family member 3 [Source:HGNC Symbol;Acc:HGNC:3051]
ENSG00000159335	-0,1779298	6,75941268	1,07113383	0,32698691	0,618415	PTMS	parathymosin [Source:HGNC Symbol;Acc:HGNC:9629]
ENSG00000159905	0,73427476	-1,0080837	1,07428294	0,32631404	0,618415	ZNF221	zinc finger protein 221 [Source:HGNC Symbol;Acc:HGNC:13014]
ENSG00000168152	0,21052247	1,61987728	1,07324519	0,32653555	0,618415	THAP9	THAP domain containing 9 [Source:HGNC Symbol;Acc:HGNC:23192]
ENSG00000169570	0,29957923	1,0138915	1,07299164	0,3265897	0,618415	DTWD2	DTW domain containing 2 [Source:HGNC Symbol;Acc:HGNC:19334]
ENSG00000196456	-0,2164818	1,72480328	1,07652222	0,32583682	0,618415	ZNF775	zinc finger protein 775 [Source:HGNC Symbol;Acc:HGNC:28501]
ENSG00000225335	-0,3540354	0,08515519	1,07296698	0,32659497	0,618415	AC016027.1	novel transcript
ENSG00000225486	-0,4693706	-1,3178488	1,07548962	0,32605675	0,618415	AL663058.1	acid phosphatase 1, soluble (ACP1) pseudogene
ENSG00000227192	0,33080059	-0,0700158	1,07592612	0,32596376	0,618415	AL023581.2	novel transcript
ENSG00000230667	-0,3909018	-0,7034387	1,07433381	0,32630319	0,618415	SETSIP	SET like protein [Source:HGNC Symbol;Acc:HGNC:42937]
ENSG00000231503	-0,3131637	0,45808044	1,07454342	0,32625848	0,618415	PTMAP4	prothymosin alpha pseudogene 4 [Source:HGNC Symbol;Acc:HGNC:9627]
ENSG00000234335	-0,2851261	0,37393054	1,0755723	0,32603914	0,618415	RPS4XP11	ribosomal protein S4X pseudogene 11 [Source:HGNC Symbol;Acc:HGNC:37013]
ENSG00000249915	0,40097006	-0,7293205	1,07724416	0,32568318	0,618415	PDCD6	programmed cell death 6 [Source:HGNC Symbol;Acc:HGNC:8765]
ENSG00000268093	-0,6860956	-2,0939018	1,07548989	0,3260567	0,618415	AC022154.1	novel transcript, antisense to SPHK2
ENSG00000285796	-0,4343279	0,24647566	1,07105075	0,32700469	0,618415	AL162458.1	novel transcript
ENSG00000134452	-0,1378911	6,15563177	1,07042977	0,32713762	0,61858057	FBH1	F-box DNA helicase 1 [Source:HGNC Symbol;Acc:HGNC:13620]
ENSG00000100865	-0,1477952	5,45873552	1,06875803	0,32749589	0,61916267	CINP	cyclin dependent kinase 2 interacting protein [Source:HGNC Symbol;Acc:HGNC:23789]
ENSG00000250571	-0,3680773	-0,0146245	1,06859795	0,32753023	0,61916267	GLI4	GLI family zinc finger 4 [Source:HGNC Symbol;Acc:HGNC:4320]
ENSG00000160325	0,19509974	3,13341757	1,06815531	0,3276252	0,61918196	CACFD1	calcium channel flower domain containing 1 [Source:HGNC Symbol;Acc:HGNC:1365]
ENSG00000235852	0,87460549	-1,9896589	1,06828936	0,32759643	0,61918196	AC005540.1	novel transcript
ENSG00000163877	0,20999371	3,9186879	1,06779246	0,32770308	0,61924904	SNIP1	Smad nuclear interacting protein 1 [Source:HGNC Symbol;Acc:HGNC:30587]
ENSG00000071246	0,7611812	-1,5427137	1,06685482	0,32790447	0,61951351	VASH1	vasohibin 1 [Source:HGNC Symbol;Acc:HGNC:19964]
ENSG00000197265	0,15269136	5,84389321	1,06674602	0,32792785	0,61951351	GTF2E2	general transcription factor IIE subunit 2 [Source:HGNC Symbol;Acc:HGNC:4651]
ENSG00000279453	0,68974239	-1,9985521	1,06653566	0,32797306	0,61951881	Z99129.4	TEC
ENSG00000141741	-0,1553914	5,12291039	1,06507315	0,32828764	0,62003286	MIEN1	migration and invasion enhancer 1 [Source:HGNC Symbol;Acc:HGNC:28230]
ENSG00000189266	-0,1436371	6,05256607	1,06458539	0,32839265	0,62011172	PNRC2	proline rich nuclear receptor coactivator 2 [Source:HGNC Symbol;Acc:HGNC:23158]
ENSG00000214026	-0,235127	4,68760019	1,06448493	0,32841429	0,62011172	MRPL23	mitochondrial ribosomal protein L23 [Source:HGNC Symbol;Acc:HGNC:10322]
ENSG00000090975	-0,1974937	2,88560025	1,0639886	0,32852121	0,6202278	PITPNM2	phosphatidylinositol transfer protein membrane associated 2 [Source:HGNC Symbol;Acc:HGNC:21044]
ENSG00000152944	0,18341792	3,75028646	1,06322666	0,32868545	0,6202278	MED21	mediator complex subunit 21 [Source:HGNC Symbol;Acc:HGNC:11473]
ENSG00000163866	-0,1479735	6,14036354	1,06301774	0,3287305	0,6202278	SMIM12	small integral membrane protein 12 [Source:HGNC Symbol;Acc:HGNC:25154]
ENSG00000240616	-0,1848974	2,43994819	1,06380421	0,32856094	0,6202278	RPS6P25	ribosomal protein S6 pseudogene 25 [Source:HGNC Symbol;Acc:HGNC:35948]
ENSG00000251867	0,69114235	-1,8463631	1,06320259	0,32869064	0,6202278	AC009812.1	novel transcript, antisense to ZBTB10
ENSG00000255993	0,49965786	-1,4473516	1,06341679	0,32864445	0,6202278	PSMC1P9	proteasome 26S subunit, ATPase 1 pseudogene 9 [Source:HGNC Symbol;Acc:HGNC:39784]

ENSG00000124224	-0,3349931	0,40219741	1,06138215	0,32908354	0,6204092	PPP4R1L	protein phosphatase 4 regulatory subunit 1 like (pseudogene) [Source:HGNC Symbol;Acc:HGNC:15755]
ENSG00000160072	-0,1681221	6,25676053	1,06155237	0,32904677	0,6204092	ATAD3B	ATPase family AAA domain containing 3B [Source:HGNC Symbol;Acc:HGNC:24007]
ENSG00000186715	-0,2063533	1,85004311	1,06127214	0,3291073	0,6204092	MST1L	macrophage stimulating 1 like [Source:HGNC Symbol;Acc:HGNC:7390]
ENSG00000197181	0,47354099	-1,1795945	1,06119522	0,32912392	0,6204092	PIWIL2	piwi like RNA-mediated gene silencing 2 [Source:HGNC Symbol;Acc:HGNC:17644]
ENSG00000226085	-0,3147413	0,08451533	1,06176118	0,32900167	0,6204092	UQCRFS1P1	ubiquinol-cytochrome c reductase, Rieske iron-sulfur polypeptide 1 pseudogene 1 [Source:HGNC Symbol;Acc:HGN
ENSG00000233098	-0,2133206	3,33023865	1,06214363	0,3289191	0,6204092	CCDC144NL-AS1	CCDC144NL antisense RNA 1 [Source:HGNC Symbol;Acc:HGNC:51340]
ENSG00000276900	-0,6273853	-1,7106024	1,06133852	0,32909296	0,6204092	AC023157.2	novel transcript, antisense to AMN1
ENSG00000104738	0,19066705	7,03115264	1,0609862	0,32916909	0,62041429	MCM4	minichromosome maintenance complex component 4 [Source:HGNC Symbol;Acc:HGNC:6947]
ENSG00000158796	0,19362704	3,95750995	1,0597923	0,32942724	0,62082075	DEDD	death effector domain containing [Source:HGNC Symbol;Acc:HGNC:2755]
ENSG00000198221	0,29788438	0,27214546	1,05914075	0,32956824	0,62100638	AFDN-DT	AFDN divergent transcript [Source:HGNC Symbol;Acc:HGNC:21236]
ENSG00000241680	-0,5252489	-1,0848478	1,05669417	0,33009852	0,62192537	RPL31P49	ribosomal protein L31 pseudogene 49 [Source:HGNC Symbol;Acc:HGNC:35909]
ENSG00000112367	0,17913033	2,99657555	1,05627403	0,33018971	0,62193676	FIG4	FIG4 phosphoinositide 5-phosphatase [Source:HGNC Symbol;Acc:HGNC:16873]
ENSG00000162840	-0,4848868	-1,2225131	1,0563455	0,3301742	0,62193676	MT2P1	metallothionein 2 pseudogene 1 [Source:HGNC Symbol;Acc:HGNC:7407]
ENSG00000175895	-0,2595317	2,24555303	1,05592036	0,3302665	0,6220012	PLEKHF2	pleckstrin homology and FYVE domain containing 2 [Source:HGNC Symbol;Acc:HGNC:20757]
ENSG00000101342	-0,4018751	-0,0902037	1,05555518	0,33034582	0,62205262	TLDC2	TBC/LysM-associated domain containing 2 [Source:HGNC Symbol;Acc:HGNC:16112]
ENSG00000259706	-0,3172079	0,04315824	1,05540263	0,33037896	0,62205262	HSP90B2P	heat shock protein 90 beta family member 2, pseudogene [Source:HGNC Symbol;Acc:HGNC:12099]
ENSG00000102595	-0,1921282	4,27952409	1,05386915	0,33071239	0,62244087	UGGT2	UDP-glucose glycoprotein glucosyltransferase 2 [Source:HGNC Symbol;Acc:HGNC:15664]
ENSG00000115129	-0,1358872	5,85203259	1,05367064	0,33075559	0,62244087	TP53I3	tumor protein p53 inducible protein 3 [Source:HGNC Symbol;Acc:HGNC:19373]
ENSG00000128585	-0,2086205	5,3426471	1,05382309	0,33072242	0,62244087	MKLN1	muskelin 1 [Source:HGNC Symbol;Acc:HGNC:7109]
ENSG00000135541	0,175193	2,2696528	1,05389504	0,33070676	0,62244087	AHI1	Abelson helper integration site 1 [Source:HGNC Symbol;Acc:HGNC:21575]
ENSG00000102580	-0,217532	6,55530378	1,05303416	0,33089416	0,6224772	DNAJC3	DnaJ heat shock protein family (Hsp40) member C3 [Source:HGNC Symbol;Acc:HGNC:9439]
ENSG00000131018	0,20341108	2,47583069	1,05280062	0,33094502	0,6224772	SYNE1	spectrin repeat containing nuclear envelope protein 1 [Source:HGNC Symbol;Acc:HGNC:17089]
ENSG00000140474	0,15489745	4,08196562	1,05279918	0,33094534	0,6224772	ULK3	unc-51 like kinase 3 [Source:HGNC Symbol;Acc:HGNC:19703]
ENSG00000179151	0,13881195	5,35633217	1,05314098	0,3308709	0,6224772	EDC3	enhancer of mRNA decapping 3 [Source:HGNC Symbol;Acc:HGNC:26114]
ENSG00000023445	0,44462352	0,01742936	1,05200818	0,3311177	0,62272123	BIRC3	baculoviral IAP repeat containing 3 [Source:HGNC Symbol;Acc:HGNC:591]
ENSG00000174748	-0,1406969	10,1689751	1,05145503	0,33123832	0,62286788	RPL15	ribosomal protein L15 [Source:HGNC Symbol;Acc:HGNC:10306]
ENSG00000165392	0,20409349	2,88935111	1,05103815	0,33132926	0,62295871	WRN	WRN RecQ like helicase [Source:HGNC Symbol;Acc:HGNC:12791]
ENSG00000088992	0,14124697	7,20692194	1,05077134	0,33138748	0,622988	TESC	tescalcin [Source:HGNC Symbol;Acc:HGNC:26065]
ENSG00000185946	0,25629046	1,44512762	1,04901787	0,33177051	0,62359833	RNPC3	RNA binding region (RNP1, RRM) containing 3 [Source:HGNC Symbol;Acc:HGNC:18666]
ENSG00000225873	-0,7116194	-1,7295172	1,0488944	0,33179751	0,62359833	C3orf86	chromosome 3 open reading frame 86 [Source:HGNC Symbol;Acc:HGNC:44570]
ENSG00000119522	0,15835895	5,31335895	1,04821772	0,33194551	0,62371601	DENND1A	DENN domain containing 1A [Source:HGNC Symbol;Acc:HGNC:29324]
ENSG00000163832	-0,1374325	5,56078343	1,04830502	0,33192641	0,62371601	ELP6	elongator acetyltransferase complex subunit 6 [Source:HGNC Symbol;Acc:HGNC:25976]
ENSG00000204619	-0,2039015	4,46843185	1,04721601	0,33216479	0,62396752	PPP1R11	protein phosphatase 1 regulatory inhibitor subunit 11 [Source:HGNC Symbol;Acc:HGNC:9285]
ENSG00000213711	0,4735894	-1,4488498	1,04729511	0,33214747	0,62396752	PHBP7	prohibitin pseudogene 7 [Source:HGNC Symbol;Acc:HGNC:39286]
ENSG00000132837	0,22127134	1,57419512	1,04695025	0,332223	0,62399664	DMGDH	dimethylglycine dehydrogenase [Source:HGNC Symbol;Acc:HGNC:24475]
ENSG00000160789	-0,2315249	8,27960947	1,04584168	0,33246599	0,62437274	LMNA	lamin A/C [Source:HGNC Symbol;Acc:HGNC:6636]
ENSG00000257727	-0,2272174	4,14084737	1,04469761	0,33271703	0,62476389	CNPY2	canopy FGF signaling regulator 2 [Source:HGNC Symbol;Acc:HGNC:13529]
ENSG00000065883	-0,1630785	4,05100144	1,04329061	0,33302614	0,62494999	CDK13	cyclin dependent kinase 13 [Source:HGNC Symbol;Acc:HGNC:1733]
ENSG00000068796	0,16064558	5,2007373	1,04191902	0,33332789	0,62494999	KIF2A	kinesin family member 2A [Source:HGNC Symbol;Acc:HGNC:6318]
ENSG00000073111	0,21105376	7,31074975	1,04397849	0,33287496	0,62494999	MCM2	minichromosome maintenance complex component 2 [Source:HGNC Symbol;Acc:HGNC:6944]
ENSG00000073417	0,16491421	4,27878278	1,04279097	0,33313602	0,62494999	PDE8A	phosphodiesterase 8A [Source:HGNC Symbol;Acc:HGNC:8793]
ENSG00000101901	-0,1622849	3,98644739	1,04197162	0,33331631	0,62494999	ALG13	ALG13 UDP-N-acetylglucosaminyltransferase subunit [Source:HGNC Symbol;Acc:HGNC:30881]
ENSG00000126243	0,1687546	2,94254238	1,04254831	0,3331894	0,62494999	LRFN3	leucine rich repeat and fibronectin type III domain containing 3 [Source:HGNC Symbol;Acc:HGNC:28370]
ENSG00000137812	-0,2438212	2,32506777	1,04224588	0,33325595	0,62494999	KNL1	kinetochore scaffold 1 [Source:HGNC Symbol;Acc:HGNC:24054]

ENSG00000144645	-0,1941609	4,77770401	1,04343567	0,33299426	0,62494999	OSBPL10	oxysterol binding protein like 10 [Source:HGNC Symbol;Acc:HGNC:16395]
ENSG00000187118	-0,1935429	3,77892839	1,04191183	0,33332948	0,62494999	CMC1	C-X9-C motif containing 1 [Source:HGNC Symbol;Acc:HGNC:28783]
ENSG00000196378	-0,2338726	1,53319737	1,04249308	0,33320155	0,62494999	ZNF34	zinc finger protein 34 [Source:HGNC Symbol;Acc:HGNC:13098]
ENSG00000197563	0,19860346	4,29197451	1,04291428	0,3331089	0,62494999	PIGN	phosphatidylinositol glycan anchor biosynthesis class N [Source:HGNC Symbol;Acc:HGNC:8967]
ENSG00000213066	-0,1735054	3,20721023	1,04207272	0,33329406	0,62494999	CEP43	centrosomal protein 43 [Source:HGNC Symbol;Acc:HGNC:17012]
ENSG00000108433	0,14575661	5,10341951	1,04129717	0,33346483	0,62496315	GOSR2	golgi SNAP receptor complex member 2 [Source:HGNC Symbol;Acc:HGNC:4431]
ENSG00000134480	0,16349765	6,35778308	1,04143815	0,33343378	0,62496315	CCNH	cyclin H [Source:HGNC Symbol;Acc:HGNC:1594]
ENSG00000228300	-0,1392344	6,88945476	1,04158026	0,33340248	0,62496315	FAM174C	family with sequence similarity 174 member C [Source:HGNC Symbol;Acc:HGNC:26073]
ENSG00000167257	0,15218491	4,15875854	1,04044494	0,33365264	0,62499441	RNF214	ring finger protein 214 [Source:HGNC Symbol;Acc:HGNC:25335]
ENSG00000171425	-0,14878	5,14624219	1,04086329	0,33356043	0,62499441	ZNF581	zinc finger protein 581 [Source:HGNC Symbol;Acc:HGNC:25017]
ENSG00000181751	0,26838937	1,10854553	1,04044626	0,33365235	0,62499441	MACIR	macrophage immunometabolism regulator [Source:HGNC Symbol;Acc:HGNC:25052]
ENSG00000182973	0,15321004	4,34187276	1,04082495	0,33356888	0,62499441	CNOT10	CCR4-NOT transcription complex subunit 10 [Source:HGNC Symbol;Acc:HGNC:23817]
ENSG00000100583	-0,6383094	-1,5304765	1,04015669	0,3337162	0,62502074	SAMD15	sterile alpha motif domain containing 15 [Source:HGNC Symbol;Acc:HGNC:18631]
ENSG00000235621	-0,6826101	-0,6457372	1,0399243	0,33376745	0,62502074	LINC00494	long intergenic non-protein coding RNA 494 [Source:HGNC Symbol;Acc:HGNC:27657]
ENSG00000236533	-0,2589246	0,53938319	1,03979921	0,33379505	0,62502074	RPS12P4	ribosomal protein S12 pseudogene 4 [Source:HGNC Symbol;Acc:HGNC:35670]
ENSG00000183655	0,16129661	3,58579795	1,03902659	0,33396555	0,62525987	KLHL25	kelch like family member 25 [Source:HGNC Symbol;Acc:HGNC:25732]
ENSG00000162419	-0,1786858	3,11298156	1,03849529	0,33408288	0,62539938	GMEB1	glucocorticoid modulatory element binding protein 1 [Source:HGNC Symbol;Acc:HGNC:4370]
ENSG00000105127	-0,2099469	5,11452156	1,03783645	0,33422845	0,62551159	AKAP8	A-kinase anchoring protein 8 [Source:HGNC Symbol;Acc:HGNC:378]
ENSG00000148908	-0,3275449	1,18529806	1,03796994	0,33419895	0,62551159	RGS10	regulator of G protein signaling 10 [Source:HGNC Symbol;Acc:HGNC:9992]
ENSG00000103876	0,14158948	7,02561639	1,03762338	0,33427555	0,6255196	FAH	fumarylacetoacetate hydrolase [Source:HGNC Symbol;Acc:HGNC:3579]
ENSG00000088876	0,15900212	4,47443257	1,036312	0,33456565	0,6258731	ZNF343	zinc finger protein 343 [Source:HGNC Symbol;Acc:HGNC:16017]
ENSG00000140326	0,17868169	4,57812669	1,03618854	0,33459298	0,6258731	CDAN1	codanin 1 [Source:HGNC Symbol;Acc:HGNC:1713]
ENSG00000229091	-0,2556116	1,17646454	1,03653163	0,33451704	0,6258731	HSPA8P8	heat shock protein family A (Hsp70) member 8 pseudogene 8 [Source:HGNC Symbol;Acc:HGNC:44923]
ENSG00000059145	-0,1664691	3,85081893	1,03570312	0,33470047	0,62599401	UNKL	unk like zinc finger [Source:HGNC Symbol;Acc:HGNC:14184]
ENSG00000135018	0,16279267	6,50880755	1,03411148	0,33505328	0,62610396	UBQLN1	ubiquilin 1 [Source:HGNC Symbol;Acc:HGNC:12508]
ENSG00000159322	0,15723588	6,46943207	1,03492028	0,33487393	0,62610396	ADPGK	ADP dependent glucokinase [Source:HGNC Symbol;Acc:HGNC:25250]
ENSG00000198300	0,34879107	7,39050873	1,03480323	0,33489988	0,62610396	PEG3	paternally expressed 3 [Source:HGNC Symbol;Acc:HGNC:8826]
ENSG00000227766	-0,4679828	-1,5016653	1,03408451	0,33505926	0,62610396	AL671277.1	HLA complex group 4 pseudogene 5
ENSG00000249673	-0,2448545	1,79080036	1,03423688	0,33502546	0,62610396	NOP14-AS1	NOP14 antisense RNA 1 [Source:HGNC Symbol;Acc:HGNC:20205]
ENSG00000254860	-0,5226299	-1,0864425	1,03414076	0,33504679	0,62610396	TMEM9B-AS1	TMEM9B antisense RNA 1 [Source:HGNC Symbol;Acc:HGNC:19230]
ENSG00000265401	-0,4288235	-0,9483571	1,03517882	0,33481663	0,62610396	AC093484.3	novel transcript
ENSG00000117228	0,33914562	1,35268318	1,03336901	0,33521805	0,62632056	GBP1	guanylate binding protein 1 [Source:HGNC Symbol;Acc:HGNC:4182]
ENSG00000175899	-0,3103054	11,8173884	1,03260716	0,33538724	0,62655655	A2M	alpha-2-macroglobulin [Source:HGNC Symbol;Acc:HGNC:7]
ENSG00000147262	-0,46217	-0,9720769	1,03204726	0,33551167	0,62662873	GPR119	G protein-coupled receptor 119 [Source:HGNC Symbol;Acc:HGNC:19060]
ENSG00000198406	0,24385145	0,88163025	1,03209019	0,33550212	0,62662873	BZW1P2	basic leucine zipper and W2 domains 1 pseudogene 2 [Source:HGNC Symbol;Acc:HGNC:33954]
ENSG00000100982	0,15200096	5,79779172	1,03114388	0,33571256	0,6266303	PCIF1	PDX1 C-terminal inhibiting factor 1 [Source:HGNC Symbol;Acc:HGNC:16200]
ENSG00000119707	0,32758039	5,30004657	1,03158869	0,33561362	0,6266303	RBM25	RNA binding motif protein 25 [Source:HGNC Symbol;Acc:HGNC:23244]
ENSG00000133624	0,23232044	1,84848692	1,03107911	0,33572697	0,6266303	ZNF767P	zinc finger family member 767, pseudogene [Source:HGNC Symbol;Acc:HGNC:21884]
ENSG00000169926	0,17596366	3,84444075	1,0317929	0,33556821	0,6266303	KLF13	Kruppel like factor 13 [Source:HGNC Symbol;Acc:HGNC:13672]
ENSG00000177025	0,49480014	-0,6589843	1,03112397	0,33571699	0,6266303	C19orf18	chromosome 19 open reading frame 18 [Source:HGNC Symbol;Acc:HGNC:28642]
ENSG00000280239	-0,5301669	-1,1770915	1,03045039	0,33586691	0,6268114	AC011498.7	TEC
ENSG00000078124	-0,1760717	2,91961953	1,02884728	0,3362241	0,62705452	ACER3	alkaline ceramidase 3 [Source:HGNC Symbol;Acc:HGNC:16066]
ENSG00000108551	-0,2995481	4,08766778	1,0295862	0,33605939	0,62705452	RASD1	ras related dexamethasone induced 1 [Source:HGNC Symbol;Acc:HGNC:15828]
ENSG00000137038	-0,1778611	6,25899116	1,0291583	0,33615476	0,62705452	DMAC1	distal membrane arm assembly complex 1 [Source:HGNC Symbol;Acc:HGNC:30536]

ENSG00000143450	0,56436892	-1,0884265	1,0288554	0,33622229	0,62705452	OAZ3	ornithine decarboxylase antizyme 3 [Source:HGNC Symbol;Acc:HGNC:8097]
ENSG00000145555	0,2864147	4,67694572	1,02871	0,33625472	0,62705452	MYO10	myosin X [Source:HGNC Symbol;Acc:HGNC:7593]
ENSG00000234337	0,43209471	-0,7680483	1,0288441	0,33622481	0,62705452	AC026462.1	tropomyosin 1 (alpha) (TPM1) pseudogene
ENSG00000269119	0,48944871	-1,502769	1,02835327	0,33633429	0,62712286	HNRNPA1P52	heterogeneous nuclear ribonucleoprotein A1 pseudogene 52 [Source:HGNC Symbol;Acc:HGNC:48782]
ENSG00000182544	0,1691048	5,77475571	1,02711536	0,33661063	0,62747795	MFSD5	major facilitator superfamily domain containing 5 [Source:HGNC Symbol;Acc:HGNC:28156]
ENSG00000263080	0,58556098	-0,747066	1,02727383	0,33657523	0,62747795	AC009121.3	novel transcript, antisense to RMI2
ENSG00000071537	0,22811935	6,08479753	1,02573701	0,33691872	0,6278904	SEL1L	SEL1L adaptor subunit of ERAD E3 ubiquitin ligase [Source:HGNC Symbol;Acc:HGNC:10717]
ENSG00000139233	-0,1564587	3,90699584	1,02534209	0,33700707	0,6278904	LLPH	LLP homolog, long-term synaptic facilitation factor [Source:HGNC Symbol;Acc:HGNC:28229]
ENSG00000155542	0,18747734	2,46145853	1,0255182	0,33696766	0,6278904	SETD9	SET domain containing 9 [Source:HGNC Symbol;Acc:HGNC:28508]
ENSG00000156374	-0,1619006	3,79137119	1,0253697	0,33700089	0,6278904	PCGF6	polycomb group ring finger 6 [Source:HGNC Symbol;Acc:HGNC:21156]
ENSG00000168303	-0,1620471	5,70702062	1,02516461	0,33704679	0,6278904	MPLKIP	M-phase specific PLK1 interacting protein [Source:HGNC Symbol;Acc:HGNC:16002]
ENSG00000260552	-0,5075803	-1,1516776	1,02465117	0,33716172	0,62802443	AC023043.1	novel transcript, antisense to MOCOS
ENSG00000101096	0,32647915	2,42945204	1,02368181	0,33737888	0,62826871	NFATC2	nuclear factor of activated T cells 2 [Source:HGNC Symbol;Acc:HGNC:7776]
ENSG00000177576	0,41609352	-1,025284	1,02381324	0,33734942	0,62826871	C18orf32	chromosome 18 open reading frame 32 [Source:HGNC Symbol;Acc:HGNC:31690]
ENSG00000150753	-0,1872389	9,03732894	1,02340389	0,33744118	0,62830463	CCT5	chaperonin containing TCP1 subunit 5 [Source:HGNC Symbol;Acc:HGNC:1618]
ENSG00000204628	-0,1456335	11,1717291	1,02228502	0,33769215	0,62869181	RACK1	receptor for activated C kinase 1 [Source:HGNC Symbol;Acc:HGNC:4399]
ENSG00000110880	-0,1689235	7,48529974	1,0218058	0,33779973	0,62869415	CORO1C	coronin 1C [Source:HGNC Symbol;Acc:HGNC:2254]
ENSG00000223460	-0,4803916	-0,9976442	1,02207748	0,33773873	0,62869415	GAPDHP69	glyceraldehyde 3 phosphate dehydrogenase pseudogene 69 [Source:HGNC Symbol;Acc:HGNC:4147]
ENSG00000274605	-0,1797968	2,40896207	1,02170436	0,33782251	0,62869415	PCCA-DT	PCCA divergent transcript [Source:HGNC Symbol;Acc:HGNC:53266]
ENSG00000168393	-0,1497293	7,05117133	1,02004476	0,3381955	0,62929164	DTYMK	deoxythymidylate kinase [Source:HGNC Symbol;Acc:HGNC:3061]
ENSG00000205861	-0,4678013	-1,5041927	1,01989265	0,33822972	0,62929164	PCOTH	Pro-X-Gly collagen triple helix like repeat containing [Source:HGNC Symbol;Acc:HGNC:39839]
ENSG00000111540	0,1973719	4,2382478	1,01882037	0,33847108	0,62964733	RAB5B	RAB5B, member RAS oncogene family [Source:HGNC Symbol;Acc:HGNC:9784]
ENSG00000176340	-0,161898	7,07744403	1,01866044	0,3385071	0,62964733	COX8A	cytochrome c oxidase subunit 8A [Source:HGNC Symbol;Acc:HGNC:2294]
ENSG00000113658	0,15959163	3,7648202	1,01818754	0,33861364	0,62976533	SMAD5	SMAD family member 5 [Source:HGNC Symbol;Acc:HGNC:6771]
ENSG00000189091	0,20889432	8,30584239	1,01796689	0,33866337	0,62977764	SF3B3	splicing factor 3b subunit 3 [Source:HGNC Symbol;Acc:HGNC:10770]
ENSG00000236081	-0,2428158	1,88902961	1,01761554	0,33874257	0,62984476	ELFN1-AS1	ELFN1 antisense RNA 1 [Source:HGNC Symbol;Acc:HGNC:39071]
ENSG00000134330	0,13739583	5,25416324	1,01740744	0,3387895	0,62985185	IAH1	isoamyl acetate hydrolyzing esterase 1 (putative) [Source:HGNC Symbol;Acc:HGNC:27696]
ENSG00000284968	-0,361665	-0,5011541	1,01678959	0,33892888	0,6300308	AC093827.4	Novel transcript, antisense to AFF1
ENSG00000133275	0,16629962	5,57189699	1,01589107	0,33913173	0,63012267	CSNK1G2	casein kinase 1 gamma 2 [Source:HGNC Symbol;Acc:HGNC:2455]
ENSG00000149428	-0,3912094	8,93325424	1,01580927	0,33915021	0,63012267	HYOU1	hypoxia up-regulated 1 [Source:HGNC Symbol;Acc:HGNC:16931]
ENSG00000225416	-0,3667543	-0,404099	1,0158172	0,33914842	0,63012267	AC104843.1	ribosomal protein L36a (RPL36A) pseudogene
ENSG00000272892	0,62007565	-1,528646	1,01580648	0,33915084	0,63012267	AL133551.1	novel transcript
ENSG00000005175	0,20536653	4,52064141	1,01522006	0,33928334	0,63018717	RPAP3	RNA polymerase II associated protein 3 [Source:HGNC Symbol;Acc:HGNC:26151]
ENSG00000100644	-0,1598007	6,15204657	1,01262184	0,33987132	0,63018717	HIF1A	hypoxia inducible factor 1 subunit alpha [Source:HGNC Symbol;Acc:HGNC:4910]
ENSG00000105643	0,16668774	3,17373767	1,01242343	0,33991628	0,63018717	ARRDC2	arrestin domain containing 2 [Source:HGNC Symbol;Acc:HGNC:25225]
ENSG00000111676	0,32688619	4,25088264	1,0116581	0,3400898	0,63018717	ATN1	atrophin 1 [Source:HGNC Symbol;Acc:HGNC:3033]
ENSG00000134265	-0,1620588	4,40260271	1,01288848	0,33981091	0,63018717	NAPG	NSF attachment protein gamma [Source:HGNC Symbol;Acc:HGNC:7642]
ENSG00000159884	-0,1388333	5,1527738	1,0117503	0,34006889	0,63018717	CCDC107	coiled-coil domain containing 107 [Source:HGNC Symbol;Acc:HGNC:28465]
ENSG00000167283	-0,1764574	4,73919943	1,01176945	0,34006455	0,63018717	ATP5MG	ATP synthase membrane subunit g [Source:HGNC Symbol;Acc:HGNC:14247]
ENSG00000168234	-0,1986317	3,77767095	1,01391676	0,33957809	0,63018717	TTC39C	tetratricopeptide repeat domain 39C [Source:HGNC Symbol;Acc:HGNC:26595]
ENSG00000169032	0,14007113	5,88459013	1,01179692	0,34005832	0,63018717	MAP2K1	mitogen-activated protein kinase kinase 1 [Source:HGNC Symbol;Acc:HGNC:6840]
ENSG00000179152	-0,1842736	3,16698449	1,01270904	0,33985156	0,63018717	TCAIM	T cell activation inhibitor, mitochondrial [Source:HGNC Symbol;Acc:HGNC:25241]
ENSG00000188986	0,13779841	6,48117092	1,01257063	0,33988293	0,63018717	NELFB	negative elongation factor complex member B [Source:HGNC Symbol;Acc:HGNC:24324]
ENSG00000189241	-0,1864963	6,08772117	1,01181219	0,34005486	0,63018717	TSPYL1	TSPY like 1 [Source:HGNC Symbol;Acc:HGNC:12382]

ENSG00000196954	0,15212687	5,80888476	1,01377748	0,33960961	0,63018717	CASP4	caspase 4 [Source:HGNC Symbol;Acc:HGNC:1505]
ENSG00000215908	-0,1705987	4,42360554	1,01295429	0,339796	0,63018717	CROCCP2	CROCC pseudogene 2 [Source:HGNC Symbol;Acc:HGNC:28170]
ENSG00000227199	-0,292696	0,68588081	1,01143846	0,34013963	0,63018717	ST7-AS1	ST7 antisense RNA 1 [Source:HGNC Symbol;Acc:HGNC:16000]
ENSG00000228502	-0,1881618	3,25761316	1,01473798	0,33939232	0,63018717	EEF1A1P11	eukaryotic translation elongation factor 1 alpha 1 pseudogene 11 [Source:HGNC Symbol;Acc:HGNC:3206]
ENSG00000229944	0,47125587	-1,3456545	1,01135552	0,34015844	0,63018717	EIF4EP2	eukaryotic translation initiation factor 4E pseudogene 2 [Source:HGNC Symbol;Acc:HGNC:32428]
ENSG00000237039	-0,4805796	-1,1439333	1,01284961	0,33981972	0,63018717	RPS28P4	ribosomal protein S28 pseudogene 4 [Source:HGNC Symbol;Acc:HGNC:36208]
ENSG00000249858	-0,3320558	-0,1816781	1,01331477	0,33971436	0,63018717	SNX5P1	sorting nexin 5 pseudogene 1 [Source:HGNC Symbol;Acc:HGNC:41512]
ENSG00000250012	0,78289521	-2,0259155	1,01294562	0,33979797	0,63018717	AC079848.1	novel transcript
ENSG00000267374	-0,3230746	0,48293202	1,01499107	0,3393351	0,63018717	MIR924HG	MIR924 host gene [Source:HGNC Symbol;Acc:HGNC:44332]
ENSG00000272501	-0,2400007	0,95745177	1,01127065	0,3401777	0,63018717	AL662844.4	novel transcript, antisense to HCG27
ENSG00000285427	-0,5219017	-1,0826769	1,0122582	0,33995373	0,63018717	SOD2-OT1	SOD2 overlapping transcript 1 [Source:HGNC Symbol;Acc:HGNC:53445]
ENSG00000260668	0,63845276	-2,0637619	1,00927236	0,34063156	0,63094795	AC093536.1	emopamil binding protein-like (EBPL) pseudogene
ENSG00000134072	0,13396482	6,01043505	1,0084908	0,34080932	0,63116229	CAMK1	calcium/calmodulin dependent protein kinase I [Source:HGNC Symbol;Acc:HGNC:1459]
ENSG00000226084	-0,1849709	2,25287309	1,0083833	0,34083378	0,63116229	AC113935.1	ribosomal protein L17 (RPL17) pseudogene
ENSG00000231831	0,47630246	-1,47483	1,00819385	0,34087689	0,63116229	MTHFD1P1	methylenetetrahydrofolate dehydrogenase (NADP+ dependent) 1 pseudogene 1 [Source:HGNC Symbol;Acc:HGNC
ENSG00000159593	0,14537238	6,1427606	1,00685746	0,34118125	0,63159544	NAE1	NEDD8 activating enzyme E1 subunit 1 [Source:HGNC Symbol;Acc:HGNC:621]
ENSG00000175104	0,18288412	2,49192566	1,00678706	0,34119729	0,63159544	TRAF6	TNF receptor associated factor 6 [Source:HGNC Symbol;Acc:HGNC:12036]
ENSG00000169756	0,16325428	4,71032198	1,00570031	0,34144511	0,63197411	LIMS1	LIM zinc finger domain containing 1 [Source:HGNC Symbol;Acc:HGNC:6616]
ENSG00000111206	0,17894619	6,04573822	1,00514708	0,34157137	0,63212771	FOXM1	forkhead box M1 [Source:HGNC Symbol;Acc:HGNC:3818]
ENSG00000250838	-0,7372656	-1,5395731	1,00424159	0,34177818	0,63243032	AC091133.3	novel transcript
ENSG00000001617	-0,1424455	4,71723219	1,00377772	0,34188419	0,63254637	SEMA3F	semaphorin 3F [Source:HGNC Symbol;Acc:HGNC:10728]
ENSG00000011638	-0,2769078	1,38912288	1,00346358	0,34195602	0,63259914	TMEM159	transmembrane protein 159 [Source:HGNC Symbol;Acc:HGNC:30136]
ENSG00000031691	0,1719192	3,37127857	1,00237822	0,34220434	0,63263459	CENPQ	centromere protein Q [Source:HGNC Symbol;Acc:HGNC:21347]
ENSG00000113845	-0,2361388	6,07674317	1,00292785	0,34207855	0,63263459	TIMMDC1	translocase of inner mitochondrial membrane domain containing 1 [Source:HGNC Symbol;Acc:HGNC:1321]
ENSG00000138642	0,31788233	-0,176997	1,00263917	0,34214461	0,63263459	HERC6	HECT and RLD domain containing E3 ubiquitin protein ligase family member 6 [Source:HGNC Symbol;Acc:HGNC:2
ENSG00000166825	0,19929466	8,33499559	1,00224427	0,342235	0,63263459	ANPEP	alanyl aminopeptidase, membrane [Source:HGNC Symbol;Acc:HGNC:500]
ENSG00000181467	-0,2334391	2,45399532	1,00248004	0,34218103	0,63263459	RAP2B	RAP2B, member of RAS oncogene family [Source:HGNC Symbol;Acc:HGNC:9862]
ENSG00000198538	-0,2828889	0,90588252	1,00253472	0,34216852	0,63263459	ZNF28	zinc finger protein 28 [Source:HGNC Symbol;Acc:HGNC:13073]
ENSG00000034152	-0,1736209	6,61326105	1,00160794	0,34238074	0,63277104	MAP2K3	mitogen-activated protein kinase kinase 3 [Source:HGNC Symbol;Acc:HGNC:6843]
ENSG00000145743	0,22733325	1,77374725	1,00154375	0,34239544	0,63277104	FBXL17	F-box and leucine rich repeat protein 17 [Source:HGNC Symbol;Acc:HGNC:13615]
ENSG00000162227	0,18195634	3,85273625	1,00112263	0,34249195	0,63286932	TAF6L	TATA-box binding protein associated factor 6 like [Source:HGNC Symbol;Acc:HGNC:17305]
ENSG00000089876	0,17520174	4,19231588	1,00083851	0,34255708	0,63290962	DHX32	DEAH-box helicase 32 (putative) [Source:HGNC Symbol;Acc:HGNC:16717]
ENSG00000129472	0,16644045	2,7934798	1,00009305	0,34272805	0,63298846	RAB2B	RAB2B, member RAS oncogene family [Source:HGNC Symbol;Acc:HGNC:20246]
ENSG00000147601	0,17256418	4,85398793	1,00035411	0,34266817	0,63298846	TERF1	telomeric repeat binding factor 1 [Source:HGNC Symbol;Acc:HGNC:11728]
ENSG00000153989	0,18304187	5,60349112	0,99984141	0,3427858	0,63298846	NUS1	NUS1 dehydrodolichyl diphosphate synthase subunit [Source:HGNC Symbol;Acc:HGNC:21042]
ENSG00000171045	-0,1975296	2,46390586	0,9997081	0,3428164	0,63298846	TSNARE1	t-SNARE domain containing 1 [Source:HGNC Symbol;Acc:HGNC:26437]
ENSG00000173064	0,31697204	3,29715384	1,00001288	0,34274645	0,63298846	HECTD4	HECT domain E3 ubiquitin protein ligase 4 [Source:HGNC Symbol;Acc:HGNC:26611]
ENSG00000196998	-0,1575457	3,44615604	0,99905458	0,34296645	0,63318549	WDR45	WD repeat domain 45 [Source:HGNC Symbol;Acc:HGNC:28912]
ENSG00000083844	0,20600537	2,23519589	0,99840299	0,34311615	0,63330182	ZNF264	zinc finger protein 264 [Source:HGNC Symbol;Acc:HGNC:13057]
ENSG00000176714	0,30693697	0,57006732	0,99853052	0,34308684	0,63330182	CCDC121	coiled-coil domain containing 121 [Source:HGNC Symbol;Acc:HGNC:25833]
ENSG00000271851	0,53806542	-1,6681445	0,99786957	0,34323878	0,63344812	AC087501.4	novel transcript, sense intronic to STX8
ENSG00000160563	0,16411873	5,16667271	0,99764544	0,34329033	0,63346323	MED27	mediator complex subunit 27 [Source:HGNC Symbol;Acc:HGNC:2377]
ENSG00000218891	-0,174595	2,84287925	0,99726099	0,34337877	0,6335464	ZNF579	zinc finger protein 579 [Source:HGNC Symbol;Acc:HGNC:26646]
ENSG00000160326	-0,1501136	5,21699233	0,99659061	0,34353307	0,63375106	SLC2A6	solute carrier family 2 member 6 [Source:HGNC Symbol;Acc:HGNC:11011]

ENSG00000234488	-0,7005751	-1,8663773	0,99606961	0,34365306	0,63389237	RPL27P6	ribosomal protein L27 pseudogene 6 [Source:HGNC Symbol;Acc:HGNC:36330]
ENSG00000253669	-0,3361291	0,60611947	0,99547957	0,34378903	0,63406311	GASAL1	growth arrest associated lncRNA 1 [Source:HGNC Symbol;Acc:HGNC:53461]
ENSG00000113318	0,16215768	3,60410006	0,99485804	0,34393234	0,63408727	MSH3	mutS homolog 3 [Source:HGNC Symbol;Acc:HGNC:7326]
ENSG00000116754	0,18343981	5,47368205	0,99487334	0,34392881	0,63408727	SRSF11	serine and arginine rich splicing factor 11 [Source:HGNC Symbol;Acc:HGNC:10782]
ENSG00000197530	-0,1689129	3,92613597	0,99507273	0,34388282	0,63408727	MIB2	mindbomb E3 ubiquitin protein ligase 2 [Source:HGNC Symbol;Acc:HGNC:30577]
ENSG00000104365	0,16280558	3,75735621	0,99409302	0,34410886	0,63433265	IKBKB	inhibitor of nuclear factor kappa B kinase subunit beta [Source:HGNC Symbol;Acc:HGNC:5960]
ENSG00000162139	-0,1731111	3,23787508	0,99307492	0,34434398	0,63452588	NEU3	neuraminidase 3 [Source:HGNC Symbol;Acc:HGNC:7760]
ENSG00000204387	0,21383749	3,34756075	0,99342907	0,34426216	0,63452588	SNHG32	small nucleolar RNA host gene 32 [Source:HGNC Symbol;Acc:HGNC:19078]
ENSG00000260852	-0,5877246	-0,1775487	0,99324384	0,34430495	0,63452588	FBXL19-AS1	FBXL19 antisense RNA 1 [Source:HGNC Symbol;Acc:HGNC:27557]
ENSG00000182165	-0,1912839	3,03942102	0,99276023	0,3444167	0,63457984	TP53TG1	TP53 target 1 [Source:HGNC Symbol;Acc:HGNC:17026]
ENSG00000230555	-0,406094	-0,7356842	0,99205628	0,34457946	0,63479967	AL450326.1	novel transcript
ENSG00000076604	0,1312665	7,2292176	0,99159168	0,34468695	0,63491284	TRAF4	TNF receptor associated factor 4 [Source:HGNC Symbol;Acc:HGNC:12034]
ENSG00000132424	-0,1839706	4,11465708	0,99141511	0,34472781	0,63491284	PNISR	PNN interacting serine and arginine rich protein [Source:HGNC Symbol;Acc:HGNC:21222]
ENSG00000113716	0,15816636	6,10016966	0,99101843	0,34481964	0,63500191	HMGXB3	HMG-box containing 3 [Source:HGNC Symbol;Acc:HGNC:28982]
ENSG00000172534	0,42430403	5,74646967	0,9903067	0,34498449	0,63522542	HCFC1	host cell factor C1 [Source:HGNC Symbol;Acc:HGNC:4839]
ENSG00000161526	-0,132299	6,61136486	0,99010499	0,34503123	0,63523142	SAP30BP	SAP30 binding protein [Source:HGNC Symbol;Acc:HGNC:30785]
ENSG00000204315	-0,2042003	3,28075361	0,98886078	0,34531976	0,63568251	FKBPL	FKBP prolyl isomerase like [Source:HGNC Symbol;Acc:HGNC:13949]
ENSG00000100304	-0,1890578	5,68546076	0,98725128	0,34569353	0,6361301	TTLL12	tubulin tyrosine ligase like 12 [Source:HGNC Symbol;Acc:HGNC:28974]
ENSG00000115307	-0,1632806	9,16489556	0,98751338	0,34563263	0,6361301	AUP1	AUP1 lipid droplet regulating VLDL assembly factor [Source:HGNC Symbol;Acc:HGNC:891]
ENSG00000150867	-0,1898499	3,4055902	0,98733368	0,34567439	0,6361301	PIP4K2A	phosphatidylinositol-5-phosphate 4-kinase type 2 alpha [Source:HGNC Symbol;Acc:HGNC:8997]
ENSG00000148339	0,17154407	5,44770897	0,9866817	0,34582595	0,63629362	SLC25A25	solute carrier family 25 member 25 [Source:HGNC Symbol;Acc:HGNC:20663]
ENSG00000268129	-0,3568285	0,9662873	0,98623343	0,34593022	0,63640532	AC026304.1	novel transcript
ENSG00000100523	0,2036232	2,78977714	0,9850729	0,34620038	0,63653121	DDHD1	DDHD domain containing 1 [Source:HGNC Symbol;Acc:HGNC:19714]
ENSG00000111275	0,18707104	7,56416622	0,98522295	0,34616544	0,63653121	ALDH2	aldehyde dehydrogenase 2 family member [Source:HGNC Symbol;Acc:HGNC:404]
ENSG00000117308	-0,1497252	6,58358764	0,98520326	0,34617002	0,63653121	GALE	UDP-galactose-4-epimerase [Source:HGNC Symbol;Acc:HGNC:4116]
ENSG00000156976	0,13769198	8,3066584	0,9856197	0,34607305	0,63653121	EIF4A2	eukaryotic translation initiation factor 4A2 [Source:HGNC Symbol;Acc:HGNC:3284]
ENSG00000198265	-0,2706382	3,8476151	0,98500368	0,34621651	0,63653121	HELZ	helicase with zinc finger [Source:HGNC Symbol;Acc:HGNC:16878]
ENSG00000141425	-0,1387677	4,82117502	0,98409614	0,34642801	0,63683992	RPRD1A	regulation of nuclear pre-mRNA domain containing 1A [Source:HGNC Symbol;Acc:HGNC:25560]
ENSG00000182326	-0,1567002	3,37101332	0,98376184	0,34650597	0,63690308	C1S	complement C1s [Source:HGNC Symbol;Acc:HGNC:1247]
ENSG00000125944	0,14691894	7,88697742	0,98243804	0,34681493	0,63731061	HNRNPR	heterogeneous nuclear ribonucleoprotein R [Source:HGNC Symbol;Acc:HGNC:5047]
ENSG00000236682	-0,2426908	2,09996069	0,9825048	0,34679934	0,63731061	MAP3K2-DT	MAP3K2 divergent transcript [Source:HGNC Symbol;Acc:HGNC:54088]
ENSG00000196405	-0,2040604	3,57142706	0,98191475	0,34693718	0,63745507	EVL	Enah/Vasp-like [Source:HGNC Symbol;Acc:HGNC:20234]
ENSG00000115738	0,14867077	7,47060905	0,98089254	0,34717616	0,63781396	ID2	inhibitor of DNA binding 2 [Source:HGNC Symbol;Acc:HGNC:5361]
ENSG00000139734	0,2049915	3,08889011	0,98064819	0,34723333	0,63783877	DIAPH3	diaphanous related formin 3 [Source:HGNC Symbol;Acc:HGNC:15480]
ENSG00000083168	0,32898902	3,97036918	0,98043323	0,34728363	0,63785096	KAT6A	lysine acetyltransferase 6A [Source:HGNC Symbol;Acc:HGNC:13013]
ENSG00000231663	-0,3639313	-0,5682313	0,98001012	0,34738267	0,63795266	COA6-AS1	COA6 antisense RNA 1 [Source:HGNC Symbol;Acc:HGNC:40825]
ENSG00000011426	0,21507709	6,19423182	0,97931274	0,347546	0,63803183	ANLN	anillin actin binding protein [Source:HGNC Symbol;Acc:HGNC:14082]
ENSG00000078246	-0,1830069	3,95053468	0,97933839	0,34753999	0,63803183	TULP3	TUB like protein 3 [Source:HGNC Symbol;Acc:HGNC:12425]
ENSG00000178199	-0,239971	1,03666518	0,97926662	0,3475568	0,63803183	ZC3H12D	zinc finger CCCH-type containing 12D [Source:HGNC Symbol;Acc:HGNC:21175]
ENSG00000188549	0,25835714	0,65773798	0,9787657	0,3476742	0,63816693	CCDC9B	coiled-coil domain containing 9B [Source:HGNC Symbol;Acc:HGNC:33488]
ENSG00000229207	0,49430223	-1,7398794	0,97857987	0,34771776	0,63816693	SERPINH1P1	serpin family H member 1 pseudogene 1 [Source:HGNC Symbol;Acc:HGNC:19917]
ENSG00000144283	-0,1322506	5,78023571	0,97783579	0,34789229	0,638345	PKP4	plakophilin 4 [Source:HGNC Symbol;Acc:HGNC:9026]
ENSG00000158411	-0,1905468	4,4217049	0,97771711	0,34792013	0,638345	MITD1	microtubule interacting and trafficking domain containing 1 [Source:HGNC Symbol;Acc:HGNC:25207]
ENSG00000179889	0,14966836	6,87031034	0,97740082	0,34799437	0,638345	PDXDC1	pyridoxal dependent decarboxylase domain containing 1 [Source:HGNC Symbol;Acc:HGNC:28995]

ENSG00000198734	0,28299107	5,96893414	0,97723514	0,34803326	0,638345	F5	coagulation factor V [Source:HGNC Symbol;Acc:HGNC:3542]
ENSG00000229167	0,7798133	-2,2923688	0,97752842	0,34796442	0,638345	AC114488.1	novel transcript
ENSG00000076555	0,18125255	2,41918757	0,97612571	0,34829388	0,63847243	ACACB	acetyl-CoA carboxylase beta [Source:HGNC Symbol;Acc:HGNC:85]
ENSG00000101343	0,18711315	6,1835361	0,97658239	0,34818657	0,63847243	CRNKL1	crooked neck pre-mRNA splicing factor 1 [Source:HGNC Symbol;Acc:HGNC:15762]
ENSG00000104427	-0,2316546	1,00004219	0,97603302	0,34831567	0,63847243	ZC2HC1A	zinc finger C2HC-type containing 1A [Source:HGNC Symbol;Acc:HGNC:24277]
ENSG00000230457	-0,30248	0,38477282	0,97600924	0,34832126	0,63847243	PA2G4P4	proliferation-associated 2G4 pseudogene 4 [Source:HGNC Symbol;Acc:HGNC:32217]
ENSG00000259943	0,1983266	2,00000417	0,97635662	0,34823962	0,63847243	AL050341.2	novel transcript, antisense to ZMPSTE24
ENSG00000259974	0,18075685	5,42764801	0,9750883	0,34853785	0,63878929	LINC00261	long intergenic non-protein coding RNA 261 [Source:HGNC Symbol;Acc:HGNC:16189]
ENSG00000130054	0,18414199	3,24079946	0,9743147	0,34871994	0,6389627	FAM155B	family with sequence similarity 155 member B [Source:HGNC Symbol;Acc:HGNC:30701]
ENSG00000162928	0,14498432	6,03951601	0,9743775	0,34870515	0,6389627	PEX13	peroxisomal biogenesis factor 13 [Source:HGNC Symbol;Acc:HGNC:8855]
ENSG00000182511	-0,1493656	4,86438696	0,97365122	0,34887623	0,6391689	FES	FES proto-oncogene, tyrosine kinase [Source:HGNC Symbol;Acc:HGNC:3657]
ENSG00000174977	-0,239012	1,61834198	0,97337471	0,34894139	0,63920813	AC026271.1	poly(A) binding protein interacting protein 1 (PAIP1) pseudogene
ENSG00000142252	-0,1787278	5,07600851	0,97293374	0,34904535	0,63929934	GEMIN7	gem nuclear organelle associated protein 7 [Source:HGNC Symbol;Acc:HGNC:20045]
ENSG00000163131	0,4546052	-0,7664898	0,9727923	0,34907871	0,63929934	CTSS	cathepsin S [Source:HGNC Symbol;Acc:HGNC:2545]
ENSG00000103275	-0,1324159	7,74571231	0,97053698	0,34961121	0,63948608	UBE2I	ubiquitin conjugating enzyme E2 I [Source:HGNC Symbol;Acc:HGNC:12485]
ENSG00000107882	0,2266177	3,58937239	0,9710693	0,34948542	0,63948608	SUFU	SUFU negative regulator of hedgehog signaling [Source:HGNC Symbol;Acc:HGNC:16466]
ENSG00000113810	0,14277537	5,34859636	0,97090363	0,34952456	0,63948608	SMC4	structural maintenance of chromosomes 4 [Source:HGNC Symbol;Acc:HGNC:14013]
ENSG00000119878	0,16022893	4,53508318	0,97032139	0,34966218	0,63948608	CRIPT	CXXC repeat containing interactor of PDZ3 domain [Source:HGNC Symbol;Acc:HGNC:14312]
ENSG00000125841	-0,1683248	6,45589365	0,97098431	0,3495055	0,63948608	NRSN2	neurensin 2 [Source:HGNC Symbol;Acc:HGNC:16229]
ENSG00000166391	-0,1539338	3,73778538	0,97078827	0,34955182	0,63948608	MOGAT2	monoacylglycerol O-acyltransferase 2 [Source:HGNC Symbol;Acc:HGNC:23248]
ENSG00000167513	0,15601397	6,12357182	0,97046422	0,34962841	0,63948608	CDT1	chromatin licensing and DNA replication factor 1 [Source:HGNC Symbol;Acc:HGNC:24576]
ENSG00000172269	-0,1393812	5,71917905	0,97044799	0,34963225	0,63948608	DPAGT1	dolichyl-phosphate N-acetylglucosaminephosphotransferase 1 [Source:HGNC Symbol;Acc:HGNC:2995]
ENSG00000180061	-0,4627204	-1,4994272	0,97096653	0,3495097	0,63948608	TMEM150B	transmembrane protein 150B [Source:HGNC Symbol;Acc:HGNC:34415]
ENSG00000216867	0,36338324	-0,4286443	0,97090572	0,34952407	0,63948608	RPL22P12	ribosomal protein L22 pseudogene 12 [Source:HGNC Symbol;Acc:HGNC:36345]
ENSG00000270959	-0,5150707	-0,9998621	0,97083046	0,34954185	0,63948608	LPP-AS2	LPP antisense RNA 2 [Source:HGNC Symbol;Acc:HGNC:27952]
ENSG00000069956	0,13457509	6,3889789	0,96992376	0,34975621	0,63954984	MAPK6	mitogen-activated protein kinase 6 [Source:HGNC Symbol;Acc:HGNC:6879]
ENSG00000100413	-0,1564321	5,56618708	0,96958011	0,34983751	0,63954984	POLR3H	RNA polymerase III subunit H [Source:HGNC Symbol;Acc:HGNC:30349]
ENSG00000174469	-0,4080585	-0,2564604	0,96944191	0,34987021	0,63954984	CNTNAP2	contactin associated protein 2 [Source:HGNC Symbol;Acc:HGNC:13830]
ENSG00000198478	0,1609865	3,76796566	0,9694337	0,34987215	0,63954984	SH3BGRL2	SH3 domain binding glutamate rich protein like 2 [Source:HGNC Symbol;Acc:HGNC:15567]
ENSG00000230716	-0,3672255	-0,6922763	0,96865184	0,35005725	0,63980813	KRT8P7	keratin 8 pseudogene 7 [Source:HGNC Symbol;Acc:HGNC:33361]
ENSG00000166887	0,16248695	4,61697595	0,96824552	0,3501535	0,63990399	VPS39	VPS39 subunit of HOPS complex [Source:HGNC Symbol;Acc:HGNC:20593]
ENSG00000167280	0,16989648	3,12131472	0,96769391	0,35028424	0,64006284	ENGASE	endo-beta-N-acetylglucosaminidase [Source:HGNC Symbol;Acc:HGNC:24622]
ENSG00000226666	0,31610075	0,62336724	0,96750811	0,35032829	0,64006327	HSPA9P1	heat shock protein family A (Hsp70) member 9 pseudogene 1 [Source:HGNC Symbol;Acc:HGNC:24915]
ENSG00000067955	0,20469997	4,44158708	0,96710014	0,35042504	0,64015999	CBFB	core-binding factor subunit beta [Source:HGNC Symbol;Acc:HGNC:1539]
ENSG00000132275	-0,1390261	4,69260679	0,96616171	0,35064775	0,64032662	RRP8	ribosomal RNA processing 8 [Source:HGNC Symbol;Acc:HGNC:29030]
ENSG00000198034	-0,1323921	10,513433	0,966167	0,3506465	0,64032662	RPS4X	ribosomal protein S4 X-linked [Source:HGNC Symbol;Acc:HGNC:10424]
ENSG00000239264	0,18511426	2,50513731	0,96640495	0,35059	0,64032662	TXNDC5	thioredoxin domain containing 5 [Source:HGNC Symbol;Acc:HGNC:21073]
ENSG00000162144	-0,1596822	3,9049538	0,96517764	0,35088152	0,64066109	CYB561A3	cytochrome b561 family member A3 [Source:HGNC Symbol;Acc:HGNC:23014]
ENSG00000214018	0,4953069	-1,7075039	0,96502159	0,35091861	0,64066109	RRM2P3	ribonucleotide reductase M2 polypeptide pseudogene 3 [Source:HGNC Symbol;Acc:HGNC:10455]
ENSG00000113460	0,13624993	6,25288333	0,96236805	0,35155025	0,64117563	BRIX1	biogenesis of ribosomes BRX1 [Source:HGNC Symbol;Acc:HGNC:24170]
ENSG00000140386	0,24522591	1,92954118	0,96342197	0,35129917	0,64117563	SCAPER	S-phase cyclin A associated protein in the ER [Source:HGNC Symbol;Acc:HGNC:13081]
ENSG00000142186	-0,1616284	6,90618915	0,96322561	0,35134593	0,64117563	SCYL1	SCY1 like pseudokinase 1 [Source:HGNC Symbol;Acc:HGNC:14372]
ENSG00000158805	-0,1774282	3,94415363	0,96199435	0,35163934	0,64117563	ZNF276	zinc finger protein 276 [Source:HGNC Symbol;Acc:HGNC:23330]
ENSG00000167772	-0,3416906	-0,3685676	0,9621657	0,35159849	0,64117563	ANGPTL4	angiopoietin like 4 [Source:HGNC Symbol;Acc:HGNC:16039]

ENSG00000171469	0,14981618	4,07378541	0,96199478	0,35163924	0,64117563	ZNF561	zinc finger protein 561 [Source:HGNC Symbol;Acc:HGNC:28684]
ENSG00000173473	0,29942785	6,31182947	0,9626177	0,35149075	0,64117563	SMARCC1	SWI/SNF related, matrix associated, actin dependent regulator of chromatin subfamily c member 1 [Source:HGNC
ENSG00000228509	0,54672446	-0,9017197	0,96272178	0,35146595	0,64117563	AC006460.1	novel transcript, antisense to NAB1
ENSG00000236144	0,24046323	1,7430031	0,96221267	0,35158729	0,64117563	TMEM147-AS1	TMEM147 antisense RNA 1 [Source:HGNC Symbol;Acc:HGNC:51273]
ENSG00000279274	-0,281663	0,16262451	0,96202518	0,35163199	0,64117563	AC012005.1	ribosomal protein L41 (RPL41) pseudogene
ENSG00000162961	0,16981986	5,22354882	0,96180205	0,3516852	0,64117922	DPY30	dpy-30 histone methyltransferase complex regulatory subunit [Source:HGNC Symbol;Acc:HGNC:24590]
ENSG00000149948	0,1638704	5,27695899	0,96157739	0,35173879	0,6411969	HMGA2	high mobility group AT-hook 2 [Source:HGNC Symbol;Acc:HGNC:5009]
ENSG00000112852	0,25962106	3,18923929	0,96066116	0,35195746	0,64148786	PCDHB2	protocadherin beta 2 [Source:HGNC Symbol;Acc:HGNC:8687]
ENSG00000121680	0,14027313	4,53209939	0,96046108	0,35200523	0,64148786	PEX16	peroxisomal biogenesis factor 16 [Source:HGNC Symbol;Acc:HGNC:8857]
ENSG00000125618	-0,2452051	1,6538178	0,96018799	0,35207046	0,64148786	PAX8	paired box 8 [Source:HGNC Symbol;Acc:HGNC:8622]
ENSG00000129696	0,14632959	3,95047752	0,95998922	0,35211795	0,64148786	TTI2	TELO2 interacting protein 2 [Source:HGNC Symbol;Acc:HGNC:26262]
ENSG00000143486	0,12987367	5,97401013	0,9600549	0,35210226	0,64148786	EIF2D	eukaryotic translation initiation factor 2D [Source:HGNC Symbol;Acc:HGNC:6583]
ENSG00000198830	0,17977654	7,05001727	0,95886206	0,35238743	0,64189874	HMGN2	high mobility group nucleosomal binding domain 2 [Source:HGNC Symbol;Acc:HGNC:4986]
ENSG00000170759	-0,1705677	6,89432879	0,95863415	0,35244195	0,64191803	KIF5B	kinesin family member 5B [Source:HGNC Symbol;Acc:HGNC:6324]
ENSG00000197568	-0,3203184	0,87823586	0,95844438	0,35248736	0,64192071	HHLA3	HERV-H LTR-associating 3 [Source:HGNC Symbol;Acc:HGNC:4906]
ENSG00000177888	0,40092382	-0,092823	0,95773905	0,35265622	0,64214817	ZBTB41	zinc finger and BTB domain containing 41 [Source:HGNC Symbol;Acc:HGNC:24819]
ENSG00000225193	0,33528016	-0,4623577	0,95703354	0,35282525	0,64237587	RPS12P26	ribosomal protein S12 pseudogene 26 [Source:HGNC Symbol;Acc:HGNC:36320]
ENSG00000063245	-0,1886355	6,14688928	0,95548606	0,35319641	0,64277604	EPN1	epsin 1 [Source:HGNC Symbol;Acc:HGNC:21604]
ENSG00000089234	0,15141864	4,90838035	0,9553835	0,35322103	0,64277604	BRAP	BRCA1 associated protein [Source:HGNC Symbol;Acc:HGNC:1099]
ENSG00000097033	-0,1417723	6,25096862	0,95558008	0,35317384	0,64277604	SH3GLB1	SH3 domain containing GRB2 like, endophilin B1 [Source:HGNC Symbol;Acc:HGNC:10833]
ENSG00000125834	0,22368679	5,36724636	0,9554624	0,35320209	0,64277604	STK35	serine/threonine kinase 35 [Source:HGNC Symbol;Acc:HGNC:16254]
ENSG00000155975	-0,1501717	4,34019626	0,95501089	0,3533105	0,64285877	VPS37A	VPS37A subunit of ESCRT-I [Source:HGNC Symbol;Acc:HGNC:24928]
ENSG00000119812	-0,1723304	5,57687197	0,95457736	0,35341464	0,64296818	FAM98A	family with sequence similarity 98 member A [Source:HGNC Symbol;Acc:HGNC:24520]
ENSG00000112981	-0,4421306	-1,2523886	0,95360691	0,35364792	0,64331247	NME5	NME/NM23 family member 5 [Source:HGNC Symbol;Acc:HGNC:7853]
ENSG00000077463	-0,1706225	4,27370464	0,95296338	0,35380274	0,64339371	SIRT6	sirtuin 6 [Source:HGNC Symbol;Acc:HGNC:14934]
ENSG00000101220	-0,1723491	7,0009242	0,95277463	0,35384817	0,64339371	C20orf27	chromosome 20 open reading frame 27 [Source:HGNC Symbol;Acc:HGNC:15873]
ENSG00000132801	-0,1959986	2,92252848	0,95268918	0,35386874	0,64339371	ZSWIM3	zinc finger SWIM-type containing 3 [Source:HGNC Symbol;Acc:HGNC:16157]
ENSG00000176907	-0,1566755	5,57938572	0,95270001	0,35386614	0,64339371	TCIM	transcriptional and immune response regulator [Source:HGNC Symbol;Acc:HGNC:1357]
ENSG00000171444	-0,4329181	0,11483927	0,95236314	0,35394725	0,64345636	MCC	MCC regulator of WNT signaling pathway [Source:HGNC Symbol;Acc:HGNC:6935]
ENSG00000238172	-0,4518185	-1,4079981	0,95212159	0,35400542	0,64348204	RPS2P35	ribosomal protein S2 pseudogene 35 [Source:HGNC Symbol;Acc:HGNC:36757]
ENSG00000204388	-0,4694358	4,70136396	0,9518467	0,35407164	0,64352235	HSPA1B	heat shock protein family A (Hsp70) member 1B [Source:HGNC Symbol;Acc:HGNC:5233]
ENSG00000131069	-0,1497709	6,99282003	0,95039171	0,35442247	0,64391965	ACSS2	acyl-CoA synthetase short chain family member 2 [Source:HGNC Symbol;Acc:HGNC:15814]
ENSG00000164587	-0,1481173	10,0062211	0,9504429	0,35441012	0,64391965	RPS14	ribosomal protein S14 [Source:HGNC Symbol;Acc:HGNC:10387]
ENSG00000241764	-0,3130739	0,62536632	0,95067516	0,35435409	0,64391965	AC002467.1	novel transcript, antisense to CBLL1
ENSG00000136877	0,15143088	6,25879301	0,94993813	0,35453195	0,64403845	FPGS	folylpolyglutamate synthase [Source:HGNC Symbol;Acc:HGNC:3824]
ENSG00000118181	-0,1641203	9,69408944	0,94964598	0,35460249	0,6440865	RPS25	ribosomal protein S25 [Source:HGNC Symbol;Acc:HGNC:10413]
ENSG00000105371	0,18808821	3,57632705	0,94945874	0,35464771	0,64408856	ICAM4	intercellular adhesion molecule 4 (Landsteiner-Wiener blood group) [Source:HGNC Symbol;Acc:HGNC:5347]
ENSG00000229719	-0,5942771	-1,7821794	0,94831222	0,35492479	0,64443156	MIR194-2HG	MIR194-2 host gene [Source:HGNC Symbol;Acc:HGNC:51946]
ENSG00000250251	0,36128042	0,79099378	0,94837695	0,35490914	0,64443156	PKD1P6	polycystin 1, transient receptor potential channel interacting pseudogene 6 [Source:HGNC Symbol;Acc:HGNC:300
ENSG00000057608	0,13721504	8,66105358	0,94576062	0,35554263	0,64473089	GDI2	GDP dissociation inhibitor 2 [Source:HGNC Symbol;Acc:HGNC:4227]
ENSG00000100024	-0,3425692	0,31014889	0,94595829	0,35549471	0,64473089	UPB1	beta-ureidopropionase 1 [Source:HGNC Symbol;Acc:HGNC:16297]
ENSG00000106052	0,2181652	6,79856465	0,94619733	0,35543677	0,64473089	TAX1BP1	Tax1 binding protein 1 [Source:HGNC Symbol;Acc:HGNC:11575]
ENSG00000112977	0,13275454	6,66520374	0,94622374	0,35543037	0,64473089	DAP	death associated protein [Source:HGNC Symbol;Acc:HGNC:2672]
ENSG00000131351	0,14016184	4,49733781	0,94501015	0,35572465	0,64473089	HAUS8	HAUS augmin like complex subunit 8 [Source:HGNC Symbol;Acc:HGNC:30532]

ENSG00000137692	-0,1623644	5,50442606	0,94556253	0,35559066	0,64473089	DCUN1D5	defective in cullin neddylation 1 domain containing 5 [Source:HGNC Symbol;Acc:HGNC:28409]
ENSG00000138443	-0,1660663	5,02560707	0,94567274	0,35556394	0,64473089	ABI2	abl interactor 2 [Source:HGNC Symbol;Acc:HGNC:24011]
ENSG00000143995	-0,2815626	0,32674414	0,94621855	0,35543163	0,64473089	MEIS1	Meis homeobox 1 [Source:HGNC Symbol;Acc:HGNC:7000]
ENSG00000148225	-0,3734332	-0,7988394	0,94734085	0,35515981	0,64473089	WDR31	WD repeat domain 31 [Source:HGNC Symbol;Acc:HGNC:21421]
ENSG00000186017	0,24493634	1,02922604	0,94494112	0,35574141	0,64473089	ZNF566	zinc finger protein 566 [Source:HGNC Symbol;Acc:HGNC:25919]
ENSG00000196081	0,39470859	-0,2039739	0,94521558	0,35567481	0,64473089	ZNF724	zinc finger protein 724 [Source:HGNC Symbol;Acc:HGNC:32460]
ENSG00000196604	-0,2623391	0,96377964	0,94684507	0,35527984	0,64473089	POTEF	POTE ankyrin domain family member F [Source:HGNC Symbol;Acc:HGNC:33905]
ENSG00000213673	-0,4666606	-1,5692563	0,94489893	0,35575164	0,64473089	SLC25A5P3	solute carrier family 25 member 5 pseudogene 3 [Source:HGNC Symbol;Acc:HGNC:507]
ENSG00000246582	0,25221482	0,90753779	0,94664013	0,35532948	0,64473089	AC100861.1	novel transcript, antisense to TNFRSF10A
ENSG00000272888	0,16813288	2,73692761	0,94647582	0,35536929	0,64473089	CHASERR	CHD2 adjacent suppressive regulatory RNA [Source:HGNC Symbol;Acc:HGNC:48626]
ENSG00000064393	0,59946108	4,43192572	0,94441612	0,35586884	0,64479846	HIPK2	homeodomain interacting protein kinase 2 [Source:HGNC Symbol;Acc:HGNC:14402]
ENSG00000135249	0,18182835	4,0060524	0,94438169	0,3558772	0,64479846	RINT1	RAD50 interactor 1 [Source:HGNC Symbol;Acc:HGNC:21876]
ENSG00000254170	-0,2667772	0,4085022	0,94332399	0,35613415	0,64518399	AC008802.1	ribosomal protein S3A (RPS3A) pseudogene
ENSG00000133872	-0,1365757	8,12018061	0,94255083	0,35632215	0,64540655	SARAF	store-operated calcium entry associated regulatory factor [Source:HGNC Symbol;Acc:HGNC:28789]
ENSG00000151276	0,24689502	4,22723891	0,94245544	0,35634535	0,64540655	MAGI1	membrane associated guanylate kinase, WW and PDZ domain containing 1 [Source:HGNC Symbol;Acc:HGNC:946]
ENSG00000171848	-0,1330184	8,1956598	0,94140095	0,35660203	0,64571134	RRM2	ribonucleotide reductase regulatory subunit M2 [Source:HGNC Symbol;Acc:HGNC:10452]
ENSG00000227969	-0,5041881	-1,9971074	0,94144799	0,35659058	0,64571134	NPM1P22	nucleophosmin 1 pseudogene 22 [Source:HGNC Symbol;Acc:HGNC:39679]
ENSG00000117481	0,18036783	5,35757866	0,94089282	0,35672582	0,64585544	NSUN4	NOP2/Sun RNA methyltransferase 4 [Source:HGNC Symbol;Acc:HGNC:31802]
ENSG00000181991	-0,1345401	6,19367507	0,94054665	0,35681019	0,64592814	MRPS11	mitochondrial ribosomal protein S11 [Source:HGNC Symbol;Acc:HGNC:14050]
ENSG00000234176	-0,2158942	1,91223881	0,9401031	0,35691834	0,64604386	HSPA8P1	heat shock protein family A (Hsp70) member 8 pseudogene 1 [Source:HGNC Symbol;Acc:HGNC:5245]
ENSG00000258512	-0,5914327	-1,4505628	0,939763	0,35700129	0,64611396	LINC00239	long intergenic non-protein coding RNA 239 [Source:HGNC Symbol;Acc:HGNC:20119]
ENSG00000114541	0,42107562	-0,0046109	0,9387673	0,35724433	0,64640026	FRMD4B	FERM domain containing 4B [Source:HGNC Symbol;Acc:HGNC:24886]
ENSG00000280649	-0,3648796	-0,2577796	0,93875236	0,35724798	0,64640026	AC245100.7	TEC
ENSG00000163827	0,71564184	-1,2694714	0,93841466	0,35733047	0,64643285	LRRC2	leucine rich repeat containing 2 [Source:HGNC Symbol;Acc:HGNC:14676]
ENSG00000204253	-0,2084529	2,06767656	0,93831635	0,35735449	0,64643285	HNRNPCP2	heterogeneous nuclear ribonucleoprotein C pseudogene 2 [Source:HGNC Symbol;Acc:HGNC:48814]
ENSG00000104946	-0,1587867	4,39097092	0,93755897	0,35753961	0,64660759	TBC1D17	TBC1 domain family member 17 [Source:HGNC Symbol;Acc:HGNC:25699]
ENSG00000171843	0,24674683	2,43369245	0,93770858	0,35750303	0,64660759	MLLT3	MLLT3 super elongation complex subunit [Source:HGNC Symbol;Acc:HGNC:7136]
ENSG00000143303	0,14858382	4,44862636	0,93736706	0,35758654	0,64661242	RRNAD1	ribosomal RNA adenine dimethylase domain containing 1 [Source:HGNC Symbol;Acc:HGNC:24273]
ENSG00000168781	0,23875622	1,405691	0,93680433	0,3577242	0,6467813	PPIP5K1	diphosphoinositol pentakisphosphate kinase 1 [Source:HGNC Symbol;Acc:HGNC:29023]
ENSG00000100350	0,2224584	6,34646798	0,93537012	0,35807543	0,64704398	FOXRED2	FAD dependent oxidoreductase domain containing 2 [Source:HGNC Symbol;Acc:HGNC:26264]
ENSG00000118508	-0,1587625	5,9226138	0,93530684	0,35809094	0,64704398	RAB32	RAB32, member RAS oncogene family [Source:HGNC Symbol;Acc:HGNC:9772]
ENSG00000120798	0,14429632	4,51268199	0,93549836	0,35804401	0,64704398	NR2C1	nuclear receptor subfamily 2 group C member 1 [Source:HGNC Symbol;Acc:HGNC:7971]
ENSG00000146066	-0,1636962	6,29043921	0,93557628	0,35802491	0,64704398	HIGD2A	HIG1 hypoxia inducible domain family member 2A [Source:HGNC Symbol;Acc:HGNC:28311]
ENSG00000213995	-0,1690498	4,65196884	0,93531063	0,35809001	0,64704398	NAXD	NAD(P)HX dehydratase [Source:HGNC Symbol;Acc:HGNC:25576]
ENSG00000113558	0,17476708	7,43979664	0,93489737	0,35819132	0,64706529	SKP1	S-phase kinase associated protein 1 [Source:HGNC Symbol;Acc:HGNC:10899]
ENSG00000115234	0,14795139	7,8683022	0,93492003	0,35818576	0,64706529	SNX17	sorting nexin 17 [Source:HGNC Symbol;Acc:HGNC:14979]
ENSG00000123146	-0,260481	2,50484956	0,93425087	0,35834989	0,64711169	ADGRE5	adhesion G protein-coupled receptor E5 [Source:HGNC Symbol;Acc:HGNC:1711]
ENSG00000142675	0,16205308	2,55164813	0,93457071	0,35827143	0,64711169	CNKSR1	connector enhancer of kinase suppressor of Ras 1 [Source:HGNC Symbol;Acc:HGNC:19700]
ENSG00000166813	-0,3163046	3,14442726	0,93431359	0,3583345	0,64711169	KIF7	kinesin family member 7 [Source:HGNC Symbol;Acc:HGNC:30497]
ENSG00000131504	0,25206553	5,70046659	0,93332871	0,35857626	0,64744044	DIAPH1	diaphanous related formin 1 [Source:HGNC Symbol;Acc:HGNC:2876]
ENSG00000165028	0,71623224	-1,2373859	0,93287939	0,35868664	0,64747968	NIPSNAP3B	nipsnap homolog 3B [Source:HGNC Symbol;Acc:HGNC:23641]
ENSG00000277791	-0,1249293	8,82361256	0,93303853	0,35864754	0,64747968	PSMB3	proteasome 20S subunit beta 3 [Source:HGNC Symbol;Acc:HGNC:9540]
ENSG00000081041	-0,6484219	-0,1920786	0,9324107	0,35880183	0,64750216	CXCL2	C-X-C motif chemokine ligand 2 [Source:HGNC Symbol;Acc:HGNC:4603]
ENSG00000169750	-0,1973191	2,17225282	0,93228774	0,35883206	0,64750216	RAC3	Rac family small GTPase 3 [Source:HGNC Symbol;Acc:HGNC:9803]

ENSG00000230551	0,3557155	-0,1649279	0,93262573	0,35874897	0,64750216	AC021078.1	novel transcript
ENSG00000103091	0,132612	6,15984473	0,93080467	0,35919697	0,64800055	WDR59	WD repeat domain 59 [Source:HGNC Symbol;Acc:HGNC:25706]
ENSG00000256594	0,49557133	-0,1395675	0,93091154	0,35917065	0,64800055	AC010186.2	C-type lectin domain family 2 (CLEC2) pseudogene
ENSG00000242553	-0,3958599	-1,0212538	0,93041628	0,35929262	0,64809309	AP001432.1	novel transcript, antisense to DSCR3
ENSG00000124608	0,17722539	5,15380393	0,92969785	0,35946967	0,64825236	AARS2	alanyl-tRNA synthetase 2, mitochondrial [Source:HGNC Symbol;Acc:HGNC:21022]
ENSG00000160446	0,14495276	5,88614878	0,92970061	0,35946899	0,64825236	ZDHHC12	zinc finger DHHC-type palmitoyltransferase 12 [Source:HGNC Symbol;Acc:HGNC:19159]
ENSG00000062716	0,14854324	7,84111641	0,92685091	0,36017255	0,64846858	VMP1	vacuole membrane protein 1 [Source:HGNC Symbol;Acc:HGNC:29559]
ENSG00000111615	0,13066587	6,04378057	0,92756955	0,35999493	0,64846858	KRR1	KRR1 small subunit processome component homolog [Source:HGNC Symbol;Acc:HGNC:5176]
ENSG00000119431	-0,1648395	4,35754911	0,92881095	0,35968841	0,64846858	HDHD3	haloacid dehalogenase like hydrolase domain containing 3 [Source:HGNC Symbol;Acc:HGNC:28171]
ENSG00000122335	0,22277135	2,86794934	0,92657715	0,36024025	0,64846858	SERAC1	serine active site containing 1 [Source:HGNC Symbol;Acc:HGNC:21061]
ENSG00000126267	-0,1561199	7,85580937	0,92665045	0,36022212	0,64846858	COX6B1	cytochrome c oxidase subunit 6B1 [Source:HGNC Symbol;Acc:HGNC:2280]
ENSG00000129292	0,18129112	3,06017408	0,92759541	0,35998854	0,64846858	PHF20L1	PHD finger protein 20 like 1 [Source:HGNC Symbol;Acc:HGNC:24280]
ENSG00000156521	0,13949098	4,04595373	0,92817712	0,35984486	0,64846858	TYSND1	trypsin domain containing 1 [Source:HGNC Symbol;Acc:HGNC:28531]
ENSG00000157734	-0,3138063	0,16651402	0,92776182	0,35994743	0,64846858	SNX22	sorting nexin 22 [Source:HGNC Symbol;Acc:HGNC:16315]
ENSG00000163002	0,14517563	5,07252348	0,92864395	0,35972962	0,64846858	NUP35	nucleoporin 35 [Source:HGNC Symbol;Acc:HGNC:29797]
ENSG00000169564	-0,1305252	9,3210261	0,92650578	0,3602579	0,64846858	PCBP1	poly(rC) binding protein 1 [Source:HGNC Symbol;Acc:HGNC:8647]
ENSG00000182054	0,14868241	7,29619629	0,92628185	0,3603133	0,64846858	IDH2	isocitrate dehydrogenase (NADP(+)) 2 [Source:HGNC Symbol;Acc:HGNC:5383]
ENSG00000184708	0,15437396	4,37104278	0,9265289	0,36025218	0,64846858	EIF4ENIF1	eukaryotic translation initiation factor 4E nuclear import factor 1 [Source:HGNC Symbol;Acc:HGNC:16687]
ENSG00000196262	-0,1429378	10,7635134	0,9286842	0,35971969	0,64846858	PPIA	peptidylprolyl isomerase A [Source:HGNC Symbol;Acc:HGNC:9253]
ENSG00000196873	0,26481793	0,58684947	0,92762255	0,35998183	0,64846858	CBWD3	COBW domain containing 3 [Source:HGNC Symbol;Acc:HGNC:18519]
ENSG00000204682	-0,2088404	2,76191811	0,92784428	0,35992706	0,64846858	MIR1915HG	MIR1915 host gene [Source:HGNC Symbol;Acc:HGNC:31448]
ENSG00000225630	0,15069525	4,47302189	0,92689886	0,3601607	0,64846858	MTND2P28	MT-ND2 pseudogene 28 [Source:HGNC Symbol;Acc:HGNC:42129]
ENSG00000281344	-0,6827773	-1,8394644	0,92615708	0,36034417	0,64846858	HELLPAR	HELLP associated long non-coding RNA [Source:HGNC Symbol;Acc:HGNC:43984]
ENSG00000250966	-0,4330558	-1,0438906	0,92589464	0,36040911	0,64850557	H3P14	H3 histone pseudogene 14 [Source:HGNC Symbol;Acc:HGNC:43805]
ENSG00000108774	-0,2027084	5,86103325	0,92523477	0,36057249	0,64871964	RAB5C	RAB5C, member RAS oncogene family [Source:HGNC Symbol;Acc:HGNC:9785]
ENSG00000125827	-0,1309428	5,17923644	0,92430079	0,36080393	0,64897772	TMX4	thioredoxin related transmembrane protein 4 [Source:HGNC Symbol;Acc:HGNC:25237]
ENSG00000135297	0,13847203	5,18330404	0,92429735	0,36080478	0,64897772	MTO1	mitochondrial tRNA translation optimization 1 [Source:HGNC Symbol;Acc:HGNC:19261]
ENSG00000135919	0,23198169	5,53002916	0,92400136	0,36087817	0,64901622	SERPINE2	serpin family E member 2 [Source:HGNC Symbol;Acc:HGNC:8951]
ENSG00000170604	-0,1471742	4,11047665	0,9235756	0,36098378	0,64901622	IRF2BP1	interferon regulatory factor 2 binding protein 1 [Source:HGNC Symbol;Acc:HGNC:21728]
ENSG00000198837	0,14061097	4,6087268	0,92349455	0,36100389	0,64901622	DENND4B	DENN domain containing 4B [Source:HGNC Symbol;Acc:HGNC:29044]
ENSG00000234450	-0,4166448	-0,9306877	0,92373884	0,36094328	0,64901622	AC005534.1	ribosomal protein L26-like 1 (RPL26L1) pseudogene
ENSG00000139597	0,25006696	1,76373375	0,9229812	0,3611313	0,64916539	N4BP2L1	NEDD4 binding protein 2 like 1 [Source:HGNC Symbol;Acc:HGNC:25037]
ENSG00000164087	-0,1867353	5,15930511	0,92247031	0,36125817	0,64931355	POC1A	POC1 centriolar protein A [Source:HGNC Symbol;Acc:HGNC:24488]
ENSG00000173638	0,14112318	5,7035013	0,922122	0,3613447	0,64933094	SLC19A1	solute carrier family 19 member 1 [Source:HGNC Symbol;Acc:HGNC:10937]
ENSG00000182318	-0,2808483	1,26085591	0,92207356	0,36135674	0,64933094	ZSCAN22	zinc finger and SCAN domain containing 22 [Source:HGNC Symbol;Acc:HGNC:4929]
ENSG00000121966	0,48950852	-0,6015128	0,92155719	0,36148509	0,64946414	CXCR4	C-X-C motif chemokine receptor 4 [Source:HGNC Symbol;Acc:HGNC:2561]
ENSG00000214050	-0,2159995	1,08845986	0,92141768	0,36151977	0,64946414	FBXO16	F-box protein 16 [Source:HGNC Symbol;Acc:HGNC:13618]
ENSG00000106992	-0,2500395	0,53272569	0,92080391	0,36167245	0,6494744	AK1	adenylate kinase 1 [Source:HGNC Symbol;Acc:HGNC:361]
ENSG00000168297	-0,218519	1,95301226	0,9211556	0,36158495	0,6494744	PXK	PX domain containing serine/threonine kinase like [Source:HGNC Symbol;Acc:HGNC:23326]
ENSG00000181284	0,23222673	1,54346481	0,92090354	0,36164766	0,6494744	TMEM102	transmembrane protein 102 [Source:HGNC Symbol;Acc:HGNC:26722]
ENSG00000236816	0,32545972	-0,0810178	0,92067985	0,36170332	0,6494744	ANKRD20A7P	ankyrin repeat domain 20 family member A7, pseudogene [Source:HGNC Symbol;Acc:HGNC:31980]
ENSG00000101190	-0,1554024	3,44315181	0,9196692	0,36195496	0,64955191	TCFL5	transcription factor like 5 [Source:HGNC Symbol;Acc:HGNC:11646]
ENSG00000137509	0,16462156	7,27023084	0,91993775	0,36188807	0,64955191	PRCP	prolylcarboxypeptidase [Source:HGNC Symbol;Acc:HGNC:9344]
ENSG00000169231	-0,5973661	-0,7501757	0,91960871	0,36197003	0,64955191	THBS3	thrombospondin 3 [Source:HGNC Symbol;Acc:HGNC:11787]

ENSG00000205937	0,15445576	7,6431981	0,9194352	0,36201326	0,64955191	RNPS1	RNA binding protein with serine rich domain 1 [Source:HGNC Symbol;Acc:HGNC:10080]
ENSG00000254827	-0,3841638	-0,9565234	0,91945315	0,36200879	0,64955191	SLC22A18AS	solute carrier family 22 member 18 antisense [Source:HGNC Symbol;Acc:HGNC:10965]
ENSG00000258153	0,29843737	-0,0420465	0,91959593	0,36197321	0,64955191	HSPE1P4	heat shock protein family E (Hsp10) member 1 pseudogene 4 [Source:HGNC Symbol;Acc:HGNC:49323]
ENSG00000138777	-0,1405278	5,08075643	0,91914926	0,36208452	0,64959999	PPA2	inorganic pyrophosphatase 2 [Source:HGNC Symbol;Acc:HGNC:28883]
ENSG00000003400	-0,1670189	4,27985396	0,91884696	0,36215988	0,64961202	CASP10	caspase 10 [Source:HGNC Symbol;Acc:HGNC:1500]
ENSG00000012983	0,14422475	3,86523385	0,91858734	0,36222462	0,64961202	MAP4K5	mitogen-activated protein kinase kinase kinase kinase 5 [Source:HGNC Symbol;Acc:HGNC:6867]
ENSG00000253522	0,38551229	0,14465163	0,91875758	0,36218217	0,64961202	MIR3142HG	MIR3142 host gene [Source:HGNC Symbol;Acc:HGNC:51944]
ENSG00000143443	-0,1696302	3,11951846	0,91829903	0,36229654	0,64962872	C1orf56	chromosome 1 open reading frame 56 [Source:HGNC Symbol;Acc:HGNC:26045]
ENSG00000277702	-0,3237524	-0,0481589	0,91819348	0,36232287	0,64962872	AC239859.5	lymphocyte-specific protein 1 (LSP1) pseudogene
ENSG00000085063	0,12454093	6,96238934	0,917203	0,36257013	0,64998612	CD59	CD59 molecule (CD59 blood group) [Source:HGNC Symbol;Acc:HGNC:1689]
ENSG00000139372	-0,1683418	4,09637741	0,91703862	0,36261119	0,64998612	TDG	thymine DNA glycosylase [Source:HGNC Symbol;Acc:HGNC:11700]
ENSG00000100425	0,1817056	4,15759819	0,91622145	0,36281542	0,65025912	BRD1	bromodomain containing 1 [Source:HGNC Symbol;Acc:HGNC:1102]
ENSG00000214199	-0,172576	2,87691406	0,91607308	0,36285251	0,65025912	EEF1A1P12	eukaryotic translation elongation factor 1 alpha 1 pseudogene 12 [Source:HGNC Symbol;Acc:HGNC:3195]
ENSG00000142867	-0,2257205	2,27714856	0,91589195	0,36289781	0,65026053	BCL10	BCL10 immune signaling adaptor [Source:HGNC Symbol;Acc:HGNC:989]
ENSG00000180979	0,16930029	2,82728838	0,91557076	0,36297816	0,65032474	LRRC57	leucine rich repeat containing 57 [Source:HGNC Symbol;Acc:HGNC:26719]
ENSG00000105290	-0,2100672	1,90792637	0,91417655	0,36332724	0,65039181	APLP1	amyloid beta precursor like protein 1 [Source:HGNC Symbol;Acc:HGNC:597]
ENSG00000108924	-0,1264502	7,13732537	0,91429952	0,36329643	0,65039181	HLF	HLF transcription factor, PAR bZIP family member [Source:HGNC Symbol;Acc:HGNC:4977]
ENSG00000132164	0,16335469	5,39439836	0,91435632	0,3632822	0,65039181	SLC6A11	solute carrier family 6 member 11 [Source:HGNC Symbol;Acc:HGNC:11044]
ENSG00000146247	0,23860563	3,3679632	0,91486632	0,36315447	0,65039181	PHIP	pleckstrin homology domain interacting protein [Source:HGNC Symbol;Acc:HGNC:15673]
ENSG00000236349	0,6847891	-1,4860308	0,91510658	0,36309432	0,65039181	SUCLG2P2	SUCLG2 pseudogene 2 [Source:HGNC Symbol;Acc:HGNC:43997]
ENSG00000247556	0,16708317	4,12304623	0,91505417	0,36310744	0,65039181	OIP5-AS1	OIP5 antisense RNA 1 [Source:HGNC Symbol;Acc:HGNC:43563]
ENSG00000285825	-0,577724	-1,8913767	0,91438304	0,3632755	0,65039181	CCDC15-DT	CCDC15 divergent transcript [Source:HGNC Symbol;Acc:HGNC:54193]
ENSG00000100473	0,15414859	7,15631162	0,91340707	0,36352011	0,65057765	COCH	cochlin [Source:HGNC Symbol;Acc:HGNC:2180]
ENSG00000235559	0,29738901	0,1580789	0,91350548	0,36349544	0,65057765	NOP56P1	NOP56 ribonucleoprotein pseudogene 1 [Source:HGNC Symbol;Acc:HGNC:13962]
ENSG00000113240	-0,1590909	3,37565704	0,91321173	0,36356911	0,65058563	CLK4	CDC like kinase 4 [Source:HGNC Symbol;Acc:HGNC:13659]
ENSG00000240445	0,24268215	0,7915788	0,91274549	0,36368607	0,65063554	FOXO3B	forkhead box O3B [Source:HGNC Symbol;Acc:HGNC:3822]
ENSG00000286024	-0,4668268	-1,01069	0,91285358	0,36365895	0,65063554	AC026803.3	novel transcript, antisense to BCAT2
ENSG00000233822	0,44742056	-0,007877	0,91247973	0,36375277	0,65067519	H2BC15	H2B clustered histone 15 [Source:HGNC Symbol;Acc:HGNC:4749]
ENSG00000010219	0,14907882	4,34216185	0,90997409	0,36438254	0,65145463	DYRK4	dual specificity tyrosine phosphorylation regulated kinase 4 [Source:HGNC Symbol;Acc:HGNC:3095]
ENSG00000120318	-0,5000846	-1,5758527	0,90956889	0,36448454	0,65145463	ARAP3	ArfGAP with RhoGAP domain, ankyrin repeat and PH domain 3 [Source:HGNC Symbol;Acc:HGNC:24097]
ENSG00000161202	0,13863262	5,70169449	0,90999249	0,36437791	0,65145463	DVL3	dishevelled segment polarity protein 3 [Source:HGNC Symbol;Acc:HGNC:3087]
ENSG00000162620	-0,4956155	-0,9673128	0,90938417	0,36453105	0,65145463	LRRIQ3	leucine rich repeats and IQ motif containing 3 [Source:HGNC Symbol;Acc:HGNC:28318]
ENSG00000164951	0,26511206	0,41123798	0,90932777	0,36454525	0,65145463	PDP1	pyruvate dehyrogenase phosphatase catalytic subunit 1 [Source:HGNC Symbol;Acc:HGNC:9279]
ENSG00000180185	-0,1299264	5,27670003	0,90972521	0,36444518	0,65145463	FAHD1	fumarylacetoacetate hydrolase domain containing 1 [Source:HGNC Symbol;Acc:HGNC:14169]
ENSG00000198301	0,14308539	5,52091729	0,90965798	0,36446211	0,65145463	SDAD1	SDA1 domain containing 1 [Source:HGNC Symbol;Acc:HGNC:25537]
ENSG00000204335	-0,1311633	7,25694078	0,91047503	0,3642565	0,65145463	SP5	Sp5 transcription factor [Source:HGNC Symbol;Acc:HGNC:14529]
ENSG00000114023	0,13384069	7,52970369	0,90890119	0,36465271	0,65156695	FAM162A	family with sequence similarity 162 member A [Source:HGNC Symbol;Acc:HGNC:17865]
ENSG00000106733	-0,3274323	1,25428539	0,90843931	0,36476911	0,65165766	NMRK1	nicotinamide riboside kinase 1 [Source:HGNC Symbol;Acc:HGNC:26057]
ENSG00000148411	0,19719994	4,03196893	0,90816887	0,36483729	0,65165766	NACC2	NACC family member 2 [Source:HGNC Symbol;Acc:HGNC:23846]
ENSG00000149716	-0,176908	3,30490016	0,90826441	0,3648132	0,65165766	LTO1	LTO1 maturation factor of ABCE1 [Source:HGNC Symbol;Acc:HGNC:17589]
ENSG00000058335	-0,6053698	-1,5958678	0,90782861	0,3649231	0,65173125	RASGRF1	Ras protein specific guanine nucleotide releasing factor 1 [Source:HGNC Symbol;Acc:HGNC:9875]
ENSG00000017797	0,16447645	4,27886979	0,90738568	0,36503485	0,65182353	RALBP1	ralA binding protein 1 [Source:HGNC Symbol;Acc:HGNC:9841]
ENSG00000174579	0,17183629	2,24083068	0,90727016	0,36506401	0,65182353	MSL2	MSL complex subunit 2 [Source:HGNC Symbol;Acc:HGNC:25544]
ENSG00000068912	-0,1272045	7,47163551	0,90596944	0,36539252	0,65209913	ERLEC1	endoplasmic reticulum lectin 1 [Source:HGNC Symbol;Acc:HGNC:25222]

ENSG00000070882	-0,1594413	4,39635081	0,90595208	0,36539691	0,65209913	OSBPL3	oxysterol binding protein like 3 [Source:HGNC Symbol;Acc:HGNC:16370]
ENSG00000127774	-0,1789814	4,71061734	0,90628406	0,36531302	0,65209913	EMC6	ER membrane protein complex subunit 6 [Source:HGNC Symbol;Acc:HGNC:28430]
ENSG00000214194	-0,1607149	3,93849061	0,90642331	0,36527784	0,65209913	SMIM30	small integral membrane protein 30 [Source:HGNC Symbol;Acc:HGNC:48953]
ENSG00000102977	-0,1496562	5,51704242	0,90500995	0,36563514	0,65236084	ACD	ACD shelterin complex subunit and telomerase recruitment factor [Source:HGNC Symbol;Acc:HGNC:25070]
ENSG00000141101	-0,1349998	7,93464868	0,90498774	0,36564076	0,65236084	NOB1	NIN1 (RPN12) binding protein 1 homolog [Source:HGNC Symbol;Acc:HGNC:29540]
ENSG00000234287	-0,271028	0,37670542	0,90484247	0,36567752	0,65236084	AC099560.2	ribosomal protein S27 (RPS27) pseudogene
ENSG00000168653	-0,1389471	8,5224134	0,9044546	0,36577569	0,65237663	NDUFS5	NADH:ubiquinone oxidoreductase subunit S5 [Source:HGNC Symbol;Acc:HGNC:7712]
ENSG00000198589	0,19129751	4,16835653	0,9045662	0,36574744	0,65237663	LRBA	LPS responsive beige-like anchor protein [Source:HGNC Symbol;Acc:HGNC:1742]
ENSG00000204604	0,17915384	2,29951622	0,90421175	0,36583717	0,65240664	ZNF320	zinc finger protein 320 [Source:HGNC Symbol;Acc:HGNC:13842]
ENSG00000125991	0,12620067	9,51119607	0,90123579	0,36659189	0,65354434	ERGIC3	ERGIC and golgi 3 [Source:HGNC Symbol;Acc:HGNC:15927]
ENSG00000144355	-0,3838195	-0,7666106	0,90149717	0,36652551	0,65354434	DLX1	distal-less homeobox 1 [Source:HGNC Symbol;Acc:HGNC:2914]
ENSG00000189007	0,16279952	2,6987215	0,9011671	0,36660934	0,65354434	ADAT2	adenosine deaminase tRNA specific 2 [Source:HGNC Symbol;Acc:HGNC:21172]
ENSG00000007384	-0,1822939	2,96912176	0,9008221	0,36669699	0,65362084	RHBDF1	rhomboid 5 homolog 1 [Source:HGNC Symbol;Acc:HGNC:20561]
ENSG00000229833	-0,145539	3,04829794	0,90045476	0,36679036	0,6537075	PET100	PET100 cytochrome c oxidase chaperone [Source:HGNC Symbol;Acc:HGNC:40038]
ENSG00000082153	0,13393776	7,75642944	0,89946588	0,36704188	0,65385139	BZW1	basic leucine zipper and W2 domains 1 [Source:HGNC Symbol;Acc:HGNC:18380]
ENSG00000104142	-0,1549835	4,68844428	0,89895746	0,3671713	0,65385139	VPS18	VPS18 core subunit of CORVET and HOPS complexes [Source:HGNC Symbol;Acc:HGNC:15972]
ENSG00000107104	0,17007341	6,20635524	0,89856697	0,36727075	0,65385139	KANK1	KN motif and ankyrin repeat domains 1 [Source:HGNC Symbol;Acc:HGNC:19309]
ENSG00000126822	0,14553915	3,58091825	0,89910462	0,36713383	0,65385139	PLEKHG3	pleckstrin homology and RhoGEF domain containing G3 [Source:HGNC Symbol;Acc:HGNC:20364]
ENSG00000127191	-0,1480406	5,28677681	0,89951362	0,36702973	0,65385139	TRAF2	TNF receptor associated factor 2 [Source:HGNC Symbol;Acc:HGNC:12032]
ENSG00000129518	0,19907555	6,40236273	0,89905244	0,36714712	0,65385139	EAPP	E2F associated phosphoprotein [Source:HGNC Symbol;Acc:HGNC:19312]
ENSG00000133433	0,41526838	-1,081473	0,89788439	0,36744468	0,65385139	GSTT2B	glutathione S-transferase theta 2B [Source:HGNC Symbol;Acc:HGNC:33437]
ENSG00000148082	0,30545608	0,127304	0,89846525	0,36729666	0,65385139	SHC3	SHC adaptor protein 3 [Source:HGNC Symbol;Acc:HGNC:18181]
ENSG00000163479	-0,1299527	9,65525533	0,89785201	0,36745293	0,65385139	SSR2	signal sequence receptor subunit 2 [Source:HGNC Symbol;Acc:HGNC:11324]
ENSG00000166295	-0,1710365	5,64428523	0,89816072	0,36737425	0,65385139	ANAPC16	anaphase promoting complex subunit 16 [Source:HGNC Symbol;Acc:HGNC:26976]
ENSG00000171466	0,15520214	3,14349125	0,89844644	0,36730145	0,65385139	ZNF562	zinc finger protein 562 [Source:HGNC Symbol;Acc:HGNC:25950]
ENSG00000187535	0,18504988	3,03231878	0,89904991	0,36714776	0,65385139	IFT140	intraflagellar transport 140 [Source:HGNC Symbol;Acc:HGNC:29077]
ENSG00000225138	-0,6723644	-2,0208233	0,89807157	0,36739697	0,65385139	SLC9A3-AS1	SLC9A3 antisense RNA 1 [Source:HGNC Symbol;Acc:HGNC:40550]
ENSG00000105793	0,18014843	3,93193961	0,89747003	0,36755033	0,65394065	GTPBP10	GTP binding protein 10 [Source:HGNC Symbol;Acc:HGNC:25106]
ENSG00000196371	-0,5624572	-0,7062026	0,8973042	0,36759262	0,65394065	FUT4	fucosyltransferase 4 [Source:HGNC Symbol;Acc:HGNC:4015]
ENSG00000134398	0,22116349	1,13870065	0,8969691	0,36767811	0,65394752	ERN2	endoplasmic reticulum to nucleus signaling 2 [Source:HGNC Symbol;Acc:HGNC:16942]
ENSG00000234494	-0,2263629	1,14349755	0,89676273	0,36773077	0,65394752	SP2-AS1	SP2 antisense RNA 1 [Source:HGNC Symbol;Acc:HGNC:51341]
ENSG00000244486	-0,1630787	3,84597168	0,89683758	0,36771167	0,65394752	SCARF2	scavenger receptor class F member 2 [Source:HGNC Symbol;Acc:HGNC:19869]
ENSG00000198743	0,32400841	1,50205214	0,8957613	0,36798649	0,65432261	SLC5A3	solute carrier family 5 member 3 [Source:HGNC Symbol;Acc:HGNC:11038]
ENSG00000241095	-0,3897869	-0,8699432	0,89522267	0,36812414	0,65448772	CYP51A1P1	cytochrome P450 family 51 subfamily A member 1 pseudogene 1 [Source:HGNC Symbol;Acc:HGNC:20245]
ENSG00000039523	0,24606629	4,37856187	0,89503315	0,36817259	0,65449421	RIPOR1	RHO family interacting cell polarization regulator 1 [Source:HGNC Symbol;Acc:HGNC:25836]
ENSG00000179335	0,14903094	5,67373429	0,89469116	0,36826005	0,65457003	CLK3	CDC like kinase 3 [Source:HGNC Symbol;Acc:HGNC:2071]
ENSG00000170085	0,17675782	2,79306083	0,89379235	0,36849005	0,65489917	SIMC1	SUMO interacting motifs containing 1 [Source:HGNC Symbol;Acc:HGNC:24779]
ENSG00000050344	0,15427136	3,91725565	0,89311769	0,36866285	0,65493515	NFE2L3	nuclear factor, erythroid 2 like 3 [Source:HGNC Symbol;Acc:HGNC:7783]
ENSG00000059122	0,14435554	4,12775224	0,89301479	0,36868921	0,65493515	FLYWCH1	FLYWCH-type zinc finger 1 [Source:HGNC Symbol;Acc:HGNC:25404]
ENSG00000176182	-0,2742981	2,29508924	0,89330682	0,36861439	0,65493515	MYPOP	Myb related transcription factor, partner of profilin [Source:HGNC Symbol;Acc:HGNC:20178]
ENSG00000275719	-0,4875894	-0,4506024	0,89301319	0,36868962	0,65493515	AC008622.2	novel transcript, antisense to SLC1A5
ENSG00000268043	0,41638114	0,81772305	0,89260467	0,36879432	0,65496186	NBPF12	NBPF member 12 [Source:HGNC Symbol;Acc:HGNC:24297]
ENSG00000286931	0,73252272	-1,1141224	0,89271841	0,36876516	0,65496186	AL136419.3	novel transcript
ENSG00000137831	-0,224602	4,12485261	0,89208412	0,3689278	0,65503965	UACA	uveal autoantigen with coiled-coil domains and ankyrin repeats [Source:HGNC Symbol;Acc:HGNC:15947]

ENSG00000228835	-0,3973709	-0,8007137	0,89225794	0,36888322	0,65503965	AC012123.1	novel transcript, antisense to KLHL14
ENSG00000110768	0,14830596	5,28716374	0,89118755	0,36915787	0,65536849	GTF2H1	general transcription factor IIH subunit 1 [Source:HGNC Symbol;Acc:HGNC:4655]
ENSG00000116473	0,19404572	4,60769943	0,89061575	0,36930471	0,65554952	RAP1A	RAP1A, member of RAS oncogene family [Source:HGNC Symbol;Acc:HGNC:9855]
ENSG00000243335	0,18332271	2,16817489	0,890397	0,36936091	0,65556962	KCTD7	potassium channel tetramerization domain containing 7 [Source:HGNC Symbol;Acc:HGNC:21957]
ENSG00000141570	0,14703946	4,06044123	0,89001586	0,36945887	0,65558419	CBX8	chromobox 8 [Source:HGNC Symbol;Acc:HGNC:15962]
ENSG00000177548	-0,1593754	4,25508279	0,89002396	0,36945679	0,65558419	RABEP2	rabaptin, RAB GTPase binding effector protein 2 [Source:HGNC Symbol;Acc:HGNC:24817]
ENSG00000250474	0,45132703	-1,2691604	0,8896594	0,36955051	0,65566717	WBP1LP2	WBP1L pseudogene 2 [Source:HGNC Symbol;Acc:HGNC:43949]
ENSG00000075568	0,22108099	5,66601559	0,8890703	0,36970205	0,65570316	TMEM131	transmembrane protein 131 [Source:HGNC Symbol;Acc:HGNC:30366]
ENSG00000101665	-0,1582381	4,22960276	0,88905709	0,36970545	0,65570316	SMAD7	SMAD family member 7 [Source:HGNC Symbol;Acc:HGNC:6773]
ENSG00000213741	-0,1659791	9,27300595	0,889336	0,36963369	0,65570316	RPS29	ribosomal protein S29 [Source:HGNC Symbol;Acc:HGNC:10419]
ENSG00000156675	0,24561147	4,13430406	0,88867398	0,36980405	0,65579842	RAB11FIP1	RAB11 family interacting protein 1 [Source:HGNC Symbol;Acc:HGNC:30265]
ENSG00000087338	0,21223757	3,35524687	0,88740728	0,37013035	0,65600949	GMCL1	germ cell-less 1, spermatogenesis associated [Source:HGNC Symbol;Acc:HGNC:23843]
ENSG00000106785	0,1753926	3,31903022	0,88798858	0,36998055	0,65600949	TRIM14	tripartite motif containing 14 [Source:HGNC Symbol;Acc:HGNC:16283]
ENSG00000156256	-0,1623271	4,63547526	0,88785939	0,37001384	0,65600949	USP16	ubiquitin specific peptidase 16 [Source:HGNC Symbol;Acc:HGNC:12614]
ENSG00000172765	-0,1837833	3,51271003	0,88736631	0,37014091	0,65600949	TMCC1	transmembrane and coiled-coil domain family 1 [Source:HGNC Symbol;Acc:HGNC:29116]
ENSG00000183844	0,15289369	5,60657309	0,88734039	0,37014759	0,65600949	FAM3B	FAM3 metabolism regulating signaling molecule B [Source:HGNC Symbol;Acc:HGNC:1253]
ENSG00000064115	-0,131003	6,85646124	0,88712492	0,37020315	0,65602836	TM7SF3	transmembrane 7 superfamily member 3 [Source:HGNC Symbol;Acc:HGNC:23049]
ENSG00000105926	0,1595386	2,65709905	0,88670262	0,37031206	0,65605095	MPP6	membrane palmitoylated protein 6 [Source:HGNC Symbol;Acc:HGNC:18167]
ENSG00000167996	0,13869119	11,1703055	0,8857602	0,3705553	0,65605095	FTH1	ferritin heavy chain 1 [Source:HGNC Symbol;Acc:HGNC:3976]
ENSG00000170448	-0,1696709	4,7779547	0,88601878	0,37048853	0,65605095	NFXL1	nuclear transcription factor, X-box binding like 1 [Source:HGNC Symbol;Acc:HGNC:18726]
ENSG00000171634	0,30502403	5,09350142	0,88590528	0,37051784	0,65605095	BPTF	bromodomain PHD finger transcription factor [Source:HGNC Symbol;Acc:HGNC:3581]
ENSG00000173011	0,137262	4,7909494	0,8856833	0,37057515	0,65605095	TADA2B	transcriptional adaptor 2B [Source:HGNC Symbol;Acc:HGNC:30781]
ENSG00000185340	-0,196509	2,35254497	0,88655031	0,37035135	0,65605095	GAS2L1	growth arrest specific 2 like 1 [Source:HGNC Symbol;Acc:HGNC:16955]
ENSG00000219085	0,40558726	-1,290695	0,88646278	0,37037394	0,65605095	NPM1P37	nucleophosmin 1 pseudogene 37 [Source:HGNC Symbol;Acc:HGNC:45216]
ENSG00000251580	-0,2702157	1,15179226	0,88596538	0,37050232	0,65605095	LINC02482	long intergenic non-protein coding RNA 2482 [Source:HGNC Symbol;Acc:HGNC:53458]
ENSG00000099917	-0,2252328	5,0660038	0,88477414	0,37081006	0,65638727	MED15	mediator complex subunit 15 [Source:HGNC Symbol;Acc:HGNC:14248]
ENSG00000270945	-0,4434963	-1,3733531	0,88447144	0,37088832	0,65644626	HSPE1P7	heat shock protein family E (Hsp10) member 1 pseudogene 7 [Source:HGNC Symbol;Acc:HGNC:49327]
ENSG00000072736	0,21671264	4,16394823	0,88413048	0,3709765	0,65652279	NFATC3	nuclear factor of activated T cells 3 [Source:HGNC Symbol;Acc:HGNC:7777]
ENSG00000122873	0,15363718	5,69664736	0,88373617	0,37107852	0,65659063	CISD1	CDGSH iron sulfur domain 1 [Source:HGNC Symbol;Acc:HGNC:30880]
ENSG00000203644	-0,3840084	-0,6489668	0,88363494	0,37110472	0,65659063	AC083799.1	novel transcript, sense intronic to TMCC1
ENSG00000110046	-0,266265	4,14140121	0,88316335	0,3712268	0,65660442	ATG2A	autophagy related 2A [Source:HGNC Symbol;Acc:HGNC:29028]
ENSG00000169251	0,15872892	5,81367771	0,88312249	0,37123738	0,65660442	NMD3	NMD3 ribosome export adaptor [Source:HGNC Symbol;Acc:HGNC:24250]
ENSG00000278311	0,16447068	5,4268621	0,88308402	0,37124735	0,65660442	GGNBP2	gametogenetin binding protein 2 [Source:HGNC Symbol;Acc:HGNC:19357]
ENSG00000135220	0,17044323	5,55131546	0,882899	0,37129527	0,65660968	UGT2A3	UDP glucuronosyltransferase family 2 member A3 [Source:HGNC Symbol;Acc:HGNC:28528]
ENSG00000051128	-0,2376518	2,39908757	0,88247415	0,37140533	0,65664879	HOMER3	homer scaffold protein 3 [Source:HGNC Symbol;Acc:HGNC:17514]
ENSG00000103496	-0,1400686	5,39236913	0,88107813	0,37176737	0,65664879	STX4	syntaxin 4 [Source:HGNC Symbol;Acc:HGNC:11439]
ENSG00000124767	0,18855465	6,54631728	0,88244064	0,37141402	0,65664879	GLO1	glyoxalase I [Source:HGNC Symbol;Acc:HGNC:4323]
ENSG00000125319	0,14288297	3,87890973	0,88159296	0,37163379	0,65664879	HROB	homologous recombination factor with OB-fold [Source:HGNC Symbol;Acc:HGNC:28460]
ENSG00000155438	-0,1215185	6,76852778	0,88134356	0,37169849	0,65664879	NIFK	nucleolar protein interacting with the FHA domain of MKI67 [Source:HGNC Symbol;Acc:HGNC:17838]
ENSG00000184983	-0,152592	6,79104571	0,88132275	0,37170389	0,65664879	NDUFA6	NADH:ubiquinone oxidoreductase subunit A6 [Source:HGNC Symbol;Acc:HGNC:7690]
ENSG00000186184	0,13834619	7,17294022	0,88191935	0,37154915	0,65664879	POLR1D	RNA polymerase I and III subunit D [Source:HGNC Symbol;Acc:HGNC:20422]
ENSG00000189319	-0,1602533	3,96836153	0,88090687	0,37181182	0,65664879	FAM53B	family with sequence similarity 53 member B [Source:HGNC Symbol;Acc:HGNC:28968]
ENSG00000198900	-0,2687501	7,79935553	0,88165877	0,37161672	0,65664879	TOP1	DNA topoisomerase I [Source:HGNC Symbol;Acc:HGNC:11986]
ENSG00000204344	-0,1452004	3,14862812	0,88094034	0,37180313	0,65664879	STK19	serine/threonine kinase 19 [Source:HGNC Symbol;Acc:HGNC:11398]

ENSG00000213996	-0,3557227	-0,37866	0,88228351	0,37145474	0,65664879	TM6SF2	transmembrane 6 superfamily member 2 [Source:HGNC Symbol;Acc:HGNC:11861]
ENSG00000175893	-0,2593592	0,82780908	0,88060199	0,37189097	0,65670919	ZDHHC21	zinc finger DHHC-type palmitoyltransferase 21 [Source:HGNC Symbol;Acc:HGNC:20750]
ENSG00000140367	0,18736988	3,12603024	0,88025037	0,37198229	0,65679106	UBE2Q2	ubiquitin conjugating enzyme E2 Q2 [Source:HGNC Symbol;Acc:HGNC:19248]
ENSG00000287688	-0,5027887	-1,7008656	0,87975479	0,37211105	0,65693901	AC011317.1	novel transcript
ENSG00000260136	-0,1781596	3,44993343	0,87954116	0,37216658	0,65695765	AC008915.2	novel transcript
ENSG00000235043	-0,2548015	0,75321205	0,87922824	0,37224794	0,65702188	TECRP1	trans-2,3-enoyl-CoA reductase pseudogene 1 [Source:HGNC Symbol;Acc:HGNC:44050]
ENSG00000236283	0,27325109	0,90191671	0,87846701	0,37244597	0,657292	AC019197.1	novel transcript, antisense to SLC38A11 and SCN3A
ENSG00000162924	0,24197108	0,7933389	0,87781822	0,37261488	0,65731977	REL	REL proto-oncogene, NF-kB subunit [Source:HGNC Symbol;Acc:HGNC:9954]
ENSG00000164609	-0,2035851	4,93484564	0,8777153	0,37264168	0,65731977	SLU7	SLU7 homolog, splicing factor [Source:HGNC Symbol;Acc:HGNC:16939]
ENSG00000186676	0,35219997	-0,6558455	0,87774106	0,37263497	0,65731977	EEF1GP1	eukaryotic translation elongation factor 1 gamma pseudogene 1 [Source:HGNC Symbol;Acc:HGNC:44556]
ENSG00000204856	-0,1563297	3,44208366	0,87773898	0,37263552	0,65731977	FAM216A	family with sequence similarity 216 member A [Source:HGNC Symbol;Acc:HGNC:30180]
ENSG00000116874	0,15425822	3,99735957	0,87728456	0,3727539	0,65743834	WARS2	tryptophanyl tRNA synthetase 2, mitochondrial [Source:HGNC Symbol;Acc:HGNC:12730]
ENSG00000123444	0,16144598	3,52231295	0,87608888	0,37306568	0,6579088	KBTBD4	kelch repeat and BTB domain containing 4 [Source:HGNC Symbol;Acc:HGNC:23761]
ENSG00000088256	0,18508888	5,49621414	0,8755536	0,37320539	0,65791692	GNA11	G protein subunit alpha 11 [Source:HGNC Symbol;Acc:HGNC:4379]
ENSG00000167699	0,14777614	6,92634725	0,87562744	0,37318611	0,65791692	GLOD4	glyoxalase domain containing 4 [Source:HGNC Symbol;Acc:HGNC:14111]
ENSG00000260917	0,49801762	-0,4343238	0,87581928	0,37313603	0,65791692	AL158212.3	novel transcript, overlapping to VTI1A
ENSG00000060069	0,14922015	4,6279301	0,87496349	0,3733595	0,65810918	CTDP1	CTD phosphatase subunit 1 [Source:HGNC Symbol;Acc:HGNC:2498]
ENSG00000068400	-0,256825	4,69449658	0,87430272	0,37353218	0,65823262	GRIPAP1	GRIP1 associated protein 1 [Source:HGNC Symbol;Acc:HGNC:18706]
ENSG00000140598	-0,1801792	3,97866984	0,87433697	0,37352322	0,65823262	EFL1	elongation factor like GTPase 1 [Source:HGNC Symbol;Acc:HGNC:25789]
ENSG00000257621	-0,1832705	2,34757921	0,87417834	0,37356469	0,65823262	PSMA3-AS1	PSMA3 antisense RNA 1 [Source:HGNC Symbol;Acc:HGNC:26445]
ENSG00000100902	-0,1219642	6,11354876	0,87368561	0,37369356	0,65838027	PSMA6	proteasome 20S subunit alpha 6 [Source:HGNC Symbol;Acc:HGNC:9535]
ENSG00000119965	0,19154364	2,67186635	0,87345468	0,37375398	0,65840731	C10orf88	chromosome 10 open reading frame 88 [Source:HGNC Symbol;Acc:HGNC:25822]
ENSG00000213563	-0,151731	3,98927137	0,87321209	0,37381746	0,65843975	C8orf82	chromosome 8 open reading frame 82 [Source:HGNC Symbol;Acc:HGNC:33826]
ENSG00000087245	0,32733555	-0,1527539	0,86830102	0,37510626	0,65853318	MMP2	matrix metallopeptidase 2 [Source:HGNC Symbol;Acc:HGNC:7166]
ENSG00000103671	0,17479266	4,71736685	0,8684327	0,37507161	0,65853318	TRIP4	thyroid hormone receptor interactor 4 [Source:HGNC Symbol;Acc:HGNC:12310]
ENSG00000104881	-0,2507361	1,80034699	0,87129256	0,37432038	0,65853318	PPP1R13L	protein phosphatase 1 regulatory subunit 13 like [Source:HGNC Symbol;Acc:HGNC:18838]
ENSG00000108821	-0,3916471	-0,1392739	0,87258412	0,37398187	0,65853318	COL1A1	collagen type I alpha 1 chain [Source:HGNC Symbol;Acc:HGNC:2197]
ENSG00000112031	0,13585328	4,61580205	0,86767953	0,37526984	0,65853318	MTRF1L	mitochondrial translational release factor 1 like [Source:HGNC Symbol;Acc:HGNC:21051]
ENSG00000112212	-0,4714405	-1,1490912	0,86804291	0,37517418	0,65853318	TSPO2	translocator protein 2 [Source:HGNC Symbol;Acc:HGNC:21256]
ENSG00000112877	-0,2057867	2,88163727	0,87068972	0,37447854	0,65853318	CEP72	centrosomal protein 72 [Source:HGNC Symbol;Acc:HGNC:25547]
ENSG00000125633	0,14721113	5,20397658	0,86655815	0,37556528	0,65853318	CCDC93	coiled-coil domain containing 93 [Source:HGNC Symbol;Acc:HGNC:25611]
ENSG00000125637	0,16214435	4,53597369	0,87129264	0,37432036	0,65853318	PSD4	pleckstrin and Sec7 domain containing 4 [Source:HGNC Symbol;Acc:HGNC:19096]
ENSG00000129636	0,15575474	7,2167402	0,86728974	0,3753725	0,65853318	ITFG1	integrin alpha FG-GAP repeat containing 1 [Source:HGNC Symbol;Acc:HGNC:30697]
ENSG00000135845	-0,1840574	4,70492012	0,87150068	0,37426581	0,65853318	PIGC	phosphatidylinositol glycan anchor biosynthesis class C [Source:HGNC Symbol;Acc:HGNC:8960]
ENSG00000136237	0,23368407	3,33411437	0,87025638	0,3745923	0,65853318	RAPGEF5	Rap guanine nucleotide exchange factor 5 [Source:HGNC Symbol;Acc:HGNC:16862]
ENSG00000137710	-0,3073113	5,2059953	0,86701542	0,37544476	0,65853318	RDX	radixin [Source:HGNC Symbol;Acc:HGNC:9944]
ENSG00000144401	-0,1672764	5,2046662	0,86698115	0,3754538	0,65853318	METTL21A	methyltransferase like 21A [Source:HGNC Symbol;Acc:HGNC:30476]
ENSG00000162980	-0,1650534	3,23873645	0,86956603	0,37477363	0,65853318	ARL5A	ADP ribosylation factor like GTPase 5A [Source:HGNC Symbol;Acc:HGNC:696]
ENSG00000163159	-0,1463798	5,24870164	0,86882948	0,37496725	0,65853318	VPS72	vacuolar protein sorting 72 homolog [Source:HGNC Symbol;Acc:HGNC:11644]
ENSG00000164051	-0,1308772	5,2637298	0,86829927	0,37510672	0,65853318	CCDC51	coiled-coil domain containing 51 [Source:HGNC Symbol;Acc:HGNC:25714]
ENSG00000164902	0,13061887	5,03829752	0,86895743	0,3749336	0,65853318	PHAX	phosphorylated adaptor for RNA export [Source:HGNC Symbol;Acc:HGNC:10241]
ENSG00000169180	0,14274325	6,77308482	0,86648927	0,37558344	0,65853318	XPO6	exportin 6 [Source:HGNC Symbol;Acc:HGNC:19733]
ENSG00000175220	0,19238231	5,81328576	0,86892944	0,37494096	0,65853318	ARHGAP1	Rho GTPase activating protein 1 [Source:HGNC Symbol;Acc:HGNC:673]
ENSG00000178952	0,13191506	10,4190607	0,86858512	0,37503152	0,65853318	TUFM	Tu translation elongation factor, mitochondrial [Source:HGNC Symbol;Acc:HGNC:12420]

ENSG00000182858	-0,1480249	5,33981196	0,8719808	0,37413994	0,65853318	ALG12	ALG12 alpha-1,6-mannosyltransferase [Source:HGNC Symbol;Acc:HGNC:19358]
ENSG00000183723	0,18359978	2,85681387	0,86987246	0,37469312	0,65853318	CMTM4	CKLF like MARVEL transmembrane domain containing 4 [Source:HGNC Symbol;Acc:HGNC:19175]
ENSG00000184220	0,21232392	5,56025498	0,87086123	0,37443353	0,65853318	CMSS1	cms1 ribosomal small subunit homolog [Source:HGNC Symbol;Acc:HGNC:28666]
ENSG00000196712	0,2326824	3,70575751	0,86662753	0,37554699	0,65853318	NF1	neurofibromin 1 [Source:HGNC Symbol;Acc:HGNC:7765]
ENSG00000197172	0,17373542	2,54985384	0,8674705	0,37532489	0,65853318	MAGEA6	MAGE family member A6 [Source:HGNC Symbol;Acc:HGNC:6804]
ENSG00000204946	0,22380286	1,81781903	0,86701676	0,37544441	0,65853318	ZNF783	zinc finger family member 783 [Source:HGNC Symbol;Acc:HGNC:27222]
ENSG00000213376	-0,3397347	-0,5628588	0,86725428	0,37538184	0,65853318	GAPDHP71	glyceraldehyde-3-phosphate dehydrogenase pseudogene 71 [Source:HGNC Symbol;Acc:HGNC:4149]
ENSG00000213432	-0,522585	-1,451337	0,86977912	0,37471764	0,65853318	RPL17P34	ribosomal protein L17 pseudogene 34 [Source:HGNC Symbol;Acc:HGNC:36756]
ENSG00000230204	-0,3910844	-1,1554391	0,86774333	0,37525304	0,65853318	FTH1P5	ferritin heavy chain 1 pseudogene 5 [Source:HGNC Symbol;Acc:HGNC:3996]
ENSG00000236534	-0,5019348	-1,8563525	0,86933631	0,374834	0,65853318	H3P36	H3 histone pseudogene 36 [Source:HGNC Symbol;Acc:HGNC:42030]
ENSG00000237456	0,47534279	-1,4614135	0,87032756	0,37457361	0,65853318	ID2B	inhibitor of DNA binding 2B, HLH protein (pseudogene) [Source:HGNC Symbol;Acc:HGNC:30656]
ENSG00000240459	-0,5124395	-1,6013207	0,87027909	0,37458634	0,65853318	AC093663.1	ribosomal protein S12 (RPS12) pseudogene
ENSG00000241316	0,33960611	0,2728781	0,86801829	0,37518066	0,65853318	SUCLG2-AS1	SUCLG2 antisense RNA 1 (head to head) [Source:HGNC Symbol;Acc:HGNC:49643]
ENSG00000243927	-0,1828699	3,92876168	0,87183376	0,37417848	0,65853318	MRPS6	mitochondrial ribosomal protein S6 [Source:HGNC Symbol;Acc:HGNC:14051]
ENSG00000244363	-0,2985423	0,37763188	0,86726751	0,37537835	0,65853318	RPL7P23	ribosomal protein L7 pseudogene 23 [Source:HGNC Symbol;Acc:HGNC:35658]
ENSG00000249193	0,37236066	0,02689136	0,87182054	0,37418195	0,65853318	HSPD1P5	heat shock protein family D (Hsp60) member 1 pseudogene 5 [Source:HGNC Symbol;Acc:HGNC:5266]
ENSG00000257576	0,24006465	2,57692147	0,8690276	0,37491515	0,65853318	HSPD1P4	heat shock protein family D (Hsp60) member 1 pseudogene 4 [Source:HGNC Symbol;Acc:HGNC:35146]
ENSG00000177084	0,18611655	5,08776966	0,86547566	0,37585082	0,65892291	POLE	DNA polymerase epsilon, catalytic subunit [Source:HGNC Symbol;Acc:HGNC:9177]
ENSG00000147050	0,16026174	2,83552968	0,86511394	0,3759463	0,65901123	KDM6A	lysine demethylase 6A [Source:HGNC Symbol;Acc:HGNC:12637]
ENSG00000027847	-0,1363081	5,84541007	0,86434039	0,37615063	0,65902225	B4GALT7	beta-1,4-galactosyltransferase 7 [Source:HGNC Symbol;Acc:HGNC:930]
ENSG00000102910	0,21845222	6,54964007	0,8640656	0,37622326	0,65902225	LONP2	lon peptidase 2, peroxisomal [Source:HGNC Symbol;Acc:HGNC:20598]
ENSG00000108448	0,29232953	0,9535003	0,86435502	0,37614677	0,65902225	TRIM16L	tripartite motif containing 16 like [Source:HGNC Symbol;Acc:HGNC:32670]
ENSG00000131473	-0,2326195	8,90368079	0,86411922	0,37620909	0,65902225	ACLY	ATP citrate lyase [Source:HGNC Symbol;Acc:HGNC:115]
ENSG00000189014	0,4494015	-0,9433402	0,86453773	0,37609849	0,65902225	SHLD2P3	shieldin complex subunit 2 pseudogene 3 [Source:HGNC Symbol;Acc:HGNC:34038]
ENSG00000197978	0,5125632	-1,3237038	0,86457109	0,37608968	0,65902225	GOLGA6L9	golgin A6 family like 9 [Source:HGNC Symbol;Acc:HGNC:37229]
ENSG00000078808	-0,1213597	8,91429182	0,86327978	0,37643107	0,65917873	SDF4	stromal cell derived factor 4 [Source:HGNC Symbol;Acc:HGNC:24188]
ENSG00000134014	0,14225826	6,0527026	0,86304544	0,37649307	0,65917873	ELP3	elongator acetyltransferase complex subunit 3 [Source:HGNC Symbol;Acc:HGNC:20696]
ENSG00000160703	0,13699636	4,12787797	0,86315626	0,37646375	0,65917873	NLRX1	NLR family member X1 [Source:HGNC Symbol;Acc:HGNC:29890]
ENSG00000237296	-0,280167	0,27566931	0,8630873	0,376482	0,65917873	SMG1P1	SMG1 pseudogene 1 [Source:HGNC Symbol;Acc:HGNC:44186]
ENSG00000067064	-0,1318621	7,40513224	0,8622396	0,37670641	0,65922103	IDI1	isopentenyl-diphosphate delta isomerase 1 [Source:HGNC Symbol;Acc:HGNC:5387]
ENSG00000130764	0,17930012	5,92261396	0,86208879	0,37674636	0,65922103	LRRC47	leucine rich repeat containing 47 [Source:HGNC Symbol;Acc:HGNC:29207]
ENSG00000143156	0,18362437	3,46838282	0,8622916	0,37669264	0,65922103	NME7	NME/NM23 family member 7 [Source:HGNC Symbol;Acc:HGNC:20461]
ENSG00000165264	-0,1253086	6,2468694	0,86215472	0,37672889	0,65922103	NDUFB6	NADH:ubiquinone oxidoreductase subunit B6 [Source:HGNC Symbol;Acc:HGNC:7701]
ENSG00000224773	-0,422526	-1,0929687	0,86257914	0,3766165	0,65922103	HSPA8P7	heat shock protein family A (Hsp70) member 8 pseudogene 7 [Source:HGNC Symbol;Acc:HGNC:44922]
ENSG00000253570	-0,2896149	0,25983493	0,86193168	0,37678798	0,65922103	RNF5P1	ring finger protein 5 pseudogene 1 [Source:HGNC Symbol;Acc:HGNC:31053]
ENSG00000075151	-0,2284945	5,10656542	0,86155222	0,37688854	0,65925638	EIF4G3	eukaryotic translation initiation factor 4 gamma 3 [Source:HGNC Symbol;Acc:HGNC:3298]
ENSG00000183840	0,21540869	1,48355174	0,86151488	0,37689844	0,65925638	GPR39	G protein-coupled receptor 39 [Source:HGNC Symbol;Acc:HGNC:4496]
ENSG00000115310	0,14248654	7,83815821	0,86108338	0,37701284	0,6592996	RTN4	reticulon 4 [Source:HGNC Symbol;Acc:HGNC:14085]
ENSG00000255986	-0,3251746	0,09946573	0,86108125	0,37701341	0,6592996	MT1JP	metallothionein 1J, pseudogene [Source:HGNC Symbol;Acc:HGNC:7402]
ENSG00000278259	0,12737495	5,38270274	0,86030983	0,37721807	0,65957855	MYO19	myosin XIX [Source:HGNC Symbol;Acc:HGNC:26234]
ENSG00000164338	0,15879675	4,78275321	0,85934979	0,37747301	0,65986637	UTP15	UTP15 small subunit processome component [Source:HGNC Symbol;Acc:HGNC:25758]
ENSG00000211451	-0,6422178	-1,0854119	0,85941028	0,37745694	0,65986637	GNRHR2	gonadotropin releasing hormone receptor 2 (pseudogene) [Source:HGNC Symbol;Acc:HGNC:16341]
ENSG00000198690	0,13514281	4,19204008	0,85913731	0,37752947	0,6598861	FAN1	FANCD2 and FANCI associated nuclease 1 [Source:HGNC Symbol;Acc:HGNC:29170]
ENSG00000156508	-0,1493155	13,3456704	0,85846407	0,37770846	0,66011997	EEF1A1	eukaryotic translation elongation factor 1 alpha 1 [Source:HGNC Symbol;Acc:HGNC:3189]

ENSG00000127511	0,17583006	4,49482914	0,85800248	0,37783125	0,66017662	SIN3B	SIN3 transcription regulator family member B [Source:HGNC Symbol;Acc:HGNC:19354]
ENSG00000165675	-0,195617	2,15790461	0,85802899	0,37782419	0,66017662	ENOX2	ecto-NOX disulfide-thiol exchanger 2 [Source:HGNC Symbol;Acc:HGNC:2259]
ENSG00000196476	0,1498075	3,99065046	0,85702166	0,37809236	0,66055386	C20orf96	chromosome 20 open reading frame 96 [Source:HGNC Symbol;Acc:HGNC:16227]
ENSG00000168890	-0,1813797	5,49556843	0,8563954	0,37825924	0,66061717	TMEM150A	transmembrane protein 150A [Source:HGNC Symbol;Acc:HGNC:24677]
ENSG00000269220	-0,2009316	1,98717245	0,85665036	0,37819129	0,66061717	LINC00528	long intergenic non-protein coding RNA 528 [Source:HGNC Symbol;Acc:HGNC:26875]
ENSG00000276291	-0,1945912	1,23299063	0,85637654	0,37826426	0,66061717	FRG1HP	FSHD region gene 1 family member H, pseudogene [Source:HGNC Symbol;Acc:HGNC:51767]
ENSG00000117713	0,36182983	4,95333269	0,85587643	0,37839761	0,66077106	ARID1A	AT-rich interaction domain 1A [Source:HGNC Symbol;Acc:HGNC:11110]
ENSG00000226696	-0,4204526	-1,1914527	0,85555188	0,37848418	0,66084324	LENG8-AS1	LENG8 antisense RNA 1 [Source:HGNC Symbol;Acc:HGNC:40705]
ENSG00000136378	-0,643538	-1,7556816	0,85537434	0,37853155	0,66084697	ADAMTS7	ADAM metallopeptidase with thrombospondin type 1 motif 7 [Source:HGNC Symbol;Acc:HGNC:223]
ENSG00000159363	-0,1251425	6,41602362	0,85508666	0,37860833	0,66087755	ATP13A2	ATPase cation transporting 13A2 [Source:HGNC Symbol;Acc:HGNC:30213]
ENSG00000178397	0,14611116	2,85201229	0,85496974	0,37863954	0,66087755	FAM220A	family with sequence similarity 220 member A [Source:HGNC Symbol;Acc:HGNC:22422]
ENSG00000118096	0,17791016	3,62307014	0,85473079	0,37870334	0,66090994	IFT46	intraflagellar transport 46 [Source:HGNC Symbol;Acc:HGNC:26146]
ENSG00000127081	-0,2480953	0,68071091	0,85447584	0,37877143	0,66094982	ZNF484	zinc finger protein 484 [Source:HGNC Symbol;Acc:HGNC:23385]
ENSG00000215784	0,32923236	-0,4794477	0,85427192	0,37882591	0,66096593	FAM72D	family with sequence similarity 72 member D [Source:HGNC Symbol;Acc:HGNC:33593]
ENSG00000147324	0,13530471	4,3952384	0,8539486	0,37891231	0,66103772	MFHAS1	malignant fibrous histiocytoma amplified sequence 1 [Source:HGNC Symbol;Acc:HGNC:16982]
ENSG00000140307	-0,1564844	5,38911382	0,85369903	0,37897901	0,66107516	GTF2A2	general transcription factor IIA subunit 2 [Source:HGNC Symbol;Acc:HGNC:4647]
ENSG00000132432	-0,1879632	5,7356109	0,85310381	0,37913819	0,66127386	SEC61G	SEC61 translocon subunit gamma [Source:HGNC Symbol;Acc:HGNC:18277]
ENSG00000171763	0,14661717	4,36446444	0,85265029	0,37925954	0,66133499	SPATA5L1	spermatogenesis associated 5 like 1 [Source:HGNC Symbol;Acc:HGNC:28762]
ENSG00000226396	-0,290809	0,41062203	0,85263445	0,37926378	0,66133499	AL031727.1	ribosomal protein S14 (RPS14) pseudogene
ENSG00000054116	-0,1453837	6,29176085	0,85153795	0,37955743	0,66147639	TRAPPC3	trafficking protein particle complex 3 [Source:HGNC Symbol;Acc:HGNC:19942]
ENSG00000075826	-0,4677391	-1,6713764	0,85112567	0,37966794	0,66147639	SEC31B	SEC31 homolog B, COPII coat complex component [Source:HGNC Symbol;Acc:HGNC:23197]
ENSG00000106483	0,34834344	-0,2044967	0,85032733	0,37988206	0,66147639	SFRP4	secreted frizzled related protein 4 [Source:HGNC Symbol;Acc:HGNC:10778]
ENSG00000114650	0,13480925	7,14198376	0,85013566	0,37993349	0,66147639	SCAP	SREBF chaperone [Source:HGNC Symbol;Acc:HGNC:30634]
ENSG00000131944	0,16838498	2,16867571	0,85065516	0,37979411	0,66147639	FAAP24	FA core complex associated protein 24 [Source:HGNC Symbol;Acc:HGNC:28467]
ENSG00000134243	0,20461721	7,356344	0,85037436	0,37986944	0,66147639	SORT1	sortilin 1 [Source:HGNC Symbol;Acc:HGNC:11186]
ENSG00000138386	0,14229947	3,79151092	0,85090758	0,37972641	0,66147639	NAB1	NGFI-A binding protein 1 [Source:HGNC Symbol;Acc:HGNC:7626]
ENSG00000167565	0,14634674	3,53752181	0,8510731	0,37968203	0,66147639	SERTAD3	SERTA domain containing 3 [Source:HGNC Symbol;Acc:HGNC:17931]
ENSG00000176896	0,46680314	-0,6348198	0,85030142	0,37988901	0,66147639	TCEANC	transcription elongation factor A N-terminal and central domain containing [Source:HGNC Symbol;Acc:HGNC:282
ENSG00000215183	-0,5413598	-2,0152256	0,85112087	0,37966922	0,66147639	MSMP	microseminoprotein, prostate associated [Source:HGNC Symbol;Acc:HGNC:29663]
ENSG00000222019	0,31034131	0,08996638	0,85028451	0,37989355	0,66147639	URAHP	urate (hydroxyiso-) hydrolase, pseudogene [Source:HGNC Symbol;Acc:HGNC:43695]
ENSG00000225972	0,26131572	0,92511371	0,85014844	0,37993006	0,66147639	MTND1P23	MT-ND1 pseudogene 23 [Source:HGNC Symbol;Acc:HGNC:42092]
ENSG00000261008	0,57724521	-0,9776338	0,85149454	0,37956907	0,66147639	LINC01572	long intergenic non-protein coding RNA 1572 [Source:HGNC Symbol;Acc:HGNC:51385]
ENSG00000091317	0,13126403	5,2798321	0,84885858	0,38027648	0,66191577	CMTM6	CKLF like MARVEL transmembrane domain containing 6 [Source:HGNC Symbol;Acc:HGNC:19177]
ENSG00000225031	0,20793	1,2547989	0,84893641	0,38025556	0,66191577	EIF4BP7	eukaryotic translation initiation factor 4B pseudogene 7 [Source:HGNC Symbol;Acc:HGNC:37940]
ENSG00000178093	-0,4458658	-1,5540202	0,84868576	0,38032293	0,66191775	TSSK6	testis specific serine kinase 6 [Source:HGNC Symbol;Acc:HGNC:30410]
ENSG00000148690	0,14135109	4,01073439	0,84762464	0,38060833	0,66225668	FRA10AC1	FRA10A associated CGG repeat 1 [Source:HGNC Symbol;Acc:HGNC:1162]
ENSG00000272599	0,2668666	0,35920984	0,84764363	0,38060322	0,66225668	AC016394.1	novel transcript to NUDT13
ENSG00000110697	-0,141261	4,16503503	0,84728543	0,38069964	0,66228748	PITPNM1	phosphatidylinositol transfer protein membrane associated 1 [Source:HGNC Symbol;Acc:HGNC:9003]
ENSG00000145088	-0,1539178	2,66949228	0,84721153	0,38071953	0,66228748	EAF2	ELL associated factor 2 [Source:HGNC Symbol;Acc:HGNC:23115]
ENSG00000196850	0,16361966	2,73692982	0,84693884	0,38079296	0,66228748	PPTC7	protein phosphatase targeting COQ7 [Source:HGNC Symbol;Acc:HGNC:30695]
ENSG00000247627	0,16754759	3,83444641	0,84688535	0,38080737	0,66228748	MTND4P12	MT-ND4 pseudogene 12 [Source:HGNC Symbol;Acc:HGNC:42199]
ENSG00000152076	0,70794781	-1,9631594	0,84505862	0,38129987	0,66298618	CCDC74B	coiled-coil domain containing 74B [Source:HGNC Symbol;Acc:HGNC:25267]
ENSG00000183022	0,55234271	-1,1573354	0,84519695	0,38126254	0,66298618	TPM3P8	tropomyosin 3 pseudogene 8 [Source:HGNC Symbol;Acc:HGNC:39171]
ENSG00000185504	-0,1364585	5,53973472	0,84431521	0,38150059	0,66325623	FAAP100	FA core complex associated protein 100 [Source:HGNC Symbol;Acc:HGNC:26171]

ENSG00000272288	-0,2445229	1,42365814	0,84413763	0,38154855	0,66326069	AL451165.2	novel transcript, antisense to C6orf106
ENSG00000241399	0,19638287	2,52460264	0,84371399	0,38166303	0,66330183	CD302	CD302 molecule [Source:HGNC Symbol;Acc:HGNC:30843]
ENSG00000257698	-0,3194596	0,53062567	0,84374402	0,38165491	0,66330183	GIHCG	GIHCG inhibitor of miR-200b/200a/429 expression [Source:HGNC Symbol;Acc:HGNC:52649]
ENSG00000185664	-0,3645227	-0,8297145	0,84301003	0,38185337	0,66355369	PMEL	premelanosome protein [Source:HGNC Symbol;Acc:HGNC:10880]
ENSG00000100387	0,15363383	5,66740838	0,84251793	0,38198651	0,66370611	RBX1	ring-box 1 [Source:HGNC Symbol;Acc:HGNC:9928]
ENSG00000239467	-0,4015431	-1,0694466	0,8422443	0,38206058	0,66375586	AC007405.3	glutamate rich 2 [Source:NCBI gene (formerly Entrezgene);Acc:285141]
ENSG00000119487	0,16730092	5,34778446	0,84110184	0,38237006	0,66421453	MAPKAP1	MAPK associated protein 1 [Source:HGNC Symbol;Acc:HGNC:18752]
ENSG00000174446	0,14993133	4,81070843	0,84091977	0,38241941	0,66422128	SNAPC5	small nuclear RNA activating complex polypeptide 5 [Source:HGNC Symbol;Acc:HGNC:15484]
ENSG00000233406	-0,2796625	0,04009672	0,84034238	0,382576	0,66441427	AL162430.1	ribosomal protein S2 (RPS2) pseudogene
ENSG00000163879	0,46631745	-0,0431772	0,8399021	0,38269547	0,66454275	DNALI1	dynein axonemal light intermediate chain 1 [Source:HGNC Symbol;Acc:HGNC:14353]
ENSG00000185453	-0,1971772	1,30885835	0,83921038	0,38288329	0,66478987	ZSWIM9	zinc finger SWIM-type containing 9 [Source:HGNC Symbol;Acc:HGNC:34495]
ENSG00000105397	-0,1312453	5,23245046	0,83855214	0,38306215	0,66501957	TYK2	tyrosine kinase 2 [Source:HGNC Symbol;Acc:HGNC:12440]
ENSG00000162614	-0,4700841	-0,0549811	0,83838851	0,38310663	0,66501957	NEXN	nexilin F-actin binding protein [Source:HGNC Symbol;Acc:HGNC:29557]
ENSG00000169021	0,11942663	7,36350026	0,83815675	0,38316965	0,66504994	UQCRFS1	ubiquinol-cytochrome c reductase, Rieske iron-sulfur polypeptide 1 [Source:HGNC Symbol;Acc:HGNC:12587]
ENSG00000274922	-0,2396871	0,73642201	0,83797603	0,3832188	0,66505624	AL139384.1	novel transcript
ENSG00000109072	0,19041807	7,813654	0,83628852	0,38367822	0,66563585	VTN	vitronectin [Source:HGNC Symbol;Acc:HGNC:12724]
ENSG00000225912	-0,2457644	0,39838883	0,83624723	0,38368947	0,66563585	RPL26P36	ribosomal protein L26 pseudogene 36 [Source:HGNC Symbol;Acc:HGNC:36685]
ENSG00000234199	-0,5203642	-1,0027286	0,83642473	0,3836411	0,66563585	LINC01191	long intergenic non-protein coding RNA 1191 [Source:HGNC Symbol;Acc:HGNC:49595]
ENSG00000125454	-0,1678953	5,22623792	0,83596953	0,38376516	0,66568811	SLC25A19	solute carrier family 25 member 19 [Source:HGNC Symbol;Acc:HGNC:14409]
ENSG00000107438	0,14461384	8,57097971	0,83442826	0,38418567	0,66618023	PDLIM1	PDZ and LIM domain 1 [Source:HGNC Symbol;Acc:HGNC:2067]
ENSG00000180901	0,13658775	5,24310691	0,83471269	0,38410801	0,66618023	KCTD2	potassium channel tetramerization domain containing 2 [Source:HGNC Symbol;Acc:HGNC:21294]
ENSG00000188522	-0,1512221	5,6047494	0,83443878	0,38418279	0,66618023	FAM83G	family with sequence similarity 83 member G [Source:HGNC Symbol;Acc:HGNC:32554]
ENSG00000165905	0,1877602	5,01315517	0,83383514	0,38434768	0,66638206	LARGE2	LARGE xylosyl- and glucuronyltransferase 2 [Source:HGNC Symbol;Acc:HGNC:16522]
ENSG00000177051	0,18530675	1,84611062	0,83309573	0,3845498	0,6664952	FBXO46	F-box protein 46 [Source:HGNC Symbol;Acc:HGNC:25069]
ENSG00000213722	0,15457281	4,71744708	0,83323003	0,38451308	0,6664952	DDAH2	dimethylarginine dimethylaminohydrolase 2 [Source:HGNC Symbol;Acc:HGNC:2716]
ENSG00000250746	-0,2868523	1,540037	0,83328599	0,38449778	0,6664952	AC105250.1	heat shock 70kDa protein 5 (glucose-regulated protein, 78kDa) (HSPA5) pseudogene
ENSG00000161013	-0,1204492	7,98263823	0,83252594	0,38470567	0,66668626	MGAT4B	alpha-1,3-mannosyl-glycoprotein 4-beta-N-acetylglucosaminyltransferase B [Source:HGNC Symbol;Acc:HGNC:704
ENSG00000183354	0,4286182	0,52386839	0,832293	0,38476942	0,66671034	KIAA2026	KIAA2026 [Source:HGNC Symbol;Acc:HGNC:23378]
ENSG00000205485	-0,2045218	1,07245514	0,831975	0,38485648	0,66671034	AC004980.1	pseudogene similar to part of postmeiotic segregation increased 2-like 3 PMS2L3
ENSG00000230314	0,6176096	-1,7852284	0,83211422	0,38481836	0,66671034	ELOVL2-AS1	ELOVL2 antisense RNA 1 [Source:HGNC Symbol;Acc:HGNC:44156]
ENSG00000148606	0,16567604	6,00028401	0,83166786	0,38494059	0,66677698	POLR3A	RNA polymerase III subunit A [Source:HGNC Symbol;Acc:HGNC:30074]
ENSG00000066379	-0,1336214	5,79191369	0,83105039	0,38510977	0,66693484	POLR1H	RNA polymerase I subunit H [Source:HGNC Symbol;Acc:HGNC:13182]
ENSG00000112339	0,13860178	6,18130515	0,83083549	0,38516868	0,66693484	HBS1L	HBS1 like translational GTPase [Source:HGNC Symbol;Acc:HGNC:4834]
ENSG00000135124	-0,1353725	4,52531712	0,83088074	0,38515627	0,66693484	P2RX4	purinergic receptor P2X 4 [Source:HGNC Symbol;Acc:HGNC:8535]
ENSG00000230592	-0,2571832	0,3759914	0,83053928	0,3852499	0,66699642	RPSAP8	ribosomal protein SA pseudogene 8 [Source:HGNC Symbol;Acc:HGNC:31462]
ENSG00000009307	-0,1197482	8,25696495	0,82970787	0,38547802	0,66700975	CSDE1	cold shock domain containing E1 [Source:HGNC Symbol;Acc:HGNC:29905]
ENSG00000062194	0,17153707	5,20300079	0,82971675	0,38547558	0,66700975	GPBP1	GC-rich promoter binding protein 1 [Source:HGNC Symbol;Acc:HGNC:29520]
ENSG00000147872	-0,1560849	8,26400871	0,82980123	0,38545239	0,66700975	PLIN2	perilipin 2 [Source:HGNC Symbol;Acc:HGNC:248]
ENSG00000170684	-0,2425362	2,36700712	0,82967919	0,38548589	0,66700975	ZNF296	zinc finger protein 296 [Source:HGNC Symbol;Acc:HGNC:15981]
ENSG00000229931	0,49173327	-1,060809	0,8302208	0,38533726	0,66700975	ATXN1-AS1	ATXN1 antisense RNA 1 [Source:HGNC Symbol;Acc:HGNC:40515]
ENSG00000127837	0,12539106	8,23178641	0,82937247	0,3855701	0,66702129	AAMP	angio associated migratory cell protein [Source:HGNC Symbol;Acc:HGNC:18]
ENSG00000165795	0,12716501	6,01522642	0,82915608	0,38562953	0,66702129	NDRG2	NDRG family member 2 [Source:HGNC Symbol;Acc:HGNC:14460]
ENSG00000170802	0,14340416	3,02342377	0,82916011	0,38562842	0,66702129	FOXN2	forkhead box N2 [Source:HGNC Symbol;Acc:HGNC:5281]
ENSG00000106355	-0,1797568	5,61260549	0,82897763	0,38567855	0,6670271	LSM5	LSM5 homolog, U6 small nuclear RNA and mRNA degradation associated [Source:HGNC Symbol;Acc:HGNC:17162

ENSG00000185262	0,15340987	3,89947038	0,82836433	0,38584709	0,6672396	UBALD2		UBA like domain containing 2 [Source:HGNC Symbol;Acc:HGNC:28438]
ENSG00000097007	0,14156037	5,7172986	0,82737438	0,38611939	0,66739448	ABL1		ABL proto-oncogene 1, non-receptor tyrosine kinase [Source:HGNC Symbol;Acc:HGNC:76]
ENSG00000105325	0,13050506	5,46347755	0,82781542	0,38599804	0,66739448	FZR1		fizzy and cell division cycle 20 related 1 [Source:HGNC Symbol;Acc:HGNC:24824]
ENSG00000165730	0,18868585	1,60333505	0,82753081	0,38607634	0,66739448	STOX1		storkhead box 1 [Source:HGNC Symbol;Acc:HGNC:23508]
ENSG00000167716	0,18812929	5,11570283	0,82753141	0,38607617	0,66739448	WDR81		WD repeat domain 81 [Source:HGNC Symbol;Acc:HGNC:26600]
ENSG00000117586	-0,1698023	2,05972764	0,82679543	0,38627877	0,66755154	TNFSF4		TNF superfamily member 4 [Source:HGNC Symbol;Acc:HGNC:11934]
ENSG00000217128	0,17033034	3,28290091	0,82671238	0,38630164	0,66755154	FNIP1		folliculin interacting protein 1 [Source:HGNC Symbol;Acc:HGNC:29418]
ENSG00000139719	0,15005873	4,21052978	0,8265079	0,38635796	0,6675699	VPS33A		VPS33A core subunit of CORVET and HOPS complexes [Source:HGNC Symbol;Acc:HGNC:18179]
ENSG00000100934	0,15400964	8,38975997	0,8248017	0,38682843	0,66809731	SEC23A		Sec23 homolog A, COPII coat complex component [Source:HGNC Symbol;Acc:HGNC:10701]
ENSG00000107020	-0,1685632	4,38903085	0,82477619	0,38683547	0,66809731	PLGRKT		plasminogen receptor with a C-terminal lysine [Source:HGNC Symbol;Acc:HGNC:23633]
ENSG00000168243	0,16739526	4,50833963	0,82504933	0,3867601	0,66809731	GNG4		G protein subunit gamma 4 [Source:HGNC Symbol;Acc:HGNC:4407]
ENSG00000179817	-0,6188064	-1,5662077	0,82457191	0,38689186	0,66809731	MRGPRX4		MAS related GPR family member X4 [Source:HGNC Symbol;Acc:HGNC:17617]
ENSG00000237749	-0,3203765	0,09758906	0,82473122	0,38684789	0,66809731	AL034379.1		ribosomal protein S27 (metallopanstimulin 1) (RPS27) pseudogene
ENSG00000165983	-0,1780275	1,83292876	0,8241914	0,38699693	0,6681208	PTER		phosphotriesterase related [Source:HGNC Symbol;Acc:HGNC:9590]
ENSG00000229036	-0,4748282	-1,5129385	0,82422341	0,38698809	0,6681208	VDAC1P8		voltage dependent anion channel 1 pseudogene 8 [Source:HGNC Symbol;Acc:HGNC:37483]
ENSG00000123104	-0,1774767	5,67798275	0,82349465	0,38718944	0,66837416	ITPR2		inositol 1,4,5-trisphosphate receptor type 2 [Source:HGNC Symbol;Acc:HGNC:6181]
ENSG00000104903	-0,387398	-0,1388336	0,82263663	0,38742671	0,66870473	LYL1		LYL1 basic helix-loop-helix family member [Source:HGNC Symbol;Acc:HGNC:6734]
ENSG00000130312	0,13590752	5,74510221	0,82208143	0,38758037	0,66889092	MRPL34		mitochondrial ribosomal protein L34 [Source:HGNC Symbol;Acc:HGNC:14488]
ENSG00000103363	-0,1191188	7,61017582	0,82096203	0,38789046	0,66889969	ELOB		elongin B [Source:HGNC Symbol;Acc:HGNC:11619]
ENSG00000121940	-0,1280114	5,38622107	0,82105757	0,38786398	0,66889969	CLCC1		chloride channel CLIC like 1 [Source:HGNC Symbol;Acc:HGNC:29675]
ENSG00000136811	-0,1497761	5,14743488	0,8209263	0,38790036	0,66889969	ODF2		outer dense fiber of sperm tails 2 [Source:HGNC Symbol;Acc:HGNC:8114]
ENSG00000136872	0,41656437	-1,4545412	0,82176633	0,38766762	0,66889969	ALDOB		aldolase, fructose-bisphosphate B [Source:HGNC Symbol;Acc:HGNC:417]
ENSG00000155090	0,28802023	4,75343219	0,82102225	0,38787377	0,66889969	KLF10		Kruppel like factor 10 [Source:HGNC Symbol;Acc:HGNC:11810]
ENSG00000162971	0,14244842	4,22836871	0,82081515	0,38793117	0,66889969	TYW5		tRNA-yW synthesizing protein 5 [Source:HGNC Symbol;Acc:HGNC:26754]
ENSG00000228672	-0,4794958	-1,3566056	0,8216194	0,38770831	0,66889969	PROB1		proline rich basic protein 1 [Source:HGNC Symbol;Acc:HGNC:41906]
ENSG00000232150	0,44301431	-1,2472527	0,82074093	0,38795175	0,66889969	ST13P4		ST13, Hsp70 interacting protein pseudogene 4 [Source:HGNC Symbol;Acc:HGNC:18487]
ENSG00000145191	-0,1284246	6,03005722	0,82031876	0,38806883	0,6689948	EIF2B5		eukaryotic translation initiation factor 2B subunit epsilon [Source:HGNC Symbol;Acc:HGNC:3261]
ENSG00000259605	-0,3953315	-1,1541604	0,8202118	0,3880985	0,6689948	AC074212.1		novel transcript, antisense to SIX5
ENSG00000099203	0,13087856	6,75082487	0,8193015	0,38835116	0,66919097	TMED1		transmembrane p24 trafficking protein 1 [Source:HGNC Symbol;Acc:HGNC:17291]
ENSG00000128609	0,13665579	5,56642473	0,81943123	0,38831514	0,66919097	NDUFA5		NADH:ubiquinone oxidoreductase subunit A5 [Source:HGNC Symbol;Acc:HGNC:7688]
ENSG00000135407	-0,5873963	-1,2905538	0,81880692	0,38848854	0,66919097	AVIL		advillin [Source:HGNC Symbol;Acc:HGNC:14188]
ENSG00000139405	-0,1744646	4,89613269	0,81850319	0,38857295	0,66919097	RITA1		RBPJ interacting and tubulin associated 1 [Source:HGNC Symbol;Acc:HGNC:25925]
ENSG00000165121	0,23082669	0,75798399	0,81798805	0,38871618	0,66919097	AL353743.1		kinesin family member 27 (KIF27) pseudogene
ENSG00000166004	0,15184787	2,54754633	0,81803378	0,38870346	0,66919097	CEP295		centrosomal protein 295 [Source:HGNC Symbol;Acc:HGNC:29366]
ENSG00000176903	0,12863609	5,07174659	0,81941777	0,38831888	0,66919097	PNMA1		PNMA family member 1 [Source:HGNC Symbol;Acc:HGNC:9158]
ENSG00000213189	-0,4401167	-1,4558999	0,81813653	0,38867489	0,66919097	BTF3L4P2		basic transcription factor 3 like 4 pseudogene 2 [Source:HGNC Symbol;Acc:HGNC:39646]
ENSG00000228933	0,61982287	-1,4687509	0,81820719	0,38865524	0,66919097	AC107419.1		novel transcript
ENSG00000264215	-0,4744872	-1,5111933	0,8188487	0,38847694	0,66919097	AC126365.1		novel transcript
ENSG00000276168	-0,2423984	0,72903446	0,81840419	0,38860047	0,66919097	RN7SL1		RNA component of signal recognition particle 7SL1 [Source:HGNC Symbol;Acc:HGNC:10038]
ENSG00000177842	0,18037729	2,19729136	0,81745634	0,38886409	0,66930388	ZNF620		zinc finger protein 620 [Source:HGNC Symbol;Acc:HGNC:28742]
ENSG00000224928	-0,3640132	-0,7109042	0,81742295	0,38887339	0,66930388	KRT8P30		keratin 8 pseudogene 30 [Source:HGNC Symbol;Acc:HGNC:33382]
ENSG00000138381	0,1825248	4,76470151	0,81654057	0,38911906	0,66964783	ASNSD1		asparagine synthetase domain containing 1 [Source:HGNC Symbol;Acc:HGNC:24910]
ENSG00000156171	0,1538081	4,37177393	0,81570765	0,38935119	0,66978482	DRAM2		DNA damage regulated autophagy modulator 2 [Source:HGNC Symbol;Acc:HGNC:28769]
ENSG00000169609	-0,1562484	4,07658344	0,81566922	0,38936191	0,66978482	C15orf40		chromosome 15 open reading frame 40 [Source:HGNC Symbol;Acc:HGNC:28443]

ENSG00000213087	-0,2272877	1,25402185	0,81559696	0,38938206	0,66978482	AL356535.1	ribosomal protein S12 (RPS12) pseudogene
ENSG00000233668	-0,2769492	0,06903775	0,81579415	0,38932707	0,66978482	AL353662.1	ribosomal protein S8 (RPS8) pseudogene
ENSG00000108039	-0,1653822	5,30209395	0,81472741	0,38962465	0,66980779	XPNPEP1	X-prolyl aminopeptidase 1 [Source:HGNC Symbol;Acc:HGNC:12822]
ENSG00000109846	0,31828572	-0,2866145	0,81494666	0,38956346	0,66980779	CRYAB	crystallin alpha B [Source:HGNC Symbol;Acc:HGNC:2389]
ENSG00000110171	-0,2144454	1,95817494	0,81489655	0,38957745	0,66980779	TRIM3	tripartite motif containing 3 [Source:HGNC Symbol;Acc:HGNC:10064]
ENSG00000119041	-0,1785301	5,40932741	0,81485579	0,38958882	0,66980779	GTF3C3	general transcription factor IIIC subunit 3 [Source:HGNC Symbol;Acc:HGNC:4666]
ENSG00000233873	-0,377358	-0,7375242	0,81512622	0,38951336	0,66980779	RPL7P44	ribosomal protein L7 pseudogene 44 [Source:HGNC Symbol;Acc:HGNC:36143]
ENSG00000134186	-0,1461576	5,46241449	0,81450676	0,38968625	0,66983486	PRPF38B	pre-mRNA processing factor 38B [Source:HGNC Symbol;Acc:HGNC:25512]
ENSG00000166143	-0,4970438	-1,8050373	0,81357029	0,38994785	0,67020567	PPP1R14D	protein phosphatase 1 regulatory inhibitor subunit 14D [Source:HGNC Symbol;Acc:HGNC:14953]
ENSG00000102172	0,17827759	6,65780455	0,81261014	0,39021635	0,67052238	SMS	spermine synthase [Source:HGNC Symbol;Acc:HGNC:11123]
ENSG00000134769	-0,1973178	1,91730963	0,81258309	0,39022392	0,67052238	DTNA	dystrobrevin alpha [Source:HGNC Symbol;Acc:HGNC:3057]
ENSG00000065457	-0,142286	2,80163689	0,81208529	0,39036324	0,67052596	ADAT1	adenosine deaminase tRNA specific 1 [Source:HGNC Symbol;Acc:HGNC:228]
ENSG00000169692	0,20531515	2,14371099	0,81208366	0,3903637	0,67052596	AGPAT2	1-acylglycerol-3-phosphate O-acyltransferase 2 [Source:HGNC Symbol;Acc:HGNC:325]
ENSG00000203809	-0,5338991	-1,7247494	0,81211759	0,3903542	0,67052596	LIN28B-AS1	LIN28B antisense RNA 1 [Source:HGNC Symbol;Acc:HGNC:21553]
ENSG00000003393	-0,1344407	4,03293791	0,81182541	0,39043601	0,67054196	ALS2	alsin Rho guanine nucleotide exchange factor ALS2 [Source:HGNC Symbol;Acc:HGNC:443]
ENSG00000033030	0,14670307	4,39744419	0,81172258	0,39046481	0,67054196	ZCCHC8	zinc finger CCHC-type containing 8 [Source:HGNC Symbol;Acc:HGNC:25265]
ENSG00000106415	0,34846722	-0,8118318	0,81028871	0,39086672	0,6710156	GLCCI1	glucocorticoid induced 1 [Source:HGNC Symbol;Acc:HGNC:18713]
ENSG00000274460	0,37838981	-0,2624131	0,81052911	0,3907993	0,6710156	AC092119.2	novel transcript
ENSG00000286715	-0,4763108	-1,6396764	0,81024704	0,39087841	0,6710156	AL589863.2	novel transcript
ENSG00000159685	0,13283721	4,7080497	0,80944544	0,39110339	0,67116522	CHCHD6	coiled-coil-helix-coiled-coil-helix domain containing 6 [Source:HGNC Symbol;Acc:HGNC:28184]
ENSG00000198040	0,16633924	2,79821861	0,80964425	0,39104758	0,67116522	ZNF84	zinc finger protein 84 [Source:HGNC Symbol;Acc:HGNC:13159]
ENSG00000237499	-0,4643232	-1,5194307	0,80947299	0,39109566	0,67116522	WAKMAR2	wound and keratinocyte migration associated lncRNA 2 [Source:HGNC Symbol;Acc:HGNC:53754]
ENSG00000136159	-0,1374589	5,55703504	0,8090824	0,39120536	0,6711857	NUDT15	nudix hydrolase 15 [Source:HGNC Symbol;Acc:HGNC:23063]
ENSG00000231684	-0,2850918	0,01177445	0,80907577	0,39120722	0,6711857	EIF1P3	eukaryotic translation initiation factor 1 pseudogene 3 [Source:HGNC Symbol;Acc:HGNC:49616]
ENSG00000116661	0,18767847	6,59160457	0,80871843	0,39130762	0,67127912	FBXO2	F-box protein 2 [Source:HGNC Symbol;Acc:HGNC:13581]
ENSG00000181826	-0,1529848	3,20840672	0,80821808	0,39144827	0,67144156	RELL1	RELT like 1 [Source:HGNC Symbol;Acc:HGNC:27379]
ENSG00000039123	0,16878655	5,62250927	0,80772782	0,39158616	0,67146893	MTREX	Mtr4 exosome RNA helicase [Source:HGNC Symbol;Acc:HGNC:18734]
ENSG00000078403	0,13274157	3,99942771	0,80749734	0,39165101	0,67146893	MLLT10	MLLT10 histone lysine methyltransferase DOT1L cofactor [Source:HGNC Symbol;Acc:HGNC:16063]
ENSG00000137338	0,14617287	3,13228651	0,80701792	0,39178596	0,67146893	PGBD1	piggyBac transposable element derived 1 [Source:HGNC Symbol;Acc:HGNC:19398]
ENSG00000138757	0,14932393	6,64462712	0,80712281	0,39175643	0,67146893	G3BP2	G3BP stress granule assembly factor 2 [Source:HGNC Symbol;Acc:HGNC:30291]
ENSG00000196535	-0,3982588	4,933561	0,80726359	0,3917168	0,67146893	MYO18A	myosin XVIIIA [Source:HGNC Symbol;Acc:HGNC:31104]
ENSG00000227827	0,36788987	0,28702817	0,8074901	0,39165305	0,67146893	PKD1P2	polycystin 1, transient receptor potential channel interacting pseudogene 2 [Source:HGNC Symbol;Acc:HGNC:300
ENSG00000231340	-0,4409687	-1,2593717	0,8074409	0,39166689	0,67146893	ACTG1P10	actin gamma 1 pseudogene 10 [Source:HGNC Symbol;Acc:HGNC:155]
ENSG00000187045	-0,1730449	2,61855746	0,80663324	0,3918943	0,67150771	TMPRSS6	transmembrane serine protease 6 [Source:HGNC Symbol;Acc:HGNC:16517]
ENSG00000270532	-0,2520585	0,28537279	0,80661114	0,39190052	0,67150771	PEBP1P2	phosphatidylethanolamine binding protein 1 pseudogene 2 [Source:HGNC Symbol;Acc:HGNC:44344]
ENSG00000134247	-0,1926122	4,68287293	0,80630019	0,39198813	0,67157905	PTGFRN	prostaglandin F2 receptor inhibitor [Source:HGNC Symbol;Acc:HGNC:9601]
ENSG00000164683	-0,3887732	0,04383645	0,8060224	0,39206642	0,67163442	HEY1	hes related family bHLH transcription factor with YRPW motif 1 [Source:HGNC Symbol;Acc:HGNC:4880]
ENSG00000188997	0,17810994	3,04398004	0,80578847	0,39213237	0,67166863	KCTD21	potassium channel tetramerization domain containing 21 [Source:HGNC Symbol;Acc:HGNC:27452]
ENSG00000100258	-0,1478175	6,24281371	0,8047368	0,39242907	0,67209804	LMF2	lipase maturation factor 2 [Source:HGNC Symbol;Acc:HGNC:25096]
ENSG00000107521	0,17425391	4,81199015	0,80400138	0,39263676	0,67233781	HPS1	HPS1 biogenesis of lysosomal organelles complex 3 subunit 1 [Source:HGNC Symbol;Acc:HGNC:5163]
ENSG00000170265	0,1516936	5,08698926	0,80391518	0,39266111	0,67233781	ZNF282	zinc finger protein 282 [Source:HGNC Symbol;Acc:HGNC:13076]
ENSG00000029153	0,15946148	3,44453522	0,80343735	0,39279616	0,67249023	ARNTL2	aryl hydrocarbon receptor nuclear translocator like 2 [Source:HGNC Symbol;Acc:HGNC:18984]
ENSG00000224831	-0,5085122	-1,621564	0,80229732	0,39311866	0,6729635	TMEM183B	transmembrane protein 183B [Source:HGNC Symbol;Acc:HGNC:33205]
ENSG00000074755	0,22601922	4,10004365	0,80173263	0,39327856	0,67308648	ZZEF1	zinc finger ZZ-type and EF-hand domain containing 1 [Source:HGNC Symbol;Acc:HGNC:29027]

ENSG00000178104	-0,1436823	3,40124795	0,80171819	0,39328265	0,67308648	PDE4DIP	phosphodiesterase 4D interacting protein [Source:HGNC Symbol;Acc:HGNC:15580]
ENSG00000135423	-0,1723523	3,54691884	0,80067078	0,39357951	0,67343677	GLS2	glutaminase 2 [Source:HGNC Symbol;Acc:HGNC:29570]
ENSG00000233778	-0,4453922	-0,8894206	0,80080652	0,39354102	0,67343677	AC104339.1	ribosomal protein S26 (RPS26) pseudogene
ENSG00000236810	-0,2821544	0,33770791	0,80040748	0,39365419	0,67348567	ELOA-AS1	ELOA antisense RNA 1 [Source:HGNC Symbol;Acc:HGNC:50582]
ENSG00000189067	-0,1713009	7,38954132	0,79985914	0,39380979	0,67367298	LITAF	lipopolysaccharide induced TNF factor [Source:HGNC Symbol;Acc:HGNC:16841]
ENSG00000076242	-0,1477453	5,71005083	0,799047	0,39404043	0,67383081	MLH1	mutL homolog 1 [Source:HGNC Symbol;Acc:HGNC:7127]
ENSG00000182827	0,20653336	4,72344528	0,79920365	0,39399593	0,67383081	ACBD3	acyl-CoA binding domain containing 3 [Source:HGNC Symbol;Acc:HGNC:15453]
ENSG00000232593	0,36303696	-0,3885631	0,7991029	0,39402455	0,67383081	KANTR	KDM5C adjacent transcript [Source:HGNC Symbol;Acc:HGNC:49510]
ENSG00000165630	0,14865503	2,5331397	0,79861699	0,39416263	0,67391496	PRPF18	pre-mRNA processing factor 18 [Source:HGNC Symbol;Acc:HGNC:17351]
ENSG00000176076	-0,5989543	-1,6973513	0,79854923	0,39418189	0,67391496	KCNE5	potassium voltage-gated channel subfamily E regulatory subunit 5 [Source:HGNC Symbol;Acc:HGNC:6241]
ENSG00000158161	0,15559641	3,82236111	0,79827445	0,39426002	0,67393195	EYA3	EYA transcriptional coactivator and phosphatase 3 [Source:HGNC Symbol;Acc:HGNC:3521]
ENSG00000214456	-0,4025015	-0,9981546	0,79818978	0,3942841	0,67393195	PLIN5	perilipin 5 [Source:HGNC Symbol;Acc:HGNC:33196]
ENSG00000219507	0,1904472	1,85046483	0,79725107	0,3945512	0,67423073	FTH1P8	ferritin heavy chain 1 pseudogene 8 [Source:HGNC Symbol;Acc:HGNC:3995]
ENSG00000243181	-0,3631605	-1,0226326	0,79741207	0,39450537	0,67423073	AC087343.1	ribosomal protein L21 (RPl21) pseudogene
ENSG00000100889	-0,2581121	9,18835576	0,79702809	0,39461469	0,67426036	PCK2	phosphoenolpyruvate carboxykinase 2, mitochondrial [Source:HGNC Symbol;Acc:HGNC:8725]
ENSG00000141994	-0,1401894	4,52493298	0,79682583	0,3946723	0,67427992	DUS3L	dihydrouridine synthase 3 like [Source:HGNC Symbol;Acc:HGNC:26920]
ENSG00000129596	-0,2315145	4,08024792	0,79661222	0,39473315	0,67430502	CDO1	cysteine dioxygenase type 1 [Source:HGNC Symbol;Acc:HGNC:1795]
ENSG00000162650	-0,1759792	2,23804605	0,79627992	0,39482784	0,67438792	ATXN7L2	ataxin 7 like 2 [Source:HGNC Symbol;Acc:HGNC:28713]
ENSG00000177663	0,17484082	3,99735569	0,7956119	0,39501831	0,67463437	IL17RA	interleukin 17 receptor A [Source:HGNC Symbol;Acc:HGNC:5985]
ENSG00000168291	0,13567443	7,32684372	0,79522427	0,39512889	0,6746655	PDHB	pyruvate dehydrogenase E1 subunit beta [Source:HGNC Symbol;Acc:HGNC:8808]
ENSG00000174851	-0,1279746	7,96413783	0,79525292	0,39512072	0,6746655	YIF1A	Yip1 interacting factor homolog A, membrane trafficking protein [Source:HGNC Symbol;Acc:HGNC:16688]
ENSG00000123094	0,55331394	-1,2473458	0,79458685	0,39531085	0,6748973	RASSF8	Ras association domain family member 8 [Source:HGNC Symbol;Acc:HGNC:13232]
ENSG00000225361	-0,3177702	-0,1977052	0,79416478	0,3954314	0,67502423	PPP1R26-AS1	PPP1R26 antisense RNA 1 [Source:HGNC Symbol;Acc:HGNC:48717]
ENSG00000001167	0,1504073	5,40518974	0,79283692	0,39581106	0,67559339	NFYA	nuclear transcription factor Y subunit alpha [Source:HGNC Symbol;Acc:HGNC:7804]
ENSG00000183935	0,3939291	-1,3730086	0,7923347	0,3959548	0,67575978	HTR7P1	5-hydroxytryptamine receptor 7 pseudogene 1 [Source:HGNC Symbol;Acc:HGNC:30411]
ENSG00000172731	0,14748533	5,91857273	0,79207468	0,39602926	0,6758079	LRRC20	leucine rich repeat containing 20 [Source:HGNC Symbol;Acc:HGNC:23421]
ENSG00000136010	-0,4615811	1,03792246	0,79181186	0,39610453	0,67585741	ALDH1L2	aldehyde dehydrogenase 1 family member L2 [Source:HGNC Symbol;Acc:HGNC:26777]
ENSG00000134508	0,11912273	5,55345918	0,7912631	0,39626178	0,67604676	CABLES1	Cdk5 and Abl enzyme substrate 1 [Source:HGNC Symbol;Acc:HGNC:25097]
ENSG00000117640	0,19377414	4,59402509	0,79050559	0,39647902	0,67625942	MTFR1L	mitochondrial fission regulator 1 like [Source:HGNC Symbol;Acc:HGNC:28836]
ENSG00000136824	0,19561752	4,22875952	0,7906395	0,3964406	0,67625942	SMC2	structural maintenance of chromosomes 2 [Source:HGNC Symbol;Acc:HGNC:14011]
ENSG00000129347	-0,2946462	4,51729263	0,78983981	0,3966701	0,6764732	KRI1	KRI1 homolog [Source:HGNC Symbol;Acc:HGNC:25769]
ENSG00000187193	-0,192304	3,88818491	0,78974626	0,39669696	0,6764732	MT1X	metallothionein 1X [Source:HGNC Symbol;Acc:HGNC:7405]
ENSG00000120694	0,21064335	7,49521491	0,78952463	0,39676061	0,67650277	HSPH1	heat shock protein family H (Hsp110) member 1 [Source:HGNC Symbol;Acc:HGNC:16969]
ENSG00000280087	0,28484164	0,35690151	0,78932341	0,39681841	0,67652236	AC011481.3	TEC
ENSG00000136280	0,13284451	4,91867078	0,78812579	0,3971627	0,67670076	CCM2	CCM2 scaffold protein [Source:HGNC Symbol;Acc:HGNC:21708]
ENSG00000143793	-0,1408511	4,13482826	0,78863564	0,39701607	0,67670076	C1orf35	chromosome 1 open reading frame 35 [Source:HGNC Symbol;Acc:HGNC:19032]
ENSG00000165886	-0,1637809	3,185959	0,78801262	0,39719526	0,67670076	UBTD1	ubiquitin domain containing 1 [Source:HGNC Symbol;Acc:HGNC:25683]
ENSG00000185485	-0,1477589	2,48323938	0,78836626	0,39709353	0,67670076	SDHAP1	succinate dehydrogenase complex flavoprotein subunit A pseudogene 1 [Source:HGNC Symbol;Acc:HGNC:32455]
ENSG00000224520	-0,2496522	1,59009725	0,78832254	0,39710611	0,67670076	KRT8P45	keratin 8 pseudogene 45 [Source:HGNC Symbol;Acc:HGNC:39879]
ENSG00000233901	-0,4687283	-1,2567059	0,78799276	0,39720097	0,67670076	LINC01503	long intergenic non-protein coding RNA 1503 [Source:HGNC Symbol;Acc:HGNC:51184]
ENSG00000094841	0,21302958	2,73720482	0,78777054	0,39726492	0,67673078	UPRT	uracil phosphoribosyltransferase homolog [Source:HGNC Symbol;Acc:HGNC:28334]
ENSG00000205913	-0,5287621	-1,7572312	0,78749832	0,39734327	0,67678534	SRRM2-AS1	SRRM2 antisense RNA 1 [Source:HGNC Symbol;Acc:HGNC:44162]
ENSG00000104879	0,22329913	1,63629208	0,78717031	0,39743772	0,67678839	CKM	creatine kinase, M-type [Source:HGNC Symbol;Acc:HGNC:1994]
ENSG00000224080	-0,4786284	-1,8740011	0,78731499	0,39739605	0,67678839	UBE2FP1	UBE2F pseudogene 1 [Source:HGNC Symbol;Acc:HGNC:44535]

ENSG00000150768	-0,1293566	6,31911362	0,78660657	0,39760012	0,67685092	DLAT	dihydrolipoamide S-acetyltransferase [Source:HGNC Symbol;Acc:HGNC:2896]
ENSG00000225680	-0,4501903	-1,376198	0,7865604	0,39761343	0,67685092	AL163953.1	novel transcript
ENSG00000244041	-0,5736052	-1,5985553	0,78656204	0,39761296	0,67685092	LINC01011	long intergenic non-protein coding RNA 1011 [Source:HGNC Symbol;Acc:HGNC:33812]
ENSG00000067596	0,17764517	6,03040501	0,78450191	0,39820737	0,67762511	DHX8	DEAH-box helicase 8 [Source:HGNC Symbol;Acc:HGNC:2749]
ENSG00000130684	0,12993315	4,40387346	0,78457716	0,39818564	0,67762511	ZNF337	zinc finger protein 337 [Source:HGNC Symbol;Acc:HGNC:15809]
ENSG00000273899	0,32103735	-0,0987437	0,7847701	0,39812991	0,67762511	NOL12	nucleolar protein 12 [Source:HGNC Symbol;Acc:HGNC:28585]
ENSG00000166477	0,17953743	6,64305147	0,78426183	0,39827673	0,6776642	LEO1	LEO1 homolog, Paf1/RNA polymerase II complex component [Source:HGNC Symbol;Acc:HGNC:30401]
ENSG00000230844	-0,1848148	1,74886037	0,7838319	0,398401	0,67776209	ZNF674-AS1	ZNF674 antisense RNA 1 (head to head) [Source:HGNC Symbol;Acc:HGNC:44266]
ENSG00000241549	-0,5182328	-1,8589129	0,78374177	0,39842705	0,67776209	GUSBP2	GUSB pseudogene 2 [Source:HGNC Symbol;Acc:HGNC:18792]
ENSG00000101473	-0,1529927	5,13617869	0,78338932	0,39852898	0,67785654	ACOT8	acyl-CoA thioesterase 8 [Source:HGNC Symbol;Acc:HGNC:15919]
ENSG00000167658	-0,1464371	11,4066319	0,78286206	0,39868153	0,67803707	EEF2	eukaryotic translation elongation factor 2 [Source:HGNC Symbol;Acc:HGNC:3214]
ENSG00000162241	0,26104959	0,15611308	0,78177731	0,39899567	0,67849235	SLC25A45	solute carrier family 25 member 45 [Source:HGNC Symbol;Acc:HGNC:27442]
ENSG00000173230	0,20131397	4,80955745	0,78146246	0,39908693	0,67856854	GOLGB1	golgin B1 [Source:HGNC Symbol;Acc:HGNC:4429]
ENSG00000176834	0,14971122	3,26222113	0,78094347	0,39923742	0,67874543	VSIG10	V-set and immunoglobulin domain containing 10 [Source:HGNC Symbol;Acc:HGNC:26078]
ENSG00000227077	-0,4144099	-1,5524381	0,7805503	0,39935149	0,67886036	AC107983.1	ribosomal protein S28 (RPS28) pseudogene
ENSG00000120896	-0,1334394	5,31506878	0,7801443	0,39946933	0,67898167	SORBS3	sorbin and SH3 domain containing 3 [Source:HGNC Symbol;Acc:HGNC:30907]
ENSG00000032219	-0,2002174	2,70808388	0,77957655	0,39963422	0,67910391	ARID4A	AT-rich interaction domain 4A [Source:HGNC Symbol;Acc:HGNC:9885]
ENSG00000267296	-0,1844896	2,5705763	0,77966657	0,39960807	0,67910391	CEBPA-DT	CEBPA divergent transcript [Source:HGNC Symbol;Acc:HGNC:25710]
ENSG00000065060	0,17571696	4,56374045	0,7785806	0,39992372	0,6791219	UHRF1BP1	UHRF1 binding protein 1 [Source:HGNC Symbol;Acc:HGNC:21216]
ENSG00000082213	0,15294119	3,75985306	0,77875232	0,39987379	0,6791219	C5orf22	chromosome 5 open reading frame 22 [Source:HGNC Symbol;Acc:HGNC:25639]
ENSG00000091732	0,12819087	5,24851064	0,77896718	0,39981131	0,6791219	ZC3HC1	zinc finger C3HC-type containing 1 [Source:HGNC Symbol;Acc:HGNC:29913]
ENSG00000131873	0,14605837	3,90769852	0,77922672	0,39973587	0,6791219	CHSY1	chondroitin sulfate synthase 1 [Source:HGNC Symbol;Acc:HGNC:17198]
ENSG00000148337	-0,1321714	5,87085734	0,77866332	0,39989967	0,6791219	CIZ1	CDKN1A interacting zinc finger protein 1 [Source:HGNC Symbol;Acc:HGNC:16744]
ENSG00000153406	-0,1486082	4,76919891	0,7788935	0,39983273	0,6791219	NMRAL1	NmrA like redox sensor 1 [Source:HGNC Symbol;Acc:HGNC:24987]
ENSG00000115317	-0,1164285	5,98155796	0,77831279	0,40000163	0,67915835	HTRA2	HtrA serine peptidase 2 [Source:HGNC Symbol;Acc:HGNC:14348]
ENSG00000167272	-0,1306756	5,95185903	0,77818721	0,40003817	0,67915835	POP5	POP5 homolog, ribonuclease P/MRP subunit [Source:HGNC Symbol;Acc:HGNC:17689]
ENSG00000214944	0,18593476	4,92846126	0,77730947	0,4002937	0,67943426	ARHGEF28	Rho guanine nucleotide exchange factor 28 [Source:HGNC Symbol;Acc:HGNC:30322]
ENSG00000225159	-0,2210999	0,93957772	0,77737671	0,40027412	0,67943426	NPM1P39	nucleophosmin 1 pseudogene 39 [Source:HGNC Symbol;Acc:HGNC:45218]
ENSG00000111877	-0,1401584	2,98284303	0,77637047	0,40056736	0,67981977	MCM9	minichromosome maintenance 9 homologous recombination repair factor [Source:HGNC Symbol;Acc:HGNC:2148
ENSG00000174780	-0,1283864	6,32770428	0,77578288	0,40073875	0,68003165	SRP72	signal recognition particle 72 [Source:HGNC Symbol;Acc:HGNC:11303]
ENSG00000101182	-0,1169447	9,51260044	0,77458	0,40108999	0,6800326	PSMA7	proteasome 20S subunit alpha 7 [Source:HGNC Symbol;Acc:HGNC:9536]
ENSG00000124160	0,13501998	5,67099572	0,7743464	0,40115826	0,6800326	NCOA5	nuclear receptor coactivator 5 [Source:HGNC Symbol;Acc:HGNC:15909]
ENSG00000147687	-0,1461763	3,93706403	0,77476455	0,40103607	0,6800326	TATDN1	TatD DNase domain containing 1 [Source:HGNC Symbol;Acc:HGNC:24220]
ENSG00000178719	-0,1191301	7,82559804	0,77482603	0,40101811	0,6800326	GRINA	glutamate ionotropic receptor NMDA type subunit associated protein 1 [Source:HGNC Symbol;Acc:HGNC:4589]
ENSG00000179295	-0,1832609	5,78393966	0,77542162	0,40084419	0,6800326	PTPN11	protein tyrosine phosphatase non-receptor type 11 [Source:HGNC Symbol;Acc:HGNC:9644]
ENSG00000196756	-0,1418372	5,08226737	0,77510566	0,40093644	0,6800326	SNHG17	small nucleolar RNA host gene 17 [Source:HGNC Symbol;Acc:HGNC:48600]
ENSG00000204778	-0,385661	-1,1710188	0,77448284	0,40111838	0,6800326	CBWD4P	COBW domain containing 4, pseudogene [Source:HGNC Symbol;Acc:HGNC:18520]
ENSG00000273906	-0,3568955	0,1875794	0,77545477	0,40083451	0,6800326	AC011297.1	novel transcript, sense intronic to TBL1Y
ENSG00000279133	0,67527926	-0,6410611	0,77465405	0,40106835	0,6800326	AC018628.1	TEC
ENSG00000125505	-0,1218615	5,84930212	0,77355197	0,40139055	0,6801461	MBOAT7	membrane bound O-acyltransferase domain containing 7 [Source:HGNC Symbol;Acc:HGNC:15505]
ENSG00000149634	-0,5945921	-0,6454335	0,77354813	0,40139168	0,6801461	SPATA25	spermatogenesis associated 25 [Source:HGNC Symbol;Acc:HGNC:16158]
ENSG00000204815	-0,4799906	-0,9840974	0,77348059	0,40141144	0,6801461	TTC25	tetratricopeptide repeat domain 25 [Source:HGNC Symbol;Acc:HGNC:25280]
ENSG00000245293	0,51099255	-1,579401	0,77349627	0,40140685	0,6801461	CYP2U1-AS1	CYP2U1 and SGMS2 antisense RNA 1 [Source:HGNC Symbol;Acc:HGNC:54817]
ENSG00000239269	-0,4092934	-1,3943182	0,77315447	0,40150687	0,6802289	RPSAP4	ribosomal protein SA pseudogene 4 [Source:HGNC Symbol;Acc:HGNC:20018]

ENSG00000134758	-0,1683301	3,06786664	0,7723222	0,40175057	0,68024735	RNF138	ring finger protein 138 [Source:HGNC Symbol;Acc:HGNC:17765]
ENSG00000148459	-0,1274799	4,14194819	0,77270424	0,40163867	0,68024735	PDSS1	decaprenyl diphosphate synthase subunit 1 [Source:HGNC Symbol;Acc:HGNC:17759]
ENSG00000164654	-0,1392952	3,89625356	0,77282442	0,40160348	0,68024735	MIOS	meiosis regulator for oocyte development [Source:HGNC Symbol;Acc:HGNC:21905]
ENSG00000213341	-0,1264766	4,53789091	0,77246365	0,40170914	0,68024735	CHUK	component of inhibitor of nuclear factor kappa B kinase complex [Source:HGNC Symbol;Acc:HGNC:1974]
ENSG00000244694	-0,1484678	3,35700585	0,77250218	0,40169785	0,68024735	PTCHD4	patched domain containing 4 [Source:HGNC Symbol;Acc:HGNC:21345]
ENSG00000106771	0,13009701	5,65225252	0,77168225	0,40193812	0,68032316	TMEM245	transmembrane protein 245 [Source:HGNC Symbol;Acc:HGNC:1363]
ENSG00000109758	-0,1584497	2,09947609	0,77140807	0,40201852	0,68032316	HGFAC	HGF activator [Source:HGNC Symbol;Acc:HGNC:4894]
ENSG00000120262	0,26116281	0,47849341	0,77160833	0,4019598	0,68032316	CCDC170	coiled-coil domain containing 170 [Source:HGNC Symbol;Acc:HGNC:21177]
ENSG00000123989	0,12483543	8,57899243	0,77186625	0,40188418	0,68032316	CHPF	chondroitin polymerizing factor [Source:HGNC Symbol;Acc:HGNC:24291]
ENSG00000170638	-0,1368806	5,17363801	0,77137509	0,40202819	0,68032316	TRABD	TraB domain containing [Source:HGNC Symbol;Acc:HGNC:28805]
ENSG00000137221	0,14583717	4,27853005	0,77057584	0,40226271	0,68064117	TJAP1	tight junction associated protein 1 [Source:HGNC Symbol;Acc:HGNC:17949]
ENSG00000114904	0,15851035	4,6197642	0,76983225	0,40248109	0,68087044	NEK4	NIMA related kinase 4 [Source:HGNC Symbol;Acc:HGNC:11399]
ENSG00000134717	0,1489752	5,49443508	0,76979708	0,40249142	0,68087044	BTF3L4	basic transcription factor 3 like 4 [Source:HGNC Symbol;Acc:HGNC:30547]
ENSG00000095794	0,13969661	3,61482096	0,76954943	0,40256419	0,6809147	CREM	cAMP responsive element modulator [Source:HGNC Symbol;Acc:HGNC:2352]
ENSG00000140829	0,16049695	6,250301	0,76909341	0,40269825	0,68106261	DHX38	DEAH-box helicase 38 [Source:HGNC Symbol;Acc:HGNC:17211]
ENSG00000135686	-0,1494815	2,86553563	0,7688705	0,40276381	0,68109463	KLHL36	kelch like family member 36 [Source:HGNC Symbol;Acc:HGNC:17844]
ENSG00000100911	0,11839321	8,97007402	0,76794169	0,40303716	0,68134004	PSME2	proteasome activator subunit 2 [Source:HGNC Symbol;Acc:HGNC:9569]
ENSG00000157954	0,17479698	5,39869723	0,76716692	0,4032654	0,68134004	WIPI2	WD repeat domain, phosphoinositide interacting 2 [Source:HGNC Symbol;Acc:HGNC:32225]
ENSG00000198342	0,2116188	1,36268762	0,76745681	0,40317997	0,68134004	ZNF442	zinc finger protein 442 [Source:HGNC Symbol;Acc:HGNC:20877]
ENSG00000223891	0,25156884	0,75387008	0,76785793	0,40306182	0,68134004	OSER1-DT	OSER1 divergent transcript [Source:HGNC Symbol;Acc:HGNC:48585]
ENSG00000233325	0,61448013	-1,8433388	0,76723607	0,40324502	0,68134004	MIPEPP3	mitochondrial intermediate peptidase pseudogene 3 [Source:HGNC Symbol;Acc:HGNC:39458]
ENSG00000261221	-0,2552548	1,78557163	0,76711045	0,40328204	0,68134004	ZNF865	zinc finger protein 865 [Source:HGNC Symbol;Acc:HGNC:38705]
ENSG00000273149	-0,2087073	1,05150169	0,76739679	0,40319766	0,68134004	AL138963.3	novel transcript, antisense to TPT1
ENSG00000275688	0,47044008	-1,7797243	0,76778683	0,40308276	0,68134004	CCL15-CCL14	CCL15-CCL14 readthrough (NMD candidate) [Source:HGNC Symbol;Acc:HGNC:44436]
ENSG00000044090	0,17143773	5,67267719	0,76657647	0,40343947	0,68136963	CUL7	cullin 7 [Source:HGNC Symbol;Acc:HGNC:21024]
ENSG00000183666	0,22715075	1,60922522	0,76672243	0,40339643	0,68136963	GUSBP1	GUSB pseudogene 1 [Source:HGNC Symbol;Acc:HGNC:13670]
ENSG00000276043	-0,2128876	6,02373875	0,76659978	0,4034326	0,68136963	UHRF1	ubiquitin like with PHD and ring finger domains 1 [Source:HGNC Symbol;Acc:HGNC:12556]
ENSG00000132825	-0,1812078	1,66745678	0,76611336	0,40357609	0,68152157	PPP1R3D	protein phosphatase 1 regulatory subunit 3D [Source:HGNC Symbol;Acc:HGNC:9294]
ENSG00000241388	0,48085292	-0,7402459	0,76592284	0,40363232	0,68153774	HNF1A-AS1	HNF1A antisense RNA 1 [Source:HGNC Symbol;Acc:HGNC:26785]
ENSG00000231940	0,25592645	0,40494766	0,76569828	0,4036986	0,68157089	RPS7P3	ribosomal protein S7 pseudogene 3 [Source:HGNC Symbol;Acc:HGNC:36518]
ENSG00000140632	0,20893195	6,19045301	0,76485379	0,40394804	0,68191321	GLYR1	glyoxylate reductase 1 homolog [Source:HGNC Symbol;Acc:HGNC:24434]
ENSG00000067113	-0,1366395	3,84112126	0,76391676	0,4042251	0,68230208	PLPP1	phospholipid phosphatase 1 [Source:HGNC Symbol;Acc:HGNC:9228]
ENSG00000114480	0,14740886	4,33584963	0,76358544	0,40432313	0,68230988	GBE1	1,4-alpha-glucan branching enzyme 1 [Source:HGNC Symbol;Acc:HGNC:4180]
ENSG00000143379	-0,1333205	4,60651714	0,76358939	0,40432196	0,68230988	SETDB1	SET domain bifurcated histone lysine methyltransferase 1 [Source:HGNC Symbol;Acc:HGNC:10761]
ENSG00000244005	0,14384621	5,55561254	0,76307022	0,40447566	0,68248844	NFS1	NFS1 cysteine desulfurase [Source:HGNC Symbol;Acc:HGNC:15910]
ENSG00000147257	0,16399179	10,9645632	0,76284772	0,40454156	0,6825208	GPC3	glypican 3 [Source:HGNC Symbol;Acc:HGNC:4451]
ENSG00000186665	-0,1750701	3,88541339	0,76256423	0,40462554	0,68258367	C17orf58	chromosome 17 open reading frame 58 [Source:HGNC Symbol;Acc:HGNC:27568]
ENSG00000167604	0,28024254	-0,1237453	0,76232355	0,40469687	0,68262517	NFKBID	NFKB inhibitor delta [Source:HGNC Symbol;Acc:HGNC:15671]
ENSG00000182795	-0,1968029	1,97116265	0,76200457	0,40479143	0,68270584	C1orf116	chromosome 1 open reading frame 116 [Source:HGNC Symbol;Acc:HGNC:28667]
ENSG00000153885	-0,138288	3,94267196	0,76142294	0,40496394	0,68291795	KCTD15	potassium channel tetramerization domain containing 15 [Source:HGNC Symbol;Acc:HGNC:23297]
ENSG00000198668	-0,1887589	6,81949417	0,75996314	0,40539743	0,68357007	CALM1	calmodulin 1 [Source:HGNC Symbol;Acc:HGNC:1442]
ENSG00000099995	-0,1927737	6,49392679	0,75959543	0,40550674	0,68367548	SF3A1	splicing factor 3a subunit 1 [Source:HGNC Symbol;Acc:HGNC:10765]
ENSG00000100220	0,15626511	7,33534452	0,75913665	0,40564319	0,68379662	RTCB	RNA 2',3'-cyclic phosphate and 5'-OH ligase [Source:HGNC Symbol;Acc:HGNC:26935]
ENSG00000117543	0,11303421	6,59004094	0,75903911	0,40567221	0,68379662	DPH5	diphthamide biosynthesis 5 [Source:HGNC Symbol;Acc:HGNC:24270]

ENSG00000240288	-0,4524506	-1,5696604	0,75848046	0,40583848	0,68399796	GHRLOS	ghrelin opposite strand/antisense RNA [Source:HGNC Symbol;Acc:HGNC:33885]
ENSG00000267541	0,16782584	1,63941361	0,75739918	0,40616061	0,68446191	MTCO2P2	MT-CO2 pseudogene 2 [Source:HGNC Symbol;Acc:HGNC:25354]
ENSG00000115306	0,31998373	6,68463993	0,75590436	0,4066066	0,68473958	SPTBN1	spectrin beta, non-erythrocytic 1 [Source:HGNC Symbol;Acc:HGNC:11275]
ENSG00000136152	0,12689097	4,36833376	0,7559336	0,40659787	0,68473958	COG3	component of oligomeric golgi complex 3 [Source:HGNC Symbol;Acc:HGNC:18619]
ENSG00000159267	-0,198833	3,95156559	0,75598495	0,40658254	0,68473958	HLCS	holocarboxylase synthetase [Source:HGNC Symbol;Acc:HGNC:4976]
ENSG00000168883	-0,1215517	6,74350371	0,75626177	0,4064999	0,68473958	USP39	ubiquitin specific peptidase 39 [Source:HGNC Symbol;Acc:HGNC:20071]
ENSG00000188243	-0,2021999	3,57042722	0,75645523	0,40644215	0,68473958	COMMD6	COMM domain containing 6 [Source:HGNC Symbol;Acc:HGNC:24015]
ENSG00000223509	0,26529787	0,316935	0,7565411	0,40641653	0,68473958	AC135983.2	WAS protein homolog associated with actin, golgi membranes and microtubules (WHAMM) pseudogene
ENSG00000272150	0,5737145	-0,7546499	0,75574325	0,40665472	0,68474168	NBPF25P	NBPF member 25, pseudogene [Source:HGNC Symbol;Acc:HGNC:45046]
ENSG00000160877	-0,1691513	5,31010398	0,75546079	0,4067391	0,68480483	NACC1	nucleus accumbens associated 1 [Source:HGNC Symbol;Acc:HGNC:20967]
ENSG00000145979	0,17468276	4,96858722	0,75441092	0,40705297	0,6850975	TBC1D7	TBC1 domain family member 7 [Source:HGNC Symbol;Acc:HGNC:21066]
ENSG00000198556	0,17428822	2,64977769	0,75440877	0,40705362	0,6850975	ZNF789	zinc finger protein 789 [Source:HGNC Symbol;Acc:HGNC:27801]
ENSG00000272674	0,47053872	-1,0052177	0,75467604	0,40697367	0,6850975	PCDHB16	protocadherin beta 16 [Source:HGNC Symbol;Acc:HGNC:14546]
ENSG00000196141	-0,1216453	8,04217409	0,75385403	0,40721962	0,685218	SPATS2L	spermatogenesis associated serine rich 2 like [Source:HGNC Symbol;Acc:HGNC:24574]
ENSG00000214022	0,1243512	6,25875077	0,75369936	0,40726592	0,685218	REPIN1	replication initiator 1 [Source:HGNC Symbol;Acc:HGNC:17922]
ENSG00000258430	-0,6295841	-1,5711407	0,75397266	0,40718411	0,685218	AL583722.1	novel transcript, antisense to AKT1
ENSG00000071626	-0,1194891	7,26344787	0,75343744	0,40734435	0,68525494	DAZAP1	DAZ associated protein 1 [Source:HGNC Symbol;Acc:HGNC:2683]
ENSG00000147140	0,16960717	8,69407652	0,75315619	0,4074286	0,68525494	NONO	non-POU domain containing octamer binding [Source:HGNC Symbol;Acc:HGNC:7871]
ENSG00000221890	0,24093977	3,46618649	0,75319441	0,40741715	0,68525494	NPTXR	neuronal pentraxin receptor [Source:HGNC Symbol;Acc:HGNC:7954]
ENSG00000070761	0,15890681	6,75914679	0,75284194	0,40752276	0,68530548	CFAP20	cilia and flagella associated protein 20 [Source:HGNC Symbol;Acc:HGNC:29523]
ENSG00000158201	0,15549777	5,36138022	0,75258633	0,40759938	0,68530548	ABHD3	abhydrolase domain containing 3, phospholipase [Source:HGNC Symbol;Acc:HGNC:18718]
ENSG00000179431	-0,2206514	1,78357802	0,75259585	0,40759653	0,68530548	FJX1	four-jointed box kinase 1 [Source:HGNC Symbol;Acc:HGNC:17166]
ENSG00000075239	0,15248895	7,54850237	0,75213732	0,40773403	0,68537411	ACAT1	acetyl-CoA acetyltransferase 1 [Source:HGNC Symbol;Acc:HGNC:93]
ENSG00000116688	0,14199514	6,47645873	0,75214468	0,40773182	0,68537411	MFN2	mitofusin 2 [Source:HGNC Symbol;Acc:HGNC:16877]
ENSG00000143924	0,16004269	8,56410391	0,75173262	0,40785544	0,68549933	EML4	EMAP like 4 [Source:HGNC Symbol;Acc:HGNC:1316]
ENSG00000235064	-0,3826597	-1,187004	0,75143411	0,40794504	0,68557105	SLC25A5P2	solute carrier family 25 member 5 pseudogene 2 [Source:HGNC Symbol;Acc:HGNC:35470]
ENSG00000103126	-0,1294492	5,62220965	0,75100594	0,40807361	0,68562937	AXIN1	axin 1 [Source:HGNC Symbol;Acc:HGNC:903]
ENSG00000164022	0,12264589	6,64250914	0,75103899	0,40806368	0,68562937	AIMP1	aminoacyl tRNA synthetase complex interacting multifunctional protein 1 [Source:HGNC Symbol;Acc:HGNC:1064[illegible]
ENSG00000177989	-0,4766268	-1,1660835	0,75077697	0,40814239	0,68566607	ODF3B	outer dense fiber of sperm tails 3B [Source:HGNC Symbol;Acc:HGNC:34388]
ENSG00000100578	0,14750589	4,00497446	0,75033146	0,40827627	0,68581212	KIAA0586	KIAA0586 [Source:HGNC Symbol;Acc:HGNC:19960]
ENSG00000125450	0,12011988	5,82786177	0,74908352	0,40865166	0,68623896	NUP85	nucleoporin 85 [Source:HGNC Symbol;Acc:HGNC:8734]
ENSG00000162997	-0,2666956	0,3863063	0,74929468	0,4085881	0,68623896	PRORSD1P	prolyl-tRNA synthetase associated domain containing 1, pseudogene [Source:HGNC Symbol;Acc:HGNC:34379]
ENSG00000198356	0,11582672	6,87535077	0,74892678	0,40869885	0,68623896	GET3	guided entry of tail-anchored proteins factor 3, ATPase [Source:HGNC Symbol;Acc:HGNC:752]
ENSG00000206530	-0,6046993	-1,5193121	0,74886229	0,40871827	0,68623896	CFAP44	cilia and flagella associated protein 44 [Source:HGNC Symbol;Acc:HGNC:25631]
ENSG00000140263	-0,1145357	6,51807849	0,74810994	0,40894491	0,68654058	SORD	sorbitol dehydrogenase [Source:HGNC Symbol;Acc:HGNC:11184]
ENSG00000143553	-0,160763	4,44143983	0,74768583	0,40907276	0,68660269	SNAPIN	SNAP associated protein [Source:HGNC Symbol;Acc:HGNC:17145]
ENSG00000149474	0,17293612	4,0800919	0,74751955	0,4091229	0,68660269	KAT14	lysine acetyltransferase 14 [Source:HGNC Symbol;Acc:HGNC:15904]
ENSG00000166169	-0,1318551	4,3436446	0,74754724	0,40911455	0,68660269	POLL	DNA polymerase lambda [Source:HGNC Symbol;Acc:HGNC:9184]
ENSG00000269113	0,5194724	-1,7428469	0,74718345	0,40922429	0,68669396	TRABD2B	TraB domain containing 2B [Source:HGNC Symbol;Acc:HGNC:44200]
ENSG00000173451	-0,2010425	1,39432221	0,74614689	0,40953721	0,68708735	THAP2	THAP domain containing 2 [Source:HGNC Symbol;Acc:HGNC:20854]
ENSG00000198270	0,18110098	2,64393612	0,74609535	0,40955278	0,68708735	TMEM116	transmembrane protein 116 [Source:HGNC Symbol;Acc:HGNC:25084]
ENSG00000164631	0,13908466	3,61611108	0,74556226	0,40971388	0,68725525	ZNF12	zinc finger protein 12 [Source:HGNC Symbol;Acc:HGNC:12902]
ENSG00000267767	0,29473843	0,06567647	0,74545286	0,40974695	0,68725525	LINC01801	long intergenic non-protein coding RNA 1801 [Source:HGNC Symbol;Acc:HGNC:52592]
ENSG00000105568	0,15164309	7,79803718	0,74494998	0,40989903	0,68740021	PPP2R1A	protein phosphatase 2 scaffold subunit Aalpha [Source:HGNC Symbol;Acc:HGNC:9302]

ENSG00000155744	0,23645063	1,83587328	0,74485591	0,40992749	0,68740021	FAM126B	family with sequence similarity 126 member B [Source:HGNC Symbol;Acc:HGNC:28593]
ENSG00000124145	0,14719049	7,18020195	0,74415838	0,41013861	0,68756515	SDC4	syndecan 4 [Source:HGNC Symbol;Acc:HGNC:10661]
ENSG00000166199	-0,1453487	4,4739989	0,74432536	0,41008805	0,68756515	ALKBH3	alkB homolog 3, alpha-ketoglutarate dependent dioxygenase [Source:HGNC Symbol;Acc:HGNC:30141]
ENSG00000198912	0,12390409	4,75657861	0,74406446	0,41016704	0,68756515	C1orf174	chromosome 1 open reading frame 174 [Source:HGNC Symbol;Acc:HGNC:27915]
ENSG00000067167	0,13714595	6,56894963	0,7438358	0,4102363	0,68760235	TRAM1	translocation associated membrane protein 1 [Source:HGNC Symbol;Acc:HGNC:20568]
ENSG00000144048	0,13566937	5,5496197	0,74329936	0,41039884	0,68771698	DUSP11	dual specificity phosphatase 11 [Source:HGNC Symbol;Acc:HGNC:3066]
ENSG00000162434	0,16545698	5,94057546	0,74333139	0,41038913	0,68771698	JAK1	Janus kinase 1 [Source:HGNC Symbol;Acc:HGNC:6190]
ENSG00000166171	0,12898001	4,05656592	0,74310305	0,41045835	0,68773781	DPCD	deleted in primary ciliary dyskinesia homolog (mouse) [Source:HGNC Symbol;Acc:HGNC:24542]
ENSG00000153551	-0,1393888	6,11783265	0,74185055	0,41083835	0,68824395	CMTM7	CKLF like MARVEL transmembrane domain containing 7 [Source:HGNC Symbol;Acc:HGNC:19178]
ENSG00000181404	-0,1518205	2,08906693	0,74179688	0,41085464	0,68824395	WASHC1	WASH complex subunit 1 [Source:HGNC Symbol;Acc:HGNC:24361]
ENSG00000139112	0,18437618	2,36913247	0,74152856	0,41093613	0,68825444	GABARAPL1	GABA type A receptor associated protein like 1 [Source:HGNC Symbol;Acc:HGNC:4068]
ENSG00000180479	0,27681047	-0,1175733	0,74146601	0,41095513	0,68825444	ZNF571	zinc finger protein 571 [Source:HGNC Symbol;Acc:HGNC:25000]
ENSG00000215464	-0,3941064	-1,1062436	0,74122101	0,41102956	0,68830019	AP000354.1	40S ribosomal protein S10 (RPS10) pseudogene
ENSG00000013503	0,16747063	4,42362181	0,73980499	0,41146017	0,68860628	POLR3B	RNA polymerase III subunit B [Source:HGNC Symbol;Acc:HGNC:30348]
ENSG00000112855	0,16805525	5,48419915	0,74036298	0,4112904	0,68860628	HARS2	histidyl-tRNA synthetase 2, mitochondrial [Source:HGNC Symbol;Acc:HGNC:4817]
ENSG00000127831	-0,2255627	8,50372329	0,74025266	0,41132396	0,68860628	VIL1	villin 1 [Source:HGNC Symbol;Acc:HGNC:12690]
ENSG00000148737	0,2072594	3,01059073	0,73990771	0,41142891	0,68860628	TCF7L2	transcription factor 7 like 2 [Source:HGNC Symbol;Acc:HGNC:11641]
ENSG00000165660	0,13739826	4,38688274	0,73984422	0,41144823	0,68860628	ABRAXAS2	abraxas 2, BRISC complex subunit [Source:HGNC Symbol;Acc:HGNC:28975]
ENSG00000260669	-0,5148711	-1,7184265	0,73969004	0,41149516	0,68860628	AL096870.2	novel transcript
ENSG00000143319	-0,1320326	4,87210617	0,73909884	0,41167518	0,68872658	ISG20L2	interferon stimulated exonuclease gene 20 like 2 [Source:HGNC Symbol;Acc:HGNC:25745]
ENSG00000152409	0,20968545	1,79579716	0,73898953	0,41170848	0,68872658	JMY	junction mediating and regulatory protein, p53 cofactor [Source:HGNC Symbol;Acc:HGNC:28916]
ENSG00000162869	-0,1307596	3,93996233	0,73929841	0,4116144	0,68872658	PPP1R21	protein phosphatase 1 regulatory subunit 21 [Source:HGNC Symbol;Acc:HGNC:30595]
ENSG00000203865	-0,3280586	-0,2907281	0,73871083	0,4117934	0,68878977	ATP1A1-AS1	ATP1A1 antisense RNA 1 [Source:HGNC Symbol;Acc:HGNC:28262]
ENSG00000112893	0,16594657	5,21752621	0,73817528	0,41195667	0,68898397	MAN2A1	mannosidase alpha class 2A member 1 [Source:HGNC Symbol;Acc:HGNC:6824]
ENSG00000167967	-0,1345091	4,08311925	0,73798441	0,41201488	0,68900245	E4F1	E4F transcription factor 1 [Source:HGNC Symbol;Acc:HGNC:3121]
ENSG00000257446	0,24556566	0,43334747	0,73782842	0,41206246	0,68900315	ZNF878	zinc finger protein 878 [Source:HGNC Symbol;Acc:HGNC:37246]
ENSG00000258605	0,41480224	-1,6721777	0,73722068	0,41224794	0,68923439	DYNLL1P2	dynein light chain LC8-type 1 pseudogene 2 [Source:HGNC Symbol;Acc:HGNC:20724]
ENSG00000249790	-0,5802662	-1,7106568	0,73673735	0,41239554	0,68932339	AC092490.1	novel transcript
ENSG00000278291	-0,1969798	1,62646318	0,7367702	0,4123855	0,68932339	AL161772.1	novel transcript, antisense to IL17D
ENSG00000141499	-0,1206047	4,87474429	0,73629436	0,41253089	0,689355	WRAP53	WD repeat containing antisense to TP53 [Source:HGNC Symbol;Acc:HGNC:25522]
ENSG00000153208	0,14346253	5,86379385	0,7363217	0,41252254	0,689355	MERTK	MER proto-oncogene, tyrosine kinase [Source:HGNC Symbol;Acc:HGNC:7027]
ENSG00000287168	-0,2599849	0,20637878	0,73621219	0,41255601	0,689355	AL512625.3	novel transcript
ENSG00000102870	-0,1475972	5,54532592	0,73573081	0,41270319	0,68944322	ZNF629	zinc finger protein 629 [Source:HGNC Symbol;Acc:HGNC:29008]
ENSG00000141642	-0,1822012	1,40420784	0,73581985	0,41267596	0,68944322	ELAC1	elaC ribonuclease Z 1 [Source:HGNC Symbol;Acc:HGNC:14197]
ENSG00000196526	-0,2180109	2,24693596	0,73515839	0,41287832	0,68965691	AFAP1	actin filament associated protein 1 [Source:HGNC Symbol;Acc:HGNC:24017]
ENSG00000157191	0,12247057	6,06892188	0,7334504	0,41340158	0,69033314	NECAP2	NECAP endocytosis associated 2 [Source:HGNC Symbol;Acc:HGNC:25528]
ENSG00000174021	-0,149495	5,04349957	0,73333768	0,41343615	0,69033314	GNG5	G protein subunit gamma 5 [Source:HGNC Symbol;Acc:HGNC:4408]
ENSG00000196656	-0,2131313	1,03389156	0,73315479	0,41349225	0,69033314	AC004057.1	ribosomal protein S26 (RPS26) pseudogene
ENSG00000198373	0,14255257	5,25162589	0,7330662	0,41351943	0,69033314	WWP2	WW domain containing E3 ubiquitin protein ligase 2 [Source:HGNC Symbol;Acc:HGNC:16804]
ENSG00000271646	0,28261609	-0,3381015	0,73357533	0,41336327	0,69033314	IRF2-DT	IRF2 divergent transcript [Source:HGNC Symbol;Acc:HGNC:54871]
ENSG00000205364	0,45738858	1,20915067	0,73282054	0,41359481	0,69038009	MT1M	metallothionein 1M [Source:HGNC Symbol;Acc:HGNC:14296]
ENSG00000116514	0,12146313	4,89789127	0,73177512	0,41391585	0,69052149	RNF19B	ring finger protein 19B [Source:HGNC Symbol;Acc:HGNC:26886]
ENSG00000163378	-0,1439551	3,33614023	0,73204381	0,4138333	0,69052149	EOGT	EGF domain specific O-linked N-acetylglucosamine transferase [Source:HGNC Symbol;Acc:HGNC:28526]
ENSG00000184489	0,21904582	1,27246138	0,73230374	0,41375346	0,69052149	PTP4A3	protein tyrosine phosphatase 4A3 [Source:HGNC Symbol;Acc:HGNC:9636]

ENSG00000237638	-0,1524682	3,03218906	0,73204404	0,41383323	0,69052149	LINC02245	long intergenic non-protein coding RNA 2245 [Source:HGNC Symbol;Acc:HGNC:53134]
ENSG00000248593	-0,2193009	1,11967728	0,73183098	0,41389869	0,69052149	DSTNP2	DSTN pseudogene 2 [Source:HGNC Symbol;Acc:HGNC:34546]
ENSG00000130921	0,13563458	3,79979953	0,73161875	0,41396391	0,6905228	C12orf65	chromosome 12 open reading frame 65 [Source:HGNC Symbol;Acc:HGNC:26784]
ENSG00000141577	-0,1972765	4,44325376	0,73085154	0,41419981	0,69065633	CEP131	centrosomal protein 131 [Source:HGNC Symbol;Acc:HGNC:29511]
ENSG00000234664	0,24147801	1,47264193	0,73074347	0,41423306	0,69065633	HMGN2P5	high mobility group nucleosomal binding domain 2 pseudogene 5 [Source:HGNC Symbol;Acc:HGNC:33568]
ENSG00000236670	0,47442904	-1,4959713	0,73084893	0,41420061	0,69065633	KRT18P5	keratin 18 pseudogene 5 [Source:HGNC Symbol;Acc:HGNC:19259]
ENSG00000259051	-0,331666	-0,5493228	0,73110332	0,41412237	0,69065633	HNRNPUP1	heterogeneous nuclear ribonucleoprotein U pseudogene 1 [Source:HGNC Symbol;Acc:HGNC:19931]
ENSG00000100243	0,12945811	8,60164025	0,73039115	0,41434148	0,69067945	CYB5R3	cytochrome b5 reductase 3 [Source:HGNC Symbol;Acc:HGNC:2873]
ENSG00000130309	0,13891938	8,04434517	0,73047339	0,41431617	0,69067945	COLGALT1	collagen beta(1-O)galactosyltransferase 1 [Source:HGNC Symbol;Acc:HGNC:26182]
ENSG00000130768	-0,4049781	0,57987093	0,73018208	0,41440584	0,69070792	SMPDL3B	sphingomyelin phosphodiesterase acid like 3B [Source:HGNC Symbol;Acc:HGNC:21416]
ENSG00000133135	0,18279214	5,58351427	0,72972516	0,41454655	0,69086363	RNF128	ring finger protein 128 [Source:HGNC Symbol;Acc:HGNC:21153]
ENSG00000111450	-0,1330498	3,38044744	0,72951641	0,41461086	0,69089199	STX2	syntaxin 2 [Source:HGNC Symbol;Acc:HGNC:3403]
ENSG00000233830	-0,2964579	-0,5511006	0,72820887	0,41501406	0,69148499	EIF4HP1	eukaryotic translation initiation factor 4H pseudogene 1 [Source:HGNC Symbol;Acc:HGNC:49035]
ENSG00000179743	-0,1973194	1,2396048	0,72801865	0,41507276	0,69150394	FLJ37453	uncharacterized LOC729614 [Source:NCBI gene (formerly Entrezgene);Acc:729614]
ENSG00000172081	0,16042738	2,8227664	0,72762248	0,41519508	0,69154999	MOB3A	MOB kinase activator 3A [Source:HGNC Symbol;Acc:HGNC:29802]
ENSG00000226380	-0,7250056	-1,3901223	0,7277088	0,41516843	0,69154999	AC016831.1	novel transcript
ENSG00000079432	0,2035437	4,62659497	0,72688737	0,4154222	0,69169169	CIC	capicua transcriptional repressor [Source:HGNC Symbol;Acc:HGNC:14214]
ENSG00000108669	0,12211308	5,23325879	0,72696979	0,41539672	0,69169169	CYTH1	cytohesin 1 [Source:HGNC Symbol;Acc:HGNC:9501]
ENSG00000215417	0,65963433	-1,5071573	0,72704075	0,4153748	0,69169169	MIR17HG	miR-17-92a-1 cluster host gene [Source:HGNC Symbol;Acc:HGNC:23564]
ENSG00000068308	-0,1257608	5,27301235	0,72655573	0,41552473	0,69178356	OTUD5	OTU deubiquitinase 5 [Source:HGNC Symbol;Acc:HGNC:25402]
ENSG00000128849	0,41570249	4,0383028	0,72614806	0,41565082	0,69191463	CGNL1	cingulin like 1 [Source:HGNC Symbol;Acc:HGNC:25931]
ENSG00000213047	-0,3054649	0,4199203	0,72591258	0,41572368	0,69195707	DENND1B	DENN domain containing 1B [Source:HGNC Symbol;Acc:HGNC:28404]
ENSG00000173801	0,20197613	5,44143827	0,72565423	0,41580364	0,69197038	JUP	junction plakoglobin [Source:HGNC Symbol;Acc:HGNC:6207]
ENSG00000174428	-0,3610128	-1,0962363	0,72558068	0,41582641	0,69197038	GTF2IRD2B	GTF2I repeat domain containing 2B [Source:HGNC Symbol;Acc:HGNC:33125]
ENSG00000088298	0,11828722	7,58134775	0,72537302	0,4158907	0,69199854	EDEM2	ER degradation enhancing alpha-mannosidase like protein 2 [Source:HGNC Symbol;Acc:HGNC:15877]
ENSG00000105664	0,38208194	-1,3117514	0,7247592	0,41608084	0,69223608	COMP	cartilage oligomeric matrix protein [Source:HGNC Symbol;Acc:HGNC:2227]
ENSG00000105202	-0,1128942	8,32576805	0,7242532	0,41623769	0,69241818	FBL	fibrillarin [Source:HGNC Symbol;Acc:HGNC:3599]
ENSG00000173207	-0,1523841	6,2524819	0,72363247	0,41643024	0,69245491	CKS1B	CDC28 protein kinase regulatory subunit 1B [Source:HGNC Symbol;Acc:HGNC:19083]
ENSG00000214297	-0,3889637	-0,8558273	0,7238338	0,41636777	0,69245491	ALDOAP2	ALDOA pseudogene 2 [Source:HGNC Symbol;Acc:HGNC:416]
ENSG00000228192	-0,2929401	0,86943823	0,72357081	0,41644937	0,69245491	AL512353.1	novel transcript, antisense to ERMAP
ENSG00000228887	-0,2259704	0,67775753	0,72369502	0,41641083	0,69245491	EEF1DP1	eukaryotic translation elongation factor 1 delta pseudogene 1 [Source:HGNC Symbol;Acc:HGNC:32474]
ENSG00000089009	-0,1383774	10,5235493	0,72266108	0,41673184	0,69256552	RPL6	ribosomal protein L6 [Source:HGNC Symbol;Acc:HGNC:10362]
ENSG00000092871	-0,1338093	3,63847999	0,72209336	0,41690827	0,69256552	RFFL	ring finger and FYVE like domain containing E3 ubiquitin protein ligase [Source:HGNC Symbol;Acc:HGNC:24821]
ENSG00000102606	0,13677262	4,46834569	0,72306675	0,41660584	0,69256552	ARHGEF7	Rho guanine nucleotide exchange factor 7 [Source:HGNC Symbol;Acc:HGNC:15607]
ENSG00000112242	0,14552504	3,92675345	0,72188786	0,41697217	0,69256552	E2F3	E2F transcription factor 3 [Source:HGNC Symbol;Acc:HGNC:3115]
ENSG00000153015	0,12022434	5,23159822	0,72259699	0,41675175	0,69256552	CWC27	CWC27 spliceosome associated cyclophilin [Source:HGNC Symbol;Acc:HGNC:10664]
ENSG00000161031	0,37291083	-0,7502291	0,72242709	0,41680455	0,69256552	PGLYRP2	peptidoglycan recognition protein 2 [Source:HGNC Symbol;Acc:HGNC:30013]
ENSG00000166689	0,15517187	4,03326876	0,72248286	0,41678721	0,69256552	PLEKHA7	pleckstrin homology domain containing A7 [Source:HGNC Symbol;Acc:HGNC:27049]
ENSG00000229740	-0,5428838	-1,6061163	0,72259332	0,41675289	0,69256552	U91324.1	novel transcript
ENSG00000232203	-0,3373193	-0,6247195	0,72183065	0,41698996	0,69256552	SLC25A6P2	solute carrier family 25 member 6 pseudogene 2 [Source:HGNC Symbol;Acc:HGNC:43850]
ENSG00000286733	-0,2555256	0,63613012	0,7219868	0,4169414	0,69256552	LINC02348	long intergenic non-protein coding RNA 2348 [Source:NCBI gene (formerly Entrezgene);Acc:101927505]
ENSG00000141664	0,13841383	4,66344869	0,72155507	0,41707567	0,69262914	ZCCHC2	zinc finger CCHC-type containing 2 [Source:HGNC Symbol;Acc:HGNC:22916]
ENSG00000248923	0,34055952	0,0439505	0,72102026	0,4172421	0,69282676	MTND5P11	MT-ND5 pseudogene 11 [Source:HGNC Symbol;Acc:HGNC:42273]
ENSG00000187954	-0,1467095	4,38326819	0,72059084	0,41737581	0,69289127	CYHR1	cysteine and histidine rich 1 [Source:HGNC Symbol;Acc:HGNC:17806]

ENSG00000197417	0,19523024	2,84032439	0,72061748	0,41736751	0,69289127	SHPK	sedoheptulokinase [Source:HGNC Symbol;Acc:HGNC:1492]
ENSG00000180089	-0,1773836	3,1749816	0,7204329	0,417425	0,6928942	TMEM86B	transmembrane protein 86B [Source:HGNC Symbol;Acc:HGNC:28448]
ENSG00000119787	0,11827866	6,12977231	0,72027209	0,4174751	0,69289863	ATL2	atlastin GTPase 2 [Source:HGNC Symbol;Acc:HGNC:24047]
ENSG00000111142	0,13857114	6,46953609	0,71946132	0,41772783	0,69309364	METAP2	methionyl aminopeptidase 2 [Source:HGNC Symbol;Acc:HGNC:16672]
ENSG00000124466	0,53504181	-1,4988005	0,71943858	0,41773492	0,69309364	LYPD3	LY6/PLAUR domain containing 3 [Source:HGNC Symbol;Acc:HGNC:24880]
ENSG00000186446	-0,1879737	1,08293019	0,71950673	0,41771367	0,69309364	ZNF501	zinc finger protein 501 [Source:HGNC Symbol;Acc:HGNC:23717]
ENSG00000130303	-0,1523744	7,52232291	0,71915624	0,41782299	0,69316104	BST2	bone marrow stromal cell antigen 2 [Source:HGNC Symbol;Acc:HGNC:1119]
ENSG00000197594	0,17354243	7,00727698	0,71890448	0,41790155	0,69321264	ENPP1	ectonucleotide pyrophosphatase/phosphodiesterase 1 [Source:HGNC Symbol;Acc:HGNC:3356]
ENSG00000198252	0,16893382	2,53630737	0,71818109	0,41812741	0,69350855	STYX	serine/threonine/tyrosine interacting protein [Source:HGNC Symbol;Acc:HGNC:11447]
ENSG00000272667	-0,2787418	-0,078651	0,71801043	0,41818072	0,69351824	AC012306.2	novel transcript
ENSG00000071553	0,1840271	7,98310741	0,71765424	0,41829202	0,69355864	ATP6AP1	ATPase H+ transporting accessory protein 1 [Source:HGNC Symbol;Acc:HGNC:868]
ENSG00000132329	-0,6234687	-1,1269701	0,71762862	0,41830003	0,69355864	RAMP1	receptor activity modifying protein 1 [Source:HGNC Symbol;Acc:HGNC:9843]
ENSG00000198182	0,15460535	2,48333973	0,71686481	0,41853888	0,69379718	ZNF607	zinc finger protein 607 [Source:HGNC Symbol;Acc:HGNC:28192]
ENSG00000267751	-0,1707991	2,12988939	0,71688695	0,41853195	0,69379718	AC009005.1	novel transcript, antisense to BSG
ENSG00000176715	0,12665611	4,98482982	0,71649971	0,41865313	0,69390782	ACSF3	acyl-CoA synthetase family member 3 [Source:HGNC Symbol;Acc:HGNC:27288]
ENSG00000228506	-0,3065561	0,44911975	0,71631406	0,41871124	0,69392542	AL513550.1	novel transcript
ENSG00000110801	0,13417224	3,16022083	0,7157899	0,41887539	0,69411871	PSMD9	proteasome 26S subunit, non-ATPase 9 [Source:HGNC Symbol;Acc:HGNC:9567]
ENSG00000144036	0,2414644	3,54042472	0,71514634	0,41907707	0,69437416	EXOC6B	exocyst complex component 6B [Source:HGNC Symbol;Acc:HGNC:17085]
ENSG00000105700	-0,1311806	5,39925834	0,7146921	0,41921952	0,69448847	KXD1	KxDL motif containing 1 [Source:HGNC Symbol;Acc:HGNC:28420]
ENSG00000216895	-0,2413956	0,57292549	0,71462317	0,41924114	0,69448847	AC009403.1	novel transcript, antisense to RBM33
ENSG00000241651	-0,3350387	-0,4682661	0,71374019	0,41951829	0,69486878	AC099340.1	ribosomal protein L15 (RPL15) pseudogene
ENSG00000126067	-0,1353027	8,15667757	0,71342848	0,4196162	0,69487338	PSMB2	proteasome 20S subunit beta 2 [Source:HGNC Symbol;Acc:HGNC:9539]
ENSG00000167881	0,13839333	7,6766326	0,71355704	0,41957581	0,69487338	SRP68	signal recognition particle 68 [Source:HGNC Symbol;Acc:HGNC:11302]
ENSG00000140995	-0,1292987	6,14525623	0,71292012	0,41977595	0,6950169	DEF8	differentially expressed in FDCP 8 homolog [Source:HGNC Symbol;Acc:HGNC:25969]
ENSG00000152270	0,12681993	4,59862643	0,71254735	0,41989316	0,6950169	PDE3B	phosphodiesterase 3B [Source:HGNC Symbol;Acc:HGNC:8779]
ENSG00000168495	-0,1174296	5,6687134	0,71273468	0,41983425	0,6950169	POLR3D	RNA polymerase III subunit D [Source:HGNC Symbol;Acc:HGNC:1080]
ENSG00000184635	0,6294251	-1,8627231	0,71260882	0,41987383	0,6950169	ZNF93	zinc finger protein 93 [Source:HGNC Symbol;Acc:HGNC:13169]
ENSG00000123416	-0,1340509	10,408934	0,71215789	0,42001567	0,69514093	TUBA1B	tubulin alpha 1b [Source:HGNC Symbol;Acc:HGNC:18809]
ENSG00000113552	0,12834168	6,64991603	0,71136481	0,42026533	0,69547533	GNPDA1	glucosamine-6-phosphate deaminase 1 [Source:HGNC Symbol;Acc:HGNC:4417]
ENSG00000100997	0,11794969	7,50660138	0,70963283	0,4208114	0,69592011	ABHD12	abhydrolase domain containing 12, lysophospholipase [Source:HGNC Symbol;Acc:HGNC:15868]
ENSG00000125144	-0,1918939	6,39532391	0,70885153	0,4210581	0,69592011	MT1G	metallothionein 1G [Source:HGNC Symbol;Acc:HGNC:7399]
ENSG00000128989	-0,1293941	4,7878959	0,71007379	0,42067226	0,69592011	ARPP19	cAMP regulated phosphoprotein 19 [Source:HGNC Symbol;Acc:HGNC:16967]
ENSG00000134569	0,16153859	3,56386901	0,70894169	0,42102962	0,69592011	LRP4	LDL receptor related protein 4 [Source:HGNC Symbol;Acc:HGNC:6696]
ENSG00000140105	-0,1474158	6,80532709	0,71005286	0,42067886	0,69592011	WARS1	tryptophanyl-tRNA synthetase 1 [Source:HGNC Symbol;Acc:HGNC:12729]
ENSG00000143970	-0,2327232	3,89335704	0,71035821	0,42058256	0,69592011	ASXL2	ASXL transcriptional regulator 2 [Source:HGNC Symbol;Acc:HGNC:23805]
ENSG00000178607	-0,2037129	3,69422379	0,70908669	0,42098382	0,69592011	ERN1	endoplasmic reticulum to nucleus signaling 1 [Source:HGNC Symbol;Acc:HGNC:3449]
ENSG00000198517	-0,1457802	3,59326337	0,70946545	0,42086423	0,69592011	MAFK	MAF bZIP transcription factor K [Source:HGNC Symbol;Acc:HGNC:6782]
ENSG00000223803	-0,2231379	0,68682213	0,70970769	0,42078777	0,69592011	RPS20P14	ribosomal protein S20 pseudogene 14 [Source:HGNC Symbol;Acc:HGNC:36692]
ENSG00000226721	-0,5094676	-1,6258546	0,70912623	0,42097134	0,69592011	EEF1DP2	eukaryotic translation elongation factor 1 delta pseudogene 2 [Source:HGNC Symbol;Acc:HGNC:33523]
ENSG00000240395	-0,2430142	0,43989949	0,70925289	0,42093134	0,69592011	RPL5P23	ribosomal protein L5 pseudogene 23 [Source:HGNC Symbol;Acc:HGNC:36553]
ENSG00000130449	-0,3528298	-0,2078274	0,70841495	0,42119606	0,6959501	ZSWIM6	zinc finger SWIM-type containing 6 [Source:HGNC Symbol;Acc:HGNC:29316]
ENSG00000164008	-0,3204164	0,11570152	0,7085231	0,42116188	0,6959501	C1orf50	chromosome 1 open reading frame 50 [Source:HGNC Symbol;Acc:HGNC:28795]
ENSG00000242616	-0,3888362	-0,7851837	0,70834187	0,42121916	0,6959501	GNG10	G protein subunit gamma 10 [Source:HGNC Symbol;Acc:HGNC:4402]
ENSG00000152242	0,13758664	3,16716746	0,70802902	0,42131808	0,69603481	C18orf25	chromosome 18 open reading frame 25 [Source:HGNC Symbol;Acc:HGNC:28172]

ENSG00000157600	0,20892092	2,73655702	0,707602	0,42145315	0,69610052	TMEM164	transmembrane protein 164 [Source:HGNC Symbol;Acc:HGNC:26217]
ENSG00000213178	-0,1681287	2,00821954	0,70765896	0,42143513	0,69610052	RPL22P1	ribosomal protein L22 pseudogene 1 [Source:HGNC Symbol;Acc:HGNC:17998]
ENSG00000188878	-0,2746346	0,46512735	0,70721134	0,42157679	0,69622601	FBF1	Fas binding factor 1 [Source:HGNC Symbol;Acc:HGNC:24674]
ENSG00000248333	-0,1867417	4,87984432	0,70681524	0,4217022	0,69627571	CDK11B	cyclin dependent kinase 11B [Source:HGNC Symbol;Acc:HGNC:1729]
ENSG00000260924	-0,2045559	0,87928536	0,70687526	0,42168319	0,69627571	LINC01311	long intergenic non-protein coding RNA 1311 [Source:HGNC Symbol;Acc:HGNC:50503]
ENSG00000108010	-0,1101671	7,49936434	0,7065939	0,42177231	0,69631277	GLRX3	glutaredoxin 3 [Source:HGNC Symbol;Acc:HGNC:15987]
ENSG00000074582	0,12645672	5,8652989	0,70630384	0,42186421	0,69632089	BCS1L	BCS1 homolog, ubiquinol-cytochrome c reductase complex chaperone [Source:HGNC Symbol;Acc:HGNC:1020]
ENSG00000162377	-0,1195832	5,57891695	0,7062775	0,42187256	0,69632089	COA7	cytochrome c oxidase assembly factor 7 (putative) [Source:HGNC Symbol;Acc:HGNC:25716]
ENSG00000074201	0,12010685	7,22146821	0,70596234	0,42197246	0,69634512	CLNS1A	chloride nucleotide-sensitive channel 1A [Source:HGNC Symbol;Acc:HGNC:2080]
ENSG00000276728	-0,3165673	-0,2624827	0,70593045	0,42198257	0,69634512	AC142472.1	novel transcript
ENSG00000066084	0,20859655	5,51820152	0,70557834	0,42209423	0,69637206	DIP2B	disco interacting protein 2 homolog B [Source:HGNC Symbol;Acc:HGNC:29284]
ENSG00000244582	-0,2906621	0,03948831	0,70572141	0,42204885	0,69637206	RPL21P120	ribosomal protein L21 pseudogene 120 [Source:HGNC Symbol;Acc:HGNC:35743]
ENSG00000064102	-0,1280136	5,15657688	0,70392053	0,42262059	0,69716172	INTS13	integrator complex subunit 13 [Source:HGNC Symbol;Acc:HGNC:20174]
ENSG00000234797	-0,1270058	3,99456833	0,70372434	0,42268295	0,69718587	RPS3AP6	RPS3A pseudogene 6 [Source:HGNC Symbol;Acc:HGNC:18630]
ENSG00000141873	0,12717417	5,6040363	0,70341473	0,4227814	0,69726952	SLC39A3	solute carrier family 39 member 3 [Source:HGNC Symbol;Acc:HGNC:17128]
ENSG00000088325	0,13647595	8,42087014	0,70295831	0,42292659	0,69735153	TPX2	TPX2 microtubule nucleation factor [Source:HGNC Symbol;Acc:HGNC:1249]
ENSG00000101752	0,1963199	3,48907875	0,70304	0,42290059	0,69735153	MIB1	mindbomb E3 ubiquitin protein ligase 1 [Source:HGNC Symbol;Acc:HGNC:21086]
ENSG00000089094	0,12889923	5,03315721	0,7024929	0,42307473	0,69743835	KDM2B	lysine demethylase 2B [Source:HGNC Symbol;Acc:HGNC:13610]
ENSG00000176978	-0,5288657	-1,140255	0,70258845	0,42304431	0,69743835	DPP7	dipeptidyl peptidase 7 [Source:HGNC Symbol;Acc:HGNC:14892]
ENSG00000145779	0,15467228	2,69321331	0,70223816	0,42315584	0,69749337	TNFAIP8	TNF alpha induced protein 8 [Source:HGNC Symbol;Acc:HGNC:17260]
ENSG00000104763	-0,1286822	7,22344176	0,70052085	0,42370336	0,69831705	ASAH1	N-acylsphingosine amidohydrolase 1 [Source:HGNC Symbol;Acc:HGNC:735]
ENSG00000155324	0,21463795	2,41456068	0,7003315	0,42376379	0,69833788	GRAMD2B	GRAM domain containing 2B [Source:HGNC Symbol;Acc:HGNC:24911]
ENSG00000159792	-0,1470111	3,65589409	0,69974646	0,42395062	0,69856696	PSKH1	protein serine kinase H1 [Source:HGNC Symbol;Acc:HGNC:9529]
ENSG00000133706	-0,1620128	6,20028837	0,69927016	0,42410283	0,69873894	LARS1	leucyl-tRNA synthetase 1 [Source:HGNC Symbol;Acc:HGNC:6512]
ENSG00000008083	0,17949886	3,18346961	0,69885086	0,42423689	0,6988022	JARID2	jumonji and AT-rich interaction domain containing 2 [Source:HGNC Symbol;Acc:HGNC:6196]
ENSG00000187243	0,33730716	-0,6322649	0,69891719	0,42421568	0,6988022	MAGED4	MAGE family member D4 [Source:HGNC Symbol;Acc:HGNC:23793]
ENSG00000102054	0,11721665	6,8131867	0,6982703	0,42442262	0,69882706	RBBP7	RB binding protein 7, chromatin remodeling factor [Source:HGNC Symbol;Acc:HGNC:9890]
ENSG00000104356	-0,1834791	4,05123438	0,69812597	0,42446882	0,69882706	POP1	POP1 homolog, ribonuclease P/MRP subunit [Source:HGNC Symbol;Acc:HGNC:30129]
ENSG00000187735	-0,1155022	6,12199957	0,69805618	0,42449116	0,69882706	TCEA1	transcription elongation factor A1 [Source:HGNC Symbol;Acc:HGNC:11612]
ENSG00000196227	-0,1810178	2,05675707	0,69812813	0,42446813	0,69882706	FAM217B	family with sequence similarity 217 member B [Source:HGNC Symbol;Acc:HGNC:16170]
ENSG00000272711	-0,214006	1,56295748	0,69809737	0,42447797	0,69882706	AC019069.1	novel transcript
ENSG00000100441	-0,1350703	5,75201166	0,6972588	0,42474654	0,69908994	KHNYN	KH and NYN domain containing [Source:HGNC Symbol;Acc:HGNC:20166]
ENSG00000237264	-0,1433599	2,27879587	0,69732587	0,42472505	0,69908994	FTH1P11	ferritin heavy chain 1 pseudogene 11 [Source:HGNC Symbol;Acc:HGNC:3981]
ENSG00000089177	-0,1921184	3,96256438	0,69670032	0,42492557	0,69922703	KIF16B	kinesin family member 16B [Source:HGNC Symbol;Acc:HGNC:15869]
ENSG00000105619	-0,1666383	3,98665716	0,6967334	0,42491496	0,69922703	TFPT	TCF3 fusion partner [Source:HGNC Symbol;Acc:HGNC:13630]
ENSG00000135250	0,19186673	4,21924774	0,6964911	0,42499266	0,69925868	SRPK2	SRSF protein kinase 2 [Source:HGNC Symbol;Acc:HGNC:11306]
ENSG00000126460	0,23390198	0,32308911	0,69622922	0,42507667	0,69931814	PRRG2	proline rich and Gla domain 2 [Source:HGNC Symbol;Acc:HGNC:9470]
ENSG00000160404	-0,1269917	4,84666785	0,6956803	0,42525285	0,69952921	TOR2A	torsin family 2 member A [Source:HGNC Symbol;Acc:HGNC:11996]
ENSG00000178996	0,13216988	2,80491879	0,69512198	0,42543217	0,69970275	SNX18	sorting nexin 18 [Source:HGNC Symbol;Acc:HGNC:19245]
ENSG00000214460	-0,4617851	-2,0158626	0,69505359	0,42545414	0,69970275	TPT1P6	tumor protein, translationally-controlled 1 pseudogene 6 [Source:HGNC Symbol;Acc:HGNC:49297]
ENSG00000064999	0,23736267	4,29666098	0,6936625	0,4259015	0,69988695	ANKS1A	ankyrin repeat and sterile alpha motif domain containing 1A [Source:HGNC Symbol;Acc:HGNC:20961]
ENSG00000075856	0,18293211	5,90876318	0,69442897	0,42565492	0,69988695	SART3	spliceosome associated factor 3, U4/U6 recycling protein [Source:HGNC Symbol;Acc:HGNC:16860]
ENSG00000170088	-0,1233038	3,41585897	0,69406999	0,42577038	0,69988695	TMEM192	transmembrane protein 192 [Source:HGNC Symbol;Acc:HGNC:26775]
ENSG00000177732	0,12535637	5,950184	0,69381402	0,42585274	0,69988695	SOX12	SRY-box transcription factor 12 [Source:HGNC Symbol;Acc:HGNC:11198]

ENSG00000180773	0,16223133	2,56804664	0,69372008	0,42588297	0,69988695	SLC36A4	solute carrier family 36 member 4 [Source:HGNC Symbol;Acc:HGNC:19660]
ENSG00000215326	-0,3994732	-1,6333307	0,69407119	0,42576999	0,69988695	GPX1P2	glutathione peroxidase pseudogene 2 [Source:HGNC Symbol;Acc:HGNC:4561]
ENSG00000274372	-0,3099308	-0,6531966	0,69383263	0,42584675	0,69988695	LINC02804	long intergenic non-protein coding RNA 2804 [Source:HGNC Symbol;Acc:HGNC:54338]
ENSG00000021488	-0,136722	2,72554208	0,69349275	0,42595614	0,69989802	SLC7A9	solute carrier family 7 member 9 [Source:HGNC Symbol;Acc:HGNC:11067]
ENSG00000126215	-0,1638285	3,51325283	0,69328144	0,42602418	0,69991573	XRCC3	X-ray repair cross complementing 3 [Source:HGNC Symbol;Acc:HGNC:12830]
ENSG00000175582	0,14004223	5,67481588	0,6931617	0,42606274	0,69991573	RAB6A	RAB6A, member RAS oncogene family [Source:HGNC Symbol;Acc:HGNC:9786]
ENSG00000068028	-0,1444564	3,88105965	0,69266437	0,42622296	0,69994072	RASSF1	Ras association domain family member 1 [Source:HGNC Symbol;Acc:HGNC:9882]
ENSG00000141551	-0,1093144	6,82577472	0,69255885	0,42625697	0,69994072	CSNK1D	casein kinase 1 delta [Source:HGNC Symbol;Acc:HGNC:2452]
ENSG00000164332	0,16807256	3,93753377	0,69257231	0,42625263	0,69994072	UBLCP1	ubiquitin like domain containing CTD phosphatase 1 [Source:HGNC Symbol;Acc:HGNC:28110]
ENSG00000182180	-0,1306279	6,80400993	0,69237102	0,42631751	0,69994072	MRPS16	mitochondrial ribosomal protein S16 [Source:HGNC Symbol;Acc:HGNC:14048]
ENSG00000260326	0,41768998	-1,191177	0,69243756	0,42629607	0,69994072	PHBP21	prohibitin pseudogene 21 [Source:HGNC Symbol;Acc:HGNC:51554]
ENSG00000165275	-0,1470032	2,16333911	0,69219722	0,42637355	0,69995406	TRMT10B	tRNA methyltransferase 10B [Source:HGNC Symbol;Acc:HGNC:26454]
ENSG00000175575	-0,1259417	5,28980385	0,69092956	0,42678263	0,700533	PAAF1	proteasomal ATPase associated factor 1 [Source:HGNC Symbol;Acc:HGNC:25687]
ENSG00000258357	-0,3652977	-1,3683254	0,69080731	0,42682211	0,700533	NSA2P2	NSA2 pseudogene 2 [Source:HGNC Symbol;Acc:HGNC:54581]
ENSG00000141068	-0,1519348	3,0645206	0,69064441	0,42687473	0,70054066	KSR1	kinase suppressor of ras 1 [Source:HGNC Symbol;Acc:HGNC:6465]
ENSG00000151773	-0,475774	-1,0244447	0,69035286	0,42696894	0,70061656	CCDC122	coiled-coil domain containing 122 [Source:HGNC Symbol;Acc:HGNC:26478]
ENSG00000184371	-0,1853219	5,41595329	0,69003824	0,42707064	0,70070474	CSF1	colony stimulating factor 1 [Source:HGNC Symbol;Acc:HGNC:2432]
ENSG00000101421	0,11274616	7,10910851	0,68950228	0,42724398	0,70081117	CHMP4B	charged multivesicular body protein 4B [Source:HGNC Symbol;Acc:HGNC:16171]
ENSG00000107669	0,14932416	4,98930267	0,6892445	0,42732739	0,70081117	ATE1	arginyltransferase 1 [Source:HGNC Symbol;Acc:HGNC:782]
ENSG00000168066	-0,1348106	7,62353718	0,6895812	0,42721845	0,70081117	SF1	splicing factor 1 [Source:HGNC Symbol;Acc:HGNC:12950]
ENSG00000184939	0,16343887	2,28259669	0,6892963	0,42731063	0,70081117	ZFP90	ZFP90 zinc finger protein [Source:HGNC Symbol;Acc:HGNC:23329]
ENSG00000040341	0,13388799	3,64115767	0,6884827	0,42757405	0,7009691	STAU2	staufen double-stranded RNA binding protein 2 [Source:HGNC Symbol;Acc:HGNC:11371]
ENSG00000167994	0,14874286	2,39256562	0,68820631	0,4276636	0,7009691	RAB3IL1	RAB3A interacting protein like 1 [Source:HGNC Symbol;Acc:HGNC:9780]
ENSG00000171720	0,11409784	6,5264081	0,68850905	0,42756552	0,7009691	HDAC3	histone deacetylase 3 [Source:HGNC Symbol;Acc:HGNC:4854]
ENSG00000188833	0,60269496	-1,4225252	0,68821121	0,42766201	0,7009691	ENTPD8	ectonucleoside triphosphate diphosphohydrolase 8 [Source:HGNC Symbol;Acc:HGNC:24860]
ENSG00000272267	0,25925174	-0,0759935	0,68838466	0,42760581	0,7009691	AC021242.3	novel transcript, antisense to TNKS
ENSG00000243759	0,38098179	-1,3692408	0,68790066	0,42776266	0,70105281	ST13P15	ST13, Hsp70 interacting protein pseudogene 15 [Source:HGNC Symbol;Acc:HGNC:38850]
ENSG00000087085	0,35618628	-0,3487686	0,68732471	0,42794944	0,70119343	ACHE	acetylcholinesterase (Cartwright blood group) [Source:HGNC Symbol;Acc:HGNC:108]
ENSG00000125648	0,13853278	3,8210681	0,68719211	0,42799246	0,70119343	SLC25A23	solute carrier family 25 member 23 [Source:HGNC Symbol;Acc:HGNC:19375]
ENSG00000212802	-0,1454971	2,51459806	0,68722949	0,42798033	0,70119343	RPL15P3	ribosomal protein L15 pseudogene 3 [Source:HGNC Symbol;Acc:HGNC:21538]
ENSG00000197548	-0,149651	3,84622651	0,68621808	0,42830868	0,70163282	ATG7	autophagy related 7 [Source:HGNC Symbol;Acc:HGNC:16935]
ENSG00000144043	-0,1152908	5,60758466	0,6857804	0,4284509	0,70176517	TEX261	testis expressed 261 [Source:HGNC Symbol;Acc:HGNC:30712]
ENSG00000224738	0,33378951	-0,3560201	0,68567381	0,42848555	0,70176517	AC099850.1	novel transcript
ENSG00000168646	-0,1245959	5,00571489	0,68519126	0,42864245	0,70186478	AXIN2	axin 2 [Source:HGNC Symbol;Acc:HGNC:904]
ENSG00000198791	0,16439638	5,73365135	0,68529022	0,42861027	0,70186478	CNOT7	CCR4-NOT transcription complex subunit 7 [Source:HGNC Symbol;Acc:HGNC:14101]
ENSG00000054282	-0,151804	2,81846846	0,68475431	0,42878462	0,7019505	SDCCAG8	SHH signaling and ciliogenesis regulator SDCCAG8 [Source:HGNC Symbol;Acc:HGNC:10671]
ENSG00000101966	0,16139874	2,82207917	0,68443979	0,428887	0,7019505	XIAP	X-linked inhibitor of apoptosis [Source:HGNC Symbol;Acc:HGNC:592]
ENSG00000105669	-0,155737	7,46319092	0,68448132	0,42887348	0,7019505	COPE	COPI coat complex subunit epsilon [Source:HGNC Symbol;Acc:HGNC:2234]
ENSG00000111554	0,14301843	2,62725449	0,68444393	0,42888565	0,7019505	MDM1	Mdm1 nuclear protein [Source:HGNC Symbol;Acc:HGNC:29917]
ENSG00000139624	0,12435528	5,65972316	0,68395916	0,42904352	0,70212802	CERS5	ceramide synthase 5 [Source:HGNC Symbol;Acc:HGNC:23749]
ENSG00000233381	0,41052167	-1,3217842	0,68361679	0,42915508	0,70223192	AK4P3	adenylate kinase 4 pseudogene 3 [Source:HGNC Symbol;Acc:HGNC:21596]
ENSG00000100228	-0,2554531	0,0315047	0,68330754	0,42925588	0,7023182	RAB36	RAB36, member RAS oncogene family [Source:HGNC Symbol;Acc:HGNC:9775]
ENSG00000047932	0,12492235	4,17431316	0,68213872	0,42963723	0,70278472	GOPC	golgi associated PDZ and coiled-coil motif containing [Source:HGNC Symbol;Acc:HGNC:17643]
ENSG00000241313	-0,4697193	-1,719188	0,68226432	0,42959623	0,70278472	WWTR1-AS1	WWTR1 antisense RNA 1 [Source:HGNC Symbol;Acc:HGNC:41035]

ENSG00000004779	-0,1150645	8,1469575	0,67975851	0,43041554	0,70281616	NDUFAB1	NADH:ubiquinone oxidoreductase subunit AB1 [Source:HGNC Symbol;Acc:HGNC:7694]
ENSG00000100852	-0,1232078	4,49813703	0,68167656	0,42978817	0,70281616	ARHGAP5	Rho GTPase activating protein 5 [Source:HGNC Symbol;Acc:HGNC:675]
ENSG00000101493	0,16705394	3,57685249	0,679726	0,43042619	0,70281616	ZNF516	zinc finger protein 516 [Source:HGNC Symbol;Acc:HGNC:28990]
ENSG00000101955	-0,6141039	-2,117321	0,68005838	0,43031736	0,70281616	SRPX	sushi repeat containing protein X-linked [Source:HGNC Symbol;Acc:HGNC:11309]
ENSG00000116679	-0,1129726	5,38777075	0,68038594	0,43021015	0,70281616	IVNS1ABP	influenza virus NS1A binding protein [Source:HGNC Symbol;Acc:HGNC:16951]
ENSG00000119777	0,16676185	7,54713636	0,67983057	0,43039195	0,70281616	TMEM214	transmembrane protein 214 [Source:HGNC Symbol;Acc:HGNC:25983]
ENSG00000119912	0,14925539	5,25442641	0,6809193	0,43003568	0,70281616	IDE	insulin degrading enzyme [Source:HGNC Symbol;Acc:HGNC:5381]
ENSG00000132254	0,15454591	6,07964516	0,68010591	0,4303018	0,70281616	ARFIP2	ADP ribosylation factor interacting protein 2 [Source:HGNC Symbol;Acc:HGNC:17160]
ENSG00000138496	0,15672923	2,52963088	0,68063542	0,43012853	0,70281616	PARP9	poly(ADP-ribose) polymerase family member 9 [Source:HGNC Symbol;Acc:HGNC:24118]
ENSG00000139505	-0,1361651	3,15691516	0,68181974	0,4297414	0,70281616	MTMR6	myotubularin related protein 6 [Source:HGNC Symbol;Acc:HGNC:7453]
ENSG00000163701	-0,1298778	4,36281662	0,68102571	0,43000089	0,70281616	IL17RE	interleukin 17 receptor E [Source:HGNC Symbol;Acc:HGNC:18439]
ENSG00000177181	-0,1580563	3,09596882	0,68064067	0,43012681	0,70281616	RIMKLA	ribosomal modification protein rimK like family member A [Source:HGNC Symbol;Acc:HGNC:28725]
ENSG00000197037	0,14048471	3,92674429	0,67976708	0,43041274	0,70281616	ZSCAN25	zinc finger and SCAN domain containing 25 [Source:HGNC Symbol;Acc:HGNC:21961]
ENSG00000210154	-0,3137917	-0,2150241	0,68119426	0,42994579	0,70281616	MT-TD	mitochondrially encoded tRNA-Asp (GAU/C) [Source:HGNC Symbol;Acc:HGNC:7478]
ENSG00000219133	-0,2598741	-0,0843951	0,67989487	0,43037089	0,70281616	AL592114.1	ribosomal protein L21 (RPL21) pseudogene
ENSG00000227388	0,7924536	-1,5171471	0,6800244	0,43032848	0,70281616	AL133410.1	novel transcript
ENSG00000232888	-0,2326215	0,60525378	0,6794726	0,43050919	0,70287313	RPS11P5	ribosomal protein S11 pseudogene 5 [Source:HGNC Symbol;Acc:HGNC:36312]
ENSG00000125870	0,11894224	7,75335255	0,67891168	0,43069301	0,70308415	SNRPB2	small nuclear ribonucleoprotein polypeptide B2 [Source:HGNC Symbol;Acc:HGNC:11155]
ENSG00000225905	-0,5717356	-1,9269663	0,67878454	0,4307347	0,70308415	AL391244.1	novel transcript
ENSG00000130396	0,22627632	6,02103471	0,67828407	0,43089884	0,70319494	AFDN	afadin, adherens junction formation factor [Source:HGNC Symbol;Acc:HGNC:7137]
ENSG00000137547	-0,1153807	6,77089294	0,67829493	0,43089528	0,70319494	MRPL15	mitochondrial ribosomal protein L15 [Source:HGNC Symbol;Acc:HGNC:14054]
ENSG00000103248	-0,1418791	3,41632027	0,67809911	0,43095953	0,70321282	MTHFSD	methenyltetrahydrofolate synthetase domain containing [Source:HGNC Symbol;Acc:HGNC:25778]
ENSG00000104863	-0,1629744	1,70562411	0,67768989	0,43109385	0,70321282	LIN7B	lin-7 homolog B, crumbs cell polarity complex component [Source:HGNC Symbol;Acc:HGNC:17788]
ENSG00000147647	0,48145537	-1,9986637	0,67775921	0,43107109	0,70321282	DPYS	dihydropyrimidinase [Source:HGNC Symbol;Acc:HGNC:3013]
ENSG00000215483	0,41458238	-1,2410984	0,67766406	0,43110234	0,70321282	LINC00598	long intergenic non-protein coding RNA 598 [Source:HGNC Symbol;Acc:HGNC:42770]
ENSG00000037757	0,17009543	3,65242609	0,67732824	0,43121262	0,70323567	MRI1	methylthioribose-1-phosphate isomerase 1 [Source:HGNC Symbol;Acc:HGNC:28469]
ENSG00000182087	-0,1236312	6,91788867	0,67745319	0,43117158	0,70323567	TMEM259	transmembrane protein 259 [Source:HGNC Symbol;Acc:HGNC:17039]
ENSG00000232938	-0,2294846	0,55304175	0,6769781	0,43132766	0,70334476	RPL23AP87	ribosomal protein L23a pseudogene 87 [Source:HGNC Symbol;Acc:HGNC:51578]
ENSG00000132478	-0,1221513	4,47650867	0,67634613	0,43153542	0,7035265	UNK	unk zinc finger [Source:HGNC Symbol;Acc:HGNC:29369]
ENSG00000213885	-0,2054621	1,70324556	0,67637279	0,43152665	0,7035265	RPL13AP7	ribosomal protein L13a pseudogene 7 [Source:HGNC Symbol;Acc:HGNC:23755]
ENSG00000121769	-0,4046937	-1,6050197	0,67409422	0,43227708	0,70449977	FABP3	fatty acid binding protein 3 [Source:HGNC Symbol;Acc:HGNC:3557]
ENSG00000147044	0,14295998	4,5846584	0,6741017	0,43227462	0,70449977	CASK	calcium/calmodulin dependent serine protein kinase [Source:HGNC Symbol;Acc:HGNC:1497]
ENSG00000173762	0,11975103	7,58663484	0,67423221	0,43223158	0,70449977	CD7	CD7 molecule [Source:HGNC Symbol;Acc:HGNC:1695]
ENSG00000108784	-0,1322769	6,26998719	0,67316502	0,43258373	0,70461171	NAGLU	N-acetyl-alpha-glucosaminidase [Source:HGNC Symbol;Acc:HGNC:7632]
ENSG00000118137	0,10999996	12,2326324	0,67315533	0,43258693	0,70461171	APOA1	apolipoprotein A1 [Source:HGNC Symbol;Acc:HGNC:600]
ENSG00000132003	-0,3111418	-0,6687306	0,67325018	0,43255561	0,70461171	ZSWIM4	zinc finger SWIM-type containing 4 [Source:HGNC Symbol;Acc:HGNC:25704]
ENSG00000171262	0,15317332	3,8825332	0,67354969	0,43245674	0,70461171	FAM98B	family with sequence similarity 98 member B [Source:HGNC Symbol;Acc:HGNC:26773]
ENSG00000231527	-0,3131098	-0,6329258	0,67333855	0,43252644	0,70461171	FAM27C	family with sequence similarity 27 member C [Source:HGNC Symbol;Acc:HGNC:23668]
ENSG00000146216	0,42369007	-1,3489352	0,67296058	0,43265125	0,70463791	TTBK1	tau tubulin kinase 1 [Source:HGNC Symbol;Acc:HGNC:19140]
ENSG00000186767	0,22601625	0,39255708	0,67276976	0,43271428	0,70466201	SPIN4	spindlin family member 4 [Source:HGNC Symbol;Acc:HGNC:27040]
ENSG00000120805	0,15741669	7,19765221	0,67212913	0,432926	0,70484966	ARL1	ADP ribosylation factor like GTPase 1 [Source:HGNC Symbol;Acc:HGNC:692]
ENSG00000204469	0,18397308	7,46910807	0,67218611	0,43290717	0,70484966	PRRC2A	proline rich coiled-coil 2A [Source:HGNC Symbol;Acc:HGNC:13918]
ENSG00000128050	-0,1403337	8,28864984	0,67165185	0,43308386	0,70502809	PAICS	phosphoribosylaminoimidazole carboxylase and phosphoribosylaminoimidazolesuccinocarboxamide synthase [So
ENSG00000101384	-0,2786505	7,4325818	0,67127058	0,43321002	0,70513207	JAG1	jagged canonical Notch ligand 1 [Source:HGNC Symbol;Acc:HGNC:6188]

ENSG00000104765	-0,1897559	4,10420746	0,67102134	0,43329253	0,70513207	BNIP3L	BCL2 interacting protein 3 like [Source:HGNC Symbol;Acc:HGNC:1085]
ENSG00000226701	0,32760859	-1,0226892	0,67109942	0,43326668	0,70513207	RPL15P14	ribosomal protein L15 pseudogene 14 [Source:HGNC Symbol;Acc:HGNC:36487]
ENSG00000184154	-0,4660288	-1,5242365	0,67084517	0,43335087	0,70514845	LRTOMT	leucine rich transmembrane and O-methyltransferase domain containing [Source:HGNC Symbol;Acc:HGNC:25033
ENSG00000135622	0,1284513	4,92213378	0,67030981	0,43352822	0,70535848	SEMA4F	ssemaphorin 4F [Source:HGNC Symbol;Acc:HGNC:10734]
ENSG00000120159	-0,1309949	4,10905225	0,66978466	0,43370231	0,70552422	CAAP1	caspase activity and apoptosis inhibitor 1 [Source:HGNC Symbol;Acc:HGNC:25834]
ENSG00000131475	0,1062197	8,59476535	0,66971119	0,43372668	0,70552422	VPS25	vacuolar protein sorting 25 homolog [Source:HGNC Symbol;Acc:HGNC:28122]
ENSG00000056558	-0,3506757	-0,9586526	0,66942084	0,43382299	0,70554316	TRAF1	TNF receptor associated factor 1 [Source:HGNC Symbol;Acc:HGNC:12031]
ENSG00000163050	0,39938478	-1,703111	0,6693849	0,43383491	0,70554316	COQ8A	coenzyme Q8A [Source:HGNC Symbol;Acc:HGNC:16812]
ENSG00000105227	0,26844077	-0,0459754	0,66834503	0,43418015	0,7056333	PRX	periaxin [Source:HGNC Symbol;Acc:HGNC:13797]
ENSG00000116731	0,18903764	2,16422589	0,66892219	0,43398847	0,7056333	PRDM2	PR/SET domain 2 [Source:HGNC Symbol;Acc:HGNC:9347]
ENSG00000178982	-0,1083396	8,31873706	0,66862877	0,4340859	0,7056333	EIF3K	eukaryotic translation initiation factor 3 subunit K [Source:HGNC Symbol;Acc:HGNC:24656]
ENSG00000257913	-0,3046715	1,11502869	0,66837555	0,43417001	0,7056333	DDN-AS1	DDN and PRKAG1 antisense RNA 1 [Source:HGNC Symbol;Acc:HGNC:53464]
ENSG00000274929	0,69518874	-2,0930278	0,66839278	0,43416428	0,7056333	AL157813.1	novel transcript, sense intronic to RCBTB2
ENSG00000280620	-0,5095301	-1,8551627	0,66866744	0,43407306	0,7056333	SCAANT1	SCA7/ATXN7 antisense RNA 1 [Source:HGNC Symbol;Acc:HGNC:43718]
ENSG00000118514	0,38158136	-1,0744774	0,66785003	0,43434464	0,70566514	ALDH8A1	aldehyde dehydrogenase 8 family member A1 [Source:HGNC Symbol;Acc:HGNC:15471]
ENSG00000179412	-0,2877408	-0,1968158	0,66803685	0,43428255	0,70566514	HNRNPCL4	heterogeneous nuclear ribonucleoprotein C like 4 [Source:HGNC Symbol;Acc:HGNC:51333]
ENSG00000203279	-0,1854473	1,49902737	0,66785535	0,43434288	0,70566514	AL590705.1	novel transcript
ENSG00000005700	-0,1428228	5,21833336	0,6675786	0,43443489	0,70570961	IBTK	inhibitor of Bruton tyrosine kinase [Source:HGNC Symbol;Acc:HGNC:17853]
ENSG00000197249	-0,1414406	13,5961928	0,66747714	0,43446863	0,70570961	SERPINA1	serpin family A member 1 [Source:HGNC Symbol;Acc:HGNC:8941]
ENSG00000103546	-0,3076738	-0,0551886	0,66731045	0,43452408	0,7057212	SLC6A2	solute carrier family 6 member 2 [Source:HGNC Symbol;Acc:HGNC:11048]
ENSG00000164181	0,17109236	2,85266213	0,66708928	0,43459766	0,70576225	ELOVL7	ELOVL fatty acid elongase 7 [Source:HGNC Symbol;Acc:HGNC:26292]
ENSG00000141564	0,15408419	5,21133694	0,66677089	0,43470362	0,70577742	RPTOR	regulatory associated protein of MTOR complex 1 [Source:HGNC Symbol;Acc:HGNC:30287]
ENSG00000166710	0,1185367	9,39053247	0,66689136	0,43466352	0,70577742	B2M	beta-2-microglobulin [Source:HGNC Symbol;Acc:HGNC:914]
ENSG00000111237	0,11691067	6,23337522	0,66622192	0,43488642	0,70599575	VPS29	VPS29 retromer complex component [Source:HGNC Symbol;Acc:HGNC:14340]
ENSG00000117242	-0,3037027	2,86127434	0,6659974	0,43496123	0,70602548	PINK1-AS	PINK1 antisense RNA [Source:HGNC Symbol;Acc:HGNC:38872]
ENSG00000188153	0,24231292	4,12202931	0,66587686	0,4350014	0,70602548	COL4A5	collagen type IV alpha 5 chain [Source:HGNC Symbol;Acc:HGNC:2207]
ENSG00000108559	-0,1241485	6,20424112	0,66544185	0,4351464	0,70602552	NUP88	nucleoporin 88 [Source:HGNC Symbol;Acc:HGNC:8067]
ENSG00000127603	0,18855285	5,13962342	0,66547779	0,43513442	0,70602552	MACF1	microtubule actin crosslinking factor 1 [Source:HGNC Symbol;Acc:HGNC:13664]
ENSG00000141349	0,13661698	7,28942517	0,66550557	0,43512516	0,70602552	G6PC3	glucose-6-phosphatase catalytic subunit 3 [Source:HGNC Symbol;Acc:HGNC:24861]
ENSG00000115816	-0,1406148	5,99116623	0,66512475	0,43525216	0,70604028	CEBPZ	CCAAT enhancer binding protein zeta [Source:HGNC Symbol;Acc:HGNC:24218]
ENSG00000188342	0,12445746	5,77242441	0,66525973	0,43520714	0,70604028	GTF2F2	general transcription factor IIF subunit 2 [Source:HGNC Symbol;Acc:HGNC:4653]
ENSG00000243406	0,45936006	-1,4387003	0,66495042	0,43531032	0,70605623	MRPS31P5	mitochondrial ribosomal protein S31 pseudogene 5 [Source:HGNC Symbol;Acc:HGNC:39253]
ENSG00000121060	0,13977609	5,73412889	0,66467312	0,43540285	0,70609474	TRIM25	tripartite motif containing 25 [Source:HGNC Symbol;Acc:HGNC:12932]
ENSG00000162105	0,26120246	3,33503471	0,66458961	0,43543073	0,70609474	SHANK2	SH3 and multiple ankyrin repeat domains 2 [Source:HGNC Symbol;Acc:HGNC:14295]
ENSG00000113732	0,12029993	7,84981762	0,66352326	0,43578692	0,70647308	ATP6V0E1	ATPase H+ transporting V0 subunit e1 [Source:HGNC Symbol;Acc:HGNC:863]
ENSG00000153815	0,18677233	5,28079102	0,66327121	0,43587118	0,70647308	CMIP	c-Maf inducing protein [Source:HGNC Symbol;Acc:HGNC:24319]
ENSG00000161638	-0,3913425	-0,8763311	0,66351976	0,43578809	0,70647308	ITGA5	integrin subunit alpha 5 [Source:HGNC Symbol;Acc:HGNC:6141]
ENSG00000196470	0,19361753	3,48745252	0,66316758	0,43590583	0,70647308	SIAH1	siah E3 ubiquitin protein ligase 1 [Source:HGNC Symbol;Acc:HGNC:10857]
ENSG00000204120	0,24895065	5,44502099	0,66324638	0,43587948	0,70647308	GIGYF2	GRB10 interacting GYF protein 2 [Source:HGNC Symbol;Acc:HGNC:11960]
ENSG00000108578	0,11473233	6,61877657	0,66238626	0,43616724	0,70669183	BLMH	bleomycin hydrolase [Source:HGNC Symbol;Acc:HGNC:1059]
ENSG00000167476	-0,3151785	-0,3660597	0,66244363	0,43614804	0,70669183	JSRP1	junctional sarcoplasmic reticulum protein 1 [Source:HGNC Symbol;Acc:HGNC:24963]
ENSG00000233461	-0,2817745	0,69160257	0,66233042	0,43618593	0,70669183	AL445524.1	novel transcript
ENSG00000038219	0,32772396	4,285805	0,66185707	0,43634444	0,70687025	BOD1L1	biorientation of chromosomes in cell division 1 like 1 [Source:HGNC Symbol;Acc:HGNC:31792]
ENSG00000180329	0,1286371	5,23481907	0,66153886	0,43645105	0,70696456	CCDC43	coiled-coil domain containing 43 [Source:HGNC Symbol;Acc:HGNC:26472]

ENSG00000262580	0,36536526	-1,6008629	0,66102547	0,43662314	0,70711941	AC087741.1	novel transcript, antisense to CARD14
ENSG00000272142	-0,2926784	-0,634352	0,6609649	0,43664345	0,70711941	LYRM4-AS1	LYRM4 antisense RNA 1 [Source:NCBI gene (formerly Entrezgene);Acc:100129461]
ENSG00000067248	0,13160332	4,56241394	0,66066526	0,43674395	0,70720377	DHX29	DExH-box helicase 29 [Source:HGNC Symbol;Acc:HGNC:15815]
ENSG00000204859	0,12772746	3,75335178	0,66050152	0,43679889	0,70721434	ZBTB48	zinc finger and BTB domain containing 48 [Source:HGNC Symbol;Acc:HGNC:4930]
ENSG00000111684	0,17957373	6,89856903	0,66017999	0,4369068	0,70731066	LPCAT3	lysophosphatidylcholine acyltransferase 3 [Source:HGNC Symbol;Acc:HGNC:30244]
ENSG00000114316	0,14175502	5,17810532	0,65959852	0,43710206	0,70754836	USP4	ubiquitin specific peptidase 4 [Source:HGNC Symbol;Acc:HGNC:12627]
ENSG00000105993	0,15847865	5,90837797	0,65904032	0,43728964	0,70772091	DNAJB6	DnaJ heat shock protein family (Hsp40) member B6 [Source:HGNC Symbol;Acc:HGNC:14888]
ENSG00000178338	0,1648319	1,43712913	0,65899301	0,43730554	0,70772091	ZNF354B	zinc finger protein 354B [Source:HGNC Symbol;Acc:HGNC:17197]
ENSG00000125485	0,13721269	5,23660919	0,65854321	0,4374568	0,70780888	DDX31	DEAD-box helicase 31 [Source:HGNC Symbol;Acc:HGNC:16715]
ENSG00000262089	-0,5350006	-1,2199473	0,65862619	0,43742889	0,70780888	AC040977.1	novel transcript
ENSG00000160439	-0,1279636	3,92491241	0,65766284	0,43775311	0,70820987	RDH13	retinol dehydrogenase 13 [Source:HGNC Symbol;Acc:HGNC:19978]
ENSG00000177830	-0,1080662	7,74885811	0,65702287	0,43796871	0,70848022	CHID1	chitinase domain containing 1 [Source:HGNC Symbol;Acc:HGNC:28474]
ENSG00000117592	0,11052219	9,16158185	0,65659711	0,43811224	0,70863394	PRDX6	peroxiredoxin 6 [Source:HGNC Symbol;Acc:HGNC:16753]
ENSG00000263766	-0,5181915	-0,9635843	0,65603847	0,4383007	0,70886027	AC025682.1	novel transcript, antisense to NPEPPS
ENSG00000265491	-0,1303064	4,85937813	0,65584571	0,43836575	0,70888701	RNF115	ring finger protein 115 [Source:HGNC Symbol;Acc:HGNC:18154]
ENSG00000065183	-0,1771199	5,85698349	0,65464295	0,43877204	0,70942391	WDR3	WD repeat domain 3 [Source:HGNC Symbol;Acc:HGNC:12755]
ENSG00000242087	0,42086357	-1,7932445	0,65457539	0,43879489	0,70942391	RPL36AP41	ribosomal protein L36a pseudogene 41 [Source:HGNC Symbol;Acc:HGNC:35813]
ENSG00000132612	-0,1135251	5,69429626	0,65427828	0,43889536	0,70950783	VPS4A	vacuolar protein sorting 4 homolog A [Source:HGNC Symbol;Acc:HGNC:13488]
ENSG00000107625	-0,1135203	5,21394537	0,65377468	0,43906574	0,70970473	DDX50	DExD-box helicase 50 [Source:HGNC Symbol;Acc:HGNC:17906]
ENSG00000100811	-0,110473	5,28916235	0,65328309	0,43923216	0,70974478	YY1	YY1 transcription factor [Source:HGNC Symbol;Acc:HGNC:12856]
ENSG00000111640	-0,1243674	12,1953556	0,65349747	0,43915957	0,70974478	GAPDH	glyceraldehyde-3-phosphate dehydrogenase [Source:HGNC Symbol;Acc:HGNC:4141]
ENSG00000196214	0,13807626	2,86407961	0,65327098	0,43923627	0,70974478	ZNF766	zinc finger protein 766 [Source:HGNC Symbol;Acc:HGNC:28063]
ENSG00000167702	-0,1536566	2,20091333	0,65292288	0,43935418	0,7097783	KIFC2	kinesin family member C2 [Source:HGNC Symbol;Acc:HGNC:29530]
ENSG00000215717	0,14363308	3,85926251	0,65303145	0,43931739	0,7097783	TMEM167B	transmembrane protein 167B [Source:HGNC Symbol;Acc:HGNC:30187]
ENSG00000002586	0,11580966	9,04543208	0,65228251	0,43957123	0,70989589	CD99	CD99 molecule (Xg blood group) [Source:HGNC Symbol;Acc:HGNC:7082]
ENSG00000075336	0,21046164	4,92161404	0,65228197	0,43957141	0,70989589	TIMM21	translocase of inner mitochondrial membrane 21 [Source:HGNC Symbol;Acc:HGNC:25010]
ENSG00000104687	-0,1160763	7,51257441	0,65227805	0,43957274	0,70989589	GSR	glutathione-disulfide reductase [Source:HGNC Symbol;Acc:HGNC:4623]
ENSG00000168273	-0,1412735	4,22978293	0,65168183	0,43977499	0,71014401	SMIM4	small integral membrane protein 4 [Source:HGNC Symbol;Acc:HGNC:37257]
ENSG00000009724	-0,1131726	4,89942749	0,65100003	0,44000646	0,71020378	MASP2	mannan binding lectin serine peptidase 2 [Source:HGNC Symbol;Acc:HGNC:6902]
ENSG00000141759	-0,1406426	7,13831675	0,65133465	0,43989283	0,71020378	TXNL4A	thioredoxin like 4A [Source:HGNC Symbol;Acc:HGNC:30551]
ENSG00000171735	-0,1427348	3,9140373	0,65110663	0,43997026	0,71020378	CAMTA1	calmodulin binding transcription activator 1 [Source:HGNC Symbol;Acc:HGNC:18806]
ENSG00000225224	-0,2159273	0,70299027	0,6510329	0,43999529	0,71020378	RPS27AP12	RPS27A pseudogene 12 [Source:HGNC Symbol;Acc:HGNC:35732]
ENSG00000114861	0,22121997	2,81467968	0,65069658	0,44010954	0,71025037	FOXP1	forkhead box P1 [Source:HGNC Symbol;Acc:HGNC:3823]
ENSG00000258282	0,37076451	-1,1739491	0,65062884	0,44013256	0,71025037	BTF3P2	BTF3 pseudogene 2 [Source:HGNC Symbol;Acc:HGNC:19924]
ENSG00000015171	-0,1241086	4,63838241	0,64969776	0,44044914	0,7104513	ZMYND11	zinc finger MYND-type containing 11 [Source:HGNC Symbol;Acc:HGNC:16966]
ENSG00000089199	0,32190623	1,007956	0,64931158	0,44058056	0,7104513	CHGB	chromogranin B [Source:HGNC Symbol;Acc:HGNC:1930]
ENSG00000103145	-0,1472626	4,60642633	0,64911898	0,44064612	0,7104513	HCFC1R1	host cell factor C1 regulator 1 [Source:HGNC Symbol;Acc:HGNC:21198]
ENSG00000123009	-0,2357831	0,36563581	0,6495645	0,44049448	0,7104513	NME2P1	NME2 pseudogene 1 [Source:HGNC Symbol;Acc:HGNC:31358]
ENSG00000144161	0,12068147	3,45363666	0,64964909	0,4404657	0,7104513	ZC3H8	zinc finger CCCH-type containing 8 [Source:HGNC Symbol;Acc:HGNC:30941]
ENSG00000175048	-0,1243521	3,42300804	0,6498178	0,4404083	0,7104513	ZDHHC14	zinc finger DHHC-type palmitoyltransferase 14 [Source:HGNC Symbol;Acc:HGNC:20341]
ENSG00000217930	-0,3159288	-0,3242469	0,64922784	0,44060906	0,7104513	PAM16	presequence translocase associated motor 16 [Source:HGNC Symbol;Acc:HGNC:29679]
ENSG00000223722	-0,3886914	-1,3324396	0,64920633	0,44061639	0,7104513	IFITM3P2	IFITM3 pseudogene 2 [Source:HGNC Symbol;Acc:HGNC:54539]
ENSG00000118004	-0,1559551	2,31111742	0,64889753	0,44072153	0,71049447	COLEC11	collectin subfamily member 11 [Source:HGNC Symbol;Acc:HGNC:17213]
ENSG00000064961	-0,1265994	5,09791438	0,6486131	0,44081842	0,71057224	HMG20B	high mobility group 20B [Source:HGNC Symbol;Acc:HGNC:5002]

ENSG00000148541	0,38595271	-0,7408572	0,64816993	0,44096944	0,71073727	FAM13C	family with sequence similarity 13 member C [Source:HGNC Symbol;Acc:HGNC:19371]
ENSG00000130997	-0,6624126	-1,7893476	0,647399	0,44123237	0,71079307	POLN	DNA polymerase nu [Source:HGNC Symbol;Acc:HGNC:18870]
ENSG00000141858	-0,123172	5,21421496	0,64714768	0,44131815	0,71079307	SAMD1	sterile alpha motif domain containing 1 [Source:HGNC Symbol;Acc:HGNC:17958]
ENSG00000142207	0,17655555	5,30709785	0,64692753	0,4413933	0,71079307	URB1	URB1 ribosome biogenesis homolog [Source:HGNC Symbol;Acc:HGNC:17344]
ENSG00000149657	0,1090265	6,11762239	0,64727016	0,44127634	0,71079307	LSM14B	LSM family member 14B [Source:HGNC Symbol;Acc:HGNC:15887]
ENSG00000157353	-0,1885408	2,87601257	0,64729029	0,44126947	0,71079307	FCSK	fucose kinase [Source:HGNC Symbol;Acc:HGNC:29500]
ENSG00000179409	-0,1234061	5,39598423	0,64782753	0,44108619	0,71079307	GEMIN4	gem nuclear organelle associated protein 4 [Source:HGNC Symbol;Acc:HGNC:15717]
ENSG00000230362	0,28591672	-0,6011005	0,64742207	0,4412245	0,71079307	ACTG1P23	actin gamma 1 pseudogene 23 [Source:HGNC Symbol;Acc:HGNC:51503]
ENSG00000232496	0,28623041	-0,5980568	0,64702122	0,44136131	0,71079307	RPL3P12	ribosomal protein L3 pseudogene 12 [Source:HGNC Symbol;Acc:HGNC:36298]
ENSG00000163558	0,12993401	4,43048323	0,6466266	0,44149607	0,7108802	PRKCI	protein kinase C iota [Source:HGNC Symbol;Acc:HGNC:9404]
ENSG00000064300	0,25033968	3,96541387	0,64586318	0,44175695	0,71089357	NGFR	nerve growth factor receptor [Source:HGNC Symbol;Acc:HGNC:7809]
ENSG00000126602	0,15444965	8,0336407	0,64574799	0,44179634	0,71089357	TRAP1	TNF receptor associated protein 1 [Source:HGNC Symbol;Acc:HGNC:16264]
ENSG00000128536	-0,2002957	1,23474056	0,64624004	0,44162814	0,71089357	CDHR3	cadherin related family member 3 [Source:HGNC Symbol;Acc:HGNC:26308]
ENSG00000146707	0,17655465	1,98698887	0,64609832	0,44167657	0,71089357	POMZP3	POM121 and ZP3 fusion [Source:HGNC Symbol;Acc:HGNC:9203]
ENSG00000150756	0,16286657	3,95216273	0,64609324	0,44167831	0,71089357	ATPSCKMT	ATP synthase c subunit lysine N-methyltransferase [Source:HGNC Symbol;Acc:HGNC:27029]
ENSG00000233719	0,37046442	-1,5868958	0,64578359	0,44178417	0,71089357	GOT2P3	GOT2 pseudogene 3 [Source:HGNC Symbol;Acc:HGNC:4436]
ENSG00000138430	-0,1069077	7,91070598	0,64504441	0,44203704	0,71120255	OLA1	Obg like ATPase 1 [Source:HGNC Symbol;Acc:HGNC:28833]
ENSG00000088682	0,1097969	6,05694937	0,64427145	0,44230173	0,71126877	COQ9	coenzyme Q9 [Source:HGNC Symbol;Acc:HGNC:25302]
ENSG00000128463	-0,1291529	7,10614353	0,64454637	0,44220756	0,71126877	EMC4	ER membrane protein complex subunit 4 [Source:HGNC Symbol;Acc:HGNC:28032]
ENSG00000136286	-0,3724796	-0,975756	0,64399522	0,44239638	0,71126877	MYO1G	myosin IG [Source:HGNC Symbol;Acc:HGNC:13880]
ENSG00000155980	-0,2365598	1,26796856	0,64392922	0,44241901	0,71126877	KIF5A	kinesin family member 5A [Source:HGNC Symbol;Acc:HGNC:6323]
ENSG00000181038	-0,1179079	4,34746503	0,64397005	0,44240501	0,71126877	METTL23	methyltransferase like 23 [Source:HGNC Symbol;Acc:HGNC:26988]
ENSG00000233382	0,33515005	0,14138812	0,64394971	0,44241198	0,71126877	NKAPP1	NFKB activating protein pseudogene 1 [Source:HGNC Symbol;Acc:HGNC:26706]
ENSG00000278916	0,38027288	-1,7016113	0,64441401	0,44225289	0,71126877	CEP83-DT	CEP83 divergent transcript [Source:HGNC Symbol;Acc:HGNC:27055]
ENSG00000115993	0,14857226	3,46976047	0,64375506	0,44247871	0,71128647	TRAK2	trafficking kinesin protein 2 [Source:HGNC Symbol;Acc:HGNC:13206]
ENSG00000110944	-0,3852511	-1,5763692	0,64337408	0,44260935	0,71132272	IL23A	interleukin 23 subunit alpha [Source:HGNC Symbol;Acc:HGNC:15488]
ENSG00000137845	0,14590951	6,36015646	0,64326337	0,44264733	0,71132272	ADAM10	ADAM metallopeptidase domain 10 [Source:HGNC Symbol;Acc:HGNC:188]
ENSG00000183684	0,11343287	8,19256696	0,64352828	0,44255647	0,71132272	ALYREF	Aly/REF export factor [Source:HGNC Symbol;Acc:HGNC:19071]
ENSG00000260742	-0,5337928	-1,8246146	0,64289406	0,44277406	0,71144811	AC009962.1	novel transcript, antisense to SSFA2
ENSG00000117318	0,11356861	7,27368774	0,64236765	0,44295479	0,71166024	ID3	inhibitor of DNA binding 3, HLH protein [Source:HGNC Symbol;Acc:HGNC:5362]
ENSG00000253953	0,30790284	1,07524526	0,64222388	0,44300417	0,71166131	PCDHGB4	protocadherin gamma subfamily B, 4 [Source:HGNC Symbol;Acc:HGNC:8711]
ENSG00000140464	0,14452246	3,62607589	0,64115973	0,44336998	0,71201407	PML	PML nuclear body scaffold [Source:HGNC Symbol;Acc:HGNC:9113]
ENSG00000172336	-0,1425635	5,5258002	0,64128971	0,44332527	0,71201407	POP7	POP7 homolog, ribonuclease P/MRP subunit [Source:HGNC Symbol;Acc:HGNC:19949]
ENSG00000176049	0,37096252	-1,2612573	0,64143304	0,44327598	0,71201407	JAKMIP2	janus kinase and microtubule interacting protein 2 [Source:HGNC Symbol;Acc:HGNC:29067]
ENSG00000092140	0,11668946	4,70074195	0,64078072	0,44350038	0,71207659	G2E3	G2/M-phase specific E3 ubiquitin protein ligase [Source:HGNC Symbol;Acc:HGNC:20338]
ENSG00000185130	-0,3854373	-0,8926535	0,64076326	0,44350639	0,71207659	H2BC13	H2B clustered histone 13 [Source:HGNC Symbol;Acc:HGNC:4748]
ENSG00000283041	-0,1313886	3,92007993	0,64054203	0,44358255	0,7121206	AC008038.1	eukaryotic translation elongation factor 1 gamma (
ENSG00000063176	-0,1596897	2,83632293	0,63997295	0,44377854	0,71212221	SPHK2	sphingosine kinase 2 [Source:HGNC Symbol;Acc:HGNC:18859]
ENSG00000067829	-0,1493804	5,16450404	0,64015863	0,44371457	0,71212221	IDH3G	isocitrate dehydrogenase (NAD(+)) 3 non-catalytic subunit gamma [Source:HGNC Symbol;Acc:HGNC:5386]
ENSG00000079387	0,12034689	3,34326163	0,64020314	0,44369924	0,71212221	SENP1	SUMO specific peptidase 1 [Source:HGNC Symbol;Acc:HGNC:17927]
ENSG00000187244	0,11551676	7,94188236	0,6400683	0,44374569	0,71212221	BCAM	basal cell adhesion molecule (Lutheran blood group) [Source:HGNC Symbol;Acc:HGNC:6722]
ENSG00000110243	-0,1093749	5,4934246	0,63946626	0,44395316	0,71216775	APOA5	apolipoprotein A5 [Source:HGNC Symbol;Acc:HGNC:17288]
ENSG00000123064	-0,1481943	6,93145265	0,6395463	0,44392557	0,71216775	DDX54	DEAD-box helicase 54 [Source:HGNC Symbol;Acc:HGNC:20084]
ENSG00000261420	-0,4494223	-1,4159123	0,63952411	0,44393322	0,71216775	AL022069.1	novel transcript, antisense to BRP44L

ENSG00000137713	0,12240697	5,16541047	0,63907552	0,4440879	0,71230568	PPP2R1B	protein phosphatase 2 scaffold subunit Abeta [Source:HGNC Symbol;Acc:HGNC:9303]
ENSG00000183155	0,16501128	3,32251273	0,63883277	0,44417164	0,71236179	RABIF	RAB interacting factor [Source:HGNC Symbol;Acc:HGNC:9797]
ENSG00000021300	0,12120796	5,99950882	0,63799232	0,44446178	0,71248518	PLEKHB1	pleckstrin homology domain containing B1 [Source:HGNC Symbol;Acc:HGNC:19079]
ENSG00000104299	-0,1417002	4,06224902	0,63806269	0,44443748	0,71248518	INTS9	integrator complex subunit 9 [Source:HGNC Symbol;Acc:HGNC:25592]
ENSG00000117480	0,15746935	2,93589485	0,63801613	0,44445356	0,71248518	FAAH	fatty acid amide hydrolase [Source:HGNC Symbol;Acc:HGNC:3553]
ENSG00000134697	-0,1643539	6,44925892	0,63788839	0,44449769	0,71248518	GNL2	G protein nucleolar 2 [Source:HGNC Symbol;Acc:HGNC:29925]
ENSG00000137491	0,13484613	2,70575158	0,6376486	0,44458053	0,71248518	SLCO2B1	solute carrier organic anion transporter family member 2B1 [Source:HGNC Symbol;Acc:HGNC:10962]
ENSG00000182150	0,13518396	3,6434408	0,6376213	0,44458997	0,71248518	ERCC6L2	ERCC excision repair 6 like 2 [Source:HGNC Symbol;Acc:HGNC:26922]
ENSG00000228158	0,37080952	-1,3062784	0,63813961	0,44441091	0,71248518	TLE1P1	TLE family member 1 pseudogene 1 [Source:HGNC Symbol;Acc:HGNC:39438]
ENSG00000286753	-0,4635699	-2,0823124	0,63724662	0,44471948	0,71261456	AC091978.1	novel transcript, antisense to ICE1
ENSG00000132466	0,23154568	5,25628883	0,63660834	0,44494026	0,7127338	ANKRD17	ankyrin repeat domain 17 [Source:HGNC Symbol;Acc:HGNC:23575]
ENSG00000167562	0,21965546	0,9010779	0,63672087	0,44490132	0,7127338	ZNF701	zinc finger protein 701 [Source:HGNC Symbol;Acc:HGNC:25597]
ENSG00000217716	-0,1716351	1,23311722	0,63664078	0,44492903	0,7127338	RPS10P3	ribosomal protein S10 pseudogene 3 [Source:HGNC Symbol;Acc:HGNC:23684]
ENSG00000178297	-0,1505188	2,04901593	0,63565327	0,44527095	0,71313777	TMPRSS9	transmembrane serine protease 9 [Source:HGNC Symbol;Acc:HGNC:30079]
ENSG00000260196	0,20358963	3,55179753	0,63559806	0,44529008	0,71313777	AC124798.1	antisense to KCNJ11 and overlapping to a novel gene
ENSG00000123131	-0,1057393	8,91617624	0,63434127	0,4457259	0,71364688	PRDX4	peroxiredoxin 4 [Source:HGNC Symbol;Acc:HGNC:17169]
ENSG00000171433	0,34283676	-0,8009871	0,63425881	0,44575452	0,71364688	GLOD5	glyoxalase domain containing 5 [Source:HGNC Symbol;Acc:HGNC:33358]
ENSG00000272886	0,19873651	3,79043221	0,63427857	0,44574766	0,71364688	DCP1A	decapping mRNA 1A [Source:HGNC Symbol;Acc:HGNC:18714]
ENSG00000183621	-0,418211	-1,274785	0,63405414	0,44582557	0,71368242	ZNF438	zinc finger protein 438 [Source:HGNC Symbol;Acc:HGNC:21029]
ENSG00000166272	0,11791174	4,51092773	0,63377756	0,44592161	0,71375795	WBP1L	WW domain binding protein 1 like [Source:HGNC Symbol;Acc:HGNC:23510]
ENSG00000232794	-0,31467	-1,0402203	0,63349087	0,4460212	0,71383915	HNRNPDP1	heterogeneous nuclear ribonucleoprotein D pseudogene 1 [Source:HGNC Symbol;Acc:HGNC:5038]
ENSG00000084072	0,11485266	4,4093599	0,63292433	0,44621812	0,71402347	PPIE	peptidylprolyl isomerase E [Source:HGNC Symbol;Acc:HGNC:9258]
ENSG00000205571	0,17799946	2,86970652	0,6328783	0,44623412	0,71402347	SMN1	survival of motor neuron 1, telomeric [Source:HGNC Symbol;Acc:HGNC:11117]
ENSG00000169925	0,21169908	4,01960488	0,63228273	0,4464413	0,71427674	BRD3	bromodomain containing 3 [Source:HGNC Symbol;Acc:HGNC:1104]
ENSG00000089818	0,11979997	4,08672483	0,63185924	0,44658871	0,71443435	NECAP1	NECAP endocytosis associated 1 [Source:HGNC Symbol;Acc:HGNC:24539]
ENSG00000188315	-0,1759697	1,30079072	0,63103731	0,44687505	0,71473589	C3orf62	chromosome 3 open reading frame 62 [Source:HGNC Symbol;Acc:HGNC:24771]
ENSG00000262160	-0,3508021	-0,0131401	0,63105935	0,44686737	0,71473589	AC020978.5	novel transcript, antisense to NFATC3
ENSG00000022976	0,13940714	3,24879887	0,63063146	0,44701655	0,7148397	ZNF839	zinc finger protein 839 [Source:HGNC Symbol;Acc:HGNC:20345]
ENSG00000086598	0,11696166	8,53756734	0,63057049	0,44703782	0,7148397	TMED2	transmembrane p24 trafficking protein 2 [Source:HGNC Symbol;Acc:HGNC:16996]
ENSG00000147459	0,2426496	3,09020798	0,6301763	0,44717534	0,71498134	DOCK5	dedicator of cytokinesis 5 [Source:HGNC Symbol;Acc:HGNC:23476]
ENSG00000100897	-0,1403612	7,6081988	0,62903902	0,4475725	0,71509734	DCAF11	DDB1 and CUL4 associated factor 11 [Source:HGNC Symbol;Acc:HGNC:20258]
ENSG00000122203	0,13790163	5,01599461	0,62940626	0,44744418	0,71509734	KIAA1191	KIAA1191 [Source:HGNC Symbol;Acc:HGNC:29209]
ENSG00000140650	-0,1052226	5,91039702	0,62950414	0,44740999	0,71509734	PMM2	phosphomannomutase 2 [Source:HGNC Symbol;Acc:HGNC:9115]
ENSG00000163659	0,15514847	2,61942153	0,62898741	0,44759053	0,71509734	TIPARP	TCDD inducible poly(ADP-ribose) polymerase [Source:HGNC Symbol;Acc:HGNC:23696]
ENSG00000167635	-0,132483	4,67264598	0,62902405	0,44757773	0,71509734	ZNF146	zinc finger protein 146 [Source:HGNC Symbol;Acc:HGNC:12931]
ENSG00000171295	0,15549616	3,15288272	0,62968914	0,44734539	0,71509734	ZNF440	zinc finger protein 440 [Source:HGNC Symbol;Acc:HGNC:20874]
ENSG00000277952	-0,4084926	-1,9537357	0,62948758	0,44741578	0,71509734	AC092745.3	small nuclear ribonucleoprotein polypeptide C (SNRPC) pseudogene
ENSG00000107537	-0,1131533	5,51008077	0,62831441	0,44782585	0,71523881	PHYH	phytanoyl-CoA 2-hydroxylase [Source:HGNC Symbol;Acc:HGNC:8940]
ENSG00000185187	0,26495615	-0,3489256	0,62837645	0,44780415	0,71523881	SIGIRR	single Ig and TIR domain containing [Source:HGNC Symbol;Acc:HGNC:30575]
ENSG00000240366	-0,3243285	-1,2106049	0,62831411	0,44782596	0,71523881	RPL36AP45	ribosomal protein L36a pseudogene 45 [Source:HGNC Symbol;Acc:HGNC:36032]
ENSG00000160190	0,50746149	-1,7259645	0,62815972	0,44787997	0,71524688	SLC37A1	solute carrier family 37 member 1 [Source:HGNC Symbol;Acc:HGNC:11024]
ENSG00000126254	0,10777459	6,64476547	0,6277013	0,44804041	0,7153176	RBM42	RNA binding motif protein 42 [Source:HGNC Symbol;Acc:HGNC:28117]
ENSG00000182704	0,10609625	6,13579898	0,62781897	0,44799922	0,7153176	TSKU	tsukushi, small leucine rich proteoglycan [Source:HGNC Symbol;Acc:HGNC:28850]
ENSG00000229344	0,13320679	3,52725332	0,62761352	0,44807114	0,7153176	MTCO2P12	MT-CO2 pseudogene 12 [Source:HGNC Symbol;Acc:HGNC:52028]

ENSG00000160551	0,21228754	3,76294926	0,62728735	0,44818537	0,71542178	TAOK1	TAO kinase 1 [Source:HGNC Symbol;Acc:HGNC:29259]
ENSG00000197635	0,12421143	6,33016466	0,62712012	0,44824396	0,71543712	DPP4	dipeptidyl peptidase 4 [Source:HGNC Symbol;Acc:HGNC:3009]
ENSG00000120322	-0,507017	-0,9173216	0,62674052	0,44837699	0,71557127	PCDHB8	protocadherin beta 8 [Source:HGNC Symbol;Acc:HGNC:8693]
ENSG00000025423	0,33096665	-0,3533507	0,62601297	0,44863214	0,71566102	HSD17B6	hydroxysteroid 17-beta dehydrogenase 6 [Source:HGNC Symbol;Acc:HGNC:23316]
ENSG00000134597	-0,1261298	4,36429792	0,62574223	0,44872715	0,71566102	RBMX2	RNA binding motif protein X-linked 2 [Source:HGNC Symbol;Acc:HGNC:24282]
ENSG00000143384	-0,1850416	7,32681151	0,62599971	0,4486368	0,71566102	MCL1	MCL1 apoptosis regulator, BCL2 family member [Source:HGNC Symbol;Acc:HGNC:6943]
ENSG00000144320	0,11849128	4,0424017	0,62581084	0,44870307	0,71566102	LNPK	lunapark, ER junction formation factor [Source:HGNC Symbol;Acc:HGNC:21610]
ENSG00000163795	-0,1939188	1,88587932	0,62625805	0,44854616	0,71566102	ZNF513	zinc finger protein 513 [Source:HGNC Symbol;Acc:HGNC:26498]
ENSG00000277459	-0,4554917	-1,2587841	0,62640817	0,44849351	0,71566102	AP001527.2	novel transcript
ENSG00000102760	-0,3677926	0,5943601	0,6252771	0,44889047	0,71576899	RGCC	regulator of cell cycle [Source:HGNC Symbol;Acc:HGNC:20369]
ENSG00000143412	-0,1350959	4,51831691	0,62527034	0,44889284	0,71576899	ANXA9	annexin A9 [Source:HGNC Symbol;Acc:HGNC:547]
ENSG00000116962	0,27615727	7,57664934	0,62439402	0,44920079	0,71579127	NID1	nidogen 1 [Source:HGNC Symbol;Acc:HGNC:7821]
ENSG00000130119	0,27143228	4,62101533	0,62451432	0,4491585	0,71579127	GNL3L	G protein nucleolar 3 like [Source:HGNC Symbol;Acc:HGNC:25553]
ENSG00000170266	0,14152842	7,1045305	0,62447492	0,44917235	0,71579127	GLB1	galactosidase beta 1 [Source:HGNC Symbol;Acc:HGNC:4298]
ENSG00000177463	0,17057454	2,95462705	0,62479989	0,44905812	0,71579127	NR2C2	nuclear receptor subfamily 2 group C member 2 [Source:HGNC Symbol;Acc:HGNC:7972]
ENSG00000225338	0,26914214	-0,1372497	0,62448661	0,44916824	0,71579127	RPL23AP18	ribosomal protein L23a pseudogene 18 [Source:HGNC Symbol;Acc:HGNC:36726]
ENSG00000246695	0,42038637	-0,7677274	0,62483353	0,4490463	0,71579127	RASSF8-AS1	RASSF8 antisense RNA 1 [Source:HGNC Symbol;Acc:HGNC:48637]
ENSG00000143622	-0,1229367	3,47887445	0,62407599	0,44931264	0,71581335	RIT1	Ras like without CAAX 1 [Source:HGNC Symbol;Acc:HGNC:10023]
ENSG00000183513	0,15369999	4,30437728	0,62418904	0,44927288	0,71581335	COA5	cytochrome c oxidase assembly factor 5 [Source:HGNC Symbol;Acc:HGNC:33848]
ENSG00000037637	0,12402616	4,18625084	0,62342738	0,44954091	0,71609891	FBXO42	F-box protein 42 [Source:HGNC Symbol;Acc:HGNC:29249]
ENSG00000225259	0,41401126	-0,964887	0,62311388	0,4496513	0,71611859	ST13P6	ST13, Hsp70 interacting protein pseudogene 6 [Source:HGNC Symbol;Acc:HGNC:18557]
ENSG00000282100	-0,2446759	0,35834596	0,62321638	0,4496152	0,71611859	HSP90AB4P	heat shock protein 90 alpha family class B member 4, pseudogene [Source:HGNC Symbol;Acc:HGNC:32538]
ENSG00000146282	-0,1075165	5,40520513	0,62255948	0,44984664	0,7163516	RARS2	arginyl-tRNA synthetase 2, mitochondrial [Source:HGNC Symbol;Acc:HGNC:21406]
ENSG00000130758	0,20567974	1,19434492	0,62234456	0,44992241	0,71639416	MAP3K10	mitogen-activated protein kinase kinase kinase 10 [Source:HGNC Symbol;Acc:HGNC:6849]
ENSG00000135324	0,27794067	-0,4342469	0,62205128	0,45002583	0,71648075	MRAP2	melanocortin 2 receptor accessory protein 2 [Source:HGNC Symbol;Acc:HGNC:21232]
ENSG00000132205	0,12911383	3,09997775	0,62117643	0,45033458	0,71673797	EMILIN2	elastin microfibril interfacer 2 [Source:HGNC Symbol;Acc:HGNC:19881]
ENSG00000135090	0,12734105	3,05170165	0,62126373	0,45030376	0,71673797	TAOK3	TAO kinase 3 [Source:HGNC Symbol;Acc:HGNC:18133]
ENSG00000235919	0,22106222	1,28820829	0,62139231	0,45025836	0,71673797	ASH1L-AS1	ASH1L antisense RNA 1 [Source:HGNC Symbol;Acc:HGNC:44146]
ENSG00000166126	-0,1755434	4,22149168	0,62103238	0,45038545	0,71674086	AMN	amnion associated transmembrane protein [Source:HGNC Symbol;Acc:HGNC:14604]
ENSG00000122386	-0,1215855	3,18478657	0,62021067	0,45067583	0,71712132	ZNF205	zinc finger protein 205 [Source:HGNC Symbol;Acc:HGNC:12996]
ENSG00000154928	0,38383385	-0,5079851	0,62007812	0,4507227	0,71712132	EPHB1	EPH receptor B1 [Source:HGNC Symbol;Acc:HGNC:3392]
ENSG00000271707	0,45081684	-1,8361133	0,61961084	0,450888	0,7173062	ATP1B3P1	ATPase Na+/K+ transporting subunit beta 3 pseudogene 1 [Source:HGNC Symbol;Acc:HGNC:807]
ENSG00000239470	-0,3389215	-1,2002827	0,61912387	0,45106037	0,71750228	AC011979.1	ribosomal protein L21 (RPL21) pseudogene
ENSG00000127540	-0,1298042	5,55380612	0,61863848	0,4512323	0,71769762	UQCR11	ubiquinol-cytochrome c reductase, complex III subunit XI [Source:HGNC Symbol;Acc:HGNC:30862]
ENSG00000231744	-0,3101274	-0,6726465	0,61825999	0,45136643	0,71783281	AL669818.1	RAN binding protein 1 (RANBP1) pseudogene
ENSG00000153976	0,22219842	0,47711502	0,61798031	0,45146559	0,71783423	HS3ST3A1	heparan sulfate-glucosamine 3-sulfotransferase 3A1 [Source:HGNC Symbol;Acc:HGNC:5196]
ENSG00000204219	0,1035024	6,86389931	0,61809955	0,45142331	0,71783423	TCEA3	transcription elongation factor A3 [Source:HGNC Symbol;Acc:HGNC:11615]
ENSG00000213695	-0,2690936	-0,4634871	0,61756169	0,45161409	0,71799219	RPS7P14	ribosomal protein S7 pseudogene 14 [Source:HGNC Symbol;Acc:HGNC:36602]
ENSG00000100023	-0,1738607	5,51186857	0,61702398	0,45180494	0,7181172	PPIL2	peptidylprolyl isomerase like 2 [Source:HGNC Symbol;Acc:HGNC:9261]
ENSG00000144118	-0,1116775	5,00981044	0,61692474	0,45184018	0,7181172	RALB	RAS like proto-oncogene B [Source:HGNC Symbol;Acc:HGNC:9840]
ENSG00000172613	-0,1221144	4,44585799	0,61694346	0,45183354	0,7181172	RAD9A	RAD9 checkpoint clamp component A [Source:HGNC Symbol;Acc:HGNC:9827]
ENSG00000162735	0,15053421	5,10164041	0,61610986	0,45212972	0,71817572	PEX19	peroxisomal biogenesis factor 19 [Source:HGNC Symbol;Acc:HGNC:9713]
ENSG00000169727	-0,1168467	7,68364363	0,61599104	0,45217197	0,71817572	GPS1	G protein pathway suppressor 1 [Source:HGNC Symbol;Acc:HGNC:4549]
ENSG00000215349	0,46361565	-1,6890795	0,61627713	0,45207026	0,71817572	MRPL3P1	mitochondrial ribosomal protein L3 pseudogene 1 [Source:HGNC Symbol;Acc:HGNC:29701]

ENSG00000225973	-0,1327974	3,02889113	0,61614897	0,45211582	0,71817572	PIGBOS1	PIGB opposite strand 1 [Source:HGNC Symbol;Acc:HGNC:50696]
ENSG00000226200	0,4414977	-1,1424046	0,61611302	0,4521286	0,71817572	SGMS1-AS1	SGMS1 antisense RNA 1 [Source:HGNC Symbol;Acc:HGNC:49683]
ENSG00000230067	0,28866973	0,87514963	0,61664552	0,45193936	0,71817572	HSPD1P6	heat shock protein family D (Hsp60) member 1 pseudogene 6 [Source:HGNC Symbol;Acc:HGNC:5267]
ENSG00000162458	-0,1445162	4,77264201	0,61582522	0,45223093	0,71819129	FBLIM1	filamin binding LIM protein 1 [Source:HGNC Symbol;Acc:HGNC:24686]
ENSG00000117877	-0,1704542	4,45105882	0,61544681	0,45236554	0,71832698	POLR1G	RNA polymerase I subunit G [Source:HGNC Symbol;Acc:HGNC:24219]
ENSG00000279392	0,27017847	-0,3911802	0,61509144	0,45249202	0,71844972	AL158801.4	ubiquitin-conjugating enzyme E2L 3 (UBE2L3) pseudogene
ENSG00000225131	0,12354047	3,71683807	0,61466565	0,45264364	0,71861236	PSME2P2	proteasome activator subunit 2 pseudogene 2 [Source:HGNC Symbol;Acc:HGNC:30160]
ENSG00000168005	0,12280117	5,74942831	0,61442649	0,45272884	0,71866952	SPINDOC	spindlin interactor and repressor of chromatin binding [Source:HGNC Symbol;Acc:HGNC:25115]
ENSG00000159216	-0,1420572	2,80489303	0,61426841	0,45278517	0,71868085	RUNX1	RUNX family transcription factor 1 [Source:HGNC Symbol;Acc:HGNC:10471]
ENSG00000231925	0,12439922	6,12372957	0,61376932	0,45296309	0,71888515	TAPBP	TAP binding protein [Source:HGNC Symbol;Acc:HGNC:11566]
ENSG00000271268	-0,4422572	-1,6445675	0,6133458	0,45311417	0,71904681	AC015849.6	ribosomal L24 domain containing 1 (RSL24D1) pseudogene
ENSG00000145860	0,16393038	4,66246383	0,61252625	0,45340676	0,71943297	RNF145	ring finger protein 145 [Source:HGNC Symbol;Acc:HGNC:20853]
ENSG00000237991	-0,1940418	0,87275861	0,61207698	0,45356729	0,71960953	RPL35P1	ribosomal protein L35 pseudogene 1 [Source:HGNC Symbol;Acc:HGNC:36695]
ENSG00000077235	0,22149466	7,56193009	0,61178581	0,45367138	0,71969651	GTF3C1	general transcription factor IIIC subunit 1 [Source:HGNC Symbol;Acc:HGNC:4664]
ENSG00000176422	0,11246599	4,68038876	0,61163178	0,45372646	0,71970574	SPRYD4	SPRY domain containing 4 [Source:HGNC Symbol;Acc:HGNC:27468]
ENSG00000074356	0,17952243	4,70914435	0,61122532	0,45387186	0,71977443	NCBP3	nuclear cap binding subunit 3 [Source:HGNC Symbol;Acc:HGNC:24612]
ENSG00000101132	0,11712311	5,87565579	0,61099216	0,45395531	0,71977443	PFDN4	prefoldin subunit 4 [Source:HGNC Symbol;Acc:HGNC:8868]
ENSG00000130772	-0,1138515	4,12698457	0,61080679	0,45402167	0,71977443	MED18	mediator complex subunit 18 [Source:HGNC Symbol;Acc:HGNC:25944]
ENSG00000165288	0,19672291	1,49771798	0,61068471	0,45406538	0,71977443	BRWD3	bromodomain and WD repeat domain containing 3 [Source:HGNC Symbol;Acc:HGNC:17342]
ENSG00000213468	-0,2230531	0,74305213	0,61112588	0,45390745	0,71977443	FIRRE	firre intergenic repeating RNA element [Source:HGNC Symbol;Acc:HGNC:49627]
ENSG00000215769	-0,3542249	-1,3731291	0,61070534	0,45405799	0,71977443	ARHGAP27P1-BPTFP1	ARHGAP27P1-BPTFP1-KPNA2P3 readthrough, transcribed pseudogene [Source:HGNC Symbol;Acc:HGNC:52873]
ENSG00000142046	-0,1855911	0,82742515	0,61048137	0,4541382	0,71981176	TMEM91	transmembrane protein 91 [Source:HGNC Symbol;Acc:HGNC:32393]
ENSG00000052749	-0,2629373	5,73152317	0,61023972	0,45422477	0,71987088	RRP12	ribosomal RNA processing 12 homolog [Source:HGNC Symbol;Acc:HGNC:29100]
ENSG00000158079	0,17955531	2,03214259	0,60856023	0,45482721	0,72074745	PTPDC1	protein tyrosine phosphatase domain containing 1 [Source:HGNC Symbol;Acc:HGNC:30184]
ENSG00000167528	-0,1900276	1,60871477	0,6082637	0,45493372	0,72083804	ZNF641	zinc finger protein 641 [Source:HGNC Symbol;Acc:HGNC:31834]
ENSG00000136868	0,12222992	6,64176544	0,60776227	0,45511392	0,72097337	SLC31A1	solute carrier family 31 member 1 [Source:HGNC Symbol;Acc:HGNC:11016]
ENSG00000215007	-0,2761669	-0,6480626	0,60775139	0,45511783	0,72097337	DNAJA1P3	DnaJ heat shock protein family (Hsp40) member A1 pseudogene 3 [Source:HGNC Symbol;Acc:HGNC:39339]
ENSG00000167645	-0,104283	6,43903314	0,6073602	0,4552585	0,72111802	YIF1B	Yip1 interacting factor homolog B, membrane trafficking protein [Source:HGNC Symbol;Acc:HGNC:30511]
ENSG00000100416	0,10298425	5,68853811	0,60683067	0,45544904	0,72131635	TRMU	tRNA 5-methylaminomethyl-2-thiouridylate methyltransferase [Source:HGNC Symbol;Acc:HGNC:25481]
ENSG00000165525	-0,240646	4,59144342	0,60662259	0,45552394	0,72131635	NEMF	nuclear export mediator factor [Source:HGNC Symbol;Acc:HGNC:10663]
ENSG00000198692	-0,1339752	5,15969515	0,60660067	0,45553184	0,72131635	EIF1AY	eukaryotic translation initiation factor 1A Y-linked [Source:HGNC Symbol;Acc:HGNC:3252]
ENSG00000227953	0,37077349	-0,3854982	0,60631888	0,45563331	0,72139884	LINC01341	long intergenic non-protein coding RNA 1341 [Source:HGNC Symbol;Acc:HGNC:49457]
ENSG00000070610	-0,1134455	5,71818973	0,60613985	0,45569781	0,72142276	GBA2	glucosylceramidase beta 2 [Source:HGNC Symbol;Acc:HGNC:18986]
ENSG00000077549	0,1087508	8,09625615	0,60557463	0,45590152	0,72151354	CAPZB	capping actin protein of muscle Z-line subunit beta [Source:HGNC Symbol;Acc:HGNC:1491]
ENSG00000100147	0,15975943	5,14970218	0,60568232	0,4558627	0,72151354	CCDC134	coiled-coil domain containing 134 [Source:HGNC Symbol;Acc:HGNC:26185]
ENSG00000106009	-0,1145126	4,29836353	0,60556967	0,45590331	0,72151354	BRAT1	BRCA1 associated ATM activator 1 [Source:HGNC Symbol;Acc:HGNC:21701]
ENSG00000102547	0,271178	1,33965948	0,60479052	0,45618439	0,7218584	CAB39L	calcium binding protein 39 like [Source:HGNC Symbol;Acc:HGNC:20290]
ENSG00000152256	-0,1361419	4,10837417	0,60469173	0,45622004	0,7218584	PDK1	pyruvate dehydrogenase kinase 1 [Source:HGNC Symbol;Acc:HGNC:8809]
ENSG00000073605	0,39085766	-1,4894772	0,60340228	0,45668592	0,72221469	GSDMB	gasdermin B [Source:HGNC Symbol;Acc:HGNC:23690]
ENSG00000108375	0,10904878	5,33747587	0,60324765	0,45674184	0,72221469	RNF43	ring finger protein 43 [Source:HGNC Symbol;Acc:HGNC:18505]
ENSG00000135775	-0,1116299	5,09972604	0,60330709	0,45672034	0,72221469	COG2	component of oligomeric golgi complex 2 [Source:HGNC Symbol;Acc:HGNC:6546]
ENSG00000159921	0,14470009	4,44689333	0,60360234	0,45661358	0,72221469	GNE	glucosamine (UDP-N-acetyl)-2-epimerase/N-acetylmannosamine kinase [Source:HGNC Symbol;Acc:HGNC:23657]
ENSG00000170315	0,10216791	10,4918998	0,60366714	0,45659016	0,72221469	UBB	ubiquitin B [Source:HGNC Symbol;Acc:HGNC:12463]
ENSG00000226221	-0,1604985	2,01176055	0,60378648	0,45654702	0,72221469	RPL26P19	ribosomal protein L26 pseudogene 19 [Source:HGNC Symbol;Acc:HGNC:36393]

ENSG00000104221	-0,1289702	4,64646955	0,60298486	0,4568369	0,72228683	BRF2	BRF2 RNA polymerase III transcription initiation factor subunit [Source:HGNC Symbol;Acc:HGNC:17298]
ENSG00000105402	0,10169439	5,65356729	0,60202978	0,45718268	0,72244298	NAPA	NSF attachment protein alpha [Source:HGNC Symbol;Acc:HGNC:7641]
ENSG00000158246	0,22677438	1,38501674	0,60250172	0,45701176	0,72244298	TENT5B	terminal nucleotidyltransferase 5B [Source:HGNC Symbol;Acc:HGNC:28273]
ENSG00000164144	-0,1279361	3,77575674	0,60214434	0,45714118	0,72244298	ARFIP1	ADP ribosylation factor interacting protein 1 [Source:HGNC Symbol;Acc:HGNC:21496]
ENSG00000175489	-0,3069173	-0,7702144	0,60202912	0,45718292	0,72244298	LRRC25	leucine rich repeat containing 25 [Source:HGNC Symbol;Acc:HGNC:29806]
ENSG00000279026	0,66862907	-2,2800868	0,60240015	0,45704854	0,72244298	AC005225.4	TEC
ENSG00000234456	-0,1229581	3,05987018	0,60152995	0,45736382	0,72265067	MAGI2-AS3	MAGI2 antisense RNA 3 [Source:HGNC Symbol;Acc:HGNC:40862]
ENSG00000213621	0,21509759	0,23893357	0,60127062	0,45745785	0,72272108	RPSAP54	ribosomal protein SA pseudogene 54 [Source:HGNC Symbol;Acc:HGNC:36373]
ENSG00000114739	0,14332375	3,39750418	0,60085545	0,45760846	0,72286541	ACVR2B	activin A receptor type 2B [Source:HGNC Symbol;Acc:HGNC:174]
ENSG00000169957	0,12915846	5,13194407	0,60071057	0,45766103	0,72286541	ZNF768	zinc finger protein 768 [Source:HGNC Symbol;Acc:HGNC:26273]
ENSG00000171867	0,15700832	8,38668719	0,60047336	0,45774714	0,72286541	PRNP	prion protein [Source:HGNC Symbol;Acc:HGNC:9449]
ENSG00000285756	-0,1793297	2,34203173	0,60059132	0,45770432	0,72286541	BX890604.1	novel transcript
ENSG00000151502	-0,104209	5,26001644	0,6001544	0,45786296	0,72297017	VPS26B	VPS26, retromer complex component B [Source:HGNC Symbol;Acc:HGNC:28119]
ENSG00000151640	-0,2092204	0,56351347	0,5995046	0,45809907	0,72326481	DPYSL4	dihydropyrimidinase like 4 [Source:HGNC Symbol;Acc:HGNC:3016]
ENSG00000197180	-0,3388939	0,12966915	0,5993588	0,45815208	0,72327034	CH17-340M24.3	uncharacterized protein BC009467 [Source:NCBI gene (formerly Entrezgene);Acc:158960]
ENSG00000135862	0,19964581	7,84756919	0,59901054	0,45827873	0,72331395	LAMC1	laminin subunit gamma 1 [Source:HGNC Symbol;Acc:HGNC:6492]
ENSG00000163121	0,37337969	-1,1960319	0,59907299	0,45825601	0,72331395	NEURL3	neuralized E3 ubiquitin protein ligase 3 [Source:HGNC Symbol;Acc:HGNC:25162]
ENSG00000067191	-0,3147738	0,16813587	0,59808835	0,4586144	0,72367168	CACNB1	calcium voltage-gated channel auxiliary subunit beta 1 [Source:HGNC Symbol;Acc:HGNC:1401]
ENSG00000102189	-0,3474785	3,65935316	0,59762028	0,45878493	0,72367168	EEA1	early endosome antigen 1 [Source:HGNC Symbol;Acc:HGNC:3185]
ENSG00000113272	0,11880506	4,26539047	0,59798814	0,4586509	0,72367168	THG1L	tRNA-histidine guanylyltransferase 1 like [Source:HGNC Symbol;Acc:HGNC:26053]
ENSG00000119927	0,11570367	4,9217967	0,59757181	0,4588026	0,72367168	GPAM	glycerol-3-phosphate acyltransferase, mitochondrial [Source:HGNC Symbol;Acc:HGNC:24865]
ENSG00000134905	-0,10062	6,09071906	0,59775387	0,45873625	0,72367168	CARS2	cysteinyl-tRNA synthetase 2, mitochondrial [Source:HGNC Symbol;Acc:HGNC:25695]
ENSG00000227354	-0,3250097	-1,1285731	0,59769597	0,45875735	0,72367168	RBM26-AS1	RBM26 antisense RNA 1 [Source:HGNC Symbol;Acc:HGNC:39805]
ENSG00000110811	-0,1753464	6,43254176	0,59739743	0,45886616	0,72369356	P3H3	prolyl 3-hydroxylase 3 [Source:HGNC Symbol;Acc:HGNC:19318]
ENSG00000128641	-0,1149898	6,07682969	0,59726199	0,45891554	0,72369356	MYO1B	myosin IB [Source:HGNC Symbol;Acc:HGNC:7596]
ENSG00000258017	0,24947366	-0,0554504	0,59685143	0,45906529	0,72385157	AC011603.2	novel transcript, antisense to TUBA1B
ENSG00000100104	-0,1183355	5,05652789	0,59647587	0,45920233	0,72398952	SRRD	SRR1 domain containing [Source:HGNC Symbol;Acc:HGNC:33910]
ENSG00000063587	0,14815116	2,36850967	0,59476038	0,45982924	0,72401877	ZNF275	zinc finger protein 275 [Source:HGNC Symbol;Acc:HGNC:13069]
ENSG00000088247	0,18014402	7,06560116	0,5947394	0,45983692	0,72401877	KHSRP	KH-type splicing regulatory protein [Source:HGNC Symbol;Acc:HGNC:6316]
ENSG00000088451	0,11586405	3,60087382	0,59480842	0,45981167	0,72401877	TGDS	TDP-glucose 4,6-dehydratase [Source:HGNC Symbol;Acc:HGNC:20324]
ENSG00000121022	0,11706927	5,93309575	0,59615018	0,45932124	0,72401877	COPS5	COP9 signalosome subunit 5 [Source:HGNC Symbol;Acc:HGNC:2240]
ENSG00000125676	-0,2321655	4,88018215	0,59565801	0,45950103	0,72401877	THOC2	THO complex 2 [Source:HGNC Symbol;Acc:HGNC:19073]
ENSG00000139835	0,10849996	4,85357361	0,59514302	0,45968929	0,72401877	GRTP1	growth hormone regulated TBC protein 1 [Source:HGNC Symbol;Acc:HGNC:20310]
ENSG00000143819	0,10966027	7,32883748	0,59614606	0,45932275	0,72401877	EPHX1	epoxide hydrolase 1 [Source:HGNC Symbol;Acc:HGNC:3401]
ENSG00000149499	-0,1167072	3,81902006	0,59469153	0,45985444	0,72401877	EML3	EMAP like 3 [Source:HGNC Symbol;Acc:HGNC:26666]
ENSG00000158373	0,20816772	0,92053236	0,59465161	0,45986904	0,72401877	H2BC5	H2B clustered histone 5 [Source:HGNC Symbol;Acc:HGNC:4747]
ENSG00000167363	-0,1057686	6,25726117	0,5958759	0,45942142	0,72401877	FN3K	fructosamine 3 kinase [Source:HGNC Symbol;Acc:HGNC:24822]
ENSG00000168096	-0,127416	2,73439342	0,59523872	0,45965429	0,72401877	ANKS3	ankyrin repeat and sterile alpha motif domain containing 3 [Source:HGNC Symbol;Acc:HGNC:29422]
ENSG00000175054	-0,1337533	2,97828593	0,5945268	0,45991472	0,72401877	ATR	ATR serine/threonine kinase [Source:HGNC Symbol;Acc:HGNC:882]
ENSG00000198324	0,12716038	4,79954672	0,59535505	0,45961176	0,72401877	PHETA1	PH domain containing endocytic trafficking adaptor 1 [Source:HGNC Symbol;Acc:HGNC:26509]
ENSG00000233514	-0,2927691	0,00101147	0,59460967	0,45988439	0,72401877	AL356653.1	small nuclear ribonucleoprotein D1 polypeptide 16kDa (SNRPD1) pseudogene
ENSG00000197903	0,38657654	-0,1581434	0,59402443	0,46009864	0,72423026	H2BC12	H2B clustered histone 12 [Source:HGNC Symbol;Acc:HGNC:13954]
ENSG00000099377	-0,1010309	6,60194208	0,59378866	0,460185	0,72424245	HSD3B7	hydroxy-delta-5-steroid dehydrogenase, 3 beta- and steroid delta-isomerase 7 [Source:HGNC Symbol;Acc:HGNC:1
ENSG00000135245	0,13376632	2,78318675	0,59373262	0,46020553	0,72424245	HILPDA	hypoxia inducible lipid droplet associated [Source:HGNC Symbol;Acc:HGNC:28859]

ENSG00000261512	-0,2880242	0,06314844	0,59346333	0,46030421	0,72431972	AC092368.3	novel transcript
ENSG00000119705	-0,1132505	7,35427427	0,59311915	0,46043039	0,72433727	SLIRP	SRA stem-loop interacting RNA binding protein [Source:HGNC Symbol;Acc:HGNC:20495]
ENSG00000128534	0,12598076	5,35166498	0,59313389	0,46042498	0,72433727	LSM8	LSM8 homolog, U6 small nuclear RNA associated [Source:HGNC Symbol;Acc:HGNC:20471]
ENSG00000215712	0,14441044	4,77759095	0,59302719	0,46046411	0,72433727	TMEM242	transmembrane protein 242 [Source:HGNC Symbol;Acc:HGNC:17206]
ENSG00000108100	0,1244832	5,10960788	0,59258577	0,46062605	0,72439028	CCNY	cyclin Y [Source:HGNC Symbol;Acc:HGNC:23354]
ENSG00000170522	-0,1518686	4,71571757	0,59243344	0,46068195	0,72439028	ELOVL6	ELOVL fatty acid elongase 6 [Source:HGNC Symbol;Acc:HGNC:15829]
ENSG00000178694	-0,1321654	2,47953534	0,59239474	0,46069615	0,72439028	NSUN3	NOP2/Sun RNA methyltransferase 3 [Source:HGNC Symbol;Acc:HGNC:26208]
ENSG00000198783	0,11672201	4,5476688	0,59266758	0,46059603	0,72439028	ZNF830	zinc finger protein 830 [Source:HGNC Symbol;Acc:HGNC:28291]
ENSG00000111110	0,16157776	4,27349425	0,59168375	0,46095725	0,72456687	PPM1H	protein phosphatase, Mg2+/Mn2+ dependent 1H [Source:HGNC Symbol;Acc:HGNC:18583]
ENSG00000180921	-0,1952515	4,10002495	0,59181445	0,46090924	0,72456687	FAM83H	family with sequence similarity 83 member H [Source:HGNC Symbol;Acc:HGNC:24797]
ENSG00000287261	-0,3495321	-0,938396	0,59171211	0,46094683	0,72456687	AL139243.1	novel transcript, antisense to HPS1
ENSG00000236675	-0,2224334	0,52709967	0,59129463	0,46110025	0,72471368	MTX1P1	metaxin 1 pseudogene 1 [Source:HGNC Symbol;Acc:HGNC:7505]
ENSG00000108509	0,13437007	3,7699531	0,59085757	0,46126097	0,72488828	CAMTA2	calmodulin binding transcription activator 2 [Source:HGNC Symbol;Acc:HGNC:18807]
ENSG00000110713	0,17834514	6,43197169	0,59040007	0,4614293	0,7248893	NUP98	nucleoporin 98 and 96 precursor [Source:HGNC Symbol;Acc:HGNC:8068]
ENSG00000153914	0,13699268	4,58278455	0,59031641	0,46146009	0,7248893	SREK1	splicing regulatory glutamic acid and lysine rich protein 1 [Source:HGNC Symbol;Acc:HGNC:17882]
ENSG00000172465	0,13857603	3,05221819	0,59033553	0,46145305	0,7248893	TCEAL1	transcription elongation factor A like 1 [Source:HGNC Symbol;Acc:HGNC:11616]
ENSG00000204435	-0,1303993	6,04053248	0,59066469	0,46133192	0,7248893	CSNK2B	casein kinase 2 beta [Source:HGNC Symbol;Acc:HGNC:2460]
ENSG00000120053	-0,1633541	8,30601202	0,59013767	0,46152589	0,72491471	GOT1	glutamic-oxaloacetic transaminase 1 [Source:HGNC Symbol;Acc:HGNC:4432]
ENSG00000015532	-0,1568125	4,97893037	0,58967025	0,46169804	0,72510715	XYLT2	xylosyltransferase 2 [Source:HGNC Symbol;Acc:HGNC:15517]
ENSG00000082996	0,11950621	5,16157272	0,58945975	0,46177561	0,72515101	RNF13	ring finger protein 13 [Source:HGNC Symbol;Acc:HGNC:10057]
ENSG00000184857	0,10252498	5,75654674	0,58904258	0,46192939	0,72531454	TMEM186	transmembrane protein 186 [Source:HGNC Symbol;Acc:HGNC:24530]
ENSG00000116337	-0,1184078	5,6281191	0,58828202	0,46220998	0,72567712	AMPD2	adenosine monophosphate deaminase 2 [Source:HGNC Symbol;Acc:HGNC:469]
ENSG00000003756	-0,1067037	5,73320854	0,58781178	0,46238361	0,72587171	RBM5	RNA binding motif protein 5 [Source:HGNC Symbol;Acc:HGNC:9902]
ENSG00000173436	-0,1692569	2,14050086	0,58673504	0,46278161	0,72641845	MICOS10	mitochondrial contact site and cristae organizing system subunit 10 [Source:HGNC Symbol;Acc:HGNC:32068]
ENSG00000116030	0,13748344	6,82455456	0,58637407	0,46291517	0,72644758	SUMO1	small ubiquitin like modifier 1 [Source:HGNC Symbol;Acc:HGNC:12502]
ENSG00000143252	-0,1060192	7,12369405	0,58654725	0,46285108	0,72644758	SDHC	succinate dehydrogenase complex subunit C [Source:HGNC Symbol;Acc:HGNC:10682]
ENSG00000262953	-0,2929685	-0,2787546	0,58628172	0,46294934	0,72644758	EIF4A1P9	eukaryotic translation initiation factor 4A1 pseudogene 9 [Source:HGNC Symbol;Acc:HGNC:37928]
ENSG00000109171	-0,1517195	3,02347296	0,58564817	0,46318395	0,72665247	SLAIN2	SLAIN motif family member 2 [Source:HGNC Symbol;Acc:HGNC:29282]
ENSG00000167088	-0,1223812	7,40260691	0,58552619	0,46322914	0,72665247	SNRPD1	small nuclear ribonucleoprotein D1 polypeptide [Source:HGNC Symbol;Acc:HGNC:11158]
ENSG00000204138	0,23331917	4,45638449	0,58554026	0,46322393	0,72665247	PHACTR4	phosphatase and actin regulator 4 [Source:HGNC Symbol;Acc:HGNC:25793]
ENSG00000166402	0,274237	0,08563266	0,58504838	0,46340624	0,72684455	TUB	TUB bipartite transcription factor [Source:HGNC Symbol;Acc:HGNC:12406]
ENSG00000264575	-0,1606589	3,09658366	0,5849274	0,46345109	0,72684455	LINC00526	long intergenic non-protein coding RNA 526 [Source:HGNC Symbol;Acc:HGNC:28278]
ENSG00000135049	-0,1476883	3,29957843	0,58467054	0,46354636	0,72691593	AGTPBP1	ATP/GTP binding protein 1 [Source:HGNC Symbol;Acc:HGNC:17258]
ENSG00000152818	0,29423106	4,95590022	0,5840845	0,46376384	0,72717892	UTRN	utrophin [Source:HGNC Symbol;Acc:HGNC:12635]
ENSG00000117525	-0,1320196	4,17406646	0,58381953	0,46386223	0,72725514	F3	coagulation factor III, tissue factor [Source:HGNC Symbol;Acc:HGNC:3541]
ENSG00000060237	-0,2717962	4,94029484	0,58348354	0,46398704	0,72737276	WNK1	WNK lysine deficient protein kinase 1 [Source:HGNC Symbol;Acc:HGNC:14540]
ENSG00000085788	0,1115864	4,49178932	0,58316089	0,46410696	0,72742079	DDHD2	DDHD domain containing 2 [Source:HGNC Symbol;Acc:HGNC:29106]
ENSG00000160310	-0,1700408	4,6860468	0,58313314	0,46411727	0,72742079	PRMT2	protein arginine methyltransferase 2 [Source:HGNC Symbol;Acc:HGNC:5186]
ENSG00000111775	0,10454402	7,03768831	0,58217605	0,4644733	0,72751403	COX6A1	cytochrome c oxidase subunit 6A1 [Source:HGNC Symbol;Acc:HGNC:2277]
ENSG00000115355	-0,1397707	4,12750329	0,58280575	0,46423901	0,72751403	CCDC88A	coiled-coil domain containing 88A [Source:HGNC Symbol;Acc:HGNC:25523]
ENSG00000122705	-0,1032274	7,91232455	0,58191425	0,46457077	0,72751403	CLTA	clathrin light chain A [Source:HGNC Symbol;Acc:HGNC:2090]
ENSG00000130811	-0,1030647	7,78888189	0,58181553	0,46460753	0,72751403	EIF3G	eukaryotic translation initiation factor 3 subunit G [Source:HGNC Symbol;Acc:HGNC:3274]
ENSG00000135631	0,11838124	4,65996899	0,58183082	0,46460184	0,72751403	RAB11FIP5	RAB11 family interacting protein 5 [Source:HGNC Symbol;Acc:HGNC:24845]
ENSG00000143164	0,13648331	5,17400394	0,58242415	0,46438096	0,72751403	DCAF6	DDB1 and CUL4 associated factor 6 [Source:HGNC Symbol;Acc:HGNC:30002]

ENSG00000153933	0,14989181	2,51796249	0,58189398	0,46457832	0,72751403	DGKE	diacylglycerol kinase epsilon [Source:HGNC Symbol;Acc:HGNC:2852]
ENSG00000229419	0,31072539	-0,6137249	0,58223713	0,46445056	0,72751403	RALGAPA1P1	RALGAPA1 pseudogene 1 [Source:HGNC Symbol;Acc:HGNC:23203]
ENSG00000285417	-0,2494361	0,43583609	0,58176876	0,46462495	0,72751403	BX571818.1	novel pseudogene
ENSG00000197937	0,3632112	0,44348219	0,58151437	0,46471971	0,72758442	ZNF347	zinc finger protein 347 [Source:HGNC Symbol;Acc:HGNC:16447]
ENSG00000124222	0,1197756	4,51371171	0,58108557	0,46487952	0,72775663	STX16	syntaxin 16 [Source:HGNC Symbol;Acc:HGNC:11431]
ENSG00000204673	-0,1103713	5,46536541	0,58039196	0,46513822	0,7280836	AKT1S1	AKT1 substrate 1 [Source:HGNC Symbol;Acc:HGNC:28426]
ENSG00000101346	0,14208045	7,36411606	0,58010037	0,46524705	0,72817593	POFUT1	protein O-fucosyltransferase 1 [Source:HGNC Symbol;Acc:HGNC:14988]
ENSG00000220323	0,24636026	0,42339519	0,57985756	0,4653377	0,72823979	H2BC19P	H2B clustered histone 19, pseudogene [Source:HGNC Symbol;Acc:HGNC:20517]
ENSG00000270504	-0,1826748	1,66818472	0,57936835	0,46552045	0,72844775	AL391422.4	novel transcript, antisense to PXDC1
ENSG00000183530	0,21160149	3,33062246	0,57880133	0,46573242	0,72870139	PRR14L	proline rich 14 like [Source:HGNC Symbol;Acc:HGNC:28738]
ENSG00000080345	0,13949907	4,6775386	0,57843394	0,46586985	0,72883836	RIF1	replication timing regulatory factor 1 [Source:HGNC Symbol;Acc:HGNC:23207]
ENSG00000106034	-0,1200021	4,82497722	0,57811829	0,46598798	0,72889342	CPED1	cadherin like and PC-esterase domain containing 1 [Source:HGNC Symbol;Acc:HGNC:26159]
ENSG00000162385	0,1032273	6,39425745	0,57807328	0,46600483	0,72889342	MAGOH	mago homolog, exon junction complex subunit [Source:HGNC Symbol;Acc:HGNC:6815]
ENSG00000155229	-0,1284691	6,23496272	0,57756243	0,46619613	0,72911458	MMS19	MMS19 homolog, cytosolic iron-sulfur assembly component [Source:HGNC Symbol;Acc:HGNC:13824]
ENSG00000137996	0,11461218	5,65182717	0,57726074	0,46630917	0,72921331	RTCA	RNA 3'-terminal phosphate cyclase [Source:HGNC Symbol;Acc:HGNC:17981]
ENSG00000055332	0,1152151	4,82878608	0,57692651	0,46643446	0,72933116	EIF2AK2	eukaryotic translation initiation factor 2 alpha kinase 2 [Source:HGNC Symbol;Acc:HGNC:9437]
ENSG00000178764	0,11164047	4,85766314	0,57678765	0,46648653	0,72933452	ZHX2	zinc fingers and homeoboxes 2 [Source:HGNC Symbol;Acc:HGNC:18513]
ENSG00000152193	0,11832426	3,7317218	0,57650113	0,466594	0,72940967	OBI1	ORC ubiquitin ligase 1 [Source:HGNC Symbol;Acc:HGNC:20308]
ENSG00000184840	-0,1185003	9,51559456	0,57633212	0,46665742	0,72940967	TMED9	transmembrane p24 trafficking protein 9 [Source:HGNC Symbol;Acc:HGNC:24878]
ENSG00000259642	-0,2383386	1,31320099	0,57626027	0,46668439	0,72940967	ST20-AS1	ST20 antisense RNA 1 [Source:HGNC Symbol;Acc:HGNC:27521]
ENSG00000005339	0,39214076	4,49843324	0,57599432	0,46678421	0,72944848	CREBBP	CREB binding protein [Source:HGNC Symbol;Acc:HGNC:2348]
ENSG00000108854	0,12556489	3,83902968	0,57581961	0,46684981	0,72944848	SMURF2	SMAD specific E3 ubiquitin protein ligase 2 [Source:HGNC Symbol;Acc:HGNC:16809]
ENSG00000213853	0,12321212	5,1880256	0,57579513	0,46685901	0,72944848	EMP2	epithelial membrane protein 2 [Source:HGNC Symbol;Acc:HGNC:3334]
ENSG00000163682	-0,1094034	10,2624787	0,57550867	0,4669666	0,72946056	RPL9	ribosomal protein L9 [Source:HGNC Symbol;Acc:HGNC:10369]
ENSG00000214244	-0,3917193	-1,3330982	0,57560943	0,46692875	0,72946056	SETP21	SET pseudogene 21 [Source:HGNC Symbol;Acc:HGNC:42940]
ENSG00000125246	-0,1324769	3,6811184	0,575224	0,46707357	0,72954964	CLYBL	citramalyl-CoA lyase [Source:HGNC Symbol;Acc:HGNC:18355]
ENSG00000226948	-0,2602597	-0,4854456	0,57500836	0,46715463	0,72959825	RPS4XP2	ribosomal protein S4X pseudogene 2 [Source:HGNC Symbol;Acc:HGNC:16563]
ENSG00000126756	-0,1174943	5,69259605	0,57460996	0,46730445	0,72975422	UXT	ubiquitously expressed prefoldin like chaperone [Source:HGNC Symbol;Acc:HGNC:12641]
ENSG00000232650	0,32788395	-0,7654493	0,57435375	0,46740084	0,72982673	LINC01780	long intergenic non-protein coding RNA 1780 [Source:HGNC Symbol;Acc:HGNC:52570]
ENSG00000179119	0,16913238	4,18940321	0,5740145	0,46752853	0,72987009	SPTY2D1	SPT2 chromatin protein domain containing 1 [Source:HGNC Symbol;Acc:HGNC:26818]
ENSG00000228078	0,36253852	-1,1658897	0,57410554	0,46749426	0,72987009	HLA-U	major histocompatibility complex, class I, U (pseudogene) [Source:HGNC Symbol;Acc:HGNC:23477]
ENSG00000006377	0,14616715	1,96782899	0,57370195	0,46764622	0,72987583	DLX6	distal-less homeobox 6 [Source:HGNC Symbol;Acc:HGNC:2919]
ENSG00000113384	0,18850059	5,6994696	0,57370216	0,46764614	0,72987583	GOLPH3	golgi phosphoprotein 3 [Source:HGNC Symbol;Acc:HGNC:15452]
ENSG00000177030	0,11118718	4,59670255	0,57360672	0,46768209	0,72987583	DEAF1	DEAF1 transcription factor [Source:HGNC Symbol;Acc:HGNC:14677]
ENSG00000146386	-0,2159717	0,62468403	0,57338527	0,46776552	0,72992806	ABRACL	ABRA C-terminal like [Source:HGNC Symbol;Acc:HGNC:21230]
ENSG00000085563	-0,1719038	5,61484066	0,57309528	0,46787481	0,72997072	ABCB1	ATP binding cassette subfamily B member 1 [Source:HGNC Symbol;Acc:HGNC:40]
ENSG00000170296	-0,1341281	3,80088169	0,57293863	0,46793387	0,72997072	GABARAP	GABA type A receptor-associated protein [Source:HGNC Symbol;Acc:HGNC:4067]
ENSG00000198836	0,11395686	5,30591002	0,57283076	0,46797454	0,72997072	OPA1	OPA1 mitochondrial dynamin like GTPase [Source:HGNC Symbol;Acc:HGNC:8140]
ENSG00000278558	-0,3054076	-0,4272333	0,57278253	0,46799273	0,72997072	TMEM191B	transmembrane protein 191B [Source:HGNC Symbol;Acc:HGNC:33600]
ENSG00000142632	0,16533307	1,69762945	0,57244673	0,46811939	0,73009034	ARHGEF19	Rho guanine nucleotide exchange factor 19 [Source:HGNC Symbol;Acc:HGNC:26604]
ENSG00000127870	0,13641825	4,60561405	0,57219412	0,46821472	0,73016106	RNF6	ring finger protein 6 [Source:HGNC Symbol;Acc:HGNC:10069]
ENSG00000180104	-0,1125628	4,00772868	0,57201676	0,46828167	0,73018752	EXOC3	exocyst complex component 3 [Source:HGNC Symbol;Acc:HGNC:30378]
ENSG00000075711	-0,1113782	4,65588174	0,57044802	0,46887455	0,73041035	DLG1	discs large MAGUK scaffold protein 1 [Source:HGNC Symbol;Acc:HGNC:2900]
ENSG00000137177	0,11037437	4,69624612	0,57055564	0,46883383	0,73041035	KIF13A	kinesin family member 13A [Source:HGNC Symbol;Acc:HGNC:14566]

ENSG00000138182	-0,1694596	3,95284657	0,57080022	0,46874132	0,73041035	KIF20B	kinesin family member 20B [Source:HGNC Symbol;Acc:HGNC:7212]
ENSG00000140553	0,17136676	5,95855201	0,57148946	0,46848081	0,73041035	UNC45A	unc-45 myosin chaperone A [Source:HGNC Symbol;Acc:HGNC:30594]
ENSG00000167291	-0,1360554	5,04928905	0,5704613	0,46886952	0,73041035	TBC1D16	TBC1 domain family member 16 [Source:HGNC Symbol;Acc:HGNC:28356]
ENSG00000172493	0,25100505	3,35876676	0,57065208	0,46879735	0,73041035	AFF1	AF4/FMR2 family member 1 [Source:HGNC Symbol;Acc:HGNC:7135]
ENSG00000188512	-0,2339	0,15937655	0,57091313	0,46869863	0,73041035	AF279873.1	cytochrome c, somatic (CYCS) pseudogene
ENSG00000226415	-0,1656401	2,31390514	0,57058669	0,46882208	0,73041035	TPI1P1	triosephosphate isomerase 1 pseudogene 1 [Source:HGNC Symbol;Acc:HGNC:35449]
ENSG00000226964	0,25577849	0,10882257	0,5705357	0,46884137	0,73041035	RHEBP2	RHEB pseudogene 2 [Source:HGNC Symbol;Acc:HGNC:44991]
ENSG00000125868	-0,1363569	7,2117464	0,57017694	0,46897713	0,73049225	DSTN	destrin, actin depolymerizing factor [Source:HGNC Symbol;Acc:HGNC:15750]
ENSG00000116521	0,11287548	7,38690989	0,56980802	0,46911679	0,73055402	SCAMP3	secretory carrier membrane protein 3 [Source:HGNC Symbol;Acc:HGNC:10565]
ENSG00000239887	0,17899117	3,45998579	0,56982679	0,46910969	0,73055402	C1orf226	chromosome 1 open reading frame 226 [Source:HGNC Symbol;Acc:HGNC:34351]
ENSG00000116350	-0,1182673	6,81295197	0,56930844	0,46930605	0,73077084	SRSF4	serine and arginine rich splicing factor 4 [Source:HGNC Symbol;Acc:HGNC:10786]
ENSG00000127804	0,12760898	4,32632206	0,56876287	0,46951287	0,73097457	METTL16	methyltransferase like 16 [Source:HGNC Symbol;Acc:HGNC:28484]
ENSG00000183747	0,23640327	0,79220353	0,56869937	0,46953695	0,73097457	ACSM2A	acyl-CoA synthetase medium chain family member 2A [Source:HGNC Symbol;Acc:HGNC:32017]
ENSG00000108175	0,31227865	4,4918926	0,5682192	0,46971913	0,73118027	ZMIZ1	zinc finger MIZ-type containing 1 [Source:HGNC Symbol;Acc:HGNC:16493]
ENSG00000204511	0,55468887	-1,1353895	0,56790405	0,46983877	0,73128857	MCCD1	mitochondrial coiled-coil domain 1 [Source:HGNC Symbol;Acc:HGNC:20668]
ENSG00000104419	-0,1609228	4,49821336	0,56776733	0,46989068	0,73129147	NDRG1	N-myc downstream regulated 1 [Source:HGNC Symbol;Acc:HGNC:7679]
ENSG00000091583	0,10451831	11,9738646	0,56709261	0,47014704	0,7314198	APOH	apolipoprotein H [Source:HGNC Symbol;Acc:HGNC:616]
ENSG00000102931	0,15593821	5,12043523	0,56733565	0,47005467	0,7314198	ARL2BP	ADP ribosylation factor like GTPase 2 binding protein [Source:HGNC Symbol;Acc:HGNC:17146]
ENSG00000166197	-0,2002618	8,12690243	0,56726835	0,47008025	0,7314198	NOLC1	nucleolar and coiled-body phosphoprotein 1 [Source:HGNC Symbol;Acc:HGNC:15608]
ENSG00000213051	-0,2161593	0,33165495	0,56702326	0,47017341	0,7314198	RPL5P5	ribosomal protein L5 pseudogene 5 [Source:HGNC Symbol;Acc:HGNC:35564]
ENSG00000233871	0,27337584	-0,3967594	0,56685232	0,4702384	0,73144302	DLG5-AS1	DLG5 antisense RNA 1 [Source:HGNC Symbol;Acc:HGNC:45109]
ENSG00000074054	0,28020481	4,60833179	0,56645883	0,47038807	0,73145834	CLASP1	cytoplasmic linker associated protein 1 [Source:HGNC Symbol;Acc:HGNC:17088]
ENSG00000074071	-0,1031355	7,36868971	0,56605333	0,4705424	0,73145834	MRPS34	mitochondrial ribosomal protein S34 [Source:HGNC Symbol;Acc:HGNC:16618]
ENSG00000131100	-0,1243561	7,07281941	0,56597965	0,47057044	0,73145834	ATP6V1E1	ATPase H+ transporting V1 subunit E1 [Source:HGNC Symbol;Acc:HGNC:857]
ENSG00000169903	0,70449743	-1,8562076	0,56590535	0,47059874	0,73145834	TM4SF4	transmembrane 4 L six family member 4 [Source:HGNC Symbol;Acc:HGNC:11856]
ENSG00000171130	-0,1329424	8,00656933	0,56627084	0,47045961	0,73145834	ATP6V0E2	ATPase H+ transporting V0 subunit e2 [Source:HGNC Symbol;Acc:HGNC:21723]
ENSG00000184612	-0,2097473	0,54042645	0,56661461	0,47032881	0,73145834	RPL7P26	ribosomal protein L7 pseudogene 26 [Source:HGNC Symbol;Acc:HGNC:36630]
ENSG00000227198	-0,3360662	-0,6957409	0,56599717	0,47056377	0,73145834	C6orf47-AS1	C6orf47 antisense RNA 1 [Source:HGNC Symbol;Acc:HGNC:39767]
ENSG00000101680	0,45780692	-1,8530765	0,56540399	0,4707897	0,73154114	LAMA1	laminin subunit alpha 1 [Source:HGNC Symbol;Acc:HGNC:6481]
ENSG00000108932	0,26497987	3,40919801	0,56553352	0,47074035	0,73154114	SLC16A6	solute carrier family 16 member 6 [Source:HGNC Symbol;Acc:HGNC:10927]
ENSG00000133812	0,13563459	3,17562522	0,5653711	0,47080223	0,73154114	SBF2	SET binding factor 2 [Source:HGNC Symbol;Acc:HGNC:2135]
ENSG00000257267	0,15662001	3,07259373	0,56523342	0,4708547	0,73154486	ZNF271P	zinc finger protein 271, pseudogene [Source:HGNC Symbol;Acc:HGNC:13065]
ENSG00000144233	0,10738436	4,61755998	0,56430134	0,47121017	0,73201929	AMMECR1L	AMMECR1 like [Source:HGNC Symbol;Acc:HGNC:28658]
ENSG00000197497	-0,8365304	-2,0344243	0,56373363	0,47142692	0,73221834	ZNF665	zinc finger protein 665 [Source:HGNC Symbol;Acc:HGNC:25885]
ENSG00000231852	-0,3882022	-1,1704125	0,56370318	0,47143854	0,73221834	CYP21A2	cytochrome P450 family 21 subfamily A member 2 [Source:HGNC Symbol;Acc:HGNC:2600]
ENSG00000196123	0,17523883	1,59109987	0,56338413	0,47156043	0,73232979	KIAA0895L	KIAA0895 like [Source:HGNC Symbol;Acc:HGNC:34408]
ENSG00000132485	0,11265504	4,73871467	0,56294818	0,47172707	0,73235502	ZRANB2	zinc finger RANBP2-type containing 2 [Source:HGNC Symbol;Acc:HGNC:13058]
ENSG00000138190	0,12640629	3,07556435	0,56301997	0,47169962	0,73235502	EXOC6	exocyst complex component 6 [Source:HGNC Symbol;Acc:HGNC:23196]
ENSG00000168137	0,22168696	4,72183669	0,56312325	0,47166014	0,73235502	SETD5	SET domain containing 5 [Source:HGNC Symbol;Acc:HGNC:25566]
ENSG00000132792	0,12386651	7,12535305	0,56223493	0,47199992	0,73238313	CTNNBL1	catenin beta like 1 [Source:HGNC Symbol;Acc:HGNC:15879]
ENSG00000142330	0,10763983	4,34673165	0,56268227	0,47182876	0,73238313	CAPN10	calpain 10 [Source:HGNC Symbol;Acc:HGNC:1477]
ENSG00000182504	-0,2150488	0,45762749	0,56230444	0,47197332	0,73238313	CEP97	centrosomal protein 97 [Source:HGNC Symbol;Acc:HGNC:26244]
ENSG00000258056	-0,2896478	0,36138822	0,56211462	0,47204597	0,73238313	AC009779.2	novel transcript, antisense to CD63
ENSG00000266563	-0,4218872	-1,3797771	0,56224825	0,47199482	0,73238313	EIF1P5	eukaryotic translation initiation factor 1 pseudogene 5 [Source:HGNC Symbol;Acc:HGNC:49618]

ENSG00000273136	0,39195282	-0,4576079	0,56258044	0,47186771	0,73238313	NBPF26	NBPF member 26 [Source:HGNC Symbol;Acc:HGNC:49571]
ENSG00000099725	0,19216409	1,17427981	0,56103617	0,47245912	0,73264341	PRKY	protein kinase Y-linked (pseudogene) [Source:HGNC Symbol;Acc:HGNC:9444]
ENSG00000113734	0,11615545	4,6306244	0,56151831	0,47227434	0,73264341	BNIP1	BCL2 interacting protein 1 [Source:HGNC Symbol;Acc:HGNC:1082]
ENSG00000124593	0,4205741	-0,8549957	0,5612463	0,47237857	0,73264341	AL365205.1	novel protein
ENSG00000179523	0,12925455	2,13372547	0,5613946	0,47232174	0,73264341	EIF3J-DT	EIF3J divergent transcript [Source:HGNC Symbol;Acc:HGNC:48616]
ENSG00000237037	-0,2734516	-0,6126778	0,5608914	0,47251463	0,73264341	NDUFA6-DT	NDUFA6 divergent transcript [Source:HGNC Symbol;Acc:HGNC:45273]
ENSG00000269940	0,36308855	0,88202399	0,56092502	0,47250174	0,73264341	AL049840.4	novel transcript, sense intronic to KLC1
ENSG00000132781	-0,3266183	-1,3662858	0,56051285	0,47265983	0,73279077	MUTYH	mutY DNA glycosylase [Source:HGNC Symbol;Acc:HGNC:7527]
ENSG00000011021	0,18646677	5,12016545	0,56014913	0,47279941	0,7329294	CLCN6	chloride voltage-gated channel 6 [Source:HGNC Symbol;Acc:HGNC:2024]
ENSG00000147223	-0,4545683	-1,4242833	0,55978994	0,47293733	0,73302533	RIPPLY1	ripply transcriptional repressor 1 [Source:HGNC Symbol;Acc:HGNC:25117]
ENSG00000164344	0,54942516	-1,9473485	0,55972662	0,47296165	0,73302533	KLKB1	kallikrein B1 [Source:HGNC Symbol;Acc:HGNC:6371]
ENSG00000270804	0,29201511	-0,2978702	0,55952244	0,47304009	0,73306912	AC010326.4	ubiquitin-conjugating enzyme E2C (UBE2C) pseudogene
ENSG00000184844	-0,3246493	-0,9845402	0,55907428	0,47321233	0,73325826	CYCSP45	CYCS pseudogene 45 [Source:HGNC Symbol;Acc:HGNC:24419]
ENSG00000148481	0,12873364	3,9772236	0,5586317	0,47338253	0,73326936	MINDY3	MINDY lysine 48 deubiquitinase 3 [Source:HGNC Symbol;Acc:HGNC:23578]
ENSG00000170144	0,09994875	9,44914154	0,55853364	0,47342026	0,73326936	HNRNPA3	heterogeneous nuclear ribonucleoprotein A3 [Source:HGNC Symbol;Acc:HGNC:24941]
ENSG00000197275	-0,1906513	0,84870481	0,55872832	0,47334537	0,73326936	RAD54B	RAD54 homolog B [Source:HGNC Symbol;Acc:HGNC:17228]
ENSG00000205763	0,14842368	2,37865923	0,55887911	0,47328737	0,73326936	RP9P	RP9 pseudogene [Source:HGNC Symbol;Acc:HGNC:33969]
ENSG00000092853	0,19930969	3,78911507	0,55814066	0,4735715	0,73342585	CLSPN	claspin [Source:HGNC Symbol;Acc:HGNC:19715]
ENSG00000004700	0,15104994	4,38478895	0,55781843	0,47369557	0,73354024	RECQL	RecQ like helicase [Source:HGNC Symbol;Acc:HGNC:9948]
ENSG00000004766	0,12689308	3,25632321	0,55668313	0,47413317	0,7336735	VPS50	VPS50 subunit of EARP/GARPII complex [Source:HGNC Symbol;Acc:HGNC:25956]
ENSG00000011295	0,10881682	4,92259378	0,55713486	0,47395897	0,7336735	TTC19	tetratricopeptide repeat domain 19 [Source:HGNC Symbol;Acc:HGNC:26006]
ENSG00000101079	-0,1038179	6,16922398	0,5570306	0,47399917	0,7336735	NDRG3	NDRG family member 3 [Source:HGNC Symbol;Acc:HGNC:14462]
ENSG00000119906	0,11332422	4,52691079	0,55670696	0,47412398	0,7336735	SLF2	SMC5-SMC6 complex localization factor 2 [Source:HGNC Symbol;Acc:HGNC:17814]
ENSG00000137168	0,12098904	7,40099383	0,55687967	0,47405737	0,7336735	PPIL1	peptidylprolyl isomerase like 1 [Source:HGNC Symbol;Acc:HGNC:9260]
ENSG00000184441	-0,4853252	-1,5571353	0,55712345	0,47396337	0,7336735	AP001062.1	novel transcript, antisense to C21orf2
ENSG00000197256	0,19342446	3,31324314	0,55741285	0,47385182	0,7336735	KANK2	KN motif and ankyrin repeat domains 2 [Source:HGNC Symbol;Acc:HGNC:29300]
ENSG00000164615	0,12063806	4,76793524	0,55649682	0,47420506	0,73370702	CAMLG	calcium modulating ligand [Source:HGNC Symbol;Acc:HGNC:1471]
ENSG00000243926	-0,3570845	-0,2078146	0,55600616	0,47439445	0,73392232	TIPARP-AS1	TIPARP antisense RNA 1 [Source:HGNC Symbol;Acc:HGNC:41028]
ENSG00000073849	0,10777832	7,00528482	0,55565058	0,47453178	0,7339743	ST6GAL1	ST6 beta-galactoside alpha-2,6-sialyltransferase 1 [Source:HGNC Symbol;Acc:HGNC:10860]
ENSG00000170166	0,16631948	1,38796698	0,55539897	0,47462901	0,7339743	HOXD4	homeobox D4 [Source:HGNC Symbol;Acc:HGNC:5138]
ENSG00000229117	-0,1238467	8,01612385	0,55550911	0,47458644	0,7339743	RPL41	ribosomal protein L41 [Source:HGNC Symbol;Acc:HGNC:10354]
ENSG00000244879	0,1270047	3,05648924	0,55558402	0,4745575	0,7339743	GABPB1-AS1	GABPB1 antisense RNA 1 [Source:HGNC Symbol;Acc:HGNC:44157]
ENSG00000103043	0,10299182	5,53514053	0,55523739	0,47469146	0,73399317	VAC14	VAC14 component of PIKFYVE complex [Source:HGNC Symbol;Acc:HGNC:25507]
ENSG00000104231	-0,1196488	4,07376062	0,55495534	0,47480051	0,7340841	ZFAND1	zinc finger AN1-type containing 1 [Source:HGNC Symbol;Acc:HGNC:25858]
ENSG00000165410	0,11551238	6,71635385	0,55450986	0,47497283	0,73427282	CFL2	cofilin 2 [Source:HGNC Symbol;Acc:HGNC:1875]
ENSG00000134897	-0,1640027	1,36364936	0,55415937	0,47510849	0,73440482	BIVM	basic, immunoglobulin-like variable motif containing [Source:HGNC Symbol;Acc:HGNC:16034]
ENSG00000156509	0,21558767	1,54139187	0,55393432	0,47519563	0,73446181	FBXO43	F-box protein 43 [Source:HGNC Symbol;Acc:HGNC:28521]
ENSG00000173621	0,14260668	2,4826292	0,55357181	0,47533606	0,73452342	LRFN4	leucine rich repeat and fibronectin type III domain containing 4 [Source:HGNC Symbol;Acc:HGNC:28456]
ENSG00000238105	0,20509036	1,35949171	0,55368782	0,47529111	0,73452342	GOLGA2P5	GOLGA2 pseudogene 5 [Source:HGNC Symbol;Acc:HGNC:25315]
ENSG00000152942	0,13579104	4,11065002	0,55307904	0,47552706	0,73466316	RAD17	RAD17 checkpoint clamp loader component [Source:HGNC Symbol;Acc:HGNC:9807]
ENSG00000153975	-0,1097047	4,22898007	0,55315809	0,47549641	0,73466316	ZUP1	zinc finger containing ubiquitin peptidase 1 [Source:HGNC Symbol;Acc:HGNC:21224]
ENSG00000172201	0,40213251	-1,0457492	0,55262692	0,47570242	0,73485637	ID4	inhibitor of DNA binding 4, HLH protein [Source:HGNC Symbol;Acc:HGNC:5363]
ENSG00000231799	-0,3629121	-1,6479319	0,55245836	0,47576783	0,7348797	PA2G4P6	proliferation-associated 2G4 pseudogene 6 [Source:HGNC Symbol;Acc:HGNC:32219]
ENSG00000167549	-0,4081155	-1,8479976	0,55207283	0,4759175	0,73503316	CORO6	coronin 6 [Source:HGNC Symbol;Acc:HGNC:21356]

ENSG00000115233	-0,1009439	7,54216902	0,55154343	0,47612314	0,73512424	PSMD14	proteasome 26S subunit, non-ATPase 14 [Source:HGNC Symbol;Acc:HGNC:16889]
ENSG00000121481	0,12994509	3,90401521	0,55153239	0,47612743	0,73512424	RNF2	ring finger protein 2 [Source:HGNC Symbol;Acc:HGNC:10061]
ENSG00000131876	-0,0985172	6,76269654	0,55177223	0,47603424	0,73512424	SNRPA1	small nuclear ribonucleoprotein polypeptide A' [Source:HGNC Symbol;Acc:HGNC:11152]
ENSG00000112343	-0,1341817	2,75356766	0,55132137	0,47620944	0,73514054	TRIM38	tripartite motif containing 38 [Source:HGNC Symbol;Acc:HGNC:10059]
ENSG00000213609	-0,2293936	0,44025133	0,55111687	0,47628895	0,73514054	RPL7AP50	ribosomal protein L7a pseudogene 50 [Source:HGNC Symbol;Acc:HGNC:36117]
ENSG00000249485	0,28779848	-0,8664276	0,55118497	0,47626247	0,73514054	RBBP4P1	RBBP4 pseudogene 1 [Source:HGNC Symbol;Acc:HGNC:42368]
ENSG00000213399	-0,2097249	0,32156496	0,55067693	0,47646006	0,73532697	RPS2P17	ribosomal protein S2 pseudogene 17 [Source:HGNC Symbol;Acc:HGNC:35764]
ENSG00000257261	-0,2430179	-0,1555547	0,55051596	0,4765227	0,73534595	AC008014.1	novel transcript
ENSG00000005844	0,33136929	-0,5555671	0,54987141	0,47677365	0,73557412	ITGAL	integrin subunit alpha L [Source:HGNC Symbol;Acc:HGNC:6148]
ENSG00000113575	0,12880862	6,70973065	0,54997976	0,47673145	0,73557412	PPP2CA	protein phosphatase 2 catalytic subunit alpha [Source:HGNC Symbol;Acc:HGNC:9299]
ENSG00000115137	0,22727575	0,39287194	0,54946002	0,47693395	0,73557412	DNAJC27	DnaJ heat shock protein family (Hsp40) member C27 [Source:HGNC Symbol;Acc:HGNC:30290]
ENSG00000118680	-0,1165007	8,19530959	0,54969989	0,47684048	0,73557412	MYL12B	myosin light chain 12B [Source:HGNC Symbol;Acc:HGNC:29827]
ENSG00000203799	0,23408395	0,23487656	0,54936071	0,47697266	0,73557412	CCDC162P	coiled-coil domain containing 162, pseudogene [Source:HGNC Symbol;Acc:HGNC:21565]
ENSG00000213331	0,27324989	-0,7263569	0,54951374	0,47691302	0,73557412	AC108073.1	peroxiredoxin 6 (PRDX6) pseudogene
ENSG00000153485	0,1319635	2,9636816	0,54879292	0,47719408	0,73562366	TMEM251	transmembrane protein 251 [Source:HGNC Symbol;Acc:HGNC:20218]
ENSG00000162341	-0,1517577	1,86231208	0,54880797	0,47718821	0,73562366	TPCN2	two pore segment channel 2 [Source:HGNC Symbol;Acc:HGNC:20820]
ENSG00000172725	-0,1649881	6,38565233	0,54876185	0,4772062	0,73562366	CORO1B	coronin 1B [Source:HGNC Symbol;Acc:HGNC:2253]
ENSG00000226086	-0,4177083	-1,6623266	0,54879948	0,47719152	0,73562366	EIF3LP3	eukaryotic translation initiation factor 3 subunit L pseudogene 3 [Source:HGNC Symbol;Acc:HGNC:44976]
ENSG00000198695	0,37565743	4,66359692	0,54795932	0,47751949	0,73602893	MT-ND6	mitochondrially encoded NADH:ubiquinone oxidoreductase core subunit 6 [Source:HGNC Symbol;Acc:HGNC:7462
ENSG00000132793	0,1298922	2,95704244	0,54747865	0,4777073	0,73624074	LPIN3	lipin 3 [Source:HGNC Symbol;Acc:HGNC:14451]
ENSG00000047365	-0,4110916	-1,2529326	0,54700413	0,47789284	0,73628608	ARAP2	ArfGAP with RhoGAP domain, ankyrin repeat and PH domain 2 [Source:HGNC Symbol;Acc:HGNC:16924]
ENSG00000163655	0,10399822	6,8231361	0,54702784	0,47788357	0,73628608	GMPS	guanine monophosphate synthase [Source:HGNC Symbol;Acc:HGNC:4378]
ENSG00000164045	0,1070793	5,72037094	0,54690508	0,47793158	0,73628608	CDC25A	cell division cycle 25A [Source:HGNC Symbol;Acc:HGNC:1725]
ENSG00000173926	0,23552361	1,96510679	0,54688787	0,47793832	0,73628608	MARCHF3	membrane associated ring-CH-type finger 3 [Source:HGNC Symbol;Acc:HGNC:28728]
ENSG00000107890	0,13878132	2,32627089	0,54652302	0,47808108	0,73634462	ANKRD26	ankyrin repeat domain 26 [Source:HGNC Symbol;Acc:HGNC:29186]
ENSG00000186204	-0,1079844	3,9314578	0,54663937	0,47803555	0,73634462	CYP4F12	cytochrome P450 family 4 subfamily F member 12 [Source:HGNC Symbol;Acc:HGNC:18857]
ENSG00000198919	0,11903981	2,85495566	0,54640436	0,47812753	0,73634462	DZIP3	DAZ interacting zinc finger protein 3 [Source:HGNC Symbol;Acc:HGNC:30938]
ENSG00000119938	0,12868566	2,98993406	0,54583369	0,47835102	0,73661116	PPP1R3C	protein phosphatase 1 regulatory subunit 3C [Source:HGNC Symbol;Acc:HGNC:9293]
ENSG00000157021	-0,3454768	-1,2771326	0,54520103	0,478599	0,73691535	CIBAR1P1	CIBAR1 pseudogene 1 [Source:HGNC Symbol;Acc:HGNC:32278]
ENSG00000149289	0,32967166	-0,0830081	0,54492012	0,47870918	0,73694144	ZC3H12C	zinc finger CCCH-type containing 12C [Source:HGNC Symbol;Acc:HGNC:29362]
ENSG00000163138	-0,1423668	2,31901177	0,54490061	0,47871684	0,73694144	PACRGL	parkin coregulated like [Source:HGNC Symbol;Acc:HGNC:28442]
ENSG00000005471	0,12100629	3,97559364	0,54426486	0,47896637	0,73715956	ABCB4	ATP binding cassette subfamily B member 4 [Source:HGNC Symbol;Acc:HGNC:45]
ENSG00000061273	-0,1186403	3,78211773	0,54394111	0,47909353	0,73715956	HDAC7	histone deacetylase 7 [Source:HGNC Symbol;Acc:HGNC:14067]
ENSG00000100504	-0,124851	6,03339652	0,54395645	0,4790875	0,73715956	PYGL	glycogen phosphorylase L [Source:HGNC Symbol;Acc:HGNC:9725]
ENSG00000188033	-0,21441	0,23266082	0,54418849	0,47899636	0,73715956	ZNF490	zinc finger protein 490 [Source:HGNC Symbol;Acc:HGNC:23705]
ENSG00000250539	-0,2595262	0,10808098	0,54389709	0,47911082	0,73715956	KRT8P33	keratin 8 pseudogene 33 [Source:HGNC Symbol;Acc:HGNC:39867]
ENSG00000077514	0,11717037	3,70367519	0,54176394	0,4799502	0,73750008	POLD3	DNA polymerase delta 3, accessory subunit [Source:HGNC Symbol;Acc:HGNC:20932]
ENSG00000084623	-0,1207161	8,77279964	0,54214524	0,47979997	0,73750008	EIF3I	eukaryotic translation initiation factor 3 subunit I [Source:HGNC Symbol;Acc:HGNC:3272]
ENSG00000104218	0,18979826	2,63943054	0,54250018	0,4796602	0,73750008	CSPP1	centrosome and spindle pole associated protein 1 [Source:HGNC Symbol;Acc:HGNC:26193]
ENSG00000112096	-0,1003576	7,52016945	0,54204787	0,47983833	0,73750008	SOD2	superoxide dismutase 2 [Source:HGNC Symbol;Acc:HGNC:11180]
ENSG00000135972	0,10930913	6,62132411	0,54141087	0,48008938	0,73750008	MRPS9	mitochondrial ribosomal protein S9 [Source:HGNC Symbol;Acc:HGNC:14501]
ENSG00000142609	0,39425446	-1,8164873	0,54184529	0,47991814	0,73750008	CFAP74	cilia and flagella associated protein 74 [Source:HGNC Symbol;Acc:HGNC:29368]
ENSG00000143106	-0,0956637	8,26541379	0,54213384	0,47980446	0,73750008	PSMA5	proteasome 20S subunit alpha 5 [Source:HGNC Symbol;Acc:HGNC:9534]
ENSG00000162391	-0,1620925	1,8366136	0,54254885	0,47964104	0,73750008	FAM151A	family with sequence similarity 151 member A [Source:HGNC Symbol;Acc:HGNC:25032]

ENSG00000165678	0,10339857	7,90081081	0,54206312	0,47983232	0,73750008	GHITM	growth hormone inducible transmembrane protein [Source:HGNC Symbol;Acc:HGNC:17281]
ENSG00000170248	0,18593701	6,2517242	0,54164119	0,47999858	0,73750008	PDCD6IP	programmed cell death 6 interacting protein [Source:HGNC Symbol;Acc:HGNC:8766]
ENSG00000170631	-0,1117505	3,59758525	0,54153041	0,48004225	0,73750008	ZNF16	zinc finger protein 16 [Source:HGNC Symbol;Acc:HGNC:12947]
ENSG00000175322	-0,3025685	0,33041259	0,54199121	0,47986065	0,73750008	ZNF519	zinc finger protein 519 [Source:HGNC Symbol;Acc:HGNC:30574]
ENSG00000213820	-0,2330159	0,50990255	0,54242882	0,4796883	0,73750008	RPL13P2	ribosomal protein L13 pseudogene 2 [Source:HGNC Symbol;Acc:HGNC:16342]
ENSG00000228501	-0,1919831	0,52006567	0,54249584	0,47966191	0,73750008	RPL15P18	ribosomal protein L15 pseudogene 18 [Source:HGNC Symbol;Acc:HGNC:36515]
ENSG00000250859	0,30946542	-0,8824081	0,5414373	0,48007896	0,73750008	HNRNPKP1	heterogeneous nuclear ribonucleoprotein K pseudogene 1 [Source:HGNC Symbol;Acc:HGNC:42374]
ENSG00000104368	-0,2464883	0,12065106	0,54090312	0,48028966	0,73768462	PLAT	plasminogen activator, tissue type [Source:HGNC Symbol;Acc:HGNC:9051]
ENSG00000158715	0,22627228	2,04333702	0,54071716	0,48036304	0,73768462	SLC45A3	solute carrier family 45 member 3 [Source:HGNC Symbol;Acc:HGNC:8642]
ENSG00000219928	-0,1249498	2,53708334	0,54059444	0,48041149	0,73768462	AL161787.1	ribosomal protein S29 (RPS29) pseudogene
ENSG00000262333	-0,3238934	-1,4872265	0,54068136	0,48037718	0,73768462	HNRNPA1P16	heterogeneous nuclear ribonucleoprotein A1 pseudogene 16 [Source:HGNC Symbol;Acc:HGNC:39134]
ENSG00000197576	0,18532101	0,85877476	0,54041921	0,48048067	0,73771331	HOXA4	homeobox A4 [Source:HGNC Symbol;Acc:HGNC:5105]
ENSG00000102125	-0,1002711	5,14022276	0,54012821	0,4805956	0,73781223	TAZ	tafazzin [Source:HGNC Symbol;Acc:HGNC:11577]
ENSG00000071243	0,13545982	2,50619938	0,53928405	0,48092928	0,73795545	ING3	inhibitor of growth family member 3 [Source:HGNC Symbol;Acc:HGNC:14587]
ENSG00000099901	-0,1675355	7,9146133	0,53908139	0,48100945	0,73795545	RANBP1	RAN binding protein 1 [Source:HGNC Symbol;Acc:HGNC:9847]
ENSG00000116922	0,10872322	4,35842813	0,53922218	0,48095375	0,73795545	C1orf109	chromosome 1 open reading frame 109 [Source:HGNC Symbol;Acc:HGNC:26039]
ENSG00000124486	-0,1129795	5,38919902	0,53939476	0,48088549	0,73795545	USP9X	ubiquitin specific peptidase 9 X-linked [Source:HGNC Symbol;Acc:HGNC:12632]
ENSG00000168288	0,10165527	7,30695361	0,53915683	0,4809796	0,73795545	MMADHC	metabolism of cobalamin associated D [Source:HGNC Symbol;Acc:HGNC:25221]
ENSG00000226121	0,2672128	-0,5914474	0,5394756	0,48085353	0,73795545	AHCTF1P1	AT-hook containing transcription factor 1 pseudogene 1 [Source:HGNC Symbol;Acc:HGNC:24620]
ENSG00000228463	0,24282961	-0,3405523	0,53899787	0,48104249	0,73795545	AP006222.1	ribosomal protein L23a (RPL23A) pseudogene
ENSG00000164241	-0,1665807	1,79412582	0,53881895	0,48111329	0,73798657	C5orf63	chromosome 5 open reading frame 63 [Source:HGNC Symbol;Acc:HGNC:40051]
ENSG00000105519	0,4152113	-1,0339498	0,53813481	0,4813842	0,73801911	CAPS	calcyphosine [Source:HGNC Symbol;Acc:HGNC:1487]
ENSG00000130762	-0,1259449	4,73584789	0,5381275	0,4813871	0,73801911	ARHGEF16	Rho guanine nucleotide exchange factor 16 [Source:HGNC Symbol;Acc:HGNC:15515]
ENSG00000142156	0,32542261	1,27636629	0,53843625	0,4812648	0,73801911	COL6A1	collagen type VI alpha 1 chain [Source:HGNC Symbol;Acc:HGNC:2211]
ENSG00000231074	-0,1452264	4,53484829	0,53817018	0,48137019	0,73801911	HCG18	HLA complex group 18 [Source:HGNC Symbol;Acc:HGNC:31337]
ENSG00000283486	0,46991615	-1,5800115	0,53833252	0,48130588	0,73801911	FAM95C	family with sequence similarity 95 member C [Source:HGNC Symbol;Acc:HGNC:45272]
ENSG00000138231	0,11090923	3,92746498	0,53694753	0,48185498	0,738349	DBR1	debranching RNA lariats 1 [Source:HGNC Symbol;Acc:HGNC:15594]
ENSG00000167842	-0,121472	4,76357662	0,53711064	0,48179026	0,738349	MIS12	MIS12 kinetochore complex component [Source:HGNC Symbol;Acc:HGNC:24967]
ENSG00000174996	0,12017775	4,51342587	0,53700621	0,48183169	0,738349	KLC2	kinesin light chain 2 [Source:HGNC Symbol;Acc:HGNC:20716]
ENSG00000232858	-0,2783811	-0,6638929	0,53734047	0,48169908	0,738349	RPL34P27	ribosomal protein L34 pseudogene 27 [Source:HGNC Symbol;Acc:HGNC:36369]
ENSG00000241431	-0,1877398	0,81175672	0,53721627	0,48174835	0,738349	RPL37P6	ribosomal protein L37 pseudogene 6 [Source:HGNC Symbol;Acc:HGNC:31080]
ENSG00000171241	0,1069806	5,10350756	0,53638114	0,48207985	0,7386161	SHCBP1	SHC binding and spindle associated 1 [Source:HGNC Symbol;Acc:HGNC:29547]
ENSG00000254470	-0,110784	3,68743731	0,53567745	0,48235949	0,73896705	AP5B1	adaptor related protein complex 5 subunit beta 1 [Source:HGNC Symbol;Acc:HGNC:25104]
ENSG00000090971	-0,1295501	4,73586914	0,53544881	0,48245041	0,73902884	NAT14	N-acetyltransferase 14 (putative) [Source:HGNC Symbol;Acc:HGNC:28918]
ENSG00000136870	0,10406721	4,81484182	0,53481196	0,48270382	0,7393395	ZNF189	zinc finger protein 189 [Source:HGNC Symbol;Acc:HGNC:12980]
ENSG00000115866	0,12927835	6,92161186	0,53410385	0,48298587	0,7393787	DARS1	aspartyl-tRNA synthetase 1 [Source:HGNC Symbol;Acc:HGNC:2678]
ENSG00000133627	-0,1229912	3,58965357	0,53347766	0,48323553	0,7393787	ACTR3B	actin related protein 3B [Source:HGNC Symbol;Acc:HGNC:17256]
ENSG00000141367	0,13705674	8,21999098	0,53386607	0,48308064	0,7393787	CLTC	clathrin heavy chain [Source:HGNC Symbol;Acc:HGNC:2092]
ENSG00000171863	-0,1059929	11,5707186	0,53392987	0,48305521	0,7393787	RPS7	ribosomal protein S7 [Source:HGNC Symbol;Acc:HGNC:10440]
ENSG00000197321	0,18321851	4,23961066	0,53362844	0,48317539	0,7393787	SVIL	supervillin [Source:HGNC Symbol;Acc:HGNC:11480]
ENSG00000213672	0,10651153	5,14277356	0,53401639	0,48302072	0,7393787	NCKIPSD	NCK interacting protein with SH3 domain [Source:HGNC Symbol;Acc:HGNC:15486]
ENSG00000226015	0,17280572	1,72185658	0,5340362	0,48301283	0,7393787	CCT8P1	chaperonin containing TCP1 subunit 8 pseudogene 1 [Source:HGNC Symbol;Acc:HGNC:35144]
ENSG00000249210	-0,3184836	0,22761437	0,5336008	0,48318641	0,7393787	GAPDHP38	glyceraldehyde 3 phosphate dehydrogenase pseudogene 38 [Source:HGNC Symbol;Acc:HGNC:37792]
ENSG00000270006	-0,4928719	-1,9424309	0,53444242	0,48285098	0,7393787	AC010531.6	novel transcript, antisense to FBXO31 and C16orf95 readthrough

ENSG00000275342	-0,164287	5,12929884	0,53384741	0,48308808	0,7393787	PRAG1	PEAK1 related, kinase-activating pseudokinase 1 [Source:HGNC Symbol;Acc:HGNC:25438]
ENSG00000136161	-0,247589	0,34151998	0,5331242	0,48337655	0,73951701	RCBTB2	RCC1 and BTB domain containing protein 2 [Source:HGNC Symbol;Acc:HGNC:1914]
ENSG00000103522	-0,1564019	2,15097108	0,53272243	0,48353693	0,73960748	IL21R	interleukin 21 receptor [Source:HGNC Symbol;Acc:HGNC:6006]
ENSG00000151465	0,10057932	7,4566781	0,53273734	0,48353098	0,73960748	CDC123	cell division cycle 123 [Source:HGNC Symbol;Acc:HGNC:16827]
ENSG00000106397	0,15949835	8,18416943	0,53167796	0,48395432	0,73993603	PLOD3	procollagen-lysine,2-oxoglutarate 5-dioxygenase 3 [Source:HGNC Symbol;Acc:HGNC:9083]
ENSG00000115761	-0,1148032	6,02646008	0,53168693	0,48395074	0,73993603	NOL10	nucleolar protein 10 [Source:HGNC Symbol;Acc:HGNC:25862]
ENSG00000138778	0,24638217	3,39097591	0,53170877	0,483942	0,73993603	CENPE	centromere protein E [Source:HGNC Symbol;Acc:HGNC:1856]
ENSG00000204564	0,10496799	4,61227965	0,53180349	0,48390413	0,73993603	C6orf136	chromosome 6 open reading frame 136 [Source:HGNC Symbol;Acc:HGNC:21301]
ENSG00000117697	-0,1082015	5,64118157	0,53128215	0,48411267	0,74010066	NSL1	NSL1 component of MIS12 kinetochore complex [Source:HGNC Symbol;Acc:HGNC:24548]
ENSG00000085982	0,10818864	4,65767713	0,53107283	0,48419644	0,74015128	USP40	ubiquitin specific peptidase 40 [Source:HGNC Symbol;Acc:HGNC:20069]
ENSG00000120699	0,09644593	6,48460418	0,53059978	0,48438586	0,74020847	EXOSC8	exosome component 8 [Source:HGNC Symbol;Acc:HGNC:17035]
ENSG00000188610	-0,2113391	1,08779976	0,53072371	0,48433622	0,74020847	FAM72B	family with sequence similarity 72 member B [Source:HGNC Symbol;Acc:HGNC:24805]
ENSG00000253721	-0,2830524	-0,6168806	0,53072665	0,48433504	0,74020847	SUMO2P20	SUMO2 pseudogene 20 [Source:HGNC Symbol;Acc:HGNC:49355]
ENSG00000237828	0,26067526	-0,1923388	0,5303962	0,48446741	0,74025567	PA2G4P1	proliferation-associated 2G4 pseudogene 1 [Source:HGNC Symbol;Acc:HGNC:8551]
ENSG00000105447	0,10412231	6,85097459	0,53020822	0,48454275	0,74026423	GRWD1	glutamate rich WD repeat containing 1 [Source:HGNC Symbol;Acc:HGNC:21270]
ENSG00000213983	-0,1195531	5,84529	0,53007323	0,48459685	0,74026423	AP1G2	adaptor related protein complex 1 subunit gamma 2 [Source:HGNC Symbol;Acc:HGNC:556]
ENSG00000214192	-0,312015	-1,1500977	0,53000294	0,48462503	0,74026423	UBE2V1P2	ubiquitin conjugating enzyme E2 V1 pseudogene 2 [Source:HGNC Symbol;Acc:HGNC:32648]
ENSG00000175137	0,1103821	4,43490218	0,52967493	0,48475656	0,74038773	SH3BP5L	SH3 binding domain protein 5 like [Source:HGNC Symbol;Acc:HGNC:29360]
ENSG00000003436	-0,1395453	9,34845482	0,52878889	0,48511219	0,74044193	TFPI	tissue factor pathway inhibitor [Source:HGNC Symbol;Acc:HGNC:11760]
ENSG00000122550	0,11395243	4,01498273	0,52883987	0,48509172	0,74044193	KLHL7	kelch like family member 7 [Source:HGNC Symbol;Acc:HGNC:15646]
ENSG00000171793	-0,1142226	6,54139977	0,52870261	0,48514684	0,74044193	CTPS1	CTP synthase 1 [Source:HGNC Symbol;Acc:HGNC:2519]
ENSG00000178082	-0,370522	-1,6605857	0,52886961	0,48507977	0,74044193	TWF1P1	twinfilin 1 pseudogene 1 [Source:HGNC Symbol;Acc:HGNC:21736]
ENSG00000186889	-0,5735596	-1,9402321	0,52898701	0,48503263	0,74044193	TMEM17	transmembrane protein 17 [Source:HGNC Symbol;Acc:HGNC:26623]
ENSG00000197879	-0,1616762	5,60128302	0,52890993	0,48506358	0,74044193	MYO1C	myosin IC [Source:HGNC Symbol;Acc:HGNC:7597]
ENSG00000263846	-0,3114838	-1,29909	0,52876046	0,48512361	0,74044193	CIAPIN1P	cytokine induced apoptosis inhibitor 1 pseudogene [Source:HGNC Symbol;Acc:HGNC:31087]
ENSG00000042445	-0,1112372	6,44644129	0,52809859	0,48538957	0,7406483	RETSAT	retinol saturase [Source:HGNC Symbol;Acc:HGNC:25991]
ENSG00000108424	0,14710477	7,97862768	0,52798771	0,48543415	0,7406483	KPNB1	karyopherin subunit beta 1 [Source:HGNC Symbol;Acc:HGNC:6400]
ENSG00000163872	0,14155863	4,83121148	0,52816011	0,48536484	0,7406483	YEATS2	YEATS domain containing 2 [Source:HGNC Symbol;Acc:HGNC:25489]
ENSG00000172458	-0,2465721	0,18235982	0,52751763	0,48562324	0,74072587	IL17D	interleukin 17D [Source:HGNC Symbol;Acc:HGNC:5984]
ENSG00000180066	-0,4225517	-0,9737273	0,52748318	0,4856371	0,74072587	LINC02870	long intergenic non-protein coding RNA 2870 [Source:HGNC Symbol;Acc:HGNC:27275]
ENSG00000277363	0,19657041	1,56681088	0,52769909	0,48555023	0,74072587	SRCIN1	SRC kinase signaling inhibitor 1 [Source:HGNC Symbol;Acc:HGNC:29506]
ENSG00000104885	-0,1702761	5,48354049	0,52722938	0,48573925	0,74080433	DOT1L	DOT1 like histone lysine methyltransferase [Source:HGNC Symbol;Acc:HGNC:24948]
ENSG00000105879	0,1200344	2,69977702	0,52698258	0,48583861	0,74087853	CBLL1	Cbl proto-oncogene like 1 [Source:HGNC Symbol;Acc:HGNC:21225]
ENSG00000149182	0,11211934	6,50723137	0,52652127	0,48602444	0,74100494	ARFGAP2	ADP ribosylation factor GTPase activating protein 2 [Source:HGNC Symbol;Acc:HGNC:13504]
ENSG00000150347	0,21403327	2,29891889	0,52648209	0,48604023	0,74100494	ARID5B	AT-rich interaction domain 5B [Source:HGNC Symbol;Acc:HGNC:17362]
ENSG00000198642	0,11164775	4,04529902	0,5263991	0,48607368	0,74100494	KLHL9	kelch like family member 9 [Source:HGNC Symbol;Acc:HGNC:18732]
ENSG00000166526	0,10009937	5,83332336	0,52619218	0,48615708	0,74105476	ZNF3	zinc finger protein 3 [Source:HGNC Symbol;Acc:HGNC:13089]
ENSG00000261737	0,39866761	-1,1476172	0,52581118	0,48631073	0,74121163	CLCA4-AS1	CLCA4 antisense RNA 1 [Source:NCBI gene (formerly Entrezgene);Acc:105378828]
ENSG00000022840	0,12460201	6,43379059	0,52471347	0,4867539	0,74160661	RNF10	ring finger protein 10 [Source:HGNC Symbol;Acc:HGNC:10055]
ENSG00000112335	-0,1125311	6,33024311	0,52461658	0,48679305	0,74160661	SNX3	sorting nexin 3 [Source:HGNC Symbol;Acc:HGNC:11174]
ENSG00000167468	-0,1195697	8,98566917	0,52454074	0,4868237	0,74160661	GPX4	glutathione peroxidase 4 [Source:HGNC Symbol;Acc:HGNC:4556]
ENSG00000181450	0,22411069	0,28627734	0,52489505	0,48668054	0,74160661	ZNF678	zinc finger protein 678 [Source:HGNC Symbol;Acc:HGNC:28652]
ENSG00000278238	0,23397135	-0,2027166	0,52464908	0,48677991	0,74160661	AL359513.1	novel transcript
ENSG00000086475	-0,1014736	6,54028678	0,5240905	0,48700572	0,74177162	SEPHS1	selenophosphate synthetase 1 [Source:HGNC Symbol;Acc:HGNC:19685]

ENSG00000100142	0,12936726	6,2624293	0,52402164	0,48703357	0,74177162	POLR2F	RNA polymerase II, I and III subunit F [Source:HGNC Symbol;Acc:HGNC:9193]
ENSG00000145819	0,11581005	3,52055562	0,52362851	0,48719262	0,74185918	ARHGAP26	Rho GTPase activating protein 26 [Source:HGNC Symbol;Acc:HGNC:17073]
ENSG00000251484	-0,3321685	-1,5993825	0,52372169	0,48715491	0,74185918	AL356488.1	poly(rC) binding protein 1 (PCBP1) pseudogene
ENSG00000168496	0,09859767	7,16611153	0,52335045	0,48730517	0,74194189	FEN1	flap structure-specific endonuclease 1 [Source:HGNC Symbol;Acc:HGNC:3650]
ENSG00000188419	0,14640754	2,61349364	0,52324341	0,48734851	0,74194189	CHM	CHM Rab escort protein [Source:HGNC Symbol;Acc:HGNC:1940]
ENSG00000122779	0,12214995	7,690564	0,52150503	0,48805333	0,74202317	TRIM24	tripartite motif containing 24 [Source:HGNC Symbol;Acc:HGNC:11812]
ENSG00000125871	0,09594897	6,26231125	0,52201361	0,48784694	0,74202317	MGME1	mitochondrial genome maintenance exonuclease 1 [Source:HGNC Symbol;Acc:HGNC:16205]
ENSG00000138050	-0,1056644	4,35719676	0,52166862	0,48798692	0,74202317	THUMPD2	THUMP domain containing 2 [Source:HGNC Symbol;Acc:HGNC:14890]
ENSG00000147604	-0,1382671	10,0198247	0,52246151	0,4876653	0,74202317	RPL7	ribosomal protein L7 [Source:HGNC Symbol;Acc:HGNC:10363]
ENSG00000152061	-0,2190617	2,75584235	0,52285195	0,48750707	0,74202317	RABGAP1L	RAB GTPase activating protein 1 like [Source:HGNC Symbol;Acc:HGNC:24663]
ENSG00000165138	0,12691096	2,37207589	0,5222643	0,48774526	0,74202317	ANKS6	ankyrin repeat and sterile alpha motif domain containing 6 [Source:HGNC Symbol;Acc:HGNC:26724]
ENSG00000179222	0,14752954	8,20883297	0,52177233	0,48794483	0,74202317	MAGED1	MAGE family member D1 [Source:HGNC Symbol;Acc:HGNC:6813]
ENSG00000185591	0,22204355	4,08931926	0,52280088	0,48752776	0,74202317	SP1	Sp1 transcription factor [Source:HGNC Symbol;Acc:HGNC:11205]
ENSG00000188859	0,24786803	-0,3022226	0,52167346	0,48798496	0,74202317	FAM78B	family with sequence similarity 78 member B [Source:HGNC Symbol;Acc:HGNC:13495]
ENSG00000229368	-0,3658002	-1,3919038	0,52205774	0,48782904	0,74202317	AC090587.1	novel transcript, sense overlapping STIM1
ENSG00000231752	0,26879749	-0,7308065	0,52199491	0,48785452	0,74202317	EMBP1	embigin pseudogene 1 [Source:HGNC Symbol;Acc:HGNC:38661]
ENSG00000258890	-0,1186165	5,17747061	0,52210989	0,48780788	0,74202317	CEP95	centrosomal protein 95 [Source:HGNC Symbol;Acc:HGNC:25141]
ENSG00000260000	-0,3587135	-0,1014383	0,52148319	0,48806219	0,74202317	AL133338.1	novel transcript, antisense to ASCC3
ENSG00000111224	-0,1729754	0,72278217	0,5206397	0,48840485	0,74221094	PARP11	poly(ADP-ribose) polymerase family member 11 [Source:HGNC Symbol;Acc:HGNC:1186]
ENSG00000130559	-0,1518295	4,25901512	0,52081802	0,48833237	0,74221094	CAMSAP1	calmodulin regulated spectrin associated protein 1 [Source:HGNC Symbol;Acc:HGNC:19946]
ENSG00000151014	-0,1415534	3,25165791	0,52078181	0,48834709	0,74221094	NOCT	nocturnin [Source:HGNC Symbol;Acc:HGNC:14254]
ENSG00000151532	0,11224015	4,45200115	0,52053675	0,4884467	0,74221094	VTI1A	vesicle transport through interaction with t-SNAREs 1A [Source:HGNC Symbol;Acc:HGNC:17792]
ENSG00000168918	-0,1292409	4,73802599	0,52017909	0,48859214	0,74221094	INPP5D	inositol polyphosphate-5-phosphatase D [Source:HGNC Symbol;Acc:HGNC:6079]
ENSG00000184110	-0,2140146	5,23336822	0,52096872	0,48827114	0,74221094	EIF3C	eukaryotic translation initiation factor 3 subunit C [Source:HGNC Symbol;Acc:HGNC:3279]
ENSG00000239440	0,21052265	0,67002786	0,52029606	0,48854457	0,74221094	LINC02008	long intergenic non-protein coding RNA 2008 [Source:HGNC Symbol;Acc:HGNC:52844]
ENSG00000259956	-0,1043031	6,28888565	0,5203891	0,48850673	0,74221094	RBM15B	RNA binding motif protein 15B [Source:HGNC Symbol;Acc:HGNC:24303]
ENSG00000143543	-0,1636169	3,04558893	0,51974856	0,48876732	0,74239986	JTB	jumping translocation breakpoint [Source:HGNC Symbol;Acc:HGNC:6201]
ENSG00000041880	0,11130365	3,36492296	0,51870368	0,48919295	0,74273746	PARP3	poly(ADP-ribose) polymerase family member 3 [Source:HGNC Symbol;Acc:HGNC:273]
ENSG00000186010	-0,3061174	-0,8406087	0,51870521	0,48919233	0,74273746	NDUFA13	NADH:ubiquinone oxidoreductase subunit A13 [Source:HGNC Symbol;Acc:HGNC:17194]
ENSG00000204178	0,11194797	5,71376409	0,51882247	0,48914453	0,74273746	MACO1	macoilin 1 [Source:HGNC Symbol;Acc:HGNC:25572]
ENSG00000268205	0,35706982	2,15952463	0,5188654	0,48912703	0,74273746	AC005261.1	novel transcript
ENSG00000106591	0,09780076	7,21251797	0,51826013	0,48937383	0,74293487	MRPL32	mitochondrial ribosomal protein L32 [Source:HGNC Symbol;Acc:HGNC:14035]
ENSG00000265393	-0,572876	-1,6451853	0,51810143	0,48943857	0,74295596	AC084125.4	novel transcript, antisense to RECQL4
ENSG00000053108	0,17773386	0,98043108	0,51745685	0,48970171	0,74313589	FSTL4	follistatin like 4 [Source:HGNC Symbol;Acc:HGNC:21389]
ENSG00000100603	-0,132502	6,26047678	0,51733271	0,48975241	0,74313589	SNW1	SNW domain containing 1 [Source:HGNC Symbol;Acc:HGNC:16696]
ENSG00000106609	0,12241283	6,26070954	0,5173127	0,48976059	0,74313589	TMEM248	transmembrane protein 248 [Source:HGNC Symbol;Acc:HGNC:25476]
ENSG00000164889	-0,1139659	7,3364988	0,5173323	0,48975258	0,74313589	SLC4A2	solute carrier family 4 member 2 [Source:HGNC Symbol;Acc:HGNC:11028]
ENSG00000105245	-0,5290888	-1,9289702	0,51706549	0,48986159	0,74321196	NUMBL	NUMB like endocytic adaptor protein [Source:HGNC Symbol;Acc:HGNC:8061]
ENSG00000090512	-0,1478287	1,75138473	0,51604937	0,49027715	0,74346253	FETUB	fetuin B [Source:HGNC Symbol;Acc:HGNC:3658]
ENSG00000130311	-0,116422	4,51645519	0,5161296	0,49024431	0,74346253	DDA1	DET1 and DDB1 associated 1 [Source:HGNC Symbol;Acc:HGNC:28360]
ENSG00000134684	-0,1389979	7,81439839	0,51603949	0,4902812	0,74346253	YARS1	tyrosyl-tRNA synthetase 1 [Source:HGNC Symbol;Acc:HGNC:12840]
ENSG00000174799	0,23446334	1,60275137	0,51644208	0,49011647	0,74346253	CEP135	centrosomal protein 135 [Source:HGNC Symbol;Acc:HGNC:29086]
ENSG00000250682	-0,4294028	-1,5536047	0,51632187	0,49016564	0,74346253	LINC00491	long intergenic non-protein coding RNA 491 [Source:HGNC Symbol;Acc:HGNC:43428]
ENSG00000103994	0,18999087	4,50165116	0,51577599	0,49038906	0,74354891	ZNF106	zinc finger protein 106 [Source:HGNC Symbol;Acc:HGNC:12886]

ENSG00000007341	-0,1099074	4,01524921	0,51548384	0,49050871	0,74365314	ST7L	suppression of tumorigenicity 7 like [Source:HGNC Symbol;Acc:HGNC:18441]
ENSG00000275029	-0,3543202	-1,4711856	0,51474435	0,49081179	0,74403543	HMGB1P24	high mobility group box 1 pseudogene 24 [Source:HGNC Symbol;Acc:HGNC:39115]
ENSG00000082781	0,09799237	7,77194349	0,51379994	0,49119934	0,74446842	ITGB5	integrin subunit beta 5 [Source:HGNC Symbol;Acc:HGNC:6160]
ENSG00000150403	-0,0975102	5,24223635	0,5138481	0,49117956	0,74446842	TMCO3	transmembrane and coiled-coil domains 3 [Source:HGNC Symbol;Acc:HGNC:20329]
ENSG00000105722	-0,1018162	5,71545157	0,51244903	0,49175466	0,74468609	ERF	ETS2 repressor factor [Source:HGNC Symbol;Acc:HGNC:3444]
ENSG00000108961	-0,1311309	4,64766489	0,512087	0,49190367	0,74468609	RANGRF	RAN guanine nucleotide release factor [Source:HGNC Symbol;Acc:HGNC:17679]
ENSG00000119314	0,10812294	4,38461919	0,51223409	0,49184312	0,74468609	PTBP3	polypyrimidine tract binding protein 3 [Source:HGNC Symbol;Acc:HGNC:10253]
ENSG00000123159	0,10234258	5,98456553	0,5129266	0,49155822	0,74468609	GIPC1	GIPC PDZ domain containing family member 1 [Source:HGNC Symbol;Acc:HGNC:1226]
ENSG00000138134	-0,4709171	-1,8649736	0,51245231	0,49175331	0,74468609	STAMBPL1	STAM binding protein like 1 [Source:HGNC Symbol;Acc:HGNC:24105]
ENSG00000165283	-0,100119	8,70898476	0,51222771	0,49184575	0,74468609	STOML2	stomatin like 2 [Source:HGNC Symbol;Acc:HGNC:14559]
ENSG00000172354	-0,1196514	7,6118535	0,51246942	0,49174627	0,74468609	GNB2	G protein subunit beta 2 [Source:HGNC Symbol;Acc:HGNC:4398]
ENSG00000176826	-0,3019241	-0,8742214	0,51263533	0,49167801	0,74468609	FKBP9P1	FKBP prolyl isomerase 9 pseudogene 1 [Source:HGNC Symbol;Acc:HGNC:23568]
ENSG00000240036	-0,1635017	1,34760757	0,51298385	0,49153468	0,74468609	AC104563.1	novel pseudogene
ENSG00000240695	0,29024886	-0,8811651	0,51216888	0,49186996	0,74468609	AC117382.1	DnaJ (Hsp40) homolog, subfamily C, member 8 (DNAJC8) pseudogene
ENSG00000250986	-0,5060855	-1,1852681	0,51322381	0,49143603	0,74468609	LINC02600	long intergenic non-protein coding RNA 2600 [Source:HGNC Symbol;Acc:HGNC:53177]
ENSG00000083828	-0,3288405	-0,8070935	0,51176976	0,49203432	0,74471956	ZNF586	zinc finger protein 586 [Source:HGNC Symbol;Acc:HGNC:25949]
ENSG00000132561	-0,3776822	-1,5797458	0,51176836	0,49203489	0,74471956	MATN2	matrilin 2 [Source:HGNC Symbol;Acc:HGNC:6908]
ENSG00000175106	0,16251184	1,61152122	0,51166198	0,49207872	0,74471956	TVP23C	trans-golgi network vesicle protein 23 homolog C [Source:HGNC Symbol;Acc:HGNC:30453]
ENSG00000101871	0,3471381	-1,3435058	0,51055815	0,49253384	0,74481232	MID1	midline 1 [Source:HGNC Symbol;Acc:HGNC:7095]
ENSG00000117143	-0,0928152	6,0706288	0,51096653	0,49236537	0,74481232	UAP1	UDP-N-acetylglucosamine pyrophosphorylase 1 [Source:HGNC Symbol;Acc:HGNC:12457]
ENSG00000135823	0,14871361	3,63143415	0,51060047	0,49251638	0,74481232	STX6	syntaxin 6 [Source:HGNC Symbol;Acc:HGNC:11441]
ENSG00000152219	0,12119103	3,76947432	0,51046707	0,49257143	0,74481232	ARL14EP	ADP ribosylation factor like GTPase 14 effector protein [Source:HGNC Symbol;Acc:HGNC:26798]
ENSG00000165716	0,31024661	-0,7295652	0,51129407	0,49223033	0,74481232	DIPK1B	divergent protein kinase domain 1B [Source:HGNC Symbol;Acc:HGNC:28290]
ENSG00000196550	-0,2466618	0,51973697	0,51108908	0,49231484	0,74481232	FAM72A	family with sequence similarity 72 member A [Source:HGNC Symbol;Acc:HGNC:24044]
ENSG00000248275	-0,1909209	1,38323595	0,51033249	0,49262698	0,74481232	TRIM52-AS1	TRIM52 antisense RNA 1 (head to head) [Source:HGNC Symbol;Acc:HGNC:49006]
ENSG00000255967	-0,3183628	-0,5410971	0,51052024	0,49254949	0,74481232	HADHAP2	HADHA pseudogene 2 [Source:HGNC Symbol;Acc:HGNC:38585]
ENSG00000265808	0,09934968	5,33115258	0,51056802	0,49252977	0,74481232	SEC22B	SEC22 homolog B, vesicle trafficking protein [Source:HGNC Symbol;Acc:HGNC:10700]
ENSG00000275160	-0,4166701	-1,8778879	0,51027713	0,49264983	0,74481232	AL354718.1	ribosomal protein L7a (RPL7A) pseudogene
ENSG00000257218	-0,1163754	4,49118348	0,51013254	0,49270953	0,74482549	GATC	glutamyl-tRNA amidotransferase subunit C [Source:HGNC Symbol;Acc:HGNC:25068]
ENSG00000115414	0,23150796	11,181963	0,5097798	0,49285523	0,74486978	FN1	fibronectin 1 [Source:HGNC Symbol;Acc:HGNC:3778]
ENSG00000125354	0,11599047	4,56046553	0,50969077	0,49289201	0,74486978	SEPTIN6	septin 6 [Source:HGNC Symbol;Acc:HGNC:15848]
ENSG00000235174	-0,1583513	1,47959203	0,50966078	0,4929044	0,74486978	RPL39P3	ribosomal protein L39 pseudogene 3 [Source:HGNC Symbol;Acc:HGNC:21383]
ENSG00000285043	0,23625299	-0,1145838	0,50956792	0,49294278	0,74486978	ALDOA	aldolase, fructose-bisphosphate A [Source:HGNC Symbol;Acc:HGNC:414]
ENSG00000157869	0,17346672	2,62314064	0,50927234	0,49306496	0,74496433	RAB28	RAB28, member RAS oncogene family [Source:HGNC Symbol;Acc:HGNC:9768]
ENSG00000205356	-0,1623138	2,41135321	0,50916986	0,49310733	0,74496433	TECPR1	tectonin beta-propeller repeat containing 1 [Source:HGNC Symbol;Acc:HGNC:22214]
ENSG00000039560	0,14116899	4,4669236	0,50898081	0,49318552	0,7450054	RAI14	retinoic acid induced 14 [Source:HGNC Symbol;Acc:HGNC:14873]
ENSG00000136536	0,0992532	5,44956994	0,50883993	0,4932438	0,74501641	MARCHF7	membrane associated ring-CH-type finger 7 [Source:HGNC Symbol;Acc:HGNC:17393]
ENSG00000091651	0,09523679	5,82596614	0,50826273	0,49348271	0,74525836	ORC6	origin recognition complex subunit 6 [Source:HGNC Symbol;Acc:HGNC:17151]
ENSG00000148229	0,12967426	5,03259108	0,50808327	0,49355703	0,74525836	POLE3	DNA polymerase epsilon 3, accessory subunit [Source:HGNC Symbol;Acc:HGNC:13546]
ENSG00000227540	0,19387987	0,54848474	0,50812249	0,49354079	0,74525836	DNAJC9-AS1	DNAJC9 antisense RNA 1 [Source:NCBI gene (formerly Entrezgene);Acc:414245]
ENSG00000163634	-0,110459	5,77548623	0,50710558	0,49396229	0,745736	THOC7	THO complex 7 [Source:HGNC Symbol;Acc:HGNC:29874]
ENSG00000283196	0,13621757	3,63722348	0,50707388	0,49397544	0,745736	LSP1P5	LSP1 pseudogene 5 [Source:NCBI gene (formerly Entrezgene);Acc:645166]
ENSG00000110330	0,11321793	4,82893394	0,50627877	0,49430548	0,7457862	BIRC2	baculoviral IAP repeat containing 2 [Source:HGNC Symbol;Acc:HGNC:590]
ENSG00000149932	0,09602349	7,61365785	0,5067121	0,49412556	0,7457862	TMEM219	transmembrane protein 219 [Source:HGNC Symbol;Acc:HGNC:25201]

ENSG00000164066	0,16743912	1,40413819	0,50647511	0,49422395	0,7457862	INTU	inturned planar cell polarity protein [Source:HGNC Symbol;Acc:HGNC:29239]
ENSG00000172977	-0,1144812	5,56905705	0,50641512	0,49424885	0,7457862	KAT5	lysine acetyltransferase 5 [Source:HGNC Symbol;Acc:HGNC:5275]
ENSG00000214253	-0,1104834	6,21815138	0,5064485	0,49423499	0,7457862	FIS1	fission, mitochondrial 1 [Source:HGNC Symbol;Acc:HGNC:21689]
ENSG00000243824	-0,21951	0,12864377	0,50625585	0,494315	0,7457862	RPL12P6	ribosomal protein L12 pseudogene 6 [Source:HGNC Symbol;Acc:HGNC:23538]
ENSG00000130173	0,11370569	5,16345383	0,50592699	0,49445162	0,74583948	ANGPTL8	angiopoietin like 8 [Source:HGNC Symbol;Acc:HGNC:24933]
ENSG00000176101	-0,119076	5,75884417	0,50580224	0,49450347	0,74583948	SSNA1	SS nuclear autoantigen 1 [Source:HGNC Symbol;Acc:HGNC:11321]
ENSG00000178429	0,13539693	1,94036622	0,50589923	0,49446316	0,74583948	RPS3AP5	RPS3A pseudogene 5 [Source:HGNC Symbol;Acc:HGNC:23744]
ENSG00000182405	-0,2272711	0,40481591	0,50506553	0,49480984	0,74622452	PGBD4	piggyBac transposable element derived 4 [Source:HGNC Symbol;Acc:HGNC:19401]
ENSG00000100280	0,15696712	5,7731007	0,50379706	0,49533815	0,74687662	AP1B1	adaptor related protein complex 1 subunit beta 1 [Source:HGNC Symbol;Acc:HGNC:554]
ENSG00000228327	0,47493493	-1,0572367	0,50365926	0,49539561	0,74687662	AL669831.1	general transcription factor IIi (GTF2I) pseudogene
ENSG00000286408	-0,4043997	-0,9706401	0,50376297	0,49535236	0,74687662	AC113410.4	novel transcript
ENSG00000235065	-0,2702173	-0,1634451	0,50327596	0,49555548	0,74704056	RPL24P2	RPL24 pseudogene 2 [Source:HGNC Symbol;Acc:HGNC:16597]
ENSG00000119929	-0,1051825	4,39356373	0,5029648	0,49568534	0,74715922	CUTC	cutC copper transporter [Source:HGNC Symbol;Acc:HGNC:24271]
ENSG00000087903	-0,270539	-0,0525236	0,50283566	0,49573925	0,74716339	RFX2	regulatory factor X2 [Source:HGNC Symbol;Acc:HGNC:9983]
ENSG00000106608	0,11397262	5,13431652	0,50183079	0,49615911	0,74741065	URGCP	upregulator of cell proliferation [Source:HGNC Symbol;Acc:HGNC:30890]
ENSG00000161048	-0,2487425	0,64278975	0,50203996	0,49607166	0,74741065	NAPEPLD	N-acyl phosphatidylethanolamine phospholipase D [Source:HGNC Symbol;Acc:HGNC:21683]
ENSG00000204099	-0,1286689	5,59664905	0,50202544	0,49607773	0,74741065	NEU4	neuraminidase 4 [Source:HGNC Symbol;Acc:HGNC:21328]
ENSG00000241478	0,24734763	-0,3367329	0,50203344	0,49607439	0,74741065	HSPA8P9	heat shock protein family A (Hsp70) member 8 pseudogene 9 [Source:HGNC Symbol;Acc:HGNC:44924]
ENSG00000243885	0,23392369	-0,1632173	0,50192727	0,49611877	0,74741065	AC108751.4	novel transcript
ENSG00000089006	-0,0927883	9,58708907	0,50135271	0,49635909	0,74749056	SNX5	sorting nexin 5 [Source:HGNC Symbol;Acc:HGNC:14969]
ENSG00000117601	-0,1591987	7,50170187	0,50141185	0,49633434	0,74749056	SERPINC1	serpin family C member 1 [Source:HGNC Symbol;Acc:HGNC:775]
ENSG00000138785	-0,1267827	4,04716933	0,50114945	0,49644416	0,74749056	INTS12	integrator complex subunit 12 [Source:HGNC Symbol;Acc:HGNC:25067]
ENSG00000185085	-0,1034453	4,62172276	0,50125888	0,49639836	0,74749056	INTS5	integrator complex subunit 5 [Source:HGNC Symbol;Acc:HGNC:29352]
ENSG00000224839	-0,3512271	-1,4464169	0,50103122	0,49649365	0,74749056	RPL12P17	ribosomal protein L12 pseudogene 17 [Source:HGNC Symbol;Acc:HGNC:36263]
ENSG00000279081	0,3375312	-0,6917458	0,5009703	0,49651916	0,74749056	AL356585.1	eukaryotic translation initiation factor 3 subunit F (EIF3F) pseudogene
ENSG00000164039	0,20115839	3,49437871	0,5000735	0,4968949	0,74790208	BDH2	3-hydroxybutyrate dehydrogenase 2 [Source:HGNC Symbol;Acc:HGNC:32389]
ENSG00000178028	0,10834545	5,53497295	0,50008639	0,49688949	0,74790208	DMAP1	DNA methyltransferase 1 associated protein 1 [Source:HGNC Symbol;Acc:HGNC:18291]
ENSG00000111199	0,17701857	1,43697013	0,49994166	0,49695018	0,74790823	TRPV4	transient receptor potential cation channel subfamily V member 4 [Source:HGNC Symbol;Acc:HGNC:18083]
ENSG00000171792	-0,1369086	4,81763018	0,49950021	0,49713537	0,74810986	RHNO1	RAD9-HUS1-RAD1 interacting nuclear orphan 1 [Source:HGNC Symbol;Acc:HGNC:28206]
ENSG00000268565	-0,4270642	-1,6514105	0,49918068	0,49726948	0,74823461	AC005339.1	novel transcript, antisense to TNFAIP8L1
ENSG00000125247	-0,1101834	4,58167266	0,4985544	0,49753254	0,74847626	TMTC4	transmembrane O-mannosyltransferase targeting cadherins 4 [Source:HGNC Symbol;Acc:HGNC:25904]
ENSG00000226752	-0,1307074	2,02514129	0,49866305	0,49748689	0,74847626	CUTALP	cutA divalent cation tolerance like, pseudogene [Source:HGNC Symbol;Acc:HGNC:27367]
ENSG00000197746	0,18629106	9,93303851	0,49836047	0,49761406	0,7485218	PSAP	prosaposin [Source:HGNC Symbol;Acc:HGNC:9498]
ENSG00000105676	-0,1025279	5,93513235	0,49773521	0,49787702	0,74871527	ARMC6	armadillo repeat containing 6 [Source:HGNC Symbol;Acc:HGNC:25049]
ENSG00000112029	-0,1062165	4,29371611	0,49783052	0,49783692	0,74871527	FBXO5	F-box protein 5 [Source:HGNC Symbol;Acc:HGNC:13584]
ENSG00000124568	0,51181382	-1,1229159	0,49756731	0,49794767	0,74871527	SLC17A1	solute carrier family 17 member 1 [Source:HGNC Symbol;Acc:HGNC:10929]
ENSG00000171314	-0,1068235	7,47047241	0,49758283	0,49794114	0,74871527	PGAM1	phosphoglycerate mutase 1 [Source:HGNC Symbol;Acc:HGNC:8888]
ENSG00000086666	0,12358853	4,5456641	0,49526787	0,49891715	0,74917181	ZFAND6	zinc finger AN1-type containing 6 [Source:HGNC Symbol;Acc:HGNC:30164]
ENSG00000102098	0,13534389	2,68795129	0,49472385	0,49914703	0,74917181	SCML2	Scm polycomb group protein like 2 [Source:HGNC Symbol;Acc:HGNC:10581]
ENSG00000103226	0,22126052	3,90834835	0,49507711	0,49899774	0,74917181	NOMO3	NODAL modulator 3 [Source:HGNC Symbol;Acc:HGNC:25242]
ENSG00000105287	0,10620198	4,43999865	0,49648146	0,49840506	0,74917181	PRKD2	protein kinase D2 [Source:HGNC Symbol;Acc:HGNC:17293]
ENSG00000105698	-0,0971573	5,97768157	0,4964305	0,49842654	0,74917181	USF2	upstream transcription factor 2, c-fos interacting [Source:HGNC Symbol;Acc:HGNC:12594]
ENSG00000107372	-0,102637	5,74056242	0,49461455	0,49919323	0,74917181	ZFAND5	zinc finger AN1-type containing 5 [Source:HGNC Symbol;Acc:HGNC:13008]
ENSG00000119638	-0,1290636	4,64988336	0,49500409	0,49902859	0,74917181	NEK9	NIMA related kinase 9 [Source:HGNC Symbol;Acc:HGNC:18591]

ENSG00000134825	-0,1033144	6,72867438	0,4953554	0,49888019	0,74917181	TMEM258	transmembrane protein 258 [Source:HGNC Symbol;Acc:HGNC:1164]
ENSG00000151665	-0,1213778	5,62600832	0,49561857	0,49876907	0,74917181	PIGF	phosphatidylinositol glycan anchor biosynthesis class F [Source:HGNC Symbol;Acc:HGNC:8962]
ENSG00000155034	0,2066209	2,3657276	0,49590956	0,49864626	0,74917181	FBXL18	F-box and leucine rich repeat protein 18 [Source:HGNC Symbol;Acc:HGNC:21874]
ENSG00000161249	-0,1474645	4,041552	0,49515414	0,49896519	0,74917181	DMKN	dermokine [Source:HGNC Symbol;Acc:HGNC:25063]
ENSG00000170291	0,10463124	6,15712379	0,49594089	0,49863305	0,74917181	ELP5	elongator acetyltransferase complex subunit 5 [Source:HGNC Symbol;Acc:HGNC:30617]
ENSG00000175336	0,16092581	3,79962031	0,49453787	0,49922565	0,74917181	APOF	apolipoprotein F [Source:HGNC Symbol;Acc:HGNC:615]
ENSG00000184470	-0,1458924	5,22258844	0,49517818	0,49895504	0,74917181	TXNRD2	thioredoxin reductase 2 [Source:HGNC Symbol;Acc:HGNC:18155]
ENSG00000197085	-0,2513918	0,15582261	0,49671797	0,49830537	0,74917181	NPSR1-AS1	NPSR1 antisense RNA 1 [Source:HGNC Symbol;Acc:HGNC:22128]
ENSG00000204923	0,35062868	-0,7273458	0,49510771	0,49898481	0,74917181	FBXO48	F-box protein 48 [Source:HGNC Symbol;Acc:HGNC:33857]
ENSG00000207650	-0,4314076	-1,3877571	0,49607473	0,49857658	0,74917181	MIR570	microRNA 570 [Source:HGNC Symbol;Acc:HGNC:32826]
ENSG00000228709	0,14344214	1,94810543	0,49482381	0,49910477	0,74917181	LINC02575	long intergenic non-protein coding RNA 2575 [Source:HGNC Symbol;Acc:HGNC:53747]
ENSG00000232385	-0,279701	-0,5515004	0,49493882	0,49905617	0,74917181	RPS3AP25	RPS3A pseudogene 25 [Source:HGNC Symbol;Acc:HGNC:36801]
ENSG00000111252	-0,1404706	2,77763668	0,49369325	0,49958302	0,74939764	SH2B3	SH2B adaptor protein 3 [Source:HGNC Symbol;Acc:HGNC:29605]
ENSG00000117016	0,18080806	1,48929728	0,4935816	0,49963029	0,74939764	RIMS3	regulating synaptic membrane exocytosis 3 [Source:HGNC Symbol;Acc:HGNC:21292]
ENSG00000172037	0,22051101	6,34576482	0,49313725	0,49981852	0,74939764	LAMB2	laminin subunit beta 2 [Source:HGNC Symbol;Acc:HGNC:6487]
ENSG00000179818	-0,1390933	2,94448538	0,49357755	0,49963201	0,74939764	PCBP1-AS1	PCBP1 antisense RNA 1 [Source:HGNC Symbol;Acc:HGNC:42948]
ENSG00000213650	-0,4305968	-1,9239708	0,49313951	0,49981756	0,74939764	AC092447.2	tyrosine 3-monooxygenase/tryptophan 5-monooxygenase activation protein, epsilon polypeptide (YWHAE) pseud
ENSG00000214429	-0,2622169	-0,5971048	0,49309173	0,49983781	0,74939764	CYCSP6	CYCS pseudogene 6 [Source:HGNC Symbol;Acc:HGNC:24421]
ENSG00000262902	0,11346097	2,89820693	0,49395833	0,49947081	0,74939764	MTCO1P40	MT-CO1 pseudogene 40 [Source:HGNC Symbol;Acc:HGNC:52105]
ENSG00000265646	-0,2323348	0,18071587	0,49317616	0,49980204	0,74939764	TUFMP1	Tu translation elongation factor, mitochondrial pseudogene 1 [Source:HGNC Symbol;Acc:HGNC:12421]
ENSG00000278768	0,38604672	-0,7024199	0,49326426	0,49976471	0,74939764	BACE1-AS	BACE1 antisense RNA [Source:HGNC Symbol;Acc:HGNC:37125]
ENSG00000137337	0,29412137	4,76174282	0,49280725	0,49995839	0,74945558	MDC1	mediator of DNA damage checkpoint 1 [Source:HGNC Symbol;Acc:HGNC:21163]
ENSG00000166681	-0,1272596	5,04823684	0,49272338	0,49999395	0,74945558	BEX3	brain expressed X-linked 3 [Source:HGNC Symbol;Acc:HGNC:13388]
ENSG00000257086	-0,2534838	-0,6603052	0,49263753	0,50003036	0,74945558	AP001453.2	novel transcript
ENSG00000276256	0,11205755	2,72906335	0,49251275	0,50008328	0,749458	nan	nan
ENSG00000183569	-0,3331132	-1,6663545	0,49227611	0,50018367	0,74953157	SERHL2	serine hydrolase like 2 [Source:HGNC Symbol;Acc:HGNC:29446]
ENSG00000050426	0,10228546	5,04860918	0,49154576	0,50049374	0,74984238	LETMD1	LETM1 domain containing 1 [Source:HGNC Symbol;Acc:HGNC:24241]
ENSG00000196655	0,09455739	6,41217268	0,49156659	0,50048489	0,74984238	TRAPPC4	trafficking protein particle complex 4 [Source:HGNC Symbol;Acc:HGNC:19943]
ENSG00000154582	-0,1112917	6,07036939	0,49138249	0,5005631	0,7498694	ELOC	elongin C [Source:HGNC Symbol;Acc:HGNC:11617]
ENSG00000090776	-0,1200766	2,77685962	0,49103363	0,50071137	0,74987239	EFNB1	ephrin B1 [Source:HGNC Symbol;Acc:HGNC:3226]
ENSG00000101474	0,10283602	9,13597031	0,49101176	0,50072067	0,74987239	APMAP	adipocyte plasma membrane associated protein [Source:HGNC Symbol;Acc:HGNC:13238]
ENSG00000144791	0,15662982	3,63345056	0,49123504	0,50062576	0,74987239	LIMD1	LIM domains containing 1 [Source:HGNC Symbol;Acc:HGNC:6612]
ENSG00000270885	-0,1189189	2,96860593	0,49089475	0,50077042	0,74987239	RASL10B	RAS like family 10 member B [Source:HGNC Symbol;Acc:HGNC:30295]
ENSG00000133619	-0,1172211	2,43698965	0,48981862	0,50122839	0,75046244	KRBA1	KRAB-A domain containing 1 [Source:HGNC Symbol;Acc:HGNC:22228]
ENSG00000272447	-0,3170061	0,23997642	0,48972751	0,5012672	0,75046244	AL135925.1	novel transcript
ENSG00000157557	-0,1394349	6,40185978	0,48920797	0,5014886	0,75071698	ETS2	ETS proto-oncogene 2, transcription factor [Source:HGNC Symbol;Acc:HGNC:3489]
ENSG00000107175	0,11385333	6,23772733	0,48835809	0,50185117	0,75102886	CREB3	cAMP responsive element binding protein 3 [Source:HGNC Symbol;Acc:HGNC:2347]
ENSG00000178409	0,12957353	2,98713179	0,48850343	0,50178913	0,75102886	BEND3	BEN domain containing 3 [Source:HGNC Symbol;Acc:HGNC:23040]
ENSG00000241837	-0,1103843	6,38178887	0,48837597	0,50184353	0,75102886	ATP5PO	ATP synthase peripheral stalk subunit OSCP [Source:HGNC Symbol;Acc:HGNC:850]
ENSG00000228057	0,38151762	-0,8205698	0,48811802	0,50195367	0,75110531	SEC63P1	SEC63 homolog, protein translocation regulator pseudogene 1 [Source:HGNC Symbol;Acc:HGNC:41953]
ENSG00000143633	-0,1327429	4,67486422	0,48771211	0,50212706	0,75113396	C1orf131	chromosome 1 open reading frame 131 [Source:HGNC Symbol;Acc:HGNC:25332]
ENSG00000184787	-0,1001608	5,24800038	0,48782841	0,50207737	0,75113396	UBE2G2	ubiquitin conjugating enzyme E2 G2 [Source:HGNC Symbol;Acc:HGNC:12483]
ENSG00000243156	0,28590759	2,66824746	0,48792235	0,50203724	0,75113396	MICAL3	microtubule associated monooxygenase, calponin and LIM domain containing 3 [Source:HGNC Symbol;Acc:HGNC
ENSG00000137274	-0,1249353	4,30190758	0,48660633	0,50259999	0,75161052	BPHL	biphenyl hydrolase like [Source:HGNC Symbol;Acc:HGNC:1094]

ENSG00000159761	-0,2166935	0,74569053	0,48666958	0,50257291	0,75161052	C16orf86	chromosome 16 open reading frame 86 [Source:HGNC Symbol;Acc:HGNC:33755]
ENSG00000235026	-0,1732257	1,05248522	0,486839	0,50250041	0,75161052	DPP10-AS1	DPP10 antisense RNA 1 [Source:HGNC Symbol;Acc:HGNC:40941]
ENSG00000112245	-0,2008261	0,34460007	0,4855746	0,50304197	0,75219449	PTP4A1	protein tyrosine phosphatase 4A1 [Source:HGNC Symbol;Acc:HGNC:9634]
ENSG00000121903	-0,1324403	1,84940587	0,48465233	0,50343767	0,75259378	ZSCAN20	zinc finger and SCAN domain containing 20 [Source:HGNC Symbol;Acc:HGNC:13093]
ENSG00000156831	-0,1145954	4,38702035	0,48460106	0,50345968	0,75259378	NSMCE2	NSE2 (MMS21) homolog, SMC5-SMC6 complex SUMO ligase [Source:HGNC Symbol;Acc:HGNC:26513]
ENSG00000184895	-0,1639214	1,47066297	0,48459205	0,50346355	0,75259378	SRY	sex determining region Y [Source:HGNC Symbol;Acc:HGNC:11311]
ENSG00000243678	0,26165775	-0,2175937	0,48433193	0,50357527	0,75268376	NME2	NME/NM23 nucleoside diphosphate kinase 2 [Source:HGNC Symbol;Acc:HGNC:7850]
ENSG00000101150	0,09424708	7,85550545	0,48370007	0,50384683	0,75286312	TPD52L2	TPD52 like 2 [Source:HGNC Symbol;Acc:HGNC:12007]
ENSG00000118507	0,12091087	3,22238708	0,48321379	0,504056	0,75286312	AKAP7	A-kinase anchoring protein 7 [Source:HGNC Symbol;Acc:HGNC:377]
ENSG00000129480	0,12596408	3,51723189	0,48356324	0,50390567	0,75286312	DTD2	D-aminoacyl-tRNA deacylase 2 [Source:HGNC Symbol;Acc:HGNC:20277]
ENSG00000133460	0,14510205	2,16926354	0,48327132	0,50403125	0,75286312	SLC2A11	solute carrier family 2 member 11 [Source:HGNC Symbol;Acc:HGNC:14239]
ENSG00000138175	-0,1093297	4,63468046	0,48328586	0,50402499	0,75286312	ARL3	ADP ribosylation factor like GTPase 3 [Source:HGNC Symbol;Acc:HGNC:694]
ENSG00000143387	-0,3834409	-1,6233422	0,48350452	0,50393092	0,75286312	CTSK	cathepsin K [Source:HGNC Symbol;Acc:HGNC:2536]
ENSG00000149262	0,11332079	3,97141387	0,48346796	0,50394665	0,75286312	INTS4	integrator complex subunit 4 [Source:HGNC Symbol;Acc:HGNC:25048]
ENSG00000084636	0,19589764	1,50222817	0,48285736	0,50420943	0,75293831	COL16A1	collagen type XVI alpha 1 chain [Source:HGNC Symbol;Acc:HGNC:2193]
ENSG00000205746	-0,401983	-0,9536266	0,4828739	0,5042023	0,75293831	PKD1P4	polycystin 1, transient receptor potential channel interacting pseudogene 4 [Source:HGNC Symbol;Acc:HGNC:300
ENSG00000023191	-0,1098848	7,16575434	0,48229432	0,50445195	0,7529644	RNH1	ribonuclease/angiogenin inhibitor 1 [Source:HGNC Symbol;Acc:HGNC:10074]
ENSG00000107140	0,1066402	3,68434357	0,48237351	0,50441783	0,7529644	TESK1	testis associated actin remodelling kinase 1 [Source:HGNC Symbol;Acc:HGNC:11731]
ENSG00000163947	0,10380989	5,65460765	0,48221856	0,5044846	0,7529644	ARHGEF3	Rho guanine nucleotide exchange factor 3 [Source:HGNC Symbol;Acc:HGNC:683]
ENSG00000228861	-0,3976449	-1,7110763	0,4824121	0,5044012	0,7529644	RPL23AP12	ribosomal protein L23a pseudogene 12 [Source:HGNC Symbol;Acc:HGNC:23840]
ENSG00000229337	-0,3118132	-1,0874353	0,48237	0,50441934	0,7529644	AC079305.2	novel transcript
ENSG00000065150	0,09730357	7,98642108	0,48124724	0,50490355	0,75297999	IPO5	importin 5 [Source:HGNC Symbol;Acc:HGNC:6402]
ENSG00000100321	0,5647241	-1,306629	0,48148941	0,50479904	0,75297999	SYNGR1	synaptogyrin 1 [Source:HGNC Symbol;Acc:HGNC:11498]
ENSG00000109066	-0,1322179	4,51343202	0,48164091	0,50473367	0,75297999	TMEM104	transmembrane protein 104 [Source:HGNC Symbol;Acc:HGNC:25984]
ENSG00000122566	-0,0996093	10,197012	0,48123835	0,50490739	0,75297999	HNRNPA2B1	heterogeneous nuclear ribonucleoprotein A2/B1 [Source:HGNC Symbol;Acc:HGNC:5033]
ENSG00000135723	0,10839235	4,84588497	0,48171727	0,50470073	0,75297999	FHOD1	formin homology 2 domain containing 1 [Source:HGNC Symbol;Acc:HGNC:17905]
ENSG00000160796	-0,1280381	3,1826668	0,48126543	0,50489569	0,75297999	NBEAL2	neurobeachin like 2 [Source:HGNC Symbol;Acc:HGNC:31928]
ENSG00000162222	-0,1317278	4,05717983	0,48152641	0,50478307	0,75297999	TTC9C	tetratricopeptide repeat domain 9C [Source:HGNC Symbol;Acc:HGNC:28432]
ENSG00000162885	0,10043272	4,85812781	0,48168313	0,50471546	0,75297999	B3GALNT2	beta-1,3-N-acetylgalactosaminyltransferase 2 [Source:HGNC Symbol;Acc:HGNC:28596]
ENSG00000166669	0,12680371	4,57570516	0,48054484	0,5052069	0,753196	ATF7IP2	activating transcription factor 7 interacting protein 2 [Source:HGNC Symbol;Acc:HGNC:20397]
ENSG00000214087	0,10371281	4,31810837	0,48059535	0,50518508	0,753196	ARL16	ADP ribosylation factor like GTPase 16 [Source:HGNC Symbol;Acc:HGNC:27902]
ENSG00000270673	-0,4410745	-0,4932102	0,48066123	0,50515661	0,753196	YTHDF3-AS1	YTHDF3 antisense RNA 1 (head to head) [Source:HGNC Symbol;Acc:HGNC:48728]
ENSG00000178585	0,10038312	6,48968273	0,48019377	0,50535864	0,75326849	CTNNBIP1	catenin beta interacting protein 1 [Source:HGNC Symbol;Acc:HGNC:16913]
ENSG00000234155	-0,3225327	-1,4908434	0,4802991	0,50531311	0,75326849	LINC02535	long intergenic non-protein coding RNA 2535 [Source:HGNC Symbol;Acc:HGNC:53569]
ENSG00000104774	0,14168884	5,64900848	0,47969387	0,50557486	0,75336018	MAN2B1	mannosidase alpha class 2B member 1 [Source:HGNC Symbol;Acc:HGNC:6826]
ENSG00000124172	-0,1282473	9,17316209	0,47992959	0,50547289	0,75336018	ATP5F1E	ATP synthase F1 subunit epsilon [Source:HGNC Symbol;Acc:HGNC:838]
ENSG00000148773	0,26606013	6,10082857	0,47980462	0,50552695	0,75336018	MKI67	marker of proliferation Ki-67 [Source:HGNC Symbol;Acc:HGNC:7107]
ENSG00000121310	0,10736019	3,62918653	0,47905378	0,50585197	0,75340284	ECHDC2	enoyl-CoA hydratase domain containing 2 [Source:HGNC Symbol;Acc:HGNC:23408]
ENSG00000152422	0,12994333	3,24495407	0,47903211	0,50586135	0,75340284	XRCC4	X-ray repair cross complementing 4 [Source:HGNC Symbol;Acc:HGNC:12831]
ENSG00000177666	0,10865924	6,63364882	0,4790986	0,50583256	0,75340284	PNPLA2	patatin like phospholipase domain containing 2 [Source:HGNC Symbol;Acc:HGNC:30802]
ENSG00000258940	-0,3301448	-0,6377699	0,47916451	0,50580401	0,75340284	AL132639.2	novel transcript, antisense to CTAGE5
ENSG00000269609	-0,1829485	2,38675518	0,479195	0,50579081	0,75340284	RPARP-AS1	RPARP antisense RNA 1 [Source:HGNC Symbol;Acc:HGNC:45238]
ENSG00000196199	-0,1262027	3,63621497	0,47888186	0,50592644	0,75342297	MPHOSPH8	M-phase phosphoprotein 8 [Source:HGNC Symbol;Acc:HGNC:29810]
ENSG00000042088	0,10260595	4,75169122	0,47817586	0,50623248	0,75380189	TDP1	tyrosyl-DNA phosphodiesterase 1 [Source:HGNC Symbol;Acc:HGNC:18884]

ENSG00000058262	0,11017484	8,90531255	0,47738605	0,50657526	0,75400489	SEC61A1	SEC61 translocon subunit alpha 1 [Source:HGNC Symbol;Acc:HGNC:18276]
ENSG00000197885	0,15123859	2,76968592	0,47748404	0,50653271	0,75400489	NKIRAS1	NFKB inhibitor interacting Ras like 1 [Source:HGNC Symbol;Acc:HGNC:17899]
ENSG00000217555	0,10867304	3,22842825	0,47745765	0,50654417	0,75400489	CKLF	chemokine like factor [Source:HGNC Symbol;Acc:HGNC:13253]
ENSG00000259972	-0,248586	0,00936639	0,47766711	0,50645323	0,75400489	AC009120.2	novel transcript, antisense to PSMD7
ENSG00000139826	0,12780101	2,9666244	0,47661642	0,50690969	0,75436211	ABHD13	abhydrolase domain containing 13 [Source:HGNC Symbol;Acc:HGNC:20293]
ENSG00000149257	0,14156189	8,70435958	0,47659607	0,50691853	0,75436211	SERPINH1	serpin family H member 1 [Source:HGNC Symbol;Acc:HGNC:1546]
ENSG00000121552	0,13114831	1,96564977	0,47630178	0,50704652	0,7544183	CSTA	cystatin A [Source:HGNC Symbol;Acc:HGNC:2481]
ENSG00000286129	-0,3485833	-1,6887414	0,47627178	0,50705958	0,7544183	AC245060.7	novel transcript
ENSG00000125898	-0,2028524	1,93191183	0,47588289	0,50722881	0,75451641	FAM110A	family with sequence similarity 110 member A [Source:HGNC Symbol;Acc:HGNC:16188]
ENSG00000158863	0,13469965	3,67416781	0,47598345	0,50718504	0,75451641	FAM160B2	family with sequence similarity 160 member B2 [Source:HGNC Symbol;Acc:HGNC:16492]
ENSG00000110075	0,11467025	4,94338154	0,47574022	0,50729091	0,75451739	PPP6R3	protein phosphatase 6 regulatory subunit 3 [Source:HGNC Symbol;Acc:HGNC:1173]
ENSG00000188375	-0,214368	0,05087134	0,47564412	0,50733276	0,75451739	H3-5	H3.5 histone [Source:HGNC Symbol;Acc:HGNC:33164]
ENSG00000101882	-0,107148	4,72388426	0,47533732	0,50746639	0,75453499	NKAP	NFKB activating protein [Source:HGNC Symbol;Acc:HGNC:29873]
ENSG00000168395	0,10083731	5,78581633	0,47530437	0,50748075	0,75453499	ING5	inhibitor of growth family member 5 [Source:HGNC Symbol;Acc:HGNC:19421]
ENSG00000173692	0,11534355	6,91032977	0,47526123	0,50749955	0,75453499	PSMD1	proteasome 26S subunit, non-ATPase 1 [Source:HGNC Symbol;Acc:HGNC:9554]
ENSG00000050555	-0,2688168	-0,3782952	0,47415487	0,50798203	0,75509865	LAMC3	laminin subunit gamma 3 [Source:HGNC Symbol;Acc:HGNC:6494]
ENSG00000057019	-0,1155291	5,05183747	0,47426124	0,50793561	0,75509865	DCBLD2	discoidin, CUB and LCCL domain containing 2 [Source:HGNC Symbol;Acc:HGNC:24627]
ENSG00000213064	0,16264342	3,89728355	0,47373296	0,50816625	0,75529564	SFT2D2	SFT2 domain containing 2 [Source:HGNC Symbol;Acc:HGNC:25140]
ENSG00000125730	-0,1975827	10,264197	0,47358515	0,50823082	0,75531476	C3	complement C3 [Source:HGNC Symbol;Acc:HGNC:1318]
ENSG00000164603	-0,1535274	1,19744531	0,47292418	0,50851974	0,75566726	BMT2	base methyltransferase of 25S rRNA 2 homolog [Source:HGNC Symbol;Acc:HGNC:26475]
ENSG00000174231	0,16594299	7,73245002	0,47203107	0,50891061	0,75617118	PRPF8	pre-mRNA processing factor 8 [Source:HGNC Symbol;Acc:HGNC:17340]
ENSG00000255397	-0,2303083	-0,3574486	0,4713448	0,50921133	0,75654106	NASPP1	nuclear autoantigenic sperm protein pseudogene 1 [Source:HGNC Symbol;Acc:HGNC:29910]
ENSG00000020129	-0,1254536	3,71612746	0,470411	0,50962104	0,7568419	NCDN	neurochondrin [Source:HGNC Symbol;Acc:HGNC:17597]
ENSG00000033867	0,23517047	2,82591176	0,47046937	0,50959541	0,7568419	SLC4A7	solute carrier family 4 member 7 [Source:HGNC Symbol;Acc:HGNC:11033]
ENSG00000113407	-0,1333616	7,51076029	0,47060124	0,50953752	0,7568419	TARS1	threonyl-tRNA synthetase 1 [Source:HGNC Symbol;Acc:HGNC:11572]
ENSG00000233859	0,15768522	0,86996051	0,47050192	0,50958112	0,7568419	ADH5P4	ADH5 pseudogene 4 [Source:HGNC Symbol;Acc:HGNC:21377]
ENSG00000143158	0,12254894	5,49424202	0,46955829	0,50999571	0,75694161	MPC2	mitochondrial pyruvate carrier 2 [Source:HGNC Symbol;Acc:HGNC:24515]
ENSG00000156136	-0,1449206	2,75005192	0,46986565	0,5098606	0,75694161	DCK	deoxycytidine kinase [Source:HGNC Symbol;Acc:HGNC:2704]
ENSG00000160445	0,10216803	3,76015807	0,46993054	0,50983208	0,75694161	ZER1	zyg-11 related cell cycle regulator [Source:HGNC Symbol;Acc:HGNC:30960]
ENSG00000161558	-0,1223811	3,10889938	0,46983638	0,50987346	0,75694161	TMEM143	transmembrane protein 143 [Source:HGNC Symbol;Acc:HGNC:25603]
ENSG00000173715	0,12401989	2,8578798	0,46955066	0,50999906	0,75694161	C11orf80	chromosome 11 open reading frame 80 [Source:HGNC Symbol;Acc:HGNC:26197]
ENSG00000272140	0,36595116	-1,85708	0,46972625	0,50992187	0,75694161	AC022400.4	novel transcript
ENSG00000128563	-0,1090054	4,44764687	0,46919884	0,5101538	0,75709436	PRKRIP1	PRKR interacting protein 1 [Source:HGNC Symbol;Acc:HGNC:21894]
ENSG00000123080	-0,1551753	3,15680575	0,46906863	0,51021109	0,75710247	CDKN2C	cyclin dependent kinase inhibitor 2C [Source:HGNC Symbol;Acc:HGNC:1789]
ENSG00000035687	0,11354508	4,76639498	0,46850266	0,51046024	0,75725339	ADSS2	adenylosuccinate synthase 2 [Source:HGNC Symbol;Acc:HGNC:292]
ENSG00000100596	0,1117864	5,43079847	0,46836668	0,51052013	0,75725339	SPTLC2	serine palmitoyltransferase long chain base subunit 2 [Source:HGNC Symbol;Acc:HGNC:11278]
ENSG00000212694	-0,163432	1,05502844	0,46845907	0,51047944	0,75725339	LINC01089	long intergenic non-protein coding RNA 1089 [Source:HGNC Symbol;Acc:HGNC:27886]
ENSG00000229887	-0,5757355	-1,6052323	0,46849111	0,51046533	0,75725339	HNRNPA1P6	heterogeneous nuclear ribonucleoprotein A1 pseudogene 6 [Source:HGNC Symbol;Acc:HGNC:5032]
ENSG00000106333	-0,1096314	4,82593378	0,46799999	0,51068172	0,75733928	PCOLCE	procollagen C-endopeptidase enhancer [Source:HGNC Symbol;Acc:HGNC:8738]
ENSG00000183979	-0,2891662	-0,9088371	0,46811603	0,51063057	0,75733928	NPB	neuropeptide B [Source:HGNC Symbol;Acc:HGNC:30099]
ENSG00000111679	0,10566004	3,71826657	0,46785269	0,51074665	0,75735869	PTPN6	protein tyrosine phosphatase non-receptor type 6 [Source:HGNC Symbol;Acc:HGNC:9658]
ENSG00000235669	0,42760115	-1,6411615	0,46698016	0,5111316	0,75785259	CHN2-AS1	CHN2 antisense RNA 1 [Source:HGNC Symbol;Acc:HGNC:40149]
ENSG00000122512	0,11956594	3,02521152	0,4665055	0,51134124	0,7579717	PMS2	PMS1 homolog 2, mismatch repair system component [Source:HGNC Symbol;Acc:HGNC:9122]
ENSG00000149639	0,28932588	4,44176006	0,46632846	0,51141947	0,7579717	SOGA1	suppressor of glucose, autophagy associated 1 [Source:HGNC Symbol;Acc:HGNC:16111]

ENSG00000189046	-0,1244909	4,40955895	0,46663626	0,51128347	0,7579717	ALKBH2	alkB homolog 2, alpha-ketoglutarate dependent dioxygenase [Source:HGNC Symbol;Acc:HGNC:32487]
ENSG00000269190	-0,1156134	3,5984808	0,46640866	0,51138403	0,7579717	FBXO17	F-box protein 17 [Source:HGNC Symbol;Acc:HGNC:18754]
ENSG00000023697	0,09722171	6,10610456	0,46580382	0,51165144	0,75800789	DERA	deoxyribose-phosphate aldolase [Source:HGNC Symbol;Acc:HGNC:24269]
ENSG00000139800	0,29932879	-0,7802414	0,46590187	0,51160807	0,75800789	ZIC5	Zic family member 5 [Source:HGNC Symbol;Acc:HGNC:20322]
ENSG00000168228	-0,1042627	3,67503633	0,46582979	0,51163995	0,75800789	ZCCHC4	zinc finger CCHC-type containing 4 [Source:HGNC Symbol;Acc:HGNC:22917]
ENSG00000224189	-0,1214003	4,1331369	0,46597797	0,51157441	0,75800789	HAGLR	HOXD antisense growth-associated long non-coding RNA [Source:HGNC Symbol;Acc:HGNC:43755]
ENSG00000185344	0,14613947	3,99266304	0,46555466	0,51176167	0,75801746	ATP6V0A2	ATPase H+ transporting V0 subunit a2 [Source:HGNC Symbol;Acc:HGNC:18481]
ENSG00000229251	0,27738761	-1,1032483	0,46564614	0,51172119	0,75801746	HNRNPA1P8	heterogeneous nuclear ribonucleoprotein A1 pseudogene 8 [Source:HGNC Symbol;Acc:HGNC:39126]
ENSG00000259032	-0,3860541	-1,7439187	0,46539334	0,51183306	0,75804635	ENSAP2	endosulfine alpha pseudogene 2 [Source:HGNC Symbol;Acc:HGNC:20011]
ENSG00000013561	-0,1136	3,90275045	0,46506967	0,51197636	0,75813696	RNF14	ring finger protein 14 [Source:HGNC Symbol;Acc:HGNC:10058]
ENSG00000261188	-0,1564267	0,91021631	0,46502073	0,51199803	0,75813696	Z95115.1	novel transcript, antisense to TFIP11
ENSG00000156482	-0,1057201	9,46551499	0,46456218	0,51220119	0,75836092	RPL30	ribosomal protein L30 [Source:HGNC Symbol;Acc:HGNC:10333]
ENSG00000038382	-0,1450196	4,59090536	0,46420328	0,5123603	0,75838592	TRIO	trio Rho guanine nucleotide exchange factor [Source:HGNC Symbol;Acc:HGNC:12303]
ENSG00000120370	0,13118293	2,78277212	0,46427742	0,51232743	0,75838592	GORAB	golgin, RAB6 interacting [Source:HGNC Symbol;Acc:HGNC:25676]
ENSG00000154229	-0,1618947	5,82065271	0,46378324	0,51254664	0,75838592	PRKCA	protein kinase C alpha [Source:HGNC Symbol;Acc:HGNC:9393]
ENSG00000167778	0,09981167	4,22674852	0,4637378	0,51256681	0,75838592	SPRYD3	SPRY domain containing 3 [Source:HGNC Symbol;Acc:HGNC:25920]
ENSG00000178226	-0,2344167	0,26879363	0,46377335	0,51255103	0,75838592	PRSS36	serine protease 36 [Source:HGNC Symbol;Acc:HGNC:26906]
ENSG00000205133	-0,1524712	2,32719085	0,46358782	0,51263337	0,75838592	TRIQK	triple QxxK/R motif containing [Source:HGNC Symbol;Acc:HGNC:27828]
ENSG00000227376	0,1740776	0,78819521	0,46365001	0,51260576	0,75838592	FTH1P16	ferritin heavy chain 1 pseudogene 16 [Source:HGNC Symbol;Acc:HGNC:3986]
ENSG00000242125	0,10481537	5,50740904	0,46385667	0,51251406	0,75838592	SNHG3	small nucleolar RNA host gene 3 [Source:HGNC Symbol;Acc:HGNC:10118]
ENSG00000135698	0,10318537	5,51477323	0,46286285	0,51295537	0,75849598	MPHOSPH6	M-phase phosphoprotein 6 [Source:HGNC Symbol;Acc:HGNC:7214]
ENSG00000162636	0,30854624	-0,7889593	0,46291631	0,51293162	0,75849598	FAM102B	family with sequence similarity 102 member B [Source:HGNC Symbol;Acc:HGNC:27637]
ENSG00000167615	0,23760938	3,75044047	0,46283586	0,51296737	0,75849598	LENG8	leukocyte receptor cluster member 8 [Source:HGNC Symbol;Acc:HGNC:15500]
ENSG00000237758	-0,2926902	-0,7346197	0,46289722	0,5129401	0,75849598	BANF1P3	BANF1 pseudogene 3 [Source:HGNC Symbol;Acc:HGNC:43883]
ENSG00000288075	0,2734537	-0,4811438	0,46297619	0,51290501	0,75849598	AC108519.1	novel transcript
ENSG00000131773	0,09910131	4,61682094	0,46246364	0,51313285	0,75866388	KHDRBS3	KH RNA binding domain containing, signal transduction associated 3 [Source:HGNC Symbol;Acc:HGNC:18117]
ENSG00000229994	-0,2003582	0,06004895	0,46230407	0,51320382	0,75869202	RPL5P4	ribosomal protein L5 pseudogene 4 [Source:HGNC Symbol;Acc:HGNC:36095]
ENSG00000243449	-0,165758	0,85129808	0,46198762	0,51334461	0,75882338	C4orf48	chromosome 4 open reading frame 48 [Source:HGNC Symbol;Acc:HGNC:34437]
ENSG00000167972	0,28464581	-0,5911067	0,46143959	0,51358862	0,7590918	ABCA3	ATP binding cassette subfamily A member 3 [Source:HGNC Symbol;Acc:HGNC:33]
ENSG00000197965	0,09498105	6,579724	0,46134643	0,51363012	0,7590918	MPZL1	myelin protein zero like 1 [Source:HGNC Symbol;Acc:HGNC:7226]
ENSG00000184162	-0,1204612	4,52534882	0,4611035	0,51373837	0,75916439	NR2C2AP	nuclear receptor 2C2 associated protein [Source:HGNC Symbol;Acc:HGNC:30763]
ENSG00000184575	0,08767034	6,64088462	0,46100299	0,51378317	0,75916439	XPOT	exportin for tRNA [Source:HGNC Symbol;Acc:HGNC:12826]
ENSG00000142599	-0,2560583	4,66016523	0,46084386	0,51385411	0,75919242	RERE	arginine-glutamic acid dipeptide repeats [Source:HGNC Symbol;Acc:HGNC:9965]
ENSG00000155827	0,17170376	5,08026284	0,46045287	0,51402849	0,75921972	RNF20	ring finger protein 20 [Source:HGNC Symbol;Acc:HGNC:10062]
ENSG00000254837	-0,3512774	-0,290096	0,46046669	0,51402233	0,75921972	AP001372.2	uncharacterized LOC100287896 [Source:NCBI gene (formerly Entrezgene);Acc:100287896]
ENSG00000280798	-0,2470076	-0,4215311	0,46060839	0,51395912	0,75921972	LINC00294	long intergenic non-protein coding RNA 294 [Source:HGNC Symbol;Acc:HGNC:27456]
ENSG00000274925	0,21105892	0,28268777	0,46020966	0,51413702	0,75930325	ZKSCAN2-DT	ZKSCAN2 divergent transcript [Source:HGNC Symbol;Acc:HGNC:53912]
ENSG00000058272	-0,2296187	3,74876151	0,4597315	0,51435052	0,759465	PPP1R12A	protein phosphatase 1 regulatory subunit 12A [Source:HGNC Symbol;Acc:HGNC:7618]
ENSG00000106025	0,46799028	-1,4952097	0,45983837	0,51430279	0,759465	TSPAN12	tetraspanin 12 [Source:HGNC Symbol;Acc:HGNC:21641]
ENSG00000100348	0,09104898	7,17809509	0,45950874	0,51445003	0,75953517	TXN2	thioredoxin 2 [Source:HGNC Symbol;Acc:HGNC:17772]
ENSG00000185527	-0,2711534	-0,3123691	0,45891213	0,51471674	0,75985215	PDE6G	phosphodiesterase 6G [Source:HGNC Symbol;Acc:HGNC:8789]
ENSG00000186111	-0,1074534	4,36925081	0,45849446	0,51490361	0,75986838	PIP5K1C	phosphatidylinositol-4-phosphate 5-kinase type 1 gamma [Source:HGNC Symbol;Acc:HGNC:8996]
ENSG00000254473	0,25038639	-0,4432373	0,45846844	0,51491526	0,75986838	AL354920.1	novel transcript, antisense to UBQLN1
ENSG00000256745	-0,2015623	0,02880024	0,45842258	0,51493579	0,75986838	ARPC3P3	actin related protein 2/3 complex subunit 3 pseudogene 3 [Source:HGNC Symbol;Acc:HGNC:39432]

ENSG00000286650	0,5618671	-2,0097023	0,45851844	0,51489288	0,75986838	AL139234.1	novel transcript
ENSG00000035499	0,09011354	5,85994966	0,45793291	0,51515506	0,76011516	DEPDC1B	DEP domain containing 1B [Source:HGNC Symbol;Acc:HGNC:24902]
ENSG00000270872	-0,3909797	-1,6221508	0,45771087	0,51525454	0,76018517	SRGAP2D	SLIT-ROBO Rho GTPase activating protein 2D (pseudogene) [Source:HGNC Symbol;Acc:HGNC:43932]
ENSG00000186481	-0,2086046	0,69328088	0,45681841	0,51565476	0,76069882	ANKRD20A5P	ankyrin repeat domain 20 family member A5, pseudogene [Source:HGNC Symbol;Acc:HGNC:33833]
ENSG00000257489	0,38326046	-1,7522537	0,45661896	0,51574429	0,76075407	AC010203.1	TBC1 domain family, member 2B (TBC1D2B) pseudogene
ENSG00000269374	-0,2074358	0,79006838	0,45629934	0,51588781	0,76088895	AC011497.2	zinc finger, AN1-type domain 5 (ZFAND5) pseudogene
ENSG00000066855	0,15499754	5,1099024	0,45597301	0,51603442	0,760914	MTFR1	mitochondrial fission regulator 1 [Source:HGNC Symbol;Acc:HGNC:29510]
ENSG00000091542	-0,1264961	4,88503808	0,45594902	0,5160452	0,760914	ALKBH5	alkB homolog 5, RNA demethylase [Source:HGNC Symbol;Acc:HGNC:25996]
ENSG00000140025	-0,1162544	3,35860805	0,45579788	0,51611313	0,760914	EFCAB11	EF-hand calcium binding domain 11 [Source:HGNC Symbol;Acc:HGNC:20357]
ENSG00000156017	-0,1223558	3,05597391	0,45585703	0,51608655	0,760914	CARNMT1	carnosine N-methyltransferase 1 [Source:HGNC Symbol;Acc:HGNC:23435]
ENSG00000181830	-0,1236151	4,63179908	0,45528448	0,51634402	0,76096705	SLC35C1	solute carrier family 35 member C1 [Source:HGNC Symbol;Acc:HGNC:20197]
ENSG00000228409	0,11628434	2,66261467	0,45525456	0,51635748	0,76096705	CCT6P1	chaperonin containing TCP1 subunit 6 pseudogene 1 [Source:HGNC Symbol;Acc:HGNC:33094]
ENSG00000237854	0,2040576	0,46807021	0,45537705	0,51630237	0,76096705	LINC00674	long intergenic non-protein coding RNA 674 [Source:HGNC Symbol;Acc:HGNC:44355]
ENSG00000271072	-0,349425	-1,3785319	0,45536714	0,51630683	0,76096705	PHBP20	prohibitin pseudogene 20 [Source:HGNC Symbol;Acc:HGNC:51553]
ENSG00000164038	-0,0979419	4,6802993	0,45459643	0,51665374	0,76132686	SLC9B2	solute carrier family 9 member B2 [Source:HGNC Symbol;Acc:HGNC:25143]
ENSG00000089639	-0,1477735	1,45618717	0,45394836	0,51694579	0,76142816	GMIP	GEM interacting protein [Source:HGNC Symbol;Acc:HGNC:24852]
ENSG00000106070	0,10565595	3,97184558	0,45379434	0,51701524	0,76142816	GRB10	growth factor receptor bound protein 10 [Source:HGNC Symbol;Acc:HGNC:4564]
ENSG00000120948	0,08985662	7,60172694	0,45392157	0,51695787	0,76142816	TARDBP	TAR DNA binding protein [Source:HGNC Symbol;Acc:HGNC:11571]
ENSG00000143653	0,08970487	7,21491883	0,45375006	0,51703521	0,76142816	SCCPDH	saccharopine dehydrogenase (putative) [Source:HGNC Symbol;Acc:HGNC:24275]
ENSG00000187486	-0,3043669	-0,1905325	0,45415508	0,5168526	0,76142816	KCNJ11	potassium inwardly rectifying channel subfamily J member 11 [Source:HGNC Symbol;Acc:HGNC:6257]
ENSG00000197779	0,25942101	-0,5749697	0,45415435	0,51685293	0,76142816	ZNF81	zinc finger protein 81 [Source:HGNC Symbol;Acc:HGNC:13156]
ENSG00000188010	0,15630931	3,66718624	0,45345164	0,51716984	0,76154966	MORN2	MORN repeat containing 2 [Source:HGNC Symbol;Acc:HGNC:30166]
ENSG00000078369	-0,1117997	7,86260191	0,45294418	0,51739892	0,76180387	GNB1	G protein subunit beta 1 [Source:HGNC Symbol;Acc:HGNC:4396]
ENSG00000101544	0,10124999	5,0255458	0,45277198	0,5174767	0,76180387	ADNP2	ADNP homeobox 2 [Source:HGNC Symbol;Acc:HGNC:23803]
ENSG00000152102	0,08984926	5,88411932	0,45260741	0,51755106	0,76180387	FAM168B	family with sequence similarity 168 member B [Source:HGNC Symbol;Acc:HGNC:27016]
ENSG00000175497	0,14789472	1,7572126	0,45261851	0,51754604	0,76180387	DPP10	dipeptidyl peptidase like 10 [Source:HGNC Symbol;Acc:HGNC:20823]
ENSG00000185127	-0,1229737	5,24819199	0,45150535	0,51804949	0,7624607	C6orf120	chromosome 6 open reading frame 120 [Source:HGNC Symbol;Acc:HGNC:21247]
ENSG00000063177	-0,0971982	10,3026337	0,4511307	0,51821913	0,76263356	RPL18	ribosomal protein L18 [Source:HGNC Symbol;Acc:HGNC:10310]
ENSG00000162009	0,47347378	-0,7872319	0,45038507	0,51855707	0,76305402	SSTR5	somatostatin receptor 5 [Source:HGNC Symbol;Acc:HGNC:11334]
ENSG00000104689	0,12848739	4,46410854	0,450174	0,51865281	0,76311803	TNFRSF10A	TNF receptor superfamily member 10a [Source:HGNC Symbol;Acc:HGNC:11904]
ENSG00000120616	0,10150411	3,50731297	0,44950451	0,51895671	0,76348827	EPC1	enhancer of polycomb homolog 1 [Source:HGNC Symbol;Acc:HGNC:19876]
ENSG00000147394	0,20627678	1,32302036	0,44926379	0,51906606	0,76349537	ZNF185	zinc finger protein 185 with LIM domain [Source:HGNC Symbol;Acc:HGNC:12976]
ENSG00000243716	0,46370138	1,20116653	0,44936807	0,51901868	0,76349537	NPIPB5	nuclear pore complex interacting protein family member B5 [Source:HGNC Symbol;Acc:HGNC:37233]
ENSG00000162430	-0,1422383	4,16399411	0,44887769	0,51924153	0,76367659	SELENON	selenoprotein N [Source:HGNC Symbol;Acc:HGNC:15999]
ENSG00000214174	-0,1388189	2,27564309	0,44856759	0,51938255	0,76380709	AMZ2P1	archaelysin family metallopeptidase 2 pseudogene 1 [Source:HGNC Symbol;Acc:HGNC:26491]
ENSG00000059377	0,22176874	-0,1758719	0,44780944	0,51972762	0,76383869	TBXAS1	thromboxane A synthase 1 [Source:HGNC Symbol;Acc:HGNC:11609]
ENSG00000143420	0,10679738	6,40447547	0,44750009	0,51986854	0,76383869	ENSA	endosulfine alpha [Source:HGNC Symbol;Acc:HGNC:3360]
ENSG00000147684	0,08655102	8,9884694	0,44764013	0,51980474	0,76383869	NDUFB9	NADH:ubiquinone oxidoreductase subunit B9 [Source:HGNC Symbol;Acc:HGNC:7704]
ENSG00000158691	0,11731269	3,55663312	0,44777416	0,51974369	0,76383869	ZSCAN12	zinc finger and SCAN domain containing 12 [Source:HGNC Symbol;Acc:HGNC:13172]
ENSG00000172301	0,11476824	6,32669396	0,44770297	0,51977611	0,76383869	COPRS	coordinator of PRMT5 and differentiation stimulator [Source:HGNC Symbol;Acc:HGNC:28848]
ENSG00000180346	0,14767779	2,67422407	0,44815325	0,51957108	0,76383869	TIGD2	tigger transposable element derived 2 [Source:HGNC Symbol;Acc:HGNC:18333]
ENSG00000181873	-0,1131229	3,95717784	0,44838898	0,51946381	0,76383869	IBA57	iron-sulfur cluster assembly factor IBA57 [Source:HGNC Symbol;Acc:HGNC:27302]
ENSG00000188739	-0,2604743	-0,7835726	0,44766014	0,51979563	0,76383869	RBM34	RNA binding motif protein 34 [Source:HGNC Symbol;Acc:HGNC:28965]
ENSG00000268034	0,27391948	-0,7374762	0,44748679	0,5198746	0,76383869	AC243960.2	tropomyosin pseudogene

ENSG00000184584	-0,1453497	2,20601236	0,44728834	0,51996505	0,76389475	STING1	stimulator of interferon response cGAMP interactor 1 [Source:HGNC Symbol;Acc:HGNC:27962]
ENSG00000000419	0,10106356	7,66370159	0,44656568	0,52029465	0,76396106	DPM1	dolichyl-phosphate mannosyltransferase subunit 1, catalytic [Source:HGNC Symbol;Acc:HGNC:3005]
ENSG00000057294	-0,1214566	5,13174363	0,44704651	0,5200753	0,76396106	PKP2	plakophilin 2 [Source:HGNC Symbol;Acc:HGNC:9024]
ENSG00000057663	0,12140396	5,12151869	0,44638691	0,52037624	0,76396106	ATG5	autophagy related 5 [Source:HGNC Symbol;Acc:HGNC:589]
ENSG00000078618	-0,1026824	6,51115595	0,44680379	0,520186	0,76396106	NRDC	nardilysin convertase [Source:HGNC Symbol;Acc:HGNC:7995]
ENSG00000189114	-0,1174655	2,56198234	0,44670588	0,52023067	0,76396106	BLOC1S3	biogenesis of lysosomal organelles complex 1 subunit 3 [Source:HGNC Symbol;Acc:HGNC:20914]
ENSG00000197183	0,1087585	4,91108423	0,44689242	0,52014558	0,76396106	NOL4L	nucleolar protein 4 like [Source:HGNC Symbol;Acc:HGNC:16106]
ENSG00000224993	-0,2112244	0,19197216	0,44643815	0,52035285	0,76396106	RPL29P12	ribosomal protein L29 pseudogene 12 [Source:HGNC Symbol;Acc:HGNC:36608]
ENSG00000002016	-0,116507	3,0429494	0,44573146	0,52067562	0,76412725	RAD52	RAD52 homolog, DNA repair protein [Source:HGNC Symbol;Acc:HGNC:9824]
ENSG00000114784	-0,1077522	5,69791079	0,44578915	0,52064926	0,76412725	EIF1B	eukaryotic translation initiation factor 1B [Source:HGNC Symbol;Acc:HGNC:30792]
ENSG00000151729	0,09839777	5,94079665	0,44568103	0,52069866	0,76412725	SLC25A4	solute carrier family 25 member 4 [Source:HGNC Symbol;Acc:HGNC:10990]
ENSG00000169710	-0,2511496	8,8323356	0,44570409	0,52068813	0,76412725	FASN	fatty acid synthase [Source:HGNC Symbol;Acc:HGNC:3594]
ENSG00000206527	0,1057849	5,47077294	0,44494329	0,52103604	0,76454556	HACD2	3-hydroxyacyl-CoA dehydratase 2 [Source:HGNC Symbol;Acc:HGNC:9640]
ENSG00000183808	-0,1293237	3,18148106	0,44444467	0,5212643	0,76480367	RBM12B	RNA binding motif protein 12B [Source:HGNC Symbol;Acc:HGNC:32310]
ENSG00000225405	0,21570482	-0,2353811	0,44384044	0,52154115	0,76513303	RPS15AP17	ribosomal protein S15a pseudogene 17 [Source:HGNC Symbol;Acc:HGNC:36888]
ENSG00000236814	0,24243248	0,06281945	0,44338386	0,52175054	0,76536336	AC046176.1	proteasome (prosome, macropain) 26S subunit, ATPase, 6 (PSMC6) pseudogene
ENSG00000137962	-0,118778	3,60179222	0,44221411	0,52228771	0,76607441	ARHGAP29	Rho GTPase activating protein 29 [Source:HGNC Symbol;Acc:HGNC:30207]
ENSG00000043093	0,11528293	3,64291556	0,44194974	0,52240926	0,76617577	DCUN1D1	defective in cullin neddylation 1 domain containing 1 [Source:HGNC Symbol;Acc:HGNC:18184]
ENSG00000137478	0,09491618	4,91170644	0,44172366	0,52251324	0,76625135	FCHSD2	FCH and double SH3 domains 2 [Source:HGNC Symbol;Acc:HGNC:29114]
ENSG00000228519	-0,1862027	0,83864858	0,44153224	0,52260132	0,76630359	RANBP1P1	RANBP1 pseudogene 1 [Source:HGNC Symbol;Acc:HGNC:54908]
ENSG00000162174	-0,0992592	6,00999026	0,44117251	0,5227669	0,76644027	ASRGL1	asparaginase and isoaspartyl peptidase 1 [Source:HGNC Symbol;Acc:HGNC:16448]
ENSG00000168488	0,15741214	7,11548007	0,44108936	0,5228052	0,76644027	ATXN2L	ataxin 2 like [Source:HGNC Symbol;Acc:HGNC:31326]
ENSG00000205683	-0,2911717	-0,323182	0,4409879	0,52285192	0,76644027	DPF3	double PHD fingers 3 [Source:HGNC Symbol;Acc:HGNC:17427]
ENSG00000136141	0,13278155	4,28104155	0,44083463	0,52292253	0,76646687	LRCH1	leucine rich repeats and calponin homology domain containing 1 [Source:HGNC Symbol;Acc:HGNC:20309]
ENSG00000171858	-0,1146232	9,89069811	0,44066376	0,52300126	0,76650536	RPS21	ribosomal protein S21 [Source:HGNC Symbol;Acc:HGNC:10409]
ENSG00000188856	-0,204916	0,23403371	0,44047839	0,5230867	0,76655368	RPSAP47	ribosomal protein SA pseudogene 47 [Source:HGNC Symbol;Acc:HGNC:36162]
ENSG00000213228	-0,4563887	-1,9407306	0,44009442	0,52326377	0,76673625	RPL12P38	ribosomal protein L12 pseudogene 38 [Source:HGNC Symbol;Acc:HGNC:36838]
ENSG00000215005	0,2663359	-0,3426011	0,43994541	0,52333251	0,76676007	HSPD1P7	heat shock protein family D (Hsp60) member 1 pseudogene 7 [Source:HGNC Symbol;Acc:HGNC:5268]
ENSG00000020426	-0,0937908	4,87011596	0,43970628	0,52344287	0,76681969	MNAT1	MNAT1 component of CDK activating kinase [Source:HGNC Symbol;Acc:HGNC:7181]
ENSG00000197312	-0,1319075	4,3921792	0,43962977	0,52347818	0,76681969	DDI2	DNA damage inducible 1 homolog 2 [Source:HGNC Symbol;Acc:HGNC:24578]
ENSG00000004399	-0,1427018	5,93604806	0,43842374	0,5240355	0,76700684	PLXND1	plexin D1 [Source:HGNC Symbol;Acc:HGNC:9107]
ENSG00000047578	0,12565569	3,53576139	0,43814873	0,52416274	0,76700684	KATNIP	katanin interacting protein [Source:HGNC Symbol;Acc:HGNC:29068]
ENSG00000082196	-0,529961	-1,8374139	0,43810393	0,52418347	0,76700684	C1QTNF3	C1q and TNF related 3 [Source:HGNC Symbol;Acc:HGNC:14326]
ENSG00000090989	0,10828439	2,96247659	0,43902508	0,52375747	0,76700684	EXOC1	exocyst complex component 1 [Source:HGNC Symbol;Acc:HGNC:30380]
ENSG00000102781	-0,1342234	1,94094126	0,43857676	0,52396472	0,76700684	KATNAL1	katanin catalytic subunit A1 like 1 [Source:HGNC Symbol;Acc:HGNC:28361]
ENSG00000105289	-0,1474832	3,62313629	0,4385416	0,52398098	0,76700684	TJP3	tight junction protein 3 [Source:HGNC Symbol;Acc:HGNC:11829]
ENSG00000139218	0,11325673	5,38498229	0,43870162	0,52390699	0,76700684	SCAF11	SR-related CTD associated factor 11 [Source:HGNC Symbol;Acc:HGNC:10784]
ENSG00000140379	-0,5029963	-1,7311556	0,43894812	0,52379304	0,76700684	BCL2A1	BCL2 related protein A1 [Source:HGNC Symbol;Acc:HGNC:991]
ENSG00000179943	0,11435309	2,92915506	0,43873692	0,52389067	0,76700684	FIZ1	FLT3 interacting zinc finger 1 [Source:HGNC Symbol;Acc:HGNC:25917]
ENSG00000225697	0,11676412	5,99035775	0,43815887	0,52415804	0,76700684	SLC26A6	solute carrier family 26 member 6 [Source:HGNC Symbol;Acc:HGNC:14472]
ENSG00000270170	-0,0957518	5,99691507	0,4391996	0,52367684	0,76700684	NCBP2AS2	NCBP2 antisense 2 (head to head) [Source:HGNC Symbol;Acc:HGNC:25121]
ENSG00000118640	0,10042204	6,13028436	0,43787409	0,52428987	0,76708569	VAMP8	vesicle associated membrane protein 8 [Source:HGNC Symbol;Acc:HGNC:12647]
ENSG00000181045	0,10808056	4,38469414	0,43745166	0,52448552	0,76729511	SLC26A11	solute carrier family 26 member 11 [Source:HGNC Symbol;Acc:HGNC:14471]
ENSG00000072310	0,13141722	6,40715446	0,43691681	0,52473344	0,76737912	SREBF1	sterol regulatory element binding transcription factor 1 [Source:HGNC Symbol;Acc:HGNC:11289]

ENSG00000170632	0,13462545	3,50961974	0,43719967	0,5246023	0,76737912	ARMC10	armadillo repeat containing 10 [Source:HGNC Symbol;Acc:HGNC:21706]
ENSG00000174371	0,14972597	3,80231191	0,4368745	0,52475306	0,76737912	EXO1	exonuclease 1 [Source:HGNC Symbol;Acc:HGNC:3511]
ENSG00000205579	-0,2170228	0,11733798	0,43699404	0,52469763	0,76737912	DYNLL1P1	dynein light chain LC8-type 1 pseudogene 1 [Source:HGNC Symbol;Acc:HGNC:20723]
ENSG00000160233	-0,1293214	3,40006787	0,43673715	0,52481677	0,76739547	LRRC3	leucine rich repeat containing 3 [Source:HGNC Symbol;Acc:HGNC:14965]
ENSG00000080845	-0,0911863	6,06011675	0,43602885	0,52514552	0,76772982	DLGAP4	DLG associated protein 4 [Source:HGNC Symbol;Acc:HGNC:24476]
ENSG00000138069	-0,137989	7,25676662	0,43601806	0,52515053	0,76772982	RAB1A	RAB1A, member RAS oncogene family [Source:HGNC Symbol;Acc:HGNC:9758]
ENSG00000126746	0,10007055	4,88839652	0,43571752	0,52529015	0,76775034	ZNF384	zinc finger protein 384 [Source:HGNC Symbol;Acc:HGNC:11955]
ENSG00000143162	0,1046826	4,83663574	0,43481519	0,52570976	0,76775034	CREG1	cellular repressor of E1A stimulated genes 1 [Source:HGNC Symbol;Acc:HGNC:2351]
ENSG00000166261	-0,1126199	3,76078673	0,43512934	0,5255636	0,76775034	ZNF202	zinc finger protein 202 [Source:HGNC Symbol;Acc:HGNC:12994]
ENSG00000175221	0,10529107	5,94696205	0,43556922	0,52535907	0,76775034	MED16	mediator complex subunit 16 [Source:HGNC Symbol;Acc:HGNC:17556]
ENSG00000187595	-0,1748923	1,26595742	0,4347445	0,52574265	0,76775034	ZNF385C	zinc finger protein 385C [Source:HGNC Symbol;Acc:HGNC:33722]
ENSG00000213864	0,17848304	0,46236054	0,43566251	0,52531571	0,76775034	EEF1B2P2	eukaryotic translation elongation factor 1 beta 2 pseudogene 2 [Source:HGNC Symbol;Acc:HGNC:3209]
ENSG00000215906	-0,4901054	-1,7336804	0,43484995	0,52569358	0,76775034	LACTBL1	lactamase beta like 1 [Source:HGNC Symbol;Acc:HGNC:35445]
ENSG00000232485	0,26967416	-0,7465579	0,43525774	0,52550388	0,76775034	RPL37A-DT	RPL37A divergent transcript [Source:HGNC Symbol;Acc:HGNC:40510]
ENSG00000248988	-0,2164542	-0,1320114	0,43482209	0,52570654	0,76775034	PSME2P3	proteasome activator subunit 2 pseudogene 3 [Source:HGNC Symbol;Acc:HGNC:30156]
ENSG00000253517	0,28156274	-1,3005614	0,4352828	0,52549223	0,76775034	XRCC6P4	X-ray repair cross complementing 6 pseudogene 4 [Source:HGNC Symbol;Acc:HGNC:45186]
ENSG00000273391	-0,269338	-0,3791276	0,43542198	0,52542751	0,76775034	AC083880.1	novel transcript, antisense to LUC7L2
ENSG00000167384	0,16106725	2,32273362	0,43453038	0,52584233	0,76781915	ZNF180	zinc finger protein 180 [Source:HGNC Symbol;Acc:HGNC:12970]
ENSG00000075420	0,1510614	6,13318096	0,43308358	0,52651677	0,76789181	FNDC3B	fibronectin type III domain containing 3B [Source:HGNC Symbol;Acc:HGNC:24670]
ENSG00000105058	0,1174056	6,28205855	0,43247548	0,52680074	0,76789181	FAM32A	family with sequence similarity 32 member A [Source:HGNC Symbol;Acc:HGNC:24563]
ENSG00000111642	0,15255623	4,2352142	0,43284081	0,52663011	0,76789181	CHD4	chromodomain helicase DNA binding protein 4 [Source:HGNC Symbol;Acc:HGNC:1919]
ENSG00000112414	0,15310059	7,30022046	0,43362241	0,5262654	0,76789181	ADGRG6	adhesion G protein-coupled receptor G6 [Source:HGNC Symbol;Acc:HGNC:13841]
ENSG00000115183	0,27991293	1,00987803	0,4325291	0,52677569	0,76789181	TANC1	tetratricopeptide repeat, ankyrin repeat and coiled-coil containing 1 [Source:HGNC Symbol;Acc:HGNC:29364]
ENSG00000116984	0,16685933	4,03168879	0,43245722	0,52680927	0,76789181	MTR	5-methyltetrahydrofolate-homocysteine methyltransferase [Source:HGNC Symbol;Acc:HGNC:7468]
ENSG00000130363	-0,1087389	2,47740919	0,43323023	0,52644834	0,76789181	RSPH3	radial spoke head 3 [Source:HGNC Symbol;Acc:HGNC:21054]
ENSG00000131067	0,11441393	4,54913377	0,43321433	0,52645576	0,76789181	GGT7	gamma-glutamyltransferase 7 [Source:HGNC Symbol;Acc:HGNC:4259]
ENSG00000160097	0,26303729	-0,039254	0,43306162	0,52652702	0,76789181	FNDC5	fibronectin type III domain containing 5 [Source:HGNC Symbol;Acc:HGNC:20240]
ENSG00000166188	-0,1143871	3,47598101	0,43262764	0,52672965	0,76789181	ZNF319	zinc finger protein 319 [Source:HGNC Symbol;Acc:HGNC:13644]
ENSG00000175354	0,09054954	5,69400371	0,43257117	0,52675603	0,76789181	PTPN2	protein tyrosine phosphatase non-receptor type 2 [Source:HGNC Symbol;Acc:HGNC:9650]
ENSG00000212978	-0,1443109	2,67589972	0,43285565	0,52662318	0,76789181	AC016747.1	novel transcript
ENSG00000229659	-0,2329598	-0,1689398	0,43332453	0,52640434	0,76789181	RPL26P6	ribosomal protein L26 pseudogene 6 [Source:HGNC Symbol;Acc:HGNC:34023]
ENSG00000236609	0,10215675	2,94495839	0,43315358	0,52648411	0,76789181	ZNF853	zinc finger protein 853 [Source:HGNC Symbol;Acc:HGNC:21767]
ENSG00000237730	0,29726849	-1,1379369	0,43239525	0,52683822	0,76789181	RPS5P8	RPS5 pseudogene 8 [Source:HGNC Symbol;Acc:HGNC:36531]
ENSG00000271602	-0,2516811	-0,5180189	0,43406952	0,52605699	0,76789181	KRT8P49	keratin 8 pseudogene 49 [Source:HGNC Symbol;Acc:HGNC:48345]
ENSG00000274750	0,55328078	-2,007799	0,43318615	0,5264689	0,76789181	H3C12	H3 clustered histone 12 [Source:HGNC Symbol;Acc:HGNC:4774]
ENSG00000279692	0,14414017	1,04920348	0,433505	0,52632015	0,76789181	AC110285.6	TEC
ENSG00000080189	-0,1222192	6,1525597	0,43152357	0,52724583	0,76813137	SLC35C2	solute carrier family 35 member C2 [Source:HGNC Symbol;Acc:HGNC:17117]
ENSG00000116044	-0,1152877	7,24379268	0,4314816	0,52726548	0,76813137	NFE2L2	nuclear factor, erythroid 2 like 2 [Source:HGNC Symbol;Acc:HGNC:7782]
ENSG00000116260	-0,1807211	4,88194223	0,43169879	0,52716385	0,76813137	QSOX1	quiescin sulfhydryl oxidase 1 [Source:HGNC Symbol;Acc:HGNC:9756]
ENSG00000197798	-0,1050966	5,07155595	0,43179493	0,52711888	0,76813137	FAM118B	family with sequence similarity 118 member B [Source:HGNC Symbol;Acc:HGNC:26110]
ENSG00000215126	0,14256666	1,98679192	0,43181105	0,52711134	0,76813137	CBWD6	COBW domain containing 6 [Source:HGNC Symbol;Acc:HGNC:31978]
ENSG00000236844	-0,4014513	-1,2784648	0,43125965	0,52736937	0,76820611	AC069213.3	ribosomal protein L24 (RPL24) pseudogene
ENSG00000166278	0,09995177	5,96984613	0,43100726	0,52748755	0,76830166	C2	complement C2 [Source:HGNC Symbol;Acc:HGNC:1248]
ENSG00000163536	0,13646502	4,01986018	0,43073766	0,52761386	0,76833242	SERPINI1	serpin family I member 1 [Source:HGNC Symbol;Acc:HGNC:8943]

ENSG00000213453	0,24739546	-0,689201	0,43084619	0,527563	0,76833242	FTH1P3	ferritin heavy chain 1 pseudogene 3 [Source:HGNC Symbol;Acc:HGNC:3990]
ENSG00000100271	0,21253883	0,24428718	0,43041334	0,52776587	0,76840059	TTLL1	tubulin tyrosine ligase like 1 [Source:HGNC Symbol;Acc:HGNC:1312]
ENSG00000130479	0,15915105	4,1763918	0,43049822	0,52772607	0,76840059	MAP1S	microtubule associated protein 1S [Source:HGNC Symbol;Acc:HGNC:15715]
ENSG00000226161	-0,2078631	-0,087892	0,4302215	0,52785582	0,76845498	EIF4A1P5	eukaryotic translation initiation factor 4A1 pseudogene 5 [Source:HGNC Symbol;Acc:HGNC:37924]
ENSG00000127990	0,10231985	5,68513134	0,42947056	0,52820823	0,76889139	SGCE	sarcoglycan epsilon [Source:HGNC Symbol;Acc:HGNC:10808]
ENSG00000140395	0,09109336	6,0813207	0,42921287	0,52832926	0,76898223	WDR61	WD repeat domain 61 [Source:HGNC Symbol;Acc:HGNC:30300]
ENSG00000219023	-0,1223221	2,07624586	0,42900158	0,52842855	0,76898223	AL033519.3	ribosomal protein S15A (RPS15A) pseudogene
ENSG00000240291	0,2576685	-0,6351525	0,42902926	0,52841554	0,76898223	AL450384.2	novel transcript, antisense to CACNB2
ENSG00000033327	-0,2281016	3,00189483	0,42857954	0,52862696	0,76913167	GAB2	GRB2 associated binding protein 2 [Source:HGNC Symbol;Acc:HGNC:14458]
ENSG00000155621	-0,1019151	3,10856011	0,42855918	0,52863653	0,76913167	C9orf85	chromosome 9 open reading frame 85 [Source:HGNC Symbol;Acc:HGNC:28784]
ENSG00000168564	0,0949743	4,31115611	0,42841307	0,52870526	0,76915506	CDKN2AIP	CDKN2A interacting protein [Source:HGNC Symbol;Acc:HGNC:24325]
ENSG00000084652	0,18871619	6,15783172	0,42781712	0,52898576	0,76915863	TXLNA	taxilin alpha [Source:HGNC Symbol;Acc:HGNC:30685]
ENSG00000181104	-0,1385329	3,64828573	0,42800187	0,52889877	0,76915863	F2R	coagulation factor II thrombin receptor [Source:HGNC Symbol;Acc:HGNC:3537]
ENSG00000196367	0,20911585	4,99471319	0,4280514	0,52887545	0,76915863	TRRAP	transformation/transcription domain associated protein [Source:HGNC Symbol;Acc:HGNC:12347]
ENSG00000214279	0,43226118	-1,5447422	0,42780558	0,52899119	0,76915863	SCART1	scavenger receptor family member expressed on T cells 1 [Source:HGNC Symbol;Acc:HGNC:32411]
ENSG00000228149	-0,3207424	-1,1983107	0,42773673	0,52902361	0,76915863	RPL3P1	ribosomal protein L3 pseudogene 1 [Source:HGNC Symbol;Acc:HGNC:10352]
ENSG00000258603	-0,5508397	-1,7633175	0,42777014	0,52900788	0,76915863	AC005225.2	novel transcript, antisense to ACOT6
ENSG00000177599	-0,2372857	-0,7083918	0,42761953	0,52907882	0,76916235	ZNF491	zinc finger protein 491 [Source:HGNC Symbol;Acc:HGNC:23706]
ENSG00000249617	-0,3043416	-1,2668727	0,42729764	0,52923049	0,76930629	AC106872.6	ribosomal protein S10 (RPS10) pseudogene
ENSG00000136689	0,13221743	5,87929842	0,42694378	0,52939732	0,76941316	IL1RN	interleukin 1 receptor antagonist [Source:HGNC Symbol;Acc:HGNC:6000]
ENSG00000275185	-0,3792132	-2,0465377	0,42691829	0,52940934	0,76941316	AC130324.3	novel transcript, antisense to TEFM
ENSG00000103534	-0,2908252	-1,2751426	0,42614872	0,52977253	0,76955821	TMC5	transmembrane channel like 5 [Source:HGNC Symbol;Acc:HGNC:22999]
ENSG00000113140	0,10128567	7,63200419	0,42628447	0,52970843	0,76955821	SPARC	secreted protein acidic and cysteine rich [Source:HGNC Symbol;Acc:HGNC:11219]
ENSG00000141198	0,09566134	5,16899762	0,42615517	0,52976948	0,76955821	TOM1L1	target of myb1 like 1 membrane trafficking protein [Source:HGNC Symbol;Acc:HGNC:11983]
ENSG00000233585	0,31697199	-0,816536	0,42646428	0,52962355	0,76955821	AC231533.2	acetyl-Coenzyme A acyltransferase 2 (mitochondrial 3-oxoacyl-Coenzyme A thiolase) (ACAA2) pseudogene
ENSG00000248873	-0,3504129	-1,5850902	0,42622925	0,5297345	0,76955821	SERBP1P6	SERPINE1 mRNA binding protein 1 pseudogene 6 [Source:HGNC Symbol;Acc:HGNC:44635]
ENSG00000214417	-0,3812903	-1,4429575	0,42588127	0,52989886	0,76966518	KRT18P13	keratin 18 pseudogene 13 [Source:HGNC Symbol;Acc:HGNC:6432]
ENSG00000080503	0,16747587	3,19570048	0,42550549	0,53007646	0,76982372	SMARCA2	SWI/SNF related, matrix associated, actin dependent regulator of chromatin, subfamily a, member 2 [Source:HGN
ENSG00000140157	0,13603189	5,75821783	0,42542735	0,5301134	0,76982372	NIPA2	NIPA magnesium transporter 2 [Source:HGNC Symbol;Acc:HGNC:17044]
ENSG00000110031	0,28014862	-0,5828824	0,42522523	0,53020898	0,769886	LPXN	leupaxin [Source:HGNC Symbol;Acc:HGNC:14061]
ENSG00000074800	-0,130557	11,4589727	0,42494686	0,53034068	0,76992418	ENO1	enolase 1 [Source:HGNC Symbol;Acc:HGNC:3350]
ENSG00000146376	0,1022663	7,08407565	0,42498563	0,53032233	0,76992418	ARHGAP18	Rho GTPase activating protein 18 [Source:HGNC Symbol;Acc:HGNC:21035]
ENSG00000123427	0,25002217	0,29752738	0,42430758	0,53064336	0,7700575	EEF1AKMT3	EEF1A lysine methyltransferase 3 [Source:HGNC Symbol;Acc:HGNC:24936]
ENSG00000174989	-0,1303192	2,23388214	0,42443577	0,53058264	0,7700575	FBXW8	F-box and WD repeat domain containing 8 [Source:HGNC Symbol;Acc:HGNC:13597]
ENSG00000197930	-0,0856531	6,60562039	0,42430772	0,5306433	0,7700575	ERO1A	endoplasmic reticulum oxidoreductase 1 alpha [Source:HGNC Symbol;Acc:HGNC:13280]
ENSG00000279827	0,37837261	-1,6642538	0,42456968	0,53051922	0,7700575	AC136469.2	novel transcript
ENSG00000107758	0,12248267	4,43076721	0,42375996	0,53090291	0,77012815	PPP3CB	protein phosphatase 3 catalytic subunit beta [Source:HGNC Symbol;Acc:HGNC:9315]
ENSG00000175073	-0,1201568	2,68526379	0,42383663	0,53086655	0,77012815	VCPIP1	valosin containing protein interacting protein 1 [Source:HGNC Symbol;Acc:HGNC:30897]
ENSG00000225178	-0,098	5,37490564	0,42386138	0,53085482	0,77012815	RPSAP58	ribosomal protein SA pseudogene 58 [Source:HGNC Symbol;Acc:HGNC:36809]
ENSG00000254331	-0,2892751	-1,1657208	0,42380465	0,53088172	0,77012815	CKS1BP7	CDC28 protein kinase regulatory subunit 1B pseudogene 7 [Source:HGNC Symbol;Acc:HGNC:1999]
ENSG00000158717	-0,103368	4,19731844	0,42349982	0,53102628	0,7701611	RNF166	ring finger protein 166 [Source:HGNC Symbol;Acc:HGNC:28856]
ENSG00000165914	0,19571482	1,27103622	0,42344515	0,53105222	0,7701611	TTC7B	tetratricopeptide repeat domain 7B [Source:HGNC Symbol;Acc:HGNC:19858]
ENSG00000188612	0,1105632	7,60936848	0,42337863	0,53108378	0,7701611	SUMO2	small ubiquitin like modifier 2 [Source:HGNC Symbol;Acc:HGNC:11125]
ENSG00000224791	-0,2634434	-0,5474914	0,42313539	0,53119922	0,77025204	KRT18P39	keratin 18 pseudogene 39 [Source:HGNC Symbol;Acc:HGNC:33408]

ENSG00000091490	-0,3087442	-0,4115128	0,42241813	0,5315399	0,77035495	SEL1L3	SEL1L family member 3 [Source:HGNC Symbol;Acc:HGNC:29108]
ENSG00000107937	-0,1244536	6,73067543	0,42203823	0,5317205	0,77035495	GTPBP4	GTP binding protein 4 [Source:HGNC Symbol;Acc:HGNC:21535]
ENSG00000147654	-0,1018814	5,23738029	0,42199276	0,53174213	0,77035495	EBAG9	estrogen receptor binding site associated antigen 9 [Source:HGNC Symbol;Acc:HGNC:3123]
ENSG00000175581	0,08786854	5,51558717	0,42203329	0,53172286	0,77035495	MRPL48	mitochondrial ribosomal protein L48 [Source:HGNC Symbol;Acc:HGNC:16653]
ENSG00000196118	-0,2640203	-0,4939309	0,4223982	0,53154937	0,77035495	CCDC189	coiled-coil domain containing 189 [Source:HGNC Symbol;Acc:HGNC:28078]
ENSG00000227542	-0,4080357	-1,4800415	0,42258526	0,53146047	0,77035495	AC092614.1	novel transcript
ENSG00000234028	-0,2698371	-0,5976537	0,42198722	0,53174477	0,77035495	EIF2AK3-DT	EIF2AK3 divergent transcript [Source:HGNC Symbol;Acc:HGNC:54066]
ENSG00000267280	-0,2095636	2,22264836	0,42269735	0,53140722	0,77035495	TBX2-AS1	TBX2 antisense RNA 1 [Source:HGNC Symbol;Acc:HGNC:50355]
ENSG00000269846	-0,5523891	-2,0831938	0,42230441	0,53159395	0,77035495	AL136172.1	novel transcript, antisense to RBL1
ENSG00000002330	-0,1410252	3,65349794	0,42183471	0,53181731	0,77038364	BAD	BCL2 associated agonist of cell death [Source:HGNC Symbol;Acc:HGNC:936]
ENSG00000101811	0,14407389	5,32940866	0,42096648	0,53223067	0,77086218	CSTF2	cleavage stimulation factor subunit 2 [Source:HGNC Symbol;Acc:HGNC:2484]
ENSG00000125966	-0,2885181	-0,9981813	0,42091921	0,53225319	0,77086218	MMP24	matrix metallopeptidase 24 [Source:HGNC Symbol;Acc:HGNC:7172]
ENSG00000004866	0,0919035	4,70970199	0,42070281	0,53235632	0,77093512	ST7	suppression of tumorigenicity 7 [Source:HGNC Symbol;Acc:HGNC:11351]
ENSG00000250616	-0,300171	-0,6020039	0,42050721	0,53244957	0,77099373	AC012645.1	novel transcript, antisense to YPEL3
ENSG00000272817	0,26524444	-0,828089	0,42013635	0,53262647	0,77117344	AL359198.1	novel transcript, antisense to FGFBP3
ENSG00000163029	-0,0940697	5,22809927	0,4199095	0,53273472	0,77125375	SMC6	structural maintenance of chromosomes 6 [Source:HGNC Symbol;Acc:HGNC:20466]
ENSG00000095906	-0,0995082	5,8099372	0,41972748	0,53282162	0,77130311	NUBP2	nucleotide binding protein 2 [Source:HGNC Symbol;Acc:HGNC:8042]
ENSG00000116918	0,10856541	3,79815583	0,41954675	0,53290792	0,77135162	TSNAX	translin associated factor X [Source:HGNC Symbol;Acc:HGNC:12380]
ENSG00000197114	-0,1192029	2,74802186	0,41931975	0,53301636	0,77143214	ZGPAT	zinc finger CCCH-type and G-patch domain containing [Source:HGNC Symbol;Acc:HGNC:15948]
ENSG00000116771	-0,095785	7,52959411	0,41861063	0,53335539	0,77179307	AGMAT	agmatinase [Source:HGNC Symbol;Acc:HGNC:18407]
ENSG00000176124	-0,1511008	1,77556676	0,41857715	0,5333714	0,77179307	DLEU1	deleted in lymphocytic leukemia 1 [Source:HGNC Symbol;Acc:HGNC:13747]
ENSG00000273702	-0,2570343	-0,5678578	0,41844442	0,53343491	0,77180851	AC091271.1	novel transcript
ENSG00000055483	0,11865802	5,83177716	0,41778978	0,53374835	0,77191056	USP36	ubiquitin specific peptidase 36 [Source:HGNC Symbol;Acc:HGNC:20062]
ENSG00000104442	-0,1217916	4,82965844	0,4177618	0,53376175	0,77191056	ARMC1	armadillo repeat containing 1 [Source:HGNC Symbol;Acc:HGNC:17684]
ENSG00000123975	0,1103955	7,30667721	0,41776026	0,53376249	0,77191056	CKS2	CDC28 protein kinase regulatory subunit 2 [Source:HGNC Symbol;Acc:HGNC:2000]
ENSG00000126461	-0,1240321	5,1380333	0,41774536	0,53376962	0,77191056	SCAF1	SR-related CTD associated factor 1 [Source:HGNC Symbol;Acc:HGNC:30403]
ENSG00000244229	-0,3845421	-1,9971007	0,41795733	0,53366809	0,77191056	RPL26P35	ribosomal protein L26 pseudogene 35 [Source:HGNC Symbol;Acc:HGNC:36278]
ENSG00000177733	0,08766754	7,20398859	0,41749627	0,53388899	0,77200675	HNRNPA0	heterogeneous nuclear ribonucleoprotein A0 [Source:HGNC Symbol;Acc:HGNC:5030]
ENSG00000134375	-0,1154277	6,62844573	0,4173722	0,53394846	0,77201634	TIMM17A	translocase of inner mitochondrial membrane 17A [Source:HGNC Symbol;Acc:HGNC:17315]
ENSG00000185986	0,11347586	2,8587311	0,41723622	0,53401366	0,7720342	SDHAP3	succinate dehydrogenase complex flavoprotein subunit A pseudogene 3 [Source:HGNC Symbol;Acc:HGNC:18781]
ENSG00000165140	0,31958339	-0,8888117	0,41704262	0,53410651	0,77209203	FBP1	fructose-bisphosphatase 1 [Source:HGNC Symbol;Acc:HGNC:3606]
ENSG00000166896	-0,1215136	2,20046488	0,41692495	0,53416296	0,77209723	ATP23	ATP23 metallopeptidase and ATP synthase assembly factor homolog [Source:HGNC Symbol;Acc:HGNC:29452]
ENSG00000164010	-0,146862	1,02212473	0,4166693	0,53428564	0,77219816	ERMAP	erythroblast membrane associated protein (Scianna blood group) [Source:HGNC Symbol;Acc:HGNC:15743]
ENSG00000111802	0,09639411	5,74026561	0,41628373	0,53447078	0,77238931	TDP2	tyrosyl-DNA phosphodiesterase 2 [Source:HGNC Symbol;Acc:HGNC:17768]
ENSG00000124201	-0,1124314	4,73198491	0,41583652	0,53468566	0,77262342	ZNFX1	zinc finger NFX1-type containing 1 [Source:HGNC Symbol;Acc:HGNC:29271]
ENSG00000100412	-0,0975194	7,37262459	0,41545515	0,53486904	0,77281196	ACO2	aconitase 2 [Source:HGNC Symbol;Acc:HGNC:118]
ENSG00000048392	0,12759991	3,46543692	0,41504186	0,5350679	0,77296402	RRM2B	ribonucleotide reductase regulatory TP53 inducible subunit M2B [Source:HGNC Symbol;Acc:HGNC:17296]
ENSG00000138834	-0,1582598	3,50620244	0,41501653	0,53508009	0,77296402	MAPK8IP3	mitogen-activated protein kinase 8 interacting protein 3 [Source:HGNC Symbol;Acc:HGNC:6884]
ENSG00000149273	-0,0866276	11,5293598	0,41456905	0,53529558	0,77304598	RPS3	ribosomal protein S3 [Source:HGNC Symbol;Acc:HGNC:10420]
ENSG00000185432	0,15611104	4,86763354	0,41462571	0,53526829	0,77304598	METTL7A	methyltransferase like 7A [Source:HGNC Symbol;Acc:HGNC:24550]
ENSG00000241547	-0,1987771	0,00277427	0,41466847	0,53524769	0,77304598	ACTG1P20	actin gamma 1 pseudogene 20 [Source:HGNC Symbol;Acc:HGNC:51500]
ENSG00000108588	-0,0967461	7,47419855	0,41355773	0,5357832	0,77333076	CCDC47	coiled-coil domain containing 47 [Source:HGNC Symbol;Acc:HGNC:24856]
ENSG00000119720	-0,1335446	3,81548153	0,41350141	0,53581038	0,77333076	NRDE2	NRDE-2, necessary for RNA interference, domain containing [Source:HGNC Symbol;Acc:HGNC:20186]
ENSG00000128039	0,18611751	0,2584597	0,4137583	0,53568642	0,77333076	SRD5A3	steroid 5 alpha-reductase 3 [Source:HGNC Symbol;Acc:HGNC:25812]

ENSG00000150991	-0,1279896	9,74062291	0,41359781	0,53576386	0,77333076	UBC	ubiquitin C [Source:HGNC Symbol;Acc:HGNC:12468]
ENSG00000160714	-0,0859984	6,08101099	0,41403169	0,53555457	0,77333076	UBE2Q1	ubiquitin conjugating enzyme E2 Q1 [Source:HGNC Symbol;Acc:HGNC:15698]
ENSG00000170846	0,1109287	4,27471476	0,41376375	0,5356838	0,77333076	AC093323.1	Putative MORF4 family-associated protein 1-like protein UPP [Source:UniProtKB/Swiss-Prot;Acc:B2RBV5]
ENSG00000101193	0,09043257	6,85835199	0,41314338	0,53598323	0,77342741	GID8	GID complex subunit 8 homolog [Source:HGNC Symbol;Acc:HGNC:15857]
ENSG00000105438	-0,136291	8,92687362	0,41322887	0,53594195	0,77342741	KDELR1	KDEL endoplasmic reticulum protein retention receptor 1 [Source:HGNC Symbol;Acc:HGNC:6304]
ENSG00000167985	0,10476255	4,30422442	0,41285011	0,5361249	0,7734342	SDHAF2	succinate dehydrogenase complex assembly factor 2 [Source:HGNC Symbol;Acc:HGNC:26034]
ENSG00000177868	-0,1205236	4,42687107	0,41301936	0,53604313	0,7734342	SVBP	small vasohibin binding protein [Source:HGNC Symbol;Acc:HGNC:29204]
ENSG00000184076	-0,1057617	7,39078829	0,41280485	0,53614677	0,7734342	UQCR10	ubiquinol-cytochrome c reductase, complex III subunit X [Source:HGNC Symbol;Acc:HGNC:30863]
ENSG00000075914	-0,0846351	6,14582972	0,41242892	0,53632849	0,77358675	EXOSC7	exosome component 7 [Source:HGNC Symbol;Acc:HGNC:28112]
ENSG00000136122	-0,1054949	3,78587263	0,41236702	0,53635842	0,77358675	BORA	BORA aurora kinase A activator [Source:HGNC Symbol;Acc:HGNC:24724]
ENSG00000269386	-0,1588474	0,90962779	0,41152389	0,53676645	0,77409882	RAB11B-AS1	RAB11B antisense RNA 1 [Source:HGNC Symbol;Acc:HGNC:44178]
ENSG00000273729	0,45105164	-1,5405381	0,41123443	0,53690667	0,77422461	AC007686.3	novel transcript, antisense to IRF2BPL
ENSG00000074935	0,09793535	3,55395312	0,41098679	0,53702669	0,77432125	TUBE1	tubulin epsilon 1 [Source:HGNC Symbol;Acc:HGNC:20775]
ENSG00000101868	0,13768999	4,02941317	0,41047074	0,53727696	0,77439038	POLA1	DNA polymerase alpha 1, catalytic subunit [Source:HGNC Symbol;Acc:HGNC:9173]
ENSG00000125651	-0,1019811	6,38302759	0,41061021	0,5372093	0,77439038	GTF2F1	general transcription factor IIF subunit 1 [Source:HGNC Symbol;Acc:HGNC:4652]
ENSG00000243964	-0,164619	0,91532468	0,41045074	0,53728667	0,77439038	RPL23AP65	ribosomal protein L23a pseudogene 65 [Source:HGNC Symbol;Acc:HGNC:36289]
ENSG00000261884	0,41014268	-1,6223774	0,4104995	0,53726301	0,77439038	AC040162.1	novel transcript
ENSG00000111639	-0,1108003	7,00745015	0,40997409	0,53751804	0,77464743	MRPL51	mitochondrial ribosomal protein L51 [Source:HGNC Symbol;Acc:HGNC:14044]
ENSG00000101230	0,11664301	2,81020082	0,40985484	0,53757595	0,77465448	ISM1	isthmin 1 [Source:HGNC Symbol;Acc:HGNC:16213]
ENSG00000111481	-0,0837916	7,13029594	0,4093906	0,53780153	0,77475028	COPZ1	COPI coat complex subunit zeta 1 [Source:HGNC Symbol;Acc:HGNC:2243]
ENSG00000183891	0,14525618	2,56352857	0,4095384	0,5377297	0,77475028	TTC32	tetratricopeptide repeat domain 32 [Source:HGNC Symbol;Acc:HGNC:32954]
ENSG00000233347	-0,1758199	0,75733257	0,40949172	0,53775238	0,77475028	ERP29P1	endoplasmic reticulum lumenal protein 29 pseudogene 1 [Source:HGNC Symbol;Acc:HGNC:24625]
ENSG00000100426	0,09812891	4,33747455	0,40897203	0,53800508	0,77496709	ZBED4	zinc finger BED-type containing 4 [Source:HGNC Symbol;Acc:HGNC:20721]
ENSG00000008300	0,24949459	3,22260194	0,40820818	0,53837692	0,77508449	CELSR3	cadherin EGF LAG seven-pass G-type receptor 3 [Source:HGNC Symbol;Acc:HGNC:3230]
ENSG00000119408	-0,0872033	6,81494873	0,40846595	0,53825138	0,77508449	NEK6	NIMA related kinase 6 [Source:HGNC Symbol;Acc:HGNC:7749]
ENSG00000161888	0,0974082	3,70448629	0,40810223	0,53842853	0,77508449	SPC24	SPC24 component of NDC80 kinetochore complex [Source:HGNC Symbol;Acc:HGNC:26913]
ENSG00000175130	-0,1059939	8,07833102	0,40829679	0,53833376	0,77508449	MARCKSL1	MARCKS like 1 [Source:HGNC Symbol;Acc:HGNC:7142]
ENSG00000177426	-0,093681	4,87684433	0,408495	0,53823724	0,77508449	TGIF1	TGFB induced factor homeobox 1 [Source:HGNC Symbol;Acc:HGNC:11776]
ENSG00000240429	-0,15313	1,0932993	0,40868871	0,53814294	0,77508449	LRRFIP1P1	LRR binding FLII interacting protein 1 pseudogene 1 [Source:HGNC Symbol;Acc:HGNC:32937]
ENSG00000259137	-0,2674388	-0,9909275	0,40804182	0,53845797	0,77508449	AL109766.1	chromosome 11 open reading frame 58 (C11orf58) pseudogene
ENSG00000071894	-0,1379684	6,32526118	0,40762358	0,53866183	0,77514318	CPSF1	cleavage and polyadenylation specific factor 1 [Source:HGNC Symbol;Acc:HGNC:2324]
ENSG00000166451	-0,1304269	5,01846658	0,40751578	0,53871441	0,77514318	CENPN	centromere protein N [Source:HGNC Symbol;Acc:HGNC:30873]
ENSG00000224616	-0,3116292	-1,3381701	0,40741403	0,53876404	0,77514318	RTCA-AS1	RTCA antisense RNA 1 [Source:HGNC Symbol;Acc:HGNC:50573]
ENSG00000226784	-0,2114907	0,00233308	0,4075755	0,53868528	0,77514318	PGAM4	phosphoglycerate mutase family member 4 [Source:HGNC Symbol;Acc:HGNC:21731]
ENSG00000229022	-0,2314874	0,27474236	0,40771198	0,53861873	0,77514318	AL365366.1	ribosomal protein L6 (RPL6) pseudogene
ENSG00000225022	-0,3591374	-1,4921238	0,4072317	0,53885299	0,77519481	UBE2D3P1	ubiquitin conjugating enzyme E2 D3 pseudogene 1 [Source:HGNC Symbol;Acc:HGNC:33349]
ENSG00000134222	0,11999895	5,34830769	0,40599643	0,5394564	0,77578296	PSRC1	proline and serine rich coiled-coil 1 [Source:HGNC Symbol;Acc:HGNC:24472]
ENSG00000165572	0,12930068	2,68605558	0,40595997	0,53947424	0,77578296	KBTBD6	kelch repeat and BTB domain containing 6 [Source:HGNC Symbol;Acc:HGNC:25340]
ENSG00000213144	-0,3236797	-1,7191893	0,40608335	0,5394139	0,77578296	AC084880.1	PDGFA associated protein 1 (PDAP1) pseudogene
ENSG00000231007	-0,3016847	-1,5041086	0,40622559	0,53934436	0,77578296	CDC20P1	cell division cycle 20 pseudogene 1 [Source:HGNC Symbol;Acc:HGNC:29487]
ENSG00000123240	-0,0922742	5,02540482	0,40555911	0,53967034	0,77591222	OPTN	optineurin [Source:HGNC Symbol;Acc:HGNC:17142]
ENSG00000167770	0,12353444	6,5397099	0,40563699	0,53963223	0,77591222	OTUB1	OTU deubiquitinase, ubiquitin aldehyde binding 1 [Source:HGNC Symbol;Acc:HGNC:23077]
ENSG00000091409	0,12888353	6,44753207	0,40512609	0,53988234	0,77614063	ITGA6	integrin subunit alpha 6 [Source:HGNC Symbol;Acc:HGNC:6142]
ENSG00000203666	-0,1470069	2,16717353	0,40414413	0,54036369	0,77675619	EFCAB2	EF-hand calcium binding domain 2 [Source:HGNC Symbol;Acc:HGNC:28166]

ENSG00000167536	-0,1025672	4,76346168	0,40300621	0,54092255	0,7774179	DHRS13	dehydrogenase/reductase 13 [Source:HGNC Symbol;Acc:HGNC:28326]
ENSG00000168040	-0,0937229	4,81879905	0,4029067	0,54097147	0,7774179	FADD	Fas associated via death domain [Source:HGNC Symbol;Acc:HGNC:3573]
ENSG00000186260	-0,1934242	2,68823796	0,4028819	0,54098366	0,7774179	MRTFB	myocardin related transcription factor B [Source:HGNC Symbol;Acc:HGNC:29819]
ENSG00000103150	0,11125581	3,2878563	0,40258943	0,54112751	0,77754813	MLYCD	malonyl-CoA decarboxylase [Source:HGNC Symbol;Acc:HGNC:7150]
ENSG00000114054	0,13825545	7,43377877	0,40205122	0,54139242	0,77785227	PCCB	propionyl-CoA carboxylase subunit beta [Source:HGNC Symbol;Acc:HGNC:8654]
ENSG00000143942	0,10071768	3,23966067	0,40181054	0,54151097	0,77786959	CHAC2	ChaC glutathione specific gamma-glutamylcyclotransferase 2 [Source:HGNC Symbol;Acc:HGNC:32363]
ENSG00000259781	-0,1026076	2,87008543	0,40189732	0,54146822	0,77786959	HMGB1P6	high mobility group box 1 pseudogene 6 [Source:HGNC Symbol;Acc:HGNC:4998]
ENSG00000251136	-0,1751997	0,43714588	0,40168546	0,5415726	0,77788164	AF117829.1	novel transcript
ENSG00000102287	0,21703668	0,82443818	0,40128228	0,54177134	0,77803034	GABRE	gamma-aminobutyric acid type A receptor subunit epsilon [Source:HGNC Symbol;Acc:HGNC:4085]
ENSG00000107338	-0,0925679	4,5314127	0,40093546	0,54194242	0,77803034	SHB	SH2 domain containing adaptor protein B [Source:HGNC Symbol;Acc:HGNC:10838]
ENSG00000143590	-0,2778874	0,03477596	0,40112455	0,54184913	0,77803034	EFNA3	ephrin A3 [Source:HGNC Symbol;Acc:HGNC:3223]
ENSG00000167674	-0,1323757	6,68684832	0,40123303	0,54179563	0,77803034	HDGFL2	HDGF like 2 [Source:HGNC Symbol;Acc:HGNC:14680]
ENSG00000178202	0,10717451	5,20462076	0,40098514	0,54191791	0,77803034	POGLUT3	protein O-glucosyltransferase 3 [Source:HGNC Symbol;Acc:HGNC:28496]
ENSG00000187504	0,35364873	-1,8545817	0,40081342	0,54200264	0,77804035	RPL7P48	ribosomal protein L7 pseudogene 48 [Source:HGNC Symbol;Acc:HGNC:35540]
ENSG00000135900	0,08847935	6,85461087	0,40047646	0,54216899	0,77815029	MRPL44	mitochondrial ribosomal protein L44 [Source:HGNC Symbol;Acc:HGNC:16650]
ENSG00000143183	0,12198187	6,99522349	0,4004425	0,54218576	0,77815029	TMCO1	transmembrane and coiled-coil domains 1 [Source:HGNC Symbol;Acc:HGNC:18188]
ENSG00000154889	0,15867063	2,51345634	0,40032815	0,54224224	0,7781549	MPPE1	metallophosphoesterase 1 [Source:HGNC Symbol;Acc:HGNC:15988]
ENSG00000011347	0,19625719	0,08479389	0,399824	0,54249138	0,77843596	SYT7	synaptotagmin 7 [Source:HGNC Symbol;Acc:HGNC:11514]
ENSG00000103657	0,18605071	3,52390554	0,39897871	0,5429096	0,7784374	HERC1	HECT and RLD domain containing E3 ubiquitin protein ligase family member 1 [Source:HGNC Symbol;Acc:HGNC:4
ENSG00000105738	0,19196231	2,88756218	0,39919552	0,54280227	0,7784374	SIPA1L3	signal induced proliferation associated 1 like 3 [Source:HGNC Symbol;Acc:HGNC:23801]
ENSG00000117245	-0,3498567	-1,521579	0,39940417	0,54269902	0,7784374	KIF17	kinesin family member 17 [Source:HGNC Symbol;Acc:HGNC:19167]
ENSG00000132394	0,11042017	4,93978384	0,39966507	0,54256997	0,7784374	EEFSEC	eukaryotic elongation factor, selenocysteine-tRNA specific [Source:HGNC Symbol;Acc:HGNC:24614]
ENSG00000146587	0,13587694	3,13190127	0,3989823	0,54290782	0,7784374	RBAK	RB associated KRAB zinc finger [Source:HGNC Symbol;Acc:HGNC:17680]
ENSG00000172315	0,09207683	5,20890524	0,3995339	0,54263484	0,7784374	TP53RK	TP53 regulating kinase [Source:HGNC Symbol;Acc:HGNC:16197]
ENSG00000197863	0,19251984	0,42899245	0,39896041	0,54291866	0,7784374	ZNF790	zinc finger protein 790 [Source:HGNC Symbol;Acc:HGNC:33114]
ENSG00000213080	0,31433206	-1,0506578	0,39914804	0,54282577	0,7784374	AL354714.2	spermine synthase (SMS) pseudogene
ENSG00000110881	-0,1480273	1,06612855	0,39844907	0,54317197	0,77872418	ASIC1	acid sensing ion channel subunit 1 [Source:HGNC Symbol;Acc:HGNC:100]
ENSG00000156639	0,08752024	5,40374362	0,39820225	0,54329433	0,77882316	ZFAND3	zinc finger AN1-type containing 3 [Source:HGNC Symbol;Acc:HGNC:18019]
ENSG00000073536	-0,0879919	5,93194415	0,3979405	0,54342414	0,77893282	NLE1	notchless homolog 1 [Source:HGNC Symbol;Acc:HGNC:19889]
ENSG00000112739	0,10332783	5,64223882	0,39774262	0,54352232	0,77899711	PRPF4B	pre-mRNA processing factor 4B [Source:HGNC Symbol;Acc:HGNC:17346]
ENSG00000103495	0,093018	7,17657124	0,39752044	0,54363259	0,77900231	MAZ	MYC associated zinc finger protein [Source:HGNC Symbol;Acc:HGNC:6914]
ENSG00000225968	0,19212617	0,54781891	0,39762307	0,54358165	0,77900231	ELFN1	extracellular leucine rich repeat and fibronectin type III domain containing 1 [Source:HGNC Symbol;Acc:HGNC:33
ENSG00000078401	0,19840172	0,86244514	0,39687964	0,54395089	0,77911828	EDN1	endothelin 1 [Source:HGNC Symbol;Acc:HGNC:3176]
ENSG00000114353	0,11876988	6,57627064	0,39712514	0,5438289	0,77911828	GNAI2	G protein subunit alpha i2 [Source:HGNC Symbol;Acc:HGNC:4385]
ENSG00000133265	-0,0824584	5,83359785	0,39686986	0,54395575	0,77911828	HSPBP1	HSPA (Hsp70) binding protein 1 [Source:HGNC Symbol;Acc:HGNC:24989]
ENSG00000138131	-0,1508849	5,11537162	0,39701894	0,54388167	0,77911828	LOXL4	lysyl oxidase like 4 [Source:HGNC Symbol;Acc:HGNC:17171]
ENSG00000161654	-0,1030949	3,93607989	0,39671343	0,54403351	0,77911828	LSM12	LSM12 homolog [Source:HGNC Symbol;Acc:HGNC:26407]
ENSG00000278611	0,43835476	-0,9427946	0,3967519	0,54401439	0,77911828	ZNF426-DT	ZNF426 divergent transcript [Source:HGNC Symbol;Acc:HGNC:53777]
ENSG00000083845	-0,1036494	10,0615495	0,39603025	0,54437336	0,77922305	RPS5	ribosomal protein S5 [Source:HGNC Symbol;Acc:HGNC:10426]
ENSG00000102103	0,08271157	6,78452435	0,39621937	0,54427924	0,77922305	PQBP1	polyglutamine binding protein 1 [Source:HGNC Symbol;Acc:HGNC:9330]
ENSG00000115504	-0,0947295	7,09358194	0,39609139	0,54434293	0,77922305	EHBP1	EH domain binding protein 1 [Source:HGNC Symbol;Acc:HGNC:29144]
ENSG00000120519	-0,1272783	2,75955837	0,39620108	0,54428835	0,77922305	SLC10A7	solute carrier family 10 member 7 [Source:HGNC Symbol;Acc:HGNC:23088]
ENSG00000134046	-0,1042849	4,55404569	0,39636776	0,54420542	0,77922305	MBD2	methyl-CpG binding domain protein 2 [Source:HGNC Symbol;Acc:HGNC:6917]
ENSG00000253982	-0,2238204	1,19577538	0,39584697	0,54446461	0,77927731	AC100810.1	novel transcript

ENSG00000186834	0,08406696	5,65641982	0,39562223	0,54457654	0,77930201	HEXIM1	HEXIM P-TEFb complex subunit 1 [Source:HGNC Symbol;Acc:HGNC:24953]
ENSG00000196531	-0,102842	8,91226204	0,3955981	0,54458855	0,77930201	NACA	nascent polypeptide associated complex subunit alpha [Source:HGNC Symbol;Acc:HGNC:7629]
ENSG00000132294	0,09674286	3,31103265	0,39525001	0,54476201	0,77935741	EFR3A	EFR3 homolog A [Source:HGNC Symbol;Acc:HGNC:28970]
ENSG00000136754	0,11534594	4,03759354	0,39519925	0,54478731	0,77935741	ABI1	abl interactor 1 [Source:HGNC Symbol;Acc:HGNC:11320]
ENSG00000163377	0,22854234	-0,2248789	0,39520394	0,54478497	0,77935741	TAFA4	TAFA chemokine like family member 4 [Source:HGNC Symbol;Acc:HGNC:21591]
ENSG00000106538	-0,1099754	6,56456178	0,39498656	0,54489336	0,77943279	RARRES2	retinoic acid receptor responder 2 [Source:HGNC Symbol;Acc:HGNC:9868]
ENSG00000198899	0,0903717	10,3351362	0,39467848	0,54504704	0,77957629	MT-ATP6	mitochondrially encoded ATP synthase membrane subunit 6 [Source:HGNC Symbol;Acc:HGNC:7414]
ENSG00000142856	-0,1346239	3,45440995	0,39448833	0,54514193	0,77963569	ITGB3BP	integrin subunit beta 3 binding protein [Source:HGNC Symbol;Acc:HGNC:6157]
ENSG00000130511	-0,1442228	4,31706886	0,39437979	0,54519612	0,77963685	SSBP4	single stranded DNA binding protein 4 [Source:HGNC Symbol;Acc:HGNC:15676]
ENSG00000079739	0,09314352	7,11591617	0,3940837	0,54534398	0,77969565	PGM1	phosphoglucomutase 1 [Source:HGNC Symbol;Acc:HGNC:8905]
ENSG00000243753	-0,1472936	1,14759906	0,39417613	0,54529781	0,77969565	HLA-L	major histocompatibility complex, class I, L (pseudogene) [Source:HGNC Symbol;Acc:HGNC:4970]
ENSG00000103375	-0,1952978	0,40601695	0,39375174	0,54550984	0,77978359	AQP8	aquaporin 8 [Source:HGNC Symbol;Acc:HGNC:642]
ENSG00000214189	0,28130426	-0,8141304	0,39374695	0,54551224	0,77978359	ZNF788P	zinc finger family member 788, pseudogene [Source:HGNC Symbol;Acc:HGNC:33112]
ENSG00000035403	-0,2330246	5,48029798	0,39346904	0,54565117	0,77985676	VCL	vinculin [Source:HGNC Symbol;Acc:HGNC:12665]
ENSG00000143862	0,14589727	2,46555982	0,39343101	0,54567019	0,77985676	ARL8A	ADP ribosylation factor like GTPase 8A [Source:HGNC Symbol;Acc:HGNC:25192]
ENSG00000163104	0,11555349	3,74392925	0,39325788	0,54575679	0,77990423	SMARCAD1	SWI/SNF-related, matrix-associated actin-dependent regulator of chromatin, subfamily a, containing DEAD/H box
ENSG00000107897	0,09715221	3,60813894	0,39237288	0,54619986	0,78037371	ACBD5	acyl-CoA binding domain containing 5 [Source:HGNC Symbol;Acc:HGNC:23338]
ENSG00000133704	-0,0950232	3,5229035	0,39229149	0,54624064	0,78037371	IPO8	importin 8 [Source:HGNC Symbol;Acc:HGNC:9853]
ENSG00000213370	-0,1944787	0,54714977	0,39228166	0,54624557	0,78037371	RANP6	RAN pseudogene 6 [Source:HGNC Symbol;Acc:HGNC:39861]
ENSG00000104324	0,14599332	5,1745984	0,39216341	0,54630484	0,78038206	CPQ	carboxypeptidase Q [Source:HGNC Symbol;Acc:HGNC:16910]
ENSG00000234964	-0,2398063	-0,8273662	0,39202089	0,54637628	0,78040781	FABP5P7	fatty acid binding protein 5 pseudogene 7 [Source:HGNC Symbol;Acc:HGNC:31070]
ENSG00000064490	-0,1493416	5,62376701	0,39111287	0,54683191	0,780463	RFXANK	regulatory factor X associated ankyrin containing protein [Source:HGNC Symbol;Acc:HGNC:9987]
ENSG00000088833	-0,0960081	7,61406863	0,3912845	0,54674573	0,780463	NSFL1C	NSFL1 cofactor [Source:HGNC Symbol;Acc:HGNC:15912]
ENSG00000150961	-0,1100515	5,65898685	0,39110613	0,54683529	0,780463	SEC24D	SEC24 homolog D, COPII coat complex component [Source:HGNC Symbol;Acc:HGNC:10706]
ENSG00000170464	0,11608269	2,85440969	0,39115786	0,54680931	0,780463	DNAJC18	DnaJ heat shock protein family (Hsp40) member C18 [Source:HGNC Symbol;Acc:HGNC:28429]
ENSG00000175591	-0,1745142	1,49040965	0,39109217	0,5468423	0,780463	P2RY2	purinergic receptor P2Y2 [Source:HGNC Symbol;Acc:HGNC:8541]
ENSG00000184619	0,25347282	-0,1961311	0,39116842	0,54680401	0,780463	KRBA2	KRAB-A domain containing 2 [Source:HGNC Symbol;Acc:HGNC:26989]
ENSG00000215105	-0,2629793	-0,7718985	0,39164761	0,54656349	0,780463	TTC3P1	tetratricopeptide repeat domain 3 pseudogene 1 [Source:HGNC Symbol;Acc:HGNC:23318]
ENSG00000223612	0,3223477	-0,9929361	0,39119499	0,54679067	0,780463	AC241585.1	EGFR-coamplified and overexpressed protein (ECOP) pseudogene
ENSG00000071655	-0,0948468	5,68806221	0,39089321	0,54694224	0,78050112	MBD3	methyl-CpG binding domain protein 3 [Source:HGNC Symbol;Acc:HGNC:6918]
ENSG00000084764	-0,2309944	-0,6754903	0,39082628	0,54697587	0,78050112	MAPRE3	microtubule associated protein RP/EB family member 3 [Source:HGNC Symbol;Acc:HGNC:6892]
ENSG00000048991	0,10318916	3,8640254	0,39035517	0,54721268	0,78076277	R3HDM1	R3H domain containing 1 [Source:HGNC Symbol;Acc:HGNC:9757]
ENSG00000117682	0,09632569	5,01802727	0,38996747	0,54740772	0,78081227	DHDDS	dehydrodolichyl diphosphate synthase subunit [Source:HGNC Symbol;Acc:HGNC:20603]
ENSG00000214535	-0,1150278	2,16156647	0,39017207	0,54730477	0,78081227	RPS15AP1	ribosomal protein S15a pseudogene 1 [Source:HGNC Symbol;Acc:HGNC:16598]
ENSG00000231764	0,16519489	1,25282833	0,39006721	0,54735753	0,78081227	DLX6-AS1	DLX6 antisense RNA 1 [Source:HGNC Symbol;Acc:HGNC:37151]
ENSG00000100297	-0,127581	6,53032557	0,38949985	0,54764314	0,78099557	MCM5	minichromosome maintenance complex component 5 [Source:HGNC Symbol;Acc:HGNC:6948]
ENSG00000196235	0,12794276	6,63890949	0,38960471	0,54759033	0,78099557	SUPT5H	SPT5 homolog, DSIF elongation factor subunit [Source:HGNC Symbol;Acc:HGNC:11469]
ENSG00000256053	-0,0943714	4,01438759	0,38920707	0,54779064	0,78112967	COA8	cytochrome c oxidase assembly factor 8 [Source:HGNC Symbol;Acc:HGNC:20492]
ENSG00000166546	-0,308611	-1,3958643	0,38899418	0,54789794	0,78120643	BEAN1	brain expressed associated with NEDD4 1 [Source:HGNC Symbol;Acc:HGNC:24160]
ENSG00000231185	0,26570865	-0,0340653	0,3888469	0,5479722	0,78123605	SPRY4-AS1	SPRY4 antisense RNA 1 [Source:HGNC Symbol;Acc:HGNC:53465]
ENSG00000130733	-0,1253668	5,01333719	0,38857645	0,54810861	0,78135428	YIPF2	Yip1 domain family member 2 [Source:HGNC Symbol;Acc:HGNC:28476]
ENSG00000064726	-0,1136011	5,05960675	0,38801154	0,54839375	0,78138133	BTBD1	BTB domain containing 1 [Source:HGNC Symbol;Acc:HGNC:1120]
ENSG00000099364	0,14259398	3,64160452	0,38798062	0,54840937	0,78138133	FBXL19	F-box and leucine rich repeat protein 19 [Source:HGNC Symbol;Acc:HGNC:25300]
ENSG00000111885	-0,1178499	7,87521984	0,38806789	0,5483653	0,78138133	MAN1A1	mannosidase alpha class 1A member 1 [Source:HGNC Symbol;Acc:HGNC:6821]

ENSG00000115844	-0,320227	-0,2582346	0,38836364	0,54821599	0,78138133	DLX2	distal-less homeobox 2 [Source:HGNC Symbol;Acc:HGNC:2915]
ENSG00000134352	0,11233884	5,69763283	0,38769142	0,54855547	0,78138133	IL6ST	interleukin 6 signal transducer [Source:HGNC Symbol;Acc:HGNC:6021]
ENSG00000152684	-0,0834335	5,59041923	0,38786616	0,54846719	0,78138133	PELO	pelota mRNA surveillance and ribosome rescue factor [Source:HGNC Symbol;Acc:HGNC:8829]
ENSG00000169131	0,14597753	1,07213747	0,38769552	0,5485534	0,78138133	ZNF354A	zinc finger protein 354A [Source:HGNC Symbol;Acc:HGNC:11628]
ENSG00000204130	-0,1209248	1,92488114	0,38817301	0,54831222	0,78138133	RUFY2	RUN and FYVE domain containing 2 [Source:HGNC Symbol;Acc:HGNC:19761]
ENSG00000134899	0,28059242	-0,5654655	0,38747794	0,54866337	0,78145882	ERCC5	ERCC excision repair 5, endonuclease [Source:HGNC Symbol;Acc:HGNC:3437]
ENSG00000145982	0,09199222	4,9734813	0,38662118	0,54909681	0,7818475	FARS2	phenylalanyl-tRNA synthetase 2, mitochondrial [Source:HGNC Symbol;Acc:HGNC:21062]
ENSG00000223546	0,42253936	-1,6251792	0,38669389	0,54906	0,7818475	LINC00630	long intergenic non-protein coding RNA 630 [Source:HGNC Symbol;Acc:HGNC:44263]
ENSG00000256338	-0,1562609	0,52271553	0,38666004	0,54907714	0,7818475	RPL41P2	ribosomal protein L41 pseudogene 2 [Source:HGNC Symbol;Acc:HGNC:10357]
ENSG00000139146	0,10060702	5,05206999	0,38647839	0,54916912	0,78187425	SINHCAF	SIN3-HDAC complex associated factor [Source:HGNC Symbol;Acc:HGNC:30702]
ENSG00000242612	-0,1106018	4,37935388	0,38631481	0,54925198	0,78191601	DECR2	2,4-dienoyl-CoA reductase 2 [Source:HGNC Symbol;Acc:HGNC:2754]
ENSG00000110048	-0,1339561	5,58993748	0,385796	0,54951492	0,78201372	OSBP	oxysterol binding protein [Source:HGNC Symbol;Acc:HGNC:8503]
ENSG00000117505	-0,0909362	4,99093993	0,38582695	0,54949923	0,78201372	DR1	down-regulator of transcription 1 [Source:HGNC Symbol;Acc:HGNC:3017]
ENSG00000179761	-0,0820511	6,38842193	0,38594578	0,54943899	0,78201372	PIPOX	pipecolic acid and sarcosine oxidase [Source:HGNC Symbol;Acc:HGNC:17804]
ENSG00000256235	-0,1515284	3,58943462	0,38575694	0,54953473	0,78201372	SMIM3	small integral membrane protein 3 [Source:HGNC Symbol;Acc:HGNC:30248]
ENSG00000245848	-0,1120119	7,21369524	0,38553494	0,54964733	0,78209778	CEBPA	CCAAT enhancer binding protein alpha [Source:HGNC Symbol;Acc:HGNC:1833]
ENSG00000229567	0,34644404	-1,7791015	0,38481394	0,55001335	0,78254236	AL139421.1	ribosomal protein L39 (RPL39) pseudogene
ENSG00000130640	-0,0933999	5,69625652	0,38432677	0,55026093	0,78259286	TUBGCP2	tubulin gamma complex associated protein 2 [Source:HGNC Symbol;Acc:HGNC:18599]
ENSG00000196689	-0,1230105	1,65270161	0,38432248	0,55026312	0,78259286	TRPV1	transient receptor potential cation channel subfamily V member 1 [Source:HGNC Symbol;Acc:HGNC:12716]
ENSG00000205352	-0,128281	5,04635599	0,38443812	0,55020432	0,78259286	PRR13	proline rich 13 [Source:HGNC Symbol;Acc:HGNC:24528]
ENSG00000238193	-0,3311394	-1,6104036	0,38441479	0,55021618	0,78259286	HADHBP1	HADHB pseudogene 1 [Source:HGNC Symbol;Acc:HGNC:54887]
ENSG00000213057	0,35181193	-1,1824344	0,38417654	0,55033733	0,78262221	C1orf220	chromosome 1 putative open reading frame 220 [Source:HGNC Symbol;Acc:HGNC:33805]
ENSG00000117602	0,12022572	3,16457643	0,38369147	0,55058413	0,78266844	RCAN3	RCAN family member 3 [Source:HGNC Symbol;Acc:HGNC:3042]
ENSG00000133121	0,10263412	3,60916995	0,38399703	0,55042864	0,78266844	STARD13	StAR related lipid transfer domain containing 13 [Source:HGNC Symbol;Acc:HGNC:19164]
ENSG00000166140	-0,0884855	4,51927637	0,38370428	0,55057761	0,78266844	ZFYVE19	zinc finger FYVE-type containing 19 [Source:HGNC Symbol;Acc:HGNC:20758]
ENSG00000259363	0,20810979	0,5079881	0,3837297	0,55056467	0,78266844	AC090825.1	novel transcript
ENSG00000114738	-0,0983407	6,01411034	0,38332885	0,55076878	0,7828108	MAPKAPK3	MAPK activated protein kinase 3 [Source:HGNC Symbol;Acc:HGNC:6888]
ENSG00000176974	-0,0877226	6,10976946	0,38307401	0,55089861	0,7828108	SHMT1	serine hydroxymethyltransferase 1 [Source:HGNC Symbol;Acc:HGNC:10850]
ENSG00000245680	0,27795443	-0,6209397	0,38317683	0,55084622	0,7828108	ZNF585B	zinc finger protein 585B [Source:HGNC Symbol;Acc:HGNC:30948]
ENSG00000258813	-0,4745268	-2,2013571	0,38316329	0,55085312	0,7828108	AL442663.3	novel transcript, antisense to Rbm25
ENSG00000115944	-0,1054419	6,72417177	0,38286103	0,55100717	0,78285724	COX7A2L	cytochrome c oxidase subunit 7A2 like [Source:HGNC Symbol;Acc:HGNC:2289]
ENSG00000225591	-0,2290516	-0,0681729	0,38279964	0,55103847	0,78285724	BX248409.1	ribosomal protein S27 (metallopanstimulin 1) (RPS27) pseudogene
ENSG00000010404	0,30297008	-1,2436374	0,38230022	0,55129322	0,78299074	IDS	iduronate 2-sulfatase [Source:HGNC Symbol;Acc:HGNC:5389]
ENSG00000111788	0,10697302	2,40599293	0,38247777	0,55120263	0,78299074	AC009533.1	DEAD/H (Asp-Glu-Ala-Asp/His) box polypeptide like pseudogene
ENSG00000120334	-0,1576033	2,14148095	0,38232821	0,55127894	0,78299074	CENPL	centromere protein L [Source:HGNC Symbol;Acc:HGNC:17879]
ENSG00000275263	-0,3122069	-1,1630451	0,3817785	0,5515596	0,78329291	AC135048.3	novel transcript
ENSG00000108883	0,12894627	7,86422205	0,38165766	0,55162134	0,78330444	EFTUD2	elongation factor Tu GTP binding domain containing 2 [Source:HGNC Symbol;Acc:HGNC:30858]
ENSG00000117450	-0,0910176	10,4321826	0,38132728	0,55179019	0,78334016	PRDX1	peroxiredoxin 1 [Source:HGNC Symbol;Acc:HGNC:9352]
ENSG00000133250	-0,1223997	2,388442	0,38148783	0,55170812	0,78334016	ZNF414	zinc finger protein 414 [Source:HGNC Symbol;Acc:HGNC:20630]
ENSG00000227404	-0,498162	-2,0519668	0,38129371	0,55180735	0,78334016	KRT8P20	keratin 8 pseudogene 20 [Source:HGNC Symbol;Acc:HGNC:33372]
ENSG00000125170	-0,1078929	5,33926917	0,38093439	0,55199113	0,78337268	DOK4	docking protein 4 [Source:HGNC Symbol;Acc:HGNC:19868]
ENSG00000236618	0,12777627	2,33207138	0,38112758	0,55189231	0,78337268	PITPNA-AS1	PITPNA antisense RNA 1 [Source:HGNC Symbol;Acc:HGNC:44116]
ENSG00000256663	-0,2373708	0,85152118	0,3809457	0,55198534	0,78337268	AC112777.1	ubiquitin-like with PHD and ring finger domains 1 (UHRF1) pseudogene
ENSG00000084674	-0,1158327	11,8073209	0,38002028	0,5524592	0,78378404	APOB	apolipoprotein B [Source:HGNC Symbol;Acc:HGNC:603]

ENSG00000128191	0,09339052	4,18165193	0,3801633	0,55238592	0,78378404	DGCR8	DGCR8 microprocessor complex subunit [Source:HGNC Symbol;Acc:HGNC:2847]
ENSG00000137497	0,18686128	5,77074869	0,37999153	0,55247393	0,78378404	NUMA1	nuclear mitotic apparatus protein 1 [Source:HGNC Symbol;Acc:HGNC:8059]
ENSG00000198929	0,15328586	1,36208644	0,37994928	0,55249559	0,78378404	NOS1AP	nitric oxide synthase 1 adaptor protein [Source:HGNC Symbol;Acc:HGNC:16859]
ENSG00000197562	-0,0949479	4,04460952	0,37981475	0,55256455	0,78380576	RAB40C	RAB40C, member RAS oncogene family [Source:HGNC Symbol;Acc:HGNC:18285]
ENSG00000110400	0,11772653	4,03197252	0,37950474	0,55272353	0,78395515	NECTIN1	nectin cell adhesion molecule 1 [Source:HGNC Symbol;Acc:HGNC:9706]
ENSG00000104613	0,09927255	6,31006051	0,37777758	0,55361093	0,78445219	INTS10	integrator complex subunit 10 [Source:HGNC Symbol;Acc:HGNC:25548]
ENSG00000125812	-0,1165711	4,55133918	0,37832676	0,55332846	0,78445219	GZF1	GDNF inducible zinc finger protein 1 [Source:HGNC Symbol;Acc:HGNC:15808]
ENSG00000144744	0,09801206	4,87552511	0,3779042	0,55354578	0,78445219	UBA3	ubiquitin like modifier activating enzyme 3 [Source:HGNC Symbol;Acc:HGNC:12470]
ENSG00000151576	0,1118722	4,98588937	0,37837642	0,55330293	0,78445219	QTRT2	queuine tRNA-ribosyltransferase accessory subunit 2 [Source:HGNC Symbol;Acc:HGNC:25771]
ENSG00000160207	0,24852665	-0,4620219	0,37856867	0,55320412	0,78445219	HSF2BP	heat shock transcription factor 2 binding protein [Source:HGNC Symbol;Acc:HGNC:5226]
ENSG00000164463	0,28554323	-0,2633308	0,37814281	0,55342304	0,78445219	CREBRF	CREB3 regulatory factor [Source:HGNC Symbol;Acc:HGNC:24050]
ENSG00000186529	0,12715724	1,4393485	0,37786334	0,5535668	0,78445219	CYP4F3	cytochrome P450 family 4 subfamily F member 3 [Source:HGNC Symbol;Acc:HGNC:2646]
ENSG00000198786	0,11823764	9,07434622	0,37855337	0,55321199	0,78445219	MT-ND5	mitochondrially encoded NADH:ubiquinone oxidoreductase core subunit 5 [Source:HGNC Symbol;Acc:HGNC:7461
ENSG00000240137	-0,3569026	-1,6926414	0,3780534	0,55346903	0,78445219	ERICH6-AS1	ERICH6 antisense RNA 1 [Source:HGNC Symbol;Acc:HGNC:41205]
ENSG00000257093	-0,1093407	2,91667385	0,37818393	0,5534019	0,78445219	DENND11	DENN domain containing 11 [Source:HGNC Symbol;Acc:HGNC:29472]
ENSG00000164849	-0,1400395	0,95708964	0,37723088	0,55389241	0,78471245	GPR146	G protein-coupled receptor 146 [Source:HGNC Symbol;Acc:HGNC:21718]
ENSG00000166845	0,17975523	0,47068634	0,37710796	0,55395574	0,78471245	C18orf54	chromosome 18 open reading frame 54 [Source:HGNC Symbol;Acc:HGNC:13796]
ENSG00000253196	-0,1755728	0,45739715	0,37715354	0,55393226	0,78471245	AC083841.1	novel transcript
ENSG00000115392	0,09596982	3,9496115	0,3767614	0,55413437	0,78486101	FANCL	FA complementation group L [Source:HGNC Symbol;Acc:HGNC:20748]
ENSG00000204147	0,17924761	0,55164388	0,37669604	0,55416807	0,78486101	ASAH2B	N-acylsphingosine amidohydrolase 2B [Source:HGNC Symbol;Acc:HGNC:23456]
ENSG00000074621	0,12108556	2,60062523	0,37643596	0,55430221	0,78489881	SLC24A1	solute carrier family 24 member 1 [Source:HGNC Symbol;Acc:HGNC:10975]
ENSG00000157895	0,09484801	4,47347965	0,37644463	0,55429774	0,78489881	C12orf43	chromosome 12 open reading frame 43 [Source:HGNC Symbol;Acc:HGNC:25719]
ENSG00000277400	0,30596326	-0,5048326	0,37627783	0,5543838	0,78493826	nan	nan
ENSG00000204084	0,11690562	2,67120657	0,37609294	0,55447923	0,78494842	INPP5B	inositol polyphosphate-5-phosphatase B [Source:HGNC Symbol;Acc:HGNC:6077]
ENSG00000273559	0,11232307	4,42591188	0,37605574	0,55449844	0,78494842	CWC25	CWC25 spliceosome associated protein homolog [Source:HGNC Symbol;Acc:HGNC:25989]
ENSG00000168301	-0,0987357	3,0905057	0,37565372	0,55470607	0,78516626	KCTD6	potassium channel tetramerization domain containing 6 [Source:HGNC Symbol;Acc:HGNC:22235]
ENSG00000224699	-0,2764968	-0,6384682	0,37550591	0,55478244	0,78519828	LAMTOR5-AS1	LAMTOR5 antisense RNA 1 [Source:HGNC Symbol;Acc:HGNC:40823]
ENSG00000125846	0,08717254	4,65665491	0,3749966	0,55504578	0,78549489	ZNF133	zinc finger protein 133 [Source:HGNC Symbol;Acc:HGNC:12917]
ENSG00000099308	0,12819544	3,32416072	0,37442082	0,55534378	0,78568829	MAST3	microtubule associated serine/threonine kinase 3 [Source:HGNC Symbol;Acc:HGNC:19036]
ENSG00000104147	0,10007036	4,5965125	0,37457191	0,55526555	0,78568829	OIP5	Opa interacting protein 5 [Source:HGNC Symbol;Acc:HGNC:20300]
ENSG00000204525	0,10734164	8,46477926	0,37443611	0,55533586	0,78568829	HLA-C	major histocompatibility complex, class I, C [Source:HGNC Symbol;Acc:HGNC:4933]
ENSG00000151748	0,13436045	2,76341053	0,37430489	0,55540382	0,78569714	SAV1	salvador family WW domain containing protein 1 [Source:HGNC Symbol;Acc:HGNC:17795]
ENSG00000159079	0,09555213	3,21526841	0,37391732	0,55560463	0,78583771	CFAP298	cilia and flagella associated protein 298 [Source:HGNC Symbol;Acc:HGNC:1301]
ENSG00000174695	-0,0912078	5,56432104	0,37390548	0,55561077	0,78583771	TMEM167A	transmembrane protein 167A [Source:HGNC Symbol;Acc:HGNC:28330]
ENSG00000189144	0,3149557	-0,2550976	0,37370258	0,55571596	0,78591041	ZNF573	zinc finger protein 573 [Source:HGNC Symbol;Acc:HGNC:26420]
ENSG00000244165	-0,1920589	0,67648724	0,3734705	0,55583633	0,78600455	P2RY11	purinergic receptor P2Y11 [Source:HGNC Symbol;Acc:HGNC:8540]
ENSG00000157978	-0,1008566	3,68550168	0,37321151	0,55597072	0,78611849	LDLRAP1	low density lipoprotein receptor adaptor protein 1 [Source:HGNC Symbol;Acc:HGNC:18640]
ENSG00000005238	-0,1272456	1,30990279	0,37274371	0,55621362	0,78623365	FAM214B	family with sequence similarity 214 member B [Source:HGNC Symbol;Acc:HGNC:25666]
ENSG00000134590	-0,1109882	2,65052493	0,37276654	0,55620176	0,78623365	RTL8C	retrotransposon Gag like 8C [Source:HGNC Symbol;Acc:HGNC:2569]
ENSG00000213383	-0,3520636	-1,7009757	0,37291992	0,5561221	0,78623365	AC104297.1	pyrophosphatase (inorganic) 1 (PPA1) pseudogene
ENSG00000014824	0,10613146	4,86909535	0,37259606	0,55629033	0,786266	SLC30A9	solute carrier family 30 member 9 [Source:HGNC Symbol;Acc:HGNC:1329]
ENSG00000070371	0,1267546	3,06986236	0,37244687	0,55636786	0,78629951	CLTCL1	clathrin heavy chain like 1 [Source:HGNC Symbol;Acc:HGNC:2093]
ENSG00000100207	0,15566484	4,41532831	0,37218247	0,55650532	0,78634164	TCF20	transcription factor 20 [Source:HGNC Symbol;Acc:HGNC:11631]
ENSG00000279069	0,1510346	1,26370977	0,37228148	0,55645384	0,78634164	AC015813.5	novel transcript

ENSG00000024526	0,12079572	3,08115229	0,37190003	0,55665224	0,78636774	DEPDC1	DEP domain containing 1 [Source:HGNC Symbol;Acc:HGNC:22949]
ENSG00000109685	0,10204091	5,98028154	0,37183653	0,55668527	0,78636774	NSD2	nuclear receptor binding SET domain protein 2 [Source:HGNC Symbol;Acc:HGNC:12766]
ENSG00000255874	-0,4182774	-2,0711266	0,37184794	0,55667934	0,78636774	PRECSIT	p53 regulated carcinoma associated Stat3 activating long intergenic non-protein coding transcript [Source:HGNC S
ENSG00000128284	0,17892343	1,6405135	0,37159958	0,5568086	0,78638987	APOL3	apolipoprotein L3 [Source:HGNC Symbol;Acc:HGNC:14868]
ENSG00000165704	0,09516067	5,0170754	0,37164819	0,55678329	0,78638987	HPRT1	hypoxanthine phosphoribosyltransferase 1 [Source:HGNC Symbol;Acc:HGNC:5157]
ENSG00000224417	-0,2878408	-0,4028203	0,37138244	0,55692166	0,78647352	AL606970.1	novel transcript
ENSG00000143494	0,41730399	-1,6918517	0,37068337	0,55728598	0,7866883	VASH2	vasohibin 2 [Source:HGNC Symbol;Acc:HGNC:25723]
ENSG00000165244	0,11885861	1,78411753	0,37047079	0,55739685	0,7866883	ZNF367	zinc finger protein 367 [Source:HGNC Symbol;Acc:HGNC:18320]
ENSG00000213839	0,26399516	-1,3986149	0,37083531	0,55720675	0,7866883	TMX2P1	thioredoxin related transmembrane protein 2 pseudogene 1 [Source:HGNC Symbol;Acc:HGNC:49916]
ENSG00000220848	-0,1124435	2,05106823	0,3705223	0,55736999	0,7866883	RPS18P9	ribosomal protein S18 pseudogene 9 [Source:HGNC Symbol;Acc:HGNC:36483]
ENSG00000255642	-0,3399475	-1,2643365	0,37075296	0,55724969	0,7866883	PABPC1P4	poly(A) binding protein cytoplasmic 1 pseudogene 4 [Source:HGNC Symbol;Acc:HGNC:8563]
ENSG00000272669	-0,4393864	-1,681209	0,37054624	0,55735749	0,7866883	AL021707.6	novel transcript, antisense to SUN2
ENSG00000214765	0,12823717	2,10716814	0,36996521	0,55766074	0,78698471	SEPTIN7P2	septin 7 pseudogene 2 [Source:HGNC Symbol;Acc:HGNC:32339]
ENSG00000130177	0,09001787	5,21530818	0,36985441	0,5577186	0,78699034	CDC16	cell division cycle 16 [Source:HGNC Symbol;Acc:HGNC:1720]
ENSG00000184009	-0,1200975	12,4480269	0,3696823	0,55780851	0,78704119	ACTG1	actin gamma 1 [Source:HGNC Symbol;Acc:HGNC:144]
ENSG00000145293	-0,0850877	6,08503781	0,36957387	0,55786517	0,78704512	ENOPH1	enolase-phosphatase 1 [Source:HGNC Symbol;Acc:HGNC:24599]
ENSG00000186566	0,2302223	4,62155421	0,36940452	0,55795368	0,78709398	GPATCH8	G-patch domain containing 8 [Source:HGNC Symbol;Acc:HGNC:29066]
ENSG00000144891	-0,2126582	0,60150652	0,36912146	0,55810169	0,78722676	AGTR1	angiotensin II receptor type 1 [Source:HGNC Symbol;Acc:HGNC:336]
ENSG00000239912	0,30718929	-1,6856339	0,368999	0,55816574	0,7872411	RPL39P36	ribosomal protein L39 pseudogene 36 [Source:HGNC Symbol;Acc:HGNC:36076]
ENSG00000220157	0,21839354	-0,2109503	0,3688888	0,5582234	0,78724642	HNRNPA1P12	heterogeneous nuclear ribonucleoprotein A1 pseudogene 12 [Source:HGNC Symbol;Acc:HGNC:39130]
ENSG00000158106	-0,2973567	-0,8662508	0,36872383	0,55830973	0,78729217	RHPN1	rhophilin Rho GTPase binding protein 1 [Source:HGNC Symbol;Acc:HGNC:19973]
ENSG00000167377	0,25414965	-0,8690992	0,36789241	0,55874525	0,78775423	ZNF23	zinc finger protein 23 [Source:HGNC Symbol;Acc:HGNC:13023]
ENSG00000178307	0,08779874	4,79432247	0,36795287	0,55871356	0,78775423	TMEM11	transmembrane protein 11 [Source:HGNC Symbol;Acc:HGNC:16823]
ENSG00000174529	0,26811471	-1,0953694	0,36749679	0,55895272	0,7879707	TMEM81	transmembrane protein 81 [Source:HGNC Symbol;Acc:HGNC:32349]
ENSG00000100401	-0,1321413	6,91809231	0,36725304	0,55908064	0,78807497	RANGAP1	Ran GTPase activating protein 1 [Source:HGNC Symbol;Acc:HGNC:9854]
ENSG00000106459	0,09660999	3,60293638	0,36681261	0,5593119	0,78824884	NRF1	nuclear respiratory factor 1 [Source:HGNC Symbol;Acc:HGNC:7996]
ENSG00000284753	-0,0997125	3,96219023	0,36687383	0,55927974	0,78824884	EEF1AKMT4	EEF1A lysine methyltransferase 4 [Source:HGNC Symbol;Acc:HGNC:53611]
ENSG00000153487	-0,1146626	3,45927972	0,36660577	0,55942057	0,78832595	ING1	inhibitor of growth family member 1 [Source:HGNC Symbol;Acc:HGNC:6062]
ENSG00000054598	0,27952106	-0,2143816	0,36641926	0,55951861	0,78838805	FOXC1	forkhead box C1 [Source:HGNC Symbol;Acc:HGNC:3800]
ENSG00000169567	-0,1006126	8,48138851	0,36624509	0,55961018	0,78844104	HINT1	histidine triad nucleotide binding protein 1 [Source:HGNC Symbol;Acc:HGNC:4912]
ENSG00000176087	-0,3948197	-1,677003	0,36573891	0,5598765	0,78874019	SLC35A4	solute carrier family 35 member A4 [Source:HGNC Symbol;Acc:HGNC:20753]
ENSG00000174938	0,13936259	6,24488372	0,36519444	0,56016325	0,78891592	SEZ6L2	seizure related 6 homolog like 2 [Source:HGNC Symbol;Acc:HGNC:30844]
ENSG00000231878	-0,2408642	-0,4886159	0,36525506	0,56013131	0,78891592	SNRPFP1	small nuclear ribonucleoprotein polypeptide F pseudogene 1 [Source:HGNC Symbol;Acc:HGNC:16551]
ENSG00000279407	-0,4862345	-1,9499665	0,3653773	0,56006691	0,78891592	AC007191.1	TEC
ENSG00000123838	0,10103431	5,01541563	0,36487904	0,56032949	0,78900596	C4BPA	complement component 4 binding protein alpha [Source:HGNC Symbol;Acc:HGNC:1325]
ENSG00000154832	0,08084276	5,70392899	0,3647658	0,5603892	0,78900596	CXXC1	CXXC finger protein 1 [Source:HGNC Symbol;Acc:HGNC:24343]
ENSG00000187257	0,100795	3,15990155	0,36476654	0,56038881	0,78900596	RSBN1L	round spermatid basic protein 1 like [Source:HGNC Symbol;Acc:HGNC:24765]
ENSG00000115289	0,08956788	4,04347864	0,36450187	0,56052842	0,78904988	PCGF1	polycomb group ring finger 1 [Source:HGNC Symbol;Acc:HGNC:17615]
ENSG00000189043	-0,0872188	8,56066262	0,36452446	0,5605165	0,78904988	NDUFA4	NDUFA4 mitochondrial complex associated [Source:HGNC Symbol;Acc:HGNC:7687]
ENSG00000152117	0,09649084	3,72304898	0,36416006	0,56070883	0,78922779	SMPD4BP	sphingomyelin phosphodiesterase 4B, pseudogene [Source:HGNC Symbol;Acc:HGNC:24761]
ENSG00000104998	0,11015181	4,33614984	0,36384416	0,56087566	0,78923449	IL27RA	interleukin 27 receptor subunit alpha [Source:HGNC Symbol;Acc:HGNC:17290]
ENSG00000138380	-0,3540613	-0,959289	0,36389828	0,56084707	0,78923449	CARF	calcium responsive transcription factor [Source:HGNC Symbol;Acc:HGNC:14435]
ENSG00000238041	0,2732208	-1,5677438	0,36399322	0,56079693	0,78923449	TMEM230P2	TMEM230 pseuogene 2 [Source:HGNC Symbol;Acc:HGNC:55044]
ENSG00000064545	-0,1320602	5,0275345	0,3615284	0,56210175	0,78925918	TMEM161A	transmembrane protein 161A [Source:HGNC Symbol;Acc:HGNC:26020]

ENSG00000088543	-0,2362195	-0,9408576	0,36182202	0,56194599	0,78925918	C3orf18	chromosome 3 open reading frame 18 [Source:HGNC Symbol;Acc:HGNC:24837]
ENSG00000099956	0,08336295	7,69405114	0,36299097	0,56132675	0,78925918	SMARCB1	SWI/SNF related, matrix associated, actin dependent regulator of chromatin, subfamily b, member 1 [Source:HGN
ENSG00000100242	-0,1354964	6,04064087	0,36316275	0,56123587	0,78925918	SUN2	Sad1 and UNC84 domain containing 2 [Source:HGNC Symbol;Acc:HGNC:14210]
ENSG00000101104	-0,1177234	4,17992218	0,36118421	0,56228444	0,78925918	PABPC1L	poly(A) binding protein cytoplasmic 1 like [Source:HGNC Symbol;Acc:HGNC:15797]
ENSG00000106346	0,10523785	2,33714518	0,36271316	0,56147379	0,78925918	USP42	ubiquitin specific peptidase 42 [Source:HGNC Symbol;Acc:HGNC:20068]
ENSG00000108523	0,08479616	7,24609061	0,36235893	0,5616614	0,78925918	RNF167	ring finger protein 167 [Source:HGNC Symbol;Acc:HGNC:24544]
ENSG00000119509	0,09618983	3,34140398	0,36116017	0,56229721	0,78925918	INVS	inversin [Source:HGNC Symbol;Acc:HGNC:17870]
ENSG00000121578	0,09319787	5,34221208	0,36127985	0,56223366	0,78925918	B4GALT4	beta-1,4-galactosyltransferase 4 [Source:HGNC Symbol;Acc:HGNC:927]
ENSG00000131943	0,11184442	4,03720636	0,36339079	0,56111527	0,78925918	C19orf12	chromosome 19 open reading frame 12 [Source:HGNC Symbol;Acc:HGNC:25443]
ENSG00000136828	0,18583069	0,6594985	0,36115892	0,56229787	0,78925918	RALGPS1	Ral GEF with PH domain and SH3 binding motif 1 [Source:HGNC Symbol;Acc:HGNC:16851]
ENSG00000160392	0,08897418	3,53066238	0,36176343	0,56197706	0,78925918	C19orf47	chromosome 19 open reading frame 47 [Source:HGNC Symbol;Acc:HGNC:26723]
ENSG00000161996	0,11688716	3,28380901	0,36276548	0,56144609	0,78925918	WDR90	WD repeat domain 90 [Source:HGNC Symbol;Acc:HGNC:26960]
ENSG00000164253	-0,0893244	4,36705767	0,36243074	0,56162335	0,78925918	WDR41	WD repeat domain 41 [Source:HGNC Symbol;Acc:HGNC:25601]
ENSG00000168765	-0,1125282	4,88120296	0,36116341	0,56229549	0,78925918	GSTM4	glutathione S-transferase mu 4 [Source:HGNC Symbol;Acc:HGNC:4636]
ENSG00000176994	0,11815741	4,09430147	0,36240332	0,56163788	0,78925918	SMCR8	SMCR8-C9orf72 complex subunit [Source:HGNC Symbol;Acc:HGNC:17921]
ENSG00000177963	-0,0787764	6,68710948	0,36194236	0,56188218	0,78925918	RIC8A	RIC8 guanine nucleotide exchange factor A [Source:HGNC Symbol;Acc:HGNC:29550]
ENSG00000179387	0,09899821	4,56266009	0,3619462	0,56188014	0,78925918	ELMOD2	ELMO domain containing 2 [Source:HGNC Symbol;Acc:HGNC:28111]
ENSG00000223723	0,29152622	-1,5708603	0,36337958	0,5611212	0,78925918	TFDP1P2	TFDP1 pseudogene 2 [Source:HGNC Symbol;Acc:HGNC:54930]
ENSG00000228195	0,30248048	-1,3104493	0,36208834	0,56180479	0,78925918	RPL5P27	ribosomal protein L5 pseudogene 27 [Source:HGNC Symbol;Acc:HGNC:36172]
ENSG00000235706	0,24301142	0,08057582	0,36158537	0,56207152	0,78925918	DICER1-AS1	DICER1 antisense RNA 1 [Source:HGNC Symbol;Acc:HGNC:43017]
ENSG00000237745	-0,2811663	-1,4457807	0,36302801	0,56130716	0,78925918	RPL22P8	ribosomal protein L22 pseudogene 8 [Source:HGNC Symbol;Acc:HGNC:36782]
ENSG00000241889	-0,2138031	-0,5225433	0,36117675	0,5622884	0,78925918	AC079944.2	dullard homolog (Xenopus laevis) (DULLARD) pseudogene
ENSG00000249348	0,19065581	0,25971731	0,36176088	0,56197841	0,78925918	UGDH-AS1	UGDH antisense RNA 1 [Source:HGNC Symbol;Acc:HGNC:40601]
ENSG00000254206	-0,3575309	-1,8256669	0,36136332	0,56218936	0,78925918	NPIPB11	nuclear pore complex interacting protein family member B11 [Source:HGNC Symbol;Acc:HGNC:37453]
ENSG00000273759	0,35660488	-1,9085066	0,36177064	0,56197324	0,78925918	AL117379.1	novel transcript
ENSG00000068650	0,18090184	4,89144113	0,36051174	0,56264174	0,78948615	ATP11A	ATPase phospholipid transporting 11A [Source:HGNC Symbol;Acc:HGNC:13552]
ENSG00000100526	-0,1357034	4,8225823	0,36027663	0,56276677	0,78948615	CDKN3	cyclin dependent kinase inhibitor 3 [Source:HGNC Symbol;Acc:HGNC:1791]
ENSG00000112378	-0,0977385	7,14243272	0,36004889	0,56288793	0,78948615	PERP	p53 apoptosis effector related to PMP22 [Source:HGNC Symbol;Acc:HGNC:17637]
ENSG00000113296	-0,2316537	-0,6506523	0,36036029	0,56272227	0,78948615	THBS4	thrombospondin 4 [Source:HGNC Symbol;Acc:HGNC:11788]
ENSG00000187522	0,08500978	4,86269412	0,36012996	0,56284479	0,78948615	HSPA14	heat shock protein family A (Hsp70) member 14 [Source:HGNC Symbol;Acc:HGNC:29526]
ENSG00000196922	0,12051741	2,42784724	0,36011192	0,56285439	0,78948615	ZNF252P	zinc finger protein 252, pseudogene [Source:HGNC Symbol;Acc:HGNC:13046]
ENSG00000257195	-0,2565943	-0,7603856	0,36071388	0,56253429	0,78948615	HNRNPA1P50	heterogeneous nuclear ribonucleoprotein A1 pseudogene 50 [Source:HGNC Symbol;Acc:HGNC:48780]
ENSG00000272459	0,33070958	-1,738529	0,36004143	0,5628919	0,78948615	AC139795.3	novel transcript
ENSG00000226742	0,11144702	2,77699174	0,35990028	0,56296702	0,78951572	HSBP1L1	heat shock factor binding protein 1 like 1 [Source:HGNC Symbol;Acc:HGNC:37243]
ENSG00000197858	-0,0809184	6,47659741	0,35969461	0,56307652	0,78959348	GPAA1	glycosylphosphatidylinositol anchor attachment 1 [Source:HGNC Symbol;Acc:HGNC:4446]
ENSG00000163950	0,11822779	6,36835756	0,35945219	0,56320564	0,78962295	SLBP	stem-loop binding protein [Source:HGNC Symbol;Acc:HGNC:10904]
ENSG00000253304	0,20890288	0,43057329	0,35949672	0,56318192	0,78962295	TMEM200B	transmembrane protein 200B [Source:HGNC Symbol;Acc:HGNC:33785]
ENSG00000153130	0,12143165	4,47216968	0,359326	0,56327287	0,78964144	SCOC	short coiled-coil protein [Source:HGNC Symbol;Acc:HGNC:20335]
ENSG00000204256	-0,0967942	7,16892021	0,35916102	0,5633608	0,78968892	BRD2	bromodomain containing 2 [Source:HGNC Symbol;Acc:HGNC:1103]
ENSG00000004487	-0,0877799	7,42293168	0,35880269	0,56355188	0,78974701	KDM1A	lysine demethylase 1A [Source:HGNC Symbol;Acc:HGNC:29079]
ENSG00000122481	0,19280906	-0,0645217	0,35881197	0,56354692	0,78974701	RWDD3	RWD domain containing 3 [Source:HGNC Symbol;Acc:HGNC:21393]
ENSG00000146143	0,08878451	4,75562801	0,35877917	0,56356442	0,78974701	PRIM2	DNA primase subunit 2 [Source:HGNC Symbol;Acc:HGNC:9370]
ENSG00000130340	0,0951693	5,44794138	0,35845847	0,56373555	0,78991105	SNX9	sorting nexin 9 [Source:HGNC Symbol;Acc:HGNC:14973]
ENSG00000075089	0,09306394	3,21706119	0,35822556	0,5638599	0,7900027	ACTR6	actin related protein 6 [Source:HGNC Symbol;Acc:HGNC:24025]

ENSG00000105176	-0,1083832	5,02647121	0,35813341	0,56390911	0,7900027	URI1	URI1 prefoldin like chaperone [Source:HGNC Symbol;Acc:HGNC:13236]
ENSG00000102879	0,09825922	3,55762088	0,3578073	0,56408334	0,79000496	CORO1A	coronin 1A [Source:HGNC Symbol;Acc:HGNC:2252]
ENSG00000108443	-0,0910494	4,52286212	0,35758871	0,56420019	0,79000496	RPS6KB1	ribosomal protein S6 kinase B1 [Source:HGNC Symbol;Acc:HGNC:10436]
ENSG00000123130	-0,1135074	5,18689877	0,35717498	0,56442148	0,79000496	ACOT9	acyl-CoA thioesterase 9 [Source:HGNC Symbol;Acc:HGNC:17152]
ENSG00000126698	0,0947327	7,50955489	0,35691435	0,56456098	0,79000496	DNAJC8	DnaJ heat shock protein family (Hsp40) member C8 [Source:HGNC Symbol;Acc:HGNC:15470]
ENSG00000130307	0,28395418	-1,5161222	0,35769799	0,56414176	0,79000496	USHBP1	USH1 protein network component harmonin binding protein 1 [Source:HGNC Symbol;Acc:HGNC:24058]
ENSG00000130812	-0,2987484	-0,8861436	0,35788945	0,56403944	0,79000496	ANGPTL6	angiopoietin like 6 [Source:HGNC Symbol;Acc:HGNC:23140]
ENSG00000142273	-0,1243333	1,67745538	0,35683344	0,5646043	0,79000496	CBLC	Cbl proto-oncogene C [Source:HGNC Symbol;Acc:HGNC:15961]
ENSG00000147874	0,09392782	4,55096836	0,35697124	0,56453052	0,79000496	HAUS6	HAUS augmin like complex subunit 6 [Source:HGNC Symbol;Acc:HGNC:25948]
ENSG00000150967	-0,129856	2,75663813	0,35764018	0,56417267	0,79000496	ABCB9	ATP binding cassette subfamily B member 9 [Source:HGNC Symbol;Acc:HGNC:50]
ENSG00000153048	0,10646119	7,27230386	0,35720642	0,56440466	0,79000496	CARHSP1	calcium regulated heat stable protein 1 [Source:HGNC Symbol;Acc:HGNC:17150]
ENSG00000163468	0,1073366	9,497797	0,35706057	0,56448271	0,79000496	CCT3	chaperonin containing TCP1 subunit 3 [Source:HGNC Symbol;Acc:HGNC:1616]
ENSG00000225356	0,31102789	-0,9394151	0,35683789	0,56460191	0,79000496	AC093840.1	epithelial cell adhesion molecule (EPCAM) pseudo
ENSG00000247373	-0,2901448	-1,5205249	0,35681585	0,56461371	0,79000496	TMED2-DT	TMED2 divergent transcript [Source:HGNC Symbol;Acc:HGNC:53346]
ENSG00000166228	-0,100689	7,32038853	0,35649161	0,56478739	0,79017228	PCBD1	pterin-4 alpha-carbinolamine dehydratase 1 [Source:HGNC Symbol;Acc:HGNC:8646]
ENSG00000107957	0,0910725	5,77129182	0,35633769	0,56486987	0,79018021	SH3PXD2A	SH3 and PX domains 2A [Source:HGNC Symbol;Acc:HGNC:23664]
ENSG00000179041	-0,0881281	5,82772902	0,35627918	0,56490123	0,79018021	RRS1	ribosome biogenesis regulator 1 homolog [Source:HGNC Symbol;Acc:HGNC:17083]
ENSG00000081760	-0,0923742	5,28762193	0,35587736	0,5651167	0,79025136	AACS	acetoacetyl-CoA synthetase [Source:HGNC Symbol;Acc:HGNC:21298]
ENSG00000103494	0,0982886	2,87307287	0,35567112	0,56522736	0,79025136	RPGRIP1L	RPGRIP1 like [Source:HGNC Symbol;Acc:HGNC:29168]
ENSG00000111731	0,0983895	3,15605344	0,35605639	0,56502068	0,79025136	C2CD5	C2 calcium dependent domain containing 5 [Source:HGNC Symbol;Acc:HGNC:29062]
ENSG00000124766	-0,1246701	2,75618944	0,35557926	0,56527666	0,79025136	SOX4	SRY-box transcription factor 4 [Source:HGNC Symbol;Acc:HGNC:11200]
ENSG00000230593	0,27004968	-1,4562776	0,35571516	0,56520372	0,79025136	PPIAP40	peptidylprolyl isomerase A pseudogene 40 [Source:HGNC Symbol;Acc:HGNC:53664]
ENSG00000237804	0,33455945	-1,6232464	0,35561678	0,56525652	0,79025136	HNRNPA1P39	heterogeneous nuclear ribonucleoprotein A1 pseudogene 39 [Source:HGNC Symbol;Acc:HGNC:48768]
ENSG00000145919	0,10294597	6,14886906	0,35537098	0,56538848	0,79033205	BOD1	biorientation of chromosomes in cell division 1 [Source:HGNC Symbol;Acc:HGNC:25114]
ENSG00000139116	-0,1782563	4,36740043	0,35504268	0,56556482	0,79050291	KIF21A	kinesin family member 21A [Source:HGNC Symbol;Acc:HGNC:19349]
ENSG00000111328	0,11291781	6,37773863	0,35463954	0,56578151	0,79072288	CDK2AP1	cyclin dependent kinase 2 associated protein 1 [Source:HGNC Symbol;Acc:HGNC:14002]
ENSG00000183291	-0,0843663	7,25489476	0,35454855	0,56583045	0,79072288	SELENOF	selenoprotein F [Source:HGNC Symbol;Acc:HGNC:17705]
ENSG00000151229	-0,1255299	1,33875745	0,35437118	0,56592585	0,79078056	SLC2A13	solute carrier family 2 member 13 [Source:HGNC Symbol;Acc:HGNC:15956]
ENSG00000213891	-0,2046004	-0,3217958	0,35366076	0,56630833	0,79123933	RPL3P6	ribosomal protein L3 pseudogene 6 [Source:HGNC Symbol;Acc:HGNC:36395]
ENSG00000129559	-0,0946557	7,03846572	0,3535009	0,56639447	0,79128401	NEDD8	NEDD8 ubiquitin like modifier [Source:HGNC Symbol;Acc:HGNC:7732]
ENSG00000127324	0,20430071	1,18259144	0,35296528	0,56668328	0,79161178	TSPAN8	tetraspanin 8 [Source:HGNC Symbol;Acc:HGNC:11855]
ENSG00000081051	0,11823276	13,9733164	0,35239384	0,56699173	0,79189123	AFP	alpha fetoprotein [Source:HGNC Symbol;Acc:HGNC:317]
ENSG00000175066	0,12429742	1,77474263	0,35240379	0,56698636	0,79189123	GK5	glycerol kinase 5 [Source:HGNC Symbol;Acc:HGNC:28635]
ENSG00000144730	0,27444207	-1,2260923	0,35208488	0,56715865	0,79197292	IL17RD	interleukin 17 receptor D [Source:HGNC Symbol;Acc:HGNC:17616]
ENSG00000185298	-0,0945638	6,3964413	0,35216557	0,56711505	0,79197292	CCDC137	coiled-coil domain containing 137 [Source:HGNC Symbol;Acc:HGNC:33451]
ENSG00000116396	0,26129547	-0,2271667	0,35186632	0,56727679	0,79206218	KCNC4	potassium voltage-gated channel subfamily C member 4 [Source:HGNC Symbol;Acc:HGNC:6236]
ENSG00000108840	0,08978595	4,82245169	0,35149488	0,56747767	0,79219124	HDAC5	histone deacetylase 5 [Source:HGNC Symbol;Acc:HGNC:14068]
ENSG00000278493	0,47124213	-1,7359936	0,35158048	0,56743137	0,79219124	AC039056.2	novel transcript, sense intronic to EHD4
ENSG00000125814	0,10841289	3,42633006	0,35122698	0,56762265	0,79221736	NAPB	NSF attachment protein beta [Source:HGNC Symbol;Acc:HGNC:15751]
ENSG00000152240	0,10408225	5,48123361	0,35126055	0,56760448	0,79221736	HAUS1	HAUS augmin like complex subunit 1 [Source:HGNC Symbol;Acc:HGNC:25174]
ENSG00000156261	0,09025268	8,23075138	0,35115972	0,56765907	0,79221736	CCT8	chaperonin containing TCP1 subunit 8 [Source:HGNC Symbol;Acc:HGNC:1623]
ENSG00000148843	0,17367194	6,43097568	0,35100465	0,56774304	0,79225886	PDCD11	programmed cell death 11 [Source:HGNC Symbol;Acc:HGNC:13408]
ENSG00000168256	0,08529308	4,40845006	0,35080561	0,56785084	0,79230888	NKIRAS2	NFKB inhibitor interacting Ras like 2 [Source:HGNC Symbol;Acc:HGNC:17898]
ENSG00000238578	0,36579658	-1,599386	0,35073824	0,56788735	0,79230888	SNORD4A	small nucleolar RNA, C/D box 4A [Source:HGNC Symbol;Acc:HGNC:10097]

ENSG00000232587	-0,2462073	-1,0879813	0,35048183	0,56802631	0,79242708	EEF1A1P3	eukaryotic translation elongation factor 1 alpha 1 pseudogene 3 [Source:HGNC Symbol;Acc:HGNC:3194]
ENSG00000116584	-0,1530421	5,52254778	0,35034083	0,56810276	0,79245806	ARHGEF2	Rho/Rac guanine nucleotide exchange factor 2 [Source:HGNC Symbol;Acc:HGNC:682]
ENSG00000068724	-0,0809442	5,7043437	0,34968118	0,5684607	0,79279895	TTC7A	tetratricopeptide repeat domain 7A [Source:HGNC Symbol;Acc:HGNC:19750]
ENSG00000110080	-0,090214	4,3276803	0,34959049	0,56850994	0,79279895	ST3GAL4	ST3 beta-galactoside alpha-2,3-sialyltransferase 4 [Source:HGNC Symbol;Acc:HGNC:10864]
ENSG00000176046	-0,1256879	2,00470308	0,34965164	0,56847674	0,79279895	NUPR1	nuclear protein 1, transcriptional regulator [Source:HGNC Symbol;Acc:HGNC:29990]
ENSG00000124641	0,07970789	5,3319371	0,34946929	0,56857577	0,79281507	MED20	mediator complex subunit 20 [Source:HGNC Symbol;Acc:HGNC:16840]
ENSG00000182359	-0,3366492	-0,1407361	0,34864933	0,56902152	0,79336089	KBTBD3	kelch repeat and BTB domain containing 3 [Source:HGNC Symbol;Acc:HGNC:22934]
ENSG00000149679	-0,1097709	3,69999836	0,34846028	0,5691244	0,79340663	CABLES2	Cdk5 and Abl enzyme substrate 2 [Source:HGNC Symbol;Acc:HGNC:16143]
ENSG00000166173	-0,1845794	0,45170685	0,34838946	0,56916295	0,79340663	LARP6	La ribonucleoprotein 6, translational regulator [Source:HGNC Symbol;Acc:HGNC:24012]
ENSG00000120686	0,08805774	4,75028904	0,34827805	0,5692236	0,79341547	UFM1	ubiquitin fold modifier 1 [Source:HGNC Symbol;Acc:HGNC:20597]
ENSG00000100060	0,18085064	1,71814009	0,34795297	0,56940065	0,79348522	MFNG	MFNG O-fucosylpeptide 3-beta-N-acetylglucosaminyltransferase [Source:HGNC Symbol;Acc:HGNC:7038]
ENSG00000130427	-0,1468351	3,59293384	0,34778734	0,5694909	0,79348522	EPO	erythropoietin [Source:HGNC Symbol;Acc:HGNC:3415]
ENSG00000165269	0,15548778	0,61805697	0,34787227	0,56944462	0,79348522	AQP7	aquaporin 7 [Source:HGNC Symbol;Acc:HGNC:640]
ENSG00000255059	-0,2354053	-0,7953645	0,34794652	0,56940416	0,79348522	PPIAP43	peptidylprolyl isomerase A pseudogene 43 [Source:HGNC Symbol;Acc:HGNC:53667]
ENSG00000023572	0,0906605	4,29469038	0,34722319	0,56979853	0,79363357	GLRX2	glutaredoxin 2 [Source:HGNC Symbol;Acc:HGNC:16065]
ENSG00000104714	-0,0882072	4,08361442	0,34719361	0,56981467	0,79363357	ERICH1	glutamate rich 1 [Source:HGNC Symbol;Acc:HGNC:27234]
ENSG00000125863	0,10599258	4,28317768	0,34732916	0,56974071	0,79363357	MKKS	McKusick-Kaufman syndrome [Source:HGNC Symbol;Acc:HGNC:7108]
ENSG00000158882	-0,1239226	4,69472004	0,34721542	0,56980276	0,79363357	TOMM40L	translocase of outer mitochondrial membrane 40 like [Source:HGNC Symbol;Acc:HGNC:25756]
ENSG00000228791	-0,3716965	-1,8197765	0,3470657	0,56988446	0,79365511	THRB-AS1	THRB antisense RNA 1 [Source:HGNC Symbol;Acc:HGNC:44515]
ENSG00000249222	0,27917843	-1,4172877	0,34680654	0,57002594	0,79377648	ATP5MGL	ATP synthase membrane subunit g like [Source:HGNC Symbol;Acc:HGNC:13213]
ENSG00000197818	-0,1162296	2,84668967	0,3465093	0,5701883	0,79392688	SLC9A8	solute carrier family 9 member A8 [Source:HGNC Symbol;Acc:HGNC:20728]
ENSG00000072071	0,20688129	-0,0162707	0,34617507	0,57037097	0,79395421	ADGRL1	adhesion G protein-coupled receptor L1 [Source:HGNC Symbol;Acc:HGNC:20973]
ENSG00000144824	0,11742261	3,19340191	0,34625633	0,57032655	0,79395421	PHLDB2	pleckstrin homology like domain family B member 2 [Source:HGNC Symbol;Acc:HGNC:29573]
ENSG00000152465	-0,0901491	4,28780408	0,34622492	0,57034372	0,79395421	NMT2	N-myristoyltransferase 2 [Source:HGNC Symbol;Acc:HGNC:7858]
ENSG00000156709	0,12202749	7,3644155	0,346007	0,57046287	0,79400649	AIFM1	apoptosis inducing factor mitochondria associated 1 [Source:HGNC Symbol;Acc:HGNC:8768]
ENSG00000127914	-0,1867273	3,9358975	0,34577408	0,57059029	0,79410818	AKAP9	A-kinase anchoring protein 9 [Source:HGNC Symbol;Acc:HGNC:379]
ENSG00000087076	0,23768981	-0,0300349	0,34503199	0,57099663	0,79410896	HSD17B14	hydroxysteroid 17-beta dehydrogenase 14 [Source:HGNC Symbol;Acc:HGNC:23238]
ENSG00000110958	0,1118183	8,17334419	0,34487595	0,57108215	0,79410896	PTGES3	prostaglandin E synthase 3 [Source:HGNC Symbol;Acc:HGNC:16049]
ENSG00000119630	-0,1070534	5,10229523	0,3454135	0,57078766	0,79410896	PGF	placental growth factor [Source:HGNC Symbol;Acc:HGNC:8893]
ENSG00000136861	0,1330189	5,5938177	0,34541438	0,57078717	0,79410896	CDK5RAP2	CDK5 regulatory subunit associated protein 2 [Source:HGNC Symbol;Acc:HGNC:18672]
ENSG00000160336	-0,0897724	3,40371415	0,3456283	0,57067006	0,79410896	ZNF761	zinc finger protein 761 [Source:HGNC Symbol;Acc:HGNC:23179]
ENSG00000176343	-0,2797757	-1,8143457	0,3447168	0,5711694	0,79410896	RPL37AP8	ribosomal protein L37a pseudogene 8 [Source:HGNC Symbol;Acc:HGNC:35645]
ENSG00000198258	-0,0950304	7,2119843	0,34507807	0,57097138	0,79410896	UBL5	ubiquitin like 5 [Source:HGNC Symbol;Acc:HGNC:13736]
ENSG00000210195	-0,3498232	0,5164837	0,34483088	0,57110685	0,79410896	MT-TT	mitochondrially encoded tRNA-Thr (ACN) [Source:HGNC Symbol;Acc:HGNC:7499]
ENSG00000237361	-0,1408134	1,56546819	0,3448519	0,57109533	0,79410896	TUSC8	tumor suppressor candidate 8 [Source:HGNC Symbol;Acc:HGNC:49111]
ENSG00000242951	-0,131293	2,07916954	0,34468144	0,57118879	0,79410896	AC007182.2	ribosomal protein S24 (RPS24) pseudogene
ENSG00000255964	0,2680085	-1,004856	0,34483425	0,57110501	0,79410896	HSPD1P12	heat shock protein family D (Hsp60) member 1 pseudogene 12 [Source:HGNC Symbol;Acc:HGNC:35140]
ENSG00000242899	-0,166429	0,46540437	0,34441753	0,57133354	0,79423463	RPL7P16	ribosomal protein L7 pseudogene 16 [Source:HGNC Symbol;Acc:HGNC:36591]
ENSG00000079134	0,09502286	4,47148005	0,34409473	0,5715107	0,79440384	THOC1	THO complex 1 [Source:HGNC Symbol;Acc:HGNC:19070]
ENSG00000143367	0,15000074	3,12361997	0,34389859	0,57161839	0,79440384	TUFT1	tuftelin 1 [Source:HGNC Symbol;Acc:HGNC:12422]
ENSG00000183549	-0,1942095	0,08153429	0,34396471	0,57158208	0,79440384	ACSM5	acyl-CoA synthetase medium chain family member 5 [Source:HGNC Symbol;Acc:HGNC:26060]
ENSG00000213465	-0,0858935	5,17164328	0,34331401	0,57193963	0,79477467	ARL2	ADP ribosylation factor like GTPase 2 [Source:HGNC Symbol;Acc:HGNC:693]
ENSG00000204338	0,42224921	-1,4396492	0,34297573	0,57212569	0,79495761	CYP21A1P	cytochrome P450 family 21 subfamily A member 1, pseudogene [Source:HGNC Symbol;Acc:HGNC:2599]
ENSG00000213293	-0,3333593	-1,9738809	0,34277619	0,57223551	0,79503457	AC012618.1	ribosomal protein L28 (RPL28) pseudogene

ENSG00000117114	0,10537252	4,36143301	0,34264447	0,57230802	0,79505971	ADGRL2	adhesion G protein-coupled receptor L2 [Source:HGNC Symbol;Acc:HGNC:18582]
ENSG00000133612	0,07905426	5,39294053	0,34235081	0,57246975	0,79520876	AGAP3	ArfGAP with GTPase domain, ankyrin repeat and PH domain 3 [Source:HGNC Symbol;Acc:HGNC:16923]
ENSG00000126883	0,15894151	4,33614334	0,34218509	0,57256106	0,79525999	NUP214	nucleoporin 214 [Source:HGNC Symbol;Acc:HGNC:8064]
ENSG00000214182	-0,2406743	0,2209683	0,34203089	0,57264605	0,79530242	PTMAP5	prothymosin alpha pseudogene 5 [Source:HGNC Symbol;Acc:HGNC:9628]
ENSG00000162817	0,08997541	6,00265056	0,34177915	0,57278485	0,79541957	C1orf115	chromosome 1 open reading frame 115 [Source:HGNC Symbol;Acc:HGNC:25873]
ENSG00000026036	-0,3435677	-1,4853163	0,34137755	0,57300644	0,79550043	RTEL1-TNFRSF6B	RTEL1-TNFRSF6B readthrough (NMD candidate) [Source:HGNC Symbol;Acc:HGNC:44095]
ENSG00000133678	0,09184435	3,91593311	0,34144347	0,57297005	0,79550043	TMEM254	transmembrane protein 254 [Source:HGNC Symbol;Acc:HGNC:25804]
ENSG00000149084	-0,0864796	5,99005508	0,34141794	0,57298414	0,79550043	HSD17B12	hydroxysteroid 17-beta dehydrogenase 12 [Source:HGNC Symbol;Acc:HGNC:18646]
ENSG00000108474	-0,0921659	4,25990884	0,34050191	0,57349019	0,79590321	PIGL	phosphatidylinositol glycan anchor biosynthesis class L [Source:HGNC Symbol;Acc:HGNC:8966]
ENSG00000133739	-0,1620892	1,29479002	0,34059297	0,57343984	0,79590321	LRRCC1	leucine rich repeat and coiled-coil centrosomal protein 1 [Source:HGNC Symbol;Acc:HGNC:29373]
ENSG00000138663	-0,0845492	5,60946403	0,34026097	0,57362344	0,79590321	COPS4	COP9 signalosome subunit 4 [Source:HGNC Symbol;Acc:HGNC:16702]
ENSG00000164576	-0,1063637	3,49433942	0,34032867	0,573586	0,79590321	SAP30L	SAP30 like [Source:HGNC Symbol;Acc:HGNC:25663]
ENSG00000248527	0,08822742	7,70779129	0,3403618	0,57356767	0,79590321	MTATP6P1	MT-ATP6 pseudogene 1 [Source:HGNC Symbol;Acc:HGNC:44575]
ENSG00000285533	-0,1631045	0,56458481	0,34042645	0,57353191	0,79590321	RELA-DT	RELA divergent transcript [Source:HGNC Symbol;Acc:HGNC:54185]
ENSG00000107186	0,11601039	3,00129596	0,34001352	0,57376037	0,795942	MPDZ	multiple PDZ domain crumbs cell polarity complex component [Source:HGNC Symbol;Acc:HGNC:7208]
ENSG00000247708	0,18530966	0,43086615	0,34007651	0,57372551	0,795942	STX18-AS1	STX18 antisense RNA 1 (head to head) [Source:HGNC Symbol;Acc:HGNC:48877]
ENSG00000224892	0,24997743	-1,2905192	0,33983045	0,57386171	0,796007	RPS4XP16	ribosomal protein S4X pseudogene 16 [Source:HGNC Symbol;Acc:HGNC:36365]
ENSG00000136270	0,07936162	7,05895931	0,33953446	0,57402564	0,79608322	TBRG4	transforming growth factor beta regulator 4 [Source:HGNC Symbol;Acc:HGNC:17443]
ENSG00000160602	-0,1119766	2,41507988	0,33953731	0,57402406	0,79608322	NEK8	NIMA related kinase 8 [Source:HGNC Symbol;Acc:HGNC:13387]
ENSG00000189337	-0,0862352	4,60124831	0,3393968	0,57410192	0,79611343	KAZN	kazrin, periplakin interacting protein [Source:HGNC Symbol;Acc:HGNC:29173]
ENSG00000198763	0,09301794	10,4223331	0,33877447	0,57444701	0,79651636	MT-ND2	mitochondrially encoded NADH:ubiquinone oxidoreductase core subunit 2 [Source:HGNC Symbol;Acc:HGNC:7456
ENSG00000155256	-0,0901214	4,73747783	0,33839593	0,57465712	0,79673208	ZFYVE27	zinc finger FYVE-type containing 27 [Source:HGNC Symbol;Acc:HGNC:26559]
ENSG00000078061	-0,112098	5,32391759	0,33808711	0,57482866	0,79674306	ARAF	A-Raf proto-oncogene, serine/threonine kinase [Source:HGNC Symbol;Acc:HGNC:646]
ENSG00000188428	0,1153825	3,78222043	0,33814611	0,57479588	0,79674306	BLOC1S5	biogenesis of lysosomal organelles complex 1 subunit 5 [Source:HGNC Symbol;Acc:HGNC:18561]
ENSG00000206195	0,12131396	1,42154571	0,33826153	0,57473176	0,79674306	DUXAP8	double homeobox A pseudogene 8 [Source:HGNC Symbol;Acc:HGNC:32187]
ENSG00000092020	-0,089255	5,21621499	0,33783759	0,57496733	0,79685966	PPP2R3C	protein phosphatase 2 regulatory subunit B''gamma [Source:HGNC Symbol;Acc:HGNC:17485]
ENSG00000100335	0,09028146	5,50049785	0,33700068	0,57543296	0,79717615	MIEF1	mitochondrial elongation factor 1 [Source:HGNC Symbol;Acc:HGNC:25979]
ENSG00000128951	0,12419626	5,73489063	0,33674738	0,57557405	0,79717615	DUT	deoxyuridine triphosphatase [Source:HGNC Symbol;Acc:HGNC:3078]
ENSG00000163125	0,17527616	3,87277345	0,33732404	0,57525296	0,79717615	RPRD2	regulation of nuclear pre-mRNA domain containing 2 [Source:HGNC Symbol;Acc:HGNC:29039]
ENSG00000172007	-0,1942353	1,04406353	0,33687318	0,57550397	0,79717615	RAB33B	RAB33B, member RAS oncogene family [Source:HGNC Symbol;Acc:HGNC:16075]
ENSG00000176809	0,2995677	-1,4757524	0,33678475	0,57555323	0,79717615	LRRC37A3	leucine rich repeat containing 37 member A3 [Source:HGNC Symbol;Acc:HGNC:32427]
ENSG00000228451	0,31634043	-1,8788651	0,3372011	0,57532138	0,79717615	SDAD1P1	SDA1 domain containing 1 pseudogene 1 [Source:HGNC Symbol;Acc:HGNC:31403]
ENSG00000230330	-0,2414266	-0,7372164	0,3367409	0,57557766	0,79717615	HMGN2P3	high mobility group nucleosomal binding domain 2 pseudogene 3 [Source:HGNC Symbol;Acc:HGNC:33566]
ENSG00000145907	0,12286395	7,62614629	0,33651643	0,57570275	0,79727382	G3BP1	G3BP stress granule assembly factor 1 [Source:HGNC Symbol;Acc:HGNC:30292]
ENSG00000198171	-0,0853754	7,94850386	0,33623483	0,57585976	0,79741566	DDRGK1	DDRGK domain containing 1 [Source:HGNC Symbol;Acc:HGNC:16110]
ENSG00000103249	0,1132826	5,92560501	0,33564843	0,57618699	0,79752131	CLCN7	chloride voltage-gated channel 7 [Source:HGNC Symbol;Acc:HGNC:2025]
ENSG00000119718	-0,0878544	5,65873431	0,33575363	0,57612825	0,79752131	EIF2B2	eukaryotic translation initiation factor 2B subunit beta [Source:HGNC Symbol;Acc:HGNC:3258]
ENSG00000131437	-0,2392385	2,09835756	0,33560898	0,57620901	0,79752131	KIF3A	kinesin family member 3A [Source:HGNC Symbol;Acc:HGNC:6319]
ENSG00000166575	-0,1028813	5,35068879	0,33562173	0,5762019	0,79752131	TMEM135	transmembrane protein 135 [Source:HGNC Symbol;Acc:HGNC:26167]
ENSG00000171320	-0,0931429	2,83349787	0,33583277	0,57608407	0,79752131	ESCO2	establishment of sister chromatid cohesion N-acetyltransferase 2 [Source:HGNC Symbol;Acc:HGNC:27230]
ENSG00000119048	0,09010747	5,72453783	0,33521886	0,57642695	0,79767181	UBE2B	ubiquitin conjugating enzyme E2 B [Source:HGNC Symbol;Acc:HGNC:12473]
ENSG00000220937	0,26180853	-1,2944185	0,33524275	0,5764136	0,79767181	HNRNPA1P41	heterogeneous nuclear ribonucleoprotein A1 pseudogene 41 [Source:HGNC Symbol;Acc:HGNC:48770]
ENSG00000087269	-0,1434072	6,63545418	0,33484754	0,57663454	0,79780794	NOP14	NOP14 nucleolar protein [Source:HGNC Symbol;Acc:HGNC:16821]
ENSG00000093217	-0,0840502	5,39587191	0,33486691	0,57662371	0,79780794	XYLB	xylulokinase [Source:HGNC Symbol;Acc:HGNC:12839]

ENSG00000023892	-0,089623	3,76296631	0,33472002	0,57670587	0,79783106	DEF6	DEF6 guanine nucleotide exchange factor [Source:HGNC Symbol;Acc:HGNC:2760]
ENSG00000155506	0,20223883	7,11495961	0,33443612	0,57686473	0,79789972	LARP1	La ribonucleoprotein 1, translational regulator [Source:HGNC Symbol;Acc:HGNC:29531]
ENSG00000182481	-0,0908035	9,15499346	0,33449453	0,57683204	0,79789972	KPNA2	karyopherin subunit alpha 2 [Source:HGNC Symbol;Acc:HGNC:6395]
ENSG00000136238	0,09217467	7,52965561	0,33398438	0,57711771	0,7980985	RAC1	Rac family small GTPase 1 [Source:HGNC Symbol;Acc:HGNC:9801]
ENSG00000182247	-0,4084624	-1,8363588	0,33398918	0,57711501	0,7980985	UBE2E2	ubiquitin conjugating enzyme E2 E2 [Source:HGNC Symbol;Acc:HGNC:12478]
ENSG00000198960	0,10421105	3,16934712	0,33381413	0,5772131	0,79815487	ARMCX6	armadillo repeat containing X-linked 6 [Source:HGNC Symbol;Acc:HGNC:26094]
ENSG00000118276	-0,1631872	1,12814224	0,33340586	0,57744201	0,79824474	B4GALT6	beta-1,4-galactosyltransferase 6 [Source:HGNC Symbol;Acc:HGNC:929]
ENSG00000213225	-0,2583056	-0,954825	0,33358359	0,57734234	0,79824474	NOC2LP1	NOC2 like nucleolar associated transcriptional repressor pseudogene 1 [Source:HGNC Symbol;Acc:HGNC:52285]
ENSG00000243660	-0,1415332	1,25581359	0,33344713	0,57741887	0,79824474	ZNF487	zinc finger protein 487 [Source:HGNC Symbol;Acc:HGNC:23488]
ENSG00000164244	0,08993455	4,50805895	0,33301899	0,5776591	0,79840205	PRRC1	proline rich coiled-coil 1 [Source:HGNC Symbol;Acc:HGNC:28164]
ENSG00000253729	0,11530844	6,71787917	0,33300828	0,57766511	0,79840205	PRKDC	protein kinase, DNA-activated, catalytic subunit [Source:HGNC Symbol;Acc:HGNC:9413]
ENSG00000042317	0,13683576	1,19520993	0,3322576	0,57808684	0,79868268	SPATA7	spermatogenesis associated 7 [Source:HGNC Symbol;Acc:HGNC:20423]
ENSG00000138092	0,09621289	5,10240556	0,33247547	0,57796438	0,79868268	CENPO	centromere protein O [Source:HGNC Symbol;Acc:HGNC:28152]
ENSG00000196981	0,1090333	1,71816929	0,33233509	0,57804328	0,79868268	WDR5B	WD repeat domain 5B [Source:HGNC Symbol;Acc:HGNC:17826]
ENSG00000280206	-0,0896179	3,1912681	0,33233551	0,57804304	0,79868268	AC026401.3	novel transcript
ENSG00000213790	-0,168235	0,24413459	0,33144227	0,57854563	0,79924095	OLA1P1	OLA1 pseudogene 1 [Source:HGNC Symbol;Acc:HGNC:45274]
ENSG00000137343	-0,0898669	3,92840232	0,33120367	0,57868003	0,79935103	ATAT1	alpha tubulin acetyltransferase 1 [Source:HGNC Symbol;Acc:HGNC:21186]
ENSG00000065911	-0,0972346	7,84868253	0,33083056	0,57889034	0,79941478	MTHFD2	methylenetetrahydrofolate dehydrogenase (NADP+ dependent) 2, methenyltetrahydrofolate cyclohydrolase [Sou
ENSG00000145632	-0,1107202	3,59880445	0,3308936	0,57885479	0,79941478	PLK2	polo like kinase 2 [Source:HGNC Symbol;Acc:HGNC:19699]
ENSG00000160285	0,08416209	7,12324231	0,33093272	0,57883274	0,79941478	LSS	lanosterol synthase [Source:HGNC Symbol;Acc:HGNC:6708]
ENSG00000049860	-0,0958556	7,15076515	0,33029855	0,57919048	0,79946799	HEXB	hexosaminidase subunit beta [Source:HGNC Symbol;Acc:HGNC:4879]
ENSG00000106105	0,1107991	8,12594812	0,33055736	0,57904443	0,79946799	GARS1	glycyl-tRNA synthetase 1 [Source:HGNC Symbol;Acc:HGNC:4162]
ENSG00000113966	0,16148097	0,36785104	0,33010928	0,57929734	0,79946799	ARL6	ADP ribosylation factor like GTPase 6 [Source:HGNC Symbol;Acc:HGNC:13210]
ENSG00000115677	-0,0961491	9,70142737	0,33065869	0,57898726	0,79946799	HDLBP	high density lipoprotein binding protein [Source:HGNC Symbol;Acc:HGNC:4857]
ENSG00000152705	-0,3037496	-0,7333639	0,33008343	0,57931194	0,79946799	CATSPER3	cation channel sperm associated 3 [Source:HGNC Symbol;Acc:HGNC:20819]
ENSG00000155428	-0,2301409	-0,9594824	0,33020914	0,57924096	0,79946799	TRIM74	tripartite motif containing 74 [Source:HGNC Symbol;Acc:HGNC:17453]
ENSG00000255959	-0,3580553	-1,8557499	0,33038759	0,57914022	0,79946799	AP000777.2	novel transcript, antisense to CCDC86
ENSG00000115541	-0,1010385	7,18730975	0,32993236	0,57939727	0,79950119	HSPE1	heat shock protein family E (Hsp10) member 1 [Source:HGNC Symbol;Acc:HGNC:5269]
ENSG00000166263	0,12184724	3,48474959	0,32975022	0,57950018	0,79950119	STXBP4	syntaxin binding protein 4 [Source:HGNC Symbol;Acc:HGNC:19694]
ENSG00000167118	-0,0902534	5,8198492	0,32978413	0,57948102	0,79950119	URM1	ubiquitin related modifier 1 [Source:HGNC Symbol;Acc:HGNC:28378]
ENSG00000144040	0,08626192	4,94657842	0,32923226	0,57979304	0,79961647	SFXN5	sideroflexin 5 [Source:HGNC Symbol;Acc:HGNC:16073]
ENSG00000158006	-0,0905285	3,93736521	0,32935186	0,57972539	0,79961647	PAFAH2	platelet activating factor acetylhydrolase 2 [Source:HGNC Symbol;Acc:HGNC:8579]
ENSG00000166394	0,22046274	-0,4436769	0,32930581	0,57975144	0,79961647	CYB5R2	cytochrome b5 reductase 2 [Source:HGNC Symbol;Acc:HGNC:24376]
ENSG00000258458	0,27126022	-1,6538762	0,32921524	0,57980267	0,79961647	AL160314.2	novel transcript, antisense to OXA1L
ENSG00000115526	0,11164444	4,36518439	0,329004	0,57992221	0,79970582	CHST10	carbohydrate sulfotransferase 10 [Source:HGNC Symbol;Acc:HGNC:19650]
ENSG00000140688	-0,0940327	5,72912962	0,32833899	0,58029885	0,79982466	RUSF1	RUS family member 1 [Source:HGNC Symbol;Acc:HGNC:25848]
ENSG00000166478	0,10404859	3,98563286	0,32874329	0,5800698	0,79982466	ZNF143	zinc finger protein 143 [Source:HGNC Symbol;Acc:HGNC:12928]
ENSG00000204745	-0,1118454	1,92023403	0,32845571	0,5802327	0,79982466	AC083899.1	anaphase promoting complex subunit 1 (ANAPC1) pseudogene
ENSG00000235472	-0,2158498	-0,8010601	0,3282719	0,58033687	0,79982466	EIF4A1P7	eukaryotic translation initiation factor 4A1 pseudogene 7 [Source:HGNC Symbol;Acc:HGNC:37926]
ENSG00000236773	0,25099983	-1,3604338	0,32830523	0,58031798	0,79982466	AC092809.3	novel pseudogene
ENSG00000242952	0,18915628	-0,2174263	0,3282928	0,58032503	0,79982466	RPSAP3	ribosomal protein SA pseudogene 3 [Source:HGNC Symbol;Acc:HGNC:19792]
ENSG00000148584	-0,108185	6,05233827	0,32801167	0,58048442	0,79995255	A1CF	APOBEC1 complementation factor [Source:HGNC Symbol;Acc:HGNC:24086]
ENSG00000103174	0,08502818	7,16978455	0,32790597	0,58054437	0,7999597	NAGPA	N-acetylglucosamine-1-phosphodiester alpha-N-acetylglucosaminidase [Source:HGNC Symbol;Acc:HGNC:17378]
ENSG00000117899	-0,0792018	8,3207493	0,32763177	0,58069996	0,80009862	MESD	mesoderm development LRP chaperone [Source:HGNC Symbol;Acc:HGNC:13520]

ENSG00000166685	0,09217064	4,92377828	0,32745797	0,58079863	0,8001591	COG1	component of oligomeric golgi complex 1 [Source:HGNC Symbol;Acc:HGNC:6545]
ENSG00000141526	-0,0779927	9,81076779	0,32723098	0,58092754	0,80024017	SLC16A3	solute carrier family 16 member 3 [Source:HGNC Symbol;Acc:HGNC:10924]
ENSG00000169519	-0,1239445	2,82227663	0,32716147	0,58096703	0,80024017	METTL15	methyltransferase like 15 [Source:HGNC Symbol;Acc:HGNC:26606]
ENSG00000163374	0,08272136	4,79921412	0,32699076	0,58106403	0,80029833	YY1AP1	YY1 associated protein 1 [Source:HGNC Symbol;Acc:HGNC:30935]
ENSG00000130589	-0,1973695	2,85075443	0,32644801	0,58137267	0,80057247	HELZ2	helicase with zinc finger 2 [Source:HGNC Symbol;Acc:HGNC:30021]
ENSG00000273061	0,34086397	-0,9755803	0,3264893	0,58134917	0,80057247	CDC37L1-DT	CDC37L1 divergent transcript [Source:HGNC Symbol;Acc:HGNC:49735]
ENSG00000123395	0,1009045	6,19314397	0,32553942	0,58189012	0,80060682	ATG101	autophagy related 101 [Source:HGNC Symbol;Acc:HGNC:25679]
ENSG00000137801	0,20152467	7,86015914	0,3257693	0,58175911	0,80060682	THBS1	thrombospondin 1 [Source:HGNC Symbol;Acc:HGNC:11785]
ENSG00000139618	0,11881409	2,57020641	0,32567488	0,58181291	0,80060682	BRCA2	BRCA2 DNA repair associated [Source:HGNC Symbol;Acc:HGNC:1101]
ENSG00000159596	0,10604613	5,02319821	0,32611079	0,5815646	0,80060682	TMEM69	transmembrane protein 69 [Source:HGNC Symbol;Acc:HGNC:28035]
ENSG00000160917	-0,1133513	5,25297781	0,32614654	0,58154425	0,80060682	CPSF4	cleavage and polyadenylation specific factor 4 [Source:HGNC Symbol;Acc:HGNC:2327]
ENSG00000171603	0,12336889	6,88888784	0,32559713	0,58185722	0,80060682	CLSTN1	calsyntenin 1 [Source:HGNC Symbol;Acc:HGNC:17447]
ENSG00000183134	0,14753177	0,65705709	0,32553816	0,58189084	0,80060682	PTGDR2	prostaglandin D2 receptor 2 [Source:HGNC Symbol;Acc:HGNC:4502]
ENSG00000186272	-0,1388649	1,72438603	0,32584557	0,58171565	0,80060682	ZNF17	zinc finger protein 17 [Source:HGNC Symbol;Acc:HGNC:12958]
ENSG00000221883	-0,1503736	0,61024343	0,32565102	0,58182651	0,80060682	ARIH2OS	ARIH2 opposite strand lncRNA [Source:HGNC Symbol;Acc:HGNC:34425]
ENSG00000013573	0,08654568	3,96873494	0,32530327	0,58202477	0,80071568	DDX11	DEAD/H-box helicase 11 [Source:HGNC Symbol;Acc:HGNC:2736]
ENSG00000141012	-0,1025573	4,75367431	0,32508244	0,58215074	0,80073818	GALNS	galactosamine (N-acetyl)-6-sulfatase [Source:HGNC Symbol;Acc:HGNC:4122]
ENSG00000148719	0,08474782	6,39170022	0,32509013	0,58214635	0,80073818	DNAJB12	DnaJ heat shock protein family (Hsp40) member B12 [Source:HGNC Symbol;Acc:HGNC:14891]
ENSG00000099821	0,10860798	6,69702852	0,32467993	0,5823805	0,80097879	POLRMT	RNA polymerase mitochondrial [Source:HGNC Symbol;Acc:HGNC:9200]
ENSG00000143184	0,18408923	0,15619729	0,32443734	0,58251907	0,80106128	XCL1	X-C motif chemokine ligand 1 [Source:HGNC Symbol;Acc:HGNC:10645]
ENSG00000271966	0,48956092	-1,7855132	0,32438296	0,58255014	0,80106128	AC021321.1	novel transcript
ENSG00000168994	0,08084163	4,94574724	0,32424533	0,5826288	0,80109404	PXDC1	PX domain containing 1 [Source:HGNC Symbol;Acc:HGNC:21361]
ENSG00000181472	-0,0805369	4,53107828	0,32413483	0,58269197	0,80110549	ZBTB2	zinc finger and BTB domain containing 2 [Source:HGNC Symbol;Acc:HGNC:20868]
ENSG00000163453	0,29548798	-0,5846991	0,3237894	0,58288952	0,80115799	IGFBP7	insulin like growth factor binding protein 7 [Source:HGNC Symbol;Acc:HGNC:5476]
ENSG00000178127	-0,307671	-1,3732134	0,32387126	0,58284269	0,80115799	NDUFV2	NADH:ubiquinone oxidoreductase core subunit V2 [Source:HGNC Symbol;Acc:HGNC:7717]
ENSG00000178761	0,10977921	3,82713595	0,32364683	0,5829711	0,80115799	FAM219B	family with sequence similarity 219 member B [Source:HGNC Symbol;Acc:HGNC:24695]
ENSG00000245571	0,26943193	-0,8714554	0,32369779	0,58294193	0,80115799	FAM111A-DT	FAM111A divergent transcript [Source:HGNC Symbol;Acc:HGNC:53752]
ENSG00000250903	-0,1270248	1,33030339	0,32358872	0,58300435	0,80115799	GMDS-DT	GMDS divergent transcript [Source:HGNC Symbol;Acc:HGNC:48993]
ENSG00000037241	-0,0849293	5,95951935	0,32263181	0,5835526	0,80140824	RPL26L1	ribosomal protein L26 like 1 [Source:HGNC Symbol;Acc:HGNC:17050]
ENSG00000093183	0,11252336	4,55940539	0,3227227	0,58350048	0,80140824	SEC22C	SEC22 homolog C, vesicle trafficking protein [Source:HGNC Symbol;Acc:HGNC:16828]
ENSG00000107798	0,10483761	6,82166538	0,32260066	0,58357046	0,80140824	LIPA	lipase A, lysosomal acid type [Source:HGNC Symbol;Acc:HGNC:6617]
ENSG00000122786	-0,1354995	6,85036983	0,32279713	0,5834578	0,80140824	CALD1	caldesmon 1 [Source:HGNC Symbol;Acc:HGNC:1441]
ENSG00000234851	-0,1046227	3,41124979	0,3230916	0,58328904	0,80140824	RPL23AP42	ribosomal protein L23a pseudogene 42 [Source:HGNC Symbol;Acc:HGNC:35942]
ENSG00000241360	0,25014672	-1,5043639	0,32284496	0,58343038	0,80140824	PDXP	pyridoxal phosphatase [Source:HGNC Symbol;Acc:HGNC:30259]
ENSG00000257758	0,20974966	-0,3238294	0,32297435	0,58335622	0,80140824	KRT18P20	keratin 18 pseudogene 20 [Source:HGNC Symbol;Acc:HGNC:33388]
ENSG00000144362	0,21898184	0,81876298	0,32192883	0,58395605	0,80186239	PHOSPHO2	phosphatase, orphan 2 [Source:HGNC Symbol;Acc:HGNC:28316]
ENSG00000019582	-0,1100862	7,59205674	0,32128274	0,58432738	0,80229687	CD74	CD74 molecule [Source:HGNC Symbol;Acc:HGNC:1697]
ENSG00000119801	0,09108637	3,63535594	0,32069594	0,58466506	0,80254973	YPEL5	yippee like 5 [Source:HGNC Symbol;Acc:HGNC:18329]
ENSG00000132780	0,09457368	8,09694113	0,32027435	0,58490794	0,80254973	NASP	nuclear autoantigenic sperm protein [Source:HGNC Symbol;Acc:HGNC:7644]
ENSG00000133985	-0,2926242	-1,0141581	0,32059301	0,58472434	0,80254973	TTC9	tetratricopeptide repeat domain 9 [Source:HGNC Symbol;Acc:HGNC:20267]
ENSG00000155368	-0,1098571	8,76708547	0,32011336	0,58500073	0,80254973	DBI	diazepam binding inhibitor, acyl-CoA binding protein [Source:HGNC Symbol;Acc:HGNC:2690]
ENSG00000177674	0,0945638	5,93480117	0,32065247	0,58469009	0,80254973	AGTRAP	angiotensin II receptor associated protein [Source:HGNC Symbol;Acc:HGNC:13539]
ENSG00000180357	0,27310526	3,76423852	0,32040374	0,58483337	0,80254973	ZNF609	zinc finger protein 609 [Source:HGNC Symbol;Acc:HGNC:29003]
ENSG00000204052	-0,2530334	-0,7836744	0,32010429	0,58500596	0,80254973	LRRC73	leucine rich repeat containing 73 [Source:HGNC Symbol;Acc:HGNC:21375]

ENSG00000213020	-0,1960659	-0,0872106	0,32043103	0,58481765	0,80254973	ZNF611	zinc finger protein 611 [Source:HGNC Symbol;Acc:HGNC:28766]
ENSG00000254598	0,20262594	-0,3564202	0,32022347	0,58493726	0,80254973	CSNK2A3	casein kinase 2 alpha 3 [Source:HGNC Symbol;Acc:HGNC:2458]
ENSG00000011275	0,0944861	5,41154528	0,31977053	0,58519845	0,80273842	RNF216	ring finger protein 216 [Source:HGNC Symbol;Acc:HGNC:21698]
ENSG00000259959	-0,2160945	-0,3405546	0,31945794	0,58537886	0,80291051	AC107068.1	novel transcript
ENSG00000106263	-0,0856594	8,70825656	0,31910897	0,58558041	0,80303615	EIF3B	eukaryotic translation initiation factor 3 subunit B [Source:HGNC Symbol;Acc:HGNC:3280]
ENSG00000174744	0,10048724	5,56860514	0,31913319	0,58556641	0,80303615	BRMS1	BRMS1 transcriptional repressor and anoikis regulator [Source:HGNC Symbol;Acc:HGNC:17262]
ENSG00000272196	-0,2730718	-1,6480074	0,31895949	0,58566678	0,80307921	H2AC18	H2A clustered histone 18 [Source:HGNC Symbol;Acc:HGNC:4736]
ENSG00000213430	0,13234346	3,3290319	0,31834106	0,58602442	0,8034942	HSPD1P1	heat shock protein family D (Hsp60) member 1 pseudogene 1 [Source:HGNC Symbol;Acc:HGNC:35133]
ENSG00000072864	-0,08216	5,69028703	0,31809733	0,5861655	0,80358561	NDE1	nudE neurodevelopment protein 1 [Source:HGNC Symbol;Acc:HGNC:17619]
ENSG00000119242	-0,1176401	2,25951753	0,31791443	0,58627141	0,80358561	CCDC92	coiled-coil domain containing 92 [Source:HGNC Symbol;Acc:HGNC:29563]
ENSG00000137776	-0,1644133	5,59521086	0,3172765	0,58664115	0,80358561	SLTM	SAFB like transcription modulator [Source:HGNC Symbol;Acc:HGNC:20709]
ENSG00000139154	-0,0887559	2,89407606	0,31758995	0,58645941	0,80358561	AEBP2	AE binding protein 2 [Source:HGNC Symbol;Acc:HGNC:24051]
ENSG00000143382	0,11292616	3,76558561	0,31727963	0,58663933	0,80358561	ADAMTSL4	ADAMTS like 4 [Source:HGNC Symbol;Acc:HGNC:19706]
ENSG00000155959	0,09605279	5,70711954	0,31730249	0,58662607	0,80358561	VBP1	VHL binding protein 1 [Source:HGNC Symbol;Acc:HGNC:12662]
ENSG00000163389	0,1037471	5,11294954	0,31772676	0,58638013	0,80358561	POGLUT1	protein O-glucosyltransferase 1 [Source:HGNC Symbol;Acc:HGNC:22954]
ENSG00000178971	-0,1398588	3,43385899	0,31747441	0,58652639	0,80358561	CTC1	CST telomere replication complex component 1 [Source:HGNC Symbol;Acc:HGNC:26169]
ENSG00000179456	0,24086474	0,26109523	0,31764141	0,58642959	0,80358561	ZBTB18	zinc finger and BTB domain containing 18 [Source:HGNC Symbol;Acc:HGNC:13030]
ENSG00000254505	-0,3200194	-1,2840299	0,31772005	0,58638402	0,80358561	CHMP4A	charged multivesicular body protein 4A [Source:HGNC Symbol;Acc:HGNC:20274]
ENSG00000204172	-0,1377026	0,92312296	0,31713104	0,58672552	0,80362584	AGAP9	ArfGAP with GTPase domain, ankyrin repeat and PH domain 9 [Source:HGNC Symbol;Acc:HGNC:23463]
ENSG00000250982	-0,1851631	0,14481576	0,31655308	0,58706104	0,80401	GAPDHP35	glyceraldehyde 3 phosphate dehydrogenase pseudogene 35 [Source:HGNC Symbol;Acc:HGNC:37789]
ENSG00000198722	0,08620772	5,07014109	0,31643734	0,58712828	0,80402672	UNC13B	unc-13 homolog B [Source:HGNC Symbol;Acc:HGNC:12566]
ENSG00000178209	-0,2684052	4,94850863	0,31595894	0,58740637	0,80433215	PLEC	plectin [Source:HGNC Symbol;Acc:HGNC:9069]
ENSG00000108561	0,09569421	9,63215656	0,31550884	0,58766828	0,80446457	C1QBP	complement C1q binding protein [Source:HGNC Symbol;Acc:HGNC:1243]
ENSG00000122565	-0,1071865	7,66353336	0,31566414	0,58757788	0,80446457	CBX3	chromobox 3 [Source:HGNC Symbol;Acc:HGNC:1553]
ENSG00000185615	0,21562388	-0,0740758	0,31553676	0,58765202	0,80446457	PDIA2	protein disulfide isomerase family A member 2 [Source:HGNC Symbol;Acc:HGNC:14180]
ENSG00000156876	-0,0971241	2,29475645	0,3154023	0,5877303	0,80447409	SASS6	SAS-6 centriolar assembly protein [Source:HGNC Symbol;Acc:HGNC:25403]
ENSG00000172738	0,23706987	-0,1431595	0,31518442	0,5878572	0,80457241	TMEM217	transmembrane protein 217 [Source:HGNC Symbol;Acc:HGNC:21238]
ENSG00000188613	-0,3170197	-1,426485	0,31465057	0,58816838	0,80492289	NANOS1	nanos C2HC-type zinc finger 1 [Source:HGNC Symbol;Acc:HGNC:23044]
ENSG00000011405	0,1178616	3,60574165	0,31413787	0,58846755	0,80495528	PIK3C2A	phosphatidylinositol-4-phosphate 3-kinase catalytic subunit type 2 alpha [Source:HGNC Symbol;Acc:HGNC:8971]
ENSG00000122359	0,07612621	6,8823573	0,31433917	0,58835005	0,80495528	ANXA11	annexin A11 [Source:HGNC Symbol;Acc:HGNC:535]
ENSG00000152348	-0,1292385	1,99187926	0,31419925	0,58843172	0,80495528	ATG10	autophagy related 10 [Source:HGNC Symbol;Acc:HGNC:20315]
ENSG00000196912	-0,1623258	0,76293683	0,31428815	0,58837982	0,80495528	ANKRD36B	ankyrin repeat domain 36B [Source:HGNC Symbol;Acc:HGNC:29333]
ENSG00000273654	0,20195867	0,00442692	0,3143751	0,58832908	0,80495528	AC020904.2	citrate synthase (CS) pseudogene
ENSG00000087884	-0,0835437	4,12711682	0,31397035	0,58856538	0,80497804	AAMDC	adipogenesis associated Mth938 domain containing [Source:HGNC Symbol;Acc:HGNC:30205]
ENSG00000232445	-0,119388	2,89697028	0,31388083	0,58861766	0,80497804	EMSLR	E2F1 mRNA stabilizing lncRNA [Source:HGNC Symbol;Acc:HGNC:54408]
ENSG00000243176	0,20490203	-0,4207645	0,31382636	0,58864949	0,80497804	AC092944.1	novel transcript
ENSG00000140043	0,18134745	-0,0723002	0,31332352	0,58894342	0,80515389	PTGR2	prostaglandin reductase 2 [Source:HGNC Symbol;Acc:HGNC:20149]
ENSG00000145348	-0,0863216	3,95323214	0,3133646	0,5889194	0,80515389	TBCK	TBC1 domain containing kinase [Source:HGNC Symbol;Acc:HGNC:28261]
ENSG00000170191	0,09048522	4,52218379	0,31340096	0,58889814	0,80515389	NANP	N-acetylneuraminic acid phosphatase [Source:HGNC Symbol;Acc:HGNC:16140]
ENSG00000108262	-0,0802892	5,0380869	0,31250895	0,58942025	0,80536511	GIT1	GIT ArfGAP 1 [Source:HGNC Symbol;Acc:HGNC:4272]
ENSG00000130475	-0,0983632	2,66379451	0,31257014	0,5893844	0,80536511	FCHO1	FCH and mu domain containing endocytic adaptor 1 [Source:HGNC Symbol;Acc:HGNC:29002]
ENSG00000135617	-0,0988155	6,09169589	0,31249824	0,58942653	0,80536511	PRADC1	protease associated domain containing 1 [Source:HGNC Symbol;Acc:HGNC:16047]
ENSG00000142546	-0,0747487	6,0031011	0,31258365	0,58937649	0,80536511	NOSIP	nitric oxide synthase interacting protein [Source:HGNC Symbol;Acc:HGNC:17946]
ENSG00000143498	-0,1008323	2,34968299	0,31249455	0,58942869	0,80536511	TAF1A	TATA-box binding protein associated factor, RNA polymerase I subunit A [Source:HGNC Symbol;Acc:HGNC:11532]

ENSG00000167543	-0,0840509	4,88425026	0,31288918	0,58919757	0,80536511	TP53I13	tumor protein p53 inducible protein 13 [Source:HGNC Symbol;Acc:HGNC:25102]
ENSG00000100938	-0,0753292	7,5711159	0,31220816	0,58959653	0,80544378	GMPR2	guanosine monophosphate reductase 2 [Source:HGNC Symbol;Acc:HGNC:4377]
ENSG00000132716	0,0921125	3,41518651	0,31221822	0,58959064	0,80544378	DCAF8	DDB1 and CUL4 associated factor 8 [Source:HGNC Symbol;Acc:HGNC:24891]
ENSG00000092295	-0,2281321	0,87275616	0,31193382	0,58975742	0,80545129	TGM1	transglutaminase 1 [Source:HGNC Symbol;Acc:HGNC:11777]
ENSG00000139668	0,07834946	5,57874852	0,31182276	0,58982257	0,80545129	WDFY2	WD repeat and FYVE domain containing 2 [Source:HGNC Symbol;Acc:HGNC:20482]
ENSG00000140284	0,08706993	4,96642965	0,31188317	0,58978713	0,80545129	SLC27A2	solute carrier family 27 member 2 [Source:HGNC Symbol;Acc:HGNC:10996]
ENSG00000173960	0,08713039	4,1729255	0,31200261	0,58971707	0,80545129	UBXN2A	UBX domain protein 2A [Source:HGNC Symbol;Acc:HGNC:27265]
ENSG00000162929	0,09322924	3,21540405	0,31151822	0,59000131	0,80562008	KIAA1841	KIAA1841 [Source:HGNC Symbol;Acc:HGNC:29387]
ENSG00000145725	-0,0823602	3,81561767	0,31106562	0,59026718	0,80583247	PPIP5K2	diphosphoinositol pentakisphosphate kinase 2 [Source:HGNC Symbol;Acc:HGNC:29035]
ENSG00000151338	0,08881535	3,87890217	0,31110823	0,59024214	0,80583247	MIPOL1	mirror-image polydactyly 1 [Source:HGNC Symbol;Acc:HGNC:21460]
ENSG00000261220	0,2554572	-0,6690776	0,31059186	0,59054575	0,80613743	AC103706.1	novel transcript
ENSG00000142453	0,10237279	6,21521294	0,31019259	0,59078073	0,80620112	CARM1	coactivator associated arginine methyltransferase 1 [Source:HGNC Symbol;Acc:HGNC:23393]
ENSG00000144504	0,11779816	2,6058545	0,31031653	0,59070777	0,80620112	ANKMY1	ankyrin repeat and MYND domain containing 1 [Source:HGNC Symbol;Acc:HGNC:20987]
ENSG00000166734	0,08592497	5,29103784	0,31013755	0,59081314	0,80620112	GOLM2	golgi membrane protein 2 [Source:HGNC Symbol;Acc:HGNC:24892]
ENSG00000187017	-0,0905569	5,32518135	0,31027879	0,59072999	0,80620112	ESPN	espin [Source:HGNC Symbol;Acc:HGNC:13281]
ENSG00000113758	0,12740153	5,59796011	0,30962146	0,59111722	0,80624783	DBN1	drebrin 1 [Source:HGNC Symbol;Acc:HGNC:2695]
ENSG00000168003	0,12298619	8,69035409	0,30961112	0,59112332	0,80624783	SLC3A2	solute carrier family 3 member 2 [Source:HGNC Symbol;Acc:HGNC:11026]
ENSG00000241255	-0,2244665	-0,9574038	0,30970676	0,59106694	0,80624783	AL136126.1	ribosomal protein S24 (RPS24) pseudogene
ENSG00000260276	-0,1509897	1,05265997	0,30997702	0,59090769	0,80624783	AC022167.2	novel transcript, antisense to CARHSP1
ENSG00000271647	-0,3276719	-1,8149444	0,30982931	0,59099472	0,80624783	KRT8P47	keratin 8 pseudogene 47 [Source:HGNC Symbol;Acc:HGNC:44461]
ENSG00000231690	0,15464445	0,54573981	0,30936889	0,59126615	0,80636737	LINC00574	long intergenic non-protein coding RNA 574 [Source:HGNC Symbol;Acc:HGNC:21598]
ENSG00000205517	0,18440153	0,20949527	0,30896097	0,59150687	0,80654507	RGL3	ral guanine nucleotide dissociation stimulator like 3 [Source:HGNC Symbol;Acc:HGNC:30282]
ENSG00000242294	0,41111607	-1,2408443	0,30899537	0,59148656	0,80654507	STAG3L5P	stromal antigen 3-like 5 pseudogene [Source:HGNC Symbol;Acc:HGNC:48896]
ENSG00000119328	0,11709939	3,16776702	0,30876562	0,59162222	0,80655696	ABITRAM	actin binding transcription modulator [Source:HGNC Symbol;Acc:HGNC:1364]
ENSG00000137133	-0,2285104	-0,7519273	0,3087592	0,59162601	0,80655696	HINT2	histidine triad nucleotide binding protein 2 [Source:HGNC Symbol;Acc:HGNC:18344]
ENSG00000160213	-0,0975159	6,41435577	0,30849208	0,59178382	0,80669682	CSTB	cystatin B [Source:HGNC Symbol;Acc:HGNC:2482]
ENSG00000158122	0,09202424	4,44894387	0,30817464	0,59197147	0,80671063	PRXL2C	peroxiredoxin like 2C [Source:HGNC Symbol;Acc:HGNC:16881]
ENSG00000198198	-0,1864803	2,32923028	0,30823577	0,59193533	0,80671063	SZT2	SZT2 subunit of KICSTOR complex [Source:HGNC Symbol;Acc:HGNC:29040]
ENSG00000198853	-0,1402562	2,5835908	0,30810133	0,59201483	0,80671063	RUSC2	RUN and SH3 domain containing 2 [Source:HGNC Symbol;Acc:HGNC:23625]
ENSG00000254681	0,27178046	1,22996984	0,30816997	0,59197424	0,80671063	PKD1P5	polycystin 1, transient receptor potential channel interacting pseudogene 5 [Source:HGNC Symbol;Acc:HGNC:300
ENSG00000125257	0,10218605	3,34036225	0,30789226	0,59213852	0,80672868	ABCC4	ATP binding cassette subfamily C member 4 [Source:HGNC Symbol;Acc:HGNC:55]
ENSG00000197302	-0,0968207	3,36056733	0,30793609	0,59211258	0,80672868	ZNF720	zinc finger protein 720 [Source:HGNC Symbol;Acc:HGNC:26987]
ENSG00000104228	0,08891357	4,05450757	0,30753036	0,59235274	0,80694528	TRIM35	tripartite motif containing 35 [Source:HGNC Symbol;Acc:HGNC:16285]
ENSG00000080618	-0,0829139	6,41957622	0,30677186	0,59280228	0,80718914	CPB2	carboxypeptidase B2 [Source:HGNC Symbol;Acc:HGNC:2300]
ENSG00000102743	-0,0903	4,80738092	0,30676304	0,59280751	0,80718914	SLC25A15	solute carrier family 25 member 15 [Source:HGNC Symbol;Acc:HGNC:10985]
ENSG00000105829	-0,0891182	4,10001579	0,30634703	0,59305439	0,80718914	BET1	Bet1 golgi vesicular membrane trafficking protein [Source:HGNC Symbol;Acc:HGNC:14562]
ENSG00000119421	-0,0994595	5,99221465	0,30629669	0,59308428	0,80718914	NDUFA8	NADH:ubiquinone oxidoreductase subunit A8 [Source:HGNC Symbol;Acc:HGNC:7692]
ENSG00000128923	0,10429145	1,94023478	0,30682399	0,59277136	0,80718914	MINDY2	MINDY lysine 48 deubiquitinase 2 [Source:HGNC Symbol;Acc:HGNC:26954]
ENSG00000162688	-0,0977471	3,08503119	0,30653748	0,59294135	0,80718914	AGL	amylo-alpha-1, 6-glucosidase, 4-alpha-glucanotransferase [Source:HGNC Symbol;Acc:HGNC:321]
ENSG00000162946	0,13639851	0,9890109	0,30649863	0,5929644	0,80718914	DISC1	DISC1 scaffold protein [Source:HGNC Symbol;Acc:HGNC:2888]
ENSG00000185619	-0,0974789	3,61458685	0,30654741	0,59293545	0,80718914	PCGF3	polycomb group ring finger 3 [Source:HGNC Symbol;Acc:HGNC:10066]
ENSG00000228889	-0,172946	-0,0112235	0,3064357	0,59300175	0,80718914	UBAC2-AS1	UBAC2 antisense RNA 1 [Source:HGNC Symbol;Acc:HGNC:42502]
ENSG00000240298	-0,2838871	-1,8166351	0,30630144	0,59308146	0,80718914	RPL36AP40	ribosomal protein L36a pseudogene 40 [Source:HGNC Symbol;Acc:HGNC:36300]
ENSG00000165804	0,0777422	5,15978341	0,30584133	0,59335479	0,80742221	ZNF219	zinc finger protein 219 [Source:HGNC Symbol;Acc:HGNC:13011]

ENSG00000214207	-0,1758678	0,14641828	0,30582236	0,59336606	0,80742221	KRT18P10	keratin 18 pseudogene 10 [Source:HGNC Symbol;Acc:HGNC:23962]
ENSG00000213015	-0,0904578	3,74856666	0,30572295	0,59342516	0,80742741	ZNF580	zinc finger protein 580 [Source:HGNC Symbol;Acc:HGNC:29473]
ENSG00000109667	-0,0751861	5,48045445	0,30461233	0,59408623	0,80779443	SLC2A9	solute carrier family 2 member 9 [Source:HGNC Symbol;Acc:HGNC:13446]
ENSG00000123561	-0,0777618	8,38088297	0,30470379	0,59403173	0,80779443	SERPINA7	serpin family A member 7 [Source:HGNC Symbol;Acc:HGNC:11583]
ENSG00000127616	-0,2221929	6,25029154	0,30462826	0,59407674	0,80779443	SMARCA4	SWI/SNF related, matrix associated, actin dependent regulator of chromatin, subfamily a, member 4 [Source:HGN
ENSG00000143028	0,32432396	-1,8082682	0,30452672	0,59413725	0,80779443	SYPL2	synaptophysin like 2 [Source:HGNC Symbol;Acc:HGNC:27638]
ENSG00000188818	0,16126615	0,38329297	0,30493438	0,59389437	0,80779443	ZDHHC11	zinc finger DHHC-type containing 11 [Source:HGNC Symbol;Acc:HGNC:19158]
ENSG00000214719	0,19979748	-0,6794024	0,30502037	0,59384317	0,80779443	AC005562.1	novel transcript
ENSG00000240230	-0,0831323	4,41648413	0,30459966	0,59409378	0,80779443	COX19	cytochrome c oxidase assembly factor COX19 [Source:HGNC Symbol;Acc:HGNC:28074]
ENSG00000260643	0,30487974	-1,437121	0,30460111	0,59409292	0,80779443	AC092718.3	novel protein
ENSG00000064607	0,0878478	5,62808971	0,30351515	0,59474087	0,80810309	SUGP2	SURP and G-patch domain containing 2 [Source:HGNC Symbol;Acc:HGNC:18641]
ENSG00000104833	-0,2279349	-0,9664232	0,30331224	0,59486211	0,80810309	TUBB4A	tubulin beta 4A class IVa [Source:HGNC Symbol;Acc:HGNC:20774]
ENSG00000114120	0,0933647	3,40695514	0,30372231	0,59461715	0,80810309	SLC25A36	solute carrier family 25 member 36 [Source:HGNC Symbol;Acc:HGNC:25554]
ENSG00000142627	-0,1318172	7,70217711	0,30368243	0,59464096	0,80810309	EPHA2	EPH receptor A2 [Source:HGNC Symbol;Acc:HGNC:3386]
ENSG00000158545	0,11641712	5,89601683	0,3033498	0,59483967	0,80810309	ZC3H18	zinc finger CCCH-type containing 18 [Source:HGNC Symbol;Acc:HGNC:25091]
ENSG00000164306	-0,1122891	2,32400676	0,303396	0,59481206	0,80810309	PRIMPOL	primase and DNA directed polymerase [Source:HGNC Symbol;Acc:HGNC:26575]
ENSG00000169714	-0,0753361	8,81932262	0,30385499	0,59453793	0,80810309	CNBP	CCHC-type zinc finger nucleic acid binding protein [Source:HGNC Symbol;Acc:HGNC:13164]
ENSG00000213676	0,09965334	5,09632557	0,30344261	0,59478421	0,80810309	ATF6B	activating transcription factor 6 beta [Source:HGNC Symbol;Acc:HGNC:2349]
ENSG00000278948	0,31891661	-0,4594763	0,30379233	0,59457534	0,80810309	AL031587.5	TEC
ENSG00000103257	0,09581133	8,43118996	0,30283059	0,59515011	0,80834399	SLC7A5	solute carrier family 7 member 5 [Source:HGNC Symbol;Acc:HGNC:11063]
ENSG00000181220	0,09172398	3,37986714	0,30288314	0,59511868	0,80834399	ZNF746	zinc finger protein 746 [Source:HGNC Symbol;Acc:HGNC:21948]
ENSG00000233937	0,11978569	1,56093286	0,30263951	0,59526445	0,80842413	CTC-338M12.4	uncharacterized LOC101928649 [Source:NCBI gene (formerly Entrezgene);Acc:101928649]
ENSG00000122085	0,0832878	5,37189005	0,30227499	0,59548272	0,80849507	MTERF4	mitochondrial transcription termination factor 4 [Source:HGNC Symbol;Acc:HGNC:28785]
ENSG00000136950	0,08192675	5,91569263	0,30232195	0,59545459	0,80849507	ARPC5L	actin related protein 2/3 complex subunit 5 like [Source:HGNC Symbol;Acc:HGNC:23366]
ENSG00000181019	0,0868185	9,62902763	0,3023387	0,59544456	0,80849507	NQO1	NAD(P)H quinone dehydrogenase 1 [Source:HGNC Symbol;Acc:HGNC:2874]
ENSG00000263465	-0,0906772	4,99171658	0,30211165	0,59558057	0,80855279	SRSF8	serine and arginine rich splicing factor 8 [Source:HGNC Symbol;Acc:HGNC:16988]
ENSG00000013810	-0,080849	6,62552255	0,3015605	0,59591103	0,80859247	TACC3	transforming acidic coiled-coil containing protein 3 [Source:HGNC Symbol;Acc:HGNC:11524]
ENSG00000165915	-0,0829427	4,97694939	0,30185108	0,59573676	0,80859247	SLC39A13	solute carrier family 39 member 13 [Source:HGNC Symbol;Acc:HGNC:20859]
ENSG00000196155	-0,1037432	2,92152329	0,30162478	0,59587246	0,80859247	PLEKHG4	pleckstrin homology and RhoGEF domain containing G4 [Source:HGNC Symbol;Acc:HGNC:24501]
ENSG00000198918	-0,0845556	7,79319406	0,30162996	0,59586936	0,80859247	RPL39	ribosomal protein L39 [Source:HGNC Symbol;Acc:HGNC:10350]
ENSG00000279865	-0,2367732	-0,6678542	0,30182576	0,59575194	0,80859247	AC006511.3	TEC
ENSG00000288559	-0,2312581	-1,1326702	0,30150905	0,5959419	0,80859247	AC016394.2	novel transcript
ENSG00000053372	0,07206886	7,14301724	0,30111205	0,5961802	0,80872138	MRTO4	MRT4 homolog, ribosome maturation factor [Source:HGNC Symbol;Acc:HGNC:18477]
ENSG00000153561	0,08525313	4,96704957	0,30107412	0,59620298	0,80872138	RMND5A	required for meiotic nuclear division 5 homolog A [Source:HGNC Symbol;Acc:HGNC:25850]
ENSG00000187607	0,14334184	1,1402047	0,30122029	0,5961152	0,80872138	ZNF286A	zinc finger protein 286A [Source:HGNC Symbol;Acc:HGNC:13501]
ENSG00000085872	0,14057599	5,40274259	0,30070171	0,59642673	0,80880874	CHERP	calcium homeostasis endoplasmic reticulum protein [Source:HGNC Symbol;Acc:HGNC:16930]
ENSG00000160211	-0,1156268	7,68638077	0,30069049	0,59643347	0,80880874	G6PD	glucose-6-phosphate dehydrogenase [Source:HGNC Symbol;Acc:HGNC:4057]
ENSG00000179820	0,1335898	6,59864437	0,30077081	0,5963852	0,80880874	MYADM	myeloid associated differentiation marker [Source:HGNC Symbol;Acc:HGNC:7544]
ENSG00000130701	-0,223929	-0,5346936	0,30037283	0,59662448	0,80891759	RBBP8NL	RBBP8 N-terminal like [Source:HGNC Symbol;Acc:HGNC:16144]
ENSG00000220205	-0,1327604	1,50527042	0,30044586	0,59658056	0,80891759	VAMP2	vesicle associated membrane protein 2 [Source:HGNC Symbol;Acc:HGNC:12643]
ENSG00000101846	0,14843295	0,83448191	0,3001053	0,59678545	0,80906074	STS	steroid sulfatase [Source:HGNC Symbol;Acc:HGNC:11425]
ENSG00000031698	-0,1145891	7,83769993	0,29902938	0,59743376	0,80948929	SARS1	seryl-tRNA synthetase 1 [Source:HGNC Symbol;Acc:HGNC:10537]
ENSG00000148346	-0,3262255	-1,6344394	0,29893713	0,59748943	0,80948929	LCN2	lipocalin 2 [Source:HGNC Symbol;Acc:HGNC:6526]
ENSG00000159131	-0,1283855	7,21990233	0,29929183	0,59727548	0,80948929	GART	phosphoribosylglycinamide formyltransferase, phosphoribosylglycinamide synthetase, phosphoribosylaminoimida

ENSG00000167978	0,28613644	8,02661229	0,29904039	0,59742713	0,80948929	SRRM2	serine/arginine repetitive matrix 2 [Source:HGNC Symbol;Acc:HGNC:16639]
ENSG00000181610	-0,07086	7,85423067	0,29936056	0,59723404	0,80948929	MRPS23	mitochondrial ribosomal protein S23 [Source:HGNC Symbol;Acc:HGNC:14509]
ENSG00000185880	-0,1601957	0,85104797	0,29904319	0,59742544	0,80948929	TRIM69	tripartite motif containing 69 [Source:HGNC Symbol;Acc:HGNC:17857]
ENSG00000247828	-0,1108275	1,47343389	0,29901398	0,59744306	0,80948929	TMEM161B-AS1	TMEM161B antisense RNA 1 [Source:HGNC Symbol;Acc:HGNC:43839]
ENSG00000168936	0,08146043	5,22525348	0,29880563	0,59756879	0,80952174	TMEM129	transmembrane protein 129, E3 ubiquitin ligase [Source:HGNC Symbol;Acc:HGNC:25137]
ENSG00000255857	0,1844208	1,72278117	0,29859018	0,59769886	0,80962287	PXN-AS1	PXN antisense RNA 1 [Source:HGNC Symbol;Acc:HGNC:44123]
ENSG00000100065	0,09923904	6,49992794	0,29826125	0,59789756	0,80968912	CARD10	caspase recruitment domain family member 10 [Source:HGNC Symbol;Acc:HGNC:16422]
ENSG00000116791	-0,074852	6,73228242	0,2980506	0,59802489	0,80968912	CRYZ	crystallin zeta [Source:HGNC Symbol;Acc:HGNC:2419]
ENSG00000119688	-0,0787866	4,7535875	0,29832901	0,59785662	0,80968912	ABCD4	ATP binding cassette subfamily D member 4 [Source:HGNC Symbol;Acc:HGNC:68]
ENSG00000167487	-0,111141	2,28075843	0,2980893	0,59800149	0,80968912	KLHL26	kelch like family member 26 [Source:HGNC Symbol;Acc:HGNC:25623]
ENSG00000274211	0,12581456	3,71107575	0,29820654	0,59793063	0,80968912	SOCS7	suppressor of cytokine signaling 7 [Source:HGNC Symbol;Acc:HGNC:29846]
ENSG00000240418	-0,2621974	-0,6204653	0,29790888	0,59811059	0,80973011	AC020917.1	ribosomal protein S2 (RPS2) pseudogene
ENSG00000120306	-0,0740416	5,99469507	0,29763481	0,59827639	0,80983006	CYSTM1	cysteine rich transmembrane module containing 1 [Source:HGNC Symbol;Acc:HGNC:30239]
ENSG00000174939	-0,0814499	5,0340248	0,29760359	0,59829528	0,80983006	ASPHD1	aspartate beta-hydroxylase domain containing 1 [Source:HGNC Symbol;Acc:HGNC:27380]
ENSG00000198231	-0,0954751	6,85452121	0,29700579	0,59865731	0,81024501	DDX42	DEAD-box helicase 42 [Source:HGNC Symbol;Acc:HGNC:18676]
ENSG00000092820	-0,1516828	7,21418798	0,29639809	0,59902583	0,81066867	EZR	ezrin [Source:HGNC Symbol;Acc:HGNC:12691]
ENSG00000234981	-0,1464066	0,34083177	0,29612993	0,5991886	0,81073874	AC244034.1	ribosomal protein L7a (RPL7A) pseudogene
ENSG00000266258	-0,1954873	-0,205297	0,29618468	0,59915535	0,81073874	LINC01909	long intergenic non-protein coding RNA 1909 [Source:HGNC Symbol;Acc:HGNC:52728]
ENSG00000143061	0,43174947	-1,9408634	0,29551749	0,59956071	0,81083333	IGSF3	immunoglobulin superfamily member 3 [Source:HGNC Symbol;Acc:HGNC:5950]
ENSG00000158711	-0,0888961	2,51119118	0,295628	0,59949353	0,81083333	ELK4	ETS transcription factor ELK4 [Source:HGNC Symbol;Acc:HGNC:3326]
ENSG00000166337	0,30603947	-1,6425294	0,29546682	0,59959152	0,81083333	TAF10	TATA-box binding protein associated factor 10 [Source:HGNC Symbol;Acc:HGNC:11543]
ENSG00000166592	-0,2216536	0,47232974	0,29580625	0,5993852	0,81083333	RRAD	RRAD, Ras related glycolysis inhibitor and calcium channel regulator [Source:HGNC Symbol;Acc:HGNC:10446]
ENSG00000233223	-0,1135648	2,26795232	0,29586194	0,59935136	0,81083333	AC016876.1	novel transcript, antisense to MPDU1 and CD68
ENSG00000263272	0,27431936	-1,6019606	0,29571903	0,5994382	0,81083333	AC004148.1	novel transcript, antisense to RPAIN
ENSG00000074219	0,08709544	3,89651365	0,29522063	0,59974126	0,8108626	TEAD2	TEA domain transcription factor 2 [Source:HGNC Symbol;Acc:HGNC:11715]
ENSG00000075275	-0,1586767	0,75593148	0,29492405	0,59992177	0,8108626	CELSR1	cadherin EGF LAG seven-pass G-type receptor 1 [Source:HGNC Symbol;Acc:HGNC:1850]
ENSG00000100626	-0,202955	0,29369165	0,29384878	0,60057719	0,8108626	GALNT16	polypeptide N-acetylgalactosaminyltransferase 16 [Source:HGNC Symbol;Acc:HGNC:23233]
ENSG00000101181	0,08110656	5,44184361	0,29422301	0,6003489	0,8108626	MTG2	mitochondrial ribosome associated GTPase 2 [Source:HGNC Symbol;Acc:HGNC:16239]
ENSG00000103051	-0,0810318	5,61179329	0,29394474	0,60051863	0,8108626	COG4	component of oligomeric golgi complex 4 [Source:HGNC Symbol;Acc:HGNC:18620]
ENSG00000108469	-0,0908843	4,44951919	0,29441961	0,60022904	0,8108626	RECQL5	RecQ like helicase 5 [Source:HGNC Symbol;Acc:HGNC:9950]
ENSG00000119636	0,16385435	0,04282958	0,29461311	0,60011113	0,8108626	BBOF1	basal body orientation factor 1 [Source:HGNC Symbol;Acc:HGNC:19855]
ENSG00000126524	-0,0920535	6,15160759	0,29436247	0,60026387	0,8108626	SBDS	SBDS ribosome maturation factor [Source:HGNC Symbol;Acc:HGNC:19440]
ENSG00000128294	0,09451967	4,58770446	0,29392872	0,60052841	0,8108626	TPST2	tyrosylprotein sulfotransferase 2 [Source:HGNC Symbol;Acc:HGNC:12021]
ENSG00000142197	0,10139836	2,38849877	0,2945096	0,6001742	0,8108626	DOP1B	DOP1 leucine zipper like protein B [Source:HGNC Symbol;Acc:HGNC:1291]
ENSG00000154511	0,08893866	4,10071742	0,29420079	0,60036245	0,8108626	DIPK1A	divergent protein kinase domain 1A [Source:HGNC Symbol;Acc:HGNC:32213]
ENSG00000159023	0,12187026	5,08238866	0,29441836	0,60022981	0,8108626	EPB41	erythrocyte membrane protein band 4.1 [Source:HGNC Symbol;Acc:HGNC:3377]
ENSG00000173581	0,09240677	3,66086663	0,29379135	0,60061224	0,8108626	CCDC106	coiled-coil domain containing 106 [Source:HGNC Symbol;Acc:HGNC:30181]
ENSG00000180228	-0,078339	5,78569489	0,29380138	0,60060612	0,8108626	PRKRA	protein activator of interferon induced protein kinase EIF2AK2 [Source:HGNC Symbol;Acc:HGNC:9438]
ENSG00000233287	-0,1754961	0,17558693	0,29440883	0,60023561	0,8108626	AC009362.1	ribosomal protein L27 (RPL27) pseudogene
ENSG00000248546	-0,2422534	-1,4698077	0,29516438	0,59977549	0,8108626	ANP32C	acidic nuclear phosphoprotein 32 family member C [Source:HGNC Symbol;Acc:HGNC:16675]
ENSG00000270605	-0,3938039	-1,2488763	0,29486713	0,59995642	0,8108626	AL353622.1	novel transcript
ENSG00000272221	0,16211785	0,54982672	0,29443676	0,6002186	0,8108626	AL645933.2	novel transcript
ENSG00000170348	-0,0809529	8,41766228	0,29353325	0,60076982	0,81092546	TMED10	transmembrane p24 trafficking protein 10 [Source:HGNC Symbol;Acc:HGNC:16998]
ENSG00000259471	-0,2574179	-1,151483	0,29354805	0,60076078	0,81092546	LINC01169	long intergenic non-protein coding RNA 1169 [Source:HGNC Symbol;Acc:HGNC:49541]

ENSG00000225921	-0,0882427	7,71044056	0,29339318	0,60085537	0,81096601	NOL7	nucleolar protein 7 [Source:HGNC Symbol;Acc:HGNC:21040]
ENSG00000117262	0,10143688	3,40936271	0,29299114	0,60110109	0,81100647	GPR89A	G protein-coupled receptor 89A [Source:HGNC Symbol;Acc:HGNC:31984]
ENSG00000226314	0,16498943	0,29132335	0,29305182	0,60106399	0,81100647	ZNF192P1	zinc finger protein 192 pseudogene 1 [Source:HGNC Symbol;Acc:HGNC:18777]
ENSG00000241612	0,23379975	-1,357419	0,29304494	0,60106819	0,81100647	AC114728.1	ribosomal protein L21 (RPL21) pseudogene
ENSG00000247240	0,13342231	0,67378824	0,29298081	0,6011074	0,81100647	UBL7-AS1	UBL7 antisense RNA 1 (head to head) [Source:HGNC Symbol;Acc:HGNC:48614]
ENSG00000048471	-0,1488627	2,61979193	0,29212154	0,60163332	0,81164108	SNX29	sorting nexin 29 [Source:HGNC Symbol;Acc:HGNC:30542]
ENSG00000239697	-0,1162545	1,20361535	0,29196551	0,60172893	0,81169511	TNFSF12	TNF superfamily member 12 [Source:HGNC Symbol;Acc:HGNC:11927]
ENSG00000112592	0,09223306	4,87142625	0,29122516	0,60218304	0,81171559	TBP	TATA-box binding protein [Source:HGNC Symbol;Acc:HGNC:11588]
ENSG00000114670	0,19579349	0,35425299	0,29088182	0,6023939	0,81171559	NEK11	NIMA related kinase 11 [Source:HGNC Symbol;Acc:HGNC:18593]
ENSG00000128283	0,08489483	6,64252769	0,29084141	0,60241872	0,81171559	CDC42EP1	CDC42 effector protein 1 [Source:HGNC Symbol;Acc:HGNC:17014]
ENSG00000130766	0,08267751	5,28121084	0,2909266	0,60236639	0,81171559	SESN2	sestrin 2 [Source:HGNC Symbol;Acc:HGNC:20746]
ENSG00000132967	-0,1114958	2,5010467	0,29104089	0,60229619	0,81171559	HMGB1P5	high mobility group box 1 pseudogene 5 [Source:HGNC Symbol;Acc:HGNC:4997]
ENSG00000158552	0,07845038	5,32138451	0,29076376	0,60246644	0,81171559	ZFAND2B	zinc finger AN1-type containing 2B [Source:HGNC Symbol;Acc:HGNC:25206]
ENSG00000172830	0,09425002	3,04004292	0,29136487	0,60209729	0,81171559	SSH3	slingshot protein phosphatase 3 [Source:HGNC Symbol;Acc:HGNC:30581]
ENSG00000173614	-0,0943093	2,82828085	0,29151983	0,60200221	0,81171559	NMNAT1	nicotinamide nucleotide adenylyltransferase 1 [Source:HGNC Symbol;Acc:HGNC:17877]
ENSG00000175279	-0,2021254	-0,4671544	0,29113751	0,60223685	0,81171559	CENPS	centromere protein S [Source:HGNC Symbol;Acc:HGNC:23163]
ENSG00000180229	-0,1502794	1,08440305	0,29108355	0,60226999	0,81171559	HERC2P3	hect domain and RLD 2 pseudogene 3 [Source:HGNC Symbol;Acc:HGNC:4871]
ENSG00000183199	-0,1472051	3,10670269	0,29178918	0,60183702	0,81171559	HSP90AB3P	heat shock protein 90 alpha family class B member 3, pseudogene [Source:HGNC Symbol;Acc:HGNC:5259]
ENSG00000239830	0,30401172	-1,4048059	0,29150675	0,60201024	0,81171559	RPS4XP22	ribosomal protein S4X pseudogene 22 [Source:HGNC Symbol;Acc:HGNC:36125]
ENSG00000249572	-0,3321364	-1,8000234	0,29080962	0,60243826	0,81171559	AC034231.1	novel transcript
ENSG00000137462	0,31698116	-1,531861	0,29054557	0,60260055	0,81182142	TLR2	toll like receptor 2 [Source:HGNC Symbol;Acc:HGNC:11848]
ENSG00000185885	-0,2899246	-1,911813	0,28998393	0,60294608	0,81216642	IFITM1	interferon induced transmembrane protein 1 [Source:HGNC Symbol;Acc:HGNC:5412]
ENSG00000234147	0,15934948	1,53866403	0,28994862	0,60296782	0,81216642	AL035446.1	novel transcript
ENSG00000113812	0,08142857	5,04372251	0,28946424	0,6032662	0,81241343	ACTR8	actin related protein 8 [Source:HGNC Symbol;Acc:HGNC:14672]
ENSG00000160305	-0,1219952	4,42046606	0,28938013	0,60331804	0,81241343	DIP2A	disco interacting protein 2 homolog A [Source:HGNC Symbol;Acc:HGNC:17217]
ENSG00000166974	-0,084962	5,56140872	0,28939611	0,60330819	0,81241343	MAPRE2	microtubule associated protein RP/EB family member 2 [Source:HGNC Symbol;Acc:HGNC:6891]
ENSG00000169641	0,16516952	4,17654186	0,2892662	0,60338828	0,81243313	LUZP1	leucine zipper protein 1 [Source:HGNC Symbol;Acc:HGNC:14985]
ENSG00000225791	0,10788804	2,01877529	0,28900581	0,60354889	0,81257448	TRAM2-AS1	TRAM2 antisense RNA 1 (head to head) [Source:HGNC Symbol;Acc:HGNC:48663]
ENSG00000112234	0,09167879	2,93441672	0,28880717	0,60367147	0,81266462	FBXL4	F-box and leucine rich repeat protein 4 [Source:HGNC Symbol;Acc:HGNC:13601]
ENSG00000184500	0,09338577	6,46119625	0,28827715	0,60399882	0,81303039	PROS1	protein S [Source:HGNC Symbol;Acc:HGNC:9456]
ENSG00000228981	-0,2273455	-1,2637038	0,28806213	0,60413174	0,81313438	AC097658.1	ribosomal protein S2 (RPS2) pseudogene
ENSG00000100068	0,09366352	2,2423109	0,28791878	0,60422038	0,81317878	LRP5L	LDL receptor related protein 5 like [Source:HGNC Symbol;Acc:HGNC:25323]
ENSG00000245317	0,23552224	-1,3125404	0,28773744	0,60433256	0,81325483	AC008393.1	novel transcript
ENSG00000106330	-0,1274388	3,64376243	0,2874869	0,60448763	0,81330452	MOSPD3	motile sperm domain containing 3 [Source:HGNC Symbol;Acc:HGNC:25078]
ENSG00000148297	0,09203393	3,76930149	0,28740797	0,6045365	0,81330452	MED22	mediator complex subunit 22 [Source:HGNC Symbol;Acc:HGNC:11477]
ENSG00000169045	0,08818869	8,56842879	0,28743378	0,60452052	0,81330452	HNRNPH1	heterogeneous nuclear ribonucleoprotein H1 [Source:HGNC Symbol;Acc:HGNC:5041]
ENSG00000184209	-0,080027	4,23308376	0,28730523	0,60460013	0,81331522	SNRNP35	small nuclear ribonucleoprotein U11/U12 subunit 35 [Source:HGNC Symbol;Acc:HGNC:30852]
ENSG00000168758	-0,0994689	5,6037868	0,28718384	0,60467532	0,81334148	SEMA4C	semaphorin 4C [Source:HGNC Symbol;Acc:HGNC:10731]
ENSG00000132182	-0,1831848	6,43448229	0,28680984	0,60490712	0,81353839	NUP210	nucleoporin 210 [Source:HGNC Symbol;Acc:HGNC:30052]
ENSG00000187840	-0,0722111	7,08646305	0,28676796	0,60493309	0,81353839	EIF4EBP1	eukaryotic translation initiation factor 4E binding protein 1 [Source:HGNC Symbol;Acc:HGNC:3288]
ENSG00000178597	-0,3242315	-1,096232	0,28637378	0,60517764	0,81379236	PSAPL1	prosaposin like 1 [Source:HGNC Symbol;Acc:HGNC:33131]
ENSG00000106244	-0,0747831	6,83392093	0,28618051	0,60529763	0,81387879	PDAP1	PDGFA associated protein 1 [Source:HGNC Symbol;Acc:HGNC:14634]
ENSG00000135655	-0,0921607	4,76612832	0,28607013	0,60536617	0,81389605	USP15	ubiquitin specific peptidase 15 [Source:HGNC Symbol;Acc:HGNC:12613]
ENSG00000139974	-0,1123358	2,86770922	0,28592104	0,60545879	0,81394566	SLC38A6	solute carrier family 38 member 6 [Source:HGNC Symbol;Acc:HGNC:19863]

ENSG00000143933	0,09594853	8,89943802	0,28581114	0,60552709	0,81396257	CALM2	calmodulin 2 [Source:HGNC Symbol;Acc:HGNC:1445]
ENSG00000272583	0,24434517	-0,8049898	0,28570456	0,60559333	0,81397672	AL592494.2	novel transcript
ENSG00000148396	0,14676013	5,7135597	0,28540812	0,60577767	0,81410327	SEC16A	SEC16 homolog A, endoplasmic reticulum export factor [Source:HGNC Symbol;Acc:HGNC:29006]
ENSG00000186187	-0,0861237	5,32303768	0,28537394	0,60579893	0,81410327	ZNRF1	zinc and ring finger 1 [Source:HGNC Symbol;Acc:HGNC:18452]
ENSG00000137073	-0,1598181	5,23913579	0,28462759	0,60626363	0,81454621	UBAP2	ubiquitin associated protein 2 [Source:HGNC Symbol;Acc:HGNC:14185]
ENSG00000142528	0,08150428	3,99074566	0,28457596	0,60629581	0,81454621	ZNF473	zinc finger protein 473 [Source:HGNC Symbol;Acc:HGNC:23239]
ENSG00000203546	0,26250229	-0,908226	0,28466624	0,60623955	0,81454621	AL139353.1	novel protein
ENSG00000178449	-0,0905217	4,3081725	0,28398926	0,60666171	0,81496284	COX14	cytochrome c oxidase assembly factor COX14 [Source:HGNC Symbol;Acc:HGNC:28216]
ENSG00000125843	-0,0880018	4,18969646	0,28373967	0,60681751	0,81502226	AP5S1	adaptor related protein complex 5 subunit sigma 1 [Source:HGNC Symbol;Acc:HGNC:15875]
ENSG00000240857	0,07634811	4,88860767	0,28378944	0,60678644	0,81502226	RDH14	retinol dehydrogenase 14 [Source:HGNC Symbol;Acc:HGNC:19979]
ENSG00000269906	0,28797775	-1,6077305	0,2836092	0,606899	0,81505676	AL606834.1	novel transcript, sense intronic to SAV1
ENSG00000076770	-0,0757696	5,79439062	0,28325535	0,60712011	0,81507877	MBNL3	muscleblind like splicing regulator 3 [Source:HGNC Symbol;Acc:HGNC:20564]
ENSG00000135482	0,09605134	2,05195512	0,28275117	0,60743547	0,81507877	ZC3H10	zinc finger CCCH-type containing 10 [Source:HGNC Symbol;Acc:HGNC:25893]
ENSG00000154305	-0,127714	5,60547632	0,2825152	0,6075832	0,81507877	MIA3	MIA SH3 domain ER export factor 3 [Source:HGNC Symbol;Acc:HGNC:24008]
ENSG00000162613	0,10960027	7,09483737	0,28251319	0,60758446	0,81507877	FUBP1	far upstream element binding protein 1 [Source:HGNC Symbol;Acc:HGNC:4004]
ENSG00000162852	-0,0915174	3,00513666	0,28283545	0,60738273	0,81507877	CNST	consortin, connexin sorting protein [Source:HGNC Symbol;Acc:HGNC:26486]
ENSG00000174547	-0,1000546	6,81659183	0,28327073	0,6071105	0,81507877	MRPL11	mitochondrial ribosomal protein L11 [Source:HGNC Symbol;Acc:HGNC:14042]
ENSG00000185222	-0,0956353	4,53045168	0,28250096	0,60759211	0,81507877	TCEAL9	transcription elongation factor A like 9 [Source:HGNC Symbol;Acc:HGNC:30084]
ENSG00000188185	0,31950522	-1,9074824	0,28273392	0,60744627	0,81507877	LINC00265	long intergenic non-protein coding RNA 265 [Source:HGNC Symbol;Acc:HGNC:28019]
ENSG00000228305	-0,1574441	1,08306438	0,28272116	0,60745426	0,81507877	PRELID1P6	PRELID1 pseudogene 6 [Source:HGNC Symbol;Acc:HGNC:54771]
ENSG00000228532	-0,1612209	0,13884178	0,28242339	0,60764069	0,81507877	SUMO2P21	SUMO2 pseudogene 21 [Source:HGNC Symbol;Acc:HGNC:55054]
ENSG00000234912	0,09516055	2,66544589	0,28250123	0,60759195	0,81507877	SNHG20	small nucleolar RNA host gene 20 [Source:HGNC Symbol;Acc:HGNC:33099]
ENSG00000244538	-0,1870116	-0,4998375	0,28267091	0,60748571	0,81507877	AC098583.1	ribosomal protein S27 (RPS27) pseudogene
ENSG00000266473	-0,2668122	-1,66832	0,28270845	0,60746221	0,81507877	AC007448.4	novel transcript
ENSG00000162413	0,08467795	6,10710994	0,28218396	0,6077907	0,81515542	KLHL21	kelch like family member 21 [Source:HGNC Symbol;Acc:HGNC:29041]
ENSG00000170734	-0,0840089	3,85877982	0,28215407	0,60780943	0,81515542	POLH	DNA polymerase eta [Source:HGNC Symbol;Acc:HGNC:9181]
ENSG00000159228	-0,087885	5,09420478	0,28199763	0,60790749	0,81521209	CBR1	carbonyl reductase 1 [Source:HGNC Symbol;Acc:HGNC:1548]
ENSG00000151247	0,13001237	4,77838768	0,28190044	0,60796843	0,81521899	EIF4E	eukaryotic translation initiation factor 4E [Source:HGNC Symbol;Acc:HGNC:3287]
ENSG00000050748	0,08287088	5,1900036	0,28158484	0,60816642	0,81525119	MAPK9	mitogen-activated protein kinase 9 [Source:HGNC Symbol;Acc:HGNC:6886]
ENSG00000120549	0,13109072	3,70754442	0,28172061	0,60808123	0,81525119	KIAA1217	KIAA1217 [Source:HGNC Symbol;Acc:HGNC:25428]
ENSG00000167311	-0,2863386	-0,984629	0,28141746	0,60827147	0,81525119	ART5	ADP-ribosyltransferase 5 [Source:HGNC Symbol;Acc:HGNC:24049]
ENSG00000259877	0,26556654	-0,1344104	0,28143643	0,60825956	0,81525119	AC009113.1	novel transcript, antisense to ZNF778
ENSG00000260063	-0,4448312	-1,9637378	0,28162955	0,60813836	0,81525119	AL512408.1	novel transcript, antisense to ARID1A
ENSG00000101558	-0,0839937	7,35354185	0,28089684	0,60859851	0,81553987	VAPA	VAMP associated protein A [Source:HGNC Symbol;Acc:HGNC:12648]
ENSG00000240371	-0,2229008	-0,9913516	0,28093392	0,60857521	0,81553987	RPS4XP13	ribosomal protein S4X pseudogene 13 [Source:HGNC Symbol;Acc:HGNC:36522]
ENSG00000165271	-0,1122217	6,01341186	0,28067338	0,608739	0,81558302	NOL6	nucleolar protein 6 [Source:HGNC Symbol;Acc:HGNC:19910]
ENSG00000168502	0,12487114	2,71839258	0,28066802	0,60874237	0,81558302	MTCL1	microtubule crosslinking factor 1 [Source:HGNC Symbol;Acc:HGNC:29121]
ENSG00000170367	-0,307378	-1,5508567	0,28049604	0,60885055	0,81565316	CST5	cystatin D [Source:HGNC Symbol;Acc:HGNC:2477]
ENSG00000186350	0,07144228	6,012249	0,28022238	0,60902277	0,81573427	RXRA	retinoid X receptor alpha [Source:HGNC Symbol;Acc:HGNC:10477]
ENSG00000213977	-0,1743114	0,01320227	0,28023701	0,60901356	0,81573427	TAX1BP3	Tax1 binding protein 3 [Source:HGNC Symbol;Acc:HGNC:30684]
ENSG00000141294	0,36915305	-0,4954724	0,28005778	0,60912641	0,81579829	LRRC46	leucine rich repeat containing 46 [Source:HGNC Symbol;Acc:HGNC:25047]
ENSG00000102978	0,06895222	7,60247965	0,27986189	0,60924981	0,81588876	POLR2C	RNA polymerase II subunit C [Source:HGNC Symbol;Acc:HGNC:9189]
ENSG00000013306	-0,0977509	8,11525023	0,27959167	0,60942011	0,81595847	SLC25A39	solute carrier family 25 member 39 [Source:HGNC Symbol;Acc:HGNC:24279]
ENSG00000066827	-0,176715	2,08794133	0,27932426	0,60958876	0,81595847	ZFAT	zinc finger and AT-hook domain containing [Source:HGNC Symbol;Acc:HGNC:19899]

ENSG00000166435	-0,086926	2,84908374	0,2794078	0,60953606	0,81595847	XRRA1	X-ray radiation resistance associated 1 [Source:HGNC Symbol;Acc:HGNC:18868]
ENSG00000182108	-0,0944675	5,2623302	0,27924782	0,60963699	0,81595847	DEXI	Dexi homolog [Source:HGNC Symbol;Acc:HGNC:13267]
ENSG00000198732	0,07696662	6,64015225	0,27940327	0,60953892	0,81595847	SMOC1	SPARC related modular calcium binding 1 [Source:HGNC Symbol;Acc:HGNC:20318]
ENSG00000204310	-0,0867176	6,46944851	0,27967092	0,60937016	0,81595847	AGPAT1	1-acylglycerol-3-phosphate O-acyltransferase 1 [Source:HGNC Symbol;Acc:HGNC:324]
ENSG00000106049	-0,0805316	5,96551363	0,27892452	0,60984105	0,81608207	HIBADH	3-hydroxyisobutyrate dehydrogenase [Source:HGNC Symbol;Acc:HGNC:4907]
ENSG00000150593	0,0919853	4,16539705	0,27900409	0,60979081	0,81608207	PDCD4	programmed cell death 4 [Source:HGNC Symbol;Acc:HGNC:8763]
ENSG00000161912	-0,299099	-1,0181992	0,27869487	0,6099861	0,81612723	ADCY10P1	ADCY10 pseudogene 1 [Source:HGNC Symbol;Acc:HGNC:44143]
ENSG00000166415	0,26409604	-0,1169319	0,27851737	0,61009826	0,81612723	WDR72	WD repeat domain 72 [Source:HGNC Symbol;Acc:HGNC:26790]
ENSG00000221914	0,073475	5,24072855	0,27852875	0,61009107	0,81612723	PPP2R2A	protein phosphatase 2 regulatory subunit Balpha [Source:HGNC Symbol;Acc:HGNC:9304]
ENSG00000274712	0,19683981	1,28737922	0,27865738	0,61000979	0,81612723	AC005332.4	bromodomain PHD finger transcription factor (BPTF) pseudogene
ENSG00000219470	-0,2482492	-1,6196004	0,27842769	0,61015495	0,81612833	AL355802.1	40S ribosomal protein S2 (RPS2) pseudogene
ENSG00000150773	0,20152549	-0,8483882	0,2780704	0,61038091	0,81635583	PIH1D2	PIH1 domain containing 2 [Source:HGNC Symbol;Acc:HGNC:25210]
ENSG00000232499	0,16438965	0,52976864	0,27795141	0,6104562	0,81638179	AL391058.1	heterogeneous nuclear ribonucleoprotein A3 (hnRNPA3) pseudogene
ENSG00000087111	0,08705082	5,8756298	0,27778648	0,6105606	0,81644667	PIGS	phosphatidylinositol glycan anchor biosynthesis class S [Source:HGNC Symbol;Acc:HGNC:14937]
ENSG00000136699	-0,0678131	6,90452122	0,27696352	0,61108213	0,81691974	SMPD4	sphingomyelin phosphodiesterase 4 [Source:HGNC Symbol;Acc:HGNC:32949]
ENSG00000197785	-0,0885816	6,28303845	0,27700841	0,61105366	0,81691974	ATAD3A	ATPase family AAA domain containing 3A [Source:HGNC Symbol;Acc:HGNC:25567]
ENSG00000198742	0,1095999	4,43237172	0,27705995	0,61102097	0,81691974	SMURF1	SMAD specific E3 ubiquitin protein ligase 1 [Source:HGNC Symbol;Acc:HGNC:16807]
ENSG00000257199	-0,2316664	-1,0961064	0,27686149	0,61114686	0,81693151	AC025161.1	RAN binding protein 1 (RANBP1) pseudogene
ENSG00000274265	-0,2365444	-0,6947768	0,27665127	0,61128028	0,81703509	AC245297.3	novel transcript
ENSG00000122729	0,09668767	5,77661166	0,27628526	0,61151272	0,81704677	ACO1	aconitase 1 [Source:HGNC Symbol;Acc:HGNC:117]
ENSG00000138468	-0,1486335	1,37166988	0,27637058	0,61145852	0,81704677	SENP7	SUMO specific peptidase 7 [Source:HGNC Symbol;Acc:HGNC:30402]
ENSG00000139531	0,07452899	5,22673794	0,27639521	0,61144287	0,81704677	SUOX	sulfite oxidase [Source:HGNC Symbol;Acc:HGNC:11460]
ENSG00000179157	-0,3238092	-1,5479458	0,27638777	0,6114476	0,81704677	RPS2P28	ribosomal protein S2 pseudogene 28 [Source:HGNC Symbol;Acc:HGNC:35780]
ENSG00000100307	-0,247305	-1,1483459	0,27575488	0,61184991	0,81735441	CBX7	chromobox 7 [Source:HGNC Symbol;Acc:HGNC:1557]
ENSG00000105464	-0,2267306	0,26552523	0,27574474	0,61185635	0,81735441	GRIN2D	glutamate ionotropic receptor NMDA type subunit 2D [Source:HGNC Symbol;Acc:HGNC:4588]
ENSG00000166922	0,21089826	-0,8007946	0,27558919	0,61195533	0,81735441	SCG5	secretogranin V [Source:HGNC Symbol;Acc:HGNC:10816]
ENSG00000258712	0,30875269	-1,6004521	0,27557121	0,61196677	0,81735441	CXADRP2	CXADR pseudogene 2 [Source:HGNC Symbol;Acc:HGNC:33973]
ENSG00000198892	-0,2224209	0,56313492	0,27540536	0,61207234	0,81742068	SHISA4	shisa family member 4 [Source:HGNC Symbol;Acc:HGNC:27139]
ENSG00000008018	0,06826783	9,34240809	0,27471847	0,61251001	0,8177297	PSMB1	proteasome 20S subunit beta 1 [Source:HGNC Symbol;Acc:HGNC:9537]
ENSG00000090266	0,0737272	6,70925145	0,27444654	0,61268347	0,8177297	NDUFB2	NADH:ubiquinone oxidoreductase subunit B2 [Source:HGNC Symbol;Acc:HGNC:7697]
ENSG00000102007	0,26708667	-1,1615412	0,27472687	0,61250465	0,8177297	PLP2	proteolipid protein 2 [Source:HGNC Symbol;Acc:HGNC:9087]
ENSG00000109320	0,07873077	5,26928615	0,27441967	0,61270062	0,8177297	NFKB1	nuclear factor kappa B subunit 1 [Source:HGNC Symbol;Acc:HGNC:7794]
ENSG00000158813	-0,0976461	4,27101806	0,27454574	0,61262018	0,8177297	EDA	ectodysplasin A [Source:HGNC Symbol;Acc:HGNC:3157]
ENSG00000255495	0,23637994	-0,9624583	0,2743399	0,61275153	0,8177297	AC145124.1	novel transcript
ENSG00000272853	-0,1401941	0,42971237	0,27485079	0,61242564	0,8177297	AC069544.1	novel transcript
ENSG00000273344	-0,131686	2,7799683	0,2743963	0,61271553	0,8177297	PAXIP1-AS1	PAXIP1 antisense RNA 1 (head to head) [Source:HGNC Symbol;Acc:HGNC:27328]
ENSG00000089154	0,13954739	6,97936581	0,274214	0,61283189	0,81774107	GCN1	GCN1 activator of EIF2AK4 [Source:HGNC Symbol;Acc:HGNC:4199]
ENSG00000177556	-0,0960087	5,92977135	0,2741512	0,612872	0,81774107	ATOX1	antioxidant 1 copper chaperone [Source:HGNC Symbol;Acc:HGNC:798]
ENSG00000089737	0,11540604	6,88665771	0,27390137	0,61303157	0,8178046	DDX24	DEAD-box helicase 24 [Source:HGNC Symbol;Acc:HGNC:13266]
ENSG00000261526	0,112498	2,29477945	0,27398707	0,61297682	0,8178046	AC012615.1	novel transcript
ENSG00000058799	0,0894395	5,34573098	0,27344976	0,61332027	0,81796566	YIPF1	Yip1 domain family member 1 [Source:HGNC Symbol;Acc:HGNC:25231]
ENSG00000100253	0,16591249	0,34885746	0,27352452	0,61327246	0,81796566	MIOX	myo-inositol oxygenase [Source:HGNC Symbol;Acc:HGNC:14522]
ENSG00000232004	-0,2594259	-0,8740844	0,27348437	0,61329813	0,81796566	CAP1P2	CAP1 pseudogene 2 [Source:HGNC Symbol;Acc:HGNC:31970]
ENSG00000054392	-0,1358914	1,93801068	0,27334435	0,6133877	0,81798091	HHAT	hedgehog acyltransferase [Source:HGNC Symbol;Acc:HGNC:18270]

ENSG00000099385	0,07904	6,07049927	0,27308133	0,61355602	0,81810428	BCL7C	BAF chromatin remodeling complex subunit BCL7C [Source:HGNC Symbol;Acc:HGNC:1006]
ENSG00000129219	-0,0860698	3,62682648	0,2730248	0,61359221	0,81810428	PLD2	phospholipase D2 [Source:HGNC Symbol;Acc:HGNC:9068]
ENSG00000101412	0,09405241	5,45937614	0,27279351	0,61374034	0,81820647	E2F1	E2F transcription factor 1 [Source:HGNC Symbol;Acc:HGNC:3113]
ENSG00000119559	-0,0811989	4,97928991	0,27273024	0,61378087	0,81820647	C19orf25	chromosome 19 open reading frame 25 [Source:HGNC Symbol;Acc:HGNC:26711]
ENSG00000083454	0,15416823	0,60588415	0,27216887	0,61414078	0,81826841	P2RX5	purinergic receptor P2X 5 [Source:HGNC Symbol;Acc:HGNC:8536]
ENSG00000106246	-0,089526	2,9633689	0,27225024	0,61408858	0,81826841	PTCD1	pentatricopeptide repeat domain 1 [Source:HGNC Symbol;Acc:HGNC:22198]
ENSG00000171155	0,08548199	5,27456252	0,2721584	0,6141475	0,81826841	C1GALT1C1	C1GALT1 specific chaperone 1 [Source:HGNC Symbol;Acc:HGNC:24338]
ENSG00000196562	0,08967143	6,88595877	0,27237064	0,61401136	0,81826841	SULF2	sulfatase 2 [Source:HGNC Symbol;Acc:HGNC:20392]
ENSG00000214654	-0,1834052	0,4664645	0,27204632	0,61421941	0,81826841	B3GNT10	UDP-GlcNAc:betaGal beta-1,3-N-acetylglucosaminyltransferase 10 (putative) [Source:HGNC Symbol;Acc:HGNC:53
ENSG00000231409	-0,1540793	0,05265652	0,27207606	0,61420033	0,81826841	AC018868.1	ribosomal protein L36a (RPL36A) pseudogene
ENSG00000260105	-0,2079539	-0,2807063	0,27250934	0,61392244	0,81826841	AOC4P	amine oxidase copper containing 4, pseudogene [Source:HGNC Symbol;Acc:HGNC:48869]
ENSG00000066427	-0,0960611	3,35618885	0,27153121	0,61455018	0,81845601	ATXN3	ataxin 3 [Source:HGNC Symbol;Acc:HGNC:7106]
ENSG00000069966	-0,0792469	3,59039853	0,27173787	0,61441743	0,81845601	GNB5	G protein subunit beta 5 [Source:HGNC Symbol;Acc:HGNC:4401]
ENSG00000100372	0,07384023	5,56780757	0,27130374	0,61469638	0,81845601	SLC25A17	solute carrier family 25 member 17 [Source:HGNC Symbol;Acc:HGNC:10987]
ENSG00000141971	-0,0943057	5,02719634	0,27136785	0,61465517	0,81845601	MVB12A	multivesicular body subunit 12A [Source:HGNC Symbol;Acc:HGNC:25153]
ENSG00000158773	-0,1173783	4,32678765	0,27131608	0,61468844	0,81845601	USF1	upstream transcription factor 1 [Source:HGNC Symbol;Acc:HGNC:12593]
ENSG00000172009	-0,082595	6,90489808	0,27135293	0,61466475	0,81845601	THOP1	thimet oligopeptidase 1 [Source:HGNC Symbol;Acc:HGNC:11793]
ENSG00000165821	0,08923971	3,07348906	0,27109197	0,61483255	0,81848813	SALL2	spalt like transcription factor 2 [Source:HGNC Symbol;Acc:HGNC:10526]
ENSG00000188002	0,1311365	2,67896512	0,27111183	0,61481978	0,81848813	AC026412.1	programmed cell death 6 (PDCD6) pseudogene
ENSG00000066044	-0,069082	6,8728567	0,27089707	0,61495794	0,81850588	ELAVL1	ELAV like RNA binding protein 1 [Source:HGNC Symbol;Acc:HGNC:3312]
ENSG00000134152	-0,1000545	2,72814048	0,27095251	0,61492227	0,81850588	KATNBL1	katanin regulatory subunit B1 like 1 [Source:HGNC Symbol;Acc:HGNC:26199]
ENSG00000168802	0,22596262	-0,8034203	0,27003794	0,61551136	0,81916784	CHTF8	chromosome transmission fidelity factor 8 [Source:HGNC Symbol;Acc:HGNC:24353]
ENSG00000141568	0,07367437	7,08306728	0,26992885	0,6155817	0,81918684	FOXK2	forkhead box K2 [Source:HGNC Symbol;Acc:HGNC:6036]
ENSG00000122140	0,06762149	6,96635931	0,26982551	0,61564836	0,81920092	MRPS2	mitochondrial ribosomal protein S2 [Source:HGNC Symbol;Acc:HGNC:14495]
ENSG00000076984	0,10603245	4,28810027	0,26968443	0,61573939	0,81921078	MAP2K7	mitogen-activated protein kinase kinase 7 [Source:HGNC Symbol;Acc:HGNC:6847]
ENSG00000166471	-0,0831691	4,29156699	0,26964023	0,61576792	0,81921078	TMEM41B	transmembrane protein 41B [Source:HGNC Symbol;Acc:HGNC:28948]
ENSG00000242697	-0,2682596	-0,9331	0,26931398	0,61597858	0,81941641	RPL5P12	ribosomal protein L5 pseudogene 12 [Source:HGNC Symbol;Acc:HGNC:36221]
ENSG00000125534	-0,0965095	4,78174972	0,26910972	0,61611055	0,81944273	PPDPF	pancreatic progenitor cell differentiation and proliferation factor [Source:HGNC Symbol;Acc:HGNC:16142]
ENSG00000141560	0,0722809	5,749395	0,26912816	0,61609863	0,81944273	FN3KRP	fructosamine 3 kinase related protein [Source:HGNC Symbol;Acc:HGNC:25700]
ENSG00000059378	0,09202356	4,78269223	0,26900174	0,61618034	0,81946095	PARP12	poly(ADP-ribose) polymerase family member 12 [Source:HGNC Symbol;Acc:HGNC:21919]
ENSG00000100813	-0,1103886	7,53050248	0,26830979	0,616628	0,81974382	ACIN1	apoptotic chromatin condensation inducer 1 [Source:HGNC Symbol;Acc:HGNC:17066]
ENSG00000105401	-0,0690013	7,97738117	0,26797952	0,61684193	0,81974382	CDC37	cell division cycle 37, HSP90 cochaperone [Source:HGNC Symbol;Acc:HGNC:1735]
ENSG00000138382	-0,0978788	5,7446255	0,26842157	0,61655563	0,81974382	METTL5	methyltransferase like 5 [Source:HGNC Symbol;Acc:HGNC:25006]
ENSG00000138385	-0,0935098	8,04498972	0,26810477	0,61676078	0,81974382	SSB	small RNA binding exonuclease protection factor La [Source:HGNC Symbol;Acc:HGNC:11316]
ENSG00000141905	0,14883428	3,50207489	0,26798706	0,61683705	0,81974382	NFIC	nuclear factor I C [Source:HGNC Symbol;Acc:HGNC:7786]
ENSG00000156697	-0,1199989	5,55570823	0,26798704	0,61683706	0,81974382	UTP14A	UTP14A small subunit processome component [Source:HGNC Symbol;Acc:HGNC:10665]
ENSG00000229965	-0,1899015	-0,1678329	0,26800448	0,61682576	0,81974382	RPS3AP7	RPS3A pseudogene 7 [Source:HGNC Symbol;Acc:HGNC:36376]
ENSG00000270629	0,31221786	-0,9437424	0,26842607	0,61655272	0,81974382	NBPF14	NBPF member 14 [Source:HGNC Symbol;Acc:HGNC:25232]
ENSG00000172663	-0,104786	2,75164952	0,26745582	0,61718151	0,82007901	TMEM134	transmembrane protein 134 [Source:HGNC Symbol;Acc:HGNC:26142]
ENSG00000197363	0,15984245	0,15367573	0,26733091	0,61726257	0,82007901	ZNF517	zinc finger protein 517 [Source:HGNC Symbol;Acc:HGNC:27984]
ENSG00000240602	-0,3524563	-1,5902938	0,26735633	0,61724607	0,82007901	AADACP1	arylacetamide deacetylase pseudogene 1 [Source:HGNC Symbol;Acc:HGNC:50305]
ENSG00000166123	-0,0883115	8,71756923	0,26721625	0,61733699	0,82010331	GPT2	glutamic--pyruvic transaminase 2 [Source:HGNC Symbol;Acc:HGNC:18062]
ENSG00000214203	-0,2124005	-1,0094016	0,26709599	0,61741508	0,82013247	RPS4XP1	ribosomal protein S4X pseudogene 1 [Source:HGNC Symbol;Acc:HGNC:32444]
ENSG00000162482	-0,2296384	-1,4424646	0,26693614	0,6175189	0,8201958	AKR7A3	aldo-keto reductase family 7 member A3 [Source:HGNC Symbol;Acc:HGNC:390]

ENSG00000121486	0,08834781	3,69899346	0,26677226	0,61762538	0,82026266	TRMT1L	tRNA methyltransferase 1 like [Source:HGNC Symbol;Acc:HGNC:16782]
ENSG00000068001	0,06827091	7,32077221	0,26640469	0,61786437	0,82035632	HYAL2	hyaluronidase 2 [Source:HGNC Symbol;Acc:HGNC:5321]
ENSG00000122484	0,07998959	4,05186276	0,26643261	0,61784621	0,82035632	RPAP2	RNA polymerase II associated protein 2 [Source:HGNC Symbol;Acc:HGNC:25791]
ENSG00000231702	0,23423871	-0,4068757	0,26653891	0,61777708	0,82035632	AL645608.3	ribosomal protein L39 (RPL39) pseudogene
ENSG00000019102	-0,1795112	1,06551349	0,26621112	0,61799031	0,82037442	VSIG2	V-set and immunoglobulin domain containing 2 [Source:HGNC Symbol;Acc:HGNC:17149]
ENSG00000079277	0,0880479	4,3551738	0,26621372	0,61798862	0,82037442	MKNK1	MAPK interacting serine/threonine kinase 1 [Source:HGNC Symbol;Acc:HGNC:7110]
ENSG00000226453	-0,168801	1,08325098	0,26608154	0,61807465	0,82041183	LINC02542	long intergenic non-protein coding RNA 2542 [Source:HGNC Symbol;Acc:HGNC:53576]
ENSG00000180071	0,13382476	1,17753964	0,26561667	0,61837744	0,82073917	ANKRD18A	ankyrin repeat domain 18A [Source:HGNC Symbol;Acc:HGNC:23643]
ENSG00000185697	0,1850516	0,2806723	0,26534071	0,61855735	0,82083443	MYBL1	MYB proto-oncogene like 1 [Source:HGNC Symbol;Acc:HGNC:7547]
ENSG00000198218	0,08215521	5,61730099	0,26519719	0,61865096	0,82083443	QRICH1	glutamine rich 1 [Source:HGNC Symbol;Acc:HGNC:24713]
ENSG00000230787	-0,2066535	-0,839873	0,26521736	0,61863781	0,82083443	PSAT1P3	phosphoserine aminotransferase 1 pseudogene 3 [Source:HGNC Symbol;Acc:HGNC:43989]
ENSG00000238005	-0,2264382	-1,082179	0,26516194	0,61867396	0,82083443	AL391832.2	novel transcript
ENSG00000146729	-0,0753907	5,31653431	0,26505423	0,61874424	0,82085313	NIPSNAP2	nipsnap homolog 2 [Source:HGNC Symbol;Acc:HGNC:4179]
ENSG00000013441	0,09156802	5,21083501	0,26472674	0,61895804	0,82099197	CLK1	CDC like kinase 1 [Source:HGNC Symbol;Acc:HGNC:2068]
ENSG00000260329	-0,3131711	-1,7286212	0,26472176	0,6189613	0,82099197	AC007541.1	novel transcript, antisense to C12orf23
ENSG00000019995	-0,0785098	4,33720141	0,26451792	0,61909447	0,82103892	ZRANB1	zinc finger RANBP2-type containing 1 [Source:HGNC Symbol;Acc:HGNC:18224]
ENSG00000049541	0,09852505	5,90463204	0,26432356	0,6192215	0,82103892	RFC2	replication factor C subunit 2 [Source:HGNC Symbol;Acc:HGNC:9970]
ENSG00000186088	-0,0901469	2,33679316	0,26439817	0,61917272	0,82103892	GSAP	gamma-secretase activating protein [Source:HGNC Symbol;Acc:HGNC:28042]
ENSG00000271780	0,17971078	0,33355351	0,2643369	0,61921278	0,82103892	AL118558.3	novel transcript
ENSG00000128891	0,09980119	4,05398206	0,2639712	0,61945196	0,82126996	CCDC32	coiled-coil domain containing 32 [Source:HGNC Symbol;Acc:HGNC:28295]
ENSG00000267288	0,3246462	-1,7897608	0,26382118	0,61955013	0,82132558	AC138150.2	novel transcript, antisense to HEXIM2
ENSG00000139579	0,08058903	5,43946441	0,26322392	0,61994136	0,82154602	NABP2	nucleic acid binding protein 2 [Source:HGNC Symbol;Acc:HGNC:28412]
ENSG00000216657	0,2741597	-1,5656065	0,26324016	0,61993072	0,82154602	GLRX3P2	glutaredoxin 3 pseudogene 2 [Source:HGNC Symbol;Acc:HGNC:49788]
ENSG00000228544	-0,2256364	-0,7860006	0,26326301	0,61991574	0,82154602	CCDC183-AS1	CCDC183 antisense RNA 1 [Source:HGNC Symbol;Acc:HGNC:44105]
ENSG00000240480	-0,2742313	-1,951616	0,26344637	0,61979558	0,82154602	RPL29P2	ribosomal protein L29 pseudogene 2 [Source:HGNC Symbol;Acc:HGNC:17334]
ENSG00000069493	-0,2302504	-1,3856256	0,26277383	0,62023656	0,8216463	CLEC2D	C-type lectin domain family 2 member D [Source:HGNC Symbol;Acc:HGNC:14351]
ENSG00000105321	-0,1330091	3,30207131	0,26255706	0,62037885	0,8216463	CCDC9	coiled-coil domain containing 9 [Source:HGNC Symbol;Acc:HGNC:24560]
ENSG00000108312	-0,1116275	6,48896397	0,26280263	0,62021766	0,8216463	UBTF	upstream binding transcription factor [Source:HGNC Symbol;Acc:HGNC:12511]
ENSG00000125952	0,0776111	3,86738711	0,26263315	0,62032889	0,8216463	MAX	MYC associated factor X [Source:HGNC Symbol;Acc:HGNC:6913]
ENSG00000136682	0,0853698	4,29525324	0,26250851	0,62041073	0,8216463	CBWD2	COBW domain containing 2 [Source:HGNC Symbol;Acc:HGNC:17907]
ENSG00000198832	-0,1212072	2,93833773	0,26298714	0,62009662	0,8216463	SELENOM	selenoprotein M [Source:HGNC Symbol;Acc:HGNC:30397]
ENSG00000270055	0,24083768	-0,3873477	0,26268876	0,62029239	0,8216463	AC127502.2	novel transcript
ENSG00000101888	0,13442439	1,37886387	0,26213833	0,62065392	0,82189386	NXT2	nuclear transport factor 2 like export factor 2 [Source:HGNC Symbol;Acc:HGNC:18151]
ENSG00000182253	0,17281927	1,97294486	0,26187097	0,6208297	0,82197762	SYNM	synemin [Source:HGNC Symbol;Acc:HGNC:24466]
ENSG00000258366	0,2482768	-1,1018747	0,26191892	0,62079817	0,82197762	RTEL1	regulator of telomere elongation helicase 1 [Source:HGNC Symbol;Acc:HGNC:15888]
ENSG00000140374	-0,0698694	7,82732394	0,26176204	0,62090135	0,82199799	ETFA	electron transfer flavoprotein subunit alpha [Source:HGNC Symbol;Acc:HGNC:3481]
ENSG00000127463	0,14653757	7,41876536	0,2616357	0,62098448	0,82203355	EMC1	ER membrane protein complex subunit 1 [Source:HGNC Symbol;Acc:HGNC:28957]
ENSG00000227694	-0,1455591	0,87333833	0,26135849	0,62116696	0,82220061	RPL23AP74	ribosomal protein L23a pseudogene 74 [Source:HGNC Symbol;Acc:HGNC:36148]
ENSG00000150907	0,10120904	3,51814042	0,26117149	0,62129013	0,8222301	FOXO1	forkhead box O1 [Source:HGNC Symbol;Acc:HGNC:3819]
ENSG00000185305	-0,1562267	0,54900726	0,26115376	0,62130181	0,8222301	ARL15	ADP ribosylation factor like GTPase 15 [Source:HGNC Symbol;Acc:HGNC:25945]
ENSG00000198380	0,11595905	7,07742222	0,2610272	0,62138521	0,82226599	GFPT1	glutamine--fructose-6-phosphate transaminase 1 [Source:HGNC Symbol;Acc:HGNC:4241]
ENSG00000103264	0,07267682	6,87768245	0,26061023	0,62166015	0,8225553	FBXO31	F-box protein 31 [Source:HGNC Symbol;Acc:HGNC:16510]
ENSG00000119537	0,08427193	4,97989994	0,26041065	0,62179185	0,82256522	KDSR	3-ketodihydrosphingosine reductase [Source:HGNC Symbol;Acc:HGNC:4021]
ENSG00000138074	0,07239398	8,14755966	0,26034291	0,62183656	0,82256522	SLC5A6	solute carrier family 5 member 6 [Source:HGNC Symbol;Acc:HGNC:11041]

ENSG00000178913	0,06816221	6,85730938	0,26043402	0,62177642	0,82256522	TAF7	TATA-box binding protein associated factor 7 [Source:HGNC Symbol;Acc:HGNC:11541]
ENSG00000166582	-0,0756905	7,36273318	0,26005598	0,62202605	0,82274138	CENPV	centromere protein V [Source:HGNC Symbol;Acc:HGNC:29920]
ENSG00000105887	-0,0749369	5,01882661	0,25988785	0,62213714	0,82281382	MTPN	myotrophin [Source:HGNC Symbol;Acc:HGNC:15667]
ENSG00000134470	0,24992747	-1,3884806	0,25944441	0,62243036	0,82292144	IL15RA	interleukin 15 receptor subunit alpha [Source:HGNC Symbol;Acc:HGNC:5978]
ENSG00000164751	-0,118728	3,79461728	0,25942406	0,62244383	0,82292144	PEX2	peroxisomal biogenesis factor 2 [Source:HGNC Symbol;Acc:HGNC:9717]
ENSG00000176108	0,06952269	5,75786303	0,25956828	0,62234842	0,82292144	CHMP6	charged multivesicular body protein 6 [Source:HGNC Symbol;Acc:HGNC:25675]
ENSG00000239445	0,28962708	-1,5717762	0,25963595	0,62230367	0,82292144	ST3GAL6-AS1	ST3GAL6 antisense RNA 1 [Source:HGNC Symbol;Acc:HGNC:40828]
ENSG00000128159	-0,1100152	3,1733607	0,25933116	0,6225053	0,82292824	TUBGCP6	tubulin gamma complex associated protein 6 [Source:HGNC Symbol;Acc:HGNC:18127]
ENSG00000162572	-0,1883781	0,23675382	0,25906965	0,62267843	0,82300816	SCNN1D	sodium channel epithelial 1 subunit delta [Source:HGNC Symbol;Acc:HGNC:10601]
ENSG00000196352	-0,1168623	5,75817358	0,25915187	0,62262398	0,82300816	CD55	CD55 molecule (Cromer blood group) [Source:HGNC Symbol;Acc:HGNC:2665]
ENSG00000033178	-0,0812689	3,95818141	0,25878623	0,62286618	0,82303292	UBA6	ubiquitin like modifier activating enzyme 6 [Source:HGNC Symbol;Acc:HGNC:25581]
ENSG00000105672	-0,2510705	-1,1189179	0,25895122	0,62275686	0,82303292	ETV2	ETS variant transcription factor 2 [Source:HGNC Symbol;Acc:HGNC:3491]
ENSG00000156463	-0,0941437	2,17254041	0,25882342	0,62284154	0,82303292	SH3RF2	SH3 domain containing ring finger 2 [Source:HGNC Symbol;Acc:HGNC:26299]
ENSG00000127957	-0,1007914	1,96151745	0,2586601	0,62294978	0,82306895	PMS2P3	PMS1 homolog 2, mismatch repair system component pseudogene 3 [Source:HGNC Symbol;Acc:HGNC:9128]
ENSG00000127125	0,09319363	4,56442767	0,25851607	0,62304527	0,82311543	PPCS	phosphopantothenoylcysteine synthetase [Source:HGNC Symbol;Acc:HGNC:25686]
ENSG00000213326	-0,0911262	3,5397014	0,2584371	0,62309765	0,82311543	RPS7P11	ribosomal protein S7 pseudogene 11 [Source:HGNC Symbol;Acc:HGNC:35841]
ENSG00000065057	-0,0677971	5,76949797	0,25787187	0,6234728	0,82344582	NTHL1	nth like DNA glycosylase 1 [Source:HGNC Symbol;Acc:HGNC:8028]
ENSG00000147400	-0,0908073	6,47063765	0,2579536	0,62341852	0,82344582	CETN2	centrin 2 [Source:HGNC Symbol;Acc:HGNC:1867]
ENSG00000177045	0,0828419	3,72065143	0,25772072	0,62357321	0,82344582	SIX5	SIX homeobox 5 [Source:HGNC Symbol;Acc:HGNC:10891]
ENSG00000178921	-0,1869467	5,209257	0,2577285	0,62356805	0,82344582	PFAS	phosphoribosylformylglycinamidine synthase [Source:HGNC Symbol;Acc:HGNC:8863]
ENSG00000142694	-0,1286306	1,35096705	0,25734585	0,62382241	0,82357265	EVA1B	eva-1 homolog B [Source:HGNC Symbol;Acc:HGNC:25558]
ENSG00000160767	0,08031778	4,92861953	0,25739656	0,62378869	0,82357265	FAM189B	family with sequence similarity 189 member B [Source:HGNC Symbol;Acc:HGNC:1233]
ENSG00000163581	-0,0762516	3,54705524	0,2572041	0,6239167	0,82357265	SLC2A2	solute carrier family 2 member 2 [Source:HGNC Symbol;Acc:HGNC:11006]
ENSG00000224908	-0,2515334	-1,7402767	0,25715234	0,62395113	0,82357265	TIMM8BP2	translocase of inner mitochondrial membrane 8B pseudogene 2 [Source:HGNC Symbol;Acc:HGNC:39212]
ENSG00000240471	0,25210209	-1,4257172	0,25722343	0,62390384	0,82357265	PHBP8	prohibitin pseudogene 8 [Source:HGNC Symbol;Acc:HGNC:39287]
ENSG00000181817	-0,0818988	4,48811345	0,25680781	0,62418047	0,82380093	LSM10	LSM10, U7 small nuclear RNA associated [Source:HGNC Symbol;Acc:HGNC:17562]
ENSG00000154370	-0,072454	4,76825995	0,25666039	0,62427867	0,8238561	TRIM11	tripartite motif containing 11 [Source:HGNC Symbol;Acc:HGNC:16281]
ENSG00000031003	0,1308702	2,11866167	0,25648324	0,62439671	0,82393745	FAM13B	family with sequence similarity 13 member B [Source:HGNC Symbol;Acc:HGNC:1335]
ENSG00000151893	0,08543218	3,61021797	0,2563215	0,62450453	0,8240053	CACUL1	CDK2 associated cullin domain 1 [Source:HGNC Symbol;Acc:HGNC:23727]
ENSG00000061936	0,09239267	5,01627053	0,25615416	0,62461613	0,8240248	SFSWAP	splicing factor SWAP [Source:HGNC Symbol;Acc:HGNC:10790]
ENSG00000082458	-0,0807396	3,51975527	0,25588094	0,62479844	0,8240248	DLG3	discs large MAGUK scaffold protein 3 [Source:HGNC Symbol;Acc:HGNC:2902]
ENSG00000154845	0,06644924	5,93890958	0,25587659	0,62480134	0,8240248	PPP4R1	protein phosphatase 4 regulatory subunit 1 [Source:HGNC Symbol;Acc:HGNC:9320]
ENSG00000171316	0,15582589	4,84000389	0,25589946	0,62478608	0,8240248	CHD7	chromodomain helicase DNA binding protein 7 [Source:HGNC Symbol;Acc:HGNC:20626]
ENSG00000243943	0,08564612	3,74203392	0,25606291	0,624677	0,8240248	ZNF512	zinc finger protein 512 [Source:HGNC Symbol;Acc:HGNC:29380]
ENSG00000267586	0,34530742	-1,4705628	0,25577117	0,62487171	0,82404323	LINC00907	long intergenic non-protein coding RNA 907 [Source:HGNC Symbol;Acc:HGNC:44327]
ENSG00000184634	0,15521141	4,15305475	0,25568629	0,62492839	0,82404358	MED12	mediator complex subunit 12 [Source:HGNC Symbol;Acc:HGNC:11957]
ENSG00000164535	0,09304173	4,85433193	0,25516915	0,62527397	0,82442486	DAGLB	diacylglycerol lipase beta [Source:HGNC Symbol;Acc:HGNC:28923]
ENSG00000172432	-0,072713	4,82875602	0,25467443	0,62560497	0,82471242	GTPBP2	GTP binding protein 2 [Source:HGNC Symbol;Acc:HGNC:4670]
ENSG00000253341	-0,1149811	1,0636942	0,25475299	0,62555238	0,82471242	PCBP2P2	poly(rC) binding protein 2 pseudogene 2 [Source:HGNC Symbol;Acc:HGNC:8650]
ENSG00000078140	0,08856773	5,91814206	0,25429231	0,62586093	0,8249754	UBE2K	ubiquitin conjugating enzyme E2 K [Source:HGNC Symbol;Acc:HGNC:4914]
ENSG00000103490	0,12710787	1,48276222	0,25410242	0,62598821	0,82502391	PYCARD	PYD and CARD domain containing [Source:HGNC Symbol;Acc:HGNC:16608]
ENSG00000146676	0,07914342	3,06915181	0,2539847	0,62606715	0,82502391	PURB	purine rich element binding protein B [Source:HGNC Symbol;Acc:HGNC:9702]
ENSG00000261840	0,16777706	0,62850757	0,25401102	0,6260495	0,82502391	AC093249.6	uncharacterized LOC730183 [Source:NCBI gene (formerly Entrezgene);Acc:730183]
ENSG00000130956	0,07450192	5,67454199	0,25386165	0,62614969	0,82505825	HABP4	hyaluronan binding protein 4 [Source:HGNC Symbol;Acc:HGNC:17062]

ENSG00000014138	-0,0886742	2,14839238	0,25367843	0,62627263	0,82514583	POLA2	DNA polymerase alpha 2, accessory subunit [Source:HGNC Symbol;Acc:HGNC:30073]
ENSG00000124226	0,07123898	7,34197693	0,25341001	0,62645285	0,82523442	RNF114	ring finger protein 114 [Source:HGNC Symbol;Acc:HGNC:13094]
ENSG00000162813	-0,0807819	4,91523939	0,25345409	0,62642324	0,82523442	BPNT1	3'(2'), 5'-bisphosphate nucleotidase 1 [Source:HGNC Symbol;Acc:HGNC:1096]
ENSG00000220804	0,14486666	0,70454927	0,25327811	0,62654145	0,82526021	LINC01881	long intergenic non-protein coding RNA 1881 [Source:HGNC Symbol;Acc:HGNC:52700]
ENSG00000261238	-0,2407974	-1,1524834	0,25321269	0,62658541	0,82526021	AC009166.1	novel transcript, antisense to SALL1
ENSG00000251192	0,17107431	0,57726803	0,25312805	0,62664229	0,82526072	ZNF674	zinc finger protein 674 [Source:HGNC Symbol;Acc:HGNC:17625]
ENSG00000151093	-0,0845839	4,16469081	0,25293639	0,62677114	0,82528162	OXSM	3-oxoacyl-ACP synthase, mitochondrial [Source:HGNC Symbol;Acc:HGNC:26063]
ENSG00000228224	0,1892233	-0,0731781	0,2529751	0,6267451	0,82528162	NACA4P	NACA family member 4, pseudogene [Source:HGNC Symbol;Acc:HGNC:24688]
ENSG00000061938	0,14214306	2,61270264	0,25232405	0,62718321	0,82539494	TNK2	tyrosine kinase non receptor 2 [Source:HGNC Symbol;Acc:HGNC:19297]
ENSG00000086062	0,11531867	5,6850134	0,25204718	0,62736974	0,82539494	B4GALT1	beta-1,4-galactosyltransferase 1 [Source:HGNC Symbol;Acc:HGNC:924]
ENSG00000140092	-0,2855891	-0,5924314	0,25223439	0,6272436	0,82539494	FBLN5	fibulin 5 [Source:HGNC Symbol;Acc:HGNC:3602]
ENSG00000143748	0,09283406	4,51342816	0,25211839	0,62732174	0,82539494	NVL	nuclear VCP like [Source:HGNC Symbol;Acc:HGNC:8070]
ENSG00000145882	-0,1662066	0,92124379	0,25261215	0,62698925	0,82539494	PCYOX1L	prenylcysteine oxidase 1 like [Source:HGNC Symbol;Acc:HGNC:28477]
ENSG00000148296	-0,0929185	5,61457273	0,25223025	0,62724639	0,82539494	SURF6	surfeit 6 [Source:HGNC Symbol;Acc:HGNC:11478]
ENSG00000160007	0,10374177	5,66925584	0,25218564	0,62727644	0,82539494	ARHGAP35	Rho GTPase activating protein 35 [Source:HGNC Symbol;Acc:HGNC:4591]
ENSG00000185236	0,07368799	5,35043902	0,25214707	0,62730242	0,82539494	RAB11B	RAB11B, member RAS oncogene family [Source:HGNC Symbol;Acc:HGNC:9761]
ENSG00000220744	-0,2045292	-0,7319545	0,25196935	0,62742219	0,82539494	RPL5P18	ribosomal protein L5 pseudogene 18 [Source:HGNC Symbol;Acc:HGNC:36640]
ENSG00000272829	0,29972913	-1,2616141	0,25225729	0,62722817	0,82539494	AC002470.1	novel transcript
ENSG00000184602	0,10755191	2,9796379	0,25174949	0,62757043	0,82551562	SNN	stannin [Source:HGNC Symbol;Acc:HGNC:11149]
ENSG00000147454	-0,0964811	2,37829589	0,25106644	0,62803151	0,82604774	SLC25A37	solute carrier family 25 member 37 [Source:HGNC Symbol;Acc:HGNC:29786]
ENSG00000006625	0,10307492	4,85787479	0,25069415	0,62828315	0,82630433	GGCT	gamma-glutamylcyclotransferase [Source:HGNC Symbol;Acc:HGNC:21705]
ENSG00000081059	-0,0829038	4,15559157	0,25059517	0,62835009	0,82631052	TCF7	transcription factor 7 [Source:HGNC Symbol;Acc:HGNC:11639]
ENSG00000203879	-0,092877	4,91371457	0,25051995	0,62840098	0,82631052	GDI1	GDP dissociation inhibitor 1 [Source:HGNC Symbol;Acc:HGNC:4226]
ENSG00000002834	-0,1293256	7,87899884	0,25025375	0,62858113	0,8264229	LASP1	LIM and SH3 protein 1 [Source:HGNC Symbol;Acc:HGNC:6513]
ENSG00000224004	0,24284844	-0,7987099	0,25015229	0,62864983	0,8264229	ATP5F1CP1	ATP synthase F1 subunit gamma pseudogene 1 [Source:HGNC Symbol;Acc:HGNC:834]
ENSG00000236603	-0,1112908	1,56008357	0,25014296	0,62865615	0,8264229	RANP1	RAN pseudogene 1 [Source:HGNC Symbol;Acc:HGNC:21631]
ENSG00000092439	-0,0830135	3,89500056	0,24994449	0,62879059	0,82645801	TRPM7	transient receptor potential cation channel subfamily M member 7 [Source:HGNC Symbol;Acc:HGNC:17994]
ENSG00000169371	0,08842502	3,24390563	0,24993649	0,628796	0,82645801	SNUPN	snurportin 1 [Source:HGNC Symbol;Acc:HGNC:14245]
ENSG00000189227	-0,0852003	3,68507425	0,24982169	0,6288738	0,8264859	C15orf61	chromosome 15 open reading frame 61 [Source:HGNC Symbol;Acc:HGNC:34453]
ENSG00000188343	-0,0783836	2,83832481	0,24963571	0,62899988	0,82657724	CIBAR1	CBY1 interacting BAR domain containing 1 [Source:HGNC Symbol;Acc:HGNC:30452]
ENSG00000185088	-0,1024717	6,89987	0,24937267	0,62917829	0,82673733	RPS27L	ribosomal protein S27 like [Source:HGNC Symbol;Acc:HGNC:18476]
ENSG00000125741	-0,1032381	4,09659274	0,24895259	0,62946347	0,82699473	OPA3	outer mitochondrial membrane lipid metabolism regulator OPA3 [Source:HGNC Symbol;Acc:HGNC:8142]
ENSG00000196345	-0,1405355	0,48266173	0,24891737	0,6294874	0,82699473	ZKSCAN7	zinc finger with KRAB and SCAN domains 7 [Source:HGNC Symbol;Acc:HGNC:12955]
ENSG00000266920	-0,1533907	0,62964881	0,24879585	0,62956996	0,82702883	ACTBP9	ACTB pseudogene 9 [Source:HGNC Symbol;Acc:HGNC:142]
ENSG00000163527	0,08298807	7,20676963	0,24832023	0,62989335	0,82730522	STT3B	STT3 oligosaccharyltransferase complex catalytic subunit B [Source:HGNC Symbol;Acc:HGNC:30611]
ENSG00000234753	-0,1954839	1,00295561	0,24831982	0,62989363	0,82730522	FOXP4-AS1	FOXP4 antisense RNA 1 [Source:HGNC Symbol;Acc:HGNC:50332]
ENSG00000127666	-0,1056525	2,32754089	0,24802141	0,63009673	0,82733099	TICAM1	toll like receptor adaptor molecule 1 [Source:HGNC Symbol;Acc:HGNC:18348]
ENSG00000142945	0,08307797	6,25500587	0,24807375	0,63006109	0,82733099	KIF2C	kinesin family member 2C [Source:HGNC Symbol;Acc:HGNC:6393]
ENSG00000151012	0,13558327	4,57899726	0,24795819	0,63013977	0,82733099	SLC7A11	solute carrier family 7 member 11 [Source:HGNC Symbol;Acc:HGNC:11059]
ENSG00000178741	-0,0665508	8,25339396	0,24801052	0,63010414	0,82733099	COX5A	cytochrome c oxidase subunit 5A [Source:HGNC Symbol;Acc:HGNC:2267]
ENSG00000197620	-0,1043114	2,53060888	0,24776854	0,63026895	0,82742623	EOLA1	endothelium and lymphocyte associated ASCH domain 1 [Source:HGNC Symbol;Acc:HGNC:28089]
ENSG00000090487	0,0740692	6,34536683	0,24745542	0,63048237	0,82742691	SPG21	SPG21 abhydrolase domain containing, maspardin [Source:HGNC Symbol;Acc:HGNC:20373]
ENSG00000101695	0,38153492	-1,8561138	0,24710329	0,63072257	0,82742691	RNF125	ring finger protein 125 [Source:HGNC Symbol;Acc:HGNC:21150]
ENSG00000117362	0,1027406	7,76595068	0,24722312	0,63064081	0,82742691	APH1A	aph-1 homolog A, gamma-secretase subunit [Source:HGNC Symbol;Acc:HGNC:29509]

ENSG00000125818	0,06654436	8,49056425	0,2471682	0,63067828	0,82742691	PSMF1	proteasome inhibitor subunit 1 [Source:HGNC Symbol;Acc:HGNC:9571]
ENSG00000134874	-0,0988578	3,9371253	0,24723898	0,63062999	0,82742691	DZIP1	DAZ interacting zinc finger protein 1 [Source:HGNC Symbol;Acc:HGNC:20908]
ENSG00000138592	0,11575793	4,21566573	0,24745188	0,63048478	0,82742691	USP8	ubiquitin specific peptidase 8 [Source:HGNC Symbol;Acc:HGNC:12631]
ENSG00000219951	-0,2588312	-1,2891199	0,24725283	0,63062054	0,82742691	AL391416.1	glyceraldehyde-3-phosphate dehydrogenase (GAPDH) pseudogene
ENSG00000257074	0,25954902	-1,9591721	0,24718706	0,63066541	0,82742691	RPL29P33	ribosomal protein L29 pseudogene 33 [Source:HGNC Symbol;Acc:HGNC:36923]
ENSG00000124097	-0,1705718	0,10480494	0,24689097	0,63086751	0,82754274	HMGB1P1	high mobility group box 1 pseudogene 1 [Source:HGNC Symbol;Acc:HGNC:4993]
ENSG00000004864	0,08205461	6,67914032	0,24677102	0,63094943	0,82757589	SLC25A13	solute carrier family 25 member 13 [Source:HGNC Symbol;Acc:HGNC:10983]
ENSG00000105656	-0,0948151	3,93662965	0,24629126	0,63127733	0,82770871	ELL	elongation factor for RNA polymerase II [Source:HGNC Symbol;Acc:HGNC:23114]
ENSG00000160214	-0,090354	6,45165103	0,24634317	0,63124183	0,82770871	RRP1	ribosomal RNA processing 1 [Source:HGNC Symbol;Acc:HGNC:18785]
ENSG00000263823	-0,1719289	0,14475977	0,24645647	0,63116437	0,82770871	AC009831.1	novel transcript, antisense to KIAA1012
ENSG00000272906	-0,2474695	-1,4304328	0,24629155	0,63127713	0,82770871	AL353708.3	novel transcript, antisense to TOR1AIP1
ENSG00000130958	0,07405085	5,29029233	0,24609959	0,63140844	0,82779282	SLC35D2	solute carrier family 35 member D2 [Source:HGNC Symbol;Acc:HGNC:20799]
ENSG00000144895	0,06589835	7,13445728	0,24594905	0,63151146	0,82779282	EIF2A	eukaryotic translation initiation factor 2A [Source:HGNC Symbol;Acc:HGNC:3254]
ENSG00000187608	-0,0845445	5,16165192	0,24595154	0,63150976	0,82779282	ISG15	ISG15 ubiquitin like modifier [Source:HGNC Symbol;Acc:HGNC:4053]
ENSG00000132773	0,07174863	5,11934602	0,24568536	0,63169202	0,82780666	TOE1	target of EGR1, exonuclease [Source:HGNC Symbol;Acc:HGNC:15954]
ENSG00000168610	0,10371582	6,18481828	0,24571776	0,63166982	0,82780666	STAT3	signal transducer and activator of transcription 3 [Source:HGNC Symbol;Acc:HGNC:11364]
ENSG00000259305	0,2856212	-1,2116388	0,24573691	0,63165671	0,82780666	ZHX1-C8orf76	ZHX1-C8orf76 readthrough [Source:HGNC Symbol;Acc:HGNC:42975]
ENSG00000111371	-0,0930486	5,63688173	0,24539969	0,63188776	0,8278404	SLC38A1	solute carrier family 38 member 1 [Source:HGNC Symbol;Acc:HGNC:13447]
ENSG00000128487	-0,0941042	2,40907476	0,24548583	0,63182872	0,8278404	SPECC1	sperm antigen with calponin homology and coiled-coil domains 1 [Source:HGNC Symbol;Acc:HGNC:30615]
ENSG00000163946	-0,0783786	4,13073806	0,24542756	0,63186865	0,8278404	TASOR	transcription activation suppressor [Source:HGNC Symbol;Acc:HGNC:30314]
ENSG00000135829	0,10298405	7,74190451	0,24486561	0,6322541	0,82824607	DHX9	DExH-box helicase 9 [Source:HGNC Symbol;Acc:HGNC:2750]
ENSG00000135722	0,17542161	0,29535954	0,24477911	0,63231348	0,82824959	FBXL8	F-box and leucine rich repeat protein 8 [Source:HGNC Symbol;Acc:HGNC:17875]
ENSG00000162368	0,08067447	7,13524621	0,24459161	0,63244224	0,82834397	CMPK1	cytidine/uridine monophosphate kinase 1 [Source:HGNC Symbol;Acc:HGNC:18170]
ENSG00000175467	-0,1110642	6,11849582	0,2442495	0,63267734	0,82836699	SART1	spliceosome associated factor 1, recruiter of U4/U6.U5 tri-snRNP [Source:HGNC Symbol;Acc:HGNC:10538]
ENSG00000180385	0,17908632	-0,3223726	0,2443343	0,63261905	0,82836699	EMC3-AS1	EMC3 antisense RNA 1 [Source:HGNC Symbol;Acc:HGNC:49223]
ENSG00000197841	-0,0979929	2,34058615	0,24423599	0,63268662	0,82836699	ZNF181	zinc finger protein 181 [Source:HGNC Symbol;Acc:HGNC:12971]
ENSG00000225953	0,15835407	-0,01846	0,24438243	0,63258596	0,82836699	SATB2-AS1	SATB2 antisense RNA 1 [Source:HGNC Symbol;Acc:HGNC:26490]
ENSG00000049618	-0,1287995	4,55416146	0,2440391	0,63282202	0,82847002	ARID1B	AT-rich interaction domain 1B [Source:HGNC Symbol;Acc:HGNC:18040]
ENSG00000099814	-0,1238628	4,73374495	0,24382046	0,63297246	0,82859272	CEP170B	centrosomal protein 170B [Source:HGNC Symbol;Acc:HGNC:20362]
ENSG00000171970	-0,0923911	3,76037129	0,24358435	0,63313502	0,82873125	ZNF57	zinc finger protein 57 [Source:HGNC Symbol;Acc:HGNC:13125]
ENSG00000127952	-0,0789282	5,34290239	0,24331954	0,63331745	0,82874728	STYXL1	serine/threonine/tyrosine interacting like 1 [Source:HGNC Symbol;Acc:HGNC:18165]
ENSG00000172728	0,10935126	3,42863712	0,24346712	0,63321576	0,82874728	FUT10	fucosyltransferase 10 [Source:HGNC Symbol;Acc:HGNC:19234]
ENSG00000176946	-0,0663005	7,13880582	0,24337454	0,63327955	0,82874728	THAP4	THAP domain containing 4 [Source:HGNC Symbol;Acc:HGNC:23187]
ENSG00000234684	0,1698197	-0,191032	0,2429688	0,63355927	0,82898947	SDCBP2-AS1	SDCBP2 antisense RNA 1 [Source:HGNC Symbol;Acc:HGNC:44314]
ENSG00000077458	0,08979686	2,29886394	0,24269061	0,63375123	0,82902143	FAM76B	family with sequence similarity 76 member B [Source:HGNC Symbol;Acc:HGNC:28492]
ENSG00000144741	0,08933696	4,41600513	0,24284393	0,63364542	0,82902143	SLC25A26	solute carrier family 25 member 26 [Source:HGNC Symbol;Acc:HGNC:20661]
ENSG00000196091	0,30384749	-1,3797008	0,24268669	0,63375394	0,82902143	MYBPC1	myosin binding protein C1 [Source:HGNC Symbol;Acc:HGNC:7549]
ENSG00000133773	-0,0735229	5,35898142	0,24246158	0,63390937	0,82914877	CCDC59	coiled-coil domain containing 59 [Source:HGNC Symbol;Acc:HGNC:25005]
ENSG00000239900	-0,0790184	3,30161523	0,24238134	0,6339648	0,82914877	ADSL	adenylosuccinate lyase [Source:HGNC Symbol;Acc:HGNC:291]
ENSG00000185436	0,09079944	3,55682304	0,24208403	0,63417027	0,82934326	IFNLR1	interferon lambda receptor 1 [Source:HGNC Symbol;Acc:HGNC:18584]
ENSG00000139160	0,20726882	-1,0678848	0,24188121	0,63431053	0,82935238	ETFBKMT	electron transfer flavoprotein subunit beta lysine methyltransferase [Source:HGNC Symbol;Acc:HGNC:28739]
ENSG00000171236	-0,1023371	6,66322856	0,24182769	0,63434756	0,82935238	LRG1	leucine rich alpha-2-glycoprotein 1 [Source:HGNC Symbol;Acc:HGNC:29480]
ENSG00000184083	-0,0920678	2,38540821	0,24191319	0,63428841	0,82935238	FAM120C	family with sequence similarity 120C [Source:HGNC Symbol;Acc:HGNC:16949]
ENSG00000143376	0,16518911	3,52590465	0,24160052	0,63450477	0,82948368	SNX27	sorting nexin 27 [Source:HGNC Symbol;Acc:HGNC:20073]

ENSG00000146425	-0,0782676	5,17942086	0,24137558	0,63466053	0,82961307	DYNLT1	dynein light chain Tctex-type 1 [Source:HGNC Symbol;Acc:HGNC:11697]
ENSG00000243955	0,09014216	5,30825891	0,2412588	0,63474143	0,82964458	GSTA1	glutathione S-transferase alpha 1 [Source:HGNC Symbol;Acc:HGNC:4626]
ENSG00000132676	-0,0771226	7,20385425	0,24112312	0,63483545	0,82969324	DAP3	death associated protein 3 [Source:HGNC Symbol;Acc:HGNC:2673]
ENSG00000134982	0,13247848	1,75997896	0,24090368	0,63498759	0,82981784	APC	APC regulator of WNT signaling pathway [Source:HGNC Symbol;Acc:HGNC:583]
ENSG00000223396	-0,1446284	0,00083318	0,24068688	0,63513798	0,82994014	RPS10P7	ribosomal protein S10 pseudogene 7 [Source:HGNC Symbol;Acc:HGNC:36423]
ENSG00000230513	-0,1799865	-0,2363936	0,2405444	0,63523687	0,82999512	THAP7-AS1	THAP7 antisense RNA 1 [Source:HGNC Symbol;Acc:HGNC:41013]
ENSG00000150995	-0,1461952	2,89317321	0,23981166	0,63574599	0,83028904	ITPR1	inositol 1,4,5-trisphosphate receptor type 1 [Source:HGNC Symbol;Acc:HGNC:6180]
ENSG00000155099	-0,106659	1,62863568	0,24006767	0,635568	0,83028904	PIP4P2	phosphatidylinositol-4,5-bisphosphate 4-phosphatase 2 [Source:HGNC Symbol;Acc:HGNC:25452]
ENSG00000160345	-0,1473672	2,19300407	0,23987094	0,63570477	0,83028904	C9orf116	chromosome 9 open reading frame 116 [Source:HGNC Symbol;Acc:HGNC:28435]
ENSG00000163539	-0,0978394	3,59568446	0,23987605	0,63570122	0,83028904	CLASP2	cytoplasmic linker associated protein 2 [Source:HGNC Symbol;Acc:HGNC:17078]
ENSG00000173821	0,15413659	4,60420439	0,23995562	0,63564589	0,83028904	RNF213	ring finger protein 213 [Source:HGNC Symbol;Acc:HGNC:14539]
ENSG00000116977	-0,0705355	5,83058	0,23935178	0,63606603	0,83028994	LGALS8	galectin 8 [Source:HGNC Symbol;Acc:HGNC:6569]
ENSG00000158296	-0,0697108	6,72377734	0,23932068	0,63608769	0,83028994	SLC13A3	solute carrier family 13 member 3 [Source:HGNC Symbol;Acc:HGNC:14430]
ENSG00000172974	-0,1281405	0,99664699	0,23966368	0,63584894	0,83028994	VDAC2P5	VDAC2 pseudogene 5 [Source:HGNC Symbol;Acc:HGNC:54753]
ENSG00000222009	-0,2399805	-0,3177744	0,23938509	0,63604284	0,83028994	BTBD19	BTB domain containing 19 [Source:HGNC Symbol;Acc:HGNC:27145]
ENSG00000238227	-0,0772125	4,57819698	0,23945241	0,63599597	0,83028994	TMEM250	transmembrane protein 250 [Source:HGNC Symbol;Acc:HGNC:31009]
ENSG00000285531	-0,1784933	-0,5093161	0,23962062	0,6358789	0,83028994	Z83840.1	LLP homolog, long-term synaptic facilitation (LLPH) pseudogene
ENSG00000117385	-0,097328	6,79948673	0,2392047	0,63616846	0,83032119	P3H1	prolyl 3-hydroxylase 1 [Source:HGNC Symbol;Acc:HGNC:19316]
ENSG00000101247	0,06640679	5,4963303	0,2390633	0,63626698	0,83037559	NDUFAF5	NADH:ubiquinone oxidoreductase complex assembly factor 5 [Source:HGNC Symbol;Acc:HGNC:15899]
ENSG00000197582	0,15076647	-0,129873	0,23892097	0,63636619	0,83043088	GPX1P1	glutathione peroxidase pseudogene 1 [Source:HGNC Symbol;Acc:HGNC:4560]
ENSG00000137054	-0,071099	5,81627941	0,23809489	0,63694269	0,83061648	POLR1E	RNA polymerase I subunit E [Source:HGNC Symbol;Acc:HGNC:17631]
ENSG00000145040	-0,2730005	-0,3336492	0,2381379	0,63691265	0,83061648	UCN2	urocortin 2 [Source:HGNC Symbol;Acc:HGNC:18414]
ENSG00000153037	-0,0901506	3,82162142	0,23844649	0,63669716	0,83061648	SRP19	signal recognition particle 19 [Source:HGNC Symbol;Acc:HGNC:11300]
ENSG00000154814	0,09132176	3,78346739	0,23835588	0,63676042	0,83061648	OXNAD1	oxidoreductase NAD binding domain containing 1 [Source:HGNC Symbol;Acc:HGNC:25128]
ENSG00000188460	0,250257	-1,4246891	0,23810099	0,63693843	0,83061648	ACTBP11	ACTB pseudogene 11 [Source:HGNC Symbol;Acc:HGNC:33971]
ENSG00000198848	0,16519409	7,25409457	0,23806544	0,63696327	0,83061648	CES1	carboxylesterase 1 [Source:HGNC Symbol;Acc:HGNC:1863]
ENSG00000235903	-0,2238639	-1,4560301	0,23822144	0,6368543	0,83061648	CPB2-AS1	CPB2 antisense RNA 1 [Source:HGNC Symbol;Acc:HGNC:39898]
ENSG00000236148	-0,1216087	0,80725876	0,23824987	0,63683444	0,83061648	RPL23AP37	ribosomal protein L23a pseudogene 37 [Source:HGNC Symbol;Acc:HGNC:35664]
ENSG00000151135	0,07256418	3,95975313	0,23775476	0,63718043	0,83082549	TMEM263	transmembrane protein 263 [Source:HGNC Symbol;Acc:HGNC:28281]
ENSG00000047410	0,1843527	6,11598513	0,23758352	0,6373002	0,8309075	TPR	translocated promoter region, nuclear basket protein [Source:HGNC Symbol;Acc:HGNC:12017]
ENSG00000287126	0,14884069	0,12135315	0,23717175	0,63758842	0,83120911	AC108488.3	novel transcript
ENSG00000163904	0,06844423	5,33992271	0,23691441	0,63776871	0,83136996	SENP2	SUMO specific peptidase 2 [Source:HGNC Symbol;Acc:HGNC:23116]
ENSG00000085832	-0,084784	3,96446287	0,23665477	0,63795074	0,83138469	EPS15	epidermal growth factor receptor pathway substrate 15 [Source:HGNC Symbol;Acc:HGNC:3419]
ENSG00000130545	-0,0911886	4,08134117	0,23669794	0,63792046	0,83138469	CRB3	crumbs cell polarity complex component 3 [Source:HGNC Symbol;Acc:HGNC:20237]
ENSG00000182173	0,07667028	5,74917105	0,23676381	0,63787428	0,83138469	TSEN54	tRNA splicing endonuclease subunit 54 [Source:HGNC Symbol;Acc:HGNC:27561]
ENSG00000224834	-0,2369063	-1,446076	0,23654917	0,63802481	0,83140705	BTF3P9	basic transcription factor 3 pseudogene 9 [Source:HGNC Symbol;Acc:HGNC:38573]
ENSG00000271525	-0,2921551	-1,8029047	0,23626938	0,63822116	0,83158873	ARF4P1	ADP ribosylation factor 4 pseudogene 1 [Source:HGNC Symbol;Acc:HGNC:657]
ENSG00000159110	0,07980398	2,78879753	0,2361611	0,63829718	0,83161362	IFNAR2	interferon alpha and beta receptor subunit 2 [Source:HGNC Symbol;Acc:HGNC:5433]
ENSG00000013275	-0,082429	8,186188	0,23574366	0,63859048	0,83162492	PSMC4	proteasome 26S subunit, ATPase 4 [Source:HGNC Symbol;Acc:HGNC:9551]
ENSG00000164088	0,11516854	1,28001709	0,23600205	0,63840889	0,83162492	PPM1M	protein phosphatase, Mg2+/Mn2+ dependent 1M [Source:HGNC Symbol;Acc:HGNC:26506]
ENSG00000213801	-0,33824	-2,0026374	0,2357518	0,63858476	0,83162492	ZNF321P	zinc finger protein 321, pseudogene [Source:HGNC Symbol;Acc:HGNC:13827]
ENSG00000223658	0,09252703	2,09348099	0,23584287	0,63852075	0,83162492	C1GALT1C1L	C1GALT1 specific chaperone 1 like [Source:HGNC Symbol;Acc:HGNC:51617]
ENSG00000248180	-0,2034885	-1,2135292	0,23597088	0,6384308	0,83162492	GAPDHP60	glyceraldehyde-3-phosphate dehydrogenase pseudogene 60 [Source:HGNC Symbol;Acc:HGNC:4153]
ENSG00000263266	-0,0803412	4,14108132	0,23564543	0,63865955	0,83164073	RPS7P1	ribosomal protein S7 pseudogene 1 [Source:HGNC Symbol;Acc:HGNC:17081]

ENSG00000079246	0,08831472	9,1248883	0,23529921	0,63890312	0,83177983	XRCC5	X-ray repair cross complementing 5 [Source:HGNC Symbol;Acc:HGNC:12833]
ENSG00000100410	-0,0658416	5,81728799	0,2352699	0,63892375	0,83177983	PHF5A	PHD finger protein 5A [Source:HGNC Symbol;Acc:HGNC:18000]
ENSG00000143368	-0,0689884	6,30608367	0,23525082	0,63893718	0,83177983	SF3B4	splicing factor 3b subunit 4 [Source:HGNC Symbol;Acc:HGNC:10771]
ENSG00000159588	0,26834962	-1,4811673	0,2349852	0,63912422	0,83194919	CCDC17	coiled-coil domain containing 17 [Source:HGNC Symbol;Acc:HGNC:26574]
ENSG00000183474	-0,071596	3,65766049	0,23481277	0,63924572	0,83203321	GTF2H2C	GTF2H2 family member C [Source:HGNC Symbol;Acc:HGNC:31394]
ENSG00000126464	-0,1324139	3,14526035	0,23436008	0,63956495	0,83237455	PRR12	proline rich 12 [Source:HGNC Symbol;Acc:HGNC:29217]
ENSG00000049769	0,25837261	-1,73779	0,23395095	0,63985379	0,8325593	PPP1R3F	protein phosphatase 1 regulatory subunit 3F [Source:HGNC Symbol;Acc:HGNC:14944]
ENSG00000261777	-0,2788882	-2,1084026	0,23391687	0,63987787	0,8325593	AC012184.3	novel transcript, antisense to DDX19B and DDX19A
ENSG00000280071	-0,1893429	-0,5848983	0,2339986	0,63982013	0,8325593	GATD3B	glutamine amidotransferase like class 1 domain containing 3B [Source:HGNC Symbol;Acc:HGNC:53816]
ENSG00000058453	-0,1359688	1,6140211	0,2333191	0,6403005	0,83266423	CROCC	ciliary rootlet coiled-coil, rootletin [Source:HGNC Symbol;Acc:HGNC:21299]
ENSG00000101224	0,06669052	5,95154878	0,23355539	0,64013336	0,83266423	CDC25B	cell division cycle 25B [Source:HGNC Symbol;Acc:HGNC:1726]
ENSG00000169223	0,07634738	9,15292855	0,23340069	0,64024277	0,83266423	LMAN2	lectin, mannose binding 2 [Source:HGNC Symbol;Acc:HGNC:16986]
ENSG00000181638	-0,143818	1,26717956	0,23337402	0,64026164	0,83266423	ZFP41	ZFP41 zinc finger protein [Source:HGNC Symbol;Acc:HGNC:26786]
ENSG00000204623	0,14431888	0,41347491	0,23338905	0,64025101	0,83266423	ZNRD1ASP	zinc ribbon domain containing 1 antisense, pseudogene [Source:HGNC Symbol;Acc:HGNC:13924]
ENSG00000251596	-0,2782602	-1,6647324	0,23355475	0,64013381	0,83266423	HADHAP1	HADHA pseudogene 1 [Source:HGNC Symbol;Acc:HGNC:4802]
ENSG00000184925	-0,2936725	-1,2811775	0,23309085	0,64046206	0,83280019	LCN12	lipocalin 12 [Source:HGNC Symbol;Acc:HGNC:28733]
ENSG00000079691	0,17532178	0,18061063	0,23268862	0,64074699	0,83309654	CARMIL1	capping protein regulator and myosin 1 linker 1 [Source:HGNC Symbol;Acc:HGNC:21581]
ENSG00000066230	-0,2325996	-1,669617	0,23249196	0,64088641	0,83320319	SLC9A3	solute carrier family 9 member A3 [Source:HGNC Symbol;Acc:HGNC:11073]
ENSG00000102226	0,12313522	5,6650967	0,23237886	0,64096663	0,83320319	USP11	ubiquitin specific peptidase 11 [Source:HGNC Symbol;Acc:HGNC:12609]
ENSG00000136770	-0,0737169	5,24029685	0,2322729	0,64104181	0,83320319	DNAJC1	DnaJ heat shock protein family (Hsp40) member C1 [Source:HGNC Symbol;Acc:HGNC:20090]
ENSG00000205534	-0,151651	0,42268229	0,23225128	0,64105715	0,83320319	SMG1P2	SMG1 pseudogene 2 [Source:HGNC Symbol;Acc:HGNC:49859]
ENSG00000122378	0,06718758	7,64888764	0,23194039	0,64127785	0,83333632	PRXL2A	peroxiredoxin like 2A [Source:HGNC Symbol;Acc:HGNC:28651]
ENSG00000198740	0,12466601	2,78710475	0,23198703	0,64124473	0,83333632	ZNF652	zinc finger protein 652 [Source:HGNC Symbol;Acc:HGNC:29147]
ENSG00000215867	-0,2704421	-1,330762	0,23186598	0,6413307	0,83333632	KRT18P57	keratin 18 pseudogene 57 [Source:HGNC Symbol;Acc:HGNC:48884]
ENSG00000136731	-0,1419522	6,88734598	0,23151559	0,64157972	0,83358575	UGGT1	UDP-glucose glycoprotein glucosyltransferase 1 [Source:HGNC Symbol;Acc:HGNC:15663]
ENSG00000281912	0,21129249	-0,2584649	0,23101291	0,64193738	0,83397628	LINC01144	long intergenic non-protein coding RNA 1144 [Source:HGNC Symbol;Acc:HGNC:49460]
ENSG00000205213	0,06710517	5,0863448	0,23038039	0,64238811	0,83448764	LGR4	leucine rich repeat containing G protein-coupled receptor 4 [Source:HGNC Symbol;Acc:HGNC:13299]
ENSG00000067334	-0,0908598	6,12591515	0,23020903	0,64251036	0,8345325	DNTTIP2	deoxynucleotidyltransferase terminal interacting protein 2 [Source:HGNC Symbol;Acc:HGNC:24013]
ENSG00000087460	-0,0684636	10,2579723	0,23017184	0,6425369	0,8345325	GNAS	GNAS complex locus [Source:HGNC Symbol;Acc:HGNC:4392]
ENSG00000101189	-0,0770391	6,25813079	0,22994836	0,64269642	0,83459129	MRGBP	MRG domain binding protein [Source:HGNC Symbol;Acc:HGNC:15866]
ENSG00000217241	0,1614879	-0,0459551	0,22997431	0,6426779	0,83459129	CBX3P9	chromobox 3 pseudogene 9 [Source:HGNC Symbol;Acc:HGNC:42881]
ENSG00000147894	0,21346198	-0,3611576	0,22981529	0,64279146	0,83464052	C9orf72	C9orf72-SMCR8 complex subunit [Source:HGNC Symbol;Acc:HGNC:28337]
ENSG00000264281	-0,1822405	-0,8804254	0,22957965	0,64295983	0,83478494	AC016596.2	ribosomal protein L41 (RPL41) pseudogene
ENSG00000224858	0,15113012	0,00443454	0,22933847	0,64313228	0,83486044	RPL29P11	ribosomal protein L29 pseudogene 11 [Source:HGNC Symbol;Acc:HGNC:36905]
ENSG00000278212	0,15530787	0,18873628	0,22940465	0,64308494	0,83486044	AC134878.2	MAFF interacting protein (pseudogene)
ENSG00000119596	0,16279532	4,63348054	0,22919512	0,64323482	0,83491937	YLPM1	YLP motif containing 1 [Source:HGNC Symbol;Acc:HGNC:17798]
ENSG00000162415	0,12771287	3,72443441	0,22907806	0,64331859	0,83495392	ZSWIM5	zinc finger SWIM-type containing 5 [Source:HGNC Symbol;Acc:HGNC:29299]
ENSG00000006453	-0,0744497	5,9230988	0,22828302	0,64388825	0,83517389	BAIAP2L1	BAR/IMD domain containing adaptor protein 2 like 1 [Source:HGNC Symbol;Acc:HGNC:21649]
ENSG00000007944	-0,1173098	1,475384	0,22875217	0,64355195	0,83517389	MYLIP	myosin regulatory light chain interacting protein [Source:HGNC Symbol;Acc:HGNC:21155]
ENSG00000116871	-0,0700105	4,89611761	0,22855936	0,64369011	0,83517389	MAP7D1	MAP7 domain containing 1 [Source:HGNC Symbol;Acc:HGNC:25514]
ENSG00000120217	0,38351994	-1,9752382	0,22859394	0,64366532	0,83517389	CD274	CD274 molecule [Source:HGNC Symbol;Acc:HGNC:17635]
ENSG00000129084	-0,082113	3,75252245	0,22841326	0,64379485	0,83517389	PSMA1	proteasome 20S subunit alpha 1 [Source:HGNC Symbol;Acc:HGNC:9530]
ENSG00000151287	0,16088968	2,17327327	0,22835613	0,64383582	0,83517389	TEX30	testis expressed 30 [Source:HGNC Symbol;Acc:HGNC:25188]
ENSG00000184178	0,10620382	3,86660903	0,2284942	0,64373682	0,83517389	SCFD2	sec1 family domain containing 2 [Source:HGNC Symbol;Acc:HGNC:30676]

ENSG00000179348	-0,0741785	4,1076193	0,22789529	0,64416652	0,83546064	GATA2	GATA binding protein 2 [Source:HGNC Symbol;Acc:HGNC:4171]
ENSG00000175455	0,09627633	2,58964076	0,2278124	0,64422604	0,83546367	CCDC14	coiled-coil domain containing 14 [Source:HGNC Symbol;Acc:HGNC:25766]
ENSG00000226733	0,29476779	-1,7878815	0,22712321	0,6447215	0,83595778	AL138826.1	uncharacterized LOC100505985 [Source:NCBI gene (formerly Entrezgene);Acc:100505985]
ENSG00000230409	-0,2111143	-0,7976361	0,22712715	0,64471866	0,83595778	TCEA1P2	transcription elongation factor A1 pseudogene 2 [Source:HGNC Symbol;Acc:HGNC:29891]
ENSG00000115484	0,07585098	9,02095196	0,22676665	0,64497819	0,8361422	CCT4	chaperonin containing TCP1 subunit 4 [Source:HGNC Symbol;Acc:HGNC:1617]
ENSG00000119125	0,18017364	-0,1519015	0,22679728	0,64495613	0,8361422	GDA	guanine deaminase [Source:HGNC Symbol;Acc:HGNC:4212]
ENSG00000114270	-0,2641209	-0,4351191	0,22636262	0,64526936	0,83637123	COL7A1	collagen type VII alpha 1 chain [Source:HGNC Symbol;Acc:HGNC:2214]
ENSG00000140443	0,13590577	5,34886044	0,22640303	0,64524023	0,83637123	IGF1R	insulin like growth factor 1 receptor [Source:HGNC Symbol;Acc:HGNC:5465]
ENSG00000213661	-0,3238582	-1,378055	0,22623996	0,64535782	0,83641168	AC106806.1	ribosomal protein L18 (RPL18) pseudogene
ENSG00000104133	-0,1027259	3,71323861	0,22596839	0,64555379	0,8364866	SPG11	SPG11 vesicle trafficking associated, spatacsin [Source:HGNC Symbol;Acc:HGNC:11226]
ENSG00000131002	0,07889112	3,59911364	0,22584252	0,64564467	0,8364866	TXLNGY	taxilin gamma pseudogene, Y-linked [Source:HGNC Symbol;Acc:HGNC:18473]
ENSG00000279088	-0,1495282	0,93506786	0,22587245	0,64562305	0,8364866	AC022400.6	TEC
ENSG00000286540	-0,1120466	1,08614596	0,22589328	0,64560801	0,8364866	AL096854.1	novel transcript
ENSG00000198242	-0,0724523	9,43741347	0,22545753	0,64592282	0,83669857	RPL23A	ribosomal protein L23a [Source:HGNC Symbol;Acc:HGNC:10317]
ENSG00000274828	-0,1057886	1,47363606	0,22548756	0,64590111	0,83669857	AC068473.5	novel transcript
ENSG00000172671	0,13635384	1,15334796	0,2253679	0,64598762	0,83670831	ZFAND4	zinc finger AN1-type containing 4 [Source:HGNC Symbol;Acc:HGNC:23504]
ENSG00000149016	0,0968511	2,89645185	0,22509446	0,6461854	0,83682738	TUT1	terminal uridylyl transferase 1, U6 snRNA-specific [Source:HGNC Symbol;Acc:HGNC:26184]
ENSG00000243667	0,12503012	1,30438499	0,22508244	0,64619411	0,83682738	WDR92	WD repeat domain 92 [Source:HGNC Symbol;Acc:HGNC:25176]
ENSG00000103415	0,06790009	6,71798663	0,22458784	0,64655225	0,83721697	HMOX2	heme oxygenase 2 [Source:HGNC Symbol;Acc:HGNC:5014]
ENSG00000077684	0,06759938	4,70530118	0,22411093	0,64689806	0,83751628	JADE1	jade family PHD finger 1 [Source:HGNC Symbol;Acc:HGNC:30027]
ENSG00000123243	0,11491181	3,49008998	0,2241344	0,64688103	0,83751628	ITIH5	inter-alpha-trypsin inhibitor heavy chain 5 [Source:HGNC Symbol;Acc:HGNC:21449]
ENSG00000177542	0,07652861	3,90950171	0,22384302	0,64709251	0,83764651	SLC25A22	solute carrier family 25 member 22 [Source:HGNC Symbol;Acc:HGNC:19954]
ENSG00000258033	0,19984803	-0,8062042	0,22381437	0,64711332	0,83764651	SNX5P2	sorting nexin 5 pseudogene 2 [Source:HGNC Symbol;Acc:HGNC:41513]
ENSG00000038532	0,08050157	4,51658379	0,22348969	0,6473492	0,8378776	CLEC16A	C-type lectin domain containing 16A [Source:HGNC Symbol;Acc:HGNC:29013]
ENSG00000228782	-0,0863727	1,91890529	0,22337635	0,64743159	0,83791001	MRPL45P2	mitochondrial ribosomal protein L45 pseudogene 2 [Source:HGNC Symbol;Acc:HGNC:29716]
ENSG00000206417	0,15274285	0,27094421	0,22321005	0,64755252	0,83799228	H1-10-AS1	H1-10 antisense RNA 1 [Source:HGNC Symbol;Acc:HGNC:27953]
ENSG00000184640	-0,0870869	8,81022805	0,22292118	0,64776273	0,83819006	SEPTIN9	septin 9 [Source:HGNC Symbol;Acc:HGNC:7323]
ENSG00000110536	0,06909952	4,92096519	0,22237202	0,64816281	0,83841071	PTPMT1	protein tyrosine phosphatase mitochondrial 1 [Source:HGNC Symbol;Acc:HGNC:26965]
ENSG00000169813	-0,0687318	8,90784397	0,22247637	0,64808675	0,83841071	HNRNPF	heterogeneous nuclear ribonucleoprotein F [Source:HGNC Symbol;Acc:HGNC:5039]
ENSG00000251095	0,1766379	-0,1269213	0,22251879	0,64805583	0,83841071	AC093866.1	novel transcript
ENSG00000271155	0,2217357	-0,8134017	0,22241391	0,64813228	0,83841071	AL161729.1	novel transcript, antisense to PTCH1
ENSG00000198947	-0,0719068	4,13922454	0,22215909	0,6483181	0,8384631	DMD	dystrophin [Source:HGNC Symbol;Acc:HGNC:2928]
ENSG00000268364	0,18059557	-0,4654259	0,22217659	0,64830534	0,8384631	SMC5-AS1	SMC5 antisense RNA 1 (head to head) [Source:HGNC Symbol;Acc:HGNC:48718]
ENSG00000062650	0,06843325	5,37495615	0,22171016	0,64864582	0,83851577	WAPL	WAPL cohesin release factor [Source:HGNC Symbol;Acc:HGNC:23293]
ENSG00000108405	-0,3140413	-1,6325884	0,22200546	0,64843021	0,83851577	P2RX1	purinergic receptor P2X 1 [Source:HGNC Symbol;Acc:HGNC:8533]
ENSG00000148660	-0,0692182	4,22977892	0,22184137	0,64854999	0,83851577	CAMK2G	calcium/calmodulin dependent protein kinase II gamma [Source:HGNC Symbol;Acc:HGNC:1463]
ENSG00000227347	-0,1491681	-0,0054439	0,22173944	0,64862443	0,83851577	HNRNPKP2	heterogeneous nuclear ribonucleoprotein K pseudogene 2 [Source:HGNC Symbol;Acc:HGNC:42375]
ENSG00000227525	0,19883604	-1,1380861	0,22188314	0,6485195	0,83851577	RPL7P6	ribosomal protein L7 pseudogene 6 [Source:HGNC Symbol;Acc:HGNC:32430]
ENSG00000100941	-0,0988479	7,43013035	0,22157187	0,64874685	0,83857217	PNN	pinin, desmosome associated protein [Source:HGNC Symbol;Acc:HGNC:9162]
ENSG00000066136	-0,0629717	5,56447475	0,22141988	0,64885794	0,83857767	NFYC	nuclear transcription factor Y subunit gamma [Source:HGNC Symbol;Acc:HGNC:7806]
ENSG00000115350	0,08145725	5,78915577	0,22134691	0,64891128	0,83857767	POLE4	DNA polymerase epsilon 4, accessory subunit [Source:HGNC Symbol;Acc:HGNC:18755]
ENSG00000143437	-0,085246	4,52311006	0,22133046	0,64892331	0,83857767	ARNT	aryl hydrocarbon receptor nuclear translocator [Source:HGNC Symbol;Acc:HGNC:700]
ENSG00000137204	0,13808697	0,48795565	0,22120942	0,64901183	0,83861789	SLC22A7	solute carrier family 22 member 7 [Source:HGNC Symbol;Acc:HGNC:10971]
ENSG00000122435	0,08150545	2,74216105	0,22108407	0,64910354	0,83866221	TRMT13	tRNA methyltransferase 13 homolog [Source:HGNC Symbol;Acc:HGNC:25502]

ENSG00000197496	-0,0738197	3,29100689	0,22066958	0,649407	0,83898009	SLC2A10	solute carrier family 2 member 10 [Source:HGNC Symbol;Acc:HGNC:13444]
ENSG00000107290	0,07913745	5,09279327	0,21994715	0,64993677	0,8390709	SETX	senataxin [Source:HGNC Symbol;Acc:HGNC:445]
ENSG00000140044	-0,0989798	2,32586891	0,22040062	0,64960411	0,8390709	JDP2	Jun dimerization protein 2 [Source:HGNC Symbol;Acc:HGNC:17546]
ENSG00000171204	-0,0708882	4,62745321	0,22044047	0,6495749	0,8390709	TMEM126B	transmembrane protein 126B [Source:HGNC Symbol;Acc:HGNC:30883]
ENSG00000183527	-0,0744652	6,67552419	0,22028545	0,64968856	0,8390709	PSMG1	proteasome assembly chaperone 1 [Source:HGNC Symbol;Acc:HGNC:3043]
ENSG00000237793	-0,1159709	0,80082405	0,22011717	0,649812	0,8390709	RPL18AP16	ribosomal protein L18a pseudogene 16 [Source:HGNC Symbol;Acc:HGNC:36288]
ENSG00000247400	0,16229136	0,76809965	0,22007772	0,64984094	0,8390709	DNAJC3-DT	DNAJC3 divergent transcript [Source:HGNC Symbol;Acc:HGNC:39808]
ENSG00000259397	-0,2275021	-0,9141351	0,21997104	0,64991924	0,8390709	AC021231.2	U2 small nuclear RNA auxiliary factor 1 (U2AF1) pseudogene
ENSG00000274512	0,21214221	-0,8535645	0,22018357	0,64976329	0,8390709	TBC1D3L	TBC1 domain family member 3L [Source:HGNC Symbol;Acc:HGNC:51246]
ENSG00000095539	0,06067662	6,19899236	0,21975793	0,65007571	0,83914489	SEMA4G	semaphorin 4G [Source:HGNC Symbol;Acc:HGNC:10735]
ENSG00000182648	-0,2089268	-1,113232	0,21971265	0,65010897	0,83914489	LINC01006	long intergenic non-protein coding RNA 1006 [Source:HGNC Symbol;Acc:HGNC:48971]
ENSG00000166902	0,06630541	5,96792595	0,21960774	0,65018604	0,83917023	MRPL16	mitochondrial ribosomal protein L16 [Source:HGNC Symbol;Acc:HGNC:14476]
ENSG00000274499	0,28104101	-1,5339907	0,21923876	0,65045729	0,83944617	AC012414.6	coxsackie virus and adenovirus receptor (CXADR) pseudogene
ENSG00000133142	-0,094515	5,03713225	0,21897586	0,65065073	0,83962164	TCEAL4	transcription elongation factor A like 4 [Source:HGNC Symbol;Acc:HGNC:26121]
ENSG00000159247	0,11931177	1,85298143	0,21876291	0,65080753	0,83962661	TUBBP5	tubulin beta pseudogene 5 [Source:HGNC Symbol;Acc:HGNC:23674]
ENSG00000160323	-0,2619972	-1,4134554	0,21863061	0,65090499	0,83962661	ADAMTS13	ADAM metallopeptidase with thrombospondin type 1 motif 13 [Source:HGNC Symbol;Acc:HGNC:1366]
ENSG00000169087	0,09169098	1,99849943	0,21861367	0,65091747	0,83962661	HSPBAP1	HSPB1 associated protein 1 [Source:HGNC Symbol;Acc:HGNC:16389]
ENSG00000226970	-0,1885668	-1,1471863	0,21858045	0,65094195	0,83962661	NEDD8P1	NEDD8 pseudogene 1 [Source:HGNC Symbol;Acc:HGNC:54555]
ENSG00000261819	-0,1583336	0,64741154	0,21875081	0,65081644	0,83962661	AC138932.2	novel pseudogene KIAA2013
ENSG00000100344	-0,0709326	4,15563305	0,21793264	0,65141975	0,83993465	PNPLA3	patatin like phospholipase domain containing 3 [Source:HGNC Symbol;Acc:HGNC:18590]
ENSG00000125945	0,07870297	2,76481373	0,21792637	0,65142438	0,83993465	ZNF436	zinc finger protein 436 [Source:HGNC Symbol;Acc:HGNC:20814]
ENSG00000141867	0,18700254	5,11142263	0,21786697	0,65146824	0,83993465	BRD4	bromodomain containing 4 [Source:HGNC Symbol;Acc:HGNC:13575]
ENSG00000160584	-0,1250762	4,01275381	0,21804786	0,6513347	0,83993465	SIK3	SIK family kinase 3 [Source:HGNC Symbol;Acc:HGNC:29165]
ENSG00000267980	0,25783843	-1,6927527	0,21796811	0,65139357	0,83993465	AC007292.1	novel transcript, antisense to SH3GL1
ENSG00000042753	-0,0633894	6,7612023	0,21772615	0,65157224	0,8399347	AP2S1	adaptor related protein complex 2 subunit sigma 1 [Source:HGNC Symbol;Acc:HGNC:565]
ENSG00000172932	0,07645681	3,51357886	0,21771122	0,65158327	0,8399347	ANKRD13D	ankyrin repeat domain 13D [Source:HGNC Symbol;Acc:HGNC:27880]
ENSG00000102312	0,09769646	3,06548054	0,21741335	0,6518034	0,84003536	PORCN	porcupine O-acyltransferase [Source:HGNC Symbol;Acc:HGNC:17652]
ENSG00000160685	0,08239704	4,14315149	0,21721667	0,65194886	0,84003536	ZBTB7B	zinc finger and BTB domain containing 7B [Source:HGNC Symbol;Acc:HGNC:18668]
ENSG00000164403	-0,0728385	4,19338416	0,21733766	0,65185937	0,84003536	SHROOM1	shroom family member 1 [Source:HGNC Symbol;Acc:HGNC:24084]
ENSG00000219487	0,21282411	-0,5072345	0,21726942	0,65190984	0,84003536	AL603766.1	small nuclear ribonucleoprotein polypeptide B'' (SNRPB2) pseudogene
ENSG00000248663	0,26331881	-1,0091441	0,21734829	0,65185151	0,84003536	LINC00992	long intergenic non-protein coding RNA 992 [Source:HGNC Symbol;Acc:HGNC:48943]
ENSG00000105988	-0,2149304	-1,4743706	0,21662483	0,65238704	0,84015535	NHP2P1	NHP2 ribonucleoprotein pseudogene 1 [Source:HGNC Symbol;Acc:HGNC:23582]
ENSG00000136518	0,06694041	5,8167977	0,21675532	0,65229036	0,84015535	ACTL6A	actin like 6A [Source:HGNC Symbol;Acc:HGNC:24124]
ENSG00000164941	-0,0711216	4,13172272	0,21683767	0,65222938	0,84015535	INTS8	integrator complex subunit 8 [Source:HGNC Symbol;Acc:HGNC:26048]
ENSG00000182162	0,1203937	1,63253084	0,21664182	0,65237445	0,84015535	P2RY8	P2Y receptor family member 8 [Source:HGNC Symbol;Acc:HGNC:15524]
ENSG00000198417	-0,1273859	5,3960661	0,2168327	0,65223306	0,84015535	MT1F	metallothionein 1F [Source:HGNC Symbol;Acc:HGNC:7398]
ENSG00000266402	0,14725842	0,3895633	0,21686211	0,65221128	0,84015535	SNHG25	small nucleolar RNA host gene 25 [Source:HGNC Symbol;Acc:HGNC:51534]
ENSG00000163584	0,07388268	6,21430643	0,2164561	0,6525121	0,84024233	RPL22L1	ribosomal protein L22 like 1 [Source:HGNC Symbol;Acc:HGNC:27610]
ENSG00000051382	0,07712398	3,98251615	0,21630719	0,65262252	0,8402471	PIK3CB	phosphatidylinositol-4,5-bisphosphate 3-kinase catalytic subunit beta [Source:HGNC Symbol;Acc:HGNC:8976]
ENSG00000126012	0,08518632	5,91536365	0,21621844	0,65268835	0,8402471	KDM5C	lysine demethylase 5C [Source:HGNC Symbol;Acc:HGNC:11114]
ENSG00000146556	-0,1253642	0,95996482	0,21627178	0,65264878	0,8402471	WASH2P	WASP family homolog 2, pseudogene [Source:HGNC Symbol;Acc:HGNC:33145]
ENSG00000107341	0,08483709	6,2198293	0,21573969	0,65304376	0,84058405	UBE2R2	ubiquitin conjugating enzyme E2 R2 [Source:HGNC Symbol;Acc:HGNC:19907]
ENSG00000155115	-0,0754381	6,9672392	0,21571088	0,65306516	0,84058405	GTF3C6	general transcription factor IIIC subunit 6 [Source:HGNC Symbol;Acc:HGNC:20872]
ENSG00000121058	0,08153367	5,21822976	0,21557355	0,65316721	0,84064133	COIL	coilin [Source:HGNC Symbol;Acc:HGNC:2184]

ENSG00000116221	0,08336867	8,03276845	0,21531591	0,65335876	0,84073973	MRPL37	mitochondrial ribosomal protein L37 [Source:HGNC Symbol;Acc:HGNC:14034]
ENSG00000163607	-0,0772042	3,67579101	0,2153177	0,65335743	0,84073973	GTPBP8	GTP binding protein 8 (putative) [Source:HGNC Symbol;Acc:HGNC:25007]
ENSG00000108384	0,06398453	5,81949055	0,21504099	0,65356332	0,84092888	RAD51C	RAD51 paralog C [Source:HGNC Symbol;Acc:HGNC:9820]
ENSG00000178105	0,12231705	4,91373317	0,21488209	0,65368162	0,84100703	DDX10	DEAD-box helicase 10 [Source:HGNC Symbol;Acc:HGNC:2735]
ENSG00000073969	-0,0882044	4,9349548	0,21449888	0,65396716	0,8413003	NSF	N-ethylmaleimide sensitive factor, vesicle fusing ATPase [Source:HGNC Symbol;Acc:HGNC:8016]
ENSG00000171206	0,06241831	5,55402356	0,21430991	0,65410808	0,84133452	TRIM8	tripartite motif containing 8 [Source:HGNC Symbol;Acc:HGNC:15579]
ENSG00000245958	0,0916088	2,63291902	0,21430876	0,65410894	0,84133452	AC093752.1	septin 4 (sept4) pseudogene
ENSG00000129749	-0,2405437	-1,271843	0,21404635	0,65430475	0,84144169	CHRNA10	cholinergic receptor nicotinic alpha 10 subunit [Source:HGNC Symbol;Acc:HGNC:13800]
ENSG00000143228	0,06932494	4,57144018	0,21404273	0,65430745	0,84144169	NUF2	NUF2 component of NDC80 kinetochore complex [Source:HGNC Symbol;Acc:HGNC:14621]
ENSG00000224114	0,06983482	3,26235218	0,21387002	0,65443641	0,84153345	AL591846.1	ribosomal protein S14 (RPS14) pseudogene
ENSG00000057935	0,06306447	6,02694118	0,2136447	0,65460475	0,84167388	MTA3	metastasis associated 1 family member 3 [Source:HGNC Symbol;Acc:HGNC:23784]
ENSG00000169740	0,10832595	3,51965406	0,21356965	0,65466085	0,84167388	ZNF32	zinc finger protein 32 [Source:HGNC Symbol;Acc:HGNC:13095]
ENSG00000109805	0,06558575	5,16433844	0,21326159	0,65489123	0,84189598	NCAPG	non-SMC condensin I complex subunit G [Source:HGNC Symbol;Acc:HGNC:24304]
ENSG00000176619	0,11710081	7,08758917	0,21301559	0,65507534	0,84198449	LMNB2	lamin B2 [Source:HGNC Symbol;Acc:HGNC:6638]
ENSG00000274528	-0,2361679	-1,3127762	0,21303074	0,65506401	0,84198449	AC090970.2	novel transcript
ENSG00000136931	0,06776025	5,85592176	0,21290162	0,65516069	0,84201653	NR5A1	nuclear receptor subfamily 5 group A member 1 [Source:HGNC Symbol;Acc:HGNC:7983]
ENSG00000189339	0,07588298	3,79192977	0,21282839	0,65521555	0,84201653	SLC35E2B	solute carrier family 35 member E2B [Source:HGNC Symbol;Acc:HGNC:33941]
ENSG00000073050	0,08696531	5,65773197	0,21250856	0,65545524	0,84225048	XRCC1	X-ray repair cross complementing 1 [Source:HGNC Symbol;Acc:HGNC:12828]
ENSG00000245149	0,21058534	-1,3036237	0,21242986	0,65551426	0,84225223	RNF139-AS1	RNF139 antisense RNA 1 (head to head) [Source:HGNC Symbol;Acc:HGNC:48940]
ENSG00000253878	-0,2555164	-1,3957004	0,21231361	0,65560146	0,84229019	AC087752.3	novel transcript
ENSG00000166454	0,06415025	5,50717982	0,21206291	0,65578961	0,84238376	ATMIN	ATM interactor [Source:HGNC Symbol;Acc:HGNC:29034]
ENSG00000187792	0,15055113	1,25850532	0,21213224	0,65573757	0,84238376	ZNF70	zinc finger protein 70 [Source:HGNC Symbol;Acc:HGNC:13140]
ENSG00000106086	0,07112529	3,77269674	0,21136883	0,65631124	0,84260928	PLEKHA8	pleckstrin homology domain containing A8 [Source:HGNC Symbol;Acc:HGNC:30037]
ENSG00000136068	-0,1155011	7,12102968	0,21160062	0,65613693	0,84260928	FLNB	filamin B [Source:HGNC Symbol;Acc:HGNC:3755]
ENSG00000138821	0,35107577	-1,9392453	0,21145232	0,65624844	0,84260928	SLC39A8	solute carrier family 39 member 8 [Source:HGNC Symbol;Acc:HGNC:20862]
ENSG00000163956	-0,082806	8,53248591	0,21139776	0,65628947	0,84260928	LRPAP1	LDL receptor related protein associated protein 1 [Source:HGNC Symbol;Acc:HGNC:6701]
ENSG00000235698	0,2167703	-1,757959	0,21162085	0,65612172	0,84260928	PA2G4P2	proliferation-associated 2G4 pseudogene 2 [Source:HGNC Symbol;Acc:HGNC:16531]
ENSG00000259045	0,21468446	-0,8672429	0,21150642	0,65620775	0,84260928	MTCO1P2	MT-CO1 pseudogene 2 [Source:HGNC Symbol;Acc:HGNC:19932]
ENSG00000122882	0,07560818	5,65564736	0,21100427	0,65658564	0,8426531	ECD	ecdysoneless cell cycle regulator [Source:HGNC Symbol;Acc:HGNC:17029]
ENSG00000197355	-0,110543	5,03559885	0,21094035	0,65663378	0,8426531	UAP1L1	UDP-N-acetylglucosamine pyrophosphorylase 1 like 1 [Source:HGNC Symbol;Acc:HGNC:28082]
ENSG00000203705	-0,0747275	2,87281927	0,21113345	0,65648838	0,8426531	TATDN3	TatD DNase domain containing 3 [Source:HGNC Symbol;Acc:HGNC:27010]
ENSG00000231341	-0,2634234	-1,8374382	0,21097813	0,65660533	0,8426531	VDAC1P6	voltage dependent anion channel 1 pseudogene 6 [Source:HGNC Symbol;Acc:HGNC:37481]
ENSG00000272645	0,13930241	0,29079969	0,21119322	0,65644339	0,8426531	GTF2IP20	general transcription factor IIi pseudogene 20 [Source:HGNC Symbol;Acc:HGNC:51732]
ENSG00000070495	0,06418841	6,28982054	0,21060802	0,65688422	0,84290045	JMJD6	jumonji domain containing 6, arginine demethylase and lysine hydroxylase [Source:HGNC Symbol;Acc:HGNC:1935
ENSG00000234420	0,11170231	1,90082036	0,21048506	0,65697695	0,84294539	ZNF37BP	zinc finger protein 37B, pseudogene [Source:HGNC Symbol;Acc:HGNC:13103]
ENSG00000227502	0,23770961	-0,9770979	0,21040263	0,65703913	0,84295115	MROCKI	MARCKS cis regulating lncRNA promoter of cytokines and inflammation [Source:HGNC Symbol;Acc:HGNC:50323]
ENSG00000111785	-0,0812699	2,83998882	0,20986956	0,6574416	0,84339343	RIC8B	RIC8 guanine nucleotide exchange factor B [Source:HGNC Symbol;Acc:HGNC:25555]
ENSG00000147383	-0,0632479	7,44496782	0,20979118	0,65750083	0,84339535	NSDHL	NAD(P) dependent steroid dehydrogenase-like [Source:HGNC Symbol;Acc:HGNC:13398]
ENSG00000187049	-0,0687166	5,12877027	0,20964112	0,65761426	0,84346679	TMEM216	transmembrane protein 216 [Source:HGNC Symbol;Acc:HGNC:25018]
ENSG00000070087	0,08435934	5,36628729	0,20933817	0,65784342	0,84349555	PFN2	profilin 2 [Source:HGNC Symbol;Acc:HGNC:8882]
ENSG00000106100	-0,112848	1,32126981	0,20926153	0,65790143	0,84349555	NOD1	nucleotide binding oligomerization domain containing 1 [Source:HGNC Symbol;Acc:HGNC:16390]
ENSG00000149547	0,07213425	7,01051634	0,20943559	0,65776971	0,84349555	EI24	EI24 autophagy associated transmembrane protein [Source:HGNC Symbol;Acc:HGNC:13276]
ENSG00000151304	0,08067198	3,48267222	0,20915364	0,65798311	0,84349555	SRFBP1	serum response factor binding protein 1 [Source:HGNC Symbol;Acc:HGNC:26333]
ENSG00000170892	-0,0686475	6,35650356	0,20926608	0,65789798	0,84349555	TSEN34	tRNA splicing endonuclease subunit 34 [Source:HGNC Symbol;Acc:HGNC:15506]

ENSG00000204186	0,22231512	-0,8725177	0,20919392	0,65795261	0,84349555	ZDBF2	zinc finger DBF-type containing 2 [Source:HGNC Symbol;Acc:HGNC:29313]
ENSG00000131187	-0,1135059	2,04291912	0,20899009	0,65810697	0,8435803	F12	coagulation factor XII [Source:HGNC Symbol;Acc:HGNC:3530]
ENSG00000226688	0,0797826	3,63785301	0,20887497	0,65819419	0,84361809	ENTPD1-AS1	ENTPD1 antisense RNA 1 [Source:HGNC Symbol;Acc:HGNC:45203]
ENSG00000014216	-0,0967384	6,81635643	0,20842552	0,65853501	0,84362098	CAPN1	calpain 1 [Source:HGNC Symbol;Acc:HGNC:1476]
ENSG00000141076	-0,076033	6,48819679	0,20855004	0,65844054	0,84362098	UTP4	UTP4 small subunit processome component [Source:HGNC Symbol;Acc:HGNC:1983]
ENSG00000144535	-0,1004729	4,77235705	0,20841509	0,65854293	0,84362098	DIS3L2	DIS3 like 3'-5' exoribonuclease 2 [Source:HGNC Symbol;Acc:HGNC:28648]
ENSG00000175463	0,13664023	0,4500056	0,20876228	0,6582796	0,84362098	TBC1D10C	TBC1 domain family member 10C [Source:HGNC Symbol;Acc:HGNC:24702]
ENSG00000213588	0,08138459	4,65269089	0,20843375	0,65852877	0,84362098	ZBTB9	zinc finger and BTB domain containing 9 [Source:HGNC Symbol;Acc:HGNC:28323]
ENSG00000215187	0,26311251	-1,0177399	0,20859576	0,65840587	0,84362098	FAM166B	family with sequence similarity 166 member B [Source:HGNC Symbol;Acc:HGNC:34242]
ENSG00000198793	-0,1004999	4,9625191	0,20826021	0,65866048	0,84362361	MTOR	mechanistic target of rapamycin kinase [Source:HGNC Symbol;Acc:HGNC:3942]
ENSG00000239622	-0,1855873	-1,0248863	0,20828271	0,65864339	0,84362361	AC005165.2	ubiquitin A-52 residue ribosomal protein fusion product 1 (UBA52) pseudogeneA
ENSG00000144815	0,09199475	1,98898843	0,2081197	0,65876717	0,8436863	NXPE3	neurexophilin and PC-esterase domain family member 3 [Source:HGNC Symbol;Acc:HGNC:28238]
ENSG00000213771	-0,1794442	-0,2654057	0,20784225	0,65897798	0,8438095	KRT8P37	keratin 8 pseudogene 37 [Source:HGNC Symbol;Acc:HGNC:39871]
ENSG00000231721	0,27535403	-1,5394541	0,20784105	0,65897889	0,8438095	LINC-PINT	long intergenic non-protein coding RNA, p53 induced transcript [Source:HGNC Symbol;Acc:HGNC:26885]
ENSG00000132313	-0,0623536	6,32781927	0,20773898	0,65905648	0,84383489	MRPL35	mitochondrial ribosomal protein L35 [Source:HGNC Symbol;Acc:HGNC:14489]
ENSG00000012660	0,0823653	6,60844659	0,20740284	0,65931219	0,84385813	ELOVL5	ELOVL fatty acid elongase 5 [Source:HGNC Symbol;Acc:HGNC:21308]
ENSG00000108861	0,0612319	7,25964853	0,20718377	0,65947897	0,84385813	DUSP3	dual specificity phosphatase 3 [Source:HGNC Symbol;Acc:HGNC:3069]
ENSG00000134748	-0,0784557	6,19551387	0,20720492	0,65946286	0,84385813	PRPF38A	pre-mRNA processing factor 38A [Source:HGNC Symbol;Acc:HGNC:25930]
ENSG00000157214	0,08964087	2,34128369	0,20721229	0,65945725	0,84385813	STEAP2	STEAP2 metalloreductase [Source:HGNC Symbol;Acc:HGNC:17885]
ENSG00000187951	-0,1195495	0,74786258	0,20726849	0,65941446	0,84385813	AC091057.1	OTU deubiquitinase 7A pseudogene [Source:NCBI gene (formerly Entrezgene);Acc:100288637]
ENSG00000196381	-0,1605798	-0,3514982	0,20760353	0,65915949	0,84385813	ZNF781	zinc finger protein 781 [Source:HGNC Symbol;Acc:HGNC:26745]
ENSG00000213467	0,1695697	-0,6049624	0,20746086	0,65926803	0,84385813	HMGB1P37	high mobility group box 1 pseudogene 37 [Source:HGNC Symbol;Acc:HGNC:39184]
ENSG00000169174	0,0984253	8,31961785	0,20707644	0,65956073	0,84388883	PCSK9	proprotein convertase subtilisin/kexin type 9 [Source:HGNC Symbol;Acc:HGNC:20001]
ENSG00000116459	0,06169218	8,51100533	0,20639426	0,66008095	0,84440653	ATP5PB	ATP synthase peripheral stalk-membrane subunit b [Source:HGNC Symbol;Acc:HGNC:840]
ENSG00000120253	-0,0941694	5,1165094	0,20643809	0,66004749	0,84440653	NUP43	nucleoporin 43 [Source:HGNC Symbol;Acc:HGNC:21182]
ENSG00000178935	0,08265799	2,88542319	0,2062833	0,66016567	0,84444096	ZNF552	zinc finger protein 552 [Source:HGNC Symbol;Acc:HGNC:26135]
ENSG00000056998	0,06526673	4,98237838	0,20605413	0,66034072	0,84451699	GYG2	glycogenin 2 [Source:HGNC Symbol;Acc:HGNC:4700]
ENSG00000135253	-0,1762421	0,16202532	0,20611984	0,66029051	0,84451699	KCP	kielin cysteine rich BMP regulator [Source:HGNC Symbol;Acc:HGNC:17585]
ENSG00000285053	-0,117825	0,45259684	0,2059652	0,66040868	0,84452997	AL357556.3	Tubulin-specific chaperone E [Source:UniProtKB/Swiss-Prot;Acc:Q15813]
ENSG00000075292	0,06450527	5,70403871	0,20541087	0,66083273	0,84459033	ZNF638	zinc finger protein 638 [Source:HGNC Symbol;Acc:HGNC:17894]
ENSG00000088035	0,10572806	2,6103499	0,20538457	0,66085286	0,84459033	ALG6	ALG6 alpha-1,3-glucosyltransferase [Source:HGNC Symbol;Acc:HGNC:23157]
ENSG00000108797	0,13325812	2,67071862	0,20545916	0,66079576	0,84459033	CNTNAP1	contactin associated protein 1 [Source:HGNC Symbol;Acc:HGNC:8011]
ENSG00000113621	0,06198404	5,94073919	0,2056789	0,66062761	0,84459033	TXNDC15	thioredoxin domain containing 15 [Source:HGNC Symbol;Acc:HGNC:20652]
ENSG00000162881	0,16064869	1,09846518	0,20529901	0,66091838	0,84459033	OXER1	oxoeicosanoid receptor 1 [Source:HGNC Symbol;Acc:HGNC:24884]
ENSG00000204390	-0,1968238	-0,8952884	0,20539056	0,66084827	0,84459033	HSPA1L	heat shock protein family A (Hsp70) member 1 like [Source:HGNC Symbol;Acc:HGNC:5234]
ENSG00000221995	0,36668961	-1,0045371	0,20535451	0,66087587	0,84459033	TIAF1	TGFB1-induced anti-apoptotic factor 1 [Source:HGNC Symbol;Acc:HGNC:11803]
ENSG00000278133	0,17115968	0,67160121	0,20546425	0,66079186	0,84459033	AC135050.6	novel transcript, sense intronic to KAT8
ENSG00000115970	0,07226503	4,82121345	0,20507742	0,66108814	0,84467464	THADA	THADA armadillo repeat containing [Source:HGNC Symbol;Acc:HGNC:19217]
ENSG00000159556	-0,0751647	3,22459236	0,20506195	0,66109999	0,84467464	ISL2	ISL LIM homeobox 2 [Source:HGNC Symbol;Acc:HGNC:18524]
ENSG00000107551	-0,101127	4,542114	0,20471946	0,66136261	0,84475963	RASSF4	Ras association domain family member 4 [Source:HGNC Symbol;Acc:HGNC:20793]
ENSG00000137960	0,0783874	3,72771073	0,20467357	0,66139782	0,84475963	GIPC2	GIPC PDZ domain containing family member 2 [Source:HGNC Symbol;Acc:HGNC:18177]
ENSG00000197566	-0,1386201	0,59372194	0,20481244	0,66129129	0,84475963	ZNF624	zinc finger protein 624 [Source:HGNC Symbol;Acc:HGNC:29254]
ENSG00000259673	0,14611615	0,5480131	0,20483873	0,66127113	0,84475963	IQCH-AS1	IQCH antisense RNA 1 [Source:HGNC Symbol;Acc:HGNC:44104]
ENSG00000006757	0,07957013	4,93360022	0,20432144	0,66166814	0,84480947	PNPLA4	patatin like phospholipase domain containing 4 [Source:HGNC Symbol;Acc:HGNC:24887]

ENSG00000141378	-0,0588513	7,24567021	0,20438569	0,6616188	0,84480947	PTRH2	peptidyl-tRNA hydrolase 2 [Source:HGNC Symbol;Acc:HGNC:24265]
ENSG00000163719	0,06953021	5,78651911	0,20441606	0,66159547	0,84480947	MTMR14	myotubularin related protein 14 [Source:HGNC Symbol;Acc:HGNC:26190]
ENSG00000184432	0,0944455	7,78832775	0,20439237	0,66161367	0,84480947	COPB2	COPI coat complex subunit beta 2 [Source:HGNC Symbol;Acc:HGNC:2232]
ENSG00000142910	0,07822433	4,57867274	0,20420589	0,66175691	0,84484897	TINAGL1	tubulointerstitial nephritis antigen like 1 [Source:HGNC Symbol;Acc:HGNC:19168]
ENSG00000116095	-0,0769984	2,54718933	0,20412342	0,66182028	0,84485604	PLEKHA3	pleckstrin homology domain containing A3 [Source:HGNC Symbol;Acc:HGNC:14338]
ENSG00000186132	0,09348565	1,5661354	0,20395211	0,66195198	0,84495032	C2orf76	chromosome 2 open reading frame 76 [Source:HGNC Symbol;Acc:HGNC:27017]
ENSG00000148700	-0,070508	4,70348623	0,20387676	0,66200992	0,84495046	ADD3	adducin 3 [Source:HGNC Symbol;Acc:HGNC:245]
ENSG00000119042	-0,1326227	1,16344103	0,20358203	0,6622367	0,84516607	SATB2	SATB homeobox 2 [Source:HGNC Symbol;Acc:HGNC:21637]
ENSG00000137992	0,08324406	3,3454518	0,20323392	0,66250481	0,84528648	DBT	dihydrolipoamide branched chain transacylase E2 [Source:HGNC Symbol;Acc:HGNC:2698]
ENSG00000183486	-0,0687485	3,83725171	0,20315907	0,6625625	0,84528648	MX2	MX dynamin like GTPase 2 [Source:HGNC Symbol;Acc:HGNC:7533]
ENSG00000226535	-0,2510071	-1,7513403	0,20317704	0,66254865	0,84528648	AL445487.1	karyopherin alpha 2 (RAG cohort 1, importin alpha 1) (KPNA2) pseudogene
ENSG00000287811	-0,2349994	-1,7743798	0,20318834	0,66253994	0,84528648	AL391840.3	novel transcript
ENSG00000027075	0,13914668	0,61802489	0,20269814	0,66291801	0,84540759	PRKCH	protein kinase C eta [Source:HGNC Symbol;Acc:HGNC:9403]
ENSG00000101265	0,29404916	-1,1807726	0,20209234	0,66338601	0,84540759	RASSF2	Ras association domain family member 2 [Source:HGNC Symbol;Acc:HGNC:9883]
ENSG00000128185	0,07868616	5,03407873	0,20181958	0,66359701	0,84540759	DGCR6L	DiGeorge syndrome critical region gene 6 like [Source:HGNC Symbol;Acc:HGNC:18551]
ENSG00000129562	-0,0622256	9,16310055	0,20187344	0,66355534	0,84540759	DAD1	defender against cell death 1 [Source:HGNC Symbol;Acc:HGNC:2664]
ENSG00000143294	0,07798772	6,55419157	0,20215733	0,66333577	0,84540759	PRCC	proline rich mitotic checkpoint control factor [Source:HGNC Symbol;Acc:HGNC:9343]
ENSG00000155304	0,09893117	5,77131936	0,2020671	0,66340554	0,84540759	HSPA13	heat shock protein family A (Hsp70) member 13 [Source:HGNC Symbol;Acc:HGNC:11375]
ENSG00000162694	0,08327224	3,77317673	0,20283179	0,66281488	0,84540759	EXTL2	exostosin like glycosyltransferase 2 [Source:HGNC Symbol;Acc:HGNC:3516]
ENSG00000168411	0,0649867	5,21115668	0,20267914	0,66293268	0,84540759	RFWD3	ring finger and WD repeat domain 3 [Source:HGNC Symbol;Acc:HGNC:25539]
ENSG00000170608	-0,0654596	6,57762126	0,20183177	0,66358758	0,84540759	FOXA3	forkhead box A3 [Source:HGNC Symbol;Acc:HGNC:5023]
ENSG00000171552	-0,059185	8,02554813	0,20217461	0,66332241	0,84540759	BCL2L1	BCL2 like 1 [Source:HGNC Symbol;Acc:HGNC:992]
ENSG00000172828	-0,1191357	3,40369489	0,20190309	0,6635324	0,84540759	CES3	carboxylesterase 3 [Source:HGNC Symbol;Acc:HGNC:1865]
ENSG00000173482	-0,1042	3,8985913	0,20223881	0,66327278	0,84540759	PTPRM	protein tyrosine phosphatase receptor type M [Source:HGNC Symbol;Acc:HGNC:9675]
ENSG00000224677	-0,2263564	-0,7980145	0,20203167	0,66343293	0,84540759	PDIA3P2	protein disulfide isomerase family A member 3 pseudogene 2 [Source:HGNC Symbol;Acc:HGNC:49403]
ENSG00000232228	-0,2063077	-1,3764439	0,20236591	0,66317456	0,84540759	RPL36AP16	ribosomal protein L36a pseudogene 16 [Source:HGNC Symbol;Acc:HGNC:36416]
ENSG00000245556	0,10134351	1,35040679	0,20202481	0,66343824	0,84540759	SCAMP1-AS1	SCAMP1 antisense RNA 1 [Source:HGNC Symbol;Acc:HGNC:50544]
ENSG00000265817	-0,1697713	-0,798546	0,20176249	0,66364119	0,84540759	FSBP	fibrinogen silencer binding protein [Source:HGNC Symbol;Acc:HGNC:43653]
ENSG00000287591	-0,2752115	-1,5284606	0,20286619	0,66278834	0,84540759	AL360169.3	novel transcript
ENSG00000187889	-0,2143444	-0,3054187	0,20158653	0,66377743	0,84550741	FYB2	FYN binding protein 2 [Source:HGNC Symbol;Acc:HGNC:27295]
ENSG00000158483	-0,0803084	2,42651293	0,20139855	0,66392305	0,84561916	FAM86C1P	family with sequence similarity 86 member C1, pseudogene [Source:HGNC Symbol;Acc:HGNC:25561]
ENSG00000138308	0,07222921	4,9098799	0,20126232	0,66402862	0,8456669	PLA2G12B	phospholipase A2 group XIIB [Source:HGNC Symbol;Acc:HGNC:18555]
ENSG00000198934	0,14825606	1,52012741	0,20120082	0,6640763	0,8456669	MAGEE1	MAGE family member E1 [Source:HGNC Symbol;Acc:HGNC:24934]
ENSG00000251359	0,22144615	-0,9475654	0,20099274	0,66423769	0,84579869	WWC2-AS2	WWC2 antisense RNA 2 [Source:HGNC Symbol;Acc:HGNC:26390]
ENSG00000275700	-0,0890656	6,32941448	0,20081673	0,66437427	0,84589888	AATF	apoptosis antagonizing transcription factor [Source:HGNC Symbol;Acc:HGNC:19235]
ENSG00000142534	-0,0644248	10,5084065	0,20072558	0,66444504	0,84591526	RPS11	ribosomal protein S11 [Source:HGNC Symbol;Acc:HGNC:10384]
ENSG00000133247	0,09723098	2,12082388	0,20039979	0,66469812	0,84597296	KMT5C	lysine methyltransferase 5C [Source:HGNC Symbol;Acc:HGNC:28405]
ENSG00000173456	-0,0679773	6,74338096	0,20036908	0,66472199	0,84597296	RNF26	ring finger protein 26 [Source:HGNC Symbol;Acc:HGNC:14646]
ENSG00000197345	-0,0597524	6,32652606	0,20050874	0,66461346	0,84597296	MRPL21	mitochondrial ribosomal protein L21 [Source:HGNC Symbol;Acc:HGNC:14479]
ENSG00000212664	0,11058698	0,69442786	0,20053062	0,66459646	0,84597296	AC064799.1	ribosomal protein L17 (RPL17) pseudogene
ENSG00000116205	-0,0769314	3,1821797	0,20013633	0,66490297	0,84604997	TCEANC2	transcription elongation factor A N-terminal and central domain containing 2 [Source:HGNC Symbol;Acc:HGNC:26
ENSG00000122042	-0,1002153	1,58280448	0,20006785	0,66495624	0,84604997	UBL3	ubiquitin like 3 [Source:HGNC Symbol;Acc:HGNC:12504]
ENSG00000228288	-0,1634732	0,14210699	0,20021656	0,66484057	0,84604997	PCAT6	prostate cancer associated transcript 6 [Source:HGNC Symbol;Acc:HGNC:43714]
ENSG00000116157	-0,0659496	4,64757897	0,19984202	0,665132	0,84619989	GPX7	glutathione peroxidase 7 [Source:HGNC Symbol;Acc:HGNC:4559]

ENSG00000048544	-0,0591344	5,79446297	0,19968919	0,66525101	0,8462776	MRPS10	mitochondrial ribosomal protein S10 [Source:HGNC Symbol;Acc:HGNC:14502]
ENSG00000181852	0,08697067	4,5331085	0,19940669	0,66547114	0,84648392	RNF41	ring finger protein 41 [Source:HGNC Symbol;Acc:HGNC:18401]
ENSG00000032444	-0,0816988	5,05804413	0,19898069	0,66580345	0,84656181	PNPLA6	patatin like phospholipase domain containing 6 [Source:HGNC Symbol;Acc:HGNC:16268]
ENSG00000099381	0,10123084	4,50339949	0,19915953	0,6656639	0,84656181	SETD1A	SET domain containing 1A, histone lysine methyltransferase [Source:HGNC Symbol;Acc:HGNC:29010]
ENSG00000131669	0,07011731	7,95214914	0,19910227	0,66570857	0,84656181	NINJ1	ninjurin 1 [Source:HGNC Symbol;Acc:HGNC:7824]
ENSG00000136463	0,06379046	6,61337645	0,19904197	0,66575562	0,84656181	TACO1	translational activator of cytochrome c oxidase I [Source:HGNC Symbol;Acc:HGNC:24316]
ENSG00000138400	-0,2036534	-1,1521719	0,19895679	0,66582211	0,84656181	MDH1B	malate dehydrogenase 1B [Source:HGNC Symbol;Acc:HGNC:17836]
ENSG00000148057	-0,1213411	1,00795048	0,19886876	0,66589084	0,84657552	IDNK	IDNK gluconokinase [Source:HGNC Symbol;Acc:HGNC:31367]
ENSG00000167740	0,09297101	3,49030781	0,19858205	0,66611482	0,84678658	CYB5D2	cytochrome b5 domain containing 2 [Source:HGNC Symbol;Acc:HGNC:28471]
ENSG00000131931	-0,0840258	3,07386607	0,19833077	0,66631127	0,84688893	THAP1	THAP domain containing 1 [Source:HGNC Symbol;Acc:HGNC:20856]
ENSG00000156853	-0,0686526	4,29014241	0,19837953	0,66627314	0,84688893	ZNF689	zinc finger protein 689 [Source:HGNC Symbol;Acc:HGNC:25173]
ENSG00000174405	0,12583872	1,41064701	0,1982298	0,66639026	0,84691564	LIG4	DNA ligase 4 [Source:HGNC Symbol;Acc:HGNC:6601]
ENSG00000145901	0,07149527	6,39411599	0,19812905	0,66646909	0,84694156	TNIP1	TNFAIP3 interacting protein 1 [Source:HGNC Symbol;Acc:HGNC:16903]
ENSG00000182022	0,11411312	3,12523806	0,19805558	0,6665266	0,84694156	CHST15	carbohydrate sulfotransferase 15 [Source:HGNC Symbol;Acc:HGNC:18137]
ENSG00000186471	0,24384446	-0,1900565	0,19768757	0,66681484	0,84723413	AKAP14	A-kinase anchoring protein 14 [Source:HGNC Symbol;Acc:HGNC:24061]
ENSG00000106635	0,06536428	5,51065812	0,19727201	0,66714072	0,84756003	BCL7B	BAF chromatin remodeling complex subunit BCL7B [Source:HGNC Symbol;Acc:HGNC:1005]
ENSG00000214578	0,25545049	-1,5108322	0,19721255	0,66718738	0,84756003	HMGN2P15	high mobility group nucleosomal binding domain 2 pseudogene 15 [Source:HGNC Symbol;Acc:HGNC:39020]
ENSG00000007923	-0,0702854	6,01091185	0,19705742	0,66730916	0,84764103	DNAJC11	DnaJ heat shock protein family (Hsp40) member C11 [Source:HGNC Symbol;Acc:HGNC:25570]
ENSG00000167548	0,31734411	4,92630461	0,19688834	0,66744195	0,8476623	KMT2D	lysine methyltransferase 2D [Source:HGNC Symbol;Acc:HGNC:7133]
ENSG00000172889	0,16531909	-0,8045304	0,19693954	0,66740173	0,8476623	EGFL7	EGF like domain multiple 7 [Source:HGNC Symbol;Acc:HGNC:20594]
ENSG00000013392	-0,0829757	3,22687735	0,19619714	0,66798554	0,84785436	RWDD2A	RWD domain containing 2A [Source:HGNC Symbol;Acc:HGNC:21385]
ENSG00000068097	0,07854599	4,41842951	0,19582659	0,66827744	0,84785436	HEATR6	HEAT repeat containing 6 [Source:HGNC Symbol;Acc:HGNC:24076]
ENSG00000101574	0,10268737	2,04066734	0,19617643	0,66800185	0,84785436	METTL4	methyltransferase like 4 [Source:HGNC Symbol;Acc:HGNC:24726]
ENSG00000112685	0,09783157	4,3173182	0,19616178	0,66801339	0,84785436	EXOC2	exocyst complex component 2 [Source:HGNC Symbol;Acc:HGNC:24968]
ENSG00000114686	0,06254206	7,44988291	0,19572657	0,66835629	0,84785436	MRPL3	mitochondrial ribosomal protein L3 [Source:HGNC Symbol;Acc:HGNC:10379]
ENSG00000125652	-0,0695991	4,8640698	0,19584712	0,66826126	0,84785436	ALKBH7	alkB homolog 7 [Source:HGNC Symbol;Acc:HGNC:21306]
ENSG00000140987	0,06888881	3,136386	0,19565513	0,66841262	0,84785436	ZSCAN32	zinc finger and SCAN domain containing 32 [Source:HGNC Symbol;Acc:HGNC:20812]
ENSG00000148384	0,06888245	3,5462687	0,19588965	0,66822775	0,84785436	INPP5E	inositol polyphosphate-5-phosphatase E [Source:HGNC Symbol;Acc:HGNC:21474]
ENSG00000160867	-0,0615518	9,20646902	0,19559034	0,66846372	0,84785436	FGFR4	fibroblast growth factor receptor 4 [Source:HGNC Symbol;Acc:HGNC:3691]
ENSG00000166788	0,0617136	5,34634149	0,19563554	0,66842807	0,84785436	SAAL1	serum amyloid A like 1 [Source:HGNC Symbol;Acc:HGNC:25158]
ENSG00000172375	-0,0795159	4,36440713	0,19564911	0,66841737	0,84785436	C2CD2L	C2CD2 like [Source:HGNC Symbol;Acc:HGNC:29000]
ENSG00000181061	-0,0856985	4,72725253	0,19661604	0,66765597	0,84785436	HIGD1A	HIG1 hypoxia inducible domain family member 1A [Source:HGNC Symbol;Acc:HGNC:29527]
ENSG00000196290	-0,0619129	5,4932551	0,19636802	0,66785105	0,84785436	NIF3L1	NGG1 interacting factor 3 like 1 [Source:HGNC Symbol;Acc:HGNC:13390]
ENSG00000232060	-0,2458853	-1,8750223	0,19602791	0,66811882	0,84785436	SLC4A1APP1	solute carrier family 4 member 1 adaptor protein pseudogene 1 [Source:HGNC Symbol;Acc:HGNC:49811]
ENSG00000269929	0,13102841	0,25518365	0,19581872	0,66828364	0,84785436	MIRLET7A1HG	miRlet-7a-1/let-7f-1/let-7d cluster host gene [Source:HGNC Symbol;Acc:HGNC:53970]
ENSG00000109680	0,12889917	0,23268596	0,1954194	0,66859859	0,8479518	TBC1D19	TBC1 domain family member 19 [Source:HGNC Symbol;Acc:HGNC:25624]
ENSG00000277581	0,24404045	-1,3117166	0,19528139	0,66870752	0,84801634	AL023803.3	novel transcript, sense intronic to DHX35
ENSG00000205542	-0,1827814	-0,6595825	0,19470653	0,6691618	0,84851877	TMSB4X	thymosin beta 4 X-linked [Source:HGNC Symbol;Acc:HGNC:11881]
ENSG00000124613	-0,1712869	-0,3988956	0,19453825	0,66929493	0,84861393	ZNF391	zinc finger protein 391 [Source:HGNC Symbol;Acc:HGNC:18779]
ENSG00000103168	0,06565088	3,935112	0,19428321	0,66949684	0,84868674	TAF1C	TATA-box binding protein associated factor, RNA polymerase I subunit C [Source:HGNC Symbol;Acc:HGNC:11534]
ENSG00000138030	-0,0709216	6,45429549	0,19423101	0,66953818	0,84868674	KHK	ketohexokinase [Source:HGNC Symbol;Acc:HGNC:6315]
ENSG00000160753	0,06612706	5,09409463	0,19416525	0,66959028	0,84868674	RUSC1	RUN and SH3 domain containing 1 [Source:HGNC Symbol;Acc:HGNC:17153]
ENSG00000213699	-0,0869023	5,67288899	0,19431455	0,66947202	0,84868674	SLC35F6	solute carrier family 35 member F6 [Source:HGNC Symbol;Acc:HGNC:26055]
ENSG00000230793	0,27635074	-1,859191	0,19402563	0,66970092	0,84868674	SMARCE1P5	SWI/SNF related, matrix associated, actin dependent regulator of chromatin, subfamily e, member 1 pseudogene

ENSG00000232995	-0,2951135	-1,6952721	0,19404432	0,6696861	0,84868674	RGS5	regulator of G protein signaling 5 [Source:NCBI gene (formerly Entrezgene);Acc:8490]
ENSG00000167657	0,08320912	5,52609079	0,19376836	0,66990491	0,8487036	DAPK3	death associated protein kinase 3 [Source:HGNC Symbol;Acc:HGNC:2676]
ENSG00000167741	-0,1666687	-0,5105583	0,1936468	0,67000136	0,8487036	GGT6	gamma-glutamyltransferase 6 [Source:HGNC Symbol;Acc:HGNC:26891]
ENSG00000168028	-0,0650122	10,5027785	0,19368646	0,66996989	0,8487036	RPSA	ribosomal protein SA [Source:HGNC Symbol;Acc:HGNC:6502]
ENSG00000228818	-0,1576665	-0,3417642	0,1936426	0,6700047	0,8487036	AL359918.1	proteasome 26S non-ATPase subunit 2 (PSMD2) pseudogene
ENSG00000242498	0,09645865	1,77162897	0,19368249	0,66997304	0,8487036	ARPIN	actin related protein 2/3 complex inhibitor [Source:HGNC Symbol;Acc:HGNC:28782]
ENSG00000164086	0,08598042	3,29240943	0,19343637	0,67016841	0,84883737	DUSP7	dual specificity phosphatase 7 [Source:HGNC Symbol;Acc:HGNC:3073]
ENSG00000235554	-0,1578034	-0,5831259	0,19336097	0,67022828	0,84883962	SRSF6P2	SRSF6 pseudogene 2 [Source:HGNC Symbol;Acc:HGNC:54947]
ENSG00000156170	-0,0743198	4,0454525	0,19310844	0,67042893	0,84902014	NDUFAF6	NADH:ubiquinone oxidoreductase complex assembly factor 6 [Source:HGNC Symbol;Acc:HGNC:28625]
ENSG00000172239	-0,0583929	6,52024211	0,19295864	0,67054804	0,84909736	PAIP1	poly(A) binding protein interacting protein 1 [Source:HGNC Symbol;Acc:HGNC:16945]
ENSG00000105819	-0,072676	6,41073595	0,19219641	0,67115494	0,84942907	PMPCB	peptidase, mitochondrial processing subunit beta [Source:HGNC Symbol;Acc:HGNC:9119]
ENSG00000128915	-0,0707531	3,8942694	0,19219149	0,67115886	0,84942907	ICE2	interactor of little elongation complex ELL subunit 2 [Source:HGNC Symbol;Acc:HGNC:29885]
ENSG00000154240	0,11231397	0,84063209	0,19243923	0,67096145	0,84942907	CEP112	centrosomal protein 112 [Source:HGNC Symbol;Acc:HGNC:28514]
ENSG00000170417	-0,1004383	2,00171044	0,19220143	0,67115094	0,84942907	TMEM182	transmembrane protein 182 [Source:HGNC Symbol;Acc:HGNC:26391]
ENSG00000171227	-0,0873437	5,74361495	0,19250107	0,67091219	0,84942907	TMEM37	transmembrane protein 37 [Source:HGNC Symbol;Acc:HGNC:18216]
ENSG00000274220	-0,2245258	-1,6492528	0,19234602	0,6710357	0,84942907	AC009163.7	novel transcript
ENSG00000108654	-0,0808741	8,86218671	0,19206749	0,67125773	0,84948061	DDX5	DEAD-box helicase 5 [Source:HGNC Symbol;Acc:HGNC:2746]
ENSG00000278834	0,09621458	1,23836023	0,1919235	0,67137259	0,84955237	AC073508.3	novel transcript, antisense to SMARCE1
ENSG00000116675	-0,1437567	0,32362223	0,19167468	0,67157119	0,84957419	DNAJC6	DnaJ heat shock protein family (Hsp40) member C6 [Source:HGNC Symbol;Acc:HGNC:15469]
ENSG00000127084	-0,2195477	-1,1369548	0,19152042	0,67169439	0,84957419	FGD3	FYVE, RhoGEF and PH domain containing 3 [Source:HGNC Symbol;Acc:HGNC:16027]
ENSG00000130779	-0,1615746	4,36344474	0,19146784	0,6717364	0,84957419	CLIP1	CAP-Gly domain containing linker protein 1 [Source:HGNC Symbol;Acc:HGNC:10461]
ENSG00000133818	-0,0645208	4,268223	0,19132563	0,67185005	0,84957419	RRAS2	RAS related 2 [Source:HGNC Symbol;Acc:HGNC:17271]
ENSG00000141956	0,10380765	2,53351518	0,19131935	0,67185507	0,84957419	PRDM15	PR/SET domain 15 [Source:HGNC Symbol;Acc:HGNC:13999]
ENSG00000151458	0,12187747	1,56446857	0,19175698	0,67150548	0,84957419	ANKRD50	ankyrin repeat domain 50 [Source:HGNC Symbol;Acc:HGNC:29223]
ENSG00000182993	0,17742681	0,19293385	0,19150732	0,67170486	0,84957419	C12orf60	chromosome 12 open reading frame 60 [Source:HGNC Symbol;Acc:HGNC:28726]
ENSG00000275774	0,1611269	-0,8024878	0,19137478	0,67181076	0,84957419	HNRNPCL2	heterogeneous nuclear ribonucleoprotein C like 2 [Source:HGNC Symbol;Acc:HGNC:48813]
ENSG00000146648	0,14790414	4,32140393	0,19085432	0,67222708	0,84997104	EGFR	epidermal growth factor receptor [Source:HGNC Symbol;Acc:HGNC:3236]
ENSG00000139324	0,07561621	2,85416738	0,19077104	0,67229376	0,84998178	TMTC3	transmembrane O-mannosyltransferase targeting cadherins 3 [Source:HGNC Symbol;Acc:HGNC:26899]
ENSG00000051180	0,0929034	4,62043872	0,19047652	0,67252971	0,85004562	RAD51	RAD51 recombinase [Source:HGNC Symbol;Acc:HGNC:9817]
ENSG00000115084	-0,0710084	6,19025085	0,19012521	0,67281144	0,85004562	SLC35F5	solute carrier family 35 member F5 [Source:HGNC Symbol;Acc:HGNC:23617]
ENSG00000162825	-0,1854963	0,07796201	0,19061622	0,67241776	0,85004562	NBPF20	NBPF member 20 [Source:HGNC Symbol;Acc:HGNC:32000]
ENSG00000166589	0,13650451	0,63873702	0,19006844	0,672857	0,85004562	CDH16	cadherin 16 [Source:HGNC Symbol;Acc:HGNC:1755]
ENSG00000168078	0,06655432	5,32687882	0,19019132	0,67275839	0,85004562	PBK	PDZ binding kinase [Source:HGNC Symbol;Acc:HGNC:18282]
ENSG00000187815	-0,0878751	2,28701806	0,19038662	0,67260177	0,85004562	ZFP69	ZFP69 zinc finger protein [Source:HGNC Symbol;Acc:HGNC:24708]
ENSG00000251381	-0,1952572	-0,2917343	0,19005482	0,67286792	0,85004562	LINC00958	long intergenic non-protein coding RNA 958 [Source:HGNC Symbol;Acc:HGNC:48671]
ENSG00000266208	0,07480086	2,37458985	0,19025089	0,67271061	0,85004562	AC080112.1	novel transcript, antisense to GJD3
ENSG00000269399	-0,1856928	-0,8091176	0,19055906	0,67246355	0,85004562	AC008764.6	novel transcript
ENSG00000108773	0,05882586	6,43201743	0,1891648	0,67358325	0,85038739	KAT2A	lysine acetyltransferase 2A [Source:HGNC Symbol;Acc:HGNC:4201]
ENSG00000171121	-0,1147302	1,15134444	0,18923035	0,67353049	0,85038739	KCNMB3	potassium calcium-activated channel subfamily M regulatory beta subunit 3 [Source:HGNC Symbol;Acc:HGNC:62
ENSG00000213906	0,2091528	0,214271	0,18927042	0,67349826	0,85038739	LTB4R2	leukotriene B4 receptor 2 [Source:HGNC Symbol;Acc:HGNC:19260]
ENSG00000227681	0,08813301	1,50055235	0,18928217	0,6734888	0,85038739	AL033504.1	novel transcript
ENSG00000233971	-0,1353492	0,05862416	0,18932227	0,67345654	0,85038739	RPS20P10	ribosomal protein S20 pseudogene 10 [Source:HGNC Symbol;Acc:HGNC:36800]
ENSG00000237238	-0,2283351	-1,735185	0,18913884	0,67360414	0,85038739	BMS1P10	BMS1 pseudogene 10 [Source:HGNC Symbol;Acc:HGNC:49154]
ENSG00000243818	0,22190989	-0,5440442	0,18923111	0,67352988	0,85038739	AC021074.3	novel transcript

ENSG00000267809	-0,0955981	1,29644691	0,1893532	0,67343166	0,85038739	NDUFV2P1	NADH:ubiquinone oxidoreductase core subunit V2 pseudogene 1 [Source:HGNC Symbol;Acc:HGNC:7718]
ENSG00000204463	0,10015598	8,34167182	0,18899333	0,67372129	0,8504618	BAG6	BAG cochaperone 6 [Source:HGNC Symbol;Acc:HGNC:13919]
ENSG00000115257	-0,1489557	0,12642336	0,1888413	0,67384376	0,8504977	PCSK4	proprotein convertase subtilisin/kexin type 4 [Source:HGNC Symbol;Acc:HGNC:8746]
ENSG00000254285	0,11889783	4,20551962	0,18881348	0,67386617	0,8504977	KRT8P3	keratin 8 pseudogene 3 [Source:HGNC Symbol;Acc:HGNC:31056]
ENSG00000227627	-0,323197	-1,4075171	0,18833353	0,67425319	0,85091265	AL080276.2	novel transcript
ENSG00000066135	0,08558731	3,88236194	0,18812645	0,67442035	0,85104502	KDM4A	lysine demethylase 4A [Source:HGNC Symbol;Acc:HGNC:22978]
ENSG00000163466	-0,0580629	8,17216444	0,18790663	0,67459793	0,85104502	ARPC2	actin related protein 2/3 complex subunit 2 [Source:HGNC Symbol;Acc:HGNC:705]
ENSG00000204348	-0,0765676	4,2721565	0,18789219	0,6746096	0,85104502	DXO	decapping exoribonuclease [Source:HGNC Symbol;Acc:HGNC:2992]
ENSG00000211454	-0,2047601	-1,2343475	0,18804955	0,67448246	0,85104502	AKR7L	aldo-keto reductase family 7 like (gene/pseudogene) [Source:HGNC Symbol;Acc:HGNC:24056]
ENSG00000224870	-0,0830033	4,20465782	0,187843	0,67464935	0,85104502	MRPL20-AS1	MRPL20 antisense RNA 1 [Source:HGNC Symbol;Acc:HGNC:27125]
ENSG00000130702	-0,1566742	6,37523188	0,18771041	0,67475654	0,85110675	LAMA5	laminin subunit alpha 5 [Source:HGNC Symbol;Acc:HGNC:6485]
ENSG00000132964	0,07469515	3,38167305	0,18730606	0,67508372	0,85144591	CDK8	cyclin dependent kinase 8 [Source:HGNC Symbol;Acc:HGNC:1779]
ENSG00000130175	-0,1130605	9,1858606	0,1870081	0,67532507	0,8516768	PRKCSH	protein kinase C substrate 80K-H [Source:HGNC Symbol;Acc:HGNC:9411]
ENSG00000129625	-0,0590491	7,96774217	0,1865409	0,67570399	0,85193403	REEP5	receptor accessory protein 5 [Source:HGNC Symbol;Acc:HGNC:30077]
ENSG00000157470	-0,2678472	-1,4514539	0,1866607	0,67560678	0,85193403	FAM81A	family with sequence similarity 81 member A [Source:HGNC Symbol;Acc:HGNC:28379]
ENSG00000168884	-0,0586436	5,50662811	0,18657596	0,67567554	0,85193403	TNIP2	TNFAIP3 interacting protein 2 [Source:HGNC Symbol;Acc:HGNC:19118]
ENSG00000025772	0,05856615	7,44172534	0,18627201	0,67592233	0,85212848	TOMM34	translocase of outer mitochondrial membrane 34 [Source:HGNC Symbol;Acc:HGNC:15746]
ENSG00000133422	0,09063662	5,57604431	0,18620733	0,67597488	0,85212848	MORC2	MORC family CW-type zinc finger 2 [Source:HGNC Symbol;Acc:HGNC:23573]
ENSG00000118900	0,1178945	4,84758442	0,18576954	0,67633085	0,85225336	UBN1	ubinuclein 1 [Source:HGNC Symbol;Acc:HGNC:12506]
ENSG00000132286	-0,0875918	3,89887175	0,18565512	0,67642397	0,85225336	TIMM10B	translocase of inner mitochondrial membrane 10B [Source:HGNC Symbol;Acc:HGNC:4022]
ENSG00000165376	-0,1120987	2,58484088	0,1857818	0,67632088	0,85225336	CLDN2	claudin 2 [Source:HGNC Symbol;Acc:HGNC:2041]
ENSG00000167721	-0,078488	7,28198584	0,1859905	0,67615113	0,85225336	TSR1	TSR1 ribosome maturation factor [Source:HGNC Symbol;Acc:HGNC:25542]
ENSG00000197081	0,16662458	7,84771364	0,18574212	0,67635316	0,85225336	IGF2R	insulin like growth factor 2 receptor [Source:HGNC Symbol;Acc:HGNC:5467]
ENSG00000254682	-0,1049403	1,46243583	0,18569135	0,67639447	0,85225336	AP002387.1	novel transcript
ENSG00000120925	0,07365178	3,81356452	0,18551884	0,67653492	0,85231964	RNF170	ring finger protein 170 [Source:HGNC Symbol;Acc:HGNC:25358]
ENSG00000089685	-0,0856893	6,79806612	0,18515296	0,67683304	0,85254819	BIRC5	baculoviral IAP repeat containing 5 [Source:HGNC Symbol;Acc:HGNC:593]
ENSG00000123200	0,15924744	5,22073627	0,18516381	0,6768242	0,85254819	ZC3H13	zinc finger CCCH-type containing 13 [Source:HGNC Symbol;Acc:HGNC:20368]
ENSG00000158008	-0,3500962	-1,9545819	0,18476114	0,6771527	0,8528773	EXTL1	exostosin like glycosyltransferase 1 [Source:HGNC Symbol;Acc:HGNC:3515]
ENSG00000004478	0,06459105	7,89482544	0,18431936	0,6775136	0,85303768	FKBP4	FKBP prolyl isomerase 4 [Source:HGNC Symbol;Acc:HGNC:3720]
ENSG00000135736	0,12808668	0,62441252	0,18438817	0,67745735	0,85303768	CCDC102A	coiled-coil domain containing 102A [Source:HGNC Symbol;Acc:HGNC:28097]
ENSG00000172667	0,09534608	1,88475615	0,18435756	0,67748238	0,85303768	ZMAT3	zinc finger matrin-type 3 [Source:HGNC Symbol;Acc:HGNC:29983]
ENSG00000279529	0,16236177	-0,6979461	0,18440679	0,67744214	0,85303768	AC008764.8	novel transcript, antisense to MED26
ENSG00000205981	0,07515639	4,72805605	0,18406386	0,67772256	0,85322724	DNAJC19	DnaJ heat shock protein family (Hsp40) member C19 [Source:HGNC Symbol;Acc:HGNC:30528]
ENSG00000075413	0,05999691	5,69774617	0,18369447	0,67802497	0,85335413	MARK3	microtubule affinity regulating kinase 3 [Source:HGNC Symbol;Acc:HGNC:6897]
ENSG00000185475	0,06088337	5,74356354	0,18378705	0,67794915	0,85335413	TMEM179B	transmembrane protein 179B [Source:HGNC Symbol;Acc:HGNC:33744]
ENSG00000222001	0,10245205	0,89050417	0,18375152	0,67797825	0,85335413	AC106876.1	novel transcript, antisense to NGEF
ENSG00000269951	0,22321443	-1,4989516	0,18365538	0,678057	0,85335413	AC090181.2	novel transcript, sense intronic to TSPAN3
ENSG00000090020	-0,0688658	3,46420373	0,1833583	0,67830052	0,85336393	SLC9A1	solute carrier family 9 member A1 [Source:HGNC Symbol;Acc:HGNC:11071]
ENSG00000112511	0,06727957	4,69590166	0,18328246	0,67836271	0,85336393	PHF1	PHD finger protein 1 [Source:HGNC Symbol;Acc:HGNC:8919]
ENSG00000129968	-0,0807427	4,31078902	0,18347691	0,67820326	0,85336393	ABHD17A	abhydrolase domain containing 17A, depalmitoylase [Source:HGNC Symbol;Acc:HGNC:28756]
ENSG00000133740	-0,086414	3,75711307	0,18330489	0,67834431	0,85336393	E2F5	E2F transcription factor 5 [Source:HGNC Symbol;Acc:HGNC:3119]
ENSG00000162972	-0,0592135	5,8392697	0,1832281	0,67840731	0,85336393	MAIP1	matrix AAA peptidase interacting protein 1 [Source:HGNC Symbol;Acc:HGNC:26198]
ENSG00000276077	-0,268722	-1,5467409	0,18314721	0,67847368	0,85336393	CU633904.1	uncharacterized LOC102724951 [Source:NCBI gene (formerly Entrezgene);Acc:102724951]
ENSG00000277067	-0,268722	-1,5467409	0,18314721	0,67847368	0,85336393	CU634019.1	uncharacterized LOC102724843 [Source:NCBI gene (formerly Entrezgene);Acc:102724843]

ENSG00000111581	0,06680253	5,20939884	0,18266401	0,67887053	0,85349566	NUP107	nucleoporin 107 [Source:HGNC Symbol;Acc:HGNC:29914]
ENSG00000155085	0,19513875	-0,1460433	0,18284446	0,67872226	0,85349566	AK9	adenylate kinase 9 [Source:HGNC Symbol;Acc:HGNC:33814]
ENSG00000223459	0,24866223	0,8289063	0,18276165	0,67879029	0,85349566	TCAF1P1	TRPM8 channel associated factor 1 pseudogene 1 [Source:HGNC Symbol;Acc:HGNC:33604]
ENSG00000246273	-0,0982159	1,49968754	0,18268025	0,67885719	0,85349566	SBF2-AS1	SBF2 antisense RNA 1 [Source:HGNC Symbol;Acc:HGNC:27438]
ENSG00000267519	0,28983267	-1,1310003	0,18287722	0,67869535	0,85349566	AC020916.1	novel transcript
ENSG00000163938	-0,0585971	7,68440669	0,18257219	0,67894601	0,85351711	GNL3	G protein nucleolar 3 [Source:HGNC Symbol;Acc:HGNC:29931]
ENSG00000147421	-0,09526	2,35967502	0,18238374	0,67910101	0,8536385	HMBOX1	homeobox containing 1 [Source:HGNC Symbol;Acc:HGNC:26137]
ENSG00000164574	-0,079942	4,24891361	0,18211556	0,67932174	0,85365907	GALNT10	polypeptide N-acetylgalactosaminyltransferase 10 [Source:HGNC Symbol;Acc:HGNC:19873]
ENSG00000166949	-0,0643117	5,01937855	0,18210164	0,6793332	0,85365907	SMAD3	SMAD family member 3 [Source:HGNC Symbol;Acc:HGNC:6769]
ENSG00000241511	0,15685887	-0,6775786	0,18211789	0,67931982	0,85365907	RPS15AP24	ribosomal protein S15a pseudogene 24 [Source:HGNC Symbol;Acc:HGNC:36174]
ENSG00000257303	-0,1636721	-0,2196386	0,1820799	0,67935111	0,85365907	AC073896.2	novel transcript, antisense to CNPY2 and CS
ENSG00000167378	0,16384637	3,9631888	0,18191519	0,67948679	0,8536827	IRGQ	immunity related GTPase Q [Source:HGNC Symbol;Acc:HGNC:24868]
ENSG00000180747	-0,1337865	1,14586198	0,18191945	0,67948327	0,8536827	SMG1P3	SMG1 pseudogene 3 [Source:HGNC Symbol;Acc:HGNC:49860]
ENSG00000062582	-0,2242929	-1,5380568	0,1816961	0,67966738	0,85368935	MRPS24	mitochondrial ribosomal protein S24 [Source:HGNC Symbol;Acc:HGNC:14510]
ENSG00000113719	-0,0593129	6,99303623	0,1818313	0,67955591	0,85368935	ERGIC1	endoplasmic reticulum-golgi intermediate compartment 1 [Source:HGNC Symbol;Acc:HGNC:29205]
ENSG00000272173	-0,2396934	-1,9169233	0,18169608	0,67966739	0,85368935	U47924.2	novel transcript, antisense to C12orf57
ENSG00000165775	-0,0693618	6,184367	0,18152003	0,67981259	0,85379833	FUNDC2	FUN14 domain containing 2 [Source:HGNC Symbol;Acc:HGNC:24925]
ENSG00000086102	-0,0650158	5,32910375	0,18142742	0,67988902	0,85382091	NFX1	nuclear transcription factor, X-box binding 1 [Source:HGNC Symbol;Acc:HGNC:7803]
ENSG00000187953	-0,0771987	2,29476973	0,18132709	0,67997183	0,85385151	PMS2CL	PMS2 C-terminal like pseudogene [Source:HGNC Symbol;Acc:HGNC:30061]
ENSG00000123992	-0,0552629	6,43734154	0,18111927	0,68014346	0,85392023	DNPEP	aspartyl aminopeptidase [Source:HGNC Symbol;Acc:HGNC:2981]
ENSG00000138363	0,08150742	8,71231079	0,18113161	0,68013327	0,85392023	ATIC	5-aminoimidazole-4-carboxamide ribonucleotide formyltransferase/IMP cyclohydrolase [Source:HGNC Symbol;Ac
ENSG00000178934	-0,306479	-1,4060053	0,18091137	0,68031528	0,85406255	LGALS7B	galectin 7B [Source:HGNC Symbol;Acc:HGNC:34447]
ENSG00000179967	-0,1269897	0,07744616	0,18042908	0,6807143	0,85448426	PPP1R14BP3	protein phosphatase 1 regulatory inhibitor subunit 14B pseudogene 3 [Source:HGNC Symbol;Acc:HGNC:16330]
ENSG00000183020	0,06235676	6,33133642	0,18036402	0,68076818	0,85448426	AP2A2	adaptor related protein complex 2 subunit alpha 2 [Source:HGNC Symbol;Acc:HGNC:562]
ENSG00000109265	0,16468399	-0,1140149	0,1802469	0,6808652	0,85453262	CRACD	capping protein inhibiting regulator of actin dynamics [Source:HGNC Symbol;Acc:HGNC:29219]
ENSG00000150636	-0,1508204	0,24840782	0,18009078	0,68099458	0,85462159	CCDC102B	coiled-coil domain containing 102B [Source:HGNC Symbol;Acc:HGNC:26295]
ENSG00000258427	-0,1816483	-0,3001125	0,18000206	0,68106814	0,85464048	RBM8B	RNA binding motif protein 8B pseudogene [Source:HGNC Symbol;Acc:HGNC:13426]
ENSG00000060762	-0,0626925	7,07679315	0,17985438	0,68119062	0,85472076	MPC1	mitochondrial pyruvate carrier 1 [Source:HGNC Symbol;Acc:HGNC:21606]
ENSG00000126214	-0,0757195	2,9185217	0,17971973	0,68130236	0,85472437	KLC1	kinesin light chain 1 [Source:HGNC Symbol;Acc:HGNC:6387]
ENSG00000136485	-0,0680635	6,5267471	0,17970991	0,68131051	0,85472437	DCAF7	DDB1 and CUL4 associated factor 7 [Source:HGNC Symbol;Acc:HGNC:30915]
ENSG00000228121	-0,1382599	-0,1507188	0,17944275	0,68153235	0,85492925	PHBP3	prohibitin pseudogene 3 [Source:HGNC Symbol;Acc:HGNC:8915]
ENSG00000113889	0,08948426	1,48804893	0,17921535	0,68172133	0,8550929	KNG1	kininogen 1 [Source:HGNC Symbol;Acc:HGNC:6383]
ENSG00000066322	-0,0605135	6,88422711	0,17905924	0,68185115	0,85512257	ELOVL1	ELOVL fatty acid elongase 1 [Source:HGNC Symbol;Acc:HGNC:14418]
ENSG00000102471	-0,0826628	4,40653311	0,17904613	0,68186206	0,85512257	NDFIP2	Nedd4 family interacting protein 2 [Source:HGNC Symbol;Acc:HGNC:18537]
ENSG00000134864	0,1743951	-1,0808349	0,17889661	0,68198647	0,85513177	GGACT	gamma-glutamylamine cyclotransferase [Source:HGNC Symbol;Acc:HGNC:25100]
ENSG00000152464	-0,0706634	4,23837331	0,17894384	0,68194716	0,85513177	RPP38	ribonuclease P/MRP subunit p38 [Source:HGNC Symbol;Acc:HGNC:30329]
ENSG00000169410	0,06382645	4,3670556	0,17863107	0,68220757	0,85529584	PTPN9	protein tyrosine phosphatase non-receptor type 9 [Source:HGNC Symbol;Acc:HGNC:9661]
ENSG00000198598	-0,0945898	1,05387634	0,17859885	0,6822344	0,85529584	MMP17	matrix metallopeptidase 17 [Source:HGNC Symbol;Acc:HGNC:7163]
ENSG00000132341	0,0709669	9,7192777	0,17846552	0,68234551	0,85534166	RAN	RAN, member RAS oncogene family [Source:HGNC Symbol;Acc:HGNC:9846]
ENSG00000223959	-0,0714431	4,09327609	0,17841448	0,68238805	0,85534166	AFG3L1P	AFG3 like matrix AAA peptidase subunit 1, pseudogene [Source:HGNC Symbol;Acc:HGNC:314]
ENSG00000168282	-0,0672028	5,89033863	0,1782624	0,68251486	0,85542721	MGAT2	alpha-1,6-mannosyl-glycoprotein 2-beta-N-acetylglucosaminyltransferase [Source:HGNC Symbol;Acc:HGNC:7045]
ENSG00000103769	0,06215406	6,67242769	0,17812232	0,68263172	0,85549354	RAB11A	RAB11A, member RAS oncogene family [Source:HGNC Symbol;Acc:HGNC:9760]
ENSG00000158315	0,09921174	3,37870058	0,17805859	0,6826849	0,85549354	RHBDL2	rhomboid like 2 [Source:HGNC Symbol;Acc:HGNC:16083]
ENSG00000053747	0,15034883	-0,2435852	0,17767082	0,68300874	0,85567916	LAMA3	laminin subunit alpha 3 [Source:HGNC Symbol;Acc:HGNC:6483]

ENSG00000126432	-0,0562008	8,18562074	0,17767971	0,68300131	0,85567916	PRDX5	peroxiredoxin 5 [Source:HGNC Symbol;Acc:HGNC:9355]
ENSG00000197323	0,12103544	4,34044048	0,17770417	0,68298087	0,85567916	TRIM33	tripartite motif containing 33 [Source:HGNC Symbol;Acc:HGNC:16290]
ENSG00000243988	0,16717877	-0,8691363	0,17755104	0,68310885	0,85570559	RPS24P17	ribosomal protein S24 pseudogene 17 [Source:HGNC Symbol;Acc:HGNC:36348]
ENSG00000288675	0,22336206	-1,6126594	0,17750544	0,68314698	0,85570559	AP006621.6	Proapoptotic nucleolar protein 1 [Source:UniProtKB/Swiss-Prot;Acc:I0J062]
ENSG00000204434	-0,1769988	-1,1326193	0,17722812	0,68337897	0,85592279	POTEKP	POTE ankyrin domain family member K, pseudogene [Source:HGNC Symbol;Acc:HGNC:30182]
ENSG00000085415	0,0577145	5,88729855	0,17686654	0,68368177	0,85594282	SEH1L	SEH1 like nucleoporin [Source:HGNC Symbol;Acc:HGNC:30379]
ENSG00000100575	-0,0753586	5,07262906	0,17679719	0,68373989	0,85594282	TIMM9	translocase of inner mitochondrial membrane 9 [Source:HGNC Symbol;Acc:HGNC:11819]
ENSG00000184574	0,30476971	-2,0627825	0,17711194	0,68347622	0,85594282	LPAR5	lysophosphatidic acid receptor 5 [Source:HGNC Symbol;Acc:HGNC:13307]
ENSG00000250899	-0,1571111	1,32741599	0,17692141	0,6836358	0,85594282	AC125807.2	novel transcript
ENSG00000263781	-0,1519669	-0,6027402	0,1767893	0,68374651	0,85594282	AC024619.4	NADH dehydrogenase (ubiquinone) Fe-S protein 5, 15kDa (NADH-coenzyme Q reductase) (NDUFS5) pseudogene
ENSG00000278600	0,15134368	0,2961743	0,17683848	0,68370528	0,85594282	AC015871.3	novel transcript, sense intronic to ST20
ENSG00000112365	0,07134564	2,568142	0,17637729	0,68409208	0,85630205	ZBTB24	zinc finger and BTB domain containing 24 [Source:HGNC Symbol;Acc:HGNC:21143]
ENSG00000134698	0,08664537	1,80631101	0,1762759	0,68417719	0,85633521	AGO4	argonaute RISC component 4 [Source:HGNC Symbol;Acc:HGNC:18424]
ENSG00000111843	-0,0672433	7,26135382	0,1759977	0,68441088	0,85648094	TMEM14C	transmembrane protein 14C [Source:HGNC Symbol;Acc:HGNC:20952]
ENSG00000115977	0,21577909	3,46899402	0,17605957	0,68435889	0,85648094	AAK1	AP2 associated kinase 1 [Source:HGNC Symbol;Acc:HGNC:19679]
ENSG00000144488	0,16453265	-0,31298	0,17559358	0,68475074	0,8566861	ESPNL	espin like [Source:HGNC Symbol;Acc:HGNC:27937]
ENSG00000145241	0,06375917	3,10664027	0,17567269	0,68468417	0,8566861	CENPC	centromere protein C [Source:HGNC Symbol;Acc:HGNC:1854]
ENSG00000164885	-0,0578753	5,32731294	0,17565713	0,68469727	0,8566861	CDK5	cyclin dependent kinase 5 [Source:HGNC Symbol;Acc:HGNC:1774]
ENSG00000242338	0,25140624	-1,9072317	0,17495102	0,68529208	0,85721655	BMS1P4	BMS1 pseudogene 4 [Source:HGNC Symbol;Acc:HGNC:23652]
ENSG00000251685	-0,0745125	1,98879974	0,1749552	0,68528856	0,85721655	UGT2B27P	UDP glucuronosyltransferase family 2 member B27, pseudogene [Source:HGNC Symbol;Acc:HGNC:12551]
ENSG00000111667	-0,1078876	6,39516296	0,17445718	0,68570894	0,85766455	USP5	ubiquitin specific peptidase 5 [Source:HGNC Symbol;Acc:HGNC:12628]
ENSG00000105483	0,07828542	2,28107574	0,17409829	0,68601232	0,85782367	CARD8	caspase recruitment domain family member 8 [Source:HGNC Symbol;Acc:HGNC:17057]
ENSG00000108622	-0,0764727	4,23048391	0,17423266	0,68589869	0,85782367	ICAM2	intercellular adhesion molecule 2 [Source:HGNC Symbol;Acc:HGNC:5345]
ENSG00000228347	0,17830819	-0,6985928	0,1741456	0,6859723	0,85782367	FTLP17	ferritin light chain pseudogene 17 [Source:HGNC Symbol;Acc:HGNC:37967]
ENSG00000154545	0,15506889	-0,3001814	0,17359387	0,68643936	0,85813731	MAGED4	MAGE family member D4 [Source:HGNC Symbol;Acc:HGNC:23793]
ENSG00000188352	0,08884211	4,70893167	0,17362883	0,68640973	0,85813731	FOCAD	focadhesin [Source:HGNC Symbol;Acc:HGNC:23377]
ENSG00000196247	0,10334464	1,33180381	0,17361288	0,68642325	0,85813731	ZNF107	zinc finger protein 107 [Source:HGNC Symbol;Acc:HGNC:12887]
ENSG00000159086	0,06982976	3,82881532	0,17304674	0,68690338	0,85864392	PAXBP1	PAX3 and PAX7 binding protein 1 [Source:HGNC Symbol;Acc:HGNC:13579]
ENSG00000105875	0,08269345	3,68507522	0,1728015	0,68711166	0,85868876	WDR91	WD repeat domain 91 [Source:HGNC Symbol;Acc:HGNC:24997]
ENSG00000118369	0,08153832	2,79101721	0,17279687	0,68711559	0,85868876	USP35	ubiquitin specific peptidase 35 [Source:HGNC Symbol;Acc:HGNC:20061]
ENSG00000197579	-0,0615689	4,08003382	0,1729066	0,68702237	0,85868876	TOPORS	TOP1 binding arginine/serine rich protein, E3 ubiquitin ligase [Source:HGNC Symbol;Acc:HGNC:21653]
ENSG00000149150	-0,0569781	7,35255012	0,17257045	0,68730804	0,85875821	SLC43A1	solute carrier family 43 member 1 [Source:HGNC Symbol;Acc:HGNC:9225]
ENSG00000167004	-0,0911496	10,5613477	0,17251342	0,68735654	0,85875821	PDIA3	protein disulfide isomerase family A member 3 [Source:HGNC Symbol;Acc:HGNC:4606]
ENSG00000178631	0,22388316	-1,6639854	0,17238582	0,68746508	0,85875821	ACTG1P1	actin gamma 1 pseudogene 1 [Source:HGNC Symbol;Acc:HGNC:146]
ENSG00000185670	-0,0842058	1,78618359	0,17247513	0,6873891	0,85875821	ZBTB3	zinc finger and BTB domain containing 3 [Source:HGNC Symbol;Acc:HGNC:22918]
ENSG00000204576	-0,0616427	3,92433974	0,17238881	0,68746253	0,85875821	PRR3	proline rich 3 [Source:HGNC Symbol;Acc:HGNC:21149]
ENSG00000006756	-0,11465	2,46152458	0,17223061	0,68759718	0,85884979	ARSD	arylsulfatase D [Source:HGNC Symbol;Acc:HGNC:717]
ENSG00000213742	-0,1632218	-0,0328101	0,17208212	0,68772362	0,85893428	ZNF337-AS1	ZNF337 antisense RNA 1 [Source:HGNC Symbol;Acc:HGNC:40759]
ENSG00000183506	-0,0777704	2,46645394	0,1719974	0,68779578	0,85895098	PI4KAP2	phosphatidylinositol 4-kinase alpha pseudogene 2 [Source:HGNC Symbol;Acc:HGNC:33577]
ENSG00000154727	-0,0634434	3,1547534	0,1717446	0,68801125	0,85914663	GABPA	GA binding protein transcription factor subunit alpha [Source:HGNC Symbol;Acc:HGNC:4071]
ENSG00000231245	0,20084752	-1,5572796	0,17162265	0,68811527	0,85920307	C1DP1	C1D nuclear receptor corepressor pseudogene 1 [Source:HGNC Symbol;Acc:HGNC:31465]
ENSG00000077312	-0,0565428	7,44529901	0,17151449	0,68820755	0,85921228	SNRPA	small nuclear ribonucleoprotein polypeptide A [Source:HGNC Symbol;Acc:HGNC:11151]
ENSG00000116815	-0,093782	3,18405803	0,17147615	0,68824027	0,85921228	CD58	CD58 molecule [Source:HGNC Symbol;Acc:HGNC:1688]
ENSG00000007866	0,06301924	5,30884358	0,17130544	0,68838602	0,85932081	TEAD3	TEA domain transcription factor 3 [Source:HGNC Symbol;Acc:HGNC:11716]

ENSG00000237882	-0,2201624	-1,8456267	0,17094795	0,68869152	0,8596287	PPIAP13	peptidylprolyl isomerase A pseudogene 13 [Source:HGNC Symbol;Acc:HGNC:9266]
ENSG00000064666	-0,0786858	8,41916597	0,17056882	0,68901591	0,85966637	CNN2	calponin 2 [Source:HGNC Symbol;Acc:HGNC:2156]
ENSG00000101310	-0,0794257	7,27674201	0,17058194	0,68900468	0,85966637	SEC23B	SEC23 homolog B, COPII coat complex component [Source:HGNC Symbol;Acc:HGNC:10702]
ENSG00000186654	-0,0635188	4,45142271	0,17069201	0,68891046	0,85966637	PRR5	proline rich 5 [Source:HGNC Symbol;Acc:HGNC:31682]
ENSG00000215067	-0,1810911	-0,0036739	0,17073466	0,68887396	0,85966637	ALOX12-AS1	ALOX12 antisense RNA 1 [Source:HGNC Symbol;Acc:HGNC:51342]
ENSG00000243646	0,209896	-1,5039449	0,17072308	0,68888387	0,85966637	IL10RB	interleukin 10 receptor subunit beta [Source:HGNC Symbol;Acc:HGNC:5965]
ENSG00000137100	0,06468887	5,04090675	0,17037142	0,68918498	0,85980388	DCTN3	dynactin subunit 3 [Source:HGNC Symbol;Acc:HGNC:2713]
ENSG00000185101	-0,0884987	1,49089895	0,17026799	0,68927362	0,85984103	ANO9	anoctamin 9 [Source:HGNC Symbol;Acc:HGNC:20679]
ENSG00000107164	0,07588733	5,79246358	0,17013028	0,68939168	0,85988772	FUBP3	far upstream element binding protein 3 [Source:HGNC Symbol;Acc:HGNC:4005]
ENSG00000124614	-0,1098573	0,40036475	0,16974734	0,68972028	0,85988772	RPS10	ribosomal protein S10 [Source:HGNC Symbol;Acc:HGNC:10383]
ENSG00000169499	0,11535223	1,14220492	0,16997677	0,68952335	0,85988772	PLEKHA2	pleckstrin homology domain containing A2 [Source:HGNC Symbol;Acc:HGNC:14336]
ENSG00000183323	-0,0727662	2,59599864	0,170051	0,68945968	0,85988772	CCDC125	coiled-coil domain containing 125 [Source:HGNC Symbol;Acc:HGNC:28924]
ENSG00000215158	-0,1461847	-0,5677883	0,16974408	0,68972307	0,85988772	AC138409.2	Putative beta-glucuronidase-like protein FLJ75287 pseudogene
ENSG00000257511	0,19692318	-1,1828435	0,16982072	0,68965727	0,85988772	AC084824.1	nucleosome assembly protein 1-like 1 (NAP1L1) pseudogene
ENSG00000288100	-0,2190458	-1,8958014	0,1697618	0,68970786	0,85988772	AL161663.2	novel transcript, antisense to STXBP6
ENSG00000011304	-0,0882473	8,18419846	0,16879632	0,69053824	0,86019599	PTBP1	polypyrimidine tract binding protein 1 [Source:HGNC Symbol;Acc:HGNC:9583]
ENSG00000099866	0,18243961	-0,2519289	0,16881739	0,69052008	0,86019599	MADCAM1	mucosal vascular addressin cell adhesion molecule 1 [Source:HGNC Symbol;Acc:HGNC:6765]
ENSG00000103326	-0,0603896	5,4743626	0,1683623	0,69091243	0,86019599	CAPN15	calpain 15 [Source:HGNC Symbol;Acc:HGNC:11182]
ENSG00000116455	-0,0547088	6,51229997	0,16895419	0,69040226	0,86019599	WDR77	WD repeat domain 77 [Source:HGNC Symbol;Acc:HGNC:29652]
ENSG00000130787	0,0873186	3,59479678	0,16843549	0,69084929	0,86019599	HIP1R	huntingtin interacting protein 1 related [Source:HGNC Symbol;Acc:HGNC:18415]
ENSG00000134318	-0,1061789	6,80413326	0,16875278	0,69057574	0,86019599	ROCK2	Rho associated coiled-coil containing protein kinase 2 [Source:HGNC Symbol;Acc:HGNC:10252]
ENSG00000147162	-0,099611	5,45235071	0,16912762	0,69025298	0,86019599	OGT	O-linked N-acetylglucosamine (GlcNAc) transferase [Source:HGNC Symbol;Acc:HGNC:8127]
ENSG00000164011	-0,0682031	3,08564289	0,1685469	0,6907532	0,86019599	ZNF691	zinc finger protein 691 [Source:HGNC Symbol;Acc:HGNC:28028]
ENSG00000165516	-0,0701286	5,39795667	0,16893705	0,69041702	0,86019599	KLHDC2	kelch domain containing 2 [Source:HGNC Symbol;Acc:HGNC:20231]
ENSG00000166508	0,07958358	8,35713505	0,16929455	0,69010937	0,86019599	MCM7	minichromosome maintenance complex component 7 [Source:HGNC Symbol;Acc:HGNC:6950]
ENSG00000168517	-0,0647434	5,38087172	0,16857259	0,69073106	0,86019599	HEXIM2	HEXIM P-TEFb complex subunit 2 [Source:HGNC Symbol;Acc:HGNC:28591]
ENSG00000169629	-0,148694	0,48768114	0,16840324	0,69087712	0,86019599	RGPD8	RANBP2 like and GRIP domain containing 8 [Source:HGNC Symbol;Acc:HGNC:9849]
ENSG00000169635	-0,1144814	3,19998768	0,1691369	0,69024499	0,86019599	HIC2	HIC ZBTB transcriptional repressor 2 [Source:HGNC Symbol;Acc:HGNC:18595]
ENSG00000197555	-0,0706928	4,45685437	0,16903151	0,6903357	0,86019599	SIPA1L1	signal induced proliferation associated 1 like 1 [Source:HGNC Symbol;Acc:HGNC:20284]
ENSG00000204632	0,08441208	2,85458675	0,16856872	0,69073439	0,86019599	HLA-G	major histocompatibility complex, class I, G [Source:HGNC Symbol;Acc:HGNC:4964]
ENSG00000226824	0,18130751	-0,3324921	0,16894338	0,69041157	0,86019599	AC006001.2	novel transcript
ENSG00000100983	0,06774088	6,72982717	0,1680967	0,69114171	0,8603348	GSS	glutathione synthetase [Source:HGNC Symbol;Acc:HGNC:4624]
ENSG00000237676	0,19998916	-1,4504066	0,1681305	0,69111252	0,8603348	RPL30P4	ribosomal protein L30 pseudogene 4 [Source:HGNC Symbol;Acc:HGNC:35871]
ENSG00000148426	0,06054061	4,16574182	0,1679187	0,69129549	0,8603796	PROSER2	proline and serine rich 2 [Source:HGNC Symbol;Acc:HGNC:23728]
ENSG00000259033	0,19052232	-1,1502008	0,16795322	0,69126565	0,8603796	AL356804.1	novel transcript
ENSG00000136273	-0,0637755	3,90327318	0,16759596	0,69157455	0,86063978	HUS1	HUS1 checkpoint clamp component [Source:HGNC Symbol;Acc:HGNC:5309]
ENSG00000215041	0,09080656	3,78540585	0,16754071	0,69162235	0,86063978	NEURL4	neuralized E3 ubiquitin protein ligase 4 [Source:HGNC Symbol;Acc:HGNC:34410]
ENSG00000100216	-0,0732243	6,72457275	0,16732855	0,69180601	0,86078239	TOMM22	translocase of outer mitochondrial membrane 22 [Source:HGNC Symbol;Acc:HGNC:18002]
ENSG00000155906	0,05341484	5,96228513	0,16727221	0,6918548	0,86078239	RMND1	required for meiotic nuclear division 1 homolog [Source:HGNC Symbol;Acc:HGNC:21176]
ENSG00000180628	-0,0682038	4,80297315	0,16718245	0,69193256	0,86080583	PCGF5	polycomb group ring finger 5 [Source:HGNC Symbol;Acc:HGNC:28264]
ENSG00000115884	0,05563254	8,67319004	0,16683888	0,69223042	0,86102973	SDC1	syndecan 1 [Source:HGNC Symbol;Acc:HGNC:10658]
ENSG00000162836	0,06105772	4,72231643	0,16690502	0,69217305	0,86102973	ACP6	acid phosphatase 6, lysophosphatidic [Source:HGNC Symbol;Acc:HGNC:29609]
ENSG00000100211	-0,0765607	3,8192472	0,16652924	0,69249918	0,86121737	CBY1	chibby family member 1, beta catenin antagonist [Source:HGNC Symbol;Acc:HGNC:1307]
ENSG00000204536	-0,067509	4,49617136	0,16654819	0,69248272	0,86121737	CCHCR1	coiled-coil alpha-helical rod protein 1 [Source:HGNC Symbol;Acc:HGNC:13930]

ENSG00000168876	0,10089237	2,10684994	0,1661794	0,69280317	0,86144877	ANKRD49	ankyrin repeat domain 49 [Source:HGNC Symbol;Acc:HGNC:25970]
ENSG00000284428	0,22593195	-0,9585501	0,16623217	0,6927573	0,86144877	AC092329.4	novel transcript
ENSG00000101019	-0,0546162	7,55816009	0,16596464	0,69298998	0,86155984	UQCC1	ubiquinol-cytochrome c reductase complex assembly factor 1 [Source:HGNC Symbol;Acc:HGNC:15891]
ENSG00000227879	-0,1504279	-0,4994635	0,16594112	0,69301045	0,86155984	PSPC1P1	paraspeckle component 1 pseudogene 1 [Source:HGNC Symbol;Acc:HGNC:42033]
ENSG00000123836	-0,0807804	2,44013136	0,165728	0,69319599	0,86161528	PFKFB2	6-phosphofructo-2-kinase/fructose-2,6-biphosphatase 2 [Source:HGNC Symbol;Acc:HGNC:8873]
ENSG00000135439	0,22171992	-1,0156903	0,16568667	0,69323198	0,86161528	AGAP2	ArfGAP with GTPase domain, ankyrin repeat and PH domain 2 [Source:HGNC Symbol;Acc:HGNC:16921]
ENSG00000204514	0,24430131	-0,8697631	0,16581425	0,69312088	0,86161528	ZNF814	zinc finger protein 814 [Source:HGNC Symbol;Acc:HGNC:33258]
ENSG00000184281	-0,0734895	5,21800993	0,16560923	0,69329945	0,86162583	TSSC4	tumor suppressing subtransferable candidate 4 [Source:HGNC Symbol;Acc:HGNC:12386]
ENSG00000084070	0,0616897	4,69486366	0,16536932	0,69350856	0,86167839	SMAP2	small ArfGAP2 [Source:HGNC Symbol;Acc:HGNC:25082]
ENSG00000127955	0,1173828	0,5055458	0,1652483	0,69361412	0,86167839	GNAI1	G protein subunit alpha i1 [Source:HGNC Symbol;Acc:HGNC:4384]
ENSG00000129317	0,06873714	3,10680474	0,16549052	0,6934029	0,86167839	PUS7L	pseudouridine synthase 7 like [Source:HGNC Symbol;Acc:HGNC:25276]
ENSG00000164620	-0,0636019	3,16425078	0,16518221	0,69367179	0,86167839	RELL2	RELT like 2 [Source:HGNC Symbol;Acc:HGNC:26902]
ENSG00000198590	0,21643953	-1,6009168	0,1650873	0,69375462	0,86167839	C3orf35	chromosome 3 open reading frame 35 [Source:HGNC Symbol;Acc:HGNC:24082]
ENSG00000225210	0,11105962	0,73403508	0,165153	0,69369727	0,86167839	DUXAP9	double homeobox A pseudogene 9 [Source:HGNC Symbol;Acc:HGNC:32188]
ENSG00000254726	-0,0689079	3,460173	0,16524977	0,69361284	0,86167839	MEX3A	mex-3 RNA binding family member A [Source:HGNC Symbol;Acc:HGNC:33482]
ENSG00000260285	-0,2271094	-1,2710168	0,16496879	0,69385809	0,86173364	AL133367.1	novel transcript, antisense to TRMT61A
ENSG00000114354	0,05691783	6,54604673	0,1647739	0,69402833	0,86185963	TFG	trafficking from ER to golgi regulator [Source:HGNC Symbol;Acc:HGNC:11758]
ENSG00000258741	-0,2321642	-1,8184301	0,16471762	0,69407752	0,86185963	H2AZ2P1	H2AZ2 pseudogene 1 [Source:HGNC Symbol;Acc:HGNC:31026]
ENSG00000272269	-0,1030948	0,86868748	0,16457882	0,69419887	0,86193705	AL138724.1	novel transcript, antisense to NUP153
ENSG00000169682	0,16796953	-1,0405232	0,16450254	0,69426559	0,86194663	SPNS1	sphingolipid transporter 1 (putative) [Source:HGNC Symbol;Acc:HGNC:30621]
ENSG00000090565	-0,0555451	5,77801006	0,16439661	0,69435827	0,86198844	RAB11FIP3	RAB11 family interacting protein 3 [Source:HGNC Symbol;Acc:HGNC:17224]
ENSG00000155275	-0,0793137	3,49535384	0,1643006	0,6944423	0,8620195	TRMT44	tRNA methyltransferase 44 homolog [Source:HGNC Symbol;Acc:HGNC:26653]
ENSG00000235275	0,13426282	-0,2469059	0,1640076	0,69469891	0,86226477	KRT18P16	keratin 18 pseudogene 16 [Source:HGNC Symbol;Acc:HGNC:33384]
ENSG00000062725	0,06567742	4,72034642	0,1639153	0,69477981	0,86229193	APPBP2	amyloid beta precursor protein binding protein 2 [Source:HGNC Symbol;Acc:HGNC:622]
ENSG00000127022	-0,076421	9,75526561	0,16380136	0,6948797	0,86234264	CANX	calnexin [Source:HGNC Symbol;Acc:HGNC:1473]
ENSG00000072756	-0,0621183	4,11353766	0,16314226	0,69545838	0,86298747	TRNT1	tRNA nucleotidyl transferase 1 [Source:HGNC Symbol;Acc:HGNC:17341]
ENSG00000131759	0,05851711	4,38888807	0,16245532	0,69606296	0,86335964	RARA	retinoic acid receptor alpha [Source:HGNC Symbol;Acc:HGNC:9864]
ENSG00000135638	-0,0813948	1,78423688	0,1625197	0,69600624	0,86335964	EMX1	empty spiracles homeobox 1 [Source:HGNC Symbol;Acc:HGNC:3340]
ENSG00000159399	0,08393624	7,3359289	0,16258255	0,69595088	0,86335964	HK2	hexokinase 2 [Source:HGNC Symbol;Acc:HGNC:4923]
ENSG00000173163	0,05718738	5,87673697	0,16267834	0,69586652	0,86335964	COMMD1	copper metabolism domain containing 1 [Source:HGNC Symbol;Acc:HGNC:23024]
ENSG00000196363	0,05517497	6,7208593	0,16247053	0,69604956	0,86335964	WDR5	WD repeat domain 5 [Source:HGNC Symbol;Acc:HGNC:12757]
ENSG00000238058	0,29280625	-1,5267421	0,16239867	0,69611289	0,86335964	AL355574.1	novel transcript
ENSG00000160710	0,08160391	6,57801926	0,16230033	0,69619958	0,86339386	ADAR	adenosine deaminase RNA specific [Source:HGNC Symbol;Acc:HGNC:225]
ENSG00000119471	0,05875489	6,03090068	0,16203901	0,69643009	0,86339852	HSDL2	hydroxysteroid dehydrogenase like 2 [Source:HGNC Symbol;Acc:HGNC:18572]
ENSG00000206341	-0,0708883	4,89387991	0,16209118	0,69638406	0,86339852	HLA-H	major histocompatibility complex, class I, H (pseudogene) [Source:HGNC Symbol;Acc:HGNC:4965]
ENSG00000230124	-0,0641487	5,1081345	0,16202808	0,69643974	0,86339852	ACBD6	acyl-CoA binding domain containing 6 [Source:HGNC Symbol;Acc:HGNC:23339]
ENSG00000259529	0,19306857	-0,231154	0,16209533	0,6963804	0,86339852	AL136295.5	novel transcript
ENSG00000135966	0,07382239	5,17336453	0,16157388	0,69684094	0,86380284	TGFBRAP1	transforming growth factor beta receptor associated protein 1 [Source:HGNC Symbol;Acc:HGNC:16836]
ENSG00000198721	-0,0576435	7,51799452	0,16152502	0,69688413	0,86380284	ECI2	enoyl-CoA delta isomerase 2 [Source:HGNC Symbol;Acc:HGNC:14601]
ENSG00000268350	-0,2064283	-1,3929418	0,16139852	0,69699601	0,86386823	FAM156B	family with sequence similarity 156 member B [Source:HGNC Symbol;Acc:HGNC:31962]
ENSG00000213793	0,09954272	1,44200673	0,16086623	0,69746733	0,86434123	ZNF888	zinc finger protein 888 [Source:HGNC Symbol;Acc:HGNC:38695]
ENSG00000232727	0,20997624	-1,6225361	0,16083391	0,69749598	0,86434123	YWHAEP1	tyrosine 3-monooxygenase/tryptophan 5-monooxygenase activation protein epsilon pseudogene 1 [Source:HGNC
ENSG00000131143	-0,0523789	9,62501751	0,1607395	0,69757968	0,86437163	COX4I1	cytochrome c oxidase subunit 4I1 [Source:HGNC Symbol;Acc:HGNC:2265]
ENSG00000065029	0,07353975	3,70954387	0,16047087	0,697818	0,86447536	ZNF76	zinc finger protein 76 [Source:HGNC Symbol;Acc:HGNC:13149]

ENSG00000125864	0,23866811	-1,0952205	0,16044505	0,69784091	0,86447536	BFSP1	beaded filament structural protein 1 [Source:HGNC Symbol;Acc:HGNC:1040]
ENSG00000188976	0,10364508	7,48310197	0,16049244	0,69779885	0,86447536	NOC2L	NOC2 like nucleolar associated transcriptional repressor [Source:HGNC Symbol;Acc:HGNC:24517]
ENSG00000129657	0,05363866	6,00741067	0,16012066	0,69812904	0,86475896	SEC14L1	SEC14 like lipid binding 1 [Source:HGNC Symbol;Acc:HGNC:10698]
ENSG00000105063	0,05503634	6,6826004	0,15986242	0,69835866	0,86476926	PPP6R1	protein phosphatase 6 regulatory subunit 1 [Source:HGNC Symbol;Acc:HGNC:29195]
ENSG00000105393	-0,0859346	4,86849316	0,15962652	0,6985686	0,86476926	BABAM1	BRISC and BRCA1 A complex member 1 [Source:HGNC Symbol;Acc:HGNC:25008]
ENSG00000109736	-0,0607157	5,2851301	0,15953146	0,69865325	0,86476926	MFSD10	major facilitator superfamily domain containing 10 [Source:HGNC Symbol;Acc:HGNC:16894]
ENSG00000136295	-0,0824492	6,25170548	0,15999517	0,69824059	0,86476926	TTYH3	tweety family member 3 [Source:HGNC Symbol;Acc:HGNC:22222]
ENSG00000142920	0,13133023	0,74221493	0,15976254	0,69844752	0,86476926	AZIN2	antizyme inhibitor 2 [Source:HGNC Symbol;Acc:HGNC:29957]
ENSG00000168769	0,15006486	0,44254149	0,15969244	0,69850992	0,86476926	TET2	tet methylcytosine dioxygenase 2 [Source:HGNC Symbol;Acc:HGNC:25941]
ENSG00000176658	0,09767116	4,02992099	0,15951253	0,69867011	0,86476926	MYO1D	myosin ID [Source:HGNC Symbol;Acc:HGNC:7598]
ENSG00000186470	-0,0606318	3,87443714	0,15986859	0,69835316	0,86476926	BTN3A2	butyrophilin subfamily 3 member A2 [Source:HGNC Symbol;Acc:HGNC:1139]
ENSG00000272764	0,17115931	-1,1517931	0,15978313	0,6984292	0,86476926	AL596094.1	novel transcript
ENSG00000103199	0,06506179	3,57193258	0,15911702	0,69902266	0,86513233	ZNF500	zinc finger protein 500 [Source:HGNC Symbol;Acc:HGNC:23716]
ENSG00000091436	-0,0777441	2,53110741	0,15855942	0,69952056	0,86535107	MAP3K20	mitogen-activated protein kinase kinase kinase 20 [Source:HGNC Symbol;Acc:HGNC:17797]
ENSG00000120662	-0,0723062	3,13210662	0,15847241	0,69959835	0,86535107	MTRF1	mitochondrial translation release factor 1 [Source:HGNC Symbol;Acc:HGNC:7469]
ENSG00000125835	0,05967913	9,57246633	0,15871883	0,69937812	0,86535107	SNRPB	small nuclear ribonucleoprotein polypeptides B and B1 [Source:HGNC Symbol;Acc:HGNC:11153]
ENSG00000132356	0,07708277	4,22900416	0,15844852	0,69961971	0,86535107	PRKAA1	protein kinase AMP-activated catalytic subunit alpha 1 [Source:HGNC Symbol;Acc:HGNC:9376]
ENSG00000135205	-0,1963689	-1,3378865	0,1587717	0,69933088	0,86535107	CCDC146	coiled-coil domain containing 146 [Source:HGNC Symbol;Acc:HGNC:29296]
ENSG00000135226	0,21632665	-2,0223628	0,15818615	0,69985445	0,86535107	UGT2B28	UDP glucuronosyltransferase family 2 member B28 [Source:HGNC Symbol;Acc:HGNC:13479]
ENSG00000156860	0,0773928	5,03086739	0,15855921	0,69952075	0,86535107	FBRS	fibrosin [Source:HGNC Symbol;Acc:HGNC:20442]
ENSG00000164024	0,06233747	6,36463023	0,15813032	0,69990442	0,86535107	METAP1	methionyl aminopeptidase 1 [Source:HGNC Symbol;Acc:HGNC:15789]
ENSG00000175334	-0,0617836	6,67293832	0,15812386	0,69991021	0,86535107	BANF1	BAF nuclear assembly factor 1 [Source:HGNC Symbol;Acc:HGNC:17397]
ENSG00000175826	0,06313928	6,6946361	0,15818241	0,69985779	0,86535107	CTDNEP1	CTD nuclear envelope phosphatase 1 [Source:HGNC Symbol;Acc:HGNC:19085]
ENSG00000248429	-0,1324637	0,90132533	0,15829531	0,69975675	0,86535107	FAM198B-AS1	FAM198B antisense RNA 1 [Source:HGNC Symbol;Acc:HGNC:53132]
ENSG00000255703	-0,208172	-1,8089766	0,15834246	0,69971457	0,86535107	AC127070.3	NHP2 non-histone chromosome protein 2-like 1 (S. cerevisiae) (NHP2L1) pseudogene
ENSG00000122257	0,08022104	5,3000162	0,15756361	0,70041232	0,86538044	RBBP6	RB binding protein 6, ubiquitin ligase [Source:HGNC Symbol;Acc:HGNC:9889]
ENSG00000142507	-0,0515364	8,52157819	0,15783228	0,7001714	0,86538044	PSMB6	proteasome 20S subunit beta 6 [Source:HGNC Symbol;Acc:HGNC:9543]
ENSG00000143315	0,06573041	3,75248035	0,15750258	0,70046709	0,86538044	PIGM	phosphatidylinositol glycan anchor biosynthesis class M [Source:HGNC Symbol;Acc:HGNC:18858]
ENSG00000144028	0,11271029	7,93924126	0,15772186	0,70027039	0,86538044	SNRNP200	small nuclear ribonucleoprotein U5 subunit 200 [Source:HGNC Symbol;Acc:HGNC:30859]
ENSG00000164975	-0,0713268	4,86731434	0,15771679	0,70027493	0,86538044	SNAPC3	small nuclear RNA activating complex polypeptide 3 [Source:HGNC Symbol;Acc:HGNC:11136]
ENSG00000166436	-0,085204	1,27340354	0,15791038	0,70010141	0,86538044	TRIM66	tripartite motif containing 66 [Source:HGNC Symbol;Acc:HGNC:29005]
ENSG00000173442	-0,0636955	3,88170254	0,15753924	0,70043418	0,86538044	EHBP1L1	EH domain binding protein 1 like 1 [Source:HGNC Symbol;Acc:HGNC:30682]
ENSG00000238103	-0,170068	-1,136959	0,15774177	0,70025254	0,86538044	RPL9P7	ribosomal protein L9 pseudogene 7 [Source:HGNC Symbol;Acc:HGNC:30335]
ENSG00000260007	0,22393505	-1,8687011	0,15764785	0,70033675	0,86538044	AC107871.1	novel protein
ENSG00000116489	-0,0559546	6,15093353	0,15724728	0,70069629	0,86555061	CAPZA1	capping actin protein of muscle Z-line subunit alpha 1 [Source:HGNC Symbol;Acc:HGNC:1488]
ENSG00000189233	-0,2543165	-0,8609897	0,15721718	0,70072333	0,86555061	NUGGC	nuclear GTPase, germinal center associated [Source:HGNC Symbol;Acc:HGNC:33550]
ENSG00000196951	-0,1237184	-0,0967148	0,15691601	0,70099403	0,86581178	SCOC-AS1	SCOC antisense RNA 1 [Source:HGNC Symbol;Acc:HGNC:50601]
ENSG00000241685	0,0558255	4,92542875	0,15680895	0,70109033	0,86585752	ARPC1A	actin related protein 2/3 complex subunit 1A [Source:HGNC Symbol;Acc:HGNC:703]
ENSG00000134900	0,0735723	5,02990061	0,15648721	0,70137998	0,86589317	TPP2	tripeptidyl peptidase 2 [Source:HGNC Symbol;Acc:HGNC:12016]
ENSG00000182518	-0,079129	3,18987543	0,1564962	0,70137188	0,86589317	FAM104B	family with sequence similarity 104 member B [Source:HGNC Symbol;Acc:HGNC:25085]
ENSG00000223668	0,14225428	-0,5200162	0,15644772	0,70141555	0,86589317	EEF1A1P24	eukaryotic translation elongation factor 1 alpha 1 pseudogene 24 [Source:HGNC Symbol;Acc:HGNC:37897]
ENSG00000269293	-0,1265334	1,81333603	0,15667557	0,70121036	0,86589317	ZSCAN16-AS1	ZSCAN16 antisense RNA 1 [Source:HGNC Symbol;Acc:HGNC:48982]
ENSG00000280383	0,10944371	0,76876326	0,1565192	0,70135116	0,86589317	Z95331.1	novel transcript, sense overlapping ATXN10
ENSG00000259353	-0,1294054	0,20882362	0,15634065	0,70151202	0,86593909	AC090515.4	novel transcript, antisense to FAM63B

ENSG00000108557	0,10996545	3,8791838	0,15614694	0,70168667	0,8660563	RAI1	retinoic acid induced 1 [Source:HGNC Symbol;Acc:HGNC:9834]
ENSG00000145833	-0,0757767	6,21239809	0,15610385	0,70172554	0,8660563	DDX46	DEAD-box helicase 46 [Source:HGNC Symbol;Acc:HGNC:18681]
ENSG00000145476	0,07836248	2,08748841	0,15573546	0,70205808	0,86607023	CYP4V2	cytochrome P450 family 4 subfamily V member 2 [Source:HGNC Symbol;Acc:HGNC:23198]
ENSG00000165661	-0,0996832	6,16294838	0,15574537	0,70204914	0,86607023	QSOX2	quiescin sulfhydryl oxidase 2 [Source:HGNC Symbol;Acc:HGNC:30249]
ENSG00000184988	-0,0705631	3,29394717	0,15576073	0,70203526	0,86607023	TMEM106A	transmembrane protein 106A [Source:HGNC Symbol;Acc:HGNC:28288]
ENSG00000232368	-0,0737458	2,07489297	0,15569733	0,70209253	0,86607023	FTLP2	ferritin light chain pseudogene 2 [Source:HGNC Symbol;Acc:HGNC:4001]
ENSG00000253173	-0,180115	-1,1251918	0,15578279	0,70201534	0,86607023	H2AZP2	H2A.Z histone pseudogene 2 [Source:HGNC Symbol;Acc:HGNC:32279]
ENSG00000260751	-0,2206202	-1,6143295	0,15590848	0,70190185	0,86607023	AC008870.2	novel transcript
ENSG00000099797	-0,067453	8,35966064	0,15525073	0,70249635	0,86626043	TECR	trans-2,3-enoyl-CoA reductase [Source:HGNC Symbol;Acc:HGNC:4551]
ENSG00000124635	-0,2166916	-1,5993298	0,1552922	0,70245882	0,86626043	H2BC11	H2B clustered histone 11 [Source:HGNC Symbol;Acc:HGNC:4761]
ENSG00000130202	0,05325294	8,37164911	0,15532469	0,70242943	0,86626043	NECTIN2	nectin cell adhesion molecule 2 [Source:HGNC Symbol;Acc:HGNC:9707]
ENSG00000167964	0,23541807	-0,6738481	0,15513346	0,7026025	0,86626043	RAB26	RAB26, member RAS oncogene family [Source:HGNC Symbol;Acc:HGNC:14259]
ENSG00000182473	-0,0778101	6,12636977	0,15522111	0,70252316	0,86626043	EXOC7	exocyst complex component 7 [Source:HGNC Symbol;Acc:HGNC:23214]
ENSG00000224051	0,07836359	4,38823558	0,15514312	0,70259375	0,86626043	CPTP	ceramide-1-phosphate transfer protein [Source:HGNC Symbol;Acc:HGNC:28116]
ENSG00000132004	-0,0590047	3,5397386	0,15498636	0,70273571	0,86628085	FBXW9	F-box and WD repeat domain containing 9 [Source:HGNC Symbol;Acc:HGNC:28136]
ENSG00000165238	0,11331265	4,31491015	0,15498422	0,70273765	0,86628085	WNK2	WNK lysine deficient protein kinase 2 [Source:HGNC Symbol;Acc:HGNC:14542]
ENSG00000125844	0,17654732	9,63194	0,15466436	0,70302758	0,86641892	RRBP1	ribosome binding protein 1 [Source:HGNC Symbol;Acc:HGNC:10448]
ENSG00000145736	-0,073809	2,60806807	0,15472265	0,70297472	0,86641892	GTF2H2	general transcription factor IIH subunit 2 [Source:HGNC Symbol;Acc:HGNC:4656]
ENSG00000169814	0,06268751	4,7666263	0,15478487	0,7029183	0,86641892	BTD	biotinidase [Source:HGNC Symbol;Acc:HGNC:1122]
ENSG00000005189	0,07269306	3,28875917	0,15458095	0,70310325	0,86643908	REXO5	RNA exonuclease 5 [Source:HGNC Symbol;Acc:HGNC:24661]
ENSG00000239779	0,15689582	-0,1754858	0,15448515	0,70319018	0,86647312	WBP1	WW domain binding protein 1 [Source:HGNC Symbol;Acc:HGNC:12737]
ENSG00000253541	0,24570406	-2,0922347	0,15441869	0,7032505	0,86647437	SEPTIN10P1	septin 10 pseudogene 1 [Source:HGNC Symbol;Acc:HGNC:40018]
ENSG00000242327	0,10538436	0,6506951	0,15408911	0,70354988	0,86669704	AC023906.1	ribosomal protein S13 (RPS13) pseudogene
ENSG00000272172	-0,1489447	-0,6559319	0,15414024	0,70350341	0,86669704	AC138696.2	novel transcript, antisense to ZNF696
ENSG00000279041	0,36879535	-1,7980581	0,15399673	0,70363387	0,86672742	AC102945.2	TEC
ENSG00000007376	-0,0615745	4,99978243	0,15382544	0,70378966	0,86677315	RPUSD1	RNA pseudouridine synthase domain containing 1 [Source:HGNC Symbol;Acc:HGNC:14173]
ENSG00000104866	0,05809627	4,0241534	0,15385622	0,70376166	0,86677315	PPP1R37	protein phosphatase 1 regulatory subunit 37 [Source:HGNC Symbol;Acc:HGNC:27607]
ENSG00000258429	-0,0765043	1,97029984	0,1535955	0,70399897	0,86695785	PDF	peptide deformylase, mitochondrial [Source:HGNC Symbol;Acc:HGNC:30012]
ENSG00000106603	-0,0661196	5,7991366	0,15336006	0,70421347	0,86701886	COA1	cytochrome c oxidase assembly factor 1 homolog [Source:HGNC Symbol;Acc:HGNC:21868]
ENSG00000177225	-0,0660143	4,64226526	0,1533457	0,70422656	0,86701886	GATD1	glutamine amidotransferase like class 1 domain containing 1 [Source:HGNC Symbol;Acc:HGNC:26616]
ENSG00000182791	-0,1991673	-1,0885563	0,15346485	0,70411797	0,86701886	CCDC87	coiled-coil domain containing 87 [Source:HGNC Symbol;Acc:HGNC:25579]
ENSG00000067601	0,16687965	-0,7741975	0,15309929	0,70445127	0,86714344	PMS2P4	PMS1 homolog 2, mismatch repair system component pseudogene 4 [Source:HGNC Symbol;Acc:HGNC:9129]
ENSG00000261335	0,1951138	-1,1094506	0,15313535	0,70441837	0,86714344	AC005837.1	novel transcript, antisense to MXRA7
ENSG00000275221	-0,2084846	-1,8911195	0,1530395	0,70450582	0,86714344	H2AC13	H2A clustered histone 13 [Source:HGNC Symbol;Acc:HGNC:4725]
ENSG00000011009	-0,060225	6,45577838	0,1529053	0,70462832	0,8672035	LYPLA2	lysophospholipase 2 [Source:HGNC Symbol;Acc:HGNC:6738]
ENSG00000147099	0,10632035	2,54153146	0,15285601	0,70467333	0,8672035	HDAC8	histone deacetylase 8 [Source:HGNC Symbol;Acc:HGNC:13315]
ENSG00000232388	-0,0565725	6,17157471	0,15259783	0,70490922	0,86742072	SMIM26	small integral membrane protein 26 [Source:HGNC Symbol;Acc:HGNC:43430]
ENSG00000171365	0,08394726	4,40291271	0,15234763	0,70513804	0,86762921	CLCN5	chloride voltage-gated channel 5 [Source:HGNC Symbol;Acc:HGNC:2023]
ENSG00000105849	0,07357489	3,23856764	0,15214863	0,7053202	0,86778025	POLR1F	RNA polymerase I subunit F [Source:HGNC Symbol;Acc:HGNC:18027]
ENSG00000121671	0,06022539	3,74831811	0,15194894	0,70550312	0,86778607	CRY2	cryptochrome circadian regulator 2 [Source:HGNC Symbol;Acc:HGNC:2385]
ENSG00000145365	-0,0795161	2,49196051	0,15195174	0,70550056	0,86778607	TIFA	TRAF interacting protein with forkhead associated domain [Source:HGNC Symbol;Acc:HGNC:19075]
ENSG00000244921	0,14181902	-0,1776472	0,15205436	0,70540654	0,86778607	MTCYBP18	MT-CYB pseudogene 18 [Source:HGNC Symbol;Acc:HGNC:51976]
ENSG00000085231	-0,0575296	4,3271379	0,15158419	0,70583761	0,86811479	AK6	adenylate kinase 6 [Source:HGNC Symbol;Acc:HGNC:49151]
ENSG00000109920	-0,0567628	5,52900016	0,15120445	0,70618634	0,86811479	FNBP4	formin binding protein 4 [Source:HGNC Symbol;Acc:HGNC:19752]

ENSG00000111011	0,06278349	6,05257778	0,15122518	0,70616729	0,86811479	RSRC2	arginine and serine rich coiled-coil 2 [Source:HGNC Symbol;Acc:HGNC:30559]
ENSG00000143554	0,06618031	2,57936988	0,15143846	0,70597138	0,86811479	SLC27A3	solute carrier family 27 member 3 [Source:HGNC Symbol;Acc:HGNC:10997]
ENSG00000197128	0,08713939	1,15846927	0,15148089	0,70593243	0,86811479	ZNF772	zinc finger protein 772 [Source:HGNC Symbol;Acc:HGNC:33106]
ENSG00000214046	0,06271981	5,66919892	0,15124612	0,70614805	0,86811479	SMIM7	small integral membrane protein 7 [Source:HGNC Symbol;Acc:HGNC:28419]
ENSG00000238150	-0,1928241	-1,2657579	0,15120726	0,70618376	0,86811479	AC008753.1	ribosomal protein, large, P1 (RPLP1) pseudogene
ENSG00000163702	-0,069422	4,49107225	0,15089713	0,70646894	0,86838913	IL17RC	interleukin 17 receptor C [Source:HGNC Symbol;Acc:HGNC:18358]
ENSG00000160766	0,10045137	0,76629435	0,15082285	0,7065373	0,86840009	GBAP1	glucosylceramidase beta pseudogene 1 [Source:HGNC Symbol;Acc:HGNC:4178]
ENSG00000138180	0,05780777	4,08851963	0,15071532	0,70663629	0,86844869	CEP55	centrosomal protein 55 [Source:HGNC Symbol;Acc:HGNC:1161]
ENSG00000102241	-0,0884247	5,61748579	0,15051701	0,70681896	0,86860011	HTATSF1	HIV-1 Tat specific factor 1 [Source:HGNC Symbol;Acc:HGNC:5276]
ENSG00000094916	-0,0691392	4,38112072	0,15016928	0,70713959	0,86892104	CBX5	chromobox 5 [Source:HGNC Symbol;Acc:HGNC:1555]
ENSG00000101109	0,07978936	5,00843395	0,14977328	0,70750526	0,86928893	STK4	serine/threonine kinase 4 [Source:HGNC Symbol;Acc:HGNC:11408]
ENSG00000141933	0,12519961	0,59428702	0,14965186	0,70761749	0,86928893	TPGS1	tubulin polyglutamylase complex subunit 1 [Source:HGNC Symbol;Acc:HGNC:25058]
ENSG00000152492	0,05258977	6,49065235	0,14969222	0,70758018	0,86928893	CCDC50	coiled-coil domain containing 50 [Source:HGNC Symbol;Acc:HGNC:18111]
ENSG00000177410	0,06824247	5,16226087	0,14936	0,70788748	0,86940128	ZFAS1	ZNFX1 antisense RNA 1 [Source:HGNC Symbol;Acc:HGNC:33101]
ENSG00000236264	-0,133593	-0,3324552	0,14941214	0,70783923	0,86940128	RPL26P30	ribosomal protein L26 pseudogene 30 [Source:HGNC Symbol;Acc:HGNC:36733]
ENSG00000251583	0,20608082	-1,3070686	0,14946162	0,70779345	0,86940128	MFFP2	MFF pseudogene 2 [Source:HGNC Symbol;Acc:HGNC:54540]
ENSG00000165609	-0,0810231	6,96936035	0,1489803	0,7082392	0,86976012	NUDT5	nudix hydrolase 5 [Source:HGNC Symbol;Acc:HGNC:8052]
ENSG00000163684	0,13161628	-0,2906321	0,14890135	0,70831239	0,86977689	RPP14	ribonuclease P/MRP subunit p14 [Source:HGNC Symbol;Acc:HGNC:30327]
ENSG00000279058	-0,4183801	-1,7023772	0,14875213	0,7084508	0,86987374	AGAP14P	ArfGAP with GTPase domain, ankyrin repeat and PH domain 14, pseudogene [Source:HGNC Symbol;Acc:HGNC:23
ENSG00000267727	0,22947331	-1,7335578	0,14866936	0,7085276	0,86989493	AC008738.5	novel transcript
ENSG00000213888	-0,1694373	-1,4730243	0,1483164	0,70885542	0,87022427	LINC01521	long intergenic non-protein coding RNA 1521 [Source:HGNC Symbol;Acc:HGNC:26029]
ENSG00000157637	0,05673884	6,87655183	0,14817795	0,70898412	0,87030914	SLC38A10	solute carrier family 38 member 10 [Source:HGNC Symbol;Acc:HGNC:28237]
ENSG00000087301	-0,074485	2,38171239	0,14796068	0,70918624	0,87042141	TXNDC16	thioredoxin domain containing 16 [Source:HGNC Symbol;Acc:HGNC:19965]
ENSG00000177119	0,08518949	5,41639075	0,14795155	0,70919474	0,87042141	ANO6	anoctamin 6 [Source:HGNC Symbol;Acc:HGNC:25240]
ENSG00000162604	-0,0564446	4,18619295	0,14769883	0,70943007	0,87063709	TM2D1	TM2 domain containing 1 [Source:HGNC Symbol;Acc:HGNC:24142]
ENSG00000013288	-0,0768052	4,40180306	0,14744964	0,70966234	0,87079952	MAN2B2	mannosidase alpha class 2B member 2 [Source:HGNC Symbol;Acc:HGNC:29623]
ENSG00000086061	-0,0592494	8,88731033	0,14712357	0,70996661	0,87079952	DNAJA1	DnaJ heat shock protein family (Hsp40) member A1 [Source:HGNC Symbol;Acc:HGNC:5229]
ENSG00000111817	-0,2156637	-0,614937	0,14704575	0,71003929	0,87079952	DSE	dermatan sulfate epimerase [Source:HGNC Symbol;Acc:HGNC:21144]
ENSG00000173681	0,08422407	1,7592351	0,14728226	0,70981849	0,87079952	BCLAF3	BCLAF1 and THRAP3 family member 3 [Source:HGNC Symbol;Acc:HGNC:27413]
ENSG00000235899	0,25643412	-1,7016587	0,14718882	0,7099057	0,87079952	LINC01564	long intergenic non-protein coding RNA 1564 [Source:HGNC Symbol;Acc:HGNC:51361]
ENSG00000242607	-0,1943811	-1,2193664	0,14707324	0,71001361	0,87079952	RPS3AP34	RPS3A pseudogene 34 [Source:HGNC Symbol;Acc:HGNC:36293]
ENSG00000244289	-0,1943811	-1,2193664	0,14707324	0,71001361	0,87079952	RPS3AP35	RPS3A pseudogene 35 [Source:HGNC Symbol;Acc:HGNC:35902]
ENSG00000279583	-0,31519	-1,7245511	0,14719081	0,70990384	0,87079952	AC009086.2	novel transcript
ENSG00000180626	0,12454049	0,57177102	0,146917	0,71015958	0,87080085	ZNF594	zinc finger protein 594 [Source:HGNC Symbol;Acc:HGNC:29392]
ENSG00000288596	0,12869674	0,20392683	0,14698016	0,71010056	0,87080085	C8orf44	chromosome 8 open reading frame 44 [Source:HGNC Symbol;Acc:HGNC:25646]
ENSG00000116285	0,05895581	7,25068666	0,14670983	0,71035326	0,87096523	ERRFI1	ERBB receptor feedback inhibitor 1 [Source:HGNC Symbol;Acc:HGNC:18185]
ENSG00000180764	-0,0935142	0,90137496	0,14649281	0,71055632	0,8711411	PIPSL	PIP5K1A and PSMD4 like (pseudogene) [Source:HGNC Symbol;Acc:HGNC:23733]
ENSG00000109181	0,05647946	6,88405616	0,14627512	0,71076019	0,87119679	UGT2B10	UDP glucuronosyltransferase family 2 member B10 [Source:HGNC Symbol;Acc:HGNC:12544]
ENSG00000175741	0,08397268	1,9420527	0,14618964	0,71084028	0,87119679	RWDD4P2	RWD domain containing 4 pseudogene 2 [Source:HGNC Symbol;Acc:HGNC:23754]
ENSG00000197157	0,10111489	9,05285538	0,14623024	0,71080224	0,87119679	SND1	staphylococcal nuclease and tudor domain containing 1 [Source:HGNC Symbol;Acc:HGNC:30646]
ENSG00000223694	0,17395213	-1,5233809	0,14626021	0,71077416	0,87119679	ADH5P3	ADH5 pseudogene 3 [Source:HGNC Symbol;Acc:HGNC:22991]
ENSG00000184898	-0,2114909	-1,2166874	0,14588117	0,71112956	0,87147821	RBM43	RNA binding motif protein 43 [Source:HGNC Symbol;Acc:HGNC:24790]
ENSG00000101199	-0,0741892	6,70203649	0,14567728	0,71132096	0,87148581	ARFGAP1	ADP ribosylation factor GTPase activating protein 1 [Source:HGNC Symbol;Acc:HGNC:15852]
ENSG00000105889	0,21526736	-1,6647999	0,14515622	0,71181081	0,87148581	STEAP1B	STEAP family member 1B [Source:HGNC Symbol;Acc:HGNC:41907]

ENSG00000111229	-0,0627026	7,7645818	0,14536761	0,71161195	0,87148581	ARPC3	actin related protein 2/3 complex subunit 3 [Source:HGNC Symbol;Acc:HGNC:706]
ENSG00000112312	-0,0677054	7,26622281	0,1451974	0,71177206	0,87148581	GMNN	geminin DNA replication inhibitor [Source:HGNC Symbol;Acc:HGNC:17493]
ENSG00000136026	0,08224223	8,07476106	0,14545262	0,71153204	0,87148581	CKAP4	cytoskeleton associated protein 4 [Source:HGNC Symbol;Acc:HGNC:16991]
ENSG00000139318	-0,0743411	6,71582134	0,14579473	0,71121069	0,87148581	DUSP6	dual specificity phosphatase 6 [Source:HGNC Symbol;Acc:HGNC:3072]
ENSG00000142188	-0,0697991	4,86829101	0,14529078	0,71168421	0,87148581	TMEM50B	transmembrane protein 50B [Source:HGNC Symbol;Acc:HGNC:1280]
ENSG00000164933	-0,1182812	0,51773074	0,14511286	0,71185161	0,87148581	SLC25A32	solute carrier family 25 member 32 [Source:HGNC Symbol;Acc:HGNC:29683]
ENSG00000184227	0,05940783	5,06096057	0,14512393	0,7118412	0,87148581	ACOT1	acyl-CoA thioesterase 1 [Source:HGNC Symbol;Acc:HGNC:33128]
ENSG00000196418	0,07973763	2,42842795	0,14519893	0,71177062	0,87148581	ZNF124	zinc finger protein 124 [Source:HGNC Symbol;Acc:HGNC:12907]
ENSG00000198081	-0,0570073	3,64183203	0,14533362	0,71164392	0,87148581	ZBTB14	zinc finger and BTB domain containing 14 [Source:HGNC Symbol;Acc:HGNC:12860]
ENSG00000268222	-0,0938702	0,76357074	0,1454632	0,71152209	0,87148581	EEF1A1P7	eukaryotic translation elongation factor 1 alpha 1 pseudogene 7 [Source:HGNC Symbol;Acc:HGNC:3202]
ENSG00000138646	0,20333325	-0,6150395	0,14498546	0,71197156	0,87155962	HERC5	HECT and RLD domain containing E3 ubiquitin protein ligase 5 [Source:HGNC Symbol;Acc:HGNC:24368]
ENSG00000198890	-0,0637144	4,17989484	0,14479687	0,71214922	0,87170406	PRMT6	protein arginine methyltransferase 6 [Source:HGNC Symbol;Acc:HGNC:18241]
ENSG00000236608	-0,2182623	-1,8281558	0,14459832	0,71233641	0,87186014	EIF4A1P6	eukaryotic translation initiation factor 4A1 pseudogene 6 [Source:HGNC Symbol;Acc:HGNC:37925]
ENSG00000205189	0,08103327	2,57659984	0,14446544	0,71246177	0,87186748	ZBTB10	zinc finger and BTB domain containing 10 [Source:HGNC Symbol;Acc:HGNC:30953]
ENSG00000248098	-0,1472518	-0,669698	0,14448315	0,71244506	0,87186748	BCKDHA	branched chain keto acid dehydrogenase E1 subunit alpha [Source:HGNC Symbol;Acc:HGNC:986]
ENSG00000203288	0,20446391	-0,5018754	0,14432543	0,71259393	0,87195617	TDRKH-AS1	TDRKH antisense RNA 1 [Source:HGNC Symbol;Acc:HGNC:40578]
ENSG00000000457	-0,0668696	2,82544161	0,14357469	0,71330385	0,87202142	SCYL3	SCY1 like pseudokinase 3 [Source:HGNC Symbol;Acc:HGNC:19285]
ENSG00000069849	0,057605	7,70868698	0,14381949	0,71307212	0,87202142	ATP1B3	ATPase Na+/K+ transporting subunit beta 3 [Source:HGNC Symbol;Acc:HGNC:806]
ENSG00000079462	0,07453405	5,45553782	0,14389664	0,71299914	0,87202142	PAFAH1B3	platelet activating factor acetylhydrolase 1b catalytic subunit 3 [Source:HGNC Symbol;Acc:HGNC:8576]
ENSG00000088727	0,07190392	2,91410237	0,14412144	0,71278662	0,87202142	KIF9	kinesin family member 9 [Source:HGNC Symbol;Acc:HGNC:16666]
ENSG00000103544	0,17525018	-0,6495182	0,14361526	0,71326544	0,87202142	VPS35L	VPS35 endosomal protein sorting factor like [Source:HGNC Symbol;Acc:HGNC:24641]
ENSG00000154265	0,092037	2,1062099	0,14394186	0,71295638	0,87202142	ABCA5	ATP binding cassette subfamily A member 5 [Source:HGNC Symbol;Acc:HGNC:35]
ENSG00000162191	0,05004865	6,90455226	0,14411255	0,71279502	0,87202142	UBXN1	UBX domain protein 1 [Source:HGNC Symbol;Acc:HGNC:18402]
ENSG00000164897	0,06155915	5,66427207	0,14358029	0,71329855	0,87202142	TMUB1	transmembrane and ubiquitin like domain containing 1 [Source:HGNC Symbol;Acc:HGNC:21709]
ENSG00000189056	0,08976517	8,39114806	0,14377073	0,71311826	0,87202142	RELN	reelin [Source:HGNC Symbol;Acc:HGNC:9957]
ENSG00000204314	-0,1394828	-0,438239	0,14371535	0,71317068	0,87202142	PRRT1	proline rich transmembrane protein 1 [Source:HGNC Symbol;Acc:HGNC:13943]
ENSG00000206159	-0,0951037	0,8876325	0,14375148	0,71313648	0,87202142	GYG2P1	glycogenin 2 pseudogene 1 [Source:HGNC Symbol;Acc:HGNC:4701]
ENSG00000004534	0,08000476	5,42534427	0,14326078	0,71360133	0,87209318	RBM6	RNA binding motif protein 6 [Source:HGNC Symbol;Acc:HGNC:9903]
ENSG00000063244	0,06152748	8,04606917	0,14330443	0,71355994	0,87209318	U2AF2	U2 small nuclear RNA auxiliary factor 2 [Source:HGNC Symbol;Acc:HGNC:23156]
ENSG00000164080	-0,1510317	2,4937514	0,14337224	0,71349566	0,87209318	RAD54L2	RAD54 like 2 [Source:HGNC Symbol;Acc:HGNC:29123]
ENSG00000204590	-0,050647	6,30909991	0,14343868	0,7134327	0,87209318	GNL1	G protein nucleolar 1 (putative) [Source:HGNC Symbol;Acc:HGNC:4413]
ENSG00000128694	-0,0601057	2,84424273	0,14272045	0,71411426	0,87225995	OSGEPL1	O-sialoglycoprotein endopeptidase like 1 [Source:HGNC Symbol;Acc:HGNC:23075]
ENSG00000134343	0,17250072	-1,3821067	0,14283246	0,71400784	0,87225995	ANO3	anoctamin 3 [Source:HGNC Symbol;Acc:HGNC:14004]
ENSG00000139613	-0,1237449	3,99632586	0,14281732	0,71402222	0,87225995	SMARCC2	SWI/SNF related, matrix associated, actin dependent regulator of chromatin subfamily c member 2 [Source:HGNC
ENSG00000148180	-0,0900476	7,79499259	0,14290464	0,71393929	0,87225995	GSN	gelsolin [Source:HGNC Symbol;Acc:HGNC:4620]
ENSG00000168214	0,07198728	3,13979653	0,14292855	0,71391658	0,87225995	RBPJ	recombination signal binding protein for immunoglobulin kappa J region [Source:HGNC Symbol;Acc:HGNC:5724]
ENSG00000235912	-0,1023471	0,41232618	0,14273601	0,71409947	0,87225995	AL031729.1	novel pseudogene
ENSG00000272767	-0,1694921	-1,3736938	0,1426768	0,71415574	0,87225995	JMJD1C-AS1	JMJD1C antisense RNA 1 [Source:HGNC Symbol;Acc:HGNC:28222]
ENSG00000164167	-0,0841562	5,0367876	0,14259429	0,71423418	0,87228283	LSM6	LSM6 homolog, U6 small nuclear RNA and mRNA degradation associated [Source:HGNC Symbol;Acc:HGNC:17017
ENSG00000004975	-0,051564	5,79977896	0,14190352	0,7148919	0,87232401	DVL2	dishevelled segment polarity protein 2 [Source:HGNC Symbol;Acc:HGNC:3086]
ENSG00000049089	-0,0868242	4,45963316	0,14186909	0,71492473	0,87232401	COL9A2	collagen type IX alpha 2 chain [Source:HGNC Symbol;Acc:HGNC:2218]
ENSG00000049759	0,0524579	7,14155418	0,14192187	0,7148744	0,87232401	NEDD4L	NEDD4 like E3 ubiquitin protein ligase [Source:HGNC Symbol;Acc:HGNC:7728]
ENSG00000099937	-0,0730833	8,35799914	0,14219577	0,71461341	0,87232401	SERPIND1	serpin family D member 1 [Source:HGNC Symbol;Acc:HGNC:4838]
ENSG00000106868	0,06615227	6,27463033	0,14210047	0,71470419	0,87232401	SUSD1	sushi domain containing 1 [Source:HGNC Symbol;Acc:HGNC:25413]

ENSG00000131381	0,06080369	3,19202622	0,14188102	0,71491335	0,87232401	RBSN	rabenosyn, RAB effector [Source:HGNC Symbol;Acc:HGNC:20759]
ENSG00000141258	-0,0677204	3,98048198	0,14192203	0,71487425	0,87232401	SGSM2	small G protein signaling modulator 2 [Source:HGNC Symbol;Acc:HGNC:29026]
ENSG00000143641	0,0665865	6,86464037	0,14190519	0,71489031	0,87232401	GALNT2	polypeptide N-acetylgalactosaminyltransferase 2 [Source:HGNC Symbol;Acc:HGNC:4124]
ENSG00000174738	-0,0766336	2,42208159	0,1423547	0,7144621	0,87232401	NR1D2	nuclear receptor subfamily 1 group D member 2 [Source:HGNC Symbol;Acc:HGNC:7963]
ENSG00000185418	-0,0711515	3,29597127	0,14241618	0,7144036	0,87232401	TARS3	threonyl-tRNA synthetase 3 [Source:HGNC Symbol;Acc:HGNC:24728]
ENSG00000275131	0,16774403	-1,2669017	0,14198683	0,71481248	0,87232401	AC241952.1	phosphodiesterase 4D interacting protein (PDE4DIP) pseudogene
ENSG00000198569	0,11666326	0,87711037	0,14180556	0,71498532	0,87232508	SLC34A3	solute carrier family 34 member 3 [Source:HGNC Symbol;Acc:HGNC:20305]
ENSG00000140455	-0,0539673	4,58452351	0,14155826	0,71522133	0,87254016	USP3	ubiquitin specific peptidase 3 [Source:HGNC Symbol;Acc:HGNC:12626]
ENSG00000105699	0,06313506	7,76016901	0,14140206	0,71537052	0,87264774	LSR	lipolysis stimulated lipoprotein receptor [Source:HGNC Symbol;Acc:HGNC:29572]
ENSG00000160294	-0,0690443	5,99023801	0,14134088	0,71542898	0,87264774	MCM3AP	minichromosome maintenance complex component 3 associated protein [Source:HGNC Symbol;Acc:HGNC:6946]
ENSG00000139514	-0,168796	5,67965021	0,14119996	0,71556369	0,87273919	SLC7A1	solute carrier family 7 member 1 [Source:HGNC Symbol;Acc:HGNC:11057]
ENSG00000172262	0,05626707	4,37784879	0,14107164	0,71568643	0,87281601	ZNF131	zinc finger protein 131 [Source:HGNC Symbol;Acc:HGNC:12915]
ENSG00000101216	-0,06047	4,81819802	0,14084438	0,71590395	0,87283392	GMEB2	glucocorticoid modulatory element binding protein 2 [Source:HGNC Symbol;Acc:HGNC:4371]
ENSG00000114859	0,0575696	4,43601166	0,14092312	0,71582857	0,87283392	CLCN2	chloride voltage-gated channel 2 [Source:HGNC Symbol;Acc:HGNC:2020]
ENSG00000184786	-0,08242	1,32053006	0,14083925	0,71590887	0,87283392	TCTE3	t-complex-associated-testis-expressed 3 [Source:HGNC Symbol;Acc:HGNC:11695]
ENSG00000214160	-0,0508063	6,36677326	0,14080664	0,71594009	0,87283392	ALG3	ALG3 alpha-1,3- mannosyltransferase [Source:HGNC Symbol;Acc:HGNC:23056]
ENSG00000113504	0,07210776	5,1910576	0,14066711	0,71607377	0,87292405	SLC12A7	solute carrier family 12 member 7 [Source:HGNC Symbol;Acc:HGNC:10915]
ENSG00000180964	-0,0711237	4,05391258	0,14053123	0,71620403	0,87293715	TCEAL8	transcription elongation factor A like 8 [Source:HGNC Symbol;Acc:HGNC:28683]
ENSG00000198546	-0,0648992	3,75914172	0,14054712	0,71618879	0,87293715	ZNF511	zinc finger protein 511 [Source:HGNC Symbol;Acc:HGNC:28445]
ENSG00000100226	0,10010111	4,66515282	0,14006104	0,7166553	0,87310199	GTPBP1	GTP binding protein 1 [Source:HGNC Symbol;Acc:HGNC:4669]
ENSG00000104497	-0,0964232	1,46683637	0,14031623	0,71641026	0,87310199	SNX16	sorting nexin 16 [Source:HGNC Symbol;Acc:HGNC:14980]
ENSG00000120254	-0,0762793	6,89265069	0,1401313	0,71658781	0,87310199	MTHFD1L	methylenetetrahydrofolate dehydrogenase (NADP+ dependent) 1 like [Source:HGNC Symbol;Acc:HGNC:21055]
ENSG00000166133	-0,0562659	4,42735166	0,14020351	0,71651847	0,87310199	RPUSD2	RNA pseudouridine synthase domain containing 2 [Source:HGNC Symbol;Acc:HGNC:24180]
ENSG00000223361	0,07650407	1,7013207	0,14009686	0,71662089	0,87310199	FTH1P10	ferritin heavy chain 1 pseudogene 10 [Source:HGNC Symbol;Acc:HGNC:3980]
ENSG00000241352	-0,0824446	1,34969531	0,14001674	0,71669786	0,87310199	AC007688.2	ribosomal protein S6 (RPS6) pseudogene
ENSG00000231043	-0,1443996	-0,8462017	0,13983217	0,71687528	0,8732453	AC007238.1	IK cytokine, down-regulator of HLA II (IK) pseudogene
ENSG00000248794	-0,1581388	-0,88	0,13974385	0,71696022	0,87327596	AC026436.1	ribosomal protein S12 (RPS12) pseudogene
ENSG00000272941	0,25968873	-1,5775745	0,13965606	0,71704469	0,87330604	AC083862.1	novel transcript, antisense to C7orf49
ENSG00000204568	0,05262683	7,70029366	0,13954284	0,71715366	0,87336595	MRPS18B	mitochondrial ribosomal protein S18B [Source:HGNC Symbol;Acc:HGNC:14516]
ENSG00000090316	0,05798227	6,30168539	0,13922491	0,71745995	0,87351213	MAEA	macrophage erythroblast attacher, E3 ubiquitin ligase [Source:HGNC Symbol;Acc:HGNC:13731]
ENSG00000119900	-0,1994509	-0,5583947	0,13917002	0,71751288	0,87351213	OGFRL1	opioid growth factor receptor like 1 [Source:HGNC Symbol;Acc:HGNC:21378]
ENSG00000124228	-0,084781	7,26794985	0,13926261	0,71742362	0,87351213	DDX27	DEAD-box helicase 27 [Source:HGNC Symbol;Acc:HGNC:15837]
ENSG00000227359	-0,2483159	-1,9208653	0,13932767	0,71736091	0,87351213	AC017074.1	novel transcript
ENSG00000072786	-0,0926005	4,10168871	0,13862182	0,71804208	0,87352634	STK10	serine/threonine kinase 10 [Source:HGNC Symbol;Acc:HGNC:11388]
ENSG00000079385	0,08303227	7,32211679	0,13896314	0,71771244	0,87352634	CEACAM1	CEA cell adhesion molecule 1 [Source:HGNC Symbol;Acc:HGNC:1814]
ENSG00000105707	0,05477277	6,62715072	0,13866249	0,71800277	0,87352634	HPN	hepsin [Source:HGNC Symbol;Acc:HGNC:5155]
ENSG00000147174	-0,2072655	-1,2983014	0,13878165	0,71788766	0,87352634	GCNA	germ cell nuclear acidic peptidase [Source:HGNC Symbol;Acc:HGNC:15805]
ENSG00000170619	-0,0617076	5,11093136	0,13865026	0,71801459	0,87352634	COMMD5	COMM domain containing 5 [Source:HGNC Symbol;Acc:HGNC:17902]
ENSG00000196924	-0,1556956	8,90158519	0,1386922	0,71797406	0,87352634	FLNA	filamin A [Source:HGNC Symbol;Acc:HGNC:3754]
ENSG00000198561	0,1355723	1,43277221	0,13902557	0,7176522	0,87352634	CTNND1	catenin delta 1 [Source:HGNC Symbol;Acc:HGNC:2515]
ENSG00000226900	-0,2676392	-1,9504029	0,13905772	0,71762118	0,87352634	AL451069.1	novel transcript
ENSG00000243547	-0,080192	1,88325287	0,13860049	0,71806268	0,87352634	HNRNPKP4	heterogeneous nuclear ribonucleoprotein K pseudogene 4 [Source:HGNC Symbol;Acc:HGNC:42377]
ENSG00000168958	0,07330418	6,65081521	0,13852269	0,71813789	0,87354509	MFF	mitochondrial fission factor [Source:HGNC Symbol;Acc:HGNC:24858]
ENSG00000074964	-0,062081	5,18687564	0,13832584	0,7183283	0,87363121	ARHGEF10L	Rho guanine nucleotide exchange factor 10 like [Source:HGNC Symbol;Acc:HGNC:25540]

ENSG00000204574	0,08802976	7,65438626	0,13837494	0,71828079	0,87363121	ABCF1	ATP binding cassette subfamily F member 1 [Source:HGNC Symbol;Acc:HGNC:70]
ENSG00000150456	-0,0818109	3,05800301	0,13825688	0,71839503	0,87363964	EEF1AKMT1	EEF1A lysine methyltransferase 1 [Source:HGNC Symbol;Acc:HGNC:27351]
ENSG00000109016	-0,0521543	5,79382064	0,13814816	0,71850028	0,87369491	DHRS7B	dehydrogenase/reductase 7B [Source:HGNC Symbol;Acc:HGNC:24547]
ENSG00000188234	0,07532949	1,68734273	0,13808389	0,71856253	0,87369787	AGAP4	ArfGAP with GTPase domain, ankyrin repeat and PH domain 4 [Source:HGNC Symbol;Acc:HGNC:23459]
ENSG00000110074	-0,0660297	6,22794419	0,1378943	0,71874624	0,87377663	FOXRED1	FAD dependent oxidoreductase domain containing 1 [Source:HGNC Symbol;Acc:HGNC:26927]
ENSG00000276141	0,11374186	0,17029664	0,1378936	0,71874692	0,87377663	WHAMMP3	WAS protein homolog associated with actin, golgi membranes and microtubules pseudogene 3 [Source:HGNC Syr
ENSG00000181544	0,10040081	0,374413	0,13755123	0,71907904	0,87410764	FANCB	FA complementation group B [Source:HGNC Symbol;Acc:HGNC:3583]
ENSG00000283078	-0,1541294	-0,6508959	0,13748251	0,71914576	0,87411601	AL137077.2	novel transcript
ENSG00000130985	-0,0937374	7,76708693	0,13735239	0,71927214	0,87412827	UBA1	ubiquitin like modifier activating enzyme 1 [Source:HGNC Symbol;Acc:HGNC:12469]
ENSG00000233608	0,17332321	-0,1050836	0,13734893	0,71927551	0,87412827	TWIST2	twist family bHLH transcription factor 2 [Source:HGNC Symbol;Acc:HGNC:20670]
ENSG00000100395	0,04853395	5,84579712	0,13722299	0,7193979	0,87413157	L3MBTL2	L3MBTL histone methyl-lysine binding protein 2 [Source:HGNC Symbol;Acc:HGNC:18594]
ENSG00000146414	0,06356008	2,82633178	0,13726324	0,71935877	0,87413157	SHPRH	SNF2 histone linker PHD RING helicase [Source:HGNC Symbol;Acc:HGNC:19336]
ENSG00000106344	-0,1061302	5,80188993	0,13714991	0,71946895	0,8741452	RBM28	RNA binding motif protein 28 [Source:HGNC Symbol;Acc:HGNC:21863]
ENSG00000072682	0,07175725	6,20462186	0,1366098	0,71999474	0,87427568	P4HA2	prolyl 4-hydroxylase subunit alpha 2 [Source:HGNC Symbol;Acc:HGNC:8547]
ENSG00000112078	-0,0536872	6,00341648	0,13671825	0,71988907	0,87427568	KCTD20	potassium channel tetramerization domain containing 20 [Source:HGNC Symbol;Acc:HGNC:21052]
ENSG00000114554	0,07927279	5,33123076	0,1369452	0,71966809	0,87427568	PLXNA1	plexin A1 [Source:HGNC Symbol;Acc:HGNC:9099]
ENSG00000144736	0,06103541	3,97682168	0,13668018	0,71992616	0,87427568	SHQ1	SHQ1, H/ACA ribonucleoprotein assembly factor [Source:HGNC Symbol;Acc:HGNC:25543]
ENSG00000164221	-0,0715085	1,83663739	0,13671216	0,719895	0,87427568	CCDC112	coiled-coil domain containing 112 [Source:HGNC Symbol;Acc:HGNC:28599]
ENSG00000261351	-0,1417651	-0,2710657	0,13660927	0,71999526	0,87427568	AC116913.1	novel transcript, antisense to MAP2K1
ENSG00000280254	-0,1563693	-1,02359	0,13673402	0,7198737	0,87427568	AC233723.2	TEC
ENSG00000278932	0,18525623	-1,5963609	0,13641462	0,72018503	0,87443344	CR381653.1	novel transcript
ENSG00000144026	0,07123749	2,35463374	0,13634569	0,72025228	0,87444242	ZNF514	zinc finger protein 514 [Source:HGNC Symbol;Acc:HGNC:25894]
ENSG00000153896	0,09885207	1,9559464	0,13591261	0,72067521	0,87488318	ZNF599	zinc finger protein 599 [Source:HGNC Symbol;Acc:HGNC:26408]
ENSG00000280287	0,1852823	-1,0669275	0,13565767	0,72092454	0,87511314	AC131212.3	novel transcript
ENSG00000128973	0,05342454	4,73957454	0,13553119	0,72104834	0,87519069	CLN6	CLN6 transmembrane ER protein [Source:HGNC Symbol;Acc:HGNC:2077]
ENSG00000186230	0,06957693	2,20943812	0,13545227	0,72112562	0,87521178	ZNF749	zinc finger protein 749 [Source:HGNC Symbol;Acc:HGNC:32783]
ENSG00000227063	-0,0921356	0,73265878	0,1353666	0,72120953	0,87524091	RPL41P1	ribosomal protein L41 pseudogene 1 [Source:HGNC Symbol;Acc:HGNC:10356]
ENSG00000287299	-0,1982692	-1,7716888	0,13520701	0,72136594	0,87535801	AC012459.1	novel transcript, antisense to SPATS2L
ENSG00000242088	-0,1487426	-0,8950541	0,13508754	0,7214831	0,87542745	AC090602.1	ribosomal protein S27 (RPS27) pseudogene
ENSG00000151500	0,0653978	5,75374489	0,13494614	0,72162184	0,8754822	THYN1	thymocyte nuclear protein 1 [Source:HGNC Symbol;Acc:HGNC:29560]
ENSG00000283023	-0,2141248	-1,2924316	0,13491941	0,72164807	0,8754822	FRG1GP	FSHD region gene 1 family member G, pseudogene [Source:HGNC Symbol;Acc:HGNC:51766]
ENSG00000168917	0,06771489	4,03873167	0,13468933	0,72187403	0,8756836	SLC35G2	solute carrier family 35 member G2 [Source:HGNC Symbol;Acc:HGNC:28480]
ENSG00000071189	0,06070633	4,02038313	0,13456313	0,72199806	0,8757029	SNX13	sorting nexin 13 [Source:HGNC Symbol;Acc:HGNC:21335]
ENSG00000096746	-0,0490232	7,10814697	0,13450183	0,72205832	0,8757029	HNRNPH3	heterogeneous nuclear ribonucleoprotein H3 [Source:HGNC Symbol;Acc:HGNC:5043]
ENSG00000102096	-0,0565888	4,1559789	0,13433644	0,72222101	0,8757029	PIM2	Pim-2 proto-oncogene, serine/threonine kinase [Source:HGNC Symbol;Acc:HGNC:8987]
ENSG00000132016	0,12618745	-0,4439068	0,13434309	0,72221447	0,8757029	C19orf57	chromosome 19 open reading frame 57 [Source:HGNC Symbol;Acc:HGNC:28153]
ENSG00000174775	0,06340748	4,80862269	0,13430741	0,72224959	0,8757029	HRAS	HRas proto-oncogene, GTPase [Source:HGNC Symbol;Acc:HGNC:5173]
ENSG00000226049	0,1440405	-1,0332476	0,13435313	0,7222046	0,8757029	TLK2P1	tousled like kinase 2 pseudogene 1 [Source:HGNC Symbol;Acc:HGNC:18048]
ENSG00000274104	-0,129518	0,10315033	0,13400215	0,7225502	0,87599468	AC020910.5	novel transcript
ENSG00000164880	0,11938111	5,64516138	0,13373058	0,72281797	0,8762466	INTS1	integrator complex subunit 1 [Source:HGNC Symbol;Acc:HGNC:24555]
ENSG00000254244	-0,2014878	-0,8572309	0,13366735	0,72288036	0,87624952	PAICSP4	phosphoribosylaminoimidazole carboxylase, phosphoribosylaminoimidazole succinocarboxamide synthetase pseu
ENSG00000013374	-0,0570087	4,81201627	0,13342769	0,72311699	0,87631822	NUB1	negative regulator of ubiquitin like proteins 1 [Source:HGNC Symbol;Acc:HGNC:17623]
ENSG00000115942	-0,0532768	5,95523017	0,13343567	0,72310911	0,87631822	ORC2	origin recognition complex subunit 2 [Source:HGNC Symbol;Acc:HGNC:8488]
ENSG00000272812	-0,1359028	-0,4845802	0,13354005	0,72300603	0,87631822	AC004908.2	novel transcript, sense intronic to ZNF596

ENSG00000160113	-0,0596028	5,76089439	0,13327518	0,7232677	0,87642815	NR2F6	nuclear receptor subfamily 2 group F member 6 [Source:HGNC Symbol;Acc:HGNC:7977]
ENSG00000150977	0,07735238	4,96206362	0,13318003	0,72336178	0,87646945	RILPL2	Rab interacting lysosomal protein like 2 [Source:HGNC Symbol;Acc:HGNC:28787]
ENSG00000146858	-0,1511186	-0,5413276	0,13258128	0,72395465	0,87711507	ZC3HAV1L	zinc finger CCCH-type containing, antiviral 1 like [Source:HGNC Symbol;Acc:HGNC:22423]
ENSG00000110888	-0,0694738	3,7660419	0,13241937	0,72411524	0,87715047	CAPRIN2	caprin family member 2 [Source:HGNC Symbol;Acc:HGNC:21259]
ENSG00000154059	-0,0725696	2,30724989	0,13240673	0,72412778	0,87715047	IMPACT	impact RWD domain protein [Source:HGNC Symbol;Acc:HGNC:20387]
ENSG00000184863	0,13294487	3,8166859	0,13237023	0,724164	0,87715047	RBM33	RNA binding motif protein 33 [Source:HGNC Symbol;Acc:HGNC:27223]
ENSG00000090861	-0,1097014	8,09618165	0,13187392	0,72465705	0,8771926	AARS1	alanyl-tRNA synthetase 1 [Source:HGNC Symbol;Acc:HGNC:20]
ENSG00000125775	-0,1271066	-0,266513	0,13223977	0,7242935	0,8771926	SDCBP2	syndecan binding protein 2 [Source:HGNC Symbol;Acc:HGNC:15756]
ENSG00000135540	0,1022126	5,92992905	0,13208616	0,72444608	0,8771926	NHSL1	NHS like 1 [Source:HGNC Symbol;Acc:HGNC:21021]
ENSG00000143429	0,06013935	3,15540515	0,13213528	0,72439728	0,8771926	LSP1P4	LSP1 pseudogene 4 [Source:HGNC Symbol;Acc:HGNC:53915]
ENSG00000149485	-0,0531729	7,42491498	0,13186411	0,72466681	0,8771926	FADS1	fatty acid desaturase 1 [Source:HGNC Symbol;Acc:HGNC:3574]
ENSG00000163132	-0,072262	3,4838765	0,13188769	0,72464336	0,8771926	MSX1	msh homeobox 1 [Source:HGNC Symbol;Acc:HGNC:7391]
ENSG00000180998	0,1488092	-0,2628645	0,13185171	0,72467914	0,8771926	GPR137C	G protein-coupled receptor 137C [Source:HGNC Symbol;Acc:HGNC:25445]
ENSG00000241458	-0,1473679	-0,919038	0,13198255	0,72454905	0,8771926	RPL7P19	ribosomal protein L7 pseudogene 19 [Source:HGNC Symbol;Acc:HGNC:36268]
ENSG00000118965	-0,0685243	4,06401105	0,13178764	0,72474288	0,87719707	WDR35	WD repeat domain 35 [Source:HGNC Symbol;Acc:HGNC:29250]
ENSG00000136098	-0,0566244	5,79496473	0,13162609	0,72490365	0,87723283	NEK3	NIMA related kinase 3 [Source:HGNC Symbol;Acc:HGNC:7746]
ENSG00000182872	-0,051876	6,49801432	0,13163263	0,72489715	0,87723283	RBM10	RNA binding motif protein 10 [Source:HGNC Symbol;Acc:HGNC:9896]
ENSG00000185522	0,13548657	-0,0663619	0,13157697	0,72495256	0,87723283	LMNTD2	lamin tail domain containing 2 [Source:HGNC Symbol;Acc:HGNC:28561]
ENSG00000100290	-0,2121899	-0,5749661	0,13133001	0,72519861	0,87734298	BIK	BCL2 interacting killer [Source:HGNC Symbol;Acc:HGNC:1051]
ENSG00000115415	0,0525898	5,39850607	0,13138573	0,72514308	0,87734298	STAT1	signal transducer and activator of transcription 1 [Source:HGNC Symbol;Acc:HGNC:11362]
ENSG00000180938	-0,075353	1,74107039	0,13121942	0,72530889	0,87734298	ZNF572	zinc finger protein 572 [Source:HGNC Symbol;Acc:HGNC:26758]
ENSG00000224032	-0,0605314	3,92248956	0,13118435	0,72534387	0,87734298	EPB41L4A-AS1	EPB41L4A antisense RNA 1 [Source:HGNC Symbol;Acc:HGNC:30749]
ENSG00000259438	0,20454696	-1,9356303	0,13123231	0,72529603	0,87734298	MAPK6-DT	MAPK6 divergent transcript [Source:HGNC Symbol;Acc:HGNC:53900]
ENSG00000155093	0,05842749	5,41217141	0,13103089	0,72549699	0,87745554	PTPRN2	protein tyrosine phosphatase receptor type N2 [Source:HGNC Symbol;Acc:HGNC:9677]
ENSG00000176986	0,07193967	6,45653307	0,13095895	0,72556881	0,87746976	SEC24C	SEC24 homolog C, COPII coat complex component [Source:HGNC Symbol;Acc:HGNC:10705]
ENSG00000119401	-0,0668823	3,5530105	0,13078041	0,72574714	0,87761278	TRIM32	tripartite motif containing 32 [Source:HGNC Symbol;Acc:HGNC:16380]
ENSG00000010292	-0,0606119	6,75710889	0,13068054	0,72584696	0,87761763	NCAPD2	non-SMC condensin I complex subunit D2 [Source:HGNC Symbol;Acc:HGNC:24305]
ENSG00000124356	-0,0498655	5,36499535	0,13065619	0,7258713	0,87761763	STAMBP	STAM binding protein [Source:HGNC Symbol;Acc:HGNC:16950]
ENSG00000123999	-0,1406246	0,59433645	0,13013067	0,72639733	0,87810828	INHA	inhibin subunit alpha [Source:HGNC Symbol;Acc:HGNC:6065]
ENSG00000164978	-0,0641194	3,8604343	0,13016122	0,72636671	0,87810828	NUDT2	nudix hydrolase 2 [Source:HGNC Symbol;Acc:HGNC:8049]
ENSG00000245205	0,06727014	2,85604833	0,12992333	0,72660519	0,87828688	EEF1A1P4	eukaryotic translation elongation factor 1 alpha 1 pseudogene 4 [Source:HGNC Symbol;Acc:HGNC:3199]
ENSG00000170584	-0,0655001	4,96370469	0,12982095	0,7267079	0,87833836	NUDCD2	NudC domain containing 2 [Source:HGNC Symbol;Acc:HGNC:30535]
ENSG00000137876	0,04874547	6,90338217	0,12960395	0,72692576	0,87852899	RSL24D1	ribosomal L24 domain containing 1 [Source:HGNC Symbol;Acc:HGNC:18479]
ENSG00000167136	-0,051195	4,72817082	0,12943007	0,72710048	0,87866745	ENDOG	endonuclease G [Source:HGNC Symbol;Acc:HGNC:3346]
ENSG00000167676	0,13482222	0,62001725	0,1291584	0,72737372	0,87892495	PLIN4	perilipin 4 [Source:HGNC Symbol;Acc:HGNC:29393]
ENSG00000176428	-0,1028831	1,26342217	0,12898527	0,72754803	0,87906287	VPS37D	VPS37D subunit of ESCRT-I [Source:HGNC Symbol;Acc:HGNC:18287]
ENSG00000105866	-0,1364398	-0,3115983	0,12852197	0,72801513	0,87955451	SP4	Sp4 transcription factor [Source:HGNC Symbol;Acc:HGNC:11209]
ENSG00000170832	0,05751266	4,26751115	0,12823262	0,72830735	0,87983383	USP32	ubiquitin specific peptidase 32 [Source:HGNC Symbol;Acc:HGNC:19143]
ENSG00000237491	-0,353285	-1,9997057	0,12817381	0,72836678	0,87983383	LINC01409	long intergenic non-protein coding RNA 1409 [Source:HGNC Symbol;Acc:HGNC:50701]
ENSG00000182888	0,19133585	-1,8179737	0,12794739	0,72859576	0,88003765	RPS27AP20	RPS27A pseudogene 20 [Source:HGNC Symbol;Acc:HGNC:54884]
ENSG00000123342	-0,2353493	-1,3757708	0,12740259	0,72914767	0,88063148	MMP19	matrix metallopeptidase 19 [Source:HGNC Symbol;Acc:HGNC:7165]
ENSG00000103148	0,05221222	5,11188476	0,12724391	0,72930867	0,88075311	NPRL3	NPR3 like, GATOR1 complex subunit [Source:HGNC Symbol;Acc:HGNC:14124]
ENSG00000131747	-0,0671026	7,08151876	0,12718	0,72937355	0,88075866	TOP2A	DNA topoisomerase II alpha [Source:HGNC Symbol;Acc:HGNC:11989]
ENSG00000085662	-0,0515221	7,54051779	0,12688057	0,72967777	0,88102568	AKR1B1	aldo-keto reductase family 1 member B [Source:HGNC Symbol;Acc:HGNC:381]

ENSG00000234702	0,15906805	-1,0818388	0,12684366	0,72971529	0,88102568	VDAC1P3	voltage dependent anion channel 1 pseudogene 3 [Source:HGNC Symbol;Acc:HGNC:37478]
ENSG00000162734	-0,0535607	4,34320408	0,126781	0,72977901	0,8810298	PEA15	proliferation and apoptosis adaptor protein 15 [Source:HGNC Symbol;Acc:HGNC:8822]
ENSG00000063660	0,07246709	4,52478906	0,1265649	0,72999892	0,8811377	GPC1	glypican 1 [Source:HGNC Symbol;Acc:HGNC:4449]
ENSG00000064932	-0,0524929	4,29860975	0,1266239	0,72993886	0,8811377	SBNO2	strawberry notch homolog 2 [Source:HGNC Symbol;Acc:HGNC:29158]
ENSG00000166548	-0,0573355	3,38517321	0,12651539	0,73004933	0,8811377	TK2	thymidine kinase 2 [Source:HGNC Symbol;Acc:HGNC:11831]
ENSG00000170909	0,15032283	-0,3176052	0,12619597	0,73037484	0,88139732	OSCAR	osteoclast associated Ig-like receptor [Source:HGNC Symbol;Acc:HGNC:29960]
ENSG00000171574	-0,0677283	2,07532974	0,12616895	0,7304024	0,88139732	ZNF584	zinc finger protein 584 [Source:HGNC Symbol;Acc:HGNC:27318]
ENSG00000260804	0,19357846	-0,6030112	0,12612676	0,73044544	0,88139732	LINC01963	long intergenic non-protein coding RNA 1963 [Source:HGNC Symbol;Acc:HGNC:25283]
ENSG00000275763	0,17131616	-1,1009539	0,12598608	0,73058898	0,88149773	ZNF516-DT	ZNF516 divergent transcript [Source:HGNC Symbol;Acc:HGNC:51248]
ENSG00000112033	0,06404554	2,88035543	0,12579293	0,73078623	0,8816629	PPARD	peroxisome proliferator activated receptor delta [Source:HGNC Symbol;Acc:HGNC:9235]
ENSG00000081154	0,05986868	5,4683653	0,12567004	0,73091182	0,8816688	PCNP	PEST proteolytic signal containing nuclear protein [Source:HGNC Symbol;Acc:HGNC:30023]
ENSG00000110871	-0,0512665	5,04859243	0,12567255	0,73090925	0,8816688	COQ5	coenzyme Q5, methyltransferase [Source:HGNC Symbol;Acc:HGNC:28722]
ENSG00000131652	-0,0581879	5,5756015	0,12545092	0,73113592	0,88173189	THOC6	THO complex 6 [Source:HGNC Symbol;Acc:HGNC:28369]
ENSG00000163032	0,06502675	5,76305397	0,12549378	0,73109206	0,88173189	VSNL1	visinin like 1 [Source:HGNC Symbol;Acc:HGNC:12722]
ENSG00000261377	0,07387589	1,58068918	0,12544186	0,73114519	0,88173189	PDCD6IPP2	PDCD6IP pseudogene 2 [Source:HGNC Symbol;Acc:HGNC:49873]
ENSG00000173349	-0,061755	4,00415889	0,12509057	0,73150495	0,88209292	SFT2D3	SFT2 domain containing 3 [Source:HGNC Symbol;Acc:HGNC:28767]
ENSG00000100347	0,04845176	6,45802074	0,12476415	0,73183974	0,88226867	SAMM50	SAMM50 sorting and assembly machinery component [Source:HGNC Symbol;Acc:HGNC:24276]
ENSG00000119397	-0,0893792	3,11330359	0,12482651	0,73177575	0,88226867	CNTRL	centriolin [Source:HGNC Symbol;Acc:HGNC:1858]
ENSG00000130717	0,07777121	3,73898575	0,12483243	0,73176967	0,88226867	UCK1	uridine-cytidine kinase 1 [Source:HGNC Symbol;Acc:HGNC:14859]
ENSG00000140905	0,1332986	0,16915931	0,12465418	0,73195265	0,88226867	GCSH	glycine cleavage system protein H [Source:HGNC Symbol;Acc:HGNC:4208]
ENSG00000228612	0,19634943	-1,2439693	0,12468942	0,73191646	0,88226867	HK2P1	hexokinase 2 pseudogene 1 [Source:HGNC Symbol;Acc:HGNC:4924]
ENSG00000054118	-0,098001	7,16103736	0,12433528	0,73228038	0,88249138	THRAP3	thyroid hormone receptor associated protein 3 [Source:HGNC Symbol;Acc:HGNC:22964]
ENSG00000165156	-0,0659734	2,65856183	0,12423936	0,73237905	0,88249138	ZHX1	zinc fingers and homeoboxes 1 [Source:HGNC Symbol;Acc:HGNC:12871]
ENSG00000197006	0,0610015	6,69567979	0,12435075	0,73226447	0,88249138	METTL9	methyltransferase like 9 [Source:HGNC Symbol;Acc:HGNC:24586]
ENSG00000226040	-0,1394269	-0,752886	0,1242904	0,73232654	0,88249138	AC005740.1	glycine cleavage system protein H (aminomethyl carrier) (GCSH) pseudogene
ENSG00000105426	0,09912012	4,82880213	0,12412016	0,73250172	0,88250838	PTPRS	protein tyrosine phosphatase receptor type S [Source:HGNC Symbol;Acc:HGNC:9681]
ENSG00000164199	0,12625276	0,24428936	0,12410826	0,73251398	0,88250838	ADGRV1	adhesion G protein-coupled receptor V1 [Source:HGNC Symbol;Acc:HGNC:17416]
ENSG00000105576	0,06245196	5,29253783	0,12384897	0,73278108	0,88263547	TNPO2	transportin 2 [Source:HGNC Symbol;Acc:HGNC:19998]
ENSG00000143373	-0,0579019	4,17677663	0,12389035	0,73273843	0,88263547	ZNF687	zinc finger protein 687 [Source:HGNC Symbol;Acc:HGNC:29277]
ENSG00000177133	-0,2529877	-1,4484433	0,12382134	0,73280956	0,88263547	PRDM16-DT	PRDM16 divergent transcript [Source:HGNC Symbol;Acc:HGNC:48664]
ENSG00000185963	0,07722093	4,56041485	0,12377132	0,73286113	0,88263547	BICD2	BICD cargo adaptor 2 [Source:HGNC Symbol;Acc:HGNC:17208]
ENSG00000175376	0,05650257	4,20651977	0,12358497	0,73305336	0,88279421	EIF1AD	eukaryotic translation initiation factor 1A domain containing [Source:HGNC Symbol;Acc:HGNC:28147]
ENSG00000044459	-0,0954996	0,90921572	0,12330361	0,7333439	0,88299493	CNTLN	centlein [Source:HGNC Symbol;Acc:HGNC:23432]
ENSG00000064933	0,05364242	3,88045582	0,12331101	0,73333625	0,88299493	PMS1	PMS1 homolog 1, mismatch repair system component [Source:HGNC Symbol;Acc:HGNC:9121]
ENSG00000092098	0,18935766	-1,4309968	0,12321661	0,73343382	0,88299493	RNF31	ring finger protein 31 [Source:HGNC Symbol;Acc:HGNC:16031]
ENSG00000101049	-0,0635825	3,85094318	0,12318954	0,73346181	0,88299493	SGK2	serum/glucocorticoid regulated kinase 2 [Source:HGNC Symbol;Acc:HGNC:13900]
ENSG00000183971	0,11031174	-0,0812072	0,12301646	0,73364081	0,88313766	NPW	neuropeptide W [Source:HGNC Symbol;Acc:HGNC:30509]
ENSG00000156650	0,20422902	2,41197712	0,12266586	0,73400388	0,88321451	KAT6B	lysine acetyltransferase 6B [Source:HGNC Symbol;Acc:HGNC:17582]
ENSG00000228589	0,09035911	0,88634449	0,1226629	0,73400694	0,88321451	SPCS2P4	signal peptidase complex subunit 2 pseudogene 4 [Source:HGNC Symbol;Acc:HGNC:45237]
ENSG00000253948	0,29331628	-1,5348739	0,12276852	0,73389751	0,88321451	VPS13B-DT	VPS13B divergent transcript [Source:HGNC Symbol;Acc:HGNC:54375]
ENSG00000275052	-0,0569304	5,30205418	0,122816	0,73384833	0,88321451	PPP4R3B	protein phosphatase 4 regulatory subunit 3B [Source:HGNC Symbol;Acc:HGNC:29267]
ENSG00000277142	0,14353375	-0,5809171	0,12283689	0,73382669	0,88321451	LINC00235	long intergenic non-protein coding RNA 235 [Source:HGNC Symbol;Acc:HGNC:14138]
ENSG00000126247	0,0479795	8,00642551	0,12239397	0,73428583	0,88340457	CAPNS1	calpain small subunit 1 [Source:HGNC Symbol;Acc:HGNC:1481]
ENSG00000152457	-0,0725999	2,33720569	0,12243157	0,73424681	0,88340457	DCLRE1C	DNA cross-link repair 1C [Source:HGNC Symbol;Acc:HGNC:17642]

ENSG00000160712	-0,1083592	4,07247884	0,12229056	0,73439315	0,88346093	IL6R	interleukin 6 receptor [Source:HGNC Symbol;Acc:HGNC:6019]
ENSG00000065559	-0,0545859	4,64354719	0,12193431	0,73476331	0,88383345	MAP2K4	mitogen-activated protein kinase kinase 4 [Source:HGNC Symbol;Acc:HGNC:6844]
ENSG00000127311	0,12900345	-0,4723927	0,12182409	0,73487796	0,88385915	HELB	DNA helicase B [Source:HGNC Symbol;Acc:HGNC:17196]
ENSG00000249853	0,12123316	-0,2487847	0,12179744	0,73490568	0,88385915	HS3ST5	heparan sulfate-glucosamine 3-sulfotransferase 5 [Source:HGNC Symbol;Acc:HGNC:19419]
ENSG00000159915	0,11760586	-0,2626802	0,12161468	0,73509594	0,88389396	ZNF233	zinc finger protein 233 [Source:HGNC Symbol;Acc:HGNC:30946]
ENSG00000173262	0,07008273	3,7667755	0,12167945	0,73502848	0,88389396	SLC2A14	solute carrier family 2 member 14 [Source:HGNC Symbol;Acc:HGNC:18301]
ENSG00000272872	-0,2938979	-2,0076201	0,12159528	0,73511614	0,88389396	AP000525.1	novel transcript, sense intronic to novel transcript
ENSG00000230832	-0,1656908	-1,7033447	0,12139082	0,73532919	0,88407737	SSBL4P	SSB like 4, pseudogene [Source:HGNC Symbol;Acc:HGNC:54985]
ENSG00000159788	-0,0506519	4,47911621	0,12126991	0,73545528	0,88408345	RGS12	regulator of G protein signaling 12 [Source:HGNC Symbol;Acc:HGNC:9994]
ENSG00000253540	0,16512174	-1,4442938	0,12131057	0,73541287	0,88408345	FAM86HP	family with sequence similarity 86 member H, pseudogene [Source:HGNC Symbol;Acc:HGNC:42359]
ENSG00000139343	-0,0576152	7,46643287	0,12118077	0,73554828	0,88412249	SNRPF	small nuclear ribonucleoprotein polypeptide F [Source:HGNC Symbol;Acc:HGNC:11162]
ENSG00000228928	0,18492879	-1,6338392	0,12108877	0,7356443	0,88416517	KPNA2P2	karyopherin subunit alpha 2 pseudogene 2 [Source:HGNC Symbol;Acc:HGNC:52872]
ENSG00000213239	0,16780586	-1,3197505	0,12102734	0,73570845	0,88416952	NPM1P32	nucleophosmin 1 pseudogene 32 [Source:HGNC Symbol;Acc:HGNC:45211]
ENSG00000124942	0,05915543	5,65852223	0,12096747	0,73577098	0,88417194	AHNAK	AHNAK nucleoprotein [Source:HGNC Symbol;Acc:HGNC:347]
ENSG00000109814	-0,0648412	8,61698251	0,12089135	0,7358505	0,88419477	UGDH	UDP-glucose 6-dehydrogenase [Source:HGNC Symbol;Acc:HGNC:12525]
ENSG00000100201	-0,0774343	6,73470051	0,12051558	0,73624351	0,88426216	DDX17	DEAD-box helicase 17 [Source:HGNC Symbol;Acc:HGNC:2740]
ENSG00000115946	0,055154	6,38418769	0,12064041	0,73611288	0,88426216	PNO1	partner of NOB1 homolog [Source:HGNC Symbol;Acc:HGNC:32790]
ENSG00000118898	-0,1152192	3,51174489	0,12069318	0,73605768	0,88426216	PPL	periplakin [Source:HGNC Symbol;Acc:HGNC:9273]
ENSG00000130560	0,05526941	5,44886893	0,12049507	0,73626498	0,88426216	UBAC1	UBA domain containing 1 [Source:HGNC Symbol;Acc:HGNC:30221]
ENSG00000185104	0,04533901	5,94907853	0,12049051	0,73626976	0,88426216	FAF1	Fas associated factor 1 [Source:HGNC Symbol;Acc:HGNC:3578]
ENSG00000218596	0,16668207	-1,1512905	0,12060338	0,73615162	0,88426216	AL162578.1	ribosomal protein S15a (RPS15A) pseudogene
ENSG00000155970	-0,1124017	0,26167074	0,12034926	0,73641769	0,88435289	MICU3	mitochondrial calcium uptake family member 3 [Source:HGNC Symbol;Acc:HGNC:27820]
ENSG00000259494	-0,0734593	3,11528258	0,12030279	0,73646637	0,88435289	MRPL46	mitochondrial ribosomal protein L46 [Source:HGNC Symbol;Acc:HGNC:1192]
ENSG00000100324	0,06436618	4,54619442	0,12017553	0,73659976	0,88444037	TAB1	TGF-beta activated kinase 1 (MAP3K7) binding protein 1 [Source:HGNC Symbol;Acc:HGNC:18157]
ENSG00000197857	0,07057737	3,99493032	0,12007891	0,73670109	0,88448933	ZNF44	zinc finger protein 44 [Source:HGNC Symbol;Acc:HGNC:13110]
ENSG00000179091	0,0455318	8,2850627	0,11990564	0,73688292	0,884566	CYC1	cytochrome c1 [Source:HGNC Symbol;Acc:HGNC:2579]
ENSG00000274904	-0,206309	-1,4192282	0,11990266	0,73688604	0,884566	AC093512.1	novel transcript, antisense to ALDOA
ENSG00000258645	0,0802862	1,14883482	0,11958409	0,73722073	0,88489505	HSPE1P2	heat shock protein family E (Hsp10) member 1 pseudogene 2 [Source:HGNC Symbol;Acc:HGNC:20052]
ENSG00000110395	0,14740768	3,13226859	0,11951176	0,7372968	0,88491365	CBL	Cbl proto-oncogene [Source:HGNC Symbol;Acc:HGNC:1541]
ENSG00000143575	-0,0531477	7,06442439	0,1194256	0,73738743	0,88494972	HAX1	HCLS1 associated protein X-1 [Source:HGNC Symbol;Acc:HGNC:16915]
ENSG00000238251	0,1654812	-1,3684363	0,11930943	0,7375097	0,88502375	NSA2P7	NSA2 pseudogene 7 [Source:HGNC Symbol;Acc:HGNC:54588]
ENSG00000123179	0,05828164	6,38411833	0,1192099	0,73761451	0,88502481	EBPL	EBP like [Source:HGNC Symbol;Acc:HGNC:18061]
ENSG00000175611	-0,1371237	0,0034053	0,11919353	0,73763174	0,88502481	LINC00476	long intergenic non-protein coding RNA 476 [Source:HGNC Symbol;Acc:HGNC:27858]
ENSG00000111319	-0,0918222	1,71737012	0,1187521	0,73809721	0,88528228	SCNN1A	sodium channel epithelial 1 subunit alpha [Source:HGNC Symbol;Acc:HGNC:10599]
ENSG00000113971	-0,1372333	0,18227713	0,11859873	0,73825916	0,88528228	NPHP3	nephrocystin 3 [Source:HGNC Symbol;Acc:HGNC:7907]
ENSG00000115827	-0,0505396	3,80966907	0,11873592	0,73811429	0,88528228	DCAF17	DDB1 and CUL4 associated factor 17 [Source:HGNC Symbol;Acc:HGNC:25784]
ENSG00000150401	0,05928998	2,78629912	0,11863501	0,73822084	0,88528228	DCUN1D2	defective in cullin neddylation 1 domain containing 2 [Source:HGNC Symbol;Acc:HGNC:20328]
ENSG00000171960	0,05208048	5,87659261	0,11858798	0,73827052	0,88528228	PPIH	peptidylprolyl isomerase H [Source:HGNC Symbol;Acc:HGNC:14651]
ENSG00000176183	0,14493326	-0,7346422	0,11872251	0,73812845	0,88528228	AC233964.1	actin, beta (ACTB) pseudogene
ENSG00000228794	-0,0756838	2,33273173	0,11885963	0,73798374	0,88528228	LINC01128	long intergenic non-protein coding RNA 1128 [Source:HGNC Symbol;Acc:HGNC:49377]
ENSG00000266282	0,15281182	-1,1999365	0,11843924	0,7384277	0,88539809	UBL5P2	ubiquitin like 5 pseudogene 2 [Source:HGNC Symbol;Acc:HGNC:44640]
ENSG00000225470	-0,0743362	2,31116572	0,11837034	0,73850056	0,88541277	JPX	JPX transcript, XIST activator [Source:HGNC Symbol;Acc:HGNC:37191]
ENSG00000213750	-0,1749846	-1,6341014	0,11822216	0,7386573	0,88552803	AP002982.1	ribosomal protein S2 (RPS2) pseudogene
ENSG00000163376	-0,0781165	1,29847157	0,118136	0,7387485	0,88556469	KBTBD8	kelch repeat and BTB domain containing 8 [Source:HGNC Symbol;Acc:HGNC:30691]

ENSG00000171148	0,04512134	7,15364888	0,11789301	0,7390059	0,88576847	TADA3	transcriptional adaptor 3 [Source:HGNC Symbol;Acc:HGNC:19422]
ENSG00000227345	-0,0510391	4,15078357	0,11786106	0,73903976	0,88576847	PARG	poly(ADP-ribose) glycohydrolase [Source:HGNC Symbol;Acc:HGNC:8605]
ENSG00000103197	0,06311852	4,16502047	0,11764937	0,73926428	0,88596487	TSC2	TSC complex subunit 2 [Source:HGNC Symbol;Acc:HGNC:12363]
ENSG00000197021	-0,0608903	4,0298357	0,11752439	0,73939694	0,88602707	EOLA2	endothelium and lymphocyte associated ASCH domain 2 [Source:HGNC Symbol;Acc:HGNC:17402]
ENSG00000243746	-0,1107341	0,45993314	0,11748622	0,73943747	0,88602707	EEF1A1P10	eukaryotic translation elongation factor 1 alpha 1 pseudogene 10 [Source:HGNC Symbol;Acc:HGNC:3205]
ENSG00000147119	0,05895102	2,65414638	0,11742496	0,73950253	0,88603235	CHST7	carbohydrate sulfotransferase 7 [Source:HGNC Symbol;Acc:HGNC:13817]
ENSG00000105538	-0,1654036	-0,799138	0,11722302	0,73971715	0,88621682	RASIP1	Ras interacting protein 1 [Source:HGNC Symbol;Acc:HGNC:24716]
ENSG00000032742	-0,0575954	3,05555844	0,11683149	0,74013387	0,88647947	IFT88	intraflagellar transport 88 [Source:HGNC Symbol;Acc:HGNC:20606]
ENSG00000163481	0,05003113	5,5536746	0,11675273	0,74021778	0,88647947	RNF25	ring finger protein 25 [Source:HGNC Symbol;Acc:HGNC:14662]
ENSG00000196812	-0,0632523	2,78350312	0,11673208	0,74023979	0,88647947	ZSCAN16	zinc finger and SCAN domain containing 16 [Source:HGNC Symbol;Acc:HGNC:20813]
ENSG00000236698	0,14057266	-0,52064	0,11687446	0,74008809	0,88647947	EIF1AXP1	EIF1AX pseudogene 1 [Source:HGNC Symbol;Acc:HGNC:19900]
ENSG00000267060	0,14017034	-0,2890755	0,11680367	0,74016351	0,88647947	PTGES3L	prostaglandin E synthase 3 like [Source:HGNC Symbol;Acc:HGNC:43943]
ENSG00000123739	-0,072124	1,63670268	0,11659661	0,74038423	0,88657977	PLA2G12A	phospholipase A2 group XIIA [Source:HGNC Symbol;Acc:HGNC:18554]
ENSG00000213104	0,10798806	0,10453341	0,11637321	0,74062262	0,88679255	NPM1P46	nucleophosmin 1 pseudogene 46 [Source:HGNC Symbol;Acc:HGNC:45225]
ENSG00000153214	-0,071475	3,79017155	0,11612064	0,74089245	0,88704292	TMEM87B	transmembrane protein 87B [Source:HGNC Symbol;Acc:HGNC:25913]
ENSG00000248568	-0,1508878	-0,8452581	0,11604427	0,7409741	0,88706799	KRT8P48	keratin 8 pseudogene 48 [Source:HGNC Symbol;Acc:HGNC:48344]
ENSG00000234160	-0,198987	-0,357625	0,11589096	0,74113811	0,88719163	AL513165.1	novel transcript
ENSG00000075624	-0,052988	11,9948543	0,11557208	0,74147963	0,88752773	ACTB	actin beta [Source:HGNC Symbol;Acc:HGNC:132]
ENSG00000110013	-0,0713993	3,24211903	0,11510767	0,74197798	0,88773211	SIAE	sialic acid acetylesterase [Source:HGNC Symbol;Acc:HGNC:18187]
ENSG00000110841	-0,087675	2,99846625	0,11516313	0,74191841	0,88773211	PPFIBP1	PPFIA binding protein 1 [Source:HGNC Symbol;Acc:HGNC:9249]
ENSG00000119203	0,04621828	6,30449253	0,11513958	0,7419437	0,88773211	CPSF3	cleavage and polyadenylation specific factor 3 [Source:HGNC Symbol;Acc:HGNC:2326]
ENSG00000165629	0,05053535	8,08704098	0,11527062	0,741803	0,88773211	ATP5F1C	ATP synthase F1 subunit gamma [Source:HGNC Symbol;Acc:HGNC:833]
ENSG00000183426	0,08451691	1,2258292	0,11516496	0,74191644	0,88773211	NPIPA1	nuclear pore complex interacting protein family member A1 [Source:HGNC Symbol;Acc:HGNC:7909]
ENSG00000198113	-0,0593444	5,23400719	0,11507323	0,74201498	0,88773211	TOR4A	torsin family 4 member A [Source:HGNC Symbol;Acc:HGNC:25981]
ENSG00000125970	0,06013543	8,84075158	0,11471388	0,74240143	0,88773988	RALY	RALY heterogeneous nuclear ribonucleoprotein [Source:HGNC Symbol;Acc:HGNC:15921]
ENSG00000131899	-0,0725135	4,76262155	0,11467169	0,74244684	0,88773988	LLGL1	LLGL scribble cell polarity complex component 1 [Source:HGNC Symbol;Acc:HGNC:6628]
ENSG00000135372	-0,0803586	6,39047955	0,11499599	0,74209799	0,88773988	NAT10	N-acetyltransferase 10 [Source:HGNC Symbol;Acc:HGNC:29830]
ENSG00000154328	-0,0575915	4,06819835	0,11480778	0,74230038	0,88773988	NEIL2	nei like DNA glycosylase 2 [Source:HGNC Symbol;Acc:HGNC:18956]
ENSG00000183208	0,14608356	-0,5467222	0,11475115	0,74236131	0,88773988	GDPGP1	GDP-D-glucose phosphorylase 1 [Source:HGNC Symbol;Acc:HGNC:34360]
ENSG00000185158	-0,0871168	1,46312221	0,11468312	0,74243454	0,88773988	LRRC37B	leucine rich repeat containing 37B [Source:HGNC Symbol;Acc:HGNC:29070]
ENSG00000229237	-0,2093453	-1,9583156	0,11468509	0,74243241	0,88773988	HMGN1P37	high mobility group nucleosome binding domain 1 pseudogene 37 [Source:HGNC Symbol;Acc:HGNC:39421]
ENSG00000165637	0,06519209	7,78098518	0,1143707	0,74277112	0,88791628	VDAC2	voltage dependent anion channel 2 [Source:HGNC Symbol;Acc:HGNC:12672]
ENSG00000233967	-0,3017808	-2,3626749	0,11436552	0,7427767	0,88791628	AL359715.1	novel transcript
ENSG00000261799	0,34070901	-1,8598941	0,1143726	0,74276908	0,88791628	AC007406.4	novel transcript
ENSG00000157873	0,08046503	3,62397238	0,11429697	0,74285063	0,88793199	TNFRSF14	TNF receptor superfamily member 14 [Source:HGNC Symbol;Acc:HGNC:11912]
ENSG00000135930	-0,056878	6,35182638	0,11417263	0,74298478	0,88798929	EIF4E2	eukaryotic translation initiation factor 4E family member 2 [Source:HGNC Symbol;Acc:HGNC:3293]
ENSG00000139636	0,05343332	3,90097706	0,11413988	0,74302013	0,88798929	LMBR1L	limb development membrane protein 1 like [Source:HGNC Symbol;Acc:HGNC:18268]
ENSG00000218739	0,05619627	4,00130565	0,11387654	0,74330455	0,88818389	CEBPZOS	CEBPZ opposite strand [Source:HGNC Symbol;Acc:HGNC:49288]
ENSG00000287817	-0,1650309	-0,6717967	0,11389383	0,74328587	0,88818389	AL096701.4	novel transcript, antisense to EIF4ENIF1
ENSG00000213707	0,11668509	-0,3145149	0,11378436	0,74340421	0,88823032	HMGB1P10	high mobility group box 1 pseudogene 10 [Source:HGNC Symbol;Acc:HGNC:4994]
ENSG00000079313	-0,0643004	4,12167111	0,11362918	0,74357207	0,88828558	REXO1	RNA exonuclease 1 homolog [Source:HGNC Symbol;Acc:HGNC:24616]
ENSG00000274180	-0,1272455	-0,2407401	0,113643	0,74355712	0,88828558	NATD1	N-acetyltransferase domain containing 1 [Source:HGNC Symbol;Acc:HGNC:30770]
ENSG00000280088	-0,1774251	-0,8756977	0,11342741	0,74379052	0,88847389	AC126474.2	TEC
ENSG00000146674	-0,136211	-0,4767307	0,11334959	0,74387483	0,88850195	IGFBP3	insulin like growth factor binding protein 3 [Source:HGNC Symbol;Acc:HGNC:5472]

ENSG00000245522	0,12893929	-0,7750242	0,11325235	0,74398023	0,88852165	LINC02709	long intergenic non-protein coding RNA 2709 [Source:HGNC Symbol;Acc:HGNC:54226]
ENSG00000280832	-0,1305453	-0,505844	0,11322216	0,74401296	0,88852165	GSEC	G-quadruplex forming sequence containing lncRNA [Source:HGNC Symbol;Acc:HGNC:48645]
ENSG00000250802	0,17763946	-1,7820903	0,11307885	0,7441684	0,88863464	ZBED3-AS1	ZBED3 antisense RNA 1 [Source:HGNC Symbol;Acc:HGNC:44188]
ENSG00000156983	0,0571981	4,49910461	0,11271613	0,74456234	0,88903238	BRPF1	bromodomain and PHD finger containing 1 [Source:HGNC Symbol;Acc:HGNC:14255]
ENSG00000136935	-0,0670325	4,1697521	0,11260196	0,74468648	0,88903528	GOLGA1	golgin A1 [Source:HGNC Symbol;Acc:HGNC:4424]
ENSG00000280161	0,15424854	-1,3462734	0,11262101	0,74466576	0,88903528	AC022413.1	TEC
ENSG00000003989	0,07673996	5,02754835	0,11242068	0,74488373	0,88919811	SLC7A2	solute carrier family 7 member 2 [Source:HGNC Symbol;Acc:HGNC:11060]
ENSG00000134291	0,07681251	6,04264928	0,11215678	0,74517122	0,88937911	TMEM106C	transmembrane protein 106C [Source:HGNC Symbol;Acc:HGNC:28775]
ENSG00000260290	-0,0905001	0,74997392	0,11210429	0,74522845	0,88937911	AC092115.1	non-POU domain containing, octamer-binding (NONO) pseudogene
ENSG00000261324	-0,1324204	-0,6613502	0,11205805	0,74527887	0,88937911	AC010168.2	novel transcript, overlapping HIST4H4
ENSG00000283341	0,19121095	-1,2240229	0,11206829	0,74526771	0,88937911	AC068205.2	novel transcript
ENSG00000086200	0,05075905	4,22698115	0,11147031	0,74592081	0,88994325	IPO11	importin 11 [Source:HGNC Symbol;Acc:HGNC:20628]
ENSG00000173905	-0,1133842	4,64906052	0,11140225	0,74599528	0,88994325	GOLIM4	golgi integral membrane protein 4 [Source:HGNC Symbol;Acc:HGNC:15448]
ENSG00000203985	-0,1869253	-0,8517551	0,11148699	0,74590257	0,88994325	LDLRAD1	low density lipoprotein receptor class A domain containing 1 [Source:HGNC Symbol;Acc:HGNC:32069]
ENSG00000285589	0,16828704	-1,4384326	0,11144983	0,74594322	0,88994325	AC010422.8	novel transcript
ENSG00000244073	-0,1131094	-0,4596912	0,11130411	0,74610269	0,88997799	RPS4XP6	ribosomal protein S4X pseudogene 6 [Source:HGNC Symbol;Acc:HGNC:36057]
ENSG00000277258	0,04363623	6,72820851	0,11126433	0,74614624	0,88997799	PCGF2	polycomb group ring finger 2 [Source:HGNC Symbol;Acc:HGNC:12929]
ENSG00000106399	-0,0637463	6,10472253	0,11118596	0,74623208	0,89000771	RPA3	replication protein A3 [Source:HGNC Symbol;Acc:HGNC:10291]
ENSG00000228399	-0,1959925	-1,7624161	0,11112775	0,74629585	0,89001111	AL109741.2	ribosomal protein S20 (RPS20) pseudogene
[truncated: 386,461 more chars]
